# Supplementary figures and images for: Sequencing at sea: challenges and experiences in Ion Torrent PGM sequencing during the 2013 Southern Line Islands Research Expedition
Source: PeerJ. 2014 Aug 19;2:e520. doi: 10.7717/peerj.520 (PMC4145072; doi:10.7717/peerj.520)

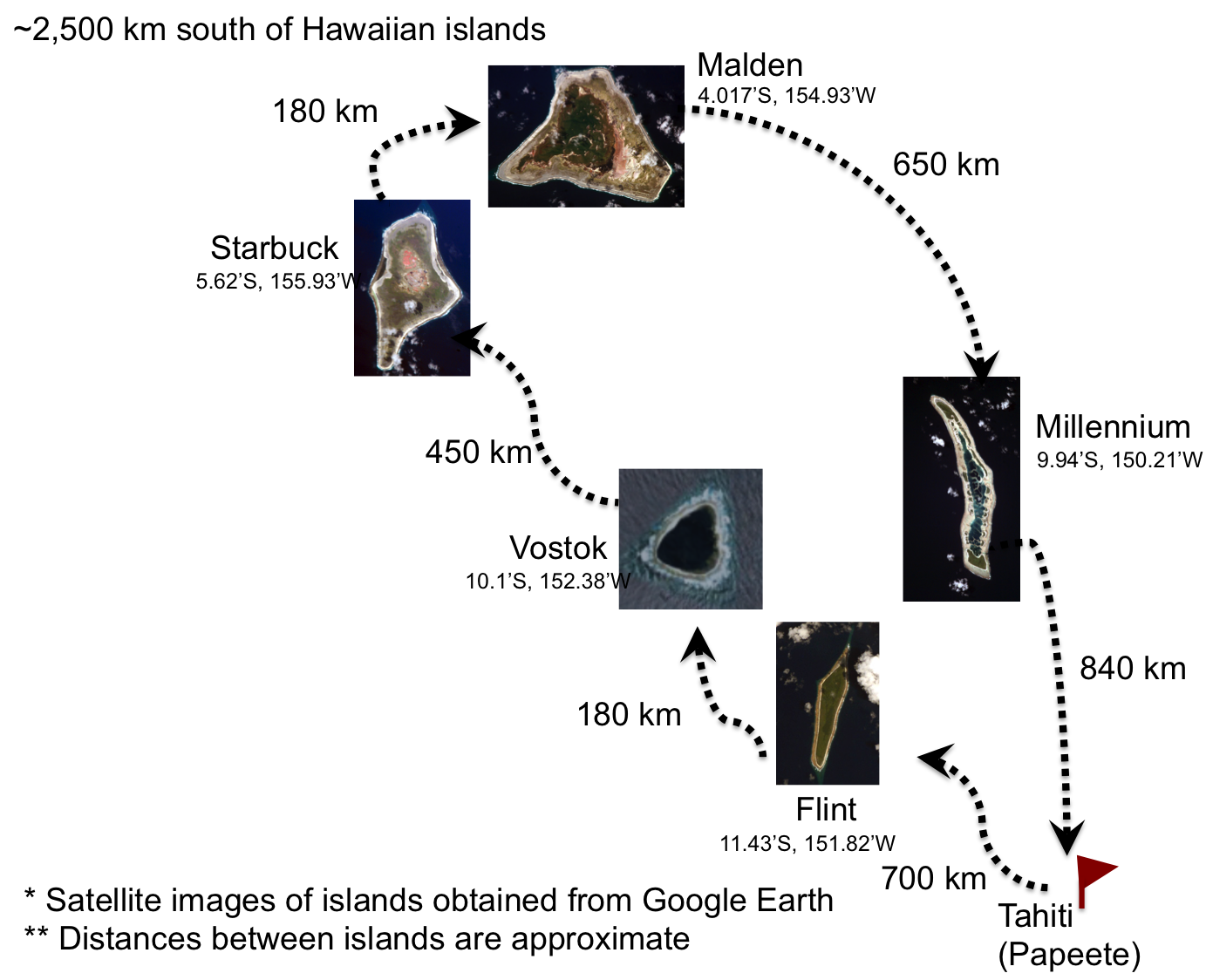

Supplement: Figure S1 — Expedition route of the 2013 Southern Line Island Expedition. The expedition left Papeete Harbor and circumnavigated the Southern Line Islands in a clockwise direction. [file peerj-02-520-s003.png]

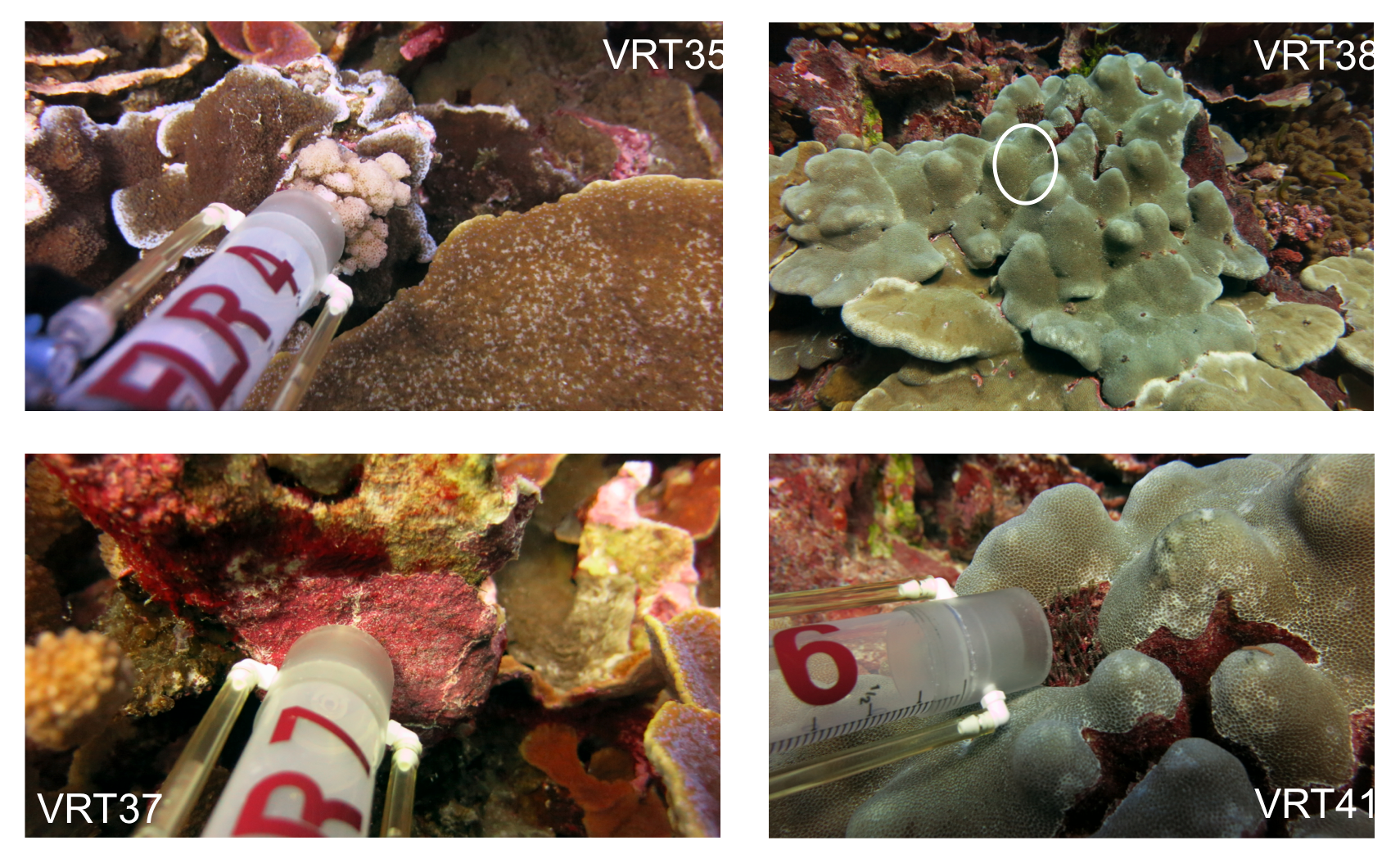

Supplement: Figure S2 — Examples of sampling surface for bacterial isolates. VRT35 and VRT38 were isolated from coral surfaces, while VRT37 and VRT41 were isolated from algae surfaces. [file peerj-02-520-s004.png]

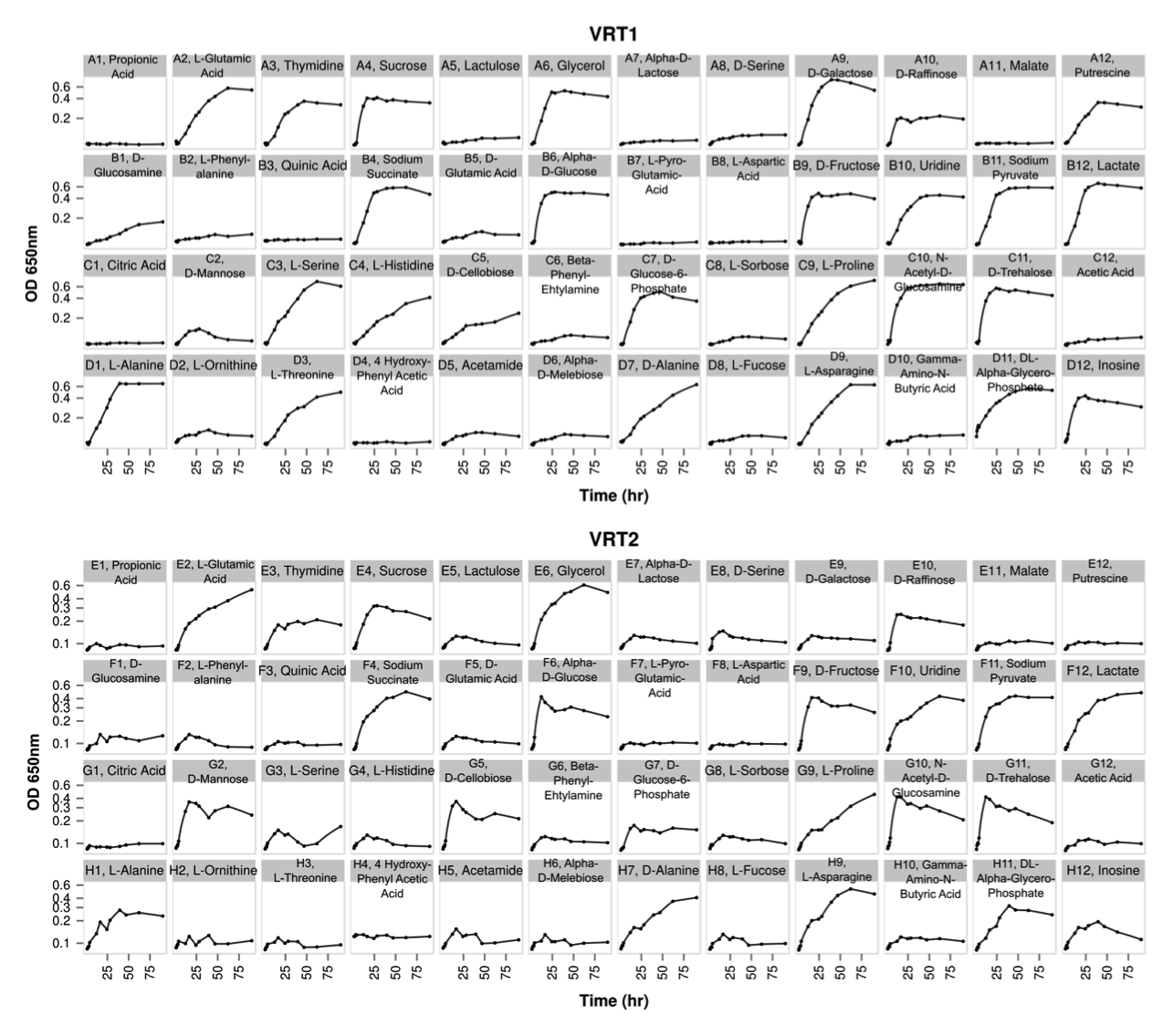

Supplement: Figure S4 — Growth curve of bacterial isolates in 48 different carbon sources indicated in Table S1. [file peerj-02-520-s006.png]

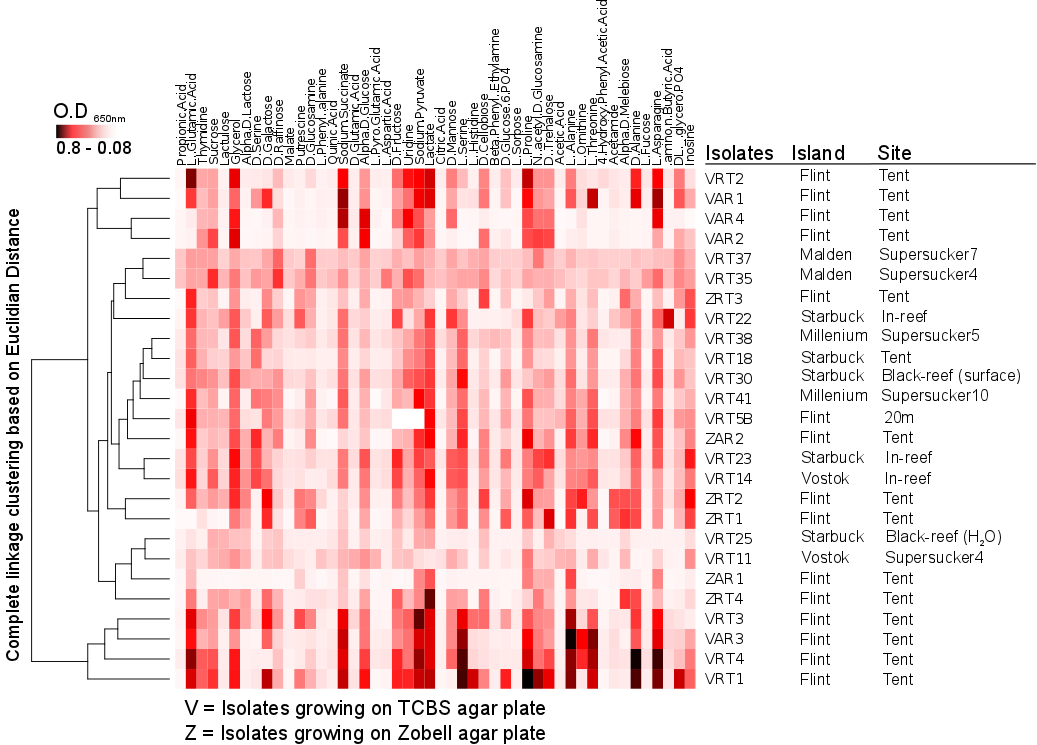

Supplement: Figure S5 — Heatmap showing the growth characteristics of each bacterial isolates from the 2013 Southern Line Island expedition in 48 different carbon sources. [file peerj-02-520-s007.png]

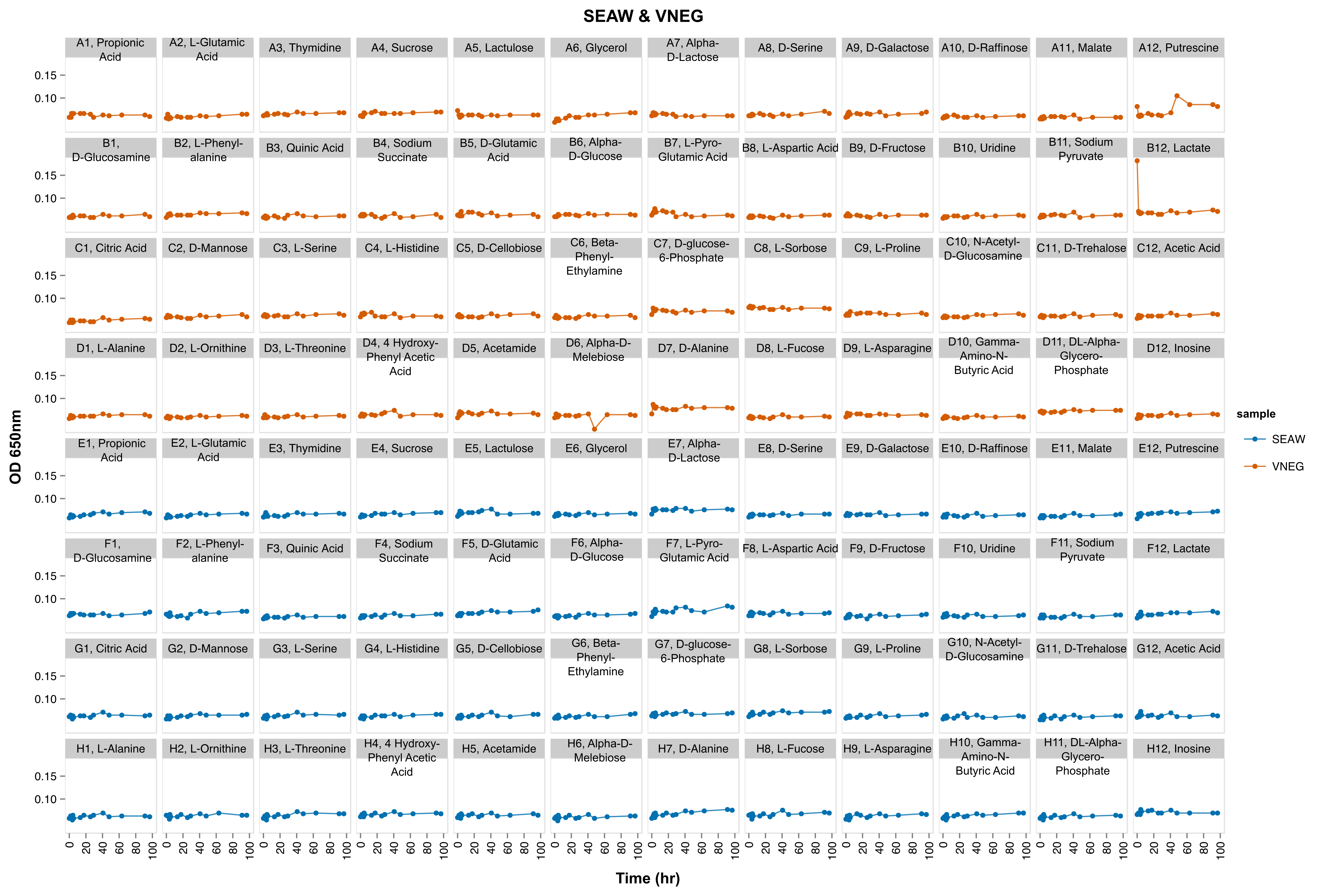

Supplement: Figure S6 — Growth curve of negative controls and filtered seawater-only samples in 48 different carbon sources indicated in Table S1. [file peerj-02-520-s008.png]

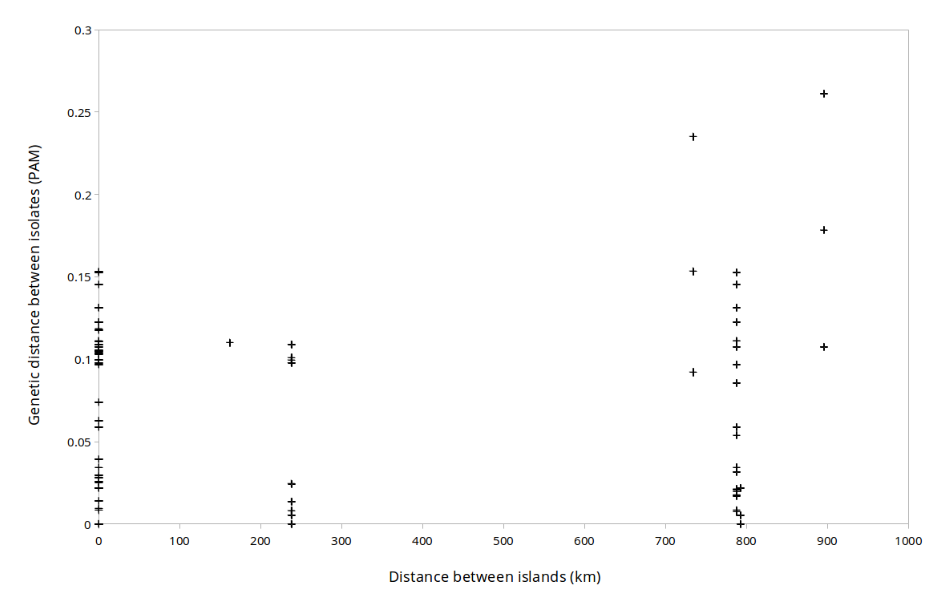

Supplement: Figure S7 — Correlation between physical distance and genetic distance in the microbial species purified on the Line Islands. [file peerj-02-520-s009.png]
