# Supplementary material for: Sequencing at sea: challenges and experiences in Ion Torrent PGM sequencing during the 2013 Southern Line Islands Research Expedition
Source: PeerJ. 2014 Aug 19;2:e520. doi: 10.7717/peerj.520 (PMC4145072; doi:10.7717/peerj.520)
Supplement: Figure S3 — Number of bases sequenced and number of contigs generated. The best-fit power regression of the data (shown with a gray line) has the equation [number of contigs] = 3.87∗1010 [base piars sequenced]−0.95 with an R2 of 0.667. [file peerj-02-520-s005.pdf]

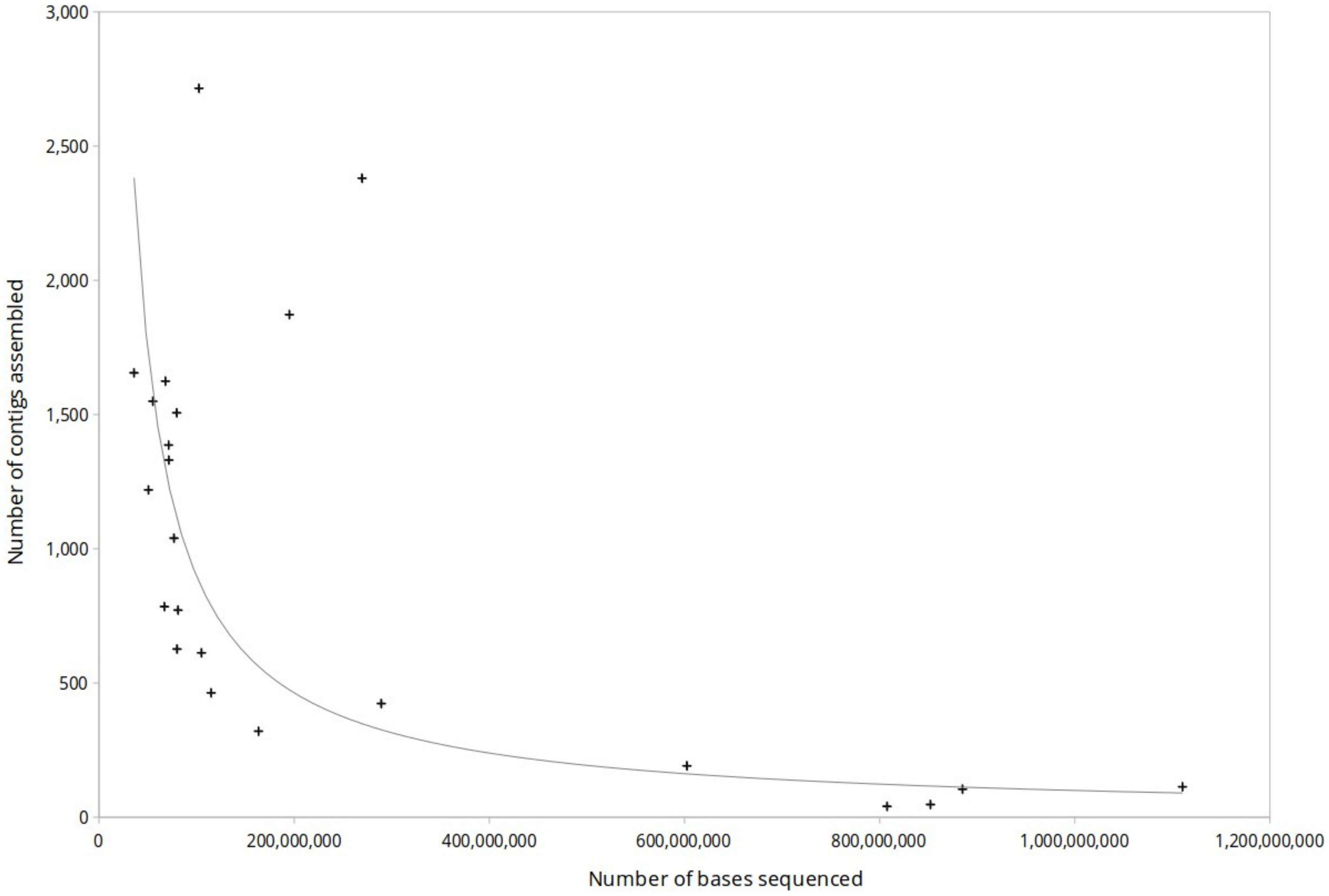

Supplemental Figure 3. Number of bases sequenced and number of contigs generated. The best-fit power regression of the data (shown with a gray line) has the equation [number of contigs] = $3.87 * 10^{10}$ [base piars sequenced]$^{-0.95}$ with an R^2 of 0.667.
