# Supplementary material for: Sequencing at sea: challenges and experiences in Ion Torrent PGM sequencing during the 2013 Southern Line Islands Research Expedition
Source: PeerJ. 2014 Aug 19;2:e520. doi: 10.7717/peerj.520 (PMC4145072; doi:10.7717/peerj.520)
Supplement: Supplementary file 5 [file peerj-02-520-s001.odt]

Supplemental Table 1: In-house phenotypic array plate. Each stock well contains 1ml of 6X basal media (6X MOPS media, 57 mM NH4Cl, 1.5 mM NaSO4, 30 μM CaCl2, 6 mM MgSO4, 1.9M NaCl, 7.92 mM K2HPO4, 60 mM KCl, 36 μM FeCl3) and 1ml of 5X carbon substrate. The substrates are used at a final concentration of 0.2% unless specified.

|  | 1 | 2 | 3 | 4 | 5 | 6 | 7 | 8 | 9 | 10 | 11 | 12 |
| --- | --- | --- | --- | --- | --- | --- | --- | --- | --- | --- | --- | --- |
| A | Propionic Acid | L- Glutamic Acid | Thymidine | Sucrose | Lactulose | Glycerol | Alpha D Lactose | D Serine | D Galactose | D Raffinose | Malate | Putrescine |
| B | D Glucosamine | L Phenyl- alanine | Quinic Acid | Sodium Succinate | D Glutamic Acid 0.1% | Alpha D Glucose | L Pyro Glutamic Acid | L Aspartic Acid 0.1% | D Fructose | Uridine | Sodium Pyruvate | Lactate |
| C | Citric Acid | D Mannose | L Serine | L- Histidine | D-Cellobiose | Beta Phenyl- Ethylamine | D Glucose 6 PO4 | L Sorbose | L Proline 0.1% | N- acetyl D Glucosamine 0.1% | D- Trehalose | Acetic Acid |
| D | L- Alanine | L Ornithine | L Threonine | 4 Hydroxy Phenyl Acetic Acid | Acetamide | Alpha D Melebiose | D Alanine | L Fucose | L Asparagine 0.1% | γ – amino-n-Butyric Acid 0.1% | DL-α-glycero PO4 | Inosine |
| E | Propionic Acid | L- Glutamic Acid | Thymidine | Sucrose | Lactulose | Glycerol | Alpha D Lactose | D Serine | D Galactose | D Raffinose | Malate | Putrescine |
| F | D Glucosamine | L Phenyl- alanine | Quinic Acid | Sodium Succinate | D Glutamic Acid 0.1% | Alpha D Glucose | L Pyro Glutamic Acid | L Aspartic Acid 0.1% | D Fructose | Uridine | Sodium Pyruvate | Lactate |
| G | Citric Acid | D Mannose | L Serine | L- Histidine | D Cellobiose | Beta Phenyl- Ethylamine | D Glucose 6 PO4 | L Sorbose | L Proline 0.1% | N- acetyl D Glucosamine 0.1% | D- Trehalose | Acetic Acid |
| H | L- Alanine | L Ornithine | L Threonine | 4 Hydroxy Phenyl Acetic Acid | Acetamide | Alpha D Melebiose | D Alanine | L Fucose | L Asparagine 0.1% | γ – amino-n-Butyric Acid 0.1% | DL-α-glycero PO4 | Inosine |
