# Supplementary material for: Sequencing at sea: challenges and experiences in Ion Torrent PGM sequencing during the 2013 Southern Line Islands Research Expedition
Source: PeerJ. 2014 Aug 19;2:e520. doi: 10.7717/peerj.520 (PMC4145072; doi:10.7717/peerj.520)
Supplement: Supplementary file 6 [file peerj-02-520-s002.odt]

OG	Function	VAR1	VAR2	VAR3	VAR4	VRT11	VRT14	VRT18	VRT1	VRT22	VRT23	VRT25	VRT2	VRT30	VRT35	VRT37	VRT38	VRT3	VRT41	VRT4	VRT5B	ZAR1	ZAR2	ZRT1	ZRT28	ZRT32	ZRT3

2681	NAD-dependent glyceraldehyde-3-phosphate dehydrogenase EC 1.2.1.12	fig|6666666.59329.peg.2353	fig|6666666.59330.peg.1550	fig|6666666.59331.peg.1204	fig|6666666.59332.peg.530	fig|6666666.59334.peg.1059	fig|6666666.59335.peg.2948	fig|6666666.59336.peg.1202	fig|6666666.59333.peg.3473	fig|6666666.59338.peg.1955	fig|6666666.59339.peg.271	fig|6666666.59340.peg.1177	fig|6666666.59337.peg.3126	fig|6666666.59342.peg.4918	fig|6666666.71181.peg.3143	fig|6666666.59344.peg.2893	fig|6666666.59345.peg.514	fig|6666666.59341.peg.644	fig|6666666.59347.peg.2434	fig|6666666.59346.peg.306	fig|6666666.59348.peg.192	fig|6666666.59349.peg.2743	fig|6666666.59350.peg.5180	fig|6666666.59351.peg.900	fig|6666666.59353.peg.1434	fig|6666666.59355.peg.3724	fig|6666666.59354.peg.3598

7452	Glutathione-regulated potassium-efflux system protein KefB	fig|6666666.59329.peg.4170	fig|6666666.59330.peg.890	fig|6666666.59331.peg.6342	fig|6666666.59332.peg.1067	fig|6666666.59334.peg.429	fig|6666666.59335.peg.1867	fig|6666666.59336.peg.2191	fig|6666666.59333.peg.1821	fig|6666666.59338.peg.5072	fig|6666666.59339.peg.8244	fig|6666666.59340.peg.2987	fig|6666666.59337.peg.5681	fig|6666666.59342.peg.6421	fig|6666666.71181.peg.409	fig|6666666.59344.peg.5044	fig|6666666.59345.peg.5562	fig|6666666.59341.peg.5670	fig|6666666.59347.peg.4967	fig|6666666.59346.peg.1998	fig|6666666.59348.peg.290	fig|6666666.59349.peg.4775	fig|6666666.59350.peg.5413	fig|6666666.59351.peg.627	fig|6666666.59353.peg.2265	fig|6666666.59355.peg.5991	fig|6666666.59354.peg.3430

4590	3-dehydroquinate synthase EC 4.2.3.4	fig|6666666.59329.peg.314	fig|6666666.59330.peg.2636	fig|6666666.59331.peg.2731	fig|6666666.59332.peg.1031	fig|6666666.59334.peg.848	fig|6666666.59335.peg.2484	fig|6666666.59336.peg.5970	fig|6666666.59333.peg.895	fig|6666666.59338.peg.3577	fig|6666666.59339.peg.8717	fig|6666666.59340.peg.365	fig|6666666.59337.peg.1702	fig|6666666.59342.peg.2759	fig|6666666.71181.peg.1831	fig|6666666.59344.peg.3065	fig|6666666.59345.peg.5380	fig|6666666.59341.peg.4080	fig|6666666.59347.peg.4945	fig|6666666.59346.peg.7	fig|6666666.59348.peg.1024	fig|6666666.59349.peg.528	fig|6666666.59350.peg.2170	fig|6666666.59351.peg.3852	fig|6666666.59353.peg.2219	fig|6666666.59355.peg.1911	fig|6666666.59354.peg.3574

2745	Bis 5'-nucleosyl -tetraphosphatase, symmetrical EC 3.6.1.41	fig|6666666.59329.peg.134	fig|6666666.59330.peg.820	fig|6666666.59331.peg.2695	fig|6666666.59332.peg.3917	fig|6666666.59334.peg.3348	fig|6666666.59335.peg.4483	fig|6666666.59336.peg.2656	fig|6666666.59333.peg.5402	fig|6666666.59338.peg.911	fig|6666666.59339.peg.7889	fig|6666666.59340.peg.431	fig|6666666.59337.peg.1382	fig|6666666.59342.peg.2179	fig|6666666.71181.peg.403	fig|6666666.59344.peg.1293	fig|6666666.59345.peg.2461	fig|6666666.59341.peg.2000	fig|6666666.59347.peg.3091	fig|6666666.59346.peg.4901	fig|6666666.59348.peg.175	fig|6666666.59349.peg.1874	fig|6666666.59350.peg.6229	fig|6666666.59351.peg.3648	fig|6666666.59353.peg.95	fig|6666666.59355.peg.705	fig|6666666.59354.peg.5334

10888	Ribosomal protein S6 glutaminyl transferase	fig|6666666.59329.peg.1208	fig|6666666.59330.peg.347	fig|6666666.59331.peg.2913	fig|6666666.59332.peg.4796	fig|6666666.59334.peg.1919	fig|6666666.59335.peg.138	fig|6666666.59336.peg.2693	fig|6666666.59333.peg.773	fig|6666666.59338.peg.449	fig|6666666.59339.peg.7835	fig|6666666.59340.peg.205	fig|6666666.59337.peg.1514	fig|6666666.59342.peg.1105	fig|6666666.71181.peg.1861	fig|6666666.59344.peg.4335	fig|6666666.59345.peg.3029	fig|6666666.59341.peg.1067	fig|6666666.59347.peg.3213	fig|6666666.59346.peg.2775	fig|6666666.59348.peg.4029	fig|6666666.59349.peg.3849	fig|6666666.59350.peg.7139	fig|6666666.59351.peg.4682	fig|6666666.59353.peg.3226	fig|6666666.59355.peg.3108	fig|6666666.59354.peg.5052

2039	Phosphoadenylyl-sulfate reductase [thioredoxin] EC 1.8.4.8	fig|6666666.59329.peg.5365	fig|6666666.59330.peg.4458	fig|6666666.59331.peg.5708	fig|6666666.59332.peg.3978	fig|6666666.59334.peg.464	fig|6666666.59335.peg.338	fig|6666666.59336.peg.1978	fig|6666666.59333.peg.5487	fig|6666666.59338.peg.338	fig|6666666.59339.peg.8335	fig|6666666.59340.peg.2945	fig|6666666.59337.peg.1727	fig|6666666.59342.peg.1448	fig|6666666.71181.peg.3407	fig|6666666.59344.peg.2209	fig|6666666.59345.peg.5357	fig|6666666.59341.peg.5977	fig|6666666.59347.peg.4914	fig|6666666.59346.peg.3211	fig|6666666.59348.peg.530	fig|6666666.59349.peg.1152	fig|6666666.59350.peg.1509	fig|6666666.59351.peg.55	fig|6666666.59353.peg.200	fig|6666666.59355.peg.2969	fig|6666666.59354.peg.2132

10395	Cytoplasmic axial filament protein CafA and Ribonuclease G EC 3.1.4.-	fig|6666666.59329.peg.710	fig|6666666.59330.peg.3719	fig|6666666.59331.peg.6382	fig|6666666.59332.peg.2168	fig|6666666.59334.peg.2738	fig|6666666.59335.peg.3030	fig|6666666.59336.peg.5711	fig|6666666.59333.peg.1601	fig|6666666.59338.peg.4840	fig|6666666.59339.peg.3164	fig|6666666.59340.peg.608	fig|6666666.59337.peg.813	fig|6666666.59342.peg.6091	fig|6666666.71181.peg.1613	fig|6666666.59344.peg.1746	fig|6666666.59345.peg.4881	fig|6666666.59341.peg.7	fig|6666666.59347.peg.4077	fig|6666666.59346.peg.2133	fig|6666666.59348.peg.5169	fig|6666666.59349.peg.8	fig|6666666.59350.peg.5720	fig|6666666.59351.peg.3425	fig|6666666.59353.peg.2296	fig|6666666.59355.peg.1344	fig|6666666.59354.peg.3169

6217	Aspartate aminotransferase EC 2.6.1.1	fig|6666666.59329.peg.4024	fig|6666666.59330.peg.308	fig|6666666.59331.peg.3529	fig|6666666.59332.peg.6244	fig|6666666.59334.peg.5078	fig|6666666.59335.peg.5460	fig|6666666.59336.peg.2082	fig|6666666.59333.peg.1424	fig|6666666.59338.peg.2207	fig|6666666.59339.peg.2020	fig|6666666.59340.peg.1753	fig|6666666.59337.peg.4869	fig|6666666.59342.peg.2823	fig|6666666.71181.peg.795	fig|6666666.59344.peg.311	fig|6666666.59345.peg.4150	fig|6666666.59341.peg.1224	fig|6666666.59347.peg.239	fig|6666666.59346.peg.2684	fig|6666666.59348.peg.4398	fig|6666666.59349.peg.3737	fig|6666666.59350.peg.3088	fig|6666666.59351.peg.2814	fig|6666666.59353.peg.3977	fig|6666666.59355.peg.3521	fig|6666666.59354.peg.3604

6811	Deoxyguanosinetriphosphate triphosphohydrolase EC 3.1.5.1	fig|6666666.59329.peg.5874	fig|6666666.59330.peg.305	fig|6666666.59331.peg.2080	fig|6666666.59332.peg.2211	fig|6666666.59334.peg.1417	fig|6666666.59335.peg.856	fig|6666666.59336.peg.6313	fig|6666666.59333.peg.4898	fig|6666666.59338.peg.1590	fig|6666666.59339.peg.3218	fig|6666666.59340.peg.3633	fig|6666666.59337.peg.1034	fig|6666666.59342.peg.5159	fig|6666666.71181.peg.1953	fig|6666666.59344.peg.1444	fig|6666666.59345.peg.4249	fig|6666666.59341.peg.3789	fig|6666666.59347.peg.2650	fig|6666666.59346.peg.2672	fig|6666666.59348.peg.4942	fig|6666666.59349.peg.4511	fig|6666666.59350.peg.7223	fig|6666666.59351.peg.3680	fig|6666666.59353.peg.166	fig|6666666.59355.peg.777	fig|6666666.59354.peg.6179

10571	2,4-dienoyl-CoA reductase [NADPH] EC 1.3.1.34	fig|6666666.59329.peg.2279	fig|6666666.59330.peg.4553	fig|6666666.59331.peg.3520	fig|6666666.59332.peg.3342	fig|6666666.59334.peg.289	fig|6666666.59335.peg.2027	fig|6666666.59336.peg.3183	fig|6666666.59333.peg.1080	fig|6666666.59338.peg.2205	fig|6666666.59339.peg.581	fig|6666666.59340.peg.3072	fig|6666666.59337.peg.3133	fig|6666666.59342.peg.1216	fig|6666666.71181.peg.4847	fig|6666666.59344.peg.2408	fig|6666666.59345.peg.366	fig|6666666.59341.peg.5703	fig|6666666.59347.peg.1434	fig|6666666.59346.peg.1042	fig|6666666.59348.peg.3680	fig|6666666.59349.peg.2652	fig|6666666.59350.peg.6584	fig|6666666.59351.peg.925	fig|6666666.59353.peg.290	fig|6666666.59355.peg.1330	fig|6666666.59354.peg.4355

9969	Cytosol aminopeptidase PepA EC 3.4.11.1	fig|6666666.59329.peg.1104	fig|6666666.59330.peg.2445	fig|6666666.59331.peg.2965	fig|6666666.59332.peg.2095	fig|6666666.59334.peg.4521	fig|6666666.59335.peg.253	fig|6666666.59336.peg.489	fig|6666666.59333.peg.2822	fig|6666666.59338.peg.3092	fig|6666666.59339.peg.8137	fig|6666666.59340.peg.1737	fig|6666666.59337.peg.4789	fig|6666666.59342.peg.1057	fig|6666666.71181.peg.1764	fig|6666666.59344.peg.1636	fig|6666666.59345.peg.4833	fig|6666666.59341.peg.3041	fig|6666666.59347.peg.3262	fig|6666666.59346.peg.1667	fig|6666666.59348.peg.2178	fig|6666666.59349.peg.2239	fig|6666666.59350.peg.4433	fig|6666666.59351.peg.889	fig|6666666.59353.peg.2443	fig|6666666.59355.peg.534	fig|6666666.59354.peg.51

251	Glycosyltransferase SypQ	fig|6666666.59329.peg.4071	fig|6666666.59330.peg.1337	fig|6666666.59331.peg.4521	fig|6666666.59332.peg.3337	fig|6666666.59334.peg.568	fig|6666666.59335.peg.526	fig|6666666.59336.peg.6017	fig|6666666.59333.peg.648	fig|6666666.59338.peg.255	fig|6666666.59339.peg.1150	fig|6666666.59340.peg.182	fig|6666666.59337.peg.5633	fig|6666666.59342.peg.4038	fig|6666666.71181.peg.4680	fig|6666666.59344.peg.792	fig|6666666.59345.peg.2599	fig|6666666.59341.peg.3698	fig|6666666.59347.peg.2342	fig|6666666.59346.peg.4450	fig|6666666.59348.peg.4285	fig|6666666.59349.peg.789	fig|6666666.59350.peg.7038	fig|6666666.59351.peg.1941	fig|6666666.59353.peg.1583	fig|6666666.59355.peg.4930	fig|6666666.59354.peg.4120

1159	Thiol peroxidase, Bcp-type EC 1.11.1.15	fig|6666666.59329.peg.3392	fig|6666666.59330.peg.3639	fig|6666666.59331.peg.1200	fig|6666666.59332.peg.4172	fig|6666666.59334.peg.1858	fig|6666666.59335.peg.1196	fig|6666666.59336.peg.2457	fig|6666666.59333.peg.4790	fig|6666666.59338.peg.786	fig|6666666.59339.peg.38	fig|6666666.59340.peg.2565	fig|6666666.59337.peg.5612	fig|6666666.59342.peg.2976	fig|6666666.71181.peg.3254	fig|6666666.59344.peg.4008	fig|6666666.59345.peg.991	fig|6666666.59341.peg.1663	fig|6666666.59347.peg.650	fig|6666666.59346.peg.2504	fig|6666666.59348.peg.1159	fig|6666666.59349.peg.1303	fig|6666666.59350.peg.4144	fig|6666666.59351.peg.491	fig|6666666.59353.peg.1226	fig|6666666.59355.peg.249	fig|6666666.59354.peg.1566

10430	Carbonic anhydrase EC 4.2.1.1	fig|6666666.59329.peg.1456	fig|6666666.59330.peg.4046	fig|6666666.59331.peg.6118	fig|6666666.59332.peg.2626	fig|6666666.59334.peg.4961	fig|6666666.59335.peg.2336	fig|6666666.59336.peg.4635	fig|6666666.59333.peg.141	fig|6666666.59338.peg.2966	fig|6666666.59339.peg.7977	fig|6666666.59340.peg.324	fig|6666666.59337.peg.2063	fig|6666666.59342.peg.4157	fig|6666666.71181.peg.2850	fig|6666666.59344.peg.4427	fig|6666666.59345.peg.2414	fig|6666666.59341.peg.4474	fig|6666666.59347.peg.3903	fig|6666666.59346.peg.4638	fig|6666666.59348.peg.5332	fig|6666666.59349.peg.1132	fig|6666666.59350.peg.304	fig|6666666.59351.peg.87	fig|6666666.59353.peg.3202	fig|6666666.59355.peg.3083	fig|6666666.59354.peg.4466

7231	ATP-dependent DNA helicase UvrD PcrA	fig|6666666.59329.peg.6409	fig|6666666.59330.peg.2134	fig|6666666.59331.peg.3358	fig|6666666.59332.peg.2863	fig|6666666.59334.peg.2767	fig|6666666.59335.peg.1124	fig|6666666.59336.peg.118	fig|6666666.59333.peg.1579	fig|6666666.59338.peg.4100	fig|6666666.59339.peg.7652	fig|6666666.59340.peg.127	fig|6666666.59337.peg.2777	fig|6666666.59342.peg.661	fig|6666666.71181.peg.2456	fig|6666666.59344.peg.2453	fig|6666666.59345.peg.2261	fig|6666666.59341.peg.4162	fig|6666666.59347.peg.2823	fig|6666666.59346.peg.920	fig|6666666.59348.peg.2193	fig|6666666.59349.peg.2648	fig|6666666.59350.peg.8224	fig|6666666.59351.peg.4828	fig|6666666.59353.peg.1700	fig|6666666.59355.peg.1453	fig|6666666.59354.peg.1846

819	O-acetylhomoserine sulfhydrylase EC 2.5.1.49 O-succinylhomoserine sulfhydrylase EC 2.5.1.48	fig|6666666.59329.peg.2907	fig|6666666.59330.peg.1672	fig|6666666.59331.peg.798	fig|6666666.59332.peg.4818	fig|6666666.59334.peg.76	fig|6666666.59335.peg.1975	fig|6666666.59336.peg.2891	fig|6666666.59333.peg.2001	fig|6666666.59338.peg.1053	fig|6666666.59339.peg.534	fig|6666666.59340.peg.211	fig|6666666.59337.peg.1675	fig|6666666.59342.peg.6426	fig|6666666.71181.peg.6055	fig|6666666.59344.peg.327	fig|6666666.59345.peg.1259	fig|6666666.59341.peg.2117	fig|6666666.59347.peg.2182	fig|6666666.59346.peg.1282	fig|6666666.59348.peg.3566	fig|6666666.59349.peg.2082	fig|6666666.59350.peg.3502	fig|6666666.59351.peg.2595	fig|6666666.59353.peg.4707	fig|6666666.59355.peg.2669	fig|6666666.59354.peg.923

11130	Ribosome-associated heat shock protein implicated in the recycling of the 50S subunit S4 paralog	fig|6666666.59329.peg.343	fig|6666666.59330.peg.415	fig|6666666.59331.peg.6023	fig|6666666.59332.peg.1677	fig|6666666.59334.peg.133	fig|6666666.59335.peg.722	fig|6666666.59336.peg.447	fig|6666666.59333.peg.501	fig|6666666.59338.peg.1798	fig|6666666.59339.peg.6193	fig|6666666.59340.peg.1433	fig|6666666.59337.peg.4240	fig|6666666.59342.peg.2913	fig|6666666.71181.peg.1842	fig|6666666.59344.peg.5476	fig|6666666.59345.peg.2679	fig|6666666.59341.peg.3430	fig|6666666.59347.peg.4461	fig|6666666.59346.peg.2598	fig|6666666.59348.peg.2078	fig|6666666.59349.peg.2177	fig|6666666.59350.peg.290	fig|6666666.59351.peg.4350	fig|6666666.59353.peg.2207	fig|6666666.59355.peg.1899	fig|6666666.59354.peg.693

9947	Transcription elongation factor GreA	fig|6666666.59329.peg.3483	fig|6666666.59330.peg.116	fig|6666666.59331.peg.5249	fig|6666666.59332.peg.4093	fig|6666666.59334.peg.2880	fig|6666666.59335.peg.705	fig|6666666.59336.peg.4515	fig|6666666.59333.peg.2902	fig|6666666.59338.peg.5978	fig|6666666.59339.peg.4855	fig|6666666.59340.peg.2161	fig|6666666.59337.peg.674	fig|6666666.59342.peg.345	fig|6666666.71181.peg.4089	fig|6666666.59344.peg.2109	fig|6666666.59345.peg.2658	fig|6666666.59341.peg.1743	fig|6666666.59347.peg.3013	fig|6666666.59346.peg.289	fig|6666666.59348.peg.4888	fig|6666666.59349.peg.2187	fig|6666666.59350.peg.6492	fig|6666666.59351.peg.3194	fig|6666666.59353.peg.3142	fig|6666666.59355.peg.454	fig|6666666.59354.peg.4042

231	Transporter, LysE family	fig|6666666.59329.peg.5040	fig|6666666.59330.peg.2103	fig|6666666.59331.peg.3474	fig|6666666.59332.peg.3952	fig|6666666.59334.peg.2138	fig|6666666.59335.peg.2151	fig|6666666.59336.peg.1422	fig|6666666.59333.peg.1440	fig|6666666.59338.peg.1037	fig|6666666.59339.peg.2372	fig|6666666.59340.peg.123	fig|6666666.59337.peg.5526	fig|6666666.59342.peg.5739	fig|6666666.71181.peg.2126	fig|6666666.59344.peg.6047	fig|6666666.59345.peg.2089	fig|6666666.59341.peg.470	fig|6666666.59347.peg.5066	fig|6666666.59346.peg.437	fig|6666666.59348.peg.4331	fig|6666666.59349.peg.2466	fig|6666666.59350.peg.462	fig|6666666.59351.peg.1925	fig|6666666.59353.peg.90	fig|6666666.59355.peg.4761	fig|6666666.59354.peg.1929

8740	Seryl-tRNA synthetase EC 6.1.1.11	fig|6666666.59329.peg.3405	fig|6666666.59330.peg.1930	fig|6666666.59331.peg.750	fig|6666666.59332.peg.5494	fig|6666666.59334.peg.2998	fig|6666666.59335.peg.3962	fig|6666666.59336.peg.2311	fig|6666666.59333.peg.2042	fig|6666666.59338.peg.3566	fig|6666666.59339.peg.3136	fig|6666666.59340.peg.2940	fig|6666666.59337.peg.4594	fig|6666666.59342.peg.2394	fig|6666666.71181.peg.3157	fig|6666666.59344.peg.2817	fig|6666666.59345.peg.1335	fig|6666666.59341.peg.6130	fig|6666666.59347.peg.2392	fig|6666666.59346.peg.1575	fig|6666666.59348.peg.4333	fig|6666666.59349.peg.1587	fig|6666666.59350.peg.7295	fig|6666666.59351.peg.1004	fig|6666666.59353.peg.1633	fig|6666666.59355.peg.1087	fig|6666666.59354.peg.2190

2536	Transcriptional regulator, AsnC family	fig|6666666.59329.peg.460	fig|6666666.59330.peg.1925	fig|6666666.59331.peg.3713	fig|6666666.59332.peg.1315	fig|6666666.59334.peg.3822	fig|6666666.59335.peg.1047	fig|6666666.59336.peg.2219	fig|6666666.59333.peg.1450	fig|6666666.59338.peg.5155	fig|6666666.59339.peg.3425	fig|6666666.59340.peg.1662	fig|6666666.59337.peg.4862	fig|6666666.59342.peg.3306	fig|6666666.71181.peg.3153	fig|6666666.59344.peg.2754	fig|6666666.59345.peg.1340	fig|6666666.59341.peg.4714	fig|6666666.59347.peg.2397	fig|6666666.59346.peg.2082	fig|6666666.59348.peg.4637	fig|6666666.59349.peg.401	fig|6666666.59350.peg.5414	fig|6666666.59351.peg.3277	fig|6666666.59353.peg.1629	fig|6666666.59355.peg.964	fig|6666666.59354.peg.2970

9560	Sensor histidine kinase	fig|6666666.59329.peg.2922	fig|6666666.59330.peg.2487	fig|6666666.59331.peg.5503	fig|6666666.59332.peg.2084	fig|6666666.59334.peg.2249	fig|6666666.59335.peg.6000	fig|6666666.59336.peg.5271	fig|6666666.59333.peg.1806	fig|6666666.59338.peg.3652	fig|6666666.59339.peg.4459	fig|6666666.59340.peg.2824	fig|6666666.59337.peg.4175	fig|6666666.59342.peg.1015	fig|6666666.71181.peg.2746	fig|6666666.59344.peg.1835	fig|6666666.59345.peg.1269	fig|6666666.59341.peg.1928	fig|6666666.59347.peg.620	fig|6666666.59346.peg.1743	fig|6666666.59348.peg.692	fig|6666666.59349.peg.3426	fig|6666666.59350.peg.3645	fig|6666666.59351.peg.2793	fig|6666666.59353.peg.1596	fig|6666666.59355.peg.4062	fig|6666666.59354.peg.536

793	Putative translation initiation inhibitor, yjgF family	fig|6666666.59329.peg.2273	fig|6666666.59330.peg.2625	fig|6666666.59331.peg.123	fig|6666666.59332.peg.6278	fig|6666666.59334.peg.3847	fig|6666666.59335.peg.2931	fig|6666666.59336.peg.4751	fig|6666666.59333.peg.1401	fig|6666666.59338.peg.4294	fig|6666666.59339.peg.784	fig|6666666.59340.peg.1836	fig|6666666.59337.peg.1480	fig|6666666.59342.peg.1582	fig|6666666.71181.peg.4699	fig|6666666.59344.peg.3317	fig|6666666.59345.peg.4017	fig|6666666.59341.peg.4194	fig|6666666.59347.peg.1518	fig|6666666.59346.peg.3729	fig|6666666.59348.peg.3228	fig|6666666.59349.peg.4092	fig|6666666.59350.peg.3302	fig|6666666.59351.peg.1772	fig|6666666.59353.peg.2763	fig|6666666.59355.peg.3679	fig|6666666.59354.peg.1996

1131	Oxidoreductase, Gfo Idh MocA family	fig|6666666.59329.peg.564	fig|6666666.59330.peg.1962	fig|6666666.59331.peg.4720	fig|6666666.59332.peg.5356	fig|6666666.59334.peg.2823	fig|6666666.59335.peg.5772	fig|6666666.59336.peg.2672	fig|6666666.59333.peg.3293	fig|6666666.59338.peg.4047	fig|6666666.59339.peg.3556	fig|6666666.59340.peg.3205	fig|6666666.59337.peg.5410	fig|6666666.59342.peg.751	fig|6666666.71181.peg.1098	fig|6666666.59344.peg.5229	fig|6666666.59345.peg.1926	fig|6666666.59341.peg.2542	fig|6666666.59347.peg.26	fig|6666666.59346.peg.4387	fig|6666666.59348.peg.2249	fig|6666666.59349.peg.626	fig|6666666.59350.peg.1974	fig|6666666.59351.peg.977	fig|6666666.59353.peg.1507	fig|6666666.59355.peg.1949	fig|6666666.59354.peg.5021

2886	Nitrite reductase [NAD P H] large subunit EC 1.7.1.4	fig|6666666.59329.peg.2968	fig|6666666.59330.peg.766	fig|6666666.59331.peg.170	fig|6666666.59332.peg.834	fig|6666666.59334.peg.5013	fig|6666666.59335.peg.4988	fig|6666666.59336.peg.2630	fig|6666666.59333.peg.3200	fig|6666666.59338.peg.5191	fig|6666666.59339.peg.4691	fig|6666666.59340.peg.1795	fig|6666666.59337.peg.5463	fig|6666666.59342.peg.6331	fig|6666666.71181.peg.3264	fig|6666666.59344.peg.3075	fig|6666666.59345.peg.43	fig|6666666.59341.peg.3397	fig|6666666.59347.peg.23	fig|6666666.59346.peg.2811	fig|6666666.59348.peg.482	fig|6666666.59349.peg.122	fig|6666666.59350.peg.3864	fig|6666666.59351.peg.2924	fig|6666666.59353.peg.5079	fig|6666666.59355.peg.6102	fig|6666666.59354.peg.2233

10454	D-3-phosphoglycerate dehydrogenase EC 1.1.1.95	fig|6666666.59329.peg.244	fig|6666666.59330.peg.5092	fig|6666666.59331.peg.2483	fig|6666666.59332.peg.2181	fig|6666666.59334.peg.4206	fig|6666666.59335.peg.5776	fig|6666666.59336.peg.1193	fig|6666666.59333.peg.4640	fig|6666666.59338.peg.4680	fig|6666666.59339.peg.8150	fig|6666666.59340.peg.224	fig|6666666.59337.peg.821	fig|6666666.59342.peg.2806	fig|6666666.71181.peg.1366	fig|6666666.59344.peg.5758	fig|6666666.59345.peg.3060	fig|6666666.59341.peg.897	fig|6666666.59347.peg.4662	fig|6666666.59346.peg.5049	fig|6666666.59348.peg.4322	fig|6666666.59349.peg.2602	fig|6666666.59350.peg.2573	fig|6666666.59351.peg.2142	fig|6666666.59353.peg.4127	fig|6666666.59355.peg.4514	fig|6666666.59354.peg.2488

1927	Probable low-affinity inorganic phosphate transporter	fig|6666666.59329.peg.381	fig|6666666.59330.peg.942	fig|6666666.59331.peg.2336	fig|6666666.59332.peg.5034	fig|6666666.59334.peg.535	fig|6666666.59335.peg.1510	fig|6666666.59336.peg.3435	fig|6666666.59333.peg.192	fig|6666666.59338.peg.213	fig|6666666.59339.peg.7398	fig|6666666.59340.peg.1092	fig|6666666.59337.peg.6	fig|6666666.59342.peg.1374	fig|6666666.71181.peg.4622	fig|6666666.59344.peg.4875	fig|6666666.59345.peg.3833	fig|6666666.59341.peg.1562	fig|6666666.59347.peg.3958	fig|6666666.59346.peg.3118	fig|6666666.59348.peg.1823	fig|6666666.59349.peg.3554	fig|6666666.59350.peg.7965	fig|6666666.59351.peg.2328	fig|6666666.59353.peg.3453	fig|6666666.59355.peg.4617	fig|6666666.59354.peg.1278

11248	Peptide transport system permease protein SapB	fig|6666666.59329.peg.970	fig|6666666.59330.peg.1184	fig|6666666.59331.peg.4051	fig|6666666.59332.peg.3807	fig|6666666.59334.peg.1126	fig|6666666.59335.peg.3752	fig|6666666.59336.peg.5495	fig|6666666.59333.peg.1325	fig|6666666.59338.peg.5553	fig|6666666.59339.peg.518	fig|6666666.59340.peg.1545	fig|6666666.59337.peg.2324	fig|6666666.59342.peg.5498	fig|6666666.71181.peg.6028	fig|6666666.59344.peg.4531	fig|6666666.59345.peg.2751	fig|6666666.59341.peg.1031	fig|6666666.59347.peg.1370	fig|6666666.59346.peg.1297	fig|6666666.59348.peg.1685	fig|6666666.59349.peg.2091	fig|6666666.59350.peg.6530	fig|6666666.59351.peg.2605	fig|6666666.59353.peg.638	fig|6666666.59355.peg.6126	fig|6666666.59354.peg.3392

5809	Signal recognition particle, subunit Ffh SRP54 TC 3.A.5.1.1	fig|6666666.59329.peg.1801	fig|6666666.59330.peg.2843	fig|6666666.59331.peg.5527	fig|6666666.59332.peg.2447	fig|6666666.59334.peg.265	fig|6666666.59335.peg.5390	fig|6666666.59336.peg.1281	fig|6666666.59333.peg.3180	fig|6666666.59338.peg.2854	fig|6666666.59339.peg.7930	fig|6666666.59340.peg.89	fig|6666666.59337.peg.2040	fig|6666666.59342.peg.2096	fig|6666666.71181.peg.3959	fig|6666666.59344.peg.3453	fig|6666666.59345.peg.5328	fig|6666666.59341.peg.1341	fig|6666666.59347.peg.2676	fig|6666666.59346.peg.567	fig|6666666.59348.peg.1774	fig|6666666.59349.peg.3789	fig|6666666.59350.peg.5150	fig|6666666.59351.peg.3395	fig|6666666.59353.peg.4815	fig|6666666.59355.peg.3362	fig|6666666.59354.peg.683

838	Sugar maltose fermentation stimulation protein homolog	fig|6666666.59329.peg.1902	fig|6666666.59330.peg.1759	fig|6666666.59331.peg.915	fig|6666666.59332.peg.6031	fig|6666666.59334.peg.824	fig|6666666.59335.peg.1517	fig|6666666.59336.peg.572	fig|6666666.59333.peg.314	fig|6666666.59338.peg.2828	fig|6666666.59339.peg.4773	fig|6666666.59340.peg.143	fig|6666666.59337.peg.626	fig|6666666.59342.peg.1568	fig|6666666.71181.peg.2837	fig|6666666.59344.peg.4443	fig|6666666.59345.peg.2402	fig|6666666.59341.peg.1550	fig|6666666.59347.peg.3969	fig|6666666.59346.peg.3825	fig|6666666.59348.peg.2949	fig|6666666.59349.peg.2478	fig|6666666.59350.peg.7937	fig|6666666.59351.peg.364	fig|6666666.59353.peg.3188	fig|6666666.59355.peg.3070	fig|6666666.59354.peg.50

1586	probable iron binding protein from the HesB IscA SufA family	fig|6666666.59329.peg.1109	fig|6666666.59330.peg.126	fig|6666666.59331.peg.4908	fig|6666666.59332.peg.1011	fig|6666666.59334.peg.2023	fig|6666666.59335.peg.4382	fig|6666666.59336.peg.241	fig|6666666.59333.peg.5479	fig|6666666.59338.peg.1324	fig|6666666.59339.peg.2175	fig|6666666.59340.peg.2650	fig|6666666.59337.peg.741	fig|6666666.59342.peg.4100	fig|6666666.71181.peg.1957	fig|6666666.59344.peg.1629	fig|6666666.59345.peg.2374	fig|6666666.59341.peg.1370	fig|6666666.59347.peg.2748	fig|6666666.59346.peg.279	fig|6666666.59348.peg.1377	fig|6666666.59349.peg.2433	fig|6666666.59350.peg.778	fig|6666666.59351.peg.2711	fig|6666666.59353.peg.162	fig|6666666.59355.peg.2518	fig|6666666.59354.peg.4570

9104	SSU ribosomal protein S8p S15Ae	fig|6666666.59329.peg.723	fig|6666666.59330.peg.564	fig|6666666.59331.peg.38	fig|6666666.59332.peg.2149	fig|6666666.59334.peg.4016	fig|6666666.59335.peg.4357	fig|6666666.59336.peg.156	fig|6666666.59333.peg.376	fig|6666666.59338.peg.1379	fig|6666666.59339.peg.8277	fig|6666666.59340.peg.66	fig|6666666.59337.peg.378	fig|6666666.59342.peg.314	fig|6666666.71181.peg.3447	fig|6666666.59344.peg.4930	fig|6666666.59345.peg.2525	fig|6666666.59341.peg.2508	fig|6666666.59347.peg.4833	fig|6666666.59346.peg.860	fig|6666666.59348.peg.1770	fig|6666666.59349.peg.3280	fig|6666666.59350.peg.3183	fig|6666666.59351.peg.4214	fig|6666666.59353.peg.4603	fig|6666666.59355.peg.4728	fig|6666666.59354.peg.3853

6411	Cold shock protein CspA	fig|6666666.59329.peg.4409	fig|6666666.59330.peg.4085	fig|6666666.59331.peg.6355	fig|6666666.59332.peg.2307	fig|6666666.59334.peg.4141	fig|6666666.59335.peg.4226	fig|6666666.59336.peg.861	fig|6666666.59333.peg.1010	fig|6666666.59338.peg.3774	fig|6666666.59339.peg.6514	fig|6666666.59340.peg.3472	fig|6666666.59337.peg.3965	fig|6666666.59342.peg.5065	fig|6666666.71181.peg.4189	fig|6666666.59344.peg.2577	fig|6666666.59345.peg.462	fig|6666666.59341.peg.1299	fig|6666666.59347.peg.1567	fig|6666666.59346.peg.4052	fig|6666666.59348.peg.2496	fig|6666666.59349.peg.4003	fig|6666666.59350.peg.2847	fig|6666666.59351.peg.1240	fig|6666666.59353.peg.1538	fig|6666666.59355.peg.2069	fig|6666666.59354.peg.3873

4786	Dihydrofolate synthase EC 6.3.2.12 Folylpolyglutamate synthase EC 6.3.2.17	fig|6666666.59329.peg.3492	fig|6666666.59330.peg.1601	fig|6666666.59331.peg.1904	fig|6666666.59332.peg.2752	fig|6666666.59334.peg.4402	fig|6666666.59335.peg.3591	fig|6666666.59336.peg.3038	fig|6666666.59333.peg.2543	fig|6666666.59338.peg.2034	fig|6666666.59339.peg.2973	fig|6666666.59340.peg.392	fig|6666666.59337.peg.5674	fig|6666666.59342.peg.4063	fig|6666666.71181.peg.1476	fig|6666666.59344.peg.1884	fig|6666666.59345.peg.1203	fig|6666666.59341.peg.2784	fig|6666666.59347.peg.2455	fig|6666666.59346.peg.54	fig|6666666.59348.peg.1715	fig|6666666.59349.peg.3460	fig|6666666.59350.peg.325	fig|6666666.59351.peg.3572	fig|6666666.59353.peg.1208	fig|6666666.59355.peg.268	fig|6666666.59354.peg.2963

5826	DNA primase EC 2.7.7.-	fig|6666666.59329.peg.680	fig|6666666.59330.peg.3132	fig|6666666.59331.peg.794	fig|6666666.59332.peg.3924	fig|6666666.59334.peg.2469	fig|6666666.59335.peg.1642	fig|6666666.59336.peg.5194	fig|6666666.59333.peg.171	fig|6666666.59338.peg.697	fig|6666666.59339.peg.7904	fig|6666666.59340.peg.1276	fig|6666666.59337.peg.1423	fig|6666666.59342.peg.1635	fig|6666666.71181.peg.3006	fig|6666666.59344.peg.4477	fig|6666666.59345.peg.3849	fig|6666666.59341.peg.3724	fig|6666666.59347.peg.3139	fig|6666666.59346.peg.2562	fig|6666666.59348.peg.2743	fig|6666666.59349.peg.1955	fig|6666666.59350.peg.3837	fig|6666666.59351.peg.2365	fig|6666666.59353.peg.4189	fig|6666666.59355.peg.1686	fig|6666666.59354.peg.11

11264	FIG000325 clustered with transcription termination protein NusA	fig|6666666.59329.peg.3445	fig|6666666.59330.peg.108	fig|6666666.59331.peg.2318	fig|6666666.59332.peg.6065	fig|6666666.59334.peg.5467	fig|6666666.59335.peg.2104	fig|6666666.59336.peg.2419	fig|6666666.59333.peg.2328	fig|6666666.59338.peg.1504	fig|6666666.59339.peg.4891	fig|6666666.59340.peg.3619	fig|6666666.59337.peg.682	fig|6666666.59342.peg.376	fig|6666666.71181.peg.4096	fig|6666666.59344.peg.2101	fig|6666666.59345.peg.2358	fig|6666666.59341.peg.2437	fig|6666666.59347.peg.3006	fig|6666666.59346.peg.297	fig|6666666.59348.peg.966	fig|6666666.59349.peg.1431	fig|6666666.59350.peg.7427	fig|6666666.59351.peg.1391	fig|6666666.59353.peg.3134	fig|6666666.59355.peg.462	fig|6666666.59354.peg.2272

6912	Transcriptional regulator, GntR family domain Aspartate aminotransferase EC 2.6.1.1	fig|6666666.59329.peg.2233	fig|6666666.59330.peg.512	fig|6666666.59331.peg.2604	fig|6666666.59332.peg.5185	fig|6666666.59334.peg.1354	fig|6666666.59335.peg.4720	fig|6666666.59336.peg.5500	fig|6666666.59333.peg.2929	fig|6666666.59338.peg.6065	fig|6666666.59339.peg.7011	fig|6666666.59340.peg.1453	fig|6666666.59337.peg.202	fig|6666666.59342.peg.3349	fig|6666666.71181.peg.4737	fig|6666666.59344.peg.4677	fig|6666666.59345.peg.524	fig|6666666.59341.peg.3326	fig|6666666.59347.peg.3647	fig|6666666.59346.peg.1273	fig|6666666.59348.peg.5364	fig|6666666.59349.peg.859	fig|6666666.59350.peg.5284	fig|6666666.59351.peg.3820	fig|6666666.59353.peg.1573	fig|6666666.59355.peg.4769	fig|6666666.59354.peg.945

7909	Acriflavin resistance protein	fig|6666666.59329.peg.1089	fig|6666666.59330.peg.1844	fig|6666666.59331.peg.5177	fig|6666666.59332.peg.4391	fig|6666666.59334.peg.3319	fig|6666666.59335.peg.5105	fig|6666666.59336.peg.2517	fig|6666666.59333.peg.4407	fig|6666666.59338.peg.1159	fig|6666666.59339.peg.6675	fig|6666666.59340.peg.661	fig|6666666.59337.peg.3547	fig|6666666.59342.peg.2728	fig|6666666.71181.peg.4365	fig|6666666.59344.peg.2913	fig|6666666.59345.peg.4001	fig|6666666.59341.peg.3891	fig|6666666.59347.peg.4732	fig|6666666.59346.peg.4014	fig|6666666.59348.peg.971	fig|6666666.59349.peg.1486	fig|6666666.59350.peg.7097	fig|6666666.59351.peg.2299	fig|6666666.59353.peg.4765	fig|6666666.59355.peg.5591	fig|6666666.59354.peg.6008

3454	Glutamate 5-kinase EC 2.7.2.11	fig|6666666.59329.peg.5830	fig|6666666.59330.peg.1064	fig|6666666.59331.peg.5884	fig|6666666.59332.peg.5764	fig|6666666.59334.peg.1666	fig|6666666.59335.peg.147	fig|6666666.59336.peg.2494	fig|6666666.59333.peg.3422	fig|6666666.59338.peg.4162	fig|6666666.59339.peg.5088	fig|6666666.59340.peg.2085	fig|6666666.59337.peg.1144	fig|6666666.59342.peg.5145	fig|6666666.71181.peg.931	fig|6666666.59344.peg.3533	fig|6666666.59345.peg.3545	fig|6666666.59341.peg.2602	fig|6666666.59347.peg.2779	fig|6666666.59346.peg.3376	fig|6666666.59348.peg.4349	fig|6666666.59349.peg.4674	fig|6666666.59350.peg.2556	fig|6666666.59351.peg.3102	fig|6666666.59353.peg.883	fig|6666666.59355.peg.821	fig|6666666.59354.peg.2653

1300	S-formylglutathione hydrolase EC 3.1.2.12	fig|6666666.59329.peg.470	fig|6666666.59330.peg.1607	fig|6666666.59331.peg.2404	fig|6666666.59332.peg.5294	fig|6666666.59334.peg.5590	fig|6666666.59335.peg.5503	fig|6666666.59336.peg.2577	fig|6666666.59333.peg.3198	fig|6666666.59338.peg.5065	fig|6666666.59339.peg.2388	fig|6666666.59340.peg.290	fig|6666666.59337.peg.4900	fig|6666666.59342.peg.3676	fig|6666666.71181.peg.186	fig|6666666.59344.peg.225	fig|6666666.59345.peg.4486	fig|6666666.59341.peg.4061	fig|6666666.59347.peg.4340	fig|6666666.59346.peg.3367	fig|6666666.59348.peg.1064	fig|6666666.59349.peg.4699	fig|6666666.59350.peg.6137	fig|6666666.59351.peg.846	fig|6666666.59353.peg.636	fig|6666666.59355.peg.119	fig|6666666.59354.peg.3822

6474	Transcriptional regulator, LacI family	fig|6666666.59329.peg.805	fig|6666666.59330.peg.2021	fig|6666666.59331.peg.1826	fig|6666666.59332.peg.3861	fig|6666666.59334.peg.306	fig|6666666.59335.peg.5753	fig|6666666.59336.peg.548	fig|6666666.59333.peg.1183	fig|6666666.59338.peg.4402	fig|6666666.59339.peg.759	fig|6666666.59340.peg.1007	fig|6666666.59337.peg.1104	fig|6666666.59342.peg.2510	fig|6666666.71181.peg.5730	fig|6666666.59344.peg.3518	fig|6666666.59345.peg.4503	fig|6666666.59341.peg.2850	fig|6666666.59347.peg.2378	fig|6666666.59346.peg.4535	fig|6666666.59348.peg.3467	fig|6666666.59349.peg.4713	fig|6666666.59350.peg.1895	fig|6666666.59351.peg.4413	fig|6666666.59353.peg.2804	fig|6666666.59355.peg.3718	fig|6666666.59354.peg.3418

3932	Phenylalanyl-tRNA synthetase beta chain EC 6.1.1.20	fig|6666666.59329.peg.1469	fig|6666666.59330.peg.2812	fig|6666666.59331.peg.1688	fig|6666666.59332.peg.2117	fig|6666666.59334.peg.4126	fig|6666666.59335.peg.3688	fig|6666666.59336.peg.6101	fig|6666666.59333.peg.535	fig|6666666.59338.peg.3866	fig|6666666.59339.peg.159	fig|6666666.59340.peg.342	fig|6666666.59337.peg.4791	fig|6666666.59342.peg.3635	fig|6666666.71181.peg.3299	fig|6666666.59344.peg.3973	fig|6666666.59345.peg.4652	fig|6666666.59341.peg.449	fig|6666666.59347.peg.1877	fig|6666666.59346.peg.1671	fig|6666666.59348.peg.1288	fig|6666666.59349.peg.3583	fig|6666666.59350.peg.3333	fig|6666666.59351.peg.4428	fig|6666666.59353.peg.4417	fig|6666666.59355.peg.684	fig|6666666.59354.peg.4282

3665	Branched-chain amino acid aminotransferase EC 2.6.1.42	fig|6666666.59329.peg.1486	fig|6666666.59330.peg.3627	fig|6666666.59331.peg.4778	fig|6666666.59332.peg.115	fig|6666666.59334.peg.4	fig|6666666.59335.peg.4367	fig|6666666.59336.peg.3337	fig|6666666.59333.peg.12	fig|6666666.59338.peg.4880	fig|6666666.59339.peg.3181	fig|6666666.59340.peg.385	fig|6666666.59337.peg.2684	fig|6666666.59342.peg.6187	fig|6666666.71181.peg.3089	fig|6666666.59344.peg.5037	fig|6666666.59345.peg.4947	fig|6666666.59341.peg.19	fig|6666666.59347.peg.4767	fig|6666666.59346.peg.110	fig|6666666.59348.peg.71	fig|6666666.59349.peg.1126	fig|6666666.59350.peg.4304	fig|6666666.59351.peg.81	fig|6666666.59353.peg.4266	fig|6666666.59355.peg.1357	fig|6666666.59354.peg.2161

3592	Magnesium and cobalt efflux protein CorC	fig|6666666.59329.peg.5002	fig|6666666.59330.peg.4037	fig|6666666.59331.peg.5436	fig|6666666.59332.peg.4720	fig|6666666.59334.peg.261	fig|6666666.59335.peg.1745	fig|6666666.59336.peg.2831	fig|6666666.59333.peg.2191	fig|6666666.59338.peg.4690	fig|6666666.59339.peg.5219	fig|6666666.59340.peg.2707	fig|6666666.59337.peg.4507	fig|6666666.59342.peg.2093	fig|6666666.71181.peg.3961	fig|6666666.59344.peg.4512	fig|6666666.59345.peg.5692	fig|6666666.59341.peg.1422	fig|6666666.59347.peg.1969	fig|6666666.59346.peg.3611	fig|6666666.59348.peg.5409	fig|6666666.59349.peg.599	fig|6666666.59350.peg.5294	fig|6666666.59351.peg.1740	fig|6666666.59353.peg.4817	fig|6666666.59355.peg.2079	fig|6666666.59354.peg.3235

4431	tolB protein precursor, periplasmic protein involved in the tonb-independent uptake of group A colicins	fig|6666666.59329.peg.1621	fig|6666666.59330.peg.3436	fig|6666666.59331.peg.4299	fig|6666666.59332.peg.5599	fig|6666666.59334.peg.2369	fig|6666666.59335.peg.2510	fig|6666666.59336.peg.2587	fig|6666666.59333.peg.1990	fig|6666666.59338.peg.4117	fig|6666666.59339.peg.399	fig|6666666.59340.peg.1633	fig|6666666.59337.peg.4379	fig|6666666.59342.peg.2249	fig|6666666.71181.peg.5007	fig|6666666.59344.peg.4572	fig|6666666.59345.peg.1506	fig|6666666.59341.peg.4883	fig|6666666.59347.peg.4209	fig|6666666.59346.peg.1441	fig|6666666.59348.peg.5115	fig|6666666.59349.peg.3861	fig|6666666.59350.peg.8323	fig|6666666.59351.peg.659	fig|6666666.59353.peg.2101	fig|6666666.59355.peg.2721	fig|6666666.59354.peg.1599

976	Rod shape-determining protein RodA	fig|6666666.59329.peg.2834	fig|6666666.59330.peg.3019	fig|6666666.59331.peg.984	fig|6666666.59332.peg.3006	fig|6666666.59334.peg.2319	fig|6666666.59335.peg.1757	fig|6666666.59336.peg.581	fig|6666666.59333.peg.5016	fig|6666666.59338.peg.4975	fig|6666666.59339.peg.4970	fig|6666666.59340.peg.403	fig|6666666.59337.peg.1089	fig|6666666.59342.peg.3060	fig|6666666.71181.peg.4760	fig|6666666.59344.peg.1480	fig|6666666.59345.peg.3495	fig|6666666.59341.peg.4751	fig|6666666.59347.peg.2732	fig|6666666.59346.peg.1239	fig|6666666.59348.peg.2549	fig|6666666.59349.peg.2765	fig|6666666.59350.peg.3070	fig|6666666.59351.peg.3622	fig|6666666.59353.peg.2019	fig|6666666.59355.peg.4426	fig|6666666.59354.peg.5711

974	Probable L-ascorbate-6-phosphate lactonase UlaG EC 3.1.1.- L-ascorbate utilization protein G	fig|6666666.59329.peg.1204	fig|6666666.59330.peg.1630	fig|6666666.59331.peg.5171	fig|6666666.59332.peg.3651	fig|6666666.59334.peg.2510	fig|6666666.59335.peg.2304	fig|6666666.59336.peg.293	fig|6666666.59333.peg.5130	fig|6666666.59338.peg.139	fig|6666666.59339.peg.6846	fig|6666666.59340.peg.2666	fig|6666666.59337.peg.1271	fig|6666666.59342.peg.1762	fig|6666666.71181.peg.1297	fig|6666666.59344.peg.2957	fig|6666666.59345.peg.3027	fig|6666666.59341.peg.1373	fig|6666666.59347.peg.3586	fig|6666666.59346.peg.5016	fig|6666666.59348.peg.4033	fig|6666666.59349.peg.4403	fig|6666666.59350.peg.1410	fig|6666666.59351.peg.4040	fig|6666666.59353.peg.3301	fig|6666666.59355.peg.3151	fig|6666666.59354.peg.2667

7101	N-acetylmuramoyl-L-alanine amidase EC 3.5.1.28	fig|6666666.59329.peg.699	fig|6666666.59330.peg.3557	fig|6666666.59331.peg.4628	fig|6666666.59332.peg.1976	fig|6666666.59334.peg.1636	fig|6666666.59335.peg.4128	fig|6666666.59336.peg.3755	fig|6666666.59333.peg.283	fig|6666666.59338.peg.3083	fig|6666666.59339.peg.8855	fig|6666666.59340.peg.102	fig|6666666.59337.peg.1779	fig|6666666.59342.peg.5106	fig|6666666.71181.peg.5248	fig|6666666.59344.peg.6251	fig|6666666.59345.peg.5667	fig|6666666.59341.peg.3765	fig|6666666.59347.peg.5350	fig|6666666.59346.peg.3044	fig|6666666.59348.peg.1940	fig|6666666.59349.peg.3080	fig|6666666.59350.peg.4250	fig|6666666.59351.peg.3072	fig|6666666.59353.peg.1068	fig|6666666.59355.peg.1449	fig|6666666.59354.peg.82

1466	sodium-solute symporter, putative	fig|6666666.59329.peg.2272	fig|6666666.59330.peg.182	fig|6666666.59331.peg.864	fig|6666666.59332.peg.5505	fig|6666666.59334.peg.1023	fig|6666666.59335.peg.457	fig|6666666.59336.peg.1471	fig|6666666.59333.peg.1063	fig|6666666.59338.peg.6056	fig|6666666.59339.peg.8357	fig|6666666.59340.peg.3388	fig|6666666.59337.peg.279	fig|6666666.59342.peg.5134	fig|6666666.71181.peg.3765	fig|6666666.59344.peg.5192	fig|6666666.59345.peg.848	fig|6666666.59341.peg.1166	fig|6666666.59347.peg.3404	fig|6666666.59346.peg.3070	fig|6666666.59348.peg.3735	fig|6666666.59349.peg.4093	fig|6666666.59350.peg.8290	fig|6666666.59351.peg.2098	fig|6666666.59353.peg.919	fig|6666666.59355.peg.48	fig|6666666.59354.peg.1176

10786	1-acyl-sn-glycerol-3-phosphate acyltransferase EC 2.3.1.51	fig|6666666.59329.peg.1448	fig|6666666.59330.peg.59	fig|6666666.59331.peg.6094	fig|6666666.59332.peg.1281	fig|6666666.59334.peg.82	fig|6666666.59335.peg.1978	fig|6666666.59336.peg.1797	fig|6666666.59333.peg.278	fig|6666666.59338.peg.121	fig|6666666.59339.peg.7032	fig|6666666.59340.peg.836	fig|6666666.59337.peg.846	fig|6666666.59342.peg.435	fig|6666666.71181.peg.3652	fig|6666666.59344.peg.5609	fig|6666666.59345.peg.4848	fig|6666666.59341.peg.2694	fig|6666666.59347.peg.3238	fig|6666666.59346.peg.708	fig|6666666.59348.peg.2326	fig|6666666.59349.peg.3784	fig|6666666.59350.peg.2322	fig|6666666.59351.peg.3391	fig|6666666.59353.peg.4930	fig|6666666.59355.peg.4974	fig|6666666.59354.peg.25

2294	DNA topoisomerase III EC 5.99.1.2	fig|6666666.59329.peg.71	fig|6666666.59330.peg.1206	fig|6666666.59331.peg.6371	fig|6666666.59332.peg.3140	fig|6666666.59334.peg.1512	fig|6666666.59335.peg.1450	fig|6666666.59336.peg.3180	fig|6666666.59333.peg.1368	fig|6666666.59338.peg.1408	fig|6666666.59339.peg.177	fig|6666666.59340.peg.1123	fig|6666666.59337.peg.3362	fig|6666666.59342.peg.125	fig|6666666.71181.peg.3427	fig|6666666.59344.peg.4908	fig|6666666.59345.peg.1156	fig|6666666.59341.peg.1116	fig|6666666.59347.peg.4119	fig|6666666.59346.peg.1829	fig|6666666.59348.peg.5271	fig|6666666.59349.peg.3890	fig|6666666.59350.peg.3318	fig|6666666.59351.peg.1375	fig|6666666.59353.peg.973	fig|6666666.59355.peg.2403	fig|6666666.59354.peg.4387

2748	Serine hydroxymethyltransferase EC 2.1.2.1	fig|6666666.59329.peg.5059	fig|6666666.59330.peg.3583	fig|6666666.59331.peg.2997	fig|6666666.59332.peg.5546	fig|6666666.59334.peg.1730	fig|6666666.59335.peg.1765	fig|6666666.59336.peg.589	fig|6666666.59333.peg.4943	fig|6666666.59338.peg.1	fig|6666666.59339.peg.5192	fig|6666666.59340.peg.2382	fig|6666666.59337.peg.1097	fig|6666666.59342.peg.2898	fig|6666666.71181.peg.1749	fig|6666666.59344.peg.1617	fig|6666666.59345.peg.3503	fig|6666666.59341.peg.436	fig|6666666.59347.peg.2913	fig|6666666.59346.peg.5244	fig|6666666.59348.peg.4760	fig|6666666.59349.peg.3478	fig|6666666.59350.peg.4039	fig|6666666.59351.peg.4255	fig|6666666.59353.peg.2686	fig|6666666.59355.peg.1793	fig|6666666.59354.peg.2934

5202	Putative inner membrane protein	fig|6666666.59329.peg.2415	fig|6666666.59330.peg.1587	fig|6666666.59331.peg.6222	fig|6666666.59332.peg.1950	fig|6666666.59334.peg.1140	fig|6666666.59335.peg.5129	fig|6666666.59336.peg.4513	fig|6666666.59333.peg.1967	fig|6666666.59338.peg.1285	fig|6666666.59339.peg.7419	fig|6666666.59340.peg.288	fig|6666666.59337.peg.67	fig|6666666.59342.peg.3272	fig|6666666.71181.peg.1956	fig|6666666.59344.peg.1448	fig|6666666.59345.peg.3776	fig|6666666.59341.peg.838	fig|6666666.59347.peg.4015	fig|6666666.59346.peg.2654	fig|6666666.59348.peg.217	fig|6666666.59349.peg.4054	fig|6666666.59350.peg.1371	fig|6666666.59351.peg.3397	fig|6666666.59353.peg.2604	fig|6666666.59355.peg.2598	fig|6666666.59354.peg.5363

3950	Multi antimicrobial extrusion protein Na + drug antiporter , MATE family of MDR efflux pumps	fig|6666666.59329.peg.4210	fig|6666666.59330.peg.3654	fig|6666666.59331.peg.2006	fig|6666666.59332.peg.6130	fig|6666666.59334.peg.5346	fig|6666666.59335.peg.4041	fig|6666666.59336.peg.4730	fig|6666666.59333.peg.5086	fig|6666666.59338.peg.3348	fig|6666666.59339.peg.8589	fig|6666666.59340.peg.1469	fig|6666666.59337.peg.1662	fig|6666666.59342.peg.5076	fig|6666666.71181.peg.3686	fig|6666666.59344.peg.3298	fig|6666666.59345.peg.2054	fig|6666666.59341.peg.2447	fig|6666666.59347.peg.646	fig|6666666.59346.peg.1210	fig|6666666.59348.peg.4280	fig|6666666.59349.peg.4343	fig|6666666.59350.peg.71	fig|6666666.59351.peg.5082	fig|6666666.59353.peg.2997	fig|6666666.59355.peg.2903	fig|6666666.59354.peg.4874

959	Periplasmic hemin-binding protein	fig|6666666.59329.peg.2767	fig|6666666.59330.peg.1294	fig|6666666.59331.peg.3301	fig|6666666.59332.peg.4375	fig|6666666.59334.peg.1574	fig|6666666.59335.peg.3631	fig|6666666.59336.peg.5482	fig|6666666.59333.peg.1502	fig|6666666.59338.peg.3295	fig|6666666.59339.peg.4290	fig|6666666.59340.peg.287	fig|6666666.59337.peg.68	fig|6666666.59342.peg.3589	fig|6666666.71181.peg.1955	fig|6666666.59344.peg.1447	fig|6666666.59345.peg.4549	fig|6666666.59341.peg.5935	fig|6666666.59347.peg.642	fig|6666666.59346.peg.3031	fig|6666666.59348.peg.1083	fig|6666666.59349.peg.2552	fig|6666666.59350.peg.884	fig|6666666.59351.peg.3913	fig|6666666.59353.peg.1330	fig|6666666.59355.peg.2541	fig|6666666.59354.peg.2753

950	Exodeoxyribonuclease V alpha chain EC 3.1.11.5	fig|6666666.59329.peg.3257	fig|6666666.59330.peg.2138	fig|6666666.59331.peg.4012	fig|6666666.59332.peg.5856	fig|6666666.59334.peg.1533	fig|6666666.59335.peg.1122	fig|6666666.59336.peg.121	fig|6666666.59333.peg.4489	fig|6666666.59338.peg.5819	fig|6666666.59339.peg.6953	fig|6666666.59340.peg.1524	fig|6666666.59337.peg.4659	fig|6666666.59342.peg.6424	fig|6666666.71181.peg.5473	fig|6666666.59344.peg.2612	fig|6666666.59345.peg.2260	fig|6666666.59341.peg.526	fig|6666666.59347.peg.2822	fig|6666666.59346.peg.1841	fig|6666666.59348.peg.300	fig|6666666.59349.peg.2645	fig|6666666.59350.peg.5827	fig|6666666.59351.peg.5189	fig|6666666.59353.peg.1956	fig|6666666.59355.peg.1451	fig|6666666.59354.peg.4721

952	Glycosyltransferase	fig|6666666.59329.peg.543	fig|6666666.59330.peg.3474	fig|6666666.59331.peg.2859	fig|6666666.59332.peg.5476	fig|6666666.59334.peg.5410	fig|6666666.59335.peg.2727	fig|6666666.59336.peg.3874	fig|6666666.59333.peg.984	fig|6666666.59338.peg.1849	fig|6666666.59339.peg.1166	fig|6666666.59340.peg.2292	fig|6666666.59337.peg.3007	fig|6666666.59342.peg.259	fig|6666666.71181.peg.240	fig|6666666.59344.peg.298	fig|6666666.59345.peg.3131	fig|6666666.59341.peg.2487	fig|6666666.59347.peg.5019	fig|6666666.59346.peg.4452	fig|6666666.59348.peg.4828	fig|6666666.59349.peg.251	fig|6666666.59350.peg.6357	fig|6666666.59351.peg.235	fig|6666666.59353.peg.579	fig|6666666.59355.peg.4301	fig|6666666.59354.peg.2249

957	Peptide chain release factor 2; programmed frameshift-containing	fig|6666666.59329.peg.215	fig|6666666.59330.peg.1743	fig|6666666.59331.peg.3607	fig|6666666.59332.peg.2621	fig|6666666.59334.peg.3495	fig|6666666.59335.peg.1179	fig|6666666.59336.peg.3155	fig|6666666.59333.peg.232	fig|6666666.59338.peg.198	fig|6666666.59339.peg.4877	fig|6666666.59340.peg.2794	fig|6666666.59337.peg.111	fig|6666666.59342.peg.462	fig|6666666.71181.peg.5102	fig|6666666.59344.peg.3671	fig|6666666.59345.peg.5417	fig|6666666.59341.peg.3044	fig|6666666.59347.peg.3042	fig|6666666.59346.peg.3102	fig|6666666.59348.peg.609	fig|6666666.59349.peg.2615	fig|6666666.59350.peg.1160	fig|6666666.59351.peg.4662	fig|6666666.59353.peg.432	fig|6666666.59355.peg.1543	fig|6666666.59354.peg.2782

593	Succinate dehydrogenase iron-sulfur protein EC 1.3.99.1	fig|6666666.59329.peg.160	fig|6666666.59330.peg.153	fig|6666666.59331.peg.74	fig|6666666.59332.peg.655	fig|6666666.59334.peg.3645	fig|6666666.59335.peg.2744	fig|6666666.59336.peg.261	fig|6666666.59333.peg.900	fig|6666666.59338.peg.812	fig|6666666.59339.peg.7192	fig|6666666.59340.peg.3441	fig|6666666.59337.peg.4109	fig|6666666.59342.peg.476	fig|6666666.71181.peg.3235	fig|6666666.59344.peg.4595	fig|6666666.59345.peg.5105	fig|6666666.59341.peg.1580	fig|6666666.59347.peg.4557	fig|6666666.59346.peg.971	fig|6666666.59348.peg.2396	fig|6666666.59349.peg.2520	fig|6666666.59350.peg.2654	fig|6666666.59351.peg.3726	fig|6666666.59353.peg.2086	fig|6666666.59355.peg.2739	fig|6666666.59354.peg.1309

2820	Cell wall endopeptidase, family M23 M37	fig|6666666.59329.peg.2553	fig|6666666.59330.peg.4012	fig|6666666.59331.peg.3331	fig|6666666.59332.peg.1726	fig|6666666.59334.peg.4569	fig|6666666.59335.peg.4556	fig|6666666.59336.peg.849	fig|6666666.59333.peg.4067	fig|6666666.59338.peg.593	fig|6666666.59339.peg.2685	fig|6666666.59340.peg.938	fig|6666666.59337.peg.668	fig|6666666.59342.peg.532	fig|6666666.71181.peg.138	fig|6666666.59344.peg.2192	fig|6666666.59345.peg.2370	fig|6666666.59341.peg.3069	fig|6666666.59347.peg.4047	fig|6666666.59346.peg.655	fig|6666666.59348.peg.1130	fig|6666666.59349.peg.4012	fig|6666666.59350.peg.1447	fig|6666666.59351.peg.1423	fig|6666666.59353.peg.1482	fig|6666666.59355.peg.2032	fig|6666666.59354.peg.3399

6109	5-Enolpyruvylshikimate-3-phosphate synthase EC 2.5.1.19	fig|6666666.59329.peg.2564	fig|6666666.59330.peg.1326	fig|6666666.59331.peg.4274	fig|6666666.59332.peg.1280	fig|6666666.59334.peg.878	fig|6666666.59335.peg.220	fig|6666666.59336.peg.868	fig|6666666.59333.peg.2553	fig|6666666.59338.peg.5483	fig|6666666.59339.peg.291	fig|6666666.59340.peg.837	fig|6666666.59337.peg.3366	fig|6666666.59342.peg.123	fig|6666666.71181.peg.1030	fig|6666666.59344.peg.1810	fig|6666666.59345.peg.1549	fig|6666666.59341.peg.1001	fig|6666666.59347.peg.3239	fig|6666666.59346.peg.2528	fig|6666666.59348.peg.2324	fig|6666666.59349.peg.379	fig|6666666.59350.peg.3400	fig|6666666.59351.peg.940	fig|6666666.59353.peg.1645	fig|6666666.59355.peg.1622	fig|6666666.59354.peg.1756

6100	RNA polymerase sigma factor RpoH	fig|6666666.59329.peg.772	fig|6666666.59330.peg.1567	fig|6666666.59331.peg.4818	fig|6666666.59332.peg.2373	fig|6666666.59334.peg.1294	fig|6666666.59335.peg.4268	fig|6666666.59336.peg.1376	fig|6666666.59333.peg.1952	fig|6666666.59338.peg.528	fig|6666666.59339.peg.8570	fig|6666666.59340.peg.1437	fig|6666666.59337.peg.1651	fig|6666666.59342.peg.2200	fig|6666666.71181.peg.4531	fig|6666666.59344.peg.3449	fig|6666666.59345.peg.5469	fig|6666666.59341.peg.5287	fig|6666666.59347.peg.5053	fig|6666666.59346.peg.1640	fig|6666666.59348.peg.585	fig|6666666.59349.peg.3785	fig|6666666.59350.peg.1703	fig|6666666.59351.peg.3392	fig|6666666.59353.peg.3770	fig|6666666.59355.peg.3365	fig|6666666.59354.peg.24

3419	Phosphomannomutase EC 5.4.2.8	fig|6666666.59329.peg.849	fig|6666666.59330.peg.3225	fig|6666666.59331.peg.5916	fig|6666666.59332.peg.241	fig|6666666.59334.peg.4820	fig|6666666.59335.peg.2734	fig|6666666.59336.peg.336	fig|6666666.59333.peg.5529	fig|6666666.59338.peg.5409	fig|6666666.59339.peg.6647	fig|6666666.59340.peg.1251	fig|6666666.59337.peg.679	fig|6666666.59342.peg.2208	fig|6666666.71181.peg.3814	fig|6666666.59344.peg.2103	fig|6666666.59345.peg.5611	fig|6666666.59341.peg.2883	fig|6666666.59347.peg.3152	fig|6666666.59346.peg.2012	fig|6666666.59348.peg.1544	fig|6666666.59349.peg.1810	fig|6666666.59350.peg.2895	fig|6666666.59351.peg.1110	fig|6666666.59353.peg.586	fig|6666666.59355.peg.69	fig|6666666.59354.peg.1197

1349	Quinolinate phosphoribosyltransferase [decarboxylating] EC 2.4.2.19	fig|6666666.59329.peg.6558	fig|6666666.59330.peg.3500	fig|6666666.59331.peg.3818	fig|6666666.59332.peg.304	fig|6666666.59334.peg.478	fig|6666666.59335.peg.3812	fig|6666666.59336.peg.1434	fig|6666666.59333.peg.150	fig|6666666.59338.peg.922	fig|6666666.59339.peg.7952	fig|6666666.59340.peg.306	fig|6666666.59337.peg.2055	fig|6666666.59342.peg.1873	fig|6666666.71181.peg.1962	fig|6666666.59344.peg.1455	fig|6666666.59345.peg.2422	fig|6666666.59341.peg.5729	fig|6666666.59347.peg.3895	fig|6666666.59346.peg.583	fig|6666666.59348.peg.4576	fig|6666666.59349.peg.2631	fig|6666666.59350.peg.408	fig|6666666.59351.peg.279	fig|6666666.59353.peg.157	fig|6666666.59355.peg.768	fig|6666666.59354.peg.6062

3978	Uncharacterized protein	fig|6666666.59329.peg.5702	fig|6666666.59330.peg.4640	fig|6666666.59331.peg.2129	fig|6666666.59332.peg.5779	fig|6666666.59334.peg.4762	fig|6666666.59335.peg.2275	fig|6666666.59336.peg.5067	fig|6666666.59333.peg.4507	fig|6666666.59338.peg.5525	fig|6666666.59339.peg.2653	fig|6666666.59340.peg.2933	fig|6666666.59337.peg.2485	fig|6666666.59342.peg.1682	fig|6666666.71181.peg.2571	fig|6666666.59344.peg.757	fig|6666666.59345.peg.110	fig|6666666.59341.peg.3247	fig|6666666.59347.peg.831	fig|6666666.59346.peg.2909	fig|6666666.59348.peg.2545	fig|6666666.59349.peg.1166	fig|6666666.59350.peg.4028	fig|6666666.59351.peg.60	fig|6666666.59353.peg.1345	fig|6666666.59355.peg.2527	fig|6666666.59354.peg.5523

9459	Peptidyl-prolyl cis-trans isomerase ppiD EC 5.2.1.8	fig|6666666.59329.peg.5402	fig|6666666.59330.peg.301	fig|6666666.59331.peg.2039	fig|6666666.59332.peg.2215	fig|6666666.59334.peg.1423	fig|6666666.59335.peg.3365	fig|6666666.59336.peg.2652	fig|6666666.59333.peg.4903	fig|6666666.59338.peg.906	fig|6666666.59339.peg.3106	fig|6666666.59340.peg.1028	fig|6666666.59337.peg.1386	fig|6666666.59342.peg.2174	fig|6666666.71181.peg.2056	fig|6666666.59344.peg.1524	fig|6666666.59345.peg.4253	fig|6666666.59341.peg.3785	fig|6666666.59347.peg.3087	fig|6666666.59346.peg.2665	fig|6666666.59348.peg.3251	fig|6666666.59349.peg.1878	fig|6666666.59350.peg.2946	fig|6666666.59351.peg.2611	fig|6666666.59353.peg.749	fig|6666666.59355.peg.955	fig|6666666.59354.peg.135

9651	Uncharacterized ABC transporter, periplasmic component YrbD	fig|6666666.59329.peg.5290	fig|6666666.59330.peg.53	fig|6666666.59331.peg.5101	fig|6666666.59332.peg.1276	fig|6666666.59334.peg.89	fig|6666666.59335.peg.216	fig|6666666.59336.peg.357	fig|6666666.59333.peg.272	fig|6666666.59338.peg.116	fig|6666666.59339.peg.8192	fig|6666666.59340.peg.260	fig|6666666.59337.peg.840	fig|6666666.59342.peg.442	fig|6666666.71181.peg.1026	fig|6666666.59344.peg.1806	fig|6666666.59345.peg.4853	fig|6666666.59341.peg.6090	fig|6666666.59347.peg.3243	fig|6666666.59346.peg.631	fig|6666666.59348.peg.3524	fig|6666666.59349.peg.29	fig|6666666.59350.peg.3305	fig|6666666.59351.peg.3450	fig|6666666.59353.peg.2345	fig|6666666.59355.peg.1618	fig|6666666.59354.peg.345

9304	RNA-binding protein Hfq	fig|6666666.59329.peg.694	fig|6666666.59330.peg.3560	fig|6666666.59331.peg.1282	fig|6666666.59332.peg.1981	fig|6666666.59334.peg.3062	fig|6666666.59335.peg.4131	fig|6666666.59336.peg.3761	fig|6666666.59333.peg.287	fig|6666666.59338.peg.1001	fig|6666666.59339.peg.8859	fig|6666666.59340.peg.462	fig|6666666.59337.peg.1782	fig|6666666.59342.peg.2598	fig|6666666.71181.peg.4634	fig|6666666.59344.peg.5884	fig|6666666.59345.peg.5664	fig|6666666.59341.peg.3760	fig|6666666.59347.peg.5347	fig|6666666.59346.peg.3040	fig|6666666.59348.peg.2815	fig|6666666.59349.peg.3171	fig|6666666.59350.peg.2273	fig|6666666.59351.peg.3094	fig|6666666.59353.peg.4990	fig|6666666.59355.peg.5537	fig|6666666.59354.peg.6041

8962	Coproporphyrinogen III oxidase, oxygen-independent EC 1.3.99.22	fig|6666666.59329.peg.1828	fig|6666666.59330.peg.336	fig|6666666.59331.peg.5804	fig|6666666.59332.peg.25	fig|6666666.59334.peg.1577	fig|6666666.59335.peg.57	fig|6666666.59336.peg.3051	fig|6666666.59333.peg.761	fig|6666666.59338.peg.3296	fig|6666666.59339.peg.7598	fig|6666666.59340.peg.1976	fig|6666666.59337.peg.517	fig|6666666.59342.peg.5702	fig|6666666.71181.peg.1870	fig|6666666.59344.peg.899	fig|6666666.59345.peg.5006	fig|6666666.59341.peg.3061	fig|6666666.59347.peg.643	fig|6666666.59346.peg.3905	fig|6666666.59348.peg.98	fig|6666666.59349.peg.492	fig|6666666.59350.peg.1501	fig|6666666.59351.peg.3815	fig|6666666.59353.peg.2628	fig|6666666.59355.peg.2576	fig|6666666.59354.peg.6328

4278	Phosphate transport system permease protein PstA TC 3.A.1.7.1	fig|6666666.59329.peg.3155	fig|6666666.59330.peg.3807	fig|6666666.59331.peg.5032	fig|6666666.59332.peg.758	fig|6666666.59334.peg.3839	fig|6666666.59335.peg.2573	fig|6666666.59336.peg.534	fig|6666666.59333.peg.351	fig|6666666.59338.peg.5466	fig|6666666.59339.peg.6926	fig|6666666.59340.peg.343	fig|6666666.59337.peg.3492	fig|6666666.59342.peg.2531	fig|6666666.71181.peg.4287	fig|6666666.59344.peg.4038	fig|6666666.59345.peg.2764	fig|6666666.59341.peg.5820	fig|6666666.59347.peg.4265	fig|6666666.59346.peg.2325	fig|6666666.59348.peg.2242	fig|6666666.59349.peg.3549	fig|6666666.59350.peg.606	fig|6666666.59351.peg.2321	fig|6666666.59353.peg.3074	fig|6666666.59355.peg.2273	fig|6666666.59354.peg.1534

5551	Glutamate synthase [NADPH] small chain EC 1.4.1.13	fig|6666666.59329.peg.6489	fig|6666666.59330.peg.2169	fig|6666666.59331.peg.1377	fig|6666666.59332.peg.6183	fig|6666666.59334.peg.4007	fig|6666666.59335.peg.2095	fig|6666666.59336.peg.3029	fig|6666666.59333.peg.5460	fig|6666666.59338.peg.3179	fig|6666666.59339.peg.3660	fig|6666666.59340.peg.282	fig|6666666.59337.peg.3878	fig|6666666.59342.peg.2413	fig|6666666.71181.peg.86	fig|6666666.59344.peg.4114	fig|6666666.59345.peg.4601	fig|6666666.59341.peg.2896	fig|6666666.59347.peg.2256	fig|6666666.59346.peg.4184	fig|6666666.59348.peg.2293	fig|6666666.59349.peg.2229	fig|6666666.59350.peg.7649	fig|6666666.59351.peg.640	fig|6666666.59353.peg.5078	fig|6666666.59355.peg.1637	fig|6666666.59354.peg.5348

4860	Integration host factor beta subunit	fig|6666666.59329.peg.671	fig|6666666.59330.peg.1386	fig|6666666.59331.peg.2071	fig|6666666.59332.peg.2851	fig|6666666.59334.peg.1580	fig|6666666.59335.peg.3370	fig|6666666.59336.peg.3381	fig|6666666.59333.peg.737	fig|6666666.59338.peg.1339	fig|6666666.59339.peg.3111	fig|6666666.59340.peg.617	fig|6666666.59337.peg.4506	fig|6666666.59342.peg.4596	fig|6666666.71181.peg.3168	fig|6666666.59344.peg.5679	fig|6666666.59345.peg.4254	fig|6666666.59341.peg.1423	fig|6666666.59347.peg.4483	fig|6666666.59346.peg.2664	fig|6666666.59348.peg.5457	fig|6666666.59349.peg.2096	fig|6666666.59350.peg.6664	fig|6666666.59351.peg.943	fig|6666666.59353.peg.750	fig|6666666.59355.peg.4555	fig|6666666.59354.peg.134

2259	Outer membrane protein Imp, required for envelope biogenesis Organic solvent tolerance protein precursor	fig|6666666.59329.peg.127	fig|6666666.59330.peg.815	fig|6666666.59331.peg.4448	fig|6666666.59332.peg.4910	fig|6666666.59334.peg.338	fig|6666666.59335.peg.1638	fig|6666666.59336.peg.2651	fig|6666666.59333.peg.129	fig|6666666.59338.peg.905	fig|6666666.59339.peg.7897	fig|6666666.59340.peg.593	fig|6666666.59337.peg.1387	fig|6666666.59342.peg.5077	fig|6666666.71181.peg.398	fig|6666666.59344.peg.1523	fig|6666666.59345.peg.2456	fig|6666666.59341.peg.5876	fig|6666666.59347.peg.3086	fig|6666666.59346.peg.821	fig|6666666.59348.peg.180	fig|6666666.59349.peg.1879	fig|6666666.59350.peg.659	fig|6666666.59351.peg.3653	fig|6666666.59353.peg.100	fig|6666666.59355.peg.710	fig|6666666.59354.peg.5415

4805	RNA methyltransferase, TrmH family	fig|6666666.59329.peg.5260	fig|6666666.59330.peg.5106	fig|6666666.59331.peg.4402	fig|6666666.59332.peg.1247	fig|6666666.59334.peg.173	fig|6666666.59335.peg.6054	fig|6666666.59336.peg.271	fig|6666666.59333.peg.2347	fig|6666666.59338.peg.4003	fig|6666666.59339.peg.8262	fig|6666666.59340.peg.1107	fig|6666666.59337.peg.4210	fig|6666666.59342.peg.5953	fig|6666666.71181.peg.2645	fig|6666666.59344.peg.921	fig|6666666.59345.peg.1619	fig|6666666.59341.peg.5787	fig|6666666.59347.peg.5339	fig|6666666.59346.peg.2618	fig|6666666.59348.peg.4193	fig|6666666.59349.peg.2493	fig|6666666.59350.peg.2738	fig|6666666.59351.peg.311	fig|6666666.59353.peg.3185	fig|6666666.59355.peg.411	fig|6666666.59354.peg.4402

2077	Flagellum-specific ATP synthase FliI	fig|6666666.59329.peg.521	fig|6666666.59330.peg.4256	fig|6666666.59331.peg.5741	fig|6666666.59332.peg.4803	fig|6666666.59334.peg.18	fig|6666666.59335.peg.2393	fig|6666666.59336.peg.1520	fig|6666666.59333.peg.5587	fig|6666666.59338.peg.3342	fig|6666666.59339.peg.8006	fig|6666666.59340.peg.2249	fig|6666666.59337.peg.2961	fig|6666666.59342.peg.2040	fig|6666666.71181.peg.4025	fig|6666666.59344.peg.4272	fig|6666666.59345.peg.4959	fig|6666666.59341.peg.2133	fig|6666666.59347.peg.4631	fig|6666666.59346.peg.2881	fig|6666666.59348.peg.340	fig|6666666.59349.peg.4963	fig|6666666.59350.peg.7634	fig|6666666.59351.peg.2049	fig|6666666.59353.peg.4560	fig|6666666.59355.peg.5158	fig|6666666.59354.peg.6426

7088	NAD-dependent malic enzyme EC 1.1.1.38	fig|6666666.59329.peg.4604	fig|6666666.59330.peg.1670	fig|6666666.59331.peg.754	fig|6666666.59332.peg.5262	fig|6666666.59334.peg.4355	fig|6666666.59335.peg.1678	fig|6666666.59336.peg.3890	fig|6666666.59333.peg.4783	fig|6666666.59338.peg.1050	fig|6666666.59339.peg.3677	fig|6666666.59340.peg.1163	fig|6666666.59337.peg.4139	fig|6666666.59342.peg.4488	fig|6666666.71181.peg.3977	fig|6666666.59344.peg.240	fig|6666666.59345.peg.4608	fig|6666666.59341.peg.1405	fig|6666666.59347.peg.4920	fig|6666666.59346.peg.3929	fig|6666666.59348.peg.4391	fig|6666666.59349.peg.1606	fig|6666666.59350.peg.4349	fig|6666666.59351.peg.1032	fig|6666666.59353.peg.624	fig|6666666.59355.peg.2795	fig|6666666.59354.peg.929

9807	6-phosphogluconate dehydrogenase, decarboxylating EC 1.1.1.44	fig|6666666.59329.peg.2871	fig|6666666.59330.peg.4020	fig|6666666.59331.peg.2189	fig|6666666.59332.peg.3862	fig|6666666.59334.peg.3014	fig|6666666.59335.peg.4778	fig|6666666.59336.peg.1156	fig|6666666.59333.peg.4044	fig|6666666.59338.peg.4878	fig|6666666.59339.peg.4635	fig|6666666.59340.peg.1326	fig|6666666.59337.peg.3103	fig|6666666.59342.peg.3364	fig|6666666.71181.peg.242	fig|6666666.59344.peg.302	fig|6666666.59345.peg.374	fig|6666666.59341.peg.3820	fig|6666666.59347.peg.1158	fig|6666666.59346.peg.3574	fig|6666666.59348.peg.3469	fig|6666666.59349.peg.2150	fig|6666666.59350.peg.3507	fig|6666666.59351.peg.3706	fig|6666666.59353.peg.577	fig|6666666.59355.peg.61	fig|6666666.59354.peg.1873

8390	3' 2' ,5'-bisphosphate nucleotidase EC 3.1.3.7	fig|6666666.59329.peg.1235	fig|6666666.59330.peg.428	fig|6666666.59331.peg.5737	fig|6666666.59332.peg.2473	fig|6666666.59334.peg.206	fig|6666666.59335.peg.694	fig|6666666.59336.peg.4430	fig|6666666.59333.peg.517	fig|6666666.59338.peg.1427	fig|6666666.59339.peg.6238	fig|6666666.59340.peg.2907	fig|6666666.59337.peg.4256	fig|6666666.59342.peg.1141	fig|6666666.71181.peg.1754	fig|6666666.59344.peg.2141	fig|6666666.59345.peg.3655	fig|6666666.59341.peg.5605	fig|6666666.59347.peg.4280	fig|6666666.59346.peg.2743	fig|6666666.59348.peg.1404	fig|6666666.59349.peg.2288	fig|6666666.59350.peg.7609	fig|6666666.59351.peg.5229	fig|6666666.59353.peg.2691	fig|6666666.59355.peg.426	fig|6666666.59354.peg.6224

11380	3',5'-cyclic-nucleotide phosphodiesterase EC 3.1.4.17	fig|6666666.59329.peg.3073	fig|6666666.59330.peg.952	fig|6666666.59331.peg.5822	fig|6666666.59332.peg.1425	fig|6666666.59334.peg.392	fig|6666666.59335.peg.3446	fig|6666666.59336.peg.1939	fig|6666666.59333.peg.630	fig|6666666.59338.peg.970	fig|6666666.59339.peg.4926	fig|6666666.59340.peg.2652	fig|6666666.59337.peg.18	fig|6666666.59342.peg.3213	fig|6666666.71181.peg.3037	fig|6666666.59344.peg.5620	fig|6666666.59345.peg.3823	fig|6666666.59341.peg.1722	fig|6666666.59347.peg.3912	fig|6666666.59346.peg.1633	fig|6666666.59348.peg.2786	fig|6666666.59349.peg.818	fig|6666666.59350.peg.3527	fig|6666666.59351.peg.3547	fig|6666666.59353.peg.4923	fig|6666666.59355.peg.4966	fig|6666666.59354.peg.662

7863	Potassium efflux system KefA protein Small-conductance mechanosensitive channel	fig|6666666.59329.peg.5718	fig|6666666.59330.peg.1232	fig|6666666.59331.peg.1929	fig|6666666.59332.peg.6058	fig|6666666.59334.peg.2652	fig|6666666.59335.peg.2311	fig|6666666.59336.peg.1198	fig|6666666.59333.peg.4936	fig|6666666.59338.peg.1039	fig|6666666.59339.peg.255	fig|6666666.59340.peg.3419	fig|6666666.59337.peg.5100	fig|6666666.59342.peg.5138	fig|6666666.71181.peg.1691	fig|6666666.59344.peg.601	fig|6666666.59345.peg.1176	fig|6666666.59341.peg.4026	fig|6666666.59347.peg.1708	fig|6666666.59346.peg.309	fig|6666666.59348.peg.888	fig|6666666.59349.peg.229	fig|6666666.59350.peg.2410	fig|6666666.59351.peg.3781	fig|6666666.59353.peg.4427	fig|6666666.59355.peg.4507	fig|6666666.59354.peg.151

10141	Ribosomal large subunit pseudouridine synthase E EC 4.2.1.70	fig|6666666.59329.peg.5268	fig|6666666.59330.peg.3693	fig|6666666.59331.peg.4857	fig|6666666.59332.peg.5737	fig|6666666.59334.peg.2570	fig|6666666.59335.peg.955	fig|6666666.59336.peg.857	fig|6666666.59333.peg.1013	fig|6666666.59338.peg.2084	fig|6666666.59339.peg.343	fig|6666666.59340.peg.1708	fig|6666666.59337.peg.1732	fig|6666666.59342.peg.4247	fig|6666666.71181.peg.487	fig|6666666.59344.peg.4807	fig|6666666.59345.peg.910	fig|6666666.59341.peg.1855	fig|6666666.59347.peg.1996	fig|6666666.59346.peg.226	fig|6666666.59348.peg.2723	fig|6666666.59349.peg.953	fig|6666666.59350.peg.5883	fig|6666666.59351.peg.796	fig|6666666.59353.peg.476	fig|6666666.59355.peg.2201	fig|6666666.59354.peg.3690

9328	Membrane-associated zinc metalloprotease	fig|6666666.59329.peg.1579	fig|6666666.59330.peg.461	fig|6666666.59331.peg.5561	fig|6666666.59332.peg.1583	fig|6666666.59334.peg.5527	fig|6666666.59335.peg.2659	fig|6666666.59336.peg.2564	fig|6666666.59333.peg.4245	fig|6666666.59338.peg.5647	fig|6666666.59339.peg.5117	fig|6666666.59340.peg.3565	fig|6666666.59337.peg.1589	fig|6666666.59342.peg.275	fig|6666666.71181.peg.28	fig|6666666.59344.peg.2661	fig|6666666.59345.peg.2194	fig|6666666.59341.peg.975	fig|6666666.59347.peg.4365	fig|6666666.59346.peg.2267	fig|6666666.59348.peg.3062	fig|6666666.59349.peg.1368	fig|6666666.59350.peg.4430	fig|6666666.59351.peg.2122	fig|6666666.59353.peg.1992	fig|6666666.59355.peg.4097	fig|6666666.59354.peg.1339

8942	LSU ribosomal protein L4p L1e	fig|6666666.59329.peg.1102	fig|6666666.59330.peg.551	fig|6666666.59331.peg.3026	fig|6666666.59332.peg.3796	fig|6666666.59334.peg.1482	fig|6666666.59335.peg.1850	fig|6666666.59336.peg.142	fig|6666666.59333.peg.2690	fig|6666666.59338.peg.1392	fig|6666666.59339.peg.8265	fig|6666666.59340.peg.1728	fig|6666666.59337.peg.391	fig|6666666.59342.peg.301	fig|6666666.71181.peg.3459	fig|6666666.59344.peg.4942	fig|6666666.59345.peg.2539	fig|6666666.59341.peg.2495	fig|6666666.59347.peg.4820	fig|6666666.59346.peg.5020	fig|6666666.59348.peg.411	fig|6666666.59349.peg.1095	fig|6666666.59350.peg.4266	fig|6666666.59351.peg.4817	fig|6666666.59353.peg.4591	fig|6666666.59355.peg.4740	fig|6666666.59354.peg.1750

1194	DNA ligase EC 6.5.1.2	fig|6666666.59329.peg.915	fig|6666666.59330.peg.3035	fig|6666666.59331.peg.3496	fig|6666666.59332.peg.633	fig|6666666.59334.peg.3263	fig|6666666.59335.peg.3017	fig|6666666.59336.peg.4670	fig|6666666.59333.peg.4114	fig|6666666.59338.peg.22	fig|6666666.59339.peg.5236	fig|6666666.59340.peg.1785	fig|6666666.59337.peg.5546	fig|6666666.59342.peg.104	fig|6666666.71181.peg.1574	fig|6666666.59344.peg.2011	fig|6666666.59345.peg.4388	fig|6666666.59341.peg.4614	fig|6666666.59347.peg.2896	fig|6666666.59346.peg.2376	fig|6666666.59348.peg.3201	fig|6666666.59349.peg.2496	fig|6666666.59350.peg.1097	fig|6666666.59351.peg.4632	fig|6666666.59353.peg.2913	fig|6666666.59355.peg.2826	fig|6666666.59354.peg.4787

1197	Carbamoyl-phosphate synthase large chain EC 6.3.5.5	fig|6666666.59329.peg.73	fig|6666666.59330.peg.1289	fig|6666666.59331.peg.5476	fig|6666666.59332.peg.1107	fig|6666666.59334.peg.2077	fig|6666666.59335.peg.5007	fig|6666666.59336.peg.2527	fig|6666666.59333.peg.540	fig|6666666.59338.peg.5278	fig|6666666.59339.peg.8099	fig|6666666.59340.peg.665	fig|6666666.59337.peg.4527	fig|6666666.59342.peg.2605	fig|6666666.71181.peg.1654	fig|6666666.59344.peg.1303	fig|6666666.59345.peg.5146	fig|6666666.59341.peg.1180	fig|6666666.59347.peg.5015	fig|6666666.59346.peg.1153	fig|6666666.59348.peg.945	fig|6666666.59349.peg.4250	fig|6666666.59350.peg.2562	fig|6666666.59351.peg.3193	fig|6666666.59353.peg.1023	fig|6666666.59355.peg.3595	fig|6666666.59354.peg.2505

4258	Putative heat shock protein YegD	fig|6666666.59329.peg.5442	fig|6666666.59330.peg.3847	fig|6666666.59331.peg.4877	fig|6666666.59332.peg.6259	fig|6666666.59334.peg.4351	fig|6666666.59335.peg.5474	fig|6666666.59336.peg.6031	fig|6666666.59333.peg.3297	fig|6666666.59338.peg.295	fig|6666666.59339.peg.4134	fig|6666666.59340.peg.1205	fig|6666666.59337.peg.4807	fig|6666666.59342.peg.1603	fig|6666666.71181.peg.4293	fig|6666666.59344.peg.4051	fig|6666666.59345.peg.314	fig|6666666.59341.peg.4127	fig|6666666.59347.peg.32	fig|6666666.59346.peg.4103	fig|6666666.59348.peg.5040	fig|6666666.59349.peg.3929	fig|6666666.59350.peg.114	fig|6666666.59351.peg.419	fig|6666666.59353.peg.4006	fig|6666666.59355.peg.5736	fig|6666666.59354.peg.4361

6576	MotA TolQ ExbB proton channel family protein	fig|6666666.59329.peg.4963	fig|6666666.59330.peg.4177	fig|6666666.59331.peg.2229	fig|6666666.59332.peg.1965	fig|6666666.59334.peg.5425	fig|6666666.59335.peg.4620	fig|6666666.59336.peg.937	fig|6666666.59333.peg.3770	fig|6666666.59338.peg.664	fig|6666666.59339.peg.978	fig|6666666.59340.peg.1868	fig|6666666.59337.peg.4383	fig|6666666.59342.peg.4165	fig|6666666.71181.peg.2251	fig|6666666.59344.peg.4576	fig|6666666.59345.peg.2643	fig|6666666.59341.peg.3490	fig|6666666.59347.peg.4608	fig|6666666.59346.peg.4089	fig|6666666.59348.peg.1086	fig|6666666.59349.peg.2931	fig|6666666.59350.peg.3041	fig|6666666.59351.peg.1688	fig|6666666.59353.peg.2098	fig|6666666.59355.peg.3627	fig|6666666.59354.peg.1594

7697	LSU ribosomal protein L14p L23e	fig|6666666.59329.peg.719	fig|6666666.59330.peg.560	fig|6666666.59331.peg.42	fig|6666666.59332.peg.2145	fig|6666666.59334.peg.1491	fig|6666666.59335.peg.4353	fig|6666666.59336.peg.152	fig|6666666.59333.peg.372	fig|6666666.59338.peg.1383	fig|6666666.59339.peg.8274	fig|6666666.59340.peg.62	fig|6666666.59337.peg.382	fig|6666666.59342.peg.310	fig|6666666.71181.peg.3450	fig|6666666.59344.peg.4933	fig|6666666.59345.peg.2529	fig|6666666.59341.peg.2504	fig|6666666.59347.peg.4829	fig|6666666.59346.peg.856	fig|6666666.59348.peg.1766	fig|6666666.59349.peg.3276	fig|6666666.59350.peg.7707	fig|6666666.59351.peg.4210	fig|6666666.59353.peg.4600	fig|6666666.59355.peg.4731	fig|6666666.59354.peg.3849

1345	Potassium uptake protein TrkH	fig|6666666.59329.peg.61	fig|6666666.59330.peg.640	fig|6666666.59331.peg.5140	fig|6666666.59332.peg.65	fig|6666666.59334.peg.1390	fig|6666666.59335.peg.6220	fig|6666666.59336.peg.2814	fig|6666666.59333.peg.4074	fig|6666666.59338.peg.1348	fig|6666666.59339.peg.7986	fig|6666666.59340.peg.1063	fig|6666666.59337.peg.855	fig|6666666.59342.peg.6061	fig|6666666.71181.peg.3324	fig|6666666.59344.peg.3396	fig|6666666.59345.peg.4997	fig|6666666.59341.peg.1948	fig|6666666.59347.peg.4815	fig|6666666.59346.peg.1819	fig|6666666.59348.peg.3710	fig|6666666.59349.peg.2812	fig|6666666.59350.peg.1772	fig|6666666.59351.peg.5048	fig|6666666.59353.peg.4231	fig|6666666.59355.peg.4193	fig|6666666.59354.peg.1435

371	C4-type zinc finger protein, DksA TraR family	fig|6666666.59329.peg.4550	fig|6666666.59330.peg.1758	fig|6666666.59331.peg.914	fig|6666666.59332.peg.6030	fig|6666666.59334.peg.823	fig|6666666.59335.peg.1516	fig|6666666.59336.peg.573	fig|6666666.59333.peg.313	fig|6666666.59338.peg.2829	fig|6666666.59339.peg.4772	fig|6666666.59340.peg.145	fig|6666666.59337.peg.625	fig|6666666.59342.peg.1569	fig|6666666.71181.peg.2838	fig|6666666.59344.peg.4442	fig|6666666.59345.peg.2403	fig|6666666.59341.peg.1551	fig|6666666.59347.peg.3968	fig|6666666.59346.peg.3824	fig|6666666.59348.peg.1701	fig|6666666.59349.peg.2477	fig|6666666.59350.peg.771	fig|6666666.59351.peg.363	fig|6666666.59353.peg.3189	fig|6666666.59355.peg.3071	fig|6666666.59354.peg.49

10975	Lysophospholipase L2 EC 3.1.1.5	fig|6666666.59329.peg.78	fig|6666666.59330.peg.1546	fig|6666666.59331.peg.6142	fig|6666666.59332.peg.473	fig|6666666.59334.peg.4430	fig|6666666.59335.peg.4120	fig|6666666.59336.peg.1477	fig|6666666.59333.peg.802	fig|6666666.59338.peg.1994	fig|6666666.59339.peg.8542	fig|6666666.59340.peg.557	fig|6666666.59337.peg.1628	fig|6666666.59342.peg.6030	fig|6666666.71181.peg.4255	fig|6666666.59344.peg.2506	fig|6666666.59345.peg.1217	fig|6666666.59341.peg.4985	fig|6666666.59347.peg.5067	fig|6666666.59346.peg.613	fig|6666666.59348.peg.743	fig|6666666.59349.peg.2587	fig|6666666.59350.peg.3852	fig|6666666.59351.peg.268	fig|6666666.59353.peg.4643	fig|6666666.59355.peg.599	fig|6666666.59354.peg.4003

6963	Translation elongation factor P	fig|6666666.59329.peg.2701	fig|6666666.59330.peg.2934	fig|6666666.59331.peg.2635	fig|6666666.59332.peg.3761	fig|6666666.59334.peg.4002	fig|6666666.59335.peg.3844	fig|6666666.59336.peg.875	fig|6666666.59333.peg.430	fig|6666666.59338.peg.3282	fig|6666666.59339.peg.8687	fig|6666666.59340.peg.220	fig|6666666.59337.peg.4328	fig|6666666.59342.peg.6110	fig|6666666.71181.peg.3173	fig|6666666.59344.peg.611	fig|6666666.59345.peg.908	fig|6666666.59341.peg.5351	fig|6666666.59347.peg.4554	fig|6666666.59346.peg.975	fig|6666666.59348.peg.3886	fig|6666666.59349.peg.2373	fig|6666666.59350.peg.3216	fig|6666666.59351.peg.134	fig|6666666.59353.peg.1051	fig|6666666.59355.peg.381	fig|6666666.59354.peg.5461

6465	putative reductase	fig|6666666.59329.peg.110	fig|6666666.59330.peg.1725	fig|6666666.59331.peg.4724	fig|6666666.59332.peg.2903	fig|6666666.59334.peg.4031	fig|6666666.59335.peg.4887	fig|6666666.59336.peg.2644	fig|6666666.59333.peg.1971	fig|6666666.59338.peg.5263	fig|6666666.59339.peg.7062	fig|6666666.59340.peg.2729	fig|6666666.59337.peg.4841	fig|6666666.59342.peg.2788	fig|6666666.71181.peg.2481	fig|6666666.59344.peg.4160	fig|6666666.59345.peg.3749	fig|6666666.59341.peg.4668	fig|6666666.59347.peg.5119	fig|6666666.59346.peg.4391	fig|6666666.59348.peg.1546	fig|6666666.59349.peg.1182	fig|6666666.59350.peg.1322	fig|6666666.59351.peg.1338	fig|6666666.59353.peg.3065	fig|6666666.59355.peg.4359	fig|6666666.59354.peg.3442

11367	LSU ribosomal protein L17p	fig|6666666.59329.peg.734	fig|6666666.59330.peg.577	fig|6666666.59331.peg.26	fig|6666666.59332.peg.3504	fig|6666666.59334.peg.3039	fig|6666666.59335.peg.4755	fig|6666666.59336.peg.167	fig|6666666.59333.peg.389	fig|6666666.59338.peg.5799	fig|6666666.59339.peg.8291	fig|6666666.59340.peg.584	fig|6666666.59337.peg.366	fig|6666666.59342.peg.327	fig|6666666.71181.peg.3438	fig|6666666.59344.peg.4921	fig|6666666.59345.peg.2513	fig|6666666.59341.peg.777	fig|6666666.59347.peg.4846	fig|6666666.59346.peg.872	fig|6666666.59348.peg.432	fig|6666666.59349.peg.3292	fig|6666666.59350.peg.4708	fig|6666666.59351.peg.4227	fig|6666666.59353.peg.4612	fig|6666666.59355.peg.4719	fig|6666666.59354.peg.3245

1812	DNA polymerase III subunits gamma and tau EC 2.7.7.7	fig|6666666.59329.peg.2503	fig|6666666.59330.peg.4120	fig|6666666.59331.peg.2652	fig|6666666.59332.peg.4654	fig|6666666.59334.peg.2756	fig|6666666.59335.peg.3290	fig|6666666.59336.peg.5174	fig|6666666.59333.peg.2534	fig|6666666.59338.peg.2297	fig|6666666.59339.peg.3064	fig|6666666.59340.peg.495	fig|6666666.59337.peg.2681	fig|6666666.59342.peg.4073	fig|6666666.71181.peg.1629	fig|6666666.59344.peg.5520	fig|6666666.59345.peg.1193	fig|6666666.59341.peg.2459	fig|6666666.59347.peg.2446	fig|6666666.59346.peg.64	fig|6666666.59348.peg.3116	fig|6666666.59349.peg.4682	fig|6666666.59350.peg.2223	fig|6666666.59351.peg.2798	fig|6666666.59353.peg.320	fig|6666666.59355.peg.1360	fig|6666666.59354.peg.5010

3224	DNA polymerase I EC 2.7.7.7	fig|6666666.59329.peg.2092	fig|6666666.59330.peg.3898	fig|6666666.59331.peg.3308	fig|6666666.59332.peg.4500	fig|6666666.59334.peg.842	fig|6666666.59335.peg.50	fig|6666666.59336.peg.1456	fig|6666666.59333.peg.836	fig|6666666.59338.peg.1651	fig|6666666.59339.peg.7584	fig|6666666.59340.peg.95	fig|6666666.59337.peg.971	fig|6666666.59342.peg.6475	fig|6666666.71181.peg.50	fig|6666666.59344.peg.895	fig|6666666.59345.peg.2704	fig|6666666.59341.peg.164	fig|6666666.59347.peg.2932	fig|6666666.59346.peg.1732	fig|6666666.59348.peg.105	fig|6666666.59349.peg.445	fig|6666666.59350.peg.7721	fig|6666666.59351.peg.94	fig|6666666.59353.peg.2633	fig|6666666.59355.peg.1439	fig|6666666.59354.peg.1893

2845	Na + -translocating NADH-quinone reductase subunit D EC 1.6.5.-	fig|6666666.59329.peg.4958	fig|6666666.59330.peg.2783	fig|6666666.59331.peg.435	fig|6666666.59332.peg.2205	fig|6666666.59334.peg.884	fig|6666666.59335.peg.122	fig|6666666.59336.peg.4523	fig|6666666.59333.peg.1801	fig|6666666.59338.peg.4864	fig|6666666.59339.peg.5049	fig|6666666.59340.peg.660	fig|6666666.59337.peg.2382	fig|6666666.59342.peg.1976	fig|6666666.71181.peg.918	fig|6666666.59344.peg.5167	fig|6666666.59345.peg.2234	fig|6666666.59341.peg.2294	fig|6666666.59347.peg.2799	fig|6666666.59346.peg.2723	fig|6666666.59348.peg.1315	fig|6666666.59349.peg.304	fig|6666666.59350.peg.4834	fig|6666666.59351.peg.3928	fig|6666666.59353.peg.897	fig|6666666.59355.peg.807	fig|6666666.59354.peg.4980

3326	Transcriptional regulator, ArsR family	fig|6666666.59329.peg.2580	fig|6666666.59330.peg.1549	fig|6666666.59331.peg.729	fig|6666666.59332.peg.470	fig|6666666.59334.peg.2143	fig|6666666.59335.peg.1013	fig|6666666.59336.peg.629	fig|6666666.59333.peg.5435	fig|6666666.59338.peg.976	fig|6666666.59339.peg.2786	fig|6666666.59340.peg.1380	fig|6666666.59337.peg.1632	fig|6666666.59342.peg.1410	fig|6666666.71181.peg.6307	fig|6666666.59344.peg.756	fig|6666666.59345.peg.5487	fig|6666666.59341.peg.637	fig|6666666.59347.peg.3934	fig|6666666.59346.peg.400	fig|6666666.59348.peg.740	fig|6666666.59349.peg.2925	fig|6666666.59350.peg.3923	fig|6666666.59351.peg.4197	fig|6666666.59353.peg.384	fig|6666666.59355.peg.2526	fig|6666666.59354.peg.5759

5479	3-methyl-2-oxobutanoate hydroxymethyltransferase EC 2.1.2.11	fig|6666666.59329.peg.4157	fig|6666666.59330.peg.4040	fig|6666666.59331.peg.2464	fig|6666666.59332.peg.3812	fig|6666666.59334.peg.819	fig|6666666.59335.peg.2329	fig|6666666.59336.peg.2746	fig|6666666.59333.peg.134	fig|6666666.59338.peg.206	fig|6666666.59339.peg.4765	fig|6666666.59340.peg.1961	fig|6666666.59337.peg.620	fig|6666666.59342.peg.1576	fig|6666666.71181.peg.2844	fig|6666666.59344.peg.4434	fig|6666666.59345.peg.2407	fig|6666666.59341.peg.1555	fig|6666666.59347.peg.3964	fig|6666666.59346.peg.3820	fig|6666666.59348.peg.1695	fig|6666666.59349.peg.2473	fig|6666666.59350.peg.5737	fig|6666666.59351.peg.359	fig|6666666.59353.peg.3196	fig|6666666.59355.peg.3077	fig|6666666.59354.peg.44

3038	Phosphotransferase system, phosphocarrier protein HPr	fig|6666666.59329.peg.907	fig|6666666.59330.peg.3031	fig|6666666.59331.peg.6006	fig|6666666.59332.peg.5051	fig|6666666.59334.peg.5240	fig|6666666.59335.peg.3057	fig|6666666.59336.peg.2338	fig|6666666.59333.peg.4293	fig|6666666.59338.peg.100	fig|6666666.59339.peg.5231	fig|6666666.59340.peg.3440	fig|6666666.59337.peg.825	fig|6666666.59342.peg.111	fig|6666666.71181.peg.1578	fig|6666666.59344.peg.2007	fig|6666666.59345.peg.4393	fig|6666666.59341.peg.1632	fig|6666666.59347.peg.4444	fig|6666666.59346.peg.645	fig|6666666.59348.peg.2213	fig|6666666.59349.peg.16	fig|6666666.59350.peg.5374	fig|6666666.59351.peg.3434	fig|6666666.59353.peg.2330	fig|6666666.59355.peg.2822	fig|6666666.59354.peg.3613

1909	Oxaloacetate decarboxylase alpha chain EC 4.1.1.3	fig|6666666.59329.peg.4822	fig|6666666.59330.peg.2826	fig|6666666.59331.peg.4729	fig|6666666.59332.peg.118	fig|6666666.59334.peg.249	fig|6666666.59335.peg.5241	fig|6666666.59336.peg.1350	fig|6666666.59333.peg.5235	fig|6666666.59338.peg.58	fig|6666666.59339.peg.6442	fig|6666666.59340.peg.419	fig|6666666.59337.peg.4290	fig|6666666.59342.peg.4973	fig|6666666.71181.peg.378	fig|6666666.59344.peg.1499	fig|6666666.59345.peg.386	fig|6666666.59341.peg.1693	fig|6666666.59347.peg.3222	fig|6666666.59346.peg.5035	fig|6666666.59348.peg.4867	fig|6666666.59349.peg.2362	fig|6666666.59350.peg.3819	fig|6666666.59351.peg.1	fig|6666666.59353.peg.121	fig|6666666.59355.peg.731	fig|6666666.59354.peg.592

6514	tRNA nucleotidyltransferase EC 2.7.7.21 EC 2.7.7.25	fig|6666666.59329.peg.690	fig|6666666.59330.peg.1755	fig|6666666.59331.peg.2592	fig|6666666.59332.peg.1506	fig|6666666.59334.peg.530	fig|6666666.59335.peg.1504	fig|6666666.59336.peg.2742	fig|6666666.59333.peg.132	fig|6666666.59338.peg.2831	fig|6666666.59339.peg.4769	fig|6666666.59340.peg.2166	fig|6666666.59337.peg.1	fig|6666666.59342.peg.1571	fig|6666666.71181.peg.3014	fig|6666666.59344.peg.4440	fig|6666666.59345.peg.3840	fig|6666666.59341.peg.4834	fig|6666666.59347.peg.3130	fig|6666666.59346.peg.3822	fig|6666666.59348.peg.1698	fig|6666666.59349.peg.1676	fig|6666666.59350.peg.255	fig|6666666.59351.peg.361	fig|6666666.59353.peg.3191	fig|6666666.59355.peg.1694	fig|6666666.59354.peg.47

7547	Guanylate kinase EC 2.7.4.8	fig|6666666.59329.peg.1269	fig|6666666.59330.peg.1193	fig|6666666.59331.peg.2947	fig|6666666.59332.peg.724	fig|6666666.59334.peg.236	fig|6666666.59335.peg.4065	fig|6666666.59336.peg.833	fig|6666666.59333.peg.4058	fig|6666666.59338.peg.2939	fig|6666666.59339.peg.6251	fig|6666666.59340.peg.156	fig|6666666.59337.peg.501	fig|6666666.59342.peg.580	fig|6666666.71181.peg.2606	fig|6666666.59344.peg.925	fig|6666666.59345.peg.2648	fig|6666666.59341.peg.4958	fig|6666666.59347.peg.5198	fig|6666666.59346.peg.1386	fig|6666666.59348.peg.1928	fig|6666666.59349.peg.3646	fig|6666666.59350.peg.600	fig|6666666.59351.peg.318	fig|6666666.59353.peg.2605	fig|6666666.59355.peg.2597	fig|6666666.59354.peg.3356

6166	C3118 Thioredoxin domain-containing protein EC-YbbN	fig|6666666.59329.peg.5190	fig|6666666.59330.peg.2193	fig|6666666.59331.peg.4006	fig|6666666.59332.peg.625	fig|6666666.59334.peg.2222	fig|6666666.59335.peg.3011	fig|6666666.59336.peg.1513	fig|6666666.59333.peg.4953	fig|6666666.59338.peg.2799	fig|6666666.59339.peg.5245	fig|6666666.59340.peg.993	fig|6666666.59337.peg.5064	fig|6666666.59342.peg.1951	fig|6666666.71181.peg.3617	fig|6666666.59344.peg.5065	fig|6666666.59345.peg.4366	fig|6666666.59341.peg.4979	fig|6666666.59347.peg.4433	fig|6666666.59346.peg.2369	fig|6666666.59348.peg.3559	fig|6666666.59349.peg.3888	fig|6666666.59350.peg.453	fig|6666666.59351.peg.1373	fig|6666666.59353.peg.4052	fig|6666666.59355.peg.6056	fig|6666666.59354.peg.4386

10999	Histidinol-phosphatase EC 3.1.3.15 Imidazoleglycerol-phosphate dehydratase EC 4.2.1.19	fig|6666666.59329.peg.1011	fig|6666666.59330.peg.1504	fig|6666666.59331.peg.2983	fig|6666666.59332.peg.1812	fig|6666666.59334.peg.4498	fig|6666666.59335.peg.591	fig|6666666.59336.peg.4085	fig|6666666.59333.peg.2717	fig|6666666.59338.peg.2164	fig|6666666.59339.peg.496	fig|6666666.59340.peg.2686	fig|6666666.59337.peg.4870	fig|6666666.59342.peg.2822	fig|6666666.71181.peg.247	fig|6666666.59344.peg.309	fig|6666666.59345.peg.1305	fig|6666666.59341.peg.500	fig|6666666.59347.peg.4146	fig|6666666.59346.peg.2687	fig|6666666.59348.peg.5344	fig|6666666.59349.peg.457	fig|6666666.59350.peg.3414	fig|6666666.59351.peg.1623	fig|6666666.59353.peg.571	fig|6666666.59355.peg.55	fig|6666666.59354.peg.892

10991	3-ketoacyl-CoA thiolase EC 2.3.1.16 Acetyl-CoA acetyltransferase EC 2.3.1.9	fig|6666666.59329.peg.4093	fig|6666666.59330.peg.2101	fig|6666666.59331.peg.2764	fig|6666666.59332.peg.2665	fig|6666666.59334.peg.4776	fig|6666666.59335.peg.3383	fig|6666666.59336.peg.2061	fig|6666666.59333.peg.4077	fig|6666666.59338.peg.1241	fig|6666666.59339.peg.4623	fig|6666666.59340.peg.1536	fig|6666666.59337.peg.858	fig|6666666.59342.peg.2721	fig|6666666.71181.peg.5872	fig|6666666.59344.peg.5841	fig|6666666.59345.peg.490	fig|6666666.59341.peg.1698	fig|6666666.59347.peg.1600	fig|6666666.59346.peg.4262	fig|6666666.59348.peg.5333	fig|6666666.59349.peg.4070	fig|6666666.59350.peg.8228	fig|6666666.59351.peg.705	fig|6666666.59353.peg.5023	fig|6666666.59355.peg.572	fig|6666666.59354.peg.4950

1015	Thioredoxin	fig|6666666.59329.peg.6275	fig|6666666.59330.peg.1002	fig|6666666.59331.peg.1507	fig|6666666.59332.peg.5851	fig|6666666.59334.peg.418	fig|6666666.59335.peg.4611	fig|6666666.59336.peg.1639	fig|6666666.59333.peg.3249	fig|6666666.59338.peg.1197	fig|6666666.59339.peg.7679	fig|6666666.59340.peg.2270	fig|6666666.59337.peg.2527	fig|6666666.59342.peg.3024	fig|6666666.71181.peg.2453	fig|6666666.59344.peg.2458	fig|6666666.59345.peg.5519	fig|6666666.59341.peg.4205	fig|6666666.59347.peg.5234	fig|6666666.59346.peg.4602	fig|6666666.59348.peg.4522	fig|6666666.59349.peg.4932	fig|6666666.59350.peg.1183	fig|6666666.59351.peg.4774	fig|6666666.59353.peg.3653	fig|6666666.59355.peg.4023	fig|6666666.59354.peg.4154

9494	Cyclic AMP receptor protein	fig|6666666.59329.peg.3002	fig|6666666.59330.peg.584	fig|6666666.59331.peg.474	fig|6666666.59332.peg.3182	fig|6666666.59334.peg.1543	fig|6666666.59335.peg.3531	fig|6666666.59336.peg.4962	fig|6666666.59333.peg.2354	fig|6666666.59338.peg.1869	fig|6666666.59339.peg.8233	fig|6666666.59340.peg.3153	fig|6666666.59337.peg.358	fig|6666666.59342.peg.596	fig|6666666.71181.peg.4347	fig|6666666.59344.peg.593	fig|6666666.59345.peg.2504	fig|6666666.59341.peg.2765	fig|6666666.59347.peg.4958	fig|6666666.59346.peg.25	fig|6666666.59348.peg.4306	fig|6666666.59349.peg.837	fig|6666666.59350.peg.2423	fig|6666666.59351.peg.827	fig|6666666.59353.peg.2243	fig|6666666.59355.peg.1935	fig|6666666.59354.peg.2722

5700	Periplasmic alpha-amylase EC 3.2.1.1	fig|6666666.59329.peg.3608	fig|6666666.59330.peg.4324	fig|6666666.59331.peg.5715	fig|6666666.59332.peg.2800	fig|6666666.59334.peg.1567	fig|6666666.59335.peg.5807	fig|6666666.59336.peg.559	fig|6666666.59333.peg.1665	fig|6666666.59338.peg.6154	fig|6666666.59339.peg.6616	fig|6666666.59340.peg.2303	fig|6666666.59337.peg.3807	fig|6666666.59342.peg.5207	fig|6666666.71181.peg.642	fig|6666666.59344.peg.4630	fig|6666666.59345.peg.4985	fig|6666666.59341.peg.1756	fig|6666666.59347.peg.2916	fig|6666666.59346.peg.3912	fig|6666666.59348.peg.3067	fig|6666666.59349.peg.3577	fig|6666666.59350.peg.7963	fig|6666666.59351.peg.1583	fig|6666666.59353.peg.3008	fig|6666666.59355.peg.3538	fig|6666666.59354.peg.5854

1057	5-methyltetrahydrofolate--homocysteine methyltransferase EC 2.1.1.13	fig|6666666.59329.peg.1313	fig|6666666.59330.peg.3916	fig|6666666.59331.peg.5585	fig|6666666.59332.peg.2533	fig|6666666.59334.peg.1600	fig|6666666.59335.peg.2144	fig|6666666.59336.peg.1289	fig|6666666.59333.peg.590	fig|6666666.59338.peg.2851	fig|6666666.59339.peg.8211	fig|6666666.59340.peg.1272	fig|6666666.59337.peg.4960	fig|6666666.59342.peg.6384	fig|6666666.71181.peg.3980	fig|6666666.59344.peg.2704	fig|6666666.59345.peg.118	fig|6666666.59341.peg.4372	fig|6666666.59347.peg.1422	fig|6666666.59346.peg.1589	fig|6666666.59348.peg.2912	fig|6666666.59349.peg.3670	fig|6666666.59350.peg.8123	fig|6666666.59351.peg.1456	fig|6666666.59353.peg.2941	fig|6666666.59355.peg.2798	fig|6666666.59354.peg.1731

2400	ADP-ribose pyrophosphatase EC 3.6.1.13	fig|6666666.59329.peg.3075	fig|6666666.59330.peg.3155	fig|6666666.59331.peg.3484	fig|6666666.59332.peg.4401	fig|6666666.59334.peg.1752	fig|6666666.59335.peg.2256	fig|6666666.59336.peg.1942	fig|6666666.59333.peg.632	fig|6666666.59338.peg.5518	fig|6666666.59339.peg.2628	fig|6666666.59340.peg.796	fig|6666666.59337.peg.16	fig|6666666.59342.peg.502	fig|6666666.71181.peg.4361	fig|6666666.59344.peg.1668	fig|6666666.59345.peg.3476	fig|6666666.59341.peg.1121	fig|6666666.59347.peg.3034	fig|6666666.59346.peg.1250	fig|6666666.59348.peg.1808	fig|6666666.59349.peg.2660	fig|6666666.59350.peg.5819	fig|6666666.59351.peg.561	fig|6666666.59353.peg.4468	fig|6666666.59355.peg.4988	fig|6666666.59354.peg.4062

2159	Sodium alanine symporter	fig|6666666.59329.peg.6532	fig|6666666.59330.peg.4313	fig|6666666.59331.peg.5714	fig|6666666.59332.peg.3054	fig|6666666.59334.peg.3171	fig|6666666.59335.peg.3633	fig|6666666.59336.peg.5288	fig|6666666.59333.peg.2158	fig|6666666.59338.peg.463	fig|6666666.59339.peg.5006	fig|6666666.59340.peg.1657	fig|6666666.59337.peg.767	fig|6666666.59342.peg.3722	fig|6666666.71181.peg.432	fig|6666666.59344.peg.751	fig|6666666.59345.peg.4205	fig|6666666.59341.peg.3457	fig|6666666.59347.peg.4308	fig|6666666.59346.peg.3475	fig|6666666.59348.peg.2033	fig|6666666.59349.peg.3517	fig|6666666.59350.peg.964	fig|6666666.59351.peg.5137	fig|6666666.59353.peg.1352	fig|6666666.59355.peg.2520	fig|6666666.59354.peg.6111

510	Putative permease PerM = YfgO	fig|6666666.59329.peg.5357	fig|6666666.59330.peg.5112	fig|6666666.59331.peg.524	fig|6666666.59332.peg.1007	fig|6666666.59334.peg.3859	fig|6666666.59335.peg.1194	fig|6666666.59336.peg.1613	fig|6666666.59333.peg.4692	fig|6666666.59338.peg.1194	fig|6666666.59339.peg.4831	fig|6666666.59340.peg.2070	fig|6666666.59337.peg.3604	fig|6666666.59342.peg.3067	fig|6666666.71181.peg.1239	fig|6666666.59344.peg.174	fig|6666666.59345.peg.2157	fig|6666666.59341.peg.5200	fig|6666666.59347.peg.3978	fig|6666666.59346.peg.276	fig|6666666.59348.peg.3893	fig|6666666.59349.peg.657	fig|6666666.59350.peg.4404	fig|6666666.59351.peg.4924	fig|6666666.59353.peg.1342	fig|6666666.59355.peg.3998	fig|6666666.59354.peg.2923

7560	TonB-dependent receptor	fig|6666666.59329.peg.5232	fig|6666666.59330.peg.1186	fig|6666666.59331.peg.2120	fig|6666666.59332.peg.1040	fig|6666666.59334.peg.4900	fig|6666666.59335.peg.683	fig|6666666.59336.peg.3235	fig|6666666.59333.peg.3516	fig|6666666.59338.peg.979	fig|6666666.59339.peg.6259	fig|6666666.59340.peg.227	fig|6666666.59337.peg.4065	fig|6666666.59342.peg.4900	fig|6666666.71181.peg.3594	fig|6666666.59344.peg.4788	fig|6666666.59345.peg.737	fig|6666666.59341.peg.3172	fig|6666666.59347.peg.609	fig|6666666.59346.peg.193	fig|6666666.59348.peg.117	fig|6666666.59349.peg.3946	fig|6666666.59350.peg.882	fig|6666666.59351.peg.905	fig|6666666.59353.peg.1731	fig|6666666.59355.peg.3168	fig|6666666.59354.peg.2803

10128	Peroxiredoxin family protein glutaredoxin	fig|6666666.59329.peg.4897	fig|6666666.59330.peg.1688	fig|6666666.59331.peg.141	fig|6666666.59332.peg.2178	fig|6666666.59334.peg.3397	fig|6666666.59335.peg.2538	fig|6666666.59336.peg.1216	fig|6666666.59333.peg.1853	fig|6666666.59338.peg.2081	fig|6666666.59339.peg.3512	fig|6666666.59340.peg.1504	fig|6666666.59337.peg.1692	fig|6666666.59342.peg.187	fig|6666666.71181.peg.4022	fig|6666666.59344.peg.355	fig|6666666.59345.peg.3943	fig|6666666.59341.peg.959	fig|6666666.59347.peg.1498	fig|6666666.59346.peg.2864	fig|6666666.59348.peg.2447	fig|6666666.59349.peg.2488	fig|6666666.59350.peg.34	fig|6666666.59351.peg.2922	fig|6666666.59353.peg.2521	fig|6666666.59355.peg.2635	fig|6666666.59354.peg.65

10127	Membrane fusion component of tripartite multidrug resistance system	fig|6666666.59329.peg.2225	fig|6666666.59330.peg.4111	fig|6666666.59331.peg.1096	fig|6666666.59332.peg.2774	fig|6666666.59334.peg.411	fig|6666666.59335.peg.2251	fig|6666666.59336.peg.794	fig|6666666.59333.peg.2245	fig|6666666.59338.peg.5738	fig|6666666.59339.peg.524	fig|6666666.59340.peg.2034	fig|6666666.59337.peg.3763	fig|6666666.59342.peg.1882	fig|6666666.71181.peg.808	fig|6666666.59344.peg.2000	fig|6666666.59345.peg.4011	fig|6666666.59341.peg.1081	fig|6666666.59347.peg.3076	fig|6666666.59346.peg.3757	fig|6666666.59348.peg.1359	fig|6666666.59349.peg.368	fig|6666666.59350.peg.4069	fig|6666666.59351.peg.2382	fig|6666666.59353.peg.4362	fig|6666666.59355.peg.4905	fig|6666666.59354.peg.1256

5933	Transcriptional regulator, AsnC family	fig|6666666.59329.peg.1908	fig|6666666.59330.peg.2154	fig|6666666.59331.peg.2073	fig|6666666.59332.peg.4355	fig|6666666.59334.peg.1189	fig|6666666.59335.peg.5910	fig|6666666.59336.peg.5292	fig|6666666.59333.peg.3393	fig|6666666.59338.peg.72	fig|6666666.59339.peg.7542	fig|6666666.59340.peg.1618	fig|6666666.59337.peg.5621	fig|6666666.59342.peg.1641	fig|6666666.71181.peg.587	fig|6666666.59344.peg.4259	fig|6666666.59345.peg.519	fig|6666666.59341.peg.4147	fig|6666666.59347.peg.4568	fig|6666666.59346.peg.4653	fig|6666666.59348.peg.937	fig|6666666.59349.peg.4707	fig|6666666.59350.peg.1414	fig|6666666.59351.peg.312	fig|6666666.59353.peg.3093	fig|6666666.59355.peg.2255	fig|6666666.59354.peg.500

2090	Topoisomerase IV subunit A EC 5.99.1.-	fig|6666666.59329.peg.2740	fig|6666666.59330.peg.2315	fig|6666666.59331.peg.3191	fig|6666666.59332.peg.1428	fig|6666666.59334.peg.1399	fig|6666666.59335.peg.3443	fig|6666666.59336.peg.1582	fig|6666666.59333.peg.4631	fig|6666666.59338.peg.5290	fig|6666666.59339.peg.945	fig|6666666.59340.peg.1448	fig|6666666.59337.peg.1756	fig|6666666.59342.peg.1083	fig|6666666.71181.peg.3653	fig|6666666.59344.peg.1723	fig|6666666.59345.peg.3820	fig|6666666.59341.peg.1726	fig|6666666.59347.peg.3915	fig|6666666.59346.peg.1638	fig|6666666.59348.peg.4318	fig|6666666.59349.peg.381	fig|6666666.59350.peg.138	fig|6666666.59351.peg.937	fig|6666666.59353.peg.4929	fig|6666666.59355.peg.4973	fig|6666666.59354.peg.1759

7020	Fumarylacetoacetate hydrolase family protein	fig|6666666.59329.peg.4106	fig|6666666.59330.peg.3232	fig|6666666.59331.peg.4734	fig|6666666.59332.peg.4545	fig|6666666.59334.peg.2344	fig|6666666.59335.peg.6174	fig|6666666.59336.peg.2959	fig|6666666.59333.peg.4724	fig|6666666.59338.peg.3702	fig|6666666.59339.peg.4139	fig|6666666.59340.peg.79	fig|6666666.59337.peg.1969	fig|6666666.59342.peg.5320	fig|6666666.71181.peg.4976	fig|6666666.59344.peg.1404	fig|6666666.59345.peg.3082	fig|6666666.59341.peg.5868	fig|6666666.59347.peg.1324	fig|6666666.59346.peg.4105	fig|6666666.59348.peg.1329	fig|6666666.59349.peg.3963	fig|6666666.59350.peg.2363	fig|6666666.59351.peg.4202	fig|6666666.59353.peg.17	fig|6666666.59355.peg.610	fig|6666666.59354.peg.6077

3330	Ketol-acid reductoisomerase EC 1.1.1.86	fig|6666666.59329.peg.3747	fig|6666666.59330.peg.4115	fig|6666666.59331.peg.4019	fig|6666666.59332.peg.2596	fig|6666666.59334.peg.703	fig|6666666.59335.peg.59	fig|6666666.59336.peg.4919	fig|6666666.59333.peg.1858	fig|6666666.59338.peg.2529	fig|6666666.59339.peg.7602	fig|6666666.59340.peg.1179	fig|6666666.59337.peg.5758	fig|6666666.59342.peg.5376	fig|6666666.71181.peg.3082	fig|6666666.59344.peg.4219	fig|6666666.59345.peg.5719	fig|6666666.59341.peg.2582	fig|6666666.59347.peg.5227	fig|6666666.59346.peg.2727	fig|6666666.59348.peg.1030	fig|6666666.59349.peg.3328	fig|6666666.59350.peg.5983	fig|6666666.59351.peg.616	fig|6666666.59353.peg.4259	fig|6666666.59355.peg.4337	fig|6666666.59354.peg.4533

1038	Gamma-glutamyl phosphate reductase EC 1.2.1.41	fig|6666666.59329.peg.5361	fig|6666666.59330.peg.1063	fig|6666666.59331.peg.6149	fig|6666666.59332.peg.5765	fig|6666666.59334.peg.5617	fig|6666666.59335.peg.148	fig|6666666.59336.peg.2495	fig|6666666.59333.peg.3423	fig|6666666.59338.peg.4163	fig|6666666.59339.peg.5089	fig|6666666.59340.peg.1141	fig|6666666.59337.peg.1143	fig|6666666.59342.peg.5143	fig|6666666.71181.peg.932	fig|6666666.59344.peg.3535	fig|6666666.59345.peg.3544	fig|6666666.59341.peg.2603	fig|6666666.59347.peg.2778	fig|6666666.59346.peg.3377	fig|6666666.59348.peg.4350	fig|6666666.59349.peg.4675	fig|6666666.59350.peg.2555	fig|6666666.59351.peg.3103	fig|6666666.59353.peg.882	fig|6666666.59355.peg.822	fig|6666666.59354.peg.4238

4378	ATP-dependent Clp protease ATP-binding subunit ClpX	fig|6666666.59329.peg.4571	fig|6666666.59330.peg.295	fig|6666666.59331.peg.688	fig|6666666.59332.peg.1189	fig|6666666.59334.peg.617	fig|6666666.59335.peg.435	fig|6666666.59336.peg.4121	fig|6666666.59333.peg.2636	fig|6666666.59338.peg.2551	fig|6666666.59339.peg.3098	fig|6666666.59340.peg.26	fig|6666666.59337.peg.1025	fig|6666666.59342.peg.5125	fig|6666666.71181.peg.2053	fig|6666666.59344.peg.1067	fig|6666666.59345.peg.4256	fig|6666666.59341.peg.4140	fig|6666666.59347.peg.2657	fig|6666666.59346.peg.2662	fig|6666666.59348.peg.155	fig|6666666.59349.peg.4767	fig|6666666.59350.peg.1190	fig|6666666.59351.peg.3830	fig|6666666.59353.peg.2541	fig|6666666.59355.peg.952	fig|6666666.59354.peg.937

8665	LSU ribosomal protein L33p LSU ribosomal protein L33p, zinc-independent	fig|6666666.59329.peg.4986	fig|6666666.59330.peg.1167	fig|6666666.59331.peg.4156	fig|6666666.59332.peg.296	fig|6666666.59334.peg.185	fig|6666666.59335.peg.1995	fig|6666666.59336.peg.54	fig|6666666.59333.peg.654	fig|6666666.59338.peg.1112	fig|6666666.59339.peg.6291	fig|6666666.59340.peg.8	fig|6666666.59337.peg.472	fig|6666666.59342.peg.5883	fig|6666666.71181.peg.3655	fig|6666666.59344.peg.942	fig|6666666.59345.peg.2619	fig|6666666.59341.peg.206	fig|6666666.59347.peg.4521	fig|6666666.59346.peg.1713	fig|6666666.59348.peg.724	fig|6666666.59349.peg.113	fig|6666666.59350.peg.1403	fig|6666666.59351.peg.169	fig|6666666.59353.peg.2591	fig|6666666.59355.peg.2610	fig|6666666.59354.peg.3706

11326	Ribosome-binding factor A	fig|6666666.59329.peg.1894	fig|6666666.59330.peg.105	fig|6666666.59331.peg.2109	fig|6666666.59332.peg.3549	fig|6666666.59334.peg.777	fig|6666666.59335.peg.2099	fig|6666666.59336.peg.2425	fig|6666666.59333.peg.2324	fig|6666666.59338.peg.3435	fig|6666666.59339.peg.4897	fig|6666666.59340.peg.953	fig|6666666.59337.peg.685	fig|6666666.59342.peg.379	fig|6666666.71181.peg.4099	fig|6666666.59344.peg.2098	fig|6666666.59345.peg.2355	fig|6666666.59341.peg.2441	fig|6666666.59347.peg.3003	fig|6666666.59346.peg.300	fig|6666666.59348.peg.2155	fig|6666666.59349.peg.1435	fig|6666666.59350.peg.921	fig|6666666.59351.peg.1388	fig|6666666.59353.peg.3131	fig|6666666.59355.peg.465	fig|6666666.59354.peg.2276

8087	DNA polymerase III alpha subunit EC 2.7.7.7	fig|6666666.59329.peg.5821	fig|6666666.59330.peg.1395	fig|6666666.59331.peg.6236	fig|6666666.59332.peg.420	fig|6666666.59334.peg.357	fig|6666666.59335.peg.2667	fig|6666666.59336.peg.3535	fig|6666666.59333.peg.4891	fig|6666666.59338.peg.2677	fig|6666666.59339.peg.5128	fig|6666666.59340.peg.2602	fig|6666666.59337.peg.2660	fig|6666666.59342.peg.5542	fig|6666666.71181.peg.20	fig|6666666.59344.peg.2669	fig|6666666.59345.peg.2186	fig|6666666.59341.peg.987	fig|6666666.59347.peg.4373	fig|6666666.59346.peg.4439	fig|6666666.59348.peg.3631	fig|6666666.59349.peg.1360	fig|6666666.59350.peg.7034	fig|6666666.59351.peg.1432	fig|6666666.59353.peg.2000	fig|6666666.59355.peg.4105	fig|6666666.59354.peg.5671

6647	Chitinase EC 3.2.1.14	fig|6666666.59329.peg.2089	fig|6666666.59330.peg.4152	fig|6666666.59331.peg.1783	fig|6666666.59332.peg.5854	fig|6666666.59334.peg.745	fig|6666666.59335.peg.5696	fig|6666666.59336.peg.3586	fig|6666666.59333.peg.1795	fig|6666666.59338.peg.3322	fig|6666666.59339.peg.6596	fig|6666666.59340.peg.1287	fig|6666666.59337.peg.5157	fig|6666666.59342.peg.2639	fig|6666666.71181.peg.3203	fig|6666666.59344.peg.3502	fig|6666666.59345.peg.3292	fig|6666666.59341.peg.6115	fig|6666666.59347.peg.2373	fig|6666666.59346.peg.2843	fig|6666666.59348.peg.1117	fig|6666666.59349.peg.1473	fig|6666666.59350.peg.2117	fig|6666666.59351.peg.3335	fig|6666666.59353.peg.2949	fig|6666666.59355.peg.2491	fig|6666666.59354.peg.956

7803	Septum formation protein Maf	fig|6666666.59329.peg.708	fig|6666666.59330.peg.27	fig|6666666.59331.peg.829	fig|6666666.59332.peg.2166	fig|6666666.59334.peg.1311	fig|6666666.59335.peg.3029	fig|6666666.59336.peg.5710	fig|6666666.59333.peg.3651	fig|6666666.59338.peg.5764	fig|6666666.59339.peg.7792	fig|6666666.59340.peg.609	fig|6666666.59337.peg.812	fig|6666666.59342.peg.3381	fig|6666666.71181.peg.981	fig|6666666.59344.peg.1745	fig|6666666.59345.peg.1069	fig|6666666.59341.peg.886	fig|6666666.59347.peg.3113	fig|6666666.59346.peg.2130	fig|6666666.59348.peg.5347	fig|6666666.59349.peg.7	fig|6666666.59350.peg.5719	fig|6666666.59351.peg.667	fig|6666666.59353.peg.2295	fig|6666666.59355.peg.1571	fig|6666666.59354.peg.3167

7806	Transcriptional regulator, TetR family	fig|6666666.59329.peg.5218	fig|6666666.59330.peg.1494	fig|6666666.59331.peg.1109	fig|6666666.59332.peg.3608	fig|6666666.59334.peg.1780	fig|6666666.59335.peg.65	fig|6666666.59336.peg.2711	fig|6666666.59333.peg.3025	fig|6666666.59338.peg.4307	fig|6666666.59339.peg.7975	fig|6666666.59340.peg.322	fig|6666666.59337.peg.5258	fig|6666666.59342.peg.3170	fig|6666666.71181.peg.6137	fig|6666666.59344.peg.1267	fig|6666666.59345.peg.638	fig|6666666.59341.peg.4548	fig|6666666.59347.peg.506	fig|6666666.59346.peg.2732	fig|6666666.59348.peg.2472	fig|6666666.59349.peg.413	fig|6666666.59350.peg.2682	fig|6666666.59351.peg.1823	fig|6666666.59353.peg.3348	fig|6666666.59355.peg.3542	fig|6666666.59354.peg.1253

6399	SSU ribosomal protein S11p S14e	fig|6666666.59329.peg.731	fig|6666666.59330.peg.574	fig|6666666.59331.peg.29	fig|6666666.59332.peg.5647	fig|6666666.59334.peg.3042	fig|6666666.59335.peg.4752	fig|6666666.59336.peg.164	fig|6666666.59333.peg.386	fig|6666666.59338.peg.4738	fig|6666666.59339.peg.8288	fig|6666666.59340.peg.2222	fig|6666666.59337.peg.369	fig|6666666.59342.peg.324	fig|6666666.71181.peg.3441	fig|6666666.59344.peg.4924	fig|6666666.59345.peg.2516	fig|6666666.59341.peg.774	fig|6666666.59347.peg.4843	fig|6666666.59346.peg.869	fig|6666666.59348.peg.435	fig|6666666.59349.peg.3289	fig|6666666.59350.peg.1553	fig|6666666.59351.peg.4224	fig|6666666.59353.peg.4609	fig|6666666.59355.peg.4722	fig|6666666.59354.peg.3248

6396	Long-chain-fatty-acid--CoA ligase EC 6.2.1.3	fig|6666666.59329.peg.4545	fig|6666666.59330.peg.4574	fig|6666666.59331.peg.1685	fig|6666666.59332.peg.1141	fig|6666666.59334.peg.1464	fig|6666666.59335.peg.3824	fig|6666666.59336.peg.969	fig|6666666.59333.peg.1433	fig|6666666.59338.peg.933	fig|6666666.59339.peg.4030	fig|6666666.59340.peg.3010	fig|6666666.59337.peg.4521	fig|6666666.59342.peg.5220	fig|6666666.71181.peg.766	fig|6666666.59344.peg.5779	fig|6666666.59345.peg.4280	fig|6666666.59341.peg.3309	fig|6666666.59347.peg.2960	fig|6666666.59346.peg.4925	fig|6666666.59348.peg.1524	fig|6666666.59349.peg.3146	fig|6666666.59350.peg.3622	fig|6666666.59351.peg.5340	fig|6666666.59353.peg.3271	fig|6666666.59355.peg.335	fig|6666666.59354.peg.2355

1855	Methylated-DNA--protein-cysteine methyltransferase EC 2.1.1.63	fig|6666666.59329.peg.467	fig|6666666.59330.peg.1829	fig|6666666.59331.peg.2865	fig|6666666.59332.peg.964	fig|6666666.59334.peg.3322	fig|6666666.59335.peg.878	fig|6666666.59336.peg.5136	fig|6666666.59333.peg.4450	fig|6666666.59338.peg.2057	fig|6666666.59339.peg.3266	fig|6666666.59340.peg.2275	fig|6666666.59337.peg.190	fig|6666666.59342.peg.6155	fig|6666666.71181.peg.2070	fig|6666666.59344.peg.1045	fig|6666666.59345.peg.4520	fig|6666666.59341.peg.2344	fig|6666666.59347.peg.2552	fig|6666666.59346.peg.989	fig|6666666.59348.peg.4718	fig|6666666.59349.peg.948	fig|6666666.59350.peg.1319	fig|6666666.59351.peg.1598	fig|6666666.59353.peg.734	fig|6666666.59355.peg.971	fig|6666666.59354.peg.3046

4961	DNA-directed RNA polymerase beta subunit EC 2.7.7.6	fig|6666666.59329.peg.627	fig|6666666.59330.peg.3409	fig|6666666.59331.peg.438	fig|6666666.59332.peg.141	fig|6666666.59334.peg.1232	fig|6666666.59335.peg.5926	fig|6666666.59336.peg.1684	fig|6666666.59333.peg.4392	fig|6666666.59338.peg.2257	fig|6666666.59339.peg.8837	fig|6666666.59340.peg.1327	fig|6666666.59337.peg.4991	fig|6666666.59342.peg.366	fig|6666666.71181.peg.3369	fig|6666666.59344.peg.5461	fig|6666666.59345.peg.5653	fig|6666666.59341.peg.3794	fig|6666666.59347.peg.5397	fig|6666666.59346.peg.1130	fig|6666666.59348.peg.2725	fig|6666666.59349.peg.1141	fig|6666666.59350.peg.7464	fig|6666666.59351.peg.5243	fig|6666666.59353.peg.4454	fig|6666666.59355.peg.4574	fig|6666666.59354.peg.4828

7944	Putative deoxyribonuclease YcfH	fig|6666666.59329.peg.2097	fig|6666666.59330.peg.86	fig|6666666.59331.peg.2653	fig|6666666.59332.peg.12	fig|6666666.59334.peg.799	fig|6666666.59335.peg.3636	fig|6666666.59336.peg.4266	fig|6666666.59333.peg.2618	fig|6666666.59338.peg.609	fig|6666666.59339.peg.5010	fig|6666666.59340.peg.494	fig|6666666.59337.peg.2680	fig|6666666.59342.peg.5178	fig|6666666.71181.peg.1630	fig|6666666.59344.peg.3409	fig|6666666.59345.peg.2708	fig|6666666.59341.peg.24	fig|6666666.59347.peg.2973	fig|6666666.59346.peg.1996	fig|6666666.59348.peg.2223	fig|6666666.59349.peg.4684	fig|6666666.59350.peg.6084	fig|6666666.59351.peg.4994	fig|6666666.59353.peg.3803	fig|6666666.59355.peg.3399	fig|6666666.59354.peg.3338

7342	2',3'-cyclic-nucleotide 2'-phosphodiesterase EC 3.1.4.16	fig|6666666.59329.peg.4952	fig|6666666.59330.peg.586	fig|6666666.59331.peg.2390	fig|6666666.59332.peg.3152	fig|6666666.59334.peg.3885	fig|6666666.59335.peg.4590	fig|6666666.59336.peg.4067	fig|6666666.59333.peg.3777	fig|6666666.59338.peg.4862	fig|6666666.59339.peg.8303	fig|6666666.59340.peg.957	fig|6666666.59337.peg.5598	fig|6666666.59342.peg.1451	fig|6666666.71181.peg.3240	fig|6666666.59344.peg.5046	fig|6666666.59345.peg.2503	fig|6666666.59341.peg.5745	fig|6666666.59347.peg.4852	fig|6666666.59346.peg.2897	fig|6666666.59348.peg.425	fig|6666666.59349.peg.3211	fig|6666666.59350.peg.7936	fig|6666666.59351.peg.2765	fig|6666666.59353.peg.4064	fig|6666666.59355.peg.2732	fig|6666666.59354.peg.3003

7008	Cysteine synthase EC 2.5.1.47	fig|6666666.59329.peg.1024	fig|6666666.59330.peg.1833	fig|6666666.59331.peg.4119	fig|6666666.59332.peg.112	fig|6666666.59334.peg.3820	fig|6666666.59335.peg.3020	fig|6666666.59336.peg.5620	fig|6666666.59333.peg.4940	fig|6666666.59338.peg.2016	fig|6666666.59339.peg.4743	fig|6666666.59340.peg.3300	fig|6666666.59337.peg.904	fig|6666666.59342.peg.1559	fig|6666666.71181.peg.5158	fig|6666666.59344.peg.330	fig|6666666.59345.peg.4391	fig|6666666.59341.peg.2124	fig|6666666.59347.peg.4769	fig|6666666.59346.peg.2379	fig|6666666.59348.peg.2214	fig|6666666.59349.peg.3451	fig|6666666.59350.peg.262	fig|6666666.59351.peg.3977	fig|6666666.59353.peg.3501	fig|6666666.59355.peg.5458	fig|6666666.59354.peg.5926

7005	D-alanyl-D-alanine carboxypeptidase EC 3.4.16.4	fig|6666666.59329.peg.3466	fig|6666666.59330.peg.2033	fig|6666666.59331.peg.371	fig|6666666.59332.peg.2691	fig|6666666.59334.peg.5470	fig|6666666.59335.peg.1759	fig|6666666.59336.peg.2720	fig|6666666.59333.peg.2215	fig|6666666.59338.peg.6	fig|6666666.59339.peg.6733	fig|6666666.59340.peg.401	fig|6666666.59337.peg.2125	fig|6666666.59342.peg.3985	fig|6666666.71181.peg.5517	fig|6666666.59344.peg.4878	fig|6666666.59345.peg.3938	fig|6666666.59341.peg.1101	fig|6666666.59347.peg.3449	fig|6666666.59346.peg.4248	fig|6666666.59348.peg.853	fig|6666666.59349.peg.2767	fig|6666666.59350.peg.1947	fig|6666666.59351.peg.3620	fig|6666666.59353.peg.3717	fig|6666666.59355.peg.3732	fig|6666666.59354.peg.5940

740	Chaperone protein DnaK	fig|6666666.59329.peg.1107	fig|6666666.59330.peg.4618	fig|6666666.59331.peg.1539	fig|6666666.59332.peg.1332	fig|6666666.59334.peg.3068	fig|6666666.59335.peg.5649	fig|6666666.59336.peg.3608	fig|6666666.59333.peg.3458	fig|6666666.59338.peg.5774	fig|6666666.59339.peg.7270	fig|6666666.59340.peg.2025	fig|6666666.59337.peg.743	fig|6666666.59342.peg.3687	fig|6666666.71181.peg.426	fig|6666666.59344.peg.1327	fig|6666666.59345.peg.3571	fig|6666666.59341.peg.4640	fig|6666666.59347.peg.4331	fig|6666666.59346.peg.5154	fig|6666666.59348.peg.1373	fig|6666666.59349.peg.4425	fig|6666666.59350.peg.164	fig|6666666.59351.peg.1548	fig|6666666.59353.peg.72	fig|6666666.59355.peg.679	fig|6666666.59354.peg.4573

2371	Phosphoribosyl-AMP cyclohydrolase EC 3.5.4.19 Phosphoribosyl-ATP pyrophosphatase EC 3.6.1.31	fig|6666666.59329.peg.1015	fig|6666666.59330.peg.1508	fig|6666666.59331.peg.3901	fig|6666666.59332.peg.1808	fig|6666666.59334.peg.3969	fig|6666666.59335.peg.586	fig|6666666.59336.peg.5022	fig|6666666.59333.peg.2721	fig|6666666.59338.peg.2160	fig|6666666.59339.peg.502	fig|6666666.59340.peg.3227	fig|6666666.59337.peg.4874	fig|6666666.59342.peg.2818	fig|6666666.71181.peg.243	fig|6666666.59344.peg.304	fig|6666666.59345.peg.1301	fig|6666666.59341.peg.504	fig|6666666.59347.peg.4150	fig|6666666.59346.peg.2692	fig|6666666.59348.peg.4651	fig|6666666.59349.peg.453	fig|6666666.59350.peg.2741	fig|6666666.59351.peg.1619	fig|6666666.59353.peg.576	fig|6666666.59355.peg.60	fig|6666666.59354.peg.898

1874	Biotin-protein ligase EC 6.3.4.15 Biotin operon repressor	fig|6666666.59329.peg.2428	fig|6666666.59330.peg.4557	fig|6666666.59331.peg.3632	fig|6666666.59332.peg.3161	fig|6666666.59334.peg.2120	fig|6666666.59335.peg.413	fig|6666666.59336.peg.3398	fig|6666666.59333.peg.5076	fig|6666666.59338.peg.1496	fig|6666666.59339.peg.6359	fig|6666666.59340.peg.3083	fig|6666666.59337.peg.5511	fig|6666666.59342.peg.1707	fig|6666666.71181.peg.4965	fig|6666666.59344.peg.6194	fig|6666666.59345.peg.5711	fig|6666666.59341.peg.5748	fig|6666666.59347.peg.5030	fig|6666666.59346.peg.4267	fig|6666666.59348.peg.25	fig|6666666.59349.peg.3360	fig|6666666.59350.peg.2608	fig|6666666.59351.peg.5023	fig|6666666.59353.peg.5081	fig|6666666.59355.peg.5133	fig|6666666.59354.peg.1664

884	Ribulose-phosphate 3-epimerase EC 5.1.3.1	fig|6666666.59329.peg.319	fig|6666666.59330.peg.2639	fig|6666666.59331.peg.4272	fig|6666666.59332.peg.3496	fig|6666666.59334.peg.4996	fig|6666666.59335.peg.2481	fig|6666666.59336.peg.593	fig|6666666.59333.peg.892	fig|6666666.59338.peg.3574	fig|6666666.59339.peg.8710	fig|6666666.59340.peg.70	fig|6666666.59337.peg.1705	fig|6666666.59342.peg.1890	fig|6666666.71181.peg.1828	fig|6666666.59344.peg.3070	fig|6666666.59345.peg.5377	fig|6666666.59341.peg.4076	fig|6666666.59347.peg.4948	fig|6666666.59346.peg.2353	fig|6666666.59348.peg.1027	fig|6666666.59349.peg.534	fig|6666666.59350.peg.985	fig|6666666.59351.peg.3857	fig|6666666.59353.peg.2222	fig|6666666.59355.peg.1916	fig|6666666.59354.peg.3568

6868	Imidazole glycerol phosphate synthase cyclase subunit EC 4.1.3.-	fig|6666666.59329.peg.1014	fig|6666666.59330.peg.1507	fig|6666666.59331.peg.4927	fig|6666666.59332.peg.1810	fig|6666666.59334.peg.3966	fig|6666666.59335.peg.588	fig|6666666.59336.peg.4089	fig|6666666.59333.peg.2720	fig|6666666.59338.peg.2161	fig|6666666.59339.peg.499	fig|6666666.59340.peg.1345	fig|6666666.59337.peg.4873	fig|6666666.59342.peg.2820	fig|6666666.71181.peg.244	fig|6666666.59344.peg.307	fig|6666666.59345.peg.1302	fig|6666666.59341.peg.502	fig|6666666.59347.peg.4149	fig|6666666.59346.peg.2690	fig|6666666.59348.peg.4650	fig|6666666.59349.peg.455	fig|6666666.59350.peg.2742	fig|6666666.59351.peg.1620	fig|6666666.59353.peg.575	fig|6666666.59355.peg.59	fig|6666666.59354.peg.896

9888	Apolipoprotein N-acyltransferase EC 2.3.1.- Copper homeostasis protein CutE	fig|6666666.59329.peg.2172	fig|6666666.59330.peg.4038	fig|6666666.59331.peg.1658	fig|6666666.59332.peg.4719	fig|6666666.59334.peg.3144	fig|6666666.59335.peg.1747	fig|6666666.59336.peg.2830	fig|6666666.59333.peg.2143	fig|6666666.59338.peg.2015	fig|6666666.59339.peg.5217	fig|6666666.59340.peg.2107	fig|6666666.59337.peg.1080	fig|6666666.59342.peg.1138	fig|6666666.71181.peg.3212	fig|6666666.59344.peg.4511	fig|6666666.59345.peg.3487	fig|6666666.59341.peg.4505	fig|6666666.59347.peg.4447	fig|6666666.59346.peg.2955	fig|6666666.59348.peg.1290	fig|6666666.59349.peg.4516	fig|6666666.59350.peg.573	fig|6666666.59351.peg.4675	fig|6666666.59353.peg.2034	fig|6666666.59355.peg.4441	fig|6666666.59354.peg.3234

665	Beta-ketoadipate enol-lactone hydrolase EC 3.1.1.24	fig|6666666.59329.peg.3411	fig|6666666.59330.peg.432	fig|6666666.59331.peg.2744	fig|6666666.59332.peg.943	fig|6666666.59334.peg.5618	fig|6666666.59335.peg.1648	fig|6666666.59336.peg.3819	fig|6666666.59333.peg.2547	fig|6666666.59338.peg.5909	fig|6666666.59339.peg.1639	fig|6666666.59340.peg.1743	fig|6666666.59337.peg.4261	fig|6666666.59342.peg.411	fig|6666666.71181.peg.1357	fig|6666666.59344.peg.6202	fig|6666666.59345.peg.2662	fig|6666666.59341.peg.701	fig|6666666.59347.peg.2383	fig|6666666.59346.peg.1479	fig|6666666.59348.peg.1058	fig|6666666.59349.peg.1385	fig|6666666.59350.peg.551	fig|6666666.59351.peg.4363	fig|6666666.59353.peg.2059	fig|6666666.59355.peg.3057	fig|6666666.59354.peg.4093

8433	Cytochrome c oxidase polypeptide I EC 1.9.3.1	fig|6666666.59329.peg.3772	fig|6666666.59330.peg.2617	fig|6666666.59331.peg.2216	fig|6666666.59332.peg.5535	fig|6666666.59334.peg.4527	fig|6666666.59335.peg.2873	fig|6666666.59336.peg.3128	fig|6666666.59333.peg.5066	fig|6666666.59338.peg.1543	fig|6666666.59339.peg.6640	fig|6666666.59340.peg.3369	fig|6666666.59337.peg.5085	fig|6666666.59342.peg.1726	fig|6666666.71181.peg.2044	fig|6666666.59344.peg.1077	fig|6666666.59345.peg.5614	fig|6666666.59341.peg.4657	fig|6666666.59347.peg.1553	fig|6666666.59346.peg.1536	fig|6666666.59348.peg.1401	fig|6666666.59349.peg.631	fig|6666666.59350.peg.6617	fig|6666666.59351.peg.4388	fig|6666666.59353.peg.761	fig|6666666.59355.peg.5540	fig|6666666.59354.peg.2412

8437	Acetate kinase EC 2.7.2.1	fig|6666666.59329.peg.5571	fig|6666666.59330.peg.3527	fig|6666666.59331.peg.1708	fig|6666666.59332.peg.1743	fig|6666666.59334.peg.1652	fig|6666666.59335.peg.1294	fig|6666666.59336.peg.6566	fig|6666666.59333.peg.4772	fig|6666666.59338.peg.3066	fig|6666666.59339.peg.3422	fig|6666666.59340.peg.2185	fig|6666666.59337.peg.1442	fig|6666666.59342.peg.5570	fig|6666666.71181.peg.552	fig|6666666.59344.peg.1914	fig|6666666.59345.peg.1099	fig|6666666.59341.peg.3138	fig|6666666.59347.peg.1215	fig|6666666.59346.peg.547	fig|6666666.59348.peg.4445	fig|6666666.59349.peg.2951	fig|6666666.59350.peg.1337	fig|6666666.59351.peg.2822	fig|6666666.59353.peg.1182	fig|6666666.59355.peg.293	fig|6666666.59354.peg.4252

9821	ATP synthase delta chain EC 3.6.3.14	fig|6666666.59329.peg.3123	fig|6666666.59330.peg.608	fig|6666666.59331.peg.403	fig|6666666.59332.peg.102	fig|6666666.59334.peg.2496	fig|6666666.59335.peg.795	fig|6666666.59336.peg.31	fig|6666666.59333.peg.26	fig|6666666.59338.peg.4018	fig|6666666.59339.peg.2292	fig|6666666.59340.peg.1172	fig|6666666.59337.peg.893	fig|6666666.59342.peg.1199	fig|6666666.71181.peg.595	fig|6666666.59344.peg.4268	fig|6666666.59345.peg.4962	fig|6666666.59341.peg.2138	fig|6666666.59347.peg.2036	fig|6666666.59346.peg.1406	fig|6666666.59348.peg.335	fig|6666666.59349.peg.1033	fig|6666666.59350.peg.1833	fig|6666666.59351.peg.5356	fig|6666666.59353.peg.3085	fig|6666666.59355.peg.2263	fig|6666666.59354.peg.1155

5971	Putative regulator protein	fig|6666666.59329.peg.4298	fig|6666666.59330.peg.3156	fig|6666666.59331.peg.2917	fig|6666666.59332.peg.3596	fig|6666666.59334.peg.680	fig|6666666.59335.peg.1052	fig|6666666.59336.peg.4199	fig|6666666.59333.peg.5589	fig|6666666.59338.peg.2766	fig|6666666.59339.peg.4370	fig|6666666.59340.peg.973	fig|6666666.59337.peg.1168	fig|6666666.59342.peg.6251	fig|6666666.71181.peg.584	fig|6666666.59344.peg.4256	fig|6666666.59345.peg.5643	fig|6666666.59341.peg.3282	fig|6666666.59347.peg.1233	fig|6666666.59346.peg.4516	fig|6666666.59348.peg.2804	fig|6666666.59349.peg.4069	fig|6666666.59350.peg.345	fig|6666666.59351.peg.703	fig|6666666.59353.peg.3096	fig|6666666.59355.peg.2252	fig|6666666.59354.peg.5242

5974	Predicted hydrolase of the metallo-beta-lactamase superfamily, clustered with KDO2-Lipid A biosynthesis genes	fig|6666666.59329.peg.3944	fig|6666666.59330.peg.1253	fig|6666666.59331.peg.4904	fig|6666666.59332.peg.4088	fig|6666666.59334.peg.5130	fig|6666666.59335.peg.2834	fig|6666666.59336.peg.4613	fig|6666666.59333.peg.1828	fig|6666666.59338.peg.2930	fig|6666666.59339.peg.3468	fig|6666666.59340.peg.489	fig|6666666.59337.peg.2197	fig|6666666.59342.peg.5661	fig|6666666.71181.peg.3169	fig|6666666.59344.peg.2798	fig|6666666.59345.peg.1592	fig|6666666.59341.peg.1850	fig|6666666.59347.peg.4103	fig|6666666.59346.peg.137	fig|6666666.59348.peg.4298	fig|6666666.59349.peg.2507	fig|6666666.59350.peg.4294	fig|6666666.59351.peg.3710	fig|6666666.59353.peg.1649	fig|6666666.59355.peg.1072	fig|6666666.59354.peg.2890

10264	LSU ribosomal protein L2p L8e	fig|6666666.59329.peg.6291	fig|6666666.59330.peg.553	fig|6666666.59331.peg.49	fig|6666666.59332.peg.3798	fig|6666666.59334.peg.1484	fig|6666666.59335.peg.4346	fig|6666666.59336.peg.144	fig|6666666.59333.peg.365	fig|6666666.59338.peg.1390	fig|6666666.59339.peg.8267	fig|6666666.59340.peg.54	fig|6666666.59337.peg.389	fig|6666666.59342.peg.303	fig|6666666.71181.peg.3457	fig|6666666.59344.peg.4940	fig|6666666.59345.peg.2537	fig|6666666.59341.peg.2497	fig|6666666.59347.peg.4822	fig|6666666.59346.peg.849	fig|6666666.59348.peg.413	fig|6666666.59349.peg.1097	fig|6666666.59350.peg.6236	fig|6666666.59351.peg.4815	fig|6666666.59353.peg.4593	fig|6666666.59355.peg.4738	fig|6666666.59354.peg.1748

10267	Polysaccharide export lipoprotein Wza	fig|6666666.59329.peg.548	fig|6666666.59330.peg.3964	fig|6666666.59331.peg.5639	fig|6666666.59332.peg.2136	fig|6666666.59334.peg.2791	fig|6666666.59335.peg.6096	fig|6666666.59336.peg.1102	fig|6666666.59333.peg.993	fig|6666666.59338.peg.1230	fig|6666666.59339.peg.4160	fig|6666666.59340.peg.1507	fig|6666666.59337.peg.3011	fig|6666666.59342.peg.2163	fig|6666666.71181.peg.3054	fig|6666666.59344.peg.264	fig|6666666.59345.peg.627	fig|6666666.59341.peg.742	fig|6666666.59347.peg.5169	fig|6666666.59346.peg.1749	fig|6666666.59348.peg.4200	fig|6666666.59349.peg.265	fig|6666666.59350.peg.5782	fig|6666666.59351.peg.228	fig|6666666.59353.peg.607	fig|6666666.59355.peg.89	fig|6666666.59354.peg.841

10853	Protein export cytoplasm protein SecA ATPase RNA helicase TC 3.A.5.1.1	fig|6666666.59329.peg.1365	fig|6666666.59330.peg.1285	fig|6666666.59331.peg.5542	fig|6666666.59332.peg.3773	fig|6666666.59334.peg.4056	fig|6666666.59335.peg.823	fig|6666666.59336.peg.959	fig|6666666.59333.peg.4205	fig|6666666.59338.peg.3157	fig|6666666.59339.peg.4982	fig|6666666.59340.peg.1284	fig|6666666.59337.peg.57	fig|6666666.59342.peg.3392	fig|6666666.71181.peg.1971	fig|6666666.59344.peg.1469	fig|6666666.59345.peg.3785	fig|6666666.59341.peg.3129	fig|6666666.59347.peg.4007	fig|6666666.59346.peg.1248	fig|6666666.59348.peg.254	fig|6666666.59349.peg.2994	fig|6666666.59350.peg.4538	fig|6666666.59351.peg.220	fig|6666666.59353.peg.148	fig|6666666.59355.peg.757	fig|6666666.59354.peg.5700

10707	LSU ribosomal protein L11p L12e	fig|6666666.59329.peg.623	fig|6666666.59330.peg.3405	fig|6666666.59331.peg.2753	fig|6666666.59332.peg.2886	fig|6666666.59334.peg.2474	fig|6666666.59335.peg.5626	fig|6666666.59336.peg.1680	fig|6666666.59333.peg.4396	fig|6666666.59338.peg.3289	fig|6666666.59339.peg.8847	fig|6666666.59340.peg.1352	fig|6666666.59337.peg.4986	fig|6666666.59342.peg.370	fig|6666666.71181.peg.5018	fig|6666666.59344.peg.5465	fig|6666666.59345.peg.5657	fig|6666666.59341.peg.5591	fig|6666666.59347.peg.5401	fig|6666666.59346.peg.4096	fig|6666666.59348.peg.5465	fig|6666666.59349.peg.1145	fig|6666666.59350.peg.3786	fig|6666666.59351.peg.5239	fig|6666666.59353.peg.4449	fig|6666666.59355.peg.4578	fig|6666666.59354.peg.4537

1795	ABC transporter, ATP-binding protein	fig|6666666.59329.peg.1802	fig|6666666.59330.peg.685	fig|6666666.59331.peg.2939	fig|6666666.59332.peg.825	fig|6666666.59334.peg.4909	fig|6666666.59335.peg.2652	fig|6666666.59336.peg.846	fig|6666666.59333.peg.3179	fig|6666666.59338.peg.1752	fig|6666666.59339.peg.547	fig|6666666.59340.peg.3457	fig|6666666.59337.peg.223	fig|6666666.59342.peg.1837	fig|6666666.71181.peg.4915	fig|6666666.59344.peg.3157	fig|6666666.59345.peg.672	fig|6666666.59341.peg.610	fig|6666666.59347.peg.142	fig|6666666.59346.peg.3836	fig|6666666.59348.peg.776	fig|6666666.59349.peg.4879	fig|6666666.59350.peg.6821	fig|6666666.59351.peg.4049	fig|6666666.59353.peg.3020	fig|6666666.59355.peg.5907	fig|6666666.59354.peg.981

3094	Alanyl-tRNA synthetase EC 6.1.1.7	fig|6666666.59329.peg.2803	fig|6666666.59330.peg.4217	fig|6666666.59331.peg.2089	fig|6666666.59332.peg.2078	fig|6666666.59334.peg.284	fig|6666666.59335.peg.264	fig|6666666.59336.peg.5563	fig|6666666.59333.peg.1723	fig|6666666.59338.peg.758	fig|6666666.59339.peg.8113	fig|6666666.59340.peg.1261	fig|6666666.59337.peg.5447	fig|6666666.59342.peg.3842	fig|6666666.71181.peg.3469	fig|6666666.59344.peg.2167	fig|6666666.59345.peg.5080	fig|6666666.59341.peg.4217	fig|6666666.59347.peg.3226	fig|6666666.59346.peg.4972	fig|6666666.59348.peg.1343	fig|6666666.59349.peg.1831	fig|6666666.59350.peg.7458	fig|6666666.59351.peg.5264	fig|6666666.59353.peg.232	fig|6666666.59355.peg.3003	fig|6666666.59354.peg.597

4104	Diaminobutyrate--2-oxoglutarate aminotransferase EC 2.6.1.76	fig|6666666.59329.peg.5627	fig|6666666.59330.peg.2944	fig|6666666.59331.peg.1966	fig|6666666.59332.peg.1010	fig|6666666.59334.peg.3858	fig|6666666.59335.peg.617	fig|6666666.59336.peg.278	fig|6666666.59333.peg.4579	fig|6666666.59338.peg.3041	fig|6666666.59339.peg.930	fig|6666666.59340.peg.1323	fig|6666666.59337.peg.2248	fig|6666666.59342.peg.4253	fig|6666666.71181.peg.5664	fig|6666666.59344.peg.3783	fig|6666666.59345.peg.4189	fig|6666666.59341.peg.355	fig|6666666.59347.peg.4954	fig|6666666.59346.peg.1062	fig|6666666.59348.peg.3473	fig|6666666.59349.peg.1622	fig|6666666.59350.peg.3861	fig|6666666.59351.peg.4297	fig|6666666.59353.peg.3309	fig|6666666.59355.peg.3144	fig|6666666.59354.peg.5688

3892	Lactoylglutathione lyase EC 4.4.1.5	fig|6666666.59329.peg.1849	fig|6666666.59330.peg.1228	fig|6666666.59331.peg.3724	fig|6666666.59332.peg.2569	fig|6666666.59334.peg.4176	fig|6666666.59335.peg.881	fig|6666666.59336.peg.5573	fig|6666666.59333.peg.4812	fig|6666666.59338.peg.4297	fig|6666666.59339.peg.51	fig|6666666.59340.peg.506	fig|6666666.59337.peg.4414	fig|6666666.59342.peg.1217	fig|6666666.71181.peg.2542	fig|6666666.59344.peg.3512	fig|6666666.59345.peg.3431	fig|6666666.59341.peg.2708	fig|6666666.59347.peg.5386	fig|6666666.59346.peg.4766	fig|6666666.59348.peg.85	fig|6666666.59349.peg.3589	fig|6666666.59350.peg.3808	fig|6666666.59351.peg.309	fig|6666666.59353.peg.1373	fig|6666666.59355.peg.2500	fig|6666666.59354.peg.5151

3891	UDP-N-acetylmuramoylalanyl-D-glutamate--2,6-diaminopimelate ligase EC 6.3.2.13	fig|6666666.59329.peg.2025	fig|6666666.59330.peg.1272	fig|6666666.59331.peg.3955	fig|6666666.59332.peg.3001	fig|6666666.59334.peg.5059	fig|6666666.59335.peg.835	fig|6666666.59336.peg.705	fig|6666666.59333.peg.571	fig|6666666.59338.peg.2626	fig|6666666.59339.peg.7730	fig|6666666.59340.peg.1191	fig|6666666.59337.peg.50	fig|6666666.59342.peg.3698	fig|6666666.71181.peg.1985	fig|6666666.59344.peg.1484	fig|6666666.59345.peg.3791	fig|6666666.59341.peg.1355	fig|6666666.59347.peg.4873	fig|6666666.59346.peg.653	fig|6666666.59348.peg.4024	fig|6666666.59349.peg.2981	fig|6666666.59350.peg.1039	fig|6666666.59351.peg.210	fig|6666666.59353.peg.136	fig|6666666.59355.peg.743	fig|6666666.59354.peg.3663

7389	Uroporphyrinogen III decarboxylase EC 4.1.1.37	fig|6666666.59329.peg.676	fig|6666666.59330.peg.3769	fig|6666666.59331.peg.3351	fig|6666666.59332.peg.137	fig|6666666.59334.peg.5292	fig|6666666.59335.peg.1946	fig|6666666.59336.peg.3842	fig|6666666.59333.peg.2188	fig|6666666.59338.peg.3035	fig|6666666.59339.peg.8831	fig|6666666.59340.peg.794	fig|6666666.59337.peg.4511	fig|6666666.59342.peg.4046	fig|6666666.71181.peg.3354	fig|6666666.59344.peg.5682	fig|6666666.59345.peg.5688	fig|6666666.59341.peg.1731	fig|6666666.59347.peg.5393	fig|6666666.59346.peg.1136	fig|6666666.59348.peg.1971	fig|6666666.59349.peg.3366	fig|6666666.59350.peg.4033	fig|6666666.59351.peg.4184	fig|6666666.59353.peg.4469	fig|6666666.59355.peg.4558	fig|6666666.59354.peg.1670

1452	Electron transport complex protein RnfA	fig|6666666.59329.peg.5221	fig|6666666.59330.peg.4049	fig|6666666.59331.peg.4891	fig|6666666.59332.peg.5656	fig|6666666.59334.peg.885	fig|6666666.59335.peg.890	fig|6666666.59336.peg.1328	fig|6666666.59333.peg.1802	fig|6666666.59338.peg.1623	fig|6666666.59339.peg.62	fig|6666666.59340.peg.659	fig|6666666.59337.peg.5096	fig|6666666.59342.peg.1805	fig|6666666.71181.peg.919	fig|6666666.59344.peg.5166	fig|6666666.59345.peg.1121	fig|6666666.59341.peg.2295	fig|6666666.59347.peg.5164	fig|6666666.59346.peg.5090	fig|6666666.59348.peg.1316	fig|6666666.59349.peg.303	fig|6666666.59350.peg.4833	fig|6666666.59351.peg.5079	fig|6666666.59353.peg.3999	fig|6666666.59355.peg.3916	fig|6666666.59354.peg.4979

3562	LSU ribosomal protein L5p L11e	fig|6666666.59329.peg.721	fig|6666666.59330.peg.562	fig|6666666.59331.peg.40	fig|6666666.59332.peg.2147	fig|6666666.59334.peg.1493	fig|6666666.59335.peg.4355	fig|6666666.59336.peg.154	fig|6666666.59333.peg.374	fig|6666666.59338.peg.1381	fig|6666666.59339.peg.8275	fig|6666666.59340.peg.64	fig|6666666.59337.peg.380	fig|6666666.59342.peg.312	fig|6666666.71181.peg.3448	fig|6666666.59344.peg.4931	fig|6666666.59345.peg.2527	fig|6666666.59341.peg.2506	fig|6666666.59347.peg.4831	fig|6666666.59346.peg.858	fig|6666666.59348.peg.1768	fig|6666666.59349.peg.3278	fig|6666666.59350.peg.3185	fig|6666666.59351.peg.4212	fig|6666666.59353.peg.4602	fig|6666666.59355.peg.4729	fig|6666666.59354.peg.3851

4678	Signal peptidase I EC 3.4.21.89	fig|6666666.59329.peg.502	fig|6666666.59330.peg.384	fig|6666666.59331.peg.5760	fig|6666666.59332.peg.729	fig|6666666.59334.peg.1877	fig|6666666.59335.peg.1488	fig|6666666.59336.peg.2101	fig|6666666.59333.peg.2808	fig|6666666.59338.peg.1514	fig|6666666.59339.peg.7369	fig|6666666.59340.peg.850	fig|6666666.59337.peg.564	fig|6666666.59342.peg.4453	fig|6666666.71181.peg.1424	fig|6666666.59344.peg.1565	fig|6666666.59345.peg.5054	fig|6666666.59341.peg.5368	fig|6666666.59347.peg.3177	fig|6666666.59346.peg.333	fig|6666666.59348.peg.650	fig|6666666.59349.peg.4317	fig|6666666.59350.peg.1613	fig|6666666.59351.peg.599	fig|6666666.59353.peg.2652	fig|6666666.59355.peg.1830	fig|6666666.59354.peg.2816

3377	Phosphate ABC transporter, periplasmic phosphate-binding protein PstS TC 3.A.1.7.1	fig|6666666.59329.peg.4009	fig|6666666.59330.peg.3808	fig|6666666.59331.peg.1050	fig|6666666.59332.peg.1269	fig|6666666.59334.peg.4029	fig|6666666.59335.peg.2531	fig|6666666.59336.peg.4849	fig|6666666.59333.peg.2087	fig|6666666.59338.peg.5535	fig|6666666.59339.peg.2361	fig|6666666.59340.peg.345	fig|6666666.59337.peg.1435	fig|6666666.59342.peg.2532	fig|6666666.71181.peg.603	fig|6666666.59344.peg.1121	fig|6666666.59345.peg.2767	fig|6666666.59341.peg.2995	fig|6666666.59347.peg.1270	fig|6666666.59346.peg.4483	fig|6666666.59348.peg.863	fig|6666666.59349.peg.2999	fig|6666666.59350.peg.605	fig|6666666.59351.peg.356	fig|6666666.59353.peg.798	fig|6666666.59355.peg.908	fig|6666666.59354.peg.37

6822	Transcriptional regulator, RpiR family	fig|6666666.59329.peg.6149	fig|6666666.59330.peg.1907	fig|6666666.59331.peg.2516	fig|6666666.59332.peg.790	fig|6666666.59334.peg.4286	fig|6666666.59335.peg.2936	fig|6666666.59336.peg.1219	fig|6666666.59333.peg.1610	fig|6666666.59338.peg.4317	fig|6666666.59339.peg.5341	fig|6666666.59340.peg.3408	fig|6666666.59337.peg.254	fig|6666666.59342.peg.190	fig|6666666.71181.peg.1931	fig|6666666.59344.peg.1574	fig|6666666.59345.peg.5246	fig|6666666.59341.peg.2538	fig|6666666.59347.peg.1980	fig|6666666.59346.peg.5319	fig|6666666.59348.peg.388	fig|6666666.59349.peg.4090	fig|6666666.59350.peg.3945	fig|6666666.59351.peg.5250	fig|6666666.59353.peg.1603	fig|6666666.59355.peg.2028	fig|6666666.59354.peg.3977

8219	Arylsulfatase EC 3.1.6.1	fig|6666666.59329.peg.4335	fig|6666666.59330.peg.2131	fig|6666666.59331.peg.5432	fig|6666666.59332.peg.3253	fig|6666666.59334.peg.4396	fig|6666666.59335.peg.1426	fig|6666666.59336.peg.5491	fig|6666666.59333.peg.5344	fig|6666666.59338.peg.3335	fig|6666666.59339.peg.215	fig|6666666.59340.peg.2976	fig|6666666.59337.peg.1855	fig|6666666.59342.peg.5292	fig|6666666.71181.peg.492	fig|6666666.59344.peg.1704	fig|6666666.59345.peg.1017	fig|6666666.59341.peg.2556	fig|6666666.59347.peg.3873	fig|6666666.59346.peg.4166	fig|6666666.59348.peg.2362	fig|6666666.59349.peg.4949	fig|6666666.59350.peg.1007	fig|6666666.59351.peg.5177	fig|6666666.59353.peg.1119	fig|6666666.59355.peg.5482	fig|6666666.59354.peg.608

1997	UDP-4-amino-4-deoxy-L-arabinose--oxoglutarate aminotransferase EC 2.6.1.-	fig|6666666.59329.peg.562	fig|6666666.59330.peg.4934	fig|6666666.59331.peg.2312	fig|6666666.59332.peg.3370	fig|6666666.59334.peg.1778	fig|6666666.59335.peg.490	fig|6666666.59336.peg.90	fig|6666666.59333.peg.1395	fig|6666666.59338.peg.155	fig|6666666.59339.peg.719	fig|6666666.59340.peg.2906	fig|6666666.59337.peg.458	fig|6666666.59342.peg.177	fig|6666666.71181.peg.2446	fig|6666666.59344.peg.793	fig|6666666.59345.peg.2607	fig|6666666.59341.peg.2064	fig|6666666.59347.peg.2352	fig|6666666.59346.peg.1699	fig|6666666.59348.peg.1044	fig|6666666.59349.peg.275	fig|6666666.59350.peg.3092	fig|6666666.59351.peg.2009	fig|6666666.59353.peg.1314	fig|6666666.59355.peg.5402	fig|6666666.59354.peg.864

1513	Uroporphyrinogen-III methyltransferase EC 2.1.1.107	fig|6666666.59329.peg.2913	fig|6666666.59330.peg.4805	fig|6666666.59331.peg.3627	fig|6666666.59332.peg.5006	fig|6666666.59334.peg.5120	fig|6666666.59335.peg.5310	fig|6666666.59336.peg.3918	fig|6666666.59333.peg.5483	fig|6666666.59338.peg.1786	fig|6666666.59339.peg.4676	fig|6666666.59340.peg.1359	fig|6666666.59337.peg.1187	fig|6666666.59342.peg.1631	fig|6666666.71181.peg.6301	fig|6666666.59344.peg.3073	fig|6666666.59345.peg.2502	fig|6666666.59341.peg.4212	fig|6666666.59347.peg.1902	fig|6666666.59346.peg.4992	fig|6666666.59348.peg.529	fig|6666666.59349.peg.1150	fig|6666666.59350.peg.1512	fig|6666666.59351.peg.5234	fig|6666666.59353.peg.5095	fig|6666666.59355.peg.2968	fig|6666666.59354.peg.2131

10446	Isocitrate lyase EC 4.1.3.1	fig|6666666.59329.peg.1841	fig|6666666.59330.peg.4918	fig|6666666.59331.peg.2351	fig|6666666.59332.peg.911	fig|6666666.59334.peg.3034	fig|6666666.59335.peg.3344	fig|6666666.59336.peg.3699	fig|6666666.59333.peg.362	fig|6666666.59338.peg.3145	fig|6666666.59339.peg.4016	fig|6666666.59340.peg.1356	fig|6666666.59337.peg.5690	fig|6666666.59342.peg.5972	fig|6666666.71181.peg.4893	fig|6666666.59344.peg.2692	fig|6666666.59345.peg.3665	fig|6666666.59341.peg.4868	fig|6666666.59347.peg.1616	fig|6666666.59346.peg.4398	fig|6666666.59348.peg.4810	fig|6666666.59349.peg.1499	fig|6666666.59350.peg.7512	fig|6666666.59351.peg.2934	fig|6666666.59353.peg.3034	fig|6666666.59355.peg.208	fig|6666666.59354.peg.1141

980	Bifunctional protein zinc-containing alcohol dehydrogenase; quinone oxidoreductase NADPH quinone reductase EC 1.1.1.- ; Similar to arginate lyase	fig|6666666.59329.peg.4312	fig|6666666.59330.peg.3491	fig|6666666.59331.peg.2969	fig|6666666.59332.peg.1567	fig|6666666.59334.peg.3425	fig|6666666.59335.peg.4370	fig|6666666.59336.peg.2578	fig|6666666.59333.peg.5584	fig|6666666.59338.peg.441	fig|6666666.59339.peg.8058	fig|6666666.59340.peg.1794	fig|6666666.59337.peg.1917	fig|6666666.59342.peg.3009	fig|6666666.71181.peg.2257	fig|6666666.59344.peg.2248	fig|6666666.59345.peg.586	fig|6666666.59341.peg.4043	fig|6666666.59347.peg.578	fig|6666666.59346.peg.2479	fig|6666666.59348.peg.3383	fig|6666666.59349.peg.3181	fig|6666666.59350.peg.8069	fig|6666666.59351.peg.188	fig|6666666.59353.peg.4966	fig|6666666.59355.peg.654	fig|6666666.59354.peg.2867

9085	Maltose maltodextrin ABC transporter, substrate binding periplasmic protein MalE	fig|6666666.59329.peg.6013	fig|6666666.59330.peg.3592	fig|6666666.59331.peg.2661	fig|6666666.59332.peg.2799	fig|6666666.59334.peg.3469	fig|6666666.59335.peg.3279	fig|6666666.59336.peg.1418	fig|6666666.59333.peg.997	fig|6666666.59338.peg.752	fig|6666666.59339.peg.3077	fig|6666666.59340.peg.1982	fig|6666666.59337.peg.2427	fig|6666666.59342.peg.1363	fig|6666666.71181.peg.5327	fig|6666666.59344.peg.2727	fig|6666666.59345.peg.623	fig|6666666.59341.peg.1969	fig|6666666.59347.peg.1474	fig|6666666.59346.peg.3319	fig|6666666.59348.peg.5005	fig|6666666.59349.peg.2259	fig|6666666.59350.peg.6539	fig|6666666.59351.peg.4777	fig|6666666.59353.peg.4147	fig|6666666.59355.peg.4268	fig|6666666.59354.peg.3659

2675	Cytochrome c-type biogenesis protein CcmG DsbE, thiol disulfide oxidoreductase	fig|6666666.59329.peg.2320	fig|6666666.59330.peg.4681	fig|6666666.59331.peg.3378	fig|6666666.59332.peg.6220	fig|6666666.59334.peg.2352	fig|6666666.59335.peg.1252	fig|6666666.59336.peg.5842	fig|6666666.59333.peg.2598	fig|6666666.59338.peg.2228	fig|6666666.59339.peg.3033	fig|6666666.59340.peg.1899	fig|6666666.59337.peg.4225	fig|6666666.59342.peg.5807	fig|6666666.71181.peg.1525	fig|6666666.59344.peg.4984	fig|6666666.59345.peg.5309	fig|6666666.59341.peg.3751	fig|6666666.59347.peg.5095	fig|6666666.59346.peg.2156	fig|6666666.59348.peg.3289	fig|6666666.59349.peg.177	fig|6666666.59350.peg.3264	fig|6666666.59351.peg.2089	fig|6666666.59353.peg.4871	fig|6666666.59355.peg.217	fig|6666666.59354.peg.1187

1167	FIG000988 Predicted permease	fig|6666666.59329.peg.6500	fig|6666666.59330.peg.2442	fig|6666666.59331.peg.2648	fig|6666666.59332.peg.2096	fig|6666666.59334.peg.5325	fig|6666666.59335.peg.255	fig|6666666.59336.peg.490	fig|6666666.59333.peg.2987	fig|6666666.59338.peg.5010	fig|6666666.59339.peg.8136	fig|6666666.59340.peg.1736	fig|6666666.59337.peg.4785	fig|6666666.59342.peg.4154	fig|6666666.71181.peg.2977	fig|6666666.59344.peg.3978	fig|6666666.59345.peg.4832	fig|6666666.59341.peg.1441	fig|6666666.59347.peg.3259	fig|6666666.59346.peg.1664	fig|6666666.59348.peg.1099	fig|6666666.59349.peg.4205	fig|6666666.59350.peg.4608	fig|6666666.59351.peg.3501	fig|6666666.59353.peg.2444	fig|6666666.59355.peg.535	fig|6666666.59354.peg.1326

2319	Beta-hexosaminidase EC 3.2.1.52	fig|6666666.59329.peg.853	fig|6666666.59330.peg.3106	fig|6666666.59331.peg.5097	fig|6666666.59332.peg.2366	fig|6666666.59334.peg.3384	fig|6666666.59335.peg.1534	fig|6666666.59336.peg.3694	fig|6666666.59333.peg.334	fig|6666666.59338.peg.3515	fig|6666666.59339.peg.1589	fig|6666666.59340.peg.2308	fig|6666666.59337.peg.646	fig|6666666.59342.peg.4055	fig|6666666.71181.peg.3284	fig|6666666.59344.peg.5092	fig|6666666.59345.peg.2388	fig|6666666.59341.peg.1806	fig|6666666.59347.peg.281	fig|6666666.59346.peg.4669	fig|6666666.59348.peg.4747	fig|6666666.59349.peg.224	fig|6666666.59350.peg.6551	fig|6666666.59351.peg.223	fig|6666666.59353.peg.2053	fig|6666666.59355.peg.516	fig|6666666.59354.peg.4993

2310	ClpB protein	fig|6666666.59329.peg.2980	fig|6666666.59330.peg.2898	fig|6666666.59331.peg.6056	fig|6666666.59332.peg.3172	fig|6666666.59334.peg.873	fig|6666666.59335.peg.4776	fig|6666666.59336.peg.5553	fig|6666666.59333.peg.3337	fig|6666666.59338.peg.4587	fig|6666666.59339.peg.7521	fig|6666666.59340.peg.3302	fig|6666666.59337.peg.5618	fig|6666666.59342.peg.114	fig|6666666.71181.peg.4128	fig|6666666.59344.peg.5879	fig|6666666.59345.peg.1554	fig|6666666.59341.peg.3631	fig|6666666.59347.peg.2412	fig|6666666.59346.peg.273	fig|6666666.59348.peg.4708	fig|6666666.59349.peg.1638	fig|6666666.59350.peg.4198	fig|6666666.59351.peg.521	fig|6666666.59353.peg.1716	fig|6666666.59355.peg.5011	fig|6666666.59354.peg.3756

5857	Na + -translocating NADH-quinone reductase subunit B EC 1.6.5.-	fig|6666666.59329.peg.4956	fig|6666666.59330.peg.2397	fig|6666666.59331.peg.433	fig|6666666.59332.peg.2203	fig|6666666.59334.peg.5554	fig|6666666.59335.peg.887	fig|6666666.59336.peg.5164	fig|6666666.59333.peg.1799	fig|6666666.59338.peg.5950	fig|6666666.59339.peg.57	fig|6666666.59340.peg.1840	fig|6666666.59337.peg.1497	fig|6666666.59342.peg.1973	fig|6666666.71181.peg.916	fig|6666666.59344.peg.5171	fig|6666666.59345.peg.3007	fig|6666666.59341.peg.4142	fig|6666666.59347.peg.5390	fig|6666666.59346.peg.2813	fig|6666666.59348.peg.1313	fig|6666666.59349.peg.4632	fig|6666666.59350.peg.4836	fig|6666666.59351.peg.3930	fig|6666666.59353.peg.1418	fig|6666666.59355.peg.2456	fig|6666666.59354.peg.6051

5852	Ferrichrome transport system permease protein FhuB TC 3.A.1.14.3	fig|6666666.59329.peg.6099	fig|6666666.59330.peg.3365	fig|6666666.59331.peg.1853	fig|6666666.59332.peg.666	fig|6666666.59334.peg.4638	fig|6666666.59335.peg.3168	fig|6666666.59336.peg.3628	fig|6666666.59333.peg.4070	fig|6666666.59338.peg.1716	fig|6666666.59339.peg.1812	fig|6666666.59340.peg.3777	fig|6666666.59337.peg.2452	fig|6666666.59342.peg.2082	fig|6666666.71181.peg.1559	fig|6666666.59344.peg.2279	fig|6666666.59345.peg.1990	fig|6666666.59341.peg.2375	fig|6666666.59347.peg.276	fig|6666666.59346.peg.4366	fig|6666666.59348.peg.2874	fig|6666666.59349.peg.4238	fig|6666666.59350.peg.885	fig|6666666.59351.peg.537	fig|6666666.59353.peg.1329	fig|6666666.59355.peg.2561	fig|6666666.59354.peg.2757

3774	Phosphoenolpyruvate synthase EC 2.7.9.2	fig|6666666.59329.peg.905	fig|6666666.59330.peg.4623	fig|6666666.59331.peg.4389	fig|6666666.59332.peg.5050	fig|6666666.59334.peg.1749	fig|6666666.59335.peg.1414	fig|6666666.59336.peg.2337	fig|6666666.59333.peg.1312	fig|6666666.59338.peg.189	fig|6666666.59339.peg.6655	fig|6666666.59340.peg.185	fig|6666666.59337.peg.4081	fig|6666666.59342.peg.3089	fig|6666666.71181.peg.72	fig|6666666.59344.peg.765	fig|6666666.59345.peg.3720	fig|6666666.59341.peg.443	fig|6666666.59347.peg.1873	fig|6666666.59346.peg.2634	fig|6666666.59348.peg.3330	fig|6666666.59349.peg.3875	fig|6666666.59350.peg.5085	fig|6666666.59351.peg.442	fig|6666666.59353.peg.1943	fig|6666666.59355.peg.2532	fig|6666666.59354.peg.3319

9744	Ferric iron ABC transporter, permease protein	fig|6666666.59329.peg.4630	fig|6666666.59330.peg.3244	fig|6666666.59331.peg.5604	fig|6666666.59332.peg.244	fig|6666666.59334.peg.2610	fig|6666666.59335.peg.774	fig|6666666.59336.peg.4765	fig|6666666.59333.peg.458	fig|6666666.59338.peg.1866	fig|6666666.59339.peg.1304	fig|6666666.59340.peg.1878	fig|6666666.59337.peg.640	fig|6666666.59342.peg.5015	fig|6666666.71181.peg.1265	fig|6666666.59344.peg.5859	fig|6666666.59345.peg.3967	fig|6666666.59341.peg.1233	fig|6666666.59347.peg.3366	fig|6666666.59346.peg.1128	fig|6666666.59348.peg.2937	fig|6666666.59349.peg.4578	fig|6666666.59350.peg.176	fig|6666666.59351.peg.3963	fig|6666666.59353.peg.4420	fig|6666666.59355.peg.2180	fig|6666666.59354.peg.375

11166	Riboflavin synthase eubacterial eukaryotic EC 2.5.1.9	fig|6666666.59329.peg.6331	fig|6666666.59330.peg.5042	fig|6666666.59331.peg.5723	fig|6666666.59332.peg.4879	fig|6666666.59334.peg.2065	fig|6666666.59335.peg.152	fig|6666666.59336.peg.2498	fig|6666666.59333.peg.4714	fig|6666666.59338.peg.3044	fig|6666666.59339.peg.5093	fig|6666666.59340.peg.1322	fig|6666666.59337.peg.2695	fig|6666666.59342.peg.4575	fig|6666666.71181.peg.2551	fig|6666666.59344.peg.3523	fig|6666666.59345.peg.3151	fig|6666666.59341.peg.381	fig|6666666.59347.peg.2769	fig|6666666.59346.peg.5304	fig|6666666.59348.peg.924	fig|6666666.59349.peg.3481	fig|6666666.59350.peg.1714	fig|6666666.59351.peg.4252	fig|6666666.59353.peg.1364	fig|6666666.59355.peg.2508	fig|6666666.59354.peg.4872

1579	Protease, insulinase family protease, insulinase family	fig|6666666.59329.peg.2128	fig|6666666.59330.peg.2835	fig|6666666.59331.peg.4200	fig|6666666.59332.peg.1570	fig|6666666.59334.peg.1701	fig|6666666.59335.peg.5953	fig|6666666.59336.peg.3149	fig|6666666.59333.peg.3510	fig|6666666.59338.peg.444	fig|6666666.59339.peg.8914	fig|6666666.59340.peg.911	fig|6666666.59337.peg.2276	fig|6666666.59342.peg.3098	fig|6666666.71181.peg.63	fig|6666666.59344.peg.5801	fig|6666666.59345.peg.2031	fig|6666666.59341.peg.2566	fig|6666666.59347.peg.2510	fig|6666666.59346.peg.2169	fig|6666666.59348.peg.3844	fig|6666666.59349.peg.2223	fig|6666666.59350.peg.913	fig|6666666.59351.peg.3745	fig|6666666.59353.peg.3658	fig|6666666.59355.peg.1454	fig|6666666.59354.peg.5656

5620	Anthranilate synthase, amidotransferase component EC 4.1.3.27	fig|6666666.59329.peg.13	fig|6666666.59330.peg.2970	fig|6666666.59331.peg.4976	fig|6666666.59332.peg.1848	fig|6666666.59334.peg.3049	fig|6666666.59335.peg.3720	fig|6666666.59336.peg.883	fig|6666666.59333.peg.307	fig|6666666.59338.peg.4716	fig|6666666.59339.peg.8224	fig|6666666.59340.peg.2675	fig|6666666.59337.peg.3497	fig|6666666.59342.peg.762	fig|6666666.71181.peg.4841	fig|6666666.59344.peg.3090	fig|6666666.59345.peg.5575	fig|6666666.59341.peg.2760	fig|6666666.59347.peg.741	fig|6666666.59346.peg.17	fig|6666666.59348.peg.400	fig|6666666.59349.peg.4272	fig|6666666.59350.peg.3548	fig|6666666.59351.peg.787	fig|6666666.59353.peg.5001	fig|6666666.59355.peg.1668	fig|6666666.59354.peg.6197

8492	4-hydroxythreonine-4-phosphate dehydrogenase EC 1.1.1.262	fig|6666666.59329.peg.131	fig|6666666.59330.peg.817	fig|6666666.59331.peg.6193	fig|6666666.59332.peg.5234	fig|6666666.59334.peg.3344	fig|6666666.59335.peg.1636	fig|6666666.59336.peg.2653	fig|6666666.59333.peg.131	fig|6666666.59338.peg.908	fig|6666666.59339.peg.7895	fig|6666666.59340.peg.435	fig|6666666.59337.peg.1385	fig|6666666.59342.peg.2175	fig|6666666.71181.peg.400	fig|6666666.59344.peg.1290	fig|6666666.59345.peg.2458	fig|6666666.59341.peg.2003	fig|6666666.59347.peg.3088	fig|6666666.59346.peg.4898	fig|6666666.59348.peg.178	fig|6666666.59349.peg.1877	fig|6666666.59350.peg.4571	fig|6666666.59351.peg.3651	fig|6666666.59353.peg.98	fig|6666666.59355.peg.708	fig|6666666.59354.peg.5331

10717	Xaa-Pro aminopeptidase EC 3.4.11.9	fig|6666666.59329.peg.5609	fig|6666666.59330.peg.983	fig|6666666.59331.peg.4519	fig|6666666.59332.peg.256	fig|6666666.59334.peg.2190	fig|6666666.59335.peg.11	fig|6666666.59336.peg.6153	fig|6666666.59333.peg.1570	fig|6666666.59338.peg.1672	fig|6666666.59339.peg.8753	fig|6666666.59340.peg.520	fig|6666666.59337.peg.2548	fig|6666666.59342.peg.5736	fig|6666666.71181.peg.5426	fig|6666666.59344.peg.4741	fig|6666666.59345.peg.5540	fig|6666666.59341.peg.3747	fig|6666666.59347.peg.5252	fig|6666666.59346.peg.929	fig|6666666.59348.peg.875	fig|6666666.59349.peg.3136	fig|6666666.59350.peg.6063	fig|6666666.59351.peg.743	fig|6666666.59353.peg.4438	fig|6666666.59355.peg.3409	fig|6666666.59354.peg.2857

10179	N-acetylglucosamine-6-phosphate deacetylase EC 3.5.1.25	fig|6666666.59329.peg.3098	fig|6666666.59330.peg.3002	fig|6666666.59331.peg.4870	fig|6666666.59332.peg.4258	fig|6666666.59334.peg.217	fig|6666666.59335.peg.4583	fig|6666666.59336.peg.325	fig|6666666.59333.peg.866	fig|6666666.59338.peg.1146	fig|6666666.59339.peg.7221	fig|6666666.59340.peg.654	fig|6666666.59337.peg.3928	fig|6666666.59342.peg.1040	fig|6666666.71181.peg.3200	fig|6666666.59344.peg.4524	fig|6666666.59345.peg.4340	fig|6666666.59341.peg.4867	fig|6666666.59347.peg.2948	fig|6666666.59346.peg.2000	fig|6666666.59348.peg.4526	fig|6666666.59349.peg.2663	fig|6666666.59350.peg.7747	fig|6666666.59351.peg.5157	fig|6666666.59353.peg.2046	fig|6666666.59355.peg.4453	fig|6666666.59354.peg.2878

769	tRNA Guanine37-N1 -methyltransferase EC 2.1.1.31	fig|6666666.59329.peg.338	fig|6666666.59330.peg.2846	fig|6666666.59331.peg.1987	fig|6666666.59332.peg.313	fig|6666666.59334.peg.268	fig|6666666.59335.peg.5393	fig|6666666.59336.peg.3003	fig|6666666.59333.peg.159	fig|6666666.59338.peg.2608	fig|6666666.59339.peg.7935	fig|6666666.59340.peg.3476	fig|6666666.59337.peg.2043	fig|6666666.59342.peg.2101	fig|6666666.71181.peg.3955	fig|6666666.59344.peg.5441	fig|6666666.59345.peg.2431	fig|6666666.59341.peg.1344	fig|6666666.59347.peg.3886	fig|6666666.59346.peg.571	fig|6666666.59348.peg.1261	fig|6666666.59349.peg.605	fig|6666666.59350.peg.2762	fig|6666666.59351.peg.542	fig|6666666.59353.peg.4812	fig|6666666.59355.peg.4866	fig|6666666.59354.peg.3769

5870	Dihydrolipoamide acetyltransferase component of pyruvate dehydrogenase complex EC 2.3.1.12	fig|6666666.59329.peg.5876	fig|6666666.59330.peg.3495	fig|6666666.59331.peg.1510	fig|6666666.59332.peg.2622	fig|6666666.59334.peg.482	fig|6666666.59335.peg.4304	fig|6666666.59336.peg.2112	fig|6666666.59333.peg.3304	fig|6666666.59338.peg.5616	fig|6666666.59339.peg.7969	fig|6666666.59340.peg.3561	fig|6666666.59337.peg.4802	fig|6666666.59342.peg.3543	fig|6666666.71181.peg.3237	fig|6666666.59344.peg.4591	fig|6666666.59345.peg.4320	fig|6666666.59341.peg.1578	fig|6666666.59347.peg.4387	fig|6666666.59346.peg.588	fig|6666666.59348.peg.3421	fig|6666666.59349.peg.2522	fig|6666666.59350.peg.3705	fig|6666666.59351.peg.2669	fig|6666666.59353.peg.2088	fig|6666666.59355.peg.5678	fig|6666666.59354.peg.774

5871	Protein of unknown function DUF81	fig|6666666.59329.peg.1935	fig|6666666.59330.peg.4674	fig|6666666.59331.peg.4388	fig|6666666.59332.peg.1352	fig|6666666.59334.peg.1748	fig|6666666.59335.peg.4842	fig|6666666.59336.peg.2363	fig|6666666.59333.peg.5501	fig|6666666.59338.peg.188	fig|6666666.59339.peg.7450	fig|6666666.59340.peg.2248	fig|6666666.59337.peg.124	fig|6666666.59342.peg.2682	fig|6666666.71181.peg.998	fig|6666666.59344.peg.1774	fig|6666666.59345.peg.3719	fig|6666666.59341.peg.5241	fig|6666666.59347.peg.3032	fig|6666666.59346.peg.5156	fig|6666666.59348.peg.4710	fig|6666666.59349.peg.4026	fig|6666666.59350.peg.2277	fig|6666666.59351.peg.1910	fig|6666666.59353.peg.2315	fig|6666666.59355.peg.1589	fig|6666666.59354.peg.1014

1074	Na + -translocating NADH-quinone reductase subunit F EC 1.6.5.-	fig|6666666.59329.peg.6140	fig|6666666.59330.peg.1856	fig|6666666.59331.peg.1034	fig|6666666.59332.peg.3584	fig|6666666.59334.peg.886	fig|6666666.59335.peg.4223	fig|6666666.59336.peg.4789	fig|6666666.59333.peg.3256	fig|6666666.59338.peg.2592	fig|6666666.59339.peg.6517	fig|6666666.59340.peg.658	fig|6666666.59337.peg.2439	fig|6666666.59342.peg.5624	fig|6666666.71181.peg.3330	fig|6666666.59344.peg.390	fig|6666666.59345.peg.5513	fig|6666666.59341.peg.2943	fig|6666666.59347.peg.1867	fig|6666666.59346.peg.5347	fig|6666666.59348.peg.518	fig|6666666.59349.peg.302	fig|6666666.59350.peg.7196	fig|6666666.59351.peg.3926	fig|6666666.59353.peg.3951	fig|6666666.59355.peg.4251	fig|6666666.59354.peg.4978

6197	Threonyl-tRNA synthetase EC 6.1.1.3	fig|6666666.59329.peg.4063	fig|6666666.59330.peg.514	fig|6666666.59331.peg.108	fig|6666666.59332.peg.5010	fig|6666666.59334.peg.317	fig|6666666.59335.peg.4083	fig|6666666.59336.peg.2086	fig|6666666.59333.peg.2258	fig|6666666.59338.peg.726	fig|6666666.59339.peg.2412	fig|6666666.59340.peg.3032	fig|6666666.59337.peg.3299	fig|6666666.59342.peg.2120	fig|6666666.71181.peg.5928	fig|6666666.59344.peg.2681	fig|6666666.59345.peg.2218	fig|6666666.59341.peg.3118	fig|6666666.59347.peg.4352	fig|6666666.59346.peg.4575	fig|6666666.59348.peg.3983	fig|6666666.59349.peg.4184	fig|6666666.59350.peg.6850	fig|6666666.59351.peg.4793	fig|6666666.59353.peg.1301	fig|6666666.59355.peg.3280	fig|6666666.59354.peg.2519

7203	Zinc uptake regulation protein ZUR	fig|6666666.59329.peg.1858	fig|6666666.59330.peg.2651	fig|6666666.59331.peg.5242	fig|6666666.59332.peg.2130	fig|6666666.59334.peg.2729	fig|6666666.59335.peg.2139	fig|6666666.59336.peg.3250	fig|6666666.59333.peg.881	fig|6666666.59338.peg.317	fig|6666666.59339.peg.6776	fig|6666666.59340.peg.2119	fig|6666666.59337.peg.1718	fig|6666666.59342.peg.3247	fig|6666666.71181.peg.1998	fig|6666666.59344.peg.2744	fig|6666666.59345.peg.3953	fig|6666666.59341.peg.2862	fig|6666666.59347.peg.4883	fig|6666666.59346.peg.3204	fig|6666666.59348.peg.536	fig|6666666.59349.peg.3245	fig|6666666.59350.peg.6087	fig|6666666.59351.peg.5440	fig|6666666.59353.peg.2995	fig|6666666.59355.peg.2901	fig|6666666.59354.peg.5100

1558	Cell division protein FtsJ Ribosomal RNA large subunit methyltransferase E EC 2.1.1.-	fig|6666666.59329.peg.3481	fig|6666666.59330.peg.114	fig|6666666.59331.peg.1134	fig|6666666.59332.peg.4095	fig|6666666.59334.peg.202	fig|6666666.59335.peg.2846	fig|6666666.59336.peg.226	fig|6666666.59333.peg.1489	fig|6666666.59338.peg.1497	fig|6666666.59339.peg.4858	fig|6666666.59340.peg.2858	fig|6666666.59337.peg.676	fig|6666666.59342.peg.2479	fig|6666666.71181.peg.4091	fig|6666666.59344.peg.2107	fig|6666666.59345.peg.2363	fig|6666666.59341.peg.3946	fig|6666666.59347.peg.3011	fig|6666666.59346.peg.291	fig|6666666.59348.peg.1540	fig|6666666.59349.peg.3698	fig|6666666.59350.peg.2981	fig|6666666.59351.peg.4938	fig|6666666.59353.peg.3140	fig|6666666.59355.peg.456	fig|6666666.59354.peg.1192

3037	Acetolactate synthase large subunit EC 2.2.1.6	fig|6666666.59329.peg.3449	fig|6666666.59330.peg.4321	fig|6666666.59331.peg.765	fig|6666666.59332.peg.4073	fig|6666666.59334.peg.1199	fig|6666666.59335.peg.3667	fig|6666666.59336.peg.4449	fig|6666666.59333.peg.4124	fig|6666666.59338.peg.547	fig|6666666.59339.peg.6557	fig|6666666.59340.peg.2265	fig|6666666.59337.peg.1404	fig|6666666.59342.peg.2927	fig|6666666.71181.peg.390	fig|6666666.59344.peg.2025	fig|6666666.59345.peg.4945	fig|6666666.59341.peg.2532	fig|6666666.59347.peg.4765	fig|6666666.59346.peg.2061	fig|6666666.59348.peg.278	fig|6666666.59349.peg.439	fig|6666666.59350.peg.956	fig|6666666.59351.peg.1606	fig|6666666.59353.peg.1145	fig|6666666.59355.peg.2838	fig|6666666.59354.peg.5782

8708	18K peptidoglycan-associated outer membrane lipoprotein; Peptidoglycan-associated lipoprotein precursor; Outer membrane protein P6; OmpA MotB precursor	fig|6666666.59329.peg.823	fig|6666666.59330.peg.3434	fig|6666666.59331.peg.4300	fig|6666666.59332.peg.4137	fig|6666666.59334.peg.4990	fig|6666666.59335.peg.1361	fig|6666666.59336.peg.4722	fig|6666666.59333.peg.1991	fig|6666666.59338.peg.1771	fig|6666666.59339.peg.401	fig|6666666.59340.peg.2092	fig|6666666.59337.peg.2580	fig|6666666.59342.peg.36	fig|6666666.71181.peg.447	fig|6666666.59344.peg.4146	fig|6666666.59345.peg.1504	fig|6666666.59341.peg.5903	fig|6666666.59347.peg.1271	fig|6666666.59346.peg.3457	fig|6666666.59348.peg.4961	fig|6666666.59349.peg.3601	fig|6666666.59350.peg.5546	fig|6666666.59351.peg.4851	fig|6666666.59353.peg.4413	fig|6666666.59355.peg.544	fig|6666666.59354.peg.1600

5609	LSU ribosomal protein L31p LSU ribosomal protein L31p, zinc-dependent	fig|6666666.59329.peg.1099	fig|6666666.59330.peg.548	fig|6666666.59331.peg.2078	fig|6666666.59332.peg.1181	fig|6666666.59334.peg.2422	fig|6666666.59335.peg.429	fig|6666666.59336.peg.138	fig|6666666.59333.peg.2686	fig|6666666.59338.peg.1049	fig|6666666.59339.peg.6380	fig|6666666.59340.peg.3758	fig|6666666.59337.peg.395	fig|6666666.59342.peg.5939	fig|6666666.71181.peg.3889	fig|6666666.59344.peg.1005	fig|6666666.59345.peg.2542	fig|6666666.59341.peg.5861	fig|6666666.59347.peg.4919	fig|6666666.59346.peg.1007	fig|6666666.59348.peg.162	fig|6666666.59349.peg.513	fig|6666666.59350.peg.2760	fig|6666666.59351.peg.3834	fig|6666666.59353.peg.2535	fig|6666666.59355.peg.5471	fig|6666666.59354.peg.931

4697	Multimodular transpeptidase-transglycosylase EC 2.4.1.129 EC 3.4.-.-	fig|6666666.59329.peg.1899	fig|6666666.59330.peg.2811	fig|6666666.59331.peg.2003	fig|6666666.59332.peg.2694	fig|6666666.59334.peg.856	fig|6666666.59335.peg.842	fig|6666666.59336.peg.6167	fig|6666666.59333.peg.1855	fig|6666666.59338.peg.11	fig|6666666.59339.peg.4782	fig|6666666.59340.peg.765	fig|6666666.59337.peg.1087	fig|6666666.59342.peg.2389	fig|6666666.71181.peg.4575	fig|6666666.59344.peg.4539	fig|6666666.59345.peg.2400	fig|6666666.59341.peg.5224	fig|6666666.59347.peg.2905	fig|6666666.59346.peg.3810	fig|6666666.59348.peg.3719	fig|6666666.59349.peg.2978	fig|6666666.59350.peg.6759	fig|6666666.59351.peg.3623	fig|6666666.59353.peg.131	fig|6666666.59355.peg.4082	fig|6666666.59354.peg.3665

9978	Type I secretion outer membrane protein, TolC precursor	fig|6666666.59329.peg.4237	fig|6666666.59330.peg.949	fig|6666666.59331.peg.5281	fig|6666666.59332.peg.1422	fig|6666666.59334.peg.1217	fig|6666666.59335.peg.5080	fig|6666666.59336.peg.2256	fig|6666666.59333.peg.2954	fig|6666666.59338.peg.3688	fig|6666666.59339.peg.4923	fig|6666666.59340.peg.900	fig|6666666.59337.peg.4948	fig|6666666.59342.peg.4966	fig|6666666.71181.peg.3033	fig|6666666.59344.peg.5624	fig|6666666.59345.peg.448	fig|6666666.59341.peg.4572	fig|6666666.59347.peg.1272	fig|6666666.59346.peg.4703	fig|6666666.59348.peg.772	fig|6666666.59349.peg.815	fig|6666666.59350.peg.6738	fig|6666666.59351.peg.2503	fig|6666666.59353.peg.589	fig|6666666.59355.peg.72	fig|6666666.59354.peg.659

228	Protease IV EC 3.4.21.-	fig|6666666.59329.peg.3070	fig|6666666.59330.peg.1390	fig|6666666.59331.peg.6238	fig|6666666.59332.peg.3150	fig|6666666.59334.peg.1081	fig|6666666.59335.peg.849	fig|6666666.59336.peg.3562	fig|6666666.59333.peg.732	fig|6666666.59338.peg.4337	fig|6666666.59339.peg.3207	fig|6666666.59340.peg.534	fig|6666666.59337.peg.3131	fig|6666666.59342.peg.1231	fig|6666666.71181.peg.4157	fig|6666666.59344.peg.2898	fig|6666666.59345.peg.1159	fig|6666666.59341.peg.45	fig|6666666.59347.peg.4165	fig|6666666.59346.peg.4458	fig|6666666.59348.peg.5420	fig|6666666.59349.peg.394	fig|6666666.59350.peg.498	fig|6666666.59351.peg.924	fig|6666666.59353.peg.1429	fig|6666666.59355.peg.1978	fig|6666666.59354.peg.6135

227	Helicase PriA essential for oriC DnaA-independent DNA replication	fig|6666666.59329.peg.1098	fig|6666666.59330.peg.547	fig|6666666.59331.peg.3288	fig|6666666.59332.peg.1183	fig|6666666.59334.peg.2420	fig|6666666.59335.peg.430	fig|6666666.59336.peg.137	fig|6666666.59333.peg.2683	fig|6666666.59338.peg.3635	fig|6666666.59339.peg.6384	fig|6666666.59340.peg.371	fig|6666666.59337.peg.397	fig|6666666.59342.peg.4526	fig|6666666.71181.peg.3891	fig|6666666.59344.peg.1003	fig|6666666.59345.peg.2543	fig|6666666.59341.peg.5844	fig|6666666.59347.peg.4917	fig|6666666.59346.peg.1008	fig|6666666.59348.peg.159	fig|6666666.59349.peg.512	fig|6666666.59350.peg.3294	fig|6666666.59351.peg.3833	fig|6666666.59353.peg.2537	fig|6666666.59355.peg.5605	fig|6666666.59354.peg.932

10057	ATP-dependent DNA helicase RecQ	fig|6666666.59329.peg.3220	fig|6666666.59330.peg.4055	fig|6666666.59331.peg.5984	fig|6666666.59332.peg.1665	fig|6666666.59334.peg.2862	fig|6666666.59335.peg.130	fig|6666666.59336.peg.2775	fig|6666666.59333.peg.1809	fig|6666666.59338.peg.3008	fig|6666666.59339.peg.7669	fig|6666666.59340.peg.468	fig|6666666.59337.peg.1553	fig|6666666.59342.peg.5634	fig|6666666.71181.peg.3799	fig|6666666.59344.peg.2501	fig|6666666.59345.peg.2227	fig|6666666.59341.peg.5929	fig|6666666.59347.peg.345	fig|6666666.59346.peg.5078	fig|6666666.59348.peg.2141	fig|6666666.59349.peg.2413	fig|6666666.59350.peg.622	fig|6666666.59351.peg.1898	fig|6666666.59353.peg.4647	fig|6666666.59355.peg.4764	fig|6666666.59354.peg.3993

10053	1-deoxy-D-xylulose 5-phosphate synthase EC 2.2.1.7	fig|6666666.59329.peg.5875	fig|6666666.59330.peg.1053	fig|6666666.59331.peg.4704	fig|6666666.59332.peg.5787	fig|6666666.59334.peg.1927	fig|6666666.59335.peg.3898	fig|6666666.59336.peg.5426	fig|6666666.59333.peg.4912	fig|6666666.59338.peg.3890	fig|6666666.59339.peg.7174	fig|6666666.59340.peg.1176	fig|6666666.59337.peg.4804	fig|6666666.59342.peg.1606	fig|6666666.71181.peg.3786	fig|6666666.59344.peg.6268	fig|6666666.59345.peg.317	fig|6666666.59341.peg.4518	fig|6666666.59347.peg.1307	fig|6666666.59346.peg.304	fig|6666666.59348.peg.2890	fig|6666666.59349.peg.2781	fig|6666666.59350.peg.3344	fig|6666666.59351.peg.5452	fig|6666666.59353.peg.4633	fig|6666666.59355.peg.4494	fig|6666666.59354.peg.3320

5810	2-amino-4-hydroxy-6-hydroxymethyldihydropteridine pyrophosphokinase EC 2.7.6.3	fig|6666666.59329.peg.4158	fig|6666666.59330.peg.1753	fig|6666666.59331.peg.2657	fig|6666666.59332.peg.1508	fig|6666666.59334.peg.820	fig|6666666.59335.peg.2327	fig|6666666.59336.peg.5409	fig|6666666.59333.peg.180	fig|6666666.59338.peg.798	fig|6666666.59339.peg.7913	fig|6666666.59340.peg.2169	fig|6666666.59337.peg.622	fig|6666666.59342.peg.1573	fig|6666666.71181.peg.2841	fig|6666666.59344.peg.4439	fig|6666666.59345.peg.2406	fig|6666666.59341.peg.1554	fig|6666666.59347.peg.3132	fig|6666666.59346.peg.4350	fig|6666666.59348.peg.1697	fig|6666666.59349.peg.2474	fig|6666666.59350.peg.1363	fig|6666666.59351.peg.5224	fig|6666666.59353.peg.3192	fig|6666666.59355.peg.3074	fig|6666666.59354.peg.45

145	DNA-directed RNA polymerase alpha subunit EC 2.7.7.6	fig|6666666.59329.peg.733	fig|6666666.59330.peg.576	fig|6666666.59331.peg.27	fig|6666666.59332.peg.3505	fig|6666666.59334.peg.3040	fig|6666666.59335.peg.4754	fig|6666666.59336.peg.166	fig|6666666.59333.peg.388	fig|6666666.59338.peg.4740	fig|6666666.59339.peg.8290	fig|6666666.59340.peg.583	fig|6666666.59337.peg.367	fig|6666666.59342.peg.326	fig|6666666.71181.peg.3439	fig|6666666.59344.peg.4922	fig|6666666.59345.peg.2514	fig|6666666.59341.peg.776	fig|6666666.59347.peg.4845	fig|6666666.59346.peg.871	fig|6666666.59348.peg.433	fig|6666666.59349.peg.3291	fig|6666666.59350.peg.1554	fig|6666666.59351.peg.4226	fig|6666666.59353.peg.4611	fig|6666666.59355.peg.5973	fig|6666666.59354.peg.3246

2713	4-diphosphocytidyl-2-C-methyl-D-erythritol kinase EC 2.7.1.148	fig|6666666.59329.peg.5528	fig|6666666.59330.peg.4668	fig|6666666.59331.peg.1440	fig|6666666.59332.peg.4262	fig|6666666.59334.peg.2240	fig|6666666.59335.peg.1175	fig|6666666.59336.peg.5361	fig|6666666.59333.peg.5422	fig|6666666.59338.peg.5373	fig|6666666.59339.peg.6	fig|6666666.59340.peg.3456	fig|6666666.59337.peg.5164	fig|6666666.59342.peg.468	fig|6666666.71181.peg.782	fig|6666666.59344.peg.105	fig|6666666.59345.peg.5413	fig|6666666.59341.peg.6007	fig|6666666.59347.peg.5282	fig|6666666.59346.peg.3188	fig|6666666.59348.peg.612	fig|6666666.59349.peg.2612	fig|6666666.59350.peg.3702	fig|6666666.59351.peg.4665	fig|6666666.59353.peg.429	fig|6666666.59355.peg.2155	fig|6666666.59354.peg.5210

824	Competence protein F homolog, phosphoribosyltransferase domain; protein YhgH required for utilization of DNA as sole source of carbon and energy	fig|6666666.59329.peg.1637	fig|6666666.59330.peg.431	fig|6666666.59331.peg.5164	fig|6666666.59332.peg.2469	fig|6666666.59334.peg.2876	fig|6666666.59335.peg.697	fig|6666666.59336.peg.4434	fig|6666666.59333.peg.520	fig|6666666.59338.peg.1424	fig|6666666.59339.peg.6233	fig|6666666.59340.peg.2257	fig|6666666.59337.peg.4259	fig|6666666.59342.peg.3593	fig|6666666.71181.peg.4485	fig|6666666.59344.peg.3637	fig|6666666.59345.peg.2663	fig|6666666.59341.peg.1414	fig|6666666.59347.peg.4477	fig|6666666.59346.peg.2235	fig|6666666.59348.peg.1407	fig|6666666.59349.peg.2194	fig|6666666.59350.peg.237	fig|6666666.59351.peg.4365	fig|6666666.59353.peg.2187	fig|6666666.59355.peg.1881	fig|6666666.59354.peg.1789

822	2-dehydro-3-deoxygluconate kinase EC 2.7.1.45	fig|6666666.59329.peg.4706	fig|6666666.59330.peg.948	fig|6666666.59331.peg.1265	fig|6666666.59332.peg.6217	fig|6666666.59334.peg.1952	fig|6666666.59335.peg.4478	fig|6666666.59336.peg.5898	fig|6666666.59333.peg.481	fig|6666666.59338.peg.683	fig|6666666.59339.peg.701	fig|6666666.59340.peg.2427	fig|6666666.59337.peg.14	fig|6666666.59342.peg.5899	fig|6666666.71181.peg.5439	fig|6666666.59344.peg.4248	fig|6666666.59345.peg.161	fig|6666666.59341.peg.5161	fig|6666666.59347.peg.1073	fig|6666666.59346.peg.3991	fig|6666666.59348.peg.4660	fig|6666666.59349.peg.4094	fig|6666666.59350.peg.7042	fig|6666666.59351.peg.1679	fig|6666666.59353.peg.3102	fig|6666666.59355.peg.4338	fig|6666666.59354.peg.5119

8166	ATP-dependent Clp protease proteolytic subunit EC 3.4.21.92	fig|6666666.59329.peg.4569	fig|6666666.59330.peg.294	fig|6666666.59331.peg.687	fig|6666666.59332.peg.5205	fig|6666666.59334.peg.510	fig|6666666.59335.peg.2709	fig|6666666.59336.peg.4123	fig|6666666.59333.peg.2635	fig|6666666.59338.peg.2552	fig|6666666.59339.peg.3096	fig|6666666.59340.peg.1683	fig|6666666.59337.peg.1024	fig|6666666.59342.peg.2791	fig|6666666.71181.peg.2052	fig|6666666.59344.peg.1068	fig|6666666.59345.peg.4258	fig|6666666.59341.peg.4432	fig|6666666.59347.peg.2658	fig|6666666.59346.peg.2781	fig|6666666.59348.peg.3841	fig|6666666.59349.peg.4766	fig|6666666.59350.peg.5489	fig|6666666.59351.peg.5101	fig|6666666.59353.peg.753	fig|6666666.59355.peg.951	fig|6666666.59354.peg.128

5257	Agmatinase EC 3.5.3.11	fig|6666666.59329.peg.1370	fig|6666666.59330.peg.1656	fig|6666666.59331.peg.5559	fig|6666666.59332.peg.4734	fig|6666666.59334.peg.2634	fig|6666666.59335.peg.1565	fig|6666666.59336.peg.1378	fig|6666666.59333.peg.3576	fig|6666666.59338.peg.5673	fig|6666666.59339.peg.3876	fig|6666666.59340.peg.42	fig|6666666.59337.peg.3304	fig|6666666.59342.peg.1110	fig|6666666.71181.peg.254	fig|6666666.59344.peg.317	fig|6666666.59345.peg.4628	fig|6666666.59341.peg.4629	fig|6666666.59347.peg.1573	fig|6666666.59346.peg.3555	fig|6666666.59348.peg.3350	fig|6666666.59349.peg.4076	fig|6666666.59350.peg.790	fig|6666666.59351.peg.3583	fig|6666666.59353.peg.564	fig|6666666.59355.peg.49	fig|6666666.59354.peg.6348

3012	3-oxoacyl-[acyl-carrier protein] reductase EC 1.1.1.100	fig|6666666.59329.peg.2683	fig|6666666.59330.peg.3272	fig|6666666.59331.peg.5110	fig|6666666.59332.peg.3474	fig|6666666.59334.peg.5298	fig|6666666.59335.peg.4174	fig|6666666.59336.peg.2130	fig|6666666.59333.peg.1588	fig|6666666.59338.peg.2788	fig|6666666.59339.peg.5434	fig|6666666.59340.peg.328	fig|6666666.59337.peg.212	fig|6666666.59342.peg.5316	fig|6666666.71181.peg.6107	fig|6666666.59344.peg.5914	fig|6666666.59345.peg.4283	fig|6666666.59341.peg.4319	fig|6666666.59347.peg.4085	fig|6666666.59346.peg.497	fig|6666666.59348.peg.1002	fig|6666666.59349.peg.3724	fig|6666666.59350.peg.4890	fig|6666666.59351.peg.5415	fig|6666666.59353.peg.3921	fig|6666666.59355.peg.4597	fig|6666666.59354.peg.1630

5664	Ribosome hibernation protein YhbH	fig|6666666.59329.peg.816	fig|6666666.59330.peg.44	fig|6666666.59331.peg.6057	fig|6666666.59332.peg.1320	fig|6666666.59334.peg.104	fig|6666666.59335.peg.6214	fig|6666666.59336.peg.839	fig|6666666.59333.peg.260	fig|6666666.59338.peg.3351	fig|6666666.59339.peg.8206	fig|6666666.59340.peg.3540	fig|6666666.59337.peg.828	fig|6666666.59342.peg.4735	fig|6666666.71181.peg.3946	fig|6666666.59344.peg.5431	fig|6666666.59345.peg.4862	fig|6666666.59341.peg.3003	fig|6666666.59347.peg.3254	fig|6666666.59346.peg.642	fig|6666666.59348.peg.4137	fig|6666666.59349.peg.2805	fig|6666666.59350.peg.5364	fig|6666666.59351.peg.264	fig|6666666.59353.peg.4803	fig|6666666.59355.peg.1606	fig|6666666.59354.peg.4907

9386	Adenylosuccinate synthetase EC 6.3.4.4	fig|6666666.59329.peg.5156	fig|6666666.59330.peg.1434	fig|6666666.59331.peg.4104	fig|6666666.59332.peg.907	fig|6666666.59334.peg.3337	fig|6666666.59335.peg.1860	fig|6666666.59336.peg.3057	fig|6666666.59333.peg.1309	fig|6666666.59338.peg.1008	fig|6666666.59339.peg.8255	fig|6666666.59340.peg.1200	fig|6666666.59337.peg.1788	fig|6666666.59342.peg.5482	fig|6666666.71181.peg.4629	fig|6666666.59344.peg.5890	fig|6666666.59345.peg.5584	fig|6666666.59341.peg.4277	fig|6666666.59347.peg.5342	fig|6666666.59346.peg.2380	fig|6666666.59348.peg.403	fig|6666666.59349.peg.3774	fig|6666666.59350.peg.4409	fig|6666666.59351.peg.3098	fig|6666666.59353.peg.4985	fig|6666666.59355.peg.5068	fig|6666666.59354.peg.35

8182	3,4-dihydroxy-2-butanone 4-phosphate synthase EC 4.1.99.12 GTP cyclohydrolase II EC 3.5.4.25	fig|6666666.59329.peg.6460	fig|6666666.59330.peg.863	fig|6666666.59331.peg.4460	fig|6666666.59332.peg.5755	fig|6666666.59334.peg.1400	fig|6666666.59335.peg.4602	fig|6666666.59336.peg.6048	fig|6666666.59333.peg.4840	fig|6666666.59338.peg.5268	fig|6666666.59339.peg.5095	fig|6666666.59340.peg.3520	fig|6666666.59337.peg.1139	fig|6666666.59342.peg.3546	fig|6666666.71181.peg.3022	fig|6666666.59344.peg.5409	fig|6666666.59345.peg.3540	fig|6666666.59341.peg.720	fig|6666666.59347.peg.4316	fig|6666666.59346.peg.4444	fig|6666666.59348.peg.2579	fig|6666666.59349.peg.3483	fig|6666666.59350.peg.440	fig|6666666.59351.peg.5260	fig|6666666.59353.peg.3601	fig|6666666.59355.peg.1703	fig|6666666.59354.peg.2929

10338	TRAP-type C4-dicarboxylate transport system, periplasmic component	fig|6666666.59329.peg.2388	fig|6666666.59330.peg.286	fig|6666666.59331.peg.4441	fig|6666666.59332.peg.2878	fig|6666666.59334.peg.3022	fig|6666666.59335.peg.5788	fig|6666666.59336.peg.505	fig|6666666.59333.peg.5399	fig|6666666.59338.peg.1947	fig|6666666.59339.peg.3086	fig|6666666.59340.peg.2846	fig|6666666.59337.peg.312	fig|6666666.59342.peg.4020	fig|6666666.71181.peg.3632	fig|6666666.59344.peg.5269	fig|6666666.59345.peg.771	fig|6666666.59341.peg.690	fig|6666666.59347.peg.3815	fig|6666666.59346.peg.3001	fig|6666666.59348.peg.4678	fig|6666666.59349.peg.4399	fig|6666666.59350.peg.4171	fig|6666666.59351.peg.3522	fig|6666666.59353.peg.4704	fig|6666666.59355.peg.4786	fig|6666666.59354.peg.2673

4408	Mg Co Ni transporter MgtE CBS domain	fig|6666666.59329.peg.5489	fig|6666666.59330.peg.40	fig|6666666.59331.peg.82	fig|6666666.59332.peg.2746	fig|6666666.59334.peg.1821	fig|6666666.59335.peg.3058	fig|6666666.59336.peg.1336	fig|6666666.59333.peg.256	fig|6666666.59338.peg.6025	fig|6666666.59339.peg.4849	fig|6666666.59340.peg.3546	fig|6666666.59337.peg.3271	fig|6666666.59342.peg.2392	fig|6666666.71181.peg.4291	fig|6666666.59344.peg.4046	fig|6666666.59345.peg.4866	fig|6666666.59341.peg.1237	fig|6666666.59347.peg.3015	fig|6666666.59346.peg.646	fig|6666666.59348.peg.961	fig|6666666.59349.peg.15	fig|6666666.59350.peg.1391	fig|6666666.59351.peg.3433	fig|6666666.59353.peg.4009	fig|6666666.59355.peg.3968	fig|6666666.59354.peg.6368

5398	LSU ribosomal protein L1p L10Ae	fig|6666666.59329.peg.624	fig|6666666.59330.peg.3406	fig|6666666.59331.peg.2752	fig|6666666.59332.peg.2887	fig|6666666.59334.peg.2475	fig|6666666.59335.peg.5627	fig|6666666.59336.peg.1681	fig|6666666.59333.peg.4395	fig|6666666.59338.peg.3290	fig|6666666.59339.peg.8846	fig|6666666.59340.peg.1351	fig|6666666.59337.peg.4987	fig|6666666.59342.peg.369	fig|6666666.71181.peg.5017	fig|6666666.59344.peg.5464	fig|6666666.59345.peg.5656	fig|6666666.59341.peg.5592	fig|6666666.59347.peg.5400	fig|6666666.59346.peg.4097	fig|6666666.59348.peg.3989	fig|6666666.59349.peg.1144	fig|6666666.59350.peg.3787	fig|6666666.59351.peg.5240	fig|6666666.59353.peg.4450	fig|6666666.59355.peg.4577	fig|6666666.59354.peg.4536

401	Putative phosphatase YqaB	fig|6666666.59329.peg.86	fig|6666666.59330.peg.3417	fig|6666666.59331.peg.550	fig|6666666.59332.peg.2413	fig|6666666.59334.peg.4851	fig|6666666.59335.peg.1543	fig|6666666.59336.peg.4013	fig|6666666.59333.peg.4620	fig|6666666.59338.peg.5542	fig|6666666.59339.peg.3835	fig|6666666.59340.peg.68	fig|6666666.59337.peg.1231	fig|6666666.59342.peg.610	fig|6666666.71181.peg.611	fig|6666666.59344.peg.2163	fig|6666666.59345.peg.5498	fig|6666666.59341.peg.6113	fig|6666666.59347.peg.5072	fig|6666666.59346.peg.2351	fig|6666666.59348.peg.1028	fig|6666666.59349.peg.2127	fig|6666666.59350.peg.1944	fig|6666666.59351.peg.666	fig|6666666.59353.peg.1283	fig|6666666.59355.peg.1917	fig|6666666.59354.peg.4554

7486	NAD P transhydrogenase alpha subunit EC 1.6.1.2	fig|6666666.59329.peg.4233	fig|6666666.59330.peg.796	fig|6666666.59331.peg.2717	fig|6666666.59332.peg.4915	fig|6666666.59334.peg.4640	fig|6666666.59335.peg.556	fig|6666666.59336.peg.2303	fig|6666666.59333.peg.1531	fig|6666666.59338.peg.2189	fig|6666666.59339.peg.3424	fig|6666666.59340.peg.3427	fig|6666666.59337.peg.4861	fig|6666666.59342.peg.1097	fig|6666666.71181.peg.1348	fig|6666666.59344.peg.589	fig|6666666.59345.peg.1341	fig|6666666.59341.peg.2224	fig|6666666.59347.peg.2398	fig|6666666.59346.peg.1899	fig|6666666.59348.peg.5339	fig|6666666.59349.peg.1581	fig|6666666.59350.peg.2937	fig|6666666.59351.peg.998	fig|6666666.59353.peg.1886	fig|6666666.59355.peg.1128	fig|6666666.59354.peg.5454

5242	Dimethyladenosine transferase EC 2.1.1.-	fig|6666666.59329.peg.132	fig|6666666.59330.peg.818	fig|6666666.59331.peg.6166	fig|6666666.59332.peg.5233	fig|6666666.59334.peg.3346	fig|6666666.59335.peg.4481	fig|6666666.59336.peg.2654	fig|6666666.59333.peg.5404	fig|6666666.59338.peg.909	fig|6666666.59339.peg.7892	fig|6666666.59340.peg.433	fig|6666666.59337.peg.1384	fig|6666666.59342.peg.2176	fig|6666666.71181.peg.401	fig|6666666.59344.peg.1291	fig|6666666.59345.peg.2459	fig|6666666.59341.peg.2002	fig|6666666.59347.peg.3089	fig|6666666.59346.peg.4899	fig|6666666.59348.peg.177	fig|6666666.59349.peg.1876	fig|6666666.59350.peg.663	fig|6666666.59351.peg.3650	fig|6666666.59353.peg.97	fig|6666666.59355.peg.707	fig|6666666.59354.peg.5332

4790	Protein involved in catabolism of external DNA	fig|6666666.59329.peg.2296	fig|6666666.59330.peg.226	fig|6666666.59331.peg.4400	fig|6666666.59332.peg.2422	fig|6666666.59334.peg.4048	fig|6666666.59335.peg.101	fig|6666666.59336.peg.765	fig|6666666.59333.peg.683	fig|6666666.59338.peg.4557	fig|6666666.59339.peg.7641	fig|6666666.59340.peg.2280	fig|6666666.59337.peg.944	fig|6666666.59342.peg.4375	fig|6666666.71181.peg.1054	fig|6666666.59344.peg.4955	fig|6666666.59345.peg.2731	fig|6666666.59341.peg.5563	fig|6666666.59347.peg.5204	fig|6666666.59346.peg.2809	fig|6666666.59348.peg.42	fig|6666666.59349.peg.1787	fig|6666666.59350.peg.4663	fig|6666666.59351.peg.2314	fig|6666666.59353.peg.4522	fig|6666666.59355.peg.4632	fig|6666666.59354.peg.3180

10324	Chromosome plasmid partitioning protein ParA	fig|6666666.59329.peg.6569	fig|6666666.59330.peg.1894	fig|6666666.59331.peg.417	fig|6666666.59332.peg.96	fig|6666666.59334.peg.3446	fig|6666666.59335.peg.3112	fig|6666666.59336.peg.5169	fig|6666666.59333.peg.32	fig|6666666.59338.peg.4856	fig|6666666.59339.peg.8018	fig|6666666.59340.peg.746	fig|6666666.59337.peg.2982	fig|6666666.59342.peg.1193	fig|6666666.71181.peg.5195	fig|6666666.59344.peg.251	fig|6666666.59345.peg.5257	fig|6666666.59341.peg.5587	fig|6666666.59347.peg.14	fig|6666666.59346.peg.1179	fig|6666666.59348.peg.1773	fig|6666666.59349.peg.3744	fig|6666666.59350.peg.3286	fig|6666666.59351.peg.2806	fig|6666666.59353.peg.616	fig|6666666.59355.peg.3529	fig|6666666.59354.peg.1146

2771	Heme O synthase, protoheme IX farnesyltransferase EC 2.5.1.- COX10-CtaB	fig|6666666.59329.peg.777	fig|6666666.59330.peg.2621	fig|6666666.59331.peg.6086	fig|6666666.59332.peg.2378	fig|6666666.59334.peg.5041	fig|6666666.59335.peg.4309	fig|6666666.59336.peg.1370	fig|6666666.59333.peg.1957	fig|6666666.59338.peg.1537	fig|6666666.59339.peg.6629	fig|6666666.59340.peg.3200	fig|6666666.59337.peg.5078	fig|6666666.59342.peg.1308	fig|6666666.71181.peg.2041	fig|6666666.59344.peg.1080	fig|6666666.59345.peg.367	fig|6666666.59341.peg.4105	fig|6666666.59347.peg.1545	fig|6666666.59346.peg.1645	fig|6666666.59348.peg.2967	fig|6666666.59349.peg.3502	fig|6666666.59350.peg.2024	fig|6666666.59351.peg.4382	fig|6666666.59353.peg.3002	fig|6666666.59355.peg.940	fig|6666666.59354.peg.2417

2774	Long-chain fatty acid transport protein	fig|6666666.59329.peg.4701	fig|6666666.59330.peg.5131	fig|6666666.59331.peg.2834	fig|6666666.59332.peg.3536	fig|6666666.59334.peg.3401	fig|6666666.59335.peg.1258	fig|6666666.59336.peg.4883	fig|6666666.59333.peg.4271	fig|6666666.59338.peg.2235	fig|6666666.59339.peg.3042	fig|6666666.59340.peg.1113	fig|6666666.59337.peg.4965	fig|6666666.59342.peg.6463	fig|6666666.71181.peg.1518	fig|6666666.59344.peg.4992	fig|6666666.59345.peg.5303	fig|6666666.59341.peg.5909	fig|6666666.59347.peg.829	fig|6666666.59346.peg.2161	fig|6666666.59348.peg.1632	fig|6666666.59349.peg.3779	fig|6666666.59350.peg.2787	fig|6666666.59351.peg.3387	fig|6666666.59353.peg.4877	fig|6666666.59355.peg.223	fig|6666666.59354.peg.28

844	Type II IV secretion system secretin RcpA CpaC, associated with Flp pilus assembly	fig|6666666.59329.peg.5897	fig|6666666.59330.peg.417	fig|6666666.59331.peg.994	fig|6666666.59332.peg.685	fig|6666666.59334.peg.2686	fig|6666666.59335.peg.3500	fig|6666666.59336.peg.700	fig|6666666.59333.peg.577	fig|6666666.59338.peg.4885	fig|6666666.59339.peg.7763	fig|6666666.59340.peg.1839	fig|6666666.59337.peg.4462	fig|6666666.59342.peg.682	fig|6666666.71181.peg.1833	fig|6666666.59344.peg.3061	fig|6666666.59345.peg.2582	fig|6666666.59341.peg.3428	fig|6666666.59347.peg.1914	fig|6666666.59346.peg.4789	fig|6666666.59348.peg.5063	fig|6666666.59349.peg.3404	fig|6666666.59350.peg.2523	fig|6666666.59351.peg.3849	fig|6666666.59353.peg.2217	fig|6666666.59355.peg.1909	fig|6666666.59354.peg.2404

7158	D-amino acid dehydrogenase EC 1.4.99.1 family protein in hydroxy-L-proline catabolic cluster	fig|6666666.59329.peg.2146	fig|6666666.59330.peg.2557	fig|6666666.59331.peg.5682	fig|6666666.59332.peg.5864	fig|6666666.59334.peg.2407	fig|6666666.59335.peg.5355	fig|6666666.59336.peg.5247	fig|6666666.59333.peg.5135	fig|6666666.59338.peg.4636	fig|6666666.59339.peg.2045	fig|6666666.59340.peg.3258	fig|6666666.59337.peg.768	fig|6666666.59342.peg.3022	fig|6666666.71181.peg.5355	fig|6666666.59344.peg.5701	fig|6666666.59345.peg.5597	fig|6666666.59341.peg.3599	fig|6666666.59347.peg.507	fig|6666666.59346.peg.1461	fig|6666666.59348.peg.4910	fig|6666666.59349.peg.2346	fig|6666666.59350.peg.3380	fig|6666666.59351.peg.1215	fig|6666666.59353.peg.4629	fig|6666666.59355.peg.5576	fig|6666666.59354.peg.6431

1735	Glutathione S-transferase EC 2.5.1.18	fig|6666666.59329.peg.5642	fig|6666666.59330.peg.967	fig|6666666.59331.peg.1846	fig|6666666.59332.peg.3731	fig|6666666.59334.peg.3272	fig|6666666.59335.peg.1552	fig|6666666.59336.peg.6579	fig|6666666.59333.peg.4283	fig|6666666.59338.peg.4558	fig|6666666.59339.peg.3852	fig|6666666.59340.peg.2155	fig|6666666.59337.peg.943	fig|6666666.59342.peg.5254	fig|6666666.71181.peg.2925	fig|6666666.59344.peg.3314	fig|6666666.59345.peg.2732	fig|6666666.59341.peg.4900	fig|6666666.59347.peg.5314	fig|6666666.59346.peg.2810	fig|6666666.59348.peg.5428	fig|6666666.59349.peg.982	fig|6666666.59350.peg.1140	fig|6666666.59351.peg.3465	fig|6666666.59353.peg.4193	fig|6666666.59355.peg.142	fig|6666666.59354.peg.3845

1597	Transcription antitermination protein NusG	fig|6666666.59329.peg.1945	fig|6666666.59330.peg.3404	fig|6666666.59331.peg.1527	fig|6666666.59332.peg.6078	fig|6666666.59334.peg.211	fig|6666666.59335.peg.5625	fig|6666666.59336.peg.320	fig|6666666.59333.peg.4397	fig|6666666.59338.peg.2348	fig|6666666.59339.peg.8848	fig|6666666.59340.peg.1353	fig|6666666.59337.peg.4985	fig|6666666.59342.peg.371	fig|6666666.71181.peg.5019	fig|6666666.59344.peg.3406	fig|6666666.59345.peg.4345	fig|6666666.59341.peg.5590	fig|6666666.59347.peg.2829	fig|6666666.59346.peg.4095	fig|6666666.59348.peg.5466	fig|6666666.59349.peg.263	fig|6666666.59350.peg.3693	fig|6666666.59351.peg.5238	fig|6666666.59353.peg.4447	fig|6666666.59355.peg.4580	fig|6666666.59354.peg.5813

6462	Ribosome small subunit-stimulated GTPase EngC	fig|6666666.59329.peg.1687	fig|6666666.59330.peg.4941	fig|6666666.59331.peg.1788	fig|6666666.59332.peg.851	fig|6666666.59334.peg.2762	fig|6666666.59335.peg.2523	fig|6666666.59336.peg.1827	fig|6666666.59333.peg.4200	fig|6666666.59338.peg.5680	fig|6666666.59339.peg.8811	fig|6666666.59340.peg.2610	fig|6666666.59337.peg.5005	fig|6666666.59342.peg.4340	fig|6666666.71181.peg.1693	fig|6666666.59344.peg.598	fig|6666666.59345.peg.5087	fig|6666666.59341.peg.4935	fig|6666666.59347.peg.3796	fig|6666666.59346.peg.963	fig|6666666.59348.peg.2382	fig|6666666.59349.peg.3226	fig|6666666.59350.peg.7696	fig|6666666.59351.peg.3890	fig|6666666.59353.peg.1063	fig|6666666.59355.peg.3555	fig|6666666.59354.peg.5760

6466	Oligopeptide ABC transporter, periplasmic oligopeptide-binding protein OppA TC 3.A.1.5.1	fig|6666666.59329.peg.5676	fig|6666666.59330.peg.1909	fig|6666666.59331.peg.1640	fig|6666666.59332.peg.5993	fig|6666666.59334.peg.4457	fig|6666666.59335.peg.4919	fig|6666666.59336.peg.3479	fig|6666666.59333.peg.2926	fig|6666666.59338.peg.3595	fig|6666666.59339.peg.2914	fig|6666666.59340.peg.1848	fig|6666666.59337.peg.488	fig|6666666.59342.peg.4904	fig|6666666.71181.peg.1562	fig|6666666.59344.peg.1059	fig|6666666.59345.peg.2638	fig|6666666.59341.peg.5471	fig|6666666.59347.peg.5127	fig|6666666.59346.peg.537	fig|6666666.59348.peg.1194	fig|6666666.59349.peg.2090	fig|6666666.59350.peg.6168	fig|6666666.59351.peg.2603	fig|6666666.59353.peg.1749	fig|6666666.59355.peg.4313	fig|6666666.59354.peg.3390

5655	Transcriptional regulator, GntR family	fig|6666666.59329.peg.1980	fig|6666666.59330.peg.4224	fig|6666666.59331.peg.2007	fig|6666666.59332.peg.2988	fig|6666666.59334.peg.3574	fig|6666666.59335.peg.1157	fig|6666666.59336.peg.5526	fig|6666666.59333.peg.5469	fig|6666666.59338.peg.3907	fig|6666666.59339.peg.2400	fig|6666666.59340.peg.330	fig|6666666.59337.peg.1410	fig|6666666.59342.peg.2427	fig|6666666.71181.peg.4228	fig|6666666.59344.peg.3953	fig|6666666.59345.peg.4099	fig|6666666.59341.peg.5725	fig|6666666.59347.peg.3819	fig|6666666.59346.peg.4880	fig|6666666.59348.peg.494	fig|6666666.59349.peg.3145	fig|6666666.59350.peg.3020	fig|6666666.59351.peg.1225	fig|6666666.59353.peg.2753	fig|6666666.59355.peg.3060	fig|6666666.59354.peg.972

3671	ATP-dependent DNA helicase RecG EC 3.6.1.-	fig|6666666.59329.peg.1264	fig|6666666.59330.peg.1246	fig|6666666.59331.peg.1758	fig|6666666.59332.peg.2404	fig|6666666.59334.peg.191	fig|6666666.59335.peg.6192	fig|6666666.59336.peg.3126	fig|6666666.59333.peg.2896	fig|6666666.59338.peg.1366	fig|6666666.59339.peg.3457	fig|6666666.59340.peg.1514	fig|6666666.59337.peg.5117	fig|6666666.59342.peg.5707	fig|6666666.71181.peg.1278	fig|6666666.59344.peg.120	fig|6666666.59345.peg.2652	fig|6666666.59341.peg.1748	fig|6666666.59347.peg.5422	fig|6666666.59346.peg.1380	fig|6666666.59348.peg.4256	fig|6666666.59349.peg.4709	fig|6666666.59350.peg.117	fig|6666666.59351.peg.310	fig|6666666.59353.peg.440	fig|6666666.59355.peg.2167	fig|6666666.59354.peg.4403

3676	Methionyl-tRNA formyltransferase EC 2.1.2.9	fig|6666666.59329.peg.65	fig|6666666.59330.peg.645	fig|6666666.59331.peg.4354	fig|6666666.59332.peg.228	fig|6666666.59334.peg.3653	fig|6666666.59335.peg.2433	fig|6666666.59336.peg.6529	fig|6666666.59333.peg.1374	fig|6666666.59338.peg.2934	fig|6666666.59339.peg.8094	fig|6666666.59340.peg.2076	fig|6666666.59337.peg.3901	fig|6666666.59342.peg.4745	fig|6666666.71181.peg.3431	fig|6666666.59344.peg.4912	fig|6666666.59345.peg.4928	fig|6666666.59341.peg.3884	fig|6666666.59347.peg.4748	fig|6666666.59346.peg.1823	fig|6666666.59348.peg.50	fig|6666666.59349.peg.2817	fig|6666666.59350.peg.6369	fig|6666666.59351.peg.5055	fig|6666666.59353.peg.4621	fig|6666666.59355.peg.4711	fig|6666666.59354.peg.1430

11447	DNA repair protein RecN	fig|6666666.59329.peg.3960	fig|6666666.59330.peg.4602	fig|6666666.59331.peg.1330	fig|6666666.59332.peg.381	fig|6666666.59334.peg.4928	fig|6666666.59335.peg.5168	fig|6666666.59336.peg.220	fig|6666666.59333.peg.2758	fig|6666666.59338.peg.1077	fig|6666666.59339.peg.7278	fig|6666666.59340.peg.2065	fig|6666666.59337.peg.5540	fig|6666666.59342.peg.852	fig|6666666.71181.peg.1441	fig|6666666.59344.peg.1545	fig|6666666.59345.peg.3576	fig|6666666.59341.peg.5538	fig|6666666.59347.peg.4327	fig|6666666.59346.peg.692	fig|6666666.59348.peg.4866	fig|6666666.59349.peg.2387	fig|6666666.59350.peg.1050	fig|6666666.59351.peg.642	fig|6666666.59353.peg.4356	fig|6666666.59355.peg.4488	fig|6666666.59354.peg.3549

4425	Transcriptional repressor for pyruvate dehydrogenase complex	fig|6666666.59329.peg.2588	fig|6666666.59330.peg.3498	fig|6666666.59331.peg.1083	fig|6666666.59332.peg.301	fig|6666666.59334.peg.480	fig|6666666.59335.peg.2342	fig|6666666.59336.peg.1436	fig|6666666.59333.peg.147	fig|6666666.59338.peg.920	fig|6666666.59339.peg.7965	fig|6666666.59340.peg.2680	fig|6666666.59337.peg.2057	fig|6666666.59342.peg.1876	fig|6666666.71181.peg.5958	fig|6666666.59344.peg.4413	fig|6666666.59345.peg.2420	fig|6666666.59341.peg.5731	fig|6666666.59347.peg.3897	fig|6666666.59346.peg.585	fig|6666666.59348.peg.665	fig|6666666.59349.peg.3762	fig|6666666.59350.peg.3950	fig|6666666.59351.peg.2672	fig|6666666.59353.peg.3213	fig|6666666.59355.peg.3095	fig|6666666.59354.peg.770

9556	DNA repair protein RadA	fig|6666666.59329.peg.1052	fig|6666666.59330.peg.76	fig|6666666.59331.peg.4344	fig|6666666.59332.peg.3864	fig|6666666.59334.peg.487	fig|6666666.59335.peg.105	fig|6666666.59336.peg.6077	fig|6666666.59333.peg.2562	fig|6666666.59338.peg.1436	fig|6666666.59339.peg.5028	fig|6666666.59340.peg.134	fig|6666666.59337.peg.1311	fig|6666666.59342.peg.3794	fig|6666666.71181.peg.453	fig|6666666.59344.peg.1364	fig|6666666.59345.peg.2328	fig|6666666.59341.peg.3910	fig|6666666.59347.peg.2963	fig|6666666.59346.peg.2457	fig|6666666.59348.peg.1795	fig|6666666.59349.peg.4323	fig|6666666.59350.peg.1435	fig|6666666.59351.peg.283	fig|6666666.59353.peg.44	fig|6666666.59355.peg.652	fig|6666666.59354.peg.2854

1476	Cyclopropane-fatty-acyl-phospholipid synthase EC 2.1.1.79 , plant type	fig|6666666.59329.peg.2105	fig|6666666.59330.peg.2875	fig|6666666.59331.peg.3979	fig|6666666.59332.peg.4994	fig|6666666.59334.peg.2691	fig|6666666.59335.peg.1774	fig|6666666.59336.peg.551	fig|6666666.59333.peg.3156	fig|6666666.59338.peg.1417	fig|6666666.59339.peg.5789	fig|6666666.59340.peg.2762	fig|6666666.59337.peg.4610	fig|6666666.59342.peg.5761	fig|6666666.71181.peg.3616	fig|6666666.59344.peg.3522	fig|6666666.59345.peg.1967	fig|6666666.59341.peg.5064	fig|6666666.59347.peg.4127	fig|6666666.59346.peg.4933	fig|6666666.59348.peg.2669	fig|6666666.59349.peg.937	fig|6666666.59350.peg.7141	fig|6666666.59351.peg.1095	fig|6666666.59353.peg.4053	fig|6666666.59355.peg.2507	fig|6666666.59354.peg.1700

11276	Purine nucleoside phosphorylase EC 2.4.2.1	fig|6666666.59329.peg.2751	fig|6666666.59330.peg.705	fig|6666666.59331.peg.4423	fig|6666666.59332.peg.1117	fig|6666666.59334.peg.3120	fig|6666666.59335.peg.4654	fig|6666666.59336.peg.3681	fig|6666666.59333.peg.2343	fig|6666666.59338.peg.3052	fig|6666666.59339.peg.5023	fig|6666666.59340.peg.3206	fig|6666666.59337.peg.70	fig|6666666.59342.peg.3266	fig|6666666.71181.peg.3859	fig|6666666.59344.peg.3423	fig|6666666.59345.peg.3773	fig|6666666.59341.peg.2257	fig|6666666.59347.peg.5136	fig|6666666.59346.peg.2451	fig|6666666.59348.peg.3451	fig|6666666.59349.peg.1668	fig|6666666.59350.peg.2649	fig|6666666.59351.peg.1193	fig|6666666.59353.peg.544	fig|6666666.59355.peg.648	fig|6666666.59354.peg.817

8311	Acyl carrier protein	fig|6666666.59329.peg.4183	fig|6666666.59330.peg.3625	fig|6666666.59331.peg.1746	fig|6666666.59332.peg.3475	fig|6666666.59334.peg.3242	fig|6666666.59335.peg.5374	fig|6666666.59336.peg.3339	fig|6666666.59333.peg.1587	fig|6666666.59338.peg.6040	fig|6666666.59339.peg.3179	fig|6666666.59340.peg.404	fig|6666666.59337.peg.2686	fig|6666666.59342.peg.1381	fig|6666666.71181.peg.1624	fig|6666666.59344.peg.2435	fig|6666666.59345.peg.1062	fig|6666666.59341.peg.17	fig|6666666.59347.peg.4086	fig|6666666.59346.peg.108	fig|6666666.59348.peg.1240	fig|6666666.59349.peg.3912	fig|6666666.59350.peg.4519	fig|6666666.59351.peg.673	fig|6666666.59353.peg.315	fig|6666666.59355.peg.1355	fig|6666666.59354.peg.2159

4823	Low molecular weight protein tyrosine phosphatase EC 3.1.3.48	fig|6666666.59329.peg.1605	fig|6666666.59330.peg.2247	fig|6666666.59331.peg.706	fig|6666666.59332.peg.2137	fig|6666666.59334.peg.1183	fig|6666666.59335.peg.5422	fig|6666666.59336.peg.1103	fig|6666666.59333.peg.4657	fig|6666666.59338.peg.5841	fig|6666666.59339.peg.6586	fig|6666666.59340.peg.1230	fig|6666666.59337.peg.3853	fig|6666666.59342.peg.1659	fig|6666666.71181.peg.215	fig|6666666.59344.peg.265	fig|6666666.59345.peg.1075	fig|6666666.59341.peg.1110	fig|6666666.59347.peg.2590	fig|6666666.59346.peg.5177	fig|6666666.59348.peg.5234	fig|6666666.59349.peg.266	fig|6666666.59350.peg.5345	fig|6666666.59351.peg.4117	fig|6666666.59353.peg.606	fig|6666666.59355.peg.88	fig|6666666.59354.peg.3097

6280	Ubiquinone menaquinone biosynthesis methyltransferase UbiE EC 2.1.1.- 2-heptaprenyl-1,4-naphthoquinone methyltransferase EC 2.1.1.163	fig|6666666.59329.peg.3193	fig|6666666.59330.peg.1937	fig|6666666.59331.peg.195	fig|6666666.59332.peg.2732	fig|6666666.59334.peg.5501	fig|6666666.59335.peg.40	fig|6666666.59336.peg.4901	fig|6666666.59333.peg.2051	fig|6666666.59338.peg.2985	fig|6666666.59339.peg.4447	fig|6666666.59340.peg.2891	fig|6666666.59337.peg.958	fig|6666666.59342.peg.6453	fig|6666666.71181.peg.238	fig|6666666.59344.peg.3417	fig|6666666.59345.peg.1842	fig|6666666.59341.peg.475	fig|6666666.59347.peg.4634	fig|6666666.59346.peg.3355	fig|6666666.59348.peg.2992	fig|6666666.59349.peg.760	fig|6666666.59350.peg.7567	fig|6666666.59351.peg.963	fig|6666666.59353.peg.583	fig|6666666.59355.peg.3238	fig|6666666.59354.peg.5288

6282	A G-specific adenine glycosylase EC 3.2.2.-	fig|6666666.59329.peg.1534	fig|6666666.59330.peg.332	fig|6666666.59331.peg.4811	fig|6666666.59332.peg.6144	fig|6666666.59334.peg.1378	fig|6666666.59335.peg.3040	fig|6666666.59336.peg.5569	fig|6666666.59333.peg.754	fig|6666666.59338.peg.5286	fig|6666666.59339.peg.7808	fig|6666666.59340.peg.1331	fig|6666666.59337.peg.511	fig|6666666.59342.peg.5166	fig|6666666.71181.peg.1877	fig|6666666.59344.peg.4353	fig|6666666.59345.peg.5001	fig|6666666.59341.peg.3065	fig|6666666.59347.peg.3876	fig|6666666.59346.peg.4581	fig|6666666.59348.peg.233	fig|6666666.59349.peg.3656	fig|6666666.59350.peg.730	fig|6666666.59351.peg.298	fig|6666666.59353.peg.3242	fig|6666666.59355.peg.3125	fig|6666666.59354.peg.3160

11255	Enolase EC 4.2.1.11	fig|6666666.59329.peg.2718	fig|6666666.59330.peg.2465	fig|6666666.59331.peg.3029	fig|6666666.59332.peg.197	fig|6666666.59334.peg.332	fig|6666666.59335.peg.1503	fig|6666666.59336.peg.4981	fig|6666666.59333.peg.4775	fig|6666666.59338.peg.2737	fig|6666666.59339.peg.7391	fig|6666666.59340.peg.48	fig|6666666.59337.peg.576	fig|6666666.59342.peg.3538	fig|6666666.71181.peg.3771	fig|6666666.59344.peg.2233	fig|6666666.59345.peg.1838	fig|6666666.59341.peg.1322	fig|6666666.59347.peg.3166	fig|6666666.59346.peg.3151	fig|6666666.59348.peg.3626	fig|6666666.59349.peg.3470	fig|6666666.59350.peg.3387	fig|6666666.59351.peg.2587	fig|6666666.59353.peg.181	fig|6666666.59355.peg.2949	fig|6666666.59354.peg.918

3059	ATP synthase gamma chain EC 3.6.3.14	fig|6666666.59329.peg.1032	fig|6666666.59330.peg.611	fig|6666666.59331.peg.406	fig|6666666.59332.peg.105	fig|6666666.59334.peg.20	fig|6666666.59335.peg.797	fig|6666666.59336.peg.1519	fig|6666666.59333.peg.23	fig|6666666.59338.peg.6206	fig|6666666.59339.peg.2296	fig|6666666.59340.peg.2144	fig|6666666.59337.peg.896	fig|6666666.59342.peg.2039	fig|6666666.71181.peg.597	fig|6666666.59344.peg.4270	fig|6666666.59345.peg.4960	fig|6666666.59341.peg.2135	fig|6666666.59347.peg.4778	fig|6666666.59346.peg.1408	fig|6666666.59348.peg.339	fig|6666666.59349.peg.1035	fig|6666666.59350.peg.6489	fig|6666666.59351.peg.5354	fig|6666666.59353.peg.3083	fig|6666666.59355.peg.2265	fig|6666666.59354.peg.6424

9628	LSU m5C1962 methyltransferase RlmI	fig|6666666.59329.peg.3298	fig|6666666.59330.peg.1039	fig|6666666.59331.peg.5445	fig|6666666.59332.peg.2230	fig|6666666.59334.peg.1646	fig|6666666.59335.peg.1227	fig|6666666.59336.peg.5608	fig|6666666.59333.peg.1640	fig|6666666.59338.peg.2751	fig|6666666.59339.peg.1569	fig|6666666.59340.peg.1188	fig|6666666.59337.peg.3679	fig|6666666.59342.peg.5213	fig|6666666.71181.peg.275	fig|6666666.59344.peg.1202	fig|6666666.59345.peg.3283	fig|6666666.59341.peg.4718	fig|6666666.59347.peg.1768	fig|6666666.59346.peg.5329	fig|6666666.59348.peg.1674	fig|6666666.59349.peg.2135	fig|6666666.59350.peg.1025	fig|6666666.59351.peg.4322	fig|6666666.59353.peg.1705	fig|6666666.59355.peg.1015	fig|6666666.59354.peg.716

9626	D-tyrosyl-tRNA Tyr deacylase	fig|6666666.59329.peg.352	fig|6666666.59330.peg.408	fig|6666666.59331.peg.4745	fig|6666666.59332.peg.2111	fig|6666666.59334.peg.3233	fig|6666666.59335.peg.730	fig|6666666.59336.peg.440	fig|6666666.59333.peg.493	fig|6666666.59338.peg.4999	fig|6666666.59339.peg.6182	fig|6666666.59340.peg.604	fig|6666666.59337.peg.993	fig|6666666.59342.peg.5960	fig|6666666.71181.peg.2597	fig|6666666.59344.peg.911	fig|6666666.59345.peg.2685	fig|6666666.59341.peg.5317	fig|6666666.59347.peg.4456	fig|6666666.59346.peg.2604	fig|6666666.59348.peg.679	fig|6666666.59349.peg.1113	fig|6666666.59350.peg.6560	fig|6666666.59351.peg.66	fig|6666666.59353.peg.2618	fig|6666666.59355.peg.2586	fig|6666666.59354.peg.5038

4058	Amidophosphoribosyltransferase EC 2.4.2.14	fig|6666666.59329.peg.3489	fig|6666666.59330.peg.4227	fig|6666666.59331.peg.3252	fig|6666666.59332.peg.2749	fig|6666666.59334.peg.5010	fig|6666666.59335.peg.2322	fig|6666666.59336.peg.3035	fig|6666666.59333.peg.109	fig|6666666.59338.peg.2038	fig|6666666.59339.peg.2976	fig|6666666.59340.peg.387	fig|6666666.59337.peg.3731	fig|6666666.59342.peg.4066	fig|6666666.71181.peg.601	fig|6666666.59344.peg.4277	fig|6666666.59345.peg.1200	fig|6666666.59341.peg.2451	fig|6666666.59347.peg.2452	fig|6666666.59346.peg.57	fig|6666666.59348.peg.1718	fig|6666666.59349.peg.3463	fig|6666666.59350.peg.1439	fig|6666666.59351.peg.3575	fig|6666666.59353.peg.3077	fig|6666666.59355.peg.5546	fig|6666666.59354.peg.2960

3615	Inosine-5'-monophosphate dehydrogenase EC 1.1.1.205	fig|6666666.59329.peg.5377	fig|6666666.59330.peg.1151	fig|6666666.59331.peg.4763	fig|6666666.59332.peg.771	fig|6666666.59334.peg.3119	fig|6666666.59335.peg.1962	fig|6666666.59336.peg.2226	fig|6666666.59333.peg.2346	fig|6666666.59338.peg.6209	fig|6666666.59339.peg.6892	fig|6666666.59340.peg.1216	fig|6666666.59337.peg.5478	fig|6666666.59342.peg.2257	fig|6666666.71181.peg.1783	fig|6666666.59344.peg.1462	fig|6666666.59345.peg.3664	fig|6666666.59341.peg.5043	fig|6666666.59347.peg.5140	fig|6666666.59346.peg.2188	fig|6666666.59348.peg.5147	fig|6666666.59349.peg.1682	fig|6666666.59350.peg.146	fig|6666666.59351.peg.384	fig|6666666.59353.peg.2723	fig|6666666.59355.peg.1758	fig|6666666.59354.peg.6037

2565	Transcriptional regulator, MerR family	fig|6666666.59329.peg.537	fig|6666666.59330.peg.4769	fig|6666666.59331.peg.5779	fig|6666666.59332.peg.1289	fig|6666666.59334.peg.1609	fig|6666666.59335.peg.2606	fig|6666666.59336.peg.5254	fig|6666666.59333.peg.5001	fig|6666666.59338.peg.1372	fig|6666666.59339.peg.6433	fig|6666666.59340.peg.254	fig|6666666.59337.peg.4634	fig|6666666.59342.peg.4775	fig|6666666.71181.peg.2293	fig|6666666.59344.peg.5060	fig|6666666.59345.peg.1721	fig|6666666.59341.peg.1593	fig|6666666.59347.peg.1406	fig|6666666.59346.peg.2372	fig|6666666.59348.peg.4144	fig|6666666.59349.peg.2359	fig|6666666.59350.peg.831	fig|6666666.59351.peg.1120	fig|6666666.59353.peg.4056	fig|6666666.59355.peg.3874	fig|6666666.59354.peg.5590

4449	TldE PmbA protein, part of proposed TldE TldD proteolytic complex PMID 12029038	fig|6666666.59329.peg.808	fig|6666666.59330.peg.31	fig|6666666.59331.peg.4072	fig|6666666.59332.peg.4520	fig|6666666.59334.peg.111	fig|6666666.59335.peg.3036	fig|6666666.59336.peg.5208	fig|6666666.59333.peg.255	fig|6666666.59338.peg.2727	fig|6666666.59339.peg.7802	fig|6666666.59340.peg.1781	fig|6666666.59337.peg.1213	fig|6666666.59342.peg.5230	fig|6666666.71181.peg.995	fig|6666666.59344.peg.1753	fig|6666666.59345.peg.4867	fig|6666666.59341.peg.1236	fig|6666666.59347.peg.4879	fig|6666666.59346.peg.1541	fig|6666666.59348.peg.4207	fig|6666666.59349.peg.14	fig|6666666.59350.peg.737	fig|6666666.59351.peg.3432	fig|6666666.59353.peg.2311	fig|6666666.59355.peg.1586	fig|6666666.59354.peg.4210

5194	Phenazine biosynthesis protein PhzF like	fig|6666666.59329.peg.1252	fig|6666666.59330.peg.2914	fig|6666666.59331.peg.4844	fig|6666666.59332.peg.2589	fig|6666666.59334.peg.1005	fig|6666666.59335.peg.3712	fig|6666666.59336.peg.3331	fig|6666666.59333.peg.4415	fig|6666666.59338.peg.5362	fig|6666666.59339.peg.2610	fig|6666666.59340.peg.1016	fig|6666666.59337.peg.1932	fig|6666666.59342.peg.5940	fig|6666666.71181.peg.1801	fig|6666666.59344.peg.3101	fig|6666666.59345.peg.4024	fig|6666666.59341.peg.1287	fig|6666666.59347.peg.1672	fig|6666666.59346.peg.209	fig|6666666.59348.peg.4181	fig|6666666.59349.peg.681	fig|6666666.59350.peg.7188	fig|6666666.59351.peg.1026	fig|6666666.59353.peg.2250	fig|6666666.59355.peg.1942	fig|6666666.59354.peg.5207

643	Pantoate--beta-alanine ligase EC 6.3.2.1	fig|6666666.59329.peg.4155	fig|6666666.59330.peg.4041	fig|6666666.59331.peg.5523	fig|6666666.59332.peg.3811	fig|6666666.59334.peg.818	fig|6666666.59335.peg.2330	fig|6666666.59336.peg.2747	fig|6666666.59333.peg.135	fig|6666666.59338.peg.207	fig|6666666.59339.peg.4764	fig|6666666.59340.peg.1960	fig|6666666.59337.peg.619	fig|6666666.59342.peg.1577	fig|6666666.71181.peg.2845	fig|6666666.59344.peg.4433	fig|6666666.59345.peg.2408	fig|6666666.59341.peg.1556	fig|6666666.59347.peg.3963	fig|6666666.59346.peg.3819	fig|6666666.59348.peg.1694	fig|6666666.59349.peg.2472	fig|6666666.59350.peg.636	fig|6666666.59351.peg.358	fig|6666666.59353.peg.3197	fig|6666666.59355.peg.3078	fig|6666666.59354.peg.42

1675	Cysteine desulfurase CsdA-CsdE EC 2.8.1.7 , main protein CsdA	fig|6666666.59329.peg.2919	fig|6666666.59330.peg.2801	fig|6666666.59331.peg.5098	fig|6666666.59332.peg.865	fig|6666666.59334.peg.1168	fig|6666666.59335.peg.106	fig|6666666.59336.peg.130	fig|6666666.59333.peg.1924	fig|6666666.59338.peg.5206	fig|6666666.59339.peg.5029	fig|6666666.59340.peg.2200	fig|6666666.59337.peg.2366	fig|6666666.59342.peg.6448	fig|6666666.71181.peg.2558	fig|6666666.59344.peg.2622	fig|6666666.59345.peg.3615	fig|6666666.59341.peg.372	fig|6666666.59347.peg.1964	fig|6666666.59346.peg.1848	fig|6666666.59348.peg.4249	fig|6666666.59349.peg.2515	fig|6666666.59350.peg.3492	fig|6666666.59351.peg.4844	fig|6666666.59353.peg.1358	fig|6666666.59355.peg.1541	fig|6666666.59354.peg.4781

11213	Adenine phosphoribosyltransferase EC 2.4.2.7	fig|6666666.59329.peg.3455	fig|6666666.59330.peg.4119	fig|6666666.59331.peg.3902	fig|6666666.59332.peg.5826	fig|6666666.59334.peg.2757	fig|6666666.59335.peg.3291	fig|6666666.59336.peg.5176	fig|6666666.59333.peg.2535	fig|6666666.59338.peg.5169	fig|6666666.59339.peg.3061	fig|6666666.59340.peg.191	fig|6666666.59337.peg.3736	fig|6666666.59342.peg.4072	fig|6666666.71181.peg.3501	fig|6666666.59344.peg.5523	fig|6666666.59345.peg.1195	fig|6666666.59341.peg.2457	fig|6666666.59347.peg.2448	fig|6666666.59346.peg.62	fig|6666666.59348.peg.454	fig|6666666.59349.peg.4591	fig|6666666.59350.peg.2036	fig|6666666.59351.peg.2753	fig|6666666.59353.peg.4079	fig|6666666.59355.peg.4155	fig|6666666.59354.peg.5009

11218	Multiple antibiotic resistance protein marC	fig|6666666.59329.peg.1798	fig|6666666.59330.peg.2242	fig|6666666.59331.peg.1040	fig|6666666.59332.peg.488	fig|6666666.59334.peg.3165	fig|6666666.59335.peg.4276	fig|6666666.59336.peg.4151	fig|6666666.59333.peg.5101	fig|6666666.59338.peg.2859	fig|6666666.59339.peg.233	fig|6666666.59340.peg.85	fig|6666666.59337.peg.1645	fig|6666666.59342.peg.4956	fig|6666666.71181.peg.1108	fig|6666666.59344.peg.420	fig|6666666.59345.peg.5475	fig|6666666.59341.peg.5362	fig|6666666.59347.peg.2569	fig|6666666.59346.peg.1097	fig|6666666.59348.peg.579	fig|6666666.59349.peg.4710	fig|6666666.59350.peg.652	fig|6666666.59351.peg.3708	fig|6666666.59353.peg.2168	fig|6666666.59355.peg.1265	fig|6666666.59354.peg.2422

4858	16S rRNA processing protein RimM	fig|6666666.59329.peg.337	fig|6666666.59330.peg.2845	fig|6666666.59331.peg.1986	fig|6666666.59332.peg.314	fig|6666666.59334.peg.267	fig|6666666.59335.peg.5392	fig|6666666.59336.peg.3004	fig|6666666.59333.peg.160	fig|6666666.59338.peg.2607	fig|6666666.59339.peg.7934	fig|6666666.59340.peg.3782	fig|6666666.59337.peg.2042	fig|6666666.59342.peg.2098	fig|6666666.71181.peg.3956	fig|6666666.59344.peg.5442	fig|6666666.59345.peg.2432	fig|6666666.59341.peg.1343	fig|6666666.59347.peg.3885	fig|6666666.59346.peg.570	fig|6666666.59348.peg.1260	fig|6666666.59349.peg.604	fig|6666666.59350.peg.5986	fig|6666666.59351.peg.541	fig|6666666.59353.peg.4813	fig|6666666.59355.peg.4867	fig|6666666.59354.peg.3771

11189	GTPase and tRNA-U34 5-formylation enzyme TrmE	fig|6666666.59329.peg.302	fig|6666666.59330.peg.1430	fig|6666666.59331.peg.2813	fig|6666666.59332.peg.4351	fig|6666666.59334.peg.1259	fig|6666666.59335.peg.4149	fig|6666666.59336.peg.1735	fig|6666666.59333.peg.915	fig|6666666.59338.peg.3021	fig|6666666.59339.peg.8025	fig|6666666.59340.peg.3684	fig|6666666.59337.peg.566	fig|6666666.59342.peg.1189	fig|6666666.71181.peg.1422	fig|6666666.59344.peg.2114	fig|6666666.59345.peg.4972	fig|6666666.59341.peg.3032	fig|6666666.59347.peg.3095	fig|6666666.59346.peg.1184	fig|6666666.59348.peg.307	fig|6666666.59349.peg.3662	fig|6666666.59350.peg.1616	fig|6666666.59351.peg.3998	fig|6666666.59353.peg.2654	fig|6666666.59355.peg.5150	fig|6666666.59354.peg.5585

1906	Gamma-glutamyltranspeptidase EC 2.3.2.2	fig|6666666.59329.peg.374	fig|6666666.59330.peg.992	fig|6666666.59331.peg.4701	fig|6666666.59332.peg.266	fig|6666666.59334.peg.2160	fig|6666666.59335.peg.4451	fig|6666666.59336.peg.192	fig|6666666.59333.peg.1582	fig|6666666.59338.peg.5823	fig|6666666.59339.peg.7659	fig|6666666.59340.peg.1342	fig|6666666.59337.peg.2539	fig|6666666.59342.peg.1370	fig|6666666.71181.peg.894	fig|6666666.59344.peg.4775	fig|6666666.59345.peg.5530	fig|6666666.59341.peg.4941	fig|6666666.59347.peg.5292	fig|6666666.59346.peg.918	fig|6666666.59348.peg.2649	fig|6666666.59349.peg.1607	fig|6666666.59350.peg.4614	fig|6666666.59351.peg.1033	fig|6666666.59353.peg.4537	fig|6666666.59355.peg.915	fig|6666666.59354.peg.1738

10846	DNA polymerase III beta subunit EC 2.7.7.7	fig|6666666.59329.peg.3810	fig|6666666.59330.peg.1101	fig|6666666.59331.peg.3667	fig|6666666.59332.peg.84	fig|6666666.59334.peg.4246	fig|6666666.59335.peg.775	fig|6666666.59336.peg.10	fig|6666666.59333.peg.50	fig|6666666.59338.peg.1267	fig|6666666.59339.peg.8038	fig|6666666.59340.peg.1132	fig|6666666.59337.peg.874	fig|6666666.59342.peg.519	fig|6666666.71181.peg.4960	fig|6666666.59344.peg.4170	fig|6666666.59345.peg.4980	fig|6666666.59341.peg.1041	fig|6666666.59347.peg.4799	fig|6666666.59346.peg.1196	fig|6666666.59348.peg.316	fig|6666666.59349.peg.1006	fig|6666666.59350.peg.2729	fig|6666666.59351.peg.5029	fig|6666666.59353.peg.3056	fig|6666666.59355.peg.5146	fig|6666666.59354.peg.590

6936	UDP-N-acetylenolpyruvoylglucosamine reductase EC 1.1.1.158	fig|6666666.59329.peg.2427	fig|6666666.59330.peg.4558	fig|6666666.59331.peg.5401	fig|6666666.59332.peg.3160	fig|6666666.59334.peg.2119	fig|6666666.59335.peg.414	fig|6666666.59336.peg.3400	fig|6666666.59333.peg.5077	fig|6666666.59338.peg.1494	fig|6666666.59339.peg.6360	fig|6666666.59340.peg.884	fig|6666666.59337.peg.5512	fig|6666666.59342.peg.1706	fig|6666666.71181.peg.4966	fig|6666666.59344.peg.6192	fig|6666666.59345.peg.5712	fig|6666666.59341.peg.5749	fig|6666666.59347.peg.5031	fig|6666666.59346.peg.4266	fig|6666666.59348.peg.27	fig|6666666.59349.peg.3361	fig|6666666.59350.peg.1042	fig|6666666.59351.peg.5022	fig|6666666.59353.peg.5080	fig|6666666.59355.peg.5132	fig|6666666.59354.peg.1666

6937	Succinate dehydrogenase flavoprotein subunit EC 1.3.99.1	fig|6666666.59329.peg.493	fig|6666666.59330.peg.377	fig|6666666.59331.peg.5303	fig|6666666.59332.peg.749	fig|6666666.59334.peg.5248	fig|6666666.59335.peg.2743	fig|6666666.59336.peg.5362	fig|6666666.59333.peg.436	fig|6666666.59338.peg.813	fig|6666666.59339.peg.6400	fig|6666666.59340.peg.1155	fig|6666666.59337.peg.4108	fig|6666666.59342.peg.475	fig|6666666.71181.peg.1431	fig|6666666.59344.peg.604	fig|6666666.59345.peg.5048	fig|6666666.59341.peg.167	fig|6666666.59347.peg.3183	fig|6666666.59346.peg.970	fig|6666666.59348.peg.262	fig|6666666.59349.peg.4311	fig|6666666.59350.peg.7947	fig|6666666.59351.peg.590	fig|6666666.59353.peg.1058	fig|6666666.59355.peg.1838	fig|6666666.59354.peg.5405

6935	Unsaturated fatty acid biosythesis repressor FabR, TetR family	fig|6666666.59329.peg.4178	fig|6666666.59330.peg.4866	fig|6666666.59331.peg.4346	fig|6666666.59332.peg.568	fig|6666666.59334.peg.1474	fig|6666666.59335.peg.4037	fig|6666666.59336.peg.5909	fig|6666666.59333.peg.1964	fig|6666666.59338.peg.3380	fig|6666666.59339.peg.8592	fig|6666666.59340.peg.231	fig|6666666.59337.peg.1664	fig|6666666.59342.peg.5691	fig|6666666.71181.peg.4440	fig|6666666.59344.peg.1025	fig|6666666.59345.peg.5633	fig|6666666.59341.peg.3564	fig|6666666.59347.peg.4651	fig|6666666.59346.peg.1651	fig|6666666.59348.peg.2990	fig|6666666.59349.peg.920	fig|6666666.59350.peg.6373	fig|6666666.59351.peg.3870	fig|6666666.59353.peg.2518	fig|6666666.59355.peg.4876	fig|6666666.59354.peg.4053

4075	Oxidoreductase, aldo keto reductase family	fig|6666666.59329.peg.220	fig|6666666.59330.peg.1747	fig|6666666.59331.peg.3412	fig|6666666.59332.peg.4209	fig|6666666.59334.peg.4629	fig|6666666.59335.peg.5340	fig|6666666.59336.peg.2947	fig|6666666.59333.peg.4648	fig|6666666.59338.peg.1806	fig|6666666.59339.peg.6613	fig|6666666.59340.peg.2488	fig|6666666.59337.peg.3646	fig|6666666.59342.peg.3289	fig|6666666.71181.peg.2138	fig|6666666.59344.peg.5254	fig|6666666.59345.peg.2040	fig|6666666.59341.peg.2324	fig|6666666.59347.peg.4576	fig|6666666.59346.peg.2616	fig|6666666.59348.peg.1894	fig|6666666.59349.peg.4161	fig|6666666.59350.peg.2193	fig|6666666.59351.peg.4277	fig|6666666.59353.peg.2833	fig|6666666.59355.peg.2492	fig|6666666.59354.peg.6454

9665	N-acetylmuramoyl-L-alanine amidase EC 3.5.1.28 AmpD	fig|6666666.59329.peg.2587	fig|6666666.59330.peg.3499	fig|6666666.59331.peg.1084	fig|6666666.59332.peg.302	fig|6666666.59334.peg.479	fig|6666666.59335.peg.3814	fig|6666666.59336.peg.1435	fig|6666666.59333.peg.149	fig|6666666.59338.peg.921	fig|6666666.59339.peg.7956	fig|6666666.59340.peg.305	fig|6666666.59337.peg.2056	fig|6666666.59342.peg.1875	fig|6666666.71181.peg.4580	fig|6666666.59344.peg.376	fig|6666666.59345.peg.2421	fig|6666666.59341.peg.5730	fig|6666666.59347.peg.3896	fig|6666666.59346.peg.584	fig|6666666.59348.peg.670	fig|6666666.59349.peg.2633	fig|6666666.59350.peg.1404	fig|6666666.59351.peg.280	fig|6666666.59353.peg.4824	fig|6666666.59355.peg.4896	fig|6666666.59354.peg.6335

9669	UDP-glucose 4-epimerase EC 5.1.3.2	fig|6666666.59329.peg.2537	fig|6666666.59330.peg.3477	fig|6666666.59331.peg.4788	fig|6666666.59332.peg.282	fig|6666666.59334.peg.1044	fig|6666666.59335.peg.500	fig|6666666.59336.peg.1923	fig|6666666.59333.peg.1142	fig|6666666.59338.peg.2496	fig|6666666.59339.peg.6318	fig|6666666.59340.peg.3015	fig|6666666.59337.peg.3033	fig|6666666.59342.peg.263	fig|6666666.71181.peg.2761	fig|6666666.59344.peg.416	fig|6666666.59345.peg.89	fig|6666666.59341.peg.1066	fig|6666666.59347.peg.4978	fig|6666666.59346.peg.1701	fig|6666666.59348.peg.12	fig|6666666.59349.peg.4883	fig|6666666.59350.peg.3910	fig|6666666.59351.peg.149	fig|6666666.59353.peg.603	fig|6666666.59355.peg.85	fig|6666666.59354.peg.4720

8973	SSU ribosomal protein S19p S15e	fig|6666666.59329.peg.6290	fig|6666666.59330.peg.554	fig|6666666.59331.peg.48	fig|6666666.59332.peg.2139	fig|6666666.59334.peg.1485	fig|6666666.59335.peg.4348	fig|6666666.59336.peg.146	fig|6666666.59333.peg.366	fig|6666666.59338.peg.1389	fig|6666666.59339.peg.8268	fig|6666666.59340.peg.55	fig|6666666.59337.peg.388	fig|6666666.59342.peg.304	fig|6666666.71181.peg.3456	fig|6666666.59344.peg.4939	fig|6666666.59345.peg.2535	fig|6666666.59341.peg.2498	fig|6666666.59347.peg.4823	fig|6666666.59346.peg.850	fig|6666666.59348.peg.415	fig|6666666.59349.peg.1098	fig|6666666.59350.peg.6235	fig|6666666.59351.peg.4814	fig|6666666.59353.peg.4594	fig|6666666.59355.peg.4737	fig|6666666.59354.peg.1747

6130	Putative diheme cytochrome c-553	fig|6666666.59329.peg.4379	fig|6666666.59330.peg.3191	fig|6666666.59331.peg.2319	fig|6666666.59332.peg.6157	fig|6666666.59334.peg.1461	fig|6666666.59335.peg.3537	fig|6666666.59336.peg.6565	fig|6666666.59333.peg.4305	fig|6666666.59338.peg.4743	fig|6666666.59339.peg.1324	fig|6666666.59340.peg.3163	fig|6666666.59337.peg.1444	fig|6666666.59342.peg.4023	fig|6666666.71181.peg.1747	fig|6666666.59344.peg.1615	fig|6666666.59345.peg.3234	fig|6666666.59341.peg.3926	fig|6666666.59347.peg.459	fig|6666666.59346.peg.1532	fig|6666666.59348.peg.2173	fig|6666666.59349.peg.138	fig|6666666.59350.peg.7328	fig|6666666.59351.peg.833	fig|6666666.59353.peg.3217	fig|6666666.59355.peg.3099	fig|6666666.59354.peg.202

9207	LSU ribosomal protein L29p L35e	fig|6666666.59329.peg.717	fig|6666666.59330.peg.558	fig|6666666.59331.peg.44	fig|6666666.59332.peg.2143	fig|6666666.59334.peg.1489	fig|6666666.59335.peg.4351	fig|6666666.59336.peg.150	fig|6666666.59333.peg.370	fig|6666666.59338.peg.1385	fig|6666666.59339.peg.8272	fig|6666666.59340.peg.60	fig|6666666.59337.peg.384	fig|6666666.59342.peg.308	fig|6666666.71181.peg.3452	fig|6666666.59344.peg.4935	fig|6666666.59345.peg.2531	fig|6666666.59341.peg.2502	fig|6666666.59347.peg.4827	fig|6666666.59346.peg.854	fig|6666666.59348.peg.1764	fig|6666666.59349.peg.1102	fig|6666666.59350.peg.7709	fig|6666666.59351.peg.4809	fig|6666666.59353.peg.4598	fig|6666666.59355.peg.4733	fig|6666666.59354.peg.1743

3401	Acetylornithine deacetylase EC 3.5.1.16	fig|6666666.59329.peg.5322	fig|6666666.59330.peg.4589	fig|6666666.59331.peg.1977	fig|6666666.59332.peg.1250	fig|6666666.59334.peg.3888	fig|6666666.59335.peg.1472	fig|6666666.59336.peg.1599	fig|6666666.59333.peg.1845	fig|6666666.59338.peg.5199	fig|6666666.59339.peg.7141	fig|6666666.59340.peg.640	fig|6666666.59337.peg.3543	fig|6666666.59342.peg.6129	fig|6666666.71181.peg.1501	fig|6666666.59344.peg.141	fig|6666666.59345.peg.5398	fig|6666666.59341.peg.950	fig|6666666.59347.peg.2845	fig|6666666.59346.peg.3544	fig|6666666.59348.peg.2135	fig|6666666.59349.peg.1416	fig|6666666.59350.peg.1746	fig|6666666.59351.peg.2237	fig|6666666.59353.peg.3902	fig|6666666.59355.peg.242	fig|6666666.59354.peg.480

10289	Endonuclease III EC 4.2.99.18	fig|6666666.59329.peg.786	fig|6666666.59330.peg.3378	fig|6666666.59331.peg.629	fig|6666666.59332.peg.2570	fig|6666666.59334.peg.630	fig|6666666.59335.peg.882	fig|6666666.59336.peg.4283	fig|6666666.59333.peg.4813	fig|6666666.59338.peg.4298	fig|6666666.59339.peg.52	fig|6666666.59340.peg.505	fig|6666666.59337.peg.4668	fig|6666666.59342.peg.1054	fig|6666666.71181.peg.2905	fig|6666666.59344.peg.3461	fig|6666666.59345.peg.1127	fig|6666666.59341.peg.2707	fig|6666666.59347.peg.5387	fig|6666666.59346.peg.5261	fig|6666666.59348.peg.86	fig|6666666.59349.peg.4635	fig|6666666.59350.peg.5497	fig|6666666.59351.peg.5073	fig|6666666.59353.peg.1415	fig|6666666.59355.peg.2459	fig|6666666.59354.peg.6159

7695	Transcriptional regulator, MarR family	fig|6666666.59329.peg.3890	fig|6666666.59330.peg.2733	fig|6666666.59331.peg.6296	fig|6666666.59332.peg.3847	fig|6666666.59334.peg.2962	fig|6666666.59335.peg.5537	fig|6666666.59336.peg.6505	fig|6666666.59333.peg.1457	fig|6666666.59338.peg.2638	fig|6666666.59339.peg.3793	fig|6666666.59340.peg.2146	fig|6666666.59337.peg.3591	fig|6666666.59342.peg.6175	fig|6666666.71181.peg.650	fig|6666666.59344.peg.2265	fig|6666666.59345.peg.2134	fig|6666666.59341.peg.5428	fig|6666666.59347.peg.3339	fig|6666666.59346.peg.1206	fig|6666666.59348.peg.2387	fig|6666666.59349.peg.4302	fig|6666666.59350.peg.5277	fig|6666666.59351.peg.1010	fig|6666666.59353.peg.3935	fig|6666666.59355.peg.181	fig|6666666.59354.peg.311

2815	Tyrosine-protein kinase Wzc EC 2.7.10.2	fig|6666666.59329.peg.2372	fig|6666666.59330.peg.1347	fig|6666666.59331.peg.620	fig|6666666.59332.peg.2831	fig|6666666.59334.peg.2794	fig|6666666.59335.peg.6097	fig|6666666.59336.peg.5031	fig|6666666.59333.peg.991	fig|6666666.59338.peg.5852	fig|6666666.59339.peg.6412	fig|6666666.59340.peg.241	fig|6666666.59337.peg.4444	fig|6666666.59342.peg.1661	fig|6666666.71181.peg.2450	fig|6666666.59344.peg.4657	fig|6666666.59345.peg.628	fig|6666666.59341.peg.2645	fig|6666666.59347.peg.4974	fig|6666666.59346.peg.3056	fig|6666666.59348.peg.5322	fig|6666666.59349.peg.256	fig|6666666.59350.peg.2160	fig|6666666.59351.peg.247	fig|6666666.59353.peg.3649	fig|6666666.59355.peg.4296	fig|6666666.59354.peg.843

4246	Putative NAD P -dependent oxidoreductase EC-YbbO	fig|6666666.59329.peg.3567	fig|6666666.59330.peg.871	fig|6666666.59331.peg.2488	fig|6666666.59332.peg.1562	fig|6666666.59334.peg.4891	fig|6666666.59335.peg.2133	fig|6666666.59336.peg.6310	fig|6666666.59333.peg.730	fig|6666666.59338.peg.2796	fig|6666666.59339.peg.1751	fig|6666666.59340.peg.3348	fig|6666666.59337.peg.5066	fig|6666666.59342.peg.1953	fig|6666666.71181.peg.3618	fig|6666666.59344.peg.471	fig|6666666.59345.peg.4159	fig|6666666.59341.peg.44	fig|6666666.59347.peg.4435	fig|6666666.59346.peg.4804	fig|6666666.59348.peg.3557	fig|6666666.59349.peg.404	fig|6666666.59350.peg.3003	fig|6666666.59351.peg.804	fig|6666666.59353.peg.4137	fig|6666666.59355.peg.3726	fig|6666666.59354.peg.2974

46	3-deoxy-manno-octulosonate cytidylyltransferase EC 2.7.7.38	fig|6666666.59329.peg.861	fig|6666666.59330.peg.1258	fig|6666666.59331.peg.966	fig|6666666.59332.peg.5354	fig|6666666.59334.peg.5500	fig|6666666.59335.peg.2829	fig|6666666.59336.peg.3391	fig|6666666.59333.peg.465	fig|6666666.59338.peg.156	fig|6666666.59339.peg.6331	fig|6666666.59340.peg.2145	fig|6666666.59337.peg.2202	fig|6666666.59342.peg.5793	fig|6666666.71181.peg.4185	fig|6666666.59344.peg.2788	fig|6666666.59345.peg.1587	fig|6666666.59341.peg.1854	fig|6666666.59347.peg.4107	fig|6666666.59346.peg.4226	fig|6666666.59348.peg.1	fig|6666666.59349.peg.2511	fig|6666666.59350.peg.4951	fig|6666666.59351.peg.2027	fig|6666666.59353.peg.1657	fig|6666666.59355.peg.1064	fig|6666666.59354.peg.875

47	ATP-dependent Clp protease adaptor protein ClpS	fig|6666666.59329.peg.5822	fig|6666666.59330.peg.1317	fig|6666666.59331.peg.4267	fig|6666666.59332.peg.4346	fig|6666666.59334.peg.871	fig|6666666.59335.peg.5597	fig|6666666.59336.peg.862	fig|6666666.59333.peg.1009	fig|6666666.59338.peg.5870	fig|6666666.59339.peg.346	fig|6666666.59340.peg.2665	fig|6666666.59337.peg.3373	fig|6666666.59342.peg.4716	fig|6666666.71181.peg.4129	fig|6666666.59344.peg.401	fig|6666666.59345.peg.1555	fig|6666666.59341.peg.994	fig|6666666.59347.peg.4111	fig|6666666.59346.peg.2521	fig|6666666.59348.peg.1385	fig|6666666.59349.peg.2892	fig|6666666.59350.peg.1943	fig|6666666.59351.peg.876	fig|6666666.59353.peg.1621	fig|6666666.59355.peg.1102	fig|6666666.59354.peg.3758

7841	Ribosomal RNA small subunit methyltransferase F EC 2.1.1.-	fig|6666666.59329.peg.6506	fig|6666666.59330.peg.1022	fig|6666666.59331.peg.5854	fig|6666666.59332.peg.4225	fig|6666666.59334.peg.5212	fig|6666666.59335.peg.3360	fig|6666666.59336.peg.4477	fig|6666666.59333.peg.1376	fig|6666666.59338.peg.2718	fig|6666666.59339.peg.8093	fig|6666666.59340.peg.921	fig|6666666.59337.peg.3900	fig|6666666.59342.peg.2354	fig|6666666.71181.peg.3432	fig|6666666.59344.peg.852	fig|6666666.59345.peg.3267	fig|6666666.59341.peg.4163	fig|6666666.59347.peg.4749	fig|6666666.59346.peg.4870	fig|6666666.59348.peg.4365	fig|6666666.59349.peg.2350	fig|6666666.59350.peg.5685	fig|6666666.59351.peg.1200	fig|6666666.59353.peg.4618	fig|6666666.59355.peg.4712	fig|6666666.59354.peg.6170

344	Transcriptional regulator of glmS gene, DeoR family	fig|6666666.59329.peg.614	fig|6666666.59330.peg.4017	fig|6666666.59331.peg.4576	fig|6666666.59332.peg.3652	fig|6666666.59334.peg.2588	fig|6666666.59335.peg.5875	fig|6666666.59336.peg.2236	fig|6666666.59333.peg.2099	fig|6666666.59338.peg.4326	fig|6666666.59339.peg.5731	fig|6666666.59340.peg.2653	fig|6666666.59337.peg.1412	fig|6666666.59342.peg.2578	fig|6666666.71181.peg.4492	fig|6666666.59344.peg.4307	fig|6666666.59345.peg.2279	fig|6666666.59341.peg.3647	fig|6666666.59347.peg.236	fig|6666666.59346.peg.4698	fig|6666666.59348.peg.2850	fig|6666666.59349.peg.1474	fig|6666666.59350.peg.2015	fig|6666666.59351.peg.2462	fig|6666666.59353.peg.3980	fig|6666666.59355.peg.3813	fig|6666666.59354.peg.5081

348	LSU ribosomal protein L16p L10e	fig|6666666.59329.peg.716	fig|6666666.59330.peg.557	fig|6666666.59331.peg.45	fig|6666666.59332.peg.2142	fig|6666666.59334.peg.1488	fig|6666666.59335.peg.4350	fig|6666666.59336.peg.149	fig|6666666.59333.peg.369	fig|6666666.59338.peg.1386	fig|6666666.59339.peg.8271	fig|6666666.59340.peg.59	fig|6666666.59337.peg.385	fig|6666666.59342.peg.307	fig|6666666.71181.peg.3453	fig|6666666.59344.peg.4936	fig|6666666.59345.peg.2532	fig|6666666.59341.peg.2501	fig|6666666.59347.peg.4826	fig|6666666.59346.peg.853	fig|6666666.59348.peg.1763	fig|6666666.59349.peg.1101	fig|6666666.59350.peg.3194	fig|6666666.59351.peg.4810	fig|6666666.59353.peg.4597	fig|6666666.59355.peg.4734	fig|6666666.59354.peg.1744

8306	Transcriptional regulator, AraC family	fig|6666666.59329.peg.3906	fig|6666666.59330.peg.5032	fig|6666666.59331.peg.5119	fig|6666666.59332.peg.5086	fig|6666666.59334.peg.4917	fig|6666666.59335.peg.5987	fig|6666666.59336.peg.2013	fig|6666666.59333.peg.1231	fig|6666666.59338.peg.3501	fig|6666666.59339.peg.7562	fig|6666666.59340.peg.2157	fig|6666666.59337.peg.5021	fig|6666666.59342.peg.6308	fig|6666666.71181.peg.3110	fig|6666666.59344.peg.3295	fig|6666666.59345.peg.615	fig|6666666.59341.peg.3090	fig|6666666.59347.peg.1460	fig|6666666.59346.peg.1211	fig|6666666.59348.peg.1972	fig|6666666.59349.peg.4959	fig|6666666.59350.peg.595	fig|6666666.59351.peg.1527	fig|6666666.59353.peg.3302	fig|6666666.59355.peg.4778	fig|6666666.59354.peg.909

10968	High-affinity choline uptake protein BetT	fig|6666666.59329.peg.3465	fig|6666666.59330.peg.3357	fig|6666666.59331.peg.3933	fig|6666666.59332.peg.3704	fig|6666666.59334.peg.1563	fig|6666666.59335.peg.1044	fig|6666666.59336.peg.6168	fig|6666666.59333.peg.3633	fig|6666666.59338.peg.2303	fig|6666666.59339.peg.4541	fig|6666666.59340.peg.1604	fig|6666666.59337.peg.3578	fig|6666666.59342.peg.6245	fig|6666666.71181.peg.2567	fig|6666666.59344.peg.3721	fig|6666666.59345.peg.3937	fig|6666666.59341.peg.4199	fig|6666666.59347.peg.2439	fig|6666666.59346.peg.3940	fig|6666666.59348.peg.2603	fig|6666666.59349.peg.3834	fig|6666666.59350.peg.388	fig|6666666.59351.peg.3690	fig|6666666.59353.peg.1349	fig|6666666.59355.peg.2523	fig|6666666.59354.peg.2108

4837	NAD P H oxidoreductase YRKL EC 1.6.99.- Putative NADPH-quinone reductase modulator of drug activity B Flavodoxin 2	fig|6666666.59329.peg.3464	fig|6666666.59330.peg.1459	fig|6666666.59331.peg.363	fig|6666666.59332.peg.3890	fig|6666666.59334.peg.3809	fig|6666666.59335.peg.4217	fig|6666666.59336.peg.5974	fig|6666666.59333.peg.5092	fig|6666666.59338.peg.1173	fig|6666666.59339.peg.3580	fig|6666666.59340.peg.3298	fig|6666666.59337.peg.1938	fig|6666666.59342.peg.6244	fig|6666666.71181.peg.1322	fig|6666666.59344.peg.2928	fig|6666666.59345.peg.2043	fig|6666666.59341.peg.3897	fig|6666666.59347.peg.4966	fig|6666666.59346.peg.4617	fig|6666666.59348.peg.4545	fig|6666666.59349.peg.3821	fig|6666666.59350.peg.2733	fig|6666666.59351.peg.1119	fig|6666666.59353.peg.4928	fig|6666666.59355.peg.4971	fig|6666666.59354.peg.4668

9439	Cobalt-zinc-cadmium resistance protein	fig|6666666.59329.peg.2014	fig|6666666.59330.peg.3235	fig|6666666.59331.peg.5738	fig|6666666.59332.peg.1313	fig|6666666.59334.peg.1683	fig|6666666.59335.peg.3547	fig|6666666.59336.peg.2321	fig|6666666.59333.peg.419	fig|6666666.59338.peg.2363	fig|6666666.59339.peg.8696	fig|6666666.59340.peg.1895	fig|6666666.59337.peg.4337	fig|6666666.59342.peg.5703	fig|6666666.71181.peg.5912	fig|6666666.59344.peg.981	fig|6666666.59345.peg.5120	fig|6666666.59341.peg.198	fig|6666666.59347.peg.2737	fig|6666666.59346.peg.3065	fig|6666666.59348.peg.1646	fig|6666666.59349.peg.2586	fig|6666666.59350.peg.1622	fig|6666666.59351.peg.266	fig|6666666.59353.peg.2106	fig|6666666.59355.peg.2692	fig|6666666.59354.peg.5109

9611	tRNA uridine 5-carboxymethylaminomethyl modification enzyme GidA	fig|6666666.59329.peg.1742	fig|6666666.59330.peg.1113	fig|6666666.59331.peg.3961	fig|6666666.59332.peg.93	fig|6666666.59334.peg.3779	fig|6666666.59335.peg.787	fig|6666666.59336.peg.22	fig|6666666.59333.peg.34	fig|6666666.59338.peg.488	fig|6666666.59339.peg.8023	fig|6666666.59340.peg.135	fig|6666666.59337.peg.885	fig|6666666.59342.peg.1191	fig|6666666.71181.peg.589	fig|6666666.59344.peg.4261	fig|6666666.59345.peg.4970	fig|6666666.59341.peg.1054	fig|6666666.59347.peg.4788	fig|6666666.59346.peg.1181	fig|6666666.59348.peg.305	fig|6666666.59349.peg.1024	fig|6666666.59350.peg.1506	fig|6666666.59351.peg.5364	fig|6666666.59353.peg.3091	fig|6666666.59355.peg.5151	fig|6666666.59354.peg.1143

9763	Acyl-[acyl-carrier-protein]--UDP-N-acetylglucosamine O-acyltransferase EC 2.3.1.129	fig|6666666.59329.peg.4483	fig|6666666.59330.peg.456	fig|6666666.59331.peg.4572	fig|6666666.59332.peg.415	fig|6666666.59334.peg.1793	fig|6666666.59335.peg.2664	fig|6666666.59336.peg.2999	fig|6666666.59333.peg.4461	fig|6666666.59338.peg.2457	fig|6666666.59339.peg.5125	fig|6666666.59340.peg.738	fig|6666666.59337.peg.1594	fig|6666666.59342.peg.280	fig|6666666.71181.peg.23	fig|6666666.59344.peg.2666	fig|6666666.59345.peg.2189	fig|6666666.59341.peg.980	fig|6666666.59347.peg.4370	fig|6666666.59346.peg.4922	fig|6666666.59348.peg.3427	fig|6666666.59349.peg.1363	fig|6666666.59350.peg.1091	fig|6666666.59351.peg.2127	fig|6666666.59353.peg.1997	fig|6666666.59355.peg.4102	fig|6666666.59354.peg.1408

4382	Glutamate synthase [NADPH] large chain EC 1.4.1.13	fig|6666666.59329.peg.1058	fig|6666666.59330.peg.2170	fig|6666666.59331.peg.2751	fig|6666666.59332.peg.187	fig|6666666.59334.peg.648	fig|6666666.59335.peg.2788	fig|6666666.59336.peg.2667	fig|6666666.59333.peg.4252	fig|6666666.59338.peg.3177	fig|6666666.59339.peg.4902	fig|6666666.59340.peg.3383	fig|6666666.59337.peg.4403	fig|6666666.59342.peg.5271	fig|6666666.71181.peg.1047	fig|6666666.59344.peg.1831	fig|6666666.59345.peg.3765	fig|6666666.59341.peg.3586	fig|6666666.59347.peg.4021	fig|6666666.59346.peg.1360	fig|6666666.59348.peg.3623	fig|6666666.59349.peg.2391	fig|6666666.59350.peg.4552	fig|6666666.59351.peg.639	fig|6666666.59353.peg.2366	fig|6666666.59355.peg.156	fig|6666666.59354.peg.5653

4030	Alcohol dehydrogenase EC 1.1.1.1	fig|6666666.59329.peg.4117	fig|6666666.59330.peg.923	fig|6666666.59331.peg.1471	fig|6666666.59332.peg.2480	fig|6666666.59334.peg.4307	fig|6666666.59335.peg.2765	fig|6666666.59336.peg.2680	fig|6666666.59333.peg.960	fig|6666666.59338.peg.4981	fig|6666666.59339.peg.3514	fig|6666666.59340.peg.2897	fig|6666666.59337.peg.4636	fig|6666666.59342.peg.3265	fig|6666666.71181.peg.3640	fig|6666666.59344.peg.5256	fig|6666666.59345.peg.436	fig|6666666.59341.peg.1884	fig|6666666.59347.peg.2567	fig|6666666.59346.peg.5193	fig|6666666.59348.peg.3992	fig|6666666.59349.peg.5025	fig|6666666.59350.peg.7376	fig|6666666.59351.peg.3368	fig|6666666.59353.peg.2504	fig|6666666.59355.peg.1266	fig|6666666.59354.peg.5518

8173	3-isopropylmalate dehydratase large subunit EC 4.2.1.33	fig|6666666.59329.peg.1835	fig|6666666.59330.peg.1765	fig|6666666.59331.peg.3525	fig|6666666.59332.peg.249	fig|6666666.59334.peg.894	fig|6666666.59335.peg.1523	fig|6666666.59336.peg.3671	fig|6666666.59333.peg.124	fig|6666666.59338.peg.56	fig|6666666.59339.peg.7321	fig|6666666.59340.peg.2301	fig|6666666.59337.peg.1391	fig|6666666.59342.peg.5864	fig|6666666.71181.peg.380	fig|6666666.59344.peg.1501	fig|6666666.59345.peg.4096	fig|6666666.59341.peg.4409	fig|6666666.59347.peg.1620	fig|6666666.59346.peg.749	fig|6666666.59348.peg.3960	fig|6666666.59349.peg.319	fig|6666666.59350.peg.5649	fig|6666666.59351.peg.1222	fig|6666666.59353.peg.3209	fig|6666666.59355.peg.1215	fig|6666666.59354.peg.838

9339	Transporter, putative	fig|6666666.59329.peg.38	fig|6666666.59330.peg.135	fig|6666666.59331.peg.2842	fig|6666666.59332.peg.753	fig|6666666.59334.peg.462	fig|6666666.59335.peg.743	fig|6666666.59336.peg.2819	fig|6666666.59333.peg.4663	fig|6666666.59338.peg.1721	fig|6666666.59339.peg.8814	fig|6666666.59340.peg.2415	fig|6666666.59337.peg.353	fig|6666666.59342.peg.2516	fig|6666666.71181.peg.1730	fig|6666666.59344.peg.1920	fig|6666666.59345.peg.761	fig|6666666.59341.peg.787	fig|6666666.59347.peg.4561	fig|6666666.59346.peg.5299	fig|6666666.59348.peg.4537	fig|6666666.59349.peg.4801	fig|6666666.59350.peg.3542	fig|6666666.59351.peg.5231	fig|6666666.59353.peg.117	fig|6666666.59355.peg.298	fig|6666666.59354.peg.2135

1613	Tyrosine recombinase XerC	fig|6666666.59329.peg.212	fig|6666666.59330.peg.3055	fig|6666666.59331.peg.2553	fig|6666666.59332.peg.4407	fig|6666666.59334.peg.2814	fig|6666666.59335.peg.3816	fig|6666666.59336.peg.3932	fig|6666666.59333.peg.3936	fig|6666666.59338.peg.4908	fig|6666666.59339.peg.7958	fig|6666666.59340.peg.3065	fig|6666666.59337.peg.1620	fig|6666666.59342.peg.3778	fig|6666666.71181.peg.5142	fig|6666666.59344.peg.2489	fig|6666666.59345.peg.5499	fig|6666666.59341.peg.3123	fig|6666666.59347.peg.5073	fig|6666666.59346.peg.3098	fig|6666666.59348.peg.668	fig|6666666.59349.peg.4391	fig|6666666.59350.peg.7958	fig|6666666.59351.peg.4748	fig|6666666.59353.peg.3838	fig|6666666.59355.peg.4995	fig|6666666.59354.peg.4888

2871	Glutamyl-tRNA synthetase EC 6.1.1.17	fig|6666666.59329.peg.4551	fig|6666666.59330.peg.1757	fig|6666666.59331.peg.2303	fig|6666666.59332.peg.3225	fig|6666666.59334.peg.221	fig|6666666.59335.peg.5210	fig|6666666.59336.peg.2866	fig|6666666.59333.peg.863	fig|6666666.59338.peg.3889	fig|6666666.59339.peg.4771	fig|6666666.59340.peg.1054	fig|6666666.59337.peg.624	fig|6666666.59342.peg.1570	fig|6666666.71181.peg.3197	fig|6666666.59344.peg.4441	fig|6666666.59345.peg.5437	fig|6666666.59341.peg.1552	fig|6666666.59347.peg.2945	fig|6666666.59346.peg.2108	fig|6666666.59348.peg.1700	fig|6666666.59349.peg.824	fig|6666666.59350.peg.4293	fig|6666666.59351.peg.3735	fig|6666666.59353.peg.2894	fig|6666666.59355.peg.2777	fig|6666666.59354.peg.48

5447	Aspartate carbamoyltransferase EC 2.1.3.2	fig|6666666.59329.peg.1445	fig|6666666.59330.peg.62	fig|6666666.59331.peg.4640	fig|6666666.59332.peg.1284	fig|6666666.59334.peg.3767	fig|6666666.59335.peg.464	fig|6666666.59336.peg.3088	fig|6666666.59333.peg.5024	fig|6666666.59338.peg.2821	fig|6666666.59339.peg.6474	fig|6666666.59340.peg.3412	fig|6666666.59337.peg.849	fig|6666666.59342.peg.429	fig|6666666.71181.peg.3294	fig|6666666.59344.peg.4649	fig|6666666.59345.peg.4843	fig|6666666.59341.peg.2699	fig|6666666.59347.peg.2613	fig|6666666.59346.peg.621	fig|6666666.59348.peg.2511	fig|6666666.59349.peg.1753	fig|6666666.59350.peg.1244	fig|6666666.59351.peg.3943	fig|6666666.59353.peg.2431	fig|6666666.59355.peg.524	fig|6666666.59354.peg.482

7685	UDP-3-O-[3-hydroxymyristoyl] glucosamine N-acyltransferase EC 2.3.1.-	fig|6666666.59329.peg.1584	fig|6666666.59330.peg.458	fig|6666666.59331.peg.2207	fig|6666666.59332.peg.413	fig|6666666.59334.peg.1795	fig|6666666.59335.peg.2662	fig|6666666.59336.peg.3001	fig|6666666.59333.peg.4464	fig|6666666.59338.peg.2455	fig|6666666.59339.peg.5122	fig|6666666.59340.peg.1316	fig|6666666.59337.peg.1592	fig|6666666.59342.peg.278	fig|6666666.71181.peg.25	fig|6666666.59344.peg.2664	fig|6666666.59345.peg.2191	fig|6666666.59341.peg.978	fig|6666666.59347.peg.4368	fig|6666666.59346.peg.4920	fig|6666666.59348.peg.4079	fig|6666666.59349.peg.1365	fig|6666666.59350.peg.582	fig|6666666.59351.peg.2125	fig|6666666.59353.peg.1995	fig|6666666.59355.peg.4100	fig|6666666.59354.peg.1344

8442	Permease of the drug metabolite transporter DMT superfamily	fig|6666666.59329.peg.3223	fig|6666666.59330.peg.3553	fig|6666666.59331.peg.4143	fig|6666666.59332.peg.3589	fig|6666666.59334.peg.2505	fig|6666666.59335.peg.1048	fig|6666666.59336.peg.4006	fig|6666666.59333.peg.3881	fig|6666666.59338.peg.2083	fig|6666666.59339.peg.4612	fig|6666666.59340.peg.3757	fig|6666666.59337.peg.1241	fig|6666666.59342.peg.3717	fig|6666666.71181.peg.5465	fig|6666666.59344.peg.2116	fig|6666666.59345.peg.4427	fig|6666666.59341.peg.4159	fig|6666666.59347.peg.901	fig|6666666.59346.peg.3353	fig|6666666.59348.peg.5153	fig|6666666.59349.peg.3857	fig|6666666.59350.peg.941	fig|6666666.59351.peg.1718	fig|6666666.59353.peg.5048	fig|6666666.59355.peg.4685	fig|6666666.59354.peg.3991

7539	Endoribonuclease L-PSP	fig|6666666.59329.peg.3708	fig|6666666.59330.peg.60	fig|6666666.59331.peg.5720	fig|6666666.59332.peg.4627	fig|6666666.59334.peg.2771	fig|6666666.59335.peg.2924	fig|6666666.59336.peg.3332	fig|6666666.59333.peg.5026	fig|6666666.59338.peg.122	fig|6666666.59339.peg.8734	fig|6666666.59340.peg.834	fig|6666666.59337.peg.5792	fig|6666666.59342.peg.434	fig|6666666.71181.peg.325	fig|6666666.59344.peg.1264	fig|6666666.59345.peg.1187	fig|6666666.59341.peg.958	fig|6666666.59347.peg.4933	fig|6666666.59346.peg.3815	fig|6666666.59348.peg.2448	fig|6666666.59349.peg.3643	fig|6666666.59350.peg.5053	fig|6666666.59351.peg.2925	fig|6666666.59353.peg.4221	fig|6666666.59355.peg.4184	fig|6666666.59354.peg.3359

6171	Menaquinone-specific isochorismate synthase EC 5.4.4.2	fig|6666666.59329.peg.5630	fig|6666666.59330.peg.309	fig|6666666.59331.peg.767	fig|6666666.59332.peg.4061	fig|6666666.59334.peg.2519	fig|6666666.59335.peg.859	fig|6666666.59336.peg.6475	fig|6666666.59333.peg.1759	fig|6666666.59338.peg.6150	fig|6666666.59339.peg.3227	fig|6666666.59340.peg.3058	fig|6666666.59337.peg.1037	fig|6666666.59342.peg.4538	fig|6666666.71181.peg.1733	fig|6666666.59344.peg.2558	fig|6666666.59345.peg.4246	fig|6666666.59341.peg.5764	fig|6666666.59347.peg.270	fig|6666666.59346.peg.3845	fig|6666666.59348.peg.3152	fig|6666666.59349.peg.3730	fig|6666666.59350.peg.2773	fig|6666666.59351.peg.788	fig|6666666.59353.peg.937	fig|6666666.59355.peg.2442	fig|6666666.59354.peg.1468

7332	GTP-binding protein HflX	fig|6666666.59329.peg.693	fig|6666666.59330.peg.3561	fig|6666666.59331.peg.1283	fig|6666666.59332.peg.4222	fig|6666666.59334.peg.3061	fig|6666666.59335.peg.4132	fig|6666666.59336.peg.3762	fig|6666666.59333.peg.288	fig|6666666.59338.peg.1002	fig|6666666.59339.peg.8861	fig|6666666.59340.peg.463	fig|6666666.59337.peg.1783	fig|6666666.59342.peg.2597	fig|6666666.71181.peg.4633	fig|6666666.59344.peg.5885	fig|6666666.59345.peg.5663	fig|6666666.59341.peg.4273	fig|6666666.59347.peg.5346	fig|6666666.59346.peg.3039	fig|6666666.59348.peg.2816	fig|6666666.59349.peg.3172	fig|6666666.59350.peg.7857	fig|6666666.59351.peg.3095	fig|6666666.59353.peg.4989	fig|6666666.59355.peg.5073	fig|6666666.59354.peg.6039

7054	Membrane-bound lytic murein transglycosylase D precursor EC 3.2.1.-	fig|6666666.59329.peg.740	fig|6666666.59330.peg.445	fig|6666666.59331.peg.3077	fig|6666666.59332.peg.5933	fig|6666666.59334.peg.5578	fig|6666666.59335.peg.4573	fig|6666666.59336.peg.5567	fig|6666666.59333.peg.1158	fig|6666666.59338.peg.1726	fig|6666666.59339.peg.8034	fig|6666666.59340.peg.697	fig|6666666.59337.peg.151	fig|6666666.59342.peg.2987	fig|6666666.71181.peg.2114	fig|6666666.59344.peg.5204	fig|6666666.59345.peg.4115	fig|6666666.59341.peg.4692	fig|6666666.59347.peg.2066	fig|6666666.59346.peg.269	fig|6666666.59348.peg.4138	fig|6666666.59349.peg.2800	fig|6666666.59350.peg.3745	fig|6666666.59351.peg.5042	fig|6666666.59353.peg.2988	fig|6666666.59355.peg.1814	fig|6666666.59354.peg.3187

6993	Enoyl-CoA hydratase EC 4.2.1.17 Delta 3 -cis-delta 2 -trans-enoyl-CoA isomerase EC 5.3.3.8 3-hydroxyacyl-CoA dehydrogenase EC 1.1.1.35 3-hydroxybutyryl-CoA epimerase EC 5.1.2.3	fig|6666666.59329.peg.5358	fig|6666666.59330.peg.1085	fig|6666666.59331.peg.763	fig|6666666.59332.peg.67	fig|6666666.59334.peg.2597	fig|6666666.59335.peg.1262	fig|6666666.59336.peg.2816	fig|6666666.59333.peg.5238	fig|6666666.59338.peg.3205	fig|6666666.59339.peg.7991	fig|6666666.59340.peg.2555	fig|6666666.59337.peg.1468	fig|6666666.59342.peg.5403	fig|6666666.71181.peg.3328	fig|6666666.59344.peg.4997	fig|6666666.59345.peg.223	fig|6666666.59341.peg.848	fig|6666666.59347.peg.5106	fig|6666666.59346.peg.2168	fig|6666666.59348.peg.460	fig|6666666.59349.peg.4071	fig|6666666.59350.peg.992	fig|6666666.59351.peg.3704	fig|6666666.59353.peg.2479	fig|6666666.59355.peg.574	fig|6666666.59354.peg.1394

11034	LSU ribosomal protein L7 L12 P1 P2	fig|6666666.59329.peg.626	fig|6666666.59330.peg.3408	fig|6666666.59331.peg.440	fig|6666666.59332.peg.2889	fig|6666666.59334.peg.2477	fig|6666666.59335.peg.5629	fig|6666666.59336.peg.1683	fig|6666666.59333.peg.4393	fig|6666666.59338.peg.3292	fig|6666666.59339.peg.8842	fig|6666666.59340.peg.1329	fig|6666666.59337.peg.4989	fig|6666666.59342.peg.367	fig|6666666.71181.peg.5015	fig|6666666.59344.peg.5462	fig|6666666.59345.peg.5654	fig|6666666.59341.peg.5594	fig|6666666.59347.peg.5398	fig|6666666.59346.peg.4099	fig|6666666.59348.peg.3991	fig|6666666.59349.peg.1142	fig|6666666.59350.peg.5399	fig|6666666.59351.peg.5242	fig|6666666.59353.peg.4452	fig|6666666.59355.peg.4575	fig|6666666.59354.peg.4534

7837	Transcriptional regulator, LysR family	fig|6666666.59329.peg.633	fig|6666666.59330.peg.4237	fig|6666666.59331.peg.2685	fig|6666666.59332.peg.5782	fig|6666666.59334.peg.3310	fig|6666666.59335.peg.2757	fig|6666666.59336.peg.2714	fig|6666666.59333.peg.4101	fig|6666666.59338.peg.5481	fig|6666666.59339.peg.1783	fig|6666666.59340.peg.1375	fig|6666666.59337.peg.3596	fig|6666666.59342.peg.5772	fig|6666666.71181.peg.2209	fig|6666666.59344.peg.5616	fig|6666666.59345.peg.4172	fig|6666666.59341.peg.5227	fig|6666666.59347.peg.465	fig|6666666.59346.peg.927	fig|6666666.59348.peg.4212	fig|6666666.59349.peg.3128	fig|6666666.59350.peg.2121	fig|6666666.59351.peg.4511	fig|6666666.59353.peg.3486	fig|6666666.59355.peg.3531	fig|6666666.59354.peg.5921

1805	DNA-directed RNA polymerase omega subunit EC 2.7.7.6	fig|6666666.59329.peg.1268	fig|6666666.59330.peg.1194	fig|6666666.59331.peg.2949	fig|6666666.59332.peg.725	fig|6666666.59334.peg.237	fig|6666666.59335.peg.4066	fig|6666666.59336.peg.834	fig|6666666.59333.peg.4057	fig|6666666.59338.peg.2938	fig|6666666.59339.peg.6250	fig|6666666.59340.peg.157	fig|6666666.59337.peg.502	fig|6666666.59342.peg.581	fig|6666666.71181.peg.2605	fig|6666666.59344.peg.924	fig|6666666.59345.peg.2649	fig|6666666.59341.peg.4959	fig|6666666.59347.peg.5199	fig|6666666.59346.peg.1384	fig|6666666.59348.peg.1929	fig|6666666.59349.peg.3645	fig|6666666.59350.peg.6798	fig|6666666.59351.peg.317	fig|6666666.59353.peg.2606	fig|6666666.59355.peg.5320	fig|6666666.59354.peg.3357

3787	Ribonuclease III EC 3.1.26.3	fig|6666666.59329.peg.503	fig|6666666.59330.peg.385	fig|6666666.59331.peg.5761	fig|6666666.59332.peg.728	fig|6666666.59334.peg.1878	fig|6666666.59335.peg.1490	fig|6666666.59336.peg.2100	fig|6666666.59333.peg.2809	fig|6666666.59338.peg.1515	fig|6666666.59339.peg.7370	fig|6666666.59340.peg.851	fig|6666666.59337.peg.565	fig|6666666.59342.peg.4454	fig|6666666.71181.peg.1423	fig|6666666.59344.peg.1566	fig|6666666.59345.peg.5055	fig|6666666.59341.peg.1334	fig|6666666.59347.peg.3176	fig|6666666.59346.peg.334	fig|6666666.59348.peg.649	fig|6666666.59349.peg.4318	fig|6666666.59350.peg.1614	fig|6666666.59351.peg.600	fig|6666666.59353.peg.2653	fig|6666666.59355.peg.1829	fig|6666666.59354.peg.2817

4994	Type II IV secretion system ATP hydrolase TadA VirB11 CpaF, TadA subfamily	fig|6666666.59329.peg.1769	fig|6666666.59330.peg.344	fig|6666666.59331.peg.2293	fig|6666666.59332.peg.1627	fig|6666666.59334.peg.1504	fig|6666666.59335.peg.718	fig|6666666.59336.peg.450	fig|6666666.59333.peg.770	fig|6666666.59338.peg.3701	fig|6666666.59339.peg.6199	fig|6666666.59340.peg.1758	fig|6666666.59337.peg.3075	fig|6666666.59342.peg.2684	fig|6666666.71181.peg.1964	fig|6666666.59344.peg.1459	fig|6666666.59345.peg.2676	fig|6666666.59341.peg.4293	fig|6666666.59347.peg.3893	fig|6666666.59346.peg.4812	fig|6666666.59348.peg.5108	fig|6666666.59349.peg.2626	fig|6666666.59350.peg.4340	fig|6666666.59351.peg.4346	fig|6666666.59353.peg.155	fig|6666666.59355.peg.765	fig|6666666.59354.peg.5980

1490	Phenylalanyl-tRNA synthetase alpha chain EC 6.1.1.20	fig|6666666.59329.peg.1470	fig|6666666.59330.peg.533	fig|6666666.59331.peg.1690	fig|6666666.59332.peg.5772	fig|6666666.59334.peg.2153	fig|6666666.59335.peg.3606	fig|6666666.59336.peg.5754	fig|6666666.59333.peg.533	fig|6666666.59338.peg.4155	fig|6666666.59339.peg.596	fig|6666666.59340.peg.3337	fig|6666666.59337.peg.3979	fig|6666666.59342.peg.4733	fig|6666666.71181.peg.2416	fig|6666666.59344.peg.804	fig|6666666.59345.peg.4651	fig|6666666.59341.peg.450	fig|6666666.59347.peg.1878	fig|6666666.59346.peg.3122	fig|6666666.59348.peg.3243	fig|6666666.59349.peg.3585	fig|6666666.59350.peg.976	fig|6666666.59351.peg.4429	fig|6666666.59353.peg.1305	fig|6666666.59355.peg.3276	fig|6666666.59354.peg.4281

525	DNA polymerase III epsilon subunit EC 2.7.7.7	fig|6666666.59329.peg.4317	fig|6666666.59330.peg.4659	fig|6666666.59331.peg.4058	fig|6666666.59332.peg.433	fig|6666666.59334.peg.3924	fig|6666666.59335.peg.5517	fig|6666666.59336.peg.1419	fig|6666666.59333.peg.1163	fig|6666666.59338.peg.3632	fig|6666666.59339.peg.5150	fig|6666666.59340.peg.635	fig|6666666.59337.peg.4476	fig|6666666.59342.peg.4295	fig|6666666.71181.peg.879	fig|6666666.59344.peg.4754	fig|6666666.59345.peg.2174	fig|6666666.59341.peg.2352	fig|6666666.59347.peg.2867	fig|6666666.59346.peg.3694	fig|6666666.59348.peg.2910	fig|6666666.59349.peg.4586	fig|6666666.59350.peg.5417	fig|6666666.59351.peg.5496	fig|6666666.59353.peg.4554	fig|6666666.59355.peg.4643	fig|6666666.59354.peg.1438

6503	Zn-dependent protease with chaperone function PA4632	fig|6666666.59329.peg.6128	fig|6666666.59330.peg.3635	fig|6666666.59331.peg.2128	fig|6666666.59332.peg.6265	fig|6666666.59334.peg.378	fig|6666666.59335.peg.1192	fig|6666666.59336.peg.1610	fig|6666666.59333.peg.4508	fig|6666666.59338.peg.3698	fig|6666666.59339.peg.2908	fig|6666666.59340.peg.1648	fig|6666666.59337.peg.3142	fig|6666666.59342.peg.1683	fig|6666666.71181.peg.5350	fig|6666666.59344.peg.4021	fig|6666666.59345.peg.1150	fig|6666666.59341.peg.3245	fig|6666666.59347.peg.1908	fig|6666666.59346.peg.2500	fig|6666666.59348.peg.5086	fig|6666666.59349.peg.3871	fig|6666666.59350.peg.3691	fig|6666666.59351.peg.4922	fig|6666666.59353.peg.5072	fig|6666666.59355.peg.3987	fig|6666666.59354.peg.5534

6501	ATP-dependent helicase HrpB	fig|6666666.59329.peg.1972	fig|6666666.59330.peg.1952	fig|6666666.59331.peg.5128	fig|6666666.59332.peg.5716	fig|6666666.59334.peg.1299	fig|6666666.59335.peg.1519	fig|6666666.59336.peg.569	fig|6666666.59333.peg.3636	fig|6666666.59338.peg.1972	fig|6666666.59339.peg.3683	fig|6666666.59340.peg.141	fig|6666666.59337.peg.628	fig|6666666.59342.peg.5059	fig|6666666.71181.peg.2835	fig|6666666.59344.peg.567	fig|6666666.59345.peg.2401	fig|6666666.59341.peg.5222	fig|6666666.59347.peg.1691	fig|6666666.59346.peg.3743	fig|6666666.59348.peg.5531	fig|6666666.59349.peg.3665	fig|6666666.59350.peg.6637	fig|6666666.59351.peg.481	fig|6666666.59353.peg.1872	fig|6666666.59355.peg.1142	fig|6666666.59354.peg.6021

4501	Heat shock protein GrpE	fig|6666666.59329.peg.1682	fig|6666666.59330.peg.4620	fig|6666666.59331.peg.3078	fig|6666666.59332.peg.385	fig|6666666.59334.peg.1384	fig|6666666.59335.peg.5165	fig|6666666.59336.peg.223	fig|6666666.59333.peg.2755	fig|6666666.59338.peg.1080	fig|6666666.59339.peg.7274	fig|6666666.59340.peg.1194	fig|6666666.59337.peg.4971	fig|6666666.59342.peg.856	fig|6666666.71181.peg.1439	fig|6666666.59344.peg.1547	fig|6666666.59345.peg.3573	fig|6666666.59341.peg.4894	fig|6666666.59347.peg.4329	fig|6666666.59346.peg.690	fig|6666666.59348.peg.4818	fig|6666666.59349.peg.2383	fig|6666666.59350.peg.1497	fig|6666666.59351.peg.646	fig|6666666.59353.peg.4353	fig|6666666.59355.peg.4490	fig|6666666.59354.peg.4834

2088	Phosphoenolpyruvate carboxykinase [ATP] EC 4.1.1.49	fig|6666666.59329.peg.346	fig|6666666.59330.peg.413	fig|6666666.59331.peg.960	fig|6666666.59332.peg.2105	fig|6666666.59334.peg.135	fig|6666666.59335.peg.725	fig|6666666.59336.peg.445	fig|6666666.59333.peg.499	fig|6666666.59338.peg.1800	fig|6666666.59339.peg.6189	fig|6666666.59340.peg.175	fig|6666666.59337.peg.4238	fig|6666666.59342.peg.2910	fig|6666666.71181.peg.3863	fig|6666666.59344.peg.860	fig|6666666.59345.peg.2681	fig|6666666.59341.peg.3432	fig|6666666.59347.peg.4459	fig|6666666.59346.peg.2600	fig|6666666.59348.peg.2080	fig|6666666.59349.peg.2180	fig|6666666.59350.peg.1623	fig|6666666.59351.peg.4354	fig|6666666.59353.peg.1257	fig|6666666.59355.peg.1896	fig|6666666.59354.peg.5144

10238	Ribosomal large subunit pseudouridine synthase D EC 4.2.1.70	fig|6666666.59329.peg.6511	fig|6666666.59330.peg.473	fig|6666666.59331.peg.2851	fig|6666666.59332.peg.2228	fig|6666666.59334.peg.1645	fig|6666666.59335.peg.244	fig|6666666.59336.peg.5973	fig|6666666.59333.peg.1599	fig|6666666.59338.peg.3398	fig|6666666.59339.peg.7517	fig|6666666.59340.peg.1187	fig|6666666.59337.peg.1576	fig|6666666.59342.peg.3353	fig|6666666.71181.peg.395	fig|6666666.59344.peg.2425	fig|6666666.59345.peg.1071	fig|6666666.59341.peg.896	fig|6666666.59347.peg.4057	fig|6666666.59346.peg.5004	fig|6666666.59348.peg.5197	fig|6666666.59349.peg.2512	fig|6666666.59350.peg.6328	fig|6666666.59351.peg.2631	fig|6666666.59353.peg.4801	fig|6666666.59355.peg.713	fig|6666666.59354.peg.2624

7783	Serine protein kinase prkA protein , P-loop containing	fig|6666666.59329.peg.3650	fig|6666666.59330.peg.1261	fig|6666666.59331.peg.2799	fig|6666666.59332.peg.6085	fig|6666666.59334.peg.1893	fig|6666666.59335.peg.2824	fig|6666666.59336.peg.3387	fig|6666666.59333.peg.1840	fig|6666666.59338.peg.5646	fig|6666666.59339.peg.3482	fig|6666666.59340.peg.1088	fig|6666666.59337.peg.2205	fig|6666666.59342.peg.4442	fig|6666666.71181.peg.2967	fig|6666666.59344.peg.2888	fig|6666666.59345.peg.1584	fig|6666666.59341.peg.3941	fig|6666666.59347.peg.4169	fig|6666666.59346.peg.3246	fig|6666666.59348.peg.1977	fig|6666666.59349.peg.2395	fig|6666666.59350.peg.7045	fig|6666666.59351.peg.635	fig|6666666.59353.peg.1437	fig|6666666.59355.peg.1986	fig|6666666.59354.peg.5163

11011	Glucose-1-phosphate adenylyltransferase EC 2.7.7.27	fig|6666666.59329.peg.3638	fig|6666666.59330.peg.49	fig|6666666.59331.peg.3600	fig|6666666.59332.peg.2535	fig|6666666.59334.peg.95	fig|6666666.59335.peg.925	fig|6666666.59336.peg.5804	fig|6666666.59333.peg.639	fig|6666666.59338.peg.1519	fig|6666666.59339.peg.297	fig|6666666.59340.peg.3657	fig|6666666.59337.peg.459	fig|6666666.59342.peg.50	fig|6666666.71181.peg.213	fig|6666666.59344.peg.270	fig|6666666.59345.peg.1545	fig|6666666.59341.peg.2737	fig|6666666.59347.peg.1290	fig|6666666.59346.peg.1596	fig|6666666.59348.peg.2005	fig|6666666.59349.peg.25	fig|6666666.59350.peg.1640	fig|6666666.59351.peg.2006	fig|6666666.59353.peg.2174	fig|6666666.59355.peg.1737	fig|6666666.59354.peg.5891

11338	tRNA delta 2 -isopentenylpyrophosphate transferase EC 2.5.1.8	fig|6666666.59329.peg.695	fig|6666666.59330.peg.3559	fig|6666666.59331.peg.1280	fig|6666666.59332.peg.1980	fig|6666666.59334.peg.3063	fig|6666666.59335.peg.4130	fig|6666666.59336.peg.3759	fig|6666666.59333.peg.286	fig|6666666.59338.peg.1000	fig|6666666.59339.peg.8858	fig|6666666.59340.peg.461	fig|6666666.59337.peg.1781	fig|6666666.59342.peg.2600	fig|6666666.71181.peg.4635	fig|6666666.59344.peg.5883	fig|6666666.59345.peg.5665	fig|6666666.59341.peg.3761	fig|6666666.59347.peg.5348	fig|6666666.59346.peg.3041	fig|6666666.59348.peg.2814	fig|6666666.59349.peg.3170	fig|6666666.59350.peg.1874	fig|6666666.59351.peg.3093	fig|6666666.59353.peg.4991	fig|6666666.59355.peg.5972	fig|6666666.59354.peg.6042

9273	2-C-methyl-D-erythritol 2,4-cyclodiphosphate synthase EC 4.6.1.12	fig|6666666.59329.peg.4460	fig|6666666.59330.peg.2462	fig|6666666.59331.peg.4167	fig|6666666.59332.peg.200	fig|6666666.59334.peg.269	fig|6666666.59335.peg.1656	fig|6666666.59336.peg.4984	fig|6666666.59333.peg.4778	fig|6666666.59338.peg.222	fig|6666666.59339.peg.7918	fig|6666666.59340.peg.1527	fig|6666666.59337.peg.579	fig|6666666.59342.peg.1274	fig|6666666.71181.peg.3417	fig|6666666.59344.peg.2196	fig|6666666.59345.peg.5070	fig|6666666.59341.peg.657	fig|6666666.59347.peg.3950	fig|6666666.59346.peg.3147	fig|6666666.59348.peg.4438	fig|6666666.59349.peg.4382	fig|6666666.59350.peg.6649	fig|6666666.59351.peg.5477	fig|6666666.59353.peg.210	fig|6666666.59355.peg.2979	fig|6666666.59354.peg.3141

7816	Multidrug resistance protein D	fig|6666666.59329.peg.2673	fig|6666666.59330.peg.1799	fig|6666666.59331.peg.532	fig|6666666.59332.peg.5473	fig|6666666.59334.peg.913	fig|6666666.59335.peg.1631	fig|6666666.59336.peg.3791	fig|6666666.59333.peg.2977	fig|6666666.59338.peg.373	fig|6666666.59339.peg.3651	fig|6666666.59340.peg.675	fig|6666666.59337.peg.5413	fig|6666666.59342.peg.1698	fig|6666666.71181.peg.486	fig|6666666.59344.peg.5283	fig|6666666.59345.peg.37	fig|6666666.59341.peg.5063	fig|6666666.59347.peg.1995	fig|6666666.59346.peg.3663	fig|6666666.59348.peg.1348	fig|6666666.59349.peg.3872	fig|6666666.59350.peg.3750	fig|6666666.59351.peg.3412	fig|6666666.59353.peg.4630	fig|6666666.59355.peg.4360	fig|6666666.59354.peg.5555

2663	Sulfate permease	fig|6666666.59329.peg.1457	fig|6666666.59330.peg.4045	fig|6666666.59331.peg.3932	fig|6666666.59332.peg.5319	fig|6666666.59334.peg.814	fig|6666666.59335.peg.2333	fig|6666666.59336.peg.4637	fig|6666666.59333.peg.1605	fig|6666666.59338.peg.5016	fig|6666666.59339.peg.3163	fig|6666666.59340.peg.1810	fig|6666666.59337.peg.3855	fig|6666666.59342.peg.5869	fig|6666666.71181.peg.786	fig|6666666.59344.peg.117	fig|6666666.59345.peg.1074	fig|6666666.59341.peg.537	fig|6666666.59347.peg.3904	fig|6666666.59346.peg.5178	fig|6666666.59348.peg.2107	fig|6666666.59349.peg.400	fig|6666666.59350.peg.644	fig|6666666.59351.peg.850	fig|6666666.59353.peg.437	fig|6666666.59355.peg.1243	fig|6666666.59354.peg.2968

1920	Transcription accessory protein S1 RNA-binding domain	fig|6666666.59329.peg.1897	fig|6666666.59330.peg.435	fig|6666666.59331.peg.2228	fig|6666666.59332.peg.2465	fig|6666666.59334.peg.4045	fig|6666666.59335.peg.5920	fig|6666666.59336.peg.4091	fig|6666666.59333.peg.3072	fig|6666666.59338.peg.4004	fig|6666666.59339.peg.6228	fig|6666666.59340.peg.2072	fig|6666666.59337.peg.688	fig|6666666.59342.peg.382	fig|6666666.71181.peg.6017	fig|6666666.59344.peg.2094	fig|6666666.59345.peg.5580	fig|6666666.59341.peg.1419	fig|6666666.59347.peg.1217	fig|6666666.59346.peg.3412	fig|6666666.59348.peg.407	fig|6666666.59349.peg.2189	fig|6666666.59350.peg.3592	fig|6666666.59351.peg.140	fig|6666666.59353.peg.2194	fig|6666666.59355.peg.1195	fig|6666666.59354.peg.5104

3869	Lead, cadmium, zinc and mercury transporting ATPase EC 3.6.3.3 EC 3.6.3.5 ; Copper-translocating P-type ATPase EC 3.6.3.4	fig|6666666.59329.peg.4584	fig|6666666.59330.peg.4062	fig|6666666.59331.peg.101	fig|6666666.59332.peg.4829	fig|6666666.59334.peg.5555	fig|6666666.59335.peg.3534	fig|6666666.59336.peg.2862	fig|6666666.59333.peg.215	fig|6666666.59338.peg.5067	fig|6666666.59339.peg.1322	fig|6666666.59340.peg.1053	fig|6666666.59337.peg.2171	fig|6666666.59342.peg.2024	fig|6666666.71181.peg.3283	fig|6666666.59344.peg.5075	fig|6666666.59345.peg.3231	fig|6666666.59341.peg.4450	fig|6666666.59347.peg.2207	fig|6666666.59346.peg.2185	fig|6666666.59348.peg.3452	fig|6666666.59349.peg.141	fig|6666666.59350.peg.1949	fig|6666666.59351.peg.3135	fig|6666666.59353.peg.4060	fig|6666666.59355.peg.4220	fig|6666666.59354.peg.199

7579	Succinate-semialdehyde dehydrogenase [NADP+] EC 1.2.1.16	fig|6666666.59329.peg.6417	fig|6666666.59330.peg.677	fig|6666666.59331.peg.119	fig|6666666.59332.peg.5801	fig|6666666.59334.peg.1350	fig|6666666.59335.peg.3573	fig|6666666.59336.peg.281	fig|6666666.59333.peg.1301	fig|6666666.59338.peg.949	fig|6666666.59339.peg.2597	fig|6666666.59340.peg.2351	fig|6666666.59337.peg.506	fig|6666666.59342.peg.4294	fig|6666666.71181.peg.4693	fig|6666666.59344.peg.5196	fig|6666666.59345.peg.4136	fig|6666666.59341.peg.3992	fig|6666666.59347.peg.2222	fig|6666666.59346.peg.1111	fig|6666666.59348.peg.1599	fig|6666666.59349.peg.1481	fig|6666666.59350.peg.6001	fig|6666666.59351.peg.2707	fig|6666666.59353.peg.4399	fig|6666666.59355.peg.3165	fig|6666666.59354.peg.5949

5901	Flavin reductase EC 1.5.1.30	fig|6666666.59329.peg.2475	fig|6666666.59330.peg.1345	fig|6666666.59331.peg.4339	fig|6666666.59332.peg.3909	fig|6666666.59334.peg.4229	fig|6666666.59335.peg.5736	fig|6666666.59336.peg.5686	fig|6666666.59333.peg.4630	fig|6666666.59338.peg.2706	fig|6666666.59339.peg.909	fig|6666666.59340.peg.2917	fig|6666666.59337.peg.2609	fig|6666666.59342.peg.253	fig|6666666.71181.peg.3913	fig|6666666.59344.peg.3739	fig|6666666.59345.peg.4221	fig|6666666.59341.peg.1652	fig|6666666.59347.peg.799	fig|6666666.59346.peg.4373	fig|6666666.59348.peg.3834	fig|6666666.59349.peg.4953	fig|6666666.59350.peg.495	fig|6666666.59351.peg.4403	fig|6666666.59353.peg.662	fig|6666666.59355.peg.3710	fig|6666666.59354.peg.1022

5908	DNA mismatch repair protein MutL	fig|6666666.59329.peg.697	fig|6666666.59330.peg.3558	fig|6666666.59331.peg.3393	fig|6666666.59332.peg.1978	fig|6666666.59334.peg.1635	fig|6666666.59335.peg.4129	fig|6666666.59336.peg.3758	fig|6666666.59333.peg.284	fig|6666666.59338.peg.999	fig|6666666.59339.peg.8856	fig|6666666.59340.peg.459	fig|6666666.59337.peg.1780	fig|6666666.59342.peg.2602	fig|6666666.71181.peg.4636	fig|6666666.59344.peg.5882	fig|6666666.59345.peg.5666	fig|6666666.59341.peg.3763	fig|6666666.59347.peg.5349	fig|6666666.59346.peg.3042	fig|6666666.59348.peg.2812	fig|6666666.59349.peg.3168	fig|6666666.59350.peg.903	fig|6666666.59351.peg.3092	fig|6666666.59353.peg.4992	fig|6666666.59355.peg.5076	fig|6666666.59354.peg.5802

4563	Diaminopimelate decarboxylase EC 4.1.1.20	fig|6666666.59329.peg.1373	fig|6666666.59330.peg.3052	fig|6666666.59331.peg.778	fig|6666666.59332.peg.4405	fig|6666666.59334.peg.2632	fig|6666666.59335.peg.3336	fig|6666666.59336.peg.1117	fig|6666666.59333.peg.5464	fig|6666666.59338.peg.3401	fig|6666666.59339.peg.8523	fig|6666666.59340.peg.3597	fig|6666666.59337.peg.1617	fig|6666666.59342.peg.1109	fig|6666666.71181.peg.4374	fig|6666666.59344.peg.3170	fig|6666666.59345.peg.3033	fig|6666666.59341.peg.2404	fig|6666666.59347.peg.5076	fig|6666666.59346.peg.600	fig|6666666.59348.peg.3352	fig|6666666.59349.peg.2206	fig|6666666.59350.peg.2327	fig|6666666.59351.peg.72	fig|6666666.59353.peg.4328	fig|6666666.59355.peg.4541	fig|6666666.59354.peg.308

6069	tRNA dihydrouridine synthase B EC 1.-.-.-	fig|6666666.59329.peg.3755	fig|6666666.59330.peg.196	fig|6666666.59331.peg.3196	fig|6666666.59332.peg.124	fig|6666666.59334.peg.3442	fig|6666666.59335.peg.2629	fig|6666666.59336.peg.2269	fig|6666666.59333.peg.2642	fig|6666666.59338.peg.5058	fig|6666666.59339.peg.6438	fig|6666666.59340.peg.819	fig|6666666.59337.peg.3839	fig|6666666.59342.peg.2608	fig|6666666.71181.peg.967	fig|6666666.59344.peg.2331	fig|6666666.59345.peg.5148	fig|6666666.59341.peg.1182	fig|6666666.59347.peg.2627	fig|6666666.59346.peg.1149	fig|6666666.59348.peg.943	fig|6666666.59349.peg.1951	fig|6666666.59350.peg.2262	fig|6666666.59351.peg.2748	fig|6666666.59353.peg.3384	fig|6666666.59355.peg.3483	fig|6666666.59354.peg.6

9037	8-amino-7-oxononanoate synthase EC 2.3.1.47	fig|6666666.59329.peg.3675	fig|6666666.59330.peg.3302	fig|6666666.59331.peg.6245	fig|6666666.59332.peg.3211	fig|6666666.59334.peg.2558	fig|6666666.59335.peg.615	fig|6666666.59336.peg.5000	fig|6666666.59333.peg.4860	fig|6666666.59338.peg.3902	fig|6666666.59339.peg.462	fig|6666666.59340.peg.2890	fig|6666666.59337.peg.4600	fig|6666666.59342.peg.1825	fig|6666666.71181.peg.3672	fig|6666666.59344.peg.4090	fig|6666666.59345.peg.3354	fig|6666666.59341.peg.5695	fig|6666666.59347.peg.1136	fig|6666666.59346.peg.2625	fig|6666666.59348.peg.1075	fig|6666666.59349.peg.1620	fig|6666666.59350.peg.4458	fig|6666666.59351.peg.3357	fig|6666666.59353.peg.2133	fig|6666666.59355.peg.2687	fig|6666666.59354.peg.2868

7014	Hypothetical metal-binding enzyme, YcbL homolog	fig|6666666.59329.peg.3544	fig|6666666.59330.peg.4727	fig|6666666.59331.peg.3309	fig|6666666.59332.peg.430	fig|6666666.59334.peg.3283	fig|6666666.59335.peg.6178	fig|6666666.59336.peg.5126	fig|6666666.59333.peg.3312	fig|6666666.59338.peg.4096	fig|6666666.59339.peg.1613	fig|6666666.59340.peg.638	fig|6666666.59337.peg.1775	fig|6666666.59342.peg.3410	fig|6666666.71181.peg.876	fig|6666666.59344.peg.2775	fig|6666666.59345.peg.869	fig|6666666.59341.peg.2349	fig|6666666.59347.peg.2871	fig|6666666.59346.peg.3697	fig|6666666.59348.peg.642	fig|6666666.59349.peg.402	fig|6666666.59350.peg.4195	fig|6666666.59351.peg.5491	fig|6666666.59353.peg.1668	fig|6666666.59355.peg.4640	fig|6666666.59354.peg.2971

1802	CsdL EC-YgdL protein of the HesA MoeB ThiF family, part of the CsdA-E-L sulfur transfer pathway	fig|6666666.59329.peg.3332	fig|6666666.59330.peg.979	fig|6666666.59331.peg.1384	fig|6666666.59332.peg.868	fig|6666666.59334.peg.1529	fig|6666666.59335.peg.1116	fig|6666666.59336.peg.176	fig|6666666.59333.peg.4700	fig|6666666.59338.peg.4248	fig|6666666.59339.peg.8496	fig|6666666.59340.peg.777	fig|6666666.59337.peg.3636	fig|6666666.59342.peg.5330	fig|6666666.71181.peg.176	fig|6666666.59344.peg.2618	fig|6666666.59345.peg.5211	fig|6666666.59341.peg.532	fig|6666666.59347.peg.5256	fig|6666666.59346.peg.1846	fig|6666666.59348.peg.2463	fig|6666666.59349.peg.2513	fig|6666666.59350.peg.2441	fig|6666666.59351.peg.3888	fig|6666666.59353.peg.1960	fig|6666666.59355.peg.4563	fig|6666666.59354.peg.4783

8094	Aspartokinase EC 2.7.2.4 Homoserine dehydrogenase EC 1.1.1.3	fig|6666666.59329.peg.1312	fig|6666666.59330.peg.1674	fig|6666666.59331.peg.3780	fig|6666666.59332.peg.2932	fig|6666666.59334.peg.655	fig|6666666.59335.peg.265	fig|6666666.59336.peg.5477	fig|6666666.59333.peg.4997	fig|6666666.59338.peg.1054	fig|6666666.59339.peg.6372	fig|6666666.59340.peg.882	fig|6666666.59337.peg.1677	fig|6666666.59342.peg.3841	fig|6666666.71181.peg.436	fig|6666666.59344.peg.1339	fig|6666666.59345.peg.5403	fig|6666666.59341.peg.4218	fig|6666666.59347.peg.3267	fig|6666666.59346.peg.1591	fig|6666666.59348.peg.1953	fig|6666666.59349.peg.2739	fig|6666666.59350.peg.7263	fig|6666666.59351.peg.4259	fig|6666666.59353.peg.2532	fig|6666666.59355.peg.2924	fig|6666666.59354.peg.922

6385	Peptidyl-prolyl cis-trans isomerase PpiB EC 5.2.1.8	fig|6666666.59329.peg.1644	fig|6666666.59330.peg.2791	fig|6666666.59331.peg.3602	fig|6666666.59332.peg.861	fig|6666666.59334.peg.2360	fig|6666666.59335.peg.115	fig|6666666.59336.peg.307	fig|6666666.59333.peg.1211	fig|6666666.59338.peg.2616	fig|6666666.59339.peg.5040	fig|6666666.59340.peg.1797	fig|6666666.59337.peg.2300	fig|6666666.59342.peg.2274	fig|6666666.71181.peg.3627	fig|6666666.59344.peg.3385	fig|6666666.59345.peg.2241	fig|6666666.59341.peg.4365	fig|6666666.59347.peg.2807	fig|6666666.59346.peg.2714	fig|6666666.59348.peg.4185	fig|6666666.59349.peg.2074	fig|6666666.59350.peg.3557	fig|6666666.59351.peg.4235	fig|6666666.59353.peg.2235	fig|6666666.59355.peg.3777	fig|6666666.59354.peg.1404

4952	Nitrogen regulatory protein P-II	fig|6666666.59329.peg.3151	fig|6666666.59330.peg.1767	fig|6666666.59331.peg.4977	fig|6666666.59332.peg.425	fig|6666666.59334.peg.891	fig|6666666.59335.peg.1525	fig|6666666.59336.peg.563	fig|6666666.59333.peg.326	fig|6666666.59338.peg.3198	fig|6666666.59339.peg.5136	fig|6666666.59340.peg.2040	fig|6666666.59337.peg.636	fig|6666666.59342.peg.4937	fig|6666666.71181.peg.4730	fig|6666666.59344.peg.1602	fig|6666666.59345.peg.2396	fig|6666666.59341.peg.5198	fig|6666666.59347.peg.3157	fig|6666666.59346.peg.751	fig|6666666.59348.peg.4964	fig|6666666.59349.peg.583	fig|6666666.59350.peg.6706	fig|6666666.59351.peg.515	fig|6666666.59353.peg.2675	fig|6666666.59355.peg.1806	fig|6666666.59354.peg.1483

3254	HflK protein	fig|6666666.59329.peg.692	fig|6666666.59330.peg.3562	fig|6666666.59331.peg.1284	fig|6666666.59332.peg.4223	fig|6666666.59334.peg.3059	fig|6666666.59335.peg.4133	fig|6666666.59336.peg.3763	fig|6666666.59333.peg.289	fig|6666666.59338.peg.1004	fig|6666666.59339.peg.8862	fig|6666666.59340.peg.3266	fig|6666666.59337.peg.1784	fig|6666666.59342.peg.2596	fig|6666666.71181.peg.4632	fig|6666666.59344.peg.5886	fig|6666666.59345.peg.5662	fig|6666666.59341.peg.4274	fig|6666666.59347.peg.5345	fig|6666666.59346.peg.3037	fig|6666666.59348.peg.5032	fig|6666666.59349.peg.3173	fig|6666666.59350.peg.3866	fig|6666666.59351.peg.3096	fig|6666666.59353.peg.4988	fig|6666666.59355.peg.5072	fig|6666666.59354.peg.5844

5139	Diaminohydroxyphosphoribosylaminopyrimidine deaminase EC 3.5.4.26 5-amino-6- 5-phosphoribosylamino uracil reductase EC 1.1.1.193	fig|6666666.59329.peg.5359	fig|6666666.59330.peg.1061	fig|6666666.59331.peg.2620	fig|6666666.59332.peg.4370	fig|6666666.59334.peg.2064	fig|6666666.59335.peg.1695	fig|6666666.59336.peg.6547	fig|6666666.59333.peg.2445	fig|6666666.59338.peg.5269	fig|6666666.59339.peg.3781	fig|6666666.59340.peg.1376	fig|6666666.59337.peg.1141	fig|6666666.59342.peg.3513	fig|6666666.71181.peg.2728	fig|6666666.59344.peg.1584	fig|6666666.59345.peg.3542	fig|6666666.59341.peg.2613	fig|6666666.59347.peg.4343	fig|6666666.59346.peg.3379	fig|6666666.59348.peg.923	fig|6666666.59349.peg.2802	fig|6666666.59350.peg.1719	fig|6666666.59351.peg.948	fig|6666666.59353.peg.779	fig|6666666.59355.peg.925	fig|6666666.59354.peg.2932

6894	Putative protease	fig|6666666.59329.peg.2303	fig|6666666.59330.peg.3675	fig|6666666.59331.peg.3855	fig|6666666.59332.peg.4667	fig|6666666.59334.peg.2955	fig|6666666.59335.peg.2111	fig|6666666.59336.peg.3979	fig|6666666.59333.peg.2308	fig|6666666.59338.peg.2723	fig|6666666.59339.peg.6911	fig|6666666.59340.peg.248	fig|6666666.59337.peg.693	fig|6666666.59342.peg.3202	fig|6666666.71181.peg.3535	fig|6666666.59344.peg.5115	fig|6666666.59345.peg.2035	fig|6666666.59341.peg.2849	fig|6666666.59347.peg.1251	fig|6666666.59346.peg.3176	fig|6666666.59348.peg.756	fig|6666666.59349.peg.3077	fig|6666666.59350.peg.5729	fig|6666666.59351.peg.3069	fig|6666666.59353.peg.4772	fig|6666666.59355.peg.484	fig|6666666.59354.peg.78

3336	Protein-export membrane protein SecF TC 3.A.5.1.1	fig|6666666.59329.peg.642	fig|6666666.59330.peg.2107	fig|6666666.59331.peg.5821	fig|6666666.59332.peg.1437	fig|6666666.59334.peg.5089	fig|6666666.59335.peg.5095	fig|6666666.59336.peg.2001	fig|6666666.59333.peg.1444	fig|6666666.59338.peg.1331	fig|6666666.59339.peg.6900	fig|6666666.59340.peg.1089	fig|6666666.59337.peg.734	fig|6666666.59342.peg.1142	fig|6666666.71181.peg.2731	fig|6666666.59344.peg.1103	fig|6666666.59345.peg.495	fig|6666666.59341.peg.1910	fig|6666666.59347.peg.4278	fig|6666666.59346.peg.2745	fig|6666666.59348.peg.1855	fig|6666666.59349.peg.1928	fig|6666666.59350.peg.6340	fig|6666666.59351.peg.2335	fig|6666666.59353.peg.783	fig|6666666.59355.peg.922	fig|6666666.59354.peg.3643

7592	SSU ribosomal protein S3p S3e	fig|6666666.59329.peg.715	fig|6666666.59330.peg.556	fig|6666666.59331.peg.46	fig|6666666.59332.peg.2141	fig|6666666.59334.peg.1487	fig|6666666.59335.peg.4349	fig|6666666.59336.peg.148	fig|6666666.59333.peg.368	fig|6666666.59338.peg.1387	fig|6666666.59339.peg.8270	fig|6666666.59340.peg.57	fig|6666666.59337.peg.386	fig|6666666.59342.peg.306	fig|6666666.71181.peg.3454	fig|6666666.59344.peg.4937	fig|6666666.59345.peg.2533	fig|6666666.59341.peg.2500	fig|6666666.59347.peg.4825	fig|6666666.59346.peg.852	fig|6666666.59348.peg.1762	fig|6666666.59349.peg.1100	fig|6666666.59350.peg.3195	fig|6666666.59351.peg.4811	fig|6666666.59353.peg.4596	fig|6666666.59355.peg.4735	fig|6666666.59354.peg.1745

7591	Exopolyphosphatase EC 3.6.1.11	fig|6666666.59329.peg.6451	fig|6666666.59330.peg.3680	fig|6666666.59331.peg.4564	fig|6666666.59332.peg.1271	fig|6666666.59334.peg.1106	fig|6666666.59335.peg.2568	fig|6666666.59336.peg.1637	fig|6666666.59333.peg.5153	fig|6666666.59338.peg.1858	fig|6666666.59339.peg.6920	fig|6666666.59340.peg.3690	fig|6666666.59337.peg.709	fig|6666666.59342.peg.5901	fig|6666666.71181.peg.2455	fig|6666666.59344.peg.2455	fig|6666666.59345.peg.3680	fig|6666666.59341.peg.3803	fig|6666666.59347.peg.4262	fig|6666666.59346.peg.4605	fig|6666666.59348.peg.3205	fig|6666666.59349.peg.4686	fig|6666666.59350.peg.6318	fig|6666666.59351.peg.4776	fig|6666666.59353.peg.4012	fig|6666666.59355.peg.3971	fig|6666666.59354.peg.1544

3847	Cysteinyl-tRNA synthetase EC 6.1.1.16	fig|6666666.59329.peg.4109	fig|6666666.59330.peg.1514	fig|6666666.59331.peg.1167	fig|6666666.59332.peg.4702	fig|6666666.59334.peg.4741	fig|6666666.59335.peg.2940	fig|6666666.59336.peg.4781	fig|6666666.59333.peg.4964	fig|6666666.59338.peg.1124	fig|6666666.59339.peg.284	fig|6666666.59340.peg.1480	fig|6666666.59337.peg.2301	fig|6666666.59342.peg.2275	fig|6666666.71181.peg.3628	fig|6666666.59344.peg.3384	fig|6666666.59345.peg.1296	fig|6666666.59341.peg.3623	fig|6666666.59347.peg.2317	fig|6666666.59346.peg.4356	fig|6666666.59348.peg.1665	fig|6666666.59349.peg.2073	fig|6666666.59350.peg.3034	fig|6666666.59351.peg.3580	fig|6666666.59353.peg.3759	fig|6666666.59355.peg.3776	fig|6666666.59354.peg.1403

2598	YrbA protein	fig|6666666.59329.peg.1450	fig|6666666.59330.peg.56	fig|6666666.59331.peg.4794	fig|6666666.59332.peg.1279	fig|6666666.59334.peg.84	fig|6666666.59335.peg.219	fig|6666666.59336.peg.359	fig|6666666.59333.peg.275	fig|6666666.59338.peg.119	fig|6666666.59339.peg.8186	fig|6666666.59340.peg.839	fig|6666666.59337.peg.843	fig|6666666.59342.peg.439	fig|6666666.71181.peg.1029	fig|6666666.59344.peg.1809	fig|6666666.59345.peg.4850	fig|6666666.59341.peg.2691	fig|6666666.59347.peg.3240	fig|6666666.59346.peg.628	fig|6666666.59348.peg.3521	fig|6666666.59349.peg.32	fig|6666666.59350.peg.3351	fig|6666666.59351.peg.3453	fig|6666666.59353.peg.2348	fig|6666666.59355.peg.1621	fig|6666666.59354.peg.348

2599	Outer membrane stress sensor protease DegS	fig|6666666.59329.peg.2741	fig|6666666.59330.peg.958	fig|6666666.59331.peg.2776	fig|6666666.59332.peg.6236	fig|6666666.59334.peg.396	fig|6666666.59335.peg.3440	fig|6666666.59336.peg.1584	fig|6666666.59333.peg.625	fig|6666666.59338.peg.5291	fig|6666666.59339.peg.4932	fig|6666666.59340.peg.1042	fig|6666666.59337.peg.24	fig|6666666.59342.peg.5933	fig|6666666.71181.peg.1952	fig|6666666.59344.peg.1818	fig|6666666.59345.peg.3818	fig|6666666.59341.peg.3552	fig|6666666.59347.peg.3917	fig|6666666.59346.peg.1639	fig|6666666.59348.peg.3436	fig|6666666.59349.peg.36	fig|6666666.59350.peg.2129	fig|6666666.59351.peg.3455	fig|6666666.59353.peg.2354	fig|6666666.59355.peg.779	fig|6666666.59354.peg.352

1041	N-acetylglucosamine-6P-responsive transcriptional repressor NagC, ROK family	fig|6666666.59329.peg.3099	fig|6666666.59330.peg.3003	fig|6666666.59331.peg.1536	fig|6666666.59332.peg.2038	fig|6666666.59334.peg.812	fig|6666666.59335.peg.5798	fig|6666666.59336.peg.4947	fig|6666666.59333.peg.4603	fig|6666666.59338.peg.5216	fig|6666666.59339.peg.1197	fig|6666666.59340.peg.653	fig|6666666.59337.peg.3927	fig|6666666.59342.peg.3667	fig|6666666.71181.peg.2177	fig|6666666.59344.peg.3827	fig|6666666.59345.peg.4342	fig|6666666.59341.peg.4084	fig|6666666.59347.peg.605	fig|6666666.59346.peg.122	fig|6666666.59348.peg.5440	fig|6666666.59349.peg.1452	fig|6666666.59350.peg.436	fig|6666666.59351.peg.2259	fig|6666666.59353.peg.808	fig|6666666.59355.peg.4452	fig|6666666.59354.peg.2554

8651	Shikimate 5-dehydrogenase I alpha EC 1.1.1.25	fig|6666666.59329.peg.5456	fig|6666666.59330.peg.655	fig|6666666.59331.peg.5656	fig|6666666.59332.peg.5273	fig|6666666.59334.peg.1518	fig|6666666.59335.peg.5011	fig|6666666.59336.peg.1982	fig|6666666.59333.peg.1363	fig|6666666.59338.peg.2071	fig|6666666.59339.peg.8106	fig|6666666.59340.peg.878	fig|6666666.59337.peg.3911	fig|6666666.59342.peg.5715	fig|6666666.71181.peg.3425	fig|6666666.59344.peg.4906	fig|6666666.59345.peg.4917	fig|6666666.59341.peg.5019	fig|6666666.59347.peg.4738	fig|6666666.59346.peg.1834	fig|6666666.59348.peg.3239	fig|6666666.59349.peg.2827	fig|6666666.59350.peg.2708	fig|6666666.59351.peg.5066	fig|6666666.59353.peg.4627	fig|6666666.59355.peg.4704	fig|6666666.59354.peg.4821

2323	PTS IIA-like nitrogen-regulatory protein PtsN	fig|6666666.59329.peg.1159	fig|6666666.59330.peg.43	fig|6666666.59331.peg.4670	fig|6666666.59332.peg.2743	fig|6666666.59334.peg.105	fig|6666666.59335.peg.3693	fig|6666666.59336.peg.3258	fig|6666666.59333.peg.259	fig|6666666.59338.peg.1178	fig|6666666.59339.peg.6876	fig|6666666.59340.peg.3539	fig|6666666.59337.peg.827	fig|6666666.59342.peg.1429	fig|6666666.71181.peg.1014	fig|6666666.59344.peg.1793	fig|6666666.59345.peg.4863	fig|6666666.59341.peg.1240	fig|6666666.59347.peg.4726	fig|6666666.59346.peg.643	fig|6666666.59348.peg.3757	fig|6666666.59349.peg.18	fig|6666666.59350.peg.1645	fig|6666666.59351.peg.3436	fig|6666666.59353.peg.2332	fig|6666666.59355.peg.1605	fig|6666666.59354.peg.3616

11003	Twin-arginine translocation protein TatA	fig|6666666.59329.peg.5192	fig|6666666.59330.peg.253	fig|6666666.59331.peg.2430	fig|6666666.59332.peg.9	fig|6666666.59334.peg.2866	fig|6666666.59335.peg.43	fig|6666666.59336.peg.894	fig|6666666.59333.peg.2510	fig|6666666.59338.peg.3943	fig|6666666.59339.peg.7574	fig|6666666.59340.peg.2540	fig|6666666.59337.peg.961	fig|6666666.59342.peg.4558	fig|6666666.71181.peg.3338	fig|6666666.59344.peg.3412	fig|6666666.59345.peg.2712	fig|6666666.59341.peg.155	fig|6666666.59347.peg.4637	fig|6666666.59346.peg.3359	fig|6666666.59348.peg.113	fig|6666666.59349.peg.3414	fig|6666666.59350.peg.1452	fig|6666666.59351.peg.4998	fig|6666666.59353.peg.3800	fig|6666666.59355.peg.3396	fig|6666666.59354.peg.2225

2102	Oligopeptide transport system permease protein OppC TC 3.A.1.5.1	fig|6666666.59329.peg.836	fig|6666666.59330.peg.3511	fig|6666666.59331.peg.3231	fig|6666666.59332.peg.2857	fig|6666666.59334.peg.5340	fig|6666666.59335.peg.3751	fig|6666666.59336.peg.6368	fig|6666666.59333.peg.1171	fig|6666666.59338.peg.3554	fig|6666666.59339.peg.4816	fig|6666666.59340.peg.2109	fig|6666666.59337.peg.4925	fig|6666666.59342.peg.2157	fig|6666666.71181.peg.483	fig|6666666.59344.peg.4532	fig|6666666.59345.peg.1106	fig|6666666.59341.peg.1955	fig|6666666.59347.peg.5129	fig|6666666.59346.peg.2400	fig|6666666.59348.peg.5427	fig|6666666.59349.peg.2092	fig|6666666.59350.peg.4010	fig|6666666.59351.peg.2606	fig|6666666.59353.peg.1751	fig|6666666.59355.peg.3472	fig|6666666.59354.peg.3393

898	RNA polymerase sigma factor RpoE	fig|6666666.59329.peg.496	fig|6666666.59330.peg.378	fig|6666666.59331.peg.3848	fig|6666666.59332.peg.1534	fig|6666666.59334.peg.4734	fig|6666666.59335.peg.4180	fig|6666666.59336.peg.1086	fig|6666666.59333.peg.2801	fig|6666666.59338.peg.3156	fig|6666666.59339.peg.7359	fig|6666666.59340.peg.1537	fig|6666666.59337.peg.2474	fig|6666666.59342.peg.3542	fig|6666666.71181.peg.3596	fig|6666666.59344.peg.5553	fig|6666666.59345.peg.579	fig|6666666.59341.peg.4052	fig|6666666.59347.peg.2810	fig|6666666.59346.peg.328	fig|6666666.59348.peg.5520	fig|6666666.59349.peg.3237	fig|6666666.59350.peg.2716	fig|6666666.59351.peg.4547	fig|6666666.59353.peg.2645	fig|6666666.59355.peg.4273	fig|6666666.59354.peg.4912

4110	UDP-N-acetylglucosamine--N-acetylmuramyl- pentapeptide pyrophosphoryl-undecaprenol N-acetylglucosamine transferase EC 2.4.1.227	fig|6666666.59329.peg.2835	fig|6666666.59330.peg.1278	fig|6666666.59331.peg.986	fig|6666666.59332.peg.3452	fig|6666666.59334.peg.949	fig|6666666.59335.peg.831	fig|6666666.59336.peg.948	fig|6666666.59333.peg.4370	fig|6666666.59338.peg.4973	fig|6666666.59339.peg.4971	fig|6666666.59340.peg.1190	fig|6666666.59337.peg.49	fig|6666666.59342.peg.3062	fig|6666666.71181.peg.1980	fig|6666666.59344.peg.1479	fig|6666666.59345.peg.3792	fig|6666666.59341.peg.4861	fig|6666666.59347.peg.4001	fig|6666666.59346.peg.1241	fig|6666666.59348.peg.2552	fig|6666666.59349.peg.2985	fig|6666666.59350.peg.1037	fig|6666666.59351.peg.208	fig|6666666.59353.peg.138	fig|6666666.59355.peg.748	fig|6666666.59354.peg.5710

9175	ATP synthase alpha chain EC 3.6.3.14	fig|6666666.59329.peg.3991	fig|6666666.59330.peg.610	fig|6666666.59331.peg.405	fig|6666666.59332.peg.103	fig|6666666.59334.peg.2497	fig|6666666.59335.peg.796	fig|6666666.59336.peg.1518	fig|6666666.59333.peg.25	fig|6666666.59338.peg.497	fig|6666666.59339.peg.2293	fig|6666666.59340.peg.1174	fig|6666666.59337.peg.2088	fig|6666666.59342.peg.2038	fig|6666666.71181.peg.596	fig|6666666.59344.peg.4269	fig|6666666.59345.peg.3327	fig|6666666.59341.peg.2137	fig|6666666.59347.peg.2037	fig|6666666.59346.peg.1407	fig|6666666.59348.peg.336	fig|6666666.59349.peg.1034	fig|6666666.59350.peg.6488	fig|6666666.59351.peg.5355	fig|6666666.59353.peg.3084	fig|6666666.59355.peg.2264	fig|6666666.59354.peg.1157

9883	FIG000859 hypothetical protein YebC	fig|6666666.59329.peg.2229	fig|6666666.59330.peg.3020	fig|6666666.59331.peg.4284	fig|6666666.59332.peg.4271	fig|6666666.59334.peg.4993	fig|6666666.59335.peg.3137	fig|6666666.59336.peg.6477	fig|6666666.59333.peg.4741	fig|6666666.59338.peg.241	fig|6666666.59339.peg.5441	fig|6666666.59340.peg.1632	fig|6666666.59337.peg.230	fig|6666666.59342.peg.5502	fig|6666666.71181.peg.1920	fig|6666666.59344.peg.2518	fig|6666666.59345.peg.5269	fig|6666666.59341.peg.3336	fig|6666666.59347.peg.9	fig|6666666.59346.peg.145	fig|6666666.59348.peg.2048	fig|6666666.59349.peg.3986	fig|6666666.59350.peg.3541	fig|6666666.59351.peg.1970	fig|6666666.59353.peg.1489	fig|6666666.59355.peg.2039	fig|6666666.59354.peg.3796

9212	CTP synthase EC 6.3.4.2	fig|6666666.59329.peg.2715	fig|6666666.59330.peg.2467	fig|6666666.59331.peg.3030	fig|6666666.59332.peg.196	fig|6666666.59334.peg.329	fig|6666666.59335.peg.1502	fig|6666666.59336.peg.2779	fig|6666666.59333.peg.4312	fig|6666666.59338.peg.2736	fig|6666666.59339.peg.7387	fig|6666666.59340.peg.1066	fig|6666666.59337.peg.574	fig|6666666.59342.peg.3536	fig|6666666.71181.peg.963	fig|6666666.59344.peg.5349	fig|6666666.59345.peg.5066	fig|6666666.59341.peg.1323	fig|6666666.59347.peg.3167	fig|6666666.59346.peg.3152	fig|6666666.59348.peg.3624	fig|6666666.59349.peg.3469	fig|6666666.59350.peg.3249	fig|6666666.59351.peg.2588	fig|6666666.59353.peg.2277	fig|6666666.59355.peg.2948	fig|6666666.59354.peg.917

9079	Pyruvate kinase EC 2.7.1.40	fig|6666666.59329.peg.1979	fig|6666666.59330.peg.1398	fig|6666666.59331.peg.4961	fig|6666666.59332.peg.5347	fig|6666666.59334.peg.3157	fig|6666666.59335.peg.4197	fig|6666666.59336.peg.6435	fig|6666666.59333.peg.111	fig|6666666.59338.peg.981	fig|6666666.59339.peg.7864	fig|6666666.59340.peg.854	fig|6666666.59337.peg.1411	fig|6666666.59342.peg.4671	fig|6666666.71181.peg.2554	fig|6666666.59344.peg.2837	fig|6666666.59345.peg.1048	fig|6666666.59341.peg.635	fig|6666666.59347.peg.3930	fig|6666666.59346.peg.121	fig|6666666.59348.peg.206	fig|6666666.59349.peg.397	fig|6666666.59350.peg.981	fig|6666666.59351.peg.920	fig|6666666.59353.peg.1361	fig|6666666.59355.peg.2511	fig|6666666.59354.peg.1348

5775	ATP-dependent RNA helicase RhlE	fig|6666666.59329.peg.1074	fig|6666666.59330.peg.2045	fig|6666666.59331.peg.3860	fig|6666666.59332.peg.2727	fig|6666666.59334.peg.4028	fig|6666666.59335.peg.4610	fig|6666666.59336.peg.5944	fig|6666666.59333.peg.5036	fig|6666666.59338.peg.1013	fig|6666666.59339.peg.8493	fig|6666666.59340.peg.3297	fig|6666666.59337.peg.317	fig|6666666.59342.peg.5582	fig|6666666.71181.peg.2454	fig|6666666.59344.peg.2456	fig|6666666.59345.peg.2777	fig|6666666.59341.peg.2202	fig|6666666.59347.peg.2486	fig|6666666.59346.peg.1914	fig|6666666.59348.peg.2164	fig|6666666.59349.peg.3266	fig|6666666.59350.peg.2965	fig|6666666.59351.peg.4007	fig|6666666.59353.peg.3125	fig|6666666.59355.peg.471	fig|6666666.59354.peg.4892

1883	Translation elongation factor G	fig|6666666.59329.peg.501	fig|6666666.59330.peg.75	fig|6666666.59331.peg.3701	fig|6666666.59332.peg.3863	fig|6666666.59334.peg.486	fig|6666666.59335.peg.6042	fig|6666666.59336.peg.5458	fig|6666666.59333.peg.489	fig|6666666.59338.peg.1489	fig|6666666.59339.peg.8879	fig|6666666.59340.peg.2334	fig|6666666.59337.peg.1826	fig|6666666.59342.peg.3793	fig|6666666.71181.peg.3408	fig|6666666.59344.peg.907	fig|6666666.59345.peg.5549	fig|6666666.59341.peg.4765	fig|6666666.59347.peg.5113	fig|6666666.59346.peg.5342	fig|6666666.59348.peg.5464	fig|6666666.59349.peg.1137	fig|6666666.59350.peg.161	fig|6666666.59351.peg.2695	fig|6666666.59353.peg.2650	fig|6666666.59355.peg.2582	fig|6666666.59354.peg.3559

130	Glutamine synthetase type I EC 6.3.1.2	fig|6666666.59329.peg.359	fig|6666666.59330.peg.403	fig|6666666.59331.peg.905	fig|6666666.59332.peg.34	fig|6666666.59334.peg.3117	fig|6666666.59335.peg.4333	fig|6666666.59336.peg.6355	fig|6666666.59333.peg.3455	fig|6666666.59338.peg.5675	fig|6666666.59339.peg.6171	fig|6666666.59340.peg.2197	fig|6666666.59337.peg.987	fig|6666666.59342.peg.3182	fig|6666666.71181.peg.2593	fig|6666666.59344.peg.195	fig|6666666.59345.peg.2691	fig|6666666.59341.peg.3715	fig|6666666.59347.peg.373	fig|6666666.59346.peg.1085	fig|6666666.59348.peg.672	fig|6666666.59349.peg.3591	fig|6666666.59350.peg.3097	fig|6666666.59351.peg.4836	fig|6666666.59353.peg.4831	fig|6666666.59355.peg.562	fig|6666666.59354.peg.1213

138	3-oxoacyl-[acyl-carrier-protein] synthase, KASIII EC 2.3.1.41	fig|6666666.59329.peg.2267	fig|6666666.59330.peg.761	fig|6666666.59331.peg.3043	fig|6666666.59332.peg.6205	fig|6666666.59334.peg.3238	fig|6666666.59335.peg.4494	fig|6666666.59336.peg.4196	fig|6666666.59333.peg.1591	fig|6666666.59338.peg.4645	fig|6666666.59339.peg.4704	fig|6666666.59340.peg.409	fig|6666666.59337.peg.1200	fig|6666666.59342.peg.1445	fig|6666666.71181.peg.1621	fig|6666666.59344.peg.2431	fig|6666666.59345.peg.1065	fig|6666666.59341.peg.14	fig|6666666.59347.peg.849	fig|6666666.59346.peg.1205	fig|6666666.59348.peg.3856	fig|6666666.59349.peg.3684	fig|6666666.59350.peg.3677	fig|6666666.59351.peg.3783	fig|6666666.59353.peg.312	fig|6666666.59355.peg.1352	fig|6666666.59354.peg.1910

7259	LSU ribosomal protein L3p L3e	fig|6666666.59329.peg.1101	fig|6666666.59330.peg.550	fig|6666666.59331.peg.3027	fig|6666666.59332.peg.3794	fig|6666666.59334.peg.1480	fig|6666666.59335.peg.1851	fig|6666666.59336.peg.140	fig|6666666.59333.peg.2688	fig|6666666.59338.peg.1394	fig|6666666.59339.peg.8263	fig|6666666.59340.peg.1727	fig|6666666.59337.peg.393	fig|6666666.59342.peg.300	fig|6666666.71181.peg.3460	fig|6666666.59344.peg.4943	fig|6666666.59345.peg.2540	fig|6666666.59341.peg.2493	fig|6666666.59347.peg.4819	fig|6666666.59346.peg.5021	fig|6666666.59348.peg.410	fig|6666666.59349.peg.1093	fig|6666666.59350.peg.4267	fig|6666666.59351.peg.4818	fig|6666666.59353.peg.4590	fig|6666666.59355.peg.4741	fig|6666666.59354.peg.1752

7628	Excinuclease ABC subunit A	fig|6666666.59329.peg.4867	fig|6666666.59330.peg.2	fig|6666666.59331.peg.5522	fig|6666666.59332.peg.3570	fig|6666666.59334.peg.4104	fig|6666666.59335.peg.195	fig|6666666.59336.peg.1293	fig|6666666.59333.peg.585	fig|6666666.59338.peg.807	fig|6666666.59339.peg.7749	fig|6666666.59340.peg.239	fig|6666666.59337.peg.788	fig|6666666.59342.peg.4463	fig|6666666.71181.peg.6039	fig|6666666.59344.peg.2761	fig|6666666.59345.peg.4909	fig|6666666.59341.peg.5794	fig|6666666.59347.peg.3126	fig|6666666.59346.peg.1595	fig|6666666.59348.peg.1966	fig|6666666.59349.peg.4446	fig|6666666.59350.peg.3848	fig|6666666.59351.peg.3410	fig|6666666.59353.peg.2980	fig|6666666.59355.peg.2886	fig|6666666.59354.peg.4687

7828	Succinyl-CoA ligase [ADP-forming] beta chain EC 6.2.1.5	fig|6666666.59329.peg.155	fig|6666666.59330.peg.2775	fig|6666666.59331.peg.1416	fig|6666666.59332.peg.659	fig|6666666.59334.peg.2834	fig|6666666.59335.peg.2747	fig|6666666.59336.peg.2113	fig|6666666.59333.peg.904	fig|6666666.59338.peg.3105	fig|6666666.59339.peg.7185	fig|6666666.59340.peg.1021	fig|6666666.59337.peg.4113	fig|6666666.59342.peg.481	fig|6666666.71181.peg.3238	fig|6666666.59344.peg.4589	fig|6666666.59345.peg.4319	fig|6666666.59341.peg.1576	fig|6666666.59347.peg.4388	fig|6666666.59346.peg.2437	fig|6666666.59348.peg.4475	fig|6666666.59349.peg.2523	fig|6666666.59350.peg.6257	fig|6666666.59351.peg.3731	fig|6666666.59353.peg.2089	fig|6666666.59355.peg.2735	fig|6666666.59354.peg.1315

1296	Fructose-bisphosphate aldolase class II EC 4.1.2.13	fig|6666666.59329.peg.4776	fig|6666666.59330.peg.358	fig|6666666.59331.peg.1206	fig|6666666.59332.peg.532	fig|6666666.59334.peg.1056	fig|6666666.59335.peg.5936	fig|6666666.59336.peg.3419	fig|6666666.59333.peg.3475	fig|6666666.59338.peg.4171	fig|6666666.59339.peg.703	fig|6666666.59340.peg.3556	fig|6666666.59337.peg.539	fig|6666666.59342.peg.754	fig|6666666.71181.peg.3141	fig|6666666.59344.peg.4727	fig|6666666.59345.peg.1929	fig|6666666.59341.peg.2537	fig|6666666.59347.peg.3203	fig|6666666.59346.peg.308	fig|6666666.59348.peg.194	fig|6666666.59349.peg.3005	fig|6666666.59350.peg.960	fig|6666666.59351.peg.352	fig|6666666.59353.peg.4425	fig|6666666.59355.peg.4505	fig|6666666.59354.peg.3123

10050	Acyl-CoA dehydrogenase, short-chain specific EC 1.3.99.2	fig|6666666.59329.peg.761	fig|6666666.59330.peg.2011	fig|6666666.59331.peg.2121	fig|6666666.59332.peg.2298	fig|6666666.59334.peg.3925	fig|6666666.59335.peg.4969	fig|6666666.59336.peg.3721	fig|6666666.59333.peg.3428	fig|6666666.59338.peg.5144	fig|6666666.59339.peg.5156	fig|6666666.59340.peg.3452	fig|6666666.59337.peg.4280	fig|6666666.59342.peg.717	fig|6666666.71181.peg.911	fig|6666666.59344.peg.65	fig|6666666.59345.peg.1209	fig|6666666.59341.peg.1696	fig|6666666.59347.peg.1986	fig|6666666.59346.peg.5189	fig|6666666.59348.peg.2810	fig|6666666.59349.peg.4970	fig|6666666.59350.peg.1620	fig|6666666.59351.peg.812	fig|6666666.59353.peg.3254	fig|6666666.59355.peg.2123	fig|6666666.59354.peg.1870

9090	Preprotein translocase subunit YajC TC 3.A.5.1.1	fig|6666666.59329.peg.646	fig|6666666.59330.peg.1406	fig|6666666.59331.peg.2298	fig|6666666.59332.peg.1439	fig|6666666.59334.peg.2714	fig|6666666.59335.peg.5094	fig|6666666.59336.peg.5539	fig|6666666.59333.peg.4134	fig|6666666.59338.peg.3404	fig|6666666.59339.peg.6897	fig|6666666.59340.peg.551	fig|6666666.59337.peg.732	fig|6666666.59342.peg.1144	fig|6666666.71181.peg.2733	fig|6666666.59344.peg.1105	fig|6666666.59345.peg.3658	fig|6666666.59341.peg.5337	fig|6666666.59347.peg.4277	fig|6666666.59346.peg.2746	fig|6666666.59348.peg.1564	fig|6666666.59349.peg.1926	fig|6666666.59350.peg.2478	fig|6666666.59351.peg.2334	fig|6666666.59353.peg.784	fig|6666666.59355.peg.920	fig|6666666.59354.peg.6510

6637	Serine acetyltransferase EC 2.3.1.30	fig|6666666.59329.peg.30	fig|6666666.59330.peg.2359	fig|6666666.59331.peg.2508	fig|6666666.59332.peg.1735	fig|6666666.59334.peg.4113	fig|6666666.59335.peg.5013	fig|6666666.59336.peg.1980	fig|6666666.59333.peg.2295	fig|6666666.59338.peg.3747	fig|6666666.59339.peg.6728	fig|6666666.59340.peg.397	fig|6666666.59337.peg.5560	fig|6666666.59342.peg.4256	fig|6666666.71181.peg.1392	fig|6666666.59344.peg.2758	fig|6666666.59345.peg.2988	fig|6666666.59341.peg.5432	fig|6666666.59347.peg.99	fig|6666666.59346.peg.4158	fig|6666666.59348.peg.1809	fig|6666666.59349.peg.258	fig|6666666.59350.peg.601	fig|6666666.59351.peg.380	fig|6666666.59353.peg.3412	fig|6666666.59355.peg.3510	fig|6666666.59354.peg.3486

4709	Fumarate hydratase class II EC 4.2.1.2	fig|6666666.59329.peg.1060	fig|6666666.59330.peg.2139	fig|6666666.59331.peg.3064	fig|6666666.59332.peg.145	fig|6666666.59334.peg.4652	fig|6666666.59335.peg.472	fig|6666666.59336.peg.6485	fig|6666666.59333.peg.5185	fig|6666666.59338.peg.5177	fig|6666666.59339.peg.6958	fig|6666666.59340.peg.3062	fig|6666666.59337.peg.4297	fig|6666666.59342.peg.3032	fig|6666666.71181.peg.6015	fig|6666666.59344.peg.3833	fig|6666666.59345.peg.5137	fig|6666666.59341.peg.5094	fig|6666666.59347.peg.4511	fig|6666666.59346.peg.785	fig|6666666.59348.peg.2451	fig|6666666.59349.peg.1756	fig|6666666.59350.peg.1572	fig|6666666.59351.peg.5290	fig|6666666.59353.peg.1045	fig|6666666.59355.peg.4884	fig|6666666.59354.peg.1614

8932	Shikimate kinase I EC 2.7.1.71	fig|6666666.59329.peg.313	fig|6666666.59330.peg.2635	fig|6666666.59331.peg.6161	fig|6666666.59332.peg.1030	fig|6666666.59334.peg.849	fig|6666666.59335.peg.2485	fig|6666666.59336.peg.5294	fig|6666666.59333.peg.896	fig|6666666.59338.peg.961	fig|6666666.59339.peg.8718	fig|6666666.59340.peg.367	fig|6666666.59337.peg.1701	fig|6666666.59342.peg.2757	fig|6666666.71181.peg.1832	fig|6666666.59344.peg.3062	fig|6666666.59345.peg.5381	fig|6666666.59341.peg.4081	fig|6666666.59347.peg.4944	fig|6666666.59346.peg.6	fig|6666666.59348.peg.1023	fig|6666666.59349.peg.527	fig|6666666.59350.peg.2171	fig|6666666.59351.peg.3850	fig|6666666.59353.peg.2218	fig|6666666.59355.peg.1910	fig|6666666.59354.peg.2406

8005	Trk system potassium uptake protein TrkA	fig|6666666.59329.peg.63	fig|6666666.59330.peg.641	fig|6666666.59331.peg.1921	fig|6666666.59332.peg.225	fig|6666666.59334.peg.1389	fig|6666666.59335.peg.2436	fig|6666666.59336.peg.3768	fig|6666666.59333.peg.1378	fig|6666666.59338.peg.583	fig|6666666.59339.peg.8091	fig|6666666.59340.peg.1064	fig|6666666.59337.peg.3899	fig|6666666.59342.peg.4142	fig|6666666.71181.peg.3433	fig|6666666.59344.peg.4916	fig|6666666.59345.peg.4930	fig|6666666.59341.peg.3886	fig|6666666.59347.peg.4750	fig|6666666.59346.peg.1820	fig|6666666.59348.peg.53	fig|6666666.59349.peg.2815	fig|6666666.59350.peg.5991	fig|6666666.59351.peg.5052	fig|6666666.59353.peg.4617	fig|6666666.59355.peg.4714	fig|6666666.59354.peg.1432

8778	Cytochrome c heme lyase subunit CcmF	fig|6666666.59329.peg.2319	fig|6666666.59330.peg.2313	fig|6666666.59331.peg.6294	fig|6666666.59332.peg.3179	fig|6666666.59334.peg.2180	fig|6666666.59335.peg.367	fig|6666666.59336.peg.4942	fig|6666666.59333.peg.5392	fig|6666666.59338.peg.5382	fig|6666666.59339.peg.3032	fig|6666666.59340.peg.1898	fig|6666666.59337.peg.4224	fig|6666666.59342.peg.2871	fig|6666666.71181.peg.1526	fig|6666666.59344.peg.4982	fig|6666666.59345.peg.5310	fig|6666666.59341.peg.5273	fig|6666666.59347.peg.1861	fig|6666666.59346.peg.5161	fig|6666666.59348.peg.3290	fig|6666666.59349.peg.178	fig|6666666.59350.peg.4236	fig|6666666.59351.peg.2087	fig|6666666.59353.peg.4870	fig|6666666.59355.peg.216	fig|6666666.59354.peg.1188

9016	Thymidylate kinase EC 2.7.4.9	fig|6666666.59329.peg.2502	fig|6666666.59330.peg.3919	fig|6666666.59331.peg.2651	fig|6666666.59332.peg.2354	fig|6666666.59334.peg.5443	fig|6666666.59335.peg.5839	fig|6666666.59336.peg.5860	fig|6666666.59333.peg.2616	fig|6666666.59338.peg.3744	fig|6666666.59339.peg.3184	fig|6666666.59340.peg.497	fig|6666666.59337.peg.2682	fig|6666666.59342.peg.3218	fig|6666666.71181.peg.1628	fig|6666666.59344.peg.5034	fig|6666666.59345.peg.1058	fig|6666666.59341.peg.22	fig|6666666.59347.peg.4090	fig|6666666.59346.peg.112	fig|6666666.59348.peg.4603	fig|6666666.59349.peg.4681	fig|6666666.59350.peg.2224	fig|6666666.59351.peg.2797	fig|6666666.59353.peg.319	fig|6666666.59355.peg.1359	fig|6666666.59354.peg.6239

5495	LSU ribosomal protein L10p P0	fig|6666666.59329.peg.625	fig|6666666.59330.peg.3407	fig|6666666.59331.peg.441	fig|6666666.59332.peg.2888	fig|6666666.59334.peg.2476	fig|6666666.59335.peg.5628	fig|6666666.59336.peg.1682	fig|6666666.59333.peg.4394	fig|6666666.59338.peg.3291	fig|6666666.59339.peg.8843	fig|6666666.59340.peg.1330	fig|6666666.59337.peg.4988	fig|6666666.59342.peg.368	fig|6666666.71181.peg.5016	fig|6666666.59344.peg.5463	fig|6666666.59345.peg.5655	fig|6666666.59341.peg.5593	fig|6666666.59347.peg.5399	fig|6666666.59346.peg.4098	fig|6666666.59348.peg.3990	fig|6666666.59349.peg.1143	fig|6666666.59350.peg.5400	fig|6666666.59351.peg.5241	fig|6666666.59353.peg.4451	fig|6666666.59355.peg.4576	fig|6666666.59354.peg.4535

5634	YgjD Kae1 Qri7 family, required for threonylcarbamoyladenosine t 6 A formation in tRNA	fig|6666666.59329.peg.3080	fig|6666666.59330.peg.3129	fig|6666666.59331.peg.1755	fig|6666666.59332.peg.940	fig|6666666.59334.peg.3132	fig|6666666.59335.peg.1646	fig|6666666.59336.peg.3816	fig|6666666.59333.peg.175	fig|6666666.59338.peg.692	fig|6666666.59339.peg.7908	fig|6666666.59340.peg.2251	fig|6666666.59337.peg.1426	fig|6666666.59342.peg.4698	fig|6666666.71181.peg.1939	fig|6666666.59344.peg.2849	fig|6666666.59345.peg.3846	fig|6666666.59341.peg.3719	fig|6666666.59347.peg.4407	fig|6666666.59346.peg.3107	fig|6666666.59348.peg.5434	fig|6666666.59349.peg.1694	fig|6666666.59350.peg.1752	fig|6666666.59351.peg.5221	fig|6666666.59353.peg.4187	fig|6666666.59355.peg.1690	fig|6666666.59354.peg.884

6294	Cytidylate kinase EC 2.7.4.14	fig|6666666.59329.peg.3386	fig|6666666.59330.peg.1388	fig|6666666.59331.peg.2069	fig|6666666.59332.peg.5810	fig|6666666.59334.peg.1078	fig|6666666.59335.peg.3368	fig|6666666.59336.peg.432	fig|6666666.59333.peg.735	fig|6666666.59338.peg.1337	fig|6666666.59339.peg.3109	None	fig|6666666.59337.peg.2876	fig|6666666.59342.peg.1796	fig|6666666.71181.peg.3166	fig|6666666.59344.peg.2801	fig|6666666.59345.peg.1037	fig|6666666.59341.peg.46	fig|6666666.59347.peg.2408	fig|6666666.59346.peg.4946	fig|6666666.59348.peg.3248	fig|6666666.59349.peg.378	fig|6666666.59350.peg.5704	fig|6666666.59351.peg.941	fig|6666666.59353.peg.1646	fig|6666666.59355.peg.1075	fig|6666666.59354.peg.4422

10619	TolA protein	fig|6666666.59329.peg.4961	fig|6666666.59330.peg.3437	fig|6666666.59331.peg.5486	fig|6666666.59332.peg.5749	fig|6666666.59334.peg.4669	fig|6666666.59335.peg.2509	fig|6666666.59336.peg.2586	fig|6666666.59333.peg.1989	fig|6666666.59338.peg.4118	fig|6666666.59339.peg.398	fig|6666666.59340.peg.1635	fig|6666666.59337.peg.4380	fig|6666666.59342.peg.2247	fig|6666666.71181.peg.5006	fig|6666666.59344.peg.170	fig|6666666.59345.peg.1507	fig|6666666.59341.peg.4884	fig|6666666.59347.peg.4208	fig|6666666.59346.peg.1442	fig|6666666.59348.peg.5114	fig|6666666.59349.peg.3862	None	fig|6666666.59351.peg.658	fig|6666666.59353.peg.2100	fig|6666666.59355.peg.2722	fig|6666666.59354.peg.1597

8017	FKBP-type peptidyl-prolyl cis-trans isomerase SlyD EC 5.2.1.8	fig|6666666.59329.peg.1375	fig|6666666.59330.peg.1462	fig|6666666.59331.peg.2365	fig|6666666.59332.peg.1082	fig|6666666.59334.peg.432	fig|6666666.59335.peg.1863	fig|6666666.59336.peg.4542	fig|6666666.59333.peg.2363	fig|6666666.59338.peg.2588	fig|6666666.59339.peg.8249	None	fig|6666666.59337.peg.135	fig|6666666.59342.peg.6498	fig|6666666.71181.peg.4306	fig|6666666.59344.peg.3124	fig|6666666.59345.peg.5560	fig|6666666.59341.peg.4625	fig|6666666.59347.peg.4970	fig|6666666.59346.peg.239	fig|6666666.59348.peg.293	fig|6666666.59349.peg.1782	fig|6666666.59350.peg.7378	fig|6666666.59351.peg.504	fig|6666666.59353.peg.2267	fig|6666666.59355.peg.1960	fig|6666666.59354.peg.2640

4592	S-adenosylmethionine-dependent methyltransferase Functionally Coupled to the MukBEF Chromosome Partitioning Mechanism	fig|6666666.59329.peg.4985	fig|6666666.59330.peg.4160	fig|6666666.59331.peg.601	fig|6666666.59332.peg.3606	fig|6666666.59334.peg.5063	fig|6666666.59335.peg.4766	fig|6666666.59336.peg.1399	fig|6666666.59333.peg.5040	fig|6666666.59338.peg.4037	fig|6666666.59339.peg.944	None	fig|6666666.59337.peg.1755	fig|6666666.59342.peg.1081	fig|6666666.71181.peg.505	fig|6666666.59344.peg.1724	fig|6666666.59345.peg.891	fig|6666666.59341.peg.5645	fig|6666666.59347.peg.2371	fig|6666666.59346.peg.1346	fig|6666666.59348.peg.5015	fig|6666666.59349.peg.1899	fig|6666666.59350.peg.233	fig|6666666.59351.peg.3599	fig|6666666.59353.peg.1661	fig|6666666.59355.peg.1060	fig|6666666.59354.peg.3967

2301	Putative stomatin prohibitin-family membrane protease subunit YbbK	fig|6666666.59329.peg.4596	fig|6666666.59330.peg.688	fig|6666666.59331.peg.4005	fig|6666666.59332.peg.5155	fig|6666666.59334.peg.2220	fig|6666666.59335.peg.3012	fig|6666666.59336.peg.4726	fig|6666666.59333.peg.2742	fig|6666666.59338.peg.2800	fig|6666666.59339.peg.3377	None	fig|6666666.59337.peg.4171	fig|6666666.59342.peg.98	fig|6666666.71181.peg.3615	fig|6666666.59344.peg.5063	fig|6666666.59345.peg.4381	fig|6666666.59341.peg.4978	fig|6666666.59347.peg.4432	fig|6666666.59346.peg.2370	fig|6666666.59348.peg.3876	fig|6666666.59349.peg.1983	fig|6666666.59350.peg.3393	fig|6666666.59351.peg.1750	fig|6666666.59353.peg.4054	fig|6666666.59355.peg.4130	fig|6666666.59354.peg.2778

10575	Thiamin biosynthesis lipoprotein ApbE	fig|6666666.59329.peg.2136	fig|6666666.59330.peg.4051	fig|6666666.59331.peg.5661	fig|6666666.59332.peg.2208	fig|6666666.59334.peg.887	fig|6666666.59335.peg.5893	fig|6666666.59336.peg.2771	fig|6666666.59333.peg.1804	fig|6666666.59338.peg.269	fig|6666666.59339.peg.5054	fig|6666666.59340.peg.946	fig|6666666.59337.peg.1548	fig|6666666.59342.peg.5121	fig|6666666.71181.peg.1503	fig|6666666.59344.peg.5812	fig|6666666.59345.peg.2231	fig|6666666.59341.peg.2297	fig|6666666.59347.peg.2796	fig|6666666.59346.peg.4251	fig|6666666.59348.peg.2980	fig|6666666.59349.peg.301	None	fig|6666666.59351.peg.3925	fig|6666666.59353.peg.1235	fig|6666666.59355.peg.811	fig|6666666.59354.peg.4977

8913	23S rRNA guanine-N-2- -methyltransferase rlmG EC 2.1.1.-	fig|6666666.59329.peg.3015	fig|6666666.59330.peg.2789	None	fig|6666666.59332.peg.4881	fig|6666666.59334.peg.3862	fig|6666666.59335.peg.117	fig|6666666.59336.peg.2436	fig|6666666.59333.peg.4691	fig|6666666.59338.peg.1999	fig|6666666.59339.peg.4830	fig|6666666.59340.peg.1799	fig|6666666.59337.peg.2377	fig|6666666.59342.peg.2128	fig|6666666.71181.peg.469	fig|6666666.59344.peg.1388	fig|6666666.59345.peg.3737	fig|6666666.59341.peg.5303	fig|6666666.59347.peg.3049	fig|6666666.59346.peg.4034	fig|6666666.59348.peg.4183	fig|6666666.59349.peg.4393	fig|6666666.59350.peg.6253	fig|6666666.59351.peg.3897	fig|6666666.59353.peg.2642	fig|6666666.59355.peg.1673	fig|6666666.59354.peg.4891

8840	Pyridoxamine 5'-phosphate oxidase EC 1.4.3.5	fig|6666666.59329.peg.232	fig|6666666.59330.peg.2286	fig|6666666.59331.peg.2848	fig|6666666.59332.peg.3999	fig|6666666.59334.peg.410	fig|6666666.59335.peg.3852	fig|6666666.59336.peg.5764	fig|6666666.59333.peg.4823	fig|6666666.59338.peg.4378	fig|6666666.59339.peg.8345	fig|6666666.59340.peg.3716	fig|6666666.59337.peg.272	None	fig|6666666.71181.peg.2928	fig|6666666.59344.peg.3490	fig|6666666.59345.peg.5230	fig|6666666.59341.peg.1512	fig|6666666.59347.peg.4672	fig|6666666.59346.peg.3999	fig|6666666.59348.peg.3929	fig|6666666.59349.peg.4221	fig|6666666.59350.peg.6076	fig|6666666.59351.peg.2693	fig|6666666.59353.peg.1391	fig|6666666.59355.peg.2481	fig|6666666.59354.peg.5176

9520	FIG001674 hypothetical protein	fig|6666666.59329.peg.4222	fig|6666666.59330.peg.1584	fig|6666666.59331.peg.4858	fig|6666666.59332.peg.4596	fig|6666666.59334.peg.2569	fig|6666666.59335.peg.1711	fig|6666666.59336.peg.4062	fig|6666666.59333.peg.4011	fig|6666666.59338.peg.1087	fig|6666666.59339.peg.7235	None	fig|6666666.59337.peg.5602	fig|6666666.59342.peg.2108	fig|6666666.71181.peg.1464	fig|6666666.59344.peg.4999	fig|6666666.59345.peg.5296	fig|6666666.59341.peg.845	fig|6666666.59347.peg.5271	fig|6666666.59346.peg.3347	fig|6666666.59348.peg.5018	fig|6666666.59349.peg.2728	fig|6666666.59350.peg.2701	fig|6666666.59351.peg.572	fig|6666666.59353.peg.4379	fig|6666666.59355.peg.4462	fig|6666666.59354.peg.2266

2519	Fructose-1,6-bisphosphatase, type I EC 3.1.3.11	fig|6666666.59329.peg.1778	fig|6666666.59330.peg.839	fig|6666666.59331.peg.6272	fig|6666666.59332.peg.5108	fig|6666666.59334.peg.57	fig|6666666.59335.peg.178	fig|6666666.59336.peg.920	fig|6666666.59333.peg.572	fig|6666666.59338.peg.306	fig|6666666.59339.peg.7728	None	fig|6666666.59337.peg.332	fig|6666666.59342.peg.5991	fig|6666666.71181.peg.2803	fig|6666666.59344.peg.2128	fig|6666666.59345.peg.2482	fig|6666666.59341.peg.5005	fig|6666666.59347.peg.4872	fig|6666666.59346.peg.654	fig|6666666.59348.peg.2843	fig|6666666.59349.peg.4977	fig|6666666.59350.peg.6634	fig|6666666.59351.peg.2269	fig|6666666.59353.peg.3161	fig|6666666.59355.peg.436	fig|6666666.59354.peg.6072

3533	Cytochrome c heme lyase subunit CcmL	fig|6666666.59329.peg.4623	fig|6666666.59330.peg.4680	None	fig|6666666.59332.peg.6222	fig|6666666.59334.peg.2353	fig|6666666.59335.peg.1253	fig|6666666.59336.peg.5504	fig|6666666.59333.peg.3690	fig|6666666.59338.peg.4418	fig|6666666.59339.peg.3034	fig|6666666.59340.peg.1901	fig|6666666.59337.peg.4226	fig|6666666.59342.peg.2828	fig|6666666.71181.peg.1524	fig|6666666.59344.peg.4985	fig|6666666.59345.peg.5308	fig|6666666.59341.peg.5696	fig|6666666.59347.peg.1860	fig|6666666.59346.peg.5157	fig|6666666.59348.peg.4227	fig|6666666.59349.peg.176	fig|6666666.59350.peg.3263	fig|6666666.59351.peg.2090	fig|6666666.59353.peg.4872	fig|6666666.59355.peg.218	fig|6666666.59354.peg.1186

6905	Decarboxylase family protein	fig|6666666.59329.peg.4174	fig|6666666.59330.peg.3896	fig|6666666.59331.peg.5551	fig|6666666.59332.peg.5831	fig|6666666.59334.peg.1983	fig|6666666.59335.peg.340	fig|6666666.59336.peg.3265	fig|6666666.59333.peg.4317	fig|6666666.59338.peg.1649	fig|6666666.59339.peg.7157	fig|6666666.59340.peg.3251	fig|6666666.59337.peg.1120	fig|6666666.59342.peg.6259	fig|6666666.71181.peg.49	fig|6666666.59344.peg.2629	fig|6666666.59345.peg.3522	fig|6666666.59341.peg.5085	fig|6666666.59347.peg.2931	fig|6666666.59346.peg.1729	fig|6666666.59348.peg.2646	fig|6666666.59349.peg.446	None	fig|6666666.59351.peg.1614	fig|6666666.59353.peg.1968	fig|6666666.59355.peg.1438	fig|6666666.59354.peg.2381

152	S-adenosylmethionine tRNA ribosyltransferase-isomerase EC 5.-.-.-	fig|6666666.59329.peg.648	fig|6666666.59330.peg.1403	fig|6666666.59331.peg.3891	fig|6666666.59332.peg.772	fig|6666666.59334.peg.2717	None	fig|6666666.59336.peg.5654	fig|6666666.59333.peg.4136	fig|6666666.59338.peg.3408	fig|6666666.59339.peg.6893	fig|6666666.59340.peg.556	fig|6666666.59337.peg.730	fig|6666666.59342.peg.1147	fig|6666666.71181.peg.2735	fig|6666666.59344.peg.1107	fig|6666666.59345.peg.3660	fig|6666666.59341.peg.5334	fig|6666666.59347.peg.4275	fig|6666666.59346.peg.2748	fig|6666666.59348.peg.1561	fig|6666666.59349.peg.1923	fig|6666666.59350.peg.3657	fig|6666666.59351.peg.2332	fig|6666666.59353.peg.787	fig|6666666.59355.peg.918	fig|6666666.59354.peg.1288

10821	FIG000557 hypothetical protein co-occurring with RecR	fig|6666666.59329.peg.5440	fig|6666666.59330.peg.4122	None	fig|6666666.59332.peg.4655	fig|6666666.59334.peg.2752	fig|6666666.59335.peg.3289	fig|6666666.59336.peg.5783	fig|6666666.59333.peg.2533	fig|6666666.59338.peg.2298	fig|6666666.59339.peg.3066	fig|6666666.59340.peg.3702	fig|6666666.59337.peg.3738	fig|6666666.59342.peg.3337	fig|6666666.71181.peg.3499	fig|6666666.59344.peg.5519	fig|6666666.59345.peg.1192	fig|6666666.59341.peg.2460	fig|6666666.59347.peg.2445	fig|6666666.59346.peg.65	fig|6666666.59348.peg.3117	fig|6666666.59349.peg.4589	fig|6666666.59350.peg.2506	fig|6666666.59351.peg.2755	fig|6666666.59353.peg.4075	fig|6666666.59355.peg.4152	fig|6666666.59354.peg.5012

7232	Methionine repressor MetJ	fig|6666666.59329.peg.4606	fig|6666666.59330.peg.1671	fig|6666666.59331.peg.6095	fig|6666666.59332.peg.1179	fig|6666666.59334.peg.2426	fig|6666666.59335.peg.427	fig|6666666.59336.peg.3891	fig|6666666.59333.peg.3016	fig|6666666.59338.peg.1051	fig|6666666.59339.peg.6377	fig|6666666.59340.peg.1162	fig|6666666.59337.peg.1674	fig|6666666.59342.peg.3918	fig|6666666.71181.peg.3888	fig|6666666.59344.peg.1006	fig|6666666.59345.peg.5405	fig|6666666.59341.peg.5322	fig|6666666.59347.peg.4921	fig|6666666.59346.peg.1005	fig|6666666.59348.peg.4560	fig|6666666.59349.peg.517	None	fig|6666666.59351.peg.3840	fig|6666666.59353.peg.2534	fig|6666666.59355.peg.2667	fig|6666666.59354.peg.927

5269	Thiamin-phosphate pyrophosphorylase EC 2.5.1.3	fig|6666666.59329.peg.3206	fig|6666666.59330.peg.4730	fig|6666666.59331.peg.2626	fig|6666666.59332.peg.2524	fig|6666666.59334.peg.903	fig|6666666.59335.peg.5	fig|6666666.59336.peg.6428	fig|6666666.59333.peg.4005	fig|6666666.59338.peg.472	fig|6666666.59339.peg.7528	fig|6666666.59340.peg.1302	fig|6666666.59337.peg.2553	fig|6666666.59342.peg.2009	fig|6666666.71181.peg.3358	fig|6666666.59344.peg.5689	fig|6666666.59345.peg.5545	fig|6666666.59341.peg.320	fig|6666666.59347.peg.2366	fig|6666666.59346.peg.934	fig|6666666.59348.peg.1091	fig|6666666.59349.peg.4743	fig|6666666.59350.peg.5867	fig|6666666.59351.peg.3887	fig|6666666.59353.peg.2743	fig|6666666.59355.peg.1739	None

11137	Thymidine kinase EC 2.7.1.21	fig|6666666.59329.peg.4110	fig|6666666.59330.peg.1515	fig|6666666.59331.peg.1166	fig|6666666.59332.peg.4703	fig|6666666.59334.peg.4842	fig|6666666.59335.peg.2939	fig|6666666.59336.peg.2602	fig|6666666.59333.peg.4965	fig|6666666.59338.peg.1125	fig|6666666.59339.peg.285	fig|6666666.59340.peg.1481	fig|6666666.59337.peg.2302	None	fig|6666666.71181.peg.2763	fig|6666666.59344.peg.418	fig|6666666.59345.peg.1295	fig|6666666.59341.peg.3624	fig|6666666.59347.peg.2316	fig|6666666.59346.peg.4357	fig|6666666.59348.peg.1666	fig|6666666.59349.peg.2960	fig|6666666.59350.peg.2572	fig|6666666.59351.peg.3801	fig|6666666.59353.peg.1745	fig|6666666.59355.peg.1267	fig|6666666.59354.peg.3093

11136	rRNA small subunit methyltransferase H	fig|6666666.59329.peg.2030	fig|6666666.59330.peg.1268	fig|6666666.59331.peg.2000	fig|6666666.59332.peg.5058	fig|6666666.59334.peg.3667	fig|6666666.59335.peg.845	fig|6666666.59336.peg.710	fig|6666666.59333.peg.3484	fig|6666666.59338.peg.536	fig|6666666.59339.peg.4958	fig|6666666.59340.peg.619	fig|6666666.59337.peg.40	fig|6666666.59342.peg.3872	None	fig|6666666.59344.peg.1490	fig|6666666.59345.peg.3802	fig|6666666.59341.peg.1358	fig|6666666.59347.peg.3992	fig|6666666.59346.peg.1229	fig|6666666.59348.peg.2586	fig|6666666.59349.peg.2976	fig|6666666.59350.peg.3519	fig|6666666.59351.peg.200	fig|6666666.59353.peg.129	fig|6666666.59355.peg.740	fig|6666666.59354.peg.3668

7936	Flavoprotein MioC	fig|6666666.59329.peg.1741	fig|6666666.59330.peg.1112	fig|6666666.59331.peg.2811	fig|6666666.59332.peg.92	fig|6666666.59334.peg.3781	fig|6666666.59335.peg.786	fig|6666666.59336.peg.21	fig|6666666.59333.peg.37	fig|6666666.59338.peg.3680	fig|6666666.59339.peg.8024	fig|6666666.59340.peg.136	fig|6666666.59337.peg.884	fig|6666666.59342.peg.1190	fig|6666666.71181.peg.588	fig|6666666.59344.peg.4260	fig|6666666.59345.peg.4971	fig|6666666.59341.peg.1053	fig|6666666.59347.peg.4789	fig|6666666.59346.peg.1182	fig|6666666.59348.peg.306	fig|6666666.59349.peg.1023	None	fig|6666666.59351.peg.5365	fig|6666666.59353.peg.3092	fig|6666666.59355.peg.2256	fig|6666666.59354.peg.1142

3024	Protein of unknown function DUF541	fig|6666666.59329.peg.245	fig|6666666.59330.peg.364	fig|6666666.59331.peg.1655	fig|6666666.59332.peg.536	fig|6666666.59334.peg.1054	fig|6666666.59335.peg.2315	fig|6666666.59336.peg.1194	fig|6666666.59333.peg.4185	fig|6666666.59338.peg.2667	fig|6666666.59339.peg.7857	None	fig|6666666.59337.peg.545	fig|6666666.59342.peg.2805	fig|6666666.71181.peg.3136	fig|6666666.59344.peg.4732	fig|6666666.59345.peg.5034	fig|6666666.59341.peg.3928	fig|6666666.59347.peg.3198	fig|6666666.59346.peg.313	fig|6666666.59348.peg.203	fig|6666666.59349.peg.1604	fig|6666666.59350.peg.5906	fig|6666666.59351.peg.1030	fig|6666666.59353.peg.4430	fig|6666666.59355.peg.4510	fig|6666666.59354.peg.2333

237	Protein of unknown function Smg	fig|6666666.59329.peg.70	fig|6666666.59330.peg.649	fig|6666666.59331.peg.4163	fig|6666666.59332.peg.232	fig|6666666.59334.peg.1511	fig|6666666.59335.peg.5004	fig|6666666.59336.peg.1988	fig|6666666.59333.peg.1369	fig|6666666.59338.peg.2064	fig|6666666.59339.peg.8097	fig|6666666.59340.peg.3347	fig|6666666.59337.peg.3905	fig|6666666.59342.peg.4246	fig|6666666.71181.peg.3428	fig|6666666.59344.peg.4909	fig|6666666.59345.peg.4923	fig|6666666.59341.peg.3879	fig|6666666.59347.peg.4744	fig|6666666.59346.peg.1828	fig|6666666.59348.peg.5270	fig|6666666.59349.peg.2821	None	fig|6666666.59351.peg.5060	fig|6666666.59353.peg.4624	fig|6666666.59355.peg.4707	fig|6666666.59354.peg.1426

9564	Adenylate cyclase EC 4.6.1.1	fig|6666666.59329.peg.385	fig|6666666.59330.peg.944	fig|6666666.59331.peg.3804	fig|6666666.59332.peg.3852	fig|6666666.59334.peg.539	fig|6666666.59335.peg.1512	fig|6666666.59336.peg.3438	fig|6666666.59333.peg.194	fig|6666666.59338.peg.210	fig|6666666.59339.peg.7402	fig|6666666.59340.peg.1193	fig|6666666.59337.peg.10	fig|6666666.59342.peg.1377	fig|6666666.71181.peg.3017	fig|6666666.59344.peg.4464	fig|6666666.59345.peg.3831	fig|6666666.59341.peg.1559	fig|6666666.59347.peg.3960	fig|6666666.59346.peg.1621	fig|6666666.59348.peg.3353	fig|6666666.59349.peg.3513	None	fig|6666666.59351.peg.3635	fig|6666666.59353.peg.4179	fig|6666666.59355.peg.1698	fig|6666666.59354.peg.3230

2889	Glucosamine-6-phosphate deaminase EC 3.5.99.6	fig|6666666.59329.peg.5495	fig|6666666.59330.peg.1477	fig|6666666.59331.peg.2260	fig|6666666.59332.peg.2457	fig|6666666.59334.peg.917	fig|6666666.59335.peg.5803	fig|6666666.59336.peg.3194	fig|6666666.59333.peg.1221	fig|6666666.59338.peg.4108	fig|6666666.59339.peg.1933	fig|6666666.59340.peg.2537	fig|6666666.59337.peg.199	fig|6666666.59342.peg.4003	fig|6666666.71181.peg.3199	fig|6666666.59344.peg.4525	fig|6666666.59345.peg.4528	fig|6666666.59341.peg.6016	fig|6666666.59347.peg.346	fig|6666666.59346.peg.4434	fig|6666666.59348.peg.3499	fig|6666666.59349.peg.2661	None	fig|6666666.59351.peg.5154	fig|6666666.59353.peg.2047	fig|6666666.59355.peg.4454	fig|6666666.59354.peg.2873

11169	tRNA pseudouridine synthase B EC 4.2.1.70	fig|6666666.59329.peg.1895	fig|6666666.59330.peg.104	fig|6666666.59331.peg.2110	fig|6666666.59332.peg.3550	fig|6666666.59334.peg.776	fig|6666666.59335.peg.2098	None	fig|6666666.59333.peg.2323	fig|6666666.59338.peg.3434	fig|6666666.59339.peg.4898	fig|6666666.59340.peg.954	fig|6666666.59337.peg.686	fig|6666666.59342.peg.380	fig|6666666.71181.peg.4100	fig|6666666.59344.peg.2097	fig|6666666.59345.peg.2354	fig|6666666.59341.peg.2442	fig|6666666.59347.peg.3002	fig|6666666.59346.peg.301	fig|6666666.59348.peg.2156	fig|6666666.59349.peg.1436	fig|6666666.59350.peg.923	fig|6666666.59351.peg.1387	fig|6666666.59353.peg.3130	fig|6666666.59355.peg.466	fig|6666666.59354.peg.5107

11247	RNA polymerase sigma factor for flagellar operon	fig|6666666.59329.peg.204	fig|6666666.59330.peg.2910	fig|6666666.59331.peg.2929	fig|6666666.59332.peg.2449	None	fig|6666666.59335.peg.914	fig|6666666.59336.peg.985	fig|6666666.59333.peg.4088	fig|6666666.59338.peg.2387	fig|6666666.59339.peg.88	fig|6666666.59340.peg.1867	fig|6666666.59337.peg.3211	fig|6666666.59342.peg.2735	fig|6666666.71181.peg.4038	fig|6666666.59344.peg.5208	fig|6666666.59345.peg.2879	fig|6666666.59341.peg.3497	fig|6666666.59347.peg.4609	fig|6666666.59346.peg.4415	fig|6666666.59348.peg.2411	fig|6666666.59349.peg.194	fig|6666666.59350.peg.6480	fig|6666666.59351.peg.2065	fig|6666666.59353.peg.4573	fig|6666666.59355.peg.3837	fig|6666666.59354.peg.685

1577	ATP synthase C chain EC 3.6.3.14	fig|6666666.59329.peg.3995	fig|6666666.59330.peg.606	fig|6666666.59331.peg.401	fig|6666666.59332.peg.100	fig|6666666.59334.peg.25	fig|6666666.59335.peg.793	fig|6666666.59336.peg.29	fig|6666666.59333.peg.28	fig|6666666.59338.peg.4016	fig|6666666.59339.peg.8013	fig|6666666.59340.peg.2444	fig|6666666.59337.peg.2084	fig|6666666.59342.peg.1197	fig|6666666.71181.peg.593	fig|6666666.59344.peg.4266	fig|6666666.59345.peg.3324	fig|6666666.59341.peg.3688	fig|6666666.59347.peg.4782	fig|6666666.59346.peg.1404	fig|6666666.59348.peg.333	fig|6666666.59349.peg.1031	None	fig|6666666.59351.peg.5358	fig|6666666.59353.peg.3087	fig|6666666.59355.peg.2261	fig|6666666.59354.peg.1153

9925	SOS-response repressor and protease LexA EC 3.4.21.88	fig|6666666.59329.peg.779	fig|6666666.59330.peg.1576	fig|6666666.59331.peg.1045	fig|6666666.59332.peg.564	fig|6666666.59334.peg.1470	fig|6666666.59335.peg.4043	fig|6666666.59336.peg.1367	fig|6666666.59333.peg.1959	fig|6666666.59338.peg.5775	fig|6666666.59339.peg.8583	fig|6666666.59340.peg.1385	fig|6666666.59337.peg.1659	None	fig|6666666.71181.peg.5546	fig|6666666.59344.peg.2740	fig|6666666.59345.peg.5627	fig|6666666.59341.peg.4287	fig|6666666.59347.peg.5045	fig|6666666.59346.peg.1647	fig|6666666.59348.peg.1071	fig|6666666.59349.peg.3511	fig|6666666.59350.peg.2781	fig|6666666.59351.peg.4390	fig|6666666.59353.peg.2999	fig|6666666.59355.peg.5828	fig|6666666.59354.peg.5143

10329	FMN-dependent NADH-azoreductase	fig|6666666.59329.peg.1968	fig|6666666.59330.peg.1950	fig|6666666.59331.peg.4109	fig|6666666.59332.peg.4443	fig|6666666.59334.peg.4919	fig|6666666.59335.peg.735	fig|6666666.59336.peg.2279	fig|6666666.59333.peg.5271	fig|6666666.59338.peg.370	fig|6666666.59339.peg.5619	None	fig|6666666.59337.peg.2650	fig|6666666.59342.peg.3377	fig|6666666.71181.peg.3218	fig|6666666.59344.peg.4502	fig|6666666.59345.peg.707	fig|6666666.59341.peg.2947	fig|6666666.59347.peg.1538	fig|6666666.59346.peg.3741	fig|6666666.59348.peg.1850	fig|6666666.59349.peg.2753	fig|6666666.59350.peg.7678	fig|6666666.59351.peg.2948	fig|6666666.59353.peg.1869	fig|6666666.59355.peg.4435	fig|6666666.59354.peg.5510

635	Flagellar biosynthesis protein FlhA	fig|6666666.59329.peg.2885	fig|6666666.59330.peg.2900	fig|6666666.59331.peg.5512	fig|6666666.59332.peg.2446	None	fig|6666666.59335.peg.4959	fig|6666666.59336.peg.4347	fig|6666666.59333.peg.2864	fig|6666666.59338.peg.5295	fig|6666666.59339.peg.3006	fig|6666666.59340.peg.2688	fig|6666666.59337.peg.3220	fig|6666666.59342.peg.3198	fig|6666666.71181.peg.2238	fig|6666666.59344.peg.3025	fig|6666666.59345.peg.1457	fig|6666666.59341.peg.670	fig|6666666.59347.peg.2677	fig|6666666.59346.peg.4412	fig|6666666.59348.peg.1778	fig|6666666.59349.peg.197	fig|6666666.59350.peg.5249	fig|6666666.59351.peg.2061	fig|6666666.59353.peg.3535	fig|6666666.59355.peg.3640	fig|6666666.59354.peg.681

8264	SSU ribosomal protein S4p S9e	fig|6666666.59329.peg.732	fig|6666666.59330.peg.575	fig|6666666.59331.peg.28	fig|6666666.59332.peg.5646	fig|6666666.59334.peg.3041	fig|6666666.59335.peg.4753	fig|6666666.59336.peg.165	fig|6666666.59333.peg.387	fig|6666666.59338.peg.4739	fig|6666666.59339.peg.8289	None	fig|6666666.59337.peg.368	fig|6666666.59342.peg.325	fig|6666666.71181.peg.3440	fig|6666666.59344.peg.4923	fig|6666666.59345.peg.2515	fig|6666666.59341.peg.775	fig|6666666.59347.peg.4844	fig|6666666.59346.peg.870	fig|6666666.59348.peg.434	fig|6666666.59349.peg.3290	fig|6666666.59350.peg.8201	fig|6666666.59351.peg.4225	fig|6666666.59353.peg.4610	fig|6666666.59355.peg.4721	fig|6666666.59354.peg.3247

9155	Pirin-related protein	fig|6666666.59329.peg.4410	fig|6666666.59330.peg.4086	fig|6666666.59331.peg.3727	fig|6666666.59332.peg.2479	fig|6666666.59334.peg.2722	fig|6666666.59335.peg.1398	fig|6666666.59336.peg.3247	fig|6666666.59333.peg.3288	fig|6666666.59338.peg.3346	fig|6666666.59339.peg.5938	None	fig|6666666.59337.peg.2137	fig|6666666.59342.peg.5064	fig|6666666.71181.peg.2208	fig|6666666.59344.peg.3579	fig|6666666.59345.peg.3951	fig|6666666.59341.peg.2145	fig|6666666.59347.peg.604	fig|6666666.59346.peg.2965	fig|6666666.59348.peg.3255	fig|6666666.59349.peg.4049	fig|6666666.59350.peg.2056	fig|6666666.59351.peg.1862	fig|6666666.59353.peg.261	fig|6666666.59355.peg.1300	fig|6666666.59354.peg.2728

2760	FIG138056 a glutathione-dependent thiol reductase	fig|6666666.59329.peg.6430	fig|6666666.59330.peg.4475	None	fig|6666666.59332.peg.3084	fig|6666666.59334.peg.3086	fig|6666666.59335.peg.356	fig|6666666.59336.peg.2162	fig|6666666.59333.peg.3551	fig|6666666.59338.peg.5356	fig|6666666.59339.peg.7138	fig|6666666.59340.peg.639	fig|6666666.59337.peg.5644	fig|6666666.59342.peg.2566	fig|6666666.71181.peg.4368	fig|6666666.59344.peg.5669	fig|6666666.59345.peg.2148	fig|6666666.59341.peg.1682	fig|6666666.59347.peg.2844	fig|6666666.59346.peg.3543	fig|6666666.59348.peg.2635	fig|6666666.59349.peg.4797	fig|6666666.59350.peg.3667	fig|6666666.59351.peg.2817	fig|6666666.59353.peg.4911	fig|6666666.59355.peg.4981	fig|6666666.59354.peg.4258

3939	FIG136845 Rhodanese-related sulfurtransferase	fig|6666666.59329.peg.34	fig|6666666.59330.peg.142	fig|6666666.59331.peg.3447	fig|6666666.59332.peg.3272	fig|6666666.59334.peg.968	fig|6666666.59335.peg.445	fig|6666666.59336.peg.5924	fig|6666666.59333.peg.2291	fig|6666666.59338.peg.1162	fig|6666666.59339.peg.6413	fig|6666666.59340.peg.3455	fig|6666666.59337.peg.4996	fig|6666666.59342.peg.3844	fig|6666666.71181.peg.4023	fig|6666666.59344.peg.971	fig|6666666.59345.peg.5095	fig|6666666.59341.peg.184	fig|6666666.59347.peg.4542	fig|6666666.59346.peg.4161	fig|6666666.59348.peg.134	fig|6666666.59349.peg.2487	None	fig|6666666.59351.peg.373	fig|6666666.59353.peg.2568	fig|6666666.59355.peg.2634	fig|6666666.59354.peg.64

6711	Glyoxalase family protein	fig|6666666.59329.peg.4947	None	fig|6666666.59331.peg.6123	fig|6666666.59332.peg.3846	fig|6666666.59334.peg.2172	fig|6666666.59335.peg.5387	fig|6666666.59336.peg.4069	fig|6666666.59333.peg.5013	fig|6666666.59338.peg.2106	fig|6666666.59339.peg.8466	fig|6666666.59340.peg.357	fig|6666666.59337.peg.3233	fig|6666666.59342.peg.6138	fig|6666666.71181.peg.3569	fig|6666666.59344.peg.3958	fig|6666666.59345.peg.2084	fig|6666666.59341.peg.3182	fig|6666666.59347.peg.3784	fig|6666666.59346.peg.1510	fig|6666666.59348.peg.1422	fig|6666666.59349.peg.615	fig|6666666.59350.peg.2833	fig|6666666.59351.peg.3290	fig|6666666.59353.peg.4390	fig|6666666.59355.peg.3064	fig|6666666.59354.peg.4409

4434	Phospho-N-acetylmuramoyl-pentapeptide-transferase EC 2.7.8.13	fig|6666666.59329.peg.863	fig|6666666.59330.peg.1334	fig|6666666.59331.peg.1116	fig|6666666.59332.peg.1901	fig|6666666.59334.peg.2905	fig|6666666.59335.peg.839	fig|6666666.59336.peg.1536	fig|6666666.59333.peg.4367	fig|6666666.59338.peg.258	fig|6666666.59339.peg.4965	fig|6666666.59340.peg.171	fig|6666666.59337.peg.425	fig|6666666.59342.peg.3696	fig|6666666.71181.peg.6315	fig|6666666.59344.peg.2461	fig|6666666.59345.peg.3796	fig|6666666.59341.peg.5948	fig|6666666.59347.peg.3997	fig|6666666.59346.peg.992	fig|6666666.59348.peg.5298	fig|6666666.59349.peg.2982	None	fig|6666666.59351.peg.230	fig|6666666.59353.peg.135	fig|6666666.59355.peg.745	fig|6666666.59354.peg.4495

10472	FIG00746831 hypothetical protein	None	fig|6666666.59330.peg.1014	fig|6666666.59331.peg.4099	fig|6666666.59332.peg.877	fig|6666666.59334.peg.925	fig|6666666.59335.peg.3356	fig|6666666.59336.peg.4553	fig|6666666.59333.peg.2670	fig|6666666.59338.peg.5099	fig|6666666.59339.peg.1538	fig|6666666.59340.peg.579	fig|6666666.59337.peg.3156	fig|6666666.59342.peg.782	fig|6666666.71181.peg.3834	fig|6666666.59344.peg.848	fig|6666666.59345.peg.3263	fig|6666666.59341.peg.4224	fig|6666666.59347.peg.1776	fig|6666666.59346.peg.3402	fig|6666666.59348.peg.4402	fig|6666666.59349.peg.4759	fig|6666666.59350.peg.459	fig|6666666.59351.peg.3148	fig|6666666.59353.peg.1267	fig|6666666.59355.peg.3316	fig|6666666.59354.peg.4706

7108	Heat shock protein 60 family chaperone GroEL	fig|6666666.59329.peg.3148	fig|6666666.59330.peg.162	None	fig|6666666.59332.peg.2922	fig|6666666.59334.peg.2270	fig|6666666.59335.peg.420	fig|6666666.59336.peg.251	fig|6666666.59333.peg.425	fig|6666666.59338.peg.3734	fig|6666666.59339.peg.6367	fig|6666666.59340.peg.3371	fig|6666666.59337.peg.4332	fig|6666666.59342.peg.3029	fig|6666666.71181.peg.1678	fig|6666666.59344.peg.614	fig|6666666.59345.peg.5114	fig|6666666.59341.peg.178	fig|6666666.59347.peg.4550	fig|6666666.59346.peg.985	fig|6666666.59348.peg.3197	fig|6666666.59349.peg.2368	fig|6666666.59350.peg.8222	fig|6666666.59351.peg.113	fig|6666666.59353.peg.1048	fig|6666666.59355.peg.3571	fig|6666666.59354.peg.3256

11200	Ferric iron ABC transporter, iron-binding protein	fig|6666666.59329.peg.2983	fig|6666666.59330.peg.1769	None	fig|6666666.59332.peg.5401	fig|6666666.59334.peg.5331	fig|6666666.59335.peg.1528	fig|6666666.59336.peg.561	fig|6666666.59333.peg.328	fig|6666666.59338.peg.3529	fig|6666666.59339.peg.4795	fig|6666666.59340.peg.970	fig|6666666.59337.peg.2250	fig|6666666.59342.peg.5014	fig|6666666.71181.peg.4165	fig|6666666.59344.peg.3270	fig|6666666.59345.peg.2394	fig|6666666.59341.peg.5540	fig|6666666.59347.peg.2604	fig|6666666.59346.peg.753	fig|6666666.59348.peg.2600	fig|6666666.59349.peg.4577	fig|6666666.59350.peg.4274	fig|6666666.59351.peg.3964	fig|6666666.59353.peg.4419	fig|6666666.59355.peg.3719	fig|6666666.59354.peg.371

879	Chorismate synthase EC 4.2.3.5	fig|6666666.59329.peg.2416	fig|6666666.59330.peg.1586	fig|6666666.59331.peg.1893	fig|6666666.59332.peg.1949	fig|6666666.59334.peg.1707	fig|6666666.59335.peg.5846	fig|6666666.59336.peg.5035	fig|6666666.59333.peg.1966	fig|6666666.59338.peg.1089	fig|6666666.59339.peg.7074	fig|6666666.59340.peg.3462	fig|6666666.59337.peg.4845	fig|6666666.59342.peg.2106	fig|6666666.71181.peg.1511	fig|6666666.59344.peg.5001	fig|6666666.59345.peg.5294	fig|6666666.59341.peg.842	fig|6666666.59347.peg.5112	fig|6666666.59346.peg.2653	None	fig|6666666.59349.peg.2730	fig|6666666.59350.peg.5538	fig|6666666.59351.peg.574	fig|6666666.59353.peg.5018	fig|6666666.59355.peg.230	fig|6666666.59354.peg.123

2744	D-alanine--D-alanine ligase EC 6.3.2.4	fig|6666666.59329.peg.5853	fig|6666666.59330.peg.4342	fig|6666666.59331.peg.3829	fig|6666666.59332.peg.5331	fig|6666666.59334.peg.2268	fig|6666666.59335.peg.571	fig|6666666.59336.peg.2247	fig|6666666.59333.peg.4298	fig|6666666.59338.peg.3816	fig|6666666.59339.peg.2928	None	fig|6666666.59337.peg.3027	fig|6666666.59342.peg.2285	fig|6666666.71181.peg.1978	fig|6666666.59344.peg.2289	fig|6666666.59345.peg.82	fig|6666666.59341.peg.5839	fig|6666666.59347.peg.964	fig|6666666.59346.peg.1888	fig|6666666.59348.peg.3864	fig|6666666.59349.peg.2987	fig|6666666.59350.peg.1043	fig|6666666.59351.peg.211	fig|6666666.59353.peg.140	fig|6666666.59355.peg.750	fig|6666666.59354.peg.6124

11197	3-oxoacyl-[acyl-carrier-protein] synthase, KASII EC 2.3.1.41	fig|6666666.59329.peg.4184	fig|6666666.59330.peg.3626	fig|6666666.59331.peg.1748	fig|6666666.59332.peg.3476	fig|6666666.59334.peg.4783	fig|6666666.59335.peg.5375	fig|6666666.59336.peg.3338	fig|6666666.59333.peg.1586	fig|6666666.59338.peg.3741	fig|6666666.59339.peg.3180	None	fig|6666666.59337.peg.2685	fig|6666666.59342.peg.1380	fig|6666666.71181.peg.1625	fig|6666666.59344.peg.5038	fig|6666666.59345.peg.1061	fig|6666666.59341.peg.18	fig|6666666.59347.peg.4087	fig|6666666.59346.peg.109	fig|6666666.59348.peg.1239	fig|6666666.59349.peg.3913	fig|6666666.59350.peg.584	fig|6666666.59351.peg.674	fig|6666666.59353.peg.316	fig|6666666.59355.peg.1356	fig|6666666.59354.peg.2160

1093	Predicted endonuclease distantly related to archaeal Holliday junction resolvase	fig|6666666.59329.peg.3487	fig|6666666.59330.peg.971	None	fig|6666666.59332.peg.641	fig|6666666.59334.peg.2332	fig|6666666.59335.peg.4931	fig|6666666.59336.peg.713	fig|6666666.59333.peg.611	fig|6666666.59338.peg.540	fig|6666666.59339.peg.4951	fig|6666666.59340.peg.1422	fig|6666666.59337.peg.36	fig|6666666.59342.peg.5571	fig|6666666.71181.peg.4572	fig|6666666.59344.peg.1844	fig|6666666.59345.peg.3805	fig|6666666.59341.peg.1362	fig|6666666.59347.peg.3989	fig|6666666.59346.peg.2329	fig|6666666.59348.peg.4625	fig|6666666.59349.peg.48	fig|6666666.59350.peg.2806	fig|6666666.59351.peg.3469	fig|6666666.59353.peg.2375	fig|6666666.59355.peg.1647	fig|6666666.59354.peg.367

1327	3-oxoacyl-[acyl-carrier-protein] synthase, KASI EC 2.3.1.41	fig|6666666.59329.peg.4401	fig|6666666.59330.peg.1592	fig|6666666.59331.peg.1351	fig|6666666.59332.peg.2180	fig|6666666.59334.peg.1834	fig|6666666.59335.peg.3933	fig|6666666.59336.peg.6169	fig|6666666.59333.peg.1973	fig|6666666.59338.peg.1852	fig|6666666.59339.peg.7055	None	fig|6666666.59337.peg.4838	fig|6666666.59342.peg.2133	fig|6666666.71181.peg.1505	fig|6666666.59344.peg.5810	fig|6666666.59345.peg.5697	fig|6666666.59341.peg.2774	fig|6666666.59347.peg.2461	fig|6666666.59346.peg.2659	fig|6666666.59348.peg.1236	fig|6666666.59349.peg.2637	fig|6666666.59350.peg.558	fig|6666666.59351.peg.4912	fig|6666666.59353.peg.1237	fig|6666666.59355.peg.238	fig|6666666.59354.peg.4676

4067	Peptide deformylase EC 3.5.1.88	fig|6666666.59329.peg.66	fig|6666666.59330.peg.646	fig|6666666.59331.peg.4355	fig|6666666.59332.peg.229	fig|6666666.59334.peg.3654	fig|6666666.59335.peg.2432	None	fig|6666666.59333.peg.3733	fig|6666666.59338.peg.2933	fig|6666666.59339.peg.8095	fig|6666666.59340.peg.2077	fig|6666666.59337.peg.3902	fig|6666666.59342.peg.3132	fig|6666666.71181.peg.3430	fig|6666666.59344.peg.4911	fig|6666666.59345.peg.4927	fig|6666666.59341.peg.3883	fig|6666666.59347.peg.4747	fig|6666666.59346.peg.1824	fig|6666666.59348.peg.5290	fig|6666666.59349.peg.2818	fig|6666666.59350.peg.6494	fig|6666666.59351.peg.5056	fig|6666666.59353.peg.4622	fig|6666666.59355.peg.4710	fig|6666666.59354.peg.1429

10663	3-dehydroquinate dehydratase II EC 4.2.1.10	fig|6666666.59329.peg.1177	fig|6666666.59330.peg.191	fig|6666666.59331.peg.1712	fig|6666666.59332.peg.513	fig|6666666.59334.peg.45	fig|6666666.59335.peg.2624	fig|6666666.59336.peg.2276	fig|6666666.59333.peg.2646	fig|6666666.59338.peg.3729	fig|6666666.59339.peg.6443	fig|6666666.59340.peg.458	fig|6666666.59337.peg.4291	None	fig|6666666.71181.peg.3868	fig|6666666.59344.peg.5337	fig|6666666.59345.peg.5143	fig|6666666.59341.peg.1178	fig|6666666.59347.peg.4497	fig|6666666.59346.peg.4315	fig|6666666.59348.peg.1813	fig|6666666.59349.peg.2363	fig|6666666.59350.peg.2546	fig|6666666.59351.peg.108	fig|6666666.59353.peg.1253	fig|6666666.59355.peg.4817	fig|6666666.59354.peg.3263

9441	Ribonuclease BN EC 3.1.-.-	fig|6666666.59329.peg.353	fig|6666666.59330.peg.407	fig|6666666.59331.peg.4744	fig|6666666.59332.peg.2112	fig|6666666.59334.peg.3234	fig|6666666.59335.peg.731	fig|6666666.59336.peg.439	fig|6666666.59333.peg.492	fig|6666666.59338.peg.5000	fig|6666666.59339.peg.6181	fig|6666666.59340.peg.605	fig|6666666.59337.peg.992	fig|6666666.59342.peg.5959	fig|6666666.71181.peg.2596	fig|6666666.59344.peg.910	fig|6666666.59345.peg.2686	fig|6666666.59341.peg.5316	fig|6666666.59347.peg.4455	None	fig|6666666.59348.peg.678	fig|6666666.59349.peg.1111	fig|6666666.59350.peg.4819	fig|6666666.59351.peg.64	fig|6666666.59353.peg.2619	fig|6666666.59355.peg.2585	fig|6666666.59354.peg.5035

451	Dihydroneopterin aldolase EC 4.1.2.25	fig|6666666.59329.peg.687	fig|6666666.59330.peg.3126	fig|6666666.59331.peg.2658	fig|6666666.59332.peg.1509	fig|6666666.59334.peg.3129	fig|6666666.59335.peg.1651	fig|6666666.59336.peg.5410	fig|6666666.59333.peg.179	fig|6666666.59338.peg.797	fig|6666666.59339.peg.7912	None	fig|6666666.59337.peg.1429	fig|6666666.59342.peg.4519	fig|6666666.71181.peg.3012	fig|6666666.59344.peg.4470	fig|6666666.59345.peg.3843	fig|6666666.59341.peg.4831	fig|6666666.59347.peg.3133	fig|6666666.59346.peg.3110	fig|6666666.59348.peg.3460	fig|6666666.59349.peg.1692	fig|6666666.59350.peg.6128	fig|6666666.59351.peg.5223	fig|6666666.59353.peg.4185	fig|6666666.59355.peg.1692	fig|6666666.59354.peg.5368

4004	Acyl-CoA thioesterase II EC 3.1.2.-	fig|6666666.59329.peg.463	fig|6666666.59330.peg.1831	fig|6666666.59331.peg.4591	fig|6666666.59332.peg.3481	fig|6666666.59334.peg.4644	fig|6666666.59335.peg.876	fig|6666666.59336.peg.1952	fig|6666666.59333.peg.4448	fig|6666666.59338.peg.2060	fig|6666666.59339.peg.3264	fig|6666666.59340.peg.1674	fig|6666666.59337.peg.1061	None	fig|6666666.71181.peg.2068	fig|6666666.59344.peg.1047	fig|6666666.59345.peg.1656	fig|6666666.59341.peg.4700	fig|6666666.59347.peg.2555	fig|6666666.59346.peg.2084	fig|6666666.59348.peg.4378	fig|6666666.59349.peg.2275	fig|6666666.59350.peg.3203	fig|6666666.59351.peg.4822	fig|6666666.59353.peg.736	fig|6666666.59355.peg.969	fig|6666666.59354.peg.4109

11413	Protein export cytoplasm chaperone protein SecB, maintains protein to be exported in unfolded state	fig|6666666.59329.peg.33	fig|6666666.59330.peg.143	fig|6666666.59331.peg.3446	fig|6666666.59332.peg.3273	None	fig|6666666.59335.peg.446	fig|6666666.59336.peg.5925	fig|6666666.59333.peg.2292	fig|6666666.59338.peg.1161	fig|6666666.59339.peg.6414	fig|6666666.59340.peg.2861	fig|6666666.59337.peg.4995	fig|6666666.59342.peg.3845	fig|6666666.71181.peg.4021	fig|6666666.59344.peg.973	fig|6666666.59345.peg.5096	fig|6666666.59341.peg.183	fig|6666666.59347.peg.4543	fig|6666666.59346.peg.4160	fig|6666666.59348.peg.135	fig|6666666.59349.peg.2489	fig|6666666.59350.peg.2705	fig|6666666.59351.peg.375	fig|6666666.59353.peg.2566	fig|6666666.59355.peg.2636	fig|6666666.59354.peg.66

6315	Phosphoglycerate kinase EC 2.7.2.3	fig|6666666.59329.peg.253	fig|6666666.59330.peg.357	fig|6666666.59331.peg.1205	fig|6666666.59332.peg.531	fig|6666666.59334.peg.1057	fig|6666666.59335.peg.2309	fig|6666666.59336.peg.1201	fig|6666666.59333.peg.3474	fig|6666666.59338.peg.2604	fig|6666666.59339.peg.7850	None	fig|6666666.59337.peg.538	fig|6666666.59342.peg.1515	fig|6666666.71181.peg.3142	fig|6666666.59344.peg.4726	fig|6666666.59345.peg.5027	fig|6666666.59341.peg.5249	fig|6666666.59347.peg.3204	fig|6666666.59346.peg.307	fig|6666666.59348.peg.193	fig|6666666.59349.peg.3006	fig|6666666.59350.peg.6090	fig|6666666.59351.peg.350	fig|6666666.59353.peg.4424	fig|6666666.59355.peg.4504	fig|6666666.59354.peg.3122

8966	Biopolymer transport protein ExbD TolR	fig|6666666.59329.peg.977	fig|6666666.59330.peg.1190	fig|6666666.59331.peg.2230	fig|6666666.59332.peg.1004	fig|6666666.59334.peg.4099	fig|6666666.59335.peg.4622	fig|6666666.59336.peg.939	fig|6666666.59333.peg.3768	fig|6666666.59338.peg.662	fig|6666666.59339.peg.4291	None	fig|6666666.59337.peg.2454	fig|6666666.59342.peg.576	fig|6666666.71181.peg.4451	fig|6666666.59344.peg.5905	fig|6666666.59345.peg.3050	fig|6666666.59341.peg.5936	fig|6666666.59347.peg.3062	fig|6666666.59346.peg.3032	fig|6666666.59348.peg.442	fig|6666666.59349.peg.2549	fig|6666666.59350.peg.8032	fig|6666666.59351.peg.2743	fig|6666666.59353.peg.2491	fig|6666666.59355.peg.585	fig|6666666.59354.peg.1115

1646	Glutamate-ammonia-ligase adenylyltransferase EC 2.7.7.42	fig|6666666.59329.peg.390	fig|6666666.59330.peg.947	fig|6666666.59331.peg.2373	fig|6666666.59332.peg.3640	fig|6666666.59334.peg.541	fig|6666666.59335.peg.1515	fig|6666666.59336.peg.4471	fig|6666666.59333.peg.199	fig|6666666.59338.peg.963	fig|6666666.59339.peg.7405	fig|6666666.59340.peg.2028	fig|6666666.59337.peg.13	None	fig|6666666.71181.peg.3018	fig|6666666.59344.peg.4463	fig|6666666.59345.peg.3828	fig|6666666.59341.peg.1716	fig|6666666.59347.peg.3907	fig|6666666.59346.peg.1627	fig|6666666.59348.peg.5268	fig|6666666.59349.peg.812	fig|6666666.59350.peg.2825	fig|6666666.59351.peg.3540	fig|6666666.59353.peg.4178	fig|6666666.59355.peg.1699	fig|6666666.59354.peg.653

1423	C1496 Uncharacterized conserved protein	fig|6666666.59329.peg.3086	fig|6666666.59330.peg.2897	None	fig|6666666.59332.peg.3171	fig|6666666.59334.peg.866	fig|6666666.59335.peg.4775	fig|6666666.59336.peg.5972	fig|6666666.59333.peg.3336	fig|6666666.59338.peg.4491	fig|6666666.59339.peg.7519	fig|6666666.59340.peg.2913	fig|6666666.59337.peg.161	fig|6666666.59342.peg.3350	fig|6666666.71181.peg.3943	fig|6666666.59344.peg.5427	fig|6666666.59345.peg.5684	fig|6666666.59341.peg.5922	fig|6666666.59347.peg.4058	fig|6666666.59346.peg.272	fig|6666666.59348.peg.2269	fig|6666666.59349.peg.586	fig|6666666.59350.peg.2582	fig|6666666.59351.peg.519	fig|6666666.59353.peg.4800	fig|6666666.59355.peg.4854	fig|6666666.59354.peg.1587

3362	tRNA-i 6 A37 methylthiotransferase	fig|6666666.59329.peg.4341	fig|6666666.59330.peg.4033	None	fig|6666666.59332.peg.2937	fig|6666666.59334.peg.4276	fig|6666666.59335.peg.3413	fig|6666666.59336.peg.2835	fig|6666666.59333.peg.2147	fig|6666666.59338.peg.3624	fig|6666666.59339.peg.8801	fig|6666666.59340.peg.861	fig|6666666.59337.peg.1072	fig|6666666.59342.peg.1133	fig|6666666.71181.peg.3207	fig|6666666.59344.peg.4516	fig|6666666.59345.peg.3483	fig|6666666.59341.peg.1640	fig|6666666.59347.peg.4451	fig|6666666.59346.peg.2961	fig|6666666.59348.peg.3121	fig|6666666.59349.peg.4521	fig|6666666.59350.peg.6421	fig|6666666.59351.peg.4671	fig|6666666.59353.peg.2039	fig|6666666.59355.peg.4446	fig|6666666.59354.peg.5216

8581	RNA polymerase sigma factor RpoD	fig|6666666.59329.peg.677	fig|6666666.59330.peg.3133	fig|6666666.59331.peg.797	fig|6666666.59332.peg.3922	fig|6666666.59334.peg.2470	fig|6666666.59335.peg.1640	fig|6666666.59336.peg.3446	fig|6666666.59333.peg.170	fig|6666666.59338.peg.4123	fig|6666666.59339.peg.7903	fig|6666666.59340.peg.1094	fig|6666666.59337.peg.1422	fig|6666666.59342.peg.1634	fig|6666666.71181.peg.3004	fig|6666666.59344.peg.4479	fig|6666666.59345.peg.3850	fig|6666666.59341.peg.3726	fig|6666666.59347.peg.3140	fig|6666666.59346.peg.2564	fig|6666666.59348.peg.248	fig|6666666.59349.peg.1954	None	fig|6666666.59351.peg.2363	fig|6666666.59353.peg.4190	fig|6666666.59355.peg.1684	fig|6666666.59354.peg.9

3223	Putative hemolysin	fig|6666666.59329.peg.3809	fig|6666666.59330.peg.320	fig|6666666.59331.peg.3682	fig|6666666.59332.peg.6128	None	fig|6666666.59335.peg.867	fig|6666666.59336.peg.2731	fig|6666666.59333.peg.1436	fig|6666666.59338.peg.2849	fig|6666666.59339.peg.1757	fig|6666666.59340.peg.547	fig|6666666.59337.peg.1048	fig|6666666.59342.peg.3977	fig|6666666.71181.peg.3939	fig|6666666.59344.peg.5379	fig|6666666.59345.peg.544	fig|6666666.59341.peg.2671	fig|6666666.59347.peg.3945	fig|6666666.59346.peg.4722	fig|6666666.59348.peg.781	fig|6666666.59349.peg.1304	fig|6666666.59350.peg.2552	fig|6666666.59351.peg.492	fig|6666666.59353.peg.5006	fig|6666666.59355.peg.5102	fig|6666666.59354.peg.2261

6495	4-hydroxybenzoyl-CoA thioesterase family active site	fig|6666666.59329.peg.3636	fig|6666666.59330.peg.3440	fig|6666666.59331.peg.1727	fig|6666666.59332.peg.6245	fig|6666666.59334.peg.4672	fig|6666666.59335.peg.2505	fig|6666666.59336.peg.2583	fig|6666666.59333.peg.1985	fig|6666666.59338.peg.1883	fig|6666666.59339.peg.394	None	fig|6666666.59337.peg.3598	fig|6666666.59342.peg.4591	fig|6666666.71181.peg.3932	fig|6666666.59344.peg.5387	fig|6666666.59345.peg.1862	fig|6666666.59341.peg.3347	fig|6666666.59347.peg.536	fig|6666666.59346.peg.3177	fig|6666666.59348.peg.5096	fig|6666666.59349.peg.4553	fig|6666666.59350.peg.142	fig|6666666.59351.peg.2172	fig|6666666.59353.peg.747	fig|6666666.59355.peg.5599	fig|6666666.59354.peg.4810

6491	SSU ribosomal protein S15p S13e	fig|6666666.59329.peg.1896	fig|6666666.59330.peg.103	fig|6666666.59331.peg.2111	fig|6666666.59332.peg.3551	fig|6666666.59334.peg.774	fig|6666666.59335.peg.5133	fig|6666666.59336.peg.6412	fig|6666666.59333.peg.2321	fig|6666666.59338.peg.3433	fig|6666666.59339.peg.7426	fig|6666666.59340.peg.956	fig|6666666.59337.peg.687	fig|6666666.59342.peg.381	fig|6666666.71181.peg.4101	fig|6666666.59344.peg.2096	fig|6666666.59345.peg.2353	fig|6666666.59341.peg.2443	fig|6666666.59347.peg.3001	fig|6666666.59346.peg.302	None	fig|6666666.59349.peg.4979	fig|6666666.59350.peg.3816	fig|6666666.59351.peg.1386	fig|6666666.59353.peg.3129	fig|6666666.59355.peg.467	fig|6666666.59354.peg.5105

2446	Transcription termination factor Rho	fig|6666666.59329.peg.6276	fig|6666666.59330.peg.1004	fig|6666666.59331.peg.1508	fig|6666666.59332.peg.3581	fig|6666666.59334.peg.419	fig|6666666.59335.peg.4612	fig|6666666.59336.peg.1640	fig|6666666.59333.peg.3250	fig|6666666.59338.peg.1199	fig|6666666.59339.peg.7681	None	fig|6666666.59337.peg.2526	fig|6666666.59342.peg.3026	fig|6666666.71181.peg.2452	fig|6666666.59344.peg.2460	fig|6666666.59345.peg.5518	fig|6666666.59341.peg.4204	fig|6666666.59347.peg.5233	fig|6666666.59346.peg.4509	fig|6666666.59348.peg.4523	fig|6666666.59349.peg.4689	fig|6666666.59350.peg.8182	fig|6666666.59351.peg.4773	fig|6666666.59353.peg.3651	fig|6666666.59355.peg.4022	fig|6666666.59354.peg.4157

7862	Putative membrane protein YfcA	fig|6666666.59329.peg.2651	fig|6666666.59330.peg.1308	fig|6666666.59331.peg.4372	fig|6666666.59332.peg.1379	fig|6666666.59334.peg.2091	fig|6666666.59335.peg.4286	fig|6666666.59336.peg.2531	fig|6666666.59333.peg.1018	fig|6666666.59338.peg.1280	fig|6666666.59339.peg.4992	fig|6666666.59340.peg.2515	fig|6666666.59337.peg.66	fig|6666666.59342.peg.2672	fig|6666666.71181.peg.1509	fig|6666666.59344.peg.5003	fig|6666666.59345.peg.4129	fig|6666666.59341.peg.4943	fig|6666666.59347.peg.1575	fig|6666666.59346.peg.1850	fig|6666666.59348.peg.4655	fig|6666666.59349.peg.1911	fig|6666666.59350.peg.199	fig|6666666.59351.peg.472	fig|6666666.59353.peg.5016	fig|6666666.59355.peg.232	None

7861	Choline dehydrogenase EC 1.1.99.1	fig|6666666.59329.peg.5916	fig|6666666.59330.peg.679	fig|6666666.59331.peg.254	fig|6666666.59332.peg.5493	fig|6666666.59334.peg.1466	fig|6666666.59335.peg.5829	fig|6666666.59336.peg.2785	fig|6666666.59333.peg.4013	fig|6666666.59338.peg.931	fig|6666666.59339.peg.2599	None	fig|6666666.59337.peg.5261	fig|6666666.59342.peg.2858	fig|6666666.71181.peg.6279	fig|6666666.59344.peg.3967	fig|6666666.59345.peg.1527	fig|6666666.59341.peg.3161	fig|6666666.59347.peg.3575	fig|6666666.59346.peg.1202	fig|6666666.59348.peg.4979	fig|6666666.59349.peg.1480	fig|6666666.59350.peg.7906	fig|6666666.59351.peg.1960	fig|6666666.59353.peg.2437	fig|6666666.59355.peg.157	fig|6666666.59354.peg.2676

9327	6,7-dimethyl-8-ribityllumazine synthase EC 2.5.1.78	fig|6666666.59329.peg.6244	fig|6666666.59330.peg.1058	fig|6666666.59331.peg.4459	fig|6666666.59332.peg.4208	fig|6666666.59334.peg.2070	fig|6666666.59335.peg.154	fig|6666666.59336.peg.3173	fig|6666666.59333.peg.4711	fig|6666666.59338.peg.5267	fig|6666666.59339.peg.5096	fig|6666666.59340.peg.965	fig|6666666.59337.peg.1138	fig|6666666.59342.peg.3331	fig|6666666.71181.peg.2727	fig|6666666.59344.peg.1099	fig|6666666.59345.peg.3539	fig|6666666.59341.peg.2617	fig|6666666.59347.peg.2767	fig|6666666.59346.peg.3382	fig|6666666.59348.peg.926	fig|6666666.59349.peg.3484	fig|6666666.59350.peg.1711	None	fig|6666666.59353.peg.778	fig|6666666.59355.peg.926	fig|6666666.59354.peg.2927

5407	SSU ribosomal protein S10p S20e	fig|6666666.59329.peg.1100	fig|6666666.59330.peg.549	fig|6666666.59331.peg.3028	fig|6666666.59332.peg.3793	fig|6666666.59334.peg.1479	fig|6666666.59335.peg.1852	fig|6666666.59336.peg.139	fig|6666666.59333.peg.2687	fig|6666666.59338.peg.1395	None	fig|6666666.59340.peg.2011	fig|6666666.59337.peg.394	fig|6666666.59342.peg.299	fig|6666666.71181.peg.3461	fig|6666666.59344.peg.4944	fig|6666666.59345.peg.2541	fig|6666666.59341.peg.2492	fig|6666666.59347.peg.4818	fig|6666666.59346.peg.5022	fig|6666666.59348.peg.409	fig|6666666.59349.peg.1092	fig|6666666.59350.peg.4268	fig|6666666.59351.peg.4819	fig|6666666.59353.peg.4589	fig|6666666.59355.peg.4742	fig|6666666.59354.peg.1753

1628	Formamidopyrimidine-DNA glycosylase EC 3.2.2.23	fig|6666666.59329.peg.552	fig|6666666.59330.peg.1164	None	fig|6666666.59332.peg.293	fig|6666666.59334.peg.184	fig|6666666.59335.peg.1997	fig|6666666.59336.peg.56	fig|6666666.59333.peg.651	fig|6666666.59338.peg.1119	fig|6666666.59339.peg.6293	fig|6666666.59340.peg.668	fig|6666666.59337.peg.469	fig|6666666.59342.peg.5553	fig|6666666.71181.peg.3656	fig|6666666.59344.peg.944	fig|6666666.59345.peg.2617	fig|6666666.59341.peg.5103	fig|6666666.59347.peg.4514	fig|6666666.59346.peg.1710	fig|6666666.59348.peg.3017	fig|6666666.59349.peg.3063	fig|6666666.59350.peg.2725	fig|6666666.59351.peg.2236	fig|6666666.59353.peg.2590	fig|6666666.59355.peg.2611	fig|6666666.59354.peg.4275

2866	Porphobilinogen synthase EC 4.2.1.24	fig|6666666.59329.peg.2095	fig|6666666.59330.peg.259	fig|6666666.59331.peg.6144	fig|6666666.59332.peg.13	fig|6666666.59334.peg.844	fig|6666666.59335.peg.47	fig|6666666.59336.peg.1459	fig|6666666.59333.peg.833	fig|6666666.59338.peg.3949	fig|6666666.59339.peg.7582	fig|6666666.59340.peg.3342	fig|6666666.59337.peg.969	None	fig|6666666.71181.peg.3334	fig|6666666.59344.peg.3408	fig|6666666.59345.peg.2706	fig|6666666.59341.peg.161	fig|6666666.59347.peg.4643	fig|6666666.59346.peg.1071	fig|6666666.59348.peg.109	fig|6666666.59349.peg.3422	fig|6666666.59350.peg.7938	fig|6666666.59351.peg.4991	fig|6666666.59353.peg.3804	fig|6666666.59355.peg.3400	fig|6666666.59354.peg.3341

551	Ribose ABC transport system, periplasmic ribose-binding protein RbsB TC 3.A.1.2.1	fig|6666666.59329.peg.4642	fig|6666666.59330.peg.712	fig|6666666.59331.peg.3105	fig|6666666.59332.peg.2872	fig|6666666.59334.peg.639	fig|6666666.59335.peg.4477	fig|6666666.59336.peg.5743	fig|6666666.59333.peg.1249	fig|6666666.59338.peg.2266	fig|6666666.59339.peg.6902	fig|6666666.59340.peg.934	fig|6666666.59337.peg.4621	None	fig|6666666.71181.peg.5012	fig|6666666.59344.peg.5978	fig|6666666.59345.peg.4803	fig|6666666.59341.peg.3733	fig|6666666.59347.peg.3379	fig|6666666.59346.peg.455	fig|6666666.59348.peg.4985	fig|6666666.59349.peg.954	fig|6666666.59350.peg.7990	fig|6666666.59351.peg.2260	fig|6666666.59353.peg.487	fig|6666666.59355.peg.1415	fig|6666666.59354.peg.2908

1344	4-Hydroxy-2-oxoglutarate aldolase EC 4.1.3.16 2-dehydro-3-deoxyphosphogluconate aldolase EC 4.1.2.14	fig|6666666.59329.peg.2746	fig|6666666.59330.peg.209	fig|6666666.59331.peg.129	fig|6666666.59332.peg.3023	fig|6666666.59334.peg.3116	fig|6666666.59335.peg.2927	fig|6666666.59336.peg.4890	fig|6666666.59333.peg.689	fig|6666666.59338.peg.3239	fig|6666666.59339.peg.8450	None	fig|6666666.59337.peg.2802	fig|6666666.59342.peg.5179	fig|6666666.71181.peg.1933	fig|6666666.59344.peg.2842	fig|6666666.59345.peg.2112	fig|6666666.59341.peg.4266	fig|6666666.59347.peg.1072	fig|6666666.59346.peg.2853	fig|6666666.59348.peg.685	fig|6666666.59349.peg.2677	fig|6666666.59350.peg.6795	fig|6666666.59351.peg.899	fig|6666666.59353.peg.3032	fig|6666666.59355.peg.2299	fig|6666666.59354.peg.5079

7524	Flagellar basal-body rod protein FlgC	fig|6666666.59329.peg.577	fig|6666666.59330.peg.2687	fig|6666666.59331.peg.2030	fig|6666666.59332.peg.1794	None	fig|6666666.59335.peg.897	fig|6666666.59336.peg.1962	fig|6666666.59333.peg.2888	fig|6666666.59338.peg.632	fig|6666666.59339.peg.68	fig|6666666.59340.peg.410	fig|6666666.59337.peg.3349	fig|6666666.59342.peg.1498	fig|6666666.71181.peg.2229	fig|6666666.59344.peg.3034	fig|6666666.59345.peg.5450	fig|6666666.59341.peg.3530	fig|6666666.59347.peg.2713	fig|6666666.59346.peg.1763	fig|6666666.59348.peg.949	fig|6666666.59349.peg.722	fig|6666666.59350.peg.5406	fig|6666666.59351.peg.1983	fig|6666666.59353.peg.3526	fig|6666666.59355.peg.3649	fig|6666666.59354.peg.1525

2278	SSU ribosomal protein S2p SAe	fig|6666666.59329.peg.5026	fig|6666666.59330.peg.469	fig|6666666.59331.peg.4432	fig|6666666.59332.peg.1592	fig|6666666.59334.peg.2977	fig|6666666.59335.peg.162	fig|6666666.59336.peg.4215	fig|6666666.59333.peg.3061	fig|6666666.59338.peg.2556	fig|6666666.59339.peg.5107	None	fig|6666666.59337.peg.1581	fig|6666666.59342.peg.265	fig|6666666.71181.peg.35	fig|6666666.59344.peg.2652	fig|6666666.59345.peg.2201	fig|6666666.59341.peg.967	fig|6666666.59347.peg.4358	fig|6666666.59346.peg.2260	fig|6666666.59348.peg.1948	fig|6666666.59349.peg.1376	fig|6666666.59350.peg.6670	fig|6666666.59351.peg.2114	fig|6666666.59353.peg.1983	fig|6666666.59355.peg.4090	fig|6666666.59354.peg.4017

5545	Isochorismatase EC 3.3.2.1	fig|6666666.59329.peg.436	fig|6666666.59330.peg.1779	fig|6666666.59331.peg.818	fig|6666666.59332.peg.3119	fig|6666666.59334.peg.4408	fig|6666666.59335.peg.3195	fig|6666666.59336.peg.1934	fig|6666666.59333.peg.3822	fig|6666666.59338.peg.5260	fig|6666666.59339.peg.1697	None	fig|6666666.59337.peg.5398	fig|6666666.59342.peg.5382	fig|6666666.71181.peg.3688	fig|6666666.59344.peg.3296	fig|6666666.59345.peg.959	fig|6666666.59341.peg.5229	fig|6666666.59347.peg.267	fig|6666666.59346.peg.370	fig|6666666.59348.peg.1488	fig|6666666.59349.peg.976	fig|6666666.59350.peg.6784	fig|6666666.59351.peg.36	fig|6666666.59353.peg.939	fig|6666666.59355.peg.3156	fig|6666666.59354.peg.2706

10087	Universal stress protein E	fig|6666666.59329.peg.3061	fig|6666666.59330.peg.3197	fig|6666666.59331.peg.197	fig|6666666.59332.peg.5669	fig|6666666.59334.peg.984	fig|6666666.59335.peg.3877	fig|6666666.59336.peg.4252	fig|6666666.59333.peg.4756	fig|6666666.59338.peg.5813	fig|6666666.59339.peg.1317	fig|6666666.59340.peg.3367	fig|6666666.59337.peg.3718	fig|6666666.59342.peg.1853	fig|6666666.71181.peg.5276	fig|6666666.59344.peg.592	fig|6666666.59345.peg.3099	fig|6666666.59341.peg.6076	fig|6666666.59347.peg.1736	fig|6666666.59346.peg.1526	fig|6666666.59348.peg.3256	fig|6666666.59349.peg.145	None	fig|6666666.59351.peg.826	fig|6666666.59353.peg.1889	fig|6666666.59355.peg.1126	fig|6666666.59354.peg.191

10400	RNA polymerase sigma-54 factor RpoN	fig|6666666.59329.peg.817	fig|6666666.59330.peg.45	fig|6666666.59331.peg.2669	fig|6666666.59332.peg.2741	fig|6666666.59334.peg.103	fig|6666666.59335.peg.3053	fig|6666666.59336.peg.348	fig|6666666.59333.peg.261	fig|6666666.59338.peg.105	fig|6666666.59339.peg.8204	fig|6666666.59340.peg.3541	fig|6666666.59337.peg.829	fig|6666666.59342.peg.5556	fig|6666666.71181.peg.1016	fig|6666666.59344.peg.1795	fig|6666666.59345.peg.4861	fig|6666666.59341.peg.1242	fig|6666666.59347.peg.3253	fig|6666666.59346.peg.641	fig|6666666.59348.peg.2011	fig|6666666.59349.peg.20	None	fig|6666666.59351.peg.3438	fig|6666666.59353.peg.2334	fig|6666666.59355.peg.1607	fig|6666666.59354.peg.3619

414	Carbamoyl-phosphate synthase small chain EC 6.3.5.5	fig|6666666.59329.peg.6010	fig|6666666.59330.peg.1288	fig|6666666.59331.peg.1253	fig|6666666.59332.peg.4398	fig|6666666.59334.peg.2081	fig|6666666.59335.peg.820	fig|6666666.59336.peg.2525	fig|6666666.59333.peg.4109	fig|6666666.59338.peg.4136	fig|6666666.59339.peg.4986	fig|6666666.59340.peg.664	fig|6666666.59337.peg.60	fig|6666666.59342.peg.4413	fig|6666666.71181.peg.412	fig|6666666.59344.peg.1305	fig|6666666.59345.peg.3781	fig|6666666.59341.peg.3132	fig|6666666.59347.peg.4010	fig|6666666.59346.peg.4834	fig|6666666.59348.peg.251	fig|6666666.59349.peg.4249	None	fig|6666666.59351.peg.3192	fig|6666666.59353.peg.86	fig|6666666.59355.peg.694	fig|6666666.59354.peg.4039

7848	tmRNA-binding protein SmpB	fig|6666666.59329.peg.3956	fig|6666666.59330.peg.2427	fig|6666666.59331.peg.1335	fig|6666666.59332.peg.377	fig|6666666.59334.peg.4413	fig|6666666.59335.peg.4164	fig|6666666.59336.peg.216	fig|6666666.59333.peg.2764	fig|6666666.59338.peg.1073	fig|6666666.59339.peg.7284	fig|6666666.59340.peg.2664	fig|6666666.59337.peg.5544	None	fig|6666666.71181.peg.1445	fig|6666666.59344.peg.1540	fig|6666666.59345.peg.3581	fig|6666666.59341.peg.5534	fig|6666666.59347.peg.4323	fig|6666666.59346.peg.696	fig|6666666.59348.peg.1843	fig|6666666.59349.peg.2636	fig|6666666.59350.peg.52	fig|6666666.59351.peg.4916	fig|6666666.59353.peg.4361	fig|6666666.59355.peg.4482	fig|6666666.59354.peg.4675

6351	Chromosomal replication initiator protein DnaA	fig|6666666.59329.peg.5727	fig|6666666.59330.peg.1102	None	fig|6666666.59332.peg.85	fig|6666666.59334.peg.4247	fig|6666666.59335.peg.776	fig|6666666.59336.peg.11	fig|6666666.59333.peg.49	fig|6666666.59338.peg.1268	fig|6666666.59339.peg.8037	fig|6666666.59340.peg.1131	fig|6666666.59337.peg.875	fig|6666666.59342.peg.518	fig|6666666.71181.peg.622	fig|6666666.59344.peg.4169	fig|6666666.59345.peg.4979	fig|6666666.59341.peg.1042	fig|6666666.59347.peg.4798	fig|6666666.59346.peg.1195	fig|6666666.59348.peg.315	fig|6666666.59349.peg.4957	fig|6666666.59350.peg.2728	fig|6666666.59351.peg.5028	fig|6666666.59353.peg.3057	fig|6666666.59355.peg.5788	fig|6666666.59354.peg.1559

9340	Ribonuclease E inhibitor RraA	fig|6666666.59329.peg.5113	fig|6666666.59330.peg.5061	fig|6666666.59331.peg.341	fig|6666666.59332.peg.170	fig|6666666.59334.peg.1737	fig|6666666.59335.peg.3556	fig|6666666.59336.peg.4988	fig|6666666.59333.peg.2393	fig|6666666.59338.peg.2429	fig|6666666.59339.peg.7410	fig|6666666.59340.peg.23	fig|6666666.59337.peg.88	fig|6666666.59342.peg.5566	fig|6666666.71181.peg.3871	fig|6666666.59344.peg.868	fig|6666666.59345.peg.3754	fig|6666666.59341.peg.85	fig|6666666.59347.peg.5024	fig|6666666.59346.peg.1016	fig|6666666.59348.peg.5352	fig|6666666.59349.peg.502	None	fig|6666666.59351.peg.3825	fig|6666666.59353.peg.1250	fig|6666666.59355.peg.2658	fig|6666666.59354.peg.949

9618	Chorismate--pyruvate lyase EC 4.1.3.40	fig|6666666.59329.peg.776	fig|6666666.59330.peg.1571	fig|6666666.59331.peg.5679	fig|6666666.59332.peg.2377	fig|6666666.59334.peg.5042	fig|6666666.59335.peg.4264	fig|6666666.59336.peg.1371	fig|6666666.59333.peg.1956	fig|6666666.59338.peg.531	fig|6666666.59339.peg.8575	fig|6666666.59340.peg.3582	fig|6666666.59337.peg.1655	fig|6666666.59342.peg.4983	fig|6666666.71181.peg.2006	fig|6666666.59344.peg.2735	fig|6666666.59345.peg.5465	fig|6666666.59341.peg.5293	fig|6666666.59347.peg.5049	fig|6666666.59346.peg.1644	fig|6666666.59348.peg.2969	fig|6666666.59349.peg.1702	None	fig|6666666.59351.peg.5214	fig|6666666.59353.peg.3003	fig|6666666.59355.peg.2910	fig|6666666.59354.peg.4023

1813	Uncharacterized protein YtfN	fig|6666666.59329.peg.1782	fig|6666666.59330.peg.845	fig|6666666.59331.peg.770	fig|6666666.59332.peg.3142	fig|6666666.59334.peg.62	fig|6666666.59335.peg.175	fig|6666666.59336.peg.925	fig|6666666.59333.peg.576	fig|6666666.59338.peg.4825	fig|6666666.59339.peg.7725	fig|6666666.59340.peg.872	fig|6666666.59337.peg.337	fig|6666666.59342.peg.4406	fig|6666666.71181.peg.2807	fig|6666666.59344.peg.2133	fig|6666666.59345.peg.2487	fig|6666666.59341.peg.5008	fig|6666666.59347.peg.4868	fig|6666666.59346.peg.1977	fig|6666666.59348.peg.2839	fig|6666666.59349.peg.1339	None	fig|6666666.59351.peg.4237	fig|6666666.59353.peg.3165	fig|6666666.59355.peg.432	fig|6666666.59354.peg.3220

9895	Iron-sulfur cluster regulator IscR	fig|6666666.59329.peg.1112	fig|6666666.59330.peg.1437	fig|6666666.59331.peg.1218	fig|6666666.59332.peg.1431	fig|6666666.59334.peg.4046	fig|6666666.59335.peg.2365	fig|6666666.59336.peg.3060	fig|6666666.59333.peg.923	fig|6666666.59338.peg.1327	fig|6666666.59339.peg.6788	fig|6666666.59340.peg.700	fig|6666666.59337.peg.1791	fig|6666666.59342.peg.6009	fig|6666666.71181.peg.1756	fig|6666666.59344.peg.1626	fig|6666666.59345.peg.3653	fig|6666666.59341.peg.4635	fig|6666666.59347.peg.4282	fig|6666666.59346.peg.2741	fig|6666666.59348.peg.1380	fig|6666666.59349.peg.2436	fig|6666666.59350.peg.1670	fig|6666666.59351.peg.2714	fig|6666666.59353.peg.2693	fig|6666666.59355.peg.1786	None

5131	Malonyl CoA-acyl carrier protein transacylase EC 2.3.1.39	fig|6666666.59329.peg.4856	fig|6666666.59330.peg.3623	fig|6666666.59331.peg.1743	fig|6666666.59332.peg.3473	fig|6666666.59334.peg.3240	fig|6666666.59335.peg.5372	fig|6666666.59336.peg.3341	fig|6666666.59333.peg.1589	None	fig|6666666.59339.peg.4096	fig|6666666.59340.peg.406	fig|6666666.59337.peg.2688	fig|6666666.59342.peg.1383	fig|6666666.71181.peg.1622	fig|6666666.59344.peg.2433	fig|6666666.59345.peg.1064	fig|6666666.59341.peg.15	fig|6666666.59347.peg.4084	fig|6666666.59346.peg.106	fig|6666666.59348.peg.1242	fig|6666666.59349.peg.3909	fig|6666666.59350.peg.1347	fig|6666666.59351.peg.671	fig|6666666.59353.peg.313	fig|6666666.59355.peg.1353	fig|6666666.59354.peg.1139

4515	Methylase of polypeptide chain release factors	fig|6666666.59329.peg.2441	fig|6666666.59330.peg.4523	fig|6666666.59331.peg.4542	fig|6666666.59332.peg.2620	fig|6666666.59334.peg.1705	fig|6666666.59335.peg.1180	fig|6666666.59336.peg.5118	fig|6666666.59333.peg.231	fig|6666666.59338.peg.1088	fig|6666666.59339.peg.11	None	fig|6666666.59337.peg.4847	fig|6666666.59342.peg.460	fig|6666666.71181.peg.1512	fig|6666666.59344.peg.5000	fig|6666666.59345.peg.5418	fig|6666666.59341.peg.3045	fig|6666666.59347.peg.5111	fig|6666666.59346.peg.3192	fig|6666666.59348.peg.4330	fig|6666666.59349.peg.2616	fig|6666666.59350.peg.665	fig|6666666.59351.peg.4661	fig|6666666.59353.peg.433	fig|6666666.59355.peg.2160	fig|6666666.59354.peg.122

9022	ATP-dependent protease La EC 3.4.21.53 Type II	fig|6666666.59329.peg.2247	fig|6666666.59330.peg.298	fig|6666666.59331.peg.4451	fig|6666666.59332.peg.5213	fig|6666666.59334.peg.3457	fig|6666666.59335.peg.5478	fig|6666666.59336.peg.3729	fig|6666666.59333.peg.4165	fig|6666666.59338.peg.4154	fig|6666666.59339.peg.1507	None	fig|6666666.59337.peg.4561	fig|6666666.59342.peg.2342	fig|6666666.71181.peg.2054	fig|6666666.59344.peg.1187	fig|6666666.59345.peg.4255	fig|6666666.59341.peg.3783	fig|6666666.59347.peg.2656	fig|6666666.59346.peg.4321	fig|6666666.59348.peg.2987	fig|6666666.59349.peg.4912	fig|6666666.59350.peg.4165	fig|6666666.59351.peg.3632	fig|6666666.59353.peg.1692	fig|6666666.59355.peg.1029	fig|6666666.59354.peg.1682

7043	Tyrosyl-tRNA synthetase EC 6.1.1.1	fig|6666666.59329.peg.1594	fig|6666666.59330.peg.121	None	fig|6666666.59332.peg.1018	fig|6666666.59334.peg.196	fig|6666666.59335.peg.2837	fig|6666666.59336.peg.234	fig|6666666.59333.peg.1315	fig|6666666.59338.peg.68	fig|6666666.59339.peg.4848	fig|6666666.59340.peg.2483	fig|6666666.59337.peg.669	fig|6666666.59342.peg.2391	fig|6666666.71181.peg.2927	fig|6666666.59344.peg.3489	fig|6666666.59345.peg.1680	fig|6666666.59341.peg.1140	fig|6666666.59347.peg.3069	fig|6666666.59346.peg.284	fig|6666666.59348.peg.979	fig|6666666.59349.peg.1670	fig|6666666.59350.peg.3149	fig|6666666.59351.peg.286	fig|6666666.59353.peg.1392	fig|6666666.59355.peg.2480	fig|6666666.59354.peg.819

1017	Glycerophosphoryl diester phosphodiesterase EC 3.1.4.46	fig|6666666.59329.peg.2671	fig|6666666.59330.peg.4538	fig|6666666.59331.peg.4128	fig|6666666.59332.peg.5784	fig|6666666.59334.peg.2115	fig|6666666.59335.peg.1737	fig|6666666.59336.peg.1659	fig|6666666.59333.peg.4530	fig|6666666.59338.peg.3835	fig|6666666.59339.peg.7338	None	fig|6666666.59337.peg.5133	fig|6666666.59342.peg.4932	fig|6666666.71181.peg.4414	fig|6666666.59344.peg.5731	fig|6666666.59345.peg.3969	fig|6666666.59341.peg.4858	fig|6666666.59347.peg.3433	fig|6666666.59346.peg.4384	fig|6666666.59348.peg.3080	fig|6666666.59349.peg.2376	fig|6666666.59350.peg.38	fig|6666666.59351.peg.137	fig|6666666.59353.peg.4631	fig|6666666.59355.peg.4748	fig|6666666.59354.peg.5945

433	TRAP-type C4-dicarboxylate transport system, large permease component	fig|6666666.59329.peg.3188	fig|6666666.59330.peg.288	fig|6666666.59331.peg.270	fig|6666666.59332.peg.4619	fig|6666666.59334.peg.2560	fig|6666666.59335.peg.227	fig|6666666.59336.peg.3781	fig|6666666.59333.peg.2723	fig|6666666.59338.peg.4526	fig|6666666.59339.peg.4754	fig|6666666.59340.peg.2574	fig|6666666.59337.peg.3582	fig|6666666.59342.peg.2506	fig|6666666.71181.peg.3634	fig|6666666.59344.peg.5267	fig|6666666.59345.peg.2475	fig|6666666.59341.peg.1984	fig|6666666.59347.peg.169	fig|6666666.59346.peg.2827	fig|6666666.59348.peg.3085	fig|6666666.59349.peg.1089	fig|6666666.59350.peg.6274	None	fig|6666666.59353.peg.4706	fig|6666666.59355.peg.4784	fig|6666666.59354.peg.5486

11345	Porphobilinogen deaminase EC 2.5.1.61	fig|6666666.59329.peg.97	fig|6666666.59330.peg.3046	fig|6666666.59331.peg.6133	fig|6666666.59332.peg.1710	fig|6666666.59334.peg.1136	fig|6666666.59335.peg.3329	fig|6666666.59336.peg.1122	fig|6666666.59333.peg.785	fig|6666666.59338.peg.4277	fig|6666666.59339.peg.8514	None	fig|6666666.59337.peg.1613	fig|6666666.59342.peg.3005	fig|6666666.71181.peg.2436	fig|6666666.59344.peg.2482	fig|6666666.59345.peg.5508	fig|6666666.59341.peg.3912	fig|6666666.59347.peg.5081	fig|6666666.59346.peg.595	fig|6666666.59348.peg.2177	fig|6666666.59349.peg.1563	fig|6666666.59350.peg.1936	fig|6666666.59351.peg.4756	fig|6666666.59353.peg.3635	fig|6666666.59355.peg.4005	fig|6666666.59354.peg.1461

2157	Cytochrome c oxidase polypeptide II EC 1.9.3.1	fig|6666666.59329.peg.3769	fig|6666666.59330.peg.2613	fig|6666666.59331.peg.2215	fig|6666666.59332.peg.4579	fig|6666666.59334.peg.992	fig|6666666.59335.peg.2874	fig|6666666.59336.peg.3606	fig|6666666.59333.peg.3090	fig|6666666.59338.peg.422	fig|6666666.59339.peg.1595	None	fig|6666666.59337.peg.1473	fig|6666666.59342.peg.1729	fig|6666666.71181.peg.5611	fig|6666666.59344.peg.1076	fig|6666666.59345.peg.5612	fig|6666666.59341.peg.2748	fig|6666666.59347.peg.1554	fig|6666666.59346.peg.4304	fig|6666666.59348.peg.1399	fig|6666666.59349.peg.3510	fig|6666666.59350.peg.2023	fig|6666666.59351.peg.4389	fig|6666666.59353.peg.760	fig|6666666.59355.peg.944	fig|6666666.59354.peg.2407

8794	Deoxyribodipyrimidine photolyase EC 4.1.99.3	fig|6666666.59329.peg.5655	fig|6666666.59330.peg.2354	fig|6666666.59331.peg.6117	fig|6666666.59332.peg.3669	fig|6666666.59334.peg.4984	fig|6666666.59335.peg.1930	fig|6666666.59336.peg.1136	fig|6666666.59333.peg.4242	fig|6666666.59338.peg.4241	fig|6666666.59339.peg.8399	None	fig|6666666.59337.peg.3587	fig|6666666.59342.peg.5066	fig|6666666.71181.peg.3221	fig|6666666.59344.peg.4612	fig|6666666.59345.peg.2993	fig|6666666.59341.peg.2260	fig|6666666.59347.peg.203	fig|6666666.59346.peg.4609	fig|6666666.59348.peg.472	fig|6666666.59349.peg.980	fig|6666666.59350.peg.1134	fig|6666666.59351.peg.1150	fig|6666666.59353.peg.2073	fig|6666666.59355.peg.2754	fig|6666666.59354.peg.2709

8795	DamX, an inner membrane protein involved in bile resistance	fig|6666666.59329.peg.316	fig|6666666.59330.peg.4344	fig|6666666.59331.peg.1889	fig|6666666.59332.peg.5330	fig|6666666.59334.peg.847	fig|6666666.59335.peg.2483	fig|6666666.59336.peg.2248	fig|6666666.59333.peg.4296	fig|6666666.59338.peg.3576	fig|6666666.59339.peg.2927	fig|6666666.59340.peg.74	fig|6666666.59337.peg.1703	fig|6666666.59342.peg.1887	fig|6666666.71181.peg.1830	fig|6666666.59344.peg.3067	fig|6666666.59345.peg.3941	fig|6666666.59341.peg.3357	fig|6666666.59347.peg.4946	fig|6666666.59346.peg.8	fig|6666666.59348.peg.3865	fig|6666666.59349.peg.529	None	fig|6666666.59351.peg.3853	fig|6666666.59353.peg.2220	fig|6666666.59355.peg.1914	fig|6666666.59354.peg.3573

3876	Cytochrome c4	fig|6666666.59329.peg.5013	fig|6666666.59330.peg.264	fig|6666666.59331.peg.158	fig|6666666.59332.peg.21	fig|6666666.59334.peg.839	fig|6666666.59335.peg.5860	fig|6666666.59336.peg.1454	fig|6666666.59333.peg.1153	fig|6666666.59338.peg.3199	fig|6666666.59339.peg.5135	fig|6666666.59340.peg.3307	fig|6666666.59337.peg.1600	fig|6666666.59342.peg.2845	fig|6666666.71181.peg.14	fig|6666666.59344.peg.2676	fig|6666666.59345.peg.2702	fig|6666666.59341.peg.4972	fig|6666666.59347.peg.4646	fig|6666666.59346.peg.4561	fig|6666666.59348.peg.103	fig|6666666.59349.peg.4625	None	fig|6666666.59351.peg.3108	fig|6666666.59353.peg.2006	fig|6666666.59355.peg.4111	fig|6666666.59354.peg.1901

8470	ATP synthase epsilon chain EC 3.6.3.14	fig|6666666.59329.peg.1030	fig|6666666.59330.peg.614	fig|6666666.59331.peg.408	fig|6666666.59332.peg.107	fig|6666666.59334.peg.17	fig|6666666.59335.peg.799	fig|6666666.59336.peg.1521	fig|6666666.59333.peg.21	fig|6666666.59338.peg.4916	fig|6666666.59339.peg.8005	None	fig|6666666.59337.peg.2093	fig|6666666.59342.peg.2041	fig|6666666.71181.peg.599	fig|6666666.59344.peg.4273	fig|6666666.59345.peg.3331	fig|6666666.59341.peg.2132	fig|6666666.59347.peg.2040	fig|6666666.59346.peg.1410	fig|6666666.59348.peg.341	fig|6666666.59349.peg.1037	fig|6666666.59350.peg.2085	fig|6666666.59351.peg.5390	fig|6666666.59353.peg.3081	fig|6666666.59355.peg.2267	fig|6666666.59354.peg.4762

4168	Membrane protein YcjF	fig|6666666.59329.peg.3383	fig|6666666.59330.peg.2086	fig|6666666.59331.peg.1238	fig|6666666.59332.peg.5660	fig|6666666.59334.peg.2328	fig|6666666.59335.peg.5171	fig|6666666.59336.peg.6280	fig|6666666.59333.peg.3930	fig|6666666.59338.peg.5532	fig|6666666.59339.peg.3721	None	fig|6666666.59337.peg.4684	fig|6666666.59342.peg.3830	fig|6666666.71181.peg.1384	fig|6666666.59344.peg.537	fig|6666666.59345.peg.821	fig|6666666.59341.peg.5971	fig|6666666.59347.peg.2141	fig|6666666.59346.peg.4343	fig|6666666.59348.peg.4731	fig|6666666.59349.peg.2084	fig|6666666.59350.peg.1078	fig|6666666.59351.peg.2597	fig|6666666.59353.peg.1844	fig|6666666.59355.peg.1170	fig|6666666.59354.peg.3550

5938	FIG004454 RNA binding protein	fig|6666666.59329.peg.3482	fig|6666666.59330.peg.115	fig|6666666.59331.peg.1131	fig|6666666.59332.peg.4094	fig|6666666.59334.peg.201	fig|6666666.59335.peg.2845	fig|6666666.59336.peg.228	fig|6666666.59333.peg.1488	fig|6666666.59338.peg.5977	fig|6666666.59339.peg.4856	fig|6666666.59340.peg.2860	fig|6666666.59337.peg.675	fig|6666666.59342.peg.2478	fig|6666666.71181.peg.4090	fig|6666666.59344.peg.2108	fig|6666666.59345.peg.2364	fig|6666666.59341.peg.3945	fig|6666666.59347.peg.3012	fig|6666666.59346.peg.290	fig|6666666.59348.peg.1539	fig|6666666.59349.peg.3699	None	fig|6666666.59351.peg.4937	fig|6666666.59353.peg.3141	fig|6666666.59355.peg.455	fig|6666666.59354.peg.1191

7025	1-hydroxy-2-methyl-2- E -butenyl 4-diphosphate synthase EC 1.17.7.1	fig|6666666.59329.peg.306	fig|6666666.59330.peg.1425	fig|6666666.59331.peg.1635	fig|6666666.59332.peg.6206	fig|6666666.59334.peg.1254	fig|6666666.59335.peg.1110	fig|6666666.59336.peg.681	fig|6666666.59333.peg.936	fig|6666666.59338.peg.2294	fig|6666666.59339.peg.6970	None	fig|6666666.59337.peg.751	fig|6666666.59342.peg.1061	fig|6666666.71181.peg.1770	fig|6666666.59344.peg.5134	fig|6666666.59345.peg.3641	fig|6666666.59341.peg.3037	fig|6666666.59347.peg.4296	fig|6666666.59346.peg.1319	fig|6666666.59348.peg.1132	fig|6666666.59349.peg.3082	fig|6666666.59350.peg.4454	fig|6666666.59351.peg.3075	fig|6666666.59353.peg.2709	fig|6666666.59355.peg.1771	fig|6666666.59354.peg.85

8667	Asparaginyl-tRNA synthetase EC 6.1.1.22	fig|6666666.59329.peg.25	fig|6666666.59330.peg.4276	fig|6666666.59331.peg.1663	fig|6666666.59332.peg.1361	fig|6666666.59334.peg.1743	fig|6666666.59335.peg.1667	fig|6666666.59336.peg.1916	fig|6666666.59333.peg.1046	fig|6666666.59338.peg.516	fig|6666666.59339.peg.4879	None	fig|6666666.59337.peg.5192	fig|6666666.59342.peg.2748	fig|6666666.71181.peg.1690	fig|6666666.59344.peg.2772	fig|6666666.59345.peg.3729	fig|6666666.59341.peg.1777	fig|6666666.59347.peg.3041	fig|6666666.59346.peg.968	fig|6666666.59348.peg.4729	fig|6666666.59349.peg.4735	fig|6666666.59350.peg.2634	fig|6666666.59351.peg.956	fig|6666666.59353.peg.1671	fig|6666666.59355.peg.1542	fig|6666666.59354.peg.3242

4087	Homolog of E. coli HemY protein	fig|6666666.59329.peg.5178	fig|6666666.59330.peg.3043	fig|6666666.59331.peg.6074	fig|6666666.59332.peg.1714	fig|6666666.59334.peg.5234	fig|6666666.59335.peg.3326	fig|6666666.59336.peg.1125	fig|6666666.59333.peg.781	fig|6666666.59338.peg.5547	fig|6666666.59339.peg.8508	fig|6666666.59340.peg.846	fig|6666666.59337.peg.1609	fig|6666666.59342.peg.5923	fig|6666666.71181.peg.2439	fig|6666666.59344.peg.2478	fig|6666666.59345.peg.5511	fig|6666666.59341.peg.5809	fig|6666666.59347.peg.5084	fig|6666666.59346.peg.592	fig|6666666.59348.peg.2174	fig|6666666.59349.peg.1565	None	fig|6666666.59351.peg.4759	fig|6666666.59353.peg.3638	fig|6666666.59355.peg.4008	fig|6666666.59354.peg.1465

8088	DNA damage-inducible gene in SOS regulon, dependent on cyclic AMP and H-NS	fig|6666666.59329.peg.2544	fig|6666666.59330.peg.2128	fig|6666666.59331.peg.2761	fig|6666666.59332.peg.5131	fig|6666666.59334.peg.3057	fig|6666666.59335.peg.1136	fig|6666666.59336.peg.109	fig|6666666.59333.peg.4534	fig|6666666.59338.peg.2351	fig|6666666.59339.peg.6933	fig|6666666.59340.peg.2391	fig|6666666.59337.peg.2346	fig|6666666.59342.peg.2449	fig|6666666.71181.peg.1894	fig|6666666.59344.peg.2550	fig|6666666.59345.peg.2266	fig|6666666.59341.peg.518	fig|6666666.59347.peg.2827	fig|6666666.59346.peg.1100	fig|6666666.59348.peg.1333	fig|6666666.59349.peg.2717	None	fig|6666666.59351.peg.554	fig|6666666.59353.peg.1517	fig|6666666.59355.peg.2067	fig|6666666.59354.peg.110

6646	DNA-3-methyladenine glycosylase EC 3.2.2.20	fig|6666666.59329.peg.3677	fig|6666666.59330.peg.1846	None	fig|6666666.59332.peg.3576	fig|6666666.59334.peg.3714	fig|6666666.59335.peg.2454	fig|6666666.59336.peg.5133	fig|6666666.59333.peg.1627	fig|6666666.59338.peg.4269	fig|6666666.59339.peg.529	fig|6666666.59340.peg.3040	fig|6666666.59337.peg.2335	fig|6666666.59342.peg.2313	fig|6666666.71181.peg.651	fig|6666666.59344.peg.4144	fig|6666666.59345.peg.1262	fig|6666666.59341.peg.3892	fig|6666666.59347.peg.2224	fig|6666666.59346.peg.1285	fig|6666666.59348.peg.1108	fig|6666666.59349.peg.217	fig|6666666.59350.peg.5754	fig|6666666.59351.peg.4855	fig|6666666.59353.peg.4120	fig|6666666.59355.peg.2320	fig|6666666.59354.peg.3813

7804	Cytochrome d ubiquinol oxidase subunit II EC 1.10.3.-	fig|6666666.59329.peg.2192	fig|6666666.59330.peg.3442	fig|6666666.59331.peg.898	fig|6666666.59332.peg.6282	fig|6666666.59334.peg.1523	fig|6666666.59335.peg.2503	fig|6666666.59336.peg.2581	fig|6666666.59333.peg.5298	fig|6666666.59338.peg.4935	fig|6666666.59339.peg.392	None	fig|6666666.59337.peg.4386	fig|6666666.59342.peg.4617	fig|6666666.71181.peg.4713	fig|6666666.59344.peg.4581	fig|6666666.59345.peg.244	fig|6666666.59341.peg.4444	fig|6666666.59347.peg.4712	fig|6666666.59346.peg.4890	fig|6666666.59348.peg.1576	fig|6666666.59349.peg.479	fig|6666666.59350.peg.1120	fig|6666666.59351.peg.1649	fig|6666666.59353.peg.2094	fig|6666666.59355.peg.4310	fig|6666666.59354.peg.5789

11239	Bacteriocin production protein	fig|6666666.59329.peg.3490	fig|6666666.59330.peg.5030	fig|6666666.59331.peg.1906	fig|6666666.59332.peg.2750	None	fig|6666666.59335.peg.3593	fig|6666666.59336.peg.3036	fig|6666666.59333.peg.2541	fig|6666666.59338.peg.2036	fig|6666666.59339.peg.2975	fig|6666666.59340.peg.388	fig|6666666.59337.peg.3730	fig|6666666.59342.peg.4065	fig|6666666.71181.peg.574	fig|6666666.59344.peg.1887	fig|6666666.59345.peg.1201	fig|6666666.59341.peg.2450	fig|6666666.59347.peg.2453	fig|6666666.59346.peg.56	fig|6666666.59348.peg.1717	fig|6666666.59349.peg.3462	fig|6666666.59350.peg.1436	fig|6666666.59351.peg.3574	fig|6666666.59353.peg.1206	fig|6666666.59355.peg.270	fig|6666666.59354.peg.2961

2394	SSU ribosomal protein S9p S16e	fig|6666666.59329.peg.2058	fig|6666666.59330.peg.962	fig|6666666.59331.peg.792	fig|6666666.59332.peg.650	fig|6666666.59334.peg.3194	fig|6666666.59335.peg.3435	fig|6666666.59336.peg.1588	fig|6666666.59333.peg.619	None	fig|6666666.59339.peg.4939	fig|6666666.59340.peg.1135	fig|6666666.59337.peg.28	fig|6666666.59342.peg.5747	fig|6666666.71181.peg.1041	fig|6666666.59344.peg.1823	fig|6666666.59345.peg.3814	fig|6666666.59341.peg.3548	fig|6666666.59347.peg.3921	fig|6666666.59346.peg.2337	fig|6666666.59348.peg.1449	fig|6666666.59349.peg.40	fig|6666666.59350.peg.1116	fig|6666666.59351.peg.3460	fig|6666666.59353.peg.2359	fig|6666666.59355.peg.1632	fig|6666666.59354.peg.357

4966	FIG000506 Hypothetical ATP-binding protein	fig|6666666.59329.peg.813	fig|6666666.59330.peg.42	fig|6666666.59331.peg.6007	fig|6666666.59332.peg.2744	fig|6666666.59334.peg.106	fig|6666666.59335.peg.3056	fig|6666666.59336.peg.345	fig|6666666.59333.peg.258	fig|6666666.59338.peg.101	fig|6666666.59339.peg.8208	None	fig|6666666.59337.peg.826	fig|6666666.59342.peg.5753	fig|6666666.71181.peg.1013	fig|6666666.59344.peg.1792	fig|6666666.59345.peg.4864	fig|6666666.59341.peg.1239	fig|6666666.59347.peg.3256	fig|6666666.59346.peg.644	fig|6666666.59348.peg.3758	fig|6666666.59349.peg.17	fig|6666666.59350.peg.1629	fig|6666666.59351.peg.3435	fig|6666666.59353.peg.2331	fig|6666666.59355.peg.1604	fig|6666666.59354.peg.3615

2179	ADP-heptose--lipooligosaccharide heptosyltransferase, putative	fig|6666666.59329.peg.834	fig|6666666.59330.peg.1357	fig|6666666.59331.peg.6134	fig|6666666.59332.peg.1731	fig|6666666.59334.peg.183	fig|6666666.59335.peg.2003	fig|6666666.59336.peg.4202	fig|6666666.59333.peg.470	fig|6666666.59338.peg.1909	fig|6666666.59339.peg.6310	fig|6666666.59340.peg.2113	fig|6666666.59337.peg.464	fig|6666666.59342.peg.2402	fig|6666666.71181.peg.3666	fig|6666666.59344.peg.954	fig|6666666.59345.peg.2820	fig|6666666.59341.peg.4927	fig|6666666.59347.peg.5178	fig|6666666.59346.peg.1707	fig|6666666.59348.peg.1749	fig|6666666.59349.peg.2787	None	fig|6666666.59351.peg.5204	fig|6666666.59353.peg.2586	fig|6666666.59355.peg.2621	fig|6666666.59354.peg.2870

5120	Nucleoside 5-triphosphatase RdgB dHAPTP, dITP, XTP-specific EC 3.6.1.15	fig|6666666.59329.peg.1540	fig|6666666.59330.peg.337	fig|6666666.59331.peg.4369	fig|6666666.59332.peg.1634	fig|6666666.59334.peg.1367	fig|6666666.59335.peg.82	fig|6666666.59336.peg.4101	fig|6666666.59333.peg.762	fig|6666666.59338.peg.2883	fig|6666666.59339.peg.7815	fig|6666666.59340.peg.3617	fig|6666666.59337.peg.518	None	fig|6666666.71181.peg.1869	fig|6666666.59344.peg.4344	fig|6666666.59345.peg.5007	fig|6666666.59341.peg.3060	fig|6666666.59347.peg.3870	fig|6666666.59346.peg.1775	fig|6666666.59348.peg.229	fig|6666666.59349.peg.796	fig|6666666.59350.peg.6027	fig|6666666.59351.peg.3526	fig|6666666.59353.peg.3235	fig|6666666.59355.peg.3117	fig|6666666.59354.peg.530

10376	Glutathione S-transferase, omega EC 2.5.1.18	fig|6666666.59329.peg.4404	fig|6666666.59330.peg.4364	fig|6666666.59331.peg.4902	fig|6666666.59332.peg.1739	fig|6666666.59334.peg.4334	fig|6666666.59335.peg.3748	fig|6666666.59336.peg.2645	fig|6666666.59333.peg.1321	fig|6666666.59338.peg.5889	fig|6666666.59339.peg.3416	fig|6666666.59340.peg.2179	fig|6666666.59337.peg.4928	fig|6666666.59342.peg.3931	fig|6666666.71181.peg.4402	fig|6666666.59344.peg.1851	fig|6666666.59345.peg.1798	fig|6666666.59341.peg.3136	fig|6666666.59347.peg.5132	fig|6666666.59346.peg.543	None	fig|6666666.59349.peg.4050	fig|6666666.59350.peg.1379	fig|6666666.59351.peg.1863	fig|6666666.59353.peg.2380	fig|6666666.59355.peg.1652	fig|6666666.59354.peg.4800

600	DNA polymerase IV EC 2.7.7.7	fig|6666666.59329.peg.4431	fig|6666666.59330.peg.4054	fig|6666666.59331.peg.2150	fig|6666666.59332.peg.1664	fig|6666666.59334.peg.4557	fig|6666666.59335.peg.129	fig|6666666.59336.peg.2774	fig|6666666.59333.peg.1808	fig|6666666.59338.peg.272	fig|6666666.59339.peg.5059	fig|6666666.59340.peg.949	fig|6666666.59337.peg.1552	None	fig|6666666.71181.peg.6365	fig|6666666.59344.peg.4322	fig|6666666.59345.peg.2228	fig|6666666.59341.peg.2299	fig|6666666.59347.peg.2794	fig|6666666.59346.peg.4254	fig|6666666.59348.peg.2144	fig|6666666.59349.peg.1209	fig|6666666.59350.peg.3114	fig|6666666.59351.peg.4274	fig|6666666.59353.peg.889	fig|6666666.59355.peg.4585	fig|6666666.59354.peg.2794

8272	Alanine racemase, biosynthetic EC 5.1.1.1	fig|6666666.59329.peg.326	fig|6666666.59330.peg.2645	fig|6666666.59331.peg.4183	fig|6666666.59332.peg.4340	fig|6666666.59334.peg.165	fig|6666666.59335.peg.3486	fig|6666666.59336.peg.602	fig|6666666.59333.peg.885	None	fig|6666666.59339.peg.7702	fig|6666666.59340.peg.576	fig|6666666.59337.peg.1711	fig|6666666.59342.peg.5626	fig|6666666.71181.peg.1992	fig|6666666.59344.peg.2751	fig|6666666.59345.peg.5370	fig|6666666.59341.peg.2414	fig|6666666.59347.peg.5373	fig|6666666.59346.peg.2391	fig|6666666.59348.peg.3105	fig|6666666.59349.peg.3242	fig|6666666.59350.peg.3891	fig|6666666.59351.peg.5437	fig|6666666.59353.peg.2989	fig|6666666.59355.peg.2895	fig|6666666.59354.peg.5103

5915	Flagellin protein FlaA	fig|6666666.59329.peg.6529	fig|6666666.59330.peg.4135	fig|6666666.59331.peg.5814	fig|6666666.59332.peg.2464	None	fig|6666666.59335.peg.375	fig|6666666.59336.peg.3470	fig|6666666.59333.peg.2861	fig|6666666.59338.peg.5388	fig|6666666.59339.peg.7115	fig|6666666.59340.peg.1100	fig|6666666.59337.peg.3361	fig|6666666.59342.peg.3200	fig|6666666.71181.peg.4037	fig|6666666.59344.peg.3046	fig|6666666.59345.peg.5463	fig|6666666.59341.peg.128	fig|6666666.59347.peg.2702	fig|6666666.59346.peg.1772	fig|6666666.59348.peg.5106	fig|6666666.59349.peg.773	fig|6666666.59350.peg.1655	fig|6666666.59351.peg.1996	fig|6666666.59353.peg.3517	fig|6666666.59355.peg.3659	fig|6666666.59354.peg.5259

2619	Aspartate aminotransferase EC 2.6.1.1	fig|6666666.59329.peg.6105	fig|6666666.59330.peg.4269	fig|6666666.59331.peg.1680	fig|6666666.59332.peg.3389	fig|6666666.59334.peg.2515	fig|6666666.59335.peg.1674	None	fig|6666666.59333.peg.4410	fig|6666666.59338.peg.2945	fig|6666666.59339.peg.2794	fig|6666666.59340.peg.3165	fig|6666666.59337.peg.3573	fig|6666666.59342.peg.5222	fig|6666666.71181.peg.5219	fig|6666666.59344.peg.2774	fig|6666666.59345.peg.852	fig|6666666.59341.peg.4425	fig|6666666.59347.peg.914	fig|6666666.59346.peg.4764	fig|6666666.59348.peg.2867	fig|6666666.59349.peg.2716	fig|6666666.59350.peg.49	fig|6666666.59351.peg.553	fig|6666666.59353.peg.1669	fig|6666666.59355.peg.1052	fig|6666666.59354.peg.109

10024	Cysteine desulfurase CsdA-CsdE, sulfur acceptor protein CsdE	fig|6666666.59329.peg.3333	fig|6666666.59330.peg.2802	fig|6666666.59331.peg.2677	fig|6666666.59332.peg.4507	fig|6666666.59334.peg.1167	fig|6666666.59335.peg.4550	fig|6666666.59336.peg.129	fig|6666666.59333.peg.5105	fig|6666666.59338.peg.4247	fig|6666666.59339.peg.2184	None	fig|6666666.59337.peg.2365	fig|6666666.59342.peg.1849	fig|6666666.71181.peg.2557	fig|6666666.59344.peg.2619	fig|6666666.59345.peg.3614	fig|6666666.59341.peg.533	fig|6666666.59347.peg.2816	fig|6666666.59346.peg.1847	fig|6666666.59348.peg.4248	fig|6666666.59349.peg.2514	fig|6666666.59350.peg.1982	fig|6666666.59351.peg.3720	fig|6666666.59353.peg.1359	fig|6666666.59355.peg.2513	fig|6666666.59354.peg.4782

741	Glutamate racemase EC 5.1.1.3	fig|6666666.59329.peg.5081	fig|6666666.59330.peg.4569	fig|6666666.59331.peg.2721	fig|6666666.59332.peg.576	fig|6666666.59334.peg.1477	fig|6666666.59335.peg.5811	fig|6666666.59336.peg.3237	fig|6666666.59333.peg.1463	fig|6666666.59338.peg.3309	fig|6666666.59339.peg.8600	fig|6666666.59340.peg.3133	fig|6666666.59337.peg.1669	fig|6666666.59342.peg.5310	fig|6666666.71181.peg.5066	fig|6666666.59344.peg.1029	fig|6666666.59345.peg.5638	fig|6666666.59341.peg.3569	fig|6666666.59347.peg.4658	None	fig|6666666.59348.peg.3644	fig|6666666.59349.peg.4289	fig|6666666.59350.peg.1730	fig|6666666.59351.peg.4327	fig|6666666.59353.peg.2514	fig|6666666.59355.peg.4872	fig|6666666.59354.peg.4942

743	Dihydropteroate synthase EC 2.5.1.15	fig|6666666.59329.peg.3479	fig|6666666.59330.peg.112	None	fig|6666666.59332.peg.5065	fig|6666666.59334.peg.204	fig|6666666.59335.peg.2849	fig|6666666.59336.peg.224	fig|6666666.59333.peg.1491	fig|6666666.59338.peg.1499	fig|6666666.59339.peg.4862	fig|6666666.59340.peg.1221	fig|6666666.59337.peg.678	fig|6666666.59342.peg.373	fig|6666666.71181.peg.4093	fig|6666666.59344.peg.2104	fig|6666666.59345.peg.2361	fig|6666666.59341.peg.3948	fig|6666666.59347.peg.3009	fig|6666666.59346.peg.293	fig|6666666.59348.peg.1543	fig|6666666.59349.peg.3696	fig|6666666.59350.peg.2893	fig|6666666.59351.peg.4940	fig|6666666.59353.peg.3138	fig|6666666.59355.peg.458	fig|6666666.59354.peg.1196

9800	6-phospho-beta-glucosidase EC 3.2.1.86	fig|6666666.59329.peg.1673	fig|6666666.59330.peg.3596	fig|6666666.59331.peg.1561	fig|6666666.59332.peg.2712	fig|6666666.59334.peg.4649	fig|6666666.59335.peg.3803	fig|6666666.59336.peg.2964	fig|6666666.59333.peg.2028	fig|6666666.59338.peg.2949	fig|6666666.59339.peg.5525	fig|6666666.59340.peg.2634	fig|6666666.59337.peg.170	fig|6666666.59342.peg.6276	fig|6666666.71181.peg.5772	fig|6666666.59344.peg.5763	fig|6666666.59345.peg.3020	fig|6666666.59341.peg.2148	fig|6666666.59347.peg.607	fig|6666666.59346.peg.2699	fig|6666666.59348.peg.1662	fig|6666666.59349.peg.2052	fig|6666666.59350.peg.3803	None	fig|6666666.59353.peg.5035	fig|6666666.59355.peg.2994	fig|6666666.59354.peg.728

5353	Uridine kinase EC 2.7.1.48 [C1]	fig|6666666.59329.peg.1601	fig|6666666.59330.peg.3713	fig|6666666.59331.peg.703	fig|6666666.59332.peg.2120	fig|6666666.59334.peg.4893	fig|6666666.59335.peg.3113	fig|6666666.59336.peg.3550	fig|6666666.59333.peg.1544	fig|6666666.59338.peg.4855	fig|6666666.59339.peg.163	fig|6666666.59340.peg.2354	fig|6666666.59337.peg.3849	fig|6666666.59342.peg.3334	fig|6666666.71181.peg.207	fig|6666666.59344.peg.255	fig|6666666.59345.peg.1079	fig|6666666.59341.peg.2391	fig|6666666.59347.peg.2618	fig|6666666.59346.peg.3934	fig|6666666.59348.peg.4674	fig|6666666.59349.peg.4716	None	fig|6666666.59351.peg.1195	fig|6666666.59353.peg.614	fig|6666666.59355.peg.96	fig|6666666.59354.peg.3747

3111	ApaG protein	fig|6666666.59329.peg.133	fig|6666666.59330.peg.819	fig|6666666.59331.peg.2696	fig|6666666.59332.peg.5232	fig|6666666.59334.peg.3347	fig|6666666.59335.peg.4482	fig|6666666.59336.peg.2655	fig|6666666.59333.peg.5403	fig|6666666.59338.peg.910	fig|6666666.59339.peg.7891	fig|6666666.59340.peg.432	fig|6666666.59337.peg.1383	fig|6666666.59342.peg.2177	fig|6666666.71181.peg.402	fig|6666666.59344.peg.1292	fig|6666666.59345.peg.2460	fig|6666666.59341.peg.2001	fig|6666666.59347.peg.3090	fig|6666666.59346.peg.4900	fig|6666666.59348.peg.176	fig|6666666.59349.peg.1875	None	fig|6666666.59351.peg.3649	fig|6666666.59353.peg.96	fig|6666666.59355.peg.706	fig|6666666.59354.peg.5333

2137	Cytochrome d ubiquinol oxidase subunit I EC 1.10.3.-	fig|6666666.59329.peg.2855	fig|6666666.59330.peg.2491	fig|6666666.59331.peg.896	fig|6666666.59332.peg.1964	fig|6666666.59334.peg.1524	fig|6666666.59335.peg.2502	fig|6666666.59336.peg.2702	fig|6666666.59333.peg.2554	fig|6666666.59338.peg.5809	fig|6666666.59339.peg.388	None	fig|6666666.59337.peg.4387	fig|6666666.59342.peg.4618	fig|6666666.71181.peg.3040	fig|6666666.59344.peg.5798	fig|6666666.59345.peg.243	fig|6666666.59341.peg.4443	fig|6666666.59347.peg.4202	fig|6666666.59346.peg.4891	fig|6666666.59348.peg.1578	fig|6666666.59349.peg.481	fig|6666666.59350.peg.1122	fig|6666666.59351.peg.1650	fig|6666666.59353.peg.2093	fig|6666666.59355.peg.2729	fig|6666666.59354.peg.5790

2378	Threonine synthase EC 4.2.3.1	fig|6666666.59329.peg.108	fig|6666666.59330.peg.1724	None	fig|6666666.59332.peg.2902	fig|6666666.59334.peg.5056	fig|6666666.59335.peg.5128	fig|6666666.59336.peg.3406	fig|6666666.59333.peg.3415	fig|6666666.59338.peg.4461	fig|6666666.59339.peg.7418	fig|6666666.59340.peg.2150	fig|6666666.59337.peg.93	fig|6666666.59342.peg.4470	fig|6666666.71181.peg.434	fig|6666666.59344.peg.1337	fig|6666666.59345.peg.3750	fig|6666666.59341.peg.90	fig|6666666.59347.peg.4034	fig|6666666.59346.peg.1372	fig|6666666.59348.peg.216	fig|6666666.59349.peg.3476	fig|6666666.59350.peg.2029	fig|6666666.59351.peg.4256	fig|6666666.59353.peg.64	fig|6666666.59355.peg.5617	fig|6666666.59354.peg.2935

6124	ATP synthase A chain EC 3.6.3.14	None	fig|6666666.59330.peg.605	fig|6666666.59331.peg.400	fig|6666666.59332.peg.99	fig|6666666.59334.peg.27	fig|6666666.59335.peg.792	fig|6666666.59336.peg.28	fig|6666666.59333.peg.29	fig|6666666.59338.peg.493	fig|6666666.59339.peg.2286	fig|6666666.59340.peg.2443	fig|6666666.59337.peg.2082	fig|6666666.59342.peg.4607	fig|6666666.71181.peg.592	fig|6666666.59344.peg.4265	fig|6666666.59345.peg.4965	fig|6666666.59341.peg.1059	fig|6666666.59347.peg.2032	fig|6666666.59346.peg.1403	fig|6666666.59348.peg.332	fig|6666666.59349.peg.1030	fig|6666666.59350.peg.2542	fig|6666666.59351.peg.5359	fig|6666666.59353.peg.3088	fig|6666666.59355.peg.2260	fig|6666666.59354.peg.1152

4120	tRNA dihydrouridine synthase A	fig|6666666.59329.peg.1856	fig|6666666.59330.peg.4452	fig|6666666.59331.peg.5233	fig|6666666.59332.peg.1878	fig|6666666.59334.peg.162	fig|6666666.59335.peg.331	fig|6666666.59336.peg.609	fig|6666666.59333.peg.879	fig|6666666.59338.peg.319	fig|6666666.59339.peg.8324	fig|6666666.59340.peg.425	fig|6666666.59337.peg.1719	fig|6666666.59342.peg.2483	fig|6666666.71181.peg.1997	fig|6666666.59344.peg.2745	fig|6666666.59345.peg.5364	fig|6666666.59341.peg.1434	fig|6666666.59347.peg.4909	fig|6666666.59346.peg.3205	None	fig|6666666.59349.peg.92	fig|6666666.59350.peg.3582	fig|6666666.59351.peg.150	fig|6666666.59353.peg.2994	fig|6666666.59355.peg.2900	fig|6666666.59354.peg.430

10006	NAD kinase EC 2.7.1.23	fig|6666666.59329.peg.1683	fig|6666666.59330.peg.4600	None	fig|6666666.59332.peg.384	fig|6666666.59334.peg.1387	fig|6666666.59335.peg.5166	fig|6666666.59336.peg.222	fig|6666666.59333.peg.2756	fig|6666666.59338.peg.1079	fig|6666666.59339.peg.7276	fig|6666666.59340.peg.1195	fig|6666666.59337.peg.4970	fig|6666666.59342.peg.855	fig|6666666.71181.peg.1440	fig|6666666.59344.peg.1546	fig|6666666.59345.peg.3574	fig|6666666.59341.peg.4895	fig|6666666.59347.peg.4328	fig|6666666.59346.peg.691	fig|6666666.59348.peg.5165	fig|6666666.59349.peg.2385	fig|6666666.59350.peg.7044	fig|6666666.59351.peg.644	fig|6666666.59353.peg.4354	fig|6666666.59355.peg.4489	fig|6666666.59354.peg.4838

9042	Thiol disulfide interchange protein DsbC	fig|6666666.59329.peg.213	fig|6666666.59330.peg.1740	fig|6666666.59331.peg.103	fig|6666666.59332.peg.3111	fig|6666666.59334.peg.2525	fig|6666666.59335.peg.2293	fig|6666666.59336.peg.2603	fig|6666666.59333.peg.1666	fig|6666666.59338.peg.200	fig|6666666.59339.peg.4153	None	fig|6666666.59337.peg.108	fig|6666666.59342.peg.3776	fig|6666666.71181.peg.1495	fig|6666666.59344.peg.3676	fig|6666666.59345.peg.1908	fig|6666666.59341.peg.3107	fig|6666666.59347.peg.649	fig|6666666.59346.peg.3099	fig|6666666.59348.peg.667	fig|6666666.59349.peg.4390	fig|6666666.59350.peg.1155	fig|6666666.59351.peg.4033	fig|6666666.59353.peg.3839	fig|6666666.59355.peg.248	fig|6666666.59354.peg.3236

2969	Outer membrane protein N, non-specific porin	fig|6666666.59329.peg.6119	fig|6666666.59330.peg.721	fig|6666666.59331.peg.5629	fig|6666666.59332.peg.3949	fig|6666666.59334.peg.3127	fig|6666666.59335.peg.2841	fig|6666666.59336.peg.1386	fig|6666666.59333.peg.4033	fig|6666666.59338.peg.5608	fig|6666666.59339.peg.2408	fig|6666666.59340.peg.1364	fig|6666666.59337.peg.3776	fig|6666666.59342.peg.1502	fig|6666666.71181.peg.4684	fig|6666666.59344.peg.5358	fig|6666666.59345.peg.1979	fig|6666666.59341.peg.5815	fig|6666666.59347.peg.1490	fig|6666666.59346.peg.4211	fig|6666666.59348.peg.4653	fig|6666666.59349.peg.2407	None	fig|6666666.59351.peg.353	fig|6666666.59353.peg.1126	fig|6666666.59355.peg.4926	fig|6666666.59354.peg.1005

2979	Probable protease htpX homolog EC 3.4.24.-	fig|6666666.59329.peg.2099	fig|6666666.59330.peg.2881	fig|6666666.59331.peg.3305	fig|6666666.59332.peg.4305	fig|6666666.59334.peg.5393	fig|6666666.59335.peg.611	fig|6666666.59336.peg.4951	fig|6666666.59333.peg.3151	fig|6666666.59338.peg.1411	fig|6666666.59339.peg.469	fig|6666666.59340.peg.2253	fig|6666666.59337.peg.4604	fig|6666666.59342.peg.2456	fig|6666666.71181.peg.3838	fig|6666666.59344.peg.842	fig|6666666.59345.peg.1325	fig|6666666.59341.peg.479	fig|6666666.59347.peg.4121	fig|6666666.59346.peg.1565	fig|6666666.59348.peg.4416	fig|6666666.59349.peg.1614	None	fig|6666666.59351.peg.3684	fig|6666666.59353.peg.1272	fig|6666666.59355.peg.3311	fig|6666666.59354.peg.1863

3737	Glycerol-3-phosphate dehydrogenase [NAD P +] EC 1.1.1.94	fig|6666666.59329.peg.31	fig|6666666.59330.peg.144	fig|6666666.59331.peg.3445	fig|6666666.59332.peg.3274	fig|6666666.59334.peg.3148	fig|6666666.59335.peg.447	fig|6666666.59336.peg.269	fig|6666666.59333.peg.2293	fig|6666666.59338.peg.3745	fig|6666666.59339.peg.6415	None	fig|6666666.59337.peg.4994	fig|6666666.59342.peg.5723	fig|6666666.71181.peg.4020	fig|6666666.59344.peg.974	fig|6666666.59345.peg.5097	fig|6666666.59341.peg.182	fig|6666666.59347.peg.4545	fig|6666666.59346.peg.4159	fig|6666666.59348.peg.138	fig|6666666.59349.peg.2490	fig|6666666.59350.peg.1753	fig|6666666.59351.peg.376	fig|6666666.59353.peg.2565	fig|6666666.59355.peg.5452	fig|6666666.59354.peg.67

7639	Lipid A biosynthesis lauroyl acyltransferase EC 2.3.1.-	fig|6666666.59329.peg.4154	fig|6666666.59330.peg.5143	fig|6666666.59331.peg.5207	fig|6666666.59332.peg.5584	fig|6666666.59334.peg.5291	fig|6666666.59335.peg.1988	fig|6666666.59336.peg.4651	fig|6666666.59333.peg.472	fig|6666666.59338.peg.1117	fig|6666666.59339.peg.6282	fig|6666666.59340.peg.670	fig|6666666.59337.peg.618	fig|6666666.59342.peg.1578	fig|6666666.71181.peg.1928	fig|6666666.59344.peg.5348	fig|6666666.59345.peg.2593	fig|6666666.59341.peg.227	fig|6666666.59347.peg.4516	fig|6666666.59346.peg.1396	fig|6666666.59348.peg.734	fig|6666666.59349.peg.814	None	fig|6666666.59351.peg.3542	fig|6666666.59353.peg.288	fig|6666666.59355.peg.4826	fig|6666666.59354.peg.656

1453	MG 2+ CHELATASE FAMILY PROTEIN ComM-related protein	fig|6666666.59329.peg.1490	fig|6666666.59330.peg.625	fig|6666666.59331.peg.3339	fig|6666666.59332.peg.212	fig|6666666.59334.peg.4506	fig|6666666.59335.peg.2450	fig|6666666.59336.peg.2733	fig|6666666.59333.peg.9	fig|6666666.59338.peg.568	fig|6666666.59339.peg.8069	fig|6666666.59340.peg.1083	fig|6666666.59337.peg.921	fig|6666666.59342.peg.4812	fig|6666666.71181.peg.5028	fig|6666666.59344.peg.4234	fig|6666666.59345.peg.4943	fig|6666666.59341.peg.1936	fig|6666666.59347.peg.4764	fig|6666666.59346.peg.4407	fig|6666666.59348.peg.67	fig|6666666.59349.peg.4055	None	fig|6666666.59351.peg.3398	fig|6666666.59353.peg.4271	fig|6666666.59355.peg.4325	fig|6666666.59354.peg.5362

5317	Octaprenyl-diphosphate synthase EC 2.5.1.- Dimethylallyltransferase EC 2.5.1.1 Geranyltranstransferase farnesyldiphosphate synthase EC 2.5.1.10 Geranylgeranyl pyrophosphate synthetase EC 2.5.1.29	fig|6666666.59329.peg.142	fig|6666666.59330.peg.827	None	fig|6666666.59332.peg.5644	fig|6666666.59334.peg.4165	fig|6666666.59335.peg.3897	fig|6666666.59336.peg.3166	fig|6666666.59333.peg.4914	fig|6666666.59338.peg.1822	fig|6666666.59339.peg.7172	fig|6666666.59340.peg.3020	fig|6666666.59337.peg.1373	fig|6666666.59342.peg.3899	fig|6666666.71181.peg.2721	fig|6666666.59344.peg.2119	fig|6666666.59345.peg.2469	fig|6666666.59341.peg.2519	fig|6666666.59347.peg.2877	fig|6666666.59346.peg.3984	fig|6666666.59348.peg.164	fig|6666666.59349.peg.2779	fig|6666666.59350.peg.856	fig|6666666.59351.peg.300	fig|6666666.59353.peg.771	fig|6666666.59355.peg.933	fig|6666666.59354.peg.4066

4679	23S rRNA Uracil-5- -methyltransferase RumA EC 2.1.1.-	fig|6666666.59329.peg.2302	fig|6666666.59330.peg.2471	None	fig|6666666.59332.peg.570	fig|6666666.59334.peg.325	fig|6666666.59335.peg.4035	fig|6666666.59336.peg.2783	fig|6666666.59333.peg.1466	fig|6666666.59338.peg.3378	fig|6666666.59339.peg.7381	fig|6666666.59340.peg.229	fig|6666666.59337.peg.1666	fig|6666666.59342.peg.4602	fig|6666666.71181.peg.5583	fig|6666666.59344.peg.367	fig|6666666.59345.peg.5063	fig|6666666.59341.peg.1326	fig|6666666.59347.peg.4653	fig|6666666.59346.peg.341	fig|6666666.59348.peg.2988	fig|6666666.59349.peg.4291	fig|6666666.59350.peg.7077	fig|6666666.59351.peg.2591	fig|6666666.59353.peg.177	fig|6666666.59355.peg.2945	fig|6666666.59354.peg.913

8188	Ribonuclease PH EC 2.7.7.56	fig|6666666.59329.peg.3643	fig|6666666.59330.peg.1174	fig|6666666.59331.peg.2612	fig|6666666.59332.peg.717	fig|6666666.59334.peg.229	fig|6666666.59335.peg.1985	fig|6666666.59336.peg.4649	fig|6666666.59333.peg.664	fig|6666666.59338.peg.4346	fig|6666666.59339.peg.6276	None	fig|6666666.59337.peg.479	fig|6666666.59342.peg.6194	fig|6666666.71181.peg.2612	fig|6666666.59344.peg.933	fig|6666666.59345.peg.2627	fig|6666666.59341.peg.6061	fig|6666666.59347.peg.5194	fig|6666666.59346.peg.1394	fig|6666666.59348.peg.736	fig|6666666.59349.peg.3070	fig|6666666.59350.peg.1499	fig|6666666.59351.peg.332	fig|6666666.59353.peg.2599	fig|6666666.59355.peg.2603	fig|6666666.59354.peg.3331

10748	Membrane alanine aminopeptidase N EC 3.4.11.2	fig|6666666.59329.peg.4711	fig|6666666.59330.peg.1009	None	fig|6666666.59332.peg.872	fig|6666666.59334.peg.934	fig|6666666.59335.peg.5674	fig|6666666.59336.peg.6498	fig|6666666.59333.peg.2666	fig|6666666.59338.peg.1772	fig|6666666.59339.peg.1534	fig|6666666.59340.peg.3656	fig|6666666.59337.peg.3151	fig|6666666.59342.peg.3552	fig|6666666.71181.peg.4353	fig|6666666.59344.peg.2767	fig|6666666.59345.peg.3259	fig|6666666.59341.peg.901	fig|6666666.59347.peg.1772	fig|6666666.59346.peg.3397	fig|6666666.59348.peg.4567	fig|6666666.59349.peg.3350	fig|6666666.59350.peg.7623	fig|6666666.59351.peg.5021	fig|6666666.59353.peg.1674	fig|6666666.59355.peg.1047	fig|6666666.59354.peg.5357

10600	FIG01200109 hypothetical protein	fig|6666666.59329.peg.5405	fig|6666666.59330.peg.3818	fig|6666666.59331.peg.3528	fig|6666666.59332.peg.478	fig|6666666.59334.peg.1418	fig|6666666.59335.peg.3858	fig|6666666.59336.peg.6314	fig|6666666.59333.peg.4899	fig|6666666.59338.peg.1591	fig|6666666.59339.peg.1195	fig|6666666.59340.peg.2704	fig|6666666.59337.peg.2710	fig|6666666.59342.peg.5160	fig|6666666.71181.peg.2647	fig|6666666.59344.peg.3921	fig|6666666.59345.peg.5495	fig|6666666.59341.peg.3788	fig|6666666.59347.peg.1808	fig|6666666.59346.peg.2670	fig|6666666.59348.peg.2096	fig|6666666.59349.peg.3739	None	fig|6666666.59351.peg.2811	fig|6666666.59353.peg.4759	fig|6666666.59355.peg.4045	fig|6666666.59354.peg.3049

3174	YcfF hinT protein a purine nucleoside phosphoramidase	fig|6666666.59329.peg.2755	fig|6666666.59330.peg.4067	fig|6666666.59331.peg.2784	fig|6666666.59332.peg.4705	fig|6666666.59334.peg.3122	fig|6666666.59335.peg.1305	fig|6666666.59336.peg.1617	fig|6666666.59333.peg.5031	fig|6666666.59338.peg.1572	fig|6666666.59339.peg.3439	None	fig|6666666.59337.peg.2176	fig|6666666.59342.peg.488	fig|6666666.71181.peg.1637	fig|6666666.59344.peg.5022	fig|6666666.59345.peg.1608	fig|6666666.59341.peg.272	fig|6666666.59347.peg.4066	fig|6666666.59346.peg.2180	fig|6666666.59348.peg.810	fig|6666666.59349.peg.2209	fig|6666666.59350.peg.6937	fig|6666666.59351.peg.5018	fig|6666666.59353.peg.330	fig|6666666.59355.peg.1369	fig|6666666.59354.peg.1618

2314	FIG002708 Protein SirB1	fig|6666666.59329.peg.2439	fig|6666666.59330.peg.4526	fig|6666666.59331.peg.5506	fig|6666666.59332.peg.2618	fig|6666666.59334.peg.3883	fig|6666666.59335.peg.1182	fig|6666666.59336.peg.5116	fig|6666666.59333.peg.229	fig|6666666.59338.peg.2088	fig|6666666.59339.peg.14	fig|6666666.59340.peg.743	fig|6666666.59337.peg.5170	fig|6666666.59342.peg.456	fig|6666666.71181.peg.5099	fig|6666666.59344.peg.113	fig|6666666.59345.peg.5420	fig|6666666.59341.peg.3047	fig|6666666.59347.peg.5276	fig|6666666.59346.peg.3194	fig|6666666.59348.peg.2374	fig|6666666.59349.peg.2619	None	fig|6666666.59351.peg.4658	fig|6666666.59353.peg.435	fig|6666666.59355.peg.2162	fig|6666666.59354.peg.2790

5850	Flagellar basal-body rod protein FlgB	fig|6666666.59329.peg.2326	fig|6666666.59330.peg.4418	fig|6666666.59331.peg.2029	fig|6666666.59332.peg.1795	None	fig|6666666.59335.peg.896	fig|6666666.59336.peg.6159	fig|6666666.59333.peg.2835	fig|6666666.59338.peg.631	fig|6666666.59339.peg.67	fig|6666666.59340.peg.3254	fig|6666666.59337.peg.3347	fig|6666666.59342.peg.3073	fig|6666666.71181.peg.2230	fig|6666666.59344.peg.3033	fig|6666666.59345.peg.5449	fig|6666666.59341.peg.2182	fig|6666666.59347.peg.2714	fig|6666666.59346.peg.1762	fig|6666666.59348.peg.948	fig|6666666.59349.peg.721	fig|6666666.59350.peg.5405	fig|6666666.59351.peg.1982	fig|6666666.59353.peg.3527	fig|6666666.59355.peg.3648	fig|6666666.59354.peg.1524

5859	Glycyl-tRNA synthetase beta chain EC 6.1.1.14	fig|6666666.59329.peg.3102	fig|6666666.59330.peg.1095	fig|6666666.59331.peg.4245	fig|6666666.59332.peg.77	fig|6666666.59334.peg.144	fig|6666666.59335.peg.3925	fig|6666666.59336.peg.2	fig|6666666.59333.peg.3476	fig|6666666.59338.peg.5459	fig|6666666.59339.peg.8045	fig|6666666.59340.peg.3191	fig|6666666.59337.peg.867	None	fig|6666666.71181.peg.659	fig|6666666.59344.peg.4131	fig|6666666.59345.peg.4986	fig|6666666.59341.peg.5113	fig|6666666.59347.peg.4804	fig|6666666.59346.peg.4286	fig|6666666.59348.peg.1207	fig|6666666.59349.peg.992	fig|6666666.59350.peg.2886	fig|6666666.59351.peg.5034	fig|6666666.59353.peg.4110	fig|6666666.59355.peg.2329	fig|6666666.59354.peg.575

7261	tRNA pseudouridine 13 synthase EC 4.2.1.-	fig|6666666.59329.peg.4461	fig|6666666.59330.peg.2460	fig|6666666.59331.peg.4166	fig|6666666.59332.peg.201	fig|6666666.59334.peg.270	fig|6666666.59335.peg.1658	fig|6666666.59336.peg.4461	fig|6666666.59333.peg.1711	fig|6666666.59338.peg.221	fig|6666666.59339.peg.7919	fig|6666666.59340.peg.1526	fig|6666666.59337.peg.581	fig|6666666.59342.peg.1273	fig|6666666.71181.peg.3418	fig|6666666.59344.peg.2195	fig|6666666.59345.peg.5071	fig|6666666.59341.peg.658	fig|6666666.59347.peg.3951	fig|6666666.59346.peg.3146	fig|6666666.59348.peg.4437	fig|6666666.59349.peg.4383	None	fig|6666666.59351.peg.5476	fig|6666666.59353.peg.211	fig|6666666.59355.peg.2980	fig|6666666.59354.peg.3142

5995	Proposed peptidoglycan lipid II flippase MurJ	fig|6666666.59329.peg.4772	fig|6666666.59330.peg.2510	fig|6666666.59331.peg.19	fig|6666666.59332.peg.3312	fig|6666666.59334.peg.2146	fig|6666666.59335.peg.1011	None	fig|6666666.59333.peg.3800	fig|6666666.59338.peg.2728	fig|6666666.59339.peg.7464	fig|6666666.59340.peg.2304	fig|6666666.59337.peg.131	fig|6666666.59342.peg.1396	fig|6666666.71181.peg.1905	fig|6666666.59344.peg.2537	fig|6666666.59345.peg.3712	fig|6666666.59341.peg.5675	fig|6666666.59347.peg.3022	fig|6666666.59346.peg.235	fig|6666666.59348.peg.1111	fig|6666666.59349.peg.566	fig|6666666.59350.peg.7336	fig|6666666.59351.peg.500	fig|6666666.59353.peg.1506	fig|6666666.59355.peg.2056	fig|6666666.59354.peg.5850

6782	Selenoprotein O and cysteine-containing homologs	fig|6666666.59329.peg.3033	fig|6666666.59330.peg.285	fig|6666666.59331.peg.5891	fig|6666666.59332.peg.3393	fig|6666666.59334.peg.3378	fig|6666666.59335.peg.5758	fig|6666666.59336.peg.4573	fig|6666666.59333.peg.3866	fig|6666666.59338.peg.4606	fig|6666666.59339.peg.3071	fig|6666666.59340.peg.1423	fig|6666666.59337.peg.1014	fig|6666666.59342.peg.4737	fig|6666666.71181.peg.2395	fig|6666666.59344.peg.778	fig|6666666.59345.peg.4266	fig|6666666.59341.peg.685	fig|6666666.59347.peg.5266	fig|6666666.59346.peg.3407	None	fig|6666666.59349.peg.3603	fig|6666666.59350.peg.7914	fig|6666666.59351.peg.4854	fig|6666666.59353.peg.1327	fig|6666666.59355.peg.2544	fig|6666666.59354.peg.3814

1246	FIG002082 Protein SirB2	fig|6666666.59329.peg.2440	fig|6666666.59330.peg.4524	fig|6666666.59331.peg.5507	fig|6666666.59332.peg.2619	fig|6666666.59334.peg.3881	fig|6666666.59335.peg.1181	fig|6666666.59336.peg.5117	fig|6666666.59333.peg.230	fig|6666666.59338.peg.951	fig|6666666.59339.peg.12	fig|6666666.59340.peg.742	fig|6666666.59337.peg.5169	fig|6666666.59342.peg.458	fig|6666666.71181.peg.5100	fig|6666666.59344.peg.112	fig|6666666.59345.peg.5419	fig|6666666.59341.peg.3046	fig|6666666.59347.peg.5277	fig|6666666.59346.peg.3193	fig|6666666.59348.peg.606	fig|6666666.59349.peg.2618	None	fig|6666666.59351.peg.4659	fig|6666666.59353.peg.434	fig|6666666.59355.peg.2161	fig|6666666.59354.peg.2788

243	Thymidylate synthase EC 2.1.1.45	fig|6666666.59329.peg.1937	fig|6666666.59330.peg.4672	fig|6666666.59331.peg.3442	fig|6666666.59332.peg.4746	fig|6666666.59334.peg.1744	fig|6666666.59335.peg.1018	fig|6666666.59336.peg.2361	fig|6666666.59333.peg.3950	fig|6666666.59338.peg.4192	fig|6666666.59339.peg.7455	fig|6666666.59340.peg.2480	fig|6666666.59337.peg.126	fig|6666666.59342.peg.3811	fig|6666666.71181.peg.70	fig|6666666.59344.peg.3197	fig|6666666.59345.peg.3717	fig|6666666.59341.peg.5244	fig|6666666.59347.peg.3030	fig|6666666.59346.peg.3087	None	fig|6666666.59349.peg.3878	fig|6666666.59350.peg.3811	fig|6666666.59351.peg.2580	fig|6666666.59353.peg.1945	fig|6666666.59355.peg.1462	fig|6666666.59354.peg.6332

3521	RNA polymerase associated protein RapA EC 3.6.1.-	fig|6666666.59329.peg.3426	fig|6666666.59330.peg.33	None	fig|6666666.59332.peg.1106	fig|6666666.59334.peg.2677	fig|6666666.59335.peg.242	fig|6666666.59336.peg.337	fig|6666666.59333.peg.245	fig|6666666.59338.peg.5770	fig|6666666.59339.peg.8155	fig|6666666.59340.peg.50	fig|6666666.59337.peg.818	fig|6666666.59342.peg.4283	fig|6666666.71181.peg.394	fig|6666666.59344.peg.1517	fig|6666666.59345.peg.4873	fig|6666666.59341.peg.895	fig|6666666.59347.peg.2004	fig|6666666.59346.peg.3808	fig|6666666.59348.peg.1300	fig|6666666.59349.peg.2595	fig|6666666.59350.peg.7574	fig|6666666.59351.peg.2539	fig|6666666.59353.peg.104	fig|6666666.59355.peg.714	fig|6666666.59354.peg.4301

2694	Flagellar basal-body P-ring formation protein FlgA	fig|6666666.59329.peg.573	fig|6666666.59330.peg.4949	fig|6666666.59331.peg.5626	fig|6666666.59332.peg.1799	None	fig|6666666.59335.peg.894	fig|6666666.59336.peg.6112	fig|6666666.59333.peg.2886	fig|6666666.59338.peg.92	fig|6666666.59339.peg.5308	fig|6666666.59340.peg.413	fig|6666666.59337.peg.5379	fig|6666666.59342.peg.3074	fig|6666666.71181.peg.2231	fig|6666666.59344.peg.3032	fig|6666666.59345.peg.5446	fig|6666666.59341.peg.4565	fig|6666666.59347.peg.4592	fig|6666666.59346.peg.1759	fig|6666666.59348.peg.4698	fig|6666666.59349.peg.718	fig|6666666.59350.peg.5476	fig|6666666.59351.peg.2562	fig|6666666.59353.peg.3528	fig|6666666.59355.peg.3647	fig|6666666.59354.peg.1520

10178	LSU ribosomal protein L24p L26e	fig|6666666.59329.peg.720	fig|6666666.59330.peg.561	fig|6666666.59331.peg.41	fig|6666666.59332.peg.2146	fig|6666666.59334.peg.1492	fig|6666666.59335.peg.4354	fig|6666666.59336.peg.153	fig|6666666.59333.peg.373	fig|6666666.59338.peg.1382	None	fig|6666666.59340.peg.63	fig|6666666.59337.peg.381	fig|6666666.59342.peg.311	fig|6666666.71181.peg.3449	fig|6666666.59344.peg.4932	fig|6666666.59345.peg.2528	fig|6666666.59341.peg.2505	fig|6666666.59347.peg.4830	fig|6666666.59346.peg.857	fig|6666666.59348.peg.1767	fig|6666666.59349.peg.3277	fig|6666666.59350.peg.3186	fig|6666666.59351.peg.4211	fig|6666666.59353.peg.4601	fig|6666666.59355.peg.4730	fig|6666666.59354.peg.3850

7448	Sigma factor RpoE negative regulatory protein RseB precursor	fig|6666666.59329.peg.498	fig|6666666.59330.peg.381	fig|6666666.59331.peg.5702	fig|6666666.59332.peg.733	fig|6666666.59334.peg.1872	fig|6666666.59335.peg.1720	fig|6666666.59336.peg.2105	fig|6666666.59333.peg.2804	fig|6666666.59338.peg.1511	fig|6666666.59339.peg.7361	fig|6666666.59340.peg.3733	fig|6666666.59337.peg.561	fig|6666666.59342.peg.2971	fig|6666666.71181.peg.1428	fig|6666666.59344.peg.1561	fig|6666666.59345.peg.5051	fig|6666666.59341.peg.5365	fig|6666666.59347.peg.3180	fig|6666666.59346.peg.330	fig|6666666.59348.peg.653	fig|6666666.59349.peg.4314	None	fig|6666666.59351.peg.594	fig|6666666.59353.peg.2647	fig|6666666.59355.peg.1835	fig|6666666.59354.peg.2811

4754	General secretion pathway protein G	fig|6666666.59329.peg.1222	fig|6666666.59330.peg.1967	fig|6666666.59331.peg.5781	fig|6666666.59332.peg.1672	fig|6666666.59334.peg.125	fig|6666666.59335.peg.210	fig|6666666.59336.peg.6353	fig|6666666.59333.peg.5457	fig|6666666.59338.peg.3287	fig|6666666.59339.peg.6204	fig|6666666.59340.peg.646	fig|6666666.59337.peg.4976	fig|6666666.59342.peg.2803	fig|6666666.71181.peg.1963	fig|6666666.59344.peg.1456	fig|6666666.59345.peg.2674	fig|6666666.59341.peg.1353	fig|6666666.59347.peg.4333	fig|6666666.59346.peg.4646	fig|6666666.59348.peg.1559	fig|6666666.59349.peg.234	None	fig|6666666.59351.peg.4344	fig|6666666.59353.peg.156	fig|6666666.59355.peg.767	fig|6666666.59354.peg.1580

1077	Phosphate starvation-inducible ATPase PhoH with RNA binding motif	fig|6666666.59329.peg.5598	fig|6666666.59330.peg.4035	fig|6666666.59331.peg.4178	fig|6666666.59332.peg.2938	fig|6666666.59334.peg.53	fig|6666666.59335.peg.1743	fig|6666666.59336.peg.4299	fig|6666666.59333.peg.2146	fig|6666666.59338.peg.2012	fig|6666666.59339.peg.5221	None	fig|6666666.59337.peg.1074	fig|6666666.59342.peg.1135	fig|6666666.71181.peg.5846	fig|6666666.59344.peg.5585	fig|6666666.59345.peg.3484	fig|6666666.59341.peg.1638	fig|6666666.59347.peg.4450	fig|6666666.59346.peg.2960	fig|6666666.59348.peg.3122	fig|6666666.59349.peg.4520	fig|6666666.59350.peg.570	fig|6666666.59351.peg.4672	fig|6666666.59353.peg.4671	fig|6666666.59355.peg.4444	fig|6666666.59354.peg.4075

9761	Transporter	fig|6666666.59329.peg.5783	fig|6666666.59330.peg.3369	fig|6666666.59331.peg.4868	fig|6666666.59332.peg.2058	fig|6666666.59334.peg.1590	fig|6666666.59335.peg.5676	fig|6666666.59336.peg.2958	fig|6666666.59333.peg.536	fig|6666666.59338.peg.3368	fig|6666666.59339.peg.799	None	fig|6666666.59337.peg.4523	fig|6666666.59342.peg.5321	fig|6666666.71181.peg.4413	fig|6666666.59344.peg.3429	fig|6666666.59345.peg.1388	fig|6666666.59341.peg.322	fig|6666666.59347.peg.1320	fig|6666666.59346.peg.3005	fig|6666666.59348.peg.2581	fig|6666666.59349.peg.4063	fig|6666666.59350.peg.19	fig|6666666.59351.peg.3405	fig|6666666.59353.peg.2714	fig|6666666.59355.peg.1766	fig|6666666.59354.peg.6413

11240	UDP-glucose dehydrogenase EC 1.1.1.22	fig|6666666.59329.peg.867	fig|6666666.59330.peg.3476	None	fig|6666666.59332.peg.4447	fig|6666666.59334.peg.4320	fig|6666666.59335.peg.5684	fig|6666666.59336.peg.1533	fig|6666666.59333.peg.5312	fig|6666666.59338.peg.164	fig|6666666.59339.peg.4243	fig|6666666.59340.peg.22	fig|6666666.59337.peg.416	fig|6666666.59342.peg.41	fig|6666666.71181.peg.2760	fig|6666666.59344.peg.415	fig|6666666.59345.peg.2560	fig|6666666.59341.peg.4137	fig|6666666.59347.peg.5023	fig|6666666.59346.peg.3053	fig|6666666.59348.peg.5302	fig|6666666.59349.peg.262	fig|6666666.59350.peg.3442	fig|6666666.59351.peg.248	fig|6666666.59353.peg.1742	fig|6666666.59355.peg.4017	fig|6666666.59354.peg.6054

10319	Twin-arginine translocation protein TatB	fig|6666666.59329.peg.5193	fig|6666666.59330.peg.254	fig|6666666.59331.peg.2431	fig|6666666.59332.peg.10	fig|6666666.59334.peg.2867	fig|6666666.59335.peg.44	fig|6666666.59336.peg.895	fig|6666666.59333.peg.2509	fig|6666666.59338.peg.2251	fig|6666666.59339.peg.7575	fig|6666666.59340.peg.3749	fig|6666666.59337.peg.962	fig|6666666.59342.peg.4557	fig|6666666.71181.peg.3337	fig|6666666.59344.peg.3411	fig|6666666.59345.peg.2711	fig|6666666.59341.peg.158	fig|6666666.59347.peg.4638	None	fig|6666666.59348.peg.112	fig|6666666.59349.peg.3415	fig|6666666.59350.peg.1451	fig|6666666.59351.peg.4997	fig|6666666.59353.peg.3801	fig|6666666.59355.peg.3397	fig|6666666.59354.peg.2226

1553	Ribonucleotide reductase of class Ia aerobic , alpha subunit EC 1.17.4.1	fig|6666666.59329.peg.5225	fig|6666666.59330.peg.2318	fig|6666666.59331.peg.1037	fig|6666666.59332.peg.4491	fig|6666666.59334.peg.5623	fig|6666666.59335.peg.4379	fig|6666666.59336.peg.5157	fig|6666666.59333.peg.5041	fig|6666666.59338.peg.3843	fig|6666666.59339.peg.942	None	fig|6666666.59337.peg.1753	fig|6666666.59342.peg.1078	fig|6666666.71181.peg.2621	fig|6666666.59344.peg.1726	fig|6666666.59345.peg.892	fig|6666666.59341.peg.3782	fig|6666666.59347.peg.2751	fig|6666666.59346.peg.1347	fig|6666666.59348.peg.4453	fig|6666666.59349.peg.384	fig|6666666.59350.peg.6771	fig|6666666.59351.peg.934	fig|6666666.59353.peg.4690	fig|6666666.59355.peg.342	fig|6666666.59354.peg.1764

1552	P pilus assembly Cpx signaling pathway, periplasmic inhibitor zinc- resistance associated protein	fig|6666666.59329.peg.2130	fig|6666666.59330.peg.168	fig|6666666.59331.peg.4672	fig|6666666.59332.peg.160	fig|6666666.59334.peg.1152	fig|6666666.59335.peg.3546	fig|6666666.59336.peg.3147	fig|6666666.59333.peg.418	fig|6666666.59338.peg.2365	fig|6666666.59339.peg.8698	fig|6666666.59340.peg.1602	fig|6666666.59337.peg.2278	fig|6666666.59342.peg.6367	fig|6666666.71181.peg.4014	fig|6666666.59344.peg.4205	fig|6666666.59345.peg.2029	fig|6666666.59341.peg.199	fig|6666666.59347.peg.4526	fig|6666666.59346.peg.792	fig|6666666.59348.peg.1647	fig|6666666.59349.peg.2590	None	fig|6666666.59351.peg.271	fig|6666666.59353.peg.2558	fig|6666666.59355.peg.4347	fig|6666666.59354.peg.4876

6420	Cell division protein BolA	fig|6666666.59329.peg.1035	fig|6666666.59330.peg.2788	None	fig|6666666.59332.peg.857	fig|6666666.59334.peg.880	fig|6666666.59335.peg.118	fig|6666666.59336.peg.4469	fig|6666666.59333.peg.1936	fig|6666666.59338.peg.1998	fig|6666666.59339.peg.5044	fig|6666666.59340.peg.2816	fig|6666666.59337.peg.2378	fig|6666666.59342.peg.2129	fig|6666666.71181.peg.2049	fig|6666666.59344.peg.1072	fig|6666666.59345.peg.2238	fig|6666666.59341.peg.5305	fig|6666666.59347.peg.2804	fig|6666666.59346.peg.2717	fig|6666666.59348.peg.1311	fig|6666666.59349.peg.325	fig|6666666.59350.peg.3028	fig|6666666.59351.peg.3933	fig|6666666.59353.peg.756	fig|6666666.59355.peg.948	fig|6666666.59354.peg.2168

224	Nucleoside-diphosphate-sugar epimerase	None	fig|6666666.59330.peg.1517	fig|6666666.59331.peg.5727	fig|6666666.59332.peg.3187	fig|6666666.59334.peg.587	fig|6666666.59335.peg.5996	fig|6666666.59336.peg.2600	fig|6666666.59333.peg.4468	fig|6666666.59338.peg.1126	fig|6666666.59339.peg.354	fig|6666666.59340.peg.1482	fig|6666666.59337.peg.2305	fig|6666666.59342.peg.4477	fig|6666666.71181.peg.252	fig|6666666.59344.peg.315	fig|6666666.59345.peg.1293	fig|6666666.59341.peg.3625	fig|6666666.59347.peg.2315	fig|6666666.59346.peg.4359	fig|6666666.59348.peg.2305	fig|6666666.59349.peg.495	fig|6666666.59350.peg.860	fig|6666666.59351.peg.3818	fig|6666666.59353.peg.566	fig|6666666.59355.peg.51	fig|6666666.59354.peg.940

4618	Heat shock protein HslJ	fig|6666666.59329.peg.1439	fig|6666666.59330.peg.3568	fig|6666666.59331.peg.5712	fig|6666666.59332.peg.1478	fig|6666666.59334.peg.2441	fig|6666666.59335.peg.5266	fig|6666666.59336.peg.2381	fig|6666666.59333.peg.1203	fig|6666666.59338.peg.507	fig|6666666.59339.peg.552	fig|6666666.59340.peg.1189	fig|6666666.59337.peg.3656	fig|6666666.59342.peg.1345	fig|6666666.71181.peg.1367	fig|6666666.59344.peg.558	fig|6666666.59345.peg.1236	fig|6666666.59341.peg.5123	fig|6666666.59347.peg.2170	fig|6666666.59346.peg.3495	fig|6666666.59348.peg.361	fig|6666666.59349.peg.3791	None	fig|6666666.59351.peg.3882	fig|6666666.59353.peg.1863	fig|6666666.59355.peg.1150	fig|6666666.59354.peg.6519

3344	33 kDa chaperonin Heat shock protein 33 HSP33	fig|6666666.59329.peg.344	fig|6666666.59330.peg.414	None	fig|6666666.59332.peg.1678	fig|6666666.59334.peg.134	fig|6666666.59335.peg.723	fig|6666666.59336.peg.446	fig|6666666.59333.peg.500	fig|6666666.59338.peg.1799	fig|6666666.59339.peg.6190	fig|6666666.59340.peg.174	fig|6666666.59337.peg.4239	fig|6666666.59342.peg.2911	fig|6666666.71181.peg.1843	fig|6666666.59344.peg.5479	fig|6666666.59345.peg.2680	fig|6666666.59341.peg.3431	fig|6666666.59347.peg.4460	fig|6666666.59346.peg.2599	fig|6666666.59348.peg.2079	fig|6666666.59349.peg.2178	fig|6666666.59350.peg.257	fig|6666666.59351.peg.4351	fig|6666666.59353.peg.2205	fig|6666666.59355.peg.1897	fig|6666666.59354.peg.692

10521	Oxygen-insensitive NAD P H nitroreductase EC 1.-.-.- Dihydropteridine reductase EC 1.5.1.34	fig|6666666.59329.peg.5465	fig|6666666.59330.peg.687	fig|6666666.59331.peg.6043	fig|6666666.59332.peg.3072	fig|6666666.59334.peg.5031	fig|6666666.59335.peg.3831	fig|6666666.59336.peg.4002	fig|6666666.59333.peg.4020	fig|6666666.59338.peg.2515	fig|6666666.59339.peg.1856	fig|6666666.59340.peg.2017	fig|6666666.59337.peg.4058	fig|6666666.59342.peg.169	fig|6666666.71181.peg.1345	fig|6666666.59344.peg.5832	fig|6666666.59345.peg.1866	fig|6666666.59341.peg.3972	fig|6666666.59347.peg.3407	fig|6666666.59346.peg.5142	fig|6666666.59348.peg.1660	fig|6666666.59349.peg.3717	fig|6666666.59350.peg.5705	None	fig|6666666.59353.peg.3255	fig|6666666.59355.peg.3198	fig|6666666.59354.peg.2901

9579	Outer membrane protein H precursor	fig|6666666.59329.peg.1582	fig|6666666.59330.peg.459	fig|6666666.59331.peg.4627	fig|6666666.59332.peg.5596	fig|6666666.59334.peg.1797	fig|6666666.59335.peg.2661	fig|6666666.59336.peg.2567	fig|6666666.59333.peg.4243	fig|6666666.59338.peg.2454	fig|6666666.59339.peg.5121	fig|6666666.59340.peg.1317	fig|6666666.59337.peg.1591	fig|6666666.59342.peg.277	fig|6666666.71181.peg.26	fig|6666666.59344.peg.2663	fig|6666666.59345.peg.2192	fig|6666666.59341.peg.977	fig|6666666.59347.peg.4367	fig|6666666.59346.peg.4919	fig|6666666.59348.peg.4080	fig|6666666.59349.peg.1366	None	fig|6666666.59351.peg.2124	fig|6666666.59353.peg.1994	fig|6666666.59355.peg.4099	fig|6666666.59354.peg.1342

8722	16S rRNA guanine 966 -N 2 -methyltransferase EC 2.1.1.171 ## SSU rRNA m 2 G966	fig|6666666.59329.peg.1800	fig|6666666.59330.peg.1563	None	fig|6666666.59332.peg.2703	fig|6666666.59334.peg.1288	fig|6666666.59335.peg.4273	fig|6666666.59336.peg.3160	fig|6666666.59333.peg.3181	fig|6666666.59338.peg.523	fig|6666666.59339.peg.8564	fig|6666666.59340.peg.88	fig|6666666.59337.peg.1647	fig|6666666.59342.peg.4953	fig|6666666.71181.peg.4506	fig|6666666.59344.peg.5709	fig|6666666.59345.peg.5473	fig|6666666.59341.peg.5364	fig|6666666.59347.peg.5057	fig|6666666.59346.peg.3709	fig|6666666.59348.peg.581	fig|6666666.59349.peg.4851	fig|6666666.59350.peg.31	fig|6666666.59351.peg.2369	fig|6666666.59353.peg.4932	fig|6666666.59355.peg.3361	fig|6666666.59354.peg.17

10330	5-methyltetrahydropteroyltriglutamate--homocysteine methyltransferase EC 2.1.1.14	fig|6666666.59329.peg.1986	fig|6666666.59330.peg.5020	fig|6666666.59331.peg.2274	fig|6666666.59332.peg.1611	None	fig|6666666.59335.peg.2451	fig|6666666.59336.peg.3415	fig|6666666.59333.peg.1914	fig|6666666.59338.peg.3268	fig|6666666.59339.peg.3549	fig|6666666.59340.peg.2466	fig|6666666.59337.peg.3511	fig|6666666.59342.peg.4058	fig|6666666.71181.peg.4885	fig|6666666.59344.peg.3425	fig|6666666.59345.peg.965	fig|6666666.59341.peg.3422	fig|6666666.59347.peg.3463	fig|6666666.59346.peg.4846	fig|6666666.59348.peg.2255	fig|6666666.59349.peg.4023	fig|6666666.59350.peg.1890	fig|6666666.59351.peg.1907	fig|6666666.59353.peg.3791	fig|6666666.59355.peg.3386	fig|6666666.59354.peg.1011

7763	Nucleoside diphosphate kinase EC 2.7.4.6	fig|6666666.59329.peg.5401	fig|6666666.59330.peg.1422	fig|6666666.59331.peg.2966	fig|6666666.59332.peg.1337	fig|6666666.59334.peg.3867	fig|6666666.59335.peg.2376	fig|6666666.59336.peg.677	fig|6666666.59333.peg.933	fig|6666666.59338.peg.4499	fig|6666666.59339.peg.6800	fig|6666666.59340.peg.3629	fig|6666666.59337.peg.747	fig|6666666.59342.peg.1058	fig|6666666.71181.peg.1766	fig|6666666.59344.peg.1640	fig|6666666.59345.peg.3644	fig|6666666.59341.peg.3040	fig|6666666.59347.peg.4291	fig|6666666.59346.peg.1322	None	fig|6666666.59349.peg.3078	fig|6666666.59350.peg.649	fig|6666666.59351.peg.3070	fig|6666666.59353.peg.2705	fig|6666666.59355.peg.1775	fig|6666666.59354.peg.80

6749	SSU ribosomal protein S17p S11e	fig|6666666.59329.peg.718	fig|6666666.59330.peg.559	fig|6666666.59331.peg.43	fig|6666666.59332.peg.2144	fig|6666666.59334.peg.1490	fig|6666666.59335.peg.4352	fig|6666666.59336.peg.151	fig|6666666.59333.peg.371	fig|6666666.59338.peg.1384	fig|6666666.59339.peg.8273	fig|6666666.59340.peg.61	fig|6666666.59337.peg.383	fig|6666666.59342.peg.309	fig|6666666.71181.peg.3451	fig|6666666.59344.peg.4934	fig|6666666.59345.peg.2530	fig|6666666.59341.peg.2503	fig|6666666.59347.peg.4828	fig|6666666.59346.peg.855	fig|6666666.59348.peg.1765	fig|6666666.59349.peg.1103	fig|6666666.59350.peg.7708	None	fig|6666666.59353.peg.4599	fig|6666666.59355.peg.4732	fig|6666666.59354.peg.1742

4400	Single-stranded-DNA-specific exonuclease RecJ EC 3.1.-.-	fig|6666666.59329.peg.214	fig|6666666.59330.peg.1741	fig|6666666.59331.peg.2467	fig|6666666.59332.peg.2000	fig|6666666.59334.peg.3494	fig|6666666.59335.peg.3818	fig|6666666.59336.peg.3156	fig|6666666.59333.peg.3275	fig|6666666.59338.peg.199	fig|6666666.59339.peg.7961	None	fig|6666666.59337.peg.110	fig|6666666.59342.peg.2751	fig|6666666.71181.peg.3738	fig|6666666.59344.peg.3675	fig|6666666.59345.peg.3732	fig|6666666.59341.peg.3449	fig|6666666.59347.peg.3044	fig|6666666.59346.peg.3100	fig|6666666.59348.peg.666	fig|6666666.59349.peg.4388	fig|6666666.59350.peg.4566	fig|6666666.59351.peg.4031	fig|6666666.59353.peg.3840	fig|6666666.59355.peg.1547	fig|6666666.59354.peg.3239

7155	FIG060670 Protein of unknown function DUF924	fig|6666666.59329.peg.5981	fig|6666666.59330.peg.4233	fig|6666666.59331.peg.2386	fig|6666666.59332.peg.4897	fig|6666666.59334.peg.340	fig|6666666.59335.peg.653	fig|6666666.59336.peg.1773	fig|6666666.59333.peg.2940	None	fig|6666666.59339.peg.4225	fig|6666666.59340.peg.3097	fig|6666666.59337.peg.1252	fig|6666666.59342.peg.6380	fig|6666666.71181.peg.178	fig|6666666.59344.peg.217	fig|6666666.59345.peg.4437	fig|6666666.59341.peg.4413	fig|6666666.59347.peg.3084	fig|6666666.59346.peg.819	fig|6666666.59348.peg.5363	fig|6666666.59349.peg.2740	fig|6666666.59350.peg.3661	fig|6666666.59351.peg.3664	fig|6666666.59353.peg.644	fig|6666666.59355.peg.126	fig|6666666.59354.peg.1297

8061	LSU ribosomal protein L27p	fig|6666666.59329.peg.139	fig|6666666.59330.peg.825	fig|6666666.59331.peg.1353	fig|6666666.59332.peg.3912	fig|6666666.59334.peg.4167	fig|6666666.59335.peg.4490	fig|6666666.59336.peg.4642	fig|6666666.59333.peg.551	fig|6666666.59338.peg.1824	fig|6666666.59339.peg.7880	fig|6666666.59340.peg.2134	fig|6666666.59337.peg.1376	fig|6666666.59342.peg.3944	fig|6666666.71181.peg.2793	fig|6666666.59344.peg.2117	fig|6666666.59345.peg.2467	fig|6666666.59341.peg.1994	fig|6666666.59347.peg.3096	fig|6666666.59346.peg.3982	fig|6666666.59348.peg.167	fig|6666666.59349.peg.3661	None	fig|6666666.59351.peg.302	fig|6666666.59353.peg.3149	fig|6666666.59355.peg.446	fig|6666666.59354.peg.3154

7484	Periplasmic thiol disulfide interchange protein DsbA	fig|6666666.59329.peg.4911	fig|6666666.59330.peg.630	fig|6666666.59331.peg.5892	fig|6666666.59332.peg.214	fig|6666666.59334.peg.1396	fig|6666666.59335.peg.2445	fig|6666666.59336.peg.2737	fig|6666666.59333.peg.59	fig|6666666.59338.peg.573	fig|6666666.59339.peg.8074	fig|6666666.59340.peg.3671	fig|6666666.59337.peg.3889	fig|6666666.59342.peg.3551	fig|6666666.71181.peg.2581	fig|6666666.59344.peg.890	fig|6666666.59345.peg.551	fig|6666666.59341.peg.1941	fig|6666666.59347.peg.4758	fig|6666666.59346.peg.4409	fig|6666666.59348.peg.3850	fig|6666666.59349.peg.4293	None	fig|6666666.59351.peg.4331	fig|6666666.59353.peg.2636	fig|6666666.59355.peg.2569	fig|6666666.59354.peg.3626

1106	Histidinol dehydrogenase EC 1.1.1.23	fig|6666666.59329.peg.1009	fig|6666666.59330.peg.1502	fig|6666666.59331.peg.4653	fig|6666666.59332.peg.1816	fig|6666666.59334.peg.2636	fig|6666666.59335.peg.3230	fig|6666666.59336.peg.4609	fig|6666666.59333.peg.2715	fig|6666666.59338.peg.5083	fig|6666666.59339.peg.775	None	fig|6666666.59337.peg.4868	fig|6666666.59342.peg.1532	fig|6666666.71181.peg.5373	fig|6666666.59344.peg.312	fig|6666666.59345.peg.1307	fig|6666666.59341.peg.497	fig|6666666.59347.peg.3423	fig|6666666.59346.peg.2682	fig|6666666.59348.peg.4400	fig|6666666.59349.peg.459	fig|6666666.59350.peg.3402	fig|6666666.59351.peg.1625	fig|6666666.59353.peg.569	fig|6666666.59355.peg.53	fig|6666666.59354.peg.890

4791	Di- tripeptide transporter	fig|6666666.59329.peg.1943	fig|6666666.59330.peg.5053	fig|6666666.59331.peg.1526	fig|6666666.59332.peg.3858	fig|6666666.59334.peg.4332	fig|6666666.59335.peg.3913	fig|6666666.59336.peg.3084	fig|6666666.59333.peg.873	fig|6666666.59338.peg.2346	fig|6666666.59339.peg.4504	fig|6666666.59340.peg.1382	fig|6666666.59337.peg.3922	fig|6666666.59342.peg.3017	fig|6666666.71181.peg.2906	fig|6666666.59344.peg.2401	fig|6666666.59345.peg.4346	fig|6666666.59341.peg.3924	fig|6666666.59347.peg.2830	fig|6666666.59346.peg.2822	fig|6666666.59348.peg.3019	fig|6666666.59349.peg.4008	None	fig|6666666.59351.peg.1417	fig|6666666.59353.peg.1414	fig|6666666.59355.peg.2460	fig|6666666.59354.peg.3681

1593	Flagellar biosynthesis protein FliP	fig|6666666.59329.peg.1320	fig|6666666.59330.peg.2229	fig|6666666.59331.peg.5221	fig|6666666.59332.peg.4087	None	fig|6666666.59335.peg.5319	fig|6666666.59336.peg.1275	fig|6666666.59333.peg.3371	fig|6666666.59338.peg.3097	fig|6666666.59339.peg.1332	fig|6666666.59340.peg.1581	fig|6666666.59337.peg.5234	fig|6666666.59342.peg.2648	fig|6666666.71181.peg.5562	fig|6666666.59344.peg.3051	fig|6666666.59345.peg.1468	fig|6666666.59341.peg.136	fig|6666666.59347.peg.4622	fig|6666666.59346.peg.2890	fig|6666666.59348.peg.3694	fig|6666666.59349.peg.201	fig|6666666.59350.peg.6525	fig|6666666.59351.peg.2056	fig|6666666.59353.peg.3513	fig|6666666.59355.peg.3663	fig|6666666.59354.peg.675

1599	Preprotein translocase secY subunit TC 3.A.5.1.1	fig|6666666.59329.peg.729	fig|6666666.59330.peg.571	fig|6666666.59331.peg.32	None	fig|6666666.59334.peg.3045	fig|6666666.59335.peg.4363	fig|6666666.59336.peg.162	fig|6666666.59333.peg.383	fig|6666666.59338.peg.5848	fig|6666666.59339.peg.8285	fig|6666666.59340.peg.2219	fig|6666666.59337.peg.372	fig|6666666.59342.peg.320	fig|6666666.71181.peg.3443	fig|6666666.59344.peg.4926	fig|6666666.59345.peg.2519	fig|6666666.59341.peg.2514	fig|6666666.59347.peg.4840	fig|6666666.59346.peg.866	fig|6666666.59348.peg.438	fig|6666666.59349.peg.3286	fig|6666666.59350.peg.1548	fig|6666666.59351.peg.4221	fig|6666666.59353.peg.4607	fig|6666666.59355.peg.4724	fig|6666666.59354.peg.3251

8065	Low-specificity L-threonine aldolase EC 4.1.2.5	fig|6666666.59329.peg.4586	fig|6666666.59330.peg.3154	fig|6666666.59331.peg.5609	fig|6666666.59332.peg.450	fig|6666666.59334.peg.683	fig|6666666.59335.peg.1056	fig|6666666.59336.peg.3797	fig|6666666.59333.peg.5337	fig|6666666.59338.peg.5508	fig|6666666.59339.peg.2080	None	fig|6666666.59337.peg.1166	fig|6666666.59342.peg.501	fig|6666666.71181.peg.3918	fig|6666666.59344.peg.386	fig|6666666.59345.peg.184	fig|6666666.59341.peg.2787	fig|6666666.59347.peg.4318	fig|6666666.59346.peg.2486	fig|6666666.59348.peg.921	fig|6666666.59349.peg.2057	fig|6666666.59350.peg.6315	fig|6666666.59351.peg.1827	fig|6666666.59353.peg.1606	fig|6666666.59355.peg.1115	fig|6666666.59354.peg.735

486	Cell division inhibitor	fig|6666666.59329.peg.4978	fig|6666666.59330.peg.2195	fig|6666666.59331.peg.6209	fig|6666666.59332.peg.623	fig|6666666.59334.peg.3366	fig|6666666.59335.peg.2430	fig|6666666.59336.peg.1509	fig|6666666.59333.peg.2053	fig|6666666.59338.peg.2795	fig|6666666.59339.peg.5248	fig|6666666.59340.peg.990	fig|6666666.59337.peg.5067	fig|6666666.59342.peg.1955	fig|6666666.71181.peg.567	fig|6666666.59344.peg.1896	fig|6666666.59345.peg.4363	fig|6666666.59341.peg.4983	fig|6666666.59347.peg.4436	fig|6666666.59346.peg.2367	fig|6666666.59348.peg.3556	fig|6666666.59349.peg.2692	None	fig|6666666.59351.peg.772	fig|6666666.59353.peg.1198	fig|6666666.59355.peg.278	fig|6666666.59354.peg.6443

484	Serine transporter	fig|6666666.59329.peg.2594	fig|6666666.59330.peg.2076	fig|6666666.59331.peg.2732	fig|6666666.59332.peg.4605	fig|6666666.59334.peg.2773	fig|6666666.59335.peg.6084	fig|6666666.59336.peg.6262	fig|6666666.59333.peg.4481	fig|6666666.59338.peg.5468	fig|6666666.59339.peg.3705	fig|6666666.59340.peg.1589	fig|6666666.59337.peg.3741	fig|6666666.59342.peg.734	fig|6666666.71181.peg.3851	fig|6666666.59344.peg.336	fig|6666666.59345.peg.296	fig|6666666.59341.peg.3522	fig|6666666.59347.peg.2156	fig|6666666.59346.peg.4501	fig|6666666.59348.peg.1442	fig|6666666.59349.peg.3976	None	fig|6666666.59351.peg.4005	fig|6666666.59353.peg.552	fig|6666666.59355.peg.37	fig|6666666.59354.peg.2552

9934	Chemotaxis protein CheV EC 2.7.3.-	fig|6666666.59329.peg.6042	fig|6666666.59330.peg.2723	fig|6666666.59331.peg.5078	fig|6666666.59332.peg.2653	None	fig|6666666.59335.peg.2078	fig|6666666.59336.peg.6020	fig|6666666.59333.peg.2833	fig|6666666.59338.peg.5309	fig|6666666.59339.peg.7030	fig|6666666.59340.peg.2473	fig|6666666.59337.peg.4057	fig|6666666.59342.peg.5507	fig|6666666.71181.peg.2248	fig|6666666.59344.peg.3012	fig|6666666.59345.peg.5319	fig|6666666.59341.peg.33	fig|6666666.59347.peg.5087	fig|6666666.59346.peg.1760	fig|6666666.59348.peg.2188	fig|6666666.59349.peg.2271	fig|6666666.59350.peg.8335	fig|6666666.59351.peg.2561	fig|6666666.59353.peg.3545	fig|6666666.59355.peg.3630	fig|6666666.59354.peg.1844

10350	Replicative DNA helicase EC 3.6.1.-	fig|6666666.59329.peg.324	fig|6666666.59330.peg.2644	fig|6666666.59331.peg.4184	fig|6666666.59332.peg.898	fig|6666666.59334.peg.166	fig|6666666.59335.peg.1777	fig|6666666.59336.peg.601	fig|6666666.59333.peg.886	fig|6666666.59338.peg.4457	fig|6666666.59339.peg.7701	fig|6666666.59340.peg.577	fig|6666666.59337.peg.1710	None	fig|6666666.71181.peg.1993	fig|6666666.59344.peg.711	fig|6666666.59345.peg.5371	fig|6666666.59341.peg.2415	fig|6666666.59347.peg.5374	fig|6666666.59346.peg.2390	fig|6666666.59348.peg.4481	fig|6666666.59349.peg.3240	fig|6666666.59350.peg.3893	fig|6666666.59351.peg.5435	fig|6666666.59353.peg.966	fig|6666666.59355.peg.2896	fig|6666666.59354.peg.1997

4427	tRNA pseudouridine synthase A EC 4.2.1.70	fig|6666666.59329.peg.5173	fig|6666666.59330.peg.1599	fig|6666666.59331.peg.4693	fig|6666666.59332.peg.2754	fig|6666666.59334.peg.1828	fig|6666666.59335.peg.3587	fig|6666666.59336.peg.6250	fig|6666666.59333.peg.4801	fig|6666666.59338.peg.4114	fig|6666666.59339.peg.2967	None	fig|6666666.59337.peg.5672	fig|6666666.59342.peg.3884	fig|6666666.71181.peg.1480	fig|6666666.59344.peg.1881	fig|6666666.59345.peg.1205	fig|6666666.59341.peg.2782	fig|6666666.59347.peg.2457	fig|6666666.59346.peg.51	fig|6666666.59348.peg.5220	fig|6666666.59349.peg.3458	fig|6666666.59350.peg.6398	fig|6666666.59351.peg.3570	fig|6666666.59353.peg.1211	fig|6666666.59355.peg.265	fig|6666666.59354.peg.2965

10152	GTP pyrophosphokinase EC 2.7.6.5 , p ppGpp synthetase II Guanosine-3',5'-bis diphosphate 3'-pyrophosphohydrolase EC 3.1.7.2	fig|6666666.59329.peg.1266	fig|6666666.59330.peg.2469	None	fig|6666666.59332.peg.194	fig|6666666.59334.peg.238	fig|6666666.59335.peg.6055	fig|6666666.59336.peg.2781	fig|6666666.59333.peg.4309	fig|6666666.59338.peg.4190	fig|6666666.59339.peg.7384	fig|6666666.59340.peg.2448	fig|6666666.59337.peg.504	fig|6666666.59342.peg.585	fig|6666666.71181.peg.3768	fig|6666666.59344.peg.922	fig|6666666.59345.peg.2650	fig|6666666.59341.peg.5726	fig|6666666.59347.peg.5200	fig|6666666.59346.peg.3155	fig|6666666.59348.peg.1931	fig|6666666.59349.peg.3644	fig|6666666.59350.peg.1360	fig|6666666.59351.peg.2590	fig|6666666.59353.peg.178	fig|6666666.59355.peg.2594	fig|6666666.59354.peg.914

5216	LSU ribosomal protein L21p	fig|6666666.59329.peg.140	fig|6666666.59330.peg.826	fig|6666666.59331.peg.1352	None	fig|6666666.59334.peg.4166	fig|6666666.59335.peg.4491	fig|6666666.59336.peg.4641	fig|6666666.59333.peg.552	fig|6666666.59338.peg.1823	fig|6666666.59339.peg.7879	fig|6666666.59340.peg.2135	fig|6666666.59337.peg.1375	fig|6666666.59342.peg.3943	fig|6666666.71181.peg.2794	fig|6666666.59344.peg.2118	fig|6666666.59345.peg.2468	fig|6666666.59341.peg.1993	fig|6666666.59347.peg.3097	fig|6666666.59346.peg.3983	fig|6666666.59348.peg.166	fig|6666666.59349.peg.3660	fig|6666666.59350.peg.5470	fig|6666666.59351.peg.301	fig|6666666.59353.peg.3150	fig|6666666.59355.peg.445	fig|6666666.59354.peg.3155

4596	Protein acetyltransferase	fig|6666666.59329.peg.5233	fig|6666666.59330.peg.4345	fig|6666666.59331.peg.1888	fig|6666666.59332.peg.4054	fig|6666666.59334.peg.2263	fig|6666666.59335.peg.574	fig|6666666.59336.peg.2251	fig|6666666.59333.peg.4981	fig|6666666.59338.peg.3814	fig|6666666.59339.peg.2920	None	fig|6666666.59337.peg.3029	fig|6666666.59342.peg.2283	fig|6666666.71181.peg.2648	fig|6666666.59344.peg.3922	fig|6666666.59345.peg.85	fig|6666666.59341.peg.3355	fig|6666666.59347.peg.966	fig|6666666.59346.peg.1886	fig|6666666.59348.peg.5482	fig|6666666.59349.peg.4156	fig|6666666.59350.peg.863	fig|6666666.59351.peg.4280	fig|6666666.59353.peg.4760	fig|6666666.59355.peg.4044	fig|6666666.59354.peg.5191

9624	LSU ribosomal protein L32p	fig|6666666.59329.peg.5962	fig|6666666.59330.peg.3618	fig|6666666.59331.peg.831	fig|6666666.59332.peg.3983	fig|6666666.59334.peg.3323	fig|6666666.59335.peg.2045	fig|6666666.59336.peg.6099	fig|6666666.59333.peg.1596	fig|6666666.59338.peg.4648	fig|6666666.59339.peg.3173	fig|6666666.59340.peg.2712	fig|6666666.59337.peg.2692	fig|6666666.59342.peg.1388	fig|6666666.71181.peg.1619	fig|6666666.59344.peg.2429	fig|6666666.59345.peg.1067	fig|6666666.59341.peg.12	fig|6666666.59347.peg.4081	fig|6666666.59346.peg.103	fig|6666666.59348.peg.4431	fig|6666666.59349.peg.3906	None	fig|6666666.59351.peg.669	fig|6666666.59353.peg.310	fig|6666666.59355.peg.1350	fig|6666666.59354.peg.2154

1312	Succinyl-CoA ligase [ADP-forming] alpha chain EC 6.2.1.5	fig|6666666.59329.peg.154	fig|6666666.59330.peg.2776	fig|6666666.59331.peg.1417	fig|6666666.59332.peg.661	fig|6666666.59334.peg.2835	None	fig|6666666.59336.peg.2114	fig|6666666.59333.peg.905	fig|6666666.59338.peg.1965	fig|6666666.59339.peg.7184	fig|6666666.59340.peg.1023	fig|6666666.59337.peg.4114	fig|6666666.59342.peg.482	fig|6666666.71181.peg.3239	fig|6666666.59344.peg.4588	fig|6666666.59345.peg.4318	fig|6666666.59341.peg.1575	fig|6666666.59347.peg.4389	fig|6666666.59346.peg.2436	fig|6666666.59348.peg.4474	fig|6666666.59349.peg.2525	fig|6666666.59350.peg.3906	fig|6666666.59351.peg.3732	fig|6666666.59353.peg.2090	fig|6666666.59355.peg.2734	fig|6666666.59354.peg.1316

7722	Methionine aminopeptidase EC 3.4.11.18	fig|6666666.59329.peg.5024	fig|6666666.59330.peg.470	fig|6666666.59331.peg.6271	fig|6666666.59332.peg.987	fig|6666666.59334.peg.2976	fig|6666666.59335.peg.160	fig|6666666.59336.peg.4216	fig|6666666.59333.peg.3062	fig|6666666.59338.peg.2557	fig|6666666.59339.peg.5106	None	fig|6666666.59337.peg.1580	fig|6666666.59342.peg.264	fig|6666666.71181.peg.36	fig|6666666.59344.peg.2651	fig|6666666.59345.peg.2202	fig|6666666.59341.peg.966	fig|6666666.59347.peg.4357	fig|6666666.59346.peg.2258	fig|6666666.59348.peg.871	fig|6666666.59349.peg.1378	fig|6666666.59350.peg.8110	fig|6666666.59351.peg.818	fig|6666666.59353.peg.1982	fig|6666666.59355.peg.4089	fig|6666666.59354.peg.4016

7727	SSU ribosomal protein S6p	fig|6666666.59329.peg.320	fig|6666666.59330.peg.2640	fig|6666666.59331.peg.5465	fig|6666666.59332.peg.902	fig|6666666.59334.peg.171	fig|6666666.59335.peg.3493	fig|6666666.59336.peg.595	fig|6666666.59333.peg.891	fig|6666666.59338.peg.6124	fig|6666666.59339.peg.7695	None	fig|6666666.59337.peg.1706	fig|6666666.59342.peg.1891	fig|6666666.71181.peg.2820	fig|6666666.59344.peg.2149	fig|6666666.59345.peg.5376	fig|6666666.59341.peg.2420	fig|6666666.59347.peg.5440	fig|6666666.59346.peg.2386	fig|6666666.59348.peg.4968	fig|6666666.59349.peg.3228	fig|6666666.59350.peg.3461	fig|6666666.59351.peg.5422	fig|6666666.59353.peg.3178	fig|6666666.59355.peg.419	fig|6666666.59354.peg.2014

3610	Glycine cleavage system H protein	fig|6666666.59329.peg.3665	fig|6666666.59330.peg.3582	fig|6666666.59331.peg.2406	fig|6666666.59332.peg.3782	fig|6666666.59334.peg.2340	fig|6666666.59335.peg.6082	fig|6666666.59336.peg.5091	fig|6666666.59333.peg.4945	fig|6666666.59338.peg.1255	fig|6666666.59339.peg.2095	None	fig|6666666.59337.peg.4824	fig|6666666.59342.peg.2900	fig|6666666.71181.peg.3125	fig|6666666.59344.peg.4746	fig|6666666.59345.peg.174	fig|6666666.59341.peg.435	fig|6666666.59347.peg.1404	fig|6666666.59346.peg.5245	fig|6666666.59348.peg.4759	fig|6666666.59349.peg.4507	fig|6666666.59350.peg.7927	fig|6666666.59351.peg.2187	fig|6666666.59353.peg.4443	fig|6666666.59355.peg.4522	fig|6666666.59354.peg.3302

11428	Nucleoside permease NupC	fig|6666666.59329.peg.4204	fig|6666666.59330.peg.4578	fig|6666666.59331.peg.4580	fig|6666666.59332.peg.1209	fig|6666666.59334.peg.1440	fig|6666666.59335.peg.1475	fig|6666666.59336.peg.1014	fig|6666666.59333.peg.5176	fig|6666666.59338.peg.4341	fig|6666666.59339.peg.4420	fig|6666666.59340.peg.2002	fig|6666666.59337.peg.1302	fig|6666666.59342.peg.5879	fig|6666666.71181.peg.2609	fig|6666666.59344.peg.930	fig|6666666.59345.peg.4290	fig|6666666.59341.peg.5002	fig|6666666.59347.peg.1231	fig|6666666.59346.peg.899	fig|6666666.59348.peg.2224	fig|6666666.59349.peg.4718	None	fig|6666666.59351.peg.1196	fig|6666666.59353.peg.36	fig|6666666.59355.peg.643	fig|6666666.59354.peg.6032

11426	Adenylylsulfate kinase EC 2.7.1.25	fig|6666666.59329.peg.282	fig|6666666.59330.peg.592	fig|6666666.59331.peg.4381	fig|6666666.59332.peg.1192	fig|6666666.59334.peg.2480	fig|6666666.59335.peg.4596	fig|6666666.59336.peg.2820	fig|6666666.59333.peg.404	fig|6666666.59338.peg.343	fig|6666666.59339.peg.8313	fig|6666666.59340.peg.1530	fig|6666666.59337.peg.351	None	fig|6666666.71181.peg.3409	fig|6666666.59344.peg.2206	fig|6666666.59345.peg.2498	fig|6666666.59341.peg.788	fig|6666666.59347.peg.4857	fig|6666666.59346.peg.1965	fig|6666666.59348.peg.534	fig|6666666.59349.peg.1155	fig|6666666.59350.peg.3322	fig|6666666.59351.peg.5230	fig|6666666.59353.peg.202	fig|6666666.59355.peg.2971	fig|6666666.59354.peg.2136

5020	Aminobenzoyl-glutamate transport protein	fig|6666666.59329.peg.1671	fig|6666666.59330.peg.5155	fig|6666666.59331.peg.2593	fig|6666666.59332.peg.977	fig|6666666.59334.peg.4195	fig|6666666.59335.peg.1383	fig|6666666.59336.peg.5786	fig|6666666.59333.peg.2255	fig|6666666.59338.peg.5733	fig|6666666.59339.peg.2779	fig|6666666.59340.peg.2888	fig|6666666.59337.peg.1569	fig|6666666.59342.peg.2185	fig|6666666.71181.peg.4801	fig|6666666.59344.peg.5943	fig|6666666.59345.peg.2214	fig|6666666.59341.peg.4065	fig|6666666.59347.peg.4338	fig|6666666.59346.peg.3609	fig|6666666.59348.peg.1472	fig|6666666.59349.peg.981	None	fig|6666666.59351.peg.714	fig|6666666.59353.peg.3904	fig|6666666.59355.peg.3784	fig|6666666.59354.peg.2715

1960	DNA mismatch repair endonuclease MutH	fig|6666666.59329.peg.6164	fig|6666666.59330.peg.1751	fig|6666666.59331.peg.5620	fig|6666666.59332.peg.1355	fig|6666666.59334.peg.4545	fig|6666666.59335.peg.1023	fig|6666666.59336.peg.2368	fig|6666666.59333.peg.4651	fig|6666666.59338.peg.191	fig|6666666.59339.peg.7442	fig|6666666.59340.peg.190	fig|6666666.59337.peg.119	fig|6666666.59342.peg.3796	fig|6666666.71181.peg.74	fig|6666666.59344.peg.3192	fig|6666666.59345.peg.3723	fig|6666666.59341.peg.3954	fig|6666666.59347.peg.3035	fig|6666666.59346.peg.3094	fig|6666666.59348.peg.1897	fig|6666666.59349.peg.3873	None	fig|6666666.59351.peg.2586	fig|6666666.59353.peg.1941	fig|6666666.59355.peg.1466	fig|6666666.59354.peg.919

1674	ATPase YjeE, predicted to have essential role in cell wall biosynthesis	fig|6666666.59329.peg.700	fig|6666666.59330.peg.3555	None	fig|6666666.59332.peg.1975	fig|6666666.59334.peg.1637	fig|6666666.59335.peg.4127	fig|6666666.59336.peg.3756	fig|6666666.59333.peg.280	fig|6666666.59338.peg.3084	fig|6666666.59339.peg.8853	fig|6666666.59340.peg.103	fig|6666666.59337.peg.1777	fig|6666666.59342.peg.5105	fig|6666666.71181.peg.1697	fig|6666666.59344.peg.6255	fig|6666666.59345.peg.5668	fig|6666666.59341.peg.3766	fig|6666666.59347.peg.5351	fig|6666666.59346.peg.3045	fig|6666666.59348.peg.1941	fig|6666666.59349.peg.3166	fig|6666666.59350.peg.4525	fig|6666666.59351.peg.3090	fig|6666666.59353.peg.1067	fig|6666666.59355.peg.3550	fig|6666666.59354.peg.3722

1677	Flagellar basal-body rod protein FlgG	fig|6666666.59329.peg.2330	fig|6666666.59330.peg.4413	fig|6666666.59331.peg.3563	fig|6666666.59332.peg.1790	None	fig|6666666.59335.peg.902	fig|6666666.59336.peg.5044	fig|6666666.59333.peg.2841	fig|6666666.59338.peg.638	fig|6666666.59339.peg.74	fig|6666666.59340.peg.1681	fig|6666666.59337.peg.3354	fig|6666666.59342.peg.1496	fig|6666666.71181.peg.2227	fig|6666666.59344.peg.3037	fig|6666666.59345.peg.5455	fig|6666666.59341.peg.6087	fig|6666666.59347.peg.2708	fig|6666666.59346.peg.1766	fig|6666666.59348.peg.951	fig|6666666.59349.peg.728	fig|6666666.59350.peg.7003	fig|6666666.59351.peg.1986	fig|6666666.59353.peg.3523	fig|6666666.59355.peg.3653	fig|6666666.59354.peg.1528

2773	FKBP-type peptidyl-prolyl cis-trans isomerase FkpA precursor EC 5.2.1.8	fig|6666666.59329.peg.1569	fig|6666666.59330.peg.4076	fig|6666666.59331.peg.5970	fig|6666666.59332.peg.6091	fig|6666666.59334.peg.5557	fig|6666666.59335.peg.6139	fig|6666666.59336.peg.2184	fig|6666666.59333.peg.5172	fig|6666666.59338.peg.5734	fig|6666666.59339.peg.8294	fig|6666666.59340.peg.586	fig|6666666.59337.peg.3551	fig|6666666.59342.peg.5615	fig|6666666.71181.peg.2815	fig|6666666.59344.peg.3127	fig|6666666.59345.peg.5556	fig|6666666.59341.peg.5932	fig|6666666.59347.peg.5336	fig|6666666.59346.peg.40	fig|6666666.59348.peg.429	fig|6666666.59349.peg.4734	None	fig|6666666.59351.peg.3633	fig|6666666.59353.peg.3173	fig|6666666.59355.peg.424	fig|6666666.59354.peg.4550

10928	LSU ribosomal protein L22p L17e	fig|6666666.59329.peg.6289	fig|6666666.59330.peg.555	fig|6666666.59331.peg.47	fig|6666666.59332.peg.2140	fig|6666666.59334.peg.1486	None	fig|6666666.59336.peg.147	fig|6666666.59333.peg.367	fig|6666666.59338.peg.1388	fig|6666666.59339.peg.8269	fig|6666666.59340.peg.56	fig|6666666.59337.peg.387	fig|6666666.59342.peg.305	fig|6666666.71181.peg.3455	fig|6666666.59344.peg.4938	fig|6666666.59345.peg.2534	fig|6666666.59341.peg.2499	fig|6666666.59347.peg.4824	fig|6666666.59346.peg.851	fig|6666666.59348.peg.416	fig|6666666.59349.peg.1099	fig|6666666.59350.peg.6234	fig|6666666.59351.peg.4813	fig|6666666.59353.peg.4595	fig|6666666.59355.peg.5917	fig|6666666.59354.peg.1746

2000	DNA mismatch repair protein MutS	fig|6666666.59329.peg.4290	fig|6666666.59330.peg.2454	fig|6666666.59331.peg.6339	fig|6666666.59332.peg.206	fig|6666666.59334.peg.277	fig|6666666.59335.peg.259	fig|6666666.59336.peg.3447	fig|6666666.59333.peg.1717	fig|6666666.59338.peg.130	fig|6666666.59339.peg.8132	None	fig|6666666.59337.peg.586	fig|6666666.59342.peg.1267	fig|6666666.71181.peg.3423	fig|6666666.59344.peg.2190	fig|6666666.59345.peg.5076	fig|6666666.59341.peg.2703	fig|6666666.59347.peg.3230	fig|6666666.59346.peg.3268	fig|6666666.59348.peg.1098	fig|6666666.59349.peg.3778	fig|6666666.59350.peg.1496	fig|6666666.59351.peg.3385	fig|6666666.59353.peg.216	fig|6666666.59355.peg.2985	fig|6666666.59354.peg.29

3962	Tol biopolymer transport system, TolR protein	fig|6666666.59329.peg.4962	fig|6666666.59330.peg.3438	fig|6666666.59331.peg.1725	fig|6666666.59332.peg.5750	fig|6666666.59334.peg.4670	fig|6666666.59335.peg.2507	fig|6666666.59336.peg.2585	fig|6666666.59333.peg.1988	fig|6666666.59338.peg.4119	fig|6666666.59339.peg.6264	None	fig|6666666.59337.peg.4382	fig|6666666.59342.peg.4593	fig|6666666.71181.peg.5005	fig|6666666.59344.peg.4575	fig|6666666.59345.peg.1508	fig|6666666.59341.peg.3349	fig|6666666.59347.peg.4207	fig|6666666.59346.peg.1443	fig|6666666.59348.peg.121	fig|6666666.59349.peg.3863	fig|6666666.59350.peg.2395	fig|6666666.59351.peg.657	fig|6666666.59353.peg.4701	fig|6666666.59355.peg.2723	fig|6666666.59354.peg.1595

4467	Inner membrane protein YrbG, predicted calcium sodium proton antiporter	fig|6666666.59329.peg.822	fig|6666666.59330.peg.50	fig|6666666.59331.peg.3258	fig|6666666.59332.peg.5661	fig|6666666.59334.peg.94	fig|6666666.59335.peg.3044	fig|6666666.59336.peg.354	fig|6666666.59333.peg.268	fig|6666666.59338.peg.2211	fig|6666666.59339.peg.8195	fig|6666666.59340.peg.263	fig|6666666.59337.peg.835	None	fig|6666666.71181.peg.1022	fig|6666666.59344.peg.1802	fig|6666666.59345.peg.4856	fig|6666666.59341.peg.1250	fig|6666666.59347.peg.3247	fig|6666666.59346.peg.634	fig|6666666.59348.peg.4000	fig|6666666.59349.peg.3537	fig|6666666.59350.peg.3914	fig|6666666.59351.peg.1137	fig|6666666.59353.peg.2341	fig|6666666.59355.peg.1614	fig|6666666.59354.peg.5893

10116	Type IV fimbrial assembly protein PilC	fig|6666666.59329.peg.3430	fig|6666666.59330.peg.15	fig|6666666.59331.peg.996	fig|6666666.59332.peg.2042	fig|6666666.59334.peg.126	fig|6666666.59335.peg.207	fig|6666666.59336.peg.1181	fig|6666666.59333.peg.507	fig|6666666.59338.peg.2614	fig|6666666.59339.peg.7945	fig|6666666.59340.peg.645	fig|6666666.59337.peg.2051	fig|6666666.59342.peg.3663	fig|6666666.71181.peg.1965	fig|6666666.59344.peg.1460	fig|6666666.59345.peg.2675	fig|6666666.59341.peg.1350	fig|6666666.59347.peg.4465	fig|6666666.59346.peg.577	fig|6666666.59348.peg.3909	fig|6666666.59349.peg.2173	None	fig|6666666.59351.peg.4345	fig|6666666.59353.peg.154	fig|6666666.59355.peg.764	fig|6666666.59354.peg.6134

8975	Anthranilate phosphoribosyltransferase EC 2.4.2.18	fig|6666666.59329.peg.400	fig|6666666.59330.peg.3335	fig|6666666.59331.peg.4609	fig|6666666.59332.peg.5515	fig|6666666.59334.peg.3050	fig|6666666.59335.peg.3721	fig|6666666.59336.peg.884	fig|6666666.59333.peg.1761	fig|6666666.59338.peg.3803	fig|6666666.59339.peg.3637	None	fig|6666666.59337.peg.3498	fig|6666666.59342.peg.761	fig|6666666.71181.peg.4145	fig|6666666.59344.peg.459	fig|6666666.59345.peg.917	fig|6666666.59341.peg.1021	fig|6666666.59347.peg.3555	fig|6666666.59346.peg.1603	fig|6666666.59348.peg.3576	fig|6666666.59349.peg.4273	fig|6666666.59350.peg.2776	fig|6666666.59351.peg.786	fig|6666666.59353.peg.1780	fig|6666666.59355.peg.1235	fig|6666666.59354.peg.5219

2830	N-acetyl-gamma-glutamyl-phosphate reductase EC 1.2.1.38	fig|6666666.59329.peg.982	fig|6666666.59330.peg.1681	None	fig|6666666.59332.peg.5907	fig|6666666.59334.peg.1802	fig|6666666.59335.peg.4743	fig|6666666.59336.peg.1600	fig|6666666.59333.peg.1846	fig|6666666.59338.peg.4221	fig|6666666.59339.peg.8742	fig|6666666.59340.peg.1451	fig|6666666.59337.peg.1684	fig|6666666.59342.peg.3125	fig|6666666.71181.peg.4432	fig|6666666.59344.peg.1013	fig|6666666.59345.peg.5397	fig|6666666.59341.peg.951	fig|6666666.59347.peg.4929	fig|6666666.59346.peg.997	fig|6666666.59348.peg.146	fig|6666666.59349.peg.1751	fig|6666666.59350.peg.7054	fig|6666666.59351.peg.2282	fig|6666666.59353.peg.2528	fig|6666666.59355.peg.4885	fig|6666666.59354.peg.481

10948	Putative membrane protein	fig|6666666.59329.peg.327	fig|6666666.59330.peg.2834	fig|6666666.59331.peg.64	fig|6666666.59332.peg.1568	fig|6666666.59334.peg.253	fig|6666666.59335.peg.3986	fig|6666666.59336.peg.1356	fig|6666666.59333.peg.3984	fig|6666666.59338.peg.442	fig|6666666.59339.peg.8918	fig|6666666.59340.peg.1761	fig|6666666.59337.peg.2031	None	fig|6666666.71181.peg.3466	fig|6666666.59344.peg.2162	fig|6666666.59345.peg.2442	fig|6666666.59341.peg.2564	fig|6666666.59347.peg.3219	fig|6666666.59346.peg.558	fig|6666666.59348.peg.1988	fig|6666666.59349.peg.4415	fig|6666666.59350.peg.6866	fig|6666666.59351.peg.4731	fig|6666666.59353.peg.235	fig|6666666.59355.peg.3006	fig|6666666.59354.peg.2032

2248	16 kDa heat shock protein B	fig|6666666.59329.peg.6136	fig|6666666.59330.peg.1097	fig|6666666.59331.peg.2605	fig|6666666.59332.peg.81	fig|6666666.59334.peg.142	fig|6666666.59335.peg.3928	fig|6666666.59336.peg.5	fig|6666666.59333.peg.3480	fig|6666666.59338.peg.4581	fig|6666666.59339.peg.8043	None	fig|6666666.59337.peg.869	fig|6666666.59342.peg.5926	fig|6666666.71181.peg.639	fig|6666666.59344.peg.4196	fig|6666666.59345.peg.4983	fig|6666666.59341.peg.5852	fig|6666666.59347.peg.4802	fig|6666666.59346.peg.1199	fig|6666666.59348.peg.1366	fig|6666666.59349.peg.3132	fig|6666666.59350.peg.2420	fig|6666666.59351.peg.2993	fig|6666666.59353.peg.3041	fig|6666666.59355.peg.2307	fig|6666666.59354.peg.3842

8786	Protein-L-isoaspartate O-methyltransferase EC 2.1.1.77	fig|6666666.59329.peg.4463	fig|6666666.59330.peg.2458	fig|6666666.59331.peg.5500	fig|6666666.59332.peg.203	fig|6666666.59334.peg.272	fig|6666666.59335.peg.1660	fig|6666666.59336.peg.4464	fig|6666666.59333.peg.1713	fig|6666666.59338.peg.218	fig|6666666.59339.peg.7921	None	fig|6666666.59337.peg.583	fig|6666666.59342.peg.1270	fig|6666666.71181.peg.3420	fig|6666666.59344.peg.2193	fig|6666666.59345.peg.5073	fig|6666666.59341.peg.1566	fig|6666666.59347.peg.3953	fig|6666666.59346.peg.3144	fig|6666666.59348.peg.3635	fig|6666666.59349.peg.4385	fig|6666666.59350.peg.7736	fig|6666666.59351.peg.5473	fig|6666666.59353.peg.213	fig|6666666.59355.peg.2982	fig|6666666.59354.peg.3146

7090	2-oxoglutarate dehydrogenase E1 component EC 1.2.4.2	fig|6666666.59329.peg.159	fig|6666666.59330.peg.2773	fig|6666666.59331.peg.75	fig|6666666.59332.peg.657	None	fig|6666666.59335.peg.2745	fig|6666666.59336.peg.2110	fig|6666666.59333.peg.901	fig|6666666.59338.peg.3103	fig|6666666.59339.peg.7189	fig|6666666.59340.peg.1726	fig|6666666.59337.peg.4110	fig|6666666.59342.peg.477	fig|6666666.71181.peg.3236	fig|6666666.59344.peg.4594	fig|6666666.59345.peg.4321	fig|6666666.59341.peg.1579	fig|6666666.59347.peg.4386	fig|6666666.59346.peg.2440	fig|6666666.59348.peg.4483	fig|6666666.59349.peg.4996	fig|6666666.59350.peg.3904	fig|6666666.59351.peg.3727	fig|6666666.59353.peg.2087	fig|6666666.59355.peg.2738	fig|6666666.59354.peg.1310

5793	DNA repair protein RadC	fig|6666666.59329.peg.3225	fig|6666666.59330.peg.1169	None	fig|6666666.59332.peg.3879	fig|6666666.59334.peg.242	fig|6666666.59335.peg.5847	fig|6666666.59336.peg.51	fig|6666666.59333.peg.657	fig|6666666.59338.peg.1114	fig|6666666.59339.peg.7304	fig|6666666.59340.peg.10	fig|6666666.59337.peg.474	fig|6666666.59342.peg.2092	fig|6666666.71181.peg.3654	fig|6666666.59344.peg.941	fig|6666666.59345.peg.2621	fig|6666666.59341.peg.208	fig|6666666.59347.peg.4519	fig|6666666.59346.peg.1715	fig|6666666.59348.peg.726	fig|6666666.59349.peg.110	fig|6666666.59350.peg.214	fig|6666666.59351.peg.167	fig|6666666.59353.peg.2593	fig|6666666.59355.peg.2609	fig|6666666.59354.peg.3708

7879	Cell division protein FtsL	fig|6666666.59329.peg.2029	fig|6666666.59330.peg.1269	fig|6666666.59331.peg.2001	fig|6666666.59332.peg.5059	fig|6666666.59334.peg.5106	fig|6666666.59335.peg.843	fig|6666666.59336.peg.709	fig|6666666.59333.peg.3485	fig|6666666.59338.peg.535	fig|6666666.59339.peg.4959	fig|6666666.59340.peg.620	fig|6666666.59337.peg.41	fig|6666666.59342.peg.3873	fig|6666666.71181.peg.1987	fig|6666666.59344.peg.1489	fig|6666666.59345.peg.3801	fig|6666666.59341.peg.1357	fig|6666666.59347.peg.3993	fig|6666666.59346.peg.1230	fig|6666666.59348.peg.2585	fig|6666666.59349.peg.2977	None	fig|6666666.59351.peg.201	fig|6666666.59353.peg.130	fig|6666666.59355.peg.741	fig|6666666.59354.peg.3666

6301	FIG003879 Predicted amidohydrolase	fig|6666666.59329.peg.714	fig|6666666.59330.peg.3274	fig|6666666.59331.peg.4525	fig|6666666.59332.peg.5378	fig|6666666.59334.peg.4838	fig|6666666.59335.peg.2638	fig|6666666.59336.peg.4295	fig|6666666.59333.peg.2006	fig|6666666.59338.peg.3662	fig|6666666.59339.peg.1987	fig|6666666.59340.peg.2324	fig|6666666.59337.peg.2813	None	fig|6666666.71181.peg.904	fig|6666666.59344.peg.1599	fig|6666666.59345.peg.4877	fig|6666666.59341.peg.925	fig|6666666.59347.peg.797	fig|6666666.59346.peg.2135	fig|6666666.59348.peg.3171	fig|6666666.59349.peg.10	fig|6666666.59350.peg.235	fig|6666666.59351.peg.2629	fig|6666666.59353.peg.2299	fig|6666666.59355.peg.1574	fig|6666666.59354.peg.4212

9317	Na + -translocating NADH-quinone reductase subunit A EC 1.6.5.-	fig|6666666.59329.peg.1033	fig|6666666.59330.peg.2787	fig|6666666.59331.peg.432	fig|6666666.59332.peg.6192	fig|6666666.59334.peg.881	fig|6666666.59335.peg.119	fig|6666666.59336.peg.4526	fig|6666666.59333.peg.1798	fig|6666666.59338.peg.5895	fig|6666666.59339.peg.5045	None	fig|6666666.59337.peg.2379	fig|6666666.59342.peg.2130	fig|6666666.71181.peg.915	fig|6666666.59344.peg.5172	fig|6666666.59345.peg.2237	fig|6666666.59341.peg.2291	fig|6666666.59347.peg.2802	fig|6666666.59346.peg.2720	fig|6666666.59348.peg.1312	fig|6666666.59349.peg.307	fig|6666666.59350.peg.6059	fig|6666666.59351.peg.3931	fig|6666666.59353.peg.902	fig|6666666.59355.peg.804	fig|6666666.59354.peg.6052

6542	DNA or RNA helicase of superfamily II	fig|6666666.59329.peg.3312	fig|6666666.59330.peg.3691	fig|6666666.59331.peg.177	fig|6666666.59332.peg.498	fig|6666666.59334.peg.4756	fig|6666666.59335.peg.1350	fig|6666666.59336.peg.1481	fig|6666666.59333.peg.1194	fig|6666666.59338.peg.4572	fig|6666666.59339.peg.901	fig|6666666.59340.peg.3023	fig|6666666.59337.peg.4265	fig|6666666.59342.peg.12	fig|6666666.71181.peg.488	fig|6666666.59344.peg.1699	fig|6666666.59345.peg.1221	fig|6666666.59341.peg.868	fig|6666666.59347.peg.1997	fig|6666666.59346.peg.225	fig|6666666.59348.peg.4530	fig|6666666.59349.peg.1335	fig|6666666.59350.peg.674	fig|6666666.59351.peg.4241	fig|6666666.59353.peg.1115	fig|6666666.59355.peg.362	None

7518	Cys-tRNA Pro deacylase YbaK	fig|6666666.59329.peg.488	fig|6666666.59330.peg.5001	None	fig|6666666.59332.peg.2615	fig|6666666.59334.peg.2574	fig|6666666.59335.peg.1726	fig|6666666.59336.peg.6193	fig|6666666.59333.peg.225	fig|6666666.59338.peg.1526	fig|6666666.59339.peg.7352	fig|6666666.59340.peg.719	fig|6666666.59337.peg.4401	fig|6666666.59342.peg.6310	fig|6666666.71181.peg.3607	fig|6666666.59344.peg.5051	fig|6666666.59345.peg.5044	fig|6666666.59341.peg.4048	fig|6666666.59347.peg.3188	fig|6666666.59346.peg.322	fig|6666666.59348.peg.266	fig|6666666.59349.peg.4474	fig|6666666.59350.peg.4593	fig|6666666.59351.peg.1550	fig|6666666.59353.peg.4062	fig|6666666.59355.peg.4138	fig|6666666.59354.peg.5566

7078	Protein of unknown function DUF414	fig|6666666.59329.peg.1826	fig|6666666.59330.peg.393	fig|6666666.59331.peg.4933	fig|6666666.59332.peg.23	fig|6666666.59334.peg.4846	fig|6666666.59335.peg.55	fig|6666666.59336.peg.1451	fig|6666666.59333.peg.841	fig|6666666.59338.peg.6158	fig|6666666.59339.peg.7596	fig|6666666.59340.peg.1145	fig|6666666.59337.peg.977	fig|6666666.59342.peg.2842	fig|6666666.71181.peg.2588	fig|6666666.59344.peg.898	fig|6666666.59345.peg.2699	fig|6666666.59341.peg.4285	fig|6666666.59347.peg.5043	fig|6666666.59346.peg.1078	fig|6666666.59348.peg.100	fig|6666666.59349.peg.4815	None	fig|6666666.59351.peg.103	fig|6666666.59353.peg.2629	fig|6666666.59355.peg.2575	fig|6666666.59354.peg.3438

7075	IMP cyclohydrolase EC 3.5.4.10 Phosphoribosylaminoimidazolecarboxamide formyltransferase EC 2.1.2.3	fig|6666666.59329.peg.668	fig|6666666.59330.peg.4027	fig|6666666.59331.peg.3425	fig|6666666.59332.peg.128	fig|6666666.59334.peg.1583	fig|6666666.59335.peg.2634	fig|6666666.59336.peg.3384	fig|6666666.59333.peg.2195	fig|6666666.59338.peg.4395	fig|6666666.59339.peg.6432	None	fig|6666666.59337.peg.4503	fig|6666666.59342.peg.5946	fig|6666666.71181.peg.3348	fig|6666666.59344.peg.5676	fig|6666666.59345.peg.5670	fig|6666666.59341.peg.1427	fig|6666666.59347.peg.4486	fig|6666666.59346.peg.1145	fig|6666666.59348.peg.3308	fig|6666666.59349.peg.1948	fig|6666666.59350.peg.7217	fig|6666666.59351.peg.2358	fig|6666666.59353.peg.4474	fig|6666666.59355.peg.4552	fig|6666666.59354.peg.3

8683	Phosphoenolpyruvate carboxylase EC 4.1.1.31	fig|6666666.59329.peg.978	fig|6666666.59330.peg.1678	fig|6666666.59331.peg.3948	fig|6666666.59332.peg.2927	fig|6666666.59334.peg.2178	fig|6666666.59335.peg.451	fig|6666666.59336.peg.1596	fig|6666666.59333.peg.5137	fig|6666666.59338.peg.1057	fig|6666666.59339.peg.6421	fig|6666666.59340.peg.1892	fig|6666666.59337.peg.1680	fig|6666666.59342.peg.4417	fig|6666666.71181.peg.3884	fig|6666666.59344.peg.1010	fig|6666666.59345.peg.5400	fig|6666666.59341.peg.948	fig|6666666.59347.peg.4927	fig|6666666.59346.peg.999	fig|6666666.59348.peg.141	fig|6666666.59349.peg.4081	None	fig|6666666.59351.peg.3766	fig|6666666.59353.peg.2530	fig|6666666.59355.peg.2672	fig|6666666.59354.peg.457

5498	2-octaprenyl-3-methyl-6-methoxy-1,4-benzoquinol hydroxylase EC 1.14.13.-	fig|6666666.59329.peg.4342	fig|6666666.59330.peg.4032	fig|6666666.59331.peg.4589	fig|6666666.59332.peg.2935	fig|6666666.59334.peg.4278	fig|6666666.59335.peg.3025	fig|6666666.59336.peg.5217	fig|6666666.59333.peg.5061	fig|6666666.59338.peg.3622	fig|6666666.59339.peg.5226	fig|6666666.59340.peg.1983	fig|6666666.59337.peg.551	None	fig|6666666.71181.peg.3127	fig|6666666.59344.peg.4742	fig|6666666.59345.peg.5042	fig|6666666.59341.peg.4045	fig|6666666.59347.peg.3191	fig|6666666.59346.peg.319	fig|6666666.59348.peg.268	fig|6666666.59349.peg.4328	fig|6666666.59350.peg.4660	fig|6666666.59351.peg.2192	fig|6666666.59353.peg.2040	fig|6666666.59355.peg.4520	fig|6666666.59354.peg.2862

2458	Glycyl-tRNA synthetase alpha chain EC 6.1.1.14	fig|6666666.59329.peg.3104	fig|6666666.59330.peg.1094	fig|6666666.59331.peg.3074	fig|6666666.59332.peg.75	fig|6666666.59334.peg.145	fig|6666666.59335.peg.3923	fig|6666666.59336.peg.1	fig|6666666.59333.peg.3962	fig|6666666.59338.peg.6018	fig|6666666.59339.peg.8048	None	fig|6666666.59337.peg.866	fig|6666666.59342.peg.2614	fig|6666666.71181.peg.658	fig|6666666.59344.peg.4133	fig|6666666.59345.peg.4987	fig|6666666.59341.peg.5112	fig|6666666.59347.peg.4805	fig|6666666.59346.peg.4287	fig|6666666.59348.peg.1209	fig|6666666.59349.peg.993	fig|6666666.59350.peg.2814	fig|6666666.59351.peg.5033	fig|6666666.59353.peg.4111	fig|6666666.59355.peg.2328	fig|6666666.59354.peg.576

5497	Autolysin sensor kinase EC 2.7.3.-	fig|6666666.59329.peg.1380	fig|6666666.59330.peg.2517	fig|6666666.59331.peg.4252	fig|6666666.59332.peg.1085	fig|6666666.59334.peg.2672	fig|6666666.59335.peg.4995	fig|6666666.59336.peg.313	fig|6666666.59333.peg.1209	fig|6666666.59338.peg.6156	fig|6666666.59339.peg.4061	fig|6666666.59340.peg.1902	fig|6666666.59337.peg.220	fig|6666666.59342.peg.2139	fig|6666666.71181.peg.1815	fig|6666666.59344.peg.3085	fig|6666666.59345.peg.3704	fig|6666666.59341.peg.103	fig|6666666.59347.peg.4044	fig|6666666.59346.peg.161	fig|6666666.59348.peg.3071	fig|6666666.59349.peg.399	None	fig|6666666.59351.peg.506	fig|6666666.59353.peg.2236	fig|6666666.59355.peg.1929	fig|6666666.59354.peg.6020

5446	Inner membrane protein translocase component YidC, long form	fig|6666666.59329.peg.2418	fig|6666666.59330.peg.1109	fig|6666666.59331.peg.1547	fig|6666666.59332.peg.90	fig|6666666.59334.peg.1675	fig|6666666.59335.peg.784	fig|6666666.59336.peg.19	fig|6666666.59333.peg.42	fig|6666666.59338.peg.3683	fig|6666666.59339.peg.8028	fig|6666666.59340.peg.139	fig|6666666.59337.peg.882	fig|6666666.59342.peg.512	fig|6666666.71181.peg.620	fig|6666666.59344.peg.4166	fig|6666666.59345.peg.4973	fig|6666666.59341.peg.1050	fig|6666666.59347.peg.4791	fig|6666666.59346.peg.1187	fig|6666666.59348.peg.308	fig|6666666.59349.peg.1011	None	fig|6666666.59351.peg.5025	fig|6666666.59353.peg.3060	fig|6666666.59355.peg.2287	fig|6666666.59354.peg.1564

3821	Cysteine desulfurase EC 2.8.1.7 , IscS subfamily	fig|6666666.59329.peg.1111	fig|6666666.59330.peg.1414	fig|6666666.59331.peg.1217	fig|6666666.59332.peg.1430	fig|6666666.59334.peg.3064	fig|6666666.59335.peg.2367	fig|6666666.59336.peg.667	fig|6666666.59333.peg.924	fig|6666666.59338.peg.1326	fig|6666666.59339.peg.6790	None	fig|6666666.59337.peg.739	fig|6666666.59342.peg.5752	fig|6666666.71181.peg.1757	fig|6666666.59344.peg.1627	fig|6666666.59345.peg.3652	fig|6666666.59341.peg.4636	fig|6666666.59347.peg.4283	fig|6666666.59346.peg.2740	fig|6666666.59348.peg.1379	fig|6666666.59349.peg.3197	fig|6666666.59350.peg.6526	fig|6666666.59351.peg.4925	fig|6666666.59353.peg.2694	fig|6666666.59355.peg.1785	fig|6666666.59354.peg.4567

5000	Membrane protein, suppressor for copper-sensitivity ScsB	fig|6666666.59329.peg.1068	fig|6666666.59330.peg.177	fig|6666666.59331.peg.1201	fig|6666666.59332.peg.3109	fig|6666666.59334.peg.2521	fig|6666666.59335.peg.2290	fig|6666666.59336.peg.2455	fig|6666666.59333.peg.409	None	fig|6666666.59339.peg.4149	fig|6666666.59340.peg.2564	fig|6666666.59337.peg.2906	fig|6666666.59342.peg.3651	fig|6666666.71181.peg.1496	fig|6666666.59344.peg.622	fig|6666666.59345.peg.5129	fig|6666666.59341.peg.5495	fig|6666666.59347.peg.651	fig|6666666.59346.peg.670	fig|6666666.59348.peg.2230	fig|6666666.59349.peg.2364	fig|6666666.59350.peg.1968	fig|6666666.59351.peg.109	fig|6666666.59353.peg.1042	fig|6666666.59355.peg.3577	fig|6666666.59354.peg.3261

7538	Orotate phosphoribosyltransferase EC 2.4.2.10	fig|6666666.59329.peg.3642	fig|6666666.59330.peg.1173	fig|6666666.59331.peg.5208	fig|6666666.59332.peg.716	fig|6666666.59334.peg.228	fig|6666666.59335.peg.1986	fig|6666666.59336.peg.4650	fig|6666666.59333.peg.663	fig|6666666.59338.peg.4348	fig|6666666.59339.peg.6277	fig|6666666.59340.peg.29	fig|6666666.59337.peg.478	None	fig|6666666.71181.peg.2613	fig|6666666.59344.peg.934	fig|6666666.59345.peg.2626	fig|6666666.59341.peg.6063	fig|6666666.59347.peg.5193	fig|6666666.59346.peg.1395	fig|6666666.59348.peg.735	fig|6666666.59349.peg.3071	fig|6666666.59350.peg.6449	fig|6666666.59351.peg.334	fig|6666666.59353.peg.2598	fig|6666666.59355.peg.2604	fig|6666666.59354.peg.3333

7052	Cell division protein FtsK	fig|6666666.59329.peg.3408	fig|6666666.59330.peg.1926	fig|6666666.59331.peg.5693	fig|6666666.59332.peg.4221	fig|6666666.59334.peg.2807	fig|6666666.59335.peg.1299	fig|6666666.59336.peg.2306	fig|6666666.59333.peg.2038	fig|6666666.59338.peg.2187	fig|6666666.59339.peg.3427	None	fig|6666666.59337.peg.4863	fig|6666666.59342.peg.2397	fig|6666666.71181.peg.3154	fig|6666666.59344.peg.2821	fig|6666666.59345.peg.1339	fig|6666666.59341.peg.60	fig|6666666.59347.peg.2396	fig|6666666.59346.peg.1578	fig|6666666.59348.peg.4388	fig|6666666.59349.peg.1583	fig|6666666.59350.peg.445	fig|6666666.59351.peg.1000	fig|6666666.59353.peg.1630	fig|6666666.59355.peg.1090	fig|6666666.59354.peg.2186

9482	Sugar transferase SypR involved in lipopolysaccharide synthesis	fig|6666666.59329.peg.550	fig|6666666.59330.peg.1336	fig|6666666.59331.peg.22	fig|6666666.59332.peg.2403	fig|6666666.59334.peg.5334	fig|6666666.59335.peg.407	fig|6666666.59336.peg.5489	fig|6666666.59333.peg.3782	fig|6666666.59338.peg.5639	fig|6666666.59339.peg.1149	None	fig|6666666.59337.peg.4716	fig|6666666.59342.peg.4037	fig|6666666.71181.peg.217	fig|6666666.59344.peg.268	fig|6666666.59345.peg.625	fig|6666666.59341.peg.739	fig|6666666.59347.peg.5021	fig|6666666.59346.peg.4449	fig|6666666.59348.peg.4956	fig|6666666.59349.peg.787	fig|6666666.59350.peg.842	fig|6666666.59351.peg.260	fig|6666666.59353.peg.604	fig|6666666.59355.peg.86	fig|6666666.59354.peg.3012

7990	Uncharacterized protein YtfM precursor	fig|6666666.59329.peg.293	fig|6666666.59330.peg.603	fig|6666666.59331.peg.1155	fig|6666666.59332.peg.3144	fig|6666666.59334.peg.2113	fig|6666666.59335.peg.172	fig|6666666.59336.peg.927	fig|6666666.59333.peg.3134	fig|6666666.59338.peg.4826	fig|6666666.59339.peg.7719	None	fig|6666666.59337.peg.340	fig|6666666.59342.peg.722	fig|6666666.71181.peg.2808	fig|6666666.59344.peg.2135	fig|6666666.59345.peg.2488	fig|6666666.59341.peg.799	fig|6666666.59347.peg.4867	fig|6666666.59346.peg.1976	fig|6666666.59348.peg.3837	fig|6666666.59349.peg.1338	fig|6666666.59350.peg.7056	fig|6666666.59351.peg.4238	fig|6666666.59353.peg.3166	fig|6666666.59355.peg.431	fig|6666666.59354.peg.3226

7337	L-serine dehydratase EC 4.3.1.17	fig|6666666.59329.peg.179	fig|6666666.59330.peg.2567	None	fig|6666666.59332.peg.5503	fig|6666666.59334.peg.1363	fig|6666666.59335.peg.5253	fig|6666666.59336.peg.3082	fig|6666666.59333.peg.1065	fig|6666666.59338.peg.4376	fig|6666666.59339.peg.4512	fig|6666666.59340.peg.2118	fig|6666666.59337.peg.4672	fig|6666666.59342.peg.3018	fig|6666666.71181.peg.3850	fig|6666666.59344.peg.2562	fig|6666666.59345.peg.2938	fig|6666666.59341.peg.5790	fig|6666666.59347.peg.2157	fig|6666666.59346.peg.2821	fig|6666666.59348.peg.2904	fig|6666666.59349.peg.2939	fig|6666666.59350.peg.3312	fig|6666666.59351.peg.2733	fig|6666666.59353.peg.553	fig|6666666.59355.peg.2076	fig|6666666.59354.peg.5966

10214	Phosphopantothenoylcysteine decarboxylase EC 4.1.1.36 Phosphopantothenoylcysteine synthetase EC 6.3.2.5	fig|6666666.59329.peg.3840	fig|6666666.59330.peg.1170	fig|6666666.59331.peg.3118	fig|6666666.59332.peg.4524	fig|6666666.59334.peg.244	fig|6666666.59335.peg.1991	fig|6666666.59336.peg.5901	fig|6666666.59333.peg.659	fig|6666666.59338.peg.1115	fig|6666666.59339.peg.6286	fig|6666666.59340.peg.12	fig|6666666.59337.peg.475	None	fig|6666666.71181.peg.2616	fig|6666666.59344.peg.938	fig|6666666.59345.peg.2623	fig|6666666.59341.peg.209	fig|6666666.59347.peg.4518	fig|6666666.59346.peg.1401	fig|6666666.59348.peg.729	fig|6666666.59349.peg.109	fig|6666666.59350.peg.2439	fig|6666666.59351.peg.165	fig|6666666.59353.peg.2594	fig|6666666.59355.peg.2608	fig|6666666.59354.peg.3709

52	RNA polymerase sigma factor RpoS	fig|6666666.59329.peg.4289	fig|6666666.59330.peg.2456	fig|6666666.59331.peg.3583	fig|6666666.59332.peg.205	fig|6666666.59334.peg.274	fig|6666666.59335.peg.1662	fig|6666666.59336.peg.2266	fig|6666666.59333.peg.1716	fig|6666666.59338.peg.3172	fig|6666666.59339.peg.7924	fig|6666666.59340.peg.1095	fig|6666666.59337.peg.585	fig|6666666.59342.peg.1268	fig|6666666.71181.peg.3422	fig|6666666.59344.peg.2191	fig|6666666.59345.peg.5075	fig|6666666.59341.peg.2099	fig|6666666.59347.peg.3955	fig|6666666.59346.peg.3271	None	fig|6666666.59349.peg.4210	fig|6666666.59350.peg.2438	fig|6666666.59351.peg.5471	fig|6666666.59353.peg.215	fig|6666666.59355.peg.2984	fig|6666666.59354.peg.3398

2430	Cytochrome c-type biogenesis protein CcmE, heme chaperone	fig|6666666.59329.peg.2318	fig|6666666.59330.peg.4377	fig|6666666.59331.peg.3837	fig|6666666.59332.peg.2662	fig|6666666.59334.peg.2181	fig|6666666.59335.peg.1250	fig|6666666.59336.peg.4941	fig|6666666.59333.peg.2595	fig|6666666.59338.peg.3956	fig|6666666.59339.peg.3029	None	fig|6666666.59337.peg.4223	fig|6666666.59342.peg.4685	fig|6666666.71181.peg.1527	fig|6666666.59344.peg.4980	fig|6666666.59345.peg.5312	fig|6666666.59341.peg.3754	fig|6666666.59347.peg.5093	fig|6666666.59346.peg.5097	fig|6666666.59348.peg.4629	fig|6666666.59349.peg.179	fig|6666666.59350.peg.1732	fig|6666666.59351.peg.2086	fig|6666666.59353.peg.4869	fig|6666666.59355.peg.215	fig|6666666.59354.peg.5194

1496	LSU ribosomal protein L19p	fig|6666666.59329.peg.339	fig|6666666.59330.peg.2847	fig|6666666.59331.peg.1988	fig|6666666.59332.peg.312	None	fig|6666666.59335.peg.5394	fig|6666666.59336.peg.3002	fig|6666666.59333.peg.158	fig|6666666.59338.peg.2609	fig|6666666.59339.peg.7936	fig|6666666.59340.peg.3477	fig|6666666.59337.peg.2044	fig|6666666.59342.peg.2102	fig|6666666.71181.peg.3954	fig|6666666.59344.peg.5440	fig|6666666.59345.peg.2430	fig|6666666.59341.peg.1345	fig|6666666.59347.peg.3887	fig|6666666.59346.peg.572	fig|6666666.59348.peg.1262	fig|6666666.59349.peg.606	fig|6666666.59350.peg.2761	fig|6666666.59351.peg.543	fig|6666666.59353.peg.4811	fig|6666666.59355.peg.4865	fig|6666666.59354.peg.3768

1498	Peptide methionine sulfoxide reductase MsrA EC 1.8.4.11	fig|6666666.59329.peg.3065	fig|6666666.59330.peg.3550	fig|6666666.59331.peg.5806	fig|6666666.59332.peg.1201	fig|6666666.59334.peg.64	fig|6666666.59335.peg.4393	fig|6666666.59336.peg.929	fig|6666666.59333.peg.2098	fig|6666666.59338.peg.3134	fig|6666666.59339.peg.2167	None	fig|6666666.59337.peg.2228	fig|6666666.59342.peg.3954	fig|6666666.71181.peg.2809	fig|6666666.59344.peg.2136	fig|6666666.59345.peg.2990	fig|6666666.59341.peg.798	fig|6666666.59347.peg.93	fig|6666666.59346.peg.1058	fig|6666666.59348.peg.2574	fig|6666666.59349.peg.2498	fig|6666666.59350.peg.1491	fig|6666666.59351.peg.2298	fig|6666666.59353.peg.3167	fig|6666666.59355.peg.430	fig|6666666.59354.peg.3473

1233	rRNA small subunit 7-methylguanosine m7G methyltransferase GidB	fig|6666666.59329.peg.1743	fig|6666666.59330.peg.1115	fig|6666666.59331.peg.3960	fig|6666666.59332.peg.95	fig|6666666.59334.peg.30	fig|6666666.59335.peg.788	fig|6666666.59336.peg.23	fig|6666666.59333.peg.33	fig|6666666.59338.peg.489	fig|6666666.59339.peg.8021	None	fig|6666666.59337.peg.886	fig|6666666.59342.peg.1192	fig|6666666.71181.peg.590	fig|6666666.59344.peg.4262	fig|6666666.59345.peg.4969	fig|6666666.59341.peg.1055	fig|6666666.59347.peg.4787	fig|6666666.59346.peg.1180	fig|6666666.59348.peg.327	fig|6666666.59349.peg.1025	fig|6666666.59350.peg.1505	fig|6666666.59351.peg.5363	fig|6666666.59353.peg.3090	fig|6666666.59355.peg.2258	fig|6666666.59354.peg.1144

1964	Cob I alamin adenosyltransferase EC 2.5.1.17	fig|6666666.59329.peg.1603	fig|6666666.59330.peg.3715	fig|6666666.59331.peg.705	fig|6666666.59332.peg.5317	fig|6666666.59334.peg.1184	fig|6666666.59335.peg.3116	fig|6666666.59336.peg.3546	fig|6666666.59333.peg.1546	fig|6666666.59338.peg.2823	fig|6666666.59339.peg.170	None	fig|6666666.59337.peg.3852	fig|6666666.59342.peg.4763	fig|6666666.71181.peg.4155	fig|6666666.59344.peg.470	fig|6666666.59345.peg.1076	fig|6666666.59341.peg.1109	fig|6666666.59347.peg.2615	fig|6666666.59346.peg.3932	fig|6666666.59348.peg.1461	fig|6666666.59349.peg.4235	fig|6666666.59350.peg.3948	fig|6666666.59351.peg.3914	fig|6666666.59353.peg.1790	fig|6666666.59355.peg.1225	fig|6666666.59354.peg.2754

8289	Hydroxymethylpyrimidine phosphate synthase ThiC	fig|6666666.59329.peg.3964	fig|6666666.59330.peg.976	fig|6666666.59331.peg.5668	fig|6666666.59332.peg.1931	fig|6666666.59334.peg.1097	fig|6666666.59335.peg.3	fig|6666666.59336.peg.174	fig|6666666.59333.peg.3927	fig|6666666.59338.peg.1664	fig|6666666.59339.peg.7526	fig|6666666.59340.peg.602	fig|6666666.59337.peg.2554	None	fig|6666666.71181.peg.3357	fig|6666666.59344.peg.5685	fig|6666666.59345.peg.5546	fig|6666666.59341.peg.5653	fig|6666666.59347.peg.5258	fig|6666666.59346.peg.935	fig|6666666.59348.peg.1093	fig|6666666.59349.peg.3790	fig|6666666.59350.peg.2933	fig|6666666.59351.peg.3883	fig|6666666.59353.peg.4466	fig|6666666.59355.peg.4561	fig|6666666.59354.peg.6178

3809	AmpG permease	fig|6666666.59329.peg.1041	fig|6666666.59330.peg.2792	fig|6666666.59331.peg.3423	fig|6666666.59332.peg.862	fig|6666666.59334.peg.1278	fig|6666666.59335.peg.113	fig|6666666.59336.peg.4677	fig|6666666.59333.peg.1932	fig|6666666.59338.peg.2003	fig|6666666.59339.peg.5039	fig|6666666.59340.peg.1805	fig|6666666.59337.peg.2374	fig|6666666.59342.peg.3223	fig|6666666.71181.peg.2047	fig|6666666.59344.peg.1074	fig|6666666.59345.peg.2242	fig|6666666.59341.peg.5028	fig|6666666.59347.peg.2808	fig|6666666.59346.peg.2713	fig|6666666.59348.peg.2932	fig|6666666.59349.peg.331	None	fig|6666666.59351.peg.3938	fig|6666666.59353.peg.758	fig|6666666.59355.peg.946	fig|6666666.59354.peg.4180

4179	hypothetical protein	fig|6666666.59329.peg.5207	fig|6666666.59330.peg.1230	fig|6666666.59331.peg.5992	fig|6666666.59332.peg.1909	fig|6666666.59334.peg.1721	fig|6666666.59335.peg.3830	fig|6666666.59336.peg.2978	fig|6666666.59333.peg.2219	fig|6666666.59338.peg.6042	fig|6666666.59339.peg.256	fig|6666666.59340.peg.3485	fig|6666666.59337.peg.3751	fig|6666666.59342.peg.4018	fig|6666666.71181.peg.331	fig|6666666.59344.peg.1270	fig|6666666.59345.peg.1175	fig|6666666.59341.peg.6040	fig|6666666.59347.peg.1171	fig|6666666.59346.peg.3852	fig|6666666.59348.peg.1659	fig|6666666.59349.peg.1650	None	fig|6666666.59351.peg.2231	fig|6666666.59353.peg.4927	fig|6666666.59355.peg.4970	fig|6666666.59354.peg.756

9130	Putative acetate efflux pump, MadN	fig|6666666.59329.peg.4667	fig|6666666.59330.peg.234	fig|6666666.59331.peg.562	fig|6666666.59332.peg.1719	fig|6666666.59334.peg.2958	fig|6666666.59335.peg.24	fig|6666666.59336.peg.6104	fig|6666666.59333.peg.676	fig|6666666.59338.peg.75	fig|6666666.59339.peg.7549	fig|6666666.59340.peg.161	fig|6666666.59337.peg.950	fig|6666666.59342.peg.5141	fig|6666666.71181.peg.4411	fig|6666666.59344.peg.3427	fig|6666666.59345.peg.2725	fig|6666666.59341.peg.4150	fig|6666666.59347.peg.4571	fig|6666666.59346.peg.3444	fig|6666666.59348.peg.2083	fig|6666666.59349.peg.2922	None	fig|6666666.59351.peg.4014	fig|6666666.59353.peg.3789	fig|6666666.59355.peg.3384	fig|6666666.59354.peg.3148

1008	DNA-directed RNA polymerase beta' subunit EC 2.7.7.6	fig|6666666.59329.peg.629	fig|6666666.59330.peg.3411	fig|6666666.59331.peg.2951	fig|6666666.59332.peg.140	fig|6666666.59334.peg.1234	fig|6666666.59335.peg.1942	fig|6666666.59336.peg.5204	None	fig|6666666.59338.peg.3039	fig|6666666.59339.peg.8835	fig|6666666.59340.peg.3398	fig|6666666.59337.peg.4993	fig|6666666.59342.peg.364	fig|6666666.71181.peg.3368	fig|6666666.59344.peg.5454	fig|6666666.59345.peg.5652	fig|6666666.59341.peg.3796	fig|6666666.59347.peg.5396	fig|6666666.59346.peg.1132	fig|6666666.59348.peg.1008	fig|6666666.59349.peg.1140	fig|6666666.59350.peg.7664	fig|6666666.59351.peg.5245	fig|6666666.59353.peg.4455	fig|6666666.59355.peg.4572	fig|6666666.59354.peg.3581

4343	Urocanate hydratase EC 4.2.1.49	fig|6666666.59329.peg.5103	fig|6666666.59330.peg.507	fig|6666666.59331.peg.3436	fig|6666666.59332.peg.3276	None	fig|6666666.59335.peg.1564	fig|6666666.59336.peg.2094	fig|6666666.59333.peg.3575	fig|6666666.59338.peg.430	fig|6666666.59339.peg.3872	fig|6666666.59340.peg.2670	fig|6666666.59337.peg.3306	fig|6666666.59342.peg.3862	fig|6666666.71181.peg.3782	fig|6666666.59344.peg.2220	fig|6666666.59345.peg.4627	fig|6666666.59341.peg.1313	fig|6666666.59347.peg.1559	fig|6666666.59346.peg.3556	fig|6666666.59348.peg.3580	fig|6666666.59349.peg.497	fig|6666666.59350.peg.4118	fig|6666666.59351.peg.4290	fig|6666666.59353.peg.191	fig|6666666.59355.peg.2116	fig|6666666.59354.peg.944

9585	LSU ribosomal protein L6p L9e	fig|6666666.59329.peg.724	fig|6666666.59330.peg.565	fig|6666666.59331.peg.37	fig|6666666.59332.peg.2150	fig|6666666.59334.peg.4017	fig|6666666.59335.peg.4358	fig|6666666.59336.peg.157	fig|6666666.59333.peg.377	fig|6666666.59338.peg.4971	fig|6666666.59339.peg.8278	None	fig|6666666.59337.peg.377	fig|6666666.59342.peg.315	fig|6666666.71181.peg.3446	fig|6666666.59344.peg.4929	fig|6666666.59345.peg.2524	fig|6666666.59341.peg.2509	fig|6666666.59347.peg.4834	fig|6666666.59346.peg.861	fig|6666666.59348.peg.1771	fig|6666666.59349.peg.3281	fig|6666666.59350.peg.3182	fig|6666666.59351.peg.4215	fig|6666666.59353.peg.4604	fig|6666666.59355.peg.4727	fig|6666666.59354.peg.3854

10825	tRNA-specific 2-thiouridylase MnmA	fig|6666666.59329.peg.5828	fig|6666666.59330.peg.2869	None	fig|6666666.59332.peg.5039	fig|6666666.59334.peg.4500	fig|6666666.59335.peg.599	fig|6666666.59336.peg.3107	fig|6666666.59333.peg.4986	fig|6666666.59338.peg.401	fig|6666666.59339.peg.482	fig|6666666.59340.peg.2663	fig|6666666.59337.peg.5578	fig|6666666.59342.peg.537	fig|6666666.71181.peg.1246	fig|6666666.59344.peg.162	fig|6666666.59345.peg.1314	fig|6666666.59341.peg.490	fig|6666666.59347.peg.4133	fig|6666666.59346.peg.1562	fig|6666666.59348.peg.3605	fig|6666666.59349.peg.2888	fig|6666666.59350.peg.1094	fig|6666666.59351.peg.872	fig|6666666.59353.peg.473	fig|6666666.59355.peg.2198	fig|6666666.59354.peg.5491

9373	Sulfite reductase [NADPH] flavoprotein alpha-component EC 1.8.1.2	fig|6666666.59329.peg.4908	fig|6666666.59330.peg.4456	fig|6666666.59331.peg.2623	fig|6666666.59332.peg.1874	fig|6666666.59334.peg.157	fig|6666666.59335.peg.336	fig|6666666.59336.peg.615	fig|6666666.59333.peg.874	fig|6666666.59338.peg.5172	fig|6666666.59339.peg.8328	fig|6666666.59340.peg.2196	fig|6666666.59337.peg.1725	fig|6666666.59342.peg.2487	fig|6666666.71181.peg.3402	fig|6666666.59344.peg.2214	fig|6666666.59345.peg.5359	fig|6666666.59341.peg.1431	fig|6666666.59347.peg.4912	fig|6666666.59346.peg.3209	fig|6666666.59348.peg.2158	fig|6666666.59349.peg.1173	None	fig|6666666.59351.peg.52	fig|6666666.59353.peg.195	fig|6666666.59355.peg.2964	fig|6666666.59354.peg.2822

2935	Ammonium transporter	fig|6666666.59329.peg.6036	fig|6666666.59330.peg.1768	None	fig|6666666.59332.peg.246	fig|6666666.59334.peg.890	fig|6666666.59335.peg.1526	fig|6666666.59336.peg.562	fig|6666666.59333.peg.327	fig|6666666.59338.peg.1915	fig|6666666.59339.peg.2035	fig|6666666.59340.peg.3351	fig|6666666.59337.peg.638	fig|6666666.59342.peg.4938	fig|6666666.71181.peg.2067	fig|6666666.59344.peg.1048	fig|6666666.59345.peg.2395	fig|6666666.59341.peg.1799	fig|6666666.59347.peg.1724	fig|6666666.59346.peg.3586	fig|6666666.59348.peg.1527	fig|6666666.59349.peg.344	fig|6666666.59350.peg.46	fig|6666666.59351.peg.3949	fig|6666666.59353.peg.737	fig|6666666.59355.peg.968	fig|6666666.59354.peg.1482

504	FIG001341 Probable Fe 2+ -trafficking protein YggX	fig|6666666.59329.peg.1533	fig|6666666.59330.peg.331	fig|6666666.59331.peg.4812	fig|6666666.59332.peg.6143	fig|6666666.59334.peg.1379	fig|6666666.59335.peg.3039	fig|6666666.59336.peg.5568	fig|6666666.59333.peg.753	fig|6666666.59338.peg.5287	fig|6666666.59339.peg.7807	fig|6666666.59340.peg.1332	fig|6666666.59337.peg.510	fig|6666666.59342.peg.5165	fig|6666666.71181.peg.1878	fig|6666666.59344.peg.4354	fig|6666666.59345.peg.5000	fig|6666666.59341.peg.3066	fig|6666666.59347.peg.3877	fig|6666666.59346.peg.4582	fig|6666666.59348.peg.234	fig|6666666.59349.peg.3657	None	fig|6666666.59351.peg.299	fig|6666666.59353.peg.3243	fig|6666666.59355.peg.3126	fig|6666666.59354.peg.3158

638	Malate dehydrogenase EC 1.1.1.37	fig|6666666.59329.peg.2970	fig|6666666.59330.peg.829	None	fig|6666666.59332.peg.5198	fig|6666666.59334.peg.4470	fig|6666666.59335.peg.234	fig|6666666.59336.peg.930	fig|6666666.59333.peg.555	fig|6666666.59338.peg.1820	fig|6666666.59339.peg.8166	fig|6666666.59340.peg.3018	fig|6666666.59337.peg.1371	fig|6666666.59342.peg.5277	fig|6666666.71181.peg.2798	fig|6666666.59344.peg.2122	fig|6666666.59345.peg.2471	fig|6666666.59341.peg.1989	fig|6666666.59347.peg.3100	fig|6666666.59346.peg.3986	fig|6666666.59348.peg.563	fig|6666666.59349.peg.352	fig|6666666.59350.peg.2852	fig|6666666.59351.peg.3958	fig|6666666.59353.peg.3155	fig|6666666.59355.peg.441	fig|6666666.59354.peg.389

6525	Adenosine deaminase EC 3.5.4.4	fig|6666666.59329.peg.4053	fig|6666666.59330.peg.4081	fig|6666666.59331.peg.1581	fig|6666666.59332.peg.1760	fig|6666666.59334.peg.4479	fig|6666666.59335.peg.4326	fig|6666666.59336.peg.3618	fig|6666666.59333.peg.1025	fig|6666666.59338.peg.4085	fig|6666666.59339.peg.6163	fig|6666666.59340.peg.3533	fig|6666666.59337.peg.982	fig|6666666.59342.peg.5700	fig|6666666.71181.peg.2690	fig|6666666.59344.peg.3858	fig|6666666.59345.peg.2696	fig|6666666.59341.peg.2150	fig|6666666.59347.peg.617	fig|6666666.59346.peg.2246	fig|6666666.59348.peg.3896	fig|6666666.59349.peg.1566	None	fig|6666666.59351.peg.4760	fig|6666666.59353.peg.3988	fig|6666666.59355.peg.3904	fig|6666666.59354.peg.1077

8405	Xanthine uracil thiamine ascorbate permease family protein	fig|6666666.59329.peg.1022	fig|6666666.59330.peg.1069	fig|6666666.59331.peg.5211	fig|6666666.59332.peg.723	fig|6666666.59334.peg.1660	fig|6666666.59335.peg.142	fig|6666666.59336.peg.2724	fig|6666666.59333.peg.3416	fig|6666666.59338.peg.6016	fig|6666666.59339.peg.5080	fig|6666666.59340.peg.151	fig|6666666.59337.peg.5782	fig|6666666.59342.peg.451	fig|6666666.71181.peg.6086	fig|6666666.59344.peg.4158	fig|6666666.59345.peg.3549	fig|6666666.59341.peg.3185	fig|6666666.59347.peg.3332	fig|6666666.59346.peg.1387	fig|6666666.59348.peg.125	fig|6666666.59349.peg.3442	None	fig|6666666.59351.peg.2728	fig|6666666.59353.peg.733	fig|6666666.59355.peg.2280	fig|6666666.59354.peg.2206

4565	Sulfate transporter, CysZ-type	fig|6666666.59329.peg.910	fig|6666666.59330.peg.3033	fig|6666666.59331.peg.3688	fig|6666666.59332.peg.636	fig|6666666.59334.peg.3261	fig|6666666.59335.peg.3019	fig|6666666.59336.peg.2341	fig|6666666.59333.peg.5510	fig|6666666.59338.peg.20	fig|6666666.59339.peg.5234	fig|6666666.59340.peg.2230	fig|6666666.59337.peg.5548	fig|6666666.59342.peg.109	fig|6666666.71181.peg.1576	fig|6666666.59344.peg.2009	fig|6666666.59345.peg.4390	fig|6666666.59341.peg.4534	fig|6666666.59347.peg.2898	fig|6666666.59346.peg.2378	fig|6666666.59348.peg.5152	fig|6666666.59349.peg.2582	None	fig|6666666.59351.peg.4642	fig|6666666.59353.peg.2915	fig|6666666.59355.peg.2824	fig|6666666.59354.peg.6112

6068	Excinuclease ABC subunit B	fig|6666666.59329.peg.2668	fig|6666666.59330.peg.1705	fig|6666666.59331.peg.5390	fig|6666666.59332.peg.1515	fig|6666666.59334.peg.290	fig|6666666.59335.peg.2387	fig|6666666.59336.peg.1325	fig|6666666.59333.peg.5220	fig|6666666.59338.peg.1625	fig|6666666.59339.peg.133	None	fig|6666666.59337.peg.4018	fig|6666666.59342.peg.4828	fig|6666666.71181.peg.2528	fig|6666666.59344.peg.4096	fig|6666666.59345.peg.1118	fig|6666666.59341.peg.5456	fig|6666666.59347.peg.5163	fig|6666666.59346.peg.527	fig|6666666.59348.peg.711	fig|6666666.59349.peg.1861	fig|6666666.59350.peg.2504	fig|6666666.59351.peg.989	fig|6666666.59353.peg.2138	fig|6666666.59355.peg.2682	fig|6666666.59354.peg.6298

2389	Ribonucleotide reductase transcriptional regulator NrdR	fig|6666666.59329.peg.5360	fig|6666666.59330.peg.1062	fig|6666666.59331.peg.6148	fig|6666666.59332.peg.5882	fig|6666666.59334.peg.5616	fig|6666666.59335.peg.149	fig|6666666.59336.peg.2496	fig|6666666.59333.peg.3424	fig|6666666.59338.peg.3399	fig|6666666.59339.peg.5090	fig|6666666.59340.peg.2102	fig|6666666.59337.peg.1142	None	fig|6666666.71181.peg.2729	fig|6666666.59344.peg.1101	fig|6666666.59345.peg.3543	fig|6666666.59341.peg.2612	fig|6666666.59347.peg.2771	fig|6666666.59346.peg.3378	fig|6666666.59348.peg.4352	fig|6666666.59349.peg.3479	fig|6666666.59350.peg.1720	fig|6666666.59351.peg.4254	fig|6666666.59353.peg.780	fig|6666666.59355.peg.924	fig|6666666.59354.peg.2933

6042	Formyltetrahydrofolate deformylase EC 3.5.1.10	fig|6666666.59329.peg.4579	fig|6666666.59330.peg.4000	None	fig|6666666.59332.peg.937	fig|6666666.59334.peg.5006	fig|6666666.59335.peg.2028	fig|6666666.59336.peg.3718	fig|6666666.59333.peg.5590	fig|6666666.59338.peg.2053	fig|6666666.59339.peg.3145	fig|6666666.59340.peg.2744	fig|6666666.59337.peg.2739	fig|6666666.59342.peg.6233	fig|6666666.71181.peg.2755	fig|6666666.59344.peg.4031	fig|6666666.59345.peg.2168	fig|6666666.59341.peg.4245	fig|6666666.59347.peg.4405	fig|6666666.59346.peg.1486	fig|6666666.59348.peg.3678	fig|6666666.59349.peg.3015	fig|6666666.59350.peg.6620	fig|6666666.59351.peg.3361	fig|6666666.59353.peg.1737	fig|6666666.59355.peg.3978	fig|6666666.59354.peg.3275

7358	Diaminopimelate epimerase EC 5.1.1.7	fig|6666666.59329.peg.89	fig|6666666.59330.peg.3053	None	fig|6666666.59332.peg.4406	fig|6666666.59334.peg.1130	fig|6666666.59335.peg.3337	fig|6666666.59336.peg.1116	fig|6666666.59333.peg.791	fig|6666666.59338.peg.1782	fig|6666666.59339.peg.8525	fig|6666666.59340.peg.1885	fig|6666666.59337.peg.1618	fig|6666666.59342.peg.3160	fig|6666666.71181.peg.2432	fig|6666666.59344.peg.2487	fig|6666666.59345.peg.5501	fig|6666666.59341.peg.3920	fig|6666666.59347.peg.5075	fig|6666666.59346.peg.601	fig|6666666.59348.peg.5065	fig|6666666.59349.peg.1556	fig|6666666.59350.peg.2362	fig|6666666.59351.peg.4751	fig|6666666.59353.peg.3631	fig|6666666.59355.peg.4993	fig|6666666.59354.peg.1453

10272	Citrate synthase si EC 2.3.3.1	fig|6666666.59329.peg.5811	fig|6666666.59330.peg.4920	fig|6666666.59331.peg.68	fig|6666666.59332.peg.3971	fig|6666666.59334.peg.3834	fig|6666666.59335.peg.3343	fig|6666666.59336.peg.5431	fig|6666666.59333.peg.2960	fig|6666666.59338.peg.3194	fig|6666666.59339.peg.7197	fig|6666666.59340.peg.2536	fig|6666666.59337.peg.4104	None	fig|6666666.71181.peg.2128	fig|6666666.59344.peg.6051	fig|6666666.59345.peg.4326	fig|6666666.59341.peg.5231	fig|6666666.59347.peg.4381	fig|6666666.59346.peg.4400	fig|6666666.59348.peg.2261	fig|6666666.59349.peg.2462	fig|6666666.59350.peg.1335	fig|6666666.59351.peg.3722	fig|6666666.59353.peg.719	fig|6666666.59355.peg.2742	fig|6666666.59354.peg.994

2754	Phosphopantetheine adenylyltransferase EC 2.7.7.3	fig|6666666.59329.peg.553	fig|6666666.59330.peg.1162	fig|6666666.59331.peg.2858	fig|6666666.59332.peg.291	fig|6666666.59334.peg.180	fig|6666666.59335.peg.1998	fig|6666666.59336.peg.58	fig|6666666.59333.peg.650	fig|6666666.59338.peg.1912	fig|6666666.59339.peg.6295	fig|6666666.59340.peg.34	fig|6666666.59337.peg.468	None	fig|6666666.71181.peg.3657	fig|6666666.59344.peg.945	fig|6666666.59345.peg.2616	fig|6666666.59341.peg.5875	fig|6666666.59347.peg.5190	fig|6666666.59346.peg.1708	fig|6666666.59348.peg.3015	fig|6666666.59349.peg.1760	fig|6666666.59350.peg.2381	fig|6666666.59351.peg.2289	fig|6666666.59353.peg.2589	fig|6666666.59355.peg.2612	fig|6666666.59354.peg.490

2023	Beta N-acetyl-glucosaminidase EC 3.2.1.52	fig|6666666.59329.peg.2556	fig|6666666.59330.peg.2523	fig|6666666.59331.peg.5063	fig|6666666.59332.peg.5998	fig|6666666.59334.peg.3604	fig|6666666.59335.peg.3651	fig|6666666.59336.peg.3922	fig|6666666.59333.peg.3354	fig|6666666.59338.peg.2826	fig|6666666.59339.peg.7484	None	fig|6666666.59337.peg.300	fig|6666666.59342.peg.3389	fig|6666666.71181.peg.2672	fig|6666666.59344.peg.5017	fig|6666666.59345.peg.3697	fig|6666666.59341.peg.3198	fig|6666666.59347.peg.3371	fig|6666666.59346.peg.4616	fig|6666666.59348.peg.389	fig|6666666.59349.peg.3998	fig|6666666.59350.peg.3957	fig|6666666.59351.peg.3785	fig|6666666.59353.peg.334	fig|6666666.59355.peg.1373	fig|6666666.59354.peg.553

4687	Glutamate--cysteine ligase EC 6.3.2.2	fig|6666666.59329.peg.330	fig|6666666.59330.peg.2837	fig|6666666.59331.peg.2192	fig|6666666.59332.peg.1572	fig|6666666.59334.peg.257	fig|6666666.59335.peg.3984	fig|6666666.59336.peg.1358	fig|6666666.59333.peg.5322	fig|6666666.59338.peg.445	fig|6666666.59339.peg.8913	fig|6666666.59340.peg.3439	fig|6666666.59337.peg.2033	fig|6666666.59342.peg.3100	fig|6666666.71181.peg.3465	fig|6666666.59344.peg.2161	fig|6666666.59345.peg.2440	fig|6666666.59341.peg.2570	fig|6666666.59347.peg.3217	fig|6666666.59346.peg.561	fig|6666666.59348.peg.3599	fig|6666666.59349.peg.589	None	fig|6666666.59351.peg.525	fig|6666666.59353.peg.236	fig|6666666.59355.peg.3007	fig|6666666.59354.peg.4885

1784	Phosphoribosylamine--glycine ligase EC 6.3.4.13	fig|6666666.59329.peg.669	fig|6666666.59330.peg.3777	fig|6666666.59331.peg.3568	fig|6666666.59332.peg.129	fig|6666666.59334.peg.1582	fig|6666666.59335.peg.2635	fig|6666666.59336.peg.3383	fig|6666666.59333.peg.2194	fig|6666666.59338.peg.3374	fig|6666666.59339.peg.6430	None	fig|6666666.59337.peg.4504	fig|6666666.59342.peg.4594	fig|6666666.71181.peg.3349	fig|6666666.59344.peg.5677	fig|6666666.59345.peg.5695	fig|6666666.59341.peg.1426	fig|6666666.59347.peg.4485	fig|6666666.59346.peg.1144	fig|6666666.59348.peg.3309	fig|6666666.59349.peg.1946	fig|6666666.59350.peg.1762	fig|6666666.59351.peg.2354	fig|6666666.59353.peg.4473	fig|6666666.59355.peg.4553	fig|6666666.59354.peg.1

10074	Proton glutamate symport protein Sodium glutamate symport protein	fig|6666666.59329.peg.6567	fig|6666666.59330.peg.2668	fig|6666666.59331.peg.3927	fig|6666666.59332.peg.387	fig|6666666.59334.peg.3162	fig|6666666.59335.peg.2524	fig|6666666.59336.peg.2662	fig|6666666.59333.peg.1770	fig|6666666.59338.peg.5240	fig|6666666.59339.peg.1920	fig|6666666.59340.peg.2057	fig|6666666.59337.peg.4360	fig|6666666.59342.peg.857	fig|6666666.71181.peg.4388	fig|6666666.59344.peg.5654	fig|6666666.59345.peg.921	fig|6666666.59341.peg.553	fig|6666666.59347.peg.1604	fig|6666666.59346.peg.2472	fig|6666666.59348.peg.3821	fig|6666666.59349.peg.4460	None	fig|6666666.59351.peg.4701	fig|6666666.59353.peg.2393	fig|6666666.59355.peg.3303	fig|6666666.59354.peg.4879

9156	LSU ribosomal protein L23p L23Ae	fig|6666666.59329.peg.1103	fig|6666666.59330.peg.552	fig|6666666.59331.peg.3025	fig|6666666.59332.peg.3797	fig|6666666.59334.peg.1483	fig|6666666.59335.peg.1849	fig|6666666.59336.peg.143	fig|6666666.59333.peg.2691	fig|6666666.59338.peg.1391	fig|6666666.59339.peg.8266	fig|6666666.59340.peg.1729	fig|6666666.59337.peg.390	fig|6666666.59342.peg.302	fig|6666666.71181.peg.3458	fig|6666666.59344.peg.4941	fig|6666666.59345.peg.2538	fig|6666666.59341.peg.2496	fig|6666666.59347.peg.4821	fig|6666666.59346.peg.848	fig|6666666.59348.peg.412	fig|6666666.59349.peg.1096	fig|6666666.59350.peg.4265	fig|6666666.59351.peg.4816	fig|6666666.59353.peg.4592	fig|6666666.59355.peg.4739	None

10753	Tryptophanyl-tRNA synthetase EC 6.1.1.2	fig|6666666.59329.peg.19	fig|6666666.59330.peg.1442	fig|6666666.59331.peg.5124	fig|6666666.59332.peg.3498	fig|6666666.59334.peg.729	fig|6666666.59335.peg.2478	fig|6666666.59336.peg.273	fig|6666666.59333.peg.302	fig|6666666.59338.peg.3147	fig|6666666.59339.peg.8706	fig|6666666.59340.peg.67	fig|6666666.59337.peg.1795	None	fig|6666666.71181.peg.1826	fig|6666666.59344.peg.3072	fig|6666666.59345.peg.5577	fig|6666666.59341.peg.4073	fig|6666666.59347.peg.4950	fig|6666666.59346.peg.13	fig|6666666.59348.peg.4012	fig|6666666.59349.peg.536	fig|6666666.59350.peg.7690	fig|6666666.59351.peg.3860	fig|6666666.59353.peg.2224	fig|6666666.59355.peg.1918	fig|6666666.59354.peg.3565

6618	Peptidyl-tRNA hydrolase EC 3.1.1.29	None	fig|6666666.59330.peg.4670	fig|6666666.59331.peg.1442	fig|6666666.59332.peg.4260	fig|6666666.59334.peg.2237	fig|6666666.59335.peg.1173	fig|6666666.59336.peg.5140	fig|6666666.59333.peg.5156	fig|6666666.59338.peg.4131	fig|6666666.59339.peg.2	fig|6666666.59340.peg.2787	fig|6666666.59337.peg.5162	fig|6666666.59342.peg.470	fig|6666666.71181.peg.785	fig|6666666.59344.peg.102	fig|6666666.59345.peg.5411	fig|6666666.59341.peg.5834	fig|6666666.59347.peg.5284	fig|6666666.59346.peg.3186	fig|6666666.59348.peg.614	fig|6666666.59349.peg.2609	fig|6666666.59350.peg.2697	fig|6666666.59351.peg.4668	fig|6666666.59353.peg.426	fig|6666666.59355.peg.2152	fig|6666666.59354.peg.4335

3506	LSU ribosomal protein L15p L27Ae	fig|6666666.59329.peg.728	fig|6666666.59330.peg.569	fig|6666666.59331.peg.33	fig|6666666.59332.peg.2154	fig|6666666.59334.peg.4021	fig|6666666.59335.peg.4362	fig|6666666.59336.peg.161	fig|6666666.59333.peg.381	fig|6666666.59338.peg.4967	fig|6666666.59339.peg.8282	fig|6666666.59340.peg.3480	fig|6666666.59337.peg.373	fig|6666666.59342.peg.319	fig|6666666.71181.peg.3444	fig|6666666.59344.peg.4927	fig|6666666.59345.peg.2520	fig|6666666.59341.peg.2513	fig|6666666.59347.peg.4838	fig|6666666.59346.peg.865	fig|6666666.59348.peg.439	fig|6666666.59349.peg.3285	None	fig|6666666.59351.peg.4219	fig|6666666.59353.peg.4606	fig|6666666.59355.peg.4725	fig|6666666.59354.peg.3253

2343	YciO family	fig|6666666.59329.peg.6312	fig|6666666.59330.peg.653	None	fig|6666666.59332.peg.5853	fig|6666666.59334.peg.4066	fig|6666666.59335.peg.3716	fig|6666666.59336.peg.1984	fig|6666666.59333.peg.4237	fig|6666666.59338.peg.3475	fig|6666666.59339.peg.3643	fig|6666666.59340.peg.876	fig|6666666.59337.peg.3909	fig|6666666.59342.peg.4876	fig|6666666.71181.peg.3426	fig|6666666.59344.peg.465	fig|6666666.59345.peg.912	fig|6666666.59341.peg.5017	fig|6666666.59347.peg.3551	fig|6666666.59346.peg.1608	fig|6666666.59348.peg.2724	fig|6666666.59349.peg.361	fig|6666666.59350.peg.1254	fig|6666666.59351.peg.793	fig|6666666.59353.peg.4626	fig|6666666.59355.peg.1230	fig|6666666.59354.peg.4183

2067	tRNA-guanine transglycosylase EC 2.4.2.29	fig|6666666.59329.peg.647	fig|6666666.59330.peg.1405	fig|6666666.59331.peg.2297	fig|6666666.59332.peg.1440	fig|6666666.59334.peg.2715	fig|6666666.59335.peg.5093	fig|6666666.59336.peg.5540	fig|6666666.59333.peg.4135	fig|6666666.59338.peg.3405	fig|6666666.59339.peg.6895	fig|6666666.59340.peg.552	fig|6666666.59337.peg.731	fig|6666666.59342.peg.1145	fig|6666666.71181.peg.2734	fig|6666666.59344.peg.1106	fig|6666666.59345.peg.3659	fig|6666666.59341.peg.5336	fig|6666666.59347.peg.4276	fig|6666666.59346.peg.2747	fig|6666666.59348.peg.1563	fig|6666666.59349.peg.1925	fig|6666666.59350.peg.7828	fig|6666666.59351.peg.2333	fig|6666666.59353.peg.786	fig|6666666.59355.peg.919	None

3700	Ubiquinone biosynthesis monooxygenase UbiB	fig|6666666.59329.peg.5191	fig|6666666.59330.peg.252	None	fig|6666666.59332.peg.7	fig|6666666.59334.peg.2865	fig|6666666.59335.peg.5137	fig|6666666.59336.peg.893	fig|6666666.59333.peg.73	fig|6666666.59338.peg.3944	fig|6666666.59339.peg.4174	fig|6666666.59340.peg.2541	fig|6666666.59337.peg.3269	fig|6666666.59342.peg.4559	fig|6666666.71181.peg.1898	fig|6666666.59344.peg.2545	fig|6666666.59345.peg.533	fig|6666666.59341.peg.153	fig|6666666.59347.peg.4636	fig|6666666.59346.peg.1866	fig|6666666.59348.peg.114	fig|6666666.59349.peg.3259	fig|6666666.59350.peg.7173	fig|6666666.59351.peg.4088	fig|6666666.59353.peg.3799	fig|6666666.59355.peg.3395	fig|6666666.59354.peg.2223

5177	HflC protein	fig|6666666.59329.peg.5158	fig|6666666.59330.peg.3563	None	fig|6666666.59332.peg.4224	fig|6666666.59334.peg.3058	fig|6666666.59335.peg.4134	fig|6666666.59336.peg.3055	fig|6666666.59333.peg.290	fig|6666666.59338.peg.1005	fig|6666666.59339.peg.8864	fig|6666666.59340.peg.1202	fig|6666666.59337.peg.1786	fig|6666666.59342.peg.2595	fig|6666666.71181.peg.4631	fig|6666666.59344.peg.5888	fig|6666666.59345.peg.5660	fig|6666666.59341.peg.4275	fig|6666666.59347.peg.5344	fig|6666666.59346.peg.3036	fig|6666666.59348.peg.3300	fig|6666666.59349.peg.3174	fig|6666666.59350.peg.3867	fig|6666666.59351.peg.3097	fig|6666666.59353.peg.4987	fig|6666666.59355.peg.5071	fig|6666666.59354.peg.5847

5339	Adenylate kinase EC 2.7.4.3	fig|6666666.59329.peg.1940	fig|6666666.59330.peg.5051	fig|6666666.59331.peg.3036	fig|6666666.59332.peg.1703	fig|6666666.59334.peg.209	fig|6666666.59335.peg.2418	fig|6666666.59336.peg.2547	fig|6666666.59333.peg.2066	fig|6666666.59338.peg.4381	fig|6666666.59339.peg.5268	None	fig|6666666.59337.peg.3920	fig|6666666.59342.peg.1793	fig|6666666.71181.peg.3496	fig|6666666.59344.peg.5514	fig|6666666.59345.peg.4348	fig|6666666.59341.peg.511	fig|6666666.59347.peg.2832	fig|6666666.59346.peg.3671	fig|6666666.59348.peg.2493	fig|6666666.59349.peg.2675	fig|6666666.59350.peg.2042	fig|6666666.59351.peg.5167	fig|6666666.59353.peg.4072	fig|6666666.59355.peg.4149	fig|6666666.59354.peg.5408

83	Ribonuclease HI EC 3.1.26.4	fig|6666666.59329.peg.411	fig|6666666.59330.peg.4725	fig|6666666.59331.peg.5746	fig|6666666.59332.peg.432	fig|6666666.59334.peg.3923	fig|6666666.59335.peg.5074	fig|6666666.59336.peg.2854	fig|6666666.59333.peg.2581	fig|6666666.59338.peg.3631	fig|6666666.59339.peg.5147	fig|6666666.59340.peg.636	fig|6666666.59337.peg.1608	None	fig|6666666.71181.peg.878	fig|6666666.59344.peg.4753	fig|6666666.59345.peg.2175	fig|6666666.59341.peg.2351	fig|6666666.59347.peg.2868	fig|6666666.59346.peg.3695	fig|6666666.59348.peg.4992	fig|6666666.59349.peg.4585	fig|6666666.59350.peg.1370	fig|6666666.59351.peg.5493	fig|6666666.59353.peg.4555	fig|6666666.59355.peg.4642	fig|6666666.59354.peg.3310

8809	Exodeoxyribonuclease VII large subunit EC 3.1.11.6	fig|6666666.59329.peg.297	fig|6666666.59330.peg.4373	fig|6666666.59331.peg.1775	fig|6666666.59332.peg.3043	fig|6666666.59334.peg.1262	fig|6666666.59335.peg.1961	fig|6666666.59336.peg.1633	fig|6666666.59333.peg.2151	fig|6666666.59338.peg.3375	fig|6666666.59339.peg.6981	fig|6666666.59340.peg.36	fig|6666666.59337.peg.760	fig|6666666.59342.peg.5783	fig|6666666.71181.peg.1782	fig|6666666.59344.peg.5151	fig|6666666.59345.peg.3632	fig|6666666.59341.peg.5883	fig|6666666.59347.peg.4303	fig|6666666.59346.peg.3217	None	fig|6666666.59349.peg.1683	fig|6666666.59350.peg.1763	fig|6666666.59351.peg.383	fig|6666666.59353.peg.2721	fig|6666666.59355.peg.1759	fig|6666666.59354.peg.3485

6580	Single-stranded DNA-binding protein	fig|6666666.59329.peg.4359	fig|6666666.59330.peg.5	fig|6666666.59331.peg.2337	fig|6666666.59332.peg.1302	fig|6666666.59334.peg.3548	fig|6666666.59335.peg.198	fig|6666666.59336.peg.4187	fig|6666666.59333.peg.582	fig|6666666.59338.peg.4639	fig|6666666.59339.peg.7755	fig|6666666.59340.peg.1861	fig|6666666.59337.peg.791	None	fig|6666666.71181.peg.4000	fig|6666666.59344.peg.699	fig|6666666.59345.peg.4905	fig|6666666.59341.peg.3144	fig|6666666.59347.peg.2007	fig|6666666.59346.peg.1598	fig|6666666.59348.peg.3072	fig|6666666.59349.peg.4450	fig|6666666.59350.peg.2168	fig|6666666.59351.peg.3414	fig|6666666.59353.peg.2979	fig|6666666.59355.peg.2401	fig|6666666.59354.peg.6194

11158	Uracil phosphoribosyltransferase EC 2.4.2.9	fig|6666666.59329.peg.6263	fig|6666666.59330.peg.4181	fig|6666666.59331.peg.5485	fig|6666666.59332.peg.2790	fig|6666666.59334.peg.384	fig|6666666.59335.peg.1709	fig|6666666.59336.peg.1603	fig|6666666.59333.peg.3285	fig|6666666.59338.peg.4858	fig|6666666.59339.peg.7240	None	fig|6666666.59337.peg.4487	fig|6666666.59342.peg.4570	fig|6666666.71181.peg.3273	fig|6666666.59344.peg.4029	fig|6666666.59345.peg.2166	fig|6666666.59341.peg.2362	fig|6666666.59347.peg.2860	fig|6666666.59346.peg.2493	fig|6666666.59348.peg.3532	fig|6666666.59349.peg.389	fig|6666666.59350.peg.2605	fig|6666666.59351.peg.3513	fig|6666666.59353.peg.4022	fig|6666666.59355.peg.3980	fig|6666666.59354.peg.5821

9098	Phosphate transport system regulatory protein PhoU	fig|6666666.59329.peg.1119	fig|6666666.59330.peg.4243	fig|6666666.59331.peg.1187	fig|6666666.59332.peg.761	fig|6666666.59334.peg.4578	fig|6666666.59335.peg.2575	fig|6666666.59336.peg.1649	fig|6666666.59333.peg.354	fig|6666666.59338.peg.1812	fig|6666666.59339.peg.6929	fig|6666666.59340.peg.2014	fig|6666666.59337.peg.715	None	fig|6666666.71181.peg.607	fig|6666666.59344.peg.4283	fig|6666666.59345.peg.3675	fig|6666666.59341.peg.3362	fig|6666666.59347.peg.4267	fig|6666666.59346.peg.4429	fig|6666666.59348.peg.4573	fig|6666666.59349.peg.3552	fig|6666666.59350.peg.610	fig|6666666.59351.peg.2324	fig|6666666.59353.peg.3072	fig|6666666.59355.peg.2275	fig|6666666.59354.peg.1276

8563	ATP synthase B chain EC 3.6.3.14	fig|6666666.59329.peg.3994	fig|6666666.59330.peg.607	fig|6666666.59331.peg.402	fig|6666666.59332.peg.101	fig|6666666.59334.peg.24	fig|6666666.59335.peg.794	fig|6666666.59336.peg.30	fig|6666666.59333.peg.27	fig|6666666.59338.peg.495	fig|6666666.59339.peg.2290	fig|6666666.59340.peg.2446	fig|6666666.59337.peg.892	fig|6666666.59342.peg.2036	fig|6666666.71181.peg.594	fig|6666666.59344.peg.4267	fig|6666666.59345.peg.4963	fig|6666666.59341.peg.1061	fig|6666666.59347.peg.2035	fig|6666666.59346.peg.1405	fig|6666666.59348.peg.334	fig|6666666.59349.peg.1032	None	fig|6666666.59351.peg.5357	fig|6666666.59353.peg.3086	fig|6666666.59355.peg.2262	fig|6666666.59354.peg.1154

8561	3-isopropylmalate dehydrogenase EC 1.1.1.85	fig|6666666.59329.peg.1699	fig|6666666.59330.peg.809	fig|6666666.59331.peg.5345	fig|6666666.59332.peg.4758	fig|6666666.59334.peg.344	fig|6666666.59335.peg.5240	fig|6666666.59336.peg.2916	fig|6666666.59333.peg.123	fig|6666666.59338.peg.57	fig|6666666.59339.peg.7324	fig|6666666.59340.peg.361	fig|6666666.59337.peg.1392	fig|6666666.59342.peg.5915	fig|6666666.71181.peg.1242	fig|6666666.59344.peg.1500	fig|6666666.59345.peg.3888	fig|6666666.59341.peg.4408	fig|6666666.59347.peg.3079	fig|6666666.59346.peg.815	None	fig|6666666.59349.peg.917	fig|6666666.59350.peg.2988	fig|6666666.59351.peg.1610	fig|6666666.59353.peg.120	fig|6666666.59355.peg.2202	fig|6666666.59354.peg.2174

4704	Outer membrane protein assembly factor YaeT precursor	fig|6666666.59329.peg.1581	fig|6666666.59330.peg.460	None	fig|6666666.59332.peg.1582	fig|6666666.59334.peg.5457	fig|6666666.59335.peg.2660	fig|6666666.59336.peg.2566	fig|6666666.59333.peg.4244	fig|6666666.59338.peg.2453	fig|6666666.59339.peg.5120	fig|6666666.59340.peg.1318	fig|6666666.59337.peg.1590	fig|6666666.59342.peg.276	fig|6666666.71181.peg.27	fig|6666666.59344.peg.2662	fig|6666666.59345.peg.2193	fig|6666666.59341.peg.976	fig|6666666.59347.peg.4366	fig|6666666.59346.peg.2268	fig|6666666.59348.peg.4081	fig|6666666.59349.peg.1367	fig|6666666.59350.peg.4427	fig|6666666.59351.peg.2123	fig|6666666.59353.peg.1993	fig|6666666.59355.peg.4098	fig|6666666.59354.peg.1341

8820	Uncharacterized ABC transporter, permease component YrbE	fig|6666666.59329.peg.5289	fig|6666666.59330.peg.52	None	fig|6666666.59332.peg.1275	fig|6666666.59334.peg.91	fig|6666666.59335.peg.3041	fig|6666666.59336.peg.356	fig|6666666.59333.peg.271	fig|6666666.59338.peg.115	fig|6666666.59339.peg.8193	fig|6666666.59340.peg.261	fig|6666666.59337.peg.839	fig|6666666.59342.peg.443	fig|6666666.71181.peg.1025	fig|6666666.59344.peg.1805	fig|6666666.59345.peg.4854	fig|6666666.59341.peg.1254	fig|6666666.59347.peg.3245	fig|6666666.59346.peg.632	fig|6666666.59348.peg.3526	fig|6666666.59349.peg.28	fig|6666666.59350.peg.8115	fig|6666666.59351.peg.3449	fig|6666666.59353.peg.2344	fig|6666666.59355.peg.1617	fig|6666666.59354.peg.343

10913	SSU ribosomal protein S16p	fig|6666666.59329.peg.336	fig|6666666.59330.peg.2844	fig|6666666.59331.peg.1985	fig|6666666.59332.peg.315	fig|6666666.59334.peg.266	fig|6666666.59335.peg.5391	fig|6666666.59336.peg.3006	fig|6666666.59333.peg.161	fig|6666666.59338.peg.2606	fig|6666666.59339.peg.7933	None	fig|6666666.59337.peg.2041	fig|6666666.59342.peg.2097	fig|6666666.71181.peg.3957	fig|6666666.59344.peg.5443	fig|6666666.59345.peg.2433	fig|6666666.59341.peg.1342	fig|6666666.59347.peg.3884	fig|6666666.59346.peg.569	fig|6666666.59348.peg.1259	fig|6666666.59349.peg.603	fig|6666666.59350.peg.2768	fig|6666666.59351.peg.540	fig|6666666.59353.peg.4814	fig|6666666.59355.peg.4868	fig|6666666.59354.peg.3773

5988	Chemotaxis protein methyltransferase CheR EC 2.1.1.80	fig|6666666.59329.peg.575	fig|6666666.59330.peg.2690	fig|6666666.59331.peg.5077	fig|6666666.59332.peg.1796	None	fig|6666666.59335.peg.2080	fig|6666666.59336.peg.1965	fig|6666666.59333.peg.2834	fig|6666666.59338.peg.629	fig|6666666.59339.peg.5310	None	fig|6666666.59337.peg.3346	fig|6666666.59342.peg.1501	fig|6666666.71181.peg.2245	fig|6666666.59344.peg.3018	fig|6666666.59345.peg.5448	fig|6666666.59341.peg.4567	fig|6666666.59347.peg.2715	fig|6666666.59346.peg.1761	fig|6666666.59348.peg.3955	fig|6666666.59349.peg.3493	fig|6666666.59350.peg.4535	fig|6666666.59351.peg.2560	fig|6666666.59353.peg.3542	fig|6666666.59355.peg.3633	fig|6666666.59354.peg.1522

9252	Flagellar biosynthesis protein FliS	fig|6666666.59329.peg.4704	fig|6666666.59330.peg.2906	fig|6666666.59331.peg.3208	fig|6666666.59332.peg.2460	None	fig|6666666.59335.peg.918	fig|6666666.59336.peg.1441	fig|6666666.59333.peg.4083	fig|6666666.59338.peg.2383	fig|6666666.59339.peg.7109	fig|6666666.59340.peg.3155	fig|6666666.59337.peg.3215	fig|6666666.59342.peg.5250	fig|6666666.71181.peg.4034	fig|6666666.59344.peg.5212	fig|6666666.59345.peg.5352	fig|6666666.59341.peg.125	fig|6666666.59347.peg.4614	fig|6666666.59346.peg.4913	fig|6666666.59348.peg.2819	fig|6666666.59349.peg.742	None	fig|6666666.59351.peg.1999	fig|6666666.59353.peg.4569	fig|6666666.59355.peg.3833	fig|6666666.59354.peg.4131

4845	UPF0125 protein yfjF	fig|6666666.59329.peg.3958	fig|6666666.59330.peg.2429	fig|6666666.59331.peg.1332	fig|6666666.59332.peg.379	None	fig|6666666.59335.peg.4162	fig|6666666.59336.peg.218	fig|6666666.59333.peg.2762	fig|6666666.59338.peg.1075	fig|6666666.59339.peg.7280	fig|6666666.59340.peg.2210	fig|6666666.59337.peg.5542	fig|6666666.59342.peg.5026	fig|6666666.71181.peg.1443	fig|6666666.59344.peg.1542	fig|6666666.59345.peg.3579	fig|6666666.59341.peg.5536	fig|6666666.59347.peg.4325	fig|6666666.59346.peg.694	fig|6666666.59348.peg.1841	fig|6666666.59349.peg.2634	None	fig|6666666.59351.peg.4918	fig|6666666.59353.peg.4358	fig|6666666.59355.peg.4485	fig|6666666.59354.peg.6254

393	Heat shock protein 60 family co-chaperone GroES	fig|6666666.59329.peg.3147	fig|6666666.59330.peg.163	fig|6666666.59331.peg.4452	fig|6666666.59332.peg.2921	fig|6666666.59334.peg.3145	fig|6666666.59335.peg.419	fig|6666666.59336.peg.250	fig|6666666.59333.peg.3602	fig|6666666.59338.peg.3751	fig|6666666.59339.peg.6366	None	fig|6666666.59337.peg.4333	fig|6666666.59342.peg.5002	fig|6666666.71181.peg.1677	None	fig|6666666.59345.peg.5115	fig|6666666.59341.peg.179	fig|6666666.59347.peg.4549	fig|6666666.59346.peg.986	fig|6666666.59348.peg.2313	fig|6666666.59349.peg.2367	fig|6666666.59350.peg.2067	fig|6666666.59351.peg.112	fig|6666666.59353.peg.1047	fig|6666666.59355.peg.3572	fig|6666666.59354.peg.3257

10396	Ferredoxin, 2Fe-2S	fig|6666666.59329.peg.1106	fig|6666666.59330.peg.1419	None	fig|6666666.59332.peg.1334	fig|6666666.59334.peg.3070	fig|6666666.59335.peg.2373	fig|6666666.59336.peg.673	fig|6666666.59333.peg.930	fig|6666666.59338.peg.5773	fig|6666666.59339.peg.6798	fig|6666666.59340.peg.3043	fig|6666666.59337.peg.744	fig|6666666.59342.peg.3302	fig|6666666.71181.peg.1762	fig|6666666.59344.peg.1634	fig|6666666.59345.peg.3647	fig|6666666.59341.peg.6095	fig|6666666.59347.peg.4288	fig|6666666.59346.peg.5153	None	fig|6666666.59349.peg.1386	fig|6666666.59350.peg.4872	fig|6666666.59351.peg.2708	fig|6666666.59353.peg.2700	fig|6666666.59355.peg.1779	fig|6666666.59354.peg.5182

7217	Ferric uptake regulation protein FUR	fig|6666666.59329.peg.1357	fig|6666666.59330.peg.2998	fig|6666666.59331.peg.2300	fig|6666666.59332.peg.3229	fig|6666666.59334.peg.223	fig|6666666.59335.peg.5791	fig|6666666.59336.peg.330	fig|6666666.59333.peg.862	fig|6666666.59338.peg.1150	fig|6666666.59339.peg.7209	None	fig|6666666.59337.peg.3932	fig|6666666.59342.peg.2214	fig|6666666.71181.peg.6318	fig|6666666.59344.peg.5089	fig|6666666.59345.peg.4335	fig|6666666.59341.peg.5924	fig|6666666.59347.peg.2944	fig|6666666.59346.peg.2005	fig|6666666.59348.peg.5168	fig|6666666.59349.peg.1817	fig|6666666.59350.peg.2964	fig|6666666.59351.peg.1116	fig|6666666.59353.peg.2056	fig|6666666.59355.peg.2771	None

9757	Ribosomal protein L11 methyltransferase EC 2.1.1.-	fig|6666666.59329.peg.3753	fig|6666666.59330.peg.195	None	fig|6666666.59332.peg.122	fig|6666666.59334.peg.49	fig|6666666.59335.peg.2628	fig|6666666.59336.peg.2271	fig|6666666.59333.peg.2643	fig|6666666.59338.peg.3732	fig|6666666.59339.peg.6439	None	fig|6666666.59337.peg.4287	fig|6666666.59342.peg.2607	fig|6666666.71181.peg.968	fig|6666666.59344.peg.5343	fig|6666666.59345.peg.5147	fig|6666666.59341.peg.1181	fig|6666666.59347.peg.4493	fig|6666666.59346.peg.1151	fig|6666666.59348.peg.944	fig|6666666.59349.peg.1953	fig|6666666.59350.peg.4759	fig|6666666.59351.peg.2362	fig|6666666.59353.peg.2282	fig|6666666.59355.peg.4822	fig|6666666.59354.peg.8

7663	UDP-3-O-[3-hydroxymyristoyl] N-acetylglucosamine deacetylase EC 3.5.1.-	fig|6666666.59329.peg.1364	fig|6666666.59330.peg.1283	fig|6666666.59331.peg.2460	fig|6666666.59332.peg.3772	fig|6666666.59334.peg.3096	fig|6666666.59335.peg.825	fig|6666666.59336.peg.957	fig|6666666.59333.peg.4202	fig|6666666.59338.peg.5664	fig|6666666.59339.peg.4980	None	fig|6666666.59337.peg.54	None	fig|6666666.71181.peg.1974	fig|6666666.59344.peg.1473	fig|6666666.59345.peg.3787	fig|6666666.59341.peg.3128	fig|6666666.59347.peg.4006	fig|6666666.59346.peg.1247	fig|6666666.59348.peg.256	fig|6666666.59349.peg.2991	fig|6666666.59350.peg.1048	fig|6666666.59351.peg.216	fig|6666666.59353.peg.144	fig|6666666.59355.peg.754	fig|6666666.59354.peg.4406

5864	Ribosomal RNA small subunit methyltransferase E EC 2.1.1.-	fig|6666666.59329.peg.1774	fig|6666666.59330.peg.348	None	fig|6666666.59332.peg.1938	fig|6666666.59334.peg.5171	fig|6666666.59335.peg.3096	None	fig|6666666.59333.peg.775	fig|6666666.59338.peg.450	fig|6666666.59339.peg.7837	fig|6666666.59340.peg.207	fig|6666666.59337.peg.529	fig|6666666.59342.peg.5969	fig|6666666.71181.peg.1860	fig|6666666.59344.peg.4334	fig|6666666.59345.peg.5019	fig|6666666.59341.peg.2577	fig|6666666.59347.peg.3212	fig|6666666.59346.peg.3519	fig|6666666.59348.peg.357	fig|6666666.59349.peg.808	fig|6666666.59350.peg.508	fig|6666666.59351.peg.3537	fig|6666666.59353.peg.3225	fig|6666666.59355.peg.3107	fig|6666666.59354.peg.650

9770	NfuA Fe-S protein maturation	fig|6666666.59329.peg.1638	fig|6666666.59330.peg.430	None	fig|6666666.59332.peg.2470	fig|6666666.59334.peg.2877	fig|6666666.59335.peg.696	fig|6666666.59336.peg.4433	fig|6666666.59333.peg.519	fig|6666666.59338.peg.1425	fig|6666666.59339.peg.6235	fig|6666666.59340.peg.2258	fig|6666666.59337.peg.4258	fig|6666666.59342.peg.3594	fig|6666666.71181.peg.4486	fig|6666666.59344.peg.3636	fig|6666666.59345.peg.2664	fig|6666666.59341.peg.1413	fig|6666666.59347.peg.4476	fig|6666666.59346.peg.2234	fig|6666666.59348.peg.1406	fig|6666666.59349.peg.2196	None	fig|6666666.59351.peg.4368	fig|6666666.59353.peg.2186	fig|6666666.59355.peg.1880	fig|6666666.59354.peg.1792

9773	Inosine-guanosine kinase EC 2.7.1.73	fig|6666666.59329.peg.2585	fig|6666666.59330.peg.2867	None	fig|6666666.59332.peg.4973	fig|6666666.59334.peg.3212	fig|6666666.59335.peg.597	fig|6666666.59336.peg.3104	fig|6666666.59333.peg.5369	fig|6666666.59338.peg.2041	fig|6666666.59339.peg.8458	fig|6666666.59340.peg.1399	fig|6666666.59337.peg.2500	fig|6666666.59342.peg.238	fig|6666666.71181.peg.3490	fig|6666666.59344.peg.5042	fig|6666666.59345.peg.1311	fig|6666666.59341.peg.5813	fig|6666666.59347.peg.4134	fig|6666666.59346.peg.1559	None	fig|6666666.59349.peg.2803	fig|6666666.59350.peg.2	fig|6666666.59351.peg.3564	fig|6666666.59353.peg.4067	fig|6666666.59355.peg.4143	fig|6666666.59354.peg.4904

8864	Protein YcgL	fig|6666666.59329.peg.4744	fig|6666666.59330.peg.4581	None	fig|6666666.59332.peg.1213	fig|6666666.59334.peg.4988	fig|6666666.59335.peg.2759	fig|6666666.59336.peg.1021	fig|6666666.59333.peg.1359	fig|6666666.59338.peg.1138	fig|6666666.59339.peg.243	fig|6666666.59340.peg.3591	fig|6666666.59337.peg.2753	fig|6666666.59342.peg.5016	fig|6666666.71181.peg.2943	fig|6666666.59344.peg.2859	fig|6666666.59345.peg.4293	fig|6666666.59341.peg.3860	fig|6666666.59347.peg.4416	fig|6666666.59346.peg.1472	fig|6666666.59348.peg.1742	fig|6666666.59349.peg.477	None	fig|6666666.59351.peg.1647	fig|6666666.59353.peg.1461	fig|6666666.59355.peg.2012	fig|6666666.59354.peg.5730

8863	Predicted transcriptional regulator	fig|6666666.59329.peg.5825	fig|6666666.59330.peg.3585	None	fig|6666666.59332.peg.3784	fig|6666666.59334.peg.5399	fig|6666666.59335.peg.5917	fig|6666666.59336.peg.5715	fig|6666666.59333.peg.4574	fig|6666666.59338.peg.1683	fig|6666666.59339.peg.2098	fig|6666666.59340.peg.1636	fig|6666666.59337.peg.4827	fig|6666666.59342.peg.2896	fig|6666666.71181.peg.3261	fig|6666666.59344.peg.3471	fig|6666666.59345.peg.172	fig|6666666.59341.peg.437	fig|6666666.59347.peg.375	fig|6666666.59346.peg.3491	fig|6666666.59348.peg.3347	fig|6666666.59349.peg.2142	fig|6666666.59350.peg.54	None	fig|6666666.59353.peg.4035	fig|6666666.59355.peg.2224	fig|6666666.59354.peg.5955

9566	Heat shock predicted periplasmic protein YciM, precursor	fig|6666666.59329.peg.2772	fig|6666666.59330.peg.1384	None	fig|6666666.59332.peg.2853	fig|6666666.59334.peg.1073	fig|6666666.59335.peg.3372	fig|6666666.59336.peg.427	fig|6666666.59333.peg.739	fig|6666666.59338.peg.5927	fig|6666666.59339.peg.3114	fig|6666666.59340.peg.614	fig|6666666.59337.peg.2880	fig|6666666.59342.peg.1800	fig|6666666.71181.peg.4196	fig|6666666.59344.peg.488	fig|6666666.59345.peg.1033	fig|6666666.59341.peg.50	fig|6666666.59347.peg.2404	fig|6666666.59346.peg.5064	fig|6666666.59348.peg.834	fig|6666666.59349.peg.374	None	fig|6666666.59351.peg.945	fig|6666666.59353.peg.1805	fig|6666666.59355.peg.1211	fig|6666666.59354.peg.4427

6253	Conserved protein YcjX with nucleoside triphosphate hydrolase domain	fig|6666666.59329.peg.3382	fig|6666666.59330.peg.2084	fig|6666666.59331.peg.1237	fig|6666666.59332.peg.5658	fig|6666666.59334.peg.2326	fig|6666666.59335.peg.5170	fig|6666666.59336.peg.6279	fig|6666666.59333.peg.3929	fig|6666666.59338.peg.5534	fig|6666666.59339.peg.3718	None	fig|6666666.59337.peg.4682	fig|6666666.59342.peg.3829	fig|6666666.71181.peg.1385	fig|6666666.59344.peg.536	fig|6666666.59345.peg.822	fig|6666666.59341.peg.5970	fig|6666666.59347.peg.2142	fig|6666666.59346.peg.3848	fig|6666666.59348.peg.5130	fig|6666666.59349.peg.2085	fig|6666666.59350.peg.1075	fig|6666666.59351.peg.2598	fig|6666666.59353.peg.1843	fig|6666666.59355.peg.1171	None

9001	LSU ribosomal protein L20p	fig|6666666.59329.peg.4067	fig|6666666.59330.peg.517	fig|6666666.59331.peg.112	fig|6666666.59332.peg.4772	fig|6666666.59334.peg.4312	fig|6666666.59335.peg.3307	fig|6666666.59336.peg.5691	fig|6666666.59333.peg.524	fig|6666666.59338.peg.5745	fig|6666666.59339.peg.2415	None	fig|6666666.59337.peg.3296	fig|6666666.59342.peg.2817	fig|6666666.71181.peg.2417	None	fig|6666666.59345.peg.4637	fig|6666666.59341.peg.3122	fig|6666666.59347.peg.3274	fig|6666666.59346.peg.4578	fig|6666666.59348.peg.1962	fig|6666666.59349.peg.405	fig|6666666.59350.peg.4679	fig|6666666.59351.peg.4791	fig|6666666.59353.peg.1304	fig|6666666.59355.peg.3277	fig|6666666.59354.peg.2975

7937	Flagellar biosynthesis protein FlhB	fig|6666666.59329.peg.199	fig|6666666.59330.peg.4264	fig|6666666.59331.peg.3272	fig|6666666.59332.peg.5895	None	fig|6666666.59335.peg.924	fig|6666666.59336.peg.5813	fig|6666666.59333.peg.1680	fig|6666666.59338.peg.1764	fig|6666666.59339.peg.7080	fig|6666666.59340.peg.2090	fig|6666666.59337.peg.2972	fig|6666666.59342.peg.2077	fig|6666666.71181.peg.2239	fig|6666666.59344.peg.3024	fig|6666666.59345.peg.2869	fig|6666666.59341.peg.3280	fig|6666666.59347.peg.2678	fig|6666666.59346.peg.2893	None	fig|6666666.59349.peg.198	fig|6666666.59350.peg.4399	fig|6666666.59351.peg.2059	fig|6666666.59353.peg.3536	fig|6666666.59355.peg.3639	fig|6666666.59354.peg.680

4785	DedD protein	fig|6666666.59329.peg.3491	None	fig|6666666.59331.peg.1905	fig|6666666.59332.peg.2751	fig|6666666.59334.peg.4400	fig|6666666.59335.peg.3592	fig|6666666.59336.peg.3037	fig|6666666.59333.peg.2542	fig|6666666.59338.peg.2035	fig|6666666.59339.peg.2974	fig|6666666.59340.peg.390	fig|6666666.59337.peg.3729	fig|6666666.59342.peg.4064	fig|6666666.71181.peg.1475	fig|6666666.59344.peg.1886	fig|6666666.59345.peg.1202	fig|6666666.59341.peg.2785	fig|6666666.59347.peg.2454	fig|6666666.59346.peg.55	fig|6666666.59348.peg.1716	fig|6666666.59349.peg.3461	None	fig|6666666.59351.peg.3573	fig|6666666.59353.peg.1207	fig|6666666.59355.peg.269	fig|6666666.59354.peg.2962

5824	Cytidine deaminase EC 3.5.4.5	fig|6666666.59329.peg.5553	fig|6666666.59330.peg.2674	fig|6666666.59331.peg.4258	fig|6666666.59332.peg.5251	None	fig|6666666.59335.peg.6001	fig|6666666.59336.peg.1687	fig|6666666.59333.peg.541	fig|6666666.59338.peg.5496	fig|6666666.59339.peg.3891	None	fig|6666666.59337.peg.4528	fig|6666666.59342.peg.391	fig|6666666.71181.peg.199	fig|6666666.59344.peg.243	fig|6666666.59345.peg.4664	fig|6666666.59341.peg.1274	fig|6666666.59347.peg.1636	fig|6666666.59346.peg.4401	fig|6666666.59348.peg.3964	fig|6666666.59349.peg.4079	fig|6666666.59350.peg.2602	fig|6666666.59351.peg.3764	fig|6666666.59353.peg.623	fig|6666666.59355.peg.106	fig|6666666.59354.peg.455

3456	Succinate dehydrogenase cytochrome b-556 subunit	fig|6666666.59329.peg.163	fig|6666666.59330.peg.2769	fig|6666666.59331.peg.70	fig|6666666.59332.peg.4294	fig|6666666.59334.peg.3835	fig|6666666.59335.peg.2740	fig|6666666.59336.peg.5302	fig|6666666.59333.peg.897	fig|6666666.59338.peg.4267	fig|6666666.59339.peg.7196	None	fig|6666666.59337.peg.4106	fig|6666666.59342.peg.473	fig|6666666.71181.peg.3232	fig|6666666.59344.peg.4599	fig|6666666.59345.peg.4325	fig|6666666.59341.peg.2528	fig|6666666.59347.peg.4382	fig|6666666.59346.peg.2445	fig|6666666.59348.peg.2400	fig|6666666.59349.peg.2517	fig|6666666.59350.peg.2863	fig|6666666.59351.peg.3723	None	fig|6666666.59355.peg.5277	fig|6666666.59354.peg.1304

5228	Flagellar basal-body rod modification protein FlgD	fig|6666666.59329.peg.2328	fig|6666666.59330.peg.2686	fig|6666666.59331.peg.2031	fig|6666666.59332.peg.1791	None	fig|6666666.59335.peg.2083	fig|6666666.59336.peg.1961	fig|6666666.59333.peg.2837	fig|6666666.59338.peg.95	fig|6666666.59339.peg.5314	None	fig|6666666.59337.peg.5375	fig|6666666.59342.peg.6437	fig|6666666.71181.peg.2228	fig|6666666.59344.peg.3035	fig|6666666.59345.peg.5451	fig|6666666.59341.peg.3531	fig|6666666.59347.peg.2712	fig|6666666.59346.peg.1764	fig|6666666.59348.peg.950	fig|6666666.59349.peg.725	fig|6666666.59350.peg.1651	fig|6666666.59351.peg.1984	fig|6666666.59353.peg.3525	fig|6666666.59355.peg.3650	fig|6666666.59354.peg.1527

3067	Uncharacterized paraquat-inducible protein B	fig|6666666.59329.peg.762	fig|6666666.59330.peg.3933	fig|6666666.59331.peg.1365	fig|6666666.59332.peg.1218	fig|6666666.59334.peg.4093	fig|6666666.59335.peg.5694	fig|6666666.59336.peg.1214	fig|6666666.59333.peg.2338	fig|6666666.59338.peg.888	fig|6666666.59339.peg.443	None	fig|6666666.59337.peg.3045	fig|6666666.59342.peg.718	fig|6666666.71181.peg.850	fig|6666666.59344.peg.27	fig|6666666.59345.peg.100	fig|6666666.59341.peg.5617	fig|6666666.59347.peg.989	fig|6666666.59346.peg.3460	fig|6666666.59348.peg.3221	fig|6666666.59349.peg.324	None	fig|6666666.59351.peg.3932	fig|6666666.59353.peg.365	fig|6666666.59355.peg.1402	fig|6666666.59354.peg.2170

10126	Uracil permease	fig|6666666.59329.peg.257	fig|6666666.59330.peg.3629	fig|6666666.59331.peg.804	fig|6666666.59332.peg.4440	fig|6666666.59334.peg.5625	fig|6666666.59335.peg.4896	fig|6666666.59336.peg.1604	fig|6666666.59333.peg.3284	fig|6666666.59338.peg.4857	fig|6666666.59339.peg.22	fig|6666666.59340.peg.2544	fig|6666666.59337.peg.4489	fig|6666666.59342.peg.5088	fig|6666666.71181.peg.322	fig|6666666.59344.peg.5187	fig|6666666.59345.peg.2655	fig|6666666.59341.peg.5968	fig|6666666.59347.peg.2859	fig|6666666.59346.peg.2494	fig|6666666.59348.peg.5218	None	fig|6666666.59350.peg.3331	fig|6666666.59351.peg.3512	fig|6666666.59353.peg.4023	fig|6666666.59355.peg.2589	None

5413	ATP-dependent protease HslV EC 3.4.25.-	fig|6666666.59329.peg.1095	fig|6666666.59330.peg.544	None	fig|6666666.59332.peg.1188	fig|6666666.59334.peg.616	fig|6666666.59335.peg.434	fig|6666666.59336.peg.132	fig|6666666.59333.peg.2680	fig|6666666.59338.peg.265	fig|6666666.59339.peg.6390	fig|6666666.59340.peg.2710	fig|6666666.59337.peg.400	fig|6666666.59342.peg.5126	fig|6666666.71181.peg.3894	fig|6666666.59344.peg.1000	fig|6666666.59345.peg.2546	None	fig|6666666.59347.peg.5028	fig|6666666.59346.peg.1011	fig|6666666.59348.peg.156	fig|6666666.59349.peg.509	fig|6666666.59350.peg.1188	fig|6666666.59351.peg.3831	fig|6666666.59353.peg.2540	fig|6666666.59355.peg.2661	fig|6666666.59354.peg.936

1464	tRNA Ile -lysidine synthetase	fig|6666666.59329.peg.5014	fig|6666666.59330.peg.450	fig|6666666.59331.peg.4957	fig|6666666.59332.peg.422	None	fig|6666666.59335.peg.5859	fig|6666666.59336.peg.3374	fig|6666666.59333.peg.1152	fig|6666666.59338.peg.3200	fig|6666666.59339.peg.5134	fig|6666666.59340.peg.374	fig|6666666.59337.peg.1599	None	fig|6666666.71181.peg.15	fig|6666666.59344.peg.2675	fig|6666666.59345.peg.2184	fig|6666666.59341.peg.5462	fig|6666666.59347.peg.4375	fig|6666666.59346.peg.4883	fig|6666666.59348.peg.634	fig|6666666.59349.peg.1358	fig|6666666.59350.peg.2402	fig|6666666.59351.peg.2132	fig|6666666.59353.peg.2005	fig|6666666.59355.peg.4110	fig|6666666.59354.peg.1417

316	SSU ribosomal protein S18p SSU ribosomal protein S18p, zinc-independent	fig|6666666.59329.peg.321	fig|6666666.59330.peg.2641	fig|6666666.59331.peg.5466	fig|6666666.59332.peg.901	fig|6666666.59334.peg.169	fig|6666666.59335.peg.3492	fig|6666666.59336.peg.596	fig|6666666.59333.peg.889	fig|6666666.59338.peg.6123	fig|6666666.59339.peg.7696	None	fig|6666666.59337.peg.1707	fig|6666666.59342.peg.1893	fig|6666666.71181.peg.2819	fig|6666666.59344.peg.2148	fig|6666666.59345.peg.5375	fig|6666666.59341.peg.2419	fig|6666666.59347.peg.5439	fig|6666666.59346.peg.2387	fig|6666666.59348.peg.4966	fig|6666666.59349.peg.3230	None	fig|6666666.59351.peg.5424	fig|6666666.59353.peg.3177	fig|6666666.59355.peg.420	fig|6666666.59354.peg.2012

444	NAD P HX epimerase NAD P HX dehydratase	None	fig|6666666.59330.peg.1149	fig|6666666.59331.peg.4687	fig|6666666.59332.peg.1295	fig|6666666.59334.peg.1345	fig|6666666.59335.peg.5995	fig|6666666.59336.peg.6269	fig|6666666.59333.peg.3979	fig|6666666.59338.peg.4496	fig|6666666.59339.peg.2513	None	fig|6666666.59337.peg.3649	fig|6666666.59342.peg.5225	fig|6666666.71181.peg.1696	fig|6666666.59344.peg.6256	fig|6666666.59345.peg.5198	fig|6666666.59341.peg.4688	fig|6666666.59347.peg.778	fig|6666666.59346.peg.2290	fig|6666666.59348.peg.4635	fig|6666666.59349.peg.3165	fig|6666666.59350.peg.2447	fig|6666666.59351.peg.3089	fig|6666666.59353.peg.1066	fig|6666666.59355.peg.3551	fig|6666666.59354.peg.3719

8691	Exonuclease SbcD	fig|6666666.59329.peg.5628	fig|6666666.59330.peg.3984	None	fig|6666666.59332.peg.3771	fig|6666666.59334.peg.3851	fig|6666666.59335.peg.4495	fig|6666666.59336.peg.4418	fig|6666666.59333.peg.1951	fig|6666666.59338.peg.2686	fig|6666666.59339.peg.4705	None	fig|6666666.59337.peg.1151	fig|6666666.59342.peg.4153	fig|6666666.71181.peg.2748	fig|6666666.59344.peg.1124	fig|6666666.59345.peg.3460	fig|6666666.59341.peg.3320	fig|6666666.59347.peg.764	fig|6666666.59346.peg.4838	fig|6666666.59348.peg.3857	fig|6666666.59349.peg.3108	fig|6666666.59350.peg.3512	fig|6666666.59351.peg.2985	fig|6666666.59353.peg.801	fig|6666666.59355.peg.905	fig|6666666.59354.peg.2688

10578	NAD FAD -utilizing dehydrogenases	fig|6666666.59329.peg.3160	fig|6666666.59330.peg.239	fig|6666666.59331.peg.947	fig|6666666.59332.peg.1723	fig|6666666.59334.peg.1196	fig|6666666.59335.peg.29	None	fig|6666666.59333.peg.672	fig|6666666.59338.peg.79	fig|6666666.59339.peg.7554	fig|6666666.59340.peg.491	fig|6666666.59337.peg.954	None	fig|6666666.71181.peg.1069	fig|6666666.59344.peg.4973	fig|6666666.59345.peg.2720	fig|6666666.59341.peg.4154	fig|6666666.59347.peg.4575	fig|6666666.59346.peg.3448	fig|6666666.59348.peg.1878	fig|6666666.59349.peg.4433	fig|6666666.59350.peg.1035	fig|6666666.59351.peg.3013	fig|6666666.59353.peg.4506	fig|6666666.59355.peg.4616	fig|6666666.59354.peg.2081

10579	Dihydrodipicolinate synthase EC 4.2.1.52	fig|6666666.59329.peg.4213	fig|6666666.59330.peg.3641	None	fig|6666666.59332.peg.5886	fig|6666666.59334.peg.1855	fig|6666666.59335.peg.1198	fig|6666666.59336.peg.5060	fig|6666666.59333.peg.460	fig|6666666.59338.peg.5997	fig|6666666.59339.peg.42	None	fig|6666666.59337.peg.5610	fig|6666666.59342.peg.3257	fig|6666666.71181.peg.3252	fig|6666666.59344.peg.4005	fig|6666666.59345.peg.5600	fig|6666666.59341.peg.1676	fig|6666666.59347.peg.2849	fig|6666666.59346.peg.3549	fig|6666666.59348.peg.79	fig|6666666.59349.peg.3139	fig|6666666.59350.peg.1330	fig|6666666.59351.peg.1565	fig|6666666.59353.peg.4205	fig|6666666.59355.peg.2477	fig|6666666.59354.peg.966

2741	Acyl-phosphate glycerol-3-phosphate O-acyltransferase PlsY	fig|6666666.59329.peg.686	fig|6666666.59330.peg.3127	fig|6666666.59331.peg.5622	fig|6666666.59332.peg.1510	fig|6666666.59334.peg.3130	fig|6666666.59335.peg.1649	fig|6666666.59336.peg.5411	fig|6666666.59333.peg.178	fig|6666666.59338.peg.796	fig|6666666.59339.peg.7911	None	fig|6666666.59337.peg.1428	None	fig|6666666.71181.peg.3011	fig|6666666.59344.peg.4472	fig|6666666.59345.peg.3844	fig|6666666.59341.peg.3716	fig|6666666.59347.peg.3134	fig|6666666.59346.peg.3109	fig|6666666.59348.peg.5493	fig|6666666.59349.peg.1693	fig|6666666.59350.peg.1240	fig|6666666.59351.peg.5222	fig|6666666.59353.peg.4186	fig|6666666.59355.peg.1691	fig|6666666.59354.peg.5369

2746	Arylesterase precursor EC 3.1.1.2	fig|6666666.59329.peg.5280	fig|6666666.59330.peg.3934	fig|6666666.59331.peg.3800	fig|6666666.59332.peg.4988	fig|6666666.59334.peg.4092	fig|6666666.59335.peg.4279	None	fig|6666666.59333.peg.2339	fig|6666666.59338.peg.1719	fig|6666666.59339.peg.2848	None	fig|6666666.59337.peg.3044	fig|6666666.59342.peg.1747	fig|6666666.71181.peg.3619	fig|6666666.59344.peg.5067	fig|6666666.59345.peg.99	fig|6666666.59341.peg.326	fig|6666666.59347.peg.3673	fig|6666666.59346.peg.3640	fig|6666666.59348.peg.4521	fig|6666666.59349.peg.4677	fig|6666666.59350.peg.1674	fig|6666666.59351.peg.1301	fig|6666666.59353.peg.4050	fig|6666666.59355.peg.4126	fig|6666666.59354.peg.4249

3046	UPF0301 protein YqgE	fig|6666666.59329.peg.1771	fig|6666666.59330.peg.346	None	fig|6666666.59332.peg.1941	fig|6666666.59334.peg.2566	fig|6666666.59335.peg.3094	fig|6666666.59336.peg.3074	fig|6666666.59333.peg.772	fig|6666666.59338.peg.448	fig|6666666.59339.peg.7834	fig|6666666.59340.peg.204	fig|6666666.59337.peg.527	fig|6666666.59342.peg.5710	fig|6666666.71181.peg.1862	fig|6666666.59344.peg.4336	fig|6666666.59345.peg.5017	fig|6666666.59341.peg.2573	fig|6666666.59347.peg.3214	fig|6666666.59346.peg.3523	fig|6666666.59348.peg.354	fig|6666666.59349.peg.806	fig|6666666.59350.peg.1252	fig|6666666.59351.peg.3535	fig|6666666.59353.peg.3227	fig|6666666.59355.peg.3109	None

1915	FIG006238 AzlC family protein	None	fig|6666666.59330.peg.3230	fig|6666666.59331.peg.3336	fig|6666666.59332.peg.4300	fig|6666666.59334.peg.3992	fig|6666666.59335.peg.4818	fig|6666666.59336.peg.1573	fig|6666666.59333.peg.4722	fig|6666666.59338.peg.2340	fig|6666666.59339.peg.4142	None	fig|6666666.59337.peg.1971	fig|6666666.59342.peg.3383	fig|6666666.71181.peg.2639	fig|6666666.59344.peg.3912	fig|6666666.59345.peg.5606	fig|6666666.59341.peg.3021	fig|6666666.59347.peg.860	fig|6666666.59346.peg.2091	fig|6666666.59348.peg.3663	fig|6666666.59349.peg.2036	fig|6666666.59350.peg.4411	fig|6666666.59351.peg.2425	fig|6666666.59353.peg.4750	fig|6666666.59355.peg.4054	fig|6666666.59354.peg.2049

1326	4-alpha-glucanotransferase amylomaltase EC 2.4.1.25	fig|6666666.59329.peg.4591	fig|6666666.59330.peg.4820	None	fig|6666666.59332.peg.3920	fig|6666666.59334.peg.2529	fig|6666666.59335.peg.4404	fig|6666666.59336.peg.804	fig|6666666.59333.peg.4751	fig|6666666.59338.peg.1082	fig|6666666.59339.peg.1717	fig|6666666.59340.peg.2511	fig|6666666.59337.peg.4349	fig|6666666.59342.peg.1906	fig|6666666.71181.peg.4487	fig|6666666.59344.peg.3635	fig|6666666.59345.peg.1914	fig|6666666.59341.peg.2596	fig|6666666.59347.peg.150	fig|6666666.59346.peg.1332	fig|6666666.59348.peg.4835	fig|6666666.59349.peg.964	None	fig|6666666.59351.peg.1582	fig|6666666.59353.peg.2185	fig|6666666.59355.peg.1879	fig|6666666.59354.peg.507

1914	2-Keto-3-deoxy-D-manno-octulosonate-8-phosphate synthase EC 2.5.1.55	fig|6666666.59329.peg.860	fig|6666666.59330.peg.4527	None	fig|6666666.59332.peg.2617	fig|6666666.59334.peg.3884	fig|6666666.59335.peg.1184	fig|6666666.59336.peg.5114	fig|6666666.59333.peg.227	fig|6666666.59338.peg.2087	fig|6666666.59339.peg.16	fig|6666666.59340.peg.1671	fig|6666666.59337.peg.5171	fig|6666666.59342.peg.4302	fig|6666666.71181.peg.5098	fig|6666666.59344.peg.115	fig|6666666.59345.peg.5421	fig|6666666.59341.peg.3048	fig|6666666.59347.peg.5275	None	fig|6666666.59348.peg.2375	fig|6666666.59349.peg.2621	fig|6666666.59350.peg.2233	fig|6666666.59351.peg.4655	fig|6666666.59353.peg.436	fig|6666666.59355.peg.2164	fig|6666666.59354.peg.2792

4069	Homoserine O-succinyltransferase EC 2.3.1.46	fig|6666666.59329.peg.1932	fig|6666666.59330.peg.4393	fig|6666666.59331.peg.1761	fig|6666666.59332.peg.4424	fig|6666666.59334.peg.4023	fig|6666666.59335.peg.2012	fig|6666666.59336.peg.4333	fig|6666666.59333.peg.2384	None	fig|6666666.59339.peg.3950	None	fig|6666666.59337.peg.2658	fig|6666666.59342.peg.4335	fig|6666666.71181.peg.4891	fig|6666666.59344.peg.2690	fig|6666666.59345.peg.698	fig|6666666.59341.peg.727	fig|6666666.59347.peg.3441	fig|6666666.59346.peg.1447	fig|6666666.59348.peg.2881	fig|6666666.59349.peg.3671	fig|6666666.59350.peg.6155	fig|6666666.59351.peg.1458	fig|6666666.59353.peg.3036	fig|6666666.59355.peg.2943	fig|6666666.59354.peg.1735

4450	Ribose-phosphate pyrophosphokinase EC 2.7.6.1	None	fig|6666666.59330.peg.4669	fig|6666666.59331.peg.1441	fig|6666666.59332.peg.4261	fig|6666666.59334.peg.2238	fig|6666666.59335.peg.1174	fig|6666666.59336.peg.5139	fig|6666666.59333.peg.5424	fig|6666666.59338.peg.4130	fig|6666666.59339.peg.3	None	fig|6666666.59337.peg.5163	fig|6666666.59342.peg.469	fig|6666666.71181.peg.783	fig|6666666.59344.peg.104	fig|6666666.59345.peg.5412	fig|6666666.59341.peg.6006	fig|6666666.59347.peg.5283	fig|6666666.59346.peg.3187	fig|6666666.59348.peg.613	fig|6666666.59349.peg.2611	fig|6666666.59350.peg.2082	fig|6666666.59351.peg.4666	fig|6666666.59353.peg.428	fig|6666666.59355.peg.2154	fig|6666666.59354.peg.4337

2829	FIG002927 hypothetical protein	fig|6666666.59329.peg.3798	fig|6666666.59330.peg.1198	fig|6666666.59331.peg.5433	fig|6666666.59332.peg.5054	fig|6666666.59334.peg.4397	fig|6666666.59335.peg.1425	fig|6666666.59336.peg.5050	fig|6666666.59333.peg.2465	fig|6666666.59338.peg.5703	fig|6666666.59339.peg.217	None	fig|6666666.59337.peg.3144	fig|6666666.59342.peg.939	fig|6666666.71181.peg.491	fig|6666666.59344.peg.1703	fig|6666666.59345.peg.1148	fig|6666666.59341.peg.1124	fig|6666666.59347.peg.2575	fig|6666666.59346.peg.97	fig|6666666.59348.peg.2361	fig|6666666.59349.peg.2702	None	fig|6666666.59351.peg.5176	fig|6666666.59353.peg.1118	fig|6666666.59355.peg.358	fig|6666666.59354.peg.5254

2824	Transcriptional regulator, HxlR family	fig|6666666.59329.peg.1261	None	fig|6666666.59331.peg.4377	fig|6666666.59332.peg.3446	fig|6666666.59334.peg.5091	fig|6666666.59335.peg.678	fig|6666666.59336.peg.5531	fig|6666666.59333.peg.5091	fig|6666666.59338.peg.5802	fig|6666666.59339.peg.1781	fig|6666666.59340.peg.2774	fig|6666666.59337.peg.1939	fig|6666666.59342.peg.3039	fig|6666666.71181.peg.1789	fig|6666666.59344.peg.5024	fig|6666666.59345.peg.4033	fig|6666666.59341.peg.3900	fig|6666666.59347.peg.2738	fig|6666666.59346.peg.414	fig|6666666.59348.peg.2702	fig|6666666.59349.peg.2527	fig|6666666.59350.peg.536	fig|6666666.59351.peg.3239	fig|6666666.59353.peg.263	fig|6666666.59355.peg.5306	None

5418	Flagellar L-ring protein FlgH	fig|6666666.59329.peg.2684	fig|6666666.59330.peg.4412	None	fig|6666666.59332.peg.2238	None	fig|6666666.59335.peg.905	fig|6666666.59336.peg.5045	fig|6666666.59333.peg.2893	fig|6666666.59338.peg.1243	fig|6666666.59339.peg.5321	fig|6666666.59340.peg.3339	fig|6666666.59337.peg.3185	fig|6666666.59342.peg.4223	fig|6666666.71181.peg.2224	fig|6666666.59344.peg.3041	fig|6666666.59345.peg.2922	fig|6666666.59341.peg.3260	fig|6666666.59347.peg.4599	fig|6666666.59346.peg.1768	fig|6666666.59348.peg.954	fig|6666666.59349.peg.729	fig|6666666.59350.peg.7004	fig|6666666.59351.peg.1988	fig|6666666.59353.peg.3521	fig|6666666.59355.peg.3654	fig|6666666.59354.peg.1532

5419	Flagellar biosynthesis protein FliQ	fig|6666666.59329.peg.1322	fig|6666666.59330.peg.1879	fig|6666666.59331.peg.5220	None	None	fig|6666666.59335.peg.1574	fig|6666666.59336.peg.5816	fig|6666666.59333.peg.2868	fig|6666666.59338.peg.1767	fig|6666666.59339.peg.1333	fig|6666666.59340.peg.3054	fig|6666666.59337.peg.2970	fig|6666666.59342.peg.4510	fig|6666666.71181.peg.4932	fig|6666666.59344.peg.3050	fig|6666666.59345.peg.2867	fig|6666666.59341.peg.3278	fig|6666666.59347.peg.4621	fig|6666666.59346.peg.2891	fig|6666666.59348.peg.3693	fig|6666666.59349.peg.200	fig|6666666.59350.peg.7929	fig|6666666.59351.peg.2057	fig|6666666.59353.peg.3514	fig|6666666.59355.peg.5745	fig|6666666.59354.peg.677

4005	Outer membrane protein NlpB, lipoprotein component of the protein assembly complex forms a complex with YaeT, YfiO, and YfgL ; Lipoprotein-34 precursor	fig|6666666.59329.peg.4214	fig|6666666.59330.peg.3642	fig|6666666.59331.peg.4758	fig|6666666.59332.peg.5885	fig|6666666.59334.peg.1854	fig|6666666.59335.peg.1199	fig|6666666.59336.peg.2167	fig|6666666.59333.peg.5425	fig|6666666.59338.peg.782	fig|6666666.59339.peg.45	None	fig|6666666.59337.peg.5609	fig|6666666.59342.peg.3258	fig|6666666.71181.peg.3251	fig|6666666.59344.peg.4004	fig|6666666.59345.peg.2153	fig|6666666.59341.peg.1677	fig|6666666.59347.peg.2848	fig|6666666.59346.peg.3548	fig|6666666.59348.peg.80	fig|6666666.59349.peg.2943	None	fig|6666666.59351.peg.608	fig|6666666.59353.peg.4045	fig|6666666.59355.peg.4002	fig|6666666.59354.peg.4935

571	Malate synthase EC 2.3.3.9	fig|6666666.59329.peg.653	fig|6666666.59330.peg.1399	fig|6666666.59331.peg.4524	fig|6666666.59332.peg.908	fig|6666666.59334.peg.4592	fig|6666666.59335.peg.3783	fig|6666666.59336.peg.4445	fig|6666666.59333.peg.361	fig|6666666.59338.peg.2470	fig|6666666.59339.peg.5389	None	fig|6666666.59337.peg.723	None	fig|6666666.71181.peg.4892	fig|6666666.59344.peg.2691	fig|6666666.59345.peg.3666	fig|6666666.59341.peg.5896	fig|6666666.59347.peg.3337	fig|6666666.59346.peg.3236	fig|6666666.59348.peg.4593	fig|6666666.59349.peg.1498	fig|6666666.59350.peg.4869	fig|6666666.59351.peg.2998	fig|6666666.59353.peg.3035	fig|6666666.59355.peg.2942	fig|6666666.59354.peg.218

6559	Rod shape-determining protein MreB	fig|6666666.59329.peg.704	fig|6666666.59330.peg.24	fig|6666666.59331.peg.5438	fig|6666666.59332.peg.4117	fig|6666666.59334.peg.4124	fig|6666666.59335.peg.1804	fig|6666666.59336.peg.2669	fig|6666666.59333.peg.2528	fig|6666666.59338.peg.5687	fig|6666666.59339.peg.3605	None	fig|6666666.59337.peg.2622	fig|6666666.59342.peg.3510	fig|6666666.71181.peg.978	fig|6666666.59344.peg.5329	fig|6666666.59345.peg.724	fig|6666666.59341.peg.882	fig|6666666.59347.peg.1682	fig|6666666.59346.peg.2126	fig|6666666.59348.peg.982	fig|6666666.59349.peg.4	None	fig|6666666.59351.peg.3421	fig|6666666.59353.peg.2292	fig|6666666.59355.peg.4811	fig|6666666.59354.peg.4572

350	Ribosomal-protein-S18p-alanine acetyltransferase EC 2.3.1.-	None	fig|6666666.59330.peg.89	fig|6666666.59331.peg.5130	fig|6666666.59332.peg.5333	fig|6666666.59334.peg.4216	fig|6666666.59335.peg.2853	fig|6666666.59336.peg.3568	fig|6666666.59333.peg.2316	None	fig|6666666.59339.peg.4868	fig|6666666.59340.peg.2613	fig|6666666.59337.peg.1297	fig|6666666.59342.peg.4541	fig|6666666.71181.peg.467	fig|6666666.59344.peg.1386	fig|6666666.59345.peg.2341	fig|6666666.59341.peg.5170	fig|6666666.59347.peg.2987	fig|6666666.59346.peg.1991	fig|6666666.59348.peg.4833	fig|6666666.59349.peg.1679	fig|6666666.59350.peg.5331	fig|6666666.59351.peg.390	fig|6666666.59353.peg.29	fig|6666666.59355.peg.636	fig|6666666.59354.peg.3479

6128	Probable exported or periplasmic protein in ApbE locus	fig|6666666.59329.peg.6438	fig|6666666.59330.peg.4052	None	fig|6666666.59332.peg.2209	fig|6666666.59334.peg.888	fig|6666666.59335.peg.127	fig|6666666.59336.peg.5235	fig|6666666.59333.peg.818	fig|6666666.59338.peg.2121	fig|6666666.59339.peg.5056	fig|6666666.59340.peg.947	fig|6666666.59337.peg.2858	fig|6666666.59342.peg.1980	fig|6666666.71181.peg.922	fig|6666666.59344.peg.5162	fig|6666666.59345.peg.1738	fig|6666666.59341.peg.2298	fig|6666666.59347.peg.2795	fig|6666666.59346.peg.4252	fig|6666666.59348.peg.2978	fig|6666666.59349.peg.4241	None	fig|6666666.59351.peg.3923	fig|6666666.59353.peg.892	fig|6666666.59355.peg.812	fig|6666666.59354.peg.6378

6496	L-proline glycine betaine binding ABC transporter protein ProX TC 3.A.1.12.1	fig|6666666.59329.peg.5412	fig|6666666.59330.peg.680	fig|6666666.59331.peg.2600	fig|6666666.59332.peg.2829	fig|6666666.59334.peg.3909	fig|6666666.59335.peg.4948	fig|6666666.59336.peg.2618	fig|6666666.59333.peg.4015	fig|6666666.59338.peg.1062	fig|6666666.59339.peg.1848	fig|6666666.59340.peg.2082	fig|6666666.59337.peg.5262	fig|6666666.59342.peg.6132	fig|6666666.71181.peg.2635	fig|6666666.59344.peg.2223	fig|6666666.59345.peg.1741	fig|6666666.59341.peg.5338	fig|6666666.59347.peg.800	fig|6666666.59346.peg.1747	fig|6666666.59348.peg.1193	None	fig|6666666.59350.peg.2239	fig|6666666.59351.peg.892	fig|6666666.59353.peg.188	fig|6666666.59355.peg.2957	None

8395	tRNA 5-methylaminomethyl-2-thiouridine synthase TusA	None	fig|6666666.59330.peg.1090	None	fig|6666666.59332.peg.72	fig|6666666.59334.peg.149	fig|6666666.59335.peg.3919	fig|6666666.59336.peg.5631	fig|6666666.59333.peg.3966	fig|6666666.59338.peg.5333	fig|6666666.59339.peg.8053	fig|6666666.59340.peg.975	fig|6666666.59337.peg.863	fig|6666666.59342.peg.2610	fig|6666666.71181.peg.4499	fig|6666666.59344.peg.5718	fig|6666666.59345.peg.4990	fig|6666666.59341.peg.5109	fig|6666666.59347.peg.4808	fig|6666666.59346.peg.4290	fig|6666666.59348.peg.1212	fig|6666666.59349.peg.990	fig|6666666.59350.peg.1967	fig|6666666.59351.peg.5038	fig|6666666.59353.peg.4940	fig|6666666.59355.peg.6030	fig|6666666.59354.peg.2375

4026	Deoxyribose-phosphate aldolase EC 4.1.2.4	fig|6666666.59329.peg.2620	fig|6666666.59330.peg.84	None	fig|6666666.59332.peg.4670	fig|6666666.59334.peg.495	fig|6666666.59335.peg.3639	fig|6666666.59336.peg.4268	fig|6666666.59333.peg.2572	fig|6666666.59338.peg.3049	fig|6666666.59339.peg.5015	None	fig|6666666.59337.peg.1303	fig|6666666.59342.peg.1786	fig|6666666.71181.peg.459	fig|6666666.59344.peg.1376	fig|6666666.59345.peg.2335	fig|6666666.59341.peg.5003	fig|6666666.59347.peg.2971	fig|6666666.59346.peg.2447	fig|6666666.59348.peg.4209	fig|6666666.59349.peg.1149	fig|6666666.59350.peg.908	fig|6666666.59351.peg.5235	fig|6666666.59353.peg.37	fig|6666666.59355.peg.645	fig|6666666.59354.peg.5810

4029	Septum site-determining protein MinC	fig|6666666.59329.peg.4743	fig|6666666.59330.peg.4583	None	fig|6666666.59332.peg.1214	fig|6666666.59334.peg.3447	fig|6666666.59335.peg.2760	fig|6666666.59336.peg.1022	fig|6666666.59333.peg.1358	fig|6666666.59338.peg.1137	fig|6666666.59339.peg.240	fig|6666666.59340.peg.1863	fig|6666666.59337.peg.2752	fig|6666666.59342.peg.5018	fig|6666666.71181.peg.2942	fig|6666666.59344.peg.2858	fig|6666666.59345.peg.4295	fig|6666666.59341.peg.3859	fig|6666666.59347.peg.4415	fig|6666666.59346.peg.1473	fig|6666666.59348.peg.1740	fig|6666666.59349.peg.476	None	fig|6666666.59351.peg.1646	fig|6666666.59353.peg.1462	fig|6666666.59355.peg.2013	fig|6666666.59354.peg.5729

3996	DNA-binding protein Fis	None	fig|6666666.59330.peg.197	fig|6666666.59331.peg.3194	fig|6666666.59332.peg.125	fig|6666666.59334.peg.51	fig|6666666.59335.peg.2630	fig|6666666.59336.peg.2268	fig|6666666.59333.peg.2641	fig|6666666.59338.peg.5021	fig|6666666.59339.peg.6437	fig|6666666.59340.peg.820	fig|6666666.59337.peg.4285	fig|6666666.59342.peg.2609	fig|6666666.71181.peg.966	fig|6666666.59344.peg.5346	fig|6666666.59345.peg.5149	fig|6666666.59341.peg.1183	fig|6666666.59347.peg.4490	fig|6666666.59346.peg.1148	fig|6666666.59348.peg.942	fig|6666666.59349.peg.1950	None	fig|6666666.59351.peg.2359	fig|6666666.59353.peg.2280	fig|6666666.59355.peg.4824	fig|6666666.59354.peg.5

1435	5-carboxymethyl-2-hydroxymuconate delta-isomerase EC 5.3.3.10	fig|6666666.59329.peg.870	fig|6666666.59330.peg.5014	fig|6666666.59331.peg.4053	fig|6666666.59332.peg.164	fig|6666666.59334.peg.961	fig|6666666.59335.peg.3559	fig|6666666.59336.peg.2173	fig|6666666.59333.peg.1390	fig|6666666.59338.peg.2360	fig|6666666.59339.peg.8679	None	fig|6666666.59337.peg.411	None	fig|6666666.71181.peg.4554	fig|6666666.59344.peg.1419	fig|6666666.59345.peg.2556	fig|6666666.59341.peg.3502	fig|6666666.59347.peg.4530	fig|6666666.59346.peg.796	fig|6666666.59348.peg.4755	fig|6666666.59349.peg.1935	fig|6666666.59350.peg.2021	fig|6666666.59351.peg.2342	fig|6666666.59353.peg.7	fig|6666666.59355.peg.613	fig|6666666.59354.peg.1714

1621	Uncharacterized protein YeaC	fig|6666666.59329.peg.3068	fig|6666666.59330.peg.1214	fig|6666666.59331.peg.6072	fig|6666666.59332.peg.5578	fig|6666666.59334.peg.1619	fig|6666666.59335.peg.2945	fig|6666666.59336.peg.3560	fig|6666666.59333.peg.1084	fig|6666666.59338.peg.4733	fig|6666666.59339.peg.274	fig|6666666.59340.peg.3644	fig|6666666.59337.peg.3129	fig|6666666.59342.peg.1234	fig|6666666.71181.peg.2972	fig|6666666.59344.peg.2895	fig|6666666.59345.peg.1161	fig|6666666.59341.peg.2	fig|6666666.59347.peg.4075	fig|6666666.59346.peg.81	None	fig|6666666.59349.peg.2747	None	fig|6666666.59351.peg.770	fig|6666666.59353.peg.1432	fig|6666666.59355.peg.1981	fig|6666666.59354.peg.4922

5533	Chorismate mutase I EC 5.4.99.5 Cyclohexadienyl dehydrogenase EC 1.3.1.12 EC 1.3.1.43	fig|6666666.59329.peg.2554	fig|6666666.59330.peg.2883	None	fig|6666666.59332.peg.2641	fig|6666666.59334.peg.1931	fig|6666666.59335.peg.2055	fig|6666666.59336.peg.850	fig|6666666.59333.peg.3356	fig|6666666.59338.peg.2967	fig|6666666.59339.peg.7493	fig|6666666.59340.peg.683	fig|6666666.59337.peg.146	fig|6666666.59342.peg.530	fig|6666666.71181.peg.3948	None	fig|6666666.59345.peg.3695	fig|6666666.59341.peg.106	fig|6666666.59347.peg.4046	fig|6666666.59346.peg.253	fig|6666666.59348.peg.393	fig|6666666.59349.peg.4859	fig|6666666.59350.peg.2408	fig|6666666.59351.peg.545	fig|6666666.59353.peg.4805	fig|6666666.59355.peg.4859	fig|6666666.59354.peg.6012

6571	Cell division protein FtsA	fig|6666666.59329.peg.1362	fig|6666666.59330.peg.1281	fig|6666666.59331.peg.4093	fig|6666666.59332.peg.3449	fig|6666666.59334.peg.3099	fig|6666666.59335.peg.827	fig|6666666.59336.peg.953	None	fig|6666666.59338.peg.2683	fig|6666666.59339.peg.4977	None	fig|6666666.59337.peg.52	fig|6666666.59342.peg.2796	fig|6666666.71181.peg.1976	fig|6666666.59344.peg.1475	fig|6666666.59345.peg.3789	fig|6666666.59341.peg.4864	fig|6666666.59347.peg.4004	fig|6666666.59346.peg.1245	fig|6666666.59348.peg.260	fig|6666666.59349.peg.2989	fig|6666666.59350.peg.6116	fig|6666666.59351.peg.213	fig|6666666.59353.peg.142	fig|6666666.59355.peg.752	fig|6666666.59354.peg.4404

7690	Chaperone protein HscB	fig|6666666.59329.peg.1108	fig|6666666.59330.peg.1417	fig|6666666.59331.peg.1214	fig|6666666.59332.peg.1331	fig|6666666.59334.peg.3067	fig|6666666.59335.peg.2370	fig|6666666.59336.peg.671	fig|6666666.59333.peg.927	fig|6666666.59338.peg.1323	fig|6666666.59339.peg.6795	None	fig|6666666.59337.peg.742	fig|6666666.59342.peg.3300	fig|6666666.71181.peg.1760	fig|6666666.59344.peg.1630	fig|6666666.59345.peg.3649	fig|6666666.59341.peg.4639	fig|6666666.59347.peg.4286	fig|6666666.59346.peg.2736	fig|6666666.59348.peg.1374	fig|6666666.59349.peg.2432	None	fig|6666666.59351.peg.2710	fig|6666666.59353.peg.2697	fig|6666666.59355.peg.1782	fig|6666666.59354.peg.4571

11102	Alkyl hydroperoxide reductase subunit C-like protein	fig|6666666.59329.peg.1126	fig|6666666.59330.peg.4240	None	fig|6666666.59332.peg.768	fig|6666666.59334.peg.4576	fig|6666666.59335.peg.3697	fig|6666666.59336.peg.5463	fig|6666666.59333.peg.359	fig|6666666.59338.peg.1815	fig|6666666.59339.peg.6883	None	fig|6666666.59337.peg.721	fig|6666666.59342.peg.3675	fig|6666666.71181.peg.2737	fig|6666666.59344.peg.1109	fig|6666666.59345.peg.3668	fig|6666666.59341.peg.3368	fig|6666666.59347.peg.4272	fig|6666666.59346.peg.5029	fig|6666666.59348.peg.1679	fig|6666666.59349.peg.2725	fig|6666666.59350.peg.938	fig|6666666.59351.peg.568	fig|6666666.59353.peg.789	fig|6666666.59355.peg.916	fig|6666666.59354.peg.118

7521	ATP-dependent helicase DinG Rad3	fig|6666666.59329.peg.2652	fig|6666666.59330.peg.1311	fig|6666666.59331.peg.1756	fig|6666666.59332.peg.5773	fig|6666666.59334.peg.3610	fig|6666666.59335.peg.2032	fig|6666666.59336.peg.5824	fig|6666666.59333.peg.1017	fig|6666666.59338.peg.5401	fig|6666666.59339.peg.3150	None	fig|6666666.59337.peg.3380	fig|6666666.59342.peg.5662	fig|6666666.71181.peg.1938	fig|6666666.59344.peg.2848	fig|6666666.59345.peg.1561	fig|6666666.59341.peg.2335	fig|6666666.59347.peg.2362	fig|6666666.59346.peg.1483	fig|6666666.59348.peg.2145	fig|6666666.59349.peg.464	None	fig|6666666.59351.peg.474	fig|6666666.59353.peg.4742	fig|6666666.59355.peg.2020	fig|6666666.59354.peg.885

373	Manganese superoxide dismutase EC 1.15.1.1	fig|6666666.59329.peg.2021	fig|6666666.59330.peg.172	fig|6666666.59331.peg.4357	fig|6666666.59332.peg.156	fig|6666666.59334.peg.1430	fig|6666666.59335.peg.3124	fig|6666666.59336.peg.2316	fig|6666666.59333.peg.415	fig|6666666.59338.peg.4235	fig|6666666.59339.peg.8704	None	fig|6666666.59337.peg.4422	fig|6666666.59342.peg.6337	fig|6666666.71181.peg.671	fig|6666666.59344.peg.4116	fig|6666666.59345.peg.5124	fig|6666666.59341.peg.2714	fig|6666666.59347.peg.1295	fig|6666666.59346.peg.789	fig|6666666.59348.peg.4014	fig|6666666.59349.peg.2394	None	fig|6666666.59351.peg.636	fig|6666666.59353.peg.4096	fig|6666666.59355.peg.2343	fig|6666666.59354.peg.5165

6967	CrcB protein	fig|6666666.59329.peg.3963	fig|6666666.59330.peg.975	None	fig|6666666.59332.peg.1929	fig|6666666.59334.peg.5046	fig|6666666.59335.peg.2	fig|6666666.59336.peg.173	fig|6666666.59333.peg.3926	fig|6666666.59338.peg.6032	fig|6666666.59339.peg.7525	fig|6666666.59340.peg.2669	fig|6666666.59337.peg.2555	None	fig|6666666.71181.peg.2378	fig|6666666.59344.peg.3342	fig|6666666.59345.peg.5547	fig|6666666.59341.peg.5654	fig|6666666.59347.peg.5259	fig|6666666.59346.peg.936	fig|6666666.59348.peg.1094	fig|6666666.59349.peg.1586	fig|6666666.59350.peg.4585	fig|6666666.59351.peg.1003	fig|6666666.59353.peg.3723	fig|6666666.59355.peg.3738	fig|6666666.59354.peg.2189

411	Protein YicC	fig|6666666.59329.peg.3644	fig|6666666.59330.peg.1175	None	fig|6666666.59332.peg.718	fig|6666666.59334.peg.230	fig|6666666.59335.peg.1984	fig|6666666.59336.peg.4647	fig|6666666.59333.peg.666	fig|6666666.59338.peg.6015	fig|6666666.59339.peg.6275	fig|6666666.59340.peg.150	fig|6666666.59337.peg.480	None	fig|6666666.71181.peg.2611	fig|6666666.59344.peg.932	fig|6666666.59345.peg.2628	fig|6666666.59341.peg.5783	fig|6666666.59347.peg.5195	fig|6666666.59346.peg.1393	fig|6666666.59348.peg.737	fig|6666666.59349.peg.3069	fig|6666666.59350.peg.599	fig|6666666.59351.peg.330	fig|6666666.59353.peg.2600	fig|6666666.59355.peg.2602	fig|6666666.59354.peg.3328

6689	Ethidium bromide-methyl viologen resistance protein EmrE	fig|6666666.59329.peg.1927	fig|6666666.59330.peg.4397	fig|6666666.59331.peg.1485	fig|6666666.59332.peg.2327	fig|6666666.59334.peg.1554	fig|6666666.59335.peg.3790	fig|6666666.59336.peg.4737	fig|6666666.59333.peg.1185	fig|6666666.59338.peg.6168	fig|6666666.59339.peg.5510	fig|6666666.59340.peg.1197	fig|6666666.59337.peg.1830	fig|6666666.59342.peg.3556	fig|6666666.71181.peg.1936	fig|6666666.59344.peg.2844	fig|6666666.59345.peg.2739	fig|6666666.59341.peg.2005	fig|6666666.59347.peg.289	fig|6666666.59346.peg.3365	None	fig|6666666.59349.peg.1544	fig|6666666.59350.peg.3845	None	fig|6666666.59353.peg.1054	fig|6666666.59355.peg.2022	fig|6666666.59354.peg.3539

5467	Excinuclease ABC subunit C	fig|6666666.59329.peg.960	fig|6666666.59330.peg.2940	None	fig|6666666.59332.peg.3932	fig|6666666.59334.peg.2911	fig|6666666.59335.peg.3765	fig|6666666.59336.peg.1775	fig|6666666.59333.peg.4266	fig|6666666.59338.peg.5901	fig|6666666.59339.peg.577	None	fig|6666666.59337.peg.1740	fig|6666666.59342.peg.2147	fig|6666666.71181.peg.354	fig|6666666.59344.peg.2382	fig|6666666.59345.peg.903	fig|6666666.59341.peg.377	fig|6666666.59347.peg.2293	fig|6666666.59346.peg.1619	fig|6666666.59348.peg.2799	fig|6666666.59349.peg.3455	fig|6666666.59350.peg.501	fig|6666666.59351.peg.3981	fig|6666666.59353.peg.268	fig|6666666.59355.peg.1307	fig|6666666.59354.peg.3384

6352	Putative cytoplasmic protein	fig|6666666.59329.peg.3074	fig|6666666.59330.peg.951	fig|6666666.59331.peg.6259	fig|6666666.59332.peg.1424	fig|6666666.59334.peg.391	fig|6666666.59335.peg.2797	fig|6666666.59336.peg.1940	fig|6666666.59333.peg.631	fig|6666666.59338.peg.969	fig|6666666.59339.peg.4925	None	fig|6666666.59337.peg.17	fig|6666666.59342.peg.3214	fig|6666666.71181.peg.3036	fig|6666666.59344.peg.5621	fig|6666666.59345.peg.3824	fig|6666666.59341.peg.1721	fig|6666666.59347.peg.3911	fig|6666666.59346.peg.1632	fig|6666666.59348.peg.2785	fig|6666666.59349.peg.817	None	fig|6666666.59351.peg.3546	fig|6666666.59353.peg.4922	fig|6666666.59355.peg.4965	fig|6666666.59354.peg.661

1819	Phosphopentomutase EC 5.4.2.7	fig|6666666.59329.peg.1046	fig|6666666.59330.peg.81	None	fig|6666666.59332.peg.4154	fig|6666666.59334.peg.492	fig|6666666.59335.peg.3643	None	fig|6666666.59333.peg.2569	fig|6666666.59338.peg.3051	fig|6666666.59339.peg.5021	fig|6666666.59340.peg.3196	fig|6666666.59337.peg.1306	fig|6666666.59342.peg.1789	fig|6666666.71181.peg.457	fig|6666666.59344.peg.1371	fig|6666666.59345.peg.2333	fig|6666666.59341.peg.4731	fig|6666666.59347.peg.2969	fig|6666666.59346.peg.2450	fig|6666666.59348.peg.3314	fig|6666666.59349.peg.4715	fig|6666666.59350.peg.2604	fig|6666666.59351.peg.1194	fig|6666666.59353.peg.39	fig|6666666.59355.peg.647	fig|6666666.59354.peg.3746

2162	Flagellar motor switch protein FliG	fig|6666666.59329.peg.1711	fig|6666666.59330.peg.2220	fig|6666666.59331.peg.6169	fig|6666666.59332.peg.5490	None	fig|6666666.59335.peg.2397	fig|6666666.59336.peg.476	fig|6666666.59333.peg.4833	fig|6666666.59338.peg.3340	fig|6666666.59339.peg.119	fig|6666666.59340.peg.1570	fig|6666666.59337.peg.2959	fig|6666666.59342.peg.4409	fig|6666666.71181.peg.4027	fig|6666666.59344.peg.6216	fig|6666666.59345.peg.5344	fig|6666666.59341.peg.145	fig|6666666.59347.peg.2690	fig|6666666.59346.peg.2878	None	fig|6666666.59349.peg.211	fig|6666666.59350.peg.7352	fig|6666666.59351.peg.2047	fig|6666666.59353.peg.4562	fig|6666666.59355.peg.5160	fig|6666666.59354.peg.1072

3320	tRNA 5-methylaminomethyl-2-thiouridine synthase TusC	fig|6666666.59329.peg.3693	fig|6666666.59330.peg.4073	fig|6666666.59331.peg.5455	fig|6666666.59332.peg.1076	fig|6666666.59334.peg.440	fig|6666666.59335.peg.5578	fig|6666666.59336.peg.2180	fig|6666666.59333.peg.3104	None	fig|6666666.59339.peg.8872	fig|6666666.59340.peg.942	fig|6666666.59337.peg.1822	fig|6666666.59342.peg.1922	fig|6666666.71181.peg.4311	fig|6666666.59344.peg.3130	fig|6666666.59345.peg.5553	fig|6666666.59341.peg.5088	fig|6666666.59347.peg.5333	fig|6666666.59346.peg.44	fig|6666666.59348.peg.3179	fig|6666666.59349.peg.4843	None	fig|6666666.59351.peg.3130	fig|6666666.59353.peg.2272	fig|6666666.59355.peg.1965	fig|6666666.59354.peg.5881

530	FIG002903 a protein of unknown function perhaps involved in purine metabolism	fig|6666666.59329.peg.5829	fig|6666666.59330.peg.2870	None	fig|6666666.59332.peg.5038	fig|6666666.59334.peg.1443	fig|6666666.59335.peg.600	fig|6666666.59336.peg.3108	fig|6666666.59333.peg.4985	fig|6666666.59338.peg.403	fig|6666666.59339.peg.480	fig|6666666.59340.peg.2662	fig|6666666.59337.peg.5579	fig|6666666.59342.peg.536	fig|6666666.71181.peg.1248	fig|6666666.59344.peg.160	fig|6666666.59345.peg.1315	fig|6666666.59341.peg.489	fig|6666666.59347.peg.4132	fig|6666666.59346.peg.1563	fig|6666666.59348.peg.3603	fig|6666666.59349.peg.2887	None	fig|6666666.59351.peg.871	fig|6666666.59353.peg.471	fig|6666666.59355.peg.2196	fig|6666666.59354.peg.5488

3816	Phosphoheptose isomerase EC 5.3.1.-	fig|6666666.59329.peg.4582	fig|6666666.59330.peg.970	fig|6666666.59331.peg.5648	fig|6666666.59332.peg.2299	fig|6666666.59334.peg.2333	fig|6666666.59335.peg.4929	fig|6666666.59336.peg.714	fig|6666666.59333.peg.612	fig|6666666.59338.peg.541	fig|6666666.59339.peg.5158	None	fig|6666666.59337.peg.35	fig|6666666.59342.peg.5572	fig|6666666.71181.peg.4573	fig|6666666.59344.peg.1842	fig|6666666.59345.peg.2170	fig|6666666.59341.peg.1363	fig|6666666.59347.peg.2864	fig|6666666.59346.peg.2489	fig|6666666.59348.peg.4777	fig|6666666.59349.peg.47	None	fig|6666666.59351.peg.3468	fig|6666666.59353.peg.905	fig|6666666.59355.peg.801	fig|6666666.59354.peg.365

3818	Phosphoribosylformylglycinamidine cyclo-ligase EC 6.3.3.1	fig|6666666.59329.peg.6330	fig|6666666.59330.peg.4182	None	fig|6666666.59332.peg.2303	fig|6666666.59334.peg.5007	fig|6666666.59335.peg.1708	fig|6666666.59336.peg.6175	fig|6666666.59333.peg.3286	fig|6666666.59338.peg.2054	fig|6666666.59339.peg.7243	None	fig|6666666.59337.peg.4486	fig|6666666.59342.peg.4571	fig|6666666.71181.peg.3274	fig|6666666.59344.peg.4030	fig|6666666.59345.peg.2167	fig|6666666.59341.peg.2361	fig|6666666.59347.peg.2861	fig|6666666.59346.peg.2492	fig|6666666.59348.peg.3531	fig|6666666.59349.peg.3026	fig|6666666.59350.peg.1978	fig|6666666.59351.peg.759	fig|6666666.59353.peg.4021	fig|6666666.59355.peg.3979	fig|6666666.59354.peg.3274

7548	Iron-sulfur cluster-binding protein	fig|6666666.59329.peg.701	fig|6666666.59330.peg.3554	None	fig|6666666.59332.peg.1974	fig|6666666.59334.peg.1639	fig|6666666.59335.peg.6191	fig|6666666.59336.peg.3757	fig|6666666.59333.peg.279	fig|6666666.59338.peg.3086	fig|6666666.59339.peg.8851	fig|6666666.59340.peg.105	fig|6666666.59337.peg.1776	None	fig|6666666.71181.peg.1695	fig|6666666.59344.peg.596	fig|6666666.59345.peg.5669	fig|6666666.59341.peg.3768	fig|6666666.59347.peg.5352	fig|6666666.59346.peg.3047	fig|6666666.59348.peg.1943	fig|6666666.59349.peg.3164	fig|6666666.59350.peg.875	fig|6666666.59351.peg.3088	fig|6666666.59353.peg.1065	fig|6666666.59355.peg.3552	fig|6666666.59354.peg.3718

1952	Ribonuclease HII EC 3.1.26.4	fig|6666666.59329.peg.4479	fig|6666666.59330.peg.454	fig|6666666.59331.peg.5347	fig|6666666.59332.peg.419	fig|6666666.59334.peg.1791	fig|6666666.59335.peg.2666	fig|6666666.59336.peg.2997	fig|6666666.59333.peg.4459	fig|6666666.59338.peg.2436	fig|6666666.59339.peg.5127	None	fig|6666666.59337.peg.1596	None	fig|6666666.71181.peg.21	fig|6666666.59344.peg.2668	fig|6666666.59345.peg.2187	fig|6666666.59341.peg.982	fig|6666666.59347.peg.4372	fig|6666666.59346.peg.4441	fig|6666666.59348.peg.3430	fig|6666666.59349.peg.1361	fig|6666666.59350.peg.725	fig|6666666.59351.peg.2129	fig|6666666.59353.peg.1999	fig|6666666.59355.peg.4104	fig|6666666.59354.peg.1411

1958	Lactam utilization protein LamB	fig|6666666.59329.peg.3375	fig|6666666.59330.peg.3708	fig|6666666.59331.peg.5562	fig|6666666.59332.peg.5639	fig|6666666.59334.peg.4240	fig|6666666.59335.peg.5836	fig|6666666.59336.peg.5313	fig|6666666.59333.peg.2695	fig|6666666.59338.peg.3024	fig|6666666.59339.peg.2855	None	fig|6666666.59337.peg.3037	fig|6666666.59342.peg.1984	fig|6666666.71181.peg.3226	fig|6666666.59344.peg.90	fig|6666666.59345.peg.92	fig|6666666.59341.peg.1567	fig|6666666.59347.peg.4397	fig|6666666.59346.peg.3632	fig|6666666.59348.peg.4486	fig|6666666.59349.peg.3995	None	fig|6666666.59351.peg.1262	fig|6666666.59353.peg.416	fig|6666666.59355.peg.2747	fig|6666666.59354.peg.547

8792	tRNA S 4 U 4-thiouridine synthase former ThiI Rhodanese-like domain required for thiamine synthesis	fig|6666666.59329.peg.444	fig|6666666.59330.peg.1049	fig|6666666.59331.peg.1845	fig|6666666.59332.peg.1967	fig|6666666.59334.peg.1922	fig|6666666.59335.peg.3893	fig|6666666.59336.peg.1870	fig|6666666.59333.peg.5011	fig|6666666.59338.peg.2742	fig|6666666.59339.peg.7167	None	fig|6666666.59337.peg.1127	fig|6666666.59342.peg.3657	fig|6666666.71181.peg.2035	fig|6666666.59344.peg.1087	fig|6666666.59345.peg.3530	fig|6666666.59341.peg.2523	fig|6666666.59347.peg.2873	fig|6666666.59346.peg.4091	fig|6666666.59348.peg.4021	fig|6666666.59349.peg.1764	None	fig|6666666.59351.peg.2292	fig|6666666.59353.peg.769	fig|6666666.59355.peg.935	fig|6666666.59354.peg.493

4579	Cell division protein FtsH EC 3.4.24.-	fig|6666666.59329.peg.3480	fig|6666666.59330.peg.113	None	fig|6666666.59332.peg.5066	fig|6666666.59334.peg.203	fig|6666666.59335.peg.2847	fig|6666666.59336.peg.225	fig|6666666.59333.peg.1490	fig|6666666.59338.peg.1498	fig|6666666.59339.peg.4859	None	fig|6666666.59337.peg.677	fig|6666666.59342.peg.2480	fig|6666666.71181.peg.4092	fig|6666666.59344.peg.2106	fig|6666666.59345.peg.2362	fig|6666666.59341.peg.3947	fig|6666666.59347.peg.3010	fig|6666666.59346.peg.292	fig|6666666.59348.peg.1541	fig|6666666.59349.peg.3697	fig|6666666.59350.peg.2404	fig|6666666.59351.peg.4939	fig|6666666.59353.peg.3139	fig|6666666.59355.peg.457	fig|6666666.59354.peg.1194

7027	Ribonuclease E inhibitor RraB	fig|6666666.59329.peg.2974	fig|6666666.59330.peg.66	None	fig|6666666.59332.peg.3193	fig|6666666.59334.peg.4128	fig|6666666.59335.peg.223	fig|6666666.59336.peg.1786	fig|6666666.59333.peg.2814	fig|6666666.59338.peg.128	fig|6666666.59339.peg.8179	fig|6666666.59340.peg.2318	fig|6666666.59337.peg.853	fig|6666666.59342.peg.6446	fig|6666666.71181.peg.3295	fig|6666666.59344.peg.3964	fig|6666666.59345.peg.4841	fig|6666666.59341.peg.2701	fig|6666666.59347.peg.3232	fig|6666666.59346.peg.1679	fig|6666666.59348.peg.553	fig|6666666.59349.peg.3783	None	fig|6666666.59351.peg.3390	fig|6666666.59353.peg.2435	fig|6666666.59355.peg.528	fig|6666666.59354.peg.26

7024	Putative oligoketide cyclase dehydratase or lipid transport protein YfjG	fig|6666666.59329.peg.3957	fig|6666666.59330.peg.2428	fig|6666666.59331.peg.1334	fig|6666666.59332.peg.378	None	fig|6666666.59335.peg.4163	fig|6666666.59336.peg.217	fig|6666666.59333.peg.2763	fig|6666666.59338.peg.1074	fig|6666666.59339.peg.7281	fig|6666666.59340.peg.2211	fig|6666666.59337.peg.5543	fig|6666666.59342.peg.5027	fig|6666666.71181.peg.1444	fig|6666666.59344.peg.1541	fig|6666666.59345.peg.3580	fig|6666666.59341.peg.5535	fig|6666666.59347.peg.4324	fig|6666666.59346.peg.695	fig|6666666.59348.peg.1842	fig|6666666.59349.peg.2635	None	fig|6666666.59351.peg.4917	fig|6666666.59353.peg.4359	fig|6666666.59355.peg.4484	fig|6666666.59354.peg.6253

5728	Allophanate hydrolase 2 subunit 1 EC 3.5.1.54	fig|6666666.59329.peg.3374	fig|6666666.59330.peg.3707	fig|6666666.59331.peg.6060	fig|6666666.59332.peg.5641	fig|6666666.59334.peg.5449	fig|6666666.59335.peg.5835	fig|6666666.59336.peg.6061	fig|6666666.59333.peg.2694	None	fig|6666666.59339.peg.2854	fig|6666666.59340.peg.1722	fig|6666666.59337.peg.3038	fig|6666666.59342.peg.1983	fig|6666666.71181.peg.3224	fig|6666666.59344.peg.87	fig|6666666.59345.peg.93	fig|6666666.59341.peg.1568	fig|6666666.59347.peg.4396	fig|6666666.59346.peg.3633	None	fig|6666666.59349.peg.2237	fig|6666666.59350.peg.2884	fig|6666666.59351.peg.888	fig|6666666.59353.peg.2076	fig|6666666.59355.peg.2749	fig|6666666.59354.peg.5972

7807	tRNA 5-methoxyuridine 34 synthase	fig|6666666.59329.peg.3556	fig|6666666.59330.peg.2482	fig|6666666.59331.peg.3080	fig|6666666.59332.peg.4761	fig|6666666.59334.peg.3030	fig|6666666.59335.peg.951	fig|6666666.59336.peg.2793	fig|6666666.59333.peg.1050	fig|6666666.59338.peg.4022	fig|6666666.59339.peg.335	None	fig|6666666.59337.peg.4544	fig|6666666.59342.peg.1782	fig|6666666.71181.peg.1913	fig|6666666.59344.peg.2526	fig|6666666.59345.peg.1521	fig|6666666.59341.peg.2248	fig|6666666.59347.peg.4192	fig|6666666.59346.peg.2772	fig|6666666.59348.peg.1589	fig|6666666.59349.peg.2671	None	fig|6666666.59351.peg.5166	fig|6666666.59353.peg.1496	fig|6666666.59355.peg.2047	fig|6666666.59354.peg.4641

1858	Arginine N-succinyltransferase EC 2.3.1.109	fig|6666666.59329.peg.10	fig|6666666.59330.peg.1449	fig|6666666.59331.peg.6189	fig|6666666.59332.peg.1850	fig|6666666.59334.peg.724	fig|6666666.59335.peg.486	fig|6666666.59336.peg.280	fig|6666666.59333.peg.309	fig|6666666.59338.peg.2646	fig|6666666.59339.peg.8227	fig|6666666.59340.peg.2243	fig|6666666.59337.peg.1801	None	fig|6666666.71181.peg.4430	fig|6666666.59344.peg.2608	fig|6666666.59345.peg.5572	fig|6666666.59341.peg.2762	fig|6666666.59347.peg.4955	fig|6666666.59346.peg.20	fig|6666666.59348.peg.402	fig|6666666.59349.peg.3804	None	fig|6666666.59351.peg.1576	fig|6666666.59353.peg.1565	fig|6666666.59355.peg.2113	fig|6666666.59354.peg.498

1853	Oligopeptidase A EC 3.4.24.70	fig|6666666.59329.peg.2295	fig|6666666.59330.peg.227	None	fig|6666666.59332.peg.2420	fig|6666666.59334.peg.1187	fig|6666666.59335.peg.102	fig|6666666.59336.peg.764	fig|6666666.59333.peg.682	fig|6666666.59338.peg.5066	fig|6666666.59339.peg.7645	fig|6666666.59340.peg.297	fig|6666666.59337.peg.945	None	fig|6666666.71181.peg.4855	fig|6666666.59344.peg.4958	fig|6666666.59345.peg.2730	fig|6666666.59341.peg.5564	fig|6666666.59347.peg.5203	fig|6666666.59346.peg.2808	fig|6666666.59348.peg.43	fig|6666666.59349.peg.4053	fig|6666666.59350.peg.3067	fig|6666666.59351.peg.3312	fig|6666666.59353.peg.4519	fig|6666666.59355.peg.4261	fig|6666666.59354.peg.1369

2176	Ferrous iron transport protein A	fig|6666666.59329.peg.145	fig|6666666.59330.peg.2782	fig|6666666.59331.peg.1506	fig|6666666.59332.peg.3925	fig|6666666.59334.peg.2840	fig|6666666.59335.peg.4151	fig|6666666.59336.peg.1732	fig|6666666.59333.peg.914	fig|6666666.59338.peg.3023	fig|6666666.59339.peg.2987	fig|6666666.59340.peg.3685	fig|6666666.59337.peg.4120	None	fig|6666666.71181.peg.4949	fig|6666666.59344.peg.6198	fig|6666666.59345.peg.4313	fig|6666666.59341.peg.5857	fig|6666666.59347.peg.4398	fig|6666666.59346.peg.4281	fig|6666666.59348.peg.2684	fig|6666666.59349.peg.2929	None	fig|6666666.59351.peg.3999	fig|6666666.59353.peg.2192	fig|6666666.59355.peg.1886	fig|6666666.59354.peg.6175

3268	Acetolactate synthase small subunit EC 2.2.1.6	None	fig|6666666.59330.peg.4323	None	fig|6666666.59332.peg.553	fig|6666666.59334.peg.3159	fig|6666666.59335.peg.1738	fig|6666666.59336.peg.967	fig|6666666.59333.peg.114	fig|6666666.59338.peg.545	fig|6666666.59339.peg.7337	fig|6666666.59340.peg.2266	fig|6666666.59337.peg.1407	fig|6666666.59342.peg.5108	fig|6666666.71181.peg.374	fig|6666666.59344.peg.1495	fig|6666666.59345.peg.3878	fig|6666666.59341.peg.1365	fig|6666666.59347.peg.3986	fig|6666666.59346.peg.804	fig|6666666.59348.peg.276	fig|6666666.59349.peg.438	fig|6666666.59350.peg.7740	fig|6666666.59351.peg.1605	fig|6666666.59353.peg.125	fig|6666666.59355.peg.735	fig|6666666.59354.peg.1102

2614	Glycerol-3-phosphate acyltransferase EC 2.3.1.15	fig|6666666.59329.peg.778	fig|6666666.59330.peg.1575	None	fig|6666666.59332.peg.563	fig|6666666.59334.peg.4302	fig|6666666.59335.peg.4045	fig|6666666.59336.peg.1369	fig|6666666.59333.peg.1958	fig|6666666.59338.peg.534	fig|6666666.59339.peg.8581	fig|6666666.59340.peg.3199	fig|6666666.59337.peg.1658	None	fig|6666666.71181.peg.2003	fig|6666666.59344.peg.2738	fig|6666666.59345.peg.5626	fig|6666666.59341.peg.5709	fig|6666666.59347.peg.5046	fig|6666666.59346.peg.1646	fig|6666666.59348.peg.2964	fig|6666666.59349.peg.1091	fig|6666666.59350.peg.3247	fig|6666666.59351.peg.4821	fig|6666666.59353.peg.3001	fig|6666666.59355.peg.2908	fig|6666666.59354.peg.1755

746	Uridylate kinase EC 2.7.4.-	fig|6666666.59329.peg.4319	fig|6666666.59330.peg.467	fig|6666666.59331.peg.3459	fig|6666666.59332.peg.1589	fig|6666666.59334.peg.5186	fig|6666666.59335.peg.165	fig|6666666.59336.peg.6240	fig|6666666.59333.peg.3059	None	fig|6666666.59339.peg.5110	None	fig|6666666.59337.peg.1583	fig|6666666.59342.peg.267	fig|6666666.71181.peg.33	fig|6666666.59344.peg.2655	fig|6666666.59345.peg.2199	fig|6666666.59341.peg.969	fig|6666666.59347.peg.4360	fig|6666666.59346.peg.2262	fig|6666666.59348.peg.4817	fig|6666666.59349.peg.1374	fig|6666666.59350.peg.1876	fig|6666666.59351.peg.2116	fig|6666666.59353.peg.1985	fig|6666666.59355.peg.4092	fig|6666666.59354.peg.1334

10874	Hypothetical lipoprotein YajG precursor	fig|6666666.59329.peg.1038	fig|6666666.59330.peg.2790	None	fig|6666666.59332.peg.860	fig|6666666.59334.peg.1275	fig|6666666.59335.peg.116	fig|6666666.59336.peg.4467	fig|6666666.59333.peg.1934	fig|6666666.59338.peg.2000	fig|6666666.59339.peg.5041	fig|6666666.59340.peg.1798	fig|6666666.59337.peg.2376	fig|6666666.59342.peg.2126	fig|6666666.71181.peg.2048	fig|6666666.59344.peg.1073	fig|6666666.59345.peg.2240	fig|6666666.59341.peg.5302	fig|6666666.59347.peg.2806	fig|6666666.59346.peg.2715	fig|6666666.59348.peg.4184	fig|6666666.59349.peg.329	None	fig|6666666.59351.peg.3936	fig|6666666.59353.peg.757	fig|6666666.59355.peg.947	fig|6666666.59354.peg.2164

3288	Na+ H+ antiporter, putative	fig|6666666.59329.peg.4517	fig|6666666.59330.peg.3917	None	fig|6666666.59332.peg.2534	fig|6666666.59334.peg.3252	fig|6666666.59335.peg.6184	fig|6666666.59336.peg.4140	fig|6666666.59333.peg.406	fig|6666666.59338.peg.2149	fig|6666666.59339.peg.8216	None	fig|6666666.59337.peg.3194	fig|6666666.59342.peg.5444	fig|6666666.71181.peg.3988	fig|6666666.59344.peg.2719	fig|6666666.59345.peg.4914	fig|6666666.59341.peg.1336	fig|6666666.59347.peg.4507	fig|6666666.59346.peg.1588	fig|6666666.59348.peg.959	fig|6666666.59349.peg.4672	fig|6666666.59350.peg.5196	fig|6666666.59351.peg.2241	fig|6666666.59353.peg.274	fig|6666666.59355.peg.2923	fig|6666666.59354.peg.4243

10498	Chemotaxis response - phosphatase CheZ	fig|6666666.59329.peg.207	fig|6666666.59330.peg.1887	fig|6666666.59331.peg.6335	fig|6666666.59332.peg.2451	None	fig|6666666.59335.peg.1239	fig|6666666.59336.peg.4766	fig|6666666.59333.peg.1687	fig|6666666.59338.peg.3653	fig|6666666.59339.peg.3014	fig|6666666.59340.peg.3160	fig|6666666.59337.peg.2979	fig|6666666.59342.peg.2737	fig|6666666.71181.peg.2242	fig|6666666.59344.peg.3021	fig|6666666.59345.peg.5324	fig|6666666.59341.peg.3499	fig|6666666.59347.peg.2672	fig|6666666.59346.peg.4526	fig|6666666.59348.peg.2413	fig|6666666.59349.peg.192	None	fig|6666666.59351.peg.2067	fig|6666666.59353.peg.3539	fig|6666666.59355.peg.3636	fig|6666666.59354.peg.687

1265	FIG065221 Holliday junction DNA helicase	fig|6666666.59329.peg.3406	fig|6666666.59330.peg.1929	fig|6666666.59331.peg.751	fig|6666666.59332.peg.4219	fig|6666666.59334.peg.2997	fig|6666666.59335.peg.3961	fig|6666666.59336.peg.2309	fig|6666666.59333.peg.2041	None	fig|6666666.59339.peg.3134	fig|6666666.59340.peg.1129	fig|6666666.59337.peg.4593	fig|6666666.59342.peg.2395	fig|6666666.71181.peg.3156	fig|6666666.59344.peg.2818	fig|6666666.59345.peg.1336	fig|6666666.59341.peg.63	fig|6666666.59347.peg.2393	fig|6666666.59346.peg.1576	None	fig|6666666.59349.peg.1585	fig|6666666.59350.peg.1558	fig|6666666.59351.peg.1002	fig|6666666.59353.peg.1632	fig|6666666.59355.peg.1088	fig|6666666.59354.peg.2188

7614	tRNA 5-methylaminomethyl-2-thiouridine synthase TusD	fig|6666666.59329.peg.3694	fig|6666666.59330.peg.4074	fig|6666666.59331.peg.5469	fig|6666666.59332.peg.1075	fig|6666666.59334.peg.439	None	fig|6666666.59336.peg.2181	fig|6666666.59333.peg.3105	fig|6666666.59338.peg.3248	fig|6666666.59339.peg.8871	fig|6666666.59340.peg.943	fig|6666666.59337.peg.1821	fig|6666666.59342.peg.1920	fig|6666666.71181.peg.4310	fig|6666666.59344.peg.3129	fig|6666666.59345.peg.5554	fig|6666666.59341.peg.5087	fig|6666666.59347.peg.5334	fig|6666666.59346.peg.43	fig|6666666.59348.peg.3180	fig|6666666.59349.peg.4842	None	fig|6666666.59351.peg.3131	fig|6666666.59353.peg.2271	fig|6666666.59355.peg.1964	fig|6666666.59354.peg.5880

5979	FIG001590 Putative conserved exported protein precursor	fig|6666666.59329.peg.239	fig|6666666.59330.peg.369	fig|6666666.59331.peg.4588	fig|6666666.59332.peg.541	fig|6666666.59334.peg.3738	fig|6666666.59335.peg.1730	fig|6666666.59336.peg.5216	fig|6666666.59333.peg.2752	fig|6666666.59338.peg.1532	fig|6666666.59339.peg.7347	None	fig|6666666.59337.peg.550	fig|6666666.59342.peg.5360	fig|6666666.71181.peg.3130	fig|6666666.59344.peg.4739	fig|6666666.59345.peg.5039	fig|6666666.59341.peg.3935	fig|6666666.59347.peg.3193	fig|6666666.59346.peg.318	fig|6666666.59348.peg.271	fig|6666666.59349.peg.4325	None	fig|6666666.59351.peg.2194	fig|6666666.59353.peg.4437	fig|6666666.59355.peg.4517	fig|6666666.59354.peg.2856

2634	membrane protein	None	fig|6666666.59330.peg.1385	fig|6666666.59331.peg.2072	fig|6666666.59332.peg.2852	fig|6666666.59334.peg.1075	fig|6666666.59335.peg.3371	fig|6666666.59336.peg.429	fig|6666666.59333.peg.738	fig|6666666.59338.peg.1340	fig|6666666.59339.peg.3112	fig|6666666.59340.peg.616	fig|6666666.59337.peg.2879	fig|6666666.59342.peg.1799	fig|6666666.71181.peg.4195	fig|6666666.59344.peg.487	fig|6666666.59345.peg.1034	fig|6666666.59341.peg.49	fig|6666666.59347.peg.2405	fig|6666666.59346.peg.5065	fig|6666666.59348.peg.833	fig|6666666.59349.peg.375	None	fig|6666666.59351.peg.944	fig|6666666.59353.peg.1804	fig|6666666.59355.peg.1212	fig|6666666.59354.peg.4426

2962	SSU ribosomal protein S21p	fig|6666666.59329.peg.682	fig|6666666.59330.peg.3130	fig|6666666.59331.peg.4679	fig|6666666.59332.peg.1513	fig|6666666.59334.peg.2466	fig|6666666.59335.peg.1645	fig|6666666.59336.peg.5192	fig|6666666.59333.peg.173	fig|6666666.59338.peg.693	fig|6666666.59339.peg.7907	None	fig|6666666.59337.peg.1425	fig|6666666.59342.peg.1638	fig|6666666.71181.peg.3007	fig|6666666.59344.peg.4476	fig|6666666.59345.peg.3847	fig|6666666.59341.peg.3721	fig|6666666.59347.peg.3137	fig|6666666.59346.peg.2560	fig|6666666.59348.peg.2746	fig|6666666.59349.peg.4852	fig|6666666.59350.peg.5898	fig|6666666.59351.peg.2368	None	fig|6666666.59355.peg.1687	fig|6666666.59354.peg.16

5375	Outer membrane lipoprotein carrier protein LolA	fig|6666666.59329.peg.3407	fig|6666666.59330.peg.1928	None	fig|6666666.59332.peg.4220	fig|6666666.59334.peg.2808	fig|6666666.59335.peg.3960	fig|6666666.59336.peg.2308	fig|6666666.59333.peg.2040	fig|6666666.59338.peg.2185	fig|6666666.59339.peg.3133	fig|6666666.59340.peg.1130	fig|6666666.59337.peg.4592	fig|6666666.59342.peg.2396	fig|6666666.71181.peg.3155	fig|6666666.59344.peg.2819	fig|6666666.59345.peg.1337	fig|6666666.59341.peg.62	fig|6666666.59347.peg.2394	fig|6666666.59346.peg.1577	fig|6666666.59348.peg.2974	fig|6666666.59349.peg.1584	None	fig|6666666.59351.peg.1001	fig|6666666.59353.peg.1631	fig|6666666.59355.peg.1089	fig|6666666.59354.peg.2187

2138	Z-ring-associated protein ZapA	fig|6666666.59329.peg.240	fig|6666666.59330.peg.368	None	fig|6666666.59332.peg.540	fig|6666666.59334.peg.1049	fig|6666666.59335.peg.1731	fig|6666666.59336.peg.5215	fig|6666666.59333.peg.2753	fig|6666666.59338.peg.1533	fig|6666666.59339.peg.7346	fig|6666666.59340.peg.3598	fig|6666666.59337.peg.549	fig|6666666.59342.peg.5359	fig|6666666.71181.peg.3131	fig|6666666.59344.peg.4738	fig|6666666.59345.peg.5038	fig|6666666.59341.peg.3933	fig|6666666.59347.peg.3194	fig|6666666.59346.peg.317	fig|6666666.59348.peg.272	fig|6666666.59349.peg.1785	None	fig|6666666.59351.peg.2312	fig|6666666.59353.peg.4436	fig|6666666.59355.peg.4516	fig|6666666.59354.peg.3182

11250	YaeQ protein	fig|6666666.59329.peg.4564	fig|6666666.59330.peg.935	fig|6666666.59331.peg.1318	fig|6666666.59332.peg.2306	fig|6666666.59334.peg.4142	fig|6666666.59335.peg.4225	fig|6666666.59336.peg.4181	fig|6666666.59333.peg.4455	fig|6666666.59338.peg.3821	fig|6666666.59339.peg.6515	None	fig|6666666.59337.peg.3966	fig|6666666.59342.peg.1858	fig|6666666.71181.peg.12	fig|6666666.59344.peg.2678	fig|6666666.59345.peg.463	fig|6666666.59341.peg.3440	fig|6666666.59347.peg.1343	fig|6666666.59346.peg.1939	fig|6666666.59348.peg.520	fig|6666666.59349.peg.387	None	fig|6666666.59351.peg.2374	fig|6666666.59353.peg.2008	fig|6666666.59355.peg.4113	fig|6666666.59354.peg.5025

6848	tRNA Cm32 Um32 methyltransferase	fig|6666666.59329.peg.1114	fig|6666666.59330.peg.1412	None	fig|6666666.59332.peg.1433	fig|6666666.59334.peg.207	fig|6666666.59335.peg.2364	fig|6666666.59336.peg.665	fig|6666666.59333.peg.922	fig|6666666.59338.peg.1328	fig|6666666.59339.peg.6787	fig|6666666.59340.peg.702	fig|6666666.59337.peg.737	None	fig|6666666.71181.peg.1755	fig|6666666.59344.peg.1625	fig|6666666.59345.peg.3654	fig|6666666.59341.peg.4634	fig|6666666.59347.peg.4281	fig|6666666.59346.peg.2742	fig|6666666.59348.peg.1381	fig|6666666.59349.peg.2437	fig|6666666.59350.peg.134	fig|6666666.59351.peg.2715	fig|6666666.59353.peg.2692	fig|6666666.59355.peg.1787	fig|6666666.59354.peg.6369

10628	FIG01200142 hypothetical protein	fig|6666666.59329.peg.4280	fig|6666666.59330.peg.480	fig|6666666.59331.peg.2595	fig|6666666.59332.peg.979	None	fig|6666666.59335.peg.3073	fig|6666666.59336.peg.5749	fig|6666666.59333.peg.2229	fig|6666666.59338.peg.4164	fig|6666666.59339.peg.5744	fig|6666666.59340.peg.1142	fig|6666666.59337.peg.1570	None	fig|6666666.71181.peg.4470	fig|6666666.59344.peg.1982	fig|6666666.59345.peg.2212	fig|6666666.59341.peg.2604	fig|6666666.59347.peg.2777	fig|6666666.59346.peg.3525	fig|6666666.59348.peg.1415	fig|6666666.59349.peg.682	fig|6666666.59350.peg.316	fig|6666666.59351.peg.4019	fig|6666666.59353.peg.2938	fig|6666666.59355.peg.2801	fig|6666666.59354.peg.5208

1025	C1451 Predicted metal-dependent hydrolase	fig|6666666.59329.peg.5287	fig|6666666.59330.peg.4648	fig|6666666.59331.peg.1261	fig|6666666.59332.peg.816	fig|6666666.59334.peg.1728	fig|6666666.59335.peg.2007	fig|6666666.59336.peg.5955	fig|6666666.59333.peg.5386	fig|6666666.59338.peg.5850	fig|6666666.59339.peg.3944	fig|6666666.59340.peg.2363	fig|6666666.59337.peg.2001	None	fig|6666666.71181.peg.4843	fig|6666666.59344.peg.5650	fig|6666666.59345.peg.5590	fig|6666666.59341.peg.721	fig|6666666.59347.peg.3448	fig|6666666.59346.peg.5073	fig|6666666.59348.peg.1727	fig|6666666.59349.peg.3870	None	fig|6666666.59351.peg.648	fig|6666666.59353.peg.2397	fig|6666666.59355.peg.1670	fig|6666666.59354.peg.5533

1249	Pantothenate kinase EC 2.7.1.33	fig|6666666.59329.peg.2429	fig|6666666.59330.peg.4556	None	fig|6666666.59332.peg.3162	fig|6666666.59334.peg.2123	fig|6666666.59335.peg.3561	fig|6666666.59336.peg.4602	fig|6666666.59333.peg.5075	fig|6666666.59338.peg.5903	fig|6666666.59339.peg.8677	fig|6666666.59340.peg.3082	fig|6666666.59337.peg.1945	fig|6666666.59342.peg.4952	fig|6666666.71181.peg.4964	fig|6666666.59344.peg.6195	fig|6666666.59345.peg.5710	fig|6666666.59341.peg.4099	fig|6666666.59347.peg.119	fig|6666666.59346.peg.4268	None	fig|6666666.59349.peg.4456	fig|6666666.59350.peg.430	fig|6666666.59351.peg.2794	fig|6666666.59353.peg.5082	fig|6666666.59355.peg.5134	fig|6666666.59354.peg.5096

5346	Phosphate acetyltransferase EC 2.3.1.8	fig|6666666.59329.peg.843	fig|6666666.59330.peg.4363	None	fig|6666666.59332.peg.1742	fig|6666666.59334.peg.1651	fig|6666666.59335.peg.1293	fig|6666666.59336.peg.2937	fig|6666666.59333.peg.1319	fig|6666666.59338.peg.3149	fig|6666666.59339.peg.3421	fig|6666666.59340.peg.3092	fig|6666666.59337.peg.4929	fig|6666666.59342.peg.801	fig|6666666.71181.peg.553	fig|6666666.59344.peg.1913	fig|6666666.59345.peg.5167	fig|6666666.59341.peg.3137	fig|6666666.59347.peg.2635	fig|6666666.59346.peg.546	None	fig|6666666.59349.peg.2952	fig|6666666.59350.peg.1336	fig|6666666.59351.peg.2821	fig|6666666.59353.peg.1183	fig|6666666.59355.peg.292	fig|6666666.59354.peg.4253

2658	Prolipoprotein diacylglyceryl transferase EC 2.4.99.-	fig|6666666.59329.peg.1936	fig|6666666.59330.peg.4673	None	fig|6666666.59332.peg.4745	fig|6666666.59334.peg.1745	fig|6666666.59335.peg.1019	fig|6666666.59336.peg.2362	fig|6666666.59333.peg.3948	fig|6666666.59338.peg.4191	fig|6666666.59339.peg.7454	None	fig|6666666.59337.peg.125	fig|6666666.59342.peg.2680	fig|6666666.71181.peg.71	fig|6666666.59344.peg.3196	fig|6666666.59345.peg.3718	fig|6666666.59341.peg.5242	fig|6666666.59347.peg.3031	fig|6666666.59346.peg.3088	fig|6666666.59348.peg.4711	fig|6666666.59349.peg.3877	fig|6666666.59350.peg.7764	fig|6666666.59351.peg.2581	fig|6666666.59353.peg.1944	fig|6666666.59355.peg.1463	fig|6666666.59354.peg.4999

7381	Phosphoserine phosphatase EC 3.1.3.3	fig|6666666.59329.peg.1049	fig|6666666.59330.peg.78	None	fig|6666666.59332.peg.4157	fig|6666666.59334.peg.489	fig|6666666.59335.peg.3646	fig|6666666.59336.peg.3738	fig|6666666.59333.peg.2565	fig|6666666.59338.peg.3781	fig|6666666.59339.peg.5025	fig|6666666.59340.peg.129	fig|6666666.59337.peg.1309	None	fig|6666666.71181.peg.454	fig|6666666.59344.peg.1367	fig|6666666.59345.peg.2330	fig|6666666.59341.peg.4734	fig|6666666.59347.peg.2966	fig|6666666.59346.peg.2453	fig|6666666.59348.peg.4661	fig|6666666.59349.peg.4661	fig|6666666.59350.peg.198	fig|6666666.59351.peg.2688	fig|6666666.59353.peg.42	fig|6666666.59355.peg.650	fig|6666666.59354.peg.762

702	Cytochrome c heme lyase subunit CcmH	fig|6666666.59329.peg.2322	fig|6666666.59330.peg.4679	fig|6666666.59331.peg.5096	fig|6666666.59332.peg.5464	fig|6666666.59334.peg.2354	fig|6666666.59335.peg.1254	fig|6666666.59336.peg.5503	fig|6666666.59333.peg.2600	fig|6666666.59338.peg.2230	fig|6666666.59339.peg.3036	None	fig|6666666.59337.peg.4227	fig|6666666.59342.peg.5729	fig|6666666.71181.peg.1523	fig|6666666.59344.peg.4986	fig|6666666.59345.peg.5307	fig|6666666.59341.peg.3749	fig|6666666.59347.peg.5097	fig|6666666.59346.peg.2158	None	fig|6666666.59349.peg.175	fig|6666666.59350.peg.3588	fig|6666666.59351.peg.2091	fig|6666666.59353.peg.4873	fig|6666666.59355.peg.219	fig|6666666.59354.peg.1183

5316	Putative Holliday junction resolvase YggF	fig|6666666.59329.peg.1770	fig|6666666.59330.peg.345	fig|6666666.59331.peg.4804	fig|6666666.59332.peg.1942	fig|6666666.59334.peg.2564	fig|6666666.59335.peg.3093	fig|6666666.59336.peg.3073	fig|6666666.59333.peg.771	fig|6666666.59338.peg.447	fig|6666666.59339.peg.7833	None	fig|6666666.59337.peg.526	None	fig|6666666.71181.peg.1863	fig|6666666.59344.peg.4337	fig|6666666.59345.peg.5016	fig|6666666.59341.peg.2572	fig|6666666.59347.peg.3215	fig|6666666.59346.peg.1784	fig|6666666.59348.peg.353	fig|6666666.59349.peg.805	fig|6666666.59350.peg.3587	fig|6666666.59351.peg.3534	fig|6666666.59353.peg.3228	fig|6666666.59355.peg.3110	fig|6666666.59354.peg.518

8575	Acetolactate synthase small subunit EC 2.2.1.6	fig|6666666.59329.peg.1487	fig|6666666.59330.peg.623	fig|6666666.59331.peg.1210	fig|6666666.59332.peg.209	fig|6666666.59334.peg.2	fig|6666666.59335.peg.4366	fig|6666666.59336.peg.4450	fig|6666666.59333.peg.11	fig|6666666.59338.peg.4881	fig|6666666.59339.peg.8065	fig|6666666.59340.peg.384	fig|6666666.59337.peg.907	None	fig|6666666.71181.peg.3090	fig|6666666.59344.peg.4229	fig|6666666.59345.peg.4946	fig|6666666.59341.peg.5897	fig|6666666.59347.peg.4766	fig|6666666.59346.peg.1419	fig|6666666.59348.peg.70	fig|6666666.59349.peg.1125	None	fig|6666666.59351.peg.80	fig|6666666.59353.peg.4267	fig|6666666.59355.peg.4329	fig|6666666.59354.peg.297

6627	Flagellar motor switch protein FliN	fig|6666666.59329.peg.3931	fig|6666666.59330.peg.1876	None	fig|6666666.59332.peg.4085	None	fig|6666666.59335.peg.2404	fig|6666666.59336.peg.1273	fig|6666666.59333.peg.4568	fig|6666666.59338.peg.3099	fig|6666666.59339.peg.110	fig|6666666.59340.peg.1580	fig|6666666.59337.peg.2967	fig|6666666.59342.peg.2650	fig|6666666.71181.peg.4935	fig|6666666.59344.peg.3053	fig|6666666.59345.peg.2865	fig|6666666.59341.peg.3274	fig|6666666.59347.peg.4623	fig|6666666.59346.peg.2887	fig|6666666.59348.peg.3696	fig|6666666.59349.peg.203	fig|6666666.59350.peg.6523	fig|6666666.59351.peg.2054	fig|6666666.59353.peg.3511	fig|6666666.59355.peg.3665	fig|6666666.59354.peg.672

6826	Flagellar hook-associated protein FlgK	fig|6666666.59329.peg.2688	fig|6666666.59330.peg.2678	fig|6666666.59331.peg.2437	fig|6666666.59332.peg.2244	None	fig|6666666.59335.peg.908	fig|6666666.59336.peg.2921	fig|6666666.59333.peg.3844	fig|6666666.59338.peg.1247	fig|6666666.59339.peg.5325	fig|6666666.59340.peg.3579	fig|6666666.59337.peg.3190	fig|6666666.59342.peg.2920	fig|6666666.71181.peg.2221	fig|6666666.59344.peg.3045	fig|6666666.59345.peg.5459	fig|6666666.59341.peg.113	fig|6666666.59347.peg.4602	fig|6666666.59346.peg.1771	fig|6666666.59348.peg.5107	fig|6666666.59349.peg.733	None	fig|6666666.59351.peg.1992	fig|6666666.59353.peg.3518	fig|6666666.59355.peg.3658	fig|6666666.59354.peg.5261

10548	Ferric siderophore transport system, periplasmic binding protein TonB	fig|6666666.59329.peg.1282	fig|6666666.59330.peg.4176	fig|6666666.59331.peg.2231	fig|6666666.59332.peg.1149	fig|6666666.59334.peg.4101	fig|6666666.59335.peg.4623	fig|6666666.59336.peg.3050	fig|6666666.59333.peg.1257	fig|6666666.59338.peg.660	fig|6666666.59339.peg.4293	None	fig|6666666.59337.peg.2456	fig|6666666.59342.peg.3436	fig|6666666.71181.peg.2783	fig|6666666.59344.peg.445	fig|6666666.59345.peg.3049	fig|6666666.59341.peg.1148	fig|6666666.59347.peg.2517	fig|6666666.59346.peg.4292	fig|6666666.59348.peg.441	fig|6666666.59349.peg.431	None	fig|6666666.59351.peg.1691	fig|6666666.59353.peg.2492	fig|6666666.59355.peg.586	fig|6666666.59354.peg.1268

8749	DNA gyrase subunit B EC 5.99.1.3	fig|6666666.59329.peg.6137	fig|6666666.59330.peg.1098	fig|6666666.59331.peg.3916	fig|6666666.59332.peg.1427	fig|6666666.59334.peg.394	fig|6666666.59335.peg.3930	fig|6666666.59336.peg.6	fig|6666666.59333.peg.628	fig|6666666.59338.peg.4582	fig|6666666.59339.peg.4929	None	fig|6666666.59337.peg.20	None	fig|6666666.71181.peg.5075	fig|6666666.59344.peg.4174	fig|6666666.59345.peg.3821	fig|6666666.59341.peg.1039	fig|6666666.59347.peg.4801	fig|6666666.59346.peg.1198	fig|6666666.59348.peg.4316	fig|6666666.59349.peg.1004	fig|6666666.59350.peg.6666	fig|6666666.59351.peg.5031	fig|6666666.59353.peg.3054	fig|6666666.59355.peg.4968	fig|6666666.59354.peg.666

7654	Glycogen synthase, ADP-glucose transglucosylase EC 2.4.1.21	fig|6666666.59329.peg.2638	fig|6666666.59330.peg.1330	fig|6666666.59331.peg.4999	fig|6666666.59332.peg.2503	fig|6666666.59334.peg.1449	fig|6666666.59335.peg.927	fig|6666666.59336.peg.3897	fig|6666666.59333.peg.2548	fig|6666666.59338.peg.3809	fig|6666666.59339.peg.301	fig|6666666.59340.peg.2140	fig|6666666.59337.peg.5388	fig|6666666.59342.peg.130	fig|6666666.71181.peg.1116	fig|6666666.59344.peg.3624	fig|6666666.59345.peg.1544	fig|6666666.59341.peg.1009	fig|6666666.59347.peg.2384	fig|6666666.59346.peg.2534	None	fig|6666666.59349.peg.958	None	fig|6666666.59351.peg.1588	fig|6666666.59353.peg.2175	fig|6666666.59355.peg.1871	fig|6666666.59354.peg.780

9263	ADP compounds hydrolase NudE EC 3.6.1.-	fig|6666666.59329.peg.1236	fig|6666666.59330.peg.429	fig|6666666.59331.peg.5259	fig|6666666.59332.peg.2472	fig|6666666.59334.peg.114	fig|6666666.59335.peg.695	fig|6666666.59336.peg.4431	fig|6666666.59333.peg.518	fig|6666666.59338.peg.1426	fig|6666666.59339.peg.6236	None	fig|6666666.59337.peg.4257	fig|6666666.59342.peg.3595	fig|6666666.71181.peg.1839	fig|6666666.59344.peg.5471	fig|6666666.59345.peg.2665	fig|6666666.59341.peg.1412	fig|6666666.59347.peg.4475	fig|6666666.59346.peg.2233	fig|6666666.59348.peg.1405	fig|6666666.59349.peg.2287	None	fig|6666666.59351.peg.4168	fig|6666666.59353.peg.2211	fig|6666666.59355.peg.1903	fig|6666666.59354.peg.6226

9081	Cytochrome c-type biogenesis protein CcmD, interacts with CcmCE	fig|6666666.59329.peg.2317	fig|6666666.59330.peg.4378	fig|6666666.59331.peg.3838	fig|6666666.59332.peg.2661	fig|6666666.59334.peg.2182	fig|6666666.59335.peg.1249	fig|6666666.59336.peg.4940	fig|6666666.59333.peg.2594	fig|6666666.59338.peg.3957	fig|6666666.59339.peg.3028	None	fig|6666666.59337.peg.4222	fig|6666666.59342.peg.4686	fig|6666666.71181.peg.1528	fig|6666666.59344.peg.4979	fig|6666666.59345.peg.5313	fig|6666666.59341.peg.3755	fig|6666666.59347.peg.5092	fig|6666666.59346.peg.5098	fig|6666666.59348.peg.4628	fig|6666666.59349.peg.180	None	fig|6666666.59351.peg.2085	fig|6666666.59353.peg.4868	fig|6666666.59355.peg.214	fig|6666666.59354.peg.5195

8009	Cytochrome B561	fig|6666666.59329.peg.590	fig|6666666.59330.peg.4718	fig|6666666.59331.peg.6362	fig|6666666.59332.peg.3516	fig|6666666.59334.peg.3071	fig|6666666.59335.peg.2349	fig|6666666.59336.peg.5110	fig|6666666.59333.peg.3746	fig|6666666.59338.peg.673	fig|6666666.59339.peg.6506	None	fig|6666666.59337.peg.2514	fig|6666666.59342.peg.1814	fig|6666666.71181.peg.946	fig|6666666.59344.peg.3550	fig|6666666.59345.peg.662	fig|6666666.59341.peg.4254	fig|6666666.59347.peg.867	fig|6666666.59346.peg.2905	fig|6666666.59348.peg.4201	fig|6666666.59349.peg.3191	fig|6666666.59350.peg.4942	None	fig|6666666.59353.peg.868	fig|6666666.59355.peg.836	fig|6666666.59354.peg.5265

8007	Microsomal dipeptidase EC 3.4.13.19	fig|6666666.59329.peg.3569	fig|6666666.59330.peg.2627	fig|6666666.59331.peg.3870	fig|6666666.59332.peg.3705	fig|6666666.59334.peg.2727	fig|6666666.59335.peg.3217	fig|6666666.59336.peg.5241	fig|6666666.59333.peg.1405	fig|6666666.59338.peg.615	fig|6666666.59339.peg.790	None	fig|6666666.59337.peg.1482	fig|6666666.59342.peg.2767	fig|6666666.71181.peg.1287	fig|6666666.59344.peg.2970	fig|6666666.59345.peg.361	fig|6666666.59341.peg.4197	None	fig|6666666.59346.peg.2644	fig|6666666.59348.peg.4780	fig|6666666.59349.peg.3383	fig|6666666.59350.peg.387	fig|6666666.59351.peg.4171	fig|6666666.59353.peg.3312	fig|6666666.59355.peg.3140	fig|6666666.59354.peg.5376

7461	Na + -translocating NADH-quinone reductase subunit C EC 1.6.5.-	fig|6666666.59329.peg.4957	fig|6666666.59330.peg.2784	fig|6666666.59331.peg.434	fig|6666666.59332.peg.2204	fig|6666666.59334.peg.883	fig|6666666.59335.peg.121	fig|6666666.59336.peg.976	fig|6666666.59333.peg.1800	fig|6666666.59338.peg.4865	fig|6666666.59339.peg.5048	None	fig|6666666.59337.peg.2381	fig|6666666.59342.peg.2978	fig|6666666.71181.peg.917	fig|6666666.59344.peg.5170	fig|6666666.59345.peg.2235	fig|6666666.59341.peg.2293	fig|6666666.59347.peg.2800	fig|6666666.59346.peg.2722	fig|6666666.59348.peg.1314	fig|6666666.59349.peg.305	fig|6666666.59350.peg.4835	fig|6666666.59351.peg.3929	fig|6666666.59353.peg.900	fig|6666666.59355.peg.806	None

4934	Tail-specific protease precursor EC 3.4.21.102	fig|6666666.59329.peg.4719	fig|6666666.59330.peg.1012	None	fig|6666666.59332.peg.874	fig|6666666.59334.peg.928	fig|6666666.59335.peg.3354	fig|6666666.59336.peg.4555	fig|6666666.59333.peg.2668	fig|6666666.59338.peg.4701	fig|6666666.59339.peg.1536	None	fig|6666666.59337.peg.3154	fig|6666666.59342.peg.785	fig|6666666.71181.peg.3836	fig|6666666.59344.peg.845	fig|6666666.59345.peg.3261	fig|6666666.59341.peg.4221	fig|6666666.59347.peg.1774	fig|6666666.59346.peg.3400	fig|6666666.59348.peg.1997	fig|6666666.59349.peg.2484	fig|6666666.59350.peg.492	fig|6666666.59351.peg.370	fig|6666666.59353.peg.1270	fig|6666666.59355.peg.3313	fig|6666666.59354.peg.59

4732	Uncharacterized protein conserved in bacteria	fig|6666666.59329.peg.3313	fig|6666666.59330.peg.1480	fig|6666666.59331.peg.2259	fig|6666666.59332.peg.2458	None	fig|6666666.59335.peg.737	fig|6666666.59336.peg.3771	fig|6666666.59333.peg.1223	fig|6666666.59338.peg.5521	fig|6666666.59339.peg.5617	fig|6666666.59340.peg.2995	fig|6666666.59337.peg.197	fig|6666666.59342.peg.4001	fig|6666666.71181.peg.4866	fig|6666666.59344.peg.4235	fig|6666666.59345.peg.4526	fig|6666666.59341.peg.2342	fig|6666666.59347.peg.978	fig|6666666.59346.peg.2592	fig|6666666.59348.peg.1846	fig|6666666.59349.peg.2409	None	fig|6666666.59351.peg.400	fig|6666666.59353.peg.4272	fig|6666666.59355.peg.4324	fig|6666666.59354.peg.5753

7513	Thiamine-monophosphate kinase EC 2.7.4.16	fig|6666666.59329.peg.437	fig|6666666.59330.peg.1056	fig|6666666.59331.peg.5989	fig|6666666.59332.peg.4206	fig|6666666.59334.peg.2074	fig|6666666.59335.peg.156	fig|6666666.59336.peg.3171	fig|6666666.59333.peg.5217	fig|6666666.59338.peg.2803	fig|6666666.59339.peg.5098	fig|6666666.59340.peg.961	fig|6666666.59337.peg.1136	None	fig|6666666.71181.peg.2725	fig|6666666.59344.peg.1097	fig|6666666.59345.peg.3537	fig|6666666.59341.peg.2619	fig|6666666.59347.peg.2765	fig|6666666.59346.peg.3385	fig|6666666.59348.peg.928	fig|6666666.59349.peg.3486	None	fig|6666666.59351.peg.4246	fig|6666666.59353.peg.775	fig|6666666.59355.peg.928	fig|6666666.59354.peg.6446

5621	3R -hydroxymyristoyl-[acyl carrier protein] dehydratase EC 4.2.1.-	None	fig|6666666.59330.peg.457	None	fig|6666666.59332.peg.414	fig|6666666.59334.peg.1794	fig|6666666.59335.peg.2663	fig|6666666.59336.peg.3000	fig|6666666.59333.peg.4462	fig|6666666.59338.peg.2456	fig|6666666.59339.peg.5124	fig|6666666.59340.peg.736	fig|6666666.59337.peg.1593	fig|6666666.59342.peg.279	fig|6666666.71181.peg.24	fig|6666666.59344.peg.2665	fig|6666666.59345.peg.2190	fig|6666666.59341.peg.979	fig|6666666.59347.peg.4369	fig|6666666.59346.peg.4921	fig|6666666.59348.peg.3426	fig|6666666.59349.peg.1364	fig|6666666.59350.peg.1090	fig|6666666.59351.peg.2126	fig|6666666.59353.peg.1996	fig|6666666.59355.peg.3443	fig|6666666.59354.peg.3636

9240	FIG01045360 hypothetical protein	fig|6666666.59329.peg.1898	fig|6666666.59330.peg.1764	fig|6666666.59331.peg.1621	fig|6666666.59332.peg.2842	fig|6666666.59334.peg.898	fig|6666666.59335.peg.1522	fig|6666666.59336.peg.566	fig|6666666.59333.peg.322	fig|6666666.59338.peg.1976	fig|6666666.59339.peg.4787	fig|6666666.59340.peg.169	fig|6666666.59337.peg.4883	None	fig|6666666.71181.peg.2629	fig|6666666.59344.peg.3898	fig|6666666.59345.peg.2399	fig|6666666.59341.peg.3967	fig|6666666.59347.peg.3973	fig|6666666.59346.peg.4050	fig|6666666.59348.peg.2608	fig|6666666.59349.peg.3840	None	fig|6666666.59351.peg.2137	fig|6666666.59353.peg.1739	fig|6666666.59355.peg.1274	fig|6666666.59354.peg.1550

9242	Adenylosuccinate lyase EC 4.3.2.2	fig|6666666.59329.peg.6294	fig|6666666.59330.peg.2871	fig|6666666.59331.peg.3303	fig|6666666.59332.peg.4306	fig|6666666.59334.peg.1445	fig|6666666.59335.peg.602	fig|6666666.59336.peg.3109	fig|6666666.59333.peg.4984	fig|6666666.59338.peg.404	fig|6666666.59339.peg.479	fig|6666666.59340.peg.3034	fig|6666666.59337.peg.5581	None	fig|6666666.71181.peg.3384	fig|6666666.59344.peg.159	fig|6666666.59345.peg.1316	fig|6666666.59341.peg.488	fig|6666666.59347.peg.4131	fig|6666666.59346.peg.1564	None	fig|6666666.59349.peg.2886	fig|6666666.59350.peg.549	fig|6666666.59351.peg.870	fig|6666666.59353.peg.470	fig|6666666.59355.peg.2195	fig|6666666.59354.peg.3065

3698	Permease of the major facilitator superfamily	None	fig|6666666.59330.peg.4102	fig|6666666.59331.peg.5205	fig|6666666.59332.peg.1606	fig|6666666.59334.peg.402	fig|6666666.59335.peg.3470	fig|6666666.59336.peg.2017	fig|6666666.59333.peg.827	fig|6666666.59338.peg.1374	fig|6666666.59339.peg.6730	fig|6666666.59340.peg.299	None	fig|6666666.59342.peg.5685	fig|6666666.71181.peg.765	fig|6666666.59344.peg.4891	fig|6666666.59345.peg.1723	fig|6666666.59341.peg.1382	fig|6666666.59347.peg.20	fig|6666666.59346.peg.5138	fig|6666666.59348.peg.3074	fig|6666666.59349.peg.1517	fig|6666666.59350.peg.517	fig|6666666.59351.peg.2165	fig|6666666.59353.peg.936	fig|6666666.59355.peg.4202	fig|6666666.59354.peg.3778

5877	Ribonucleotide reductase of class Ia aerobic , beta subunit EC 1.17.4.1	fig|6666666.59329.peg.6203	fig|6666666.59330.peg.2320	fig|6666666.59331.peg.1230	fig|6666666.59332.peg.2067	fig|6666666.59334.peg.2018	fig|6666666.59335.peg.4378	fig|6666666.59336.peg.5402	fig|6666666.59333.peg.3809	fig|6666666.59338.peg.3844	fig|6666666.59339.peg.2170	None	fig|6666666.59337.peg.1752	fig|6666666.59342.peg.1077	fig|6666666.71181.peg.507	fig|6666666.59344.peg.1728	fig|6666666.59345.peg.893	fig|6666666.59341.peg.3780	fig|6666666.59347.peg.2195	fig|6666666.59346.peg.1349	fig|6666666.59348.peg.1904	fig|6666666.59349.peg.385	None	fig|6666666.59351.peg.933	fig|6666666.59353.peg.4689	fig|6666666.59355.peg.4071	fig|6666666.59354.peg.5023

5271	Arginyl-tRNA synthetase EC 6.1.1.19	fig|6666666.59329.peg.5182	fig|6666666.59330.peg.4512	None	fig|6666666.59332.peg.934	fig|6666666.59334.peg.3428	fig|6666666.59335.peg.4146	fig|6666666.59336.peg.3093	fig|6666666.59333.peg.1338	fig|6666666.59338.peg.2372	fig|6666666.59339.peg.2996	fig|6666666.59340.peg.1412	fig|6666666.59337.peg.2735	fig|6666666.59342.peg.4854	fig|6666666.71181.peg.1906	fig|6666666.59344.peg.2536	fig|6666666.59345.peg.4309	fig|6666666.59341.peg.5059	fig|6666666.59347.peg.4402	None	fig|6666666.59348.peg.4214	fig|6666666.59349.peg.4220	fig|6666666.59350.peg.783	fig|6666666.59351.peg.2694	fig|6666666.59353.peg.1505	fig|6666666.59355.peg.5848	fig|6666666.59354.peg.3558

10194	Imidazole glycerol phosphate synthase amidotransferase subunit EC 2.4.2.-	fig|6666666.59329.peg.1012	fig|6666666.59330.peg.1505	None	fig|6666666.59332.peg.1811	fig|6666666.59334.peg.4499	fig|6666666.59335.peg.590	fig|6666666.59336.peg.4086	fig|6666666.59333.peg.2718	fig|6666666.59338.peg.2163	fig|6666666.59339.peg.498	fig|6666666.59340.peg.1343	fig|6666666.59337.peg.4871	fig|6666666.59342.peg.2821	fig|6666666.71181.peg.246	fig|6666666.59344.peg.308	fig|6666666.59345.peg.1304	fig|6666666.59341.peg.501	fig|6666666.59347.peg.4147	fig|6666666.59346.peg.2688	None	fig|6666666.59349.peg.456	fig|6666666.59350.peg.7448	fig|6666666.59351.peg.1622	fig|6666666.59353.peg.572	fig|6666666.59355.peg.56	fig|6666666.59354.peg.894

4776	FIG010427 hypothetical protein	fig|6666666.59329.peg.2918	fig|6666666.59330.peg.3727	fig|6666666.59331.peg.709	fig|6666666.59332.peg.4864	fig|6666666.59334.peg.3958	fig|6666666.59335.peg.5576	fig|6666666.59336.peg.6642	fig|6666666.59333.peg.5387	None	fig|6666666.59339.peg.3662	fig|6666666.59340.peg.2203	fig|6666666.59337.peg.4131	fig|6666666.59342.peg.1549	fig|6666666.71181.peg.144	fig|6666666.59344.peg.3668	fig|6666666.59345.peg.4602	fig|6666666.59341.peg.371	fig|6666666.59347.peg.1965	fig|6666666.59346.peg.2514	fig|6666666.59348.peg.895	fig|6666666.59349.peg.3599	None	fig|6666666.59351.peg.4845	fig|6666666.59353.peg.3846	fig|6666666.59355.peg.1540	fig|6666666.59354.peg.2389

4614	Fe-S protein, homolog of lactate dehydrogenase SO1521	fig|6666666.59329.peg.4760	fig|6666666.59330.peg.495	fig|6666666.59331.peg.1162	None	fig|6666666.59334.peg.5164	fig|6666666.59335.peg.1560	fig|6666666.59336.peg.5018	fig|6666666.59333.peg.4354	fig|6666666.59338.peg.1059	fig|6666666.59339.peg.2801	fig|6666666.59340.peg.686	fig|6666666.59337.peg.3317	None	fig|6666666.71181.peg.2572	fig|6666666.59344.peg.5722	fig|6666666.59345.peg.4622	fig|6666666.59341.peg.1394	fig|6666666.59347.peg.1949	fig|6666666.59346.peg.4467	fig|6666666.59348.peg.4096	fig|6666666.59349.peg.4151	fig|6666666.59350.peg.2001	fig|6666666.59351.peg.1701	fig|6666666.59353.peg.1344	fig|6666666.59355.peg.3349	fig|6666666.59354.peg.5384

2710	rRNA small subunit methyltransferase I	fig|6666666.59329.peg.3484	fig|6666666.59330.peg.973	fig|6666666.59331.peg.5694	fig|6666666.59332.peg.638	fig|6666666.59334.peg.3669	fig|6666666.59335.peg.4934	fig|6666666.59336.peg.711	fig|6666666.59333.peg.608	fig|6666666.59338.peg.537	fig|6666666.59339.peg.4955	None	fig|6666666.59337.peg.39	None	fig|6666666.71181.peg.4570	fig|6666666.59344.peg.1847	fig|6666666.59345.peg.3803	fig|6666666.59341.peg.1360	fig|6666666.59347.peg.3991	fig|6666666.59346.peg.2326	fig|6666666.59348.peg.2588	fig|6666666.59349.peg.51	fig|6666666.59350.peg.2807	fig|6666666.59351.peg.3473	fig|6666666.59353.peg.2377	fig|6666666.59355.peg.1649	fig|6666666.59354.peg.3735

6241	Glycine dehydrogenase [decarboxylating] glycine cleavage system P protein EC 1.4.4.2	fig|6666666.59329.peg.3664	fig|6666666.59330.peg.3581	fig|6666666.59331.peg.2407	fig|6666666.59332.peg.3781	None	fig|6666666.59335.peg.5905	fig|6666666.59336.peg.5090	fig|6666666.59333.peg.5015	fig|6666666.59338.peg.1258	fig|6666666.59339.peg.2094	None	fig|6666666.59337.peg.4822	fig|6666666.59342.peg.1967	fig|6666666.71181.peg.5253	fig|6666666.59344.peg.4747	fig|6666666.59345.peg.175	fig|6666666.59341.peg.434	fig|6666666.59347.peg.1405	fig|6666666.59346.peg.5183	fig|6666666.59348.peg.4758	fig|6666666.59349.peg.4508	fig|6666666.59350.peg.534	fig|6666666.59351.peg.2185	fig|6666666.59353.peg.4444	fig|6666666.59355.peg.4523	fig|6666666.59354.peg.3305

6246	DNA recombination and repair protein RecF	fig|6666666.59329.peg.3811	fig|6666666.59330.peg.1100	None	fig|6666666.59332.peg.83	fig|6666666.59334.peg.138	fig|6666666.59335.peg.3931	fig|6666666.59336.peg.9	fig|6666666.59333.peg.51	fig|6666666.59338.peg.1266	fig|6666666.59339.peg.8039	None	fig|6666666.59337.peg.872	fig|6666666.59342.peg.520	fig|6666666.71181.peg.624	fig|6666666.59344.peg.4171	fig|6666666.59345.peg.4981	fig|6666666.59341.peg.1040	fig|6666666.59347.peg.4800	fig|6666666.59346.peg.1197	fig|6666666.59348.peg.1364	fig|6666666.59349.peg.1005	fig|6666666.59350.peg.2730	fig|6666666.59351.peg.5030	fig|6666666.59353.peg.3055	fig|6666666.59355.peg.2291	fig|6666666.59354.peg.588

9709	Ferrochelatase, protoheme ferro-lyase EC 4.99.1.1	fig|6666666.59329.peg.1942	fig|6666666.59330.peg.5052	fig|6666666.59331.peg.1525	fig|6666666.59332.peg.1704	fig|6666666.59334.peg.210	fig|6666666.59335.peg.2417	fig|6666666.59336.peg.316	fig|6666666.59333.peg.2067	fig|6666666.59338.peg.2345	fig|6666666.59339.peg.5269	fig|6666666.59340.peg.2604	fig|6666666.59337.peg.3921	fig|6666666.59342.peg.1792	fig|6666666.71181.peg.3491	fig|6666666.59344.peg.5041	fig|6666666.59345.peg.4347	fig|6666666.59341.peg.512	fig|6666666.59347.peg.2831	None	None	fig|6666666.59349.peg.1238	fig|6666666.59350.peg.241	fig|6666666.59351.peg.1659	fig|6666666.59353.peg.4068	fig|6666666.59355.peg.4144	fig|6666666.59354.peg.5006

8872	Translation initiation factor 3	fig|6666666.59329.peg.4065	fig|6666666.59330.peg.515	fig|6666666.59331.peg.110	fig|6666666.59332.peg.4138	fig|6666666.59334.peg.4314	fig|6666666.59335.peg.3305	fig|6666666.59336.peg.5689	fig|6666666.59333.peg.522	fig|6666666.59338.peg.5743	fig|6666666.59339.peg.2413	None	fig|6666666.59337.peg.3298	fig|6666666.59342.peg.2815	fig|6666666.71181.peg.2419	fig|6666666.59344.peg.806	fig|6666666.59345.peg.4635	fig|6666666.59341.peg.3120	fig|6666666.59347.peg.3276	fig|6666666.59346.peg.4576	None	fig|6666666.59349.peg.407	fig|6666666.59350.peg.3158	fig|6666666.59351.peg.4792	fig|6666666.59353.peg.1302	fig|6666666.59355.peg.3279	fig|6666666.59354.peg.2977

8167	Putative heme iron utilization protein	fig|6666666.59329.peg.4535	fig|6666666.59330.peg.4173	fig|6666666.59331.peg.6187	fig|6666666.59332.peg.5734	fig|6666666.59334.peg.1578	fig|6666666.59335.peg.3625	fig|6666666.59336.peg.3052	fig|6666666.59333.peg.3773	fig|6666666.59338.peg.3297	fig|6666666.59339.peg.4296	None	fig|6666666.59337.peg.2460	fig|6666666.59342.peg.3206	fig|6666666.71181.peg.2391	fig|6666666.59344.peg.774	fig|6666666.59345.peg.593	fig|6666666.59341.peg.3098	fig|6666666.59347.peg.644	fig|6666666.59346.peg.3907	fig|6666666.59348.peg.5365	fig|6666666.59349.peg.2547	fig|6666666.59350.peg.883	None	fig|6666666.59353.peg.1331	fig|6666666.59355.peg.2540	fig|6666666.59354.peg.1989

5253	Polyphosphate kinase EC 2.7.4.1	fig|6666666.59329.peg.2868	fig|6666666.59330.peg.3681	fig|6666666.59331.peg.4030	fig|6666666.59332.peg.5064	fig|6666666.59334.peg.1105	fig|6666666.59335.peg.2569	fig|6666666.59336.peg.4851	fig|6666666.59333.peg.348	fig|6666666.59338.peg.2549	fig|6666666.59339.peg.6921	None	fig|6666666.59337.peg.711	None	fig|6666666.71181.peg.4288	fig|6666666.59344.peg.4042	fig|6666666.59345.peg.3679	fig|6666666.59341.peg.3804	fig|6666666.59347.peg.4263	fig|6666666.59346.peg.2536	fig|6666666.59348.peg.3425	fig|6666666.59349.peg.3545	fig|6666666.59350.peg.5871	fig|6666666.59351.peg.2318	fig|6666666.59353.peg.4013	fig|6666666.59355.peg.3972	fig|6666666.59354.peg.1543

3016	SeqA protein, negative modulator of initiation of replication	fig|6666666.59329.peg.1352	fig|6666666.59330.peg.2993	None	fig|6666666.59332.peg.5172	fig|6666666.59334.peg.4536	fig|6666666.59335.peg.2733	fig|6666666.59336.peg.335	fig|6666666.59333.peg.857	fig|6666666.59338.peg.3506	fig|6666666.59339.peg.7202	fig|6666666.59340.peg.1745	fig|6666666.59337.peg.3937	fig|6666666.59342.peg.2209	fig|6666666.71181.peg.3813	fig|6666666.59344.peg.5085	fig|6666666.59345.peg.4330	fig|6666666.59341.peg.4746	fig|6666666.59347.peg.2939	fig|6666666.59346.peg.2011	fig|6666666.59348.peg.1057	fig|6666666.59349.peg.1811	None	fig|6666666.59351.peg.1111	fig|6666666.59353.peg.2061	fig|6666666.59355.peg.2767	fig|6666666.59354.peg.4944

3014	ABC-type multidrug transport system, permease component	fig|6666666.59329.peg.4385	fig|6666666.59330.peg.3305	fig|6666666.59331.peg.4721	fig|6666666.59332.peg.4010	fig|6666666.59334.peg.2403	fig|6666666.59335.peg.2018	fig|6666666.59336.peg.4384	fig|6666666.59333.peg.3343	fig|6666666.59338.peg.1234	fig|6666666.59339.peg.3958	None	fig|6666666.59337.peg.3094	fig|6666666.59342.peg.6177	fig|6666666.71181.peg.3540	fig|6666666.59344.peg.2080	fig|6666666.59345.peg.4157	fig|6666666.59341.peg.2024	fig|6666666.59347.peg.1606	fig|6666666.59346.peg.3482	fig|6666666.59348.peg.4971	fig|6666666.59349.peg.2862	None	fig|6666666.59351.peg.2865	fig|6666666.59353.peg.3117	fig|6666666.59355.peg.479	fig|6666666.59354.peg.3638

8724	Cell division trigger factor EC 5.2.1.8	None	fig|6666666.59330.peg.293	fig|6666666.59331.peg.686	fig|6666666.59332.peg.2006	fig|6666666.59334.peg.509	fig|6666666.59335.peg.2708	fig|6666666.59336.peg.4126	fig|6666666.59333.peg.2634	fig|6666666.59338.peg.2553	fig|6666666.59339.peg.3095	None	fig|6666666.59337.peg.1023	fig|6666666.59342.peg.2792	fig|6666666.71181.peg.2051	fig|6666666.59344.peg.1069	fig|6666666.59345.peg.4259	fig|6666666.59341.peg.4430	fig|6666666.59347.peg.2659	fig|6666666.59346.peg.2782	fig|6666666.59348.peg.3842	fig|6666666.59349.peg.4765	fig|6666666.59350.peg.1862	fig|6666666.59351.peg.5100	fig|6666666.59353.peg.754	fig|6666666.59355.peg.950	fig|6666666.59354.peg.126

8180	GTP-binding and nucleic acid-binding protein YchF	fig|6666666.59329.peg.5982	fig|6666666.59330.peg.4671	fig|6666666.59331.peg.1443	fig|6666666.59332.peg.4259	None	fig|6666666.59335.peg.1172	fig|6666666.59336.peg.5141	fig|6666666.59333.peg.5157	fig|6666666.59338.peg.4132	fig|6666666.59339.peg.1	None	fig|6666666.59337.peg.5161	fig|6666666.59342.peg.471	fig|6666666.71181.peg.787	fig|6666666.59344.peg.99	fig|6666666.59345.peg.5410	fig|6666666.59341.peg.5835	fig|6666666.59347.peg.5285	fig|6666666.59346.peg.3185	fig|6666666.59348.peg.615	fig|6666666.59349.peg.2608	fig|6666666.59350.peg.2692	fig|6666666.59351.peg.4669	fig|6666666.59353.peg.424	fig|6666666.59355.peg.2149	fig|6666666.59354.peg.4333

8885	4-hydroxy-3-methylbut-2-enyl diphosphate reductase EC 1.17.1.2	fig|6666666.59329.peg.1376	fig|6666666.59330.peg.2515	None	fig|6666666.59332.peg.1083	fig|6666666.59334.peg.2850	fig|6666666.59335.peg.1003	fig|6666666.59336.peg.4540	fig|6666666.59333.peg.2498	fig|6666666.59338.peg.2587	fig|6666666.59339.peg.7472	fig|6666666.59340.peg.3473	fig|6666666.59337.peg.136	None	fig|6666666.71181.peg.418	fig|6666666.59344.peg.1313	fig|6666666.59345.peg.3706	fig|6666666.59341.peg.4626	fig|6666666.59347.peg.3017	fig|6666666.59346.peg.241	fig|6666666.59348.peg.503	fig|6666666.59349.peg.572	fig|6666666.59350.peg.1365	fig|6666666.59351.peg.505	fig|6666666.59353.peg.80	fig|6666666.59355.peg.687	fig|6666666.59354.peg.2639

3659	Thiazole biosynthesis protein ThiG	fig|6666666.59329.peg.3986	fig|6666666.59330.peg.981	fig|6666666.59331.peg.1382	fig|6666666.59332.peg.253	fig|6666666.59334.peg.2193	fig|6666666.59335.peg.9	fig|6666666.59336.peg.178	fig|6666666.59333.peg.1568	fig|6666666.59338.peg.1669	fig|6666666.59339.peg.7533	fig|6666666.59340.peg.3659	fig|6666666.59337.peg.2550	None	fig|6666666.71181.peg.3361	None	fig|6666666.59345.peg.5542	fig|6666666.59341.peg.3745	fig|6666666.59347.peg.5254	fig|6666666.59346.peg.931	fig|6666666.59348.peg.3769	fig|6666666.59349.peg.4744	fig|6666666.59350.peg.2928	fig|6666666.59351.peg.3886	fig|6666666.59353.peg.4462	fig|6666666.59355.peg.4565	fig|6666666.59354.peg.6331

922	ATP phosphoribosyltransferase EC 2.4.2.17	fig|6666666.59329.peg.1008	fig|6666666.59330.peg.2864	fig|6666666.59331.peg.2271	fig|6666666.59332.peg.5630	None	fig|6666666.59335.peg.594	fig|6666666.59336.peg.4610	fig|6666666.59333.peg.2714	fig|6666666.59338.peg.3963	fig|6666666.59339.peg.491	None	fig|6666666.59337.peg.4867	fig|6666666.59342.peg.544	fig|6666666.71181.peg.250	fig|6666666.59344.peg.313	fig|6666666.59345.peg.1308	fig|6666666.59341.peg.495	fig|6666666.59347.peg.4143	fig|6666666.59346.peg.2681	fig|6666666.59348.peg.5105	fig|6666666.59349.peg.460	fig|6666666.59350.peg.5368	fig|6666666.59351.peg.1626	fig|6666666.59353.peg.568	fig|6666666.59355.peg.52	fig|6666666.59354.peg.889

8602	Thiol peroxidase, Tpx-type EC 1.11.1.15	fig|6666666.59329.peg.6176	fig|6666666.59330.peg.4463	fig|6666666.59331.peg.6344	fig|6666666.59332.peg.2065	fig|6666666.59334.peg.845	fig|6666666.59335.peg.5437	fig|6666666.59336.peg.6054	fig|6666666.59333.peg.4674	fig|6666666.59338.peg.3674	fig|6666666.59339.peg.7657	None	fig|6666666.59337.peg.2942	fig|6666666.59342.peg.4122	fig|6666666.71181.peg.1382	fig|6666666.59344.peg.539	fig|6666666.59345.peg.2066	fig|6666666.59341.peg.5576	fig|6666666.59347.peg.79	fig|6666666.59346.peg.2520	fig|6666666.59348.peg.571	fig|6666666.59349.peg.2400	None	fig|6666666.59351.peg.630	fig|6666666.59353.peg.1846	fig|6666666.59355.peg.1168	fig|6666666.59354.peg.1500

1458	Lipoprotein signal peptidase EC 3.4.23.36	fig|6666666.59329.peg.1374	fig|6666666.59330.peg.2513	None	fig|6666666.59332.peg.1081	fig|6666666.59334.peg.3208	fig|6666666.59335.peg.1007	fig|6666666.59336.peg.4543	fig|6666666.59333.peg.2495	fig|6666666.59338.peg.2589	fig|6666666.59339.peg.7470	None	fig|6666666.59337.peg.134	fig|6666666.59342.peg.3634	fig|6666666.71181.peg.420	fig|6666666.59344.peg.1315	fig|6666666.59345.peg.3709	fig|6666666.59341.peg.4624	fig|6666666.59347.peg.3019	fig|6666666.59346.peg.238	fig|6666666.59348.peg.505	fig|6666666.59349.peg.570	fig|6666666.59350.peg.915	fig|6666666.59351.peg.503	fig|6666666.59353.peg.78	fig|6666666.59355.peg.685	fig|6666666.59354.peg.2641

1897	Transaldolase EC 2.2.1.2	fig|6666666.59329.peg.920	fig|6666666.59330.peg.1128	fig|6666666.59331.peg.718	None	fig|6666666.59334.peg.2703	fig|6666666.59335.peg.5296	fig|6666666.59336.peg.4820	fig|6666666.59333.peg.4281	fig|6666666.59338.peg.2783	fig|6666666.59339.peg.8504	fig|6666666.59340.peg.3317	fig|6666666.59337.peg.5301	fig|6666666.59342.peg.565	fig|6666666.71181.peg.431	fig|6666666.59344.peg.1333	fig|6666666.59345.peg.1830	fig|6666666.59341.peg.3653	fig|6666666.59347.peg.1595	fig|6666666.59346.peg.3860	None	fig|6666666.59349.peg.4229	fig|6666666.59350.peg.4694	fig|6666666.59351.peg.3908	fig|6666666.59353.peg.1815	fig|6666666.59355.peg.2780	fig|6666666.59354.peg.2749

11253	DNA recombination-dependent growth factor C	fig|6666666.59329.peg.6050	fig|6666666.59330.peg.3676	fig|6666666.59331.peg.4766	fig|6666666.59332.peg.1264	fig|6666666.59334.peg.2954	fig|6666666.59335.peg.2563	fig|6666666.59336.peg.4270	fig|6666666.59333.peg.342	fig|6666666.59338.peg.1862	fig|6666666.59339.peg.6912	fig|6666666.59340.peg.247	fig|6666666.59337.peg.703	None	fig|6666666.71181.peg.2184	fig|6666666.59344.peg.1145	fig|6666666.59345.peg.3684	fig|6666666.59341.peg.3798	fig|6666666.59347.peg.4258	fig|6666666.59346.peg.2545	fig|6666666.59348.peg.2589	fig|6666666.59349.peg.3858	None	fig|6666666.59351.peg.4262	fig|6666666.59353.peg.816	fig|6666666.59355.peg.890	fig|6666666.59354.peg.1170

9726	Integration host factor alpha subunit	fig|6666666.59329.peg.5784	fig|6666666.59330.peg.3371	None	fig|6666666.59332.peg.2613	fig|6666666.59334.peg.2151	fig|6666666.59335.peg.3842	None	fig|6666666.59333.peg.537	fig|6666666.59338.peg.3300	fig|6666666.59339.peg.566	fig|6666666.59340.peg.3211	fig|6666666.59337.peg.4524	fig|6666666.59342.peg.386	fig|6666666.71181.peg.2414	fig|6666666.59344.peg.800	fig|6666666.59345.peg.4660	fig|6666666.59341.peg.448	fig|6666666.59347.peg.1876	fig|6666666.59346.peg.2927	fig|6666666.59348.peg.3889	fig|6666666.59349.peg.3581	fig|6666666.59350.peg.3676	fig|6666666.59351.peg.4427	fig|6666666.59353.peg.1307	fig|6666666.59355.peg.2564	fig|6666666.59354.peg.4285

2407	Ribosomal RNA large subunit methyltransferase N EC 2.1.1.-	fig|6666666.59329.peg.5400	fig|6666666.59330.peg.1423	None	fig|6666666.59332.peg.1338	fig|6666666.59334.peg.3868	fig|6666666.59335.peg.1114	fig|6666666.59336.peg.679	fig|6666666.59333.peg.934	fig|6666666.59338.peg.4500	fig|6666666.59339.peg.6965	None	fig|6666666.59337.peg.749	fig|6666666.59342.peg.1059	fig|6666666.71181.peg.1767	fig|6666666.59344.peg.1641	fig|6666666.59345.peg.3643	fig|6666666.59341.peg.3039	fig|6666666.59347.peg.4292	fig|6666666.59346.peg.1321	fig|6666666.59348.peg.1134	fig|6666666.59349.peg.3079	fig|6666666.59350.peg.205	fig|6666666.59351.peg.3071	fig|6666666.59353.peg.2706	fig|6666666.59355.peg.1774	fig|6666666.59354.peg.81

5647	Dihydroxy-acid dehydratase EC 4.2.1.9	fig|6666666.59329.peg.630	fig|6666666.59330.peg.621	None	fig|6666666.59332.peg.113	fig|6666666.59334.peg.2811	fig|6666666.59335.peg.4368	fig|6666666.59336.peg.48	fig|6666666.59333.peg.13	fig|6666666.59338.peg.2478	fig|6666666.59339.peg.8061	fig|6666666.59340.peg.1005	fig|6666666.59337.peg.905	None	fig|6666666.71181.peg.3088	fig|6666666.59344.peg.2697	fig|6666666.59345.peg.2737	fig|6666666.59341.peg.2122	fig|6666666.59347.peg.4768	fig|6666666.59346.peg.1417	fig|6666666.59348.peg.73	fig|6666666.59349.peg.1127	fig|6666666.59350.peg.426	fig|6666666.59351.peg.898	fig|6666666.59353.peg.3030	fig|6666666.59355.peg.2937	fig|6666666.59354.peg.295

3920	Aspartate-semialdehyde dehydrogenase EC 1.2.1.11	fig|6666666.59329.peg.4397	fig|6666666.59330.peg.1596	None	fig|6666666.59332.peg.2182	fig|6666666.59334.peg.1831	fig|6666666.59335.peg.5775	fig|6666666.59336.peg.3663	fig|6666666.59333.peg.5481	fig|6666666.59338.peg.1856	fig|6666666.59339.peg.7052	fig|6666666.59340.peg.2798	fig|6666666.59337.peg.3149	None	fig|6666666.71181.peg.1110	fig|6666666.59344.peg.3617	fig|6666666.59345.peg.5700	fig|6666666.59341.peg.2778	fig|6666666.59347.peg.2459	fig|6666666.59346.peg.5107	fig|6666666.59348.peg.3375	fig|6666666.59349.peg.4524	fig|6666666.59350.peg.3125	fig|6666666.59351.peg.2141	fig|6666666.59353.peg.2170	fig|6666666.59355.peg.264	fig|6666666.59354.peg.2619

10293	LSU ribosomal protein L31p LSU ribosomal protein L31p, zinc-independent	fig|6666666.59329.peg.1824	fig|6666666.59330.peg.483	None	fig|6666666.59332.peg.975	fig|6666666.59334.peg.1546	fig|6666666.59335.peg.4081	fig|6666666.59336.peg.5788	fig|6666666.59333.peg.2256	fig|6666666.59338.peg.1210	fig|6666666.59339.peg.5162	fig|6666666.59340.peg.310	fig|6666666.59337.peg.1567	fig|6666666.59342.peg.3445	fig|6666666.71181.peg.2083	fig|6666666.59344.peg.1030	fig|6666666.59345.peg.2215	fig|6666666.59341.peg.2832	fig|6666666.59347.peg.284	fig|6666666.59346.peg.3530	fig|6666666.59348.peg.1151	fig|6666666.59349.peg.4610	None	fig|6666666.59351.peg.4603	fig|6666666.59353.peg.722	fig|6666666.59355.peg.984	fig|6666666.59354.peg.2912

2549	Dihydrofolate reductase EC 1.5.1.3	fig|6666666.59329.peg.135	fig|6666666.59330.peg.821	fig|6666666.59331.peg.2693	fig|6666666.59332.peg.3916	None	fig|6666666.59335.peg.4484	fig|6666666.59336.peg.2657	fig|6666666.59333.peg.5030	fig|6666666.59338.peg.913	fig|6666666.59339.peg.7888	fig|6666666.59340.peg.430	fig|6666666.59337.peg.1381	fig|6666666.59342.peg.2180	fig|6666666.71181.peg.405	fig|6666666.59344.peg.1296	fig|6666666.59345.peg.2463	fig|6666666.59341.peg.1999	fig|6666666.59347.peg.3092	fig|6666666.59346.peg.3977	fig|6666666.59348.peg.173	fig|6666666.59349.peg.3663	fig|6666666.59350.peg.3812	fig|6666666.59351.peg.304	fig|6666666.59353.peg.93	fig|6666666.59355.peg.703	None

9558	Periplasmic thiol disulfide oxidoreductase DsbB, required for DsbA reoxidation	fig|6666666.59329.peg.1002	fig|6666666.59330.peg.3949	fig|6666666.59331.peg.4842	fig|6666666.59332.peg.4456	fig|6666666.59334.peg.1209	fig|6666666.59335.peg.1300	fig|6666666.59336.peg.4023	fig|6666666.59333.peg.1537	None	fig|6666666.59339.peg.3429	fig|6666666.59340.peg.2256	fig|6666666.59337.peg.3842	fig|6666666.59342.peg.1965	fig|6666666.71181.peg.2947	fig|6666666.59344.peg.2865	fig|6666666.59345.peg.1086	fig|6666666.59341.peg.2385	fig|6666666.59347.peg.2625	fig|6666666.59346.peg.555	fig|6666666.59348.peg.4389	fig|6666666.59349.peg.2720	None	fig|6666666.59351.peg.560	fig|6666666.59353.peg.1457	fig|6666666.59355.peg.2007	fig|6666666.59354.peg.113

1693	Membrane-bound lytic murein transglycosylase B EC 3.2.1.-	fig|6666666.59329.peg.4745	fig|6666666.59330.peg.4580	fig|6666666.59331.peg.5127	fig|6666666.59332.peg.1212	fig|6666666.59334.peg.4987	fig|6666666.59335.peg.2758	fig|6666666.59336.peg.1020	fig|6666666.59333.peg.1112	fig|6666666.59338.peg.1139	fig|6666666.59339.peg.244	None	fig|6666666.59337.peg.2754	None	fig|6666666.71181.peg.2944	fig|6666666.59344.peg.2861	fig|6666666.59345.peg.4292	fig|6666666.59341.peg.3861	fig|6666666.59347.peg.4417	fig|6666666.59346.peg.1471	fig|6666666.59348.peg.1744	fig|6666666.59349.peg.478	fig|6666666.59350.peg.1777	fig|6666666.59351.peg.1648	fig|6666666.59353.peg.1460	fig|6666666.59355.peg.2011	fig|6666666.59354.peg.5787

4227	Acetyl-coenzyme A carboxyl transferase beta chain EC 6.4.1.2	fig|6666666.59329.peg.5764	fig|6666666.59330.peg.1600	fig|6666666.59331.peg.4694	fig|6666666.59332.peg.2965	None	fig|6666666.59335.peg.3590	None	fig|6666666.59333.peg.4802	fig|6666666.59338.peg.2033	fig|6666666.59339.peg.2970	fig|6666666.59340.peg.3635	fig|6666666.59337.peg.3452	fig|6666666.59342.peg.3885	fig|6666666.71181.peg.1477	fig|6666666.59344.peg.1883	fig|6666666.59345.peg.1204	fig|6666666.59341.peg.3864	fig|6666666.59347.peg.2456	fig|6666666.59346.peg.52	fig|6666666.59348.peg.5541	fig|6666666.59349.peg.3459	fig|6666666.59350.peg.327	fig|6666666.59351.peg.3571	fig|6666666.59353.peg.1209	fig|6666666.59355.peg.267	fig|6666666.59354.peg.2964

11271	Sigma factor RpoE negative regulatory protein RseA	fig|6666666.59329.peg.497	fig|6666666.59330.peg.380	fig|6666666.59331.peg.3849	fig|6666666.59332.peg.734	fig|6666666.59334.peg.4732	fig|6666666.59335.peg.1721	fig|6666666.59336.peg.2106	fig|6666666.59333.peg.2803	fig|6666666.59338.peg.1510	fig|6666666.59339.peg.7360	fig|6666666.59340.peg.856	fig|6666666.59337.peg.560	fig|6666666.59342.peg.2972	fig|6666666.71181.peg.1429	fig|6666666.59344.peg.1559	fig|6666666.59345.peg.5050	fig|6666666.59341.peg.4053	fig|6666666.59347.peg.3181	fig|6666666.59346.peg.329	None	fig|6666666.59349.peg.4313	None	fig|6666666.59351.peg.593	fig|6666666.59353.peg.2646	fig|6666666.59355.peg.1836	fig|6666666.59354.peg.2810

7720	GTP-binding protein EngB	fig|6666666.59329.peg.6579	fig|6666666.59330.peg.263	fig|6666666.59331.peg.5603	fig|6666666.59332.peg.20	fig|6666666.59334.peg.841	fig|6666666.59335.peg.52	fig|6666666.59336.peg.1455	fig|6666666.59333.peg.837	fig|6666666.59338.peg.1968	fig|6666666.59339.peg.7590	fig|6666666.59340.peg.98	fig|6666666.59337.peg.972	None	fig|6666666.71181.peg.2586	fig|6666666.59344.peg.896	fig|6666666.59345.peg.2703	fig|6666666.59341.peg.4973	fig|6666666.59347.peg.4645	fig|6666666.59346.peg.1075	fig|6666666.59348.peg.104	fig|6666666.59349.peg.4488	None	fig|6666666.59351.peg.99	fig|6666666.59353.peg.2631	fig|6666666.59355.peg.2573	fig|6666666.59354.peg.1898

3943	3-polyprenyl-4-hydroxybenzoate carboxy-lyase UbiX EC 4.1.1.-	fig|6666666.59329.peg.6477	fig|6666666.59330.peg.837	fig|6666666.59331.peg.1731	fig|6666666.59332.peg.2332	fig|6666666.59334.peg.54	fig|6666666.59335.peg.182	fig|6666666.59336.peg.918	fig|6666666.59333.peg.570	fig|6666666.59338.peg.309	fig|6666666.59339.peg.7732	fig|6666666.59340.peg.515	fig|6666666.59337.peg.330	None	fig|6666666.71181.peg.572	fig|6666666.59344.peg.1889	fig|6666666.59345.peg.2480	fig|6666666.59341.peg.1230	fig|6666666.59347.peg.4874	fig|6666666.59346.peg.652	fig|6666666.59348.peg.1996	fig|6666666.59349.peg.3088	None	fig|6666666.59351.peg.336	fig|6666666.59353.peg.1204	fig|6666666.59355.peg.272	fig|6666666.59354.peg.96

1670	membrane protein	fig|6666666.59329.peg.323	fig|6666666.59330.peg.2643	fig|6666666.59331.peg.3745	fig|6666666.59332.peg.1820	fig|6666666.59334.peg.167	fig|6666666.59335.peg.3311	fig|6666666.59336.peg.598	fig|6666666.59333.peg.887	fig|6666666.59338.peg.4455	fig|6666666.59339.peg.895	None	fig|6666666.59337.peg.1709	fig|6666666.59342.peg.1895	fig|6666666.71181.peg.3950	fig|6666666.59344.peg.5435	fig|6666666.59345.peg.5372	fig|6666666.59341.peg.2417	fig|6666666.59347.peg.5375	fig|6666666.59346.peg.518	fig|6666666.59348.peg.3802	fig|6666666.59349.peg.230	None	fig|6666666.59351.peg.227	fig|6666666.59353.peg.4807	fig|6666666.59355.peg.4861	fig|6666666.59354.peg.2526

10923	Type IV fimbrial biogenesis protein FimT	fig|6666666.59329.peg.1665	fig|6666666.59330.peg.4788	fig|6666666.59331.peg.1069	fig|6666666.59332.peg.391	None	fig|6666666.59335.peg.1690	fig|6666666.59336.peg.3611	fig|6666666.59333.peg.3463	fig|6666666.59338.peg.3970	fig|6666666.59339.peg.7266	fig|6666666.59340.peg.1148	fig|6666666.59337.peg.4977	fig|6666666.59342.peg.2191	fig|6666666.71181.peg.68	fig|6666666.59344.peg.3199	fig|6666666.59345.peg.3568	fig|6666666.59341.peg.4069	fig|6666666.59347.peg.4334	fig|6666666.59346.peg.4645	fig|6666666.59348.peg.1567	fig|6666666.59349.peg.3233	None	fig|6666666.59351.peg.5427	fig|6666666.59353.peg.1947	fig|6666666.59355.peg.1460	fig|6666666.59354.peg.2010

10929	Translation initiation factor 2	fig|6666666.59329.peg.3442	fig|6666666.59330.peg.106	fig|6666666.59331.peg.2125	fig|6666666.59332.peg.3548	fig|6666666.59334.peg.780	fig|6666666.59335.peg.2100	fig|6666666.59336.peg.2424	fig|6666666.59333.peg.2326	fig|6666666.59338.peg.4600	fig|6666666.59339.peg.4894	None	fig|6666666.59337.peg.684	fig|6666666.59342.peg.378	fig|6666666.71181.peg.4098	fig|6666666.59344.peg.2099	fig|6666666.59345.peg.2356	fig|6666666.59341.peg.2440	fig|6666666.59347.peg.3004	fig|6666666.59346.peg.299	None	fig|6666666.59349.peg.1434	fig|6666666.59350.peg.224	fig|6666666.59351.peg.1389	fig|6666666.59353.peg.3132	fig|6666666.59355.peg.464	fig|6666666.59354.peg.2275

10587	Membrane Protein Functionally coupled to the MukBEF Chromosome Partitioning Mechanism	fig|6666666.59329.peg.5352	fig|6666666.59330.peg.4159	fig|6666666.59331.peg.600	fig|6666666.59332.peg.3605	fig|6666666.59334.peg.596	fig|6666666.59335.peg.935	fig|6666666.59336.peg.1402	fig|6666666.59333.peg.4424	fig|6666666.59338.peg.2427	fig|6666666.59339.peg.311	None	fig|6666666.59337.peg.4935	fig|6666666.59342.peg.140	fig|6666666.71181.peg.4183	None	fig|6666666.59345.peg.1536	fig|6666666.59341.peg.2326	fig|6666666.59347.peg.2372	fig|6666666.59346.peg.2020	fig|6666666.59348.peg.1189	fig|6666666.59349.peg.1329	fig|6666666.59350.peg.5189	fig|6666666.59351.peg.5090	fig|6666666.59353.peg.1659	fig|6666666.59355.peg.1062	fig|6666666.59354.peg.158

11185	Predicted dye-decolorizing peroxidase DyP , YfeX-like subgroup	fig|6666666.59329.peg.1406	fig|6666666.59330.peg.4470	fig|6666666.59331.peg.6263	fig|6666666.59332.peg.3088	fig|6666666.59334.peg.3082	fig|6666666.59335.peg.374	fig|6666666.59336.peg.2157	fig|6666666.59333.peg.3556	fig|6666666.59338.peg.3388	fig|6666666.59339.peg.7121	fig|6666666.59340.peg.1760	fig|6666666.59337.peg.5639	fig|6666666.59342.peg.2561	fig|6666666.71181.peg.4710	fig|6666666.59344.peg.1990	fig|6666666.59345.peg.2143	fig|6666666.59341.peg.1689	fig|6666666.59347.peg.2839	fig|6666666.59346.peg.3538	None	fig|6666666.59349.peg.4580	None	fig|6666666.59351.peg.3960	fig|6666666.59353.peg.4953	fig|6666666.59355.peg.2809	fig|6666666.59354.peg.378

1904	Riboflavin kinase EC 2.7.1.26 FMN adenylyltransferase EC 2.7.7.2	None	fig|6666666.59330.peg.2511	fig|6666666.59331.peg.4570	fig|6666666.59332.peg.3311	fig|6666666.59334.peg.2148	fig|6666666.59335.peg.1010	fig|6666666.59336.peg.5212	fig|6666666.59333.peg.3802	fig|6666666.59338.peg.2591	fig|6666666.59339.peg.7466	None	fig|6666666.59337.peg.132	fig|6666666.59342.peg.1397	fig|6666666.71181.peg.422	fig|6666666.59344.peg.1318	fig|6666666.59345.peg.3711	fig|6666666.59341.peg.5676	fig|6666666.59347.peg.3021	fig|6666666.59346.peg.236	fig|6666666.59348.peg.1110	fig|6666666.59349.peg.567	fig|6666666.59350.peg.2076	fig|6666666.59351.peg.501	fig|6666666.59353.peg.76	fig|6666666.59355.peg.5932	fig|6666666.59354.peg.2645

1337	tRNA 5-methylaminomethyl-2-thiouridine synthase TusB	fig|6666666.59329.peg.3692	fig|6666666.59330.peg.4072	fig|6666666.59331.peg.5456	fig|6666666.59332.peg.1077	fig|6666666.59334.peg.441	fig|6666666.59335.peg.5579	fig|6666666.59336.peg.2179	fig|6666666.59333.peg.3103	fig|6666666.59338.peg.3251	fig|6666666.59339.peg.8873	None	fig|6666666.59337.peg.1823	fig|6666666.59342.peg.1923	fig|6666666.71181.peg.4312	fig|6666666.59344.peg.3131	fig|6666666.59345.peg.5552	fig|6666666.59341.peg.5089	fig|6666666.59347.peg.5332	fig|6666666.59346.peg.45	fig|6666666.59348.peg.3178	fig|6666666.59349.peg.4844	None	fig|6666666.59351.peg.3129	fig|6666666.59353.peg.2273	fig|6666666.59355.peg.1966	fig|6666666.59354.peg.5882

8122	GlpG protein membrane protein of glp regulon	fig|6666666.59329.peg.774	fig|6666666.59330.peg.1569	fig|6666666.59331.peg.6361	fig|6666666.59332.peg.2375	fig|6666666.59334.peg.1296	fig|6666666.59335.peg.4266	fig|6666666.59336.peg.1374	fig|6666666.59333.peg.1954	fig|6666666.59338.peg.530	fig|6666666.59339.peg.8572	fig|6666666.59340.peg.3674	fig|6666666.59337.peg.1653	fig|6666666.59342.peg.2198	fig|6666666.71181.peg.4491	fig|6666666.59344.peg.3629	fig|6666666.59345.peg.5467	fig|6666666.59341.peg.5291	fig|6666666.59347.peg.5051	fig|6666666.59346.peg.1642	fig|6666666.59348.peg.587	fig|6666666.59349.peg.2782	None	fig|6666666.59351.peg.5211	fig|6666666.59353.peg.2181	fig|6666666.59355.peg.1875	None

3963	Primosomal replication protein N prime prime	fig|6666666.59329.peg.2650	fig|6666666.59330.peg.1307	fig|6666666.59331.peg.4371	fig|6666666.59332.peg.4846	fig|6666666.59334.peg.5023	fig|6666666.59335.peg.4287	fig|6666666.59336.peg.4224	fig|6666666.59333.peg.1019	fig|6666666.59338.peg.772	fig|6666666.59339.peg.449	fig|6666666.59340.peg.2331	fig|6666666.59337.peg.3383	None	fig|6666666.71181.peg.3503	fig|6666666.59344.peg.5525	fig|6666666.59345.peg.1566	fig|6666666.59341.peg.2332	fig|6666666.59347.peg.2364	fig|6666666.59346.peg.2201	fig|6666666.59348.peg.3381	fig|6666666.59349.peg.1910	None	fig|6666666.59351.peg.471	fig|6666666.59353.peg.4081	fig|6666666.59355.peg.4157	fig|6666666.59354.peg.5737

11406	Putative esterase, FIGfam005057	fig|6666666.59329.peg.3072	fig|6666666.59330.peg.953	fig|6666666.59331.peg.6242	fig|6666666.59332.peg.1426	fig|6666666.59334.peg.393	fig|6666666.59335.peg.3445	fig|6666666.59336.peg.1938	fig|6666666.59333.peg.629	fig|6666666.59338.peg.971	fig|6666666.59339.peg.4928	None	fig|6666666.59337.peg.19	fig|6666666.59342.peg.3212	fig|6666666.71181.peg.3038	fig|6666666.59344.peg.5619	fig|6666666.59345.peg.3822	fig|6666666.59341.peg.1723	fig|6666666.59347.peg.3913	fig|6666666.59346.peg.1634	fig|6666666.59348.peg.2787	fig|6666666.59349.peg.819	None	fig|6666666.59351.peg.3548	fig|6666666.59353.peg.4924	fig|6666666.59355.peg.4967	fig|6666666.59354.peg.664

4465	Probable component of the lipoprotein assembly complex forms a complex with YaeT, YfgL, and NlpB	fig|6666666.59329.peg.3084	fig|6666666.59330.peg.2895	fig|6666666.59331.peg.2325	fig|6666666.59332.peg.1318	fig|6666666.59334.peg.863	fig|6666666.59335.peg.4773	None	fig|6666666.59333.peg.3334	fig|6666666.59338.peg.3261	fig|6666666.59339.peg.7514	None	fig|6666666.59337.peg.159	fig|6666666.59342.peg.3354	fig|6666666.71181.peg.3945	fig|6666666.59344.peg.5429	fig|6666666.59345.peg.5682	fig|6666666.59341.peg.3001	fig|6666666.59347.peg.4056	fig|6666666.59346.peg.270	fig|6666666.59348.peg.2267	fig|6666666.59349.peg.584	fig|6666666.59350.peg.1049	fig|6666666.59351.peg.516	fig|6666666.59353.peg.4802	fig|6666666.59355.peg.4856	fig|6666666.59354.peg.1585

1652	Ribosome recycling factor	fig|6666666.59329.peg.4320	fig|6666666.59330.peg.466	fig|6666666.59331.peg.3458	fig|6666666.59332.peg.1587	fig|6666666.59334.peg.5188	fig|6666666.59335.peg.166	fig|6666666.59336.peg.6241	fig|6666666.59333.peg.3058	fig|6666666.59338.peg.4901	fig|6666666.59339.peg.5111	None	fig|6666666.59337.peg.1584	fig|6666666.59342.peg.269	fig|6666666.71181.peg.32	fig|6666666.59344.peg.2656	fig|6666666.59345.peg.2198	fig|6666666.59341.peg.970	fig|6666666.59347.peg.4361	fig|6666666.59346.peg.2263	None	fig|6666666.59349.peg.1373	fig|6666666.59350.peg.1877	fig|6666666.59351.peg.2117	fig|6666666.59353.peg.1986	fig|6666666.59355.peg.4093	fig|6666666.59354.peg.1335

2832	Histidine ammonia-lyase EC 4.3.1.3	fig|6666666.59329.peg.2409	fig|6666666.59330.peg.506	fig|6666666.59331.peg.3434	None	None	fig|6666666.59335.peg.1563	fig|6666666.59336.peg.1048	fig|6666666.59333.peg.3574	fig|6666666.59338.peg.432	fig|6666666.59339.peg.3867	fig|6666666.59340.peg.1249	fig|6666666.59337.peg.3308	fig|6666666.59342.peg.3864	fig|6666666.71181.peg.3781	fig|6666666.59344.peg.2221	fig|6666666.59345.peg.4625	fig|6666666.59341.peg.1312	fig|6666666.59347.peg.1560	fig|6666666.59346.peg.2120	fig|6666666.59348.peg.2535	fig|6666666.59349.peg.496	fig|6666666.59350.peg.3593	fig|6666666.59351.peg.4289	fig|6666666.59353.peg.190	fig|6666666.59355.peg.2954	fig|6666666.59354.peg.4847

8596	Cell division protein FtsQ	fig|6666666.59329.peg.1361	fig|6666666.59330.peg.1280	fig|6666666.59331.peg.5410	fig|6666666.59332.peg.3450	None	fig|6666666.59335.peg.828	fig|6666666.59336.peg.952	fig|6666666.59333.peg.4864	fig|6666666.59338.peg.2682	fig|6666666.59339.peg.4975	fig|6666666.59340.peg.1430	fig|6666666.59337.peg.51	fig|6666666.59342.peg.2797	fig|6666666.71181.peg.1977	fig|6666666.59344.peg.1476	fig|6666666.59345.peg.3790	fig|6666666.59341.peg.4863	fig|6666666.59347.peg.4003	fig|6666666.59346.peg.1244	None	fig|6666666.59349.peg.2988	fig|6666666.59350.peg.5231	fig|6666666.59351.peg.212	fig|6666666.59353.peg.141	fig|6666666.59355.peg.751	fig|6666666.59354.peg.6125

4871	Pyruvate dehydrogenase E1 component EC 1.2.4.1	fig|6666666.59329.peg.2591	fig|6666666.59330.peg.3497	fig|6666666.59331.peg.1082	fig|6666666.59332.peg.300	fig|6666666.59334.peg.481	fig|6666666.59335.peg.2341	fig|6666666.59336.peg.6545	fig|6666666.59333.peg.146	fig|6666666.59338.peg.918	fig|6666666.59339.peg.7967	None	fig|6666666.59337.peg.2058	fig|6666666.59342.peg.1877	fig|6666666.71181.peg.1847	fig|6666666.59344.peg.4415	fig|6666666.59345.peg.2419	fig|6666666.59341.peg.5781	fig|6666666.59347.peg.3898	fig|6666666.59346.peg.587	fig|6666666.59348.peg.664	fig|6666666.59349.peg.3765	None	fig|6666666.59351.peg.2671	fig|6666666.59353.peg.3212	fig|6666666.59355.peg.3094	fig|6666666.59354.peg.772

11393	Flavodoxin 2	fig|6666666.59329.peg.211	fig|6666666.59330.peg.1738	None	fig|6666666.59332.peg.2003	fig|6666666.59334.peg.2815	fig|6666666.59335.peg.3815	fig|6666666.59336.peg.2434	fig|6666666.59333.peg.4878	fig|6666666.59338.peg.202	fig|6666666.59339.peg.7957	fig|6666666.59340.peg.379	fig|6666666.59337.peg.106	fig|6666666.59342.peg.3779	fig|6666666.71181.peg.3735	fig|6666666.59344.peg.3678	fig|6666666.59345.peg.3735	fig|6666666.59341.peg.3452	fig|6666666.59347.peg.3047	fig|6666666.59346.peg.3097	fig|6666666.59348.peg.669	fig|6666666.59349.peg.4392	None	fig|6666666.59351.peg.4035	fig|6666666.59353.peg.3837	fig|6666666.59355.peg.1550	fig|6666666.59354.peg.4890

4012	ABC-type multidrug transport system, permease component	fig|6666666.59329.peg.4152	fig|6666666.59330.peg.4043	fig|6666666.59331.peg.5525	fig|6666666.59332.peg.3810	fig|6666666.59334.peg.816	fig|6666666.59335.peg.2331	fig|6666666.59336.peg.2749	fig|6666666.59333.peg.137	fig|6666666.59338.peg.5018	fig|6666666.59339.peg.4763	None	fig|6666666.59337.peg.2068	None	fig|6666666.71181.peg.2848	fig|6666666.59344.peg.4429	fig|6666666.59345.peg.2410	fig|6666666.59341.peg.1715	fig|6666666.59347.peg.3906	fig|6666666.59346.peg.3818	fig|6666666.59348.peg.2109	fig|6666666.59349.peg.3090	fig|6666666.59350.peg.497	fig|6666666.59351.peg.338	fig|6666666.59353.peg.3200	fig|6666666.59355.peg.3081	fig|6666666.59354.peg.99

7873	CDP-diacylglycerol--serine O-phosphatidyltransferase EC 2.7.8.8	fig|6666666.59329.peg.2424	fig|6666666.59330.peg.4561	fig|6666666.59331.peg.4391	fig|6666666.59332.peg.3158	fig|6666666.59334.peg.2116	fig|6666666.59335.peg.411	fig|6666666.59336.peg.3402	fig|6666666.59333.peg.4948	fig|6666666.59338.peg.1492	fig|6666666.59339.peg.6356	None	fig|6666666.59337.peg.5514	fig|6666666.59342.peg.1702	fig|6666666.71181.peg.2650	fig|6666666.59344.peg.3924	fig|6666666.59345.peg.5714	fig|6666666.59341.peg.4652	fig|6666666.59347.peg.5033	fig|6666666.59346.peg.4264	fig|6666666.59348.peg.23	fig|6666666.59349.peg.2231	None	fig|6666666.59351.peg.882	fig|6666666.59353.peg.4762	fig|6666666.59355.peg.4042	fig|6666666.59354.peg.4575

10135	Protein Implicated in DNA repair function with RecA and MutS	fig|6666666.59329.peg.5901	fig|6666666.59330.peg.4221	None	fig|6666666.59332.peg.208	fig|6666666.59334.peg.279	fig|6666666.59335.peg.260	fig|6666666.59336.peg.3449	fig|6666666.59333.peg.1719	fig|6666666.59338.peg.762	fig|6666666.59339.peg.8119	fig|6666666.59340.peg.1921	fig|6666666.59337.peg.587	fig|6666666.59342.peg.3966	fig|6666666.71181.peg.3473	fig|6666666.59344.peg.2171	fig|6666666.59345.peg.5077	None	fig|6666666.59347.peg.3229	fig|6666666.59346.peg.3267	fig|6666666.59348.peg.1097	fig|6666666.59349.peg.3776	fig|6666666.59350.peg.4989	fig|6666666.59351.peg.3384	fig|6666666.59353.peg.228	fig|6666666.59355.peg.2999	fig|6666666.59354.peg.32

6547	Acetyl-coenzyme A carboxyl transferase alpha chain EC 6.4.1.2	None	fig|6666666.59330.peg.452	fig|6666666.59331.peg.5543	fig|6666666.59332.peg.421	fig|6666666.59334.peg.4623	fig|6666666.59335.peg.2668	fig|6666666.59336.peg.3373	fig|6666666.59333.peg.1150	fig|6666666.59338.peg.2438	fig|6666666.59339.peg.5132	fig|6666666.59340.peg.373	fig|6666666.59337.peg.1598	fig|6666666.59342.peg.2437	fig|6666666.71181.peg.19	fig|6666666.59344.peg.2670	fig|6666666.59345.peg.2185	None	fig|6666666.59347.peg.4374	fig|6666666.59346.peg.4882	fig|6666666.59348.peg.633	fig|6666666.59349.peg.1359	fig|6666666.59350.peg.3578	fig|6666666.59351.peg.2131	fig|6666666.59353.peg.2001	fig|6666666.59355.peg.4106	fig|6666666.59354.peg.1416

6157	Phosphate acyl-ACP acyltransferase PlsX	fig|6666666.59329.peg.5963	fig|6666666.59330.peg.3619	fig|6666666.59331.peg.832	fig|6666666.59332.peg.3982	None	fig|6666666.59335.peg.2047	fig|6666666.59336.peg.6100	fig|6666666.59333.peg.1595	fig|6666666.59338.peg.4647	fig|6666666.59339.peg.3174	fig|6666666.59340.peg.2711	fig|6666666.59337.peg.2691	fig|6666666.59342.peg.1387	fig|6666666.71181.peg.1620	fig|6666666.59344.peg.2430	fig|6666666.59345.peg.1066	fig|6666666.59341.peg.13	fig|6666666.59347.peg.4082	fig|6666666.59346.peg.104	None	fig|6666666.59349.peg.3907	fig|6666666.59350.peg.3678	fig|6666666.59351.peg.670	fig|6666666.59353.peg.311	fig|6666666.59355.peg.1351	fig|6666666.59354.peg.2155

4833	Glucose-6-phosphate isomerase EC 5.3.1.9	fig|6666666.59329.peg.1860	fig|6666666.59330.peg.2648	None	fig|6666666.59332.peg.5930	fig|6666666.59334.peg.164	fig|6666666.59335.peg.3484	fig|6666666.59336.peg.605	fig|6666666.59333.peg.883	fig|6666666.59338.peg.6119	fig|6666666.59339.peg.7706	None	fig|6666666.59337.peg.1716	fig|6666666.59342.peg.3252	fig|6666666.71181.peg.2018	fig|6666666.59344.peg.2721	fig|6666666.59345.peg.5367	fig|6666666.59341.peg.2411	fig|6666666.59347.peg.5371	fig|6666666.59346.peg.3202	fig|6666666.59348.peg.1205	fig|6666666.59349.peg.659	fig|6666666.59350.peg.8351	fig|6666666.59351.peg.4928	fig|6666666.59353.peg.3014	fig|6666666.59355.peg.2922	fig|6666666.59354.peg.2926

8301	Dihydroorotate dehydrogenase EC 1.3.3.1	fig|6666666.59329.peg.6392	fig|6666666.59330.peg.3161	fig|6666666.59331.peg.4489	fig|6666666.59332.peg.4531	fig|6666666.59334.peg.3625	fig|6666666.59335.peg.1232	None	fig|6666666.59333.peg.5096	fig|6666666.59338.peg.5654	fig|6666666.59339.peg.7211	None	fig|6666666.59337.peg.4552	fig|6666666.59342.peg.4319	fig|6666666.71181.peg.4772	fig|6666666.59344.peg.1174	fig|6666666.59345.peg.3257	fig|6666666.59341.peg.905	fig|6666666.59347.peg.1770	fig|6666666.59346.peg.2050	fig|6666666.59348.peg.1672	fig|6666666.59349.peg.3357	fig|6666666.59350.peg.73	fig|6666666.59351.peg.3163	fig|6666666.59353.peg.1680	fig|6666666.59355.peg.1040	fig|6666666.59354.peg.713

2048	Transcription termination protein NusB	fig|6666666.59329.peg.6243	fig|6666666.59330.peg.1057	None	fig|6666666.59332.peg.4207	fig|6666666.59334.peg.2071	fig|6666666.59335.peg.155	fig|6666666.59336.peg.3172	fig|6666666.59333.peg.4710	fig|6666666.59338.peg.5266	fig|6666666.59339.peg.5097	fig|6666666.59340.peg.964	fig|6666666.59337.peg.1137	fig|6666666.59342.peg.3332	fig|6666666.71181.peg.2726	fig|6666666.59344.peg.1098	fig|6666666.59345.peg.3538	fig|6666666.59341.peg.2618	fig|6666666.59347.peg.2766	fig|6666666.59346.peg.3383	fig|6666666.59348.peg.927	fig|6666666.59349.peg.3485	fig|6666666.59350.peg.1710	fig|6666666.59351.peg.4247	fig|6666666.59353.peg.777	fig|6666666.59355.peg.927	None

10090	Leucyl phenylalanyl-tRNA--protein transferase EC 2.3.2.6	fig|6666666.59329.peg.2567	fig|6666666.59330.peg.1322	None	fig|6666666.59332.peg.2651	fig|6666666.59334.peg.876	fig|6666666.59335.peg.5949	fig|6666666.59336.peg.866	fig|6666666.59333.peg.1005	fig|6666666.59338.peg.5089	fig|6666666.59339.peg.289	None	fig|6666666.59337.peg.3368	fig|6666666.59342.peg.118	fig|6666666.71181.peg.4126	fig|6666666.59344.peg.404	fig|6666666.59345.peg.1551	fig|6666666.59341.peg.999	fig|6666666.59347.peg.4115	fig|6666666.59346.peg.2526	fig|6666666.59348.peg.1669	fig|6666666.59349.peg.2228	fig|6666666.59350.peg.3307	fig|6666666.59351.peg.880	fig|6666666.59353.peg.1624	fig|6666666.59355.peg.5550	fig|6666666.59354.peg.5345

6972	C1399 protein, clustered with ribosomal protein L32p	fig|6666666.59329.peg.5961	fig|6666666.59330.peg.3617	fig|6666666.59331.peg.830	fig|6666666.59332.peg.3984	fig|6666666.59334.peg.3324	fig|6666666.59335.peg.2044	fig|6666666.59336.peg.6098	fig|6666666.59333.peg.1597	fig|6666666.59338.peg.5765	fig|6666666.59339.peg.3172	None	fig|6666666.59337.peg.2693	fig|6666666.59342.peg.1389	fig|6666666.71181.peg.1618	fig|6666666.59344.peg.2428	fig|6666666.59345.peg.1068	fig|6666666.59341.peg.11	fig|6666666.59347.peg.4080	fig|6666666.59346.peg.102	fig|6666666.59348.peg.4430	fig|6666666.59349.peg.3905	None	fig|6666666.59351.peg.668	fig|6666666.59353.peg.309	fig|6666666.59355.peg.1349	fig|6666666.59354.peg.2153

6979	Cytochrome c-type biogenesis protein CcmC, putative heme lyase for CcmE	fig|6666666.59329.peg.2316	fig|6666666.59330.peg.4379	fig|6666666.59331.peg.3839	fig|6666666.59332.peg.2659	fig|6666666.59334.peg.2183	fig|6666666.59335.peg.1248	fig|6666666.59336.peg.4939	fig|6666666.59333.peg.2593	fig|6666666.59338.peg.3959	fig|6666666.59339.peg.3027	None	fig|6666666.59337.peg.4221	None	fig|6666666.71181.peg.1529	fig|6666666.59344.peg.4978	fig|6666666.59345.peg.5314	fig|6666666.59341.peg.3756	fig|6666666.59347.peg.5091	fig|6666666.59346.peg.5179	fig|6666666.59348.peg.4627	fig|6666666.59349.peg.181	fig|6666666.59350.peg.2484	fig|6666666.59351.peg.2083	fig|6666666.59353.peg.4867	fig|6666666.59355.peg.213	fig|6666666.59354.peg.5196

4388	FIG004453 protein YceG like	fig|6666666.59329.peg.2501	fig|6666666.59330.peg.3918	None	fig|6666666.59332.peg.2353	fig|6666666.59334.peg.5442	fig|6666666.59335.peg.5838	fig|6666666.59336.peg.5859	fig|6666666.59333.peg.2615	fig|6666666.59338.peg.3743	fig|6666666.59339.peg.3183	fig|6666666.59340.peg.499	fig|6666666.59337.peg.2683	None	fig|6666666.71181.peg.1627	fig|6666666.59344.peg.5036	fig|6666666.59345.peg.1059	fig|6666666.59341.peg.20	fig|6666666.59347.peg.4089	fig|6666666.59346.peg.111	fig|6666666.59348.peg.5436	fig|6666666.59349.peg.3915	fig|6666666.59350.peg.2243	fig|6666666.59351.peg.676	fig|6666666.59353.peg.318	fig|6666666.59355.peg.5886	fig|6666666.59354.peg.6482

4385	Dethiobiotin synthetase EC 6.3.3.3	fig|6666666.59329.peg.5564	fig|6666666.59330.peg.1938	fig|6666666.59331.peg.5879	fig|6666666.59332.peg.4303	fig|6666666.59334.peg.5391	fig|6666666.59335.peg.613	fig|6666666.59336.peg.4953	fig|6666666.59333.peg.3149	fig|6666666.59338.peg.3904	fig|6666666.59339.peg.465	None	fig|6666666.59337.peg.4602	fig|6666666.59342.peg.2454	fig|6666666.71181.peg.2525	fig|6666666.59344.peg.3825	fig|6666666.59345.peg.1327	fig|6666666.59341.peg.476	fig|6666666.59347.peg.2386	fig|6666666.59346.peg.1568	fig|6666666.59348.peg.1073	fig|6666666.59349.peg.1617	None	fig|6666666.59351.peg.965	fig|6666666.59353.peg.2135	fig|6666666.59355.peg.2685	fig|6666666.59354.peg.1866

7856	Argininosuccinate synthase EC 6.3.4.5	fig|6666666.59329.peg.984	fig|6666666.59330.peg.1683	None	fig|6666666.59332.peg.3468	fig|6666666.59334.peg.1806	fig|6666666.59335.peg.4747	fig|6666666.59336.peg.1602	fig|6666666.59333.peg.1848	fig|6666666.59338.peg.5176	fig|6666666.59339.peg.8738	fig|6666666.59340.peg.3094	fig|6666666.59337.peg.1686	None	fig|6666666.71181.peg.4434	fig|6666666.59344.peg.1015	fig|6666666.59345.peg.5395	fig|6666666.59341.peg.955	fig|6666666.59347.peg.4931	fig|6666666.59346.peg.994	fig|6666666.59348.peg.3926	fig|6666666.59349.peg.1755	fig|6666666.59350.peg.1486	fig|6666666.59351.peg.2286	fig|6666666.59353.peg.2525	fig|6666666.59355.peg.6129	fig|6666666.59354.peg.483

1618	Nucleoside triphosphate pyrophosphohydrolase MazG EC 3.6.1.8	fig|6666666.59329.peg.2713	fig|6666666.59330.peg.2468	fig|6666666.59331.peg.2974	fig|6666666.59332.peg.195	fig|6666666.59334.peg.328	fig|6666666.59335.peg.1501	fig|6666666.59336.peg.2780	fig|6666666.59333.peg.4310	fig|6666666.59338.peg.2735	fig|6666666.59339.peg.7385	None	fig|6666666.59337.peg.573	None	fig|6666666.71181.peg.3769	fig|6666666.59344.peg.2236	fig|6666666.59345.peg.5065	fig|6666666.59341.peg.1324	fig|6666666.59347.peg.3168	fig|6666666.59346.peg.3154	fig|6666666.59348.peg.3647	fig|6666666.59349.peg.3468	fig|6666666.59350.peg.672	fig|6666666.59351.peg.2589	fig|6666666.59353.peg.179	fig|6666666.59355.peg.2947	fig|6666666.59354.peg.916

3826	FIG005080 Possible exported protein	fig|6666666.59329.peg.713	fig|6666666.59330.peg.29	fig|6666666.59331.peg.2675	fig|6666666.59332.peg.2170	fig|6666666.59334.peg.1308	fig|6666666.59335.peg.3031	fig|6666666.59336.peg.4293	fig|6666666.59333.peg.236	fig|6666666.59338.peg.2888	fig|6666666.59339.peg.7797	None	fig|6666666.59337.peg.814	fig|6666666.59342.peg.4997	fig|6666666.71181.peg.983	fig|6666666.59344.peg.1749	fig|6666666.59345.peg.4879	fig|6666666.59341.peg.889	fig|6666666.59347.peg.3111	fig|6666666.59346.peg.2134	fig|6666666.59348.peg.1634	fig|6666666.59349.peg.9	None	fig|6666666.59351.peg.3427	fig|6666666.59353.peg.2298	fig|6666666.59355.peg.1573	fig|6666666.59354.peg.3172

5005	hypothetical protein	fig|6666666.59329.peg.3820	fig|6666666.59330.peg.2333	fig|6666666.59331.peg.1174	fig|6666666.59332.peg.1382	fig|6666666.59334.peg.4759	fig|6666666.59335.peg.4835	fig|6666666.59336.peg.4503	fig|6666666.59333.peg.2221	fig|6666666.59338.peg.4354	None	fig|6666666.59340.peg.446	fig|6666666.59337.peg.3597	fig|6666666.59342.peg.3446	fig|6666666.71181.peg.2529	fig|6666666.59344.peg.4097	fig|6666666.59345.peg.1801	fig|6666666.59341.peg.614	fig|6666666.59347.peg.1658	fig|6666666.59346.peg.4928	fig|6666666.59348.peg.873	fig|6666666.59349.peg.3223	None	fig|6666666.59351.peg.3759	fig|6666666.59353.peg.2139	fig|6666666.59355.peg.2681	fig|6666666.59354.peg.446

2205	Lipoate synthase	fig|6666666.59329.peg.3583	fig|6666666.59330.peg.2030	None	fig|6666666.59332.peg.1670	None	fig|6666666.59335.peg.1762	fig|6666666.59336.peg.588	fig|6666666.59333.peg.5117	fig|6666666.59338.peg.3	fig|6666666.59339.peg.5197	fig|6666666.59340.peg.2380	fig|6666666.59337.peg.1095	fig|6666666.59342.peg.6090	fig|6666666.71181.peg.6109	fig|6666666.59344.peg.3339	fig|6666666.59345.peg.3501	fig|6666666.59341.peg.5012	fig|6666666.59347.peg.2911	fig|6666666.59346.peg.3288	fig|6666666.59348.peg.4158	fig|6666666.59349.peg.2772	fig|6666666.59350.peg.6764	fig|6666666.59351.peg.3617	fig|6666666.59353.peg.3720	fig|6666666.59355.peg.3735	fig|6666666.59354.peg.5642

425	Flagellar hook-basal body complex protein FliE	fig|6666666.59329.peg.6201	fig|6666666.59330.peg.1864	None	fig|6666666.59332.peg.4857	None	fig|6666666.59335.peg.387	fig|6666666.59336.peg.1448	fig|6666666.59333.peg.4264	fig|6666666.59338.peg.5181	fig|6666666.59339.peg.7102	fig|6666666.59340.peg.1712	fig|6666666.59337.peg.1881	fig|6666666.59342.peg.2666	fig|6666666.71181.peg.4029	fig|6666666.59344.peg.5217	fig|6666666.59345.peg.2861	fig|6666666.59341.peg.141	fig|6666666.59347.peg.4627	fig|6666666.59346.peg.4717	fig|6666666.59348.peg.3871	fig|6666666.59349.peg.213	fig|6666666.59350.peg.6220	fig|6666666.59351.peg.2044	fig|6666666.59353.peg.4564	fig|6666666.59355.peg.3828	fig|6666666.59354.peg.1069

3782	FIG004684 SpoVR-like protein	fig|6666666.59329.peg.3293	fig|6666666.59330.peg.1259	fig|6666666.59331.peg.6237	fig|6666666.59332.peg.5422	None	fig|6666666.59335.peg.2828	fig|6666666.59336.peg.3390	fig|6666666.59333.peg.1836	fig|6666666.59338.peg.4945	fig|6666666.59339.peg.3479	fig|6666666.59340.peg.2490	fig|6666666.59337.peg.2203	None	fig|6666666.71181.peg.2951	fig|6666666.59344.peg.2871	fig|6666666.59345.peg.1586	fig|6666666.59341.peg.3939	fig|6666666.59347.peg.4167	fig|6666666.59346.peg.4459	fig|6666666.59348.peg.1980	fig|6666666.59349.peg.2397	fig|6666666.59350.peg.4529	fig|6666666.59351.peg.633	fig|6666666.59353.peg.1453	fig|6666666.59355.peg.2002	fig|6666666.59354.peg.1507

3781	Uncharacterized protein YrbK clustered with lipopolysaccharide transporters	fig|6666666.59329.peg.820	fig|6666666.59330.peg.48	None	fig|6666666.59332.peg.5622	fig|6666666.59334.peg.98	fig|6666666.59335.peg.3049	fig|6666666.59336.peg.352	fig|6666666.59333.peg.265	fig|6666666.59338.peg.109	fig|6666666.59339.peg.8199	fig|6666666.59340.peg.1368	fig|6666666.59337.peg.832	fig|6666666.59342.peg.5644	fig|6666666.71181.peg.1019	fig|6666666.59344.peg.1799	fig|6666666.59345.peg.4858	fig|6666666.59341.peg.1245	fig|6666666.59347.peg.3250	fig|6666666.59346.peg.637	fig|6666666.59348.peg.2007	fig|6666666.59349.peg.23	None	fig|6666666.59351.peg.3443	fig|6666666.59353.peg.2337	fig|6666666.59355.peg.1611	fig|6666666.59354.peg.5888

1237	3-isopropylmalate dehydratase small subunit EC 4.2.1.33	fig|6666666.59329.peg.122	fig|6666666.59330.peg.812	fig|6666666.59331.peg.2384	fig|6666666.59332.peg.5762	fig|6666666.59334.peg.342	fig|6666666.59335.peg.5238	fig|6666666.59336.peg.3670	fig|6666666.59333.peg.125	fig|6666666.59338.peg.55	fig|6666666.59339.peg.7320	fig|6666666.59340.peg.2302	fig|6666666.59337.peg.1390	None	fig|6666666.71181.peg.381	fig|6666666.59344.peg.1502	fig|6666666.59345.peg.3890	fig|6666666.59341.peg.4411	fig|6666666.59347.peg.3082	fig|6666666.59346.peg.817	None	fig|6666666.59349.peg.323	fig|6666666.59350.peg.2985	fig|6666666.59351.peg.4	fig|6666666.59353.peg.118	fig|6666666.59355.peg.5903	fig|6666666.59354.peg.3463

1230	FIG002095 hypothetical protein	fig|6666666.59329.peg.2171	fig|6666666.59330.peg.4039	fig|6666666.59331.peg.1659	fig|6666666.59332.peg.4718	fig|6666666.59334.peg.4637	fig|6666666.59335.peg.1749	None	fig|6666666.59333.peg.2142	fig|6666666.59338.peg.19	fig|6666666.59339.peg.5216	fig|6666666.59340.peg.903	fig|6666666.59337.peg.1081	fig|6666666.59342.peg.1139	fig|6666666.71181.peg.3219	fig|6666666.59344.peg.4501	fig|6666666.59345.peg.3488	fig|6666666.59341.peg.4535	fig|6666666.59347.peg.2899	fig|6666666.59346.peg.2953	fig|6666666.59348.peg.1289	fig|6666666.59349.peg.4126	None	fig|6666666.59351.peg.3630	fig|6666666.59353.peg.2027	fig|6666666.59355.peg.4434	fig|6666666.59354.peg.1681

1239	membrane protein	fig|6666666.59329.peg.1404	fig|6666666.59330.peg.4471	None	fig|6666666.59332.peg.3087	fig|6666666.59334.peg.3083	fig|6666666.59335.peg.373	fig|6666666.59336.peg.2158	fig|6666666.59333.peg.3555	fig|6666666.59338.peg.3389	fig|6666666.59339.peg.7122	fig|6666666.59340.peg.1759	fig|6666666.59337.peg.5640	fig|6666666.59342.peg.2562	fig|6666666.71181.peg.4465	fig|6666666.59344.peg.1987	fig|6666666.59345.peg.2144	fig|6666666.59341.peg.1688	fig|6666666.59347.peg.2840	fig|6666666.59346.peg.3539	fig|6666666.59348.peg.3287	fig|6666666.59349.peg.2497	None	fig|6666666.59351.peg.4631	fig|6666666.59353.peg.2933	fig|6666666.59355.peg.2807	fig|6666666.59354.peg.4786

6502	Catalase EC 1.11.1.6 Peroxidase EC 1.11.1.7	fig|6666666.59329.peg.4034	None	fig|6666666.59331.peg.5179	fig|6666666.59332.peg.958	fig|6666666.59334.peg.3328	fig|6666666.59335.peg.5715	fig|6666666.59336.peg.365	fig|6666666.59333.peg.3111	fig|6666666.59338.peg.6048	fig|6666666.59339.peg.2301	None	fig|6666666.59337.peg.2832	fig|6666666.59342.peg.4331	fig|6666666.71181.peg.1717	fig|6666666.59344.peg.5237	fig|6666666.59345.peg.1371	fig|6666666.59341.peg.3591	fig|6666666.59347.peg.371	fig|6666666.59346.peg.4352	fig|6666666.59348.peg.5264	fig|6666666.59349.peg.4033	fig|6666666.59350.peg.4373	fig|6666666.59351.peg.4957	fig|6666666.59353.peg.3697	fig|6666666.59355.peg.3019	fig|6666666.59354.peg.4560

4502	GTP cyclohydrolase I EC 3.5.4.16 type 1	fig|6666666.59329.peg.1811	fig|6666666.59330.peg.1140	fig|6666666.59331.peg.6255	fig|6666666.59332.peg.2304	fig|6666666.59334.peg.4963	fig|6666666.59335.peg.3118	fig|6666666.59336.peg.6133	fig|6666666.59333.peg.5408	fig|6666666.59338.peg.4081	fig|6666666.59339.peg.8494	fig|6666666.59340.peg.3703	fig|6666666.59337.peg.3639	None	fig|6666666.71181.peg.190	fig|6666666.59344.peg.231	fig|6666666.59345.peg.5209	fig|6666666.59341.peg.1540	fig|6666666.59347.peg.694	fig|6666666.59346.peg.1794	fig|6666666.59348.peg.3772	fig|6666666.59349.peg.4417	None	fig|6666666.59351.peg.4733	fig|6666666.59353.peg.632	fig|6666666.59355.peg.115	fig|6666666.59354.peg.2034

2083	Chorismate mutase I EC 5.4.99.5 Prephenate dehydratase EC 4.2.1.51	fig|6666666.59329.peg.2739	fig|6666666.59330.peg.2890	fig|6666666.59331.peg.5847	fig|6666666.59332.peg.1321	fig|6666666.59334.peg.858	fig|6666666.59335.peg.2064	fig|6666666.59336.peg.841	fig|6666666.59333.peg.4107	fig|6666666.59338.peg.3256	fig|6666666.59339.peg.7511	fig|6666666.59340.peg.2739	fig|6666666.59337.peg.155	None	fig|6666666.71181.peg.3947	fig|6666666.59344.peg.5432	fig|6666666.59345.peg.5678	fig|6666666.59341.peg.3004	fig|6666666.59347.peg.4053	fig|6666666.59346.peg.265	fig|6666666.59348.peg.4136	fig|6666666.59349.peg.2709	None	fig|6666666.59351.peg.546	fig|6666666.59353.peg.4804	fig|6666666.59355.peg.4858	fig|6666666.59354.peg.6102

7780	Putative cytochrome d ubiquinol oxidase subunit III EC 1.10.3.- Cytochrome bd-I oxidase subunit III	fig|6666666.59329.peg.2743	fig|6666666.59330.peg.959	fig|6666666.59331.peg.1475	fig|6666666.59332.peg.653	fig|6666666.59334.peg.400	fig|6666666.59335.peg.3439	fig|6666666.59336.peg.1585	fig|6666666.59333.peg.622	fig|6666666.59338.peg.4445	fig|6666666.59339.peg.4936	fig|6666666.59340.peg.1041	fig|6666666.59337.peg.25	fig|6666666.59342.peg.5737	fig|6666666.71181.peg.1037	fig|6666666.59344.peg.1820	fig|6666666.59345.peg.3817	fig|6666666.59341.peg.3551	fig|6666666.59347.peg.3918	fig|6666666.59346.peg.2340	None	fig|6666666.59349.peg.37	None	fig|6666666.59351.peg.3457	fig|6666666.59353.peg.2356	fig|6666666.59355.peg.1629	fig|6666666.59354.peg.353

11016	FIG01200735 hypothetical protein	fig|6666666.59329.peg.3152	fig|6666666.59330.peg.1766	None	fig|6666666.59332.peg.248	fig|6666666.59334.peg.892	fig|6666666.59335.peg.1524	fig|6666666.59336.peg.564	fig|6666666.59333.peg.325	fig|6666666.59338.peg.2993	fig|6666666.59339.peg.4791	fig|6666666.59340.peg.2041	fig|6666666.59337.peg.635	fig|6666666.59342.peg.4936	fig|6666666.71181.peg.2856	fig|6666666.59344.peg.4421	fig|6666666.59345.peg.2397	fig|6666666.59341.peg.5197	fig|6666666.59347.peg.3158	fig|6666666.59346.peg.750	fig|6666666.59348.peg.4965	fig|6666666.59349.peg.4064	None	fig|6666666.59351.peg.3406	fig|6666666.59353.peg.3208	fig|6666666.59355.peg.3090	fig|6666666.59354.peg.6414

9274	RecA protein	fig|6666666.59329.peg.4356	fig|6666666.59330.peg.4219	fig|6666666.59331.peg.5248	fig|6666666.59332.peg.2074	fig|6666666.59334.peg.280	fig|6666666.59335.peg.261	fig|6666666.59336.peg.3451	fig|6666666.59333.peg.1720	fig|6666666.59338.peg.761	fig|6666666.59339.peg.8118	None	fig|6666666.59337.peg.589	fig|6666666.59342.peg.3967	fig|6666666.71181.peg.3472	fig|6666666.59344.peg.2170	fig|6666666.59345.peg.5078	fig|6666666.59341.peg.4215	fig|6666666.59347.peg.3228	fig|6666666.59346.peg.3266	None	fig|6666666.59349.peg.3775	fig|6666666.59350.peg.153	fig|6666666.59351.peg.3383	fig|6666666.59353.peg.229	fig|6666666.59355.peg.3000	fig|6666666.59354.peg.33

7817	N-acetylglucosamine-1-phosphate uridyltransferase EC 2.7.7.23 Glucosamine-1-phosphate N-acetyltransferase EC 2.3.1.157	fig|6666666.59329.peg.1029	fig|6666666.59330.peg.615	None	fig|6666666.59332.peg.108	fig|6666666.59334.peg.16	fig|6666666.59335.peg.800	fig|6666666.59336.peg.38	fig|6666666.59333.peg.20	fig|6666666.59338.peg.501	fig|6666666.59339.peg.8003	None	fig|6666666.59337.peg.899	fig|6666666.59342.peg.5131	fig|6666666.71181.peg.600	fig|6666666.59344.peg.4274	fig|6666666.59345.peg.4957	fig|6666666.59341.peg.2131	fig|6666666.59347.peg.4775	fig|6666666.59346.peg.1411	fig|6666666.59348.peg.342	fig|6666666.59349.peg.1038	fig|6666666.59350.peg.1914	fig|6666666.59351.peg.2486	fig|6666666.59353.peg.3078	fig|6666666.59355.peg.2269	fig|6666666.59354.peg.4763

2140	FIG009095 D,D-carboxypeptidase family protein	fig|6666666.59329.peg.4216	fig|6666666.59330.peg.4477	fig|6666666.59331.peg.4989	fig|6666666.59332.peg.3080	None	fig|6666666.59335.peg.354	fig|6666666.59336.peg.2165	fig|6666666.59333.peg.5427	fig|6666666.59338.peg.778	fig|6666666.59339.peg.7142	fig|6666666.59340.peg.641	fig|6666666.59337.peg.5607	fig|6666666.59342.peg.3260	fig|6666666.71181.peg.4370	fig|6666666.59344.peg.5671	fig|6666666.59345.peg.2151	fig|6666666.59341.peg.1679	fig|6666666.59347.peg.2846	fig|6666666.59346.peg.3546	fig|6666666.59348.peg.3292	fig|6666666.59349.peg.4795	None	fig|6666666.59351.peg.2815	fig|6666666.59353.peg.4909	fig|6666666.59355.peg.4979	fig|6666666.59354.peg.5827

5585	Putative TEGT family carrier transport protein	fig|6666666.59329.peg.3253	fig|6666666.59330.peg.1034	None	fig|6666666.59332.peg.3261	fig|6666666.59334.peg.3985	fig|6666666.59335.peg.3578	fig|6666666.59336.peg.6011	fig|6666666.59333.peg.2076	fig|6666666.59338.peg.2700	fig|6666666.59339.peg.1563	None	fig|6666666.59337.peg.3175	fig|6666666.59342.peg.4047	fig|6666666.71181.peg.278	fig|6666666.59344.peg.4105	fig|6666666.59345.peg.3278	fig|6666666.59341.peg.4207	fig|6666666.59347.peg.1794	fig|6666666.59346.peg.4462	fig|6666666.59348.peg.5545	fig|6666666.59349.peg.4841	fig|6666666.59350.peg.1401	fig|6666666.59351.peg.3132	fig|6666666.59353.peg.2146	fig|6666666.59355.peg.1012	fig|6666666.59354.peg.5879

8335	Coproporphyrinogen III oxidase, aerobic EC 1.3.3.3	None	fig|6666666.59330.peg.654	None	fig|6666666.59332.peg.5272	fig|6666666.59334.peg.1517	fig|6666666.59335.peg.5010	fig|6666666.59336.peg.1983	fig|6666666.59333.peg.1364	fig|6666666.59338.peg.2070	fig|6666666.59339.peg.8104	fig|6666666.59340.peg.877	fig|6666666.59337.peg.3910	fig|6666666.59342.peg.4877	fig|6666666.71181.peg.4469	fig|6666666.59344.peg.1983	fig|6666666.59345.peg.4918	fig|6666666.59341.peg.5018	fig|6666666.59347.peg.4739	fig|6666666.59346.peg.1833	fig|6666666.59348.peg.3238	fig|6666666.59349.peg.2824	fig|6666666.59350.peg.1939	fig|6666666.59351.peg.5063	fig|6666666.59353.peg.2937	fig|6666666.59355.peg.2802	fig|6666666.59354.peg.4817

4564	Thiosulfate sulfurtransferase GlpE EC 2.8.1.1	fig|6666666.59329.peg.773	fig|6666666.59330.peg.1568	fig|6666666.59331.peg.4819	fig|6666666.59332.peg.2374	fig|6666666.59334.peg.1295	fig|6666666.59335.peg.4267	fig|6666666.59336.peg.1375	fig|6666666.59333.peg.1953	fig|6666666.59338.peg.529	fig|6666666.59339.peg.8571	None	fig|6666666.59337.peg.1652	fig|6666666.59342.peg.2199	fig|6666666.71181.peg.4490	fig|6666666.59344.peg.3630	fig|6666666.59345.peg.5468	fig|6666666.59341.peg.5289	fig|6666666.59347.peg.5052	fig|6666666.59346.peg.1641	fig|6666666.59348.peg.586	fig|6666666.59349.peg.2783	None	fig|6666666.59351.peg.5209	fig|6666666.59353.peg.2182	fig|6666666.59355.peg.1876	fig|6666666.59354.peg.2866

6060	UPF0246 protein YaaA	fig|6666666.59329.peg.119	fig|6666666.59330.peg.1732	None	fig|6666666.59332.peg.3055	fig|6666666.59334.peg.5047	fig|6666666.59335.peg.3634	fig|6666666.59336.peg.6244	fig|6666666.59333.peg.5334	fig|6666666.59338.peg.3450	fig|6666666.59339.peg.5008	None	fig|6666666.59337.peg.101	fig|6666666.59342.peg.1354	fig|6666666.71181.peg.433	fig|6666666.59344.peg.1336	fig|6666666.59345.peg.3741	fig|6666666.59341.peg.3456	fig|6666666.59347.peg.3051	fig|6666666.59346.peg.4037	fig|6666666.59348.peg.2221	fig|6666666.59349.peg.4228	fig|6666666.59350.peg.619	fig|6666666.59351.peg.3907	fig|6666666.59353.peg.65	fig|6666666.59355.peg.672	fig|6666666.59354.peg.2748

4091	Arsenate reductase EC 1.20.4.1	fig|6666666.59329.peg.4265	fig|6666666.59330.peg.3634	None	fig|6666666.59332.peg.2795	fig|6666666.59334.peg.380	fig|6666666.59335.peg.1191	fig|6666666.59336.peg.1609	fig|6666666.59333.peg.3279	fig|6666666.59338.peg.790	fig|6666666.59339.peg.28	fig|6666666.59340.peg.1649	fig|6666666.59337.peg.4493	fig|6666666.59342.peg.2938	fig|6666666.71181.peg.3267	fig|6666666.59344.peg.4023	fig|6666666.59345.peg.2160	fig|6666666.59341.peg.1671	fig|6666666.59347.peg.2855	fig|6666666.59346.peg.2499	None	fig|6666666.59349.peg.651	fig|6666666.59350.peg.72	fig|6666666.59351.peg.4921	fig|6666666.59353.peg.4028	fig|6666666.59355.peg.3986	fig|6666666.59354.peg.2916

4097	Succinate dehydrogenase hydrophobic membrane anchor protein	fig|6666666.59329.peg.162	fig|6666666.59330.peg.2770	fig|6666666.59331.peg.71	fig|6666666.59332.peg.4295	fig|6666666.59334.peg.3837	fig|6666666.59335.peg.2741	fig|6666666.59336.peg.5303	fig|6666666.59333.peg.898	fig|6666666.59338.peg.4266	fig|6666666.59339.peg.7195	None	fig|6666666.59337.peg.4107	fig|6666666.59342.peg.474	fig|6666666.71181.peg.3233	fig|6666666.59344.peg.4598	fig|6666666.59345.peg.4324	fig|6666666.59341.peg.2530	fig|6666666.59347.peg.4383	fig|6666666.59346.peg.2444	fig|6666666.59348.peg.2399	fig|6666666.59349.peg.2518	None	fig|6666666.59351.peg.3724	fig|6666666.59353.peg.2084	fig|6666666.59355.peg.2741	fig|6666666.59354.peg.1305

10803	Glutaminase EC 3.5.1.2	fig|6666666.59329.peg.1537	fig|6666666.59330.peg.335	fig|6666666.59331.peg.6353	fig|6666666.59332.peg.1636	fig|6666666.59334.peg.1372	fig|6666666.59335.peg.4696	fig|6666666.59336.peg.4099	fig|6666666.59333.peg.759	fig|6666666.59338.peg.2885	fig|6666666.59339.peg.7811	None	fig|6666666.59337.peg.516	None	fig|6666666.71181.peg.1873	fig|6666666.59344.peg.4349	fig|6666666.59345.peg.5004	fig|6666666.59341.peg.3063	fig|6666666.59347.peg.3874	fig|6666666.59346.peg.4579	fig|6666666.59348.peg.231	fig|6666666.59349.peg.2804	fig|6666666.59350.peg.5969	fig|6666666.59351.peg.3563	fig|6666666.59353.peg.3239	fig|6666666.59355.peg.3122	fig|6666666.59354.peg.4906

3299	cytosolic long-chain acyl-CoA thioester hydrolase family protein	fig|6666666.59329.peg.5752	fig|6666666.59330.peg.3517	fig|6666666.59331.peg.5462	fig|6666666.59332.peg.3310	None	fig|6666666.59335.peg.5261	fig|6666666.59336.peg.5349	fig|6666666.59333.peg.1918	fig|6666666.59338.peg.5325	fig|6666666.59339.peg.4309	fig|6666666.59340.peg.3260	fig|6666666.59337.peg.2432	fig|6666666.59342.peg.4062	fig|6666666.71181.peg.2784	fig|6666666.59344.peg.446	fig|6666666.59345.peg.926	fig|6666666.59341.peg.3419	fig|6666666.59347.peg.1463	fig|6666666.59346.peg.4963	fig|6666666.59348.peg.2349	fig|6666666.59349.peg.1591	None	fig|6666666.59351.peg.1077	fig|6666666.59353.peg.1767	fig|6666666.59355.peg.1245	fig|6666666.59354.peg.2512

6897	Iojap protein	fig|6666666.59329.peg.2163	fig|6666666.59330.peg.4796	fig|6666666.59331.peg.5579	None	fig|6666666.59334.peg.2315	fig|6666666.59335.peg.1753	fig|6666666.59336.peg.578	fig|6666666.59333.peg.2136	fig|6666666.59338.peg.13	fig|6666666.59339.peg.5209	None	fig|6666666.59337.peg.1085	fig|6666666.59342.peg.907	fig|6666666.71181.peg.4763	fig|6666666.59344.peg.4493	fig|6666666.59345.peg.3492	fig|6666666.59341.peg.4748	fig|6666666.59347.peg.2903	fig|6666666.59346.peg.3300	fig|6666666.59348.peg.846	fig|6666666.59349.peg.2762	fig|6666666.59350.peg.2411	fig|6666666.59351.peg.3625	fig|6666666.59353.peg.2022	fig|6666666.59355.peg.4429	fig|6666666.59354.peg.2322

8249	Paraquat-inducible protein A	fig|6666666.59329.peg.5557	fig|6666666.59330.peg.1375	fig|6666666.59331.peg.2088	fig|6666666.59332.peg.5116	fig|6666666.59334.peg.4418	fig|6666666.59335.peg.4833	fig|6666666.59336.peg.4039	fig|6666666.59333.peg.748	fig|6666666.59338.peg.2721	fig|6666666.59339.peg.678	None	fig|6666666.59337.peg.3160	fig|6666666.59342.peg.778	fig|6666666.71181.peg.256	fig|6666666.59344.peg.1180	fig|6666666.59345.peg.3265	fig|6666666.59341.peg.4227	fig|6666666.59347.peg.1778	fig|6666666.59346.peg.4867	fig|6666666.59348.peg.4886	fig|6666666.59349.peg.4423	None	fig|6666666.59351.peg.3741	fig|6666666.59353.peg.1266	fig|6666666.59355.peg.1035	fig|6666666.59354.peg.2306

7590	Frataxin homolog CyaY, facilitates iron supply for heme A synthesis or Fe-S cluster assembly	fig|6666666.59329.peg.92	fig|6666666.59330.peg.3050	None	fig|6666666.59332.peg.1707	fig|6666666.59334.peg.1133	fig|6666666.59335.peg.3334	fig|6666666.59336.peg.1119	fig|6666666.59333.peg.788	fig|6666666.59338.peg.1779	fig|6666666.59339.peg.8521	fig|6666666.59340.peg.791	fig|6666666.59337.peg.1615	fig|6666666.59342.peg.3156	fig|6666666.71181.peg.2434	fig|6666666.59344.peg.2485	fig|6666666.59345.peg.5504	fig|6666666.59341.peg.3917	fig|6666666.59347.peg.5078	fig|6666666.59346.peg.598	fig|6666666.59348.peg.3774	fig|6666666.59349.peg.1560	None	fig|6666666.59351.peg.4753	fig|6666666.59353.peg.3633	fig|6666666.59355.peg.4991	fig|6666666.59354.peg.1457

7910	Ribose 5-phosphate isomerase A EC 5.3.1.6	fig|6666666.59329.peg.242	fig|6666666.59330.peg.366	None	fig|6666666.59332.peg.538	fig|6666666.59334.peg.1051	fig|6666666.59335.peg.1733	fig|6666666.59336.peg.1192	fig|6666666.59333.peg.4182	fig|6666666.59338.peg.5636	fig|6666666.59339.peg.7344	None	fig|6666666.59337.peg.547	fig|6666666.59342.peg.2808	fig|6666666.71181.peg.3134	fig|6666666.59344.peg.4735	fig|6666666.59345.peg.5036	fig|6666666.59341.peg.3931	fig|6666666.59347.peg.3196	fig|6666666.59346.peg.315	fig|6666666.59348.peg.274	fig|6666666.59349.peg.350	fig|6666666.59350.peg.2122	fig|6666666.59351.peg.3956	fig|6666666.59353.peg.4433	fig|6666666.59355.peg.4513	fig|6666666.59354.peg.1492

2594	Metal-dependent hydrolase YbeY, involved in rRNA and or ribosome maturation and assembly	fig|6666666.59329.peg.5003	fig|6666666.59330.peg.4036	fig|6666666.59331.peg.1656	None	fig|6666666.59334.peg.3139	fig|6666666.59335.peg.1744	fig|6666666.59336.peg.2832	fig|6666666.59333.peg.2145	fig|6666666.59338.peg.2013	fig|6666666.59339.peg.5220	fig|6666666.59340.peg.2709	fig|6666666.59337.peg.1075	fig|6666666.59342.peg.1136	fig|6666666.71181.peg.3210	fig|6666666.59344.peg.4513	fig|6666666.59345.peg.3485	fig|6666666.59341.peg.1637	fig|6666666.59347.peg.4449	fig|6666666.59346.peg.2958	fig|6666666.59348.peg.3124	fig|6666666.59349.peg.4519	fig|6666666.59350.peg.5296	fig|6666666.59351.peg.4673	fig|6666666.59353.peg.2036	fig|6666666.59355.peg.4443	None

4300	Hypothetical protein YggS, proline synthase co-transcribed bacterial homolog PROSC	None	fig|6666666.59330.peg.342	None	fig|6666666.59332.peg.1628	fig|6666666.59334.peg.2561	fig|6666666.59335.peg.3089	fig|6666666.59336.peg.3070	fig|6666666.59333.peg.767	fig|6666666.59338.peg.4440	fig|6666666.59339.peg.7826	fig|6666666.59340.peg.1034	fig|6666666.59337.peg.523	fig|6666666.59342.peg.2685	fig|6666666.71181.peg.1865	fig|6666666.59344.peg.4339	fig|6666666.59345.peg.5013	fig|6666666.59341.peg.5679	fig|6666666.59347.peg.3865	fig|6666666.59346.peg.1780	fig|6666666.59348.peg.223	fig|6666666.59349.peg.800	fig|6666666.59350.peg.6053	fig|6666666.59351.peg.3530	fig|6666666.59353.peg.3230	fig|6666666.59355.peg.3113	fig|6666666.59354.peg.525

4701	tRNA- ms[2]io[6]A -hydroxylase EC 1.-.-.-	fig|6666666.59329.peg.1618	fig|6666666.59330.peg.1919	fig|6666666.59331.peg.1624	fig|6666666.59332.peg.3345	fig|6666666.59334.peg.1071	fig|6666666.59335.peg.3375	None	fig|6666666.59333.peg.2401	fig|6666666.59338.peg.6055	fig|6666666.59339.peg.3117	fig|6666666.59340.peg.2648	fig|6666666.59337.peg.4856	fig|6666666.59342.peg.4927	fig|6666666.71181.peg.3296	fig|6666666.59344.peg.3965	fig|6666666.59345.peg.1345	fig|6666666.59341.peg.52	fig|6666666.59347.peg.2402	fig|6666666.59346.peg.4662	fig|6666666.59348.peg.840	fig|6666666.59349.peg.2078	None	fig|6666666.59351.peg.1027	fig|6666666.59353.peg.2436	fig|6666666.59355.peg.529	fig|6666666.59354.peg.3557

4688	Carbon starvation protein A	fig|6666666.59329.peg.1381	fig|6666666.59330.peg.2518	None	fig|6666666.59332.peg.1086	fig|6666666.59334.peg.2852	fig|6666666.59335.peg.1002	fig|6666666.59336.peg.5870	fig|6666666.59333.peg.2503	fig|6666666.59338.peg.4458	fig|6666666.59339.peg.7473	fig|6666666.59340.peg.1171	fig|6666666.59337.peg.139	fig|6666666.59342.peg.2141	fig|6666666.71181.peg.4209	fig|6666666.59344.peg.503	fig|6666666.59345.peg.3703	fig|6666666.59341.peg.97	fig|6666666.59347.peg.4037	fig|6666666.59346.peg.244	fig|6666666.59348.peg.501	fig|6666666.59349.peg.4165	None	fig|6666666.59351.peg.4272	fig|6666666.59353.peg.1818	fig|6666666.59355.peg.1198	fig|6666666.59354.peg.5458

9269	Electron transport complex protein RnfB	fig|6666666.59329.peg.5222	fig|6666666.59330.peg.1696	fig|6666666.59331.peg.4892	fig|6666666.59332.peg.5657	fig|6666666.59334.peg.4473	fig|6666666.59335.peg.889	fig|6666666.59336.peg.1329	fig|6666666.59333.peg.3623	None	fig|6666666.59339.peg.61	fig|6666666.59340.peg.2954	fig|6666666.59337.peg.5097	fig|6666666.59342.peg.1803	fig|6666666.71181.peg.4981	fig|6666666.59344.peg.3875	fig|6666666.59345.peg.1122	fig|6666666.59341.peg.5686	fig|6666666.59347.peg.5165	fig|6666666.59346.peg.5091	fig|6666666.59348.peg.93	fig|6666666.59349.peg.1856	None	fig|6666666.59351.peg.5078	fig|6666666.59353.peg.4000	fig|6666666.59355.peg.3917	fig|6666666.59354.peg.5667

6020	Cytochrome c oxidase polypeptide III EC 1.9.3.1	fig|6666666.59329.peg.5242	fig|6666666.59330.peg.3219	fig|6666666.59331.peg.607	fig|6666666.59332.peg.5161	fig|6666666.59334.peg.996	fig|6666666.59335.peg.2868	fig|6666666.59336.peg.3602	fig|6666666.59333.peg.3094	fig|6666666.59338.peg.3611	fig|6666666.59339.peg.6636	None	fig|6666666.59337.peg.1475	fig|6666666.59342.peg.6203	fig|6666666.71181.peg.5444	fig|6666666.59344.peg.1078	fig|6666666.59345.peg.369	fig|6666666.59341.peg.3235	fig|6666666.59347.peg.1550	fig|6666666.59346.peg.4307	None	fig|6666666.59349.peg.3506	fig|6666666.59350.peg.2027	fig|6666666.59351.peg.4386	fig|6666666.59353.peg.762	fig|6666666.59355.peg.5557	fig|6666666.59354.peg.2414

9215	Undecaprenyl-diphosphatase EC 3.6.1.27	fig|6666666.59329.peg.689	fig|6666666.59330.peg.3124	fig|6666666.59331.peg.2656	fig|6666666.59332.peg.1507	None	fig|6666666.59335.peg.1653	fig|6666666.59336.peg.3314	fig|6666666.59333.peg.181	fig|6666666.59338.peg.799	fig|6666666.59339.peg.7914	fig|6666666.59340.peg.2167	fig|6666666.59337.peg.1432	fig|6666666.59342.peg.4521	fig|6666666.71181.peg.3013	fig|6666666.59344.peg.4469	fig|6666666.59345.peg.3841	fig|6666666.59341.peg.4833	fig|6666666.59347.peg.3131	fig|6666666.59346.peg.3112	fig|6666666.59348.peg.3458	fig|6666666.59349.peg.1690	fig|6666666.59350.peg.3686	fig|6666666.59351.peg.5225	fig|6666666.59353.peg.4184	fig|6666666.59355.peg.1693	None

6612	Isopenicillin N synthase	fig|6666666.59329.peg.3499	fig|6666666.59330.peg.3928	fig|6666666.59331.peg.1925	fig|6666666.59332.peg.5338	None	fig|6666666.59335.peg.6106	fig|6666666.59336.peg.4319	fig|6666666.59333.peg.2330	fig|6666666.59338.peg.5882	fig|6666666.59339.peg.2839	None	fig|6666666.59337.peg.5670	fig|6666666.59342.peg.1670	fig|6666666.71181.peg.617	fig|6666666.59344.peg.326	fig|6666666.59345.peg.104	fig|6666666.59341.peg.6127	fig|6666666.59347.peg.837	fig|6666666.59346.peg.3465	fig|6666666.59348.peg.3746	fig|6666666.59349.peg.2666	fig|6666666.59350.peg.3892	fig|6666666.59351.peg.5160	fig|6666666.59353.peg.557	fig|6666666.59355.peg.42	fig|6666666.59354.peg.2882

1635	Uncharacterized protein conserved in bacteria, NMA0228-like	fig|6666666.59329.peg.2198	fig|6666666.59330.peg.989	None	fig|6666666.59332.peg.263	fig|6666666.59334.peg.2972	fig|6666666.59335.peg.6172	fig|6666666.59336.peg.4075	fig|6666666.59333.peg.1577	fig|6666666.59338.peg.2869	fig|6666666.59339.peg.7649	None	fig|6666666.59337.peg.2542	fig|6666666.59342.peg.2493	fig|6666666.71181.peg.2298	fig|6666666.59344.peg.4702	fig|6666666.59345.peg.1810	fig|6666666.59341.peg.2311	fig|6666666.59347.peg.5244	fig|6666666.59346.peg.922	fig|6666666.59348.peg.48	fig|6666666.59349.peg.2335	fig|6666666.59350.peg.3671	fig|6666666.59351.peg.2996	fig|6666666.59353.peg.4483	fig|6666666.59355.peg.4590	fig|6666666.59354.peg.6371

1636	Phosphatidate cytidylyltransferase EC 2.7.7.41	fig|6666666.59329.peg.1575	fig|6666666.59330.peg.463	None	fig|6666666.59332.peg.1585	fig|6666666.59334.peg.5275	fig|6666666.59335.peg.2656	fig|6666666.59336.peg.2561	fig|6666666.59333.peg.3055	fig|6666666.59338.peg.4899	fig|6666666.59339.peg.5113	fig|6666666.59340.peg.3364	fig|6666666.59337.peg.1586	fig|6666666.59342.peg.272	fig|6666666.71181.peg.29	fig|6666666.59344.peg.2659	fig|6666666.59345.peg.2196	fig|6666666.59341.peg.973	fig|6666666.59347.peg.4363	fig|6666666.59346.peg.2265	fig|6666666.59348.peg.3060	fig|6666666.59349.peg.1371	None	fig|6666666.59351.peg.2120	fig|6666666.59353.peg.1989	fig|6666666.59355.peg.4096	fig|6666666.59354.peg.1336

3504	Outer membrane lipoprotein SmpA, a component of the essential YaeT outer-membrane protein assembly complex	fig|6666666.59329.peg.3959	fig|6666666.59330.peg.4603	fig|6666666.59331.peg.1331	fig|6666666.59332.peg.380	None	fig|6666666.59335.peg.5169	fig|6666666.59336.peg.219	fig|6666666.59333.peg.2761	fig|6666666.59338.peg.1076	fig|6666666.59339.peg.7279	fig|6666666.59340.peg.2209	fig|6666666.59337.peg.5541	fig|6666666.59342.peg.850	fig|6666666.71181.peg.1442	fig|6666666.59344.peg.1543	fig|6666666.59345.peg.3578	fig|6666666.59341.peg.5537	fig|6666666.59347.peg.4326	fig|6666666.59346.peg.693	fig|6666666.59348.peg.4865	fig|6666666.59349.peg.3332	None	fig|6666666.59351.peg.612	fig|6666666.59353.peg.4357	fig|6666666.59355.peg.4486	fig|6666666.59354.peg.6255

2340	3-polyprenyl-4-hydroxybenzoate carboxy-lyase EC 4.1.1.-	fig|6666666.59329.peg.4167	fig|6666666.59330.peg.1006	fig|6666666.59331.peg.5143	fig|6666666.59332.peg.3583	fig|6666666.59334.peg.421	fig|6666666.59335.peg.4614	fig|6666666.59336.peg.1643	fig|6666666.59333.peg.3253	fig|6666666.59338.peg.1201	fig|6666666.59339.peg.7685	fig|6666666.59340.peg.2578	fig|6666666.59337.peg.2523	None	fig|6666666.71181.peg.3331	fig|6666666.59344.peg.3404	fig|6666666.59345.peg.5514	fig|6666666.59341.peg.4202	fig|6666666.59347.peg.5231	fig|6666666.59346.peg.4511	fig|6666666.59348.peg.3057	fig|6666666.59349.peg.1570	None	fig|6666666.59351.peg.4764	fig|6666666.59353.peg.3807	fig|6666666.59355.peg.3403	fig|6666666.59354.peg.1083

1502	Chaperone protein DnaJ	fig|6666666.59329.peg.1662	fig|6666666.59330.peg.4617	fig|6666666.59331.peg.5730	fig|6666666.59332.peg.389	fig|6666666.59334.peg.1381	fig|6666666.59335.peg.5647	fig|6666666.59336.peg.3609	fig|6666666.59333.peg.3460	fig|6666666.59338.peg.3968	fig|6666666.59339.peg.7268	None	fig|6666666.59337.peg.4974	None	fig|6666666.71181.peg.425	fig|6666666.59344.peg.1325	fig|6666666.59345.peg.3570	fig|6666666.59341.peg.5138	fig|6666666.59347.peg.4332	fig|6666666.59346.peg.4647	fig|6666666.59348.peg.1569	fig|6666666.59349.peg.4426	fig|6666666.59350.peg.6495	fig|6666666.59351.peg.1549	fig|6666666.59353.peg.2197	fig|6666666.59355.peg.1890	fig|6666666.59354.peg.5562

1504	LPS-assembly lipoprotein RlpB precursor Rare lipoprotein B	fig|6666666.59329.peg.2167	fig|6666666.59330.peg.4793	fig|6666666.59331.peg.5001	fig|6666666.59332.peg.5140	fig|6666666.59334.peg.4465	fig|6666666.59335.peg.1751	fig|6666666.59336.peg.576	fig|6666666.59333.peg.2140	fig|6666666.59338.peg.15	fig|6666666.59339.peg.5211	fig|6666666.59340.peg.2004	fig|6666666.59337.peg.1083	None	fig|6666666.71181.peg.4766	fig|6666666.59344.peg.4498	fig|6666666.59345.peg.3490	fig|6666666.59341.peg.4537	fig|6666666.59347.peg.2901	fig|6666666.59346.peg.2950	fig|6666666.59348.peg.844	fig|6666666.59349.peg.2759	None	fig|6666666.59351.peg.3628	fig|6666666.59353.peg.2025	fig|6666666.59355.peg.4432	fig|6666666.59354.peg.2325

3884	Biotin synthase EC 2.8.1.6	fig|6666666.59329.peg.5959	fig|6666666.59330.peg.1935	None	fig|6666666.59332.peg.5874	None	fig|6666666.59335.peg.616	fig|6666666.59336.peg.5101	fig|6666666.59333.peg.2049	fig|6666666.59338.peg.3569	fig|6666666.59339.peg.461	fig|6666666.59340.peg.3468	fig|6666666.59337.peg.4599	fig|6666666.59342.peg.4200	fig|6666666.71181.peg.2522	fig|6666666.59344.peg.4089	fig|6666666.59345.peg.1330	fig|6666666.59341.peg.473	fig|6666666.59347.peg.2389	fig|6666666.59346.peg.1572	fig|6666666.59348.peg.1077	fig|6666666.59349.peg.1621	fig|6666666.59350.peg.2935	fig|6666666.59351.peg.961	fig|6666666.59353.peg.2132	fig|6666666.59355.peg.2688	fig|6666666.59354.peg.3536

992	Exodeoxyribonuclease III EC 3.1.11.2	fig|6666666.59329.peg.2647	fig|6666666.59330.peg.1304	fig|6666666.59331.peg.2014	fig|6666666.59332.peg.2268	fig|6666666.59334.peg.4812	None	fig|6666666.59336.peg.6209	fig|6666666.59333.peg.1022	fig|6666666.59338.peg.1157	fig|6666666.59339.peg.444	None	fig|6666666.59337.peg.3385	fig|6666666.59342.peg.2768	fig|6666666.71181.peg.5939	fig|6666666.59344.peg.2905	fig|6666666.59345.peg.1569	fig|6666666.59341.peg.2241	fig|6666666.59347.peg.4183	fig|6666666.59346.peg.3841	fig|6666666.59348.peg.2152	fig|6666666.59349.peg.2947	fig|6666666.59350.peg.442	fig|6666666.59351.peg.2826	fig|6666666.59353.peg.1422	fig|6666666.59355.peg.1971	fig|6666666.59354.peg.5605

5097	Glutamyl-tRNA reductase EC 1.2.1.70	fig|6666666.59329.peg.5131	fig|6666666.59330.peg.4521	None	fig|6666666.59332.peg.6209	fig|6666666.59334.peg.2242	fig|6666666.59335.peg.1178	fig|6666666.59336.peg.5933	fig|6666666.59333.peg.233	fig|6666666.59338.peg.5313	fig|6666666.59339.peg.8	fig|6666666.59340.peg.3596	fig|6666666.59337.peg.5166	fig|6666666.59342.peg.463	fig|6666666.71181.peg.779	fig|6666666.59344.peg.109	fig|6666666.59345.peg.5416	fig|6666666.59341.peg.3043	fig|6666666.59347.peg.5280	fig|6666666.59346.peg.3190	fig|6666666.59348.peg.610	fig|6666666.59349.peg.2614	None	fig|6666666.59351.peg.4663	fig|6666666.59353.peg.431	fig|6666666.59355.peg.2158	fig|6666666.59354.peg.5215

4497	DNA recombination and repair protein RecO	fig|6666666.59329.peg.2797	fig|6666666.59330.peg.388	fig|6666666.59331.peg.4033	fig|6666666.59332.peg.189	fig|6666666.59334.peg.320	fig|6666666.59335.peg.1493	fig|6666666.59336.peg.2096	fig|6666666.59333.peg.2811	fig|6666666.59338.peg.4040	fig|6666666.59339.peg.7373	None	fig|6666666.59337.peg.567	None	fig|6666666.71181.peg.1421	fig|6666666.59344.peg.1568	fig|6666666.59345.peg.5058	fig|6666666.59341.peg.1331	fig|6666666.59347.peg.3174	fig|6666666.59346.peg.336	fig|6666666.59348.peg.647	fig|6666666.59349.peg.4321	fig|6666666.59350.peg.1618	fig|6666666.59351.peg.604	fig|6666666.59353.peg.2655	fig|6666666.59355.peg.1827	fig|6666666.59354.peg.2820

4987	2-aminoethylphosphonate pyruvate aminotransferase EC 2.6.1.37	fig|6666666.59329.peg.2984	fig|6666666.59330.peg.3914	fig|6666666.59331.peg.1156	fig|6666666.59332.peg.5207	fig|6666666.59334.peg.1602	fig|6666666.59335.peg.959	fig|6666666.59336.peg.3727	fig|6666666.59333.peg.2483	fig|6666666.59338.peg.3528	fig|6666666.59339.peg.4491	None	fig|6666666.59337.peg.786	fig|6666666.59342.peg.4805	fig|6666666.71181.peg.899	fig|6666666.59344.peg.4781	fig|6666666.59345.peg.2957	fig|6666666.59341.peg.1448	fig|6666666.59347.peg.3266	fig|6666666.59346.peg.1064	fig|6666666.59348.peg.1963	fig|6666666.59349.peg.2120	fig|6666666.59350.peg.6888	None	fig|6666666.59353.peg.4531	fig|6666666.59355.peg.3307	fig|6666666.59354.peg.4450

7473	Methylenetetrahydrofolate dehydrogenase NADP+ EC 1.5.1.5 Methenyltetrahydrofolate cyclohydrolase EC 3.5.4.9	None	fig|6666666.59330.peg.2493	fig|6666666.59331.peg.4581	fig|6666666.59332.peg.1206	fig|6666666.59334.peg.4985	fig|6666666.59335.peg.3286	fig|6666666.59336.peg.1012	fig|6666666.59333.peg.1108	fig|6666666.59338.peg.1141	fig|6666666.59339.peg.3068	fig|6666666.59340.peg.3385	fig|6666666.59337.peg.2760	fig|6666666.59342.peg.6007	fig|6666666.71181.peg.3630	fig|6666666.59344.peg.3381	fig|6666666.59345.peg.4288	fig|6666666.59341.peg.3862	fig|6666666.59347.peg.4419	fig|6666666.59346.peg.1467	fig|6666666.59348.peg.4605	fig|6666666.59349.peg.2732	None	fig|6666666.59351.peg.5098	fig|6666666.59353.peg.3756	fig|6666666.59355.peg.3773	fig|6666666.59354.peg.125

112	LppC putative lipoprotein	fig|6666666.59329.peg.3485	fig|6666666.59330.peg.972	fig|6666666.59331.peg.4495	fig|6666666.59332.peg.640	fig|6666666.59334.peg.2330	fig|6666666.59335.peg.4932	fig|6666666.59336.peg.712	fig|6666666.59333.peg.610	fig|6666666.59338.peg.539	fig|6666666.59339.peg.4953	fig|6666666.59340.peg.1421	fig|6666666.59337.peg.37	None	fig|6666666.71181.peg.4571	fig|6666666.59344.peg.1845	fig|6666666.59345.peg.3804	fig|6666666.59341.peg.1361	fig|6666666.59347.peg.3990	fig|6666666.59346.peg.2328	fig|6666666.59348.peg.4624	fig|6666666.59349.peg.50	None	fig|6666666.59351.peg.3470	fig|6666666.59353.peg.2376	fig|6666666.59355.peg.1648	fig|6666666.59354.peg.368

7643	Twin-arginine translocation protein TatC	fig|6666666.59329.peg.5194	fig|6666666.59330.peg.255	fig|6666666.59331.peg.2434	fig|6666666.59332.peg.11	fig|6666666.59334.peg.2868	fig|6666666.59335.peg.45	fig|6666666.59336.peg.896	None	fig|6666666.59338.peg.3941	fig|6666666.59339.peg.7576	None	fig|6666666.59337.peg.963	fig|6666666.59342.peg.5187	fig|6666666.71181.peg.3336	fig|6666666.59344.peg.3410	fig|6666666.59345.peg.2710	fig|6666666.59341.peg.159	fig|6666666.59347.peg.4639	fig|6666666.59346.peg.3361	fig|6666666.59348.peg.111	fig|6666666.59349.peg.3417	fig|6666666.59350.peg.1450	fig|6666666.59351.peg.4996	fig|6666666.59353.peg.3802	fig|6666666.59355.peg.3398	fig|6666666.59354.peg.2227

11177	Protein slyX	None	fig|6666666.59330.peg.4078	fig|6666666.59331.peg.4124	fig|6666666.59332.peg.1071	fig|6666666.59334.peg.435	fig|6666666.59335.peg.6126	fig|6666666.59336.peg.2186	fig|6666666.59333.peg.3109	fig|6666666.59338.peg.4002	fig|6666666.59339.peg.8866	fig|6666666.59340.peg.2693	fig|6666666.59337.peg.1817	fig|6666666.59342.peg.6264	fig|6666666.71181.peg.4307	fig|6666666.59344.peg.3125	fig|6666666.59345.peg.5558	fig|6666666.59341.peg.5786	fig|6666666.59347.peg.5338	fig|6666666.59346.peg.38	fig|6666666.59348.peg.3298	fig|6666666.59349.peg.3370	None	fig|6666666.59351.peg.4178	fig|6666666.59353.peg.2268	fig|6666666.59355.peg.1961	fig|6666666.59354.peg.1675

10709	Phosphoribosylformylglycinamidine synthase, synthetase subunit EC 6.3.5.3 Phosphoribosylformylglycinamidine synthase, glutamine amidotransferase subunit EC 6.3.5.3	fig|6666666.59329.peg.1869	fig|6666666.59330.peg.1077	None	fig|6666666.59332.peg.3751	None	fig|6666666.59335.peg.1702	fig|6666666.59336.peg.3996	fig|6666666.59333.peg.2832	fig|6666666.59338.peg.719	fig|6666666.59339.peg.7250	fig|6666666.59340.peg.3763	fig|6666666.59337.peg.5442	None	fig|6666666.71181.peg.4872	fig|6666666.59344.peg.1588	fig|6666666.59345.peg.3558	fig|6666666.59341.peg.1479	fig|6666666.59347.peg.4348	fig|6666666.59346.peg.1090	fig|6666666.59348.peg.2262	fig|6666666.59349.peg.2798	fig|6666666.59350.peg.1724	fig|6666666.59351.peg.3568	fig|6666666.59353.peg.2669	fig|6666666.59355.peg.1813	fig|6666666.59354.peg.2663

1172	Thiamin ABC transporter, ATPase component	fig|6666666.59329.peg.1476	fig|6666666.59330.peg.834	fig|6666666.59331.peg.2537	fig|6666666.59332.peg.2335	None	fig|6666666.59335.peg.189	fig|6666666.59336.peg.4763	fig|6666666.59333.peg.4956	fig|6666666.59338.peg.312	fig|6666666.59339.peg.7737	fig|6666666.59340.peg.3108	fig|6666666.59337.peg.1366	fig|6666666.59342.peg.2205	fig|6666666.71181.peg.388	fig|6666666.59344.peg.1511	fig|6666666.59345.peg.2477	fig|6666666.59341.peg.1234	fig|6666666.59347.peg.4877	fig|6666666.59346.peg.649	fig|6666666.59348.peg.1993	None	fig|6666666.59350.peg.7013	None	fig|6666666.59353.peg.111	fig|6666666.59355.peg.720	fig|6666666.59354.peg.2100

7459	S-adenosylmethionine synthetase EC 2.5.1.6	fig|6666666.59329.peg.5226	fig|6666666.59330.peg.352	fig|6666666.59331.peg.1128	None	fig|6666666.59334.peg.3899	fig|6666666.59335.peg.3101	fig|6666666.59336.peg.3964	fig|6666666.59333.peg.779	fig|6666666.59338.peg.4711	None	None	fig|6666666.59337.peg.533	fig|6666666.59342.peg.3319	fig|6666666.71181.peg.2829	fig|6666666.59344.peg.4792	fig|6666666.59345.peg.5023	fig|6666666.59341.peg.5884	fig|6666666.59347.peg.3208	fig|6666666.59346.peg.3514	fig|6666666.59348.peg.4377	fig|6666666.59349.peg.1871	fig|6666666.59350.peg.574	fig|6666666.59351.peg.3639	fig|6666666.59353.peg.4326	fig|6666666.59355.peg.4543	fig|6666666.59354.peg.1958

8841	4Fe-4S ferredoxin, iron-sulfur binding	fig|6666666.59329.peg.5298	fig|6666666.59330.peg.3674	None	fig|6666666.59332.peg.1262	fig|6666666.59334.peg.2956	fig|6666666.59335.peg.2560	fig|6666666.59336.peg.4273	fig|6666666.59333.peg.340	fig|6666666.59338.peg.1864	fig|6666666.59339.peg.6907	None	fig|6666666.59337.peg.701	fig|6666666.59342.peg.4187	fig|6666666.71181.peg.1418	fig|6666666.59344.peg.1571	fig|6666666.59345.peg.3686	fig|6666666.59341.peg.6002	fig|6666666.59347.peg.4256	fig|6666666.59346.peg.2548	fig|6666666.59348.peg.2591	fig|6666666.59349.peg.3075	None	fig|6666666.59351.peg.3068	fig|6666666.59353.peg.2658	fig|6666666.59355.peg.1824	fig|6666666.59354.peg.76

1768	Aldose 1-epimerase family protein YeaD	fig|6666666.59329.peg.871	fig|6666666.59330.peg.1218	fig|6666666.59331.peg.1772	fig|6666666.59332.peg.1349	fig|6666666.59334.peg.1622	fig|6666666.59335.peg.2949	fig|6666666.59336.peg.1816	fig|6666666.59333.peg.1088	fig|6666666.59338.peg.1954	fig|6666666.59339.peg.267	None	fig|6666666.59337.peg.3125	None	fig|6666666.71181.peg.2969	fig|6666666.59344.peg.2891	fig|6666666.59345.peg.1165	fig|6666666.59341.peg.5424	fig|6666666.59347.peg.2435	fig|6666666.59346.peg.75	fig|6666666.59348.peg.2576	fig|6666666.59349.peg.1745	None	fig|6666666.59351.peg.2399	fig|6666666.59353.peg.1435	fig|6666666.59355.peg.1984	fig|6666666.59354.peg.5742

10253	Nucleoprotein polynucleotide-associated enzyme	fig|6666666.59329.peg.1881	fig|6666666.59330.peg.3210	None	fig|6666666.59332.peg.4883	fig|6666666.59334.peg.3486	fig|6666666.59335.peg.4024	fig|6666666.59336.peg.2049	fig|6666666.59333.peg.1303	fig|6666666.59338.peg.5706	fig|6666666.59339.peg.3817	fig|6666666.59340.peg.2517	fig|6666666.59337.peg.5149	fig|6666666.59342.peg.5779	fig|6666666.71181.peg.940	fig|6666666.59344.peg.3543	fig|6666666.59345.peg.3893	fig|6666666.59341.peg.4944	fig|6666666.59347.peg.1574	fig|6666666.59346.peg.3939	fig|6666666.59348.peg.2497	fig|6666666.59349.peg.4626	None	fig|6666666.59351.peg.3107	fig|6666666.59353.peg.875	fig|6666666.59355.peg.829	None

8038	Tetraacyldisaccharide 4'-kinase EC 2.7.1.130	fig|6666666.59329.peg.3288	fig|6666666.59330.peg.1256	fig|6666666.59331.peg.5379	fig|6666666.59332.peg.3687	None	fig|6666666.59335.peg.2831	fig|6666666.59336.peg.3393	fig|6666666.59333.peg.1833	fig|6666666.59338.peg.2928	fig|6666666.59339.peg.3475	None	fig|6666666.59337.peg.2200	fig|6666666.59342.peg.5791	fig|6666666.71181.peg.4191	fig|6666666.59344.peg.2795	fig|6666666.59345.peg.1589	fig|6666666.59341.peg.1852	fig|6666666.59347.peg.4105	fig|6666666.59346.peg.5296	None	fig|6666666.59349.peg.2509	fig|6666666.59350.peg.734	fig|6666666.59351.peg.3713	fig|6666666.59353.peg.1651	fig|6666666.59355.peg.1070	fig|6666666.59354.peg.2887

8528	Acetoacetyl-CoA reductase EC 1.1.1.36	fig|6666666.59329.peg.2626	fig|6666666.59330.peg.2102	fig|6666666.59331.peg.3656	fig|6666666.59332.peg.3881	None	fig|6666666.59335.peg.1371	fig|6666666.59336.peg.1998	fig|6666666.59333.peg.4835	fig|6666666.59338.peg.4532	fig|6666666.59339.peg.2765	None	fig|6666666.59337.peg.3527	fig|6666666.59342.peg.2581	fig|6666666.71181.peg.5492	fig|6666666.59344.peg.5395	fig|6666666.59345.peg.491	fig|6666666.59341.peg.4966	fig|6666666.59347.peg.1599	fig|6666666.59346.peg.2111	fig|6666666.59348.peg.5474	fig|6666666.59349.peg.4440	fig|6666666.59350.peg.3234	None	fig|6666666.59353.peg.4783	fig|6666666.59355.peg.4836	fig|6666666.59354.peg.1117

10435	Glucose-6-phosphate 1-dehydrogenase EC 1.1.1.49	fig|6666666.59329.peg.3056	fig|6666666.59330.peg.3315	None	fig|6666666.59332.peg.1388	fig|6666666.59334.peg.3016	fig|6666666.59335.peg.3884	None	fig|6666666.59333.peg.1646	fig|6666666.59338.peg.4403	fig|6666666.59339.peg.2436	None	fig|6666666.59337.peg.3101	fig|6666666.59342.peg.2267	fig|6666666.71181.peg.1932	fig|6666666.59344.peg.2840	fig|6666666.59345.peg.4147	fig|6666666.59341.peg.3393	fig|6666666.59347.peg.3469	fig|6666666.59346.peg.3576	fig|6666666.59348.peg.3036	fig|6666666.59349.peg.660	fig|6666666.59350.peg.424	fig|6666666.59351.peg.4929	fig|6666666.59353.peg.1477	fig|6666666.59355.peg.2027	fig|6666666.59354.peg.4959

2726	Translation elongation factor Ts	fig|6666666.59329.peg.5027	fig|6666666.59330.peg.468	None	fig|6666666.59332.peg.1591	fig|6666666.59334.peg.2978	fig|6666666.59335.peg.163	fig|6666666.59336.peg.4214	fig|6666666.59333.peg.3060	fig|6666666.59338.peg.2555	fig|6666666.59339.peg.5108	None	fig|6666666.59337.peg.1582	fig|6666666.59342.peg.266	fig|6666666.71181.peg.34	fig|6666666.59344.peg.2654	fig|6666666.59345.peg.2200	fig|6666666.59341.peg.968	fig|6666666.59347.peg.4359	fig|6666666.59346.peg.2261	None	fig|6666666.59349.peg.1375	fig|6666666.59350.peg.6671	fig|6666666.59351.peg.2115	fig|6666666.59353.peg.1984	fig|6666666.59355.peg.4091	fig|6666666.59354.peg.1333

2720	SSU ribosomal protein S13p S18e	None	fig|6666666.59330.peg.573	fig|6666666.59331.peg.30	fig|6666666.59332.peg.5648	fig|6666666.59334.peg.3043	None	fig|6666666.59336.peg.163	fig|6666666.59333.peg.385	fig|6666666.59338.peg.4737	fig|6666666.59339.peg.8287	fig|6666666.59340.peg.2221	fig|6666666.59337.peg.370	fig|6666666.59342.peg.323	fig|6666666.71181.peg.3442	fig|6666666.59344.peg.4925	fig|6666666.59345.peg.2517	None	fig|6666666.59347.peg.4842	fig|6666666.59346.peg.868	fig|6666666.59348.peg.436	fig|6666666.59349.peg.4943	fig|6666666.59350.peg.4705	fig|6666666.59351.peg.4223	fig|6666666.59353.peg.4608	fig|6666666.59355.peg.4723	fig|6666666.59354.peg.3249

810	Phosphoribosylaminoimidazole-succinocarboxamide synthase EC 6.3.2.6	fig|6666666.59329.peg.3048	fig|6666666.59330.peg.5063	fig|6666666.59331.peg.4044	fig|6666666.59332.peg.4266	fig|6666666.59334.peg.3557	None	fig|6666666.59336.peg.2902	fig|6666666.59333.peg.3667	fig|6666666.59338.peg.5317	fig|6666666.59339.peg.964	None	fig|6666666.59337.peg.4145	fig|6666666.59342.peg.1066	fig|6666666.71181.peg.3250	fig|6666666.59344.peg.4003	fig|6666666.59345.peg.4613	fig|6666666.59341.peg.1402	fig|6666666.59347.peg.1955	fig|6666666.59346.peg.3924	None	fig|6666666.59349.peg.3631	fig|6666666.59350.peg.5372	fig|6666666.59351.peg.2246	fig|6666666.59353.peg.4046	fig|6666666.59355.peg.5305	fig|6666666.59354.peg.4602

6751	DNA polymerase III chi subunit EC 2.7.7.7	fig|6666666.59329.peg.2117	fig|6666666.59330.peg.2447	fig|6666666.59331.peg.5428	fig|6666666.59332.peg.2092	fig|6666666.59334.peg.4520	fig|6666666.59335.peg.252	fig|6666666.59336.peg.488	fig|6666666.59333.peg.2821	fig|6666666.59338.peg.3093	fig|6666666.59339.peg.8140	fig|6666666.59340.peg.340	fig|6666666.59337.peg.4790	fig|6666666.59342.peg.5095	None	fig|6666666.59344.peg.3974	fig|6666666.59345.peg.4835	fig|6666666.59341.peg.1446	fig|6666666.59347.peg.3263	fig|6666666.59346.peg.1669	fig|6666666.59348.peg.2180	None	None	fig|6666666.59351.peg.3498	fig|6666666.59353.peg.2441	fig|6666666.59355.peg.533	fig|6666666.59354.peg.3395

6755	proteinase inhibitor, putative	fig|6666666.59329.peg.2598	fig|6666666.59330.peg.4127	fig|6666666.59331.peg.5086	fig|6666666.59332.peg.4658	fig|6666666.59334.peg.2895	fig|6666666.59335.peg.2755	fig|6666666.59336.peg.1242	fig|6666666.59333.peg.5291	fig|6666666.59338.peg.2301	fig|6666666.59339.peg.250	None	fig|6666666.59337.peg.3745	fig|6666666.59342.peg.3905	fig|6666666.71181.peg.3176	fig|6666666.59344.peg.1674	fig|6666666.59345.peg.1184	fig|6666666.59341.peg.2463	fig|6666666.59347.peg.2442	fig|6666666.59346.peg.68	fig|6666666.59348.peg.3119	fig|6666666.59349.peg.3734	None	fig|6666666.59351.peg.817	fig|6666666.59353.peg.1094	fig|6666666.59355.peg.385	None

933	Triosephosphate isomerase EC 5.3.1.1	fig|6666666.59329.peg.869	fig|6666666.59330.peg.5015	None	fig|6666666.59332.peg.163	fig|6666666.59334.peg.960	fig|6666666.59335.peg.3558	fig|6666666.59336.peg.2174	fig|6666666.59333.peg.1391	fig|6666666.59338.peg.2025	fig|6666666.59339.peg.8681	None	fig|6666666.59337.peg.412	None	fig|6666666.71181.peg.4009	fig|6666666.59344.peg.985	fig|6666666.59345.peg.2558	fig|6666666.59341.peg.196	fig|6666666.59347.peg.4529	fig|6666666.59346.peg.795	fig|6666666.59348.peg.4205	fig|6666666.59349.peg.3694	fig|6666666.59350.peg.1522	fig|6666666.59351.peg.4942	fig|6666666.59353.peg.2553	fig|6666666.59355.peg.2648	fig|6666666.59354.peg.1198

10791	Glutaredoxin-related protein	fig|6666666.59329.peg.795	fig|6666666.59330.peg.3385	None	fig|6666666.59332.peg.5919	fig|6666666.59334.peg.1429	fig|6666666.59335.peg.3125	fig|6666666.59336.peg.3555	fig|6666666.59333.peg.3172	fig|6666666.59338.peg.4233	fig|6666666.59339.peg.176	None	fig|6666666.59337.peg.4421	fig|6666666.59342.peg.1044	fig|6666666.71181.peg.2544	fig|6666666.59344.peg.3514	fig|6666666.59345.peg.1134	None	fig|6666666.59347.peg.2563	fig|6666666.59346.peg.4470	fig|6666666.59348.peg.4312	fig|6666666.59349.peg.2393	fig|6666666.59350.peg.3349	fig|6666666.59351.peg.637	fig|6666666.59353.peg.1371	fig|6666666.59355.peg.2502	fig|6666666.59354.peg.5655

8053	Alkylphosphonate utilization operon protein PhnA	fig|6666666.59329.peg.5127	fig|6666666.59330.peg.5090	fig|6666666.59331.peg.5962	None	fig|6666666.59334.peg.2136	fig|6666666.59335.peg.2271	fig|6666666.59336.peg.1002	fig|6666666.59333.peg.3780	fig|6666666.59338.peg.5825	fig|6666666.59339.peg.3803	None	fig|6666666.59337.peg.2818	None	fig|6666666.71181.peg.6392	fig|6666666.59344.peg.4515	fig|6666666.59345.peg.117	fig|6666666.59341.peg.3103	fig|6666666.59347.peg.5294	fig|6666666.59346.peg.2918	fig|6666666.59348.peg.3728	fig|6666666.59349.peg.1788	fig|6666666.59350.peg.3753	fig|6666666.59351.peg.3259	fig|6666666.59353.peg.2038	fig|6666666.59355.peg.4445	fig|6666666.59354.peg.3178

5801	Asparagine synthetase [glutamine-hydrolyzing] EC 6.3.5.4	fig|6666666.59329.peg.1946	fig|6666666.59330.peg.3007	None	fig|6666666.59332.peg.6077	fig|6666666.59334.peg.212	fig|6666666.59335.peg.4589	fig|6666666.59336.peg.321	fig|6666666.59333.peg.870	fig|6666666.59338.peg.5967	fig|6666666.59339.peg.7226	None	fig|6666666.59337.peg.3924	fig|6666666.59342.peg.6112	fig|6666666.71181.peg.3205	fig|6666666.59344.peg.4519	fig|6666666.59345.peg.4344	fig|6666666.59341.peg.286	fig|6666666.59347.peg.2951	fig|6666666.59346.peg.5246	fig|6666666.59348.peg.3020	fig|6666666.59349.peg.2232	None	fig|6666666.59351.peg.883	fig|6666666.59353.peg.2041	fig|6666666.59355.peg.4448	fig|6666666.59354.peg.4577

9717	Putative transporting ATPase	fig|6666666.59329.peg.2414	fig|6666666.59330.peg.1588	fig|6666666.59331.peg.1895	fig|6666666.59332.peg.1952	None	fig|6666666.59335.peg.3941	fig|6666666.59336.peg.5507	fig|6666666.59333.peg.1969	fig|6666666.59338.peg.1093	fig|6666666.59339.peg.7069	None	fig|6666666.59337.peg.4843	fig|6666666.59342.peg.2103	fig|6666666.71181.peg.1508	fig|6666666.59344.peg.5004	fig|6666666.59345.peg.5292	fig|6666666.59341.peg.837	fig|6666666.59347.peg.5117	fig|6666666.59346.peg.2655	fig|6666666.59348.peg.1550	fig|6666666.59349.peg.4073	None	fig|6666666.59351.peg.708	fig|6666666.59353.peg.5015	fig|6666666.59355.peg.233	fig|6666666.59354.peg.5588

7453	Allophanate hydrolase 2 subunit 2 EC 3.5.1.54	fig|6666666.59329.peg.3373	fig|6666666.59330.peg.3706	None	fig|6666666.59332.peg.5642	fig|6666666.59334.peg.4238	fig|6666666.59335.peg.5834	fig|6666666.59336.peg.6060	fig|6666666.59333.peg.2693	None	fig|6666666.59339.peg.2853	fig|6666666.59340.peg.1721	fig|6666666.59337.peg.3039	fig|6666666.59342.peg.1982	fig|6666666.71181.peg.3225	fig|6666666.59344.peg.4607	fig|6666666.59345.peg.94	fig|6666666.59341.peg.1569	fig|6666666.59347.peg.4395	fig|6666666.59346.peg.3634	None	fig|6666666.59349.peg.2236	fig|6666666.59350.peg.6894	fig|6666666.59351.peg.887	fig|6666666.59353.peg.2077	fig|6666666.59355.peg.2748	fig|6666666.59354.peg.5973

5246	DNA-binding protein H-NS	fig|6666666.59329.peg.2583	fig|6666666.59330.peg.2866	None	fig|6666666.59332.peg.4974	fig|6666666.59334.peg.3410	fig|6666666.59335.peg.596	fig|6666666.59336.peg.3101	fig|6666666.59333.peg.5187	fig|6666666.59338.peg.399	fig|6666666.59339.peg.487	fig|6666666.59340.peg.1400	None	fig|6666666.59342.peg.540	fig|6666666.71181.peg.2762	fig|6666666.59344.peg.417	fig|6666666.59345.peg.1310	fig|6666666.59341.peg.493	fig|6666666.59347.peg.4135	fig|6666666.59346.peg.2679	None	fig|6666666.59349.peg.1334	fig|6666666.59350.peg.2648	fig|6666666.59351.peg.4242	fig|6666666.59353.peg.1744	fig|6666666.59355.peg.1268	fig|6666666.59354.peg.165

5227	Chaperone protein HtpG	None	fig|6666666.59330.peg.2203	fig|6666666.59331.peg.3037	fig|6666666.59332.peg.1702	None	fig|6666666.59335.peg.2419	fig|6666666.59336.peg.2548	fig|6666666.59333.peg.2064	fig|6666666.59338.peg.4380	fig|6666666.59339.peg.5266	fig|6666666.59340.peg.674	fig|6666666.59337.peg.3919	fig|6666666.59342.peg.1794	fig|6666666.71181.peg.3497	fig|6666666.59344.peg.5516	fig|6666666.59345.peg.4349	fig|6666666.59341.peg.510	fig|6666666.59347.peg.2833	fig|6666666.59346.peg.3672	fig|6666666.59348.peg.2492	fig|6666666.59349.peg.2401	None	fig|6666666.59351.peg.2781	fig|6666666.59353.peg.4073	fig|6666666.59355.peg.4150	fig|6666666.59354.peg.1498

3065	Rod shape-determining protein MreD	fig|6666666.59329.peg.707	fig|6666666.59330.peg.26	fig|6666666.59331.peg.5595	fig|6666666.59332.peg.2165	fig|6666666.59334.peg.1312	fig|6666666.59335.peg.3028	None	fig|6666666.59333.peg.3652	fig|6666666.59338.peg.3607	fig|6666666.59339.peg.7790	fig|6666666.59340.peg.611	fig|6666666.59337.peg.811	fig|6666666.59342.peg.3380	fig|6666666.71181.peg.980	fig|6666666.59344.peg.1744	fig|6666666.59345.peg.4883	fig|6666666.59341.peg.884	fig|6666666.59347.peg.3114	fig|6666666.59346.peg.2129	fig|6666666.59348.peg.5348	fig|6666666.59349.peg.6	None	fig|6666666.59351.peg.3423	fig|6666666.59353.peg.2294	fig|6666666.59355.peg.1570	None

498	Cell division protein FtsX	fig|6666666.59329.peg.771	fig|6666666.59330.peg.1566	None	fig|6666666.59332.peg.2372	fig|6666666.59334.peg.1292	fig|6666666.59335.peg.4270	fig|6666666.59336.peg.3157	None	fig|6666666.59338.peg.527	fig|6666666.59339.peg.8567	fig|6666666.59340.peg.93	fig|6666666.59337.peg.1650	fig|6666666.59342.peg.2202	fig|6666666.71181.peg.4530	fig|6666666.59344.peg.3450	fig|6666666.59345.peg.5470	fig|6666666.59341.peg.5663	fig|6666666.59347.peg.5054	fig|6666666.59346.peg.3712	fig|6666666.59348.peg.584	fig|6666666.59349.peg.3787	None	fig|6666666.59351.peg.3393	fig|6666666.59353.peg.3769	fig|6666666.59355.peg.3364	fig|6666666.59354.peg.21

6470	DNA recombination protein RmuC	fig|6666666.59329.peg.3192	fig|6666666.59330.peg.249	None	fig|6666666.59332.peg.4	fig|6666666.59334.peg.3549	fig|6666666.59335.peg.39	fig|6666666.59336.peg.890	fig|6666666.59333.peg.2514	fig|6666666.59338.peg.2984	None	fig|6666666.59340.peg.865	fig|6666666.59337.peg.957	None	fig|6666666.71181.peg.3342	fig|6666666.59344.peg.3419	fig|6666666.59345.peg.2716	fig|6666666.59341.peg.150	fig|6666666.59347.peg.4633	fig|6666666.59346.peg.3354	fig|6666666.59348.peg.2991	fig|6666666.59349.peg.1504	fig|6666666.59350.peg.7374	fig|6666666.59351.peg.3003	fig|6666666.59353.peg.3796	fig|6666666.59355.peg.3392	fig|6666666.59354.peg.225

9901	Arginine ornithine ABC transporter, permease protein AotQ	fig|6666666.59329.peg.1028	fig|6666666.59330.peg.616	fig|6666666.59331.peg.4407	fig|6666666.59332.peg.5443	fig|6666666.59334.peg.4802	fig|6666666.59335.peg.4094	fig|6666666.59336.peg.39	fig|6666666.59333.peg.4859	fig|6666666.59338.peg.5592	fig|6666666.59339.peg.1560	fig|6666666.59340.peg.2803	fig|6666666.59337.peg.3387	fig|6666666.59342.peg.1735	fig|6666666.71181.peg.6271	fig|6666666.59344.peg.1892	fig|6666666.59345.peg.4977	fig|6666666.59341.peg.2081	fig|6666666.59347.peg.1544	fig|6666666.59346.peg.1192	fig|6666666.59348.peg.1799	None	fig|6666666.59350.peg.2443	None	fig|6666666.59353.peg.521	fig|6666666.59355.peg.276	None

445	Glutamate synthase [NADPH] small chain EC 1.4.1.13	fig|6666666.59329.peg.334	fig|6666666.59330.peg.2842	None	fig|6666666.59332.peg.317	fig|6666666.59334.peg.262	fig|6666666.59335.peg.1665	fig|6666666.59336.peg.3009	fig|6666666.59333.peg.163	fig|6666666.59338.peg.2853	fig|6666666.59339.peg.7928	None	fig|6666666.59337.peg.2039	fig|6666666.59342.peg.2094	fig|6666666.71181.peg.3960	fig|6666666.59344.peg.5445	fig|6666666.59345.peg.2435	fig|6666666.59341.peg.1340	fig|6666666.59347.peg.3882	fig|6666666.59346.peg.566	fig|6666666.59348.peg.2208	fig|6666666.59349.peg.600	None	fig|6666666.59351.peg.532	fig|6666666.59353.peg.4816	fig|6666666.59355.peg.4870	fig|6666666.59354.peg.4902

3433	Rhodanese-related sulfurtransferase	fig|6666666.59329.peg.6364	fig|6666666.59330.peg.1131	None	fig|6666666.59332.peg.3283	fig|6666666.59334.peg.4151	fig|6666666.59335.peg.5291	fig|6666666.59336.peg.5679	fig|6666666.59333.peg.4276	fig|6666666.59338.peg.4700	fig|6666666.59339.peg.8497	None	fig|6666666.59337.peg.3661	fig|6666666.59342.peg.3394	fig|6666666.71181.peg.4733	fig|6666666.59344.peg.6259	fig|6666666.59345.peg.5216	fig|6666666.59341.peg.1544	fig|6666666.59347.peg.690	fig|6666666.59346.peg.3867	fig|6666666.59348.peg.2470	None	fig|6666666.59350.peg.4533	fig|6666666.59351.peg.2258	fig|6666666.59353.peg.5060	fig|6666666.59355.peg.4668	fig|6666666.59354.peg.6073

4847	Aspartyl-tRNA synthetase EC 6.1.1.12	fig|6666666.59329.peg.3404	fig|6666666.59330.peg.2485	fig|6666666.59331.peg.1969	fig|6666666.59332.peg.1957	None	fig|6666666.59335.peg.954	fig|6666666.59336.peg.3634	fig|6666666.59333.peg.1047	fig|6666666.59338.peg.4014	fig|6666666.59339.peg.340	fig|6666666.59340.peg.1631	fig|6666666.59337.peg.4547	fig|6666666.59342.peg.4874	fig|6666666.71181.peg.1918	fig|6666666.59344.peg.2521	fig|6666666.59345.peg.1518	fig|6666666.59341.peg.2251	fig|6666666.59347.peg.4195	fig|6666666.59346.peg.2769	None	fig|6666666.59349.peg.3868	None	fig|6666666.59351.peg.652	fig|6666666.59353.peg.1491	fig|6666666.59355.peg.2041	fig|6666666.59354.peg.1589

1091	Undecaprenyl diphosphate synthase EC 2.5.1.31	fig|6666666.59329.peg.4321	fig|6666666.59330.peg.464	fig|6666666.59331.peg.3457	fig|6666666.59332.peg.1586	None	fig|6666666.59335.peg.2655	fig|6666666.59336.peg.2560	fig|6666666.59333.peg.3056	fig|6666666.59338.peg.4900	fig|6666666.59339.peg.5112	None	fig|6666666.59337.peg.1585	fig|6666666.59342.peg.270	fig|6666666.71181.peg.30	fig|6666666.59344.peg.2658	fig|6666666.59345.peg.2197	fig|6666666.59341.peg.971	fig|6666666.59347.peg.4362	fig|6666666.59346.peg.2264	fig|6666666.59348.peg.4880	fig|6666666.59349.peg.1372	fig|6666666.59350.peg.1878	fig|6666666.59351.peg.2118	fig|6666666.59353.peg.1988	fig|6666666.59355.peg.4095	None

9677	Transcriptional regulator, SorC family	fig|6666666.59329.peg.4716	fig|6666666.59330.peg.1129	fig|6666666.59331.peg.717	fig|6666666.59332.peg.5586	fig|6666666.59334.peg.1605	fig|6666666.59335.peg.5867	fig|6666666.59336.peg.4821	fig|6666666.59333.peg.4277	fig|6666666.59338.peg.3164	fig|6666666.59339.peg.8503	fig|6666666.59340.peg.1620	fig|6666666.59337.peg.5300	fig|6666666.59342.peg.4584	fig|6666666.71181.peg.4343	fig|6666666.59344.peg.1963	fig|6666666.59345.peg.5219	fig|6666666.59341.peg.1548	fig|6666666.59347.peg.3625	fig|6666666.59346.peg.3861	fig|6666666.59348.peg.4666	None	fig|6666666.59350.peg.2960	None	fig|6666666.59353.peg.2953	fig|6666666.59355.peg.2787	None

3621	DnaJ-like protein DjlA	fig|6666666.59329.peg.124	fig|6666666.59330.peg.814	fig|6666666.59331.peg.2387	fig|6666666.59332.peg.4898	fig|6666666.59334.peg.339	fig|6666666.59335.peg.1639	fig|6666666.59336.peg.3668	fig|6666666.59333.peg.127	fig|6666666.59338.peg.904	fig|6666666.59339.peg.7900	None	fig|6666666.59337.peg.1388	None	fig|6666666.71181.peg.396	fig|6666666.59344.peg.1520	fig|6666666.59345.peg.3892	fig|6666666.59341.peg.4414	fig|6666666.59347.peg.3085	fig|6666666.59346.peg.820	fig|6666666.59348.peg.3705	fig|6666666.59349.peg.1882	fig|6666666.59350.peg.2293	None	fig|6666666.59353.peg.102	fig|6666666.59355.peg.712	fig|6666666.59354.peg.1383

2462	Homoserine kinase EC 2.7.1.39	fig|6666666.59329.peg.107	fig|6666666.59330.peg.1723	fig|6666666.59331.peg.4925	fig|6666666.59332.peg.2901	fig|6666666.59334.peg.5057	fig|6666666.59335.peg.5127	fig|6666666.59336.peg.3403	fig|6666666.59333.peg.4998	fig|6666666.59338.peg.3316	None	None	fig|6666666.59337.peg.92	fig|6666666.59342.peg.4467	fig|6666666.71181.peg.435	fig|6666666.59344.peg.1338	fig|6666666.59345.peg.3751	fig|6666666.59341.peg.89	fig|6666666.59347.peg.4033	fig|6666666.59346.peg.1371	fig|6666666.59348.peg.215	fig|6666666.59349.peg.3474	None	fig|6666666.59351.peg.4257	fig|6666666.59353.peg.63	fig|6666666.59355.peg.670	fig|6666666.59354.peg.4523

2806	Isoaspartyl aminopeptidase EC 3.4.19.5 Asp-X dipeptidase	None	fig|6666666.59330.peg.4079	fig|6666666.59331.peg.2366	fig|6666666.59332.peg.1070	fig|6666666.59334.peg.433	fig|6666666.59335.peg.1862	fig|6666666.59336.peg.2188	fig|6666666.59333.peg.3110	fig|6666666.59338.peg.3091	fig|6666666.59339.peg.8253	None	fig|6666666.59337.peg.1816	fig|6666666.59342.peg.6300	fig|6666666.71181.peg.181	fig|6666666.59344.peg.220	fig|6666666.59345.peg.5559	fig|6666666.59341.peg.2421	fig|6666666.59347.peg.4971	fig|6666666.59346.peg.37	fig|6666666.59348.peg.294	fig|6666666.59349.peg.674	None	fig|6666666.59351.peg.2786	fig|6666666.59353.peg.641	fig|6666666.59355.peg.124	fig|6666666.59354.peg.6036

6558	ATPase, AFG1 family	None	fig|6666666.59330.peg.960	None	fig|6666666.59332.peg.652	fig|6666666.59334.peg.3196	fig|6666666.59335.peg.3437	fig|6666666.59336.peg.1586	fig|6666666.59333.peg.621	fig|6666666.59338.peg.4446	fig|6666666.59339.peg.4937	fig|6666666.59340.peg.1137	fig|6666666.59337.peg.26	None	fig|6666666.71181.peg.1038	fig|6666666.59344.peg.1821	fig|6666666.59345.peg.3816	fig|6666666.59341.peg.3550	fig|6666666.59347.peg.3919	fig|6666666.59346.peg.2339	fig|6666666.59348.peg.1451	fig|6666666.59349.peg.38	fig|6666666.59350.peg.324	fig|6666666.59351.peg.3458	fig|6666666.59353.peg.2357	fig|6666666.59355.peg.1630	fig|6666666.59354.peg.354

359	Methyl-accepting chemotaxis protein	fig|6666666.59329.peg.1218	None	fig|6666666.59331.peg.4945	fig|6666666.59332.peg.1667	fig|6666666.59334.peg.4558	fig|6666666.59335.peg.133	fig|6666666.59336.peg.2777	fig|6666666.59333.peg.1811	fig|6666666.59338.peg.275	fig|6666666.59339.peg.5063	None	fig|6666666.59337.peg.1555	fig|6666666.59342.peg.6497	fig|6666666.71181.peg.1872	fig|6666666.59344.peg.4347	fig|6666666.59345.peg.2225	fig|6666666.59341.peg.2301	fig|6666666.59347.peg.2791	fig|6666666.59346.peg.5207	fig|6666666.59348.peg.3542	fig|6666666.59349.peg.1779	None	fig|6666666.59351.peg.5312	fig|6666666.59353.peg.3238	fig|6666666.59355.peg.3120	fig|6666666.59354.peg.2027

2193	Lysine 2,3-aminomutase EC 5.4.3.2	fig|6666666.59329.peg.4449	fig|6666666.59330.peg.159	fig|6666666.59331.peg.2634	fig|6666666.59332.peg.5991	None	fig|6666666.59335.peg.3552	fig|6666666.59336.peg.254	fig|6666666.59333.peg.429	fig|6666666.59338.peg.3737	fig|6666666.59339.peg.8689	fig|6666666.59340.peg.219	fig|6666666.59337.peg.4329	None	fig|6666666.71181.peg.1680	fig|6666666.59344.peg.612	fig|6666666.59345.peg.5111	fig|6666666.59341.peg.173	fig|6666666.59347.peg.4553	fig|6666666.59346.peg.976	fig|6666666.59348.peg.2570	fig|6666666.59349.peg.2372	None	fig|6666666.59351.peg.133	fig|6666666.59353.peg.1050	fig|6666666.59355.peg.3569	fig|6666666.59354.peg.5462

459	Preprotein translocase subunit SecG TC 3.A.5.1.1	fig|6666666.59329.peg.3446	fig|6666666.59330.peg.109	fig|6666666.59331.peg.2317	None	fig|6666666.59334.peg.3207	fig|6666666.59335.peg.2105	fig|6666666.59336.peg.2418	fig|6666666.59333.peg.5530	fig|6666666.59338.peg.1503	fig|6666666.59339.peg.4890	fig|6666666.59340.peg.3314	fig|6666666.59337.peg.681	fig|6666666.59342.peg.375	fig|6666666.71181.peg.4095	fig|6666666.59344.peg.2102	fig|6666666.59345.peg.2359	None	fig|6666666.59347.peg.3007	fig|6666666.59346.peg.296	fig|6666666.59348.peg.965	fig|6666666.59349.peg.3693	None	fig|6666666.59351.peg.4943	fig|6666666.59353.peg.3135	fig|6666666.59355.peg.461	fig|6666666.59354.peg.1199

3835	FIG137360 hypothetical protein	fig|6666666.59329.peg.3236	fig|6666666.59330.peg.4622	None	fig|6666666.59332.peg.5250	fig|6666666.59334.peg.4065	fig|6666666.59335.peg.1415	fig|6666666.59336.peg.3286	fig|6666666.59333.peg.1311	fig|6666666.59338.peg.3489	fig|6666666.59339.peg.6654	None	fig|6666666.59337.peg.4079	fig|6666666.59342.peg.5175	fig|6666666.71181.peg.2385	fig|6666666.59344.peg.767	fig|6666666.59345.peg.1676	fig|6666666.59341.peg.442	fig|6666666.59347.peg.1872	fig|6666666.59346.peg.2635	fig|6666666.59348.peg.1079	fig|6666666.59349.peg.4153	None	fig|6666666.59351.peg.1704	fig|6666666.59353.peg.1337	fig|6666666.59355.peg.2534	fig|6666666.59354.peg.3317

9764	ADA regulatory protein	fig|6666666.59329.peg.3319	fig|6666666.59330.peg.1484	fig|6666666.59331.peg.683	fig|6666666.59332.peg.963	None	fig|6666666.59335.peg.6195	fig|6666666.59336.peg.5134	None	fig|6666666.59338.peg.5081	fig|6666666.59339.peg.5642	fig|6666666.59340.peg.1242	fig|6666666.59337.peg.191	None	fig|6666666.71181.peg.4292	fig|6666666.59344.peg.4047	fig|6666666.59345.peg.4521	fig|6666666.59341.peg.2343	fig|6666666.59347.peg.197	fig|6666666.59346.peg.2589	fig|6666666.59348.peg.1852	fig|6666666.59349.peg.2957	fig|6666666.59350.peg.485	fig|6666666.59351.peg.3804	fig|6666666.59353.peg.4008	fig|6666666.59355.peg.3967	fig|6666666.59354.peg.3045

11237	C1683 Uncharacterized conserved protein FIG143828 Hypothetical protein YbgA	fig|6666666.59329.peg.1960	fig|6666666.59330.peg.741	fig|6666666.59331.peg.1425	fig|6666666.59332.peg.595	fig|6666666.59334.peg.2488	fig|6666666.59335.peg.1926	fig|6666666.59336.peg.2762	None	fig|6666666.59338.peg.1481	fig|6666666.59339.peg.8397	None	fig|6666666.59337.peg.3470	fig|6666666.59342.peg.5885	fig|6666666.71181.peg.4735	fig|6666666.59344.peg.6262	fig|6666666.59345.peg.2782	fig|6666666.59341.peg.931	fig|6666666.59347.peg.437	fig|6666666.59346.peg.2986	fig|6666666.59348.peg.1214	fig|6666666.59349.peg.4292	None	fig|6666666.59351.peg.718	fig|6666666.59353.peg.5062	fig|6666666.59355.peg.4670	fig|6666666.59354.peg.1692

9989	Predicted glutamine amidotransferase	fig|6666666.59329.peg.4581	fig|6666666.59330.peg.4185	None	fig|6666666.59332.peg.2301	fig|6666666.59334.peg.4266	fig|6666666.59335.peg.4084	fig|6666666.59336.peg.3719	fig|6666666.59333.peg.4585	fig|6666666.59338.peg.2052	fig|6666666.59339.peg.4344	fig|6666666.59340.peg.2742	fig|6666666.59337.peg.4483	None	fig|6666666.71181.peg.913	None	fig|6666666.59345.peg.2169	fig|6666666.59341.peg.570	fig|6666666.59347.peg.2863	fig|6666666.59346.peg.2490	fig|6666666.59348.peg.1155	fig|6666666.59349.peg.3023	fig|6666666.59350.peg.4019	fig|6666666.59351.peg.755	fig|6666666.59353.peg.904	fig|6666666.59355.peg.802	fig|6666666.59354.peg.3278

9981	Peptidyl-prolyl cis-trans isomerase PpiC EC 5.2.1.8	fig|6666666.59329.peg.2454	fig|6666666.59330.peg.4169	fig|6666666.59331.peg.2474	fig|6666666.59332.peg.3363	fig|6666666.59334.peg.2127	fig|6666666.59335.peg.1064	None	fig|6666666.59333.peg.5371	fig|6666666.59338.peg.6180	fig|6666666.59339.peg.4521	fig|6666666.59340.peg.3494	fig|6666666.59337.peg.2466	None	fig|6666666.71181.peg.2457	fig|6666666.59344.peg.2451	fig|6666666.59345.peg.588	fig|6666666.59341.peg.4788	fig|6666666.59347.peg.1530	fig|6666666.59346.peg.3911	fig|6666666.59348.peg.918	fig|6666666.59349.peg.1301	None	fig|6666666.59351.peg.3083	fig|6666666.59353.peg.3657	fig|6666666.59355.peg.4027	fig|6666666.59354.peg.2834

7845	Transporter	fig|6666666.59329.peg.5736	fig|6666666.59330.peg.4948	None	fig|6666666.59332.peg.1536	fig|6666666.59334.peg.4298	fig|6666666.59335.peg.766	fig|6666666.59336.peg.1855	fig|6666666.59333.peg.1706	fig|6666666.59338.peg.6198	fig|6666666.59339.peg.5585	None	fig|6666666.59337.peg.2476	fig|6666666.59342.peg.4286	fig|6666666.71181.peg.3517	fig|6666666.59344.peg.4623	fig|6666666.59345.peg.1367	fig|6666666.59341.peg.555	fig|6666666.59347.peg.3360	fig|6666666.59346.peg.3669	None	fig|6666666.59349.peg.2318	fig|6666666.59350.peg.5275	fig|6666666.59351.peg.2932	fig|6666666.59353.peg.2411	fig|6666666.59355.peg.502	fig|6666666.59354.peg.285

2847	Protein YigP C3165 clustered with ubiquinone biosynthetic genes	fig|6666666.59329.peg.3194	fig|6666666.59330.peg.251	None	fig|6666666.59332.peg.6	fig|6666666.59334.peg.2864	fig|6666666.59335.peg.41	fig|6666666.59336.peg.892	fig|6666666.59333.peg.2512	fig|6666666.59338.peg.3945	fig|6666666.59339.peg.7571	fig|6666666.59340.peg.867	fig|6666666.59337.peg.959	None	fig|6666666.71181.peg.3340	fig|6666666.59344.peg.3416	fig|6666666.59345.peg.2714	fig|6666666.59341.peg.152	fig|6666666.59347.peg.4635	fig|6666666.59346.peg.3356	fig|6666666.59348.peg.2993	fig|6666666.59349.peg.3412	None	fig|6666666.59351.peg.5000	fig|6666666.59353.peg.3798	fig|6666666.59355.peg.3394	fig|6666666.59354.peg.2222

532	Flagellar biosynthesis protein FliQ	fig|6666666.59329.peg.193	fig|6666666.59330.peg.1877	None	fig|6666666.59332.peg.4086	None	fig|6666666.59335.peg.398	fig|6666666.59336.peg.1274	fig|6666666.59333.peg.1676	fig|6666666.59338.peg.3098	fig|6666666.59339.peg.7086	fig|6666666.59340.peg.3179	fig|6666666.59337.peg.2968	fig|6666666.59342.peg.2649	fig|6666666.71181.peg.4934	fig|6666666.59344.peg.3052	fig|6666666.59345.peg.5334	fig|6666666.59341.peg.3275	fig|6666666.59347.peg.2682	fig|6666666.59346.peg.2888	fig|6666666.59348.peg.3695	fig|6666666.59349.peg.202	None	fig|6666666.59351.peg.2055	fig|6666666.59353.peg.3512	fig|6666666.59355.peg.3664	fig|6666666.59354.peg.674

3812	FIG003003 hypothetical protein	fig|6666666.59329.peg.5095	fig|6666666.59330.peg.2484	fig|6666666.59331.peg.1968	fig|6666666.59332.peg.5475	None	fig|6666666.59335.peg.953	fig|6666666.59336.peg.3635	fig|6666666.59333.peg.1048	fig|6666666.59338.peg.4013	fig|6666666.59339.peg.338	None	fig|6666666.59337.peg.4546	fig|6666666.59342.peg.1780	fig|6666666.71181.peg.1916	fig|6666666.59344.peg.2523	fig|6666666.59345.peg.1519	fig|6666666.59341.peg.2250	fig|6666666.59347.peg.4194	fig|6666666.59346.peg.2770	fig|6666666.59348.peg.3784	fig|6666666.59349.peg.3869	None	fig|6666666.59351.peg.651	fig|6666666.59353.peg.1493	fig|6666666.59355.peg.2043	fig|6666666.59354.peg.5532

3814	Hydroxymethylpyrimidine ABC transporter, substrate-binding component	fig|6666666.59329.peg.3210	fig|6666666.59330.peg.5171	None	fig|6666666.59332.peg.2531	fig|6666666.59334.peg.906	fig|6666666.59335.peg.6212	fig|6666666.59336.peg.4114	fig|6666666.59333.peg.3402	fig|6666666.59338.peg.467	fig|6666666.59339.peg.5551	None	fig|6666666.59337.peg.1219	None	fig|6666666.71181.peg.4177	fig|6666666.59344.peg.6271	fig|6666666.59345.peg.4404	fig|6666666.59341.peg.3560	fig|6666666.59347.peg.4311	fig|6666666.59346.peg.4173	fig|6666666.59348.peg.2770	fig|6666666.59349.peg.1130	fig|6666666.59350.peg.2045	fig|6666666.59351.peg.85	fig|6666666.59353.peg.2817	fig|6666666.59355.peg.3919	fig|6666666.59354.peg.291

4518	2,3-bisphosphoglycerate-independent phosphoglycerate mutase EC 5.4.2.1	fig|6666666.59329.peg.35	fig|6666666.59330.peg.141	None	fig|6666666.59332.peg.1902	fig|6666666.59334.peg.967	fig|6666666.59335.peg.1950	fig|6666666.59336.peg.1820	fig|6666666.59333.peg.2289	fig|6666666.59338.peg.1164	fig|6666666.59339.peg.8824	fig|6666666.59340.peg.935	fig|6666666.59337.peg.4997	None	fig|6666666.71181.peg.3676	fig|6666666.59344.peg.969	fig|6666666.59345.peg.5093	fig|6666666.59341.peg.185	fig|6666666.59347.peg.4540	fig|6666666.59346.peg.990	None	fig|6666666.59349.peg.2486	fig|6666666.59350.peg.7058	fig|6666666.59351.peg.372	fig|6666666.59353.peg.2569	fig|6666666.59355.peg.2632	fig|6666666.59354.peg.62

9283	Dephospho-CoA kinase EC 2.7.1.24	fig|6666666.59329.peg.342	None	None	fig|6666666.59332.peg.309	fig|6666666.59334.peg.5122	fig|6666666.59335.peg.3805	fig|6666666.59336.peg.1425	fig|6666666.59333.peg.155	fig|6666666.59338.peg.2612	fig|6666666.59339.peg.7941	None	fig|6666666.59337.peg.2047	fig|6666666.59342.peg.3661	fig|6666666.71181.peg.1967	fig|6666666.59344.peg.1464	fig|6666666.59345.peg.2427	fig|6666666.59341.peg.1348	fig|6666666.59347.peg.3890	fig|6666666.59346.peg.575	fig|6666666.59348.peg.1265	fig|6666666.59349.peg.2627	fig|6666666.59350.peg.2707	fig|6666666.59351.peg.275	fig|6666666.59353.peg.152	fig|6666666.59355.peg.762	fig|6666666.59354.peg.5549

11346	FIG01199944 hypothetical protein	fig|6666666.59329.peg.845	fig|6666666.59330.peg.4361	fig|6666666.59331.peg.1707	fig|6666666.59332.peg.1744	fig|6666666.59334.peg.1653	fig|6666666.59335.peg.1295	fig|6666666.59336.peg.2811	fig|6666666.59333.peg.3613	fig|6666666.59338.peg.3067	fig|6666666.59339.peg.3423	fig|6666666.59340.peg.1300	fig|6666666.59337.peg.4931	fig|6666666.59342.peg.804	fig|6666666.71181.peg.551	fig|6666666.59344.peg.1916	fig|6666666.59345.peg.1098	fig|6666666.59341.peg.3139	fig|6666666.59347.peg.2633	fig|6666666.59346.peg.548	None	fig|6666666.59349.peg.2950	None	fig|6666666.59351.peg.2823	fig|6666666.59353.peg.1181	fig|6666666.59355.peg.294	None

3796	Arylsulfatase EC 3.1.6.1	fig|6666666.59329.peg.380	fig|6666666.59330.peg.941	None	fig|6666666.59332.peg.5033	fig|6666666.59334.peg.534	fig|6666666.59335.peg.1509	None	fig|6666666.59333.peg.191	fig|6666666.59338.peg.214	fig|6666666.59339.peg.7396	fig|6666666.59340.peg.1093	fig|6666666.59337.peg.5	fig|6666666.59342.peg.1373	fig|6666666.71181.peg.3015	fig|6666666.59344.peg.4466	fig|6666666.59345.peg.3834	fig|6666666.59341.peg.1563	fig|6666666.59347.peg.3957	fig|6666666.59346.peg.3117	fig|6666666.59348.peg.1824	fig|6666666.59349.peg.3514	None	fig|6666666.59351.peg.3634	fig|6666666.59353.peg.4181	fig|6666666.59355.peg.1696	fig|6666666.59354.peg.5698

2064	Folate-dependent protein for Fe S cluster synthesis repair in oxidative stress	fig|6666666.59329.peg.489	fig|6666666.59330.peg.374	None	fig|6666666.59332.peg.739	fig|6666666.59334.peg.4790	fig|6666666.59335.peg.1725	fig|6666666.59336.peg.6192	fig|6666666.59333.peg.2747	fig|6666666.59338.peg.1525	fig|6666666.59339.peg.7353	None	fig|6666666.59337.peg.555	fig|6666666.59342.peg.5128	fig|6666666.71181.peg.3729	fig|6666666.59344.peg.3687	fig|6666666.59345.peg.5045	fig|6666666.59341.peg.4049	fig|6666666.59347.peg.3187	fig|6666666.59346.peg.323	fig|6666666.59348.peg.265	fig|6666666.59349.peg.2062	None	fig|6666666.59351.peg.587	fig|6666666.59353.peg.3830	fig|6666666.59355.peg.1557	fig|6666666.59354.peg.1389

629	D-3-phosphoglycerate dehydrogenase EC 1.1.1.95	fig|6666666.59329.peg.674	fig|6666666.59330.peg.3771	None	fig|6666666.59332.peg.134	fig|6666666.59334.peg.227	fig|6666666.59335.peg.5709	fig|6666666.59336.peg.3839	fig|6666666.59333.peg.2189	fig|6666666.59338.peg.1359	fig|6666666.59339.peg.6424	None	fig|6666666.59337.peg.4509	None	fig|6666666.71181.peg.870	fig|6666666.59344.peg.3	fig|6666666.59345.peg.5690	fig|6666666.59341.peg.1734	fig|6666666.59347.peg.5428	fig|6666666.59346.peg.1139	fig|6666666.59348.peg.3195	fig|6666666.59349.peg.1305	fig|6666666.59350.peg.3068	fig|6666666.59351.peg.493	fig|6666666.59353.peg.344	fig|6666666.59355.peg.1382	fig|6666666.59354.peg.2264

6865	Uracil-DNA glycosylase, family 1	fig|6666666.59329.peg.114	fig|6666666.59330.peg.1728	fig|6666666.59331.peg.5319	None	None	fig|6666666.59335.peg.811	None	fig|6666666.59333.peg.3410	fig|6666666.59338.peg.5640	fig|6666666.59339.peg.5000	fig|6666666.59340.peg.716	fig|6666666.59337.peg.97	fig|6666666.59342.peg.1359	fig|6666666.71181.peg.1438	fig|6666666.59344.peg.1548	fig|6666666.59345.peg.3746	fig|6666666.59341.peg.1145	fig|6666666.59347.peg.3066	fig|6666666.59346.peg.4041	fig|6666666.59348.peg.2699	fig|6666666.59349.peg.4232	fig|6666666.59350.peg.2777	fig|6666666.59351.peg.3912	fig|6666666.59353.peg.4351	fig|6666666.59355.peg.4491	fig|6666666.59354.peg.2752

5937	Exodeoxyribonuclease V gamma chain EC 3.1.11.5	fig|6666666.59329.peg.3860	fig|6666666.59330.peg.2133	fig|6666666.59331.peg.2934	fig|6666666.59332.peg.2484	fig|6666666.59334.peg.2766	fig|6666666.59335.peg.1127	fig|6666666.59336.peg.117	fig|6666666.59333.peg.4523	fig|6666666.59338.peg.1277	fig|6666666.59339.peg.6946	None	fig|6666666.59337.peg.2350	None	fig|6666666.71181.peg.64	fig|6666666.59344.peg.5804	fig|6666666.59345.peg.2262	fig|6666666.59341.peg.523	fig|6666666.59347.peg.2824	fig|6666666.59346.peg.1839	fig|6666666.59348.peg.298	fig|6666666.59349.peg.2649	None	fig|6666666.59351.peg.5191	fig|6666666.59353.peg.1951	fig|6666666.59355.peg.1456	fig|6666666.59354.peg.3085

2091	Arginine pathway regulatory protein ArgR, repressor of arg regulon	None	fig|6666666.59330.peg.830	fig|6666666.59331.peg.4159	fig|6666666.59332.peg.5200	fig|6666666.59334.peg.4468	fig|6666666.59335.peg.233	fig|6666666.59336.peg.5428	fig|6666666.59333.peg.556	fig|6666666.59338.peg.1819	fig|6666666.59339.peg.8169	None	fig|6666666.59337.peg.1370	fig|6666666.59342.peg.3583	fig|6666666.71181.peg.2799	fig|6666666.59344.peg.2124	fig|6666666.59345.peg.2472	fig|6666666.59341.peg.1988	fig|6666666.59347.peg.3101	fig|6666666.59346.peg.3987	fig|6666666.59348.peg.561	fig|6666666.59349.peg.353	None	fig|6666666.59351.peg.3959	fig|6666666.59353.peg.3157	fig|6666666.59355.peg.440	fig|6666666.59354.peg.388

9121	Putative metal chaperone, involved in Zn homeostasis, GTPase of C0523 family	fig|6666666.59329.peg.2708	fig|6666666.59330.peg.4519	fig|6666666.59331.peg.2713	fig|6666666.59332.peg.5222	fig|6666666.59334.peg.2009	fig|6666666.59335.peg.5330	fig|6666666.59336.peg.6340	fig|6666666.59333.peg.3497	fig|6666666.59338.peg.5831	fig|6666666.59339.peg.4726	fig|6666666.59340.peg.1809	fig|6666666.59337.peg.2145	fig|6666666.59342.peg.2262	fig|6666666.71181.peg.2999	fig|6666666.59344.peg.3999	fig|6666666.59345.peg.414	fig|6666666.59341.peg.1534	fig|6666666.59347.peg.2487	fig|6666666.59346.peg.1913	fig|6666666.59348.peg.3584	None	fig|6666666.59350.peg.3109	None	fig|6666666.59353.peg.1101	fig|6666666.59355.peg.556	None

9836	Paraquat-inducible protein B	fig|6666666.59329.peg.6531	fig|6666666.59330.peg.1020	fig|6666666.59331.peg.2980	fig|6666666.59332.peg.883	fig|6666666.59334.peg.4419	fig|6666666.59335.peg.4834	fig|6666666.59336.peg.4476	fig|6666666.59333.peg.746	fig|6666666.59338.peg.2720	fig|6666666.59339.peg.1543	None	fig|6666666.59337.peg.3161	fig|6666666.59342.peg.2359	fig|6666666.71181.peg.3832	fig|6666666.59344.peg.1181	fig|6666666.59345.peg.1014	fig|6666666.59341.peg.5902	fig|6666666.59347.peg.1779	fig|6666666.59346.peg.4869	None	fig|6666666.59349.peg.4422	None	fig|6666666.59351.peg.3742	fig|6666666.59353.peg.1686	fig|6666666.59355.peg.1034	fig|6666666.59354.peg.2304

1035	5-formyltetrahydrofolate cyclo-ligase EC 6.3.3.2	fig|6666666.59329.peg.241	fig|6666666.59330.peg.367	None	fig|6666666.59332.peg.539	fig|6666666.59334.peg.1050	fig|6666666.59335.peg.1732	fig|6666666.59336.peg.1191	fig|6666666.59333.peg.2754	fig|6666666.59338.peg.5637	fig|6666666.59339.peg.7345	None	fig|6666666.59337.peg.548	None	fig|6666666.71181.peg.5902	fig|6666666.59344.peg.4737	fig|6666666.59345.peg.5037	fig|6666666.59341.peg.3932	fig|6666666.59347.peg.3195	fig|6666666.59346.peg.316	fig|6666666.59348.peg.273	fig|6666666.59349.peg.349	fig|6666666.59350.peg.6383	fig|6666666.59351.peg.3955	fig|6666666.59353.peg.4435	fig|6666666.59355.peg.4515	fig|6666666.59354.peg.1491

4372	Flagellar M-ring protein FliF	fig|6666666.59329.peg.2733	fig|6666666.59330.peg.4699	None	fig|6666666.59332.peg.4855	None	fig|6666666.59335.peg.2398	fig|6666666.59336.peg.1449	fig|6666666.59333.peg.2998	fig|6666666.59338.peg.3339	fig|6666666.59339.peg.7101	fig|6666666.59340.peg.2770	fig|6666666.59337.peg.2958	fig|6666666.59342.peg.4160	fig|6666666.71181.peg.4028	fig|6666666.59344.peg.5218	fig|6666666.59345.peg.1480	fig|6666666.59341.peg.5967	fig|6666666.59347.peg.4628	fig|6666666.59346.peg.4718	fig|6666666.59348.peg.3872	fig|6666666.59349.peg.212	None	fig|6666666.59351.peg.2046	fig|6666666.59353.peg.4563	fig|6666666.59355.peg.5161	fig|6666666.59354.peg.1070

11066	Phage shock protein A	fig|6666666.59329.peg.4969	fig|6666666.59330.peg.1838	fig|6666666.59331.peg.1883	fig|6666666.59332.peg.4751	fig|6666666.59334.peg.5389	fig|6666666.59335.peg.4628	fig|6666666.59336.peg.5890	fig|6666666.59333.peg.5106	None	fig|6666666.59339.peg.521	None	fig|6666666.59337.peg.2327	fig|6666666.59342.peg.3326	fig|6666666.71181.peg.1389	fig|6666666.59344.peg.532	fig|6666666.59345.peg.1268	fig|6666666.59341.peg.1035	fig|6666666.59347.peg.3540	fig|6666666.59346.peg.1293	fig|6666666.59348.peg.2616	fig|6666666.59349.peg.2088	None	fig|6666666.59351.peg.2601	fig|6666666.59353.peg.1839	fig|6666666.59355.peg.1175	fig|6666666.59354.peg.3388

2173	Flagellar P-ring protein FlgI	fig|6666666.59329.peg.2685	fig|6666666.59330.peg.4411	None	fig|6666666.59332.peg.2239	None	fig|6666666.59335.peg.2089	fig|6666666.59336.peg.5046	fig|6666666.59333.peg.2844	fig|6666666.59338.peg.640	fig|6666666.59339.peg.5322	fig|6666666.59340.peg.2227	fig|6666666.59337.peg.3356	fig|6666666.59342.peg.6216	fig|6666666.71181.peg.2223	fig|6666666.59344.peg.3043	fig|6666666.59345.peg.2921	fig|6666666.59341.peg.5343	fig|6666666.59347.peg.4600	fig|6666666.59346.peg.1769	fig|6666666.59348.peg.956	fig|6666666.59349.peg.730	fig|6666666.59350.peg.5975	fig|6666666.59351.peg.1989	fig|6666666.59353.peg.3520	fig|6666666.59355.peg.3656	None

1930	Proline iminopeptidase EC 3.4.11.5	fig|6666666.59329.peg.1288	fig|6666666.59330.peg.2327	fig|6666666.59331.peg.2788	fig|6666666.59332.peg.6241	fig|6666666.59334.peg.2349	fig|6666666.59335.peg.1390	fig|6666666.59336.peg.1269	fig|6666666.59333.peg.2214	fig|6666666.59338.peg.5030	fig|6666666.59339.peg.6742	None	fig|6666666.59337.peg.3592	None	fig|6666666.71181.peg.861	fig|6666666.59344.peg.12	fig|6666666.59345.peg.1871	fig|6666666.59341.peg.2803	fig|6666666.59347.peg.3488	fig|6666666.59346.peg.4590	None	fig|6666666.59349.peg.1112	fig|6666666.59350.peg.232	fig|6666666.59351.peg.65	fig|6666666.59353.peg.353	fig|6666666.59355.peg.1391	fig|6666666.59354.peg.5037

1703	Indole-3-glycerol phosphate synthase EC 4.1.1.48 Phosphoribosylanthranilate isomerase EC 5.3.1.24	fig|6666666.59329.peg.399	fig|6666666.59330.peg.3336	None	fig|6666666.59332.peg.5516	fig|6666666.59334.peg.3051	None	fig|6666666.59336.peg.885	fig|6666666.59333.peg.1762	fig|6666666.59338.peg.3802	fig|6666666.59339.peg.3635	None	fig|6666666.59337.peg.3499	fig|6666666.59342.peg.760	fig|6666666.71181.peg.4144	fig|6666666.59344.peg.458	fig|6666666.59345.peg.918	fig|6666666.59341.peg.1020	fig|6666666.59347.peg.3556	fig|6666666.59346.peg.1601	fig|6666666.59348.peg.1832	fig|6666666.59349.peg.4275	fig|6666666.59350.peg.2778	fig|6666666.59351.peg.785	fig|6666666.59353.peg.1779	fig|6666666.59355.peg.1236	fig|6666666.59354.peg.5221

8431	Utilization protein for unknown catechol-siderophore X	fig|6666666.59329.peg.476	fig|6666666.59330.peg.1611	fig|6666666.59331.peg.4189	fig|6666666.59332.peg.3859	fig|6666666.59334.peg.1562	fig|6666666.59335.peg.3298	fig|6666666.59336.peg.3687	fig|6666666.59333.peg.3193	fig|6666666.59338.peg.5259	fig|6666666.59339.peg.2403	None	fig|6666666.59337.peg.4908	fig|6666666.59342.peg.695	fig|6666666.71181.peg.3755	fig|6666666.59344.peg.5579	fig|6666666.59345.peg.4477	fig|6666666.59341.peg.5778	fig|6666666.59347.peg.268	fig|6666666.59346.peg.2823	fig|6666666.59348.peg.4133	fig|6666666.59349.peg.4864	fig|6666666.59350.peg.8174	None	fig|6666666.59353.peg.4666	fig|6666666.59355.peg.3857	None

2586	Predicted membrane fusion protein MFP component of efflux pump, membrane anchor protein YbhG	fig|6666666.59329.peg.6350	fig|6666666.59330.peg.3308	None	fig|6666666.59332.peg.4009	fig|6666666.59334.peg.2402	fig|6666666.59335.peg.2019	fig|6666666.59336.peg.4380	fig|6666666.59333.peg.3340	fig|6666666.59338.peg.1231	fig|6666666.59339.peg.3961	None	fig|6666666.59337.peg.3096	fig|6666666.59342.peg.4711	fig|6666666.71181.peg.3541	fig|6666666.59344.peg.2082	fig|6666666.59345.peg.4155	fig|6666666.59341.peg.2023	fig|6666666.59347.peg.1607	fig|6666666.59346.peg.4738	fig|6666666.59348.peg.2887	fig|6666666.59349.peg.1467	None	fig|6666666.59351.peg.3032	fig|6666666.59353.peg.3119	fig|6666666.59355.peg.478	fig|6666666.59354.peg.1924

280	Phage shock protein E	fig|6666666.59329.peg.2380	fig|6666666.59330.peg.3541	None	fig|6666666.59332.peg.4713	fig|6666666.59334.peg.3377	fig|6666666.59335.peg.5512	fig|6666666.59336.peg.1067	fig|6666666.59333.peg.3603	fig|6666666.59338.peg.3062	fig|6666666.59339.peg.2006	fig|6666666.59340.peg.1046	fig|6666666.59337.peg.3241	None	fig|6666666.71181.peg.1499	fig|6666666.59344.peg.5818	fig|6666666.59345.peg.1787	fig|6666666.59341.peg.4391	fig|6666666.59347.peg.2137	fig|6666666.59346.peg.1342	fig|6666666.59348.peg.4022	fig|6666666.59349.peg.1895	None	fig|6666666.59351.peg.3593	fig|6666666.59353.peg.1231	fig|6666666.59355.peg.244	fig|6666666.59354.peg.3971

1071	tRNA 5-methylaminomethyl-2-thiouridylate -methyltransferase EC 2.1.1.61	fig|6666666.59329.peg.2411	fig|6666666.59330.peg.1591	None	fig|6666666.59332.peg.1956	fig|6666666.59334.peg.4033	fig|6666666.59335.peg.3935	fig|6666666.59336.peg.6220	fig|6666666.59333.peg.1972	fig|6666666.59338.peg.2575	fig|6666666.59339.peg.7060	None	fig|6666666.59337.peg.4840	fig|6666666.59342.peg.2132	fig|6666666.71181.peg.1506	fig|6666666.59344.peg.5006	fig|6666666.59345.peg.5696	fig|6666666.59341.peg.2434	fig|6666666.59347.peg.2462	fig|6666666.59346.peg.2658	fig|6666666.59348.peg.1545	fig|6666666.59349.peg.2638	None	fig|6666666.59351.peg.4911	fig|6666666.59353.peg.5013	fig|6666666.59355.peg.235	fig|6666666.59354.peg.4677

10480	Translation initiation factor 1	fig|6666666.59329.peg.2570	fig|6666666.59330.peg.1320	fig|6666666.59331.peg.2255	None	fig|6666666.59334.peg.874	fig|6666666.59335.peg.5951	fig|6666666.59336.peg.864	fig|6666666.59333.peg.1007	fig|6666666.59338.peg.5087	fig|6666666.59339.peg.286	fig|6666666.59340.peg.3707	fig|6666666.59337.peg.3371	fig|6666666.59342.peg.116	fig|6666666.71181.peg.4127	fig|6666666.59344.peg.403	fig|6666666.59345.peg.1553	fig|6666666.59341.peg.997	fig|6666666.59347.peg.4113	fig|6666666.59346.peg.2524	fig|6666666.59348.peg.1667	fig|6666666.59349.peg.2226	None	fig|6666666.59351.peg.878	fig|6666666.59353.peg.1623	fig|6666666.59355.peg.1100	None

3738	2-methylaconitate isomerase	None	fig|6666666.59330.peg.4304	fig|6666666.59331.peg.342	fig|6666666.59332.peg.516	None	fig|6666666.59335.peg.5769	fig|6666666.59336.peg.6432	fig|6666666.59333.peg.2963	fig|6666666.59338.peg.5494	fig|6666666.59339.peg.4028	None	fig|6666666.59337.peg.5505	fig|6666666.59342.peg.2944	fig|6666666.71181.peg.3872	fig|6666666.59344.peg.869	fig|6666666.59345.peg.4095	fig|6666666.59341.peg.5235	fig|6666666.59347.peg.1621	fig|6666666.59346.peg.181	fig|6666666.59348.peg.4216	fig|6666666.59349.peg.2464	fig|6666666.59350.peg.4086	fig|6666666.59351.peg.1220	fig|6666666.59353.peg.1249	fig|6666666.59355.peg.3336	fig|6666666.59354.peg.992

2781	Hydrolase, alpha beta fold family functionally coupled to Phosphoribulokinase	fig|6666666.59329.peg.4	fig|6666666.59330.peg.1455	None	fig|6666666.59332.peg.3185	fig|6666666.59334.peg.719	fig|6666666.59335.peg.1872	fig|6666666.59336.peg.287	fig|6666666.59333.peg.2357	fig|6666666.59338.peg.2639	fig|6666666.59339.peg.8236	fig|6666666.59340.peg.3263	fig|6666666.59337.peg.1807	None	fig|6666666.71181.peg.1803	fig|6666666.59344.peg.3099	fig|6666666.59345.peg.5566	fig|6666666.59341.peg.2768	fig|6666666.59347.peg.4961	fig|6666666.59346.peg.28	fig|6666666.59348.peg.284	fig|6666666.59349.peg.3377	None	fig|6666666.59351.peg.4173	fig|6666666.59353.peg.2248	fig|6666666.59355.peg.1940	fig|6666666.59354.peg.5899

2780	Flavodoxin 1	fig|6666666.59329.peg.1355	fig|6666666.59330.peg.2996	None	fig|6666666.59332.peg.5169	fig|6666666.59334.peg.4533	fig|6666666.59335.peg.5790	fig|6666666.59336.peg.332	fig|6666666.59333.peg.860	fig|6666666.59338.peg.1152	fig|6666666.59339.peg.7207	fig|6666666.59340.peg.2463	fig|6666666.59337.peg.3934	fig|6666666.59342.peg.2212	fig|6666666.71181.peg.3809	fig|6666666.59344.peg.5088	fig|6666666.59345.peg.4333	None	fig|6666666.59347.peg.2942	fig|6666666.59346.peg.2007	fig|6666666.59348.peg.1060	fig|6666666.59349.peg.1816	None	fig|6666666.59351.peg.1115	fig|6666666.59353.peg.2057	fig|6666666.59355.peg.2770	fig|6666666.59354.peg.4090

1241	UPF0229 protein YeaH	None	fig|6666666.59330.peg.1260	None	fig|6666666.59332.peg.5423	fig|6666666.59334.peg.5436	fig|6666666.59335.peg.2826	fig|6666666.59336.peg.3389	fig|6666666.59333.peg.1838	fig|6666666.59338.peg.4948	fig|6666666.59339.peg.3480	None	fig|6666666.59337.peg.2204	fig|6666666.59342.peg.4443	fig|6666666.71181.peg.2966	fig|6666666.59344.peg.2887	fig|6666666.59345.peg.1585	fig|6666666.59341.peg.3940	fig|6666666.59347.peg.4168	fig|6666666.59346.peg.3247	fig|6666666.59348.peg.1978	fig|6666666.59349.peg.2396	fig|6666666.59350.peg.4530	fig|6666666.59351.peg.634	fig|6666666.59353.peg.1438	fig|6666666.59355.peg.1987	fig|6666666.59354.peg.6416

9798	ABC transporter, periplasmic spermidine putrescine-binding protein PotD TC 3.A.1.11.1	fig|6666666.59329.peg.5250	fig|6666666.59330.peg.2603	fig|6666666.59331.peg.4046	fig|6666666.59332.peg.4161	fig|6666666.59334.peg.889	fig|6666666.59335.peg.2208	fig|6666666.59336.peg.6139	fig|6666666.59333.peg.3975	fig|6666666.59338.peg.4143	fig|6666666.59339.peg.1301	fig|6666666.59340.peg.2764	fig|6666666.59337.peg.3708	fig|6666666.59342.peg.224	fig|6666666.71181.peg.4484	fig|6666666.59344.peg.361	fig|6666666.59345.peg.3218	fig|6666666.59341.peg.4673	fig|6666666.59347.peg.679	fig|6666666.59346.peg.264	fig|6666666.59348.peg.2861	None	fig|6666666.59350.peg.179	None	fig|6666666.59353.peg.448	fig|6666666.59355.peg.2175	None

9163	YgfY C2938	fig|6666666.59329.peg.491	fig|6666666.59330.peg.375	fig|6666666.59331.peg.5301	fig|6666666.59332.peg.738	fig|6666666.59334.peg.5247	fig|6666666.59335.peg.1724	fig|6666666.59336.peg.5364	fig|6666666.59333.peg.2746	fig|6666666.59338.peg.1524	None	None	fig|6666666.59337.peg.556	fig|6666666.59342.peg.4753	fig|6666666.71181.peg.3730	fig|6666666.59344.peg.3686	fig|6666666.59345.peg.5046	fig|6666666.59341.peg.4050	fig|6666666.59347.peg.3185	fig|6666666.59346.peg.324	fig|6666666.59348.peg.264	fig|6666666.59349.peg.2061	None	fig|6666666.59351.peg.588	fig|6666666.59353.peg.3831	fig|6666666.59355.peg.1556	fig|6666666.59354.peg.1388

6820	N-acetylmuramic acid 6-phosphate etherase EC 4.2.-.-	fig|6666666.59329.peg.1384	fig|6666666.59330.peg.2521	fig|6666666.59331.peg.2740	fig|6666666.59332.peg.1089	fig|6666666.59334.peg.1016	fig|6666666.59335.peg.1000	fig|6666666.59336.peg.5943	fig|6666666.59333.peg.3501	fig|6666666.59338.peg.5737	fig|6666666.59339.peg.7476	fig|6666666.59340.peg.1168	fig|6666666.59337.peg.253	fig|6666666.59342.peg.5924	fig|6666666.71181.peg.1414	fig|6666666.59344.peg.1576	fig|6666666.59345.peg.3700	fig|6666666.59341.peg.99	fig|6666666.59347.peg.4040	fig|6666666.59346.peg.247	fig|6666666.59348.peg.5101	None	fig|6666666.59350.peg.4668	None	fig|6666666.59353.peg.2663	fig|6666666.59355.peg.1820	None

1511	Transcriptional regulator, IclR family	fig|6666666.59329.peg.1890	fig|6666666.59330.peg.4973	fig|6666666.59331.peg.783	None	fig|6666666.59334.peg.947	fig|6666666.59335.peg.3661	fig|6666666.59336.peg.3531	fig|6666666.59333.peg.3558	fig|6666666.59338.peg.4625	fig|6666666.59339.peg.3291	fig|6666666.59340.peg.771	fig|6666666.59337.peg.5509	fig|6666666.59342.peg.3681	fig|6666666.71181.peg.4666	fig|6666666.59344.peg.3819	fig|6666666.59345.peg.408	fig|6666666.59341.peg.1265	fig|6666666.59347.peg.3837	fig|6666666.59346.peg.2359	fig|6666666.59348.peg.507	fig|6666666.59349.peg.4954	fig|6666666.59350.peg.4383	None	fig|6666666.59353.peg.2726	fig|6666666.59355.peg.3309	None

6017	ATP synthase protein I	fig|6666666.59329.peg.1746	fig|6666666.59330.peg.1119	fig|6666666.59331.peg.399	fig|6666666.59332.peg.98	None	fig|6666666.59335.peg.791	fig|6666666.59336.peg.27	None	fig|6666666.59338.peg.492	fig|6666666.59339.peg.8015	fig|6666666.59340.peg.748	fig|6666666.59337.peg.889	fig|6666666.59342.peg.1195	fig|6666666.71181.peg.591	fig|6666666.59344.peg.4264	fig|6666666.59345.peg.4966	fig|6666666.59341.peg.1058	fig|6666666.59347.peg.4784	fig|6666666.59346.peg.1402	fig|6666666.59348.peg.331	fig|6666666.59349.peg.1029	None	fig|6666666.59351.peg.5360	fig|6666666.59353.peg.3089	fig|6666666.59355.peg.2259	fig|6666666.59354.peg.1150

3175	C0613, Predicted metal-dependent phosphoesterases PHP family	fig|6666666.59329.peg.6313	fig|6666666.59330.peg.4899	fig|6666666.59331.peg.2409	None	None	fig|6666666.59335.peg.3717	fig|6666666.59336.peg.880	fig|6666666.59333.peg.4238	fig|6666666.59338.peg.3476	fig|6666666.59339.peg.3642	fig|6666666.59340.peg.3090	fig|6666666.59337.peg.1729	fig|6666666.59342.peg.766	fig|6666666.71181.peg.4149	fig|6666666.59344.peg.464	fig|6666666.59345.peg.913	fig|6666666.59341.peg.1024	fig|6666666.59347.peg.3552	fig|6666666.59346.peg.1607	fig|6666666.59348.peg.4338	fig|6666666.59349.peg.4270	None	fig|6666666.59351.peg.790	fig|6666666.59353.peg.1784	fig|6666666.59355.peg.1231	fig|6666666.59354.peg.3827

8555	Aminomethyltransferase glycine cleavage system T protein EC 2.1.2.10	fig|6666666.59329.peg.6077	fig|6666666.59330.peg.3587	None	fig|6666666.59332.peg.5073	fig|6666666.59334.peg.2342	fig|6666666.59335.peg.5916	None	fig|6666666.59333.peg.4347	fig|6666666.59338.peg.1399	fig|6666666.59339.peg.2100	None	fig|6666666.59337.peg.4829	fig|6666666.59342.peg.6241	fig|6666666.71181.peg.3126	fig|6666666.59344.peg.4745	fig|6666666.59345.peg.171	fig|6666666.59341.peg.438	fig|6666666.59347.peg.1401	fig|6666666.59346.peg.3490	fig|6666666.59348.peg.3348	fig|6666666.59349.peg.4506	fig|6666666.59350.peg.1062	fig|6666666.59351.peg.2188	fig|6666666.59353.peg.4442	fig|6666666.59355.peg.4521	fig|6666666.59354.peg.3301

2032	OsmC Ohr family protein	fig|6666666.59329.peg.1502	fig|6666666.59330.peg.2358	fig|6666666.59331.peg.3742	fig|6666666.59332.peg.4905	None	fig|6666666.59335.peg.4343	fig|6666666.59336.peg.1131	fig|6666666.59333.peg.3079	fig|6666666.59338.peg.3132	fig|6666666.59339.peg.4356	None	fig|6666666.59337.peg.2229	fig|6666666.59342.peg.4784	fig|6666666.71181.peg.1807	fig|6666666.59344.peg.3095	fig|6666666.59345.peg.2989	fig|6666666.59341.peg.767	fig|6666666.59347.peg.842	fig|6666666.59346.peg.1059	fig|6666666.59348.peg.2517	fig|6666666.59349.peg.3801	None	fig|6666666.59351.peg.3878	fig|6666666.59353.peg.2244	fig|6666666.59355.peg.1936	fig|6666666.59354.peg.3998

4935	LSU ribosomal protein L28p	fig|6666666.59329.peg.4987	fig|6666666.59330.peg.1168	fig|6666666.59331.peg.4157	fig|6666666.59332.peg.297	fig|6666666.59334.peg.186	fig|6666666.59335.peg.1994	fig|6666666.59336.peg.53	fig|6666666.59333.peg.655	fig|6666666.59338.peg.1113	fig|6666666.59339.peg.6290	fig|6666666.59340.peg.9	fig|6666666.59337.peg.473	fig|6666666.59342.peg.5882	None	None	fig|6666666.59345.peg.2620	fig|6666666.59341.peg.207	fig|6666666.59347.peg.4520	fig|6666666.59346.peg.1714	fig|6666666.59348.peg.725	fig|6666666.59349.peg.112	fig|6666666.59350.peg.4900	fig|6666666.59351.peg.168	None	fig|6666666.59355.peg.6007	fig|6666666.59354.peg.3707

10240	LSU rRNA 2'-O-methyl-C2498 methyltransferase RlmM	fig|6666666.59329.peg.450	fig|6666666.59330.peg.1043	fig|6666666.59331.peg.3306	fig|6666666.59332.peg.1973	fig|6666666.59334.peg.1986	fig|6666666.59335.peg.5508	fig|6666666.59336.peg.1876	fig|6666666.59333.peg.4315	fig|6666666.59338.peg.1652	fig|6666666.59339.peg.7163	None	fig|6666666.59337.peg.1122	fig|6666666.59342.peg.6120	fig|6666666.71181.peg.51	fig|6666666.59344.peg.2627	fig|6666666.59345.peg.3525	fig|6666666.59341.peg.5083	fig|6666666.59347.peg.2934	fig|6666666.59346.peg.1734	None	fig|6666666.59349.peg.2625	None	fig|6666666.59351.peg.4651	fig|6666666.59353.peg.1966	fig|6666666.59355.peg.1440	fig|6666666.59354.peg.5474

5628	ProQ influences osmotic activation of compatible solute ProP	None	fig|6666666.59330.peg.1013	fig|6666666.59331.peg.4098	fig|6666666.59332.peg.876	fig|6666666.59334.peg.926	fig|6666666.59335.peg.3355	fig|6666666.59336.peg.4554	fig|6666666.59333.peg.2669	fig|6666666.59338.peg.5100	fig|6666666.59339.peg.1537	None	fig|6666666.59337.peg.3155	fig|6666666.59342.peg.783	fig|6666666.71181.peg.3835	fig|6666666.59344.peg.847	fig|6666666.59345.peg.3262	fig|6666666.59341.peg.4222	fig|6666666.59347.peg.1775	fig|6666666.59346.peg.3401	fig|6666666.59348.peg.4403	fig|6666666.59349.peg.4757	None	fig|6666666.59351.peg.3150	fig|6666666.59353.peg.1268	fig|6666666.59355.peg.3315	fig|6666666.59354.peg.4704

5623	Sulfur carrier protein ThiS	fig|6666666.59329.peg.3985	fig|6666666.59330.peg.980	fig|6666666.59331.peg.1272	fig|6666666.59332.peg.252	fig|6666666.59334.peg.2194	fig|6666666.59335.peg.8	fig|6666666.59336.peg.177	fig|6666666.59333.peg.1567	None	None	fig|6666666.59340.peg.3416	fig|6666666.59337.peg.2551	None	fig|6666666.71181.peg.3360	fig|6666666.59344.peg.5691	fig|6666666.59345.peg.5543	fig|6666666.59341.peg.3743	fig|6666666.59347.peg.5255	fig|6666666.59346.peg.932	fig|6666666.59348.peg.3770	fig|6666666.59349.peg.4746	fig|6666666.59350.peg.2930	fig|6666666.59351.peg.3885	fig|6666666.59353.peg.4463	fig|6666666.59355.peg.4564	fig|6666666.59354.peg.6291

5997	Tricarboxylate transport membrane protein TctA	fig|6666666.59329.peg.5314	fig|6666666.59330.peg.4107	fig|6666666.59331.peg.1171	fig|6666666.59332.peg.1378	fig|6666666.59334.peg.4011	fig|6666666.59335.peg.2023	fig|6666666.59336.peg.6385	fig|6666666.59333.peg.3042	fig|6666666.59338.peg.4563	fig|6666666.59339.peg.3964	fig|6666666.59340.peg.2424	fig|6666666.59337.peg.3114	fig|6666666.59342.peg.2644	fig|6666666.71181.peg.1490	fig|6666666.59344.peg.1867	fig|6666666.59345.peg.4128	fig|6666666.59341.peg.5964	fig|6666666.59347.peg.1312	fig|6666666.59346.peg.3012	fig|6666666.59348.peg.2884	None	fig|6666666.59350.peg.2219	None	fig|6666666.59353.peg.1222	fig|6666666.59355.peg.254	None

9247	Formate efflux transporter TC 2.A.44 family	fig|6666666.59329.peg.5880	fig|6666666.59330.peg.4786	fig|6666666.59331.peg.1982	fig|6666666.59332.peg.3191	fig|6666666.59334.peg.552	fig|6666666.59335.peg.2351	fig|6666666.59336.peg.2594	fig|6666666.59333.peg.4473	fig|6666666.59338.peg.4544	fig|6666666.59339.peg.2878	fig|6666666.59340.peg.1972	fig|6666666.59337.peg.1185	fig|6666666.59342.peg.556	fig|6666666.71181.peg.1531	fig|6666666.59344.peg.2036	fig|6666666.59345.peg.128	fig|6666666.59341.peg.5209	fig|6666666.59347.peg.870	fig|6666666.59346.peg.4994	fig|6666666.59348.peg.5480	None	fig|6666666.59350.peg.4821	None	fig|6666666.59353.peg.2893	fig|6666666.59355.peg.2846	None

3522	Glucosamine kinase GpsK EC 2.7.1.8	fig|6666666.59329.peg.854	None	fig|6666666.59331.peg.2096	fig|6666666.59332.peg.238	fig|6666666.59334.peg.1246	fig|6666666.59335.peg.1535	fig|6666666.59336.peg.2389	fig|6666666.59333.peg.335	fig|6666666.59338.peg.5008	fig|6666666.59339.peg.4809	None	fig|6666666.59337.peg.648	None	fig|6666666.71181.peg.2662	fig|6666666.59344.peg.4313	fig|6666666.59345.peg.2387	fig|6666666.59341.peg.1809	fig|6666666.59347.peg.3149	fig|6666666.59346.peg.763	fig|6666666.59348.peg.3736	fig|6666666.59349.peg.2665	fig|6666666.59350.peg.2802	fig|6666666.59351.peg.5158	fig|6666666.59353.peg.3877	fig|6666666.59355.peg.5954	fig|6666666.59354.peg.2881

2695	Methyl-directed repair DNA adenine methylase EC 2.1.1.72	fig|6666666.59329.peg.318	fig|6666666.59330.peg.2638	None	None	fig|6666666.59334.peg.3699	fig|6666666.59335.peg.2482	fig|6666666.59336.peg.592	fig|6666666.59333.peg.893	fig|6666666.59338.peg.3575	fig|6666666.59339.peg.8711	fig|6666666.59340.peg.73	fig|6666666.59337.peg.1704	fig|6666666.59342.peg.1888	fig|6666666.71181.peg.1829	fig|6666666.59344.peg.3068	fig|6666666.59345.peg.5378	fig|6666666.59341.peg.4077	fig|6666666.59347.peg.4947	fig|6666666.59346.peg.9	fig|6666666.59348.peg.1026	fig|6666666.59349.peg.531	None	fig|6666666.59351.peg.3854	fig|6666666.59353.peg.2221	fig|6666666.59355.peg.1915	fig|6666666.59354.peg.3572

1146	H + Cl - exchange transporter ClcA	fig|6666666.59329.peg.4471	fig|6666666.59330.peg.1143	fig|6666666.59331.peg.5719	fig|6666666.59332.peg.5893	fig|6666666.59334.peg.458	fig|6666666.59335.peg.1880	fig|6666666.59336.peg.2483	None	fig|6666666.59338.peg.6145	fig|6666666.59339.peg.4724	None	fig|6666666.59337.peg.3643	fig|6666666.59342.peg.5031	fig|6666666.71181.peg.2720	fig|6666666.59344.peg.3823	fig|6666666.59345.peg.5204	fig|6666666.59341.peg.1536	fig|6666666.59347.peg.698	fig|6666666.59346.peg.1912	None	fig|6666666.59349.peg.336	fig|6666666.59350.peg.1051	fig|6666666.59351.peg.3941	fig|6666666.59353.peg.161	fig|6666666.59355.peg.772	fig|6666666.59354.peg.4176

7192	LptA, protein essential for LPS transport across the periplasm	fig|6666666.59329.peg.819	fig|6666666.59330.peg.47	None	None	fig|6666666.59334.peg.99	fig|6666666.59335.peg.3050	fig|6666666.59336.peg.351	fig|6666666.59333.peg.264	fig|6666666.59338.peg.108	fig|6666666.59339.peg.8200	None	fig|6666666.59337.peg.831	fig|6666666.59342.peg.5646	fig|6666666.71181.peg.1018	fig|6666666.59344.peg.1798	fig|6666666.59345.peg.4859	fig|6666666.59341.peg.1244	fig|6666666.59347.peg.3251	fig|6666666.59346.peg.638	fig|6666666.59348.peg.2008	fig|6666666.59349.peg.22	fig|6666666.59350.peg.1642	fig|6666666.59351.peg.3441	fig|6666666.59353.peg.2336	fig|6666666.59355.peg.1610	fig|6666666.59354.peg.3621

4289	TRAP-type transport system, small permease component, predicted N-acetylneuraminate transporter	fig|6666666.59329.peg.3540	fig|6666666.59330.peg.287	fig|6666666.59331.peg.3202	fig|6666666.59332.peg.5780	fig|6666666.59334.peg.3505	fig|6666666.59335.peg.2703	fig|6666666.59336.peg.504	fig|6666666.59333.peg.2184	fig|6666666.59338.peg.1949	fig|6666666.59339.peg.3750	fig|6666666.59340.peg.756	fig|6666666.59337.peg.3583	fig|6666666.59342.peg.3879	fig|6666666.71181.peg.3633	fig|6666666.59344.peg.5268	fig|6666666.59345.peg.865	fig|6666666.59341.peg.5074	fig|6666666.59347.peg.4425	fig|6666666.59346.peg.4540	fig|6666666.59348.peg.5357	None	fig|6666666.59350.peg.1520	None	fig|6666666.59353.peg.4705	fig|6666666.59355.peg.4785	None

10366	Carbon storage regulator	fig|6666666.59329.peg.2807	fig|6666666.59330.peg.4214	None	fig|6666666.59332.peg.2080	fig|6666666.59334.peg.287	fig|6666666.59335.peg.266	fig|6666666.59336.peg.5560	fig|6666666.59333.peg.1726	fig|6666666.59338.peg.756	fig|6666666.59339.peg.8110	None	fig|6666666.59337.peg.5449	fig|6666666.59342.peg.3839	fig|6666666.71181.peg.3468	fig|6666666.59344.peg.2164	fig|6666666.59345.peg.5083	fig|6666666.59341.peg.4219	fig|6666666.59347.peg.3224	fig|6666666.59346.peg.4974	fig|6666666.59348.peg.1340	fig|6666666.59349.peg.1833	None	fig|6666666.59351.peg.5266	fig|6666666.59353.peg.233	fig|6666666.59355.peg.3004	fig|6666666.59354.peg.595

7204	PQQ-dependent oxidoreductase, gdhB family	fig|6666666.59329.peg.1151	fig|6666666.59330.peg.924	fig|6666666.59331.peg.4834	fig|6666666.59332.peg.2308	fig|6666666.59334.peg.4150	fig|6666666.59335.peg.6086	None	fig|6666666.59333.peg.2166	fig|6666666.59338.peg.3068	fig|6666666.59339.peg.583	fig|6666666.59340.peg.1301	fig|6666666.59337.peg.3950	fig|6666666.59342.peg.5852	fig|6666666.71181.peg.5121	fig|6666666.59344.peg.322	fig|6666666.59345.peg.437	fig|6666666.59341.peg.3140	fig|6666666.59347.peg.2632	fig|6666666.59346.peg.1937	fig|6666666.59348.peg.2959	None	fig|6666666.59350.peg.594	fig|6666666.59351.peg.739	fig|6666666.59353.peg.560	fig|6666666.59355.peg.45	None

10506	1-aminocyclopropane-1-carboxylate deaminase EC 3.5.99.7	fig|6666666.59329.peg.3875	fig|6666666.59330.peg.4841	fig|6666666.59331.peg.5028	fig|6666666.59332.peg.5406	fig|6666666.59334.peg.4975	fig|6666666.59335.peg.5800	fig|6666666.59336.peg.1929	fig|6666666.59333.peg.2012	None	fig|6666666.59339.peg.423	fig|6666666.59340.peg.768	fig|6666666.59337.peg.2406	None	fig|6666666.71181.peg.4040	fig|6666666.59344.peg.5205	fig|6666666.59345.peg.954	fig|6666666.59341.peg.2065	fig|6666666.59347.peg.2353	fig|6666666.59346.peg.2414	fig|6666666.59348.peg.2661	fig|6666666.59349.peg.2	None	fig|6666666.59351.peg.3419	fig|6666666.59353.peg.4575	fig|6666666.59355.peg.3839	fig|6666666.59354.peg.4694

2502	Ribosomal RNA large subunit methyltransferase F EC 2.1.1.51	fig|6666666.59329.peg.5285	fig|6666666.59330.peg.4647	fig|6666666.59331.peg.1765	fig|6666666.59332.peg.4426	fig|6666666.59334.peg.1595	fig|6666666.59335.peg.2009	None	fig|6666666.59333.peg.3532	fig|6666666.59338.peg.5851	fig|6666666.59339.peg.3946	None	fig|6666666.59337.peg.2002	fig|6666666.59342.peg.4707	fig|6666666.71181.peg.2118	fig|6666666.59344.peg.5320	fig|6666666.59345.peg.5591	fig|6666666.59341.peg.723	fig|6666666.59347.peg.3447	fig|6666666.59346.peg.5072	fig|6666666.59348.peg.1726	fig|6666666.59349.peg.3691	None	fig|6666666.59351.peg.3800	fig|6666666.59353.peg.708	fig|6666666.59355.peg.195	fig|6666666.59354.peg.2809

2869	Short chain dehydrogenase	fig|6666666.59329.peg.6555	fig|6666666.59330.peg.1204	fig|6666666.59331.peg.6150	fig|6666666.59332.peg.3138	fig|6666666.59334.peg.755	fig|6666666.59335.peg.1443	fig|6666666.59336.peg.2979	fig|6666666.59333.peg.4497	fig|6666666.59338.peg.4939	fig|6666666.59339.peg.184	fig|6666666.59340.peg.1124	fig|6666666.59337.peg.3138	fig|6666666.59342.peg.4017	fig|6666666.71181.peg.5160	None	fig|6666666.59345.peg.179	fig|6666666.59341.peg.1118	fig|6666666.59347.peg.2582	fig|6666666.59346.peg.92	fig|6666666.59348.peg.2423	fig|6666666.59349.peg.2340	fig|6666666.59350.peg.2819	fig|6666666.59351.peg.1217	None	None	fig|6666666.59354.peg.2103

7229	Conserved uncharacterized protein CreA	fig|6666666.59329.peg.3580	fig|6666666.59330.peg.3420	fig|6666666.59331.peg.5850	fig|6666666.59332.peg.4694	fig|6666666.59334.peg.3213	fig|6666666.59335.peg.4515	fig|6666666.59336.peg.5937	fig|6666666.59333.peg.4911	fig|6666666.59338.peg.2043	fig|6666666.59339.peg.8456	None	fig|6666666.59337.peg.2501	fig|6666666.59342.peg.237	fig|6666666.71181.peg.439	fig|6666666.59344.peg.1343	fig|6666666.59345.peg.143	fig|6666666.59341.peg.5296	fig|6666666.59347.peg.818	fig|6666666.59346.peg.4547	fig|6666666.59348.peg.5139	fig|6666666.59349.peg.1853	None	fig|6666666.59351.peg.5126	fig|6666666.59353.peg.59	fig|6666666.59355.peg.666	None

9174	FIG137478 Hypothetical protein YbgI	fig|6666666.59329.peg.1348	fig|6666666.59330.peg.4932	fig|6666666.59331.peg.67	fig|6666666.59332.peg.5672	None	fig|6666666.59335.peg.2736	fig|6666666.59336.peg.5430	fig|6666666.59333.peg.854	fig|6666666.59338.peg.3195	fig|6666666.59339.peg.7198	fig|6666666.59340.peg.2534	fig|6666666.59337.peg.4103	fig|6666666.59342.peg.6496	fig|6666666.71181.peg.3223	fig|6666666.59344.peg.4609	fig|6666666.59345.peg.4327	fig|6666666.59341.peg.2526	fig|6666666.59347.peg.4380	fig|6666666.59346.peg.5330	None	fig|6666666.59349.peg.1900	None	fig|6666666.59351.peg.3600	fig|6666666.59353.peg.2075	fig|6666666.59355.peg.2751	fig|6666666.59354.peg.3966

10756	Protein ydjA	fig|6666666.59329.peg.2277	fig|6666666.59330.peg.1208	None	None	None	fig|6666666.59335.peg.1263	fig|6666666.59336.peg.3182	fig|6666666.59333.peg.1078	fig|6666666.59338.peg.2203	fig|6666666.59339.peg.3053	fig|6666666.59340.peg.2236	fig|6666666.59337.peg.3134	fig|6666666.59342.peg.6495	fig|6666666.71181.peg.2976	fig|6666666.59344.peg.2899	fig|6666666.59345.peg.1157	fig|6666666.59341.peg.1115	fig|6666666.59347.peg.2584	fig|6666666.59346.peg.86	fig|6666666.59348.peg.459	fig|6666666.59349.peg.390	fig|6666666.59350.peg.2625	fig|6666666.59351.peg.929	fig|6666666.59353.peg.1428	fig|6666666.59355.peg.1977	fig|6666666.59354.peg.5823

8721	SSU ribosomal protein S7p S5e	fig|6666666.59329.peg.4638	fig|6666666.59330.peg.4070	fig|6666666.59331.peg.4324	fig|6666666.59332.peg.1079	fig|6666666.59334.peg.443	fig|6666666.59335.peg.5581	None	fig|6666666.59333.peg.3101	fig|6666666.59338.peg.3253	fig|6666666.59339.peg.8875	fig|6666666.59340.peg.895	fig|6666666.59337.peg.1825	fig|6666666.59342.peg.1925	fig|6666666.71181.peg.4313	None	fig|6666666.59345.peg.5550	fig|6666666.59341.peg.5091	fig|6666666.59347.peg.5330	fig|6666666.59346.peg.47	fig|6666666.59348.peg.3176	fig|6666666.59349.peg.1138	fig|6666666.59350.peg.3772	fig|6666666.59351.peg.5247	None	fig|6666666.59355.peg.6104	fig|6666666.59354.peg.3579

10539	1-acyl-sn-glycerol-3-phosphate acyltransferase EC 2.3.1.51	fig|6666666.59329.peg.49	fig|6666666.59330.peg.629	None	fig|6666666.59332.peg.2575	fig|6666666.59334.peg.4503	fig|6666666.59335.peg.2446	fig|6666666.59336.peg.2736	fig|6666666.59333.peg.7	fig|6666666.59338.peg.572	fig|6666666.59339.peg.8072	fig|6666666.59340.peg.2095	fig|6666666.59337.peg.3888	None	fig|6666666.71181.peg.2582	fig|6666666.59344.peg.891	fig|6666666.59345.peg.4940	fig|6666666.59341.peg.2189	fig|6666666.59347.peg.4761	fig|6666666.59346.peg.3198	fig|6666666.59348.peg.64	fig|6666666.59349.peg.2204	None	fig|6666666.59351.peg.4189	fig|6666666.59353.peg.2635	fig|6666666.59355.peg.2570	fig|6666666.59354.peg.3021

9957	Cell division protein FtsB	fig|6666666.59329.peg.6546	fig|6666666.59330.peg.2464	None	fig|6666666.59332.peg.198	fig|6666666.59334.peg.333	fig|6666666.59335.peg.1654	fig|6666666.59336.peg.4982	fig|6666666.59333.peg.4776	fig|6666666.59338.peg.224	fig|6666666.59339.peg.7916	None	fig|6666666.59337.peg.577	fig|6666666.59342.peg.1277	fig|6666666.71181.peg.3415	fig|6666666.59344.peg.2198	fig|6666666.59345.peg.5068	fig|6666666.59341.peg.655	fig|6666666.59347.peg.3948	fig|6666666.59346.peg.3150	fig|6666666.59348.peg.3457	fig|6666666.59349.peg.4380	None	fig|6666666.59351.peg.5479	fig|6666666.59353.peg.208	fig|6666666.59355.peg.2977	fig|6666666.59354.peg.3139

209	Phosphoserine aminotransferase EC 2.6.1.52	fig|6666666.59329.peg.1190	fig|6666666.59330.peg.1991	None	fig|6666666.59332.peg.5989	fig|6666666.59334.peg.4042	fig|6666666.59335.peg.5929	fig|6666666.59336.peg.5709	fig|6666666.59333.peg.5552	fig|6666666.59338.peg.3919	fig|6666666.59339.peg.3536	None	fig|6666666.59337.peg.4650	fig|6666666.59342.peg.205	fig|6666666.71181.peg.3164	fig|6666666.59344.peg.2803	fig|6666666.59345.peg.4587	fig|6666666.59341.peg.365	fig|6666666.59347.peg.1970	fig|6666666.59346.peg.5225	fig|6666666.59348.peg.5131	fig|6666666.59349.peg.380	None	fig|6666666.59351.peg.938	fig|6666666.59353.peg.1644	fig|6666666.59355.peg.1077	fig|6666666.59354.peg.1757

903	Ribonuclease T EC 3.1.13.-	fig|6666666.59329.peg.790	fig|6666666.59330.peg.3382	fig|6666666.59331.peg.3023	fig|6666666.59332.peg.2566	None	fig|6666666.59335.peg.1202	fig|6666666.59336.peg.3559	fig|6666666.59333.peg.3176	fig|6666666.59338.peg.1829	fig|6666666.59339.peg.47	fig|6666666.59340.peg.3608	fig|6666666.59337.peg.4417	fig|6666666.59342.peg.1049	fig|6666666.71181.peg.2543	fig|6666666.59344.peg.3513	fig|6666666.59345.peg.1131	fig|6666666.59341.peg.2710	fig|6666666.59347.peg.5383	fig|6666666.59346.peg.4474	fig|6666666.59348.peg.82	None	None	fig|6666666.59351.peg.4908	fig|6666666.59353.peg.1372	fig|6666666.59355.peg.2501	fig|6666666.59354.peg.6500

2285	Transcription termination protein NusA	fig|6666666.59329.peg.3444	fig|6666666.59330.peg.107	None	None	fig|6666666.59334.peg.5468	fig|6666666.59335.peg.2103	fig|6666666.59336.peg.2420	fig|6666666.59333.peg.2327	fig|6666666.59338.peg.1505	fig|6666666.59339.peg.4892	None	fig|6666666.59337.peg.683	fig|6666666.59342.peg.377	fig|6666666.71181.peg.4097	fig|6666666.59344.peg.2100	fig|6666666.59345.peg.2357	fig|6666666.59341.peg.2438	fig|6666666.59347.peg.3005	fig|6666666.59346.peg.298	fig|6666666.59348.peg.967	fig|6666666.59349.peg.1432	fig|6666666.59350.peg.227	fig|6666666.59351.peg.1390	fig|6666666.59353.peg.3133	fig|6666666.59355.peg.463	fig|6666666.59354.peg.2273

10903	LSU m3Psi1915 methyltransferase RlmH	None	fig|6666666.59330.peg.2038	fig|6666666.59331.peg.5580	None	fig|6666666.59334.peg.2316	fig|6666666.59335.peg.1754	fig|6666666.59336.peg.579	fig|6666666.59333.peg.2135	fig|6666666.59338.peg.12	fig|6666666.59339.peg.5207	None	fig|6666666.59337.peg.1086	fig|6666666.59342.peg.908	fig|6666666.71181.peg.4762	fig|6666666.59344.peg.4492	fig|6666666.59345.peg.3493	fig|6666666.59341.peg.4749	fig|6666666.59347.peg.2904	fig|6666666.59346.peg.3298	fig|6666666.59348.peg.847	fig|6666666.59349.peg.2763	fig|6666666.59350.peg.7968	fig|6666666.59351.peg.3624	fig|6666666.59353.peg.2021	fig|6666666.59355.peg.5615	fig|6666666.59354.peg.2321

9126	3'-to-5' oligoribonuclease orn	fig|6666666.59329.peg.45	fig|6666666.59330.peg.4940	None	fig|6666666.59332.peg.756	fig|6666666.59334.peg.2761	fig|6666666.59335.peg.1958	fig|6666666.59336.peg.1829	fig|6666666.59333.peg.4201	fig|6666666.59338.peg.823	fig|6666666.59339.peg.8810	None	fig|6666666.59337.peg.5006	fig|6666666.59342.peg.5563	fig|6666666.71181.peg.1694	fig|6666666.59344.peg.597	fig|6666666.59345.peg.5085	fig|6666666.59341.peg.4608	fig|6666666.59347.peg.4565	fig|6666666.59346.peg.5238	fig|6666666.59348.peg.1654	fig|6666666.59349.peg.3227	None	fig|6666666.59351.peg.3891	fig|6666666.59353.peg.1064	fig|6666666.59355.peg.3553	fig|6666666.59354.peg.5761

9403	Tyrosine-specific transport protein	fig|6666666.59329.peg.3140	fig|6666666.59330.peg.2863	fig|6666666.59331.peg.3120	fig|6666666.59332.peg.1056	fig|6666666.59334.peg.2389	fig|6666666.59335.peg.5251	fig|6666666.59336.peg.6430	fig|6666666.59333.peg.961	None	fig|6666666.59339.peg.8487	None	fig|6666666.59337.peg.1950	fig|6666666.59342.peg.2669	fig|6666666.71181.peg.3174	fig|6666666.59344.peg.1677	fig|6666666.59345.peg.258	fig|6666666.59341.peg.6020	fig|6666666.59347.peg.824	fig|6666666.59346.peg.4011	fig|6666666.59348.peg.2953	fig|6666666.59349.peg.2774	None	fig|6666666.59351.peg.5420	fig|6666666.59353.peg.1096	fig|6666666.59355.peg.383	fig|6666666.59354.peg.4072

7536	FIG005121 SAM-dependent methyltransferase EC 2.1.1.-	fig|6666666.59329.peg.5809	fig|6666666.59330.peg.4726	None	fig|6666666.59332.peg.431	fig|6666666.59334.peg.3922	fig|6666666.59335.peg.5519	fig|6666666.59336.peg.2853	fig|6666666.59333.peg.1161	fig|6666666.59338.peg.3630	fig|6666666.59339.peg.5145	fig|6666666.59340.peg.637	fig|6666666.59337.peg.1607	None	fig|6666666.71181.peg.877	fig|6666666.59344.peg.4752	fig|6666666.59345.peg.2176	fig|6666666.59341.peg.2350	fig|6666666.59347.peg.2869	fig|6666666.59346.peg.3696	fig|6666666.59348.peg.644	fig|6666666.59349.peg.4584	None	fig|6666666.59351.peg.5492	fig|6666666.59353.peg.4556	fig|6666666.59355.peg.4641	fig|6666666.59354.peg.3311

2561	Flagellar assembly protein FliH	fig|6666666.59329.peg.6285	fig|6666666.59330.peg.2218	None	fig|6666666.59332.peg.5489	None	fig|6666666.59335.peg.2395	fig|6666666.59336.peg.2869	fig|6666666.59333.peg.2847	fig|6666666.59338.peg.2981	fig|6666666.59339.peg.7098	fig|6666666.59340.peg.1513	fig|6666666.59337.peg.2960	fig|6666666.59342.peg.4345	fig|6666666.71181.peg.4026	fig|6666666.59344.peg.6214	fig|6666666.59345.peg.5343	fig|6666666.59341.peg.3268	fig|6666666.59347.peg.4630	fig|6666666.59346.peg.2880	fig|6666666.59348.peg.4348	fig|6666666.59349.peg.210	None	fig|6666666.59351.peg.2048	fig|6666666.59353.peg.4561	fig|6666666.59355.peg.5159	fig|6666666.59354.peg.1073

5026	Zinc ABC transporter, inner membrane permease protein ZnuB	fig|6666666.59329.peg.152	fig|6666666.59330.peg.1533	fig|6666666.59331.peg.1418	fig|6666666.59332.peg.2609	fig|6666666.59334.peg.1847	fig|6666666.59335.peg.3595	fig|6666666.59336.peg.4534	fig|6666666.59333.peg.906	fig|6666666.59338.peg.5700	fig|6666666.59339.peg.2979	fig|6666666.59340.peg.1024	fig|6666666.59337.peg.4116	fig|6666666.59342.peg.4907	fig|6666666.71181.peg.6087	fig|6666666.59344.peg.2830	fig|6666666.59345.peg.3039	fig|6666666.59341.peg.2864	fig|6666666.59347.peg.3293	fig|6666666.59346.peg.2935	fig|6666666.59348.peg.1720	None	fig|6666666.59350.peg.3802	None	fig|6666666.59353.peg.1288	fig|6666666.59355.peg.980	None

1614	Uncharacterized conserved protein	fig|6666666.59329.peg.4291	None	fig|6666666.59331.peg.1705	fig|6666666.59332.peg.5023	fig|6666666.59334.peg.2437	fig|6666666.59335.peg.3854	fig|6666666.59336.peg.1530	fig|6666666.59333.peg.3469	None	fig|6666666.59339.peg.8350	fig|6666666.59340.peg.2568	fig|6666666.59337.peg.597	fig|6666666.59342.peg.3406	fig|6666666.71181.peg.2476	fig|6666666.59344.peg.5407	fig|6666666.59345.peg.1635	fig|6666666.59341.peg.1529	fig|6666666.59347.peg.748	fig|6666666.59346.peg.746	fig|6666666.59348.peg.3927	fig|6666666.59349.peg.3	None	fig|6666666.59351.peg.3420	fig|6666666.59353.peg.3603	fig|6666666.59355.peg.4354	fig|6666666.59354.peg.4692

302	FIG00809136 hypothetical protein	fig|6666666.59329.peg.5449	fig|6666666.59330.peg.3703	fig|6666666.59331.peg.3129	fig|6666666.59332.peg.5942	fig|6666666.59334.peg.5364	fig|6666666.59335.peg.4277	None	None	fig|6666666.59338.peg.2499	fig|6666666.59339.peg.2850	None	fig|6666666.59337.peg.3042	fig|6666666.59342.peg.3692	fig|6666666.71181.peg.3621	fig|6666666.59344.peg.3394	fig|6666666.59345.peg.96	fig|6666666.59341.peg.1063	fig|6666666.59347.peg.985	fig|6666666.59346.peg.3637	fig|6666666.59348.peg.3027	fig|6666666.59349.peg.4679	fig|6666666.59350.peg.1671	fig|6666666.59351.peg.1300	fig|6666666.59353.peg.3766	fig|6666666.59355.peg.3783	fig|6666666.59354.peg.4246

11186	Glycine cleavage system transcriptional antiactivator GcvR	fig|6666666.59329.peg.6168	fig|6666666.59330.peg.3640	None	fig|6666666.59332.peg.4173	fig|6666666.59334.peg.1857	fig|6666666.59335.peg.1197	fig|6666666.59336.peg.1616	fig|6666666.59333.peg.4789	fig|6666666.59338.peg.784	fig|6666666.59339.peg.39	None	fig|6666666.59337.peg.5611	fig|6666666.59342.peg.3256	fig|6666666.71181.peg.3253	fig|6666666.59344.peg.4007	fig|6666666.59345.peg.2155	fig|6666666.59341.peg.1675	fig|6666666.59347.peg.2850	fig|6666666.59346.peg.2506	fig|6666666.59348.peg.78	fig|6666666.59349.peg.2944	None	fig|6666666.59351.peg.607	fig|6666666.59353.peg.4043	fig|6666666.59355.peg.4000	fig|6666666.59354.peg.4934

1334	Glyoxylase family protein	fig|6666666.59329.peg.1549	fig|6666666.59330.peg.2205	fig|6666666.59331.peg.3050	fig|6666666.59332.peg.4450	fig|6666666.59334.peg.3541	fig|6666666.59335.peg.4709	fig|6666666.59336.peg.2540	fig|6666666.59333.peg.4379	fig|6666666.59338.peg.4567	fig|6666666.59339.peg.4393	None	fig|6666666.59337.peg.1898	None	fig|6666666.71181.peg.16	fig|6666666.59344.peg.2673	fig|6666666.59345.peg.2836	fig|6666666.59341.peg.5413	fig|6666666.59347.peg.1138	None	fig|6666666.59348.peg.2202	fig|6666666.59349.peg.2148	fig|6666666.59350.peg.1141	fig|6666666.59351.peg.3053	fig|6666666.59353.peg.2003	fig|6666666.59355.peg.4108	fig|6666666.59354.peg.1875

5230	Membrane protein, distant similarity to thiosulphate quinone oxidoreductase DoxD	fig|6666666.59329.peg.3315	fig|6666666.59330.peg.1482	fig|6666666.59331.peg.5437	fig|6666666.59332.peg.4942	fig|6666666.59334.peg.3784	fig|6666666.59335.peg.4872	fig|6666666.59336.peg.3775	fig|6666666.59333.peg.1226	fig|6666666.59338.peg.4280	fig|6666666.59339.peg.4607	None	fig|6666666.59337.peg.195	fig|6666666.59342.peg.2496	fig|6666666.71181.peg.4401	fig|6666666.59344.peg.1852	fig|6666666.59345.peg.4523	fig|6666666.59341.peg.2309	fig|6666666.59347.peg.4714	fig|6666666.59346.peg.4017	None	fig|6666666.59349.peg.391	None	fig|6666666.59351.peg.927	fig|6666666.59353.peg.2381	fig|6666666.59355.peg.1653	fig|6666666.59354.peg.5825

5548	Non-specific DNA-binding protein Dps Iron-binding ferritin-like antioxidant protein Ferroxidase EC 1.16.3.1	fig|6666666.59329.peg.1147	fig|6666666.59330.peg.928	fig|6666666.59331.peg.1935	fig|6666666.59332.peg.1405	fig|6666666.59334.peg.1162	fig|6666666.59335.peg.4738	fig|6666666.59336.peg.4014	fig|6666666.59333.peg.2164	fig|6666666.59338.peg.2514	fig|6666666.59339.peg.3761	None	fig|6666666.59337.peg.3954	fig|6666666.59342.peg.839	fig|6666666.71181.peg.2775	fig|6666666.59344.peg.436	fig|6666666.59345.peg.440	fig|6666666.59341.peg.3331	fig|6666666.59347.peg.3696	fig|6666666.59346.peg.4596	None	fig|6666666.59349.peg.1727	None	fig|6666666.59351.peg.2379	fig|6666666.59353.peg.1757	fig|6666666.59355.peg.1254	fig|6666666.59354.peg.5311

8590	Putative formate dehydrogenase-specific chaperone	fig|6666666.59329.peg.1966	fig|6666666.59330.peg.4156	fig|6666666.59331.peg.3929	fig|6666666.59332.peg.3601	fig|6666666.59334.peg.597	fig|6666666.59335.peg.2191	fig|6666666.59336.peg.3297	fig|6666666.59333.peg.4422	fig|6666666.59338.peg.2422	fig|6666666.59339.peg.1282	fig|6666666.59340.peg.986	fig|6666666.59337.peg.2647	fig|6666666.59342.peg.210	fig|6666666.71181.peg.868	fig|6666666.59344.peg.5	fig|6666666.59345.peg.709	fig|6666666.59341.peg.1016	fig|6666666.59347.peg.1718	fig|6666666.59346.peg.2023	fig|6666666.59348.peg.5316	None	fig|6666666.59350.peg.7643	None	fig|6666666.59353.peg.346	fig|6666666.59355.peg.1384	None

4663	Pyrroline-5-carboxylate reductase EC 1.5.1.2	fig|6666666.59329.peg.6335	fig|6666666.59330.peg.341	None	fig|6666666.59332.peg.1629	fig|6666666.59334.peg.3006	fig|6666666.59335.peg.3087	fig|6666666.59336.peg.3068	fig|6666666.59333.peg.766	None	fig|6666666.59339.peg.7825	None	fig|6666666.59337.peg.522	fig|6666666.59342.peg.2686	fig|6666666.71181.peg.1866	fig|6666666.59344.peg.4341	fig|6666666.59345.peg.5012	fig|6666666.59341.peg.3055	fig|6666666.59347.peg.3866	fig|6666666.59346.peg.1779	fig|6666666.59348.peg.225	fig|6666666.59349.peg.799	fig|6666666.59350.peg.2575	fig|6666666.59351.peg.3529	fig|6666666.59353.peg.3232	fig|6666666.59355.peg.3114	fig|6666666.59354.peg.527

324	tRNA guanine46-N7- -methyltransferase EC 2.1.1.33	fig|6666666.59329.peg.1535	fig|6666666.59330.peg.333	None	fig|6666666.59332.peg.1637	fig|6666666.59334.peg.1376	fig|6666666.59335.peg.4695	fig|6666666.59336.peg.5908	fig|6666666.59333.peg.756	fig|6666666.59338.peg.2886	fig|6666666.59339.peg.7810	None	fig|6666666.59337.peg.513	None	fig|6666666.71181.peg.1875	fig|6666666.59344.peg.4352	fig|6666666.59345.peg.5002	fig|6666666.59341.peg.3064	fig|6666666.59347.peg.3875	fig|6666666.59346.peg.4580	fig|6666666.59348.peg.232	fig|6666666.59349.peg.3655	fig|6666666.59350.peg.576	fig|6666666.59351.peg.297	fig|6666666.59353.peg.3241	fig|6666666.59355.peg.3124	fig|6666666.59354.peg.3162

9209	Uncharacterized ABC transporter, auxiliary component YrbC	None	fig|6666666.59330.peg.54	fig|6666666.59331.peg.5801	fig|6666666.59332.peg.1277	fig|6666666.59334.peg.87	fig|6666666.59335.peg.217	fig|6666666.59336.peg.358	fig|6666666.59333.peg.273	fig|6666666.59338.peg.117	fig|6666666.59339.peg.8190	None	fig|6666666.59337.peg.841	fig|6666666.59342.peg.441	fig|6666666.71181.peg.1027	fig|6666666.59344.peg.1807	fig|6666666.59345.peg.4852	fig|6666666.59341.peg.6089	fig|6666666.59347.peg.3242	fig|6666666.59346.peg.630	fig|6666666.59348.peg.3523	fig|6666666.59349.peg.30	None	fig|6666666.59351.peg.3451	fig|6666666.59353.peg.2346	fig|6666666.59355.peg.1619	fig|6666666.59354.peg.346

8367	Zinc ABC transporter, periplasmic-binding protein ZnuA	fig|6666666.59329.peg.1652	fig|6666666.59330.peg.1530	fig|6666666.59331.peg.2203	fig|6666666.59332.peg.701	fig|6666666.59334.peg.1844	fig|6666666.59335.peg.3598	fig|6666666.59336.peg.1392	fig|6666666.59333.peg.909	fig|6666666.59338.peg.1961	fig|6666666.59339.peg.2981	fig|6666666.59340.peg.1630	fig|6666666.59337.peg.4118	fig|6666666.59342.peg.1755	fig|6666666.71181.peg.4321	fig|6666666.59344.peg.2832	fig|6666666.59345.peg.3922	fig|6666666.59341.peg.1571	fig|6666666.59347.peg.1120	fig|6666666.59346.peg.3786	fig|6666666.59348.peg.4702	None	fig|6666666.59350.peg.6608	None	fig|6666666.59353.peg.1290	fig|6666666.59355.peg.981	None

4019	Maltose maltodextrin ABC transporter, permease protein MalG	fig|6666666.59329.peg.2190	fig|6666666.59330.peg.3242	fig|6666666.59331.peg.1879	fig|6666666.59332.peg.3662	fig|6666666.59334.peg.3929	fig|6666666.59335.peg.5922	fig|6666666.59336.peg.1563	fig|6666666.59333.peg.1000	fig|6666666.59338.peg.43	fig|6666666.59339.peg.4307	fig|6666666.59340.peg.3008	fig|6666666.59337.peg.171	fig|6666666.59342.peg.5776	fig|6666666.71181.peg.3599	fig|6666666.59344.peg.2725	fig|6666666.59345.peg.620	fig|6666666.59341.peg.4494	fig|6666666.59347.peg.3527	fig|6666666.59346.peg.1172	fig|6666666.59348.peg.2346	None	fig|6666666.59350.peg.277	None	fig|6666666.59353.peg.3784	fig|6666666.59355.peg.3232	None

7871	D-glycero-D-manno-heptose 1,7-bisphosphate phosphatase EC 3.1.1.-	fig|6666666.59329.peg.3212	fig|6666666.59330.peg.2020	fig|6666666.59331.peg.3882	fig|6666666.59332.peg.404	fig|6666666.59334.peg.309	fig|6666666.59335.peg.4420	fig|6666666.59336.peg.2329	fig|6666666.59333.peg.4061	None	fig|6666666.59339.peg.5171	None	fig|6666666.59337.peg.1105	fig|6666666.59342.peg.5510	fig|6666666.71181.peg.1	fig|6666666.59344.peg.2689	fig|6666666.59345.peg.3510	fig|6666666.59341.peg.2852	fig|6666666.59347.peg.2920	fig|6666666.59346.peg.4068	fig|6666666.59348.peg.705	fig|6666666.59349.peg.2786	None	fig|6666666.59351.peg.5205	fig|6666666.59353.peg.2018	fig|6666666.59355.peg.4124	fig|6666666.59354.peg.2869

566	Hypothetical nudix hydrolase YeaB	fig|6666666.59329.peg.5818	fig|6666666.59330.peg.2077	fig|6666666.59331.peg.4647	fig|6666666.59332.peg.4604	fig|6666666.59334.peg.2518	fig|6666666.59335.peg.5690	None	fig|6666666.59333.peg.3213	None	fig|6666666.59339.peg.3708	None	fig|6666666.59337.peg.4675	fig|6666666.59342.peg.733	fig|6666666.71181.peg.4232	fig|6666666.59344.peg.2560	fig|6666666.59345.peg.829	fig|6666666.59341.peg.2097	fig|6666666.59347.peg.2155	fig|6666666.59346.peg.4500	fig|6666666.59348.peg.1441	fig|6666666.59349.peg.2938	fig|6666666.59350.peg.4438	fig|6666666.59351.peg.2734	fig|6666666.59353.peg.1525	fig|6666666.59355.peg.2074	fig|6666666.59354.peg.5401

5420	Queuosine Biosynthesis QueC ATPase	fig|6666666.59329.peg.5162	fig|6666666.59330.peg.3937	None	fig|6666666.59332.peg.3323	fig|6666666.59334.peg.457	fig|6666666.59335.peg.5077	fig|6666666.59336.peg.4108	fig|6666666.59333.peg.3166	fig|6666666.59338.peg.5630	fig|6666666.59339.peg.4041	None	fig|6666666.59337.peg.4952	fig|6666666.59342.peg.1400	fig|6666666.71181.peg.5495	fig|6666666.59344.peg.1060	fig|6666666.59345.peg.4087	fig|6666666.59341.peg.4723	fig|6666666.59347.peg.1801	fig|6666666.59346.peg.4333	None	fig|6666666.59349.peg.3448	fig|6666666.59350.peg.1069	fig|6666666.59351.peg.3970	fig|6666666.59353.peg.746	fig|6666666.59355.peg.958	fig|6666666.59354.peg.2198

4241	Diacylglycerol kinase EC 2.7.1.107	fig|6666666.59329.peg.558	fig|6666666.59330.peg.876	None	fig|6666666.59332.peg.286	None	fig|6666666.59335.peg.4044	fig|6666666.59336.peg.64	fig|6666666.59333.peg.644	fig|6666666.59338.peg.2582	fig|6666666.59339.peg.8582	fig|6666666.59340.peg.2385	fig|6666666.59337.peg.3295	fig|6666666.59342.peg.2989	fig|6666666.71181.peg.2002	fig|6666666.59344.peg.2739	fig|6666666.59345.peg.2611	fig|6666666.59341.peg.2429	fig|6666666.59347.peg.2467	fig|6666666.59346.peg.1704	fig|6666666.59348.peg.4252	fig|6666666.59349.peg.1686	fig|6666666.59350.peg.8099	None	fig|6666666.59353.peg.3000	fig|6666666.59355.peg.2906	fig|6666666.59354.peg.4596

6544	Molybdopterin-guanine dinucleotide biosynthesis protein MobB Molybdopterin biosynthesis protein MoeA	fig|6666666.59329.peg.2463	fig|6666666.59330.peg.1139	fig|6666666.59331.peg.6175	fig|6666666.59332.peg.4971	fig|6666666.59334.peg.2014	fig|6666666.59335.peg.5614	fig|6666666.59336.peg.4575	fig|6666666.59333.peg.4699	fig|6666666.59338.peg.3884	fig|6666666.59339.peg.1208	fig|6666666.59340.peg.2628	fig|6666666.59337.peg.3637	fig|6666666.59342.peg.3664	fig|6666666.71181.peg.177	fig|6666666.59344.peg.886	fig|6666666.59345.peg.3192	fig|6666666.59341.peg.4010	fig|6666666.59347.peg.693	fig|6666666.59346.peg.3682	fig|6666666.59348.peg.2826	None	fig|6666666.59350.peg.7111	None	fig|6666666.59353.peg.645	fig|6666666.59355.peg.127	None

2263	L-proline glycine betaine ABC transport system permease protein ProW TC 3.A.1.12.1	fig|6666666.59329.peg.3988	fig|6666666.59330.peg.2017	fig|6666666.59331.peg.5309	fig|6666666.59332.peg.401	fig|6666666.59334.peg.905	fig|6666666.59335.peg.5793	fig|6666666.59336.peg.5790	fig|6666666.59333.peg.814	fig|6666666.59338.peg.873	fig|6666666.59339.peg.2602	fig|6666666.59340.peg.3394	fig|6666666.59337.peg.5263	fig|6666666.59342.peg.4946	fig|6666666.71181.peg.5177	fig|6666666.59344.peg.3904	fig|6666666.59345.peg.4757	fig|6666666.59341.peg.3559	fig|6666666.59347.peg.2078	fig|6666666.59346.peg.483	fig|6666666.59348.peg.2071	None	fig|6666666.59350.peg.6194	None	fig|6666666.59353.peg.2816	fig|6666666.59355.peg.4285	None

2042	Outer membrane protein YfgL, lipoprotein component of the protein assembly complex forms a complex with YaeT, YfiO, and NlpB	fig|6666666.59329.peg.303	fig|6666666.59330.peg.1429	None	fig|6666666.59332.peg.5279	fig|6666666.59334.peg.1257	fig|6666666.59335.peg.5521	fig|6666666.59336.peg.1628	fig|6666666.59333.peg.939	fig|6666666.59338.peg.2289	fig|6666666.59339.peg.6973	None	fig|6666666.59337.peg.755	None	fig|6666666.71181.peg.1773	fig|6666666.59344.peg.5137	fig|6666666.59345.peg.3638	fig|6666666.59341.peg.3033	fig|6666666.59347.peg.4299	fig|6666666.59346.peg.1315	fig|6666666.59348.peg.3132	fig|6666666.59349.peg.3085	fig|6666666.59350.peg.5123	fig|6666666.59351.peg.3078	fig|6666666.59353.peg.2712	fig|6666666.59355.peg.1768	fig|6666666.59354.peg.88

7070	DNA uptake protein	fig|6666666.59329.peg.5403	fig|6666666.59330.peg.302	None	fig|6666666.59332.peg.2214	fig|6666666.59334.peg.1421	fig|6666666.59335.peg.3367	fig|6666666.59336.peg.3733	fig|6666666.59333.peg.4901	fig|6666666.59338.peg.1593	fig|6666666.59339.peg.3107	None	fig|6666666.59337.peg.1031	fig|6666666.59342.peg.1928	fig|6666666.71181.peg.2057	fig|6666666.59344.peg.1062	fig|6666666.59345.peg.4252	fig|6666666.59341.peg.3786	fig|6666666.59347.peg.2653	fig|6666666.59346.peg.2667	fig|6666666.59348.peg.3250	fig|6666666.59349.peg.2718	None	fig|6666666.59351.peg.555	fig|6666666.59353.peg.748	fig|6666666.59355.peg.956	fig|6666666.59354.peg.111

4039	Hypoxanthine-guanine phosphoribosyltransferase EC 2.4.2.8	fig|6666666.59329.peg.6353	fig|6666666.59330.peg.4047	fig|6666666.59331.peg.6119	fig|6666666.59332.peg.2625	fig|6666666.59334.peg.461	fig|6666666.59335.peg.2337	fig|6666666.59336.peg.4634	fig|6666666.59333.peg.3418	fig|6666666.59338.peg.280	fig|6666666.59339.peg.7976	fig|6666666.59340.peg.3332	fig|6666666.59337.peg.2062	fig|6666666.59342.peg.453	fig|6666666.71181.peg.2851	fig|6666666.59344.peg.4426	fig|6666666.59345.peg.2415	fig|6666666.59341.peg.1651	fig|6666666.59347.peg.3902	fig|6666666.59346.peg.2253	fig|6666666.59348.peg.5331	None	fig|6666666.59350.peg.3453	None	fig|6666666.59353.peg.886	fig|6666666.59355.peg.3084	None

1601	PlcB, ORFX, ORFP, ORFB, ORFA, ldh gene	fig|6666666.59329.peg.6346	fig|6666666.59330.peg.522	fig|6666666.59331.peg.4464	fig|6666666.59332.peg.4984	fig|6666666.59334.peg.2919	fig|6666666.59335.peg.3074	fig|6666666.59336.peg.5088	fig|6666666.59333.peg.525	fig|6666666.59338.peg.5312	fig|6666666.59339.peg.5743	fig|6666666.59340.peg.688	fig|6666666.59337.peg.5817	fig|6666666.59342.peg.2195	fig|6666666.71181.peg.3911	fig|6666666.59344.peg.375	fig|6666666.59345.peg.4642	fig|6666666.59341.peg.2994	fig|6666666.59347.peg.1220	fig|6666666.59346.peg.5169	fig|6666666.59348.peg.1416	None	fig|6666666.59350.peg.3487	None	fig|6666666.59353.peg.517	fig|6666666.59355.peg.2	None

3233	3-deoxy-D-manno-octulosonic-acid transferase EC 2.-.-.-	None	fig|6666666.59330.peg.1358	None	fig|6666666.59332.peg.1733	fig|6666666.59334.peg.177	fig|6666666.59335.peg.492	fig|6666666.59336.peg.74	fig|6666666.59333.peg.469	fig|6666666.59338.peg.1906	fig|6666666.59339.peg.6306	fig|6666666.59340.peg.267	fig|6666666.59337.peg.449	fig|6666666.59342.peg.3741	fig|6666666.71181.peg.3659	fig|6666666.59344.peg.947	fig|6666666.59345.peg.2596	fig|6666666.59341.peg.4932	fig|6666666.59347.peg.5182	None	fig|6666666.59348.peg.1751	fig|6666666.59349.peg.2792	fig|6666666.59350.peg.1921	fig|6666666.59351.peg.5199	fig|6666666.59353.peg.2587	fig|6666666.59355.peg.2614	fig|6666666.59354.peg.5498

540	Lipid-A-disaccharide synthase EC 2.4.1.182	fig|6666666.59329.peg.4481	fig|6666666.59330.peg.455	None	fig|6666666.59332.peg.417	fig|6666666.59334.peg.1792	fig|6666666.59335.peg.2665	fig|6666666.59336.peg.2998	fig|6666666.59333.peg.4460	fig|6666666.59338.peg.2458	fig|6666666.59339.peg.5126	None	fig|6666666.59337.peg.1595	None	fig|6666666.71181.peg.22	fig|6666666.59344.peg.2667	fig|6666666.59345.peg.2188	fig|6666666.59341.peg.981	fig|6666666.59347.peg.4371	fig|6666666.59346.peg.4442	fig|6666666.59348.peg.3429	fig|6666666.59349.peg.1362	fig|6666666.59350.peg.1093	fig|6666666.59351.peg.2128	fig|6666666.59353.peg.1998	fig|6666666.59355.peg.4103	fig|6666666.59354.peg.1409

10986	Anhydro-N-acetylmuramic acid kinase EC 2.7.1.-	fig|6666666.59329.peg.5653	fig|6666666.59330.peg.2522	fig|6666666.59331.peg.2741	fig|6666666.59332.peg.1090	fig|6666666.59334.peg.1018	fig|6666666.59335.peg.999	fig|6666666.59336.peg.854	None	fig|6666666.59338.peg.584	fig|6666666.59339.peg.7478	None	fig|6666666.59337.peg.143	fig|6666666.59342.peg.522	fig|6666666.71181.peg.2930	fig|6666666.59344.peg.3492	fig|6666666.59345.peg.3699	fig|6666666.59341.peg.100	fig|6666666.59347.peg.4041	fig|6666666.59346.peg.248	None	fig|6666666.59349.peg.3432	fig|6666666.59350.peg.2970	fig|6666666.59351.peg.1671	fig|6666666.59353.peg.1389	fig|6666666.59355.peg.2483	fig|6666666.59354.peg.952

4529	[Protein-PII] uridylyltransferase EC 2.7.7.59	fig|6666666.59329.peg.4767	fig|6666666.59330.peg.471	fig|6666666.59331.peg.2853	fig|6666666.59332.peg.986	fig|6666666.59334.peg.1038	fig|6666666.59335.peg.159	fig|6666666.59336.peg.6282	fig|6666666.59333.peg.2238	None	fig|6666666.59339.peg.5104	None	fig|6666666.59337.peg.1579	None	fig|6666666.71181.peg.37	fig|6666666.59344.peg.2650	fig|6666666.59345.peg.2204	fig|6666666.59341.peg.1470	fig|6666666.59347.peg.4356	fig|6666666.59346.peg.2257	fig|6666666.59348.peg.931	fig|6666666.59349.peg.1394	fig|6666666.59350.peg.2809	fig|6666666.59351.peg.2111	fig|6666666.59353.peg.1981	fig|6666666.59355.peg.5296	fig|6666666.59354.peg.3035

7331	YihE protein, a ser thr kinase implicated in LPS synthesis and Cpx signalling	fig|6666666.59329.peg.51	fig|6666666.59330.peg.631	None	fig|6666666.59332.peg.215	fig|6666666.59334.peg.1395	fig|6666666.59335.peg.2444	fig|6666666.59336.peg.2738	fig|6666666.59333.peg.5	fig|6666666.59338.peg.574	fig|6666666.59339.peg.8076	None	fig|6666666.59337.peg.3890	fig|6666666.59342.peg.5868	fig|6666666.71181.peg.2580	fig|6666666.59344.peg.889	fig|6666666.59345.peg.4938	fig|6666666.59341.peg.1942	fig|6666666.59347.peg.4757	fig|6666666.59346.peg.4410	fig|6666666.59348.peg.61	fig|6666666.59349.peg.4295	None	fig|6666666.59351.peg.4332	fig|6666666.59353.peg.2637	fig|6666666.59355.peg.2568	fig|6666666.59354.peg.3628

851	Nucleoid-associated protein NdpA	fig|6666666.59329.peg.3797	fig|6666666.59330.peg.1197	fig|6666666.59331.peg.2919	None	fig|6666666.59334.peg.3298	fig|6666666.59335.peg.1424	fig|6666666.59336.peg.5051	fig|6666666.59333.peg.2464	fig|6666666.59338.peg.6013	fig|6666666.59339.peg.219	None	fig|6666666.59337.peg.3145	fig|6666666.59342.peg.938	fig|6666666.71181.peg.490	fig|6666666.59344.peg.1702	fig|6666666.59345.peg.1147	fig|6666666.59341.peg.1125	fig|6666666.59347.peg.2574	fig|6666666.59346.peg.98	fig|6666666.59348.peg.2360	fig|6666666.59349.peg.2701	None	fig|6666666.59351.peg.5174	fig|6666666.59353.peg.1117	fig|6666666.59355.peg.359	fig|6666666.59354.peg.5686

11358	Lipoprotein YcfM, part of a salvage pathway of unknown substrate	fig|6666666.59329.peg.5648	fig|6666666.59330.peg.1239	None	fig|6666666.59332.peg.4925	fig|6666666.59334.peg.5215	fig|6666666.59335.peg.1310	fig|6666666.59336.peg.1620	fig|6666666.59333.peg.1871	fig|6666666.59338.peg.1569	fig|6666666.59339.peg.3445	None	fig|6666666.59337.peg.2182	fig|6666666.59342.peg.493	fig|6666666.71181.peg.1639	fig|6666666.59344.peg.5020	fig|6666666.59345.peg.1605	fig|6666666.59341.peg.269	fig|6666666.59347.peg.4070	fig|6666666.59346.peg.4967	fig|6666666.59348.peg.815	fig|6666666.59349.peg.2697	None	fig|6666666.59351.peg.5172	fig|6666666.59353.peg.332	fig|6666666.59355.peg.1371	fig|6666666.59354.peg.4453

11355	Oligosaccharide translocase SypK	fig|6666666.59329.peg.2534	fig|6666666.59330.peg.1342	fig|6666666.59331.peg.383	fig|6666666.59332.peg.3462	fig|6666666.59334.peg.2802	fig|6666666.59335.peg.2724	fig|6666666.59336.peg.3872	fig|6666666.59333.peg.4128	fig|6666666.59338.peg.2155	fig|6666666.59339.peg.6340	None	fig|6666666.59337.peg.4435	fig|6666666.59342.peg.181	fig|6666666.71181.peg.3052	fig|6666666.59344.peg.4658	fig|6666666.59345.peg.2569	fig|6666666.59341.peg.505	fig|6666666.59347.peg.2327	fig|6666666.59346.peg.1738	fig|6666666.59348.peg.8	fig|6666666.59349.peg.246	fig|6666666.59350.peg.1031	None	fig|6666666.59353.peg.1588	fig|6666666.59355.peg.4297	None

1806	Pyridoxine 5'-phosphate synthase EC 2.6.99.2	fig|6666666.59329.peg.2798	fig|6666666.59330.peg.389	fig|6666666.59331.peg.4034	fig|6666666.59332.peg.190	fig|6666666.59334.peg.321	fig|6666666.59335.peg.1494	fig|6666666.59336.peg.5324	None	fig|6666666.59338.peg.4042	fig|6666666.59339.peg.7374	fig|6666666.59340.peg.1438	fig|6666666.59337.peg.568	None	fig|6666666.71181.peg.1420	fig|6666666.59344.peg.1569	fig|6666666.59345.peg.5059	fig|6666666.59341.peg.1330	fig|6666666.59347.peg.3173	fig|6666666.59346.peg.338	fig|6666666.59348.peg.646	fig|6666666.59349.peg.1397	fig|6666666.59350.peg.1358	fig|6666666.59351.peg.605	fig|6666666.59353.peg.2656	fig|6666666.59355.peg.1826	None

6506	AsmA protein	fig|6666666.59329.peg.1602	fig|6666666.59330.peg.3714	fig|6666666.59331.peg.704	None	None	fig|6666666.59335.peg.3114	fig|6666666.59336.peg.3548	fig|6666666.59333.peg.1545	fig|6666666.59338.peg.5379	fig|6666666.59339.peg.166	fig|6666666.59340.peg.1401	fig|6666666.59337.peg.3850	None	fig|6666666.71181.peg.209	fig|6666666.59344.peg.257	fig|6666666.59345.peg.1078	fig|6666666.59341.peg.2392	fig|6666666.59347.peg.2617	fig|6666666.59346.peg.3933	fig|6666666.59348.peg.1457	fig|6666666.59349.peg.3271	fig|6666666.59350.peg.2235	fig|6666666.59351.peg.4208	fig|6666666.59353.peg.612	fig|6666666.59355.peg.94	fig|6666666.59354.peg.3785

2662	GMP synthase [glutamine-hydrolyzing], amidotransferase subunit EC 6.3.5.2 GMP synthase [glutamine-hydrolyzing], ATP pyrophosphatase subunit EC 6.3.5.2	fig|6666666.59329.peg.295	fig|6666666.59330.peg.4371	None	fig|6666666.59332.peg.4932	fig|6666666.59334.peg.3487	fig|6666666.59335.peg.1964	None	fig|6666666.59333.peg.2153	fig|6666666.59338.peg.459	fig|6666666.59339.peg.6985	fig|6666666.59340.peg.1217	fig|6666666.59337.peg.763	fig|6666666.59342.peg.3012	fig|6666666.71181.peg.1785	fig|6666666.59344.peg.5155	fig|6666666.59345.peg.3630	fig|6666666.59341.peg.5042	fig|6666666.59347.peg.4306	fig|6666666.59346.peg.3213	None	fig|6666666.59349.peg.1681	fig|6666666.59350.peg.2784	fig|6666666.59351.peg.385	fig|6666666.59353.peg.2725	fig|6666666.59355.peg.1756	fig|6666666.59354.peg.3481

8218	FIG028593 membrane protein	fig|6666666.59329.peg.2487	fig|6666666.59330.peg.1605	fig|6666666.59331.peg.4222	fig|6666666.59332.peg.165	fig|6666666.59334.peg.962	fig|6666666.59335.peg.3560	fig|6666666.59336.peg.2171	fig|6666666.59333.peg.1388	fig|6666666.59338.peg.2359	fig|6666666.59339.peg.8678	fig|6666666.59340.peg.2571	fig|6666666.59337.peg.4898	fig|6666666.59342.peg.4402	fig|6666666.71181.peg.4007	fig|6666666.59344.peg.987	fig|6666666.59345.peg.2555	fig|6666666.59341.peg.3641	fig|6666666.59347.peg.4531	fig|6666666.59346.peg.797	fig|6666666.59348.peg.4754	None	None	fig|6666666.59351.peg.4416	fig|6666666.59353.peg.2551	fig|6666666.59355.peg.2650	None

10037	Lipoprotein	fig|6666666.59329.peg.4698	fig|6666666.59330.peg.4678	fig|6666666.59331.peg.2836	fig|6666666.59332.peg.5465	fig|6666666.59334.peg.2355	fig|6666666.59335.peg.1255	fig|6666666.59336.peg.5502	None	fig|6666666.59338.peg.2232	fig|6666666.59339.peg.3038	fig|6666666.59340.peg.1115	fig|6666666.59337.peg.4228	fig|6666666.59342.peg.5728	fig|6666666.71181.peg.1522	fig|6666666.59344.peg.4987	fig|6666666.59345.peg.5306	None	fig|6666666.59347.peg.5099	fig|6666666.59346.peg.2159	None	fig|6666666.59349.peg.174	fig|6666666.59350.peg.42	fig|6666666.59351.peg.2093	fig|6666666.59353.peg.4874	fig|6666666.59355.peg.220	fig|6666666.59354.peg.1182

10800	Rossmann fold nucleotide-binding protein Smf possibly involved in DNA uptake	fig|6666666.59329.peg.69	fig|6666666.59330.peg.648	None	fig|6666666.59332.peg.231	fig|6666666.59334.peg.1510	fig|6666666.59335.peg.2431	fig|6666666.59336.peg.1989	fig|6666666.59333.peg.1370	fig|6666666.59338.peg.2931	fig|6666666.59339.peg.8096	fig|6666666.59340.peg.2279	fig|6666666.59337.peg.3904	None	fig|6666666.71181.peg.3429	fig|6666666.59344.peg.4910	fig|6666666.59345.peg.4924	fig|6666666.59341.peg.3880	fig|6666666.59347.peg.4745	fig|6666666.59346.peg.1827	None	fig|6666666.59349.peg.2820	fig|6666666.59350.peg.3317	fig|6666666.59351.peg.5059	fig|6666666.59353.peg.4623	fig|6666666.59355.peg.4709	fig|6666666.59354.peg.1427

2387	tRNA cytosine34-2'-O- -methyltransferase EC 2.1.1.-	fig|6666666.59329.peg.2022	fig|6666666.59330.peg.173	None	fig|6666666.59332.peg.155	fig|6666666.59334.peg.1155	fig|6666666.59335.peg.2477	fig|6666666.59336.peg.2315	fig|6666666.59333.peg.413	fig|6666666.59338.peg.1111	fig|6666666.59339.peg.8705	fig|6666666.59340.peg.7	fig|6666666.59337.peg.4343	None	fig|6666666.71181.peg.4017	fig|6666666.59344.peg.977	fig|6666666.59345.peg.5125	fig|6666666.59341.peg.205	fig|6666666.59347.peg.4522	fig|6666666.59346.peg.788	fig|6666666.59348.peg.4013	fig|6666666.59349.peg.2585	None	fig|6666666.59351.peg.265	fig|6666666.59353.peg.2561	fig|6666666.59355.peg.2640	fig|6666666.59354.peg.4006

1841	Putative preQ0 transporter	fig|6666666.59329.peg.3218	fig|6666666.59330.peg.999	fig|6666666.59331.peg.4565	fig|6666666.59332.peg.273	fig|6666666.59334.peg.414	fig|6666666.59335.peg.4458	fig|6666666.59336.peg.3812	fig|6666666.59333.peg.3885	fig|6666666.59338.peg.3005	fig|6666666.59339.peg.2145	fig|6666666.59340.peg.465	fig|6666666.59337.peg.2530	fig|6666666.59342.peg.5515	fig|6666666.71181.peg.4498	fig|6666666.59344.peg.5719	fig|6666666.59345.peg.5523	fig|6666666.59341.peg.4156	fig|6666666.59347.peg.5237	fig|6666666.59346.peg.4607	fig|6666666.59348.peg.2235	None	fig|6666666.59350.peg.628	None	fig|6666666.59353.peg.4941	fig|6666666.59355.peg.3352	None

2129	Histidyl-tRNA synthetase EC 6.1.1.21	fig|6666666.59329.peg.305	fig|6666666.59330.peg.1426	fig|6666666.59331.peg.1636	None	fig|6666666.59334.peg.1255	fig|6666666.59335.peg.1109	fig|6666666.59336.peg.682	fig|6666666.59333.peg.937	fig|6666666.59338.peg.2292	fig|6666666.59339.peg.6971	None	fig|6666666.59337.peg.753	fig|6666666.59342.peg.1062	fig|6666666.71181.peg.1771	fig|6666666.59344.peg.5135	fig|6666666.59345.peg.3640	fig|6666666.59341.peg.3036	fig|6666666.59347.peg.4297	fig|6666666.59346.peg.1318	fig|6666666.59348.peg.1131	fig|6666666.59349.peg.3083	None	fig|6666666.59351.peg.3076	fig|6666666.59353.peg.2710	fig|6666666.59355.peg.1770	fig|6666666.59354.peg.86

4546	Ribonuclease P protein component EC 3.1.26.5	fig|6666666.59329.peg.2419	fig|6666666.59330.peg.1108	fig|6666666.59331.peg.1546	fig|6666666.59332.peg.89	fig|6666666.59334.peg.1674	fig|6666666.59335.peg.783	fig|6666666.59336.peg.17	fig|6666666.59333.peg.43	None	fig|6666666.59339.peg.8029	None	fig|6666666.59337.peg.881	fig|6666666.59342.peg.513	fig|6666666.71181.peg.621	fig|6666666.59344.peg.4167	fig|6666666.59345.peg.4974	fig|6666666.59341.peg.1047	fig|6666666.59347.peg.4792	fig|6666666.59346.peg.1189	fig|6666666.59348.peg.310	fig|6666666.59349.peg.1010	None	fig|6666666.59351.peg.5027	fig|6666666.59353.peg.3058	fig|6666666.59355.peg.2288	fig|6666666.59354.peg.1562

5363	Aminoacyl-histidine dipeptidase Peptidase D EC 3.4.13.3	fig|6666666.59329.peg.1021	fig|6666666.59330.peg.1071	None	fig|6666666.59332.peg.1765	fig|6666666.59334.peg.4015	fig|6666666.59335.peg.141	fig|6666666.59336.peg.2723	fig|6666666.59333.peg.2825	fig|6666666.59338.peg.278	fig|6666666.59339.peg.5076	fig|6666666.59340.peg.1321	fig|6666666.59337.peg.5648	fig|6666666.59342.peg.449	fig|6666666.71181.peg.926	fig|6666666.59344.peg.3527	fig|6666666.59345.peg.3550	fig|6666666.59341.peg.2306	fig|6666666.59347.peg.2787	fig|6666666.59346.peg.2251	None	fig|6666666.59349.peg.3686	None	fig|6666666.59351.peg.3784	fig|6666666.59353.peg.888	fig|6666666.59355.peg.816	fig|6666666.59354.peg.1911

3128	Iron-sulfur cluster assembly scaffold protein IscU	fig|6666666.59329.peg.1110	fig|6666666.59330.peg.1415	fig|6666666.59331.peg.1216	fig|6666666.59332.peg.1329	fig|6666666.59334.peg.3065	fig|6666666.59335.peg.2368	fig|6666666.59336.peg.669	fig|6666666.59333.peg.925	fig|6666666.59338.peg.1325	fig|6666666.59339.peg.6793	None	fig|6666666.59337.peg.740	None	fig|6666666.71181.peg.1758	fig|6666666.59344.peg.1628	fig|6666666.59345.peg.3651	fig|6666666.59341.peg.4637	fig|6666666.59347.peg.4284	fig|6666666.59346.peg.2739	fig|6666666.59348.peg.1378	fig|6666666.59349.peg.2434	None	fig|6666666.59351.peg.2712	fig|6666666.59353.peg.2695	fig|6666666.59355.peg.1784	fig|6666666.59354.peg.4569

676	Selenoprotein W-related protein	fig|6666666.59329.peg.4687	fig|6666666.59330.peg.2198	fig|6666666.59331.peg.3021	fig|6666666.59332.peg.1699	None	fig|6666666.59335.peg.2424	fig|6666666.59336.peg.2554	fig|6666666.59333.peg.2060	fig|6666666.59338.peg.5514	fig|6666666.59339.peg.5260	None	fig|6666666.59337.peg.3915	fig|6666666.59342.peg.4086	fig|6666666.71181.peg.3117	fig|6666666.59344.peg.5965	fig|6666666.59345.peg.4354	None	fig|6666666.59347.peg.2836	fig|6666666.59346.peg.3676	fig|6666666.59348.peg.1276	fig|6666666.59349.peg.3559	fig|6666666.59350.peg.864	fig|6666666.59351.peg.1278	fig|6666666.59353.peg.3355	fig|6666666.59355.peg.3440	fig|6666666.59354.peg.5631

10676	Cardiolipin synthetase EC 2.7.8.-	fig|6666666.59329.peg.2603	fig|6666666.59330.peg.1852	fig|6666666.59331.peg.4507	fig|6666666.59332.peg.5541	fig|6666666.59334.peg.4214	fig|6666666.59335.peg.5083	fig|6666666.59336.peg.2126	fig|6666666.59333.peg.1632	fig|6666666.59338.peg.5354	fig|6666666.59339.peg.535	fig|6666666.59340.peg.3421	fig|6666666.59337.peg.2871	fig|6666666.59342.peg.1818	fig|6666666.71181.peg.6053	fig|6666666.59344.peg.433	fig|6666666.59345.peg.1714	fig|6666666.59341.peg.4964	fig|6666666.59347.peg.1645	fig|6666666.59346.peg.1162	fig|6666666.59348.peg.3219	None	fig|6666666.59350.peg.2669	None	fig|6666666.59353.peg.1755	fig|6666666.59355.peg.1257	None

7256	CDP-diacylglycerol--glycerol-3-phosphate 3-phosphatidyltransferase EC 2.7.8.5	fig|6666666.59329.peg.961	fig|6666666.59330.peg.2941	None	fig|6666666.59332.peg.3929	fig|6666666.59334.peg.2914	fig|6666666.59335.peg.3767	fig|6666666.59336.peg.1776	fig|6666666.59333.peg.4268	fig|6666666.59338.peg.5902	fig|6666666.59339.peg.579	None	fig|6666666.59337.peg.1742	fig|6666666.59342.peg.2149	fig|6666666.71181.peg.355	fig|6666666.59344.peg.2357	fig|6666666.59345.peg.902	fig|6666666.59341.peg.379	fig|6666666.59347.peg.2292	fig|6666666.59346.peg.1620	fig|6666666.59348.peg.2801	fig|6666666.59349.peg.3456	fig|6666666.59350.peg.502	None	fig|6666666.59353.peg.249	fig|6666666.59355.peg.1289	fig|6666666.59354.peg.3386

8806	1-deoxy-D-xylulose 5-phosphate reductoisomerase EC 1.1.1.267	fig|6666666.59329.peg.1577	fig|6666666.59330.peg.462	None	fig|6666666.59332.peg.1584	None	fig|6666666.59335.peg.2657	fig|6666666.59336.peg.2562	fig|6666666.59333.peg.3054	fig|6666666.59338.peg.5994	fig|6666666.59339.peg.5114	None	fig|6666666.59337.peg.1587	fig|6666666.59342.peg.273	fig|6666666.71181.peg.31	fig|6666666.59344.peg.2657	fig|6666666.59345.peg.2195	fig|6666666.59341.peg.974	fig|6666666.59347.peg.4364	fig|6666666.59346.peg.2266	fig|6666666.59348.peg.3061	fig|6666666.59349.peg.1369	fig|6666666.59350.peg.1881	fig|6666666.59351.peg.2121	fig|6666666.59353.peg.1987	fig|6666666.59355.peg.4094	fig|6666666.59354.peg.1338

8801	Extracellular deoxyribonuclease Dns EC 3.1.21.-	fig|6666666.59329.peg.1775	fig|6666666.59330.peg.349	fig|6666666.59331.peg.3187	fig|6666666.59332.peg.1936	fig|6666666.59334.peg.5172	fig|6666666.59335.peg.3097	fig|6666666.59336.peg.3968	fig|6666666.59333.peg.776	fig|6666666.59338.peg.452	fig|6666666.59339.peg.7838	fig|6666666.59340.peg.208	fig|6666666.59337.peg.530	None	fig|6666666.71181.peg.1859	fig|6666666.59344.peg.4333	fig|6666666.59345.peg.5020	fig|6666666.59341.peg.2578	fig|6666666.59347.peg.3211	fig|6666666.59346.peg.3517	fig|6666666.59348.peg.358	fig|6666666.59349.peg.1178	None	fig|6666666.59351.peg.4902	fig|6666666.59353.peg.3224	fig|6666666.59355.peg.3106	None

3369	FIG021952 putative membrane protein	fig|6666666.59329.peg.307	fig|6666666.59330.peg.1424	fig|6666666.59331.peg.1632	fig|6666666.59332.peg.1339	fig|6666666.59334.peg.1252	fig|6666666.59335.peg.1112	fig|6666666.59336.peg.680	fig|6666666.59333.peg.935	None	fig|6666666.59339.peg.6968	fig|6666666.59340.peg.1714	fig|6666666.59337.peg.750	fig|6666666.59342.peg.1060	fig|6666666.71181.peg.1769	None	fig|6666666.59345.peg.3642	fig|6666666.59341.peg.3038	fig|6666666.59347.peg.4294	fig|6666666.59346.peg.1320	fig|6666666.59348.peg.1133	fig|6666666.59349.peg.3081	None	fig|6666666.59351.peg.3074	fig|6666666.59353.peg.2708	fig|6666666.59355.peg.1772	fig|6666666.59354.peg.83

3883	3-hydroxydecanoyl-[acyl-carrier-protein] dehydratase EC 4.2.1.60	fig|6666666.59329.peg.2245	fig|6666666.59330.peg.3169	None	fig|6666666.59332.peg.5212	fig|6666666.59334.peg.1329	fig|6666666.59335.peg.1221	None	fig|6666666.59333.peg.2794	fig|6666666.59338.peg.6069	fig|6666666.59339.peg.1509	None	fig|6666666.59337.peg.4559	fig|6666666.59342.peg.2344	fig|6666666.71181.peg.261	fig|6666666.59344.peg.1186	fig|6666666.59345.peg.3249	fig|6666666.59341.peg.914	fig|6666666.59347.peg.1762	fig|6666666.59346.peg.2058	fig|6666666.59348.peg.2352	fig|6666666.59349.peg.1588	fig|6666666.59350.peg.559	fig|6666666.59351.peg.1005	fig|6666666.59353.peg.1690	fig|6666666.59355.peg.1030	fig|6666666.59354.peg.2192

9091	Cell division protein FtsZ EC 3.4.24.-	fig|6666666.59329.peg.1363	fig|6666666.59330.peg.1282	None	None	fig|6666666.59334.peg.3097	fig|6666666.59335.peg.826	fig|6666666.59336.peg.955	fig|6666666.59333.peg.5472	fig|6666666.59338.peg.2684	fig|6666666.59339.peg.4979	None	fig|6666666.59337.peg.53	fig|6666666.59342.peg.2794	fig|6666666.71181.peg.1975	fig|6666666.59344.peg.1474	fig|6666666.59345.peg.3788	fig|6666666.59341.peg.6097	fig|6666666.59347.peg.4005	fig|6666666.59346.peg.1246	fig|6666666.59348.peg.259	fig|6666666.59349.peg.2990	fig|6666666.59350.peg.1046	fig|6666666.59351.peg.214	fig|6666666.59353.peg.143	fig|6666666.59355.peg.753	fig|6666666.59354.peg.4405

2404	TPR repeat containing exported protein; Putative periplasmic protein contains a protein prenylyltransferase domain	fig|6666666.59329.peg.1623	fig|6666666.59330.peg.3433	None	fig|6666666.59332.peg.5920	fig|6666666.59334.peg.2371	fig|6666666.59335.peg.2512	fig|6666666.59336.peg.5478	fig|6666666.59333.peg.1993	fig|6666666.59338.peg.5913	fig|6666666.59339.peg.402	None	fig|6666666.59337.peg.4377	fig|6666666.59342.peg.2251	fig|6666666.71181.peg.5009	fig|6666666.59344.peg.4569	fig|6666666.59345.peg.1503	fig|6666666.59341.peg.4880	fig|6666666.59347.peg.4211	fig|6666666.59346.peg.1439	fig|6666666.59348.peg.3008	fig|6666666.59349.peg.3859	None	fig|6666666.59351.peg.661	fig|6666666.59353.peg.2103	fig|6666666.59355.peg.2718	fig|6666666.59354.peg.1601

1299	LSU ribosomal protein L35p	fig|6666666.59329.peg.4066	fig|6666666.59330.peg.516	fig|6666666.59331.peg.111	fig|6666666.59332.peg.4771	fig|6666666.59334.peg.4313	fig|6666666.59335.peg.3306	fig|6666666.59336.peg.5690	fig|6666666.59333.peg.523	fig|6666666.59338.peg.5744	fig|6666666.59339.peg.2414	None	fig|6666666.59337.peg.3297	fig|6666666.59342.peg.2816	fig|6666666.71181.peg.2418	fig|6666666.59344.peg.805	fig|6666666.59345.peg.4636	fig|6666666.59341.peg.3121	fig|6666666.59347.peg.3275	fig|6666666.59346.peg.4577	None	fig|6666666.59349.peg.406	fig|6666666.59350.peg.4678	None	fig|6666666.59353.peg.1303	fig|6666666.59355.peg.3278	fig|6666666.59354.peg.2976

5492	Ribose ABC transport system, permease protein RbsC TC 3.A.1.2.1	fig|6666666.59329.peg.4641	fig|6666666.59330.peg.710	None	fig|6666666.59332.peg.2874	fig|6666666.59334.peg.638	fig|6666666.59335.peg.5366	fig|6666666.59336.peg.5742	fig|6666666.59333.peg.1251	fig|6666666.59338.peg.2265	fig|6666666.59339.peg.647	fig|6666666.59340.peg.3692	fig|6666666.59337.peg.4619	fig|6666666.59342.peg.4506	fig|6666666.71181.peg.580	fig|6666666.59344.peg.42	fig|6666666.59345.peg.4802	fig|6666666.59341.peg.300	fig|6666666.59347.peg.2062	fig|6666666.59346.peg.456	fig|6666666.59348.peg.3053	None	fig|6666666.59350.peg.4649	fig|6666666.59351.peg.2261	fig|6666666.59353.peg.379	fig|6666666.59355.peg.1417	None

271	Dihydrodipicolinate reductase EC 1.3.1.26	fig|6666666.59329.peg.6357	fig|6666666.59330.peg.1287	None	fig|6666666.59332.peg.4399	None	fig|6666666.59335.peg.821	fig|6666666.59336.peg.961	fig|6666666.59333.peg.4110	fig|6666666.59338.peg.3159	fig|6666666.59339.peg.4985	None	fig|6666666.59337.peg.59	None	fig|6666666.71181.peg.413	fig|6666666.59344.peg.1306	fig|6666666.59345.peg.3783	fig|6666666.59341.peg.3131	fig|6666666.59347.peg.4009	fig|6666666.59346.peg.1251	fig|6666666.59348.peg.252	fig|6666666.59349.peg.4248	fig|6666666.59350.peg.920	fig|6666666.59351.peg.3191	fig|6666666.59353.peg.85	fig|6666666.59355.peg.693	fig|6666666.59354.peg.5908

9256	FIG01201176 hypothetical protein	fig|6666666.59329.peg.6106	fig|6666666.59330.peg.4785	None	fig|6666666.59332.peg.3515	fig|6666666.59334.peg.1270	fig|6666666.59335.peg.2350	fig|6666666.59336.peg.5111	fig|6666666.59333.peg.3747	fig|6666666.59338.peg.4411	fig|6666666.59339.peg.2877	None	fig|6666666.59337.peg.2515	fig|6666666.59342.peg.557	fig|6666666.71181.peg.1600	fig|6666666.59344.peg.2410	fig|6666666.59345.peg.129	fig|6666666.59341.peg.4256	fig|6666666.59347.peg.868	fig|6666666.59346.peg.2904	None	fig|6666666.59349.peg.3190	fig|6666666.59350.peg.6765	None	fig|6666666.59353.peg.292	fig|6666666.59355.peg.1333	fig|6666666.59354.peg.1233

4739	Cytochrome oxidase biogenesis protein Cox11-CtaG, copper delivery to Cox1	fig|6666666.59329.peg.1241	fig|6666666.59330.peg.3220	fig|6666666.59331.peg.606	fig|6666666.59332.peg.3960	fig|6666666.59334.peg.995	fig|6666666.59335.peg.2871	fig|6666666.59336.peg.3604	fig|6666666.59333.peg.5067	fig|6666666.59338.peg.3613	fig|6666666.59339.peg.6638	fig|6666666.59340.peg.3370	fig|6666666.59337.peg.5084	fig|6666666.59342.peg.1725	None	None	fig|6666666.59345.peg.5615	fig|6666666.59341.peg.3234	fig|6666666.59347.peg.1551	fig|6666666.59346.peg.4306	fig|6666666.59348.peg.1403	fig|6666666.59349.peg.3507	fig|6666666.59350.peg.2026	fig|6666666.59351.peg.4387	None	None	fig|6666666.59354.peg.2068

8713	Proposed lipoate regulatory protein YbeD	fig|6666666.59329.peg.3586	fig|6666666.59330.peg.2032	None	fig|6666666.59332.peg.2690	fig|6666666.59334.peg.5471	fig|6666666.59335.peg.1760	fig|6666666.59336.peg.585	None	fig|6666666.59338.peg.5	fig|6666666.59339.peg.5200	fig|6666666.59340.peg.400	fig|6666666.59337.peg.1092	fig|6666666.59342.peg.918	fig|6666666.71181.peg.2383	fig|6666666.59344.peg.3336	fig|6666666.59345.peg.3499	fig|6666666.59341.peg.5015	fig|6666666.59347.peg.2909	fig|6666666.59346.peg.3291	None	fig|6666666.59349.peg.2770	None	fig|6666666.59351.peg.3619	fig|6666666.59353.peg.3718	fig|6666666.59355.peg.3733	fig|6666666.59354.peg.5640

9503	C2363	fig|6666666.59329.peg.449	fig|6666666.59330.peg.1044	None	fig|6666666.59332.peg.1972	None	fig|6666666.59335.peg.5509	fig|6666666.59336.peg.1875	fig|6666666.59333.peg.4314	fig|6666666.59338.peg.1654	fig|6666666.59339.peg.7164	None	fig|6666666.59337.peg.1123	fig|6666666.59342.peg.5987	fig|6666666.71181.peg.52	fig|6666666.59344.peg.2626	fig|6666666.59345.peg.3526	fig|6666666.59341.peg.4741	fig|6666666.59347.peg.2935	fig|6666666.59346.peg.1735	fig|6666666.59348.peg.5012	fig|6666666.59349.peg.2624	None	fig|6666666.59351.peg.4652	fig|6666666.59353.peg.1965	fig|6666666.59355.peg.1441	fig|6666666.59354.peg.5472

2516	Thymidine phosphorylase EC 2.4.2.4	fig|6666666.59329.peg.1045	fig|6666666.59330.peg.82	fig|6666666.59331.peg.2879	fig|6666666.59332.peg.4671	fig|6666666.59334.peg.494	fig|6666666.59335.peg.3641	fig|6666666.59336.peg.6268	fig|6666666.59333.peg.2570	fig|6666666.59338.peg.3050	fig|6666666.59339.peg.5019	None	fig|6666666.59337.peg.1305	fig|6666666.59342.peg.1787	fig|6666666.71181.peg.458	fig|6666666.59344.peg.1374	fig|6666666.59345.peg.2334	fig|6666666.59341.peg.4730	fig|6666666.59347.peg.2970	fig|6666666.59346.peg.2449	fig|6666666.59348.peg.3313	None	fig|6666666.59350.peg.5246	None	fig|6666666.59353.peg.38	fig|6666666.59355.peg.646	None

153	FIG004064 hypothetical protein	fig|6666666.59329.peg.1862	fig|6666666.59330.peg.2647	fig|6666666.59331.peg.2699	fig|6666666.59332.peg.4342	None	fig|6666666.59335.peg.3485	fig|6666666.59336.peg.604	fig|6666666.59333.peg.884	None	fig|6666666.59339.peg.7704	None	fig|6666666.59337.peg.1714	None	fig|6666666.71181.peg.5218	fig|6666666.59344.peg.2756	fig|6666666.59345.peg.5368	fig|6666666.59341.peg.2412	fig|6666666.59347.peg.5372	fig|6666666.59346.peg.2393	fig|6666666.59348.peg.3661	fig|6666666.59349.peg.3243	fig|6666666.59350.peg.1721	fig|6666666.59351.peg.5438	fig|6666666.59353.peg.2984	fig|6666666.59355.peg.2890	fig|6666666.59354.peg.5102

2721	Putative cytoplasmic protein ,probably associated with Glutathione-regulated potassium-efflux	fig|6666666.59329.peg.5568	fig|6666666.59330.peg.1461	fig|6666666.59331.peg.5868	fig|6666666.59332.peg.1068	None	fig|6666666.59335.peg.1864	fig|6666666.59336.peg.2190	fig|6666666.59333.peg.2362	fig|6666666.59338.peg.3089	fig|6666666.59339.peg.8248	None	fig|6666666.59337.peg.1813	None	fig|6666666.71181.peg.4305	fig|6666666.59344.peg.3123	fig|6666666.59345.peg.5561	fig|6666666.59341.peg.5672	fig|6666666.59347.peg.4969	fig|6666666.59346.peg.35	fig|6666666.59348.peg.292	fig|6666666.59349.peg.3371	None	fig|6666666.59351.peg.4177	fig|6666666.59353.peg.2266	fig|6666666.59355.peg.1959	fig|6666666.59354.peg.6086

5265	PTS system, fructose-specific IIB component EC 2.7.1.69 PTS system, fructose-specific IIC component EC 2.7.1.69	fig|6666666.59329.peg.1161	fig|6666666.59330.peg.3548	fig|6666666.59331.peg.1111	fig|6666666.59332.peg.5125	fig|6666666.59334.peg.4290	fig|6666666.59335.peg.3694	fig|6666666.59336.peg.2235	fig|6666666.59333.peg.3711	fig|6666666.59338.peg.3765	fig|6666666.59339.peg.1791	fig|6666666.59340.peg.2241	fig|6666666.59337.peg.3254	fig|6666666.59342.peg.1432	fig|6666666.71181.peg.3183	fig|6666666.59344.peg.1664	fig|6666666.59345.peg.2026	fig|6666666.59341.peg.4091	fig|6666666.59347.peg.398	fig|6666666.59346.peg.3955	fig|6666666.59348.peg.1172	None	None	None	fig|6666666.59353.peg.1085	fig|6666666.59355.peg.394	None

1706	ABC transporter involved in cytochrome c biogenesis, CcmB subunit	None	fig|6666666.59330.peg.4380	None	fig|6666666.59332.peg.2657	fig|6666666.59334.peg.2184	fig|6666666.59335.peg.1247	fig|6666666.59336.peg.5998	fig|6666666.59333.peg.2592	fig|6666666.59338.peg.3960	fig|6666666.59339.peg.3026	None	fig|6666666.59337.peg.4220	None	fig|6666666.71181.peg.1530	fig|6666666.59344.peg.4977	fig|6666666.59345.peg.5315	fig|6666666.59341.peg.3757	fig|6666666.59347.peg.5090	fig|6666666.59346.peg.5180	fig|6666666.59348.peg.3303	fig|6666666.59349.peg.182	fig|6666666.59350.peg.2483	fig|6666666.59351.peg.2082	fig|6666666.59353.peg.4866	fig|6666666.59355.peg.212	fig|6666666.59354.peg.5197

6756	FIG039061 hypothetical protein related to heme utilization	fig|6666666.59329.peg.3454	fig|6666666.59330.peg.4118	fig|6666666.59331.peg.3904	fig|6666666.59332.peg.6169	None	fig|6666666.59335.peg.3292	None	fig|6666666.59333.peg.2536	None	fig|6666666.59339.peg.3059	fig|6666666.59340.peg.192	fig|6666666.59337.peg.3735	fig|6666666.59342.peg.4071	fig|6666666.71181.peg.3502	fig|6666666.59344.peg.5524	fig|6666666.59345.peg.1196	fig|6666666.59341.peg.2456	None	fig|6666666.59346.peg.61	fig|6666666.59348.peg.455	fig|6666666.59349.peg.1660	fig|6666666.59350.peg.1502	fig|6666666.59351.peg.2403	fig|6666666.59353.peg.4080	fig|6666666.59355.peg.4156	fig|6666666.59354.peg.2060

9390	tRNA 2-thiouridine synthesizing protein E EC 2.8.1.-	fig|6666666.59329.peg.3252	fig|6666666.59330.peg.1035	None	fig|6666666.59332.peg.3262	fig|6666666.59334.peg.3984	fig|6666666.59335.peg.3577	fig|6666666.59336.peg.6225	fig|6666666.59333.peg.2073	fig|6666666.59338.peg.2699	fig|6666666.59339.peg.1565	None	fig|6666666.59337.peg.3177	fig|6666666.59342.peg.2779	fig|6666666.71181.peg.277	fig|6666666.59344.peg.1204	fig|6666666.59345.peg.3280	None	fig|6666666.59347.peg.1795	fig|6666666.59346.peg.4461	fig|6666666.59348.peg.5046	fig|6666666.59349.peg.4845	None	fig|6666666.59351.peg.3128	fig|6666666.59353.peg.1707	fig|6666666.59355.peg.1013	fig|6666666.59354.peg.5883

1575	FIG01200173 hypothetical protein	fig|6666666.59329.peg.673	fig|6666666.59330.peg.3772	None	fig|6666666.59332.peg.133	fig|6666666.59334.peg.226	fig|6666666.59335.peg.5708	None	fig|6666666.59333.peg.2190	fig|6666666.59338.peg.1360	fig|6666666.59339.peg.6425	fig|6666666.59340.peg.2330	fig|6666666.59337.peg.4508	fig|6666666.59342.peg.6147	fig|6666666.71181.peg.3352	fig|6666666.59344.peg.5680	fig|6666666.59345.peg.5691	fig|6666666.59341.peg.1735	fig|6666666.59347.peg.5427	fig|6666666.59346.peg.1140	fig|6666666.59348.peg.3194	fig|6666666.59349.peg.1695	None	fig|6666666.59351.peg.5220	fig|6666666.59353.peg.4471	fig|6666666.59355.peg.4556	None

1723	Conserved domain associated with flavoprotein oxygenases, DIM6 NTAB family	fig|6666666.59329.peg.4683	fig|6666666.59330.peg.2239	fig|6666666.59331.peg.929	fig|6666666.59332.peg.4597	fig|6666666.59334.peg.4324	fig|6666666.59335.peg.5900	fig|6666666.59336.peg.4148	None	fig|6666666.59338.peg.4721	fig|6666666.59339.peg.3903	None	fig|6666666.59337.peg.5047	fig|6666666.59342.peg.2637	fig|6666666.71181.peg.2119	fig|6666666.59344.peg.5321	fig|6666666.59345.peg.1885	None	fig|6666666.59347.peg.500	fig|6666666.59346.peg.2297	None	fig|6666666.59349.peg.4084	fig|6666666.59350.peg.5199	fig|6666666.59351.peg.1146	fig|6666666.59353.peg.709	fig|6666666.59355.peg.196	fig|6666666.59354.peg.464

7771	FIG01199637 hypothetical protein	None	fig|6666666.59330.peg.3108	fig|6666666.59331.peg.4228	fig|6666666.59332.peg.2338	None	fig|6666666.59335.peg.1714	fig|6666666.59336.peg.3692	fig|6666666.59333.peg.220	fig|6666666.59338.peg.29	fig|6666666.59339.peg.7232	fig|6666666.59340.peg.1992	fig|6666666.59337.peg.5628	None	fig|6666666.71181.peg.3608	fig|6666666.59344.peg.5052	fig|6666666.59345.peg.5429	fig|6666666.59341.peg.4016	fig|6666666.59347.peg.2891	fig|6666666.59346.peg.3349	fig|6666666.59348.peg.1642	fig|6666666.59349.peg.1123	None	fig|6666666.59351.peg.78	fig|6666666.59353.peg.4061	fig|6666666.59355.peg.4137	fig|6666666.59354.peg.302

6081	Universal stress protein family 3	fig|6666666.59329.peg.1477	fig|6666666.59330.peg.833	fig|6666666.59331.peg.2484	fig|6666666.59332.peg.4620	fig|6666666.59334.peg.4205	fig|6666666.59335.peg.226	fig|6666666.59336.peg.4762	fig|6666666.59333.peg.559	fig|6666666.59338.peg.4679	fig|6666666.59339.peg.8175	fig|6666666.59340.peg.2644	fig|6666666.59337.peg.1367	None	fig|6666666.71181.peg.335	fig|6666666.59344.peg.1275	fig|6666666.59345.peg.2476	fig|6666666.59341.peg.898	fig|6666666.59347.peg.3104	fig|6666666.59346.peg.3803	fig|6666666.59348.peg.556	None	fig|6666666.59350.peg.812	None	fig|6666666.59353.peg.4210	fig|6666666.59355.peg.4173	None

4417	Imidazolonepropionase EC 3.5.2.7	fig|6666666.59329.peg.4895	fig|6666666.59330.peg.510	fig|6666666.59331.peg.2413	fig|6666666.59332.peg.3277	None	fig|6666666.59335.peg.1566	fig|6666666.59336.peg.2093	fig|6666666.59333.peg.3577	fig|6666666.59338.peg.427	fig|6666666.59339.peg.3877	None	fig|6666666.59337.peg.3303	None	fig|6666666.71181.peg.1206	fig|6666666.59344.peg.209	fig|6666666.59345.peg.4629	fig|6666666.59341.peg.1315	fig|6666666.59347.peg.1557	fig|6666666.59346.peg.3554	None	fig|6666666.59349.peg.499	fig|6666666.59350.peg.1594	fig|6666666.59351.peg.3822	fig|6666666.59353.peg.513	fig|6666666.59355.peg.2239	fig|6666666.59354.peg.946

4413	NAD synthetase EC 6.3.1.5	fig|6666666.59329.peg.2818	fig|6666666.59330.peg.2711	fig|6666666.59331.peg.5472	fig|6666666.59332.peg.989	fig|6666666.59334.peg.2855	fig|6666666.59335.peg.1625	fig|6666666.59336.peg.5656	fig|6666666.59333.peg.3907	fig|6666666.59338.peg.1876	fig|6666666.59339.peg.6544	None	fig|6666666.59337.peg.2440	fig|6666666.59342.peg.2953	fig|6666666.71181.peg.4731	None	fig|6666666.59345.peg.609	fig|6666666.59341.peg.5417	fig|6666666.59347.peg.544	fig|6666666.59346.peg.3018	None	None	fig|6666666.59350.peg.1664	fig|6666666.59351.peg.4735	fig|6666666.59353.peg.2674	fig|6666666.59355.peg.1807	fig|6666666.59354.peg.5910

9150	Lipopolysaccharide biosynthesis glycosyltransferase	fig|6666666.59329.peg.556	fig|6666666.59330.peg.1160	fig|6666666.59331.peg.2860	fig|6666666.59332.peg.289	fig|6666666.59334.peg.1774	fig|6666666.59335.peg.2001	fig|6666666.59336.peg.61	fig|6666666.59333.peg.647	fig|6666666.59338.peg.1910	fig|6666666.59339.peg.6299	fig|6666666.59340.peg.2656	fig|6666666.59337.peg.465	fig|6666666.59342.peg.5929	fig|6666666.71181.peg.3658	fig|6666666.59344.peg.946	fig|6666666.59345.peg.2613	fig|6666666.59341.peg.4928	fig|6666666.59347.peg.5188	fig|6666666.59346.peg.1706	fig|6666666.59348.peg.876	None	None	None	fig|6666666.59353.peg.2588	fig|6666666.59355.peg.2613	None

3062	tRNA uridine-5-oxyacetic acid methyl ester 34 synthase	fig|6666666.59329.peg.5096	fig|6666666.59330.peg.2483	fig|6666666.59331.peg.6376	fig|6666666.59332.peg.4762	fig|6666666.59334.peg.1880	fig|6666666.59335.peg.952	fig|6666666.59336.peg.3636	fig|6666666.59333.peg.1049	None	fig|6666666.59339.peg.337	None	fig|6666666.59337.peg.4545	fig|6666666.59342.peg.1781	fig|6666666.71181.peg.1914	fig|6666666.59344.peg.2525	fig|6666666.59345.peg.1520	None	fig|6666666.59347.peg.4193	fig|6666666.59346.peg.2771	fig|6666666.59348.peg.1588	fig|6666666.59349.peg.2670	None	fig|6666666.59351.peg.5165	fig|6666666.59353.peg.1495	fig|6666666.59355.peg.2045	fig|6666666.59354.peg.4643

7751	FIG003276 zinc-binding protein	fig|6666666.59329.peg.340	fig|6666666.59330.peg.4891	None	fig|6666666.59332.peg.311	fig|6666666.59334.peg.5124	fig|6666666.59335.peg.5395	fig|6666666.59336.peg.1423	fig|6666666.59333.peg.157	fig|6666666.59338.peg.2610	fig|6666666.59339.peg.7937	None	fig|6666666.59337.peg.2045	None	fig|6666666.71181.peg.1969	fig|6666666.59344.peg.1466	fig|6666666.59345.peg.2429	fig|6666666.59341.peg.1346	fig|6666666.59347.peg.3888	fig|6666666.59346.peg.573	fig|6666666.59348.peg.1263	fig|6666666.59349.peg.2592	None	fig|6666666.59351.peg.272	fig|6666666.59353.peg.150	fig|6666666.59355.peg.760	fig|6666666.59354.peg.4878

9904	ThiJ PfpI family protein	None	fig|6666666.59330.peg.4534	fig|6666666.59331.peg.2486	fig|6666666.59332.peg.318	fig|6666666.59334.peg.829	fig|6666666.59335.peg.677	fig|6666666.59336.peg.5633	fig|6666666.59333.peg.4284	fig|6666666.59338.peg.6079	fig|6666666.59339.peg.1782	None	fig|6666666.59337.peg.5029	None	fig|6666666.71181.peg.1330	fig|6666666.59344.peg.2920	fig|6666666.59345.peg.325	fig|6666666.59341.peg.5644	fig|6666666.59347.peg.1267	fig|6666666.59346.peg.349	None	fig|6666666.59349.peg.4656	fig|6666666.59350.peg.2940	fig|6666666.59351.peg.1331	fig|6666666.59353.peg.3270	fig|6666666.59355.peg.3182	fig|6666666.59354.peg.2434

4042	Glycerol kinase EC 2.7.1.30	fig|6666666.59329.peg.1138	fig|6666666.59330.peg.2822	fig|6666666.59331.peg.1943	fig|6666666.59332.peg.2482	fig|6666666.59334.peg.4282	None	fig|6666666.59336.peg.4686	fig|6666666.59333.peg.3441	fig|6666666.59338.peg.5275	fig|6666666.59339.peg.2809	fig|6666666.59340.peg.2580	fig|6666666.59337.peg.5136	fig|6666666.59342.peg.5480	fig|6666666.71181.peg.4716	fig|6666666.59344.peg.992	fig|6666666.59345.peg.2277	fig|6666666.59341.peg.3984	fig|6666666.59347.peg.2053	fig|6666666.59346.peg.2362	fig|6666666.59348.peg.1603	None	fig|6666666.59350.peg.6021	None	fig|6666666.59353.peg.4386	fig|6666666.59355.peg.4752	None

977	Regulator of sigma D	fig|6666666.59329.peg.5034	fig|6666666.59330.peg.3764	fig|6666666.59331.peg.6373	fig|6666666.59332.peg.139	fig|6666666.59334.peg.1235	fig|6666666.59335.peg.1943	fig|6666666.59336.peg.3844	None	fig|6666666.59338.peg.3037	fig|6666666.59339.peg.8834	None	fig|6666666.59337.peg.4516	None	fig|6666666.71181.peg.3356	fig|6666666.59344.peg.5684	fig|6666666.59345.peg.5686	fig|6666666.59341.peg.1728	fig|6666666.59347.peg.5395	fig|6666666.59346.peg.1134	fig|6666666.59348.peg.1007	fig|6666666.59349.peg.3367	None	fig|6666666.59351.peg.4182	fig|6666666.59353.peg.4467	fig|6666666.59355.peg.4560	fig|6666666.59354.peg.1671

8922	Adenylate cyclase EC 4.6.1.1	fig|6666666.59329.peg.93	fig|6666666.59330.peg.3048	fig|6666666.59331.peg.6229	fig|6666666.59332.peg.1709	fig|6666666.59334.peg.1134	fig|6666666.59335.peg.3332	fig|6666666.59336.peg.1121	fig|6666666.59333.peg.786	fig|6666666.59338.peg.1776	fig|6666666.59339.peg.8519	fig|6666666.59340.peg.789	fig|6666666.59337.peg.1614	fig|6666666.59342.peg.3155	fig|6666666.71181.peg.2435	fig|6666666.59344.peg.2484	fig|6666666.59345.peg.5505	fig|6666666.59341.peg.3916	fig|6666666.59347.peg.5080	fig|6666666.59346.peg.597	fig|6666666.59348.peg.3775	None	None	None	fig|6666666.59353.peg.3634	fig|6666666.59355.peg.4990	None

5206	LSU ribosomal protein L30p L7e	fig|6666666.59329.peg.727	fig|6666666.59330.peg.568	fig|6666666.59331.peg.34	fig|6666666.59332.peg.2153	fig|6666666.59334.peg.4020	fig|6666666.59335.peg.4361	fig|6666666.59336.peg.160	fig|6666666.59333.peg.380	fig|6666666.59338.peg.4968	fig|6666666.59339.peg.8281	fig|6666666.59340.peg.3479	fig|6666666.59337.peg.374	fig|6666666.59342.peg.318	None	None	fig|6666666.59345.peg.2521	fig|6666666.59341.peg.2512	fig|6666666.59347.peg.4837	fig|6666666.59346.peg.864	fig|6666666.59348.peg.440	fig|6666666.59349.peg.3284	fig|6666666.59350.peg.6334	fig|6666666.59351.peg.4218	None	None	fig|6666666.59354.peg.3254

3953	Glutathione peroxidase EC 1.11.1.9	fig|6666666.59329.peg.4914	fig|6666666.59330.peg.864	fig|6666666.59331.peg.1577	fig|6666666.59332.peg.3563	fig|6666666.59334.peg.4632	None	fig|6666666.59336.peg.5906	fig|6666666.59333.peg.62	fig|6666666.59338.peg.2488	None	None	fig|6666666.59337.peg.3284	fig|6666666.59342.peg.3547	fig|6666666.71181.peg.2388	fig|6666666.59344.peg.770	fig|6666666.59345.peg.548	fig|6666666.59341.peg.5137	fig|6666666.59347.peg.431	fig|6666666.59346.peg.1871	None	fig|6666666.59349.peg.3159	fig|6666666.59350.peg.1132	fig|6666666.59351.peg.3371	fig|6666666.59353.peg.1311	fig|6666666.59355.peg.2560	fig|6666666.59354.peg.4505

11430	Cell division protein ZipA	fig|6666666.59329.peg.912	fig|6666666.59330.peg.3034	None	fig|6666666.59332.peg.635	fig|6666666.59334.peg.3262	fig|6666666.59335.peg.3018	fig|6666666.59336.peg.2342	fig|6666666.59333.peg.4115	fig|6666666.59338.peg.21	fig|6666666.59339.peg.5235	None	fig|6666666.59337.peg.5547	fig|6666666.59342.peg.108	fig|6666666.71181.peg.1575	fig|6666666.59344.peg.2010	fig|6666666.59345.peg.4389	None	fig|6666666.59347.peg.2897	fig|6666666.59346.peg.2377	fig|6666666.59348.peg.3200	fig|6666666.59349.peg.2495	None	fig|6666666.59351.peg.4633	fig|6666666.59353.peg.2914	fig|6666666.59355.peg.2825	fig|6666666.59354.peg.4788

958	Crossover junction endodeoxyribonuclease RuvC EC 3.1.22.4	fig|6666666.59329.peg.3403	fig|6666666.59330.peg.2486	None	fig|6666666.59332.peg.1958	None	fig|6666666.59335.peg.2495	fig|6666666.59336.peg.3633	fig|6666666.59333.peg.1044	fig|6666666.59338.peg.5806	fig|6666666.59339.peg.380	None	fig|6666666.59337.peg.4548	fig|6666666.59342.peg.4935	fig|6666666.71181.peg.1921	fig|6666666.59344.peg.2516	fig|6666666.59345.peg.1517	fig|6666666.59341.peg.3337	fig|6666666.59347.peg.4197	fig|6666666.59346.peg.2768	None	fig|6666666.59349.peg.3867	fig|6666666.59350.peg.4001	fig|6666666.59351.peg.653	fig|6666666.59353.peg.1488	fig|6666666.59355.peg.2038	fig|6666666.59354.peg.1590

11227	Uncharacterized iron-regulated membrane protein; Iron-uptake factor PiuB	fig|6666666.59329.peg.1547	fig|6666666.59330.peg.2207	fig|6666666.59331.peg.3049	fig|6666666.59332.peg.705	fig|6666666.59334.peg.3394	fig|6666666.59335.peg.3236	fig|6666666.59336.peg.2539	fig|6666666.59333.peg.4380	fig|6666666.59338.peg.2267	fig|6666666.59339.peg.2453	fig|6666666.59340.peg.708	fig|6666666.59337.peg.1897	None	fig|6666666.71181.peg.1292	fig|6666666.59344.peg.2962	fig|6666666.59345.peg.2837	None	fig|6666666.59347.peg.3297	fig|6666666.59346.peg.3641	None	fig|6666666.59349.peg.3723	None	fig|6666666.59351.peg.1932	fig|6666666.59353.peg.3306	fig|6666666.59355.peg.3146	fig|6666666.59354.peg.189

5126	Protein sprT	fig|6666666.59329.peg.1776	fig|6666666.59330.peg.350	fig|6666666.59331.peg.3185	fig|6666666.59332.peg.1935	fig|6666666.59334.peg.3897	fig|6666666.59335.peg.3098	fig|6666666.59336.peg.3966	fig|6666666.59333.peg.777	fig|6666666.59338.peg.453	fig|6666666.59339.peg.7839	fig|6666666.59340.peg.209	fig|6666666.59337.peg.531	fig|6666666.59342.peg.3321	fig|6666666.71181.peg.1858	fig|6666666.59344.peg.4332	fig|6666666.59345.peg.5021	fig|6666666.59341.peg.2579	fig|6666666.59347.peg.3210	fig|6666666.59346.peg.3516	fig|6666666.59348.peg.359	None	None	None	fig|6666666.59353.peg.3223	fig|6666666.59355.peg.3105	None

9448	Rod shape-determining protein MreC	fig|6666666.59329.peg.706	fig|6666666.59330.peg.25	fig|6666666.59331.peg.5596	None	fig|6666666.59334.peg.1313	fig|6666666.59335.peg.3027	fig|6666666.59336.peg.3614	fig|6666666.59333.peg.3653	fig|6666666.59338.peg.3606	fig|6666666.59339.peg.7789	None	fig|6666666.59337.peg.810	fig|6666666.59342.peg.3379	fig|6666666.71181.peg.979	None	fig|6666666.59345.peg.4884	fig|6666666.59341.peg.883	fig|6666666.59347.peg.3115	fig|6666666.59346.peg.2127	fig|6666666.59348.peg.2479	fig|6666666.59349.peg.5	fig|6666666.59350.peg.3073	fig|6666666.59351.peg.3422	fig|6666666.59353.peg.2293	None	fig|6666666.59354.peg.3166

450	Thioredoxin	fig|6666666.59329.peg.793	fig|6666666.59330.peg.3384	fig|6666666.59331.peg.1279	fig|6666666.59332.peg.5918	fig|6666666.59334.peg.4572	fig|6666666.59335.peg.5001	fig|6666666.59336.peg.3556	fig|6666666.59333.peg.3753	fig|6666666.59338.peg.1696	fig|6666666.59339.peg.4068	None	fig|6666666.59337.peg.4419	fig|6666666.59342.peg.1045	fig|6666666.71181.peg.97	fig|6666666.59344.peg.3165	fig|6666666.59345.peg.1133	fig|6666666.59341.peg.5489	fig|6666666.59347.peg.2187	fig|6666666.59346.peg.4471	fig|6666666.59348.peg.3068	None	None	fig|6666666.59351.peg.4400	fig|6666666.59353.peg.1916	fig|6666666.59355.peg.1492	None

6928	Predicted exporter of the RND superfamily	fig|6666666.59329.peg.3774	fig|6666666.59330.peg.528	fig|6666666.59331.peg.3159	fig|6666666.59332.peg.2173	None	fig|6666666.59335.peg.3613	fig|6666666.59336.peg.1701	fig|6666666.59333.peg.1102	None	fig|6666666.59339.peg.587	None	fig|6666666.59337.peg.3983	fig|6666666.59342.peg.4833	fig|6666666.71181.peg.3930	fig|6666666.59344.peg.5389	fig|6666666.59345.peg.4645	fig|6666666.59341.peg.4582	fig|6666666.59347.peg.2359	fig|6666666.59346.peg.3127	fig|6666666.59348.peg.1820	fig|6666666.59349.peg.3355	None	fig|6666666.59351.peg.3159	fig|6666666.59353.peg.4787	fig|6666666.59355.peg.4841	fig|6666666.59354.peg.3910

6924	L-alanine-DL-glutamate epimerase	fig|6666666.59329.peg.4791	fig|6666666.59330.peg.4099	fig|6666666.59331.peg.5145	fig|6666666.59332.peg.2701	fig|6666666.59334.peg.4779	fig|6666666.59335.peg.4392	fig|6666666.59336.peg.5646	fig|6666666.59333.peg.823	fig|6666666.59338.peg.3710	fig|6666666.59339.peg.4128	None	fig|6666666.59337.peg.2861	fig|6666666.59342.peg.5911	fig|6666666.71181.peg.1381	fig|6666666.59344.peg.541	fig|6666666.59345.peg.1730	fig|6666666.59341.peg.2424	fig|6666666.59347.peg.2470	fig|6666666.59346.peg.4781	fig|6666666.59348.peg.4920	None	fig|6666666.59350.peg.2943	None	fig|6666666.59353.peg.1847	fig|6666666.59355.peg.1166	None

3625	LSU ribosomal protein L9p	fig|6666666.59329.peg.322	fig|6666666.59330.peg.2642	fig|6666666.59331.peg.5467	fig|6666666.59332.peg.900	fig|6666666.59334.peg.168	fig|6666666.59335.peg.3491	fig|6666666.59336.peg.597	fig|6666666.59333.peg.888	None	fig|6666666.59339.peg.7697	None	fig|6666666.59337.peg.1708	fig|6666666.59342.peg.1894	fig|6666666.71181.peg.2818	fig|6666666.59344.peg.2147	fig|6666666.59345.peg.5374	fig|6666666.59341.peg.2418	None	fig|6666666.59346.peg.2388	fig|6666666.59348.peg.5050	fig|6666666.59349.peg.3231	fig|6666666.59350.peg.1867	fig|6666666.59351.peg.5425	fig|6666666.59353.peg.3176	fig|6666666.59355.peg.421	None

3627	4-hydroxybenzoate transporter	fig|6666666.59329.peg.1874	None	fig|6666666.59331.peg.3605	fig|6666666.59332.peg.5341	fig|6666666.59334.peg.3793	fig|6666666.59335.peg.3389	fig|6666666.59336.peg.2056	fig|6666666.59333.peg.2171	fig|6666666.59338.peg.4509	fig|6666666.59339.peg.692	None	fig|6666666.59337.peg.3417	fig|6666666.59342.peg.969	fig|6666666.71181.peg.3726	fig|6666666.59344.peg.3592	fig|6666666.59345.peg.1935	fig|6666666.59341.peg.5804	fig|6666666.59347.peg.3473	fig|6666666.59346.peg.1019	fig|6666666.59348.peg.3135	fig|6666666.59349.peg.2055	None	None	fig|6666666.59353.peg.2152	fig|6666666.59355.peg.3218	fig|6666666.59354.peg.731

8587	YciL protein	fig|6666666.59329.peg.5751	fig|6666666.59330.peg.3347	None	fig|6666666.59332.peg.6227	None	fig|6666666.59335.peg.5262	fig|6666666.59336.peg.4859	fig|6666666.59333.peg.1917	fig|6666666.59338.peg.3266	fig|6666666.59339.peg.557	None	fig|6666666.59337.peg.3509	fig|6666666.59342.peg.4061	fig|6666666.71181.peg.2780	fig|6666666.59344.peg.443	fig|6666666.59345.peg.927	fig|6666666.59341.peg.3420	fig|6666666.59347.peg.3563	fig|6666666.59346.peg.4964	fig|6666666.59348.peg.368	fig|6666666.59349.peg.2685	fig|6666666.59350.peg.1754	fig|6666666.59351.peg.779	fig|6666666.59353.peg.1763	fig|6666666.59355.peg.5451	None

355	FIG00921969 hypothetical protein	fig|6666666.59329.peg.3283	fig|6666666.59330.peg.1313	fig|6666666.59331.peg.2478	fig|6666666.59332.peg.5696	fig|6666666.59334.peg.2408	fig|6666666.59335.peg.5208	fig|6666666.59336.peg.856	fig|6666666.59333.peg.210	fig|6666666.59338.peg.768	fig|6666666.59339.peg.456	fig|6666666.59340.peg.2941	fig|6666666.59337.peg.3331	fig|6666666.59342.peg.1503	fig|6666666.71181.peg.272	fig|6666666.59344.peg.1199	fig|6666666.59345.peg.1559	fig|6666666.59341.peg.4616	fig|6666666.59347.peg.2360	fig|6666666.59346.peg.724	fig|6666666.59348.peg.3487	None	None	None	fig|6666666.59353.peg.1702	fig|6666666.59355.peg.1018	None

9791	Predicted hydrolase	fig|6666666.59329.peg.2158	fig|6666666.59330.peg.4963	fig|6666666.59331.peg.3153	fig|6666666.59332.peg.1603	fig|6666666.59334.peg.1229	fig|6666666.59335.peg.5824	fig|6666666.59336.peg.4110	fig|6666666.59333.peg.3757	fig|6666666.59338.peg.2307	fig|6666666.59339.peg.1764	fig|6666666.59340.peg.3525	fig|6666666.59337.peg.4954	fig|6666666.59342.peg.5756	fig|6666666.71181.peg.2515	fig|6666666.59344.peg.4078	fig|6666666.59345.peg.4493	fig|6666666.59341.peg.4852	fig|6666666.59347.peg.5152	fig|6666666.59346.peg.2758	fig|6666666.59348.peg.2201	None	None	None	fig|6666666.59353.peg.3053	fig|6666666.59355.peg.2293	None

4021	Phosphonoacetaldehyde hydrolase EC 3.11.1.1	fig|6666666.59329.peg.3785	fig|6666666.59330.peg.2372	fig|6666666.59331.peg.1159	fig|6666666.59332.peg.4903	fig|6666666.59334.peg.4039	fig|6666666.59335.peg.962	fig|6666666.59336.peg.3723	None	fig|6666666.59338.peg.3456	fig|6666666.59339.peg.4487	None	fig|6666666.59337.peg.2246	fig|6666666.59342.peg.5147	fig|6666666.71181.peg.3843	fig|6666666.59344.peg.836	fig|6666666.59345.peg.2959	fig|6666666.59341.peg.5667	fig|6666666.59347.peg.484	fig|6666666.59346.peg.1061	fig|6666666.59348.peg.4750	fig|6666666.59349.peg.2103	None	None	fig|6666666.59353.peg.1277	fig|6666666.59355.peg.3306	fig|6666666.59354.peg.2217

378	DNA polymerase II EC 2.7.7.7	fig|6666666.59329.peg.2703	fig|6666666.59330.peg.2936	None	fig|6666666.59332.peg.3764	fig|6666666.59334.peg.4509	fig|6666666.59335.peg.3761	fig|6666666.59336.peg.871	fig|6666666.59333.peg.4975	fig|6666666.59338.peg.5152	fig|6666666.59339.peg.572	None	fig|6666666.59337.peg.1736	fig|6666666.59342.peg.2144	fig|6666666.71181.peg.393	fig|6666666.59344.peg.1516	fig|6666666.59345.peg.906	fig|6666666.59341.peg.375	fig|6666666.59347.peg.2295	fig|6666666.59346.peg.1614	fig|6666666.59348.peg.3413	fig|6666666.59349.peg.1913	None	fig|6666666.59351.peg.475	fig|6666666.59353.peg.105	fig|6666666.59355.peg.715	None

9346	IcmF-related protein	fig|6666666.59329.peg.4246	fig|6666666.59330.peg.2152	fig|6666666.59331.peg.5549	fig|6666666.59332.peg.5877	None	fig|6666666.59335.peg.4105	fig|6666666.59336.peg.5811	fig|6666666.59333.peg.3392	fig|6666666.59338.peg.4304	fig|6666666.59339.peg.3976	fig|6666666.59340.peg.1617	fig|6666666.59337.peg.5620	fig|6666666.59342.peg.5217	fig|6666666.71181.peg.2265	fig|6666666.59344.peg.2991	fig|6666666.59345.peg.4120	fig|6666666.59341.peg.4554	fig|6666666.59347.peg.1018	None	fig|6666666.59348.peg.2012	fig|6666666.59349.peg.1645	None	None	fig|6666666.59353.peg.3561	fig|6666666.59355.peg.997	fig|6666666.59354.peg.747

3328	SAM-dependent methyltransferase EC 2.1.1.-	None	fig|6666666.59330.peg.231	fig|6666666.59331.peg.558	fig|6666666.59332.peg.1717	fig|6666666.59334.peg.1190	fig|6666666.59335.peg.20	None	fig|6666666.59333.peg.679	fig|6666666.59338.peg.73	fig|6666666.59339.peg.7547	fig|6666666.59340.peg.164	fig|6666666.59337.peg.948	None	fig|6666666.71181.peg.1057	fig|6666666.59344.peg.4959	fig|6666666.59345.peg.2727	fig|6666666.59341.peg.4148	fig|6666666.59347.peg.4569	fig|6666666.59346.peg.3441	fig|6666666.59348.peg.2081	fig|6666666.59349.peg.126	None	fig|6666666.59351.peg.3310	fig|6666666.59353.peg.4518	fig|6666666.59355.peg.4628	fig|6666666.59354.peg.5373

11100	SSU ribosomal protein S20p	fig|6666666.59329.peg.4770	fig|6666666.59330.peg.2509	fig|6666666.59331.peg.3000	fig|6666666.59332.peg.3314	fig|6666666.59334.peg.2144	fig|6666666.59335.peg.1012	fig|6666666.59336.peg.5785	fig|6666666.59333.peg.3798	fig|6666666.59338.peg.2729	fig|6666666.59339.peg.7463	fig|6666666.59340.peg.3304	fig|6666666.59337.peg.130	fig|6666666.59342.peg.1395	None	None	fig|6666666.59345.peg.3713	fig|6666666.59341.peg.5674	fig|6666666.59347.peg.3023	fig|6666666.59346.peg.3082	fig|6666666.59348.peg.1112	fig|6666666.59349.peg.564	fig|6666666.59350.peg.2727	fig|6666666.59351.peg.499	None	None	fig|6666666.59354.peg.5851

7543	FIG01199739 hypothetical protein	fig|6666666.59329.peg.3851	fig|6666666.59330.peg.745	fig|6666666.59331.peg.4772	fig|6666666.59332.peg.3679	fig|6666666.59334.peg.5246	fig|6666666.59335.peg.1931	fig|6666666.59336.peg.2759	fig|6666666.59333.peg.2428	fig|6666666.59338.peg.1485	fig|6666666.59339.peg.8402	None	fig|6666666.59337.peg.3474	fig|6666666.59342.peg.5067	fig|6666666.71181.peg.2556	fig|6666666.59344.peg.742	fig|6666666.59345.peg.2779	fig|6666666.59341.peg.2261	fig|6666666.59347.peg.440	fig|6666666.59346.peg.5045	fig|6666666.59348.peg.2764	None	fig|6666666.59350.peg.1676	None	fig|6666666.59353.peg.1360	fig|6666666.59355.peg.5206	None

9428	L-ribulose-5-phosphate 4-epimerase EC 5.1.3.4	fig|6666666.59329.peg.2157	fig|6666666.59330.peg.4023	fig|6666666.59331.peg.1414	fig|6666666.59332.peg.2909	fig|6666666.59334.peg.1231	fig|6666666.59335.peg.4912	fig|6666666.59336.peg.3186	fig|6666666.59333.peg.3730	None	None	fig|6666666.59340.peg.2193	fig|6666666.59337.peg.2936	fig|6666666.59342.peg.1768	fig|6666666.71181.peg.906	fig|6666666.59344.peg.5297	fig|6666666.59345.peg.4534	fig|6666666.59341.peg.718	None	fig|6666666.59346.peg.2345	None	fig|6666666.59349.peg.2217	fig|6666666.59350.peg.807	fig|6666666.59351.peg.5009	fig|6666666.59353.peg.912	fig|6666666.59355.peg.3426	fig|6666666.59354.peg.3603

1482	21 kDa hemolysin precursor	fig|6666666.59329.peg.2051	fig|6666666.59330.peg.969	fig|6666666.59331.peg.4186	fig|6666666.59332.peg.643	fig|6666666.59334.peg.2334	fig|6666666.59335.peg.4928	fig|6666666.59336.peg.715	fig|6666666.59333.peg.613	fig|6666666.59338.peg.542	fig|6666666.59339.peg.4949	None	fig|6666666.59337.peg.34	None	fig|6666666.71181.peg.4141	fig|6666666.59344.peg.452	fig|6666666.59345.peg.3808	fig|6666666.59341.peg.1364	fig|6666666.59347.peg.3987	fig|6666666.59346.peg.2331	None	fig|6666666.59349.peg.46	None	fig|6666666.59351.peg.3467	fig|6666666.59353.peg.1773	fig|6666666.59355.peg.1239	fig|6666666.59354.peg.364

10611	L-asparaginase EC 3.5.1.1	fig|6666666.59329.peg.3069	fig|6666666.59330.peg.1213	None	None	fig|6666666.59334.peg.1618	fig|6666666.59335.peg.3965	fig|6666666.59336.peg.3561	fig|6666666.59333.peg.1083	fig|6666666.59338.peg.4338	fig|6666666.59339.peg.3138	None	fig|6666666.59337.peg.3130	fig|6666666.59342.peg.1233	fig|6666666.71181.peg.2974	fig|6666666.59344.peg.2897	fig|6666666.59345.peg.1160	fig|6666666.59341.peg.1111	fig|6666666.59347.peg.2587	fig|6666666.59346.peg.82	fig|6666666.59348.peg.4873	fig|6666666.59349.peg.395	None	fig|6666666.59351.peg.923	fig|6666666.59353.peg.1430	fig|6666666.59355.peg.1979	fig|6666666.59354.peg.1351

3872	C1720 Uncharacterized conserved protein	fig|6666666.59329.peg.1825	fig|6666666.59330.peg.484	None	None	fig|6666666.59334.peg.316	fig|6666666.59335.peg.4082	fig|6666666.59336.peg.2410	fig|6666666.59333.peg.2257	fig|6666666.59338.peg.728	fig|6666666.59339.peg.5161	None	fig|6666666.59337.peg.1566	fig|6666666.59342.peg.2999	fig|6666666.71181.peg.7	fig|6666666.59344.peg.2683	fig|6666666.59345.peg.2216	fig|6666666.59341.peg.3977	fig|6666666.59347.peg.4353	fig|6666666.59346.peg.3532	fig|6666666.59348.peg.4504	fig|6666666.59349.peg.4183	None	fig|6666666.59351.peg.1081	fig|6666666.59353.peg.2013	fig|6666666.59355.peg.4118	fig|6666666.59354.peg.2518

9654	Dihydroorotase EC 3.5.2.3	fig|6666666.59329.peg.2821	fig|6666666.59330.peg.2708	None	fig|6666666.59332.peg.4636	None	fig|6666666.59335.peg.3237	fig|6666666.59336.peg.4242	fig|6666666.59333.peg.3903	fig|6666666.59338.peg.1881	fig|6666666.59339.peg.2456	None	fig|6666666.59337.peg.2437	fig|6666666.59342.peg.5834	fig|6666666.71181.peg.1611	fig|6666666.59344.peg.2420	fig|6666666.59345.peg.612	fig|6666666.59341.peg.1876	fig|6666666.59347.peg.540	fig|6666666.59346.peg.3603	None	fig|6666666.59349.peg.1333	fig|6666666.59350.peg.6450	fig|6666666.59351.peg.5093	fig|6666666.59353.peg.302	fig|6666666.59355.peg.1342	fig|6666666.59354.peg.164

6398	possible efflux permease	None	fig|6666666.59330.peg.4651	fig|6666666.59331.peg.5228	fig|6666666.59332.peg.819	fig|6666666.59334.peg.3570	fig|6666666.59335.peg.2181	fig|6666666.59336.peg.3974	fig|6666666.59333.peg.1772	None	fig|6666666.59339.peg.3923	fig|6666666.59340.peg.2214	fig|6666666.59337.peg.1992	fig|6666666.59342.peg.3505	fig|6666666.71181.peg.2714	fig|6666666.59344.peg.3910	fig|6666666.59345.peg.4683	fig|6666666.59341.peg.2730	fig|6666666.59347.peg.3343	fig|6666666.59346.peg.2047	fig|6666666.59348.peg.1908	fig|6666666.59349.peg.4628	None	fig|6666666.59351.peg.3106	fig|6666666.59353.peg.3964	fig|6666666.59355.peg.3880	None

5430	Flagellar protein FlgJ [peptidoglycan hydrolase] EC 3.2.1.-	fig|6666666.59329.peg.2686	fig|6666666.59330.peg.2679	None	fig|6666666.59332.peg.2243	None	fig|6666666.59335.peg.2091	fig|6666666.59336.peg.2919	fig|6666666.59333.peg.2895	fig|6666666.59338.peg.1245	fig|6666666.59339.peg.5323	fig|6666666.59340.peg.1767	fig|6666666.59337.peg.3187	fig|6666666.59342.peg.6217	fig|6666666.71181.peg.2222	fig|6666666.59344.peg.3044	fig|6666666.59345.peg.5458	fig|6666666.59341.peg.112	fig|6666666.59347.peg.4601	fig|6666666.59346.peg.1770	fig|6666666.59348.peg.957	fig|6666666.59349.peg.731	None	fig|6666666.59351.peg.1990	fig|6666666.59353.peg.3519	fig|6666666.59355.peg.3657	None

3855	NAD-dependent protein deacetylase of SIR2 family	None	fig|6666666.59330.peg.2600	fig|6666666.59331.peg.3962	fig|6666666.59332.peg.6027	fig|6666666.59334.peg.2463	fig|6666666.59335.peg.2204	None	fig|6666666.59333.peg.3974	fig|6666666.59338.peg.1302	fig|6666666.59339.peg.1297	None	fig|6666666.59337.peg.3707	fig|6666666.59342.peg.220	fig|6666666.71181.peg.1271	fig|6666666.59344.peg.128	fig|6666666.59345.peg.3216	fig|6666666.59341.peg.4675	fig|6666666.59347.peg.1725	fig|6666666.59346.peg.3588	fig|6666666.59348.peg.2864	fig|6666666.59349.peg.1867	fig|6666666.59350.peg.5343	None	fig|6666666.59353.peg.447	fig|6666666.59355.peg.2174	fig|6666666.59354.peg.1953

2373	Succinylglutamate desuccinylase EC 3.5.1.96	fig|6666666.59329.peg.3348	fig|6666666.59330.peg.2658	None	fig|6666666.59332.peg.829	fig|6666666.59334.peg.573	fig|6666666.59335.peg.2172	fig|6666666.59336.peg.5584	fig|6666666.59333.peg.1781	None	fig|6666666.59339.peg.3912	None	fig|6666666.59337.peg.1985	fig|6666666.59342.peg.2063	fig|6666666.71181.peg.4525	fig|6666666.59344.peg.2604	fig|6666666.59345.peg.4677	fig|6666666.59341.peg.543	fig|6666666.59347.peg.3350	fig|6666666.59346.peg.2040	fig|6666666.59348.peg.3246	fig|6666666.59349.peg.1809	None	fig|6666666.59351.peg.1108	fig|6666666.59353.peg.1561	fig|6666666.59355.peg.2110	fig|6666666.59354.peg.4947

1877	Ubiquinol--cytochrome c reductase, cytochrome B subunit EC 1.10.2.2	fig|6666666.59329.peg.2056	fig|6666666.59330.peg.964	fig|6666666.59331.peg.790	fig|6666666.59332.peg.647	fig|6666666.59334.peg.2338	fig|6666666.59335.peg.3433	fig|6666666.59336.peg.1591	fig|6666666.59333.peg.617	fig|6666666.59338.peg.4707	fig|6666666.59339.peg.4944	fig|6666666.59340.peg.522	fig|6666666.59337.peg.30	fig|6666666.59342.peg.4825	None	None	fig|6666666.59345.peg.3812	fig|6666666.59341.peg.3546	fig|6666666.59347.peg.3925	fig|6666666.59346.peg.2335	fig|6666666.59348.peg.1446	fig|6666666.59349.peg.42	fig|6666666.59350.peg.2358	fig|6666666.59351.peg.3463	None	None	fig|6666666.59354.peg.360

1871	Outer membrane lipoprotein LolB precursor	fig|6666666.59329.peg.5527	fig|6666666.59330.peg.4667	fig|6666666.59331.peg.5278	None	fig|6666666.59334.peg.2241	fig|6666666.59335.peg.1176	fig|6666666.59336.peg.5360	fig|6666666.59333.peg.234	fig|6666666.59338.peg.5374	fig|6666666.59339.peg.7	None	fig|6666666.59337.peg.5165	None	fig|6666666.71181.peg.781	fig|6666666.59344.peg.106	fig|6666666.59345.peg.5414	fig|6666666.59341.peg.3042	fig|6666666.59347.peg.5281	fig|6666666.59346.peg.3189	fig|6666666.59348.peg.611	fig|6666666.59349.peg.2613	None	fig|6666666.59351.peg.4664	fig|6666666.59353.peg.430	fig|6666666.59355.peg.2156	fig|6666666.59354.peg.5213

9824	Flagellar biosynthesis protein FliR	fig|6666666.59329.peg.196	fig|6666666.59330.peg.2231	None	None	None	fig|6666666.59335.peg.5317	fig|6666666.59336.peg.1277	fig|6666666.59333.peg.1679	fig|6666666.59338.peg.1765	fig|6666666.59339.peg.107	fig|6666666.59340.peg.1874	fig|6666666.59337.peg.2971	fig|6666666.59342.peg.4508	fig|6666666.71181.peg.5561	fig|6666666.59344.peg.3048	fig|6666666.59345.peg.5331	fig|6666666.59341.peg.134	fig|6666666.59347.peg.4620	fig|6666666.59346.peg.2892	None	fig|6666666.59349.peg.199	fig|6666666.59350.peg.4400	fig|6666666.59351.peg.2058	fig|6666666.59353.peg.3515	fig|6666666.59355.peg.3661	fig|6666666.59354.peg.678

9823	Hypothetical protein YqcC clustered with tRNA pseudouridine synthase C	fig|6666666.59329.peg.3893	fig|6666666.59330.peg.474	None	fig|6666666.59332.peg.983	fig|6666666.59334.peg.1034	fig|6666666.59335.peg.3902	fig|6666666.59336.peg.4977	fig|6666666.59333.peg.2235	fig|6666666.59338.peg.3397	fig|6666666.59339.peg.7178	None	fig|6666666.59337.peg.1575	None	fig|6666666.71181.peg.44	fig|6666666.59344.peg.2635	fig|6666666.59345.peg.2207	fig|6666666.59341.peg.2609	fig|6666666.59347.peg.2773	fig|6666666.59346.peg.5003	fig|6666666.59348.peg.5198	fig|6666666.59349.peg.1352	None	fig|6666666.59351.peg.5487	fig|6666666.59353.peg.1973	fig|6666666.59355.peg.1434	fig|6666666.59354.peg.2625

9229	ADP-L-glycero-D-manno-heptose-6-epimerase EC 5.1.3.20	fig|6666666.59329.peg.830	fig|6666666.59330.peg.1354	fig|6666666.59331.peg.3328	fig|6666666.59332.peg.1728	fig|6666666.59334.peg.620	fig|6666666.59335.peg.499	fig|6666666.59336.peg.78	fig|6666666.59333.peg.474	fig|6666666.59338.peg.1221	fig|6666666.59339.peg.6315	fig|6666666.59340.peg.180	fig|6666666.59337.peg.444	fig|6666666.59342.peg.5449	fig|6666666.71181.peg.3671	fig|6666666.59344.peg.961	fig|6666666.59345.peg.2592	fig|6666666.59341.peg.3705	fig|6666666.59347.peg.5175	fig|6666666.59346.peg.4802	fig|6666666.59348.peg.1047	None	None	None	fig|6666666.59353.peg.2575	fig|6666666.59355.peg.2626	None

10021	Uncharacterized protein ImpB	fig|6666666.59329.peg.5785	fig|6666666.59330.peg.3111	fig|6666666.59331.peg.4414	fig|6666666.59332.peg.1092	None	fig|6666666.59335.peg.2687	fig|6666666.59336.peg.3825	fig|6666666.59333.peg.5465	fig|6666666.59338.peg.5253	fig|6666666.59339.peg.2718	fig|6666666.59340.peg.3235	fig|6666666.59337.peg.5015	fig|6666666.59342.peg.1248	fig|6666666.71181.peg.4825	fig|6666666.59344.peg.2986	fig|6666666.59345.peg.3374	fig|6666666.59341.peg.5190	fig|6666666.59347.peg.1031	None	fig|6666666.59348.peg.4634	fig|6666666.59349.peg.1632	None	None	fig|6666666.59353.peg.3565	fig|6666666.59355.peg.3608	fig|6666666.59354.peg.401

7638	SSU ribosomal protein S5p S2e	fig|6666666.59329.peg.726	fig|6666666.59330.peg.567	fig|6666666.59331.peg.35	fig|6666666.59332.peg.2152	fig|6666666.59334.peg.4019	fig|6666666.59335.peg.4360	fig|6666666.59336.peg.159	fig|6666666.59333.peg.379	fig|6666666.59338.peg.4969	fig|6666666.59339.peg.8280	None	fig|6666666.59337.peg.375	fig|6666666.59342.peg.317	fig|6666666.71181.peg.3445	fig|6666666.59344.peg.4928	fig|6666666.59345.peg.2522	fig|6666666.59341.peg.2511	fig|6666666.59347.peg.4836	fig|6666666.59346.peg.863	None	fig|6666666.59349.peg.3283	None	fig|6666666.59351.peg.4217	fig|6666666.59353.peg.4605	fig|6666666.59355.peg.4726	None

7437	Mlr7403 protein	fig|6666666.59329.peg.304	fig|6666666.59330.peg.1428	None	fig|6666666.59332.peg.5278	fig|6666666.59334.peg.1256	fig|6666666.59335.peg.1107	None	fig|6666666.59333.peg.938	fig|6666666.59338.peg.2290	fig|6666666.59339.peg.6972	None	fig|6666666.59337.peg.754	fig|6666666.59342.peg.1064	fig|6666666.71181.peg.1772	fig|6666666.59344.peg.5136	fig|6666666.59345.peg.3639	fig|6666666.59341.peg.3034	fig|6666666.59347.peg.4298	fig|6666666.59346.peg.1316	fig|6666666.59348.peg.3131	fig|6666666.59349.peg.3084	None	fig|6666666.59351.peg.3077	fig|6666666.59353.peg.2711	fig|6666666.59355.peg.1769	fig|6666666.59354.peg.87

10402	FxsA protein	None	fig|6666666.59330.peg.174	None	fig|6666666.59332.peg.154	fig|6666666.59334.peg.1156	fig|6666666.59335.peg.473	fig|6666666.59336.peg.2314	fig|6666666.59333.peg.412	fig|6666666.59338.peg.2356	fig|6666666.59339.peg.6493	fig|6666666.59340.peg.666	fig|6666666.59337.peg.4344	None	fig|6666666.71181.peg.1676	None	fig|6666666.59345.peg.5126	fig|6666666.59341.peg.5105	fig|6666666.59347.peg.4512	fig|6666666.59346.peg.787	fig|6666666.59348.peg.398	fig|6666666.59349.peg.2366	fig|6666666.59350.peg.2704	fig|6666666.59351.peg.111	fig|6666666.59353.peg.1046	fig|6666666.59355.peg.3573	fig|6666666.59354.peg.3259

9869	putative exported protein	fig|6666666.59329.peg.2630	fig|6666666.59330.peg.2059	fig|6666666.59331.peg.6381	fig|6666666.59332.peg.4788	fig|6666666.59334.peg.363	fig|6666666.59335.peg.3411	fig|6666666.59336.peg.6512	fig|6666666.59333.peg.3917	fig|6666666.59338.peg.4473	fig|6666666.59339.peg.1735	fig|6666666.59340.peg.2461	fig|6666666.59337.peg.1201	fig|6666666.59342.peg.5675	None	None	fig|6666666.59345.peg.3925	fig|6666666.59341.peg.1222	fig|6666666.59347.peg.3633	fig|6666666.59346.peg.352	fig|6666666.59348.peg.4299	fig|6666666.59349.peg.3265	fig|6666666.59350.peg.4824	fig|6666666.59351.peg.3116	None	None	fig|6666666.59354.peg.5852

265	putative transport protein	fig|6666666.59329.peg.3728	fig|6666666.59330.peg.4285	None	fig|6666666.59332.peg.709	fig|6666666.59334.peg.4547	fig|6666666.59335.peg.2305	fig|6666666.59336.peg.6397	None	fig|6666666.59338.peg.2446	fig|6666666.59339.peg.869	None	fig|6666666.59337.peg.4030	None	fig|6666666.71181.peg.202	fig|6666666.59344.peg.2542	fig|6666666.59345.peg.203	fig|6666666.59341.peg.4328	fig|6666666.59347.peg.890	fig|6666666.59346.peg.2641	fig|6666666.59348.peg.4432	fig|6666666.59349.peg.1490	fig|6666666.59350.peg.12	fig|6666666.59351.peg.3229	fig|6666666.59353.peg.1510	fig|6666666.59355.peg.103	fig|6666666.59354.peg.205

9083	Organic hydroperoxide resistance protein	fig|6666666.59329.peg.6538	fig|6666666.59330.peg.4467	fig|6666666.59331.peg.2618	fig|6666666.59332.peg.5100	None	fig|6666666.59335.peg.2452	None	fig|6666666.59333.peg.4672	fig|6666666.59338.peg.1933	fig|6666666.59339.peg.3550	None	fig|6666666.59337.peg.2944	fig|6666666.59342.peg.5118	fig|6666666.71181.peg.2468	fig|6666666.59344.peg.5290	fig|6666666.59345.peg.2063	fig|6666666.59341.peg.6017	fig|6666666.59347.peg.348	fig|6666666.59346.peg.3616	fig|6666666.59348.peg.1103	fig|6666666.59349.peg.2255	fig|6666666.59350.peg.850	None	fig|6666666.59353.peg.3612	fig|6666666.59355.peg.4797	fig|6666666.59354.peg.3656

1380	Bacterioferritin	fig|6666666.59329.peg.4603	fig|6666666.59330.peg.1669	fig|6666666.59331.peg.5763	fig|6666666.59332.peg.3792	None	fig|6666666.59335.peg.3494	fig|6666666.59336.peg.3889	fig|6666666.59333.peg.3018	fig|6666666.59338.peg.1396	fig|6666666.59339.peg.7694	fig|6666666.59340.peg.2010	fig|6666666.59337.peg.1672	fig|6666666.59342.peg.5306	fig|6666666.71181.peg.3462	fig|6666666.59344.peg.4945	fig|6666666.59345.peg.5408	fig|6666666.59341.peg.2491	None	fig|6666666.59346.peg.5023	None	fig|6666666.59349.peg.4533	None	fig|6666666.59351.peg.3806	fig|6666666.59353.peg.4588	fig|6666666.59355.peg.4743	fig|6666666.59354.peg.6277

6207	Permease of the major facilitator superfamily	None	fig|6666666.59330.peg.759	fig|6666666.59331.peg.250	None	None	fig|6666666.59335.peg.4993	fig|6666666.59336.peg.4193	fig|6666666.59333.peg.3914	fig|6666666.59338.peg.3980	fig|6666666.59339.peg.4698	None	fig|6666666.59337.peg.1198	fig|6666666.59342.peg.1442	fig|6666666.71181.peg.3158	fig|6666666.59344.peg.2172	fig|6666666.59345.peg.18	fig|6666666.59341.peg.1227	fig|6666666.59347.peg.3629	fig|6666666.59346.peg.4508	fig|6666666.59348.peg.4511	fig|6666666.59349.peg.4175	fig|6666666.59350.peg.8167	fig|6666666.59351.peg.2435	fig|6666666.59353.peg.227	fig|6666666.59355.peg.2998	fig|6666666.59354.peg.3767

8832	Molybdenum cofactor biosynthesis protein MoaA	fig|6666666.59329.peg.5054	fig|6666666.59330.peg.1709	fig|6666666.59331.peg.5275	fig|6666666.59332.peg.1521	fig|6666666.59334.peg.296	fig|6666666.59335.peg.5489	fig|6666666.59336.peg.2031	fig|6666666.59333.peg.1332	fig|6666666.59338.peg.2312	fig|6666666.59339.peg.139	None	fig|6666666.59337.peg.4024	fig|6666666.59342.peg.871	fig|6666666.71181.peg.2531	fig|6666666.59344.peg.4099	fig|6666666.59345.peg.1113	fig|6666666.59341.peg.2167	fig|6666666.59347.peg.5157	fig|6666666.59346.peg.532	fig|6666666.59348.peg.716	None	fig|6666666.59350.peg.1920	None	fig|6666666.59353.peg.2141	fig|6666666.59355.peg.2679	None

1280	Uroporphyrinogen-III methyltransferase EC 2.1.1.107	fig|6666666.59329.peg.5177	fig|6666666.59330.peg.3044	None	fig|6666666.59332.peg.1712	None	fig|6666666.59335.peg.3327	fig|6666666.59336.peg.1124	fig|6666666.59333.peg.782	fig|6666666.59338.peg.4275	fig|6666666.59339.peg.8512	fig|6666666.59340.peg.847	fig|6666666.59337.peg.1611	None	fig|6666666.71181.peg.2438	fig|6666666.59344.peg.2479	fig|6666666.59345.peg.5510	fig|6666666.59341.peg.5810	fig|6666666.59347.peg.5083	fig|6666666.59346.peg.593	fig|6666666.59348.peg.2175	fig|6666666.59349.peg.1564	None	fig|6666666.59351.peg.4758	fig|6666666.59353.peg.3637	fig|6666666.59355.peg.4007	fig|6666666.59354.peg.1464

682	Glycogen phosphorylase EC 2.4.1.1	None	fig|6666666.59330.peg.3469	fig|6666666.59331.peg.5769	fig|6666666.59332.peg.4185	fig|6666666.59334.peg.2530	fig|6666666.59335.peg.4405	fig|6666666.59336.peg.803	fig|6666666.59333.peg.4752	fig|6666666.59338.peg.1081	fig|6666666.59339.peg.1716	None	fig|6666666.59337.peg.4348	fig|6666666.59342.peg.1908	fig|6666666.71181.peg.1117	fig|6666666.59344.peg.3634	fig|6666666.59345.peg.1916	fig|6666666.59341.peg.2598	fig|6666666.59347.peg.149	fig|6666666.59346.peg.1331	None	fig|6666666.59349.peg.960	None	fig|6666666.59351.peg.1586	fig|6666666.59353.peg.2176	fig|6666666.59355.peg.1878	fig|6666666.59354.peg.6283

2153	Branched-chain amino acid transport system carrier protein	fig|6666666.59329.peg.1680	fig|6666666.59330.peg.2405	fig|6666666.59331.peg.5600	fig|6666666.59332.peg.2004	fig|6666666.59334.peg.2817	fig|6666666.59335.peg.6095	fig|6666666.59336.peg.2435	fig|6666666.59333.peg.4879	fig|6666666.59338.peg.203	fig|6666666.59339.peg.4876	fig|6666666.59340.peg.380	fig|6666666.59337.peg.105	fig|6666666.59342.peg.6414	fig|6666666.71181.peg.2743	fig|6666666.59344.peg.6292	fig|6666666.59345.peg.3736	fig|6666666.59341.peg.3453	fig|6666666.59347.peg.3048	fig|6666666.59346.peg.3096	fig|6666666.59348.peg.2946	None	None	None	fig|6666666.59353.peg.4414	fig|6666666.59355.peg.4495	None

4285	Cytosine deaminase EC 3.5.4.1	fig|6666666.59329.peg.4929	fig|6666666.59330.peg.855	fig|6666666.59331.peg.5460	None	fig|6666666.59334.peg.3758	fig|6666666.59335.peg.3406	fig|6666666.59336.peg.3850	fig|6666666.59333.peg.68	fig|6666666.59338.peg.3775	fig|6666666.59339.peg.4324	None	fig|6666666.59337.peg.3274	fig|6666666.59342.peg.6368	fig|6666666.71181.peg.1821	fig|6666666.59344.peg.3077	fig|6666666.59345.peg.538	fig|6666666.59341.peg.1300	fig|6666666.59347.peg.1566	fig|6666666.59346.peg.4053	fig|6666666.59348.peg.2540	fig|6666666.59349.peg.4998	fig|6666666.59350.peg.2723	None	fig|6666666.59353.peg.2229	fig|6666666.59355.peg.1923	None

4753	D-alanyl-D-alanine carboxypeptidase EC 3.4.16.4	fig|6666666.59329.peg.1596	fig|6666666.59330.peg.119	fig|6666666.59331.peg.3894	fig|6666666.59332.peg.6237	fig|6666666.59334.peg.198	fig|6666666.59335.peg.2840	fig|6666666.59336.peg.232	fig|6666666.59333.peg.1484	fig|6666666.59338.peg.6026	fig|6666666.59339.peg.4851	fig|6666666.59340.peg.3547	fig|6666666.59337.peg.672	fig|6666666.59342.peg.2393	fig|6666666.71181.peg.4088	fig|6666666.59344.peg.2110	fig|6666666.59345.peg.2367	None	fig|6666666.59347.peg.3014	fig|6666666.59346.peg.286	fig|6666666.59348.peg.4787	None	fig|6666666.59350.peg.2491	None	fig|6666666.59353.peg.4063	fig|6666666.59355.peg.452	None

11295	Ribonuclease D EC 3.1.26.3	fig|6666666.59329.peg.3414	fig|6666666.59330.peg.4011	None	fig|6666666.59332.peg.946	fig|6666666.59334.peg.5483	fig|6666666.59335.peg.2038	fig|6666666.59336.peg.1025	fig|6666666.59333.peg.1355	fig|6666666.59338.peg.1134	fig|6666666.59339.peg.3160	fig|6666666.59340.peg.2189	fig|6666666.59337.peg.2749	None	fig|6666666.71181.peg.5197	fig|6666666.59344.peg.2854	fig|6666666.59345.peg.4298	fig|6666666.59341.peg.3856	fig|6666666.59347.peg.4412	fig|6666666.59346.peg.1476	None	fig|6666666.59349.peg.472	fig|6666666.59350.peg.1980	fig|6666666.59351.peg.1643	fig|6666666.59353.peg.1465	fig|6666666.59355.peg.2016	None

2197	Predicted esterase of the alpha-beta hydrolase superfamily	fig|6666666.59329.peg.2888	fig|6666666.59330.peg.2240	fig|6666666.59331.peg.6324	fig|6666666.59332.peg.5346	fig|6666666.59334.peg.448	fig|6666666.59335.peg.4737	fig|6666666.59336.peg.6433	fig|6666666.59333.peg.2626	fig|6666666.59338.peg.506	fig|6666666.59339.peg.3191	None	fig|6666666.59337.peg.2140	None	fig|6666666.71181.peg.462	fig|6666666.59344.peg.1380	fig|6666666.59345.peg.1049	fig|6666666.59341.peg.3316	fig|6666666.59347.peg.4160	fig|6666666.59346.peg.5048	fig|6666666.59348.peg.2021	fig|6666666.59349.peg.366	None	None	fig|6666666.59353.peg.34	fig|6666666.59355.peg.641	fig|6666666.59354.peg.4430

2199	Fumarate hydratase class I, aerobic EC 4.2.1.2	fig|6666666.59329.peg.6372	fig|6666666.59330.peg.2082	fig|6666666.59331.peg.6260	None	fig|6666666.59334.peg.2323	fig|6666666.59335.peg.5970	fig|6666666.59336.peg.4803	fig|6666666.59333.peg.3217	fig|6666666.59338.peg.4259	fig|6666666.59339.peg.3715	None	fig|6666666.59337.peg.4680	None	fig|6666666.71181.peg.1857	fig|6666666.59344.peg.4331	fig|6666666.59345.peg.824	fig|6666666.59341.peg.5712	fig|6666666.59347.peg.2144	fig|6666666.59346.peg.3846	fig|6666666.59348.peg.5238	fig|6666666.59349.peg.2936	None	fig|6666666.59351.peg.2736	fig|6666666.59353.peg.3222	fig|6666666.59355.peg.3104	fig|6666666.59354.peg.3195

9768	Protein of unknown function DUF55	fig|6666666.59329.peg.80	fig|6666666.59330.peg.1542	fig|6666666.59331.peg.826	fig|6666666.59332.peg.475	None	fig|6666666.59335.peg.4118	fig|6666666.59336.peg.622	fig|6666666.59333.peg.798	fig|6666666.59338.peg.1997	fig|6666666.59339.peg.8539	fig|6666666.59340.peg.1994	fig|6666666.59337.peg.1625	fig|6666666.59342.peg.5621	None	None	fig|6666666.59345.peg.5492	fig|6666666.59341.peg.3298	fig|6666666.59347.peg.5069	fig|6666666.59346.peg.611	fig|6666666.59348.peg.746	fig|6666666.59349.peg.2274	fig|6666666.59350.peg.1755	fig|6666666.59351.peg.4824	None	fig|6666666.59355.peg.5450	fig|6666666.59354.peg.1840

5276	Antiholin-like protein LrgA	fig|6666666.59329.peg.6478	fig|6666666.59330.peg.2672	fig|6666666.59331.peg.4024	fig|6666666.59332.peg.3200	fig|6666666.59334.peg.5295	fig|6666666.59335.peg.5724	fig|6666666.59336.peg.1689	fig|6666666.59333.peg.543	fig|6666666.59338.peg.5879	fig|6666666.59339.peg.3895	None	fig|6666666.59337.peg.4530	fig|6666666.59342.peg.393	fig|6666666.71181.peg.201	fig|6666666.59344.peg.245	fig|6666666.59345.peg.4666	fig|6666666.59341.peg.1276	fig|6666666.59347.peg.1638	fig|6666666.59346.peg.4403	None	fig|6666666.59349.peg.2068	None	None	fig|6666666.59353.peg.621	fig|6666666.59355.peg.2870	fig|6666666.59354.peg.1396

10318	Flagellar hook-associated protein FliD	fig|6666666.59329.peg.2522	fig|6666666.59330.peg.2905	fig|6666666.59331.peg.2106	None	None	fig|6666666.59335.peg.919	fig|6666666.59336.peg.991	fig|6666666.59333.peg.4082	fig|6666666.59338.peg.2382	fig|6666666.59339.peg.97	fig|6666666.59340.peg.3154	fig|6666666.59337.peg.3216	fig|6666666.59342.peg.5249	fig|6666666.71181.peg.4035	fig|6666666.59344.peg.5211	fig|6666666.59345.peg.2874	fig|6666666.59341.peg.675	fig|6666666.59347.peg.4615	None	fig|6666666.59348.peg.2817	fig|6666666.59349.peg.741	None	fig|6666666.59351.peg.1998	fig|6666666.59353.peg.4570	fig|6666666.59355.peg.3834	fig|6666666.59354.peg.4130

8703	2-keto-3-deoxy-D-arabino-heptulosonate-7-phosphate synthase I alpha EC 2.5.1.54	fig|6666666.59329.peg.3886	fig|6666666.59330.peg.1123	fig|6666666.59331.peg.4197	fig|6666666.59332.peg.2640	fig|6666666.59334.peg.3586	fig|6666666.59335.peg.2053	fig|6666666.59336.peg.6273	fig|6666666.59333.peg.3355	fig|6666666.59338.peg.881	fig|6666666.59339.peg.1553	fig|6666666.59340.peg.2691	fig|6666666.59337.peg.5076	fig|6666666.59342.peg.526	fig|6666666.71181.peg.2501	fig|6666666.59344.peg.5434	fig|6666666.59345.peg.3696	fig|6666666.59341.peg.105	fig|6666666.59347.peg.994	fig|6666666.59346.peg.3921	fig|6666666.59348.peg.4596	None	None	None	fig|6666666.59353.peg.4806	fig|6666666.59355.peg.2711	None

223	Orotidine 5'-phosphate decarboxylase EC 4.1.1.23	fig|6666666.59329.peg.2773	fig|6666666.59330.peg.1383	None	fig|6666666.59332.peg.2854	fig|6666666.59334.peg.1072	fig|6666666.59335.peg.3373	fig|6666666.59336.peg.426	fig|6666666.59333.peg.740	None	fig|6666666.59339.peg.3115	None	fig|6666666.59337.peg.2881	fig|6666666.59342.peg.1801	fig|6666666.71181.peg.4199	fig|6666666.59344.peg.492	fig|6666666.59345.peg.1032	fig|6666666.59341.peg.51	fig|6666666.59347.peg.2403	fig|6666666.59346.peg.5063	fig|6666666.59348.peg.836	fig|6666666.59349.peg.373	fig|6666666.59350.peg.3116	fig|6666666.59351.peg.946	fig|6666666.59353.peg.1808	fig|6666666.59355.peg.1208	None

9515	Phosphohistidine phosphatase SixA	fig|6666666.59329.peg.6418	fig|6666666.59330.peg.1583	fig|6666666.59331.peg.5104	fig|6666666.59332.peg.4595	fig|6666666.59334.peg.1702	fig|6666666.59335.peg.5965	fig|6666666.59336.peg.3322	fig|6666666.59333.peg.4010	None	fig|6666666.59339.peg.3003	None	None	fig|6666666.59342.peg.2110	fig|6666666.71181.peg.1514	fig|6666666.59344.peg.4998	fig|6666666.59345.peg.5297	fig|6666666.59341.peg.846	fig|6666666.59347.peg.5109	fig|6666666.59346.peg.3411	fig|6666666.59348.peg.1780	fig|6666666.59349.peg.3947	None	fig|6666666.59351.peg.414	fig|6666666.59353.peg.5021	fig|6666666.59355.peg.227	fig|6666666.59354.peg.4564

4610	Lipoprotein nlpI precursor	fig|6666666.59329.peg.4208	fig|6666666.59330.peg.101	None	fig|6666666.59332.peg.4052	fig|6666666.59334.peg.772	fig|6666666.59335.peg.1034	fig|6666666.59336.peg.4092	fig|6666666.59333.peg.2319	fig|6666666.59338.peg.5943	fig|6666666.59339.peg.7429	None	fig|6666666.59337.peg.689	None	fig|6666666.71181.peg.4793	fig|6666666.59344.peg.2093	fig|6666666.59345.peg.2351	fig|6666666.59341.peg.2445	fig|6666666.59347.peg.2999	fig|6666666.59346.peg.1981	fig|6666666.59348.peg.3716	fig|6666666.59349.peg.3900	None	fig|6666666.59351.peg.1384	fig|6666666.59353.peg.3127	fig|6666666.59355.peg.469	fig|6666666.59354.peg.6491

3908	Phosphatidylglycerophosphatase A EC 3.1.3.27	fig|6666666.59329.peg.438	fig|6666666.59330.peg.1054	None	fig|6666666.59332.peg.4205	fig|6666666.59334.peg.2076	fig|6666666.59335.peg.157	fig|6666666.59336.peg.3170	fig|6666666.59333.peg.5216	fig|6666666.59338.peg.2804	fig|6666666.59339.peg.5099	None	fig|6666666.59337.peg.1134	None	fig|6666666.71181.peg.2724	fig|6666666.59344.peg.1095	fig|6666666.59345.peg.3536	fig|6666666.59341.peg.2188	fig|6666666.59347.peg.2764	fig|6666666.59346.peg.3386	fig|6666666.59348.peg.929	fig|6666666.59349.peg.3487	fig|6666666.59350.peg.4990	fig|6666666.59351.peg.4245	fig|6666666.59353.peg.774	fig|6666666.59355.peg.929	None

208	Inosine xanthosine triphosphatase EC 3.6.1.- ; Hypothetical cytoplasmic protein in cluster with NspS	fig|6666666.59329.peg.2737	fig|6666666.59330.peg.2889	fig|6666666.59331.peg.933	fig|6666666.59332.peg.1326	None	fig|6666666.59335.peg.2061	fig|6666666.59336.peg.843	fig|6666666.59333.peg.4106	None	fig|6666666.59339.peg.7502	fig|6666666.59340.peg.699	fig|6666666.59337.peg.153	None	fig|6666666.71181.peg.442	fig|6666666.59344.peg.1349	fig|6666666.59345.peg.5677	fig|6666666.59341.peg.3008	fig|6666666.59347.peg.4051	fig|6666666.59346.peg.261	fig|6666666.59348.peg.2856	fig|6666666.59349.peg.2126	None	fig|6666666.59351.peg.144	fig|6666666.59353.peg.56	fig|6666666.59355.peg.663	fig|6666666.59354.peg.4717

5068	5-nucleotidase SurE EC 3.1.3.5 Exopolyphosphatase EC 3.6.1.11	fig|6666666.59329.peg.4462	fig|6666666.59330.peg.2459	None	fig|6666666.59332.peg.202	fig|6666666.59334.peg.271	fig|6666666.59335.peg.1659	fig|6666666.59336.peg.4462	fig|6666666.59333.peg.1712	fig|6666666.59338.peg.220	fig|6666666.59339.peg.7920	None	fig|6666666.59337.peg.582	fig|6666666.59342.peg.1272	fig|6666666.71181.peg.3419	fig|6666666.59344.peg.2194	fig|6666666.59345.peg.5072	None	fig|6666666.59347.peg.3952	fig|6666666.59346.peg.3145	None	fig|6666666.59349.peg.4384	fig|6666666.59350.peg.1445	fig|6666666.59351.peg.5475	fig|6666666.59353.peg.212	fig|6666666.59355.peg.2981	fig|6666666.59354.peg.3144

402	Peptidyl-prolyl cis-trans isomerase EC 5.2.1.8	fig|6666666.59329.peg.670	fig|6666666.59330.peg.3776	fig|6666666.59331.peg.5198	fig|6666666.59332.peg.130	fig|6666666.59334.peg.1581	fig|6666666.59335.peg.5705	fig|6666666.59336.peg.3382	fig|6666666.59333.peg.2193	fig|6666666.59338.peg.3370	fig|6666666.59339.peg.6429	fig|6666666.59340.peg.1214	fig|6666666.59337.peg.4505	fig|6666666.59342.peg.4595	fig|6666666.71181.peg.3350	fig|6666666.59344.peg.5678	fig|6666666.59345.peg.5694	fig|6666666.59341.peg.1425	fig|6666666.59347.peg.4484	fig|6666666.59346.peg.1143	fig|6666666.59348.peg.3310	None	None	None	fig|6666666.59353.peg.4472	fig|6666666.59355.peg.4554	None

5643	Phage integrase	fig|6666666.59329.peg.5453	fig|6666666.59330.peg.2415	fig|6666666.59331.peg.2194	fig|6666666.59332.peg.376	None	fig|6666666.59335.peg.4166	None	fig|6666666.59333.peg.547	fig|6666666.59338.peg.729	fig|6666666.59339.peg.2518	None	fig|6666666.59337.peg.4197	fig|6666666.59342.peg.2888	fig|6666666.71181.peg.1670	fig|6666666.59344.peg.624	fig|6666666.59345.peg.5158	fig|6666666.59341.peg.1858	None	fig|6666666.59346.peg.697	fig|6666666.59348.peg.2370	fig|6666666.59349.peg.3043	fig|6666666.59350.peg.8388	fig|6666666.59351.peg.5149	fig|6666666.59353.peg.3753	fig|6666666.59355.peg.3770	fig|6666666.59354.peg.1765

10358	N-hydroxyarylamine O-acetyltransferase EC 2.3.1.118	fig|6666666.59329.peg.473	None	None	fig|6666666.59332.peg.2784	fig|6666666.59334.peg.3223	fig|6666666.59335.peg.5721	fig|6666666.59336.peg.4301	fig|6666666.59333.peg.3195	fig|6666666.59338.peg.4460	fig|6666666.59339.peg.2148	fig|6666666.59340.peg.2149	fig|6666666.59337.peg.4903	fig|6666666.59342.peg.705	fig|6666666.71181.peg.1199	fig|6666666.59344.peg.2329	fig|6666666.59345.peg.4484	fig|6666666.59341.peg.93	fig|6666666.59347.peg.4035	fig|6666666.59346.peg.4392	None	fig|6666666.59349.peg.1157	None	fig|6666666.59351.peg.2559	fig|6666666.59353.peg.3386	fig|6666666.59355.peg.3484	fig|6666666.59354.peg.2138

1065	Fructose-specific phosphocarrier protein HPr EC 2.7.1.69 PTS system, fructose-specific IIA component EC 2.7.1.69	fig|6666666.59329.peg.6092	fig|6666666.59330.peg.4754	fig|6666666.59331.peg.951	fig|6666666.59332.peg.2	fig|6666666.59334.peg.921	fig|6666666.59335.peg.541	fig|6666666.59336.peg.2729	fig|6666666.59333.peg.2786	fig|6666666.59338.peg.1407	fig|6666666.59339.peg.8424	fig|6666666.59340.peg.1493	fig|6666666.59337.peg.2481	fig|6666666.59342.peg.776	fig|6666666.71181.peg.662	fig|6666666.59344.peg.4127	fig|6666666.59345.peg.4045	fig|6666666.59341.peg.2826	fig|6666666.59347.peg.493	fig|6666666.59346.peg.714	fig|6666666.59348.peg.1788	None	None	None	fig|6666666.59353.peg.1191	fig|6666666.59355.peg.392	None

9120	Ribosomal RNA large subunit methyltransferase A EC 2.1.1.51	fig|6666666.59329.peg.876	fig|6666666.59330.peg.1221	None	fig|6666666.59332.peg.1346	fig|6666666.59334.peg.2899	fig|6666666.59335.peg.2953	fig|6666666.59336.peg.1810	fig|6666666.59333.peg.1090	fig|6666666.59338.peg.1953	fig|6666666.59339.peg.263	fig|6666666.59340.peg.2154	fig|6666666.59337.peg.3121	None	fig|6666666.71181.peg.4242	fig|6666666.59344.peg.2574	fig|6666666.59345.peg.1169	fig|6666666.59341.peg.5423	fig|6666666.59347.peg.2436	fig|6666666.59346.peg.73	None	fig|6666666.59349.peg.2949	None	fig|6666666.59351.peg.2824	fig|6666666.59353.peg.1536	fig|6666666.59355.peg.2085	fig|6666666.59354.peg.5603

3449	Phosphoribosylaminoimidazole carboxylase catalytic subunit EC 4.1.1.21	fig|6666666.59329.peg.74	fig|6666666.59330.peg.652	None	fig|6666666.59332.peg.5852	fig|6666666.59334.peg.1514	fig|6666666.59335.peg.5008	fig|6666666.59336.peg.1985	fig|6666666.59333.peg.1366	fig|6666666.59338.peg.2068	fig|6666666.59339.peg.8100	None	fig|6666666.59337.peg.3908	None	fig|6666666.71181.peg.3625	None	fig|6666666.59345.peg.4920	fig|6666666.59341.peg.5016	fig|6666666.59347.peg.4741	fig|6666666.59346.peg.1831	fig|6666666.59348.peg.3867	fig|6666666.59349.peg.1611	fig|6666666.59350.peg.3332	fig|6666666.59351.peg.1038	fig|6666666.59353.peg.3762	fig|6666666.59355.peg.3779	fig|6666666.59354.peg.1859

4053	Iron compound ABC uptake transporter substrate-binding protein PiuA	fig|6666666.59329.peg.3423	fig|6666666.59330.peg.4197	fig|6666666.59331.peg.1855	fig|6666666.59332.peg.4231	fig|6666666.59334.peg.4591	fig|6666666.59335.peg.2650	fig|6666666.59336.peg.1897	fig|6666666.59333.peg.3824	fig|6666666.59338.peg.4093	fig|6666666.59339.peg.1044	None	fig|6666666.59337.peg.4518	fig|6666666.59342.peg.5442	fig|6666666.71181.peg.5117	fig|6666666.59344.peg.2033	fig|6666666.59345.peg.740	fig|6666666.59341.peg.2373	fig|6666666.59347.peg.363	fig|6666666.59346.peg.1156	fig|6666666.59348.peg.4790	None	fig|6666666.59350.peg.6531	None	fig|6666666.59353.peg.2896	fig|6666666.59355.peg.2843	None

4442	Lactoylglutathione lyase	fig|6666666.59329.peg.1501	fig|6666666.59330.peg.4497	fig|6666666.59331.peg.213	fig|6666666.59332.peg.5957	fig|6666666.59334.peg.1753	fig|6666666.59335.peg.4054	fig|6666666.59336.peg.759	fig|6666666.59333.peg.5128	fig|6666666.59338.peg.1216	fig|6666666.59339.peg.5542	fig|6666666.59340.peg.1622	fig|6666666.59337.peg.1257	fig|6666666.59342.peg.3727	None	None	fig|6666666.59345.peg.585	fig|6666666.59341.peg.3223	fig|6666666.59347.peg.290	fig|6666666.59346.peg.4018	fig|6666666.59348.peg.4647	fig|6666666.59349.peg.3811	fig|6666666.59350.peg.1388	fig|6666666.59351.peg.3261	None	None	fig|6666666.59354.peg.341

304	TRAP transporter solute receptor, TAXI family precursor	fig|6666666.59329.peg.1480	fig|6666666.59330.peg.2403	fig|6666666.59331.peg.2598	fig|6666666.59332.peg.5201	fig|6666666.59334.peg.5100	fig|6666666.59335.peg.231	fig|6666666.59336.peg.2206	fig|6666666.59333.peg.2037	fig|6666666.59338.peg.4149	fig|6666666.59339.peg.8371	fig|6666666.59340.peg.1561	fig|6666666.59337.peg.1369	fig|6666666.59342.peg.3582	None	None	fig|6666666.59345.peg.2473	fig|6666666.59341.peg.2111	fig|6666666.59347.peg.3102	fig|6666666.59346.peg.490	fig|6666666.59348.peg.560	fig|6666666.59349.peg.1088	fig|6666666.59350.peg.809	None	None	fig|6666666.59355.peg.5397	fig|6666666.59354.peg.5487

7280	Cytochrome c-type protein NapC	fig|6666666.59329.peg.4424	fig|6666666.59330.peg.1525	fig|6666666.59331.peg.6284	fig|6666666.59332.peg.3683	fig|6666666.59334.peg.4551	fig|6666666.59335.peg.5112	fig|6666666.59336.peg.4806	fig|6666666.59333.peg.3616	fig|6666666.59338.peg.3076	fig|6666666.59339.peg.361	fig|6666666.59340.peg.1755	fig|6666666.59337.peg.2314	fig|6666666.59342.peg.963	fig|6666666.71181.peg.3179	fig|6666666.59344.peg.1669	fig|6666666.59345.peg.486	fig|6666666.59341.peg.5106	fig|6666666.59347.peg.2306	fig|6666666.59346.peg.4258	fig|6666666.59348.peg.2525	None	None	None	fig|6666666.59353.peg.1090	fig|6666666.59355.peg.390	None

1089	Universal stress protein A	fig|6666666.59329.peg.3802	fig|6666666.59330.peg.235	fig|6666666.59331.peg.5395	fig|6666666.59332.peg.1720	fig|6666666.59334.peg.1555	fig|6666666.59335.peg.3802	fig|6666666.59336.peg.5074	fig|6666666.59333.peg.1823	fig|6666666.59338.peg.409	fig|6666666.59339.peg.5523	fig|6666666.59340.peg.160	fig|6666666.59337.peg.2159	fig|6666666.59342.peg.5142	fig|6666666.71181.peg.831	fig|6666666.59344.peg.47	fig|6666666.59345.peg.2724	fig|6666666.59341.peg.4151	fig|6666666.59347.peg.4572	fig|6666666.59346.peg.3445	fig|6666666.59348.peg.2084	None	None	None	fig|6666666.59353.peg.383	fig|6666666.59355.peg.4619	None

8345	Maltose-6'-phosphate glucosidase EC 3.2.1.122	fig|6666666.59329.peg.3179	fig|6666666.59330.peg.2435	fig|6666666.59331.peg.2572	fig|6666666.59332.peg.5834	fig|6666666.59334.peg.601	fig|6666666.59335.peg.3266	fig|6666666.59336.peg.3061	fig|6666666.59333.peg.3569	fig|6666666.59338.peg.298	fig|6666666.59339.peg.6833	fig|6666666.59340.peg.3631	fig|6666666.59337.peg.4178	fig|6666666.59342.peg.2115	fig|6666666.71181.peg.5070	fig|6666666.59344.peg.133	fig|6666666.59345.peg.2140	fig|6666666.59341.peg.3887	fig|6666666.59347.peg.4577	None	fig|6666666.59348.peg.3191	None	fig|6666666.59350.peg.4795	None	fig|6666666.59353.peg.2777	fig|6666666.59355.peg.2177	None

11400	UPF0234 protein YajQ	fig|6666666.59329.peg.5984	fig|6666666.59330.peg.1027	None	fig|6666666.59332.peg.5007	fig|6666666.59334.peg.3584	fig|6666666.59335.peg.3585	fig|6666666.59336.peg.6271	fig|6666666.59333.peg.4846	fig|6666666.59338.peg.4718	fig|6666666.59339.peg.1554	None	fig|6666666.59337.peg.3169	fig|6666666.59342.peg.1629	fig|6666666.71181.peg.2038	fig|6666666.59344.peg.1084	fig|6666666.59345.peg.3272	fig|6666666.59341.peg.4213	fig|6666666.59347.peg.1788	fig|6666666.59346.peg.3922	None	fig|6666666.59349.peg.1768	None	fig|6666666.59351.peg.2296	fig|6666666.59353.peg.766	fig|6666666.59355.peg.938	fig|6666666.59354.peg.3477

2247	Flagellar motor switch protein FliM	fig|6666666.59329.peg.191	fig|6666666.59330.peg.1875	None	fig|6666666.59332.peg.4084	None	fig|6666666.59335.peg.396	fig|6666666.59336.peg.1271	fig|6666666.59333.peg.4567	fig|6666666.59338.peg.3100	fig|6666666.59339.peg.7088	None	fig|6666666.59337.peg.2966	fig|6666666.59342.peg.2651	fig|6666666.71181.peg.4936	fig|6666666.59344.peg.3054	fig|6666666.59345.peg.5336	fig|6666666.59341.peg.3273	fig|6666666.59347.peg.2684	fig|6666666.59346.peg.2886	fig|6666666.59348.peg.5274	fig|6666666.59349.peg.204	None	fig|6666666.59351.peg.2053	fig|6666666.59353.peg.3510	fig|6666666.59355.peg.3666	fig|6666666.59354.peg.671

2240	hypothetical protein	fig|6666666.59329.peg.2627	fig|6666666.59330.peg.2063	fig|6666666.59331.peg.5213	fig|6666666.59332.peg.4790	fig|6666666.59334.peg.361	fig|6666666.59335.peg.4257	fig|6666666.59336.peg.6499	fig|6666666.59333.peg.3918	fig|6666666.59338.peg.3470	fig|6666666.59339.peg.5631	fig|6666666.59340.peg.3772	fig|6666666.59337.peg.2535	fig|6666666.59342.peg.3924	None	None	fig|6666666.59345.peg.22	fig|6666666.59341.peg.1220	fig|6666666.59347.peg.2607	fig|6666666.59346.peg.351	fig|6666666.59348.peg.3146	fig|6666666.59349.peg.3264	fig|6666666.59350.peg.2633	fig|6666666.59351.peg.3266	None	None	fig|6666666.59354.peg.4957

4877	Holo-[acyl-carrier protein] synthase EC 2.7.8.7	fig|6666666.59329.peg.2799	fig|6666666.59330.peg.390	fig|6666666.59331.peg.6198	fig|6666666.59332.peg.191	fig|6666666.59334.peg.322	fig|6666666.59335.peg.1495	fig|6666666.59336.peg.5323	fig|6666666.59333.peg.4157	fig|6666666.59338.peg.4044	fig|6666666.59339.peg.7376	fig|6666666.59340.peg.1440	fig|6666666.59337.peg.569	None	fig|6666666.71181.peg.1419	fig|6666666.59344.peg.1570	fig|6666666.59345.peg.5060	fig|6666666.59341.peg.1329	fig|6666666.59347.peg.3172	fig|6666666.59346.peg.339	fig|6666666.59348.peg.645	None	fig|6666666.59350.peg.8398	None	fig|6666666.59353.peg.2657	fig|6666666.59355.peg.1825	None

5455	FIG001826 putative inner membrane protein	fig|6666666.59329.peg.136	fig|6666666.59330.peg.822	fig|6666666.59331.peg.1356	fig|6666666.59332.peg.3915	fig|6666666.59334.peg.3956	fig|6666666.59335.peg.4485	fig|6666666.59336.peg.2658	fig|6666666.59333.peg.5029	fig|6666666.59338.peg.914	fig|6666666.59339.peg.7887	fig|6666666.59340.peg.429	fig|6666666.59337.peg.1379	fig|6666666.59342.peg.2181	fig|6666666.71181.peg.406	fig|6666666.59344.peg.1297	fig|6666666.59345.peg.2464	fig|6666666.59341.peg.1998	fig|6666666.59347.peg.3093	fig|6666666.59346.peg.3979	fig|6666666.59348.peg.171	None	None	None	fig|6666666.59353.peg.92	fig|6666666.59355.peg.702	None

6480	VgrG protein	fig|6666666.59329.peg.3545	fig|6666666.59330.peg.3121	fig|6666666.59331.peg.6202	fig|6666666.59332.peg.1097	None	fig|6666666.59335.peg.3427	fig|6666666.59336.peg.4905	fig|6666666.59333.peg.1948	fig|6666666.59338.peg.1335	fig|6666666.59339.peg.2704	None	fig|6666666.59337.peg.5616	fig|6666666.59342.peg.1625	fig|6666666.71181.peg.287	fig|6666666.59344.peg.2184	fig|6666666.59345.peg.4101	fig|6666666.59341.peg.5436	fig|6666666.59347.peg.1037	None	fig|6666666.59348.peg.4108	fig|6666666.59349.peg.1997	None	fig|6666666.59351.peg.1473	fig|6666666.59353.peg.4894	fig|6666666.59355.peg.5009	fig|6666666.59354.peg.2843

3180	Phosphoribulokinase EC 2.7.1.19 homolog, function unknown	fig|6666666.59329.peg.6	fig|6666666.59330.peg.1453	fig|6666666.59331.peg.4175	fig|6666666.59332.peg.3183	fig|6666666.59334.peg.720	None	fig|6666666.59336.peg.284	fig|6666666.59333.peg.2355	fig|6666666.59338.peg.2641	fig|6666666.59339.peg.8234	None	fig|6666666.59337.peg.1805	None	fig|6666666.71181.peg.1806	fig|6666666.59344.peg.3096	fig|6666666.59345.peg.5568	fig|6666666.59341.peg.2766	fig|6666666.59347.peg.4959	fig|6666666.59346.peg.26	fig|6666666.59348.peg.282	fig|6666666.59349.peg.2125	None	fig|6666666.59351.peg.145	fig|6666666.59353.peg.2245	fig|6666666.59355.peg.1937	fig|6666666.59354.peg.4718

3981	FIG01199774 hypothetical protein	fig|6666666.59329.peg.5	fig|6666666.59330.peg.1454	None	fig|6666666.59332.peg.3184	None	fig|6666666.59335.peg.1873	fig|6666666.59336.peg.285	fig|6666666.59333.peg.2356	fig|6666666.59338.peg.2640	fig|6666666.59339.peg.8235	fig|6666666.59340.peg.3262	fig|6666666.59337.peg.1806	None	fig|6666666.71181.peg.1804	fig|6666666.59344.peg.3098	fig|6666666.59345.peg.5567	fig|6666666.59341.peg.2767	fig|6666666.59347.peg.4960	fig|6666666.59346.peg.27	fig|6666666.59348.peg.283	fig|6666666.59349.peg.3378	None	fig|6666666.59351.peg.4172	fig|6666666.59353.peg.2247	fig|6666666.59355.peg.1939	fig|6666666.59354.peg.5901

3982	Tryptophan synthase beta chain EC 4.2.1.20	fig|6666666.59329.peg.398	fig|6666666.59330.peg.3337	None	fig|6666666.59332.peg.5153	None	fig|6666666.59335.peg.5380	None	fig|6666666.59333.peg.1763	fig|6666666.59338.peg.6173	fig|6666666.59339.peg.3633	None	fig|6666666.59337.peg.3500	fig|6666666.59342.peg.6427	fig|6666666.71181.peg.4143	fig|6666666.59344.peg.456	fig|6666666.59345.peg.919	fig|6666666.59341.peg.1019	fig|6666666.59347.peg.3557	fig|6666666.59346.peg.1600	fig|6666666.59348.peg.1830	fig|6666666.59349.peg.4277	fig|6666666.59350.peg.2700	fig|6666666.59351.peg.784	fig|6666666.59353.peg.1778	fig|6666666.59355.peg.5635	fig|6666666.59354.peg.6419

287	Flagellar hook-length control protein FliK	fig|6666666.59329.peg.5862	fig|6666666.59330.peg.2908	fig|6666666.59331.peg.2242	fig|6666666.59332.peg.4082	None	fig|6666666.59335.peg.393	fig|6666666.59336.peg.987	fig|6666666.59333.peg.4562	fig|6666666.59338.peg.2385	fig|6666666.59339.peg.90	fig|6666666.59340.peg.2039	fig|6666666.59337.peg.3213	fig|6666666.59342.peg.3228	fig|6666666.71181.peg.4938	fig|6666666.59344.peg.3056	fig|6666666.59345.peg.2877	fig|6666666.59341.peg.123	fig|6666666.59347.peg.4612	fig|6666666.59346.peg.2884	fig|6666666.59348.peg.3264	None	fig|6666666.59350.peg.5763	fig|6666666.59351.peg.2051	None	None	fig|6666666.59354.peg.669

43	YcgN Fragment	None	fig|6666666.59330.peg.4128	None	fig|6666666.59332.peg.1341	fig|6666666.59334.peg.2896	fig|6666666.59335.peg.2753	fig|6666666.59336.peg.1245	fig|6666666.59333.peg.4934	fig|6666666.59338.peg.2302	fig|6666666.59339.peg.252	None	fig|6666666.59337.peg.3746	None	fig|6666666.71181.peg.2946	fig|6666666.59344.peg.2864	fig|6666666.59345.peg.1182	fig|6666666.59341.peg.2464	fig|6666666.59347.peg.2441	fig|6666666.59346.peg.69	fig|6666666.59348.peg.2014	fig|6666666.59349.peg.3437	fig|6666666.59350.peg.2077	fig|6666666.59351.peg.1676	fig|6666666.59353.peg.1458	fig|6666666.59355.peg.2008	fig|6666666.59354.peg.4314

8387	Cell division topological specificity factor MinE	fig|6666666.59329.peg.4740	None	fig|6666666.59331.peg.5866	fig|6666666.59332.peg.1217	fig|6666666.59334.peg.3445	fig|6666666.59335.peg.2763	fig|6666666.59336.peg.1024	fig|6666666.59333.peg.1356	fig|6666666.59338.peg.1135	fig|6666666.59339.peg.238	fig|6666666.59340.peg.2190	fig|6666666.59337.peg.2750	None	fig|6666666.71181.peg.5196	fig|6666666.59344.peg.2855	fig|6666666.59345.peg.4297	fig|6666666.59341.peg.3857	fig|6666666.59347.peg.4413	fig|6666666.59346.peg.1475	fig|6666666.59348.peg.2716	fig|6666666.59349.peg.474	None	fig|6666666.59351.peg.1644	fig|6666666.59353.peg.1464	None	fig|6666666.59354.peg.5725

3351	Tricarboxylate transport protein TctC	fig|6666666.59329.peg.6185	fig|6666666.59330.peg.4109	fig|6666666.59331.peg.4351	None	fig|6666666.59334.peg.2670	fig|6666666.59335.peg.2025	fig|6666666.59336.peg.5377	fig|6666666.59333.peg.3045	fig|6666666.59338.peg.3055	fig|6666666.59339.peg.3970	fig|6666666.59340.peg.2458	fig|6666666.59337.peg.3117	fig|6666666.59342.peg.2864	fig|6666666.71181.peg.6170	fig|6666666.59344.peg.1870	fig|6666666.59345.peg.4126	fig|6666666.59341.peg.4871	fig|6666666.59347.peg.1310	fig|6666666.59346.peg.3014	fig|6666666.59348.peg.3363	None	fig|6666666.59350.peg.6741	None	fig|6666666.59353.peg.1220	fig|6666666.59355.peg.256	None

5476	Quinolinate synthetase EC 2.5.1.72	fig|6666666.59329.peg.1624	fig|6666666.59330.peg.3432	fig|6666666.59331.peg.6093	None	fig|6666666.59334.peg.2373	fig|6666666.59335.peg.2513	fig|6666666.59336.peg.5480	fig|6666666.59333.peg.1995	None	fig|6666666.59339.peg.404	None	fig|6666666.59337.peg.4376	fig|6666666.59342.peg.2252	fig|6666666.71181.peg.2495	None	fig|6666666.59345.peg.1502	fig|6666666.59341.peg.4879	fig|6666666.59347.peg.4212	fig|6666666.59346.peg.1437	fig|6666666.59348.peg.3007	fig|6666666.59349.peg.2657	fig|6666666.59350.peg.406	fig|6666666.59351.peg.5193	fig|6666666.59353.peg.2104	fig|6666666.59355.peg.2717	fig|6666666.59354.peg.4359

7683	Predicted acetyltransferase	fig|6666666.59329.peg.2931	fig|6666666.59330.peg.93	fig|6666666.59331.peg.2068	fig|6666666.59332.peg.5580	fig|6666666.59334.peg.4218	fig|6666666.59335.peg.2068	fig|6666666.59336.peg.2464	fig|6666666.59333.peg.2313	fig|6666666.59338.peg.1553	fig|6666666.59339.peg.4888	None	fig|6666666.59337.peg.696	fig|6666666.59342.peg.4544	fig|6666666.71181.peg.162	fig|6666666.59344.peg.3746	fig|6666666.59345.peg.3993	fig|6666666.59341.peg.2867	fig|6666666.59347.peg.582	fig|6666666.59346.peg.4016	fig|6666666.59348.peg.2205	None	fig|6666666.59350.peg.2822	None	fig|6666666.59353.peg.3111	fig|6666666.59355.peg.139	None

9158	Amino acid transporter	fig|6666666.59329.peg.4408	fig|6666666.59330.peg.4030	fig|6666666.59331.peg.1313	fig|6666666.59332.peg.4569	fig|6666666.59334.peg.4207	fig|6666666.59335.peg.4740	fig|6666666.59336.peg.5518	fig|6666666.59333.peg.4117	fig|6666666.59338.peg.5219	fig|6666666.59339.peg.4346	None	fig|6666666.59337.peg.4571	fig|6666666.59342.peg.3606	fig|6666666.71181.peg.4586	fig|6666666.59344.peg.5494	fig|6666666.59345.peg.5673	fig|6666666.59341.peg.5569	fig|6666666.59347.peg.1238	fig|6666666.59346.peg.4651	fig|6666666.59348.peg.3157	None	fig|6666666.59350.peg.5832	None	fig|6666666.59353.peg.1541	fig|6666666.59355.peg.4890	None

51	6-phosphofructokinase EC 2.7.1.11	fig|6666666.59329.peg.2013	fig|6666666.59330.peg.166	None	fig|6666666.59332.peg.162	fig|6666666.59334.peg.959	fig|6666666.59335.peg.3549	None	fig|6666666.59333.peg.421	fig|6666666.59338.peg.2362	fig|6666666.59339.peg.8695	fig|6666666.59340.peg.1896	fig|6666666.59337.peg.4336	None	fig|6666666.71181.peg.4011	fig|6666666.59344.peg.983	fig|6666666.59345.peg.5118	fig|6666666.59341.peg.197	fig|6666666.59347.peg.4528	fig|6666666.59346.peg.794	fig|6666666.59348.peg.1644	fig|6666666.59349.peg.4837	None	fig|6666666.59351.peg.406	fig|6666666.59353.peg.2555	fig|6666666.59355.peg.2646	fig|6666666.59354.peg.1373

2437	Potassium voltage-gated channel subfamily KQT; possible potassium channel, VIC family	fig|6666666.59329.peg.386	fig|6666666.59330.peg.4924	fig|6666666.59331.peg.3805	fig|6666666.59332.peg.3851	fig|6666666.59334.peg.3303	fig|6666666.59335.peg.2767	fig|6666666.59336.peg.3660	fig|6666666.59333.peg.196	fig|6666666.59338.peg.3265	fig|6666666.59339.peg.7403	fig|6666666.59340.peg.2795	fig|6666666.59337.peg.11	fig|6666666.59342.peg.2381	None	None	fig|6666666.59345.peg.1140	fig|6666666.59341.peg.1558	fig|6666666.59347.peg.3961	fig|6666666.59346.peg.4374	fig|6666666.59348.peg.3377	fig|6666666.59349.peg.3773	fig|6666666.59350.peg.4638	fig|6666666.59351.peg.4281	None	None	fig|6666666.59354.peg.3692

6340	Dihydrofolate reductase homolog	fig|6666666.59329.peg.5217	fig|6666666.59330.peg.4428	fig|6666666.59331.peg.2959	fig|6666666.59332.peg.2034	fig|6666666.59334.peg.3529	fig|6666666.59335.peg.2154	None	None	fig|6666666.59338.peg.3828	fig|6666666.59339.peg.6757	None	fig|6666666.59337.peg.2120	None	fig|6666666.71181.peg.4746	fig|6666666.59344.peg.5897	fig|6666666.59345.peg.3931	fig|6666666.59341.peg.3654	fig|6666666.59347.peg.505	fig|6666666.59346.peg.1165	fig|6666666.59348.peg.1623	fig|6666666.59349.peg.2442	fig|6666666.59350.peg.6975	fig|6666666.59351.peg.1918	fig|6666666.59353.peg.5053	fig|6666666.59355.peg.5081	fig|6666666.59354.peg.5664

9608	Transcriptional regulator, ArsR family	fig|6666666.59329.peg.5735	fig|6666666.59330.peg.4947	fig|6666666.59331.peg.5359	fig|6666666.59332.peg.1535	fig|6666666.59334.peg.4297	fig|6666666.59335.peg.755	fig|6666666.59336.peg.4335	fig|6666666.59333.peg.1819	fig|6666666.59338.peg.3573	fig|6666666.59339.peg.5600	fig|6666666.59340.peg.2294	fig|6666666.59337.peg.2475	fig|6666666.59342.peg.4285	None	None	fig|6666666.59345.peg.578	fig|6666666.59341.peg.2139	fig|6666666.59347.peg.596	fig|6666666.59346.peg.3668	fig|6666666.59348.peg.1124	fig|6666666.59349.peg.2317	fig|6666666.59350.peg.5141	fig|6666666.59351.peg.2930	None	None	fig|6666666.59354.peg.284

4997	SanA protein	fig|6666666.59329.peg.2771	fig|6666666.59330.peg.3884	fig|6666666.59331.peg.753	fig|6666666.59332.peg.5259	fig|6666666.59334.peg.4353	fig|6666666.59335.peg.1680	fig|6666666.59336.peg.6264	fig|6666666.59333.peg.5493	fig|6666666.59338.peg.3880	fig|6666666.59339.peg.3675	fig|6666666.59340.peg.2756	fig|6666666.59337.peg.4138	fig|6666666.59342.peg.4487	fig|6666666.71181.peg.197	fig|6666666.59344.peg.239	fig|6666666.59345.peg.4607	fig|6666666.59341.peg.1408	fig|6666666.59347.peg.1961	fig|6666666.59346.peg.3930	fig|6666666.59348.peg.4393	None	None	None	fig|6666666.59353.peg.625	fig|6666666.59355.peg.108	None

3338	Preprotein translocase subunit SecE TC 3.A.5.1.1	fig|6666666.59329.peg.6519	fig|6666666.59330.peg.3403	fig|6666666.59331.peg.2755	fig|6666666.59332.peg.2884	fig|6666666.59334.peg.2472	fig|6666666.59335.peg.5624	fig|6666666.59336.peg.1678	fig|6666666.59333.peg.4398	None	fig|6666666.59339.peg.8849	None	fig|6666666.59337.peg.4984	fig|6666666.59342.peg.372	fig|6666666.71181.peg.5020	fig|6666666.59344.peg.5468	fig|6666666.59345.peg.5659	fig|6666666.59341.peg.5589	fig|6666666.59347.peg.5403	fig|6666666.59346.peg.4094	None	fig|6666666.59349.peg.1147	None	fig|6666666.59351.peg.5237	fig|6666666.59353.peg.4446	fig|6666666.59355.peg.4581	fig|6666666.59354.peg.5812

1234	F TPR repeat protein, SEL1 subfamily	fig|6666666.59329.peg.4198	fig|6666666.59330.peg.1435	fig|6666666.59331.peg.415	fig|6666666.59332.peg.4263	None	fig|6666666.59335.peg.1858	fig|6666666.59336.peg.2647	fig|6666666.59333.peg.3670	fig|6666666.59338.peg.5329	fig|6666666.59339.peg.1936	fig|6666666.59340.peg.766	fig|6666666.59337.peg.4538	fig|6666666.59342.peg.3474	fig|6666666.71181.peg.5200	None	fig|6666666.59345.peg.4618	fig|6666666.59341.peg.335	fig|6666666.59347.peg.3661	fig|6666666.59346.peg.4895	fig|6666666.59348.peg.2384	fig|6666666.59349.peg.3441	fig|6666666.59350.peg.5730	fig|6666666.59351.peg.2727	None	None	fig|6666666.59354.peg.4266

6504	Molybdenum cofactor biosynthesis protein MoaB	fig|6666666.59329.peg.5053	fig|6666666.59330.peg.1711	None	fig|6666666.59332.peg.1522	fig|6666666.59334.peg.297	fig|6666666.59335.peg.5488	fig|6666666.59336.peg.2032	fig|6666666.59333.peg.1331	fig|6666666.59338.peg.2310	fig|6666666.59339.peg.140	fig|6666666.59340.peg.3677	fig|6666666.59337.peg.4025	fig|6666666.59342.peg.873	fig|6666666.71181.peg.2532	fig|6666666.59344.peg.4101	fig|6666666.59345.peg.1112	fig|6666666.59341.peg.2166	fig|6666666.59347.peg.5156	fig|6666666.59346.peg.533	fig|6666666.59348.peg.717	None	fig|6666666.59350.peg.1246	None	fig|6666666.59353.peg.2142	fig|6666666.59355.peg.2677	None

4907	glutathione S-transfersae-related protein	fig|6666666.59329.peg.5721	None	fig|6666666.59331.peg.5540	fig|6666666.59332.peg.970	None	fig|6666666.59335.peg.751	fig|6666666.59336.peg.5599	fig|6666666.59333.peg.5356	fig|6666666.59338.peg.4484	fig|6666666.59339.peg.5603	None	fig|6666666.59337.peg.5900	fig|6666666.59342.peg.4238	fig|6666666.71181.peg.1915	fig|6666666.59344.peg.2524	fig|6666666.59345.peg.3406	fig|6666666.59341.peg.5265	fig|6666666.59347.peg.161	fig|6666666.59346.peg.2580	fig|6666666.59348.peg.4018	fig|6666666.59349.peg.1603	None	fig|6666666.59351.peg.1029	fig|6666666.59353.peg.1494	fig|6666666.59355.peg.2044	fig|6666666.59354.peg.2334

7515	Sigma factor RpoE regulatory protein RseC	fig|6666666.59329.peg.499	fig|6666666.59330.peg.382	None	fig|6666666.59332.peg.731	fig|6666666.59334.peg.1873	fig|6666666.59335.peg.1719	fig|6666666.59336.peg.2104	fig|6666666.59333.peg.2805	fig|6666666.59338.peg.1512	fig|6666666.59339.peg.7363	None	fig|6666666.59337.peg.562	fig|6666666.59342.peg.2970	fig|6666666.71181.peg.1427	fig|6666666.59344.peg.1562	fig|6666666.59345.peg.5052	fig|6666666.59341.peg.5366	fig|6666666.59347.peg.3179	fig|6666666.59346.peg.331	fig|6666666.59348.peg.652	fig|6666666.59349.peg.4315	None	fig|6666666.59351.peg.595	fig|6666666.59353.peg.2648	fig|6666666.59355.peg.1834	None

7578	Exodeoxyribonuclease I EC 3.1.11.1	fig|6666666.59329.peg.6072	fig|6666666.59330.peg.2671	fig|6666666.59331.peg.4888	fig|6666666.59332.peg.3202	fig|6666666.59334.peg.5202	fig|6666666.59335.peg.5725	fig|6666666.59336.peg.1691	fig|6666666.59333.peg.544	None	fig|6666666.59339.peg.3896	None	fig|6666666.59337.peg.4531	fig|6666666.59342.peg.395	fig|6666666.71181.peg.4585	fig|6666666.59344.peg.5495	fig|6666666.59345.peg.4667	fig|6666666.59341.peg.1277	fig|6666666.59347.peg.1639	fig|6666666.59346.peg.4404	None	fig|6666666.59349.peg.2251	None	fig|6666666.59351.peg.5150	fig|6666666.59353.peg.4829	fig|6666666.59355.peg.4891	fig|6666666.59354.peg.2759

9602	Intracellular septation protein IspA	None	fig|6666666.59330.peg.3345	fig|6666666.59331.peg.6247	None	fig|6666666.59334.peg.4571	fig|6666666.59335.peg.3836	fig|6666666.59336.peg.4861	fig|6666666.59333.peg.1919	fig|6666666.59338.peg.5858	fig|6666666.59339.peg.560	fig|6666666.59340.peg.2397	fig|6666666.59337.peg.3507	None	fig|6666666.71181.peg.2785	fig|6666666.59344.peg.447	fig|6666666.59345.peg.925	fig|6666666.59341.peg.3418	fig|6666666.59347.peg.3561	fig|6666666.59346.peg.4962	fig|6666666.59348.peg.370	fig|6666666.59349.peg.2682	fig|6666666.59350.peg.1768	None	fig|6666666.59353.peg.1768	fig|6666666.59355.peg.1244	fig|6666666.59354.peg.2577

753	Queuosine Biosynthesis QueE Radical SAM	None	fig|6666666.59330.peg.3936	fig|6666666.59331.peg.3156	fig|6666666.59332.peg.5611	None	fig|6666666.59335.peg.5075	fig|6666666.59336.peg.4109	fig|6666666.59333.peg.3167	fig|6666666.59338.peg.5629	fig|6666666.59339.peg.4037	None	fig|6666666.59337.peg.4953	fig|6666666.59342.peg.1399	fig|6666666.71181.peg.3400	fig|6666666.59344.peg.2216	fig|6666666.59345.peg.4089	fig|6666666.59341.peg.4724	fig|6666666.59347.peg.1802	fig|6666666.59346.peg.4334	fig|6666666.59348.peg.4895	fig|6666666.59349.peg.3449	None	fig|6666666.59351.peg.3971	fig|6666666.59353.peg.193	fig|6666666.59355.peg.2962	fig|6666666.59354.peg.2197

8678	PTS system, maltose and glucose-specific IIC component EC 2.7.1.69 PTS system, maltose and glucose-specific IIB component EC 2.7.1.69	fig|6666666.59329.peg.2659	fig|6666666.59330.peg.1905	fig|6666666.59331.peg.5778	fig|6666666.59332.peg.3826	fig|6666666.59334.peg.220	fig|6666666.59335.peg.4424	fig|6666666.59336.peg.5499	fig|6666666.59333.peg.3818	fig|6666666.59338.peg.2415	fig|6666666.59339.peg.3239	fig|6666666.59340.peg.1208	fig|6666666.59337.peg.1044	fig|6666666.59342.peg.3348	fig|6666666.71181.peg.1631	fig|6666666.59344.peg.134	fig|6666666.59345.peg.1444	fig|6666666.59341.peg.1208	fig|6666666.59347.peg.28	fig|6666666.59346.peg.116	fig|6666666.59348.peg.710	None	None	None	fig|6666666.59353.peg.323	fig|6666666.59355.peg.1117	None

4095	Sulfite reductase [NADPH] hemoprotein beta-component EC 1.8.1.2	fig|6666666.59329.peg.5366	fig|6666666.59330.peg.4457	fig|6666666.59331.peg.3317	fig|6666666.59332.peg.1873	fig|6666666.59334.peg.156	fig|6666666.59335.peg.337	None	None	fig|6666666.59338.peg.5762	fig|6666666.59339.peg.8334	None	fig|6666666.59337.peg.1726	None	fig|6666666.71181.peg.3403	fig|6666666.59344.peg.2213	fig|6666666.59345.peg.5358	fig|6666666.59341.peg.5976	fig|6666666.59347.peg.4913	fig|6666666.59346.peg.3210	fig|6666666.59348.peg.2265	fig|6666666.59349.peg.1172	fig|6666666.59350.peg.1510	fig|6666666.59351.peg.53	fig|6666666.59353.peg.196	fig|6666666.59355.peg.2965	fig|6666666.59354.peg.2823

5137	FIG000605 protein co-occurring with transport systems C1739	fig|6666666.59329.peg.1990	fig|6666666.59330.peg.1084	None	fig|6666666.59332.peg.66	fig|6666666.59334.peg.3368	fig|6666666.59335.peg.6091	fig|6666666.59336.peg.2815	fig|6666666.59333.peg.4075	fig|6666666.59338.peg.1346	fig|6666666.59339.peg.7989	fig|6666666.59340.peg.568	fig|6666666.59337.peg.856	None	fig|6666666.71181.peg.3325	fig|6666666.59344.peg.3398	fig|6666666.59345.peg.4996	fig|6666666.59341.peg.4040	fig|6666666.59347.peg.4814	fig|6666666.59346.peg.3715	None	fig|6666666.59349.peg.2811	None	fig|6666666.59351.peg.5046	fig|6666666.59353.peg.3813	fig|6666666.59355.peg.3410	fig|6666666.59354.peg.6222

2757	Protein of unknown function YceH	fig|6666666.59329.peg.6066	fig|6666666.59330.peg.2118	fig|6666666.59331.peg.5915	None	fig|6666666.59334.peg.5201	fig|6666666.59335.peg.5030	fig|6666666.59336.peg.6203	fig|6666666.59333.peg.100	None	fig|6666666.59339.peg.2813	None	fig|6666666.59337.peg.4587	fig|6666666.59342.peg.2318	fig|6666666.71181.peg.1903	fig|6666666.59344.peg.2540	fig|6666666.59345.peg.507	fig|6666666.59341.peg.5601	fig|6666666.59347.peg.882	fig|6666666.59346.peg.3308	fig|6666666.59348.peg.829	fig|6666666.59349.peg.1537	None	fig|6666666.59351.peg.4736	fig|6666666.59353.peg.1508	fig|6666666.59355.peg.2058	fig|6666666.59354.peg.5911

4111	Thiamin ABC transporter, substrate-binding component	fig|6666666.59329.peg.1474	fig|6666666.59330.peg.836	fig|6666666.59331.peg.1732	fig|6666666.59332.peg.2333	None	fig|6666666.59335.peg.186	fig|6666666.59336.peg.916	fig|6666666.59333.peg.569	fig|6666666.59338.peg.310	fig|6666666.59339.peg.7733	fig|6666666.59340.peg.516	fig|6666666.59337.peg.329	fig|6666666.59342.peg.4721	fig|6666666.71181.peg.386	fig|6666666.59344.peg.1509	fig|6666666.59345.peg.2479	fig|6666666.59341.peg.1232	fig|6666666.59347.peg.4875	fig|6666666.59346.peg.651	fig|6666666.59348.peg.1995	None	fig|6666666.59350.peg.3009	None	fig|6666666.59353.peg.113	fig|6666666.59355.peg.722	None

3146	Hypothetical protein YaeJ with similarity to translation release factor	fig|6666666.59329.peg.1085	fig|6666666.59330.peg.4586	fig|6666666.59331.peg.3516	fig|6666666.59332.peg.2412	fig|6666666.59334.peg.3722	fig|6666666.59335.peg.2147	None	fig|6666666.59333.peg.5197	fig|6666666.59338.peg.1035	fig|6666666.59339.peg.6766	None	fig|6666666.59337.peg.3549	fig|6666666.59342.peg.648	fig|6666666.71181.peg.11	fig|6666666.59344.peg.2679	fig|6666666.59345.peg.470	fig|6666666.59341.peg.3605	fig|6666666.59347.peg.1823	fig|6666666.59346.peg.3486	None	fig|6666666.59349.peg.3574	fig|6666666.59350.peg.629	fig|6666666.59351.peg.1271	fig|6666666.59353.peg.2009	fig|6666666.59355.peg.4114	None

2346	Transamidase GatB domain protein	fig|6666666.59329.peg.681	fig|6666666.59330.peg.3131	None	fig|6666666.59332.peg.1514	fig|6666666.59334.peg.2467	fig|6666666.59335.peg.1644	fig|6666666.59336.peg.5193	fig|6666666.59333.peg.172	fig|6666666.59338.peg.694	fig|6666666.59339.peg.7906	fig|6666666.59340.peg.1274	fig|6666666.59337.peg.1424	fig|6666666.59342.peg.1637	None	None	fig|6666666.59345.peg.3848	fig|6666666.59341.peg.3722	fig|6666666.59347.peg.3138	fig|6666666.59346.peg.2561	fig|6666666.59348.peg.2745	fig|6666666.59349.peg.4853	fig|6666666.59350.peg.5954	fig|6666666.59351.peg.2366	None	fig|6666666.59355.peg.5326	fig|6666666.59354.peg.15

6852	Uroporphyrinogen-III synthase EC 4.2.1.75	fig|6666666.59329.peg.98	fig|6666666.59330.peg.3045	None	fig|6666666.59332.peg.1711	fig|6666666.59334.peg.1137	fig|6666666.59335.peg.3328	fig|6666666.59336.peg.1123	fig|6666666.59333.peg.783	fig|6666666.59338.peg.4276	fig|6666666.59339.peg.8513	fig|6666666.59340.peg.3017	fig|6666666.59337.peg.1612	fig|6666666.59342.peg.3003	fig|6666666.71181.peg.2437	fig|6666666.59344.peg.2481	fig|6666666.59345.peg.5509	fig|6666666.59341.peg.3911	fig|6666666.59347.peg.5082	fig|6666666.59346.peg.594	fig|6666666.59348.peg.2176	None	None	None	fig|6666666.59353.peg.3636	fig|6666666.59355.peg.4006	fig|6666666.59354.peg.1463

3887	Stringent starvation protein B	fig|6666666.59329.peg.2052	fig|6666666.59330.peg.968	fig|6666666.59331.peg.4187	fig|6666666.59332.peg.644	fig|6666666.59334.peg.2335	fig|6666666.59335.peg.4927	fig|6666666.59336.peg.716	fig|6666666.59333.peg.614	None	fig|6666666.59339.peg.4948	fig|6666666.59340.peg.525	fig|6666666.59337.peg.33	None	fig|6666666.71181.peg.1043	fig|6666666.59344.peg.1825	fig|6666666.59345.peg.3809	fig|6666666.59341.peg.3543	fig|6666666.59347.peg.3928	fig|6666666.59346.peg.2332	None	fig|6666666.59349.peg.45	None	fig|6666666.59351.peg.3466	fig|6666666.59353.peg.2361	fig|6666666.59355.peg.1634	fig|6666666.59354.peg.363

2484	Phage shock protein B	fig|6666666.59329.peg.4968	fig|6666666.59330.peg.1839	None	fig|6666666.59332.peg.4750	None	fig|6666666.59335.peg.4629	fig|6666666.59336.peg.5891	fig|6666666.59333.peg.5107	fig|6666666.59338.peg.1640	fig|6666666.59339.peg.522	None	fig|6666666.59337.peg.2328	fig|6666666.59342.peg.3325	fig|6666666.71181.peg.1388	fig|6666666.59344.peg.533	fig|6666666.59345.peg.1267	fig|6666666.59341.peg.1036	fig|6666666.59347.peg.3539	fig|6666666.59346.peg.1292	fig|6666666.59348.peg.2617	fig|6666666.59349.peg.2087	None	fig|6666666.59351.peg.2600	fig|6666666.59353.peg.1840	fig|6666666.59355.peg.1174	fig|6666666.59354.peg.3387

8564	Lysine efflux permease	fig|6666666.59329.peg.247	fig|6666666.59330.peg.362	fig|6666666.59331.peg.4075	fig|6666666.59332.peg.534	fig|6666666.59334.peg.2547	fig|6666666.59335.peg.2313	fig|6666666.59336.peg.1196	fig|6666666.59333.peg.4188	fig|6666666.59338.peg.1031	fig|6666666.59339.peg.7855	None	fig|6666666.59337.peg.543	fig|6666666.59342.peg.1509	fig|6666666.71181.peg.3137	fig|6666666.59344.peg.4731	fig|6666666.59345.peg.4451	fig|6666666.59341.peg.5253	fig|6666666.59347.peg.1817	fig|6666666.59346.peg.3790	fig|6666666.59348.peg.198	None	fig|6666666.59350.peg.7691	None	fig|6666666.59353.peg.4429	fig|6666666.59355.peg.4509	None

4706	Recombination protein RecR	None	fig|6666666.59330.peg.4123	None	fig|6666666.59332.peg.4656	fig|6666666.59334.peg.2751	None	fig|6666666.59336.peg.5782	fig|6666666.59333.peg.5289	fig|6666666.59338.peg.2299	fig|6666666.59339.peg.3067	None	fig|6666666.59337.peg.3739	fig|6666666.59342.peg.3338	fig|6666666.71181.peg.3498	fig|6666666.59344.peg.5518	fig|6666666.59345.peg.1191	fig|6666666.59341.peg.2461	fig|6666666.59347.peg.2444	fig|6666666.59346.peg.66	fig|6666666.59348.peg.3118	fig|6666666.59349.peg.2402	fig|6666666.59350.peg.2507	fig|6666666.59351.peg.2780	fig|6666666.59353.peg.4074	fig|6666666.59355.peg.4151	fig|6666666.59354.peg.1497

8821	FIG152265 Sodium solute symporter associated protein	fig|6666666.59329.peg.1065	fig|6666666.59330.peg.180	fig|6666666.59331.peg.865	fig|6666666.59332.peg.148	fig|6666666.59334.peg.33	fig|6666666.59335.peg.458	fig|6666666.59336.peg.1303	fig|6666666.59333.peg.2213	fig|6666666.59338.peg.2147	fig|6666666.59339.peg.6464	fig|6666666.59340.peg.453	fig|6666666.59337.peg.4302	fig|6666666.59342.peg.2743	None	None	fig|6666666.59345.peg.5133	fig|6666666.59341.peg.5493	fig|6666666.59347.peg.4506	fig|6666666.59346.peg.779	fig|6666666.59348.peg.1232	fig|6666666.59349.peg.3011	fig|6666666.59350.peg.5631	fig|6666666.59351.peg.345	None	None	fig|6666666.59354.peg.3591

10910	FIG01200298 hypothetical protein	fig|6666666.59329.peg.2649	fig|6666666.59330.peg.1306	fig|6666666.59331.peg.4370	fig|6666666.59332.peg.4847	None	None	fig|6666666.59336.peg.4225	fig|6666666.59333.peg.1020	fig|6666666.59338.peg.773	None	fig|6666666.59340.peg.2332	fig|6666666.59337.peg.3384	fig|6666666.59342.peg.2772	fig|6666666.71181.peg.3504	fig|6666666.59344.peg.5526	fig|6666666.59345.peg.1567	fig|6666666.59341.peg.2331	fig|6666666.59347.peg.2365	fig|6666666.59346.peg.2202	fig|6666666.59348.peg.3380	fig|6666666.59349.peg.1907	None	fig|6666666.59351.peg.468	fig|6666666.59353.peg.4082	fig|6666666.59355.peg.4158	fig|6666666.59354.peg.5733

8775	Predicted signal-transduction protein containing cAMP-binding and CBS domains	fig|6666666.59329.peg.4504	fig|6666666.59330.peg.3652	fig|6666666.59331.peg.4277	fig|6666666.59332.peg.4067	fig|6666666.59334.peg.41	fig|6666666.59335.peg.1041	fig|6666666.59336.peg.375	fig|6666666.59333.peg.3260	fig|6666666.59338.peg.510	fig|6666666.59339.peg.6451	fig|6666666.59340.peg.331	fig|6666666.59337.peg.4294	fig|6666666.59342.peg.424	None	None	fig|6666666.59345.peg.854	fig|6666666.59341.peg.1175	fig|6666666.59347.peg.4500	fig|6666666.59346.peg.5274	fig|6666666.59348.peg.1817	fig|6666666.59349.peg.3832	fig|6666666.59350.peg.5421	fig|6666666.59351.peg.3687	None	None	fig|6666666.59354.peg.2111

7646	Pyruvate formate-lyase EC 2.3.1.54	fig|6666666.59329.peg.922	fig|6666666.59330.peg.1266	fig|6666666.59331.peg.1598	fig|6666666.59332.peg.810	fig|6666666.59334.peg.451	fig|6666666.59335.peg.4088	fig|6666666.59336.peg.462	fig|6666666.59333.peg.3023	fig|6666666.59338.peg.2141	fig|6666666.59339.peg.3496	fig|6666666.59340.peg.2713	fig|6666666.59337.peg.5094	fig|6666666.59342.peg.5550	fig|6666666.71181.peg.4204	fig|6666666.59344.peg.2810	fig|6666666.59345.peg.1119	fig|6666666.59341.peg.3536	fig|6666666.59347.peg.4176	fig|6666666.59346.peg.3238	fig|6666666.59348.peg.1011	None	None	None	fig|6666666.59353.peg.1639	fig|6666666.59355.peg.1082	None

7644	Formate dehydrogenase-O, iron-sulfur subunit EC 1.2.1.2 ; Putative formate dehydrogenase iron-sulfur subunit EC 1.2.1.2	fig|6666666.59329.peg.4230	fig|6666666.59330.peg.2595	fig|6666666.59331.peg.1225	fig|6666666.59332.peg.6279	fig|6666666.59334.peg.3554	fig|6666666.59335.peg.5590	fig|6666666.59336.peg.2285	fig|6666666.59333.peg.3875	fig|6666666.59338.peg.4850	fig|6666666.59339.peg.7128	None	fig|6666666.59337.peg.1761	fig|6666666.59342.peg.2873	fig|6666666.71181.peg.673	fig|6666666.59344.peg.4112	fig|6666666.59345.peg.1005	fig|6666666.59341.peg.2625	fig|6666666.59347.peg.1721	fig|6666666.59346.peg.504	fig|6666666.59348.peg.5260	None	fig|6666666.59350.peg.353	None	fig|6666666.59353.peg.4092	fig|6666666.59355.peg.2347	None

6790	Uncharacterized protein ImpJ VasE	fig|6666666.59329.peg.5545	fig|6666666.59330.peg.4163	None	fig|6666666.59332.peg.4136	None	fig|6666666.59335.peg.4108	fig|6666666.59336.peg.5273	fig|6666666.59333.peg.3048	fig|6666666.59338.peg.4068	fig|6666666.59339.peg.2738	fig|6666666.59340.peg.3576	fig|6666666.59337.peg.4072	fig|6666666.59342.peg.4393	fig|6666666.71181.peg.2263	fig|6666666.59344.peg.2995	fig|6666666.59345.peg.4118	fig|6666666.59341.peg.4557	fig|6666666.59347.peg.1020	None	fig|6666666.59348.peg.4272	fig|6666666.59349.peg.1643	None	None	fig|6666666.59353.peg.3559	fig|6666666.59355.peg.3615	fig|6666666.59354.peg.743

9250	FIG01200087 hypothetical protein	fig|6666666.59329.peg.2142	fig|6666666.59330.peg.4314	None	fig|6666666.59332.peg.4056	fig|6666666.59334.peg.4373	fig|6666666.59335.peg.1968	fig|6666666.59336.peg.3990	fig|6666666.59333.peg.2157	fig|6666666.59338.peg.461	fig|6666666.59339.peg.6992	fig|6666666.59340.peg.1656	fig|6666666.59337.peg.766	fig|6666666.59342.peg.3721	fig|6666666.71181.peg.428	fig|6666666.59344.peg.1330	fig|6666666.59345.peg.3626	fig|6666666.59341.peg.6080	fig|6666666.59347.peg.4307	fig|6666666.59346.peg.716	fig|6666666.59348.peg.2034	None	None	None	fig|6666666.59353.peg.70	fig|6666666.59355.peg.677	None

179	Ribulosamine erythrulosamine 3-kinase potentially involved in protein deglycation	None	fig|6666666.59330.peg.4654	None	fig|6666666.59332.peg.4877	fig|6666666.59334.peg.4878	fig|6666666.59335.peg.3865	fig|6666666.59336.peg.6043	fig|6666666.59333.peg.4689	fig|6666666.59338.peg.1028	fig|6666666.59339.peg.1182	None	fig|6666666.59337.peg.2697	fig|6666666.59342.peg.4572	fig|6666666.71181.peg.4327	fig|6666666.59344.peg.826	fig|6666666.59345.peg.3153	fig|6666666.59341.peg.3613	fig|6666666.59347.peg.1813	fig|6666666.59346.peg.5227	None	fig|6666666.59349.peg.4346	None	fig|6666666.59351.peg.912	fig|6666666.59353.peg.1285	fig|6666666.59355.peg.3297	fig|6666666.59354.peg.4870

3680	AttF component of AttEFGH ABC transport system AttG component of AttEFGH ABC transport system	fig|6666666.59329.peg.4997	fig|6666666.59330.peg.3857	fig|6666666.59331.peg.2499	fig|6666666.59332.peg.4129	fig|6666666.59334.peg.3921	fig|6666666.59335.peg.1342	fig|6666666.59336.peg.649	fig|6666666.59333.peg.2654	fig|6666666.59338.peg.4992	fig|6666666.59339.peg.917	None	fig|6666666.59337.peg.5246	fig|6666666.59342.peg.4531	None	None	fig|6666666.59345.peg.2051	fig|6666666.59341.peg.584	fig|6666666.59347.peg.2102	fig|6666666.59346.peg.1545	fig|6666666.59348.peg.752	fig|6666666.59349.peg.2342	fig|6666666.59350.peg.814	fig|6666666.59351.peg.3991	None	None	fig|6666666.59354.peg.2099

2722	Chromosome partition protein MukF	fig|6666666.59329.peg.5349	fig|6666666.59330.peg.4161	fig|6666666.59331.peg.602	fig|6666666.59332.peg.5452	fig|6666666.59334.peg.594	fig|6666666.59335.peg.938	fig|6666666.59336.peg.1397	fig|6666666.59333.peg.1059	fig|6666666.59338.peg.4367	fig|6666666.59339.peg.315	None	fig|6666666.59337.peg.4938	fig|6666666.59342.peg.143	fig|6666666.71181.peg.4181	fig|6666666.59344.peg.2783	fig|6666666.59345.peg.1534	fig|6666666.59341.peg.2328	fig|6666666.59347.peg.2370	fig|6666666.59346.peg.2018	fig|6666666.59348.peg.5341	None	None	None	fig|6666666.59353.peg.1662	fig|6666666.59355.peg.1059	None

7233	Sterol desaturase	fig|6666666.59329.peg.5018	fig|6666666.59330.peg.695	fig|6666666.59331.peg.3869	fig|6666666.59332.peg.5673	None	fig|6666666.59335.peg.2471	fig|6666666.59336.peg.6483	fig|6666666.59333.peg.5599	fig|6666666.59338.peg.2406	fig|6666666.59339.peg.3581	fig|6666666.59340.peg.1823	fig|6666666.59337.peg.2709	fig|6666666.59342.peg.4990	None	None	fig|6666666.59345.peg.4776	fig|6666666.59341.peg.4848	fig|6666666.59347.peg.2208	fig|6666666.59346.peg.4405	fig|6666666.59348.peg.2854	fig|6666666.59349.peg.3320	fig|6666666.59350.peg.4441	fig|6666666.59351.peg.624	None	None	fig|6666666.59354.peg.3980

1704	2-dehydropantoate 2-reductase EC 1.1.1.169	None	fig|6666666.59330.peg.2798	fig|6666666.59331.peg.5831	fig|6666666.59332.peg.864	fig|6666666.59334.peg.4947	fig|6666666.59335.peg.108	fig|6666666.59336.peg.3461	fig|6666666.59333.peg.1926	fig|6666666.59338.peg.3153	fig|6666666.59339.peg.5032	None	fig|6666666.59337.peg.2368	None	fig|6666666.71181.peg.2037	fig|6666666.59344.peg.1085	fig|6666666.59345.peg.2250	fig|6666666.59341.peg.536	fig|6666666.59347.peg.2813	fig|6666666.59346.peg.2710	None	fig|6666666.59349.peg.1766	None	fig|6666666.59351.peg.2293	fig|6666666.59353.peg.767	fig|6666666.59355.peg.937	fig|6666666.59354.peg.494

6435	Thiazole biosynthesis protein ThiH	fig|6666666.59329.peg.360	fig|6666666.59330.peg.982	fig|6666666.59331.peg.1274	fig|6666666.59332.peg.255	fig|6666666.59334.peg.2192	fig|6666666.59335.peg.10	fig|6666666.59336.peg.179	fig|6666666.59333.peg.1569	fig|6666666.59338.peg.1670	fig|6666666.59339.peg.7534	fig|6666666.59340.peg.521	fig|6666666.59337.peg.2549	None	fig|6666666.71181.peg.3362	fig|6666666.59344.peg.5447	fig|6666666.59345.peg.5541	fig|6666666.59341.peg.3746	fig|6666666.59347.peg.5253	fig|6666666.59346.peg.930	fig|6666666.59348.peg.3766	None	None	None	fig|6666666.59353.peg.4461	fig|6666666.59355.peg.4566	None

2531	ATPase of the AAA+ class	fig|6666666.59329.peg.4177	fig|6666666.59330.peg.4865	fig|6666666.59331.peg.4347	fig|6666666.59332.peg.569	fig|6666666.59334.peg.1475	fig|6666666.59335.peg.4036	fig|6666666.59336.peg.3233	fig|6666666.59333.peg.1965	fig|6666666.59338.peg.3379	fig|6666666.59339.peg.8593	fig|6666666.59340.peg.230	fig|6666666.59337.peg.1665	None	fig|6666666.71181.peg.4441	fig|6666666.59344.peg.1026	fig|6666666.59345.peg.5634	fig|6666666.59341.peg.3565	fig|6666666.59347.peg.4652	fig|6666666.59346.peg.1652	fig|6666666.59348.peg.2989	None	None	None	fig|6666666.59353.peg.2517	fig|6666666.59355.peg.4875	None

10799	Phosphate transport regulator distant homolog of PhoU	fig|6666666.59329.peg.382	fig|6666666.59330.peg.943	fig|6666666.59331.peg.2335	fig|6666666.59332.peg.3853	fig|6666666.59334.peg.537	fig|6666666.59335.peg.1511	fig|6666666.59336.peg.3436	fig|6666666.59333.peg.193	fig|6666666.59338.peg.212	fig|6666666.59339.peg.7399	fig|6666666.59340.peg.1192	fig|6666666.59337.peg.7	fig|6666666.59342.peg.1375	None	None	fig|6666666.59345.peg.3832	fig|6666666.59341.peg.1561	fig|6666666.59347.peg.3959	fig|6666666.59346.peg.3119	fig|6666666.59348.peg.5143	fig|6666666.59349.peg.3553	None	fig|6666666.59351.peg.2327	None	None	fig|6666666.59354.peg.1277

7143	DNA polymerase III delta subunit EC 2.7.7.7	fig|6666666.59329.peg.2165	fig|6666666.59330.peg.4795	None	fig|6666666.59332.peg.5141	None	fig|6666666.59335.peg.1752	fig|6666666.59336.peg.577	fig|6666666.59333.peg.2139	fig|6666666.59338.peg.14	fig|6666666.59339.peg.5210	None	fig|6666666.59337.peg.1084	fig|6666666.59342.peg.905	fig|6666666.71181.peg.4765	fig|6666666.59344.peg.4497	fig|6666666.59345.peg.3491	None	fig|6666666.59347.peg.2902	fig|6666666.59346.peg.2949	fig|6666666.59348.peg.845	fig|6666666.59349.peg.2760	None	fig|6666666.59351.peg.3627	fig|6666666.59353.peg.2024	fig|6666666.59355.peg.4431	fig|6666666.59354.peg.2324

8054	FIG01202089 hypothetical protein	None	fig|6666666.59330.peg.3396	fig|6666666.59331.peg.5040	fig|6666666.59332.peg.3334	fig|6666666.59334.peg.1921	fig|6666666.59335.peg.4539	fig|6666666.59336.peg.5449	fig|6666666.59333.peg.1905	fig|6666666.59338.peg.3531	fig|6666666.59339.peg.3539	None	fig|6666666.59337.peg.3517	fig|6666666.59342.peg.4269	fig|6666666.71181.peg.3647	fig|6666666.59344.peg.5247	fig|6666666.59345.peg.936	fig|6666666.59341.peg.2650	fig|6666666.59347.peg.2324	fig|6666666.59346.peg.3901	fig|6666666.59348.peg.1308	None	None	fig|6666666.59351.peg.161	fig|6666666.59353.peg.4720	fig|6666666.59355.peg.4770	None

10792	Putative cell envelope opacity-associated protein A	fig|6666666.59329.peg.5639	fig|6666666.59330.peg.580	fig|6666666.59331.peg.6300	fig|6666666.59332.peg.3501	fig|6666666.59334.peg.74	fig|6666666.59335.peg.4758	fig|6666666.59336.peg.170	fig|6666666.59333.peg.392	fig|6666666.59338.peg.4860	fig|6666666.59339.peg.8296	None	fig|6666666.59337.peg.363	fig|6666666.59342.peg.5061	fig|6666666.71181.peg.2816	fig|6666666.59344.peg.2146	fig|6666666.59345.peg.2509	fig|6666666.59341.peg.780	fig|6666666.59347.peg.4849	fig|6666666.59346.peg.875	fig|6666666.59348.peg.428	None	None	None	fig|6666666.59353.peg.3174	fig|6666666.59355.peg.423	None

3478	FIG01199554 hypothetical protein	fig|6666666.59329.peg.3969	fig|6666666.59330.peg.2715	fig|6666666.59331.peg.4434	fig|6666666.59332.peg.993	fig|6666666.59334.peg.2859	fig|6666666.59335.peg.4527	fig|6666666.59336.peg.4899	fig|6666666.59333.peg.5169	fig|6666666.59338.peg.6007	fig|6666666.59339.peg.2485	fig|6666666.59340.peg.2098	fig|6666666.59337.peg.2444	fig|6666666.59342.peg.2958	None	None	fig|6666666.59345.peg.605	fig|6666666.59341.peg.224	fig|6666666.59347.peg.548	fig|6666666.59346.peg.3022	fig|6666666.59348.peg.4374	fig|6666666.59349.peg.2605	None	fig|6666666.59351.peg.2195	None	None	fig|6666666.59354.peg.2482

5249	Aldose 1-epimerase EC 5.1.3.3	fig|6666666.59329.peg.1331	None	fig|6666666.59331.peg.3390	fig|6666666.59332.peg.1176	fig|6666666.59334.peg.2609	fig|6666666.59335.peg.3686	fig|6666666.59336.peg.3474	fig|6666666.59333.peg.1145	fig|6666666.59338.peg.3547	fig|6666666.59339.peg.6863	fig|6666666.59340.peg.3392	fig|6666666.59337.peg.1354	None	fig|6666666.71181.peg.2504	fig|6666666.59344.peg.4062	fig|6666666.59345.peg.2290	fig|6666666.59341.peg.6116	fig|6666666.59347.peg.4222	fig|6666666.59346.peg.2240	fig|6666666.59348.peg.568	None	fig|6666666.59350.peg.6677	None	fig|6666666.59353.peg.2113	fig|6666666.59355.peg.2708	None

916	TPR domain protein, putative component of TonB system	fig|6666666.59329.peg.1271	fig|6666666.59330.peg.1640	fig|6666666.59331.peg.4863	fig|6666666.59332.peg.2288	fig|6666666.59334.peg.4103	fig|6666666.59335.peg.4624	fig|6666666.59336.peg.941	fig|6666666.59333.peg.1259	fig|6666666.59338.peg.5642	fig|6666666.59339.peg.984	fig|6666666.59340.peg.714	fig|6666666.59337.peg.1534	fig|6666666.59342.peg.579	None	None	fig|6666666.59345.peg.2647	fig|6666666.59341.peg.1147	fig|6666666.59347.peg.3064	fig|6666666.59346.peg.2464	fig|6666666.59348.peg.5027	fig|6666666.59349.peg.432	None	fig|6666666.59351.peg.1692	None	None	fig|6666666.59354.peg.5865

8637	Permease of the drug metabolite transporter DMT superfamily	fig|6666666.59329.peg.36	fig|6666666.59330.peg.736	fig|6666666.59331.peg.2845	fig|6666666.59332.peg.752	fig|6666666.59334.peg.1742	fig|6666666.59335.peg.4661	fig|6666666.59336.peg.1822	fig|6666666.59333.peg.4197	fig|6666666.59338.peg.815	fig|6666666.59339.peg.8385	fig|6666666.59340.peg.2062	fig|6666666.59337.peg.4998	fig|6666666.59342.peg.1880	None	None	fig|6666666.59345.peg.5092	fig|6666666.59341.peg.4602	fig|6666666.59347.peg.434	fig|6666666.59346.peg.967	fig|6666666.59348.peg.5103	fig|6666666.59349.peg.3672	None	fig|6666666.59351.peg.4568	None	None	fig|6666666.59354.peg.1736

4583	Molybdenum ABC transporter, periplasmic molybdenum-binding protein ModA TC 3.A.1.8.1	fig|6666666.59329.peg.3509	fig|6666666.59330.peg.786	None	fig|6666666.59332.peg.2425	fig|6666666.59334.peg.1945	fig|6666666.59335.peg.546	fig|6666666.59336.peg.4484	fig|6666666.59333.peg.1522	fig|6666666.59338.peg.3677	fig|6666666.59339.peg.2963	None	fig|6666666.59337.peg.3815	fig|6666666.59342.peg.942	fig|6666666.71181.peg.2512	fig|6666666.59344.peg.4075	fig|6666666.59345.peg.58	fig|6666666.59341.peg.2235	fig|6666666.59347.peg.918	fig|6666666.59346.peg.1222	fig|6666666.59348.peg.4932	None	fig|6666666.59350.peg.3428	None	fig|6666666.59353.peg.2122	fig|6666666.59355.peg.2698	None

5594	Polyphosphate kinase 2 EC 2.7.4.1	fig|6666666.59329.peg.1123	fig|6666666.59330.peg.4242	None	None	fig|6666666.59334.peg.2833	fig|6666666.59335.peg.2577	fig|6666666.59336.peg.5466	fig|6666666.59333.peg.356	fig|6666666.59338.peg.1813	fig|6666666.59339.peg.6930	fig|6666666.59340.peg.2015	fig|6666666.59337.peg.719	None	fig|6666666.71181.peg.4623	fig|6666666.59344.peg.4876	fig|6666666.59345.peg.3670	None	fig|6666666.59347.peg.4270	None	fig|6666666.59348.peg.1335	fig|6666666.59349.peg.973	fig|6666666.59350.peg.4486	fig|6666666.59351.peg.1572	fig|6666666.59353.peg.3452	fig|6666666.59355.peg.3271	fig|6666666.59354.peg.495

10713	Holliday junction DNA helicase RuvA	None	fig|6666666.59330.peg.2489	fig|6666666.59331.peg.4436	fig|6666666.59332.peg.1960	None	fig|6666666.59335.peg.2498	fig|6666666.59336.peg.5352	fig|6666666.59333.peg.1043	fig|6666666.59338.peg.5530	fig|6666666.59339.peg.384	None	fig|6666666.59337.peg.4550	None	fig|6666666.71181.peg.1922	fig|6666666.59344.peg.2515	fig|6666666.59345.peg.1515	fig|6666666.59341.peg.3339	fig|6666666.59347.peg.4199	fig|6666666.59346.peg.2766	None	fig|6666666.59349.peg.3866	fig|6666666.59350.peg.8198	fig|6666666.59351.peg.654	fig|6666666.59353.peg.1487	fig|6666666.59355.peg.2037	fig|6666666.59354.peg.1591

10914	FIG01200701 hypothetical protein	fig|6666666.59329.peg.3748	fig|6666666.59330.peg.4114	fig|6666666.59331.peg.4020	fig|6666666.59332.peg.2598	fig|6666666.59334.peg.701	fig|6666666.59335.peg.60	None	fig|6666666.59333.peg.1859	fig|6666666.59338.peg.2530	fig|6666666.59339.peg.7603	fig|6666666.59340.peg.1180	None	None	fig|6666666.71181.peg.3021	None	fig|6666666.59345.peg.2760	fig|6666666.59341.peg.2584	fig|6666666.59347.peg.5225	fig|6666666.59346.peg.2728	fig|6666666.59348.peg.1031	fig|6666666.59349.peg.1134	None	fig|6666666.59351.peg.88	fig|6666666.59353.peg.4175	fig|6666666.59355.peg.1702	fig|6666666.59354.peg.4465

3436	Uncharacterized protein ImpC	fig|6666666.59329.peg.2962	fig|6666666.59330.peg.3113	fig|6666666.59331.peg.3968	fig|6666666.59332.peg.2973	None	fig|6666666.59335.peg.2688	fig|6666666.59336.peg.5662	fig|6666666.59333.peg.1937	fig|6666666.59338.peg.5731	fig|6666666.59339.peg.4000	None	fig|6666666.59337.peg.5337	fig|6666666.59342.peg.1246	fig|6666666.71181.peg.4824	fig|6666666.59344.peg.2985	fig|6666666.59345.peg.4106	fig|6666666.59341.peg.5950	fig|6666666.59347.peg.1029	None	fig|6666666.59348.peg.4705	fig|6666666.59349.peg.1634	None	None	fig|6666666.59353.peg.1711	fig|6666666.59355.peg.3607	fig|6666666.59354.peg.404

3049	Cytosine permease	None	fig|6666666.59330.peg.854	None	fig|6666666.59332.peg.6224	fig|6666666.59334.peg.5290	fig|6666666.59335.peg.3408	fig|6666666.59336.peg.1335	fig|6666666.59333.peg.69	fig|6666666.59338.peg.3776	fig|6666666.59339.peg.4327	fig|6666666.59340.peg.1104	fig|6666666.59337.peg.3272	fig|6666666.59342.peg.5244	fig|6666666.71181.peg.2678	fig|6666666.59344.peg.4293	fig|6666666.59345.peg.537	fig|6666666.59341.peg.1301	fig|6666666.59347.peg.1565	fig|6666666.59346.peg.4054	fig|6666666.59348.peg.2541	None	None	fig|6666666.59351.peg.4149	fig|6666666.59353.peg.3893	fig|6666666.59355.peg.3799	None

8907	FIG01199611 hypothetical protein	fig|6666666.59329.peg.5455	fig|6666666.59330.peg.656	fig|6666666.59331.peg.5657	fig|6666666.59332.peg.5274	fig|6666666.59334.peg.1519	fig|6666666.59335.peg.5012	fig|6666666.59336.peg.1981	fig|6666666.59333.peg.1362	fig|6666666.59338.peg.2072	fig|6666666.59339.peg.8107	fig|6666666.59340.peg.880	fig|6666666.59337.peg.3912	fig|6666666.59342.peg.5714	None	None	fig|6666666.59345.peg.4916	fig|6666666.59341.peg.5020	fig|6666666.59347.peg.4737	fig|6666666.59346.peg.1835	fig|6666666.59348.peg.3240	fig|6666666.59349.peg.2829	None	fig|6666666.59351.peg.5067	None	None	fig|6666666.59354.peg.4823

1666	Flagellar biosynthesis protein FliL	fig|6666666.59329.peg.189	fig|6666666.59330.peg.1874	fig|6666666.59331.peg.2241	fig|6666666.59332.peg.4083	None	fig|6666666.59335.peg.394	fig|6666666.59336.peg.1270	fig|6666666.59333.peg.4565	fig|6666666.59338.peg.3101	fig|6666666.59339.peg.8573	fig|6666666.59340.peg.3581	fig|6666666.59337.peg.1654	fig|6666666.59342.peg.2652	None	None	fig|6666666.59345.peg.5337	fig|6666666.59341.peg.5292	fig|6666666.59347.peg.5050	fig|6666666.59346.peg.2885	fig|6666666.59348.peg.588	fig|6666666.59349.peg.205	fig|6666666.59350.peg.5762	fig|6666666.59351.peg.2052	None	None	fig|6666666.59354.peg.670

2828	FIG01199837 hypothetical protein	fig|6666666.59329.peg.6488	fig|6666666.59330.peg.3157	None	fig|6666666.59332.peg.4868	fig|6666666.59334.peg.4236	fig|6666666.59335.peg.1051	fig|6666666.59336.peg.4198	fig|6666666.59333.peg.4867	fig|6666666.59338.peg.2767	fig|6666666.59339.peg.4530	fig|6666666.59340.peg.2000	fig|6666666.59337.peg.1169	fig|6666666.59342.peg.505	fig|6666666.71181.peg.585	fig|6666666.59344.peg.4257	fig|6666666.59345.peg.3478	fig|6666666.59341.peg.5762	fig|6666666.59347.peg.1232	fig|6666666.59346.peg.4517	fig|6666666.59348.peg.2805	None	None	None	fig|6666666.59353.peg.3095	fig|6666666.59355.peg.2253	None

3417	Nucleotidase YfbR, HD superfamily	fig|6666666.59329.peg.5871	fig|6666666.59330.peg.306	fig|6666666.59331.peg.5067	fig|6666666.59332.peg.6243	fig|6666666.59334.peg.1416	fig|6666666.59335.peg.857	fig|6666666.59336.peg.2480	fig|6666666.59333.peg.1423	fig|6666666.59338.peg.1589	fig|6666666.59339.peg.3223	fig|6666666.59340.peg.2513	fig|6666666.59337.peg.1035	None	fig|6666666.71181.peg.547	fig|6666666.59344.peg.1921	fig|6666666.59345.peg.4248	fig|6666666.59341.peg.3791	fig|6666666.59347.peg.2649	fig|6666666.59346.peg.2673	fig|6666666.59348.peg.3150	None	None	None	fig|6666666.59353.peg.1176	fig|6666666.59355.peg.299	None

3626	FIG01199954 hypothetical protein	fig|6666666.59329.peg.4802	fig|6666666.59330.peg.497	fig|6666666.59331.peg.3057	fig|6666666.59332.peg.4482	fig|6666666.59334.peg.5163	fig|6666666.59335.peg.1562	fig|6666666.59336.peg.5016	fig|6666666.59333.peg.4352	fig|6666666.59338.peg.939	fig|6666666.59339.peg.3866	fig|6666666.59340.peg.3459	fig|6666666.59337.peg.3315	fig|6666666.59342.peg.6320	None	None	fig|6666666.59345.peg.4624	fig|6666666.59341.peg.1390	fig|6666666.59347.peg.3581	fig|6666666.59346.peg.3559	fig|6666666.59348.peg.4513	fig|6666666.59349.peg.3389	None	fig|6666666.59351.peg.3365	None	None	fig|6666666.59354.peg.4510

5875	FIG023911 putative membrane protein	fig|6666666.59329.peg.137	fig|6666666.59330.peg.823	fig|6666666.59331.peg.1355	fig|6666666.59332.peg.3914	fig|6666666.59334.peg.3954	fig|6666666.59335.peg.4486	fig|6666666.59336.peg.2659	fig|6666666.59333.peg.5028	fig|6666666.59338.peg.915	fig|6666666.59339.peg.7886	None	fig|6666666.59337.peg.1378	fig|6666666.59342.peg.2182	fig|6666666.71181.peg.407	fig|6666666.59344.peg.1298	fig|6666666.59345.peg.2465	fig|6666666.59341.peg.1997	fig|6666666.59347.peg.3094	fig|6666666.59346.peg.3980	fig|6666666.59348.peg.170	None	None	None	fig|6666666.59353.peg.91	fig|6666666.59355.peg.701	None

357	Multimeric flavodoxin WrbA	fig|6666666.59329.peg.3592	fig|6666666.59330.peg.1910	None	fig|6666666.59332.peg.1111	None	fig|6666666.59335.peg.1190	fig|6666666.59336.peg.1317	fig|6666666.59333.peg.3505	fig|6666666.59338.peg.791	fig|6666666.59339.peg.27	None	fig|6666666.59337.peg.258	fig|6666666.59342.peg.2937	fig|6666666.71181.peg.2365	fig|6666666.59344.peg.1254	fig|6666666.59345.peg.5243	fig|6666666.59341.peg.1670	fig|6666666.59347.peg.2856	fig|6666666.59346.peg.1852	None	fig|6666666.59349.peg.650	None	fig|6666666.59351.peg.1771	fig|6666666.59353.peg.4230	fig|6666666.59355.peg.4192	fig|6666666.59354.peg.2915

6946	Fructose-1,6-bisphosphatase, GlpX type EC 3.1.3.11	fig|6666666.59329.peg.5116	fig|6666666.59330.peg.5059	fig|6666666.59331.peg.5846	fig|6666666.59332.peg.168	fig|6666666.59334.peg.963	fig|6666666.59335.peg.422	fig|6666666.59336.peg.4986	fig|6666666.59333.peg.4830	fig|6666666.59338.peg.1170	fig|6666666.59339.peg.6368	None	fig|6666666.59337.peg.407	None	fig|6666666.71181.peg.4003	fig|6666666.59344.peg.991	fig|6666666.59345.peg.2551	fig|6666666.59341.peg.190	fig|6666666.59347.peg.4534	fig|6666666.59346.peg.800	fig|6666666.59348.peg.1950	None	fig|6666666.59350.peg.4567	None	fig|6666666.59353.peg.2547	fig|6666666.59355.peg.2654	None

5406	Ribose ABC transport system, high affinity permease RbsD TC 3.A.1.2.1	fig|6666666.59329.peg.5942	fig|6666666.59330.peg.707	None	fig|6666666.59332.peg.1646	fig|6666666.59334.peg.633	fig|6666666.59335.peg.637	fig|6666666.59336.peg.3807	fig|6666666.59333.peg.1253	fig|6666666.59338.peg.2262	fig|6666666.59339.peg.4246	None	fig|6666666.59337.peg.4616	fig|6666666.59342.peg.5744	fig|6666666.71181.peg.582	fig|6666666.59344.peg.4253	fig|6666666.59345.peg.4800	fig|6666666.59341.peg.2323	fig|6666666.59347.peg.1263	fig|6666666.59346.peg.459	fig|6666666.59348.peg.1530	None	fig|6666666.59350.peg.4559	None	fig|6666666.59353.peg.3098	fig|6666666.59355.peg.2250	None

10976	Galactose-1-phosphate uridylyltransferase EC 2.7.7.10	fig|6666666.59329.peg.1333	None	fig|6666666.59331.peg.4308	fig|6666666.59332.peg.2255	fig|6666666.59334.peg.2605	fig|6666666.59335.peg.4069	fig|6666666.59336.peg.1925	fig|6666666.59333.peg.1143	fig|6666666.59338.peg.3462	fig|6666666.59339.peg.6858	fig|6666666.59340.peg.3361	fig|6666666.59337.peg.1352	fig|6666666.59342.peg.5377	fig|6666666.71181.peg.2506	fig|6666666.59344.peg.4066	fig|6666666.59345.peg.2292	fig|6666666.59341.peg.298	fig|6666666.59347.peg.3770	fig|6666666.59346.peg.4537	fig|6666666.59348.peg.566	None	None	None	fig|6666666.59353.peg.2115	fig|6666666.59355.peg.2706	None

9982	Na+ H+ antiporter	fig|6666666.59329.peg.2581	fig|6666666.59330.peg.2865	fig|6666666.59331.peg.904	fig|6666666.59332.peg.2919	fig|6666666.59334.peg.5405	fig|6666666.59335.peg.1419	fig|6666666.59336.peg.1840	fig|6666666.59333.peg.2712	fig|6666666.59338.peg.398	fig|6666666.59339.peg.227	fig|6666666.59340.peg.3528	fig|6666666.59337.peg.3148	fig|6666666.59342.peg.2434	None	None	fig|6666666.59345.peg.3609	fig|6666666.59341.peg.3712	fig|6666666.59347.peg.372	fig|6666666.59346.peg.101	fig|6666666.59348.peg.4292	fig|6666666.59349.peg.1821	None	fig|6666666.59351.peg.4566	None	None	fig|6666666.59354.peg.5649

5513	Methylglyoxal synthase EC 4.2.3.3	None	fig|6666666.59330.peg.3178	fig|6666666.59331.peg.6234	fig|6666666.59332.peg.2860	fig|6666666.59334.peg.459	fig|6666666.59335.peg.5651	fig|6666666.59336.peg.817	fig|6666666.59333.peg.4931	fig|6666666.59338.peg.6001	fig|6666666.59339.peg.8449	None	fig|6666666.59337.peg.5472	fig|6666666.59342.peg.659	fig|6666666.71181.peg.271	fig|6666666.59344.peg.1198	fig|6666666.59345.peg.3297	fig|6666666.59341.peg.772	fig|6666666.59347.peg.976	fig|6666666.59346.peg.3655	None	None	fig|6666666.59350.peg.4860	fig|6666666.59351.peg.420	fig|6666666.59353.peg.1701	fig|6666666.59355.peg.1019	None

9422	tRNA Cytosine32 -2-thiocytidine synthetase	None	fig|6666666.59330.peg.3198	None	fig|6666666.59332.peg.5667	fig|6666666.59334.peg.2626	fig|6666666.59335.peg.2218	fig|6666666.59336.peg.3525	fig|6666666.59333.peg.4758	None	fig|6666666.59339.peg.1315	None	fig|6666666.59337.peg.3716	None	fig|6666666.71181.peg.1372	fig|6666666.59344.peg.551	fig|6666666.59345.peg.3225	fig|6666666.59341.peg.6077	fig|6666666.59347.peg.1735	fig|6666666.59346.peg.1524	fig|6666666.59348.peg.3258	fig|6666666.59349.peg.150	fig|6666666.59350.peg.3643	fig|6666666.59351.peg.825	fig|6666666.59353.peg.1856	fig|6666666.59355.peg.1157	fig|6666666.59354.peg.183

430	Positive regulator of competence TfoX	fig|6666666.59329.peg.3462	fig|6666666.59330.peg.1999	None	fig|6666666.59332.peg.2509	fig|6666666.59334.peg.3558	fig|6666666.59335.peg.931	fig|6666666.59336.peg.3902	fig|6666666.59333.peg.1618	fig|6666666.59338.peg.2419	fig|6666666.59339.peg.2903	fig|6666666.59340.peg.2678	fig|6666666.59337.peg.4644	fig|6666666.59342.peg.134	fig|6666666.71181.peg.267	fig|6666666.59344.peg.1194	fig|6666666.59345.peg.1540	fig|6666666.59341.peg.1014	fig|6666666.59347.peg.1283	fig|6666666.59346.peg.4113	fig|6666666.59348.peg.1185	None	None	None	fig|6666666.59353.peg.1697	fig|6666666.59355.peg.1023	None

5701	Predicted deacylase	fig|6666666.59329.peg.1019	fig|6666666.59330.peg.1073	fig|6666666.59331.peg.4553	fig|6666666.59332.peg.1762	fig|6666666.59334.peg.1890	fig|6666666.59335.peg.137	fig|6666666.59336.peg.3792	fig|6666666.59333.peg.2827	fig|6666666.59338.peg.4812	fig|6666666.59339.peg.1965	None	fig|6666666.59337.peg.5650	fig|6666666.59342.peg.6388	None	None	fig|6666666.59345.peg.3472	fig|6666666.59341.peg.1155	fig|6666666.59347.peg.980	fig|6666666.59346.peg.2249	fig|6666666.59348.peg.4512	fig|6666666.59349.peg.2418	fig|6666666.59350.peg.1995	fig|6666666.59351.peg.4681	None	None	fig|6666666.59354.peg.5053

6530	Molybdopterin-guanine dinucleotide biosynthesis protein MobA	fig|6666666.59329.peg.2465	fig|6666666.59330.peg.3815	None	fig|6666666.59332.peg.4969	fig|6666666.59334.peg.4179	fig|6666666.59335.peg.5611	fig|6666666.59336.peg.4576	fig|6666666.59333.peg.3242	fig|6666666.59338.peg.3886	fig|6666666.59339.peg.1211	None	fig|6666666.59337.peg.2715	fig|6666666.59342.peg.1129	fig|6666666.71181.peg.2578	fig|6666666.59344.peg.887	fig|6666666.59345.peg.3193	fig|6666666.59341.peg.4009	fig|6666666.59347.peg.1699	fig|6666666.59346.peg.4521	fig|6666666.59348.peg.2829	None	fig|6666666.59350.peg.3230	None	fig|6666666.59353.peg.2639	fig|6666666.59355.peg.2566	None

11328	C2907 Amine oxidase, flavin-containing	fig|6666666.59329.peg.2102	fig|6666666.59330.peg.2877	fig|6666666.59331.peg.6136	fig|6666666.59332.peg.3672	fig|6666666.59334.peg.2689	fig|6666666.59335.peg.608	fig|6666666.59336.peg.6187	fig|6666666.59333.peg.3155	fig|6666666.59338.peg.1415	fig|6666666.59339.peg.472	None	fig|6666666.59337.peg.4607	fig|6666666.59342.peg.6371	None	None	fig|6666666.59345.peg.1322	fig|6666666.59341.peg.482	fig|6666666.59347.peg.4125	fig|6666666.59346.peg.2980	fig|6666666.59348.peg.2672	fig|6666666.59349.peg.939	fig|6666666.59350.peg.4080	fig|6666666.59351.peg.1097	None	None	fig|6666666.59354.peg.1697

10029	Protein ytfJ precursor	fig|6666666.59329.peg.289	fig|6666666.59330.peg.600	None	fig|6666666.59332.peg.1199	fig|6666666.59334.peg.65	fig|6666666.59335.peg.169	fig|6666666.59336.peg.2828	fig|6666666.59333.peg.3088	fig|6666666.59338.peg.352	fig|6666666.59339.peg.7716	fig|6666666.59340.peg.2478	fig|6666666.59337.peg.343	fig|6666666.59342.peg.3955	fig|6666666.71181.peg.2812	fig|6666666.59344.peg.2140	fig|6666666.59345.peg.2491	fig|6666666.59341.peg.796	fig|6666666.59347.peg.4864	fig|6666666.59346.peg.1972	fig|6666666.59348.peg.3608	None	None	None	fig|6666666.59353.peg.3170	fig|6666666.59355.peg.427	None

6951	Putative cytochrome c oxidase, subunit I	None	fig|6666666.59330.peg.351	fig|6666666.59331.peg.3184	fig|6666666.59332.peg.1934	fig|6666666.59334.peg.3898	fig|6666666.59335.peg.3099	fig|6666666.59336.peg.3965	fig|6666666.59333.peg.778	fig|6666666.59338.peg.455	fig|6666666.59339.peg.7840	fig|6666666.59340.peg.210	fig|6666666.59337.peg.532	fig|6666666.59342.peg.3320	None	None	fig|6666666.59345.peg.5022	fig|6666666.59341.peg.2580	fig|6666666.59347.peg.3209	fig|6666666.59346.peg.3515	fig|6666666.59348.peg.360	fig|6666666.59349.peg.4077	fig|6666666.59350.peg.1162	fig|6666666.59351.peg.3762	None	None	fig|6666666.59354.peg.452

1051	C2827 putative endonuclease containing a URI domain	fig|6666666.59329.peg.4253	fig|6666666.59330.peg.2117	fig|6666666.59331.peg.5914	fig|6666666.59332.peg.5180	fig|6666666.59334.peg.5200	fig|6666666.59335.peg.5031	fig|6666666.59336.peg.6204	fig|6666666.59333.peg.101	fig|6666666.59338.peg.3933	fig|6666666.59339.peg.2814	None	fig|6666666.59337.peg.4588	fig|6666666.59342.peg.2319	fig|6666666.71181.peg.3532	fig|6666666.59344.peg.2071	fig|6666666.59345.peg.506	fig|6666666.59341.peg.5600	fig|6666666.59347.peg.883	fig|6666666.59346.peg.3309	fig|6666666.59348.peg.830	None	None	None	fig|6666666.59353.peg.3110	fig|6666666.59355.peg.487	None

11041	Aspartate carbamoyltransferase regulatory chain PyrI	fig|6666666.59329.peg.1446	fig|6666666.59330.peg.61	fig|6666666.59331.peg.3490	fig|6666666.59332.peg.1283	fig|6666666.59334.peg.79	fig|6666666.59335.peg.237	fig|6666666.59336.peg.1794	fig|6666666.59333.peg.5025	fig|6666666.59338.peg.123	fig|6666666.59339.peg.8163	None	fig|6666666.59337.peg.848	fig|6666666.59342.peg.433	fig|6666666.71181.peg.3291	fig|6666666.59344.peg.4648	fig|6666666.59345.peg.4846	fig|6666666.59341.peg.2697	fig|6666666.59347.peg.3236	fig|6666666.59346.peg.623	fig|6666666.59348.peg.2509	None	None	None	fig|6666666.59353.peg.2430	fig|6666666.59355.peg.523	None

8250	membrane protein	fig|6666666.59329.peg.2662	fig|6666666.59330.peg.319	fig|6666666.59331.peg.5833	fig|6666666.59332.peg.3429	fig|6666666.59334.peg.1205	fig|6666666.59335.peg.866	None	fig|6666666.59333.peg.1435	fig|6666666.59338.peg.2847	fig|6666666.59339.peg.3244	fig|6666666.59340.peg.545	fig|6666666.59337.peg.1047	None	fig|6666666.71181.peg.3186	fig|6666666.59344.peg.1660	fig|6666666.59345.peg.4235	fig|6666666.59341.peg.3595	fig|6666666.59347.peg.2640	fig|6666666.59346.peg.2072	fig|6666666.59348.peg.908	None	None	fig|6666666.59351.peg.1036	fig|6666666.59353.peg.1082	fig|6666666.59355.peg.397	None

8434	Periplasmic membrane protein associated with DUF414	fig|6666666.59329.peg.1827	fig|6666666.59330.peg.394	fig|6666666.59331.peg.4932	fig|6666666.59332.peg.24	fig|6666666.59334.peg.4843	fig|6666666.59335.peg.56	fig|6666666.59336.peg.1450	fig|6666666.59333.peg.843	fig|6666666.59338.peg.1322	fig|6666666.59339.peg.7597	fig|6666666.59340.peg.1146	fig|6666666.59337.peg.978	fig|6666666.59342.peg.2841	None	None	fig|6666666.59345.peg.2698	fig|6666666.59341.peg.4284	fig|6666666.59347.peg.5042	fig|6666666.59346.peg.1079	fig|6666666.59348.peg.99	fig|6666666.59349.peg.4816	None	fig|6666666.59351.peg.104	None	None	fig|6666666.59354.peg.3440

8438	LrgA-associated membrane protein LrgB	fig|6666666.59329.peg.5552	fig|6666666.59330.peg.2673	fig|6666666.59331.peg.5697	fig|6666666.59332.peg.3199	fig|6666666.59334.peg.4069	None	fig|6666666.59336.peg.1688	fig|6666666.59333.peg.542	None	fig|6666666.59339.peg.3892	None	fig|6666666.59337.peg.4529	fig|6666666.59342.peg.392	fig|6666666.71181.peg.200	fig|6666666.59344.peg.5643	fig|6666666.59345.peg.4665	fig|6666666.59341.peg.1275	fig|6666666.59347.peg.1637	fig|6666666.59346.peg.4402	None	fig|6666666.59349.peg.2069	None	fig|6666666.59351.peg.581	fig|6666666.59353.peg.622	fig|6666666.59355.peg.2869	fig|6666666.59354.peg.1398

2632	Chromosome plasmid partitioning protein ParB Stage 0 sporulation protein J	fig|6666666.59329.peg.1745	fig|6666666.59330.peg.1118	None	fig|6666666.59332.peg.97	fig|6666666.59334.peg.28	fig|6666666.59335.peg.790	fig|6666666.59336.peg.26	fig|6666666.59333.peg.31	fig|6666666.59338.peg.491	fig|6666666.59339.peg.8016	fig|6666666.59340.peg.747	fig|6666666.59337.peg.888	fig|6666666.59342.peg.1194	None	None	fig|6666666.59345.peg.4967	fig|6666666.59341.peg.1057	fig|6666666.59347.peg.4785	fig|6666666.59346.peg.1178	fig|6666666.59348.peg.329	fig|6666666.59349.peg.1027	fig|6666666.59350.peg.1503	fig|6666666.59351.peg.5361	None	None	fig|6666666.59354.peg.1148

765	Cell division inhibitor	fig|6666666.59329.peg.1646	fig|6666666.59330.peg.5148	fig|6666666.59331.peg.312	fig|6666666.59332.peg.5030	fig|6666666.59334.peg.5140	fig|6666666.59335.peg.4250	fig|6666666.59336.peg.305	fig|6666666.59333.peg.1213	fig|6666666.59338.peg.232	fig|6666666.59339.peg.1703	fig|6666666.59340.peg.1325	fig|6666666.59337.peg.216	fig|6666666.59342.peg.5041	None	None	fig|6666666.59345.peg.5286	fig|6666666.59341.peg.3821	fig|6666666.59347.peg.4	fig|6666666.59346.peg.4989	fig|6666666.59348.peg.2835	fig|6666666.59349.peg.4082	None	fig|6666666.59351.peg.4951	None	None	fig|6666666.59354.peg.460

4331	Signal transduction histidine kinase CheA EC 2.7.3.-	fig|6666666.59329.peg.5231	fig|6666666.59330.peg.4877	None	fig|6666666.59332.peg.5809	None	fig|6666666.59335.peg.1240	fig|6666666.59336.peg.4767	fig|6666666.59333.peg.1688	None	fig|6666666.59339.peg.5471	None	fig|6666666.59337.peg.2980	fig|6666666.59342.peg.5328	fig|6666666.71181.peg.2249	fig|6666666.59344.peg.3010	fig|6666666.59345.peg.5323	None	fig|6666666.59347.peg.2671	fig|6666666.59346.peg.4525	fig|6666666.59348.peg.4446	fig|6666666.59349.peg.191	fig|6666666.59350.peg.4205	fig|6666666.59351.peg.2068	fig|6666666.59353.peg.3546	fig|6666666.59355.peg.3629	fig|6666666.59354.peg.2080

1791	Formate dehydrogenase -O, gamma subunit EC 1.2.1.2	fig|6666666.59329.peg.6497	fig|6666666.59330.peg.2596	fig|6666666.59331.peg.5430	None	fig|6666666.59334.peg.3092	fig|6666666.59335.peg.2199	fig|6666666.59336.peg.3292	fig|6666666.59333.peg.3221	fig|6666666.59338.peg.1305	fig|6666666.59339.peg.1294	None	fig|6666666.59337.peg.3704	fig|6666666.59342.peg.217	fig|6666666.71181.peg.674	fig|6666666.59344.peg.4111	fig|6666666.59345.peg.3214	fig|6666666.59341.peg.2893	fig|6666666.59347.peg.1722	fig|6666666.59346.peg.3582	fig|6666666.59348.peg.3796	None	fig|6666666.59350.peg.354	None	fig|6666666.59353.peg.4091	fig|6666666.59355.peg.2348	None

9847	Protein of unknown function DUF484	fig|6666666.59329.peg.88	fig|6666666.59330.peg.3054	fig|6666666.59331.peg.2552	None	fig|6666666.59334.peg.4853	fig|6666666.59335.peg.3338	fig|6666666.59336.peg.1115	fig|6666666.59333.peg.792	fig|6666666.59338.peg.1783	fig|6666666.59339.peg.8526	None	fig|6666666.59337.peg.1619	fig|6666666.59342.peg.3162	fig|6666666.71181.peg.2431	fig|6666666.59344.peg.2488	fig|6666666.59345.peg.5500	fig|6666666.59341.peg.3304	fig|6666666.59347.peg.5074	None	None	fig|6666666.59349.peg.1555	None	fig|6666666.59351.peg.4749	fig|6666666.59353.peg.3630	fig|6666666.59355.peg.4994	fig|6666666.59354.peg.1452

10282	FIG01289452 hypothetical protein	fig|6666666.59329.peg.79	fig|6666666.59330.peg.1544	fig|6666666.59331.peg.827	fig|6666666.59332.peg.474	fig|6666666.59334.peg.4429	fig|6666666.59335.peg.4119	fig|6666666.59336.peg.624	fig|6666666.59333.peg.799	fig|6666666.59338.peg.1995	fig|6666666.59339.peg.8540	None	fig|6666666.59337.peg.1626	fig|6666666.59342.peg.5620	fig|6666666.71181.peg.3795	fig|6666666.59344.peg.2507	fig|6666666.59345.peg.5491	fig|6666666.59341.peg.3297	fig|6666666.59347.peg.5068	fig|6666666.59346.peg.612	fig|6666666.59348.peg.745	None	None	None	fig|6666666.59353.peg.4642	fig|6666666.59355.peg.4759	None

4488	Phosphatidylserine decarboxylase EC 4.1.1.65	fig|6666666.59329.peg.40	fig|6666666.59330.peg.4943	None	fig|6666666.59332.peg.754	fig|6666666.59334.peg.2763	None	fig|6666666.59336.peg.1826	fig|6666666.59333.peg.4199	fig|6666666.59338.peg.821	fig|6666666.59339.peg.8812	fig|6666666.59340.peg.3131	fig|6666666.59337.peg.5003	None	fig|6666666.71181.peg.1692	None	fig|6666666.59345.peg.5088	fig|6666666.59341.peg.4605	fig|6666666.59347.peg.4563	fig|6666666.59346.peg.964	fig|6666666.59348.peg.1651	fig|6666666.59349.peg.3225	fig|6666666.59350.peg.1110	fig|6666666.59351.peg.3889	fig|6666666.59353.peg.1062	fig|6666666.59355.peg.3556	None

3155	Octanoate-[acyl-carrier-protein]-protein-N-octanoyltransferase	fig|6666666.59329.peg.3584	fig|6666666.59330.peg.2031	None	None	None	fig|6666666.59335.peg.1761	None	fig|6666666.59333.peg.5118	fig|6666666.59338.peg.4	fig|6666666.59339.peg.5199	fig|6666666.59340.peg.399	fig|6666666.59337.peg.1093	fig|6666666.59342.peg.919	fig|6666666.71181.peg.2382	fig|6666666.59344.peg.3337	fig|6666666.59345.peg.3500	fig|6666666.59341.peg.5014	fig|6666666.59347.peg.2910	fig|6666666.59346.peg.3289	None	fig|6666666.59349.peg.2771	fig|6666666.59350.peg.2906	fig|6666666.59351.peg.3618	fig|6666666.59353.peg.3719	fig|6666666.59355.peg.3734	fig|6666666.59354.peg.5641

568	Arginine ornithine antiporter ArcD	fig|6666666.59329.peg.6089	fig|6666666.59330.peg.3152	fig|6666666.59331.peg.1146	fig|6666666.59332.peg.5310	fig|6666666.59334.peg.4317	fig|6666666.59335.peg.2808	fig|6666666.59336.peg.3793	fig|6666666.59333.peg.4159	fig|6666666.59338.peg.4392	fig|6666666.59339.peg.1962	fig|6666666.59340.peg.1640	fig|6666666.59337.peg.1163	fig|6666666.59342.peg.497	None	None	fig|6666666.59345.peg.1399	fig|6666666.59341.peg.4696	fig|6666666.59347.peg.2610	fig|6666666.59346.peg.4586	fig|6666666.59348.peg.1860	fig|6666666.59349.peg.488	None	fig|6666666.59351.peg.903	None	None	fig|6666666.59354.peg.5447

564	Maltose operon periplasmic protein MalM	fig|6666666.59329.peg.6179	fig|6666666.59330.peg.1806	fig|6666666.59331.peg.3265	fig|6666666.59332.peg.2263	fig|6666666.59334.peg.2577	fig|6666666.59335.peg.5449	fig|6666666.59336.peg.2396	fig|6666666.59333.peg.1068	fig|6666666.59338.peg.388	fig|6666666.59339.peg.2105	fig|6666666.59340.peg.3137	fig|6666666.59337.peg.3425	fig|6666666.59342.peg.1711	fig|6666666.71181.peg.2008	fig|6666666.59344.peg.2733	fig|6666666.59345.peg.1957	fig|6666666.59341.peg.334	fig|6666666.59347.peg.3664	fig|6666666.59346.peg.3315	None	None	None	None	fig|6666666.59353.peg.3005	fig|6666666.59355.peg.2912	None

7246	Putative cytoplasmic protein	fig|6666666.59329.peg.5114	fig|6666666.59330.peg.5060	None	fig|6666666.59332.peg.169	fig|6666666.59334.peg.964	fig|6666666.59335.peg.423	fig|6666666.59336.peg.4987	fig|6666666.59333.peg.4827	fig|6666666.59338.peg.1169	fig|6666666.59339.peg.6369	fig|6666666.59340.peg.1592	fig|6666666.59337.peg.406	fig|6666666.59342.peg.5565	fig|6666666.71181.peg.3898	fig|6666666.59344.peg.995	fig|6666666.59345.peg.2550	fig|6666666.59341.peg.189	fig|6666666.59347.peg.4536	fig|6666666.59346.peg.1017	fig|6666666.59348.peg.1951	None	None	None	fig|6666666.59353.peg.2544	fig|6666666.59355.peg.2657	None

10541	Cell-cell signaling protein, C-factor	fig|6666666.59329.peg.5385	fig|6666666.59330.peg.2356	fig|6666666.59331.peg.3110	fig|6666666.59332.peg.6172	fig|6666666.59334.peg.4420	fig|6666666.59335.peg.4555	fig|6666666.59336.peg.1133	fig|6666666.59333.peg.4240	fig|6666666.59338.peg.6165	fig|6666666.59339.peg.2189	fig|6666666.59340.peg.1017	fig|6666666.59337.peg.2227	None	None	None	fig|6666666.59345.peg.2991	fig|6666666.59341.peg.5246	fig|6666666.59347.peg.205	fig|6666666.59346.peg.4613	fig|6666666.59348.peg.4861	fig|6666666.59349.peg.4083	fig|6666666.59350.peg.6777	fig|6666666.59351.peg.2964	None	None	fig|6666666.59354.peg.463

8811	Syd protein	fig|6666666.59329.peg.2944	fig|6666666.59330.peg.3892	None	fig|6666666.59332.peg.396	fig|6666666.59334.peg.1904	fig|6666666.59335.peg.350	None	fig|6666666.59333.peg.3269	fig|6666666.59338.peg.2027	fig|6666666.59339.peg.7146	None	fig|6666666.59337.peg.1114	None	fig|6666666.71181.peg.47	fig|6666666.59344.peg.2631	fig|6666666.59345.peg.3517	fig|6666666.59341.peg.2858	fig|6666666.59347.peg.2926	fig|6666666.59346.peg.1724	fig|6666666.59348.peg.3295	fig|6666666.59349.peg.1343	None	fig|6666666.59351.peg.4233	fig|6666666.59353.peg.1970	fig|6666666.59355.peg.1436	fig|6666666.59354.peg.2634

11147	LSU ribosomal protein L25p	fig|6666666.59329.peg.1431	fig|6666666.59330.peg.3684	fig|6666666.59331.peg.5135	fig|6666666.59332.peg.5460	fig|6666666.59334.peg.4755	fig|6666666.59335.peg.3321	fig|6666666.59336.peg.2374	fig|6666666.59333.peg.1199	fig|6666666.59338.peg.4200	fig|6666666.59339.peg.876	None	fig|6666666.59337.peg.5631	fig|6666666.59342.peg.1209	fig|6666666.71181.peg.489	fig|6666666.59344.peg.1701	fig|6666666.59345.peg.1229	fig|6666666.59341.peg.864	fig|6666666.59347.peg.2001	fig|6666666.59346.peg.216	fig|6666666.59348.peg.2030	None	None	None	fig|6666666.59353.peg.1116	fig|6666666.59355.peg.360	None

988	Fumarate reductase subunit D	fig|6666666.59329.peg.5432	fig|6666666.59330.peg.155	fig|6666666.59331.peg.2529	fig|6666666.59332.peg.745	fig|6666666.59334.peg.4003	fig|6666666.59335.peg.439	fig|6666666.59336.peg.259	fig|6666666.59333.peg.432	fig|6666666.59338.peg.809	fig|6666666.59339.peg.6395	None	fig|6666666.59337.peg.5188	fig|6666666.59342.peg.2306	fig|6666666.71181.peg.1685	fig|6666666.59344.peg.607	fig|6666666.59345.peg.5107	fig|6666666.59341.peg.170	fig|6666666.59347.peg.4555	fig|6666666.59346.peg.973	fig|6666666.59348.peg.151	None	None	None	fig|6666666.59353.peg.1055	fig|6666666.59355.peg.3563	None

11258	Alpha-ribazole-5'-phosphate phosphatase EC 3.1.3.73	fig|6666666.59329.peg.3346	fig|6666666.59330.peg.2660	None	fig|6666666.59332.peg.6075	None	fig|6666666.59335.peg.2169	fig|6666666.59336.peg.5746	fig|6666666.59333.peg.1783	fig|6666666.59338.peg.3165	fig|6666666.59339.peg.3908	None	fig|6666666.59337.peg.1983	fig|6666666.59342.peg.2061	fig|6666666.71181.peg.441	fig|6666666.59344.peg.1346	fig|6666666.59345.peg.2130	fig|6666666.59341.peg.546	fig|6666666.59347.peg.3352	fig|6666666.59346.peg.2036	fig|6666666.59348.peg.3564	None	fig|6666666.59350.peg.1316	fig|6666666.59351.peg.3922	fig|6666666.59353.peg.57	fig|6666666.59355.peg.664	None

723	Arginine-tRNA-protein transferase EC 2.3.2.8	fig|6666666.59329.peg.2569	fig|6666666.59330.peg.1321	fig|6666666.59331.peg.2256	None	fig|6666666.59334.peg.875	fig|6666666.59335.peg.5950	fig|6666666.59336.peg.865	fig|6666666.59333.peg.1006	fig|6666666.59338.peg.5088	fig|6666666.59339.peg.287	fig|6666666.59340.peg.3708	fig|6666666.59337.peg.3370	fig|6666666.59342.peg.117	None	None	fig|6666666.59345.peg.1552	fig|6666666.59341.peg.998	fig|6666666.59347.peg.4114	fig|6666666.59346.peg.2525	fig|6666666.59348.peg.1668	fig|6666666.59349.peg.2227	fig|6666666.59350.peg.2832	fig|6666666.59351.peg.879	None	None	fig|6666666.59354.peg.5343

2033	Lipoate-protein ligase A	fig|6666666.59329.peg.1048	fig|6666666.59330.peg.79	fig|6666666.59331.peg.4424	fig|6666666.59332.peg.4156	fig|6666666.59334.peg.490	fig|6666666.59335.peg.3645	fig|6666666.59336.peg.3737	fig|6666666.59333.peg.2567	None	fig|6666666.59339.peg.5024	None	fig|6666666.59337.peg.1308	fig|6666666.59342.peg.5605	fig|6666666.71181.peg.455	fig|6666666.59344.peg.1368	fig|6666666.59345.peg.2331	fig|6666666.59341.peg.4733	fig|6666666.59347.peg.2967	fig|6666666.59346.peg.2452	fig|6666666.59348.peg.4663	fig|6666666.59349.peg.4660	None	None	fig|6666666.59353.peg.41	fig|6666666.59355.peg.649	None

4653	CopG protein	fig|6666666.59329.peg.4442	fig|6666666.59330.peg.3103	None	fig|6666666.59332.peg.2342	fig|6666666.59334.peg.4231	fig|6666666.59335.peg.2413	fig|6666666.59336.peg.2861	fig|6666666.59333.peg.216	fig|6666666.59338.peg.33	fig|6666666.59339.peg.5275	fig|6666666.59340.peg.1044	fig|6666666.59337.peg.3325	fig|6666666.59342.peg.1158	None	None	fig|6666666.59345.peg.5432	fig|6666666.59341.peg.4020	fig|6666666.59347.peg.2883	fig|6666666.59346.peg.4671	fig|6666666.59348.peg.1371	fig|6666666.59349.peg.2568	fig|6666666.59350.peg.3636	fig|6666666.59351.peg.3517	None	None	fig|6666666.59354.peg.1885

6202	Uncharacterized conserved protein	fig|6666666.59329.peg.3862	fig|6666666.59330.peg.1622	fig|6666666.59331.peg.164	fig|6666666.59332.peg.4766	fig|6666666.59334.peg.1079	fig|6666666.59335.peg.660	fig|6666666.59336.peg.2202	fig|6666666.59333.peg.3996	fig|6666666.59338.peg.990	fig|6666666.59339.peg.4217	fig|6666666.59340.peg.3795	fig|6666666.59337.peg.1278	fig|6666666.59342.peg.4632	None	None	fig|6666666.59345.peg.4468	fig|6666666.59341.peg.1077	fig|6666666.59347.peg.1282	fig|6666666.59346.peg.718	fig|6666666.59348.peg.1480	fig|6666666.59349.peg.1477	None	fig|6666666.59351.peg.1337	None	None	fig|6666666.59354.peg.2682

1366	Anti anti-sigma regulatory factor SypA	fig|6666666.59329.peg.2339	None	fig|6666666.59331.peg.5123	fig|6666666.59332.peg.4861	fig|6666666.59334.peg.2251	fig|6666666.59335.peg.2590	fig|6666666.59336.peg.4970	fig|6666666.59333.peg.4609	fig|6666666.59338.peg.1518	fig|6666666.59339.peg.1176	fig|6666666.59340.peg.1724	fig|6666666.59337.peg.4448	fig|6666666.59342.peg.6377	None	None	fig|6666666.59345.peg.3147	fig|6666666.59341.peg.2649	fig|6666666.59347.peg.3288	fig|6666666.59346.peg.5127	fig|6666666.59348.peg.2622	fig|6666666.59349.peg.2713	fig|6666666.59350.peg.457	fig|6666666.59351.peg.549	None	None	fig|6666666.59354.peg.5440

7197	FIG003461 hypothetical protein	fig|6666666.59329.peg.284	fig|6666666.59330.peg.594	None	fig|6666666.59332.peg.1194	fig|6666666.59334.peg.71	fig|6666666.59335.peg.3481	fig|6666666.59336.peg.2823	fig|6666666.59333.peg.3124	fig|6666666.59338.peg.347	fig|6666666.59339.peg.7709	fig|6666666.59340.peg.3628	fig|6666666.59337.peg.349	fig|6666666.59342.peg.3495	None	None	fig|6666666.59345.peg.2496	fig|6666666.59341.peg.790	fig|6666666.59347.peg.4860	fig|6666666.59346.peg.1967	fig|6666666.59348.peg.1202	fig|6666666.59349.peg.3638	fig|6666666.59350.peg.1857	fig|6666666.59351.peg.2243	None	None	fig|6666666.59354.peg.6314

8539	FIG002473 Protein YcaR in KDO2-Lipid A biosynthesis cluster	fig|6666666.59329.peg.3289	fig|6666666.59330.peg.1257	None	fig|6666666.59332.peg.3688	None	fig|6666666.59335.peg.2830	fig|6666666.59336.peg.3392	fig|6666666.59333.peg.1834	fig|6666666.59338.peg.2927	fig|6666666.59339.peg.3477	None	fig|6666666.59337.peg.2201	fig|6666666.59342.peg.5792	fig|6666666.71181.peg.4186	fig|6666666.59344.peg.2789	fig|6666666.59345.peg.1588	fig|6666666.59341.peg.1853	fig|6666666.59347.peg.4106	fig|6666666.59346.peg.5295	None	fig|6666666.59349.peg.2510	fig|6666666.59350.peg.7012	fig|6666666.59351.peg.3715	fig|6666666.59353.peg.1656	fig|6666666.59355.peg.1065	None

2198	FIG00921810 hypothetical protein	fig|6666666.59329.peg.5968	fig|6666666.59330.peg.3890	fig|6666666.59331.peg.4043	fig|6666666.59332.peg.4267	fig|6666666.59334.peg.5177	fig|6666666.59335.peg.1676	fig|6666666.59336.peg.2905	fig|6666666.59333.peg.3664	fig|6666666.59338.peg.5760	fig|6666666.59339.peg.3684	None	fig|6666666.59337.peg.4143	fig|6666666.59342.peg.4179	fig|6666666.71181.peg.1352	fig|6666666.59344.peg.581	fig|6666666.59345.peg.4611	fig|6666666.59341.peg.1403	fig|6666666.59347.peg.1956	fig|6666666.59346.peg.3925	fig|6666666.59348.peg.5084	None	None	None	fig|6666666.59353.peg.1881	fig|6666666.59355.peg.1132	None

2731	Protein ImpG VasA	fig|6666666.59329.peg.4242	fig|6666666.59330.peg.3116	fig|6666666.59331.peg.3297	fig|6666666.59332.peg.2969	None	fig|6666666.59335.peg.2690	fig|6666666.59336.peg.4626	fig|6666666.59333.peg.3395	fig|6666666.59338.peg.1732	fig|6666666.59339.peg.3995	None	fig|6666666.59337.peg.5334	fig|6666666.59342.peg.2711	fig|6666666.71181.peg.2272	fig|6666666.59344.peg.5877	fig|6666666.59345.peg.4111	fig|6666666.59341.peg.3634	fig|6666666.59347.peg.2414	None	fig|6666666.59348.peg.3066	fig|6666666.59349.peg.1636	None	None	fig|6666666.59353.peg.4900	fig|6666666.59355.peg.995	fig|6666666.59354.peg.408

7200	Murein endopeptidase	fig|6666666.59329.peg.5296	fig|6666666.59330.peg.3672	fig|6666666.59331.peg.5416	fig|6666666.59332.peg.1261	None	fig|6666666.59335.peg.2559	fig|6666666.59336.peg.5775	fig|6666666.59333.peg.338	fig|6666666.59338.peg.6073	fig|6666666.59339.peg.6906	None	fig|6666666.59337.peg.699	fig|6666666.59342.peg.4189	fig|6666666.71181.peg.1510	fig|6666666.59344.peg.5002	fig|6666666.59345.peg.3688	fig|6666666.59341.peg.2152	fig|6666666.59347.peg.618	fig|6666666.59346.peg.2550	fig|6666666.59348.peg.2592	None	fig|6666666.59350.peg.626	None	fig|6666666.59353.peg.5017	fig|6666666.59355.peg.231	None

6221	Permease of the drug metabolite transporter DMT superfamily	fig|6666666.59329.peg.5987	fig|6666666.59330.peg.2552	fig|6666666.59331.peg.3953	fig|6666666.59332.peg.5184	fig|6666666.59334.peg.2554	fig|6666666.59335.peg.3910	fig|6666666.59336.peg.2149	fig|6666666.59333.peg.2478	fig|6666666.59338.peg.4454	fig|6666666.59339.peg.4502	None	fig|6666666.59337.peg.2254	None	fig|6666666.71181.peg.2150	fig|6666666.59344.peg.3221	fig|6666666.59345.peg.2951	fig|6666666.59341.peg.4710	fig|6666666.59347.peg.491	fig|6666666.59346.peg.5007	fig|6666666.59348.peg.900	None	fig|6666666.59350.peg.2012	None	fig|6666666.59353.peg.2837	fig|6666666.59355.peg.3941	None

10410	FIG01200495 hypothetical protein	fig|6666666.59329.peg.4069	fig|6666666.59330.peg.4403	fig|6666666.59331.peg.6181	fig|6666666.59332.peg.3336	None	fig|6666666.59335.peg.6018	fig|6666666.59336.peg.6233	fig|6666666.59333.peg.2021	fig|6666666.59338.peg.4349	fig|6666666.59339.peg.434	fig|6666666.59340.peg.32	fig|6666666.59337.peg.2412	fig|6666666.59342.peg.4662	fig|6666666.71181.peg.2403	fig|6666666.59344.peg.788	fig|6666666.59345.peg.949	fig|6666666.59341.peg.5874	fig|6666666.59347.peg.2347	fig|6666666.59346.peg.3903	fig|6666666.59348.peg.4284	None	None	None	fig|6666666.59353.peg.1319	fig|6666666.59355.peg.2552	None

6242	Glycosyltransferase EC 2.4.1.-	fig|6666666.59329.peg.1695	fig|6666666.59330.peg.4318	fig|6666666.59331.peg.5982	fig|6666666.59332.peg.561	fig|6666666.59334.peg.2236	fig|6666666.59335.peg.5065	fig|6666666.59336.peg.2911	fig|6666666.59333.peg.118	fig|6666666.59338.peg.64	fig|6666666.59339.peg.7328	fig|6666666.59340.peg.1223	fig|6666666.59337.peg.1400	fig|6666666.59342.peg.1945	None	None	fig|6666666.59345.peg.3884	fig|6666666.59341.peg.1135	fig|6666666.59347.peg.3073	fig|6666666.59346.peg.808	fig|6666666.59348.peg.1183	fig|6666666.59349.peg.504	None	fig|6666666.59351.peg.3827	None	None	fig|6666666.59354.peg.5615

8161	Ribonucleotide reductase of class III anaerobic , large subunit EC 1.17.4.2	fig|6666666.59329.peg.4715	fig|6666666.59330.peg.772	fig|6666666.59331.peg.1552	fig|6666666.59332.peg.4417	fig|6666666.59334.peg.1940	fig|6666666.59335.peg.3461	fig|6666666.59336.peg.4571	fig|6666666.59333.peg.2778	fig|6666666.59338.peg.2948	fig|6666666.59339.peg.2792	None	fig|6666666.59337.peg.5363	fig|6666666.59342.peg.2297	fig|6666666.71181.peg.3513	fig|6666666.59344.peg.4627	fig|6666666.59345.peg.48	fig|6666666.59341.peg.4422	fig|6666666.59347.peg.912	fig|6666666.59346.peg.1208	fig|6666666.59348.peg.1867	None	None	None	fig|6666666.59353.peg.2415	fig|6666666.59355.peg.506	None

11125	DNA polymerase III epsilon subunit EC 2.7.7.7	fig|6666666.59329.peg.4503	fig|6666666.59330.peg.3651	fig|6666666.59331.peg.1834	fig|6666666.59332.peg.4068	fig|6666666.59334.peg.4386	fig|6666666.59335.peg.1042	fig|6666666.59336.peg.5914	fig|6666666.59333.peg.3259	fig|6666666.59338.peg.509	fig|6666666.59339.peg.4545	fig|6666666.59340.peg.3127	fig|6666666.59337.peg.3576	fig|6666666.59342.peg.422	None	None	fig|6666666.59345.peg.855	fig|6666666.59341.peg.1772	fig|6666666.59347.peg.2169	fig|6666666.59346.peg.5273	fig|6666666.59348.peg.2728	fig|6666666.59349.peg.3833	None	fig|6666666.59351.peg.3688	None	None	fig|6666666.59354.peg.2110

10202	Molybdenum cofactor biosynthesis protein MoaD	fig|6666666.59329.peg.5051	fig|6666666.59330.peg.1713	fig|6666666.59331.peg.2796	fig|6666666.59332.peg.1524	fig|6666666.59334.peg.300	fig|6666666.59335.peg.5486	fig|6666666.59336.peg.2034	fig|6666666.59333.peg.1329	None	fig|6666666.59339.peg.142	None	fig|6666666.59337.peg.4027	fig|6666666.59342.peg.875	fig|6666666.71181.peg.2534	fig|6666666.59344.peg.5788	fig|6666666.59345.peg.1110	fig|6666666.59341.peg.2164	fig|6666666.59347.peg.5154	fig|6666666.59346.peg.535	fig|6666666.59348.peg.719	None	fig|6666666.59350.peg.7873	None	fig|6666666.59353.peg.2144	fig|6666666.59355.peg.2675	None

404	Molybdopterin binding motif, CinA N-terminal domain C-terminal domain of CinA type E	fig|6666666.59329.peg.6557	fig|6666666.59330.peg.2323	fig|6666666.59331.peg.1033	fig|6666666.59332.peg.5686	fig|6666666.59334.peg.4901	fig|6666666.59335.peg.1323	fig|6666666.59336.peg.5400	fig|6666666.59333.peg.3807	fig|6666666.59338.peg.2401	fig|6666666.59339.peg.937	None	fig|6666666.59337.peg.1750	None	fig|6666666.71181.peg.510	fig|6666666.59344.peg.1731	fig|6666666.59345.peg.895	fig|6666666.59341.peg.3771	fig|6666666.59347.peg.2211	fig|6666666.59346.peg.1351	fig|6666666.59348.peg.3319	None	fig|6666666.59350.peg.25	None	fig|6666666.59353.peg.1137	fig|6666666.59355.peg.339	None

1731	LSU ribosomal protein L13p L13Ae	fig|6666666.59329.peg.2059	fig|6666666.59330.peg.961	fig|6666666.59331.peg.793	fig|6666666.59332.peg.651	fig|6666666.59334.peg.3195	None	None	fig|6666666.59333.peg.620	None	fig|6666666.59339.peg.4938	None	fig|6666666.59337.peg.27	fig|6666666.59342.peg.5748	fig|6666666.71181.peg.1040	fig|6666666.59344.peg.1822	fig|6666666.59345.peg.3815	fig|6666666.59341.peg.3549	fig|6666666.59347.peg.3920	fig|6666666.59346.peg.2338	None	fig|6666666.59349.peg.39	fig|6666666.59350.peg.5607	fig|6666666.59351.peg.3459	fig|6666666.59353.peg.2358	fig|6666666.59355.peg.1631	fig|6666666.59354.peg.355

1450	SSU ribosomal protein S14p S29e SSU ribosomal protein S14p S29e , zinc-independent	fig|6666666.59329.peg.722	fig|6666666.59330.peg.563	fig|6666666.59331.peg.39	fig|6666666.59332.peg.2148	None	fig|6666666.59335.peg.4356	fig|6666666.59336.peg.155	fig|6666666.59333.peg.375	fig|6666666.59338.peg.1380	fig|6666666.59339.peg.8276	fig|6666666.59340.peg.65	fig|6666666.59337.peg.379	fig|6666666.59342.peg.313	None	None	fig|6666666.59345.peg.2526	fig|6666666.59341.peg.2507	fig|6666666.59347.peg.4832	fig|6666666.59346.peg.859	fig|6666666.59348.peg.1769	fig|6666666.59349.peg.3279	fig|6666666.59350.peg.3184	fig|6666666.59351.peg.4213	None	None	fig|6666666.59354.peg.3852

7177	hypothetical protein	fig|6666666.59329.peg.1815	fig|6666666.59330.peg.1133	fig|6666666.59331.peg.1178	fig|6666666.59332.peg.3286	fig|6666666.59334.peg.5271	fig|6666666.59335.peg.3938	fig|6666666.59336.peg.6408	fig|6666666.59333.peg.5264	fig|6666666.59338.peg.5192	fig|6666666.59339.peg.7066	None	fig|6666666.59337.peg.3626	fig|6666666.59342.peg.1654	None	None	fig|6666666.59345.peg.1805	fig|6666666.59341.peg.2472	fig|6666666.59347.peg.903	fig|6666666.59346.peg.5307	fig|6666666.59348.peg.1548	fig|6666666.59349.peg.783	fig|6666666.59350.peg.5442	fig|6666666.59351.peg.720	None	None	fig|6666666.59354.peg.3016

2287	Maltoporin maltose maltodextrin high-affinity receptor, phage lambda receptor protein	fig|6666666.59329.peg.5814	fig|6666666.59330.peg.3590	fig|6666666.59331.peg.547	fig|6666666.59332.peg.399	fig|6666666.59334.peg.2576	fig|6666666.59335.peg.5450	fig|6666666.59336.peg.2394	fig|6666666.59333.peg.1067	fig|6666666.59338.peg.2585	fig|6666666.59339.peg.829	None	fig|6666666.59337.peg.2894	fig|6666666.59342.peg.1712	fig|6666666.71181.peg.2009	fig|6666666.59344.peg.2732	fig|6666666.59345.peg.1958	fig|6666666.59341.peg.6091	fig|6666666.59347.peg.3392	fig|6666666.59346.peg.1721	fig|6666666.59348.peg.3409	None	None	None	fig|6666666.59353.peg.3006	fig|6666666.59355.peg.2913	None

3057	Sodium-dependent transporter	fig|6666666.59329.peg.5532	fig|6666666.59330.peg.1853	fig|6666666.59331.peg.1302	fig|6666666.59332.peg.5323	fig|6666666.59334.peg.4269	fig|6666666.59335.peg.4193	fig|6666666.59336.peg.2415	fig|6666666.59333.peg.2262	fig|6666666.59338.peg.276	fig|6666666.59339.peg.539	fig|6666666.59340.peg.2128	fig|6666666.59337.peg.2676	fig|6666666.59342.peg.2124	None	None	fig|6666666.59345.peg.1052	fig|6666666.59341.peg.28	fig|6666666.59347.peg.4152	fig|6666666.59346.peg.1278	fig|6666666.59348.peg.4688	fig|6666666.59349.peg.2684	None	fig|6666666.59351.peg.4931	None	None	fig|6666666.59354.peg.1296

6440	Heme A synthase, cytochrome oxidase biogenesis protein Cox15-CtaA	fig|6666666.59329.peg.6048	fig|6666666.59330.peg.1120	fig|6666666.59331.peg.611	fig|6666666.59332.peg.6249	fig|6666666.59334.peg.1001	fig|6666666.59335.peg.2864	fig|6666666.59336.peg.6072	fig|6666666.59333.peg.5454	fig|6666666.59338.peg.355	fig|6666666.59339.peg.6630	fig|6666666.59340.peg.3552	fig|6666666.59337.peg.5079	fig|6666666.59342.peg.5323	None	None	fig|6666666.59345.peg.5622	None	fig|6666666.59347.peg.1546	fig|6666666.59346.peg.1046	fig|6666666.59348.peg.1804	fig|6666666.59349.peg.3503	fig|6666666.59350.peg.135	fig|6666666.59351.peg.4383	None	None	fig|6666666.59354.peg.2073

10016	FIG002577 Putative lipoprotein precursor	fig|6666666.59329.peg.6295	fig|6666666.59330.peg.2872	fig|6666666.59331.peg.5924	fig|6666666.59332.peg.4990	fig|6666666.59334.peg.2694	fig|6666666.59335.peg.603	fig|6666666.59336.peg.4706	fig|6666666.59333.peg.4983	fig|6666666.59338.peg.405	fig|6666666.59339.peg.478	fig|6666666.59340.peg.3033	fig|6666666.59337.peg.5582	None	None	None	fig|6666666.59345.peg.1317	fig|6666666.59341.peg.487	fig|6666666.59347.peg.4130	fig|6666666.59346.peg.4936	fig|6666666.59348.peg.5087	fig|6666666.59349.peg.4304	fig|6666666.59350.peg.3642	fig|6666666.59351.peg.1091	None	None	fig|6666666.59354.peg.3925

4203	Putative efflux PET family inner membrane protein YccS	fig|6666666.59329.peg.4189	fig|6666666.59330.peg.3669	fig|6666666.59331.peg.2702	fig|6666666.59332.peg.6140	fig|6666666.59334.peg.2410	fig|6666666.59335.peg.5405	fig|6666666.59336.peg.213	fig|6666666.59333.peg.5473	fig|6666666.59338.peg.4212	fig|6666666.59339.peg.2829	None	fig|6666666.59337.peg.781	fig|6666666.59342.peg.663	fig|6666666.71181.peg.1809	fig|6666666.59344.peg.3093	fig|6666666.59345.peg.3295	fig|6666666.59341.peg.2478	fig|6666666.59347.peg.894	fig|6666666.59346.peg.3168	fig|6666666.59348.peg.4950	None	None	None	fig|6666666.59353.peg.1698	fig|6666666.59355.peg.1022	None

4851	FIG00922491 hypothetical protein	fig|6666666.59329.peg.2413	fig|6666666.59330.peg.1589	fig|6666666.59331.peg.1896	fig|6666666.59332.peg.1953	None	fig|6666666.59335.peg.3937	fig|6666666.59336.peg.5508	fig|6666666.59333.peg.1970	fig|6666666.59338.peg.1094	fig|6666666.59339.peg.7063	None	fig|6666666.59337.peg.4842	None	fig|6666666.71181.peg.1507	fig|6666666.59344.peg.5005	fig|6666666.59345.peg.5291	fig|6666666.59341.peg.836	fig|6666666.59347.peg.5118	fig|6666666.59346.peg.2656	fig|6666666.59348.peg.1547	fig|6666666.59349.peg.4075	None	fig|6666666.59351.peg.711	fig|6666666.59353.peg.5014	fig|6666666.59355.peg.234	None

10117	Transcriptional regulator for fatty acid degradation FadR, GntR family	fig|6666666.59329.peg.999	fig|6666666.59330.peg.3951	None	fig|6666666.59332.peg.2115	fig|6666666.59334.peg.2533	fig|6666666.59335.peg.1302	fig|6666666.59336.peg.5172	fig|6666666.59333.peg.1540	fig|6666666.59338.peg.3863	fig|6666666.59339.peg.3433	None	fig|6666666.59337.peg.3845	None	fig|6666666.71181.peg.2950	fig|6666666.59344.peg.2870	fig|6666666.59345.peg.1083	fig|6666666.59341.peg.2387	fig|6666666.59347.peg.2623	fig|6666666.59346.peg.5326	None	fig|6666666.59349.peg.2724	None	fig|6666666.59351.peg.567	fig|6666666.59353.peg.1454	fig|6666666.59355.peg.2003	fig|6666666.59354.peg.117

8971	Oxidoreductase, short-chain dehydrogenase reductase family EC 1.1.1.-	fig|6666666.59329.peg.2101	fig|6666666.59330.peg.2879	fig|6666666.59331.peg.6264	fig|6666666.59332.peg.3671	fig|6666666.59334.peg.5460	fig|6666666.59335.peg.609	fig|6666666.59336.peg.4949	fig|6666666.59333.peg.3153	fig|6666666.59338.peg.1414	fig|6666666.59339.peg.471	None	fig|6666666.59337.peg.4606	fig|6666666.59342.peg.2458	None	None	fig|6666666.59345.peg.1323	fig|6666666.59341.peg.481	fig|6666666.59347.peg.4123	fig|6666666.59346.peg.2981	fig|6666666.59348.peg.4414	fig|6666666.59349.peg.940	fig|6666666.59350.peg.363	fig|6666666.59351.peg.1098	None	None	fig|6666666.59354.peg.1696

7132	Pyruvate formate-lyase activating enzyme EC 1.97.1.4	fig|6666666.59329.peg.3716	fig|6666666.59330.peg.1264	None	fig|6666666.59332.peg.5840	fig|6666666.59334.peg.453	fig|6666666.59335.peg.2820	fig|6666666.59336.peg.466	fig|6666666.59333.peg.3022	fig|6666666.59338.peg.3826	fig|6666666.59339.peg.956	fig|6666666.59340.peg.1085	fig|6666666.59337.peg.2208	fig|6666666.59342.peg.1091	fig|6666666.71181.peg.3159	fig|6666666.59344.peg.2811	fig|6666666.59345.peg.1829	fig|6666666.59341.peg.5380	fig|6666666.59347.peg.1593	fig|6666666.59346.peg.3241	fig|6666666.59348.peg.2042	None	None	None	fig|6666666.59353.peg.1637	fig|6666666.59355.peg.1202	None

2127	Glycerate kinase EC 2.7.1.31	fig|6666666.59329.peg.5457	fig|6666666.59330.peg.3255	fig|6666666.59331.peg.1516	fig|6666666.59332.peg.2915	fig|6666666.59334.peg.5566	fig|6666666.59335.peg.3175	fig|6666666.59336.peg.4757	fig|6666666.59333.peg.2931	fig|6666666.59338.peg.2996	fig|6666666.59339.peg.1040	fig|6666666.59340.peg.41	fig|6666666.59337.peg.1247	None	fig|6666666.71181.peg.1943	fig|6666666.59344.peg.5040	fig|6666666.59345.peg.4429	fig|6666666.59341.peg.1317	fig|6666666.59347.peg.3161	fig|6666666.59346.peg.5047	fig|6666666.59348.peg.2410	None	None	None	fig|6666666.59353.peg.4069	fig|6666666.59355.peg.4145	None

4878	Phosphoserine phosphatase EC 3.1.3.3	fig|6666666.59329.peg.6256	fig|6666666.59330.peg.2390	fig|6666666.59331.peg.2515	fig|6666666.59332.peg.3295	fig|6666666.59334.peg.4038	fig|6666666.59335.peg.5416	fig|6666666.59336.peg.792	fig|6666666.59333.peg.4028	fig|6666666.59338.peg.3185	fig|6666666.59339.peg.2221	None	fig|6666666.59337.peg.3997	fig|6666666.59342.peg.5295	fig|6666666.71181.peg.3845	fig|6666666.59344.peg.324	fig|6666666.59345.peg.1707	None	fig|6666666.59347.peg.4690	fig|6666666.59346.peg.5320	fig|6666666.59348.peg.95	None	None	fig|6666666.59351.peg.177	fig|6666666.59353.peg.558	fig|6666666.59355.peg.43	None

4836	Predicted P-loop ATPase fused to an acetyltransferase C1444	fig|6666666.59329.peg.664	fig|6666666.59330.peg.1897	fig|6666666.59331.peg.1890	fig|6666666.59332.peg.440	fig|6666666.59334.peg.1710	fig|6666666.59335.peg.4138	None	fig|6666666.59333.peg.5209	fig|6666666.59338.peg.1370	fig|6666666.59339.peg.5459	fig|6666666.59340.peg.304	fig|6666666.59337.peg.245	None	fig|6666666.71181.peg.4373	None	fig|6666666.59345.peg.5255	fig|6666666.59341.peg.1386	fig|6666666.59347.peg.16	fig|6666666.59346.peg.2557	fig|6666666.59348.peg.4026	fig|6666666.59349.peg.2269	None	fig|6666666.59351.peg.4830	fig|6666666.59353.peg.4906	None	fig|6666666.59354.peg.1848

2046	FIG01199897 hypothetical protein	fig|6666666.59329.peg.6457	fig|6666666.59330.peg.1946	fig|6666666.59331.peg.3986	fig|6666666.59332.peg.5474	fig|6666666.59334.peg.5125	fig|6666666.59335.peg.1800	fig|6666666.59336.peg.5083	fig|6666666.59333.peg.5179	fig|6666666.59338.peg.3193	fig|6666666.59339.peg.3614	None	fig|6666666.59337.peg.5455	fig|6666666.59342.peg.843	None	None	fig|6666666.59345.peg.717	fig|6666666.59341.peg.3521	fig|6666666.59347.peg.1688	fig|6666666.59346.peg.2421	fig|6666666.59348.peg.1452	fig|6666666.59349.peg.4685	fig|6666666.59350.peg.1118	fig|6666666.59351.peg.2801	None	None	fig|6666666.59354.peg.4527

1606	Formate dehydrogenase chain D EC 1.2.1.2	fig|6666666.59329.peg.5151	fig|6666666.59330.peg.2586	fig|6666666.59331.peg.2356	fig|6666666.59332.peg.6064	fig|6666666.59334.peg.4601	fig|6666666.59335.peg.5747	fig|6666666.59336.peg.4877	fig|6666666.59333.peg.2456	fig|6666666.59338.peg.5159	fig|6666666.59339.peg.1274	None	fig|6666666.59337.peg.2729	fig|6666666.59342.peg.2885	fig|6666666.71181.peg.4709	fig|6666666.59344.peg.5790	fig|6666666.59345.peg.3206	fig|6666666.59341.peg.2903	fig|6666666.59347.peg.1714	fig|6666666.59346.peg.3279	fig|6666666.59348.peg.3912	None	None	None	fig|6666666.59353.peg.4952	fig|6666666.59355.peg.5035	None

6562	2-C-methyl-D-erythritol 4-phosphate cytidylyltransferase EC 2.7.7.60	None	fig|6666666.59330.peg.2463	None	fig|6666666.59332.peg.199	fig|6666666.59334.peg.334	fig|6666666.59335.peg.1655	fig|6666666.59336.peg.4983	fig|6666666.59333.peg.4777	fig|6666666.59338.peg.223	fig|6666666.59339.peg.7917	None	fig|6666666.59337.peg.578	fig|6666666.59342.peg.1276	fig|6666666.71181.peg.3416	fig|6666666.59344.peg.2197	fig|6666666.59345.peg.5069	fig|6666666.59341.peg.656	fig|6666666.59347.peg.3949	fig|6666666.59346.peg.3149	None	fig|6666666.59349.peg.4381	None	fig|6666666.59351.peg.5478	fig|6666666.59353.peg.209	fig|6666666.59355.peg.2978	fig|6666666.59354.peg.3140

5510	Na+ H+ antiporter NhaA type	fig|6666666.59329.peg.5891	fig|6666666.59330.peg.3853	fig|6666666.59331.peg.842	fig|6666666.59332.peg.1227	fig|6666666.59334.peg.5128	fig|6666666.59335.peg.1337	fig|6666666.59336.peg.4349	fig|6666666.59333.peg.3425	None	fig|6666666.59339.peg.919	None	fig|6666666.59337.peg.4277	fig|6666666.59342.peg.711	fig|6666666.71181.peg.5721	fig|6666666.59344.peg.1321	fig|6666666.59345.peg.1211	fig|6666666.59341.peg.346	fig|6666666.59347.peg.1989	fig|6666666.59346.peg.4497	fig|6666666.59348.peg.4959	None	fig|6666666.59350.peg.6286	None	fig|6666666.59353.peg.74	fig|6666666.59355.peg.681	None

9996	Aminopeptidase N	fig|6666666.59329.peg.3219	fig|6666666.59330.peg.998	fig|6666666.59331.peg.5983	fig|6666666.59332.peg.272	fig|6666666.59334.peg.413	fig|6666666.59335.peg.4457	fig|6666666.59336.peg.3811	fig|6666666.59333.peg.3884	fig|6666666.59338.peg.3007	fig|6666666.59339.peg.7672	fig|6666666.59340.peg.467	fig|6666666.59337.peg.2531	fig|6666666.59342.peg.5635	None	None	fig|6666666.59345.peg.5524	fig|6666666.59341.peg.4157	fig|6666666.59347.peg.5238	fig|6666666.59346.peg.5079	fig|6666666.59348.peg.2236	fig|6666666.59349.peg.2414	None	fig|6666666.59351.peg.396	None	None	fig|6666666.59354.peg.3994

11031	FIG111991 hypothetical protein	fig|6666666.59329.peg.1042	fig|6666666.59330.peg.2793	fig|6666666.59331.peg.5705	fig|6666666.59332.peg.4036	fig|6666666.59334.peg.1279	fig|6666666.59335.peg.112	fig|6666666.59336.peg.3468	fig|6666666.59333.peg.1931	fig|6666666.59338.peg.2004	fig|6666666.59339.peg.5036	fig|6666666.59340.peg.1804	fig|6666666.59337.peg.2373	None	None	None	fig|6666666.59345.peg.2245	fig|6666666.59341.peg.5025	fig|6666666.59347.peg.2809	fig|6666666.59346.peg.2989	fig|6666666.59348.peg.2931	fig|6666666.59349.peg.945	fig|6666666.59350.peg.371	fig|6666666.59351.peg.1100	None	None	fig|6666666.59354.peg.1691

9351	Type cbb3 cytochrome oxidase biogenesis protein CcoG, involved in Cu oxidation	fig|6666666.59329.peg.2578	fig|6666666.59330.peg.632	None	fig|6666666.59332.peg.216	fig|6666666.59334.peg.1394	fig|6666666.59335.peg.2443	fig|6666666.59336.peg.2739	fig|6666666.59333.peg.4	fig|6666666.59338.peg.4851	fig|6666666.59339.peg.8079	fig|6666666.59340.peg.1392	fig|6666666.59337.peg.3891	fig|6666666.59342.peg.2029	None	None	fig|6666666.59345.peg.4937	fig|6666666.59341.peg.4176	fig|6666666.59347.peg.4756	fig|6666666.59346.peg.4411	fig|6666666.59348.peg.59	fig|6666666.59349.peg.4296	fig|6666666.59350.peg.1952	fig|6666666.59351.peg.4333	None	None	fig|6666666.59354.peg.6025

4178	FIG00920084 hypothetical protein	fig|6666666.59329.peg.2700	fig|6666666.59330.peg.2933	None	fig|6666666.59332.peg.3759	None	fig|6666666.59335.peg.3843	fig|6666666.59336.peg.876	fig|6666666.59333.peg.4234	fig|6666666.59338.peg.3281	fig|6666666.59339.peg.567	None	fig|6666666.59337.peg.1733	None	fig|6666666.71181.peg.2772	fig|6666666.59344.peg.432	fig|6666666.59345.peg.909	fig|6666666.59341.peg.5350	fig|6666666.59347.peg.2298	fig|6666666.59346.peg.1611	fig|6666666.59348.peg.3888	fig|6666666.59349.peg.2066	None	fig|6666666.59351.peg.583	fig|6666666.59353.peg.1754	fig|6666666.59355.peg.1258	fig|6666666.59354.peg.1393

5920	Fumarate reductase subunit C	fig|6666666.59329.peg.5433	fig|6666666.59330.peg.154	fig|6666666.59331.peg.2528	fig|6666666.59332.peg.746	fig|6666666.59334.peg.4004	fig|6666666.59335.peg.440	fig|6666666.59336.peg.260	fig|6666666.59333.peg.433	fig|6666666.59338.peg.811	fig|6666666.59339.peg.6397	None	fig|6666666.59337.peg.5189	fig|6666666.59342.peg.2305	fig|6666666.71181.peg.1686	fig|6666666.59344.peg.606	fig|6666666.59345.peg.5106	fig|6666666.59341.peg.169	fig|6666666.59347.peg.4556	fig|6666666.59346.peg.972	fig|6666666.59348.peg.150	None	None	None	fig|6666666.59353.peg.1056	fig|6666666.59355.peg.3562	None

7310	AttH component of AttEFGH ABC transport system	fig|6666666.59329.peg.4995	fig|6666666.59330.peg.4334	fig|6666666.59331.peg.5382	fig|6666666.59332.peg.5094	fig|6666666.59334.peg.4338	fig|6666666.59335.peg.1338	fig|6666666.59336.peg.646	fig|6666666.59333.peg.2656	fig|6666666.59338.peg.5148	fig|6666666.59339.peg.918	None	fig|6666666.59337.peg.4276	fig|6666666.59342.peg.4027	None	None	fig|6666666.59345.peg.1212	fig|6666666.59341.peg.879	fig|6666666.59347.peg.2101	fig|6666666.59346.peg.1546	fig|6666666.59348.peg.750	fig|6666666.59349.peg.2343	fig|6666666.59350.peg.813	fig|6666666.59351.peg.3992	None	None	fig|6666666.59354.peg.2096

10823	Thiamine kinase EC 2.7.1.89 Adenosylcobinamide kinase EC 2.7.1.156	fig|6666666.59329.peg.5646	fig|6666666.59330.peg.1240	fig|6666666.59331.peg.5142	fig|6666666.59332.peg.4924	None	fig|6666666.59335.peg.1311	fig|6666666.59336.peg.1621	fig|6666666.59333.peg.1873	fig|6666666.59338.peg.1567	fig|6666666.59339.peg.3447	fig|6666666.59340.peg.3759	fig|6666666.59337.peg.2183	fig|6666666.59342.peg.494	fig|6666666.71181.peg.1640	fig|6666666.59344.peg.5019	fig|6666666.59345.peg.1604	fig|6666666.59341.peg.268	fig|6666666.59347.peg.4071	fig|6666666.59346.peg.4966	fig|6666666.59348.peg.816	None	None	None	fig|6666666.59353.peg.333	fig|6666666.59355.peg.1372	None

3860	Predicted signal transduction protein	fig|6666666.59329.peg.2351	fig|6666666.59330.peg.1510	fig|6666666.59331.peg.727	fig|6666666.59332.peg.1804	fig|6666666.59334.peg.3972	fig|6666666.59335.peg.4936	fig|6666666.59336.peg.5020	fig|6666666.59333.peg.5225	fig|6666666.59338.peg.1121	fig|6666666.59339.peg.8544	fig|6666666.59340.peg.560	fig|6666666.59337.peg.4876	fig|6666666.59342.peg.2272	None	None	fig|6666666.59345.peg.5488	fig|6666666.59341.peg.3620	fig|6666666.59347.peg.5065	fig|6666666.59346.peg.615	fig|6666666.59348.peg.741	fig|6666666.59349.peg.957	None	fig|6666666.59351.peg.2096	None	None	fig|6666666.59354.peg.783

5905	Electron transport complex protein RnfE	fig|6666666.59329.peg.785	fig|6666666.59330.peg.3377	None	fig|6666666.59332.peg.2571	fig|6666666.59334.peg.4174	fig|6666666.59335.peg.883	fig|6666666.59336.peg.4282	fig|6666666.59333.peg.4814	fig|6666666.59338.peg.4299	fig|6666666.59339.peg.53	fig|6666666.59340.peg.2887	fig|6666666.59337.peg.4669	None	fig|6666666.71181.peg.2904	fig|6666666.59344.peg.3460	fig|6666666.59345.peg.1126	None	fig|6666666.59347.peg.5388	fig|6666666.59346.peg.4768	fig|6666666.59348.peg.88	fig|6666666.59349.peg.4634	None	fig|6666666.59351.peg.5074	fig|6666666.59353.peg.1416	fig|6666666.59355.peg.2458	None

7018	Cytochrome c551 peroxidase EC 1.11.1.5	fig|6666666.59329.peg.3738	fig|6666666.59330.peg.1913	fig|6666666.59331.peg.5591	fig|6666666.59332.peg.3716	fig|6666666.59334.peg.2217	fig|6666666.59335.peg.4951	fig|6666666.59336.peg.4598	fig|6666666.59333.peg.454	fig|6666666.59338.peg.3001	fig|6666666.59339.peg.859	fig|6666666.59340.peg.632	fig|6666666.59337.peg.2167	fig|6666666.59342.peg.5030	None	None	fig|6666666.59345.peg.3986	fig|6666666.59341.peg.4685	fig|6666666.59347.peg.1509	None	fig|6666666.59348.peg.3814	fig|6666666.59349.peg.4400	fig|6666666.59350.peg.839	fig|6666666.59351.peg.5105	None	None	fig|6666666.59354.peg.4744

1027	NADH dehydrogenase EC 1.6.99.3	fig|6666666.59329.peg.4437	fig|6666666.59330.peg.3700	fig|6666666.59331.peg.5090	fig|6666666.59332.peg.4195	fig|6666666.59334.peg.3658	fig|6666666.59335.peg.1315	fig|6666666.59336.peg.4262	fig|6666666.59333.peg.1879	fig|6666666.59338.peg.4736	fig|6666666.59339.peg.3449	None	fig|6666666.59337.peg.2185	fig|6666666.59342.peg.496	fig|6666666.71181.peg.1644	fig|6666666.59344.peg.5015	fig|6666666.59345.peg.1602	fig|6666666.59341.peg.1098	fig|6666666.59347.peg.4073	fig|6666666.59346.peg.4455	None	None	fig|6666666.59350.peg.82	None	fig|6666666.59353.peg.336	fig|6666666.59355.peg.1375	None

4099	hypothetical protein PA3071	fig|6666666.59329.peg.4299	fig|6666666.59330.peg.2526	fig|6666666.59331.peg.2134	fig|6666666.59332.peg.4022	fig|6666666.59334.peg.4832	fig|6666666.59335.peg.5363	fig|6666666.59336.peg.3300	fig|6666666.59333.peg.4807	fig|6666666.59338.peg.4897	fig|6666666.59339.peg.4369	fig|6666666.59340.peg.3261	fig|6666666.59337.peg.2559	fig|6666666.59342.peg.6250	None	None	fig|6666666.59345.peg.5642	fig|6666666.59341.peg.5453	fig|6666666.59347.peg.1485	fig|6666666.59346.peg.732	None	fig|6666666.59349.peg.4068	fig|6666666.59350.peg.7133	fig|6666666.59351.peg.700	None	None	fig|6666666.59354.peg.5243

8096	Secreted trypsin-like serine protease	fig|6666666.59329.peg.1716	fig|6666666.59330.peg.5118	fig|6666666.59331.peg.4291	fig|6666666.59332.peg.4483	fig|6666666.59334.peg.4348	fig|6666666.59335.peg.3106	fig|6666666.59336.peg.3098	fig|6666666.59333.peg.5528	fig|6666666.59338.peg.1401	fig|6666666.59339.peg.410	fig|6666666.59340.peg.2060	fig|6666666.59337.peg.3447	fig|6666666.59342.peg.4925	None	None	fig|6666666.59345.peg.4092	fig|6666666.59341.peg.5798	fig|6666666.59347.peg.1400	fig|6666666.59346.peg.3489	fig|6666666.59348.peg.5252	fig|6666666.59349.peg.972	None	fig|6666666.59351.peg.1867	None	None	fig|6666666.59354.peg.496

6654	SSU ribosomal protein S1p	None	fig|6666666.59330.peg.1387	fig|6666666.59331.peg.2070	None	fig|6666666.59334.peg.1077	fig|6666666.59335.peg.3369	fig|6666666.59336.peg.431	fig|6666666.59333.peg.736	fig|6666666.59338.peg.1338	fig|6666666.59339.peg.3110	None	fig|6666666.59337.peg.2877	fig|6666666.59342.peg.1797	fig|6666666.71181.peg.3167	fig|6666666.59344.peg.2800	fig|6666666.59345.peg.1036	fig|6666666.59341.peg.47	fig|6666666.59347.peg.2407	fig|6666666.59346.peg.4945	None	fig|6666666.59349.peg.377	None	fig|6666666.59351.peg.942	fig|6666666.59353.peg.1647	fig|6666666.59355.peg.1074	fig|6666666.59354.peg.4423

10801	Isocitrate dehydrogenase [NADP] EC 1.1.1.42 ; Monomeric isocitrate dehydrogenase [NADP] EC 1.1.1.42	None	fig|6666666.59330.peg.1315	fig|6666666.59331.peg.6356	fig|6666666.59332.peg.4343	fig|6666666.59334.peg.3659	fig|6666666.59335.peg.5595	fig|6666666.59336.peg.859	fig|6666666.59333.peg.1011	fig|6666666.59338.peg.4182	fig|6666666.59339.peg.344	fig|6666666.59340.peg.1707	fig|6666666.59337.peg.3375	fig|6666666.59342.peg.2497	None	None	fig|6666666.59345.peg.1557	fig|6666666.59341.peg.1856	fig|6666666.59347.peg.4109	fig|6666666.59346.peg.2195	fig|6666666.59348.peg.1383	fig|6666666.59349.peg.2890	fig|6666666.59350.peg.4857	fig|6666666.59351.peg.874	None	None	fig|6666666.59354.peg.3760

616	Adenosylcobinamide-phosphate synthase	fig|6666666.59329.peg.2768	fig|6666666.59330.peg.1295	fig|6666666.59331.peg.5253	fig|6666666.59332.peg.1116	None	fig|6666666.59335.peg.5131	fig|6666666.59336.peg.4511	fig|6666666.59333.peg.1503	fig|6666666.59338.peg.1287	fig|6666666.59339.peg.7423	fig|6666666.59340.peg.285	fig|6666666.59337.peg.69	fig|6666666.59342.peg.3270	None	None	fig|6666666.59345.peg.3774	fig|6666666.59341.peg.70	fig|6666666.59347.peg.4017	fig|6666666.59346.peg.1856	fig|6666666.59348.peg.5023	fig|6666666.59349.peg.1669	fig|6666666.59350.peg.1382	fig|6666666.59351.peg.287	None	None	fig|6666666.59354.peg.818

8428	SSU ribosomal protein S12p S23e	None	fig|6666666.59330.peg.4071	fig|6666666.59331.peg.4325	fig|6666666.59332.peg.1078	fig|6666666.59334.peg.442	fig|6666666.59335.peg.5580	fig|6666666.59336.peg.2178	fig|6666666.59333.peg.3102	fig|6666666.59338.peg.3252	fig|6666666.59339.peg.8874	fig|6666666.59340.peg.894	fig|6666666.59337.peg.1824	fig|6666666.59342.peg.1924	None	None	fig|6666666.59345.peg.5551	fig|6666666.59341.peg.5090	fig|6666666.59347.peg.5331	fig|6666666.59346.peg.46	fig|6666666.59348.peg.3177	fig|6666666.59349.peg.1139	fig|6666666.59350.peg.3773	fig|6666666.59351.peg.5246	None	None	fig|6666666.59354.peg.3580

9839	Diadenosine tetraphosphate Ap4A hydrolase and other HIT family hydrolases	fig|6666666.59329.peg.5181	fig|6666666.59330.peg.4511	fig|6666666.59331.peg.1503	fig|6666666.59332.peg.3928	fig|6666666.59334.peg.5194	fig|6666666.59335.peg.4147	fig|6666666.59336.peg.1737	fig|6666666.59333.peg.1337	fig|6666666.59338.peg.2373	fig|6666666.59339.peg.2993	fig|6666666.59340.peg.1411	fig|6666666.59337.peg.2734	None	None	None	fig|6666666.59345.peg.4310	fig|6666666.59341.peg.5058	fig|6666666.59347.peg.4401	fig|6666666.59346.peg.4284	fig|6666666.59348.peg.2687	fig|6666666.59349.peg.3852	None	fig|6666666.59351.peg.4265	None	fig|6666666.59355.peg.5441	fig|6666666.59354.peg.1162

4545	Phage shock protein C	fig|6666666.59329.peg.4967	fig|6666666.59330.peg.1840	None	None	None	fig|6666666.59335.peg.4630	fig|6666666.59336.peg.5893	fig|6666666.59333.peg.5108	fig|6666666.59338.peg.1641	fig|6666666.59339.peg.523	None	fig|6666666.59337.peg.2330	fig|6666666.59342.peg.3324	fig|6666666.71181.peg.1387	fig|6666666.59344.peg.534	fig|6666666.59345.peg.1266	fig|6666666.59341.peg.1037	fig|6666666.59347.peg.3538	fig|6666666.59346.peg.1291	fig|6666666.59348.peg.2618	fig|6666666.59349.peg.2086	None	fig|6666666.59351.peg.2599	fig|6666666.59353.peg.1841	fig|6666666.59355.peg.1173	fig|6666666.59354.peg.6442

5752	Transcriptional regulator, MarR family	fig|6666666.59329.peg.5861	fig|6666666.59330.peg.3355	fig|6666666.59331.peg.2491	fig|6666666.59332.peg.4150	None	fig|6666666.59335.peg.1045	fig|6666666.59336.peg.5267	fig|6666666.59333.peg.2728	fig|6666666.59338.peg.5011	fig|6666666.59339.peg.4540	None	fig|6666666.59337.peg.3579	fig|6666666.59342.peg.2588	fig|6666666.71181.peg.5047	fig|6666666.59344.peg.1426	fig|6666666.59345.peg.858	fig|6666666.59341.peg.1765	fig|6666666.59347.peg.1330	fig|6666666.59346.peg.4533	fig|6666666.59348.peg.4091	None	fig|6666666.59350.peg.2020	None	fig|6666666.59353.peg.2	fig|6666666.59355.peg.607	None

2362	Probable 3-phenylpropionic acid transporter	fig|6666666.59329.peg.1169	fig|6666666.59330.peg.187	fig|6666666.59331.peg.447	fig|6666666.59332.peg.4380	fig|6666666.59334.peg.39	fig|6666666.59335.peg.2617	fig|6666666.59336.peg.1294	fig|6666666.59333.peg.2203	fig|6666666.59338.peg.3123	fig|6666666.59339.peg.6455	None	fig|6666666.59337.peg.4295	fig|6666666.59342.peg.6483	fig|6666666.71181.peg.1256	fig|6666666.59344.peg.148	fig|6666666.59345.peg.5139	fig|6666666.59341.peg.1173	fig|6666666.59347.peg.4501	fig|6666666.59346.peg.1270	fig|6666666.59348.peg.868	None	None	None	fig|6666666.59353.peg.2253	fig|6666666.59355.peg.1792	None

3723	NADPH dependent preQ0 reductase EC 1.7.1.13	fig|6666666.59329.peg.2942	fig|6666666.59330.peg.3893	None	fig|6666666.59332.peg.395	fig|6666666.59334.peg.1905	fig|6666666.59335.peg.349	fig|6666666.59336.peg.3262	fig|6666666.59333.peg.3271	fig|6666666.59338.peg.2026	fig|6666666.59339.peg.7149	None	fig|6666666.59337.peg.1116	fig|6666666.59342.peg.5326	fig|6666666.71181.peg.48	fig|6666666.59344.peg.2630	fig|6666666.59345.peg.3519	fig|6666666.59341.peg.2859	fig|6666666.59347.peg.2927	fig|6666666.59346.peg.1725	None	fig|6666666.59349.peg.1342	None	fig|6666666.59351.peg.4234	fig|6666666.59353.peg.1969	fig|6666666.59355.peg.3794	None

10485	RNA-binding protein	fig|6666666.59329.peg.223	fig|6666666.59330.peg.4568	fig|6666666.59331.peg.2720	fig|6666666.59332.peg.577	fig|6666666.59334.peg.2250	fig|6666666.59335.peg.5810	fig|6666666.59336.peg.3238	fig|6666666.59333.peg.1462	fig|6666666.59338.peg.930	fig|6666666.59339.peg.8601	fig|6666666.59340.peg.3132	fig|6666666.59337.peg.282	fig|6666666.59342.peg.5311	None	None	fig|6666666.59345.peg.2135	fig|6666666.59341.peg.4367	fig|6666666.59347.peg.4659	fig|6666666.59346.peg.5332	fig|6666666.59348.peg.3645	fig|6666666.59349.peg.4288	None	fig|6666666.59351.peg.4326	None	None	fig|6666666.59354.peg.4943

7600	O-succinylbenzoate synthase EC 4.2.1.113	fig|6666666.59329.peg.5758	fig|6666666.59330.peg.314	fig|6666666.59331.peg.3852	fig|6666666.59332.peg.4285	fig|6666666.59334.peg.1202	fig|6666666.59335.peg.863	fig|6666666.59336.peg.6249	fig|6666666.59333.peg.1432	fig|6666666.59338.peg.3206	fig|6666666.59339.peg.3235	None	fig|6666666.59337.peg.1042	fig|6666666.59342.peg.3965	fig|6666666.71181.peg.515	fig|6666666.59344.peg.1735	fig|6666666.59345.peg.4240	fig|6666666.59341.peg.2668	fig|6666666.59347.peg.2643	fig|6666666.59346.peg.2065	fig|6666666.59348.peg.3813	None	None	None	fig|6666666.59353.peg.1142	fig|6666666.59355.peg.334	None

3148	FolM Alternative dihydrofolate reductase 1	None	None	fig|6666666.59331.peg.5560	fig|6666666.59332.peg.4584	fig|6666666.59334.peg.4964	fig|6666666.59335.peg.3117	fig|6666666.59336.peg.511	fig|6666666.59333.peg.2178	fig|6666666.59338.peg.5834	fig|6666666.59339.peg.776	fig|6666666.59340.peg.1738	None	fig|6666666.59342.peg.1533	fig|6666666.71181.peg.1353	fig|6666666.59344.peg.579	fig|6666666.59345.peg.767	None	fig|6666666.59347.peg.3422	fig|6666666.59346.peg.1795	None	fig|6666666.59349.peg.4418	fig|6666666.59350.peg.500	fig|6666666.59351.peg.4734	fig|6666666.59353.peg.1880	fig|6666666.59355.peg.1133	fig|6666666.59354.peg.2035

10848	UPF0225 protein YchJ	fig|6666666.59329.peg.1016	fig|6666666.59330.peg.1509	fig|6666666.59331.peg.3900	None	fig|6666666.59334.peg.3970	fig|6666666.59335.peg.2944	fig|6666666.59336.peg.5021	fig|6666666.59333.peg.2722	fig|6666666.59338.peg.5930	fig|6666666.59339.peg.275	None	fig|6666666.59337.peg.4875	fig|6666666.59342.peg.2270	fig|6666666.71181.peg.2756	fig|6666666.59344.peg.411	fig|6666666.59345.peg.1300	fig|6666666.59341.peg.3619	fig|6666666.59347.peg.2321	fig|6666666.59346.peg.2693	None	fig|6666666.59349.peg.3439	None	fig|6666666.59351.peg.2725	fig|6666666.59353.peg.1738	fig|6666666.59355.peg.1275	None

87	Putative inner membrane protein	fig|6666666.59329.peg.3417	fig|6666666.59330.peg.4147	fig|6666666.59331.peg.5373	fig|6666666.59332.peg.2706	None	fig|6666666.59335.peg.6248	fig|6666666.59336.peg.2682	fig|6666666.59333.peg.4259	fig|6666666.59338.peg.2861	fig|6666666.59339.peg.8560	None	fig|6666666.59337.peg.2492	fig|6666666.59342.peg.245	fig|6666666.71181.peg.4503	fig|6666666.59344.peg.5714	fig|6666666.59345.peg.151	fig|6666666.59341.peg.5360	fig|6666666.59347.peg.5061	fig|6666666.59346.peg.3705	fig|6666666.59348.peg.577	None	fig|6666666.59350.peg.4771	None	fig|6666666.59353.peg.4936	fig|6666666.59355.peg.3357	None

8327	Ubiquinol-cytochrome C reductase iron-sulfur subunit EC 1.10.2.2	fig|6666666.59329.peg.2057	fig|6666666.59330.peg.963	fig|6666666.59331.peg.791	fig|6666666.59332.peg.649	fig|6666666.59334.peg.3192	fig|6666666.59335.peg.3434	fig|6666666.59336.peg.1589	fig|6666666.59333.peg.618	fig|6666666.59338.peg.5690	None	fig|6666666.59340.peg.2267	fig|6666666.59337.peg.29	fig|6666666.59342.peg.4827	None	None	fig|6666666.59345.peg.3813	fig|6666666.59341.peg.3547	fig|6666666.59347.peg.3924	fig|6666666.59346.peg.2336	fig|6666666.59348.peg.1448	fig|6666666.59349.peg.41	fig|6666666.59350.peg.3002	fig|6666666.59351.peg.3462	None	None	fig|6666666.59354.peg.358

993	C1272 Predicted membrane protein hemolysin III homolog	fig|6666666.59329.peg.59	fig|6666666.59330.peg.637	fig|6666666.59331.peg.5137	fig|6666666.59332.peg.221	fig|6666666.59334.peg.5601	fig|6666666.59335.peg.2439	fig|6666666.59336.peg.3764	fig|6666666.59333.peg.1382	fig|6666666.59338.peg.579	fig|6666666.59339.peg.8085	fig|6666666.59340.peg.1060	fig|6666666.59337.peg.3896	None	fig|6666666.71181.peg.3728	fig|6666666.59344.peg.3688	fig|6666666.59345.peg.4933	fig|6666666.59341.peg.1947	fig|6666666.59347.peg.4752	fig|6666666.59346.peg.1816	fig|6666666.59348.peg.55	None	None	None	fig|6666666.59353.peg.3829	fig|6666666.59355.peg.1558	None

10051	low-specificity D-threonine aldolase	fig|6666666.59329.peg.2275	fig|6666666.59330.peg.3479	fig|6666666.59331.peg.120	fig|6666666.59332.peg.3293	fig|6666666.59334.peg.4358	fig|6666666.59335.peg.2934	fig|6666666.59336.peg.4473	None	fig|6666666.59338.peg.4319	fig|6666666.59339.peg.5339	fig|6666666.59340.peg.2021	fig|6666666.59337.peg.1911	fig|6666666.59342.peg.1584	None	None	fig|6666666.59345.peg.4015	fig|6666666.59341.peg.1902	fig|6666666.59347.peg.1520	fig|6666666.59346.peg.2806	fig|6666666.59348.peg.1466	fig|6666666.59349.peg.4089	fig|6666666.59350.peg.6931	fig|6666666.59351.peg.1751	None	None	fig|6666666.59354.peg.6100

3765	Sigma cross-reacting protein 27A	fig|6666666.59329.peg.1154	fig|6666666.59330.peg.922	fig|6666666.59331.peg.1898	None	fig|6666666.59334.peg.667	fig|6666666.59335.peg.971	fig|6666666.59336.peg.3354	fig|6666666.59333.peg.2169	None	fig|6666666.59339.peg.4478	None	fig|6666666.59337.peg.3948	fig|6666666.59342.peg.3263	fig|6666666.71181.peg.4576	fig|6666666.59344.peg.1837	fig|6666666.59345.peg.435	fig|6666666.59341.peg.1891	fig|6666666.59347.peg.1524	None	fig|6666666.59348.peg.3278	fig|6666666.59349.peg.4492	None	fig|6666666.59351.peg.93	fig|6666666.59353.peg.2371	fig|6666666.59355.peg.1643	fig|6666666.59354.peg.1892

1295	Methionine ABC transporter substrate-binding protein	fig|6666666.59329.peg.3215	fig|6666666.59330.peg.2016	fig|6666666.59331.peg.1605	fig|6666666.59332.peg.400	fig|6666666.59334.peg.312	fig|6666666.59335.peg.4077	fig|6666666.59336.peg.2326	fig|6666666.59333.peg.3267	fig|6666666.59338.peg.2029	fig|6666666.59339.peg.5167	None	fig|6666666.59337.peg.1108	fig|6666666.59342.peg.3833	fig|6666666.71181.peg.833	fig|6666666.59344.peg.2686	fig|6666666.59345.peg.3513	fig|6666666.59341.peg.2856	fig|6666666.59347.peg.2924	fig|6666666.59346.peg.1719	fig|6666666.59348.peg.700	None	None	None	fig|6666666.59353.peg.2015	fig|6666666.59355.peg.4121	None

8558	FIG026291 Hypothetical periplasmic protein	fig|6666666.59329.peg.6297	fig|6666666.59330.peg.2874	fig|6666666.59331.peg.5925	fig|6666666.59332.peg.4991	fig|6666666.59334.peg.2693	fig|6666666.59335.peg.604	fig|6666666.59336.peg.4707	fig|6666666.59333.peg.3158	fig|6666666.59338.peg.406	fig|6666666.59339.peg.476	None	fig|6666666.59337.peg.4611	fig|6666666.59342.peg.4457	None	None	fig|6666666.59345.peg.1318	fig|6666666.59341.peg.486	fig|6666666.59347.peg.4129	fig|6666666.59346.peg.4935	fig|6666666.59348.peg.5088	fig|6666666.59349.peg.4303	fig|6666666.59350.peg.6365	fig|6666666.59351.peg.1093	None	None	fig|6666666.59354.peg.1702

11173	Outer membrane protein OmpV	fig|6666666.59329.peg.1075	fig|6666666.59330.peg.2756	fig|6666666.59331.peg.489	fig|6666666.59332.peg.2104	fig|6666666.59334.peg.1896	fig|6666666.59335.peg.5145	fig|6666666.59336.peg.5750	fig|6666666.59333.peg.811	None	fig|6666666.59339.peg.1029	None	fig|6666666.59337.peg.319	fig|6666666.59342.peg.3890	fig|6666666.71181.peg.2968	fig|6666666.59344.peg.2890	fig|6666666.59345.peg.2213	None	None	fig|6666666.59346.peg.3526	fig|6666666.59348.peg.5055	fig|6666666.59349.peg.2882	None	fig|6666666.59351.peg.1267	fig|6666666.59353.peg.1436	fig|6666666.59355.peg.1985	fig|6666666.59354.peg.3058

2684	Putative cytoplasmic protein	None	fig|6666666.59330.peg.906	fig|6666666.59331.peg.4196	fig|6666666.59332.peg.5936	fig|6666666.59334.peg.2552	fig|6666666.59335.peg.3515	fig|6666666.59336.peg.6374	fig|6666666.59333.peg.4677	fig|6666666.59338.peg.992	fig|6666666.59339.peg.6503	None	fig|6666666.59337.peg.5352	fig|6666666.59342.peg.3816	fig|6666666.71181.peg.1573	fig|6666666.59344.peg.2012	fig|6666666.59345.peg.420	fig|6666666.59341.peg.1078	fig|6666666.59347.peg.1280	fig|6666666.59346.peg.1051	fig|6666666.59348.peg.5241	None	None	None	fig|6666666.59353.peg.2912	fig|6666666.59355.peg.2827	None

7454	Hypothetical protein UPF0052	fig|6666666.59329.peg.4647	fig|6666666.59330.peg.1708	fig|6666666.59331.peg.2085	fig|6666666.59332.peg.1519	fig|6666666.59334.peg.295	fig|6666666.59335.peg.5490	fig|6666666.59336.peg.1321	fig|6666666.59333.peg.1333	fig|6666666.59338.peg.2314	fig|6666666.59339.peg.138	None	fig|6666666.59337.peg.4023	None	fig|6666666.71181.peg.2530	fig|6666666.59344.peg.4098	fig|6666666.59345.peg.1114	fig|6666666.59341.peg.2169	fig|6666666.59347.peg.5159	fig|6666666.59346.peg.531	fig|6666666.59348.peg.715	None	None	None	fig|6666666.59353.peg.2140	fig|6666666.59355.peg.2680	None

2308	FIG008443 hypothetical protein	fig|6666666.59329.peg.1209	fig|6666666.59330.peg.778	fig|6666666.59331.peg.1681	fig|6666666.59332.peg.4797	fig|6666666.59334.peg.1943	fig|6666666.59335.peg.949	fig|6666666.59336.peg.5548	fig|6666666.59333.peg.1517	fig|6666666.59338.peg.2944	fig|6666666.59339.peg.2795	fig|6666666.59340.peg.2928	fig|6666666.59337.peg.1515	fig|6666666.59342.peg.1783	None	None	fig|6666666.59345.peg.3030	fig|6666666.59341.peg.1069	fig|6666666.59347.peg.4190	fig|6666666.59346.peg.2776	fig|6666666.59348.peg.1590	fig|6666666.59349.peg.2420	None	fig|6666666.59351.peg.4683	None	None	None

5614	hypothetical protein	fig|6666666.59329.peg.4395	fig|6666666.59330.peg.2100	fig|6666666.59331.peg.6036	fig|6666666.59332.peg.2669	None	fig|6666666.59335.peg.1374	fig|6666666.59336.peg.1996	fig|6666666.59333.peg.3957	fig|6666666.59338.peg.1239	fig|6666666.59339.peg.2768	fig|6666666.59340.peg.3787	fig|6666666.59337.peg.3530	fig|6666666.59342.peg.4011	None	None	fig|6666666.59345.peg.489	fig|6666666.59341.peg.2029	fig|6666666.59347.peg.1601	fig|6666666.59346.peg.4261	fig|6666666.59348.peg.3269	fig|6666666.59349.peg.4439	None	fig|6666666.59351.peg.3589	None	None	fig|6666666.59354.peg.1119

2518	Protein YciN	fig|6666666.59329.peg.4073	fig|6666666.59330.peg.1327	fig|6666666.59331.peg.4273	fig|6666666.59332.peg.2646	None	fig|6666666.59335.peg.5622	fig|6666666.59336.peg.869	fig|6666666.59333.peg.2552	fig|6666666.59338.peg.5484	fig|6666666.59339.peg.292	fig|6666666.59340.peg.959	fig|6666666.59337.peg.3364	fig|6666666.59342.peg.124	fig|6666666.71181.peg.4158	fig|6666666.59344.peg.473	fig|6666666.59345.peg.1548	fig|6666666.59341.peg.1002	fig|6666666.59347.peg.4117	fig|6666666.59346.peg.2529	None	None	None	None	fig|6666666.59353.peg.1793	fig|6666666.59355.peg.1222	None

4298	Putative activity regulator of membrane protease YbbK	None	fig|6666666.59330.peg.3038	fig|6666666.59331.peg.5780	fig|6666666.59332.peg.627	fig|6666666.59334.peg.2219	fig|6666666.59335.peg.3013	fig|6666666.59336.peg.4727	fig|6666666.59333.peg.4950	fig|6666666.59338.peg.2801	fig|6666666.59339.peg.5242	None	fig|6666666.59337.peg.4913	fig|6666666.59342.peg.100	fig|6666666.71181.peg.3614	fig|6666666.59344.peg.5061	fig|6666666.59345.peg.4382	fig|6666666.59341.peg.4977	fig|6666666.59347.peg.4431	fig|6666666.59346.peg.2371	fig|6666666.59348.peg.3877	None	None	None	fig|6666666.59353.peg.4055	fig|6666666.59355.peg.4131	None

158	FIG002994 Putative transcriptional regulator	fig|6666666.59329.peg.2100	fig|6666666.59330.peg.2880	fig|6666666.59331.peg.6266	fig|6666666.59332.peg.3670	None	fig|6666666.59335.peg.610	fig|6666666.59336.peg.4950	fig|6666666.59333.peg.3152	fig|6666666.59338.peg.1412	fig|6666666.59339.peg.470	fig|6666666.59340.peg.2252	fig|6666666.59337.peg.4605	fig|6666666.59342.peg.2457	None	None	fig|6666666.59345.peg.1324	fig|6666666.59341.peg.480	fig|6666666.59347.peg.4122	fig|6666666.59346.peg.2982	fig|6666666.59348.peg.4415	fig|6666666.59349.peg.941	None	fig|6666666.59351.peg.1099	None	None	fig|6666666.59354.peg.1695

5673	FIG002842 hypothetical protein	fig|6666666.59329.peg.341	fig|6666666.59330.peg.4890	None	fig|6666666.59332.peg.310	fig|6666666.59334.peg.5123	fig|6666666.59335.peg.5396	fig|6666666.59336.peg.1424	fig|6666666.59333.peg.156	fig|6666666.59338.peg.2611	fig|6666666.59339.peg.7939	None	fig|6666666.59337.peg.2046	fig|6666666.59342.peg.3659	fig|6666666.71181.peg.1968	fig|6666666.59344.peg.1465	fig|6666666.59345.peg.2428	fig|6666666.59341.peg.1347	fig|6666666.59347.peg.3889	fig|6666666.59346.peg.574	fig|6666666.59348.peg.1264	None	None	None	fig|6666666.59353.peg.151	fig|6666666.59355.peg.761	None

5674	FIG01200175 hypothetical protein	fig|6666666.59329.peg.6352	fig|6666666.59330.peg.1066	None	fig|6666666.59332.peg.5056	fig|6666666.59334.peg.1663	fig|6666666.59335.peg.145	fig|6666666.59336.peg.2491	fig|6666666.59333.peg.3419	fig|6666666.59338.peg.285	fig|6666666.59339.peg.5083	None	fig|6666666.59337.peg.1147	fig|6666666.59342.peg.454	fig|6666666.71181.peg.929	fig|6666666.59344.peg.3530	fig|6666666.59345.peg.3547	fig|6666666.59341.peg.5280	fig|6666666.59347.peg.2781	fig|6666666.59346.peg.2255	fig|6666666.59348.peg.1018	None	None	None	fig|6666666.59353.peg.885	fig|6666666.59355.peg.819	None

9940	Manganese-dependent inorganic pyrophosphatase EC 3.6.1.1	None	fig|6666666.59330.peg.1534	fig|6666666.59331.peg.1804	fig|6666666.59332.peg.4286	fig|6666666.59334.peg.5039	fig|6666666.59335.peg.583	None	fig|6666666.59333.peg.2917	fig|6666666.59338.peg.2116	fig|6666666.59339.peg.509	fig|6666666.59340.peg.472	fig|6666666.59337.peg.2318	fig|6666666.59342.peg.4993	None	None	fig|6666666.59345.peg.1276	fig|6666666.59341.peg.5347	fig|6666666.59347.peg.3792	fig|6666666.59346.peg.4748	fig|6666666.59348.peg.5375	fig|6666666.59349.peg.3061	fig|6666666.59350.peg.2069	fig|6666666.59351.peg.193	None	None	fig|6666666.59354.peg.4272

10342	Pyruvate formate-lyase EC 2.3.1.54	fig|6666666.59329.peg.111	fig|6666666.59330.peg.1726	fig|6666666.59331.peg.4725	fig|6666666.59332.peg.2904	fig|6666666.59334.peg.4081	fig|6666666.59335.peg.813	None	fig|6666666.59333.peg.3413	fig|6666666.59338.peg.69	None	fig|6666666.59340.peg.2714	fig|6666666.59337.peg.4011	fig|6666666.59342.peg.1361	fig|6666666.71181.peg.1436	fig|6666666.59344.peg.1550	fig|6666666.59345.peg.3748	fig|6666666.59341.peg.1142	fig|6666666.59347.peg.3068	fig|6666666.59346.peg.1374	fig|6666666.59348.peg.2696	None	None	None	fig|6666666.59353.peg.4350	fig|6666666.59355.peg.4492	None

931	Lipid A core-O-antigen ligase	fig|6666666.59329.peg.6046	None	fig|6666666.59331.peg.3134	fig|6666666.59332.peg.3569	fig|6666666.59334.peg.4107	fig|6666666.59335.peg.194	fig|6666666.59336.peg.1292	fig|6666666.59333.peg.586	fig|6666666.59338.peg.806	fig|6666666.59339.peg.7747	fig|6666666.59340.peg.237	fig|6666666.59337.peg.787	fig|6666666.59342.peg.4465	None	fig|6666666.59344.peg.5999	fig|6666666.59345.peg.4910	fig|6666666.59341.peg.5793	fig|6666666.59347.peg.3127	fig|6666666.59346.peg.1594	fig|6666666.59348.peg.1964	None	None	None	fig|6666666.59353.peg.4981	fig|6666666.59355.peg.5055	None

7149	Rare lipoprotein A precursor	fig|6666666.59329.peg.3588	fig|6666666.59330.peg.2034	fig|6666666.59331.peg.3710	fig|6666666.59332.peg.2692	fig|6666666.59334.peg.2321	fig|6666666.59335.peg.1758	fig|6666666.59336.peg.583	fig|6666666.59333.peg.4851	fig|6666666.59338.peg.7	fig|6666666.59339.peg.5202	fig|6666666.59340.peg.402	fig|6666666.59337.peg.1090	fig|6666666.59342.peg.916	None	None	fig|6666666.59345.peg.3496	fig|6666666.59341.peg.4752	fig|6666666.59347.peg.2907	fig|6666666.59346.peg.3293	fig|6666666.59348.peg.852	fig|6666666.59349.peg.2766	None	fig|6666666.59351.peg.3621	None	None	None

2880	Cytochrome c5	fig|6666666.59329.peg.2526	fig|6666666.59330.peg.3505	fig|6666666.59331.peg.3357	fig|6666666.59332.peg.3610	None	fig|6666666.59335.peg.68	fig|6666666.59336.peg.1207	fig|6666666.59333.peg.1865	fig|6666666.59338.peg.4101	fig|6666666.59339.peg.7618	fig|6666666.59340.peg.2	fig|6666666.59337.peg.2778	fig|6666666.59342.peg.5531	None	None	fig|6666666.59345.peg.2755	fig|6666666.59341.peg.2592	fig|6666666.59347.peg.5219	fig|6666666.59346.peg.2735	fig|6666666.59348.peg.2194	fig|6666666.59349.peg.4493	None	fig|6666666.59351.peg.92	None	None	fig|6666666.59354.peg.1891

4764	Periplasmic divalent cation tolerance protein CutA	fig|6666666.59329.peg.3502	fig|6666666.59330.peg.4644	None	None	None	fig|6666666.59335.peg.5407	fig|6666666.59336.peg.6604	fig|6666666.59333.peg.5550	fig|6666666.59338.peg.6098	fig|6666666.59339.peg.2837	None	fig|6666666.59337.peg.5668	fig|6666666.59342.peg.1674	fig|6666666.71181.peg.1673	fig|6666666.59344.peg.621	fig|6666666.59345.peg.106	fig|6666666.59341.peg.3252	fig|6666666.59347.peg.835	None	fig|6666666.59348.peg.4794	fig|6666666.59349.peg.2365	None	fig|6666666.59351.peg.110	fig|6666666.59353.peg.1043	fig|6666666.59355.peg.3576	fig|6666666.59354.peg.3260

9170	Permease of the major facilitator superfamily	fig|6666666.59329.peg.2355	fig|6666666.59330.peg.1552	fig|6666666.59331.peg.732	fig|6666666.59332.peg.467	fig|6666666.59334.peg.3875	fig|6666666.59335.peg.6239	fig|6666666.59336.peg.633	fig|6666666.59333.peg.4897	fig|6666666.59338.peg.3695	fig|6666666.59339.peg.8551	None	fig|6666666.59337.peg.1635	fig|6666666.59342.peg.6451	None	None	fig|6666666.59345.peg.5484	fig|6666666.59341.peg.646	fig|6666666.59347.peg.3938	fig|6666666.59346.peg.619	None	fig|6666666.59349.peg.2927	fig|6666666.59350.peg.3921	fig|6666666.59351.peg.823	None	None	fig|6666666.59354.peg.3152

5480	FIG01201122 hypothetical protein	fig|6666666.59329.peg.2489	fig|6666666.59330.peg.1604	fig|6666666.59331.peg.159	None	None	fig|6666666.59335.peg.2525	fig|6666666.59336.peg.2573	fig|6666666.59333.peg.3199	fig|6666666.59338.peg.4643	fig|6666666.59339.peg.1919	None	fig|6666666.59337.peg.4897	fig|6666666.59342.peg.4400	fig|6666666.71181.peg.1051	fig|6666666.59344.peg.5451	fig|6666666.59345.peg.4490	fig|6666666.59341.peg.5187	fig|6666666.59347.peg.682	fig|6666666.59346.peg.4562	fig|6666666.59348.peg.1498	None	fig|6666666.59350.peg.4244	None	fig|6666666.59353.peg.4525	fig|6666666.59355.peg.5724	None

6410	FIG01199549 hypothetical protein	fig|6666666.59329.peg.1213	fig|6666666.59330.peg.487	None	fig|6666666.59332.peg.5550	None	fig|6666666.59335.peg.1706	fig|6666666.59336.peg.2412	fig|6666666.59333.peg.2260	fig|6666666.59338.peg.724	fig|6666666.59339.peg.7246	fig|6666666.59340.peg.3575	fig|6666666.59337.peg.1562	fig|6666666.59342.peg.2121	fig|6666666.71181.peg.867	fig|6666666.59344.peg.6	fig|6666666.59345.peg.2219	fig|6666666.59341.peg.1475	fig|6666666.59347.peg.4350	fig|6666666.59346.peg.3534	fig|6666666.59348.peg.2783	None	None	None	fig|6666666.59353.peg.347	fig|6666666.59355.peg.1385	None

6082	FIG139928 Putative protease	fig|6666666.59329.peg.4212	fig|6666666.59330.peg.98	fig|6666666.59331.peg.5035	fig|6666666.59332.peg.4779	fig|6666666.59334.peg.771	fig|6666666.59335.peg.2113	fig|6666666.59336.peg.3978	fig|6666666.59333.peg.2307	fig|6666666.59338.peg.2636	fig|6666666.59339.peg.4880	None	fig|6666666.59337.peg.692	fig|6666666.59342.peg.4565	fig|6666666.71181.peg.3536	fig|6666666.59344.peg.2076	fig|6666666.59345.peg.2348	None	fig|6666666.59347.peg.2996	fig|6666666.59346.peg.1984	fig|6666666.59348.peg.5349	None	None	None	fig|6666666.59353.peg.3114	fig|6666666.59355.peg.483	None

9489	ATPase involved in DNA repair	fig|6666666.59329.peg.4554	fig|6666666.59330.peg.4382	fig|6666666.59331.peg.4293	fig|6666666.59332.peg.2655	None	fig|6666666.59335.peg.1245	fig|6666666.59336.peg.6001	fig|6666666.59333.peg.2590	fig|6666666.59338.peg.5308	fig|6666666.59339.peg.3024	fig|6666666.59340.peg.3064	fig|6666666.59337.peg.4218	fig|6666666.59342.peg.3627	None	None	fig|6666666.59345.peg.5318	fig|6666666.59341.peg.3759	fig|6666666.59347.peg.5088	fig|6666666.59346.peg.5119	fig|6666666.59348.peg.3305	fig|6666666.59349.peg.186	None	fig|6666666.59351.peg.2073	None	None	fig|6666666.59354.peg.2938

5224	TrkA, Potassium channel-family protein	fig|6666666.59329.peg.764	fig|6666666.59330.peg.2009	None	fig|6666666.59332.peg.4556	fig|6666666.59334.peg.2872	fig|6666666.59335.peg.4097	fig|6666666.59336.peg.1215	fig|6666666.59333.peg.3430	fig|6666666.59338.peg.4072	fig|6666666.59339.peg.3511	fig|6666666.59340.peg.2046	fig|6666666.59337.peg.4282	fig|6666666.59342.peg.5278	fig|6666666.71181.peg.4477	fig|6666666.59344.peg.353	fig|6666666.59345.peg.4572	fig|6666666.59341.peg.1837	fig|6666666.59347.peg.1984	fig|6666666.59346.peg.5236	None	None	None	None	fig|6666666.59353.peg.535	fig|6666666.59355.peg.20	None

4817	FIG023103 Predicted transmembrane protein	fig|6666666.59329.peg.5576	fig|6666666.59330.peg.2441	fig|6666666.59331.peg.2650	fig|6666666.59332.peg.2866	fig|6666666.59334.peg.4692	fig|6666666.59335.peg.3981	fig|6666666.59336.peg.492	fig|6666666.59333.peg.2985	fig|6666666.59338.peg.6120	fig|6666666.59339.peg.8906	fig|6666666.59340.peg.3103	fig|6666666.59337.peg.4784	None	None	None	fig|6666666.59345.peg.4830	fig|6666666.59341.peg.2410	fig|6666666.59347.peg.5370	fig|6666666.59346.peg.1663	fig|6666666.59348.peg.3602	fig|6666666.59349.peg.4203	None	fig|6666666.59351.peg.3504	None	None	fig|6666666.59354.peg.1325

1309	Qnr	fig|6666666.59329.peg.3861	fig|6666666.59330.peg.1623	fig|6666666.59331.peg.1811	fig|6666666.59332.peg.3423	fig|6666666.59334.peg.4475	fig|6666666.59335.peg.5682	fig|6666666.59336.peg.2203	fig|6666666.59333.peg.3997	fig|6666666.59338.peg.3003	fig|6666666.59339.peg.2168	None	fig|6666666.59337.peg.1277	None	fig|6666666.71181.peg.1228	fig|6666666.59344.peg.184	fig|6666666.59345.peg.4467	fig|6666666.59341.peg.5751	fig|6666666.59347.peg.96	fig|6666666.59346.peg.1120	fig|6666666.59348.peg.4234	None	None	None	fig|6666666.59353.peg.492	fig|6666666.59355.peg.2216	None

7754	Acylphosphate phosphohydrolase EC 3.6.1.7 , putative	fig|6666666.59329.peg.3251	fig|6666666.59330.peg.1036	None	fig|6666666.59332.peg.3263	fig|6666666.59334.peg.3983	fig|6666666.59335.peg.3576	fig|6666666.59336.peg.6226	fig|6666666.59333.peg.2072	fig|6666666.59338.peg.2698	fig|6666666.59339.peg.1566	None	fig|6666666.59337.peg.3178	fig|6666666.59342.peg.2778	fig|6666666.71181.peg.276	fig|6666666.59344.peg.1203	fig|6666666.59345.peg.3281	None	fig|6666666.59347.peg.1796	fig|6666666.59346.peg.4460	fig|6666666.59348.peg.5047	None	fig|6666666.59350.peg.6035	None	fig|6666666.59353.peg.1706	fig|6666666.59355.peg.1014	None

4234	Ribonucleotide reductase of class III anaerobic , activating protein EC 1.97.1.4	fig|6666666.59329.peg.4714	fig|6666666.59330.peg.773	fig|6666666.59331.peg.1551	fig|6666666.59332.peg.4415	fig|6666666.59334.peg.1941	fig|6666666.59335.peg.3459	fig|6666666.59336.peg.4569	None	fig|6666666.59338.peg.2946	fig|6666666.59339.peg.2793	None	fig|6666666.59337.peg.5364	fig|6666666.59342.peg.2299	fig|6666666.71181.peg.3514	fig|6666666.59344.peg.4626	fig|6666666.59345.peg.49	fig|6666666.59341.peg.4424	fig|6666666.59347.peg.913	fig|6666666.59346.peg.1209	fig|6666666.59348.peg.1868	None	None	None	fig|6666666.59353.peg.2414	fig|6666666.59355.peg.505	None

313	putative membrane protein	fig|6666666.59329.peg.3883	fig|6666666.59330.peg.1124	fig|6666666.59331.peg.3951	fig|6666666.59332.peg.2847	fig|6666666.59334.peg.3534	fig|6666666.59335.peg.1378	fig|6666666.59336.peg.5424	fig|6666666.59333.peg.4141	fig|6666666.59338.peg.2777	fig|6666666.59339.peg.2771	fig|6666666.59340.peg.1003	fig|6666666.59337.peg.5305	fig|6666666.59342.peg.2590	None	None	fig|6666666.59345.peg.5721	fig|6666666.59341.peg.599	fig|6666666.59347.peg.2730	fig|6666666.59346.peg.5032	fig|6666666.59348.peg.3478	None	fig|6666666.59350.peg.3037	fig|6666666.59351.peg.3906	None	None	None

10314	Hypothetical protein yfgJ	fig|6666666.59329.peg.298	fig|6666666.59330.peg.4374	None	fig|6666666.59332.peg.4348	fig|6666666.59334.peg.1261	fig|6666666.59335.peg.1960	fig|6666666.59336.peg.1632	fig|6666666.59333.peg.2150	None	fig|6666666.59339.peg.6980	fig|6666666.59340.peg.37	fig|6666666.59337.peg.759	fig|6666666.59342.peg.5784	fig|6666666.71181.peg.1776	fig|6666666.59344.peg.5142	fig|6666666.59345.peg.3633	fig|6666666.59341.peg.5882	fig|6666666.59347.peg.195	fig|6666666.59346.peg.1311	fig|6666666.59348.peg.5525	None	None	None	fig|6666666.59353.peg.2715	fig|6666666.59355.peg.1765	None

4840	DNA polymerase III psi subunit EC 2.7.7.7	fig|6666666.59329.peg.2308	fig|6666666.59330.peg.90	fig|6666666.59331.peg.3405	fig|6666666.59332.peg.5335	fig|6666666.59334.peg.4217	fig|6666666.59335.peg.2852	fig|6666666.59336.peg.3567	fig|6666666.59333.peg.2315	None	fig|6666666.59339.peg.4866	None	fig|6666666.59337.peg.1296	fig|6666666.59342.peg.4542	fig|6666666.71181.peg.468	fig|6666666.59344.peg.1387	fig|6666666.59345.peg.2342	fig|6666666.59341.peg.5171	fig|6666666.59347.peg.2988	fig|6666666.59346.peg.1990	fig|6666666.59348.peg.4832	None	None	None	fig|6666666.59353.peg.28	fig|6666666.59355.peg.635	None

3952	TPR domain protein in aerotolerance operon	fig|6666666.59329.peg.4302	fig|6666666.59330.peg.2524	fig|6666666.59331.peg.593	fig|6666666.59332.peg.4027	fig|6666666.59334.peg.1811	fig|6666666.59335.peg.4337	fig|6666666.59336.peg.3298	fig|6666666.59333.peg.4803	fig|6666666.59338.peg.4730	fig|6666666.59339.peg.4365	fig|6666666.59340.peg.2247	fig|6666666.59337.peg.2078	fig|6666666.59342.peg.3801	None	None	fig|6666666.59345.peg.3318	fig|6666666.59341.peg.4002	fig|6666666.59347.peg.706	fig|6666666.59346.peg.2269	None	fig|6666666.59349.peg.4065	None	fig|6666666.59351.peg.697	None	None	fig|6666666.59354.peg.6345

5787	Galactokinase EC 2.7.1.6	fig|6666666.59329.peg.1332	None	fig|6666666.59331.peg.3389	fig|6666666.59332.peg.2256	fig|6666666.59334.peg.1042	fig|6666666.59335.peg.4068	fig|6666666.59336.peg.1926	fig|6666666.59333.peg.1144	fig|6666666.59338.peg.3548	fig|6666666.59339.peg.6859	fig|6666666.59340.peg.1731	fig|6666666.59337.peg.1353	None	fig|6666666.71181.peg.2505	fig|6666666.59344.peg.4065	fig|6666666.59345.peg.2291	fig|6666666.59341.peg.297	fig|6666666.59347.peg.3769	fig|6666666.59346.peg.2239	fig|6666666.59348.peg.567	None	None	None	fig|6666666.59353.peg.2114	fig|6666666.59355.peg.2707	None

6333	Glutamate Aspartate periplasmic binding protein precursor GltI TC 3.A.1.3.4	fig|6666666.59329.peg.6252	fig|6666666.59330.peg.1029	fig|6666666.59331.peg.1848	fig|6666666.59332.peg.5445	None	fig|6666666.59335.peg.3582	fig|6666666.59336.peg.3917	fig|6666666.59333.peg.2081	None	fig|6666666.59339.peg.1557	None	fig|6666666.59337.peg.3171	fig|6666666.59342.peg.1632	fig|6666666.71181.peg.799	fig|6666666.59344.peg.4510	fig|6666666.59345.peg.3274	fig|6666666.59341.peg.4211	fig|6666666.59347.peg.1790	fig|6666666.59346.peg.5310	fig|6666666.59348.peg.3776	None	fig|6666666.59350.peg.1563	None	fig|6666666.59353.peg.413	fig|6666666.59355.peg.2138	None

7124	Cell division protein FtsN	fig|6666666.59329.peg.1096	fig|6666666.59330.peg.545	None	fig|6666666.59332.peg.1186	fig|6666666.59334.peg.615	fig|6666666.59335.peg.433	fig|6666666.59336.peg.134	fig|6666666.59333.peg.2681	fig|6666666.59338.peg.3638	fig|6666666.59339.peg.6389	fig|6666666.59340.peg.369	fig|6666666.59337.peg.399	fig|6666666.59342.peg.5127	None	None	fig|6666666.59345.peg.2545	fig|6666666.59341.peg.5842	fig|6666666.59347.peg.4915	fig|6666666.59346.peg.1010	fig|6666666.59348.peg.157	fig|6666666.59349.peg.511	None	fig|6666666.59351.peg.3832	None	None	fig|6666666.59354.peg.935

3380	Uncharacterized protein ImpH VasB	fig|6666666.59329.peg.4243	fig|6666666.59330.peg.2155	None	fig|6666666.59332.peg.2968	None	fig|6666666.59335.peg.4186	fig|6666666.59336.peg.4704	fig|6666666.59333.peg.3394	fig|6666666.59338.peg.4585	fig|6666666.59339.peg.2727	None	fig|6666666.59337.peg.5217	fig|6666666.59342.peg.2423	fig|6666666.71181.peg.296	fig|6666666.59344.peg.1228	fig|6666666.59345.peg.4112	fig|6666666.59341.peg.3633	fig|6666666.59347.peg.1026	None	fig|6666666.59348.peg.2401	fig|6666666.59349.peg.1637	None	None	fig|6666666.59353.peg.4898	fig|6666666.59355.peg.5012	fig|6666666.59354.peg.409

337	Predicted transcriptional regulator	fig|6666666.59329.peg.2176	fig|6666666.59330.peg.3785	None	fig|6666666.59332.peg.2729	fig|6666666.59334.peg.2732	fig|6666666.59335.peg.4780	fig|6666666.59336.peg.1140	fig|6666666.59333.peg.5524	fig|6666666.59338.peg.5400	fig|6666666.59339.peg.4200	fig|6666666.59340.peg.2276	fig|6666666.59337.peg.2222	fig|6666666.59342.peg.1479	None	None	fig|6666666.59345.peg.2996	fig|6666666.59341.peg.2345	fig|6666666.59347.peg.199	fig|6666666.59346.peg.890	fig|6666666.59348.peg.4030	fig|6666666.59349.peg.2641	None	fig|6666666.59351.peg.4139	None	None	fig|6666666.59354.peg.6427

8532	Beta-galactosidase EC 3.2.1.23	fig|6666666.59329.peg.2063	fig|6666666.59330.peg.2392	fig|6666666.59331.peg.4937	fig|6666666.59332.peg.6155	fig|6666666.59334.peg.523	fig|6666666.59335.peg.4074	fig|6666666.59336.peg.1235	fig|6666666.59333.peg.5340	fig|6666666.59338.peg.5767	fig|6666666.59339.peg.2743	None	fig|6666666.59337.peg.1346	None	fig|6666666.71181.peg.5879	fig|6666666.59344.peg.5240	fig|6666666.59345.peg.1001	fig|6666666.59341.peg.5805	fig|6666666.59347.peg.3285	fig|6666666.59346.peg.3262	fig|6666666.59348.peg.2270	None	None	None	fig|6666666.59353.peg.3694	fig|6666666.59355.peg.3016	None

3971	Cytochrome oxidase biogenesis protein Sco1 SenC PrrC, putative copper metallochaperone	fig|6666666.59329.peg.1691	fig|6666666.59330.peg.904	fig|6666666.59331.peg.1790	fig|6666666.59332.peg.854	fig|6666666.59334.peg.2929	fig|6666666.59335.peg.3070	fig|6666666.59336.peg.3745	fig|6666666.59333.peg.5287	fig|6666666.59338.peg.1536	fig|6666666.59339.peg.5748	fig|6666666.59340.peg.3760	fig|6666666.59337.peg.5425	None	None	None	fig|6666666.59345.peg.1921	fig|6666666.59341.peg.806	fig|6666666.59347.peg.3794	fig|6666666.59346.peg.356	None	fig|6666666.59349.peg.3501	fig|6666666.59350.peg.5112	fig|6666666.59351.peg.4381	None	None	fig|6666666.59354.peg.2075

8530	Curlin genes transcriptional activator	None	fig|6666666.59330.peg.1065	None	fig|6666666.59332.peg.5057	fig|6666666.59334.peg.1665	fig|6666666.59335.peg.146	fig|6666666.59336.peg.2492	fig|6666666.59333.peg.3420	fig|6666666.59338.peg.286	fig|6666666.59339.peg.5086	fig|6666666.59340.peg.2087	fig|6666666.59337.peg.1145	fig|6666666.59342.peg.455	fig|6666666.71181.peg.930	fig|6666666.59344.peg.3531	fig|6666666.59345.peg.3546	fig|6666666.59341.peg.5281	fig|6666666.59347.peg.2780	fig|6666666.59346.peg.3375	fig|6666666.59348.peg.1015	None	None	None	fig|6666666.59353.peg.884	fig|6666666.59355.peg.820	None

1644	Conserved domain protein	fig|6666666.59329.peg.367	fig|6666666.59330.peg.988	fig|6666666.59331.peg.2342	fig|6666666.59332.peg.262	fig|6666666.59334.peg.2973	None	fig|6666666.59336.peg.187	fig|6666666.59333.peg.1576	fig|6666666.59338.peg.5212	fig|6666666.59339.peg.7648	None	fig|6666666.59337.peg.2543	fig|6666666.59342.peg.2495	None	None	fig|6666666.59345.peg.5535	fig|6666666.59341.peg.4840	fig|6666666.59347.peg.4715	fig|6666666.59346.peg.923	fig|6666666.59348.peg.46	fig|6666666.59349.peg.4593	fig|6666666.59350.peg.3670	fig|6666666.59351.peg.2995	None	None	fig|6666666.59354.peg.4166

5884	Predicted hydrolase	fig|6666666.59329.peg.2179	fig|6666666.59330.peg.2089	None	fig|6666666.59332.peg.3845	fig|6666666.59334.peg.4898	fig|6666666.59335.peg.5174	fig|6666666.59336.peg.3930	fig|6666666.59333.peg.3934	fig|6666666.59338.peg.1632	fig|6666666.59339.peg.3724	None	fig|6666666.59337.peg.5692	fig|6666666.59342.peg.6334	fig|6666666.71181.peg.3727	fig|6666666.59344.peg.3689	fig|6666666.59345.peg.818	fig|6666666.59341.peg.4374	fig|6666666.59347.peg.3480	fig|6666666.59346.peg.1509	fig|6666666.59348.peg.3650	None	None	None	fig|6666666.59353.peg.3828	fig|6666666.59355.peg.1559	None

353	FIG01201461 hypothetical protein	fig|6666666.59329.peg.2357	fig|6666666.59330.peg.1554	fig|6666666.59331.peg.3909	fig|6666666.59332.peg.2710	fig|6666666.59334.peg.3685	None	fig|6666666.59336.peg.636	fig|6666666.59333.peg.3189	fig|6666666.59338.peg.3488	fig|6666666.59339.peg.8553	fig|6666666.59340.peg.108	fig|6666666.59337.peg.1638	fig|6666666.59342.peg.6257	None	fig|6666666.59344.peg.6117	fig|6666666.59345.peg.5481	fig|6666666.59341.peg.4302	fig|6666666.59347.peg.5213	fig|6666666.59346.peg.3702	fig|6666666.59348.peg.2613	None	None	None	fig|6666666.59353.peg.3576	fig|6666666.59355.peg.4380	None

4806	Type IV pilus biogenesis protein PilO	fig|6666666.59329.peg.310	fig|6666666.59330.peg.2632	fig|6666666.59331.peg.4501	fig|6666666.59332.peg.1027	None	fig|6666666.59335.peg.2488	fig|6666666.59336.peg.4162	fig|6666666.59333.peg.4149	fig|6666666.59338.peg.958	fig|6666666.59339.peg.8722	fig|6666666.59340.peg.2524	fig|6666666.59337.peg.1698	fig|6666666.59342.peg.6159	None	None	fig|6666666.59345.peg.5384	fig|6666666.59341.peg.964	fig|6666666.59347.peg.4940	fig|6666666.59346.peg.3	fig|6666666.59348.peg.1915	fig|6666666.59349.peg.524	None	fig|6666666.59351.peg.3847	None	None	fig|6666666.59354.peg.2402

8165	Regulatory protein RecX	None	fig|6666666.59330.peg.4218	None	None	fig|6666666.59334.peg.281	fig|6666666.59335.peg.262	fig|6666666.59336.peg.3452	fig|6666666.59333.peg.1721	fig|6666666.59338.peg.760	fig|6666666.59339.peg.8116	fig|6666666.59340.peg.989	fig|6666666.59337.peg.5446	None	fig|6666666.71181.peg.3471	fig|6666666.59344.peg.2169	fig|6666666.59345.peg.5079	fig|6666666.59341.peg.4216	fig|6666666.59347.peg.3227	None	fig|6666666.59348.peg.1344	fig|6666666.59349.peg.1830	None	fig|6666666.59351.peg.5263	fig|6666666.59353.peg.230	fig|6666666.59355.peg.3001	fig|6666666.59354.peg.598

6141	FIG004436 Protein related to deoxyribodipyrimidine photolyase	None	fig|6666666.59330.peg.2355	fig|6666666.59331.peg.4849	fig|6666666.59332.peg.5348	fig|6666666.59334.peg.2900	fig|6666666.59335.peg.4567	fig|6666666.59336.peg.1135	fig|6666666.59333.peg.4241	fig|6666666.59338.peg.4633	fig|6666666.59339.peg.2013	fig|6666666.59340.peg.1018	fig|6666666.59337.peg.2226	None	None	None	fig|6666666.59345.peg.2992	fig|6666666.59341.peg.5247	fig|6666666.59347.peg.204	fig|6666666.59346.peg.4611	fig|6666666.59348.peg.470	fig|6666666.59349.peg.1425	fig|6666666.59350.peg.1135	fig|6666666.59351.peg.1148	None	None	fig|6666666.59354.peg.5280

4531	metal-dependent phosphohydrolase	fig|6666666.59329.peg.6186	fig|6666666.59330.peg.857	None	None	fig|6666666.59334.peg.2774	None	fig|6666666.59336.peg.3853	fig|6666666.59333.peg.67	None	None	None	fig|6666666.59337.peg.3277	fig|6666666.59342.peg.5513	fig|6666666.71181.peg.2920	fig|6666666.59344.peg.3482	fig|6666666.59345.peg.541	fig|6666666.59341.peg.5374	fig|6666666.59347.peg.1655	fig|6666666.59346.peg.3564	fig|6666666.59348.peg.1479	fig|6666666.59349.peg.2133	fig|6666666.59350.peg.5541	fig|6666666.59351.peg.2721	fig|6666666.59353.peg.1399	fig|6666666.59355.peg.2474	fig|6666666.59354.peg.1974

7980	LSU ribosomal protein L18p L5e	fig|6666666.59329.peg.725	fig|6666666.59330.peg.566	fig|6666666.59331.peg.36	fig|6666666.59332.peg.2151	fig|6666666.59334.peg.4018	fig|6666666.59335.peg.4359	fig|6666666.59336.peg.158	fig|6666666.59333.peg.378	fig|6666666.59338.peg.4970	fig|6666666.59339.peg.8279	None	fig|6666666.59337.peg.376	fig|6666666.59342.peg.316	None	None	fig|6666666.59345.peg.2523	fig|6666666.59341.peg.2510	fig|6666666.59347.peg.4835	fig|6666666.59346.peg.862	None	fig|6666666.59349.peg.3282	fig|6666666.59350.peg.3181	fig|6666666.59351.peg.4216	None	None	fig|6666666.59354.peg.3855

1978	FIG01201012 hypothetical protein	fig|6666666.59329.peg.290	fig|6666666.59330.peg.372	fig|6666666.59331.peg.5808	fig|6666666.59332.peg.741	None	fig|6666666.59335.peg.170	fig|6666666.59336.peg.2829	fig|6666666.59333.peg.3131	fig|6666666.59338.peg.1528	fig|6666666.59339.peg.7351	None	fig|6666666.59337.peg.342	fig|6666666.59342.peg.5130	fig|6666666.71181.peg.2811	fig|6666666.59344.peg.2139	fig|6666666.59345.peg.2490	fig|6666666.59341.peg.797	fig|6666666.59347.peg.3190	fig|6666666.59346.peg.1973	fig|6666666.59348.peg.267	None	None	None	fig|6666666.59353.peg.3169	fig|6666666.59355.peg.428	None

11106	PTS system, glucose-specific IIA component EC 2.7.1.69	fig|6666666.59329.peg.904	fig|6666666.59330.peg.3029	None	None	fig|6666666.59334.peg.3081	fig|6666666.59335.peg.3024	fig|6666666.59336.peg.2336	fig|6666666.59333.peg.4291	fig|6666666.59338.peg.3602	fig|6666666.59339.peg.5228	None	fig|6666666.59337.peg.5711	fig|6666666.59342.peg.113	fig|6666666.71181.peg.1580	fig|6666666.59344.peg.2004	fig|6666666.59345.peg.4395	fig|6666666.59341.peg.1630	fig|6666666.59347.peg.4442	fig|6666666.59346.peg.4794	None	fig|6666666.59349.peg.4798	None	fig|6666666.59351.peg.2818	fig|6666666.59353.peg.2919	fig|6666666.59355.peg.2820	None

9108	YheO-like PAS domain	fig|6666666.59329.peg.3695	fig|6666666.59330.peg.4075	None	fig|6666666.59332.peg.1074	fig|6666666.59334.peg.438	fig|6666666.59335.peg.6138	fig|6666666.59336.peg.2182	fig|6666666.59333.peg.3106	None	fig|6666666.59339.peg.8870	fig|6666666.59340.peg.944	fig|6666666.59337.peg.1820	fig|6666666.59342.peg.1919	fig|6666666.71181.peg.4309	fig|6666666.59344.peg.3128	fig|6666666.59345.peg.5555	fig|6666666.59341.peg.5931	fig|6666666.59347.peg.5335	fig|6666666.59346.peg.42	fig|6666666.59348.peg.3181	None	None	None	fig|6666666.59353.peg.2270	fig|6666666.59355.peg.1963	None

9363	Membrane-bound lytic murein transglycosylase A precursor EC 3.2.1.-	fig|6666666.59329.peg.3331	None	None	fig|6666666.59332.peg.869	fig|6666666.59334.peg.1530	fig|6666666.59335.peg.1118	fig|6666666.59336.peg.127	fig|6666666.59333.peg.5102	fig|6666666.59338.peg.4249	fig|6666666.59339.peg.6961	fig|6666666.59340.peg.778	fig|6666666.59337.peg.2363	None	fig|6666666.71181.peg.58	fig|6666666.59344.peg.2617	fig|6666666.59345.peg.2255	fig|6666666.59341.peg.531	fig|6666666.59347.peg.2818	fig|6666666.59346.peg.1845	fig|6666666.59348.peg.4824	None	fig|6666666.59350.peg.2702	None	fig|6666666.59353.peg.1959	fig|6666666.59355.peg.1447	None

614	Flagellar protein FliJ	fig|6666666.59329.peg.186	fig|6666666.59330.peg.1870	None	fig|6666666.59332.peg.4804	None	fig|6666666.59335.peg.392	fig|6666666.59336.peg.2872	None	fig|6666666.59338.peg.5670	None	None	fig|6666666.59337.peg.2962	fig|6666666.59342.peg.4348	fig|6666666.71181.peg.4024	fig|6666666.59344.peg.6210	fig|6666666.59345.peg.5341	fig|6666666.59341.peg.3270	fig|6666666.59347.peg.2687	fig|6666666.59346.peg.2882	fig|6666666.59348.peg.3259	fig|6666666.59349.peg.208	None	fig|6666666.59351.peg.2050	fig|6666666.59353.peg.4559	fig|6666666.59355.peg.5157	fig|6666666.59354.peg.1075

2065	ATPase involved in DNA repair	fig|6666666.59329.peg.1617	fig|6666666.59330.peg.1920	fig|6666666.59331.peg.1623	fig|6666666.59332.peg.3346	None	fig|6666666.59335.peg.3376	fig|6666666.59336.peg.6469	fig|6666666.59333.peg.2400	fig|6666666.59338.peg.4464	fig|6666666.59339.peg.3118	fig|6666666.59340.peg.2649	fig|6666666.59337.peg.4857	fig|6666666.59342.peg.4929	None	None	fig|6666666.59345.peg.1344	fig|6666666.59341.peg.53	fig|6666666.59347.peg.2401	fig|6666666.59346.peg.4150	fig|6666666.59348.peg.3442	fig|6666666.59349.peg.3443	None	fig|6666666.59351.peg.2730	None	None	fig|6666666.59354.peg.2203

1229	FIG01200899 hypothetical protein	fig|6666666.59329.peg.1051	fig|6666666.59330.peg.77	fig|6666666.59331.peg.3323	fig|6666666.59332.peg.4158	None	fig|6666666.59335.peg.3647	fig|6666666.59336.peg.3739	fig|6666666.59333.peg.2563	fig|6666666.59338.peg.3783	fig|6666666.59339.peg.5026	fig|6666666.59340.peg.131	fig|6666666.59337.peg.1310	fig|6666666.59342.peg.5390	None	None	fig|6666666.59345.peg.2329	fig|6666666.59341.peg.4735	fig|6666666.59347.peg.2965	fig|6666666.59346.peg.2455	fig|6666666.59348.peg.1796	fig|6666666.59349.peg.4322	None	fig|6666666.59351.peg.282	None	None	fig|6666666.59354.peg.5425

11062	Tryptophan synthase alpha chain EC 4.2.1.20	fig|6666666.59329.peg.397	fig|6666666.59330.peg.3338	None	fig|6666666.59332.peg.5154	fig|6666666.59334.peg.4447	fig|6666666.59335.peg.5379	fig|6666666.59336.peg.5397	fig|6666666.59333.peg.1764	None	fig|6666666.59339.peg.3631	None	fig|6666666.59337.peg.3502	None	fig|6666666.71181.peg.4142	fig|6666666.59344.peg.454	fig|6666666.59345.peg.920	fig|6666666.59341.peg.1018	fig|6666666.59347.peg.3558	None	fig|6666666.59348.peg.1828	fig|6666666.59349.peg.4278	fig|6666666.59350.peg.2694	fig|6666666.59351.peg.783	fig|6666666.59353.peg.1774	fig|6666666.59355.peg.1238	None

6391	TonB system biopolymer transport component; Chromosome segregation ATPase	fig|6666666.59329.peg.973	fig|6666666.59330.peg.1635	None	fig|6666666.59332.peg.3458	fig|6666666.59334.peg.5523	fig|6666666.59335.peg.4619	fig|6666666.59336.peg.827	fig|6666666.59333.peg.4614	fig|6666666.59338.peg.3521	fig|6666666.59339.peg.975	fig|6666666.59340.peg.512	fig|6666666.59337.peg.493	fig|6666666.59342.peg.572	None	None	fig|6666666.59345.peg.3053	fig|6666666.59341.peg.3488	fig|6666666.59347.peg.3059	fig|6666666.59346.peg.2469	fig|6666666.59348.peg.446	fig|6666666.59349.peg.2933	None	fig|6666666.59351.peg.2740	None	None	fig|6666666.59354.peg.3192

606	ABC-type sulfate transport system, permease component	fig|6666666.59329.peg.3231	None	fig|6666666.59331.peg.1401	fig|6666666.59332.peg.6165	fig|6666666.59334.peg.2302	fig|6666666.59335.peg.3400	fig|6666666.59336.peg.4007	fig|6666666.59333.peg.5281	fig|6666666.59338.peg.653	fig|6666666.59339.peg.4315	fig|6666666.59340.peg.362	fig|6666666.59337.peg.4704	fig|6666666.59342.peg.4326	None	None	fig|6666666.59345.peg.3118	fig|6666666.59341.peg.5260	fig|6666666.59347.peg.45	fig|6666666.59346.peg.4659	fig|6666666.59348.peg.2689	fig|6666666.59349.peg.2700	None	fig|6666666.59351.peg.3181	None	None	fig|6666666.59354.peg.5684

461	1,4-dihydroxy-2-naphthoate octaprenyltransferase EC 2.5.1.74	fig|6666666.59329.peg.6027	fig|6666666.59330.peg.5097	None	fig|6666666.59332.peg.171	fig|6666666.59334.peg.2765	fig|6666666.59335.peg.3554	fig|6666666.59336.peg.4990	fig|6666666.59333.peg.4825	fig|6666666.59338.peg.261	fig|6666666.59339.peg.8686	fig|6666666.59340.peg.24	fig|6666666.59337.peg.404	None	fig|6666666.71181.peg.3896	fig|6666666.59344.peg.997	fig|6666666.59345.peg.2548	fig|6666666.59341.peg.4139	fig|6666666.59347.peg.5025	fig|6666666.59346.peg.1013	fig|6666666.59348.peg.5351	None	None	None	fig|6666666.59353.peg.2542	fig|6666666.59355.peg.2659	None

7583	SAM-dependent methyltransferases	fig|6666666.59329.peg.1614	fig|6666666.59330.peg.1923	fig|6666666.59331.peg.5062	fig|6666666.59332.peg.3205	fig|6666666.59334.peg.4641	fig|6666666.59335.peg.3379	fig|6666666.59336.peg.6584	fig|6666666.59333.peg.4328	fig|6666666.59338.peg.2191	fig|6666666.59339.peg.3122	None	fig|6666666.59337.peg.4860	fig|6666666.59342.peg.3304	None	None	fig|6666666.59345.peg.1342	fig|6666666.59341.peg.55	fig|6666666.59347.peg.2399	fig|6666666.59346.peg.4153	fig|6666666.59348.peg.5423	fig|6666666.59349.peg.1131	None	fig|6666666.59351.peg.86	None	None	fig|6666666.59354.peg.4467

6074	Extracellular deoxyribonuclease Xds	fig|6666666.59329.peg.14	fig|6666666.59330.peg.1444	fig|6666666.59331.peg.5689	fig|6666666.59332.peg.4080	fig|6666666.59334.peg.677	fig|6666666.59335.peg.474	fig|6666666.59336.peg.276	fig|6666666.59333.peg.303	fig|6666666.59338.peg.3146	fig|6666666.59339.peg.6495	None	fig|6666666.59337.peg.1797	fig|6666666.59342.peg.5740	None	None	fig|6666666.59345.peg.5576	fig|6666666.59341.peg.5795	fig|6666666.59347.peg.4951	fig|6666666.59346.peg.15	fig|6666666.59348.peg.399	fig|6666666.59349.peg.876	None	fig|6666666.59351.peg.4990	None	None	fig|6666666.59354.peg.1264

2610	Predicted ATP-dependent endonuclease of the OLD family, YbjD subgroup	fig|6666666.59329.peg.5392	fig|6666666.59330.peg.4937	fig|6666666.59331.peg.3648	fig|6666666.59332.peg.3153	fig|6666666.59334.peg.1356	fig|6666666.59335.peg.2354	fig|6666666.59336.peg.3665	None	fig|6666666.59338.peg.5056	fig|6666666.59339.peg.2882	None	fig|6666666.59337.peg.2520	fig|6666666.59342.peg.553	fig|6666666.71181.peg.4134	fig|6666666.59344.peg.394	fig|6666666.59345.peg.123	fig|6666666.59341.peg.5711	fig|6666666.59347.peg.873	fig|6666666.59346.peg.2899	fig|6666666.59348.peg.3433	None	None	None	fig|6666666.59353.peg.1614	fig|6666666.59355.peg.1107	None

1055	FIG01200117 hypothetical protein	fig|6666666.59329.peg.2	fig|6666666.59330.peg.1457	fig|6666666.59331.peg.2882	fig|6666666.59332.peg.3186	fig|6666666.59334.peg.718	fig|6666666.59335.peg.1871	fig|6666666.59336.peg.288	fig|6666666.59333.peg.2358	fig|6666666.59338.peg.3243	fig|6666666.59339.peg.8237	fig|6666666.59340.peg.1011	fig|6666666.59337.peg.1808	fig|6666666.59342.peg.5741	None	None	fig|6666666.59345.peg.5565	fig|6666666.59341.peg.2772	fig|6666666.59347.peg.4964	fig|6666666.59346.peg.29	fig|6666666.59348.peg.285	fig|6666666.59349.peg.3374	None	fig|6666666.59351.peg.4174	None	None	None

5973	Leader peptidase Prepilin peptidase EC 3.4.23.43 N-methyltransferase EC 2.1.1.-	fig|6666666.59329.peg.3429	fig|6666666.59330.peg.4887	None	fig|6666666.59332.peg.308	fig|6666666.59334.peg.5394	fig|6666666.59335.peg.3806	fig|6666666.59336.peg.1426	fig|6666666.59333.peg.154	fig|6666666.59338.peg.2613	fig|6666666.59339.peg.7942	None	fig|6666666.59337.peg.2048	fig|6666666.59342.peg.3662	fig|6666666.71181.peg.3065	None	fig|6666666.59345.peg.2426	fig|6666666.59341.peg.1349	fig|6666666.59347.peg.3891	fig|6666666.59346.peg.576	fig|6666666.59348.peg.1266	fig|6666666.59349.peg.2628	None	fig|6666666.59351.peg.276	None	None	fig|6666666.59354.peg.5548

5976	Putative Bax protein	fig|6666666.59329.peg.4263	fig|6666666.59330.peg.3200	fig|6666666.59331.peg.6080	fig|6666666.59332.peg.5500	fig|6666666.59334.peg.879	fig|6666666.59335.peg.2215	fig|6666666.59336.peg.3527	fig|6666666.59333.peg.5506	fig|6666666.59338.peg.4111	fig|6666666.59339.peg.1313	None	fig|6666666.59337.peg.3714	fig|6666666.59342.peg.232	None	None	fig|6666666.59345.peg.3223	fig|6666666.59341.peg.5626	fig|6666666.59347.peg.1733	fig|6666666.59346.peg.1522	fig|6666666.59348.peg.4798	fig|6666666.59349.peg.167	None	fig|6666666.59351.peg.3682	None	None	fig|6666666.59354.peg.3883

5975	FIG01200634 hypothetical protein	fig|6666666.59329.peg.4416	fig|6666666.59330.peg.780	fig|6666666.59331.peg.5448	fig|6666666.59332.peg.6118	None	fig|6666666.59335.peg.544	fig|6666666.59336.peg.5546	fig|6666666.59333.peg.1519	fig|6666666.59338.peg.4414	fig|6666666.59339.peg.5772	None	fig|6666666.59337.peg.2099	fig|6666666.59342.peg.5525	fig|6666666.71181.peg.4466	fig|6666666.59344.peg.1986	fig|6666666.59345.peg.3335	fig|6666666.59341.peg.5546	fig|6666666.59347.peg.3323	fig|6666666.59346.peg.1220	fig|6666666.59348.peg.1793	None	None	None	fig|6666666.59353.peg.2934	fig|6666666.59355.peg.2806	None

2631	FIG01199556 hypothetical protein	None	fig|6666666.59330.peg.447	fig|6666666.59331.peg.5510	fig|6666666.59332.peg.426	fig|6666666.59334.peg.3278	fig|6666666.59335.peg.5862	fig|6666666.59336.peg.3377	fig|6666666.59333.peg.1155	fig|6666666.59338.peg.3197	fig|6666666.59339.peg.5137	None	fig|6666666.59337.peg.1602	fig|6666666.59342.peg.2983	fig|6666666.71181.peg.3921	fig|6666666.59344.peg.5401	fig|6666666.59345.peg.2181	fig|6666666.59341.peg.5458	fig|6666666.59347.peg.4378	fig|6666666.59346.peg.4887	fig|6666666.59348.peg.637	None	None	None	fig|6666666.59353.peg.4778	fig|6666666.59355.peg.4829	None

1533	FIG00920830 hypothetical protein	fig|6666666.59329.peg.3649	fig|6666666.59330.peg.1262	fig|6666666.59331.peg.2801	fig|6666666.59332.peg.2103	fig|6666666.59334.peg.1897	fig|6666666.59335.peg.2822	fig|6666666.59336.peg.468	fig|6666666.59333.peg.1842	fig|6666666.59338.peg.2133	fig|6666666.59339.peg.3486	fig|6666666.59340.peg.1086	fig|6666666.59337.peg.2206	None	fig|6666666.71181.peg.550	fig|6666666.59344.peg.1917	fig|6666666.59345.peg.1583	fig|6666666.59341.peg.3942	fig|6666666.59347.peg.4171	fig|6666666.59346.peg.3243	None	None	None	None	fig|6666666.59353.peg.1180	fig|6666666.59355.peg.295	None

8191	NnrS protein involved in response to NO	fig|6666666.59329.peg.1134	fig|6666666.59330.peg.2815	fig|6666666.59331.peg.4824	fig|6666666.59332.peg.1759	fig|6666666.59334.peg.3308	fig|6666666.59335.peg.3695	fig|6666666.59336.peg.3243	fig|6666666.59333.peg.3446	fig|6666666.59338.peg.5966	fig|6666666.59339.peg.6879	None	fig|6666666.59337.peg.1360	fig|6666666.59342.peg.3739	None	None	fig|6666666.59345.peg.2283	fig|6666666.59341.peg.288	fig|6666666.59347.peg.2952	fig|6666666.59346.peg.2245	fig|6666666.59348.peg.2554	fig|6666666.59349.peg.2966	None	fig|6666666.59351.peg.4121	None	None	fig|6666666.59354.peg.3100

7421	LSU ribosomal protein L36p	fig|6666666.59329.peg.730	fig|6666666.59330.peg.572	fig|6666666.59331.peg.31	fig|6666666.59332.peg.5649	fig|6666666.59334.peg.3044	fig|6666666.59335.peg.4364	None	fig|6666666.59333.peg.384	fig|6666666.59338.peg.5849	fig|6666666.59339.peg.8286	fig|6666666.59340.peg.2220	fig|6666666.59337.peg.371	fig|6666666.59342.peg.322	None	None	fig|6666666.59345.peg.2518	fig|6666666.59341.peg.2516	fig|6666666.59347.peg.4841	fig|6666666.59346.peg.867	fig|6666666.59348.peg.437	fig|6666666.59349.peg.3287	None	fig|6666666.59351.peg.4222	None	None	fig|6666666.59354.peg.3250

8239	4-hydroxyphenylpyruvate dioxygenase EC 1.13.11.27	fig|6666666.59329.peg.6044	fig|6666666.59330.peg.3831	None	fig|6666666.59332.peg.4543	None	fig|6666666.59335.peg.1549	fig|6666666.59336.peg.6441	fig|6666666.59333.peg.441	fig|6666666.59338.peg.4988	None	fig|6666666.59340.peg.76	fig|6666666.59337.peg.2013	fig|6666666.59342.peg.4678	None	None	fig|6666666.59345.peg.3080	fig|6666666.59341.peg.607	fig|6666666.59347.peg.3732	fig|6666666.59346.peg.4918	fig|6666666.59348.peg.4632	fig|6666666.59349.peg.4010	fig|6666666.59350.peg.299	fig|6666666.59351.peg.1420	None	fig|6666666.59355.peg.5860	fig|6666666.59354.peg.1516

9643	membrane protein	fig|6666666.59329.peg.3150	fig|6666666.59330.peg.160	fig|6666666.59331.peg.3428	fig|6666666.59332.peg.2926	None	fig|6666666.59335.peg.3550	fig|6666666.59336.peg.253	fig|6666666.59333.peg.428	fig|6666666.59338.peg.3736	fig|6666666.59339.peg.8691	None	fig|6666666.59337.peg.4330	None	fig|6666666.71181.peg.5137	fig|6666666.59344.peg.3791	fig|6666666.59345.peg.5112	fig|6666666.59341.peg.175	fig|6666666.59347.peg.4552	fig|6666666.59346.peg.979	fig|6666666.59348.peg.2572	None	None	fig|6666666.59351.peg.3271	fig|6666666.59353.peg.1861	fig|6666666.59355.peg.1152	None

5952	UPF0265 protein YeeX	fig|6666666.59329.peg.6063	fig|6666666.59330.peg.2120	None	fig|6666666.59332.peg.4383	fig|6666666.59334.peg.4478	fig|6666666.59335.peg.6199	fig|6666666.59336.peg.4933	fig|6666666.59333.peg.98	fig|6666666.59338.peg.2274	fig|6666666.59339.peg.2810	None	fig|6666666.59337.peg.4584	fig|6666666.59342.peg.2316	fig|6666666.71181.peg.3558	fig|6666666.59344.peg.5505	fig|6666666.59345.peg.509	fig|6666666.59341.peg.5741	fig|6666666.59347.peg.880	fig|6666666.59346.peg.3306	fig|6666666.59348.peg.827	None	None	None	fig|6666666.59353.peg.4822	fig|6666666.59355.peg.4898	None

3567	Uncharacterized protein YrbB	fig|6666666.59329.peg.1451	fig|6666666.59330.peg.55	None	fig|6666666.59332.peg.1278	None	fig|6666666.59335.peg.218	None	fig|6666666.59333.peg.274	fig|6666666.59338.peg.118	None	None	fig|6666666.59337.peg.842	fig|6666666.59342.peg.440	fig|6666666.71181.peg.1028	fig|6666666.59344.peg.1808	fig|6666666.59345.peg.4851	fig|6666666.59341.peg.6088	fig|6666666.59347.peg.3241	fig|6666666.59346.peg.629	fig|6666666.59348.peg.3522	fig|6666666.59349.peg.31	None	fig|6666666.59351.peg.3452	fig|6666666.59353.peg.2347	fig|6666666.59355.peg.1620	fig|6666666.59354.peg.347

8571	Dienelactone hydrolase family	fig|6666666.59329.peg.6058	None	fig|6666666.59331.peg.458	fig|6666666.59332.peg.5405	None	fig|6666666.59335.peg.979	fig|6666666.59336.peg.3024	fig|6666666.59333.peg.1038	fig|6666666.59338.peg.4256	fig|6666666.59339.peg.4470	fig|6666666.59340.peg.1608	fig|6666666.59337.peg.3882	fig|6666666.59342.peg.4149	fig|6666666.71181.peg.3346	fig|6666666.59344.peg.2935	fig|6666666.59345.peg.287	fig|6666666.59341.peg.2270	fig|6666666.59347.peg.1506	fig|6666666.59346.peg.2413	fig|6666666.59348.peg.3993	None	None	None	fig|6666666.59353.peg.3792	fig|6666666.59355.peg.3169	None

11141	Resolvase integrase Bin	fig|6666666.59329.peg.3473	fig|6666666.59330.peg.1800	fig|6666666.59331.peg.218	None	None	fig|6666666.59335.peg.4848	fig|6666666.59336.peg.3284	fig|6666666.59333.peg.2276	fig|6666666.59338.peg.735	fig|6666666.59339.peg.3939	None	fig|6666666.59337.peg.3411	fig|6666666.59342.peg.3919	fig|6666666.71181.peg.5365	None	fig|6666666.59345.peg.1981	fig|6666666.59341.peg.3875	fig|6666666.59347.peg.2595	fig|6666666.59346.peg.837	fig|6666666.59348.peg.1934	fig|6666666.59349.peg.4894	fig|6666666.59350.peg.7815	fig|6666666.59351.peg.2497	None	fig|6666666.59355.peg.6067	None

1514	Integral membrane protein YggT, involved in response to extracytoplasmic stress osmotic shock	None	fig|6666666.59330.peg.340	None	fig|6666666.59332.peg.1631	None	fig|6666666.59335.peg.3086	fig|6666666.59336.peg.4104	fig|6666666.59333.peg.765	None	fig|6666666.59339.peg.7823	None	fig|6666666.59337.peg.521	fig|6666666.59342.peg.2688	fig|6666666.71181.peg.1867	fig|6666666.59344.peg.4342	fig|6666666.59345.peg.5010	None	fig|6666666.59347.peg.3867	fig|6666666.59346.peg.1778	fig|6666666.59348.peg.226	fig|6666666.59349.peg.798	fig|6666666.59350.peg.8090	fig|6666666.59351.peg.3528	fig|6666666.59353.peg.3233	fig|6666666.59355.peg.3115	fig|6666666.59354.peg.528

3396	YbbM seven transmembrane helix protein	fig|6666666.59329.peg.6513	fig|6666666.59330.peg.1522	None	fig|6666666.59332.peg.3192	None	fig|6666666.59335.peg.5543	fig|6666666.59336.peg.2591	fig|6666666.59333.peg.3614	fig|6666666.59338.peg.4546	fig|6666666.59339.peg.359	fig|6666666.59340.peg.3215	fig|6666666.59337.peg.2311	fig|6666666.59342.peg.3908	fig|6666666.71181.peg.1088	fig|6666666.59344.peg.3590	fig|6666666.59345.peg.1287	fig|6666666.59341.peg.5395	fig|6666666.59347.peg.2308	fig|6666666.59346.peg.4558	None	None	None	fig|6666666.59351.peg.4592	fig|6666666.59353.peg.2150	fig|6666666.59355.peg.1845	None

2672	Predicted acyltransferase	fig|6666666.59329.peg.2426	fig|6666666.59330.peg.4559	None	fig|6666666.59332.peg.3159	fig|6666666.59334.peg.2118	fig|6666666.59335.peg.417	fig|6666666.59336.peg.3401	fig|6666666.59333.peg.4949	fig|6666666.59338.peg.1493	fig|6666666.59339.peg.6363	fig|6666666.59340.peg.887	fig|6666666.59337.peg.5513	fig|6666666.59342.peg.1704	None	None	fig|6666666.59345.peg.5713	fig|6666666.59341.peg.4653	fig|6666666.59347.peg.5032	fig|6666666.59346.peg.4265	fig|6666666.59348.peg.2308	fig|6666666.59349.peg.3771	None	fig|6666666.59351.peg.4284	None	None	fig|6666666.59354.peg.3698

5333	Exodeoxyribonuclease VII small subunit EC 3.1.11.6	fig|6666666.59329.peg.441	None	None	None	fig|6666666.59334.peg.1923	fig|6666666.59335.peg.3896	fig|6666666.59336.peg.3165	fig|6666666.59333.peg.4916	fig|6666666.59338.peg.3893	fig|6666666.59339.peg.7171	None	fig|6666666.59337.peg.1131	fig|6666666.59342.peg.3898	None	fig|6666666.59344.peg.1088	fig|6666666.59345.peg.3533	fig|6666666.59341.peg.2520	fig|6666666.59347.peg.2876	fig|6666666.59346.peg.4088	fig|6666666.59348.peg.2888	fig|6666666.59349.peg.2778	fig|6666666.59350.peg.855	fig|6666666.59351.peg.5454	fig|6666666.59353.peg.770	fig|6666666.59355.peg.934	None

10898	Cytochrome c oxidase subunit CcoO EC 1.9.3.1	fig|6666666.59329.peg.1338	fig|6666666.59330.peg.3189	fig|6666666.59331.peg.1450	fig|6666666.59332.peg.5536	fig|6666666.59334.peg.5217	fig|6666666.59335.peg.3539	fig|6666666.59336.peg.6481	fig|6666666.59333.peg.4303	fig|6666666.59338.peg.846	fig|6666666.59339.peg.1326	None	fig|6666666.59337.peg.1446	fig|6666666.59342.peg.588	None	None	fig|6666666.59345.peg.3236	fig|6666666.59341.peg.4656	fig|6666666.59347.peg.458	fig|6666666.59346.peg.5322	None	fig|6666666.59349.peg.136	fig|6666666.59350.peg.1956	fig|6666666.59351.peg.835	None	None	fig|6666666.59354.peg.2591

1754	General secretion pathway protein H	fig|6666666.59329.peg.1224	fig|6666666.59330.peg.421	fig|6666666.59331.peg.6366	fig|6666666.59332.peg.1671	fig|6666666.59334.peg.124	fig|6666666.59335.peg.714	fig|6666666.59336.peg.3118	fig|6666666.59333.peg.509	None	fig|6666666.59339.peg.6205	fig|6666666.59340.peg.647	fig|6666666.59337.peg.4247	fig|6666666.59342.peg.3766	None	None	fig|6666666.59345.peg.2673	fig|6666666.59341.peg.4540	fig|6666666.59347.peg.4467	fig|6666666.59346.peg.2224	fig|6666666.59348.peg.4328	fig|6666666.59349.peg.2171	None	fig|6666666.59351.peg.4343	None	None	fig|6666666.59354.peg.701

6783	ubiquinol cytochrome C oxidoreductase, cytochrome C1 subunit	fig|6666666.59329.peg.2054	fig|6666666.59330.peg.966	fig|6666666.59331.peg.788	fig|6666666.59332.peg.646	fig|6666666.59334.peg.2337	fig|6666666.59335.peg.4925	None	fig|6666666.59333.peg.616	fig|6666666.59338.peg.4706	fig|6666666.59339.peg.4945	fig|6666666.59340.peg.523	fig|6666666.59337.peg.31	None	None	None	fig|6666666.59345.peg.3811	fig|6666666.59341.peg.3545	fig|6666666.59347.peg.3926	fig|6666666.59346.peg.2334	fig|6666666.59348.peg.1445	fig|6666666.59349.peg.43	fig|6666666.59350.peg.2359	fig|6666666.59351.peg.3464	None	None	fig|6666666.59354.peg.361

3693	FIG01199667 hypothetical protein	fig|6666666.59329.peg.3892	fig|6666666.59330.peg.476	fig|6666666.59331.peg.5782	fig|6666666.59332.peg.982	fig|6666666.59334.peg.1033	fig|6666666.59335.peg.3903	fig|6666666.59336.peg.4976	fig|6666666.59333.peg.2234	fig|6666666.59338.peg.3396	fig|6666666.59339.peg.7179	None	fig|6666666.59337.peg.1574	fig|6666666.59342.peg.3469	None	None	fig|6666666.59345.peg.2208	fig|6666666.59341.peg.2608	fig|6666666.59347.peg.2774	fig|6666666.59346.peg.5002	fig|6666666.59348.peg.3853	fig|6666666.59349.peg.1351	None	fig|6666666.59351.peg.5486	None	None	fig|6666666.59354.peg.2626

3696	Endonuclease IV EC 3.1.21.2	fig|6666666.59329.peg.112	fig|6666666.59330.peg.1727	None	fig|6666666.59332.peg.2905	fig|6666666.59334.peg.4080	fig|6666666.59335.peg.812	fig|6666666.59336.peg.3410	fig|6666666.59333.peg.3412	fig|6666666.59338.peg.70	fig|6666666.59339.peg.4998	None	fig|6666666.59337.peg.96	fig|6666666.59342.peg.1360	fig|6666666.71181.peg.3185	fig|6666666.59344.peg.1662	fig|6666666.59345.peg.3747	fig|6666666.59341.peg.1143	fig|6666666.59347.peg.3067	fig|6666666.59346.peg.1375	fig|6666666.59348.peg.2697	None	None	None	fig|6666666.59353.peg.1083	fig|6666666.59355.peg.396	None

2156	Exoenzymes regulatory protein AepA in lipid-linked oligosaccharide synthesis cluster	fig|6666666.59329.peg.3619	None	fig|6666666.59331.peg.1594	fig|6666666.59332.peg.1178	None	fig|6666666.59335.peg.3160	fig|6666666.59336.peg.4315	None	fig|6666666.59338.peg.4413	fig|6666666.59339.peg.2345	fig|6666666.59340.peg.822	fig|6666666.59337.peg.5315	fig|6666666.59342.peg.6340	None	None	fig|6666666.59345.peg.4432	fig|6666666.59341.peg.4354	fig|6666666.59347.peg.2759	fig|6666666.59346.peg.2099	fig|6666666.59348.peg.5413	fig|6666666.59349.peg.835	fig|6666666.59350.peg.1393	fig|6666666.59351.peg.3379	None	fig|6666666.59355.peg.5247	fig|6666666.59354.peg.5230

3529	Electron transport complex protein RnfG	fig|6666666.59329.peg.784	fig|6666666.59330.peg.3376	None	fig|6666666.59332.peg.2572	None	fig|6666666.59335.peg.884	fig|6666666.59336.peg.4281	fig|6666666.59333.peg.4815	None	fig|6666666.59339.peg.54	None	fig|6666666.59337.peg.4670	fig|6666666.59342.peg.5226	fig|6666666.71181.peg.2903	fig|6666666.59344.peg.3459	fig|6666666.59345.peg.1125	fig|6666666.59341.peg.2704	fig|6666666.59347.peg.5389	fig|6666666.59346.peg.4769	fig|6666666.59348.peg.89	fig|6666666.59349.peg.4633	None	fig|6666666.59351.peg.5075	fig|6666666.59353.peg.1417	fig|6666666.59355.peg.2457	None

1143	FIG018429 hypothetical protein	fig|6666666.59329.peg.5010	fig|6666666.59330.peg.3021	None	fig|6666666.59332.peg.4270	fig|6666666.59334.peg.2615	fig|6666666.59335.peg.3138	fig|6666666.59336.peg.773	fig|6666666.59333.peg.4740	fig|6666666.59338.peg.242	fig|6666666.59339.peg.5442	fig|6666666.59340.peg.712	fig|6666666.59337.peg.231	fig|6666666.59342.peg.5501	None	None	fig|6666666.59345.peg.5268	fig|6666666.59341.peg.3816	fig|6666666.59347.peg.10	fig|6666666.59346.peg.144	fig|6666666.59348.peg.2047	fig|6666666.59349.peg.3770	None	fig|6666666.59351.peg.4285	None	None	fig|6666666.59354.peg.779

766	Flagellar protein FlgP	fig|6666666.59329.peg.570	fig|6666666.59330.peg.2696	None	fig|6666666.59332.peg.2633	None	fig|6666666.59335.peg.2074	fig|6666666.59336.peg.3754	fig|6666666.59333.peg.203	fig|6666666.59338.peg.624	fig|6666666.59339.peg.5305	fig|6666666.59340.peg.3724	fig|6666666.59337.peg.3340	fig|6666666.59342.peg.5345	None	None	fig|6666666.59345.peg.5443	fig|6666666.59341.peg.4562	fig|6666666.59347.peg.2720	fig|6666666.59346.peg.1756	fig|6666666.59348.peg.4695	fig|6666666.59349.peg.714	fig|6666666.59350.peg.6887	fig|6666666.59351.peg.2567	None	None	fig|6666666.59354.peg.4323

4281	FIG01199629 hypothetical protein	fig|6666666.59329.peg.5391	fig|6666666.59330.peg.4938	fig|6666666.59331.peg.3646	fig|6666666.59332.peg.3154	None	fig|6666666.59335.peg.2353	fig|6666666.59336.peg.3664	fig|6666666.59333.peg.3751	fig|6666666.59338.peg.5055	fig|6666666.59339.peg.2881	None	fig|6666666.59337.peg.2519	fig|6666666.59342.peg.554	fig|6666666.71181.peg.524	fig|6666666.59344.peg.1953	fig|6666666.59345.peg.126	fig|6666666.59341.peg.4260	fig|6666666.59347.peg.872	fig|6666666.59346.peg.2900	fig|6666666.59348.peg.3432	None	None	None	fig|6666666.59353.peg.1153	fig|6666666.59355.peg.324	None

8701	FIG138517 Putative lipid carrier protein	fig|6666666.59329.peg.2305	fig|6666666.59330.peg.94	None	fig|6666666.59332.peg.4404	fig|6666666.59334.peg.4221	fig|6666666.59335.peg.2108	fig|6666666.59336.peg.3564	fig|6666666.59333.peg.2312	fig|6666666.59338.peg.6022	fig|6666666.59339.peg.4887	None	fig|6666666.59337.peg.695	fig|6666666.59342.peg.3205	fig|6666666.71181.peg.3534	fig|6666666.59344.peg.2074	fig|6666666.59345.peg.2345	fig|6666666.59341.peg.5174	fig|6666666.59347.peg.2991	fig|6666666.59346.peg.1988	fig|6666666.59348.peg.962	None	None	None	fig|6666666.59353.peg.3112	fig|6666666.59355.peg.485	None

4770	Copper metallochaperone, bacterial analog of Cox17 protein	fig|6666666.59329.peg.1690	fig|6666666.59330.peg.3461	fig|6666666.59331.peg.1789	fig|6666666.59332.peg.4435	fig|6666666.59334.peg.3515	fig|6666666.59335.peg.3071	fig|6666666.59336.peg.5146	None	fig|6666666.59338.peg.1535	fig|6666666.59339.peg.5747	None	fig|6666666.59337.peg.4808	fig|6666666.59342.peg.4628	None	None	fig|6666666.59345.peg.312	fig|6666666.59341.peg.807	fig|6666666.59347.peg.3795	fig|6666666.59346.peg.1326	None	fig|6666666.59349.peg.97	fig|6666666.59350.peg.5342	fig|6666666.59351.peg.154	None	fig|6666666.59355.peg.5209	fig|6666666.59354.peg.424

828	putative lipoprotein	fig|6666666.59329.peg.5419	fig|6666666.59330.peg.1236	None	fig|6666666.59332.peg.4927	fig|6666666.59334.peg.5265	fig|6666666.59335.peg.1307	fig|6666666.59336.peg.1618	fig|6666666.59333.peg.1868	fig|6666666.59338.peg.1571	fig|6666666.59339.peg.3441	fig|6666666.59340.peg.2950	fig|6666666.59337.peg.2177	fig|6666666.59342.peg.490	None	None	fig|6666666.59345.peg.1607	fig|6666666.59341.peg.271	fig|6666666.59347.peg.4068	fig|6666666.59346.peg.4969	fig|6666666.59348.peg.812	fig|6666666.59349.peg.2695	None	fig|6666666.59351.peg.5171	None	None	fig|6666666.59354.peg.4455

10702	General secretion pathway protein C	fig|6666666.59329.peg.3574	fig|6666666.59330.peg.416	None	fig|6666666.59332.peg.1676	fig|6666666.59334.peg.132	fig|6666666.59335.peg.721	fig|6666666.59336.peg.448	fig|6666666.59333.peg.503	fig|6666666.59338.peg.1797	fig|6666666.59339.peg.6194	fig|6666666.59340.peg.1434	fig|6666666.59337.peg.4241	fig|6666666.59342.peg.2914	None	None	fig|6666666.59345.peg.2678	fig|6666666.59341.peg.3429	fig|6666666.59347.peg.4462	fig|6666666.59346.peg.2597	fig|6666666.59348.peg.2077	fig|6666666.59349.peg.2176	None	fig|6666666.59351.peg.4349	None	None	fig|6666666.59354.peg.694

7765	FIG01199926 hypothetical protein	fig|6666666.59329.peg.1799	fig|6666666.59330.peg.1562	None	None	fig|6666666.59334.peg.1287	fig|6666666.59335.peg.4275	fig|6666666.59336.peg.3161	fig|6666666.59333.peg.3182	fig|6666666.59338.peg.522	fig|6666666.59339.peg.8563	fig|6666666.59340.peg.87	fig|6666666.59337.peg.1646	fig|6666666.59342.peg.4955	fig|6666666.71181.peg.4505	fig|6666666.59344.peg.5710	fig|6666666.59345.peg.5474	fig|6666666.59341.peg.5363	fig|6666666.59347.peg.5058	fig|6666666.59346.peg.3708	fig|6666666.59348.peg.580	None	None	None	fig|6666666.59353.peg.4933	fig|6666666.59355.peg.3360	None

928	DNA polymerase-like protein PA0670	fig|6666666.59329.peg.4531	fig|6666666.59330.peg.1394	fig|6666666.59331.peg.1739	None	fig|6666666.59334.peg.358	fig|6666666.59335.peg.5297	fig|6666666.59336.peg.4096	fig|6666666.59333.peg.728	fig|6666666.59338.peg.2678	fig|6666666.59339.peg.3201	fig|6666666.59340.peg.1057	fig|6666666.59337.peg.2659	fig|6666666.59342.peg.5541	None	None	fig|6666666.59345.peg.1043	fig|6666666.59341.peg.37	fig|6666666.59347.peg.4158	fig|6666666.59346.peg.126	fig|6666666.59348.peg.4122	fig|6666666.59349.peg.560	fig|6666666.59350.peg.487	fig|6666666.59351.peg.1433	None	None	None

9576	Protein yihD	fig|6666666.59329.peg.54	fig|6666666.59330.peg.633	None	fig|6666666.59332.peg.217	fig|6666666.59334.peg.1393	fig|6666666.59335.peg.2442	fig|6666666.59336.peg.2740	fig|6666666.59333.peg.3	fig|6666666.59338.peg.576	fig|6666666.59339.peg.8081	fig|6666666.59340.peg.1390	fig|6666666.59337.peg.3892	fig|6666666.59342.peg.4033	fig|6666666.71181.peg.2579	fig|6666666.59344.peg.888	fig|6666666.59345.peg.4936	fig|6666666.59341.peg.1944	fig|6666666.59347.peg.4755	None	fig|6666666.59348.peg.58	None	None	None	fig|6666666.59353.peg.2638	fig|6666666.59355.peg.2567	None

7153	FIG01199889 hypothetical protein	fig|6666666.59329.peg.3945	fig|6666666.59330.peg.1252	fig|6666666.59331.peg.5112	fig|6666666.59332.peg.4089	fig|6666666.59334.peg.3914	fig|6666666.59335.peg.5116	fig|6666666.59336.peg.2757	fig|6666666.59333.peg.1827	fig|6666666.59338.peg.5854	fig|6666666.59339.peg.3464	fig|6666666.59340.peg.488	fig|6666666.59337.peg.2194	None	None	None	fig|6666666.59345.peg.1593	fig|6666666.59341.peg.249	fig|6666666.59347.peg.4101	fig|6666666.59346.peg.136	fig|6666666.59348.peg.4297	fig|6666666.59349.peg.2506	None	fig|6666666.59351.peg.3709	None	None	fig|6666666.59354.peg.722

2896	Endonuclease exonuclease phosphatase family protein	fig|6666666.59329.peg.5930	fig|6666666.59330.peg.4751	None	fig|6666666.59332.peg.1641	fig|6666666.59334.peg.3951	fig|6666666.59335.peg.2605	fig|6666666.59336.peg.4695	fig|6666666.59333.peg.3991	fig|6666666.59338.peg.3361	fig|6666666.59339.peg.2250	fig|6666666.59340.peg.775	fig|6666666.59337.peg.2483	fig|6666666.59342.peg.2892	None	None	fig|6666666.59345.peg.158	fig|6666666.59341.peg.3708	fig|6666666.59347.peg.369	fig|6666666.59346.peg.466	fig|6666666.59348.peg.3484	fig|6666666.59349.peg.409	None	fig|6666666.59351.peg.4794	None	None	fig|6666666.59354.peg.1247

11257	FIG01199930 hypothetical protein	fig|6666666.59329.peg.6118	fig|6666666.59330.peg.1578	fig|6666666.59331.peg.2786	fig|6666666.59332.peg.3840	fig|6666666.59334.peg.3403	fig|6666666.59335.peg.1259	fig|6666666.59336.peg.4644	fig|6666666.59333.peg.4273	fig|6666666.59338.peg.4177	fig|6666666.59339.peg.3043	None	fig|6666666.59337.peg.4231	fig|6666666.59342.peg.2723	None	None	fig|6666666.59345.peg.5302	fig|6666666.59341.peg.852	fig|6666666.59347.peg.5103	fig|6666666.59346.peg.2164	fig|6666666.59348.peg.5519	fig|6666666.59349.peg.4454	None	fig|6666666.59351.peg.693	None	None	fig|6666666.59354.peg.4915

1733	Thiaminase II EC 3.5.99.2	fig|6666666.59329.peg.3208	fig|6666666.59330.peg.4728	fig|6666666.59331.peg.4526	fig|6666666.59332.peg.2529	fig|6666666.59334.peg.909	fig|6666666.59335.peg.6213	fig|6666666.59336.peg.3926	fig|6666666.59333.peg.3401	fig|6666666.59338.peg.466	fig|6666666.59339.peg.5552	None	fig|6666666.59337.peg.1221	None	fig|6666666.71181.peg.4174	fig|6666666.59344.peg.3257	fig|6666666.59345.peg.4405	fig|6666666.59341.peg.3561	fig|6666666.59347.peg.4310	fig|6666666.59346.peg.4174	fig|6666666.59348.peg.2768	None	None	None	fig|6666666.59353.peg.2814	fig|6666666.59355.peg.3729	None

9933	Carboxynorspermidine decarboxylase, putative EC 4.1.1.-	fig|6666666.59329.peg.4748	fig|6666666.59330.peg.4909	fig|6666666.59331.peg.5305	fig|6666666.59332.peg.2221	fig|6666666.59334.peg.4484	fig|6666666.59335.peg.1327	fig|6666666.59336.peg.1780	fig|6666666.59333.peg.3804	fig|6666666.59338.peg.2523	fig|6666666.59339.peg.933	fig|6666666.59340.peg.3668	fig|6666666.59337.peg.1747	fig|6666666.59342.peg.3716	None	None	fig|6666666.59345.peg.898	fig|6666666.59341.peg.387	fig|6666666.59347.peg.2284	fig|6666666.59346.peg.1356	fig|6666666.59348.peg.4166	fig|6666666.59349.peg.4909	None	None	None	fig|6666666.59355.peg.5189	None

9550	Sodium glutamate symport protein	fig|6666666.59329.peg.1393	fig|6666666.59330.peg.1463	fig|6666666.59331.peg.1801	fig|6666666.59332.peg.3068	fig|6666666.59334.peg.4529	fig|6666666.59335.peg.5641	fig|6666666.59336.peg.2523	fig|6666666.59333.peg.1215	fig|6666666.59338.peg.228	fig|6666666.59339.peg.8377	None	fig|6666666.59337.peg.213	fig|6666666.59342.peg.4301	fig|6666666.71181.peg.2601	fig|6666666.59344.peg.918	fig|6666666.59345.peg.5289	fig|6666666.59341.peg.5640	fig|6666666.59347.peg.1881	fig|6666666.59346.peg.1801	None	None	None	None	fig|6666666.59353.peg.2612	fig|6666666.59355.peg.2591	None

4828	General secretion pathway protein M	fig|6666666.59329.peg.1232	fig|6666666.59330.peg.426	fig|6666666.59331.peg.5440	fig|6666666.59332.peg.2998	fig|6666666.59334.peg.117	fig|6666666.59335.peg.709	fig|6666666.59336.peg.3111	fig|6666666.59333.peg.514	fig|6666666.59338.peg.1430	fig|6666666.59339.peg.6211	fig|6666666.59340.peg.725	fig|6666666.59337.peg.4254	None	None	None	fig|6666666.59345.peg.2668	fig|6666666.59341.peg.4546	fig|6666666.59347.peg.4472	fig|6666666.59346.peg.2229	fig|6666666.59348.peg.1394	fig|6666666.59349.peg.2166	None	fig|6666666.59351.peg.4338	None	None	fig|6666666.59354.peg.707

180	FIG01203079 hypothetical protein	fig|6666666.59329.peg.3232	None	fig|6666666.59331.peg.1399	fig|6666666.59332.peg.4648	fig|6666666.59334.peg.2538	fig|6666666.59335.peg.3401	fig|6666666.59336.peg.4008	fig|6666666.59333.peg.5283	fig|6666666.59338.peg.651	fig|6666666.59339.peg.4316	fig|6666666.59340.peg.363	fig|6666666.59337.peg.4705	fig|6666666.59342.peg.4325	None	None	fig|6666666.59345.peg.3119	fig|6666666.59341.peg.5259	fig|6666666.59347.peg.44	fig|6666666.59346.peg.4660	fig|6666666.59348.peg.2688	fig|6666666.59349.peg.1825	None	fig|6666666.59351.peg.5258	None	None	fig|6666666.59354.peg.39

9334	RecA RadA recombinase	fig|6666666.59329.peg.4533	fig|6666666.59330.peg.1393	fig|6666666.59331.peg.1741	fig|6666666.59332.peg.2409	fig|6666666.59334.peg.360	fig|6666666.59335.peg.847	fig|6666666.59336.peg.4095	fig|6666666.59333.peg.729	fig|6666666.59338.peg.2680	fig|6666666.59339.peg.3205	None	fig|6666666.59337.peg.5675	fig|6666666.59342.peg.4104	None	None	fig|6666666.59345.peg.1042	fig|6666666.59341.peg.40	fig|6666666.59347.peg.4159	fig|6666666.59346.peg.127	fig|6666666.59348.peg.4124	fig|6666666.59349.peg.559	None	fig|6666666.59351.peg.1434	None	None	fig|6666666.59354.peg.6288

2563	Soluble cytochrome b562	fig|6666666.59329.peg.1545	fig|6666666.59330.peg.2209	None	fig|6666666.59332.peg.4144	None	fig|6666666.59335.peg.5889	fig|6666666.59336.peg.2535	fig|6666666.59333.peg.2857	fig|6666666.59338.peg.5185	fig|6666666.59339.peg.2444	None	fig|6666666.59337.peg.1895	None	fig|6666666.71181.peg.996	None	fig|6666666.59345.peg.2840	fig|6666666.59341.peg.2469	fig|6666666.59347.peg.906	fig|6666666.59346.peg.3643	fig|6666666.59348.peg.5157	fig|6666666.59349.peg.4608	None	fig|6666666.59351.peg.1558	fig|6666666.59353.peg.2312	fig|6666666.59355.peg.1587	fig|6666666.59354.peg.2910

4891	YcfL protein an outer membrane lipoprotein that is part of a salvage cluster	fig|6666666.59329.peg.5420	fig|6666666.59330.peg.1238	None	fig|6666666.59332.peg.4926	fig|6666666.59334.peg.5266	fig|6666666.59335.peg.1309	fig|6666666.59336.peg.1619	fig|6666666.59333.peg.1869	fig|6666666.59338.peg.1570	fig|6666666.59339.peg.3443	None	fig|6666666.59337.peg.2180	fig|6666666.59342.peg.491	fig|6666666.71181.peg.1638	fig|6666666.59344.peg.5021	fig|6666666.59345.peg.1606	fig|6666666.59341.peg.270	fig|6666666.59347.peg.4069	fig|6666666.59346.peg.4968	fig|6666666.59348.peg.814	None	None	None	fig|6666666.59353.peg.331	fig|6666666.59355.peg.1370	None

6117	General secretion pathway protein I	fig|6666666.59329.peg.1225	fig|6666666.59330.peg.422	fig|6666666.59331.peg.2900	fig|6666666.59332.peg.2994	fig|6666666.59334.peg.123	fig|6666666.59335.peg.713	fig|6666666.59336.peg.3117	fig|6666666.59333.peg.510	fig|6666666.59338.peg.1434	fig|6666666.59339.peg.6206	None	fig|6666666.59337.peg.4248	fig|6666666.59342.peg.3767	None	None	fig|6666666.59345.peg.2672	fig|6666666.59341.peg.4541	fig|6666666.59347.peg.4468	fig|6666666.59346.peg.2225	fig|6666666.59348.peg.4327	fig|6666666.59349.peg.2170	None	fig|6666666.59351.peg.4342	None	None	fig|6666666.59354.peg.702

7282	Heavy metal RND efflux outer membrane protein, CzcC family	fig|6666666.59329.peg.5238	fig|6666666.59330.peg.4688	fig|6666666.59331.peg.3676	fig|6666666.59332.peg.5524	None	fig|6666666.59335.peg.1407	fig|6666666.59336.peg.3445	fig|6666666.59333.peg.3853	fig|6666666.59338.peg.4547	fig|6666666.59339.peg.6667	None	fig|6666666.59337.peg.5735	fig|6666666.59342.peg.3809	fig|6666666.71181.peg.5881	None	fig|6666666.59345.peg.4003	fig|6666666.59341.peg.1493	fig|6666666.59347.peg.3763	fig|6666666.59346.peg.4075	fig|6666666.59348.peg.4577	fig|6666666.59349.peg.2569	None	fig|6666666.59351.peg.3519	None	None	fig|6666666.59354.peg.1886

9547	Starvation sensing protein RspA	fig|6666666.59329.peg.4465	None	fig|6666666.59331.peg.1269	fig|6666666.59332.peg.3027	fig|6666666.59334.peg.3896	None	fig|6666666.59336.peg.3509	None	fig|6666666.59338.peg.2441	fig|6666666.59339.peg.4563	None	fig|6666666.59337.peg.2292	fig|6666666.59342.peg.1752	fig|6666666.71181.peg.4285	fig|6666666.59344.peg.4186	None	fig|6666666.59341.peg.4325	fig|6666666.59347.peg.3834	fig|6666666.59346.peg.1869	None	fig|6666666.59349.peg.4622	fig|6666666.59350.peg.521	fig|6666666.59351.peg.3110	fig|6666666.59353.peg.4774	fig|6666666.59355.peg.5383	fig|6666666.59354.peg.5693

943	putative outer membrane lipoprotein	fig|6666666.59329.peg.1401	fig|6666666.59330.peg.4473	fig|6666666.59331.peg.5273	fig|6666666.59332.peg.3086	fig|6666666.59334.peg.3530	fig|6666666.59335.peg.358	fig|6666666.59336.peg.2160	fig|6666666.59333.peg.3553	fig|6666666.59338.peg.3391	fig|6666666.59339.peg.7136	None	fig|6666666.59337.peg.5642	fig|6666666.59342.peg.2564	fig|6666666.71181.peg.1679	fig|6666666.59344.peg.613	fig|6666666.59345.peg.2146	fig|6666666.59341.peg.1685	None	fig|6666666.59346.peg.3541	fig|6666666.59348.peg.2633	None	None	None	fig|6666666.59353.peg.1049	fig|6666666.59355.peg.3570	None

6326	Uncharacterized protein conserved in bacteria	fig|6666666.59329.peg.5680	fig|6666666.59330.peg.1499	fig|6666666.59331.peg.3729	fig|6666666.59332.peg.2478	None	fig|6666666.59335.peg.5138	fig|6666666.59336.peg.1160	fig|6666666.59333.peg.1740	fig|6666666.59338.peg.3345	fig|6666666.59339.peg.4176	None	fig|6666666.59337.peg.179	fig|6666666.59342.peg.3128	fig|6666666.71181.peg.2185	fig|6666666.59344.peg.1146	fig|6666666.59345.peg.4508	fig|6666666.59341.peg.2143	fig|6666666.59347.peg.603	fig|6666666.59346.peg.2964	fig|6666666.59348.peg.3274	None	None	None	fig|6666666.59353.peg.817	fig|6666666.59355.peg.889	None

6560	FIG00919961 hypothetical protein	fig|6666666.59329.peg.1985	fig|6666666.59330.peg.5021	fig|6666666.59331.peg.1143	fig|6666666.59332.peg.1612	None	fig|6666666.59335.peg.4840	fig|6666666.59336.peg.3416	fig|6666666.59333.peg.5020	fig|6666666.59338.peg.1978	fig|6666666.59339.peg.3795	None	fig|6666666.59337.peg.2395	None	None	fig|6666666.59344.peg.3935	fig|6666666.59345.peg.966	None	fig|6666666.59347.peg.3462	fig|6666666.59346.peg.4847	fig|6666666.59348.peg.2254	fig|6666666.59349.peg.4025	None	fig|6666666.59351.peg.1908	fig|6666666.59353.peg.4410	fig|6666666.59355.peg.3043	fig|6666666.59354.peg.1012

7139	Putative inner membrane protein	fig|6666666.59329.peg.1855	fig|6666666.59330.peg.4453	None	fig|6666666.59332.peg.1877	fig|6666666.59334.peg.161	fig|6666666.59335.peg.333	fig|6666666.59336.peg.611	fig|6666666.59333.peg.878	fig|6666666.59338.peg.321	fig|6666666.59339.peg.8325	fig|6666666.59340.peg.426	fig|6666666.59337.peg.1722	fig|6666666.59342.peg.2484	fig|6666666.71181.peg.1996	fig|6666666.59344.peg.2746	fig|6666666.59345.peg.5362	fig|6666666.59341.peg.1433	fig|6666666.59347.peg.4910	fig|6666666.59346.peg.3206	None	None	None	None	fig|6666666.59353.peg.2993	fig|6666666.59355.peg.2899	None

4661	Hypothetical Transcriptional Regulator	fig|6666666.59329.peg.57	fig|6666666.59330.peg.636	fig|6666666.59331.peg.2791	fig|6666666.59332.peg.3280	fig|6666666.59334.peg.1392	fig|6666666.59335.peg.2440	fig|6666666.59336.peg.6423	fig|6666666.59333.peg.3580	fig|6666666.59338.peg.578	fig|6666666.59339.peg.8083	None	fig|6666666.59337.peg.3895	fig|6666666.59342.peg.4031	None	None	fig|6666666.59345.peg.4934	fig|6666666.59341.peg.1946	fig|6666666.59347.peg.4753	fig|6666666.59346.peg.3550	fig|6666666.59348.peg.56	fig|6666666.59349.peg.3928	None	fig|6666666.59351.peg.114	None	None	fig|6666666.59354.peg.4360

9898	Putative cytoplasmic protein USSDB7A	fig|6666666.59329.peg.5786	fig|6666666.59330.peg.3119	fig|6666666.59331.peg.4416	fig|6666666.59332.peg.1093	None	fig|6666666.59335.peg.3351	fig|6666666.59336.peg.5368	fig|6666666.59333.peg.1946	fig|6666666.59338.peg.1334	fig|6666666.59339.peg.4004	None	fig|6666666.59337.peg.5226	fig|6666666.59342.peg.2905	fig|6666666.71181.peg.4823	fig|6666666.59344.peg.2983	fig|6666666.59345.peg.3454	fig|6666666.59341.peg.5191	fig|6666666.59347.peg.2411	None	None	fig|6666666.59349.peg.1631	None	None	fig|6666666.59353.peg.3567	fig|6666666.59355.peg.3606	fig|6666666.59354.peg.400

2798	Cytochrome O ubiquinol oxidase subunit IV EC 1.10.3.-	fig|6666666.59329.peg.5243	fig|6666666.59330.peg.901	None	fig|6666666.59332.peg.4822	fig|6666666.59334.peg.2933	fig|6666666.59335.peg.4310	fig|6666666.59336.peg.5094	fig|6666666.59333.peg.3095	fig|6666666.59338.peg.1538	fig|6666666.59339.peg.1602	None	fig|6666666.59337.peg.1476	fig|6666666.59342.peg.1310	fig|6666666.71181.peg.2042	fig|6666666.59344.peg.1079	fig|6666666.59345.peg.368	fig|6666666.59341.peg.4106	fig|6666666.59347.peg.4680	None	None	fig|6666666.59349.peg.634	None	None	fig|6666666.59353.peg.763	fig|6666666.59355.peg.5585	fig|6666666.59354.peg.2415

3256	FIG01200265 hypothetical protein	fig|6666666.59329.peg.4435	fig|6666666.59330.peg.1244	fig|6666666.59331.peg.5536	fig|6666666.59332.peg.2407	fig|6666666.59334.peg.3657	fig|6666666.59335.peg.1319	fig|6666666.59336.peg.1626	fig|6666666.59333.peg.1881	fig|6666666.59338.peg.3640	fig|6666666.59339.peg.3454	fig|6666666.59340.peg.693	fig|6666666.59337.peg.2187	None	None	None	fig|6666666.59345.peg.1600	fig|6666666.59341.peg.257	fig|6666666.59347.peg.4095	fig|6666666.59346.peg.129	fig|6666666.59348.peg.5094	fig|6666666.59349.peg.1802	None	fig|6666666.59351.peg.986	None	None	fig|6666666.59354.peg.5337

4013	FIG01202327 hypothetical protein	fig|6666666.59329.peg.6378	fig|6666666.59330.peg.3604	fig|6666666.59331.peg.113	fig|6666666.59332.peg.4773	fig|6666666.59334.peg.4608	fig|6666666.59335.peg.5497	fig|6666666.59336.peg.1148	fig|6666666.59333.peg.5443	None	fig|6666666.59339.peg.2315	None	fig|6666666.59337.peg.3000	fig|6666666.59342.peg.1335	fig|6666666.71181.peg.1895	fig|6666666.59344.peg.2549	fig|6666666.59345.peg.637	fig|6666666.59341.peg.3317	fig|6666666.59347.peg.766	fig|6666666.59346.peg.4679	fig|6666666.59348.peg.2593	None	None	None	fig|6666666.59353.peg.1516	fig|6666666.59355.peg.2066	None

3988	Type IV pilus biogenesis protein PilN	fig|6666666.59329.peg.309	fig|6666666.59330.peg.1694	fig|6666666.59331.peg.890	fig|6666666.59332.peg.1026	None	fig|6666666.59335.peg.2489	fig|6666666.59336.peg.4163	fig|6666666.59333.peg.4148	fig|6666666.59338.peg.957	fig|6666666.59339.peg.8723	fig|6666666.59340.peg.2525	fig|6666666.59337.peg.1697	fig|6666666.59342.peg.6157	None	None	fig|6666666.59345.peg.5385	fig|6666666.59341.peg.963	fig|6666666.59347.peg.4939	fig|6666666.59346.peg.2	fig|6666666.59348.peg.1916	fig|6666666.59349.peg.523	None	fig|6666666.59351.peg.3846	None	None	fig|6666666.59354.peg.2401

9314	Universal stress protein B	fig|6666666.59329.peg.4663	fig|6666666.59330.peg.238	None	fig|6666666.59332.peg.1722	fig|6666666.59334.peg.1195	fig|6666666.59335.peg.28	fig|6666666.59336.peg.5076	fig|6666666.59333.peg.673	fig|6666666.59338.peg.78	fig|6666666.59339.peg.7553	None	fig|6666666.59337.peg.953	fig|6666666.59342.peg.5888	fig|6666666.71181.peg.1067	fig|6666666.59344.peg.4970	fig|6666666.59345.peg.2722	fig|6666666.59341.peg.4153	fig|6666666.59347.peg.4574	fig|6666666.59346.peg.3447	fig|6666666.59348.peg.2086	None	None	None	fig|6666666.59353.peg.4508	fig|6666666.59355.peg.4618	None

49	biphenyl-2,3-diol 1,2-dioxygenase III-related protein	fig|6666666.59329.peg.1495	fig|6666666.59330.peg.4505	fig|6666666.59331.peg.2425	fig|6666666.59332.peg.1410	fig|6666666.59334.peg.2949	fig|6666666.59335.peg.656	fig|6666666.59336.peg.750	fig|6666666.59333.peg.5129	None	fig|6666666.59339.peg.5347	None	fig|6666666.59337.peg.5818	fig|6666666.59342.peg.2194	fig|6666666.71181.peg.1463	fig|6666666.59344.peg.5744	fig|6666666.59345.peg.4641	fig|6666666.59341.peg.1286	None	fig|6666666.59346.peg.1498	fig|6666666.59348.peg.4931	None	fig|6666666.59350.peg.5997	None	fig|6666666.59353.peg.4378	fig|6666666.59355.peg.4463	None

6153	Chitobiose phosphorylase EC 2.4.1.-	fig|6666666.59329.peg.852	fig|6666666.59330.peg.1773	None	fig|6666666.59332.peg.240	fig|6666666.59334.peg.3726	fig|6666666.59335.peg.1532	fig|6666666.59336.peg.6148	fig|6666666.59333.peg.333	fig|6666666.59338.peg.5392	fig|6666666.59339.peg.4803	fig|6666666.59340.peg.3555	fig|6666666.59337.peg.644	None	fig|6666666.71181.peg.519	fig|6666666.59344.peg.1958	fig|6666666.59345.peg.2389	fig|6666666.59341.peg.1805	fig|6666666.59347.peg.3151	fig|6666666.59346.peg.759	fig|6666666.59348.peg.660	None	None	None	fig|6666666.59353.peg.1148	fig|6666666.59355.peg.329	None

4038	FIG01200153 hypothetical protein	fig|6666666.59329.peg.2889	None	fig|6666666.59331.peg.5321	fig|6666666.59332.peg.2361	fig|6666666.59334.peg.807	fig|6666666.59335.peg.4194	fig|6666666.59336.peg.4353	fig|6666666.59333.peg.2624	fig|6666666.59338.peg.2159	fig|6666666.59339.peg.3190	fig|6666666.59340.peg.3229	fig|6666666.59337.peg.2675	fig|6666666.59342.peg.5487	None	None	fig|6666666.59345.peg.1051	fig|6666666.59341.peg.29	fig|6666666.59347.peg.4153	fig|6666666.59346.peg.118	fig|6666666.59348.peg.2019	fig|6666666.59349.peg.2565	None	fig|6666666.59351.peg.5527	None	None	fig|6666666.59354.peg.1882

2453	Molybdenum cofactor biosynthesis protein MoaC	fig|6666666.59329.peg.5052	fig|6666666.59330.peg.1712	None	fig|6666666.59332.peg.1523	None	fig|6666666.59335.peg.5487	fig|6666666.59336.peg.2033	fig|6666666.59333.peg.1330	fig|6666666.59338.peg.2309	fig|6666666.59339.peg.141	None	fig|6666666.59337.peg.4026	fig|6666666.59342.peg.874	fig|6666666.71181.peg.2533	fig|6666666.59344.peg.4102	fig|6666666.59345.peg.1111	fig|6666666.59341.peg.2165	fig|6666666.59347.peg.5155	fig|6666666.59346.peg.534	fig|6666666.59348.peg.718	None	fig|6666666.59350.peg.2779	None	fig|6666666.59353.peg.2143	fig|6666666.59355.peg.2676	None

3825	hypothetical protein	None	fig|6666666.59330.peg.2378	fig|6666666.59331.peg.2960	fig|6666666.59332.peg.2035	None	fig|6666666.59335.peg.5223	fig|6666666.59336.peg.2122	fig|6666666.59333.peg.831	fig|6666666.59338.peg.4421	fig|6666666.59339.peg.3882	None	fig|6666666.59337.peg.2867	fig|6666666.59342.peg.6085	fig|6666666.71181.peg.3828	fig|6666666.59344.peg.855	fig|6666666.59345.peg.1718	fig|6666666.59341.peg.5699	fig|6666666.59347.peg.1857	fig|6666666.59346.peg.1166	fig|6666666.59348.peg.3725	None	fig|6666666.59350.peg.2561	None	fig|6666666.59353.peg.1261	fig|6666666.59355.peg.3323	None

7333	Putative surface protein	fig|6666666.59329.peg.5795	fig|6666666.59330.peg.2001	None	fig|6666666.59332.peg.793	fig|6666666.59334.peg.3559	fig|6666666.59335.peg.2550	fig|6666666.59336.peg.1223	fig|6666666.59333.peg.1615	fig|6666666.59338.peg.2380	fig|6666666.59339.peg.3521	None	fig|6666666.59337.peg.4641	fig|6666666.59342.peg.195	fig|6666666.71181.peg.4136	fig|6666666.59344.peg.392	fig|6666666.59345.peg.4579	fig|6666666.59341.peg.357	fig|6666666.59347.peg.1977	fig|6666666.59346.peg.4116	fig|6666666.59348.peg.3884	None	None	None	fig|6666666.59353.peg.1612	fig|6666666.59355.peg.1109	None

6694	Aquaporin Z	fig|6666666.59329.peg.2593	fig|6666666.59330.peg.4124	fig|6666666.59331.peg.1942	fig|6666666.59332.peg.4657	fig|6666666.59334.peg.2750	fig|6666666.59335.peg.6083	fig|6666666.59336.peg.1854	fig|6666666.59333.peg.3440	fig|6666666.59338.peg.2300	None	None	fig|6666666.59337.peg.3740	fig|6666666.59342.peg.5072	fig|6666666.71181.peg.2199	fig|6666666.59344.peg.994	fig|6666666.59345.peg.1190	fig|6666666.59341.peg.2462	fig|6666666.59347.peg.2443	fig|6666666.59346.peg.4876	None	None	fig|6666666.59350.peg.6505	None	fig|6666666.59353.peg.832	fig|6666666.59355.peg.872	None

7839	Gfa-like protein	fig|6666666.59329.peg.2367	fig|6666666.59330.peg.2858	fig|6666666.59331.peg.1095	fig|6666666.59332.peg.510	fig|6666666.59334.peg.4380	fig|6666666.59335.peg.4400	fig|6666666.59336.peg.905	fig|6666666.59333.peg.1731	None	fig|6666666.59339.peg.8479	None	fig|6666666.59337.peg.3198	fig|6666666.59342.peg.5347	None	None	fig|6666666.59345.peg.2904	fig|6666666.59341.peg.2910	fig|6666666.59347.peg.5414	fig|6666666.59346.peg.4231	fig|6666666.59348.peg.3794	fig|6666666.59349.peg.683	fig|6666666.59350.peg.5271	fig|6666666.59351.peg.4081	None	None	fig|6666666.59354.peg.5209

1236	Putative exported protein	fig|6666666.59329.peg.348	fig|6666666.59330.peg.411	fig|6666666.59331.peg.3263	fig|6666666.59332.peg.2109	fig|6666666.59334.peg.3230	fig|6666666.59335.peg.726	fig|6666666.59336.peg.444	fig|6666666.59333.peg.497	None	fig|6666666.59339.peg.6185	None	fig|6666666.59337.peg.996	fig|6666666.59342.peg.6183	fig|6666666.71181.peg.2599	fig|6666666.59344.peg.914	fig|6666666.59345.peg.2683	fig|6666666.59341.peg.5319	fig|6666666.59347.peg.4458	fig|6666666.59346.peg.2601	fig|6666666.59348.peg.4497	None	None	None	fig|6666666.59353.peg.2615	fig|6666666.59355.peg.2588	None

4172	C4-dicarboxylate transporter DcuA	fig|6666666.59329.peg.2847	fig|6666666.59330.peg.4802	fig|6666666.59331.peg.3121	fig|6666666.59332.peg.152	None	fig|6666666.59335.peg.471	fig|6666666.59336.peg.6556	fig|6666666.59333.peg.3320	fig|6666666.59338.peg.4105	fig|6666666.59339.peg.6489	None	fig|6666666.59337.peg.4346	fig|6666666.59342.peg.3031	fig|6666666.71181.peg.1674	fig|6666666.59344.peg.620	fig|6666666.59345.peg.5128	fig|6666666.59341.peg.1605	fig|6666666.59347.peg.4510	fig|6666666.59346.peg.784	fig|6666666.59348.peg.1862	None	None	None	fig|6666666.59353.peg.1044	fig|6666666.59355.peg.3575	None

2222	2-succinyl-6-hydroxy-2,4-cyclohexadiene-1-carboxylate synthase EC 4.2.99.20	fig|6666666.59329.peg.4194	fig|6666666.59330.peg.312	fig|6666666.59331.peg.764	fig|6666666.59332.peg.5076	fig|6666666.59334.peg.1200	fig|6666666.59335.peg.861	fig|6666666.59336.peg.2474	fig|6666666.59333.peg.1430	fig|6666666.59338.peg.3204	fig|6666666.59339.peg.3232	None	fig|6666666.59337.peg.1039	fig|6666666.59342.peg.3962	None	fig|6666666.59344.peg.1738	fig|6666666.59345.peg.4243	fig|6666666.59341.peg.2666	fig|6666666.59347.peg.2645	fig|6666666.59346.peg.2063	fig|6666666.59348.peg.3371	None	None	None	fig|6666666.59353.peg.1144	fig|6666666.59355.peg.332	None

4346	4'-phosphopantetheinyl transferase entD EC 2.7.8.-	fig|6666666.59329.peg.432	fig|6666666.59330.peg.273	None	fig|6666666.59332.peg.1135	None	fig|6666666.59335.peg.3828	fig|6666666.59336.peg.5485	fig|6666666.59333.peg.4385	fig|6666666.59338.peg.1205	fig|6666666.59339.peg.1041	None	fig|6666666.59337.peg.1001	None	fig|6666666.71181.peg.1732	fig|6666666.59344.peg.6142	fig|6666666.59345.peg.4275	None	fig|6666666.59347.peg.280	None	fig|6666666.59348.peg.5484	fig|6666666.59349.peg.697	fig|6666666.59350.peg.4555	fig|6666666.59351.peg.42	fig|6666666.59353.peg.3681	fig|6666666.59355.peg.4945	fig|6666666.59354.peg.3441

7813	Hydroxypyruvate isomerase EC 5.3.1.22	None	fig|6666666.59330.peg.4024	fig|6666666.59331.peg.4497	fig|6666666.59332.peg.6100	fig|6666666.59334.peg.2709	fig|6666666.59335.peg.2787	None	fig|6666666.59333.peg.3732	fig|6666666.59338.peg.5864	fig|6666666.59339.peg.5381	None	fig|6666666.59337.peg.2937	fig|6666666.59342.peg.3363	fig|6666666.71181.peg.3100	fig|6666666.59344.peg.6044	fig|6666666.59345.peg.2073	fig|6666666.59341.peg.3175	fig|6666666.59347.peg.3335	fig|6666666.59346.peg.2106	fig|6666666.59348.peg.1614	None	fig|6666666.59350.peg.342	None	fig|6666666.59353.peg.693	fig|6666666.59355.peg.179	None

1827	NAD P transhydrogenase subunit beta EC 1.6.1.2	fig|6666666.59329.peg.4234	fig|6666666.59330.peg.797	fig|6666666.59331.peg.5550	None	fig|6666666.59334.peg.1011	fig|6666666.59335.peg.557	fig|6666666.59336.peg.5342	fig|6666666.59333.peg.1532	fig|6666666.59338.peg.1747	fig|6666666.59339.peg.2947	None	fig|6666666.59337.peg.3824	fig|6666666.59342.peg.1098	fig|6666666.71181.peg.4349	fig|6666666.59344.peg.590	fig|6666666.59345.peg.72	fig|6666666.59341.peg.2223	fig|6666666.59347.peg.927	fig|6666666.59346.peg.1898	None	None	fig|6666666.59350.peg.4664	None	fig|6666666.59353.peg.1887	fig|6666666.59355.peg.1127	None

5587	Type VI secretion lipoprotein VasD	fig|6666666.59329.peg.3364	fig|6666666.59330.peg.4162	None	fig|6666666.59332.peg.3511	None	fig|6666666.59335.peg.1359	fig|6666666.59336.peg.3640	fig|6666666.59333.peg.3047	fig|6666666.59338.peg.4067	fig|6666666.59339.peg.3983	None	fig|6666666.59337.peg.4727	fig|6666666.59342.peg.2556	fig|6666666.71181.peg.2261	fig|6666666.59344.peg.2997	fig|6666666.59345.peg.4117	fig|6666666.59341.peg.4558	fig|6666666.59347.peg.1021	None	fig|6666666.59348.peg.2403	fig|6666666.59349.peg.1641	None	None	fig|6666666.59353.peg.3558	fig|6666666.59355.peg.3617	fig|6666666.59354.peg.740

8995	Na+ H+ antiporter NhaB	fig|6666666.59329.peg.1001	fig|6666666.59330.peg.3950	fig|6666666.59331.peg.6269	fig|6666666.59332.peg.4455	None	fig|6666666.59335.peg.1301	fig|6666666.59336.peg.4024	fig|6666666.59333.peg.1538	fig|6666666.59338.peg.5196	fig|6666666.59339.peg.3430	fig|6666666.59340.peg.2255	fig|6666666.59337.peg.3843	fig|6666666.59342.peg.1966	fig|6666666.71181.peg.2948	fig|6666666.59344.peg.2868	fig|6666666.59345.peg.1084	fig|6666666.59341.peg.2386	fig|6666666.59347.peg.2624	fig|6666666.59346.peg.556	None	None	None	None	fig|6666666.59353.peg.1456	fig|6666666.59355.peg.2006	None

6651	Putative potassium channel protein	fig|6666666.59329.peg.1626	fig|6666666.59330.peg.3430	fig|6666666.59331.peg.5121	fig|6666666.59332.peg.4697	fig|6666666.59334.peg.2939	fig|6666666.59335.peg.2516	fig|6666666.59336.peg.5028	fig|6666666.59333.peg.1996	fig|6666666.59338.peg.1477	fig|6666666.59339.peg.407	fig|6666666.59340.peg.3488	fig|6666666.59337.peg.4375	None	None	None	fig|6666666.59345.peg.1501	fig|6666666.59341.peg.4520	fig|6666666.59347.peg.4213	fig|6666666.59346.peg.1436	fig|6666666.59348.peg.2751	fig|6666666.59349.peg.2272	None	fig|6666666.59351.peg.4826	None	None	fig|6666666.59354.peg.1843

11228	Predicted nucleoside-diphosphate-sugar epimerases	fig|6666666.59329.peg.6021	fig|6666666.59330.peg.2666	None	fig|6666666.59332.peg.3377	fig|6666666.59334.peg.572	fig|6666666.59335.peg.2164	fig|6666666.59336.peg.1696	fig|6666666.59333.peg.1787	fig|6666666.59338.peg.3171	fig|6666666.59339.peg.3902	None	fig|6666666.59337.peg.4534	fig|6666666.59342.peg.2636	fig|6666666.71181.peg.3530	fig|6666666.59344.peg.3284	fig|6666666.59345.peg.4670	fig|6666666.59341.peg.552	fig|6666666.59347.peg.3358	fig|6666666.59346.peg.2031	fig|6666666.59348.peg.3822	None	None	None	fig|6666666.59353.peg.3108	fig|6666666.59355.peg.3709	None

6048	Chemotaxis response regulator protein-glutamate methylesterase CheB EC 3.1.1.61	None	fig|6666666.59330.peg.4878	None	None	None	fig|6666666.59335.peg.1920	fig|6666666.59336.peg.6367	fig|6666666.59333.peg.1690	None	fig|6666666.59339.peg.3017	None	fig|6666666.59337.peg.2981	fig|6666666.59342.peg.5329	fig|6666666.71181.peg.2244	fig|6666666.59344.peg.3019	fig|6666666.59345.peg.5322	fig|6666666.59341.peg.5586	fig|6666666.59347.peg.2670	fig|6666666.59346.peg.4524	fig|6666666.59348.peg.5038	fig|6666666.59349.peg.190	fig|6666666.59350.peg.7719	fig|6666666.59351.peg.2069	fig|6666666.59353.peg.3541	fig|6666666.59355.peg.3634	fig|6666666.59354.peg.2943

771	FIG138315 Putative alpha helix protein	None	fig|6666666.59330.peg.37	None	fig|6666666.59332.peg.2336	fig|6666666.59334.peg.110	fig|6666666.59335.peg.190	fig|6666666.59336.peg.341	fig|6666666.59333.peg.253	fig|6666666.59338.peg.313	None	None	fig|6666666.59337.peg.822	None	fig|6666666.71181.peg.994	fig|6666666.59344.peg.1766	fig|6666666.59345.peg.4868	fig|6666666.59341.peg.1235	fig|6666666.59347.peg.4878	fig|6666666.59346.peg.648	fig|6666666.59348.peg.4097	fig|6666666.59349.peg.13	None	fig|6666666.59351.peg.3430	fig|6666666.59353.peg.2310	fig|6666666.59355.peg.1585	fig|6666666.59354.peg.4209

5362	Chromosome plasmid partitioning protein ParB	fig|6666666.59329.peg.663	fig|6666666.59330.peg.1895	fig|6666666.59331.peg.1891	fig|6666666.59332.peg.1130	fig|6666666.59334.peg.1714	fig|6666666.59335.peg.4684	fig|6666666.59336.peg.788	None	fig|6666666.59338.peg.5651	fig|6666666.59339.peg.5457	fig|6666666.59340.peg.1050	fig|6666666.59337.peg.244	fig|6666666.59342.peg.6404	None	None	fig|6666666.59345.peg.5256	fig|6666666.59341.peg.1387	fig|6666666.59347.peg.15	fig|6666666.59346.peg.5241	fig|6666666.59348.peg.3516	fig|6666666.59349.peg.1508	None	fig|6666666.59351.peg.3007	None	None	fig|6666666.59354.peg.4143

2322	Cytochrome c oxidase subunit CcoQ EC 1.9.3.1	fig|6666666.59329.peg.1339	fig|6666666.59330.peg.3190	fig|6666666.59331.peg.1451	fig|6666666.59332.peg.5537	fig|6666666.59334.peg.1460	fig|6666666.59335.peg.3538	fig|6666666.59336.peg.6480	fig|6666666.59333.peg.4304	fig|6666666.59338.peg.4744	fig|6666666.59339.peg.1325	None	fig|6666666.59337.peg.3726	fig|6666666.59342.peg.589	None	None	fig|6666666.59345.peg.3235	fig|6666666.59341.peg.5717	fig|6666666.59347.peg.1743	fig|6666666.59346.peg.1534	fig|6666666.59348.peg.4792	fig|6666666.59349.peg.137	None	fig|6666666.59351.peg.834	None	None	fig|6666666.59354.peg.2590

6029	Guanylate cyclase-related protein	None	fig|6666666.59330.peg.2078	fig|6666666.59331.peg.4648	fig|6666666.59332.peg.5888	fig|6666666.59334.peg.2245	fig|6666666.59335.peg.5689	fig|6666666.59336.peg.6474	fig|6666666.59333.peg.3214	fig|6666666.59338.peg.4692	fig|6666666.59339.peg.3709	fig|6666666.59340.peg.3761	fig|6666666.59337.peg.4676	fig|6666666.59342.peg.732	None	None	fig|6666666.59345.peg.828	fig|6666666.59341.peg.2105	fig|6666666.59347.peg.2147	fig|6666666.59346.peg.3842	fig|6666666.59348.peg.1440	fig|6666666.59349.peg.2146	None	fig|6666666.59351.peg.1662	None	None	fig|6666666.59354.peg.1878

10840	RND efflux system, outer membrane lipoprotein CmeC	fig|6666666.59329.peg.3922	None	fig|6666666.59331.peg.1861	fig|6666666.59332.peg.1230	fig|6666666.59334.peg.2278	fig|6666666.59335.peg.4953	fig|6666666.59336.peg.2519	fig|6666666.59333.peg.3353	None	fig|6666666.59339.peg.743	fig|6666666.59340.peg.985	fig|6666666.59337.peg.3608	fig|6666666.59342.peg.6408	fig|6666666.71181.peg.5804	fig|6666666.59344.peg.2377	fig|6666666.59345.peg.1695	fig|6666666.59341.peg.2484	fig|6666666.59347.peg.2116	fig|6666666.59346.peg.4907	None	None	fig|6666666.59350.peg.6690	None	fig|6666666.59353.peg.265	fig|6666666.59355.peg.3186	None

2344	Glycine cleavage system regulatory protein	fig|6666666.59329.peg.4747	fig|6666666.59330.peg.4910	fig|6666666.59331.peg.1433	fig|6666666.59332.peg.6035	fig|6666666.59334.peg.4483	fig|6666666.59335.peg.1326	None	fig|6666666.59333.peg.3805	fig|6666666.59338.peg.4870	fig|6666666.59339.peg.934	fig|6666666.59340.peg.3041	fig|6666666.59337.peg.1748	fig|6666666.59342.peg.3715	None	None	fig|6666666.59345.peg.897	fig|6666666.59341.peg.4468	fig|6666666.59347.peg.2223	fig|6666666.59346.peg.1353	fig|6666666.59348.peg.3316	fig|6666666.59349.peg.3547	None	fig|6666666.59351.peg.2319	None	None	fig|6666666.59354.peg.1539

9681	Uncharacterized protein, similar to the N-terminal domain of Lon protease	fig|6666666.59329.peg.4848	fig|6666666.59330.peg.2796	fig|6666666.59331.peg.2168	fig|6666666.59332.peg.4034	fig|6666666.59334.peg.1283	fig|6666666.59335.peg.110	fig|6666666.59336.peg.3464	fig|6666666.59333.peg.1928	fig|6666666.59338.peg.3155	None	None	fig|6666666.59337.peg.2370	fig|6666666.59342.peg.3539	None	None	fig|6666666.59345.peg.2248	fig|6666666.59341.peg.5023	fig|6666666.59347.peg.2811	fig|6666666.59346.peg.2991	fig|6666666.59348.peg.2929	fig|6666666.59349.peg.947	fig|6666666.59350.peg.452	fig|6666666.59351.peg.1103	None	None	fig|6666666.59354.peg.793

8805	NAD-specific glutamate dehydrogenase EC 1.4.1.2 , large form	fig|6666666.59329.peg.5482	fig|6666666.59330.peg.4682	fig|6666666.59331.peg.4490	fig|6666666.59332.peg.4530	fig|6666666.59334.peg.3627	fig|6666666.59335.peg.1233	fig|6666666.59336.peg.3838	fig|6666666.59333.peg.4536	fig|6666666.59338.peg.1775	fig|6666666.59339.peg.1529	None	fig|6666666.59337.peg.5625	fig|6666666.59342.peg.3554	None	None	fig|6666666.59345.peg.3258	fig|6666666.59341.peg.904	fig|6666666.59347.peg.1771	fig|6666666.59346.peg.2049	fig|6666666.59348.peg.4569	fig|6666666.59349.peg.3356	None	fig|6666666.59351.peg.3161	None	None	fig|6666666.59354.peg.712

10636	Lipoprotein-related protein	fig|6666666.59329.peg.466	fig|6666666.59330.peg.1830	None	fig|6666666.59332.peg.3483	fig|6666666.59334.peg.4646	fig|6666666.59335.peg.877	fig|6666666.59336.peg.1950	fig|6666666.59333.peg.4449	fig|6666666.59338.peg.2058	fig|6666666.59339.peg.3265	fig|6666666.59340.peg.1677	fig|6666666.59337.peg.1062	fig|6666666.59342.peg.4451	fig|6666666.71181.peg.2069	fig|6666666.59344.peg.1046	fig|6666666.59345.peg.1655	fig|6666666.59341.peg.4701	fig|6666666.59347.peg.2553	fig|6666666.59346.peg.2085	None	None	None	None	fig|6666666.59353.peg.735	fig|6666666.59355.peg.970	None

6636	putative histidinol phosphatase and related hydrolases of the PHP family	fig|6666666.59329.peg.6359	fig|6666666.59330.peg.2210	None	fig|6666666.59332.peg.4143	fig|6666666.59334.peg.4394	fig|6666666.59335.peg.5888	fig|6666666.59336.peg.4601	fig|6666666.59333.peg.2856	fig|6666666.59338.peg.5186	fig|6666666.59339.peg.2443	None	fig|6666666.59337.peg.1894	fig|6666666.59342.peg.6394	fig|6666666.71181.peg.869	fig|6666666.59344.peg.4	fig|6666666.59345.peg.2841	fig|6666666.59341.peg.4098	fig|6666666.59347.peg.905	fig|6666666.59346.peg.3644	fig|6666666.59348.peg.5156	None	None	None	fig|6666666.59353.peg.345	fig|6666666.59355.peg.1383	None

11178	UDP-2,3-diacylglucosamine hydrolase EC 3.6.1.-	None	fig|6666666.59330.peg.1511	None	fig|6666666.59332.peg.1803	None	fig|6666666.59335.peg.2942	fig|6666666.59336.peg.5019	fig|6666666.59333.peg.5227	fig|6666666.59338.peg.1122	fig|6666666.59339.peg.279	None	None	fig|6666666.59342.peg.2273	fig|6666666.71181.peg.3626	fig|6666666.59344.peg.3387	fig|6666666.59345.peg.1298	fig|6666666.59341.peg.3621	fig|6666666.59347.peg.2319	fig|6666666.59346.peg.4353	fig|6666666.59348.peg.4785	fig|6666666.59349.peg.2075	None	fig|6666666.59351.peg.3578	fig|6666666.59353.peg.3761	fig|6666666.59355.peg.3778	fig|6666666.59354.peg.1407

3762	Zn-ribbon-containing, possibly nucleic-acid-binding protein	fig|6666666.59329.peg.2945	fig|6666666.59330.peg.3891	fig|6666666.59331.peg.5813	fig|6666666.59332.peg.397	fig|6666666.59334.peg.1902	fig|6666666.59335.peg.352	fig|6666666.59336.peg.6452	fig|6666666.59333.peg.3268	fig|6666666.59338.peg.2028	fig|6666666.59339.peg.7144	fig|6666666.59340.peg.3271	fig|6666666.59337.peg.1113	None	None	None	fig|6666666.59345.peg.3516	fig|6666666.59341.peg.2857	fig|6666666.59347.peg.2925	fig|6666666.59346.peg.1723	fig|6666666.59348.peg.3294	fig|6666666.59349.peg.1344	None	fig|6666666.59351.peg.4232	None	None	fig|6666666.59354.peg.2633

4644	Glycerol-3-phosphate ABC transporter, periplasmic glycerol-3-phosphate-binding protein TC 3.A.1.1.3	None	fig|6666666.59330.peg.3245	fig|6666666.59331.peg.2546	fig|6666666.59332.peg.5723	None	fig|6666666.59335.peg.3796	fig|6666666.59336.peg.1660	None	fig|6666666.59338.peg.6021	fig|6666666.59339.peg.5516	fig|6666666.59340.peg.2557	fig|6666666.59337.peg.2155	fig|6666666.59342.peg.4934	fig|6666666.71181.peg.4418	fig|6666666.59344.peg.3434	fig|6666666.59345.peg.3968	fig|6666666.59341.peg.4856	fig|6666666.59347.peg.3525	fig|6666666.59346.peg.4931	fig|6666666.59348.peg.1357	None	fig|6666666.59350.peg.6612	None	fig|6666666.59353.peg.3782	fig|6666666.59355.peg.3377	None

5152	membrane protein	fig|6666666.59329.peg.1694	fig|6666666.59330.peg.3464	fig|6666666.59331.peg.5653	fig|6666666.59332.peg.5173	fig|6666666.59334.peg.3510	fig|6666666.59335.peg.5643	fig|6666666.59336.peg.3748	fig|6666666.59333.peg.5284	None	fig|6666666.59339.peg.8374	fig|6666666.59340.peg.3170	fig|6666666.59337.peg.5422	fig|6666666.59342.peg.6190	None	None	fig|6666666.59345.peg.1918	fig|6666666.59341.peg.4389	fig|6666666.59347.peg.47	fig|6666666.59346.peg.1329	fig|6666666.59348.peg.1350	fig|6666666.59349.peg.1885	None	fig|6666666.59351.peg.404	None	None	fig|6666666.59354.peg.1380

10552	Thioredoxin 2 EC 1.8.1.8	fig|6666666.59329.peg.2269	fig|6666666.59330.peg.760	fig|6666666.59331.peg.255	fig|6666666.59332.peg.5847	fig|6666666.59334.peg.3216	fig|6666666.59335.peg.4994	fig|6666666.59336.peg.4195	fig|6666666.59333.peg.3915	fig|6666666.59338.peg.5134	fig|6666666.59339.peg.4701	None	fig|6666666.59337.peg.1199	fig|6666666.59342.peg.1444	fig|6666666.71181.peg.2646	fig|6666666.59344.peg.3920	fig|6666666.59345.peg.19	fig|6666666.59341.peg.1074	fig|6666666.59347.peg.848	fig|6666666.59346.peg.1203	None	None	None	None	fig|6666666.59353.peg.4758	fig|6666666.59355.peg.4046	None

1745	Polyketide synthase modules and related proteins	fig|6666666.59329.peg.3107	fig|6666666.59330.peg.697	fig|6666666.59331.peg.3350	fig|6666666.59332.peg.135	None	None	fig|6666666.59336.peg.4501	fig|6666666.59333.peg.4320	fig|6666666.59338.peg.3034	None	fig|6666666.59340.peg.793	fig|6666666.59337.peg.4153	None	fig|6666666.71181.peg.852	fig|6666666.59344.peg.24	fig|6666666.59345.peg.4779	fig|6666666.59341.peg.1732	fig|6666666.59347.peg.5392	fig|6666666.59346.peg.1137	None	fig|6666666.59349.peg.2733	None	fig|6666666.59351.peg.2829	fig|6666666.59353.peg.362	fig|6666666.59355.peg.1400	fig|6666666.59354.peg.5122

623	Uncharacterized conserved protein	fig|6666666.59329.peg.4567	fig|6666666.59330.peg.931	fig|6666666.59331.peg.5853	fig|6666666.59332.peg.4791	fig|6666666.59334.peg.4059	fig|6666666.59335.peg.4229	fig|6666666.59336.peg.4178	None	fig|6666666.59338.peg.3824	fig|6666666.59339.peg.6512	None	fig|6666666.59337.peg.3963	fig|6666666.59342.peg.1862	None	None	fig|6666666.59345.peg.460	fig|6666666.59341.peg.3442	fig|6666666.59347.peg.1341	fig|6666666.59346.peg.939	fig|6666666.59348.peg.523	fig|6666666.59349.peg.4870	None	fig|6666666.59351.peg.1714	None	None	fig|6666666.59354.peg.2429

4849	Chromosome segregation ATPase	fig|6666666.59329.peg.4766	fig|6666666.59330.peg.472	fig|6666666.59331.peg.2852	fig|6666666.59332.peg.985	fig|6666666.59334.peg.1036	fig|6666666.59335.peg.158	fig|6666666.59336.peg.4979	fig|6666666.59333.peg.2237	None	fig|6666666.59339.peg.5100	None	fig|6666666.59337.peg.1577	fig|6666666.59342.peg.6151	fig|6666666.71181.peg.39	fig|6666666.59344.peg.2647	fig|6666666.59345.peg.2205	fig|6666666.59341.peg.1471	fig|6666666.59347.peg.4355	None	fig|6666666.59348.peg.930	None	None	None	fig|6666666.59353.peg.1979	fig|6666666.59355.peg.4086	None

8297	FIG01200110 hypothetical protein	fig|6666666.59329.peg.283	fig|6666666.59330.peg.593	fig|6666666.59331.peg.4383	fig|6666666.59332.peg.1193	None	fig|6666666.59335.peg.3483	fig|6666666.59336.peg.2821	fig|6666666.59333.peg.405	fig|6666666.59338.peg.344	fig|6666666.59339.peg.7708	fig|6666666.59340.peg.1531	fig|6666666.59337.peg.350	None	None	None	fig|6666666.59345.peg.2497	fig|6666666.59341.peg.789	fig|6666666.59347.peg.4858	fig|6666666.59346.peg.1966	fig|6666666.59348.peg.1204	fig|6666666.59349.peg.100	None	fig|6666666.59351.peg.157	None	None	fig|6666666.59354.peg.419

2304	Holliday junction DNA helicase RuvB	fig|6666666.59329.peg.2857	fig|6666666.59330.peg.2490	fig|6666666.59331.peg.5226	fig|6666666.59332.peg.1961	fig|6666666.59334.peg.1525	fig|6666666.59335.peg.2499	None	None	None	fig|6666666.59339.peg.385	None	fig|6666666.59337.peg.4551	None	fig|6666666.71181.peg.1923	None	fig|6666666.59345.peg.1514	fig|6666666.59341.peg.3341	fig|6666666.59347.peg.4200	fig|6666666.59346.peg.2765	None	fig|6666666.59349.peg.3865	fig|6666666.59350.peg.4003	fig|6666666.59351.peg.655	fig|6666666.59353.peg.1486	fig|6666666.59355.peg.2036	fig|6666666.59354.peg.1592

3746	putative DNA-binding protein	fig|6666666.59329.peg.248	fig|6666666.59330.peg.361	None	None	fig|6666666.59334.peg.3255	fig|6666666.59335.peg.2312	fig|6666666.59336.peg.1197	fig|6666666.59333.peg.4189	fig|6666666.59338.peg.2664	fig|6666666.59339.peg.7853	fig|6666666.59340.peg.3420	fig|6666666.59337.peg.542	fig|6666666.59342.peg.1510	None	None	fig|6666666.59345.peg.5030	fig|6666666.59341.peg.5252	fig|6666666.59347.peg.3201	None	fig|6666666.59348.peg.196	fig|6666666.59349.peg.4629	fig|6666666.59350.peg.2534	fig|6666666.59351.peg.3105	None	None	fig|6666666.59354.peg.6200

3743	membrane protein	fig|6666666.59329.peg.4451	None	fig|6666666.59331.peg.675	fig|6666666.59332.peg.969	None	fig|6666666.59335.peg.5783	fig|6666666.59336.peg.2606	fig|6666666.59333.peg.1669	fig|6666666.59338.peg.2660	fig|6666666.59339.peg.5649	None	fig|6666666.59337.peg.4357	fig|6666666.59342.peg.6235	fig|6666666.71181.peg.739	fig|6666666.59344.peg.717	fig|6666666.59345.peg.1905	fig|6666666.59341.peg.1950	fig|6666666.59347.peg.1352	fig|6666666.59346.peg.2581	fig|6666666.59348.peg.2136	None	None	None	fig|6666666.59353.peg.962	fig|6666666.59355.peg.2414	None

10391	FIG01201247 hypothetical protein	fig|6666666.59329.peg.5165	fig|6666666.59330.peg.2055	fig|6666666.59331.peg.5664	fig|6666666.59332.peg.3910	fig|6666666.59334.peg.902	fig|6666666.59335.peg.3132	fig|6666666.59336.peg.740	fig|6666666.59333.peg.4177	fig|6666666.59338.peg.3546	fig|6666666.59339.peg.5430	None	fig|6666666.59337.peg.4325	fig|6666666.59342.peg.4674	None	None	fig|6666666.59345.peg.3920	fig|6666666.59341.peg.3572	fig|6666666.59347.peg.3767	fig|6666666.59346.peg.498	fig|6666666.59348.peg.4262	None	fig|6666666.59350.peg.1886	fig|6666666.59351.peg.4729	None	None	None

9526	FIG01199668 hypothetical protein	fig|6666666.59329.peg.930	fig|6666666.59330.peg.2706	fig|6666666.59331.peg.6109	None	fig|6666666.59334.peg.545	fig|6666666.59335.peg.3246	fig|6666666.59336.peg.4239	fig|6666666.59333.peg.5207	fig|6666666.59338.peg.3666	fig|6666666.59339.peg.2471	None	fig|6666666.59337.peg.2435	fig|6666666.59342.peg.5350	None	None	fig|6666666.59345.peg.614	fig|6666666.59341.peg.1880	fig|6666666.59347.peg.530	fig|6666666.59346.peg.3601	fig|6666666.59348.peg.3144	fig|6666666.59349.peg.3038	None	fig|6666666.59351.peg.5185	None	None	fig|6666666.59354.peg.4826

3580	Chemotaxis protein CheX	fig|6666666.59329.peg.1859	fig|6666666.59330.peg.2650	None	fig|6666666.59332.peg.5929	None	fig|6666666.59335.peg.4597	fig|6666666.59336.peg.606	fig|6666666.59333.peg.882	fig|6666666.59338.peg.316	fig|6666666.59339.peg.8314	fig|6666666.59340.peg.1782	fig|6666666.59337.peg.1717	fig|6666666.59342.peg.3248	None	None	fig|6666666.59345.peg.5366	fig|6666666.59341.peg.1440	fig|6666666.59347.peg.4882	fig|6666666.59346.peg.3203	fig|6666666.59348.peg.535	fig|6666666.59349.peg.3244	None	fig|6666666.59351.peg.5439	None	None	fig|6666666.59354.peg.5101

9778	Protein YbgE	fig|6666666.59329.peg.2850	fig|6666666.59330.peg.3441	fig|6666666.59331.peg.1728	None	None	fig|6666666.59335.peg.2504	fig|6666666.59336.peg.2582	fig|6666666.59333.peg.1984	fig|6666666.59338.peg.4934	fig|6666666.59339.peg.393	None	fig|6666666.59337.peg.4385	fig|6666666.59342.peg.4590	fig|6666666.71181.peg.3246	fig|6666666.59344.peg.4578	fig|6666666.59345.peg.1511	fig|6666666.59341.peg.3346	fig|6666666.59347.peg.4204	fig|6666666.59346.peg.1446	fig|6666666.59348.peg.3510	None	None	None	fig|6666666.59353.peg.2096	fig|6666666.59355.peg.2726	None

815	Malate synthase-related protein	fig|6666666.59329.peg.2175	fig|6666666.59330.peg.3786	fig|6666666.59331.peg.6110	fig|6666666.59332.peg.2730	None	fig|6666666.59335.peg.4781	fig|6666666.59336.peg.1139	fig|6666666.59333.peg.5142	fig|6666666.59338.peg.1067	fig|6666666.59339.peg.4201	None	fig|6666666.59337.peg.2223	fig|6666666.59342.peg.1477	None	None	fig|6666666.59345.peg.2995	fig|6666666.59341.peg.1162	fig|6666666.59347.peg.2075	fig|6666666.59346.peg.889	fig|6666666.59348.peg.4031	fig|6666666.59349.peg.2642	None	fig|6666666.59351.peg.4138	None	None	fig|6666666.59354.peg.4724

3277	Flp pilus assembly protein TadB	fig|6666666.59329.peg.5665	fig|6666666.59330.peg.667	fig|6666666.59331.peg.2292	fig|6666666.59332.peg.691	fig|6666666.59334.peg.1506	fig|6666666.59335.peg.1101	fig|6666666.59336.peg.4290	None	fig|6666666.59338.peg.4888	fig|6666666.59339.peg.1887	None	fig|6666666.59337.peg.1336	fig|6666666.59342.peg.354	fig|6666666.71181.peg.5661	None	fig|6666666.59345.peg.2311	fig|6666666.59341.peg.6053	fig|6666666.59347.peg.1926	fig|6666666.59346.peg.4741	fig|6666666.59348.peg.1165	None	fig|6666666.59350.peg.6478	None	None	fig|6666666.59355.peg.5344	None

9003	Nitrite reductase [NAD P H] small subunit EC 1.7.1.4	fig|6666666.59329.peg.2901	None	None	fig|6666666.59332.peg.6180	fig|6666666.59334.peg.5390	fig|6666666.59335.peg.4989	fig|6666666.59336.peg.4029	fig|6666666.59333.peg.3201	fig|6666666.59338.peg.877	fig|6666666.59339.peg.1001	fig|6666666.59340.peg.1913	fig|6666666.59337.peg.5465	fig|6666666.59342.peg.6332	fig|6666666.71181.peg.1824	None	fig|6666666.59345.peg.281	fig|6666666.59341.peg.5210	fig|6666666.59347.peg.1900	fig|6666666.59346.peg.4995	fig|6666666.59348.peg.5479	None	None	None	fig|6666666.59353.peg.2226	fig|6666666.59355.peg.1920	None

3646	FIG01199864 hypothetical protein	fig|6666666.59329.peg.5620	fig|6666666.59330.peg.2114	fig|6666666.59331.peg.2284	fig|6666666.59332.peg.6266	None	fig|6666666.59335.peg.4731	fig|6666666.59336.peg.2216	fig|6666666.59333.peg.4034	fig|6666666.59338.peg.225	fig|6666666.59339.peg.1941	fig|6666666.59340.peg.3306	fig|6666666.59337.peg.5486	fig|6666666.59342.peg.1469	None	None	fig|6666666.59345.peg.502	fig|6666666.59341.peg.1818	fig|6666666.59347.peg.3589	fig|6666666.59346.peg.3313	fig|6666666.59348.peg.3501	fig|6666666.59349.peg.2814	None	fig|6666666.59351.peg.5050	None	None	None

8076	3-deoxy-D-manno-octulosonic acid kinase EC 2.7.1.-	fig|6666666.59329.peg.559	fig|6666666.59330.peg.1157	None	fig|6666666.59332.peg.284	fig|6666666.59334.peg.178	fig|6666666.59335.peg.487	fig|6666666.59336.peg.66	fig|6666666.59333.peg.643	fig|6666666.59338.peg.1908	fig|6666666.59339.peg.6302	fig|6666666.59340.peg.981	fig|6666666.59337.peg.462	fig|6666666.59342.peg.4336	None	None	fig|6666666.59345.peg.2610	fig|6666666.59341.peg.4931	fig|6666666.59347.peg.5186	fig|6666666.59346.peg.1703	fig|6666666.59348.peg.879	fig|6666666.59349.peg.2788	None	fig|6666666.59351.peg.5203	None	None	None

1306	FIG01200654 hypothetical protein	fig|6666666.59329.peg.4860	fig|6666666.59330.peg.803	fig|6666666.59331.peg.566	fig|6666666.59332.peg.4466	fig|6666666.59334.peg.1006	fig|6666666.59335.peg.565	None	fig|6666666.59333.peg.3897	fig|6666666.59338.peg.4949	fig|6666666.59339.peg.2938	None	fig|6666666.59337.peg.3830	None	fig|6666666.71181.peg.1890	fig|6666666.59344.peg.2555	fig|6666666.59345.peg.77	fig|6666666.59341.peg.5355	fig|6666666.59347.peg.933	fig|6666666.59346.peg.1893	fig|6666666.59348.peg.5216	None	None	None	fig|6666666.59353.peg.1521	fig|6666666.59355.peg.2071	None

2571	L,D-transpeptidase YcbB	None	fig|6666666.59330.peg.2302	None	fig|6666666.59332.peg.2680	fig|6666666.59334.peg.1401	fig|6666666.59335.peg.6023	fig|6666666.59336.peg.5130	fig|6666666.59333.peg.3314	fig|6666666.59338.peg.4098	fig|6666666.59339.peg.3741	None	fig|6666666.59337.peg.1773	fig|6666666.59342.peg.2336	fig|6666666.71181.peg.4178	fig|6666666.59344.peg.2777	fig|6666666.59345.peg.871	None	fig|6666666.59347.peg.2241	fig|6666666.59346.peg.1681	fig|6666666.59348.peg.4889	None	fig|6666666.59350.peg.581	None	fig|6666666.59353.peg.1666	fig|6666666.59355.peg.1055	None

7290	Potential queD like 2	fig|6666666.59329.peg.6473	fig|6666666.59330.peg.3387	fig|6666666.59331.peg.4356	fig|6666666.59332.peg.5682	None	fig|6666666.59335.peg.3123	fig|6666666.59336.peg.3553	fig|6666666.59333.peg.3171	fig|6666666.59338.peg.4236	fig|6666666.59339.peg.175	fig|6666666.59340.peg.1039	fig|6666666.59337.peg.4423	fig|6666666.59342.peg.1042	None	None	fig|6666666.59345.peg.1136	fig|6666666.59341.peg.2715	fig|6666666.59347.peg.2565	fig|6666666.59346.peg.4468	None	fig|6666666.59349.peg.3438	None	fig|6666666.59351.peg.2724	None	None	fig|6666666.59354.peg.6513

5200	Homogentisate 1,2-dioxygenase EC 1.13.11.5	None	fig|6666666.59330.peg.3832	fig|6666666.59331.peg.4884	fig|6666666.59332.peg.4544	None	fig|6666666.59335.peg.1550	fig|6666666.59336.peg.6022	fig|6666666.59333.peg.440	fig|6666666.59338.peg.4987	fig|6666666.59339.peg.3849	fig|6666666.59340.peg.77	fig|6666666.59337.peg.2014	fig|6666666.59342.peg.4681	None	None	fig|6666666.59345.peg.3081	fig|6666666.59341.peg.606	fig|6666666.59347.peg.3733	fig|6666666.59346.peg.4202	None	fig|6666666.59349.peg.3219	None	fig|6666666.59351.peg.3755	None	fig|6666666.59355.peg.5878	fig|6666666.59354.peg.442

11435	FIG008480 hypothetical protein	fig|6666666.59329.peg.8	fig|6666666.59330.peg.1451	fig|6666666.59331.peg.2837	fig|6666666.59332.peg.3181	fig|6666666.59334.peg.722	fig|6666666.59335.peg.1878	fig|6666666.59336.peg.282	fig|6666666.59333.peg.2353	fig|6666666.59338.peg.2644	fig|6666666.59339.peg.8231	None	fig|6666666.59337.peg.1803	None	None	None	fig|6666666.59345.peg.5570	fig|6666666.59341.peg.2764	fig|6666666.59347.peg.4957	fig|6666666.59346.peg.24	fig|6666666.59348.peg.280	fig|6666666.59349.peg.971	None	fig|6666666.59351.peg.1575	None	None	fig|6666666.59354.peg.497

8908	Predicted arginine uptake transporter	fig|6666666.59329.peg.5696	fig|6666666.59330.peg.2860	None	fig|6666666.59332.peg.1918	fig|6666666.59334.peg.4739	fig|6666666.59335.peg.1824	fig|6666666.59336.peg.5006	fig|6666666.59333.peg.4221	fig|6666666.59338.peg.2331	fig|6666666.59339.peg.1986	fig|6666666.59340.peg.1404	fig|6666666.59337.peg.1954	fig|6666666.59342.peg.3217	None	None	fig|6666666.59345.peg.4058	fig|6666666.59341.peg.1215	fig|6666666.59347.peg.3640	fig|6666666.59346.peg.4229	None	fig|6666666.59349.peg.2941	None	fig|6666666.59351.peg.609	None	None	fig|6666666.59354.peg.5968

7129	Protoporphyrinogen IX oxidase, oxygen-independent, HemG EC 1.3.-.-	fig|6666666.59329.peg.5547	fig|6666666.59330.peg.1082	fig|6666666.59331.peg.6090	fig|6666666.59332.peg.64	fig|6666666.59334.peg.3370	fig|6666666.59335.peg.6221	fig|6666666.59336.peg.2812	None	fig|6666666.59338.peg.1349	fig|6666666.59339.peg.7985	None	fig|6666666.59337.peg.854	None	fig|6666666.71181.peg.3323	fig|6666666.59344.peg.3395	fig|6666666.59345.peg.4998	fig|6666666.59341.peg.4038	fig|6666666.59347.peg.4816	fig|6666666.59346.peg.3713	fig|6666666.59348.peg.3711	None	None	None	fig|6666666.59353.peg.3815	fig|6666666.59355.peg.3412	None

1662	FIG01200127 hypothetical protein	fig|6666666.59329.peg.3786	fig|6666666.59330.peg.2750	None	fig|6666666.59332.peg.1310	None	fig|6666666.59335.peg.3566	fig|6666666.59336.peg.3091	fig|6666666.59333.peg.3233	fig|6666666.59338.peg.3421	fig|6666666.59339.peg.8368	fig|6666666.59340.peg.478	fig|6666666.59337.peg.293	None	None	None	fig|6666666.59345.peg.2118	fig|6666666.59341.peg.613	fig|6666666.59347.peg.3718	fig|6666666.59346.peg.3061	fig|6666666.59348.peg.2039	fig|6666666.59349.peg.3518	fig|6666666.59350.peg.789	fig|6666666.59351.peg.4577	None	None	fig|6666666.59354.peg.6323

5415	Protein yecM	fig|6666666.59329.peg.3076	fig|6666666.59330.peg.4513	None	fig|6666666.59332.peg.935	fig|6666666.59334.peg.3430	fig|6666666.59335.peg.4145	fig|6666666.59336.peg.3094	fig|6666666.59333.peg.1339	fig|6666666.59338.peg.2371	fig|6666666.59339.peg.2997	None	fig|6666666.59337.peg.2736	None	fig|6666666.71181.peg.1908	fig|6666666.59344.peg.2534	fig|6666666.59345.peg.4308	fig|6666666.59341.peg.5061	fig|6666666.59347.peg.4403	fig|6666666.59346.peg.1488	fig|6666666.59348.peg.2547	None	None	None	fig|6666666.59353.peg.1503	fig|6666666.59355.peg.2053	None

10933	DNA polymerase III epsilon subunit EC 2.7.7.7	fig|6666666.59329.peg.1173	fig|6666666.59330.peg.189	fig|6666666.59331.peg.3321	fig|6666666.59332.peg.5612	fig|6666666.59334.peg.43	fig|6666666.59335.peg.2621	fig|6666666.59336.peg.377	fig|6666666.59333.peg.4503	fig|6666666.59338.peg.3120	None	fig|6666666.59340.peg.914	fig|6666666.59337.peg.4293	None	None	None	fig|6666666.59345.peg.1378	fig|6666666.59341.peg.2337	fig|6666666.59347.peg.4499	fig|6666666.59346.peg.5087	fig|6666666.59348.peg.1816	fig|6666666.59349.peg.3008	None	fig|6666666.59351.peg.348	None	None	fig|6666666.59354.peg.3596

2465	Zinc carboxypeptidase domain protein	fig|6666666.59329.peg.166	fig|6666666.59330.peg.2559	fig|6666666.59331.peg.6341	fig|6666666.59332.peg.1488	None	fig|6666666.59335.peg.3914	fig|6666666.59336.peg.2143	fig|6666666.59333.peg.2876	fig|6666666.59338.peg.1110	fig|6666666.59339.peg.4507	fig|6666666.59340.peg.6	fig|6666666.59337.peg.2259	fig|6666666.59342.peg.3021	None	None	fig|6666666.59345.peg.2946	fig|6666666.59341.peg.204	fig|6666666.59347.peg.4523	fig|6666666.59346.peg.3501	fig|6666666.59348.peg.904	fig|6666666.59349.peg.2744	None	fig|6666666.59351.peg.765	None	None	None

1641	FIG01200539 hypothetical protein	None	None	fig|6666666.59331.peg.4224	fig|6666666.59332.peg.821	fig|6666666.59334.peg.3125	fig|6666666.59335.peg.2180	fig|6666666.59336.peg.3973	fig|6666666.59333.peg.1774	fig|6666666.59338.peg.6109	fig|6666666.59339.peg.3922	None	fig|6666666.59337.peg.1991	fig|6666666.59342.peg.3507	None	None	fig|6666666.59345.peg.4682	fig|6666666.59341.peg.2723	fig|6666666.59347.peg.3344	fig|6666666.59346.peg.2046	fig|6666666.59348.peg.1909	fig|6666666.59349.peg.2793	fig|6666666.59350.peg.6627	fig|6666666.59351.peg.5198	None	None	fig|6666666.59354.peg.5499

4272	UDP-N-acetylglucosamine 2-epimerase EC 5.1.3.14	None	None	fig|6666666.59331.peg.2091	fig|6666666.59332.peg.4448	fig|6666666.59334.peg.2796	fig|6666666.59335.peg.5686	fig|6666666.59336.peg.1534	None	fig|6666666.59338.peg.4631	fig|6666666.59339.peg.6585	fig|6666666.59340.peg.812	fig|6666666.59337.peg.424	fig|6666666.59342.peg.40	fig|6666666.71181.peg.2449	fig|6666666.59344.peg.2464	None	None	fig|6666666.59347.peg.3120	fig|6666666.59346.peg.4221	None	fig|6666666.59349.peg.271	None	fig|6666666.59351.peg.243	fig|6666666.59353.peg.3648	fig|6666666.59355.peg.4019	fig|6666666.59354.peg.849

4862	Putative molybdenum transport ATP-binding protein modF	fig|6666666.59329.peg.1887	fig|6666666.59330.peg.4892	None	None	fig|6666666.59334.peg.1972	fig|6666666.59335.peg.2185	fig|6666666.59336.peg.3534	fig|6666666.59333.peg.4745	fig|6666666.59338.peg.1283	fig|6666666.59339.peg.3928	fig|6666666.59340.peg.1071	fig|6666666.59337.peg.5746	fig|6666666.59342.peg.2376	fig|6666666.71181.peg.2509	fig|6666666.59344.peg.4070	fig|6666666.59345.peg.406	fig|6666666.59341.peg.66	fig|6666666.59347.peg.4014	None	fig|6666666.59348.peg.1731	None	None	None	fig|6666666.59353.peg.2118	fig|6666666.59355.peg.2702	None

2192	protein of unknown function DUF1244	fig|6666666.59329.peg.457	fig|6666666.59330.peg.329	fig|6666666.59331.peg.835	fig|6666666.59332.peg.1132	fig|6666666.59334.peg.5102	fig|6666666.59335.peg.872	fig|6666666.59336.peg.5622	fig|6666666.59333.peg.4941	fig|6666666.59338.peg.1827	fig|6666666.59339.peg.3256	fig|6666666.59340.peg.1686	fig|6666666.59337.peg.1058	fig|6666666.59342.peg.5805	None	None	fig|6666666.59345.peg.1661	fig|6666666.59341.peg.4945	fig|6666666.59347.peg.5381	fig|6666666.59346.peg.2080	fig|6666666.59348.peg.912	None	fig|6666666.59350.peg.983	None	None	None	None

2076	Predicted signal transduction protein	fig|6666666.59329.peg.5796	fig|6666666.59330.peg.2000	fig|6666666.59331.peg.4702	fig|6666666.59332.peg.794	None	fig|6666666.59335.peg.4053	fig|6666666.59336.peg.1224	fig|6666666.59333.peg.1617	fig|6666666.59338.peg.2379	fig|6666666.59339.peg.3524	None	fig|6666666.59337.peg.4643	fig|6666666.59342.peg.196	None	None	fig|6666666.59345.peg.4581	fig|6666666.59341.peg.359	fig|6666666.59347.peg.1976	fig|6666666.59346.peg.4114	fig|6666666.59348.peg.1875	fig|6666666.59349.peg.3856	None	fig|6666666.59351.peg.4263	None	None	fig|6666666.59354.peg.1956

413	FIG01199937 hypothetical protein	fig|6666666.59329.peg.2575	fig|6666666.59330.peg.724	None	fig|6666666.59332.peg.4724	None	fig|6666666.59335.peg.4733	fig|6666666.59336.peg.3041	fig|6666666.59333.peg.4036	fig|6666666.59338.peg.5548	fig|6666666.59339.peg.1944	fig|6666666.59340.peg.845	fig|6666666.59337.peg.3772	fig|6666666.59342.peg.2034	None	None	fig|6666666.59345.peg.2805	fig|6666666.59341.peg.5808	fig|6666666.59347.peg.5085	fig|6666666.59346.peg.3225	fig|6666666.59348.peg.2566	fig|6666666.59349.peg.4471	None	fig|6666666.59351.peg.1554	None	None	fig|6666666.59354.peg.2904

11361	Hypothetical protein, restriction endonuclease-like VRR-NUC domain	fig|6666666.59329.peg.5000	fig|6666666.59330.peg.3294	fig|6666666.59331.peg.5172	None	fig|6666666.59334.peg.5104	fig|6666666.59335.peg.2672	fig|6666666.59336.peg.3673	fig|6666666.59333.peg.2450	fig|6666666.59338.peg.880	fig|6666666.59339.peg.2673	None	fig|6666666.59337.peg.2106	fig|6666666.59342.peg.6411	None	None	fig|6666666.59345.peg.3343	fig|6666666.59341.peg.761	fig|6666666.59347.peg.995	fig|6666666.59346.peg.2937	fig|6666666.59348.peg.1432	fig|6666666.59349.peg.4791	None	fig|6666666.59351.peg.4132	None	None	fig|6666666.59354.peg.1439

536	Gluconokinase EC 2.7.1.12	fig|6666666.59329.peg.2257	None	fig|6666666.59331.peg.5423	fig|6666666.59332.peg.5794	fig|6666666.59334.peg.4940	fig|6666666.59335.peg.5979	fig|6666666.59336.peg.772	fig|6666666.59333.peg.693	fig|6666666.59338.peg.1602	fig|6666666.59339.peg.4192	None	fig|6666666.59337.peg.2800	None	fig|6666666.71181.peg.5652	fig|6666666.59344.peg.3614	fig|6666666.59345.peg.2736	fig|6666666.59341.peg.4190	fig|6666666.59347.peg.4427	fig|6666666.59346.peg.2826	fig|6666666.59348.peg.4274	None	None	None	fig|6666666.59353.peg.3028	fig|6666666.59355.peg.2935	None

4519	Alkaline serine protease	fig|6666666.59329.peg.3741	fig|6666666.59330.peg.3645	fig|6666666.59331.peg.2782	fig|6666666.59332.peg.327	None	fig|6666666.59335.peg.1979	fig|6666666.59336.peg.4374	fig|6666666.59333.peg.703	None	fig|6666666.59339.peg.7033	None	fig|6666666.59337.peg.3869	fig|6666666.59342.peg.1903	fig|6666666.71181.peg.5333	fig|6666666.59344.peg.6284	fig|6666666.59345.peg.4181	fig|6666666.59341.peg.3214	None	fig|6666666.59346.peg.396	fig|6666666.59348.peg.2778	fig|6666666.59349.peg.1659	None	fig|6666666.59351.peg.2718	None	None	fig|6666666.59354.peg.908

7301	Chromosome partition protein MukE	fig|6666666.59329.peg.6324	None	fig|6666666.59331.peg.605	fig|6666666.59332.peg.5453	fig|6666666.59334.peg.591	fig|6666666.59335.peg.939	fig|6666666.59336.peg.1396	fig|6666666.59333.peg.1058	fig|6666666.59338.peg.4365	fig|6666666.59339.peg.317	None	fig|6666666.59337.peg.4939	fig|6666666.59342.peg.144	fig|6666666.71181.peg.4180	fig|6666666.59344.peg.2781	fig|6666666.59345.peg.1533	fig|6666666.59341.peg.2329	fig|6666666.59347.peg.2369	fig|6666666.59346.peg.2016	None	None	None	None	fig|6666666.59353.peg.1664	fig|6666666.59355.peg.1057	None

1436	Thermostable carboxypeptidase 1 EC 3.4.17.19	fig|6666666.59329.peg.2785	fig|6666666.59330.peg.3747	fig|6666666.59331.peg.488	fig|6666666.59332.peg.5741	fig|6666666.59334.peg.3152	fig|6666666.59335.peg.4906	None	fig|6666666.59333.peg.2380	fig|6666666.59338.peg.3896	fig|6666666.59339.peg.804	None	fig|6666666.59337.peg.1841	None	fig|6666666.71181.peg.1356	fig|6666666.59344.peg.576	fig|6666666.59345.peg.4212	fig|6666666.59341.peg.4013	fig|6666666.59347.peg.1693	fig|6666666.59346.peg.3472	None	None	fig|6666666.59350.peg.8151	None	fig|6666666.59353.peg.1877	fig|6666666.59355.peg.1136	None

1832	TRAP transporter solute receptor, unknown substrate 6	fig|6666666.59329.peg.3541	fig|6666666.59330.peg.3350	fig|6666666.59331.peg.3251	fig|6666666.59332.peg.544	fig|6666666.59334.peg.1406	fig|6666666.59335.peg.1290	fig|6666666.59336.peg.4654	fig|6666666.59333.peg.3308	fig|6666666.59338.peg.3466	fig|6666666.59339.peg.3749	fig|6666666.59340.peg.1312	fig|6666666.59337.peg.3584	fig|6666666.59342.peg.2959	None	None	fig|6666666.59345.peg.866	fig|6666666.59341.peg.469	fig|6666666.59347.peg.2235	fig|6666666.59346.peg.1686	fig|6666666.59348.peg.2950	None	fig|6666666.59350.peg.4381	None	None	None	None

1481	General secretion pathway protein A	fig|6666666.59329.peg.606	fig|6666666.59330.peg.939	fig|6666666.59331.peg.3461	fig|6666666.59332.peg.1505	fig|6666666.59334.peg.737	fig|6666666.59335.peg.204	fig|6666666.59336.peg.3311	fig|6666666.59333.peg.185	fig|6666666.59338.peg.802	fig|6666666.59339.peg.7765	None	fig|6666666.59337.peg.2	None	None	None	fig|6666666.59345.peg.4899	fig|6666666.59341.peg.3656	fig|6666666.59347.peg.3129	fig|6666666.59346.peg.3115	fig|6666666.59348.peg.1248	fig|6666666.59349.peg.1675	None	fig|6666666.59351.peg.2635	None	None	fig|6666666.59354.peg.5978

9658	Tellurite resistance protein	fig|6666666.59329.peg.4468	fig|6666666.59330.peg.1141	fig|6666666.59331.peg.5092	fig|6666666.59332.peg.2483	fig|6666666.59334.peg.2011	fig|6666666.59335.peg.1128	fig|6666666.59336.peg.113	fig|6666666.59333.peg.5490	fig|6666666.59338.peg.2200	fig|6666666.59339.peg.6942	fig|6666666.59340.peg.1659	fig|6666666.59337.peg.2348	fig|6666666.59342.peg.2451	None	None	fig|6666666.59345.peg.2264	fig|6666666.59341.peg.520	fig|6666666.59347.peg.3709	fig|6666666.59346.peg.3451	fig|6666666.59348.peg.2667	None	fig|6666666.59350.peg.943	None	None	None	None

2099	Fatty acid desaturase EC 1.14.19.1 ; Delta-9 fatty acid desaturase EC 1.14.19.1	fig|6666666.59329.peg.1388	fig|6666666.59330.peg.3298	fig|6666666.59331.peg.6101	fig|6666666.59332.peg.4664	None	fig|6666666.59335.peg.4402	fig|6666666.59336.peg.3544	fig|6666666.59333.peg.3433	fig|6666666.59338.peg.3849	fig|6666666.59339.peg.8476	None	fig|6666666.59337.peg.2112	fig|6666666.59342.peg.4729	None	None	fig|6666666.59345.peg.3350	fig|6666666.59341.peg.5444	fig|6666666.59347.peg.3530	fig|6666666.59346.peg.4736	fig|6666666.59348.peg.2160	fig|6666666.59349.peg.921	None	fig|6666666.59351.peg.3869	None	None	fig|6666666.59354.peg.629

7801	Starvation lipoprotein Slp paralog	fig|6666666.59329.peg.3410	fig|6666666.59330.peg.4008	None	fig|6666666.59332.peg.942	fig|6666666.59334.peg.3608	fig|6666666.59335.peg.2035	fig|6666666.59336.peg.3818	fig|6666666.59333.peg.1350	fig|6666666.59338.peg.2734	None	fig|6666666.59340.peg.627	fig|6666666.59337.peg.2746	None	fig|6666666.71181.peg.1940	fig|6666666.59344.peg.2850	fig|6666666.59345.peg.4302	fig|6666666.59341.peg.5237	fig|6666666.59347.peg.3027	fig|6666666.59346.peg.1480	fig|6666666.59348.peg.5230	None	None	None	fig|6666666.59353.peg.1467	fig|6666666.59355.peg.2018	None

2396	Transcriptional repressor protein TrpR	fig|6666666.59329.peg.2736	fig|6666666.59330.peg.2888	fig|6666666.59331.peg.932	fig|6666666.59332.peg.1327	None	fig|6666666.59335.peg.2060	fig|6666666.59336.peg.844	fig|6666666.59333.peg.4104	None	fig|6666666.59339.peg.7501	fig|6666666.59340.peg.698	fig|6666666.59337.peg.152	None	fig|6666666.71181.peg.443	fig|6666666.59344.peg.1350	fig|6666666.59345.peg.3690	fig|6666666.59341.peg.3009	fig|6666666.59347.peg.4050	fig|6666666.59346.peg.260	fig|6666666.59348.peg.2857	None	None	None	fig|6666666.59353.peg.55	fig|6666666.59355.peg.662	None

4960	UPF0235 protein VC0458	None	fig|6666666.59330.peg.339	None	fig|6666666.59332.peg.1632	None	fig|6666666.59335.peg.3085	fig|6666666.59336.peg.4103	fig|6666666.59333.peg.764	fig|6666666.59338.peg.2881	None	fig|6666666.59340.peg.3460	fig|6666666.59337.peg.520	fig|6666666.59342.peg.2689	fig|6666666.71181.peg.1868	fig|6666666.59344.peg.4343	fig|6666666.59345.peg.5009	fig|6666666.59341.peg.3058	fig|6666666.59347.peg.3868	fig|6666666.59346.peg.1777	fig|6666666.59348.peg.227	None	fig|6666666.59350.peg.8302	None	fig|6666666.59353.peg.3234	fig|6666666.59355.peg.3116	None

5434	putative lipase	fig|6666666.59329.peg.5065	fig|6666666.59330.peg.4367	fig|6666666.59331.peg.1113	fig|6666666.59332.peg.3537	fig|6666666.59334.peg.4595	fig|6666666.59335.peg.3755	None	fig|6666666.59333.peg.4999	fig|6666666.59338.peg.2093	fig|6666666.59339.peg.3404	fig|6666666.59340.peg.3425	fig|6666666.59337.peg.4963	fig|6666666.59342.peg.4371	None	None	fig|6666666.59345.peg.115	None	fig|6666666.59347.peg.827	fig|6666666.59346.peg.2915	fig|6666666.59348.peg.1631	fig|6666666.59349.peg.4465	None	fig|6666666.59351.peg.805	None	None	fig|6666666.59354.peg.4814

1206	lysin, extracellular zinc protease EC 3.4.24.25 Pseudolysin, extracellular zinc protease EC 3.4.24.26	fig|6666666.59329.peg.3742	fig|6666666.59330.peg.3644	None	None	None	fig|6666666.59335.peg.5743	fig|6666666.59336.peg.6134	None	fig|6666666.59338.peg.5953	fig|6666666.59339.peg.3354	fig|6666666.59340.peg.2056	fig|6666666.59337.peg.1487	fig|6666666.59342.peg.847	fig|6666666.71181.peg.1778	fig|6666666.59344.peg.5144	fig|6666666.59345.peg.357	None	fig|6666666.59347.peg.3560	None	fig|6666666.59348.peg.491	fig|6666666.59349.peg.4370	None	fig|6666666.59351.peg.2719	fig|6666666.59353.peg.2717	fig|6666666.59355.peg.2967	fig|6666666.59354.peg.3931

3859	Protease II	fig|6666666.59329.peg.4818	fig|6666666.59330.peg.752	fig|6666666.59331.peg.3418	fig|6666666.59332.peg.1780	None	fig|6666666.59335.peg.5737	fig|6666666.59336.peg.1384	fig|6666666.59333.peg.1272	None	fig|6666666.59339.peg.3347	None	fig|6666666.59337.peg.1290	fig|6666666.59342.peg.5964	fig|6666666.71181.peg.866	fig|6666666.59344.peg.3470	fig|6666666.59345.peg.4476	None	None	fig|6666666.59346.peg.2206	None	fig|6666666.59349.peg.4933	None	fig|6666666.59351.peg.4552	fig|6666666.59353.peg.1408	fig|6666666.59355.peg.1386	fig|6666666.59354.peg.1007

11304	Arsenical-resistance protein ACR3	fig|6666666.59329.peg.1257	fig|6666666.59330.peg.3869	fig|6666666.59331.peg.5162	fig|6666666.59332.peg.2814	fig|6666666.59334.peg.2445	fig|6666666.59335.peg.1466	fig|6666666.59336.peg.3327	fig|6666666.59333.peg.5497	fig|6666666.59338.peg.3692	fig|6666666.59339.peg.4429	fig|6666666.59340.peg.1709	fig|6666666.59337.peg.1936	fig|6666666.59342.peg.5260	None	None	fig|6666666.59345.peg.4028	fig|6666666.59341.peg.650	fig|6666666.59347.peg.3942	fig|6666666.59346.peg.2751	fig|6666666.59348.peg.5524	None	fig|6666666.59350.peg.3919	None	None	None	None

10872	Phosphatidylglycerophosphatase B EC 3.1.3.27	fig|6666666.59329.peg.3205	fig|6666666.59330.peg.4733	fig|6666666.59331.peg.2044	fig|6666666.59332.peg.1527	None	fig|6666666.59335.peg.5569	fig|6666666.59336.peg.6378	None	fig|6666666.59338.peg.3572	fig|6666666.59339.peg.5559	None	fig|6666666.59337.peg.1224	fig|6666666.59342.peg.4344	fig|6666666.71181.peg.4194	fig|6666666.59344.peg.486	fig|6666666.59345.peg.4410	fig|6666666.59341.peg.2140	fig|6666666.59347.peg.597	fig|6666666.59346.peg.3658	fig|6666666.59348.peg.5249	None	None	None	fig|6666666.59353.peg.1803	fig|6666666.59355.peg.1213	None

2115	Acyl dehydratase	fig|6666666.59329.peg.2809	fig|6666666.59330.peg.3535	fig|6666666.59331.peg.5745	fig|6666666.59332.peg.2351	fig|6666666.59334.peg.3940	fig|6666666.59335.peg.619	fig|6666666.59336.peg.4306	fig|6666666.59333.peg.3078	fig|6666666.59338.peg.879	fig|6666666.59339.peg.4279	None	fig|6666666.59337.peg.1433	fig|6666666.59342.peg.282	None	None	fig|6666666.59345.peg.403	fig|6666666.59341.peg.760	fig|6666666.59347.peg.996	fig|6666666.59346.peg.3417	fig|6666666.59348.peg.4032	None	fig|6666666.59350.peg.7885	fig|6666666.59351.peg.2870	None	None	None

3131	FIG01202929 hypothetical protein	fig|6666666.59329.peg.3307	fig|6666666.59330.peg.3686	None	fig|6666666.59332.peg.1992	fig|6666666.59334.peg.567	fig|6666666.59335.peg.3319	fig|6666666.59336.peg.5357	None	fig|6666666.59338.peg.2215	fig|6666666.59339.peg.879	None	fig|6666666.59337.peg.5635	fig|6666666.59342.peg.1211	fig|6666666.71181.peg.1191	fig|6666666.59344.peg.1515	fig|6666666.59345.peg.1227	fig|6666666.59341.peg.3307	fig|6666666.59347.peg.516	fig|6666666.59346.peg.220	fig|6666666.59348.peg.2026	None	None	None	fig|6666666.59353.peg.1210	fig|6666666.59355.peg.266	None

5766	Mannose-6-phosphate isomerase EC 5.3.1.8	fig|6666666.59329.peg.5869	None	fig|6666666.59331.peg.5519	fig|6666666.59332.peg.3105	fig|6666666.59334.peg.923	fig|6666666.59335.peg.5817	fig|6666666.59336.peg.4836	None	fig|6666666.59338.peg.3046	fig|6666666.59339.peg.420	None	fig|6666666.59337.peg.4657	fig|6666666.59342.peg.4111	fig|6666666.71181.peg.2709	fig|6666666.59344.peg.3835	fig|6666666.59345.peg.1766	fig|6666666.59341.peg.3994	fig|6666666.59347.peg.4996	fig|6666666.59346.peg.3958	fig|6666666.59348.peg.618	None	None	None	fig|6666666.59353.peg.3969	fig|6666666.59355.peg.3885	None

5765	Acyl carrier protein phosphodiesterase EC 3.1.4.14	fig|6666666.59329.peg.3196	fig|6666666.59330.peg.3414	None	fig|6666666.59332.peg.1059	fig|6666666.59334.peg.688	fig|6666666.59335.peg.4396	None	None	fig|6666666.59338.peg.4854	fig|6666666.59339.peg.8483	None	fig|6666666.59337.peg.2509	fig|6666666.59342.peg.888	fig|6666666.71181.peg.2736	fig|6666666.59344.peg.1108	fig|6666666.59345.peg.136	fig|6666666.59341.peg.1464	fig|6666666.59347.peg.1241	fig|6666666.59346.peg.4008	fig|6666666.59348.peg.5075	fig|6666666.59349.peg.1848	None	None	fig|6666666.59353.peg.788	fig|6666666.59355.peg.917	None

5958	FIG004599 Hypothetical protein	fig|6666666.59329.peg.4316	fig|6666666.59330.peg.4722	fig|6666666.59331.peg.3611	fig|6666666.59332.peg.2296	None	fig|6666666.59335.peg.5515	fig|6666666.59336.peg.2856	fig|6666666.59333.peg.1164	fig|6666666.59338.peg.3633	fig|6666666.59339.peg.5152	fig|6666666.59340.peg.3076	fig|6666666.59337.peg.4477	fig|6666666.59342.peg.4232	None	None	fig|6666666.59345.peg.2173	fig|6666666.59341.peg.2354	fig|6666666.59347.peg.2866	fig|6666666.59346.peg.3693	None	fig|6666666.59349.peg.4587	None	fig|6666666.59351.peg.5497	None	None	fig|6666666.59354.peg.3306

3565	FIG003551 hypothetical protein	fig|6666666.59329.peg.1541	fig|6666666.59330.peg.338	None	fig|6666666.59332.peg.1633	fig|6666666.59334.peg.1366	fig|6666666.59335.peg.4700	fig|6666666.59336.peg.4102	fig|6666666.59333.peg.763	fig|6666666.59338.peg.2882	fig|6666666.59339.peg.7816	fig|6666666.59340.peg.3461	fig|6666666.59337.peg.519	None	None	None	fig|6666666.59345.peg.5008	fig|6666666.59341.peg.3059	fig|6666666.59347.peg.3869	fig|6666666.59346.peg.1776	fig|6666666.59348.peg.228	fig|6666666.59349.peg.797	None	fig|6666666.59351.peg.3527	None	None	fig|6666666.59354.peg.529

3156	Predicted hydrolase or acyltransferase	fig|6666666.59329.peg.2360	fig|6666666.59330.peg.1495	fig|6666666.59331.peg.4105	fig|6666666.59332.peg.2056	fig|6666666.59334.peg.4135	fig|6666666.59335.peg.744	fig|6666666.59336.peg.1163	fig|6666666.59333.peg.1736	fig|6666666.59338.peg.1722	fig|6666666.59339.peg.5608	None	fig|6666666.59337.peg.182	None	None	None	fig|6666666.59345.peg.4511	fig|6666666.59341.peg.324	fig|6666666.59347.peg.3676	fig|6666666.59346.peg.4240	fig|6666666.59348.peg.2473	fig|6666666.59349.peg.3300	None	fig|6666666.59351.peg.5142	None	None	fig|6666666.59354.peg.802

10720	hypothetical protein	fig|6666666.59329.peg.6573	fig|6666666.59330.peg.894	fig|6666666.59331.peg.6028	fig|6666666.59332.peg.3107	None	fig|6666666.59335.peg.3523	fig|6666666.59336.peg.5940	fig|6666666.59333.peg.5158	fig|6666666.59338.peg.3358	fig|6666666.59339.peg.4053	fig|6666666.59340.peg.772	fig|6666666.59337.peg.5349	None	None	None	fig|6666666.59345.peg.417	fig|6666666.59341.peg.5097	fig|6666666.59347.peg.366	fig|6666666.59346.peg.672	fig|6666666.59348.peg.5187	fig|6666666.59349.peg.4642	None	fig|6666666.59351.peg.4622	None	None	fig|6666666.59354.peg.5043

3196	hypothetical protein	fig|6666666.59329.peg.2614	None	None	fig|6666666.59332.peg.3061	fig|6666666.59334.peg.4574	fig|6666666.59335.peg.2117	fig|6666666.59336.peg.738	fig|6666666.59333.peg.4178	fig|6666666.59338.peg.1878	fig|6666666.59339.peg.5718	None	fig|6666666.59337.peg.4324	fig|6666666.59342.peg.4673	None	None	fig|6666666.59345.peg.3919	fig|6666666.59341.peg.1874	fig|6666666.59347.peg.542	fig|6666666.59346.peg.4839	fig|6666666.59348.peg.5033	fig|6666666.59349.peg.3027	fig|6666666.59350.peg.7442	fig|6666666.59351.peg.3196	None	None	fig|6666666.59354.peg.3272

4659	5,10-methylenetetrahydrofolate reductase EC 1.5.1.20	fig|6666666.59329.peg.2452	fig|6666666.59330.peg.1676	None	fig|6666666.59332.peg.2930	fig|6666666.59334.peg.2176	fig|6666666.59335.peg.424	None	fig|6666666.59333.peg.3010	fig|6666666.59338.peg.1055	fig|6666666.59339.peg.6371	None	fig|6666666.59337.peg.1678	None	fig|6666666.71181.peg.3885	fig|6666666.59344.peg.1009	fig|6666666.59345.peg.5402	fig|6666666.59341.peg.945	fig|6666666.59347.peg.4924	fig|6666666.59346.peg.1002	fig|6666666.59348.peg.1952	None	fig|6666666.59350.peg.6782	None	fig|6666666.59353.peg.2531	fig|6666666.59355.peg.2671	None

7885	Molybdenum cofactor biosynthesis protein MoaE; Molybdopterin converting factor subunit 2	None	fig|6666666.59330.peg.1714	fig|6666666.59331.peg.2795	fig|6666666.59332.peg.1525	fig|6666666.59334.peg.301	None	None	fig|6666666.59333.peg.1328	fig|6666666.59338.peg.2308	fig|6666666.59339.peg.143	None	fig|6666666.59337.peg.4028	fig|6666666.59342.peg.876	fig|6666666.71181.peg.2535	fig|6666666.59344.peg.4104	fig|6666666.59345.peg.1109	fig|6666666.59341.peg.2163	fig|6666666.59347.peg.5153	fig|6666666.59346.peg.536	fig|6666666.59348.peg.720	None	fig|6666666.59350.peg.504	None	fig|6666666.59353.peg.2145	fig|6666666.59355.peg.2674	None

10000	Ornithine cyclodeaminase EC 4.3.1.12	fig|6666666.59329.peg.5733	None	fig|6666666.59331.peg.212	None	None	fig|6666666.59335.peg.5357	fig|6666666.59336.peg.5063	fig|6666666.59333.peg.461	fig|6666666.59338.peg.4638	fig|6666666.59339.peg.8781	None	None	fig|6666666.59342.peg.3601	fig|6666666.71181.peg.627	fig|6666666.59344.peg.4179	fig|6666666.59345.peg.5599	None	fig|6666666.59347.peg.140	fig|6666666.59346.peg.4019	None	fig|6666666.59349.peg.4105	fig|6666666.59350.peg.319	fig|6666666.59351.peg.682	fig|6666666.59353.peg.3052	fig|6666666.59355.peg.2294	fig|6666666.59354.peg.1946

7262	Oxaloacetate decarboxylase beta chain EC 4.1.1.3	fig|6666666.59329.peg.4373	fig|6666666.59330.peg.2830	None	fig|6666666.59332.peg.1566	fig|6666666.59334.peg.250	fig|6666666.59335.peg.5710	fig|6666666.59336.peg.5265	fig|6666666.59333.peg.4924	fig|6666666.59338.peg.439	fig|6666666.59339.peg.8922	None	fig|6666666.59337.peg.2029	fig|6666666.59342.peg.2696	None	None	fig|6666666.59345.peg.2446	fig|6666666.59341.peg.2561	fig|6666666.59347.peg.3221	fig|6666666.59346.peg.5037	fig|6666666.59348.peg.3339	fig|6666666.59349.peg.1837	None	fig|6666666.59351.peg.5271	None	None	fig|6666666.59354.peg.6503

1285	Permease of the major facilitator superfamily	None	fig|6666666.59330.peg.728	fig|6666666.59331.peg.3947	fig|6666666.59332.peg.4723	fig|6666666.59334.peg.2883	fig|6666666.59335.peg.6133	fig|6666666.59336.peg.2852	fig|6666666.59333.peg.5078	None	fig|6666666.59339.peg.1946	None	fig|6666666.59337.peg.3768	fig|6666666.59342.peg.5773	None	None	fig|6666666.59345.peg.2795	fig|6666666.59341.peg.3710	fig|6666666.59347.peg.370	fig|6666666.59346.peg.3226	fig|6666666.59348.peg.2564	fig|6666666.59349.peg.931	fig|6666666.59350.peg.3291	fig|6666666.59351.peg.3863	None	None	fig|6666666.59354.peg.643

10310	Glutaredoxin	fig|6666666.59329.peg.1565	fig|6666666.59330.peg.937	fig|6666666.59331.peg.5907	fig|6666666.59332.peg.5243	fig|6666666.59334.peg.3833	fig|6666666.59335.peg.4219	fig|6666666.59336.peg.4183	fig|6666666.59333.peg.4458	fig|6666666.59338.peg.4427	fig|6666666.59339.peg.6521	None	fig|6666666.59337.peg.3554	None	None	None	fig|6666666.59345.peg.465	fig|6666666.59341.peg.3436	fig|6666666.59347.peg.1347	fig|6666666.59346.peg.3456	fig|6666666.59348.peg.515	fig|6666666.59349.peg.826	None	fig|6666666.59351.peg.3374	None	None	fig|6666666.59354.peg.2655

5044	FIG01200791 hypothetical protein	fig|6666666.59329.peg.6305	fig|6666666.59330.peg.2090	None	fig|6666666.59332.peg.4119	fig|6666666.59334.peg.4899	fig|6666666.59335.peg.5176	fig|6666666.59336.peg.3931	fig|6666666.59333.peg.3935	fig|6666666.59338.peg.1631	fig|6666666.59339.peg.3726	None	fig|6666666.59337.peg.4688	fig|6666666.59342.peg.6333	None	None	fig|6666666.59345.peg.817	fig|6666666.59341.peg.4373	fig|6666666.59347.peg.3479	fig|6666666.59346.peg.4339	fig|6666666.59348.peg.3649	fig|6666666.59349.peg.4378	None	fig|6666666.59351.peg.5481	None	None	fig|6666666.59354.peg.3136

5188	Putative phosphatase	fig|6666666.59329.peg.1672	fig|6666666.59330.peg.2409	None	fig|6666666.59332.peg.5533	fig|6666666.59334.peg.4761	fig|6666666.59335.peg.4048	fig|6666666.59336.peg.6119	fig|6666666.59333.peg.2027	fig|6666666.59338.peg.4203	fig|6666666.59339.peg.5533	None	fig|6666666.59337.peg.1508	fig|6666666.59342.peg.6277	fig|6666666.71181.peg.404	fig|6666666.59344.peg.1295	fig|6666666.59345.peg.3021	fig|6666666.59341.peg.3640	fig|6666666.59347.peg.5291	fig|6666666.59346.peg.1917	None	None	None	None	fig|6666666.59353.peg.94	fig|6666666.59355.peg.704	None

5254	INTEGRAL MEMBRANE PROTEIN Rhomboid family	fig|6666666.59329.peg.366	fig|6666666.59330.peg.987	fig|6666666.59331.peg.2280	fig|6666666.59332.peg.261	fig|6666666.59334.peg.2188	fig|6666666.59335.peg.103	fig|6666666.59336.peg.186	fig|6666666.59333.peg.1575	fig|6666666.59338.peg.2871	fig|6666666.59339.peg.7647	fig|6666666.59340.peg.2895	fig|6666666.59337.peg.2544	fig|6666666.59342.peg.6108	None	None	fig|6666666.59345.peg.5536	fig|6666666.59341.peg.4839	fig|6666666.59347.peg.5248	fig|6666666.59346.peg.924	fig|6666666.59348.peg.45	None	fig|6666666.59350.peg.5212	None	None	None	None

8880	FIG01200309 hypothetical protein	fig|6666666.59329.peg.2093	fig|6666666.59330.peg.260	fig|6666666.59331.peg.6145	fig|6666666.59332.peg.15	None	fig|6666666.59335.peg.48	fig|6666666.59336.peg.1458	fig|6666666.59333.peg.834	None	fig|6666666.59339.peg.7583	fig|6666666.59340.peg.3230	fig|6666666.59337.peg.970	None	fig|6666666.71181.peg.4497	fig|6666666.59344.peg.5720	fig|6666666.59345.peg.2705	fig|6666666.59341.peg.162	None	fig|6666666.59346.peg.1072	fig|6666666.59348.peg.108	fig|6666666.59349.peg.2918	None	None	fig|6666666.59353.peg.4942	fig|6666666.59355.peg.3351	None

9571	FIG01200138 hypothetical protein	fig|6666666.59329.peg.5522	fig|6666666.59330.peg.322	fig|6666666.59331.peg.4560	None	fig|6666666.59334.peg.1206	fig|6666666.59335.peg.870	fig|6666666.59336.peg.5407	fig|6666666.59333.peg.1439	fig|6666666.59338.peg.5098	None	None	fig|6666666.59337.peg.1050	fig|6666666.59342.peg.5414	None	None	fig|6666666.59345.peg.4232	fig|6666666.59341.peg.2674	fig|6666666.59347.peg.2637	fig|6666666.59346.peg.2075	fig|6666666.59348.peg.911	fig|6666666.59349.peg.4455	fig|6666666.59350.peg.2703	fig|6666666.59351.peg.692	None	None	fig|6666666.59354.peg.4916

921	Predicted transcriptional regulator	fig|6666666.59329.peg.5910	fig|6666666.59330.peg.5161	fig|6666666.59331.peg.4220	fig|6666666.59332.peg.167	None	None	fig|6666666.59336.peg.2169	fig|6666666.59333.peg.1385	fig|6666666.59338.peg.1171	None	fig|6666666.59340.peg.2573	fig|6666666.59337.peg.408	fig|6666666.59342.peg.5400	fig|6666666.71181.peg.4116	fig|6666666.59344.peg.989	fig|6666666.59345.peg.2553	fig|6666666.59341.peg.191	fig|6666666.59347.peg.4533	fig|6666666.59346.peg.799	fig|6666666.59348.peg.2309	None	None	None	fig|6666666.59353.peg.4732	fig|6666666.59355.peg.4688	None

842	FIG01199803 hypothetical protein	fig|6666666.59329.peg.3439	fig|6666666.59330.peg.2747	fig|6666666.59331.peg.5327	fig|6666666.59332.peg.1314	None	fig|6666666.59335.peg.3568	fig|6666666.59336.peg.3268	fig|6666666.59333.peg.3228	fig|6666666.59338.peg.646	fig|6666666.59339.peg.8363	None	fig|6666666.59337.peg.289	fig|6666666.59342.peg.4128	None	None	fig|6666666.59345.peg.2126	fig|6666666.59341.peg.4030	fig|6666666.59347.peg.40	fig|6666666.59346.peg.3066	fig|6666666.59348.peg.5097	fig|6666666.59349.peg.4347	None	fig|6666666.59351.peg.913	None	None	fig|6666666.59354.peg.6470

2406	Sodium-dependent phosphate transporter	fig|6666666.59329.peg.5205	None	fig|6666666.59331.peg.3441	fig|6666666.59332.peg.4747	None	fig|6666666.59335.peg.1016	fig|6666666.59336.peg.5471	fig|6666666.59333.peg.3953	fig|6666666.59338.peg.2733	fig|6666666.59339.peg.7456	fig|6666666.59340.peg.626	fig|6666666.59337.peg.127	fig|6666666.59342.peg.1391	None	None	fig|6666666.59345.peg.3716	fig|6666666.59341.peg.5238	fig|6666666.59347.peg.3026	fig|6666666.59346.peg.3086	fig|6666666.59348.peg.1115	fig|6666666.59349.peg.3434	None	fig|6666666.59351.peg.1673	None	None	fig|6666666.59354.peg.6029

6721	General secretion pathway protein L	fig|6666666.59329.peg.1231	fig|6666666.59330.peg.425	None	fig|6666666.59332.peg.2997	fig|6666666.59334.peg.120	fig|6666666.59335.peg.710	fig|6666666.59336.peg.3113	fig|6666666.59333.peg.513	fig|6666666.59338.peg.1431	fig|6666666.59339.peg.6210	fig|6666666.59340.peg.723	fig|6666666.59337.peg.4253	None	None	None	fig|6666666.59345.peg.2669	fig|6666666.59341.peg.4545	fig|6666666.59347.peg.4471	fig|6666666.59346.peg.2228	fig|6666666.59348.peg.1391	fig|6666666.59349.peg.2167	None	fig|6666666.59351.peg.4339	None	None	fig|6666666.59354.peg.705

6725	FIG01200995 hypothetical protein	fig|6666666.59329.peg.1354	fig|6666666.59330.peg.2995	None	fig|6666666.59332.peg.5170	fig|6666666.59334.peg.4534	fig|6666666.59335.peg.2731	fig|6666666.59336.peg.333	fig|6666666.59333.peg.859	fig|6666666.59338.peg.1153	fig|6666666.59339.peg.7206	fig|6666666.59340.peg.1742	fig|6666666.59337.peg.3935	fig|6666666.59342.peg.2211	None	None	fig|6666666.59345.peg.4332	None	fig|6666666.59347.peg.2941	fig|6666666.59346.peg.2008	fig|6666666.59348.peg.1059	fig|6666666.59349.peg.1814	None	fig|6666666.59351.peg.1113	None	None	fig|6666666.59354.peg.4092

2281	Cytoplasmic copper homeostasis protein cutC	fig|6666666.59329.peg.1125	fig|6666666.59330.peg.4241	fig|6666666.59331.peg.4048	fig|6666666.59332.peg.767	fig|6666666.59334.peg.4577	fig|6666666.59335.peg.1137	fig|6666666.59336.peg.5464	fig|6666666.59333.peg.357	fig|6666666.59338.peg.1814	None	None	fig|6666666.59337.peg.720	None	fig|6666666.71181.peg.1910	fig|6666666.59344.peg.2531	fig|6666666.59345.peg.3669	fig|6666666.59341.peg.3367	fig|6666666.59347.peg.4271	fig|6666666.59346.peg.5030	fig|6666666.59348.peg.1334	None	None	None	fig|6666666.59353.peg.1500	fig|6666666.59355.peg.2051	None

860	Putative integral membrane protein	fig|6666666.59329.peg.2154	None	fig|6666666.59331.peg.1412	fig|6666666.59332.peg.1	fig|6666666.59334.peg.5519	None	fig|6666666.59336.peg.3184	fig|6666666.59333.peg.2784	fig|6666666.59338.peg.3431	fig|6666666.59339.peg.2337	fig|6666666.59340.peg.2368	None	fig|6666666.59342.peg.1766	fig|6666666.71181.peg.557	fig|6666666.59344.peg.1908	fig|6666666.59345.peg.4536	fig|6666666.59341.peg.2869	fig|6666666.59347.peg.494	fig|6666666.59346.peg.3351	fig|6666666.59348.peg.2984	None	None	None	fig|6666666.59353.peg.1189	fig|6666666.59355.peg.288	None

2024	Histone acetyltransferase HPA2	fig|6666666.59329.peg.1249	fig|6666666.59330.peg.3226	fig|6666666.59331.peg.2521	fig|6666666.59332.peg.1912	fig|6666666.59334.peg.3800	fig|6666666.59335.peg.2287	fig|6666666.59336.peg.1577	fig|6666666.59333.peg.3546	fig|6666666.59338.peg.4459	fig|6666666.59339.peg.4146	None	fig|6666666.59337.peg.1974	fig|6666666.59342.peg.1731	fig|6666666.71181.peg.5122	fig|6666666.59344.peg.321	fig|6666666.59345.peg.5610	None	fig|6666666.59347.peg.5302	None	fig|6666666.59348.peg.2226	None	None	None	fig|6666666.59353.peg.561	fig|6666666.59355.peg.46	None

10154	Predicted protein-tyrosine phosphatase	fig|6666666.59329.peg.2354	fig|6666666.59330.peg.1551	fig|6666666.59331.peg.731	fig|6666666.59332.peg.468	fig|6666666.59334.peg.3876	fig|6666666.59335.peg.6238	fig|6666666.59336.peg.632	fig|6666666.59333.peg.4896	fig|6666666.59338.peg.973	fig|6666666.59339.peg.8550	None	fig|6666666.59337.peg.1634	fig|6666666.59342.peg.4919	None	None	fig|6666666.59345.peg.5485	fig|6666666.59341.peg.645	fig|6666666.59347.peg.3937	fig|6666666.59346.peg.618	fig|6666666.59348.peg.738	None	fig|6666666.59350.peg.3061	fig|6666666.59351.peg.1974	None	None	None

6702	Transcriptional regulator, LuxR family	fig|6666666.59329.peg.231	fig|6666666.59330.peg.2068	fig|6666666.59331.peg.887	fig|6666666.59332.peg.5605	None	fig|6666666.59335.peg.3853	fig|6666666.59336.peg.1911	fig|6666666.59333.peg.4821	None	fig|6666666.59339.peg.8347	None	fig|6666666.59337.peg.273	fig|6666666.59342.peg.742	fig|6666666.71181.peg.2677	fig|6666666.59344.peg.4294	fig|6666666.59345.peg.5229	fig|6666666.59341.peg.2658	None	fig|6666666.59346.peg.3998	fig|6666666.59348.peg.3928	None	fig|6666666.59350.peg.5269	None	fig|6666666.59353.peg.3892	fig|6666666.59355.peg.3800	None

11210	General secretion pathway protein N	fig|6666666.59329.peg.1234	fig|6666666.59330.peg.427	None	fig|6666666.59332.peg.2999	fig|6666666.59334.peg.116	fig|6666666.59335.peg.708	fig|6666666.59336.peg.4429	fig|6666666.59333.peg.515	fig|6666666.59338.peg.1428	fig|6666666.59339.peg.6213	None	fig|6666666.59337.peg.4255	fig|6666666.59342.peg.3597	None	None	fig|6666666.59345.peg.2667	fig|6666666.59341.peg.1410	fig|6666666.59347.peg.4473	fig|6666666.59346.peg.2230	fig|6666666.59348.peg.1395	fig|6666666.59349.peg.2165	None	fig|6666666.59351.peg.4337	None	None	fig|6666666.59354.peg.708

4897	Sugar diacid utilization regulator SdaR	fig|6666666.59329.peg.6221	fig|6666666.59330.peg.3254	None	None	fig|6666666.59334.peg.5133	fig|6666666.59335.peg.3176	fig|6666666.59336.peg.4756	fig|6666666.59333.peg.2935	fig|6666666.59338.peg.2994	fig|6666666.59339.peg.1038	fig|6666666.59340.peg.1138	fig|6666666.59337.peg.1248	fig|6666666.59342.peg.4636	fig|6666666.71181.peg.3494	fig|6666666.59344.peg.5513	fig|6666666.59345.peg.4430	fig|6666666.59341.peg.5196	fig|6666666.59347.peg.3159	None	fig|6666666.59348.peg.2114	None	None	None	fig|6666666.59353.peg.168	fig|6666666.59355.peg.780	None

4265	HAD superfamily hydrolase	fig|6666666.59329.peg.1391	fig|6666666.59330.peg.3300	None	fig|6666666.59332.peg.4631	None	fig|6666666.59335.peg.4796	fig|6666666.59336.peg.3542	fig|6666666.59333.peg.3431	fig|6666666.59338.peg.1630	None	None	fig|6666666.59337.peg.2114	None	fig|6666666.71181.peg.2595	fig|6666666.59344.peg.909	fig|6666666.59345.peg.3352	fig|6666666.59341.peg.5972	fig|6666666.59347.peg.3478	None	fig|6666666.59348.peg.1420	fig|6666666.59349.peg.3677	fig|6666666.59350.peg.318	None	fig|6666666.59353.peg.2621	fig|6666666.59355.peg.2583	fig|6666666.59354.peg.6241

10617	putative exported protein	fig|6666666.59329.peg.3007	None	fig|6666666.59331.peg.3296	fig|6666666.59332.peg.4843	fig|6666666.59334.peg.3764	fig|6666666.59335.peg.5588	fig|6666666.59336.peg.3197	None	fig|6666666.59338.peg.1599	fig|6666666.59339.peg.4213	fig|6666666.59340.peg.3806	None	fig|6666666.59342.peg.3237	fig|6666666.71181.peg.1752	fig|6666666.59344.peg.1621	fig|6666666.59345.peg.1429	fig|6666666.59341.peg.3071	fig|6666666.59347.peg.4422	fig|6666666.59346.peg.4188	fig|6666666.59348.peg.4703	None	None	None	fig|6666666.59353.peg.2689	fig|6666666.59355.peg.1790	None

7874	Cytochrome oxidase biogenesis protein Surf1, facilitates heme A insertion	fig|6666666.59329.peg.1238	fig|6666666.59330.peg.3217	fig|6666666.59331.peg.4626	fig|6666666.59332.peg.3956	fig|6666666.59334.peg.999	fig|6666666.59335.peg.2866	fig|6666666.59336.peg.3600	fig|6666666.59333.peg.5070	None	None	fig|6666666.59340.peg.2271	fig|6666666.59337.peg.5081	None	None	None	fig|6666666.59345.peg.5618	fig|6666666.59341.peg.5973	fig|6666666.59347.peg.1548	fig|6666666.59346.peg.4309	fig|6666666.59348.peg.5502	fig|6666666.59349.peg.3505	fig|6666666.59350.peg.2028	fig|6666666.59351.peg.4385	None	None	fig|6666666.59354.peg.2070

347	Mannitol operon repressor	fig|6666666.59329.peg.5466	None	fig|6666666.59331.peg.1346	fig|6666666.59332.peg.2455	fig|6666666.59334.peg.919	fig|6666666.59335.peg.1284	fig|6666666.59336.peg.4603	fig|6666666.59333.peg.102	fig|6666666.59338.peg.150	None	None	fig|6666666.59337.peg.1944	fig|6666666.59342.peg.2784	fig|6666666.71181.peg.664	fig|6666666.59344.peg.4124	fig|6666666.59345.peg.4048	fig|6666666.59341.peg.4100	fig|6666666.59347.peg.235	fig|6666666.59346.peg.4436	fig|6666666.59348.peg.4466	None	None	None	fig|6666666.59353.peg.4105	fig|6666666.59355.peg.2335	None

9410	FIG01201121 hypothetical protein	fig|6666666.59329.peg.5060	fig|6666666.59330.peg.1487	fig|6666666.59331.peg.5361	fig|6666666.59332.peg.1532	fig|6666666.59334.peg.1732	fig|6666666.59335.peg.5101	fig|6666666.59336.peg.590	fig|6666666.59333.peg.4193	fig|6666666.59338.peg.3425	fig|6666666.59339.peg.5191	fig|6666666.59340.peg.2176	fig|6666666.59337.peg.188	fig|6666666.59342.peg.4484	None	None	fig|6666666.59345.peg.4518	fig|6666666.59341.peg.1463	fig|6666666.59347.peg.2914	fig|6666666.59346.peg.4848	fig|6666666.59348.peg.2169	None	fig|6666666.59350.peg.5183	None	None	None	None

11070	Anthranilate phosphoribosyltransferase like EC 2.4.2.18	fig|6666666.59329.peg.6537	None	None	fig|6666666.59332.peg.3439	fig|6666666.59334.peg.415	fig|6666666.59335.peg.2888	fig|6666666.59336.peg.3026	fig|6666666.59333.peg.716	fig|6666666.59338.peg.869	fig|6666666.59339.peg.4677	None	fig|6666666.59337.peg.3881	fig|6666666.59342.peg.6232	fig|6666666.71181.peg.2122	fig|6666666.59344.peg.5324	fig|6666666.59345.peg.288	fig|6666666.59341.peg.2160	fig|6666666.59347.peg.5148	fig|6666666.59346.peg.2190	fig|6666666.59348.peg.1984	None	None	None	fig|6666666.59353.peg.712	fig|6666666.59355.peg.199	None

7857	FIG01200104 hypothetical protein	fig|6666666.59329.peg.4344	fig|6666666.59330.peg.1024	fig|6666666.59331.peg.3402	fig|6666666.59332.peg.4228	fig|6666666.59334.peg.5595	fig|6666666.59335.peg.3363	fig|6666666.59336.peg.5981	fig|6666666.59333.peg.4842	fig|6666666.59338.peg.4556	fig|6666666.59339.peg.1549	None	fig|6666666.59337.peg.3165	fig|6666666.59342.peg.2352	None	None	fig|6666666.59345.peg.3269	fig|6666666.59341.peg.4165	fig|6666666.59347.peg.1783	fig|6666666.59346.peg.3918	fig|6666666.59348.peg.4247	fig|6666666.59349.peg.4639	None	None	None	None	fig|6666666.59354.peg.6142

7855	Lipase chaperone	fig|6666666.59329.peg.3678	fig|6666666.59330.peg.1850	fig|6666666.59331.peg.4608	fig|6666666.59332.peg.4578	None	fig|6666666.59335.peg.5323	None	fig|6666666.59333.peg.1628	fig|6666666.59338.peg.4195	fig|6666666.59339.peg.531	fig|6666666.59340.peg.3510	fig|6666666.59337.peg.2339	fig|6666666.59342.peg.2314	None	None	fig|6666666.59345.peg.1261	fig|6666666.59341.peg.4677	fig|6666666.59347.peg.88	fig|6666666.59346.peg.3433	fig|6666666.59348.peg.4903	fig|6666666.59349.peg.4564	None	fig|6666666.59351.peg.4950	None	None	fig|6666666.59354.peg.3726

3088	Transcriptional regulator, TetR family	fig|6666666.59329.peg.27	fig|6666666.59330.peg.149	fig|6666666.59331.peg.2665	fig|6666666.59332.peg.1727	fig|6666666.59334.peg.2764	fig|6666666.59335.peg.1947	fig|6666666.59336.peg.265	fig|6666666.59333.peg.2300	fig|6666666.59338.peg.1084	fig|6666666.59339.peg.8830	None	fig|6666666.59337.peg.5194	None	None	None	fig|6666666.59345.peg.5101	fig|6666666.59341.peg.188	fig|6666666.59347.peg.4537	fig|6666666.59346.peg.4156	fig|6666666.59348.peg.1970	fig|6666666.59349.peg.1762	None	fig|6666666.59351.peg.2290	None	None	fig|6666666.59354.peg.491

2872	Rhodanese domain protein UPF0176	fig|6666666.59329.peg.5094	fig|6666666.59330.peg.2325	None	fig|6666666.59332.peg.3630	None	fig|6666666.59335.peg.2603	fig|6666666.59336.peg.1262	None	None	fig|6666666.59339.peg.2251	None	fig|6666666.59337.peg.3589	fig|6666666.59342.peg.5272	fig|6666666.71181.peg.1599	fig|6666666.59344.peg.2409	fig|6666666.59345.peg.1877	None	None	fig|6666666.59346.peg.2976	fig|6666666.59348.peg.4447	fig|6666666.59349.peg.363	fig|6666666.59350.peg.4210	fig|6666666.59351.peg.1053	fig|6666666.59353.peg.291	fig|6666666.59355.peg.1332	fig|6666666.59354.peg.4185

9483	Chromosome partition protein MukB	fig|6666666.59329.peg.3295	fig|6666666.59330.peg.2474	None	fig|6666666.59332.peg.3714	fig|6666666.59334.peg.588	fig|6666666.59335.peg.941	fig|6666666.59336.peg.6255	fig|6666666.59333.peg.1056	fig|6666666.59338.peg.776	fig|6666666.59339.peg.319	None	fig|6666666.59337.peg.4940	fig|6666666.59342.peg.5357	fig|6666666.71181.peg.4179	fig|6666666.59344.peg.2780	fig|6666666.59345.peg.1532	fig|6666666.59341.peg.2330	fig|6666666.59347.peg.2367	fig|6666666.59346.peg.2014	None	None	None	None	fig|6666666.59353.peg.1665	fig|6666666.59355.peg.1056	None

3339	Flagellin protein FlaG	fig|6666666.59329.peg.2520	fig|6666666.59330.peg.4629	fig|6666666.59331.peg.2107	fig|6666666.59332.peg.2463	None	fig|6666666.59335.peg.378	fig|6666666.59336.peg.1438	fig|6666666.59333.peg.5166	fig|6666666.59338.peg.5544	fig|6666666.59339.peg.7113	None	fig|6666666.59337.peg.2947	fig|6666666.59342.peg.4081	None	None	fig|6666666.59345.peg.5355	fig|6666666.59341.peg.5154	fig|6666666.59347.peg.2699	fig|6666666.59346.peg.4634	fig|6666666.59348.peg.2116	fig|6666666.59349.peg.740	None	fig|6666666.59351.peg.1997	None	None	fig|6666666.59354.peg.4128

2228	Predicted hydrolase of the alpha beta-hydrolase fold	fig|6666666.59329.peg.447	fig|6666666.59330.peg.1046	None	fig|6666666.59332.peg.1971	fig|6666666.59334.peg.2046	fig|6666666.59335.peg.5510	fig|6666666.59336.peg.1873	fig|6666666.59333.peg.4313	fig|6666666.59338.peg.1655	fig|6666666.59339.peg.7165	fig|6666666.59340.peg.1932	fig|6666666.59337.peg.1124	fig|6666666.59342.peg.5986	None	None	fig|6666666.59345.peg.3527	fig|6666666.59341.peg.4742	fig|6666666.59347.peg.2936	fig|6666666.59346.peg.1736	None	fig|6666666.59349.peg.2623	None	fig|6666666.59351.peg.4653	None	None	fig|6666666.59354.peg.5470

5714	Membrane-associated phospholipid phosphatase	fig|6666666.59329.peg.1696	fig|6666666.59330.peg.805	fig|6666666.59331.peg.2272	fig|6666666.59332.peg.5415	None	fig|6666666.59335.peg.5066	fig|6666666.59336.peg.2912	fig|6666666.59333.peg.119	fig|6666666.59338.peg.63	fig|6666666.59339.peg.7327	None	fig|6666666.59337.peg.1398	fig|6666666.59342.peg.1946	None	None	fig|6666666.59345.peg.3885	fig|6666666.59341.peg.1134	fig|6666666.59347.peg.3074	fig|6666666.59346.peg.809	fig|6666666.59348.peg.1184	fig|6666666.59349.peg.503	None	fig|6666666.59351.peg.3826	None	None	fig|6666666.59354.peg.950

1219	General secretion pathway protein J	fig|6666666.59329.peg.1226	fig|6666666.59330.peg.423	fig|6666666.59331.peg.4621	fig|6666666.59332.peg.2995	fig|6666666.59334.peg.122	fig|6666666.59335.peg.712	fig|6666666.59336.peg.3116	fig|6666666.59333.peg.511	fig|6666666.59338.peg.1433	fig|6666666.59339.peg.6207	None	fig|6666666.59337.peg.4249	None	None	None	fig|6666666.59345.peg.2671	fig|6666666.59341.peg.4542	fig|6666666.59347.peg.4469	fig|6666666.59346.peg.2226	fig|6666666.59348.peg.4326	fig|6666666.59349.peg.2169	None	fig|6666666.59351.peg.4341	None	None	fig|6666666.59354.peg.703

8814	GTPase EC 3.6.1.-	fig|6666666.59329.peg.3090	fig|6666666.59330.peg.735	None	fig|6666666.59332.peg.3046	fig|6666666.59334.peg.2881	fig|6666666.59335.peg.4662	fig|6666666.59336.peg.2846	fig|6666666.59333.peg.2246	fig|6666666.59338.peg.2493	fig|6666666.59339.peg.8384	None	fig|6666666.59337.peg.3762	fig|6666666.59342.peg.1881	fig|6666666.71181.peg.1447	fig|6666666.59344.peg.1535	fig|6666666.59345.peg.2789	fig|6666666.59341.peg.2253	fig|6666666.59347.peg.433	fig|6666666.59346.peg.1339	None	None	None	None	fig|6666666.59353.peg.4363	fig|6666666.59355.peg.4480	None

4159	F WD40 repeat	None	fig|6666666.59330.peg.4077	fig|6666666.59331.peg.2368	fig|6666666.59332.peg.1072	None	fig|6666666.59335.peg.6124	fig|6666666.59336.peg.2185	fig|6666666.59333.peg.3108	fig|6666666.59338.peg.4001	fig|6666666.59339.peg.8867	fig|6666666.59340.peg.2694	fig|6666666.59337.peg.1818	fig|6666666.59342.peg.5616	None	None	fig|6666666.59345.peg.5557	fig|6666666.59341.peg.5785	fig|6666666.59347.peg.5337	fig|6666666.59346.peg.39	fig|6666666.59348.peg.3297	fig|6666666.59349.peg.2353	None	fig|6666666.59351.peg.4179	None	None	fig|6666666.59354.peg.3809

5906	Outer membrane lipoprotein	fig|6666666.59329.peg.3850	fig|6666666.59330.peg.746	fig|6666666.59331.peg.4773	fig|6666666.59332.peg.3681	fig|6666666.59334.peg.5245	fig|6666666.59335.peg.1932	fig|6666666.59336.peg.2758	fig|6666666.59333.peg.2429	fig|6666666.59338.peg.1486	fig|6666666.59339.peg.8404	None	fig|6666666.59337.peg.3475	fig|6666666.59342.peg.5069	fig|6666666.71181.peg.2555	fig|6666666.59344.peg.741	fig|6666666.59345.peg.2778	fig|6666666.59341.peg.2262	fig|6666666.59347.peg.441	fig|6666666.59346.peg.4825	fig|6666666.59348.peg.2765	None	None	None	None	None	None

750	YcfP protein probably an esterase that is part of a salvage cluster	fig|6666666.59329.peg.5645	fig|6666666.59330.peg.1241	fig|6666666.59331.peg.5141	fig|6666666.59332.peg.4194	fig|6666666.59334.peg.5216	fig|6666666.59335.peg.1313	fig|6666666.59336.peg.1622	fig|6666666.59333.peg.1874	fig|6666666.59338.peg.5907	fig|6666666.59339.peg.3448	None	fig|6666666.59337.peg.2184	fig|6666666.59342.peg.495	fig|6666666.71181.peg.1642	fig|6666666.59344.peg.5016	fig|6666666.59345.peg.1603	fig|6666666.59341.peg.267	fig|6666666.59347.peg.4072	None	None	None	None	None	fig|6666666.59353.peg.335	fig|6666666.59355.peg.1374	None

5960	membrane protein	fig|6666666.59329.peg.5404	fig|6666666.59330.peg.303	None	fig|6666666.59332.peg.2213	fig|6666666.59334.peg.1420	fig|6666666.59335.peg.852	fig|6666666.59336.peg.3734	fig|6666666.59333.peg.4900	fig|6666666.59338.peg.1592	fig|6666666.59339.peg.3211	fig|6666666.59340.peg.2703	fig|6666666.59337.peg.1032	None	None	None	fig|6666666.59345.peg.4251	fig|6666666.59341.peg.3787	fig|6666666.59347.peg.2652	fig|6666666.59346.peg.2668	fig|6666666.59348.peg.5145	fig|6666666.59349.peg.1608	None	fig|6666666.59351.peg.1034	None	None	fig|6666666.59354.peg.6477

2591	FIG01201502 hypothetical protein	fig|6666666.59329.peg.1183	fig|6666666.59330.peg.4274	fig|6666666.59331.peg.2928	fig|6666666.59332.peg.4786	None	fig|6666666.59335.peg.1669	fig|6666666.59336.peg.1461	fig|6666666.59333.peg.4526	fig|6666666.59338.peg.3107	fig|6666666.59339.peg.3693	None	fig|6666666.59337.peg.3567	fig|6666666.59342.peg.1176	None	None	fig|6666666.59345.peg.842	fig|6666666.59341.peg.2872	fig|6666666.59347.peg.1152	fig|6666666.59346.peg.3250	fig|6666666.59348.peg.4727	fig|6666666.59349.peg.831	None	fig|6666666.59351.peg.3377	None	None	fig|6666666.59354.peg.4303

2627	FIG01199732 hypothetical protein	None	fig|6666666.59330.peg.4474	None	fig|6666666.59332.peg.3085	fig|6666666.59334.peg.3381	fig|6666666.59335.peg.357	fig|6666666.59336.peg.2161	fig|6666666.59333.peg.3552	fig|6666666.59338.peg.5358	fig|6666666.59339.peg.7137	None	fig|6666666.59337.peg.5643	fig|6666666.59342.peg.2565	fig|6666666.71181.peg.4364	fig|6666666.59344.peg.5665	fig|6666666.59345.peg.2147	fig|6666666.59341.peg.1684	fig|6666666.59347.peg.2842	fig|6666666.59346.peg.3542	fig|6666666.59348.peg.2634	None	None	None	fig|6666666.59353.peg.4915	fig|6666666.59355.peg.4985	None

11059	Cobyric acid synthase	fig|6666666.59329.peg.4415	fig|6666666.59330.peg.784	fig|6666666.59331.peg.3177	fig|6666666.59332.peg.2424	None	fig|6666666.59335.peg.545	None	fig|6666666.59333.peg.1521	fig|6666666.59338.peg.3678	fig|6666666.59339.peg.8419	None	fig|6666666.59337.peg.3814	fig|6666666.59342.peg.941	None	None	fig|6666666.59345.peg.57	fig|6666666.59341.peg.2236	fig|6666666.59347.peg.917	fig|6666666.59346.peg.1221	fig|6666666.59348.peg.4933	fig|6666666.59349.peg.4236	fig|6666666.59350.peg.3213	fig|6666666.59351.peg.3915	None	None	fig|6666666.59354.peg.2755

7435	Translation initiation factor SUI1-related protein	fig|6666666.59329.peg.5855	fig|6666666.59330.peg.4340	None	None	None	fig|6666666.59335.peg.568	fig|6666666.59336.peg.2245	fig|6666666.59333.peg.4300	fig|6666666.59338.peg.5426	fig|6666666.59339.peg.2933	None	fig|6666666.59337.peg.3025	fig|6666666.59342.peg.2287	fig|6666666.71181.peg.4200	fig|6666666.59344.peg.493	fig|6666666.59345.peg.80	fig|6666666.59341.peg.5836	fig|6666666.59347.peg.961	None	fig|6666666.59348.peg.5283	fig|6666666.59349.peg.2712	None	None	fig|6666666.59353.peg.1809	fig|6666666.59355.peg.1207	fig|6666666.59354.peg.5439

4686	Phosphodiesterase yfcE EC 3.1.4.-	fig|6666666.59329.peg.4688	fig|6666666.59330.peg.2197	fig|6666666.59331.peg.3020	None	fig|6666666.59334.peg.5430	fig|6666666.59335.peg.2425	fig|6666666.59336.peg.2555	fig|6666666.59333.peg.2059	fig|6666666.59338.peg.3601	fig|6666666.59339.peg.5257	None	None	fig|6666666.59342.peg.4087	fig|6666666.71181.peg.562	fig|6666666.59344.peg.1902	fig|6666666.59345.peg.4355	fig|6666666.59341.peg.1629	fig|6666666.59347.peg.4441	fig|6666666.59346.peg.3677	fig|6666666.59348.peg.1275	None	None	None	fig|6666666.59353.peg.1194	fig|6666666.59355.peg.283	None

10678	Phosphonate ABC transporter phosphate-binding periplasmic component TC 3.A.1.9.1	None	None	fig|6666666.59331.peg.4304	fig|6666666.59332.peg.4863	None	fig|6666666.59335.peg.6098	None	fig|6666666.59333.peg.2387	fig|6666666.59338.peg.827	fig|6666666.59339.peg.7007	fig|6666666.59340.peg.3358	fig|6666666.59337.peg.3227	None	fig|6666666.71181.peg.1126	fig|6666666.59344.peg.2246	None	fig|6666666.59341.peg.4664	fig|6666666.59347.peg.342	fig|6666666.59346.peg.478	fig|6666666.59348.peg.4220	fig|6666666.59349.peg.4602	fig|6666666.59350.peg.4077	None	fig|6666666.59353.peg.4964	fig|6666666.59355.peg.5048	fig|6666666.59354.peg.3409

10298	Thiol-disulfide isomerase and thioredoxin	fig|6666666.59329.peg.5793	fig|6666666.59330.peg.3165	fig|6666666.59331.peg.1502	fig|6666666.59332.peg.4929	fig|6666666.59334.peg.1334	fig|6666666.59335.peg.1226	fig|6666666.59336.peg.5250	fig|6666666.59333.peg.2799	fig|6666666.59338.peg.2750	fig|6666666.59339.peg.1518	None	fig|6666666.59337.peg.4555	fig|6666666.59342.peg.5214	None	None	fig|6666666.59345.peg.3254	fig|6666666.59341.peg.910	fig|6666666.59347.peg.1766	fig|6666666.59346.peg.2054	fig|6666666.59348.peg.1676	fig|6666666.59349.peg.2503	None	None	None	None	fig|6666666.59354.peg.717

9685	FIG01199643 hypothetical protein	None	fig|6666666.59330.peg.1023	fig|6666666.59331.peg.1558	fig|6666666.59332.peg.4226	fig|6666666.59334.peg.5213	fig|6666666.59335.peg.3362	fig|6666666.59336.peg.4478	fig|6666666.59333.peg.5430	fig|6666666.59338.peg.2716	fig|6666666.59339.peg.1548	None	fig|6666666.59337.peg.3164	fig|6666666.59342.peg.2353	None	None	fig|6666666.59345.peg.3268	fig|6666666.59341.peg.4164	fig|6666666.59347.peg.1782	fig|6666666.59346.peg.3917	fig|6666666.59348.peg.4363	fig|6666666.59349.peg.365	None	fig|6666666.59351.peg.1055	None	None	fig|6666666.59354.peg.4432

8321	Serine threonine protein kinase	fig|6666666.59329.peg.6387	fig|6666666.59330.peg.2164	fig|6666666.59331.peg.2728	fig|6666666.59332.peg.5878	fig|6666666.59334.peg.1840	fig|6666666.59335.peg.2684	fig|6666666.59336.peg.3821	fig|6666666.59333.peg.4572	fig|6666666.59338.peg.6135	fig|6666666.59339.peg.2714	None	fig|6666666.59337.peg.5012	fig|6666666.59342.peg.2558	None	None	fig|6666666.59345.peg.4121	fig|6666666.59341.peg.1389	fig|6666666.59347.peg.1017	fig|6666666.59346.peg.3960	fig|6666666.59348.peg.5160	None	None	fig|6666666.59351.peg.5349	None	None	fig|6666666.59354.peg.3847

5328	FIG01199806 hypothetical protein	None	fig|6666666.59330.peg.3162	None	None	fig|6666666.59334.peg.3624	fig|6666666.59335.peg.1230	fig|6666666.59336.peg.6511	fig|6666666.59333.peg.5095	fig|6666666.59338.peg.5655	fig|6666666.59339.peg.1522	None	fig|6666666.59337.peg.4553	None	fig|6666666.71181.peg.4771	fig|6666666.59344.peg.1175	fig|6666666.59345.peg.3256	fig|6666666.59341.peg.906	fig|6666666.59347.peg.1769	fig|6666666.59346.peg.2052	fig|6666666.59348.peg.1673	fig|6666666.59349.peg.3358	None	fig|6666666.59351.peg.3164	fig|6666666.59353.peg.1681	fig|6666666.59355.peg.1039	None

7891	FIG01200900 hypothetical protein	fig|6666666.59329.peg.117	fig|6666666.59330.peg.1729	None	fig|6666666.59332.peg.3053	fig|6666666.59334.peg.4722	fig|6666666.59335.peg.809	fig|6666666.59336.peg.5038	fig|6666666.59333.peg.3408	fig|6666666.59338.peg.3478	fig|6666666.59339.peg.5002	fig|6666666.59340.peg.113	fig|6666666.59337.peg.99	fig|6666666.59342.peg.1357	None	None	fig|6666666.59345.peg.3744	fig|6666666.59341.peg.3482	fig|6666666.59347.peg.3054	fig|6666666.59346.peg.4039	fig|6666666.59348.peg.4178	fig|6666666.59349.peg.4231	None	fig|6666666.59351.peg.3911	None	None	None

116	Cell wall-associated hydrolase	fig|6666666.59329.peg.1738	fig|6666666.59330.peg.4922	fig|6666666.59331.peg.5938	None	fig|6666666.59334.peg.4876	fig|6666666.59335.peg.3863	fig|6666666.59336.peg.5534	fig|6666666.59333.peg.3698	None	fig|6666666.59339.peg.1184	None	fig|6666666.59337.peg.2701	fig|6666666.59342.peg.2244	fig|6666666.71181.peg.477	fig|6666666.59344.peg.1685	fig|6666666.59345.peg.3178	fig|6666666.59341.peg.3615	fig|6666666.59347.peg.1811	fig|6666666.59346.peg.3690	None	None	fig|6666666.59350.peg.5934	None	fig|6666666.59353.peg.1323	fig|6666666.59355.peg.2548	None

9251	Na+ H+ antiporter NhaD type	fig|6666666.59329.peg.2364	fig|6666666.59330.peg.1490	fig|6666666.59331.peg.678	fig|6666666.59332.peg.966	None	fig|6666666.59335.peg.5785	fig|6666666.59336.peg.1170	fig|6666666.59333.peg.1732	fig|6666666.59338.peg.6162	fig|6666666.59339.peg.5646	None	fig|6666666.59337.peg.186	None	None	None	fig|6666666.59345.peg.4516	fig|6666666.59341.peg.315	fig|6666666.59347.peg.3684	fig|6666666.59346.peg.2585	fig|6666666.59348.peg.4714	fig|6666666.59349.peg.1887	None	fig|6666666.59351.peg.3662	None	None	fig|6666666.59354.peg.1376

4591	FIG01201264 hypothetical protein	None	fig|6666666.59330.peg.4541	fig|6666666.59331.peg.2171	None	fig|6666666.59334.peg.2259	fig|6666666.59335.peg.4245	fig|6666666.59336.peg.6304	fig|6666666.59333.peg.4641	None	fig|6666666.59339.peg.1708	fig|6666666.59340.peg.1802	fig|6666666.59337.peg.1547	None	fig|6666666.71181.peg.667	fig|6666666.59344.peg.4120	fig|6666666.59345.peg.3061	fig|6666666.59341.peg.4787	fig|6666666.59347.peg.1527	fig|6666666.59346.peg.2994	fig|6666666.59348.peg.4837	None	None	None	fig|6666666.59353.peg.4101	fig|6666666.59355.peg.2338	None

8293	FIG01199743 hypothetical protein	fig|6666666.59329.peg.4279	fig|6666666.59330.peg.479	fig|6666666.59331.peg.2596	fig|6666666.59332.peg.980	fig|6666666.59334.peg.1029	fig|6666666.59335.peg.3905	fig|6666666.59336.peg.5748	fig|6666666.59333.peg.2230	fig|6666666.59338.peg.4165	fig|6666666.59339.peg.7182	fig|6666666.59340.peg.1143	fig|6666666.59337.peg.1571	fig|6666666.59342.peg.3472	None	None	fig|6666666.59345.peg.2211	fig|6666666.59341.peg.2605	fig|6666666.59347.peg.2776	fig|6666666.59346.peg.3524	fig|6666666.59348.peg.3851	None	None	None	None	None	None

10574	Multicopper oxidase	fig|6666666.59329.peg.3962	fig|6666666.59330.peg.974	fig|6666666.59331.peg.1571	fig|6666666.59332.peg.1928	None	fig|6666666.59335.peg.1	fig|6666666.59336.peg.172	fig|6666666.59333.peg.3924	None	fig|6666666.59339.peg.7524	None	fig|6666666.59337.peg.2556	None	fig|6666666.71181.peg.5563	fig|6666666.59344.peg.5608	fig|6666666.59345.peg.5548	fig|6666666.59341.peg.5655	None	fig|6666666.59346.peg.937	fig|6666666.59348.peg.1095	None	fig|6666666.59350.peg.5656	None	fig|6666666.59353.peg.4931	fig|6666666.59355.peg.3086	None

9758	Putative signal peptide protein	fig|6666666.59329.peg.369	fig|6666666.59330.peg.990	None	fig|6666666.59332.peg.264	fig|6666666.59334.peg.2971	fig|6666666.59335.peg.5433	fig|6666666.59336.peg.189	fig|6666666.59333.peg.1578	fig|6666666.59338.peg.2868	fig|6666666.59339.peg.7650	fig|6666666.59340.peg.2242	fig|6666666.59337.peg.2541	fig|6666666.59342.peg.4056	None	None	fig|6666666.59345.peg.5533	fig|6666666.59341.peg.4842	fig|6666666.59347.peg.5243	fig|6666666.59346.peg.921	fig|6666666.59348.peg.49	None	fig|6666666.59350.peg.6851	None	None	None	None

8845	Flagellar protein FlgO	fig|6666666.59329.peg.569	fig|6666666.59330.peg.2697	None	fig|6666666.59332.peg.2632	None	fig|6666666.59335.peg.2073	fig|6666666.59336.peg.3753	fig|6666666.59333.peg.204	fig|6666666.59338.peg.2320	None	fig|6666666.59340.peg.3723	fig|6666666.59337.peg.3339	fig|6666666.59342.peg.5346	None	None	fig|6666666.59345.peg.5442	fig|6666666.59341.peg.4561	fig|6666666.59347.peg.2721	fig|6666666.59346.peg.4726	fig|6666666.59348.peg.317	fig|6666666.59349.peg.710	None	fig|6666666.59351.peg.2570	None	None	fig|6666666.59354.peg.4319

1549	Probable transmembrane protein	fig|6666666.59329.peg.3105	fig|6666666.59330.peg.1093	fig|6666666.59331.peg.6249	fig|6666666.59332.peg.74	fig|6666666.59334.peg.148	fig|6666666.59335.peg.3921	fig|6666666.59336.peg.5629	fig|6666666.59333.peg.3963	fig|6666666.59338.peg.5331	fig|6666666.59339.peg.8050	fig|6666666.59340.peg.978	fig|6666666.59337.peg.865	fig|6666666.59342.peg.2612	None	None	fig|6666666.59345.peg.4988	fig|6666666.59341.peg.5111	fig|6666666.59347.peg.4806	fig|6666666.59346.peg.4288	fig|6666666.59348.peg.1210	None	None	None	None	None	None

9521	FIG01201286 hypothetical protein	fig|6666666.59329.peg.3025	fig|6666666.59330.peg.3939	None	fig|6666666.59332.peg.3325	fig|6666666.59334.peg.2433	fig|6666666.59335.peg.5078	fig|6666666.59336.peg.4724	fig|6666666.59333.peg.3562	fig|6666666.59338.peg.5925	fig|6666666.59339.peg.4042	None	fig|6666666.59337.peg.4950	fig|6666666.59342.peg.1404	None	None	fig|6666666.59345.peg.4085	fig|6666666.59341.peg.4722	fig|6666666.59347.peg.1800	None	fig|6666666.59348.peg.3579	fig|6666666.59349.peg.3156	None	fig|6666666.59351.peg.3081	None	None	fig|6666666.59354.peg.2836

8133	Predicted polymerase, ortholog to Borrelia burgdorferi BB0267	fig|6666666.59329.peg.1704	fig|6666666.59330.peg.1898	fig|6666666.59331.peg.2058	fig|6666666.59332.peg.441	None	fig|6666666.59335.peg.2593	fig|6666666.59336.peg.3947	fig|6666666.59333.peg.4400	fig|6666666.59338.peg.1371	fig|6666666.59339.peg.5486	fig|6666666.59340.peg.301	fig|6666666.59337.peg.246	None	None	None	fig|6666666.59345.peg.5254	fig|6666666.59341.peg.1385	fig|6666666.59347.peg.17	fig|6666666.59346.peg.2556	fig|6666666.59348.peg.2908	fig|6666666.59349.peg.2330	None	fig|6666666.59351.peg.4907	None	None	None

10531	L-lactate dehydrogenase EC 1.1.2.3	fig|6666666.59329.peg.4457	fig|6666666.59330.peg.3961	None	fig|6666666.59332.peg.1783	fig|6666666.59334.peg.4336	fig|6666666.59335.peg.5283	fig|6666666.59336.peg.4201	fig|6666666.59333.peg.3626	None	fig|6666666.59339.peg.2611	None	fig|6666666.59337.peg.1922	fig|6666666.59342.peg.5957	fig|6666666.71181.peg.126	fig|6666666.59344.peg.3648	fig|6666666.59345.peg.2819	None	None	fig|6666666.59346.peg.3624	fig|6666666.59348.peg.4182	None	fig|6666666.59350.peg.5653	None	fig|6666666.59353.peg.3864	fig|6666666.59355.peg.5803	None

7777	LSU ribosomal protein L36p	None	fig|6666666.59330.peg.482	None	fig|6666666.59332.peg.976	fig|6666666.59334.peg.1545	None	fig|6666666.59336.peg.5787	None	fig|6666666.59338.peg.1209	None	fig|6666666.59340.peg.311	fig|6666666.59337.peg.1568	None	fig|6666666.71181.peg.2082	fig|6666666.59344.peg.1031	None	None	fig|6666666.59347.peg.283	fig|6666666.59346.peg.3529	fig|6666666.59348.peg.1150	fig|6666666.59349.peg.4609	fig|6666666.59350.peg.3111	fig|6666666.59351.peg.4604	fig|6666666.59353.peg.723	fig|6666666.59355.peg.983	fig|6666666.59354.peg.2911

212	Potential queD like	fig|6666666.59329.peg.4104	fig|6666666.59330.peg.3835	fig|6666666.59331.peg.3058	fig|6666666.59332.peg.4239	None	fig|6666666.59335.peg.4801	fig|6666666.59336.peg.6046	fig|6666666.59333.peg.4971	fig|6666666.59338.peg.4506	fig|6666666.59339.peg.994	None	fig|6666666.59337.peg.2017	fig|6666666.59342.peg.5253	None	None	fig|6666666.59345.peg.3084	fig|6666666.59341.peg.738	fig|6666666.59347.peg.3430	fig|6666666.59346.peg.1457	None	fig|6666666.59349.peg.1667	None	fig|6666666.59351.peg.289	None	None	fig|6666666.59354.peg.816

630	Possible membrane protein	fig|6666666.59329.peg.747	None	fig|6666666.59331.peg.93	fig|6666666.59332.peg.5255	fig|6666666.59334.peg.4095	fig|6666666.59335.peg.679	fig|6666666.59336.peg.5702	fig|6666666.59333.peg.1410	fig|6666666.59338.peg.4577	fig|6666666.59339.peg.1779	fig|6666666.59340.peg.1769	None	fig|6666666.59342.peg.2655	None	None	fig|6666666.59345.peg.780	fig|6666666.59341.peg.2960	fig|6666666.59347.peg.1080	fig|6666666.59346.peg.413	fig|6666666.59348.peg.2703	None	fig|6666666.59350.peg.6638	fig|6666666.59351.peg.2438	None	None	None

9731	Putative inner membrane protein YjeT clustered with HflC	fig|6666666.59329.peg.5157	fig|6666666.59330.peg.1433	None	None	fig|6666666.59334.peg.4994	fig|6666666.59335.peg.6127	fig|6666666.59336.peg.3056	fig|6666666.59333.peg.291	fig|6666666.59338.peg.1007	fig|6666666.59339.peg.8865	fig|6666666.59340.peg.1201	fig|6666666.59337.peg.1787	None	fig|6666666.71181.peg.4630	fig|6666666.59344.peg.5889	fig|6666666.59345.peg.5585	fig|6666666.59341.peg.4276	fig|6666666.59347.peg.5343	None	fig|6666666.59348.peg.3299	None	None	None	fig|6666666.59353.peg.4986	fig|6666666.59355.peg.5069	None

7750	Oxaloacetate decarboxylase gamma chain EC 4.1.1.3	fig|6666666.59329.peg.5635	fig|6666666.59330.peg.2825	fig|6666666.59331.peg.5856	fig|6666666.59332.peg.6285	fig|6666666.59334.peg.247	fig|6666666.59335.peg.5713	fig|6666666.59336.peg.2513	None	fig|6666666.59338.peg.755	fig|6666666.59339.peg.8926	None	fig|6666666.59337.peg.2027	fig|6666666.59342.peg.2228	None	None	fig|6666666.59345.peg.2449	fig|6666666.59341.peg.5656	fig|6666666.59347.peg.3223	None	fig|6666666.59348.peg.4868	fig|6666666.59349.peg.1835	None	fig|6666666.59351.peg.5268	None	None	fig|6666666.59354.peg.593

8693	Aspartate racemase EC 5.1.1.13	None	None	fig|6666666.59331.peg.5363	fig|6666666.59332.peg.5506	fig|6666666.59334.peg.1185	fig|6666666.59335.peg.1376	fig|6666666.59336.peg.1554	fig|6666666.59333.peg.2770	fig|6666666.59338.peg.2889	fig|6666666.59339.peg.2770	None	None	None	fig|6666666.71181.peg.1547	fig|6666666.59344.peg.2048	fig|6666666.59345.peg.4796	fig|6666666.59341.peg.1585	fig|6666666.59347.peg.674	None	fig|6666666.59348.peg.3476	None	fig|6666666.59350.peg.5384	fig|6666666.59351.peg.1831	fig|6666666.59353.peg.2884	fig|6666666.59355.peg.2854	None

4936	Thiopurine S-methyltransferase EC 2.1.1.67	fig|6666666.59329.peg.5265	fig|6666666.59330.peg.3373	fig|6666666.59331.peg.5475	fig|6666666.59332.peg.5254	None	fig|6666666.59335.peg.5220	fig|6666666.59336.peg.5899	fig|6666666.59333.peg.539	fig|6666666.59338.peg.3303	fig|6666666.59339.peg.3885	None	fig|6666666.59337.peg.4526	fig|6666666.59342.peg.388	None	None	fig|6666666.59345.peg.4662	fig|6666666.59341.peg.446	fig|6666666.59347.peg.1874	fig|6666666.59346.peg.2924	fig|6666666.59348.peg.4373	fig|6666666.59349.peg.4835	None	None	None	None	fig|6666666.59354.peg.4379

4063	lipopeptide	fig|6666666.59329.peg.91	fig|6666666.59330.peg.3051	None	fig|6666666.59332.peg.1706	None	fig|6666666.59335.peg.3335	fig|6666666.59336.peg.1118	fig|6666666.59333.peg.789	fig|6666666.59338.peg.1780	fig|6666666.59339.peg.8522	fig|6666666.59340.peg.792	fig|6666666.59337.peg.1616	fig|6666666.59342.peg.3157	None	None	fig|6666666.59345.peg.5503	fig|6666666.59341.peg.3918	fig|6666666.59347.peg.5077	fig|6666666.59346.peg.599	fig|6666666.59348.peg.3773	fig|6666666.59349.peg.1558	None	None	None	None	fig|6666666.59354.peg.1456

2822	S-ribosylhomocysteine lyase EC 4.4.1.21 Autoinducer-2 production protein LuxS	fig|6666666.59329.peg.332	fig|6666666.59330.peg.2839	None	fig|6666666.59332.peg.6003	fig|6666666.59334.peg.258	fig|6666666.59335.peg.3982	None	fig|6666666.59333.peg.5320	fig|6666666.59338.peg.446	fig|6666666.59339.peg.8907	None	fig|6666666.59337.peg.2036	None	fig|6666666.71181.peg.3464	fig|6666666.59344.peg.2160	fig|6666666.59345.peg.2438	fig|6666666.59341.peg.2571	fig|6666666.59347.peg.3216	fig|6666666.59346.peg.564	fig|6666666.59348.peg.3601	None	None	None	fig|6666666.59353.peg.237	fig|6666666.59355.peg.3008	None

6920	FIG01200042 hypothetical protein	fig|6666666.59329.peg.3966	fig|6666666.59330.peg.1267	fig|6666666.59331.peg.4114	fig|6666666.59332.peg.5858	fig|6666666.59334.peg.3307	fig|6666666.59335.peg.4091	fig|6666666.59336.peg.460	fig|6666666.59333.peg.5165	fig|6666666.59338.peg.5782	fig|6666666.59339.peg.3501	fig|6666666.59340.peg.2326	fig|6666666.59337.peg.3091	fig|6666666.59342.peg.1084	None	None	fig|6666666.59345.peg.4165	fig|6666666.59341.peg.3539	fig|6666666.59347.peg.4177	fig|6666666.59346.peg.4045	fig|6666666.59348.peg.1012	None	None	None	None	None	None

3624	putative exported protein	fig|6666666.59329.peg.5741	fig|6666666.59330.peg.2303	fig|6666666.59331.peg.3397	fig|6666666.59332.peg.2677	fig|6666666.59334.peg.3632	fig|6666666.59335.peg.4608	fig|6666666.59336.peg.4390	fig|6666666.59333.peg.5592	fig|6666666.59338.peg.4099	fig|6666666.59339.peg.3739	None	fig|6666666.59337.peg.1772	fig|6666666.59342.peg.2335	None	None	fig|6666666.59345.peg.872	fig|6666666.59341.peg.4885	fig|6666666.59347.peg.2243	fig|6666666.59346.peg.1680	fig|6666666.59348.peg.5490	None	fig|6666666.59350.peg.1275	None	None	None	None

4956	hypothetical protein	fig|6666666.59329.peg.6112	fig|6666666.59330.peg.4717	fig|6666666.59331.peg.5111	None	fig|6666666.59334.peg.5423	fig|6666666.59335.peg.4824	fig|6666666.59336.peg.5399	None	fig|6666666.59338.peg.4720	fig|6666666.59339.peg.1256	fig|6666666.59340.peg.2636	fig|6666666.59337.peg.4042	fig|6666666.59342.peg.3064	None	None	fig|6666666.59345.peg.232	fig|6666666.59341.peg.6048	fig|6666666.59347.peg.594	None	None	fig|6666666.59349.peg.4490	fig|6666666.59350.peg.2798	fig|6666666.59351.peg.97	None	None	fig|6666666.59354.peg.1896

4398	Melibiose carrier protein, Na+ melibiose symporter	fig|6666666.59329.peg.1412	None	fig|6666666.59331.peg.893	fig|6666666.59332.peg.3447	None	fig|6666666.59335.peg.3268	fig|6666666.59336.peg.5155	None	fig|6666666.59338.peg.3072	fig|6666666.59339.peg.6836	None	fig|6666666.59337.peg.2898	fig|6666666.59342.peg.4875	fig|6666666.71181.peg.1850	fig|6666666.59344.peg.4409	fig|6666666.59345.peg.2139	fig|6666666.59341.peg.3426	fig|6666666.59347.peg.155	fig|6666666.59346.peg.3972	fig|6666666.59348.peg.5227	None	None	None	fig|6666666.59353.peg.3215	fig|6666666.59355.peg.2136	None

10144	Chitinase EC 3.2.1.14	fig|6666666.59329.peg.2141	fig|6666666.59330.peg.4315	None	fig|6666666.59332.peg.4933	None	fig|6666666.59335.peg.1966	fig|6666666.59336.peg.5153	fig|6666666.59333.peg.2156	fig|6666666.59338.peg.2324	fig|6666666.59339.peg.6990	fig|6666666.59340.peg.109	fig|6666666.59337.peg.765	None	None	None	fig|6666666.59345.peg.3627	fig|6666666.59341.peg.6000	fig|6666666.59347.peg.5208	fig|6666666.59346.peg.3212	fig|6666666.59348.peg.2035	fig|6666666.59349.peg.2348	None	fig|6666666.59351.peg.1212	None	None	fig|6666666.59354.peg.3804

1810	Dihydroorotase EC 3.5.2.3	fig|6666666.59329.peg.4272	fig|6666666.59330.peg.4089	fig|6666666.59331.peg.2165	fig|6666666.59332.peg.4290	fig|6666666.59334.peg.4112	None	fig|6666666.59336.peg.3251	fig|6666666.59333.peg.3495	fig|6666666.59338.peg.1945	fig|6666666.59339.peg.6777	None	fig|6666666.59337.peg.2142	fig|6666666.59342.peg.2260	None	None	fig|6666666.59345.peg.3954	fig|6666666.59341.peg.5574	None	fig|6666666.59346.peg.2144	None	fig|6666666.59349.peg.3523	fig|6666666.59350.peg.1241	fig|6666666.59351.peg.3354	None	None	fig|6666666.59354.peg.4609

2215	Type cbb3 cytochrome oxidase biogenesis protein CcoS, involved in heme b insertion	fig|6666666.59329.peg.1345	fig|6666666.59330.peg.3194	fig|6666666.59331.peg.2238	None	fig|6666666.59334.peg.987	fig|6666666.59335.peg.3533	fig|6666666.59336.peg.5823	fig|6666666.59333.peg.5338	fig|6666666.59338.peg.4062	fig|6666666.59339.peg.1321	None	fig|6666666.59337.peg.3721	fig|6666666.59342.peg.594	None	None	fig|6666666.59345.peg.3230	fig|6666666.59341.peg.5608	fig|6666666.59347.peg.1739	fig|6666666.59346.peg.1529	None	fig|6666666.59349.peg.142	None	fig|6666666.59351.peg.829	None	None	fig|6666666.59354.peg.197

1951	FIG01199542 hypothetical protein	fig|6666666.59329.peg.4763	fig|6666666.59330.peg.3912	fig|6666666.59331.peg.4816	fig|6666666.59332.peg.4423	fig|6666666.59334.peg.2937	fig|6666666.59335.peg.2013	None	fig|6666666.59333.peg.3781	fig|6666666.59338.peg.5797	fig|6666666.59339.peg.3951	None	fig|6666666.59337.peg.4715	None	None	None	fig|6666666.59345.peg.3128	fig|6666666.59341.peg.728	fig|6666666.59347.peg.3440	fig|6666666.59346.peg.1448	fig|6666666.59348.peg.2880	fig|6666666.59349.peg.4736	None	fig|6666666.59351.peg.957	None	None	fig|6666666.59354.peg.4547

519	Heavy-metal-associated domain N-terminus and membrane-bounded cytochrome biogenesis cycZ-like domain, possible membrane copper tolerance protein	fig|6666666.59329.peg.1346	fig|6666666.59330.peg.3195	None	fig|6666666.59332.peg.5966	fig|6666666.59334.peg.986	fig|6666666.59335.peg.3532	None	fig|6666666.59333.peg.4753	fig|6666666.59338.peg.4061	fig|6666666.59339.peg.1320	None	fig|6666666.59337.peg.3720	fig|6666666.59342.peg.595	None	None	fig|6666666.59345.peg.3229	fig|6666666.59341.peg.5607	fig|6666666.59347.peg.1738	fig|6666666.59346.peg.1528	fig|6666666.59348.peg.5394	fig|6666666.59349.peg.143	None	fig|6666666.59351.peg.828	None	None	fig|6666666.59354.peg.196

4572	FIG01200668 hypothetical protein	None	fig|6666666.59330.peg.1945	fig|6666666.59331.peg.2370	None	fig|6666666.59334.peg.4377	fig|6666666.59335.peg.1801	fig|6666666.59336.peg.3785	fig|6666666.59333.peg.2520	fig|6666666.59338.peg.3192	fig|6666666.59339.peg.3611	None	fig|6666666.59337.peg.5454	None	None	None	fig|6666666.59345.peg.718	fig|6666666.59341.peg.3826	fig|6666666.59347.peg.1687	fig|6666666.59346.peg.2420	fig|6666666.59348.peg.4268	fig|6666666.59349.peg.911	fig|6666666.59350.peg.3337	fig|6666666.59351.peg.7	None	None	fig|6666666.59354.peg.3468

4605	Uncharacterized protein conserved in bacteria	fig|6666666.59329.peg.2972	fig|6666666.59330.peg.4539	fig|6666666.59331.peg.394	fig|6666666.59332.peg.4112	None	fig|6666666.59335.peg.4340	fig|6666666.59336.peg.6023	fig|6666666.59333.peg.4697	None	fig|6666666.59339.peg.4359	fig|6666666.59340.peg.570	fig|6666666.59337.peg.1544	fig|6666666.59342.peg.6017	None	fig|6666666.59344.peg.5174	fig|6666666.59345.peg.3059	fig|6666666.59341.peg.3574	fig|6666666.59347.peg.216	fig|6666666.59346.peg.4385	fig|6666666.59348.peg.2514	None	fig|6666666.59350.peg.247	None	None	None	None

1854	FIGfam010717	fig|6666666.59329.peg.2794	fig|6666666.59330.peg.2282	fig|6666666.59331.peg.5452	fig|6666666.59332.peg.445	fig|6666666.59334.peg.2768	fig|6666666.59335.peg.3850	fig|6666666.59336.peg.1307	fig|6666666.59333.peg.2490	fig|6666666.59338.peg.3586	fig|6666666.59339.peg.8343	None	fig|6666666.59337.peg.267	None	fig|6666666.71181.peg.5209	None	fig|6666666.59345.peg.5235	fig|6666666.59341.peg.1510	fig|6666666.59347.peg.4670	fig|6666666.59346.peg.2552	fig|6666666.59348.peg.5258	None	None	None	None	fig|6666666.59355.peg.5237	None

9884	dTDP-4-dehydrorhamnose reductase EC 1.1.1.133	None	None	None	None	fig|6666666.59334.peg.3135	fig|6666666.59335.peg.503	fig|6666666.59336.peg.1635	fig|6666666.59333.peg.5155	fig|6666666.59338.peg.2782	fig|6666666.59339.peg.6322	None	None	None	fig|6666666.71181.peg.222	fig|6666666.59344.peg.272	fig|6666666.59345.peg.5522	fig|6666666.59341.peg.4792	fig|6666666.59347.peg.5008	None	fig|6666666.59348.peg.883	fig|6666666.59349.peg.1550	fig|6666666.59350.peg.7356	fig|6666666.59351.peg.242	fig|6666666.59353.peg.600	fig|6666666.59355.peg.82	fig|6666666.59354.peg.3687

1203	FIG01200261 hypothetical protein	fig|6666666.59329.peg.4269	fig|6666666.59330.peg.3631	fig|6666666.59331.peg.5667	fig|6666666.59332.peg.2792	fig|6666666.59334.peg.382	fig|6666666.59335.peg.1188	fig|6666666.59336.peg.1605	fig|6666666.59333.peg.3282	fig|6666666.59338.peg.795	fig|6666666.59339.peg.23	None	fig|6666666.59337.peg.4490	fig|6666666.59342.peg.5073	None	None	fig|6666666.59345.peg.2164	fig|6666666.59341.peg.1667	fig|6666666.59347.peg.2858	fig|6666666.59346.peg.2495	None	fig|6666666.59349.peg.3587	None	fig|6666666.59351.peg.4431	None	None	None

9691	Negative regulator of flagellin synthesis FlgM	fig|6666666.59329.peg.572	fig|6666666.59330.peg.2694	fig|6666666.59331.peg.5627	fig|6666666.59332.peg.1800	None	fig|6666666.59335.peg.2076	fig|6666666.59336.peg.1968	None	fig|6666666.59338.peg.626	fig|6666666.59339.peg.5307	None	fig|6666666.59337.peg.3342	fig|6666666.59342.peg.4044	None	None	fig|6666666.59345.peg.5445	fig|6666666.59341.peg.4564	fig|6666666.59347.peg.2718	fig|6666666.59346.peg.1758	fig|6666666.59348.peg.4697	fig|6666666.59349.peg.717	None	fig|6666666.59351.peg.2563	None	None	fig|6666666.59354.peg.1519

3151	Type IV pilus biogenesis protein PilM	fig|6666666.59329.peg.308	fig|6666666.59330.peg.1692	fig|6666666.59331.peg.889	fig|6666666.59332.peg.1024	None	fig|6666666.59335.peg.2490	fig|6666666.59336.peg.4164	fig|6666666.59333.peg.4147	fig|6666666.59338.peg.956	fig|6666666.59339.peg.8726	None	fig|6666666.59337.peg.1695	None	None	None	fig|6666666.59345.peg.5386	fig|6666666.59341.peg.962	fig|6666666.59347.peg.4938	fig|6666666.59346.peg.1	fig|6666666.59348.peg.1918	fig|6666666.59349.peg.522	None	fig|6666666.59351.peg.3845	None	None	fig|6666666.59354.peg.2397

1515	FIG01201035 hypothetical protein	fig|6666666.59329.peg.2250	fig|6666666.59330.peg.3171	fig|6666666.59331.peg.352	None	None	fig|6666666.59335.peg.1219	None	fig|6666666.59333.peg.2792	fig|6666666.59338.peg.5195	fig|6666666.59339.peg.1503	None	fig|6666666.59337.peg.4562	fig|6666666.59342.peg.2340	fig|6666666.71181.peg.264	fig|6666666.59344.peg.1191	fig|6666666.59345.peg.3246	fig|6666666.59341.peg.916	fig|6666666.59347.peg.1760	fig|6666666.59346.peg.5099	fig|6666666.59348.peg.4461	None	None	None	fig|6666666.59353.peg.1694	fig|6666666.59355.peg.1026	None

2679	DnaJ-related protein	fig|6666666.59329.peg.3498	fig|6666666.59330.peg.3929	fig|6666666.59331.peg.1924	fig|6666666.59332.peg.5339	None	fig|6666666.59335.peg.6107	fig|6666666.59336.peg.4320	fig|6666666.59333.peg.2331	fig|6666666.59338.peg.884	fig|6666666.59339.peg.2840	None	fig|6666666.59337.peg.5806	None	None	None	fig|6666666.59345.peg.103	fig|6666666.59341.peg.5324	fig|6666666.59347.peg.992	fig|6666666.59346.peg.3464	fig|6666666.59348.peg.3747	fig|6666666.59349.peg.1577	None	fig|6666666.59351.peg.4769	None	None	fig|6666666.59354.peg.1091

4654	Pyridoxamine 5'-phosphate oxidase-related putative heme iron utilization protein	fig|6666666.59329.peg.4536	fig|6666666.59330.peg.4172	None	fig|6666666.59332.peg.3360	fig|6666666.59334.peg.1579	fig|6666666.59335.peg.3624	fig|6666666.59336.peg.3053	fig|6666666.59333.peg.3774	None	fig|6666666.59339.peg.4298	None	fig|6666666.59337.peg.2461	fig|6666666.59342.peg.3207	None	None	fig|6666666.59345.peg.592	fig|6666666.59341.peg.3099	fig|6666666.59347.peg.645	fig|6666666.59346.peg.3908	fig|6666666.59348.peg.5366	fig|6666666.59349.peg.4549	None	fig|6666666.59351.peg.535	None	None	fig|6666666.59354.peg.5862

1283	CheW domain protein	fig|6666666.59329.peg.4552	fig|6666666.59330.peg.4384	fig|6666666.59331.peg.4295	fig|6666666.59332.peg.2652	None	fig|6666666.59335.peg.1243	fig|6666666.59336.peg.6019	fig|6666666.59333.peg.2588	fig|6666666.59338.peg.5310	fig|6666666.59339.peg.3020	None	fig|6666666.59337.peg.4216	fig|6666666.59342.peg.3625	None	None	fig|6666666.59345.peg.5320	fig|6666666.59341.peg.5588	fig|6666666.59347.peg.5086	fig|6666666.59346.peg.5116	None	fig|6666666.59349.peg.188	None	fig|6666666.59351.peg.2071	None	None	fig|6666666.59354.peg.2941

2699	LSU ribosomal protein L34p	fig|6666666.59329.peg.2420	fig|6666666.59330.peg.1107	fig|6666666.59331.peg.1545	fig|6666666.59332.peg.88	fig|6666666.59334.peg.1673	fig|6666666.59335.peg.782	fig|6666666.59336.peg.16	fig|6666666.59333.peg.44	fig|6666666.59338.peg.1274	fig|6666666.59339.peg.8030	None	fig|6666666.59337.peg.880	fig|6666666.59342.peg.514	None	None	fig|6666666.59345.peg.4975	fig|6666666.59341.peg.1046	fig|6666666.59347.peg.4793	None	None	fig|6666666.59349.peg.1009	None	None	None	fig|6666666.59355.peg.5689	fig|6666666.59354.peg.1561

7199	FIG01204079 hypothetical protein	fig|6666666.59329.peg.1647	None	fig|6666666.59331.peg.2170	fig|6666666.59332.peg.4032	fig|6666666.59334.peg.3942	fig|6666666.59335.peg.4249	fig|6666666.59336.peg.304	fig|6666666.59333.peg.1214	fig|6666666.59338.peg.231	fig|6666666.59339.peg.1704	fig|6666666.59340.peg.1324	fig|6666666.59337.peg.215	fig|6666666.59342.peg.5040	None	None	fig|6666666.59345.peg.5287	fig|6666666.59341.peg.3822	fig|6666666.59347.peg.3	fig|6666666.59346.peg.2993	fig|6666666.59348.peg.2836	None	fig|6666666.59350.peg.6240	None	None	None	None

767	GTP pyrophosphokinase EC 2.7.6.5 , p ppGpp synthetase II Guanosine-3',5'-bis diphosphate 3'-pyrophosphohydrolase EC 3.1.7.2	fig|6666666.59329.peg.1267	fig|6666666.59330.peg.2470	fig|6666666.59331.peg.2950	None	fig|6666666.59334.peg.326	None	fig|6666666.59336.peg.2782	fig|6666666.59333.peg.4056	None	fig|6666666.59339.peg.7382	fig|6666666.59340.peg.158	fig|6666666.59337.peg.503	fig|6666666.59342.peg.582	fig|6666666.71181.peg.2469	fig|6666666.59344.peg.5292	None	fig|6666666.59341.peg.4960	None	fig|6666666.59346.peg.1383	fig|6666666.59348.peg.1930	None	fig|6666666.59350.peg.1361	None	fig|6666666.59353.peg.3610	fig|6666666.59355.peg.4795	None

10426	FIG01200504 hypothetical protein	fig|6666666.59329.peg.356	fig|6666666.59330.peg.406	None	fig|6666666.59332.peg.2113	None	fig|6666666.59335.peg.732	fig|6666666.59336.peg.438	fig|6666666.59333.peg.491	fig|6666666.59338.peg.4352	fig|6666666.59339.peg.6180	fig|6666666.59340.peg.3807	fig|6666666.59337.peg.991	fig|6666666.59342.peg.6228	None	None	fig|6666666.59345.peg.2688	None	fig|6666666.59347.peg.400	fig|6666666.59346.peg.1089	fig|6666666.59348.peg.677	fig|6666666.59349.peg.1110	None	fig|6666666.59351.peg.63	None	None	fig|6666666.59354.peg.5033

381	Lipase precursor EC 3.1.1.3	fig|6666666.59329.peg.3679	None	fig|6666666.59331.peg.6347	fig|6666666.59332.peg.4576	None	fig|6666666.59335.peg.5324	fig|6666666.59336.peg.2893	fig|6666666.59333.peg.1630	None	fig|6666666.59339.peg.532	fig|6666666.59340.peg.3512	fig|6666666.59337.peg.2341	fig|6666666.59342.peg.1821	None	None	fig|6666666.59345.peg.1260	fig|6666666.59341.peg.4679	fig|6666666.59347.peg.87	fig|6666666.59346.peg.3432	fig|6666666.59348.peg.4904	fig|6666666.59349.peg.4565	None	fig|6666666.59351.peg.4949	None	None	fig|6666666.59354.peg.3729

10365	Outer membrane protein W precursor	fig|6666666.59329.peg.4816	fig|6666666.59330.peg.1624	None	None	None	fig|6666666.59335.peg.4557	fig|6666666.59336.peg.2205	fig|6666666.59333.peg.3999	None	fig|6666666.59339.peg.2054	None	fig|6666666.59337.peg.1276	None	fig|6666666.71181.peg.4140	fig|6666666.59344.peg.451	fig|6666666.59345.peg.4466	None	None	fig|6666666.59346.peg.487	fig|6666666.59348.peg.3329	fig|6666666.59349.peg.1415	fig|6666666.59350.peg.1907	fig|6666666.59351.peg.1403	fig|6666666.59353.peg.1772	fig|6666666.59355.peg.1240	fig|6666666.59354.peg.2310

7209	Plasma membrane protein involved in salt tolerance	fig|6666666.59329.peg.2820	fig|6666666.59330.peg.2709	None	fig|6666666.59332.peg.4637	None	None	fig|6666666.59336.peg.4244	fig|6666666.59333.peg.3904	None	None	None	fig|6666666.59337.peg.2438	fig|6666666.59342.peg.5833	fig|6666666.71181.peg.2962	fig|6666666.59344.peg.2882	fig|6666666.59345.peg.611	None	None	fig|6666666.59346.peg.3604	fig|6666666.59348.peg.5144	fig|6666666.59349.peg.3295	fig|6666666.59350.peg.647	fig|6666666.59351.peg.3272	fig|6666666.59353.peg.1442	fig|6666666.59355.peg.1991	fig|6666666.59354.peg.796

9767	FIG00920463 hypothetical protein	fig|6666666.59329.peg.6332	fig|6666666.59330.peg.4655	None	fig|6666666.59332.peg.4878	fig|6666666.59334.peg.4879	fig|6666666.59335.peg.3867	fig|6666666.59336.peg.6041	fig|6666666.59333.peg.4687	fig|6666666.59338.peg.1032	fig|6666666.59339.peg.1181	None	fig|6666666.59337.peg.2696	fig|6666666.59342.peg.4574	None	None	fig|6666666.59345.peg.3152	fig|6666666.59341.peg.3609	fig|6666666.59347.peg.1819	fig|6666666.59346.peg.5228	fig|6666666.59348.peg.5072	fig|6666666.59349.peg.4345	None	fig|6666666.59351.peg.911	None	None	None

4774	D-aminoacylase EC 3.5.1.81	fig|6666666.59329.peg.2274	None	fig|6666666.59331.peg.121	fig|6666666.59332.peg.3904	fig|6666666.59334.peg.3182	fig|6666666.59335.peg.2932	fig|6666666.59336.peg.4750	None	fig|6666666.59338.peg.4295	fig|6666666.59339.peg.5335	fig|6666666.59340.peg.1835	fig|6666666.59337.peg.5493	fig|6666666.59342.peg.1583	None	None	fig|6666666.59345.peg.4016	fig|6666666.59341.peg.1903	fig|6666666.59347.peg.1519	fig|6666666.59346.peg.2803	fig|6666666.59348.peg.3229	fig|6666666.59349.peg.4091	None	None	None	None	fig|6666666.59354.peg.4851

7223	FIG01199558 hypothetical protein	fig|6666666.59329.peg.2359	fig|6666666.59330.peg.1556	fig|6666666.59331.peg.3911	fig|6666666.59332.peg.2709	fig|6666666.59334.peg.3683	fig|6666666.59335.peg.6064	fig|6666666.59336.peg.637	fig|6666666.59333.peg.3188	fig|6666666.59338.peg.1988	fig|6666666.59339.peg.8556	fig|6666666.59340.peg.2875	fig|6666666.59337.peg.1639	fig|6666666.59342.peg.4797	None	None	fig|6666666.59345.peg.5480	fig|6666666.59341.peg.3292	fig|6666666.59347.peg.5063	fig|6666666.59346.peg.3703	fig|6666666.59348.peg.2612	None	None	None	None	None	None

3654	Cob I alamin adenosyltransferase EC 2.5.1.17	fig|6666666.59329.peg.4275	fig|6666666.59330.peg.862	fig|6666666.59331.peg.1515	fig|6666666.59332.peg.6006	fig|6666666.59334.peg.868	fig|6666666.59335.peg.6013	None	fig|6666666.59333.peg.64	fig|6666666.59338.peg.3078	fig|6666666.59339.peg.2210	None	fig|6666666.59337.peg.3282	None	None	None	fig|6666666.59345.peg.545	fig|6666666.59341.peg.5856	fig|6666666.59347.peg.209	fig|6666666.59346.peg.3060	None	fig|6666666.59349.peg.1725	fig|6666666.59350.peg.5602	fig|6666666.59351.peg.2375	None	None	fig|6666666.59354.peg.5313

781	Hypothetical protein, bax gene locus	fig|6666666.59329.peg.4261	fig|6666666.59330.peg.3199	None	None	fig|6666666.59334.peg.2623	fig|6666666.59335.peg.2217	fig|6666666.59336.peg.3526	fig|6666666.59333.peg.5507	fig|6666666.59338.peg.6163	fig|6666666.59339.peg.1314	None	fig|6666666.59337.peg.3715	fig|6666666.59342.peg.234	None	None	fig|6666666.59345.peg.3224	fig|6666666.59341.peg.5627	fig|6666666.59347.peg.1734	fig|6666666.59346.peg.1523	fig|6666666.59348.peg.4796	fig|6666666.59349.peg.166	None	fig|6666666.59351.peg.3683	None	None	fig|6666666.59354.peg.3884

2890	Thermolabile hemolysin precursor	fig|6666666.59329.peg.1426	fig|6666666.59330.peg.2368	fig|6666666.59331.peg.3861	fig|6666666.59332.peg.5650	fig|6666666.59334.peg.642	fig|6666666.59335.peg.1379	fig|6666666.59336.peg.1740	fig|6666666.59333.peg.2781	None	fig|6666666.59339.peg.2775	None	fig|6666666.59337.peg.2242	fig|6666666.59342.peg.3758	fig|6666666.71181.peg.653	fig|6666666.59344.peg.4142	fig|6666666.59345.peg.2967	None	None	fig|6666666.59346.peg.4822	fig|6666666.59348.peg.3480	None	None	None	fig|6666666.59353.peg.4118	fig|6666666.59355.peg.2321	None

8893	DNA-binding protein inhibitor Id-2-related protein	fig|6666666.59329.peg.5694	fig|6666666.59330.peg.1519	fig|6666666.59331.peg.1979	fig|6666666.59332.peg.3188	fig|6666666.59334.peg.3388	fig|6666666.59335.peg.5547	fig|6666666.59336.peg.2596	fig|6666666.59333.peg.4471	fig|6666666.59338.peg.1130	None	fig|6666666.59340.peg.3504	fig|6666666.59337.peg.2308	fig|6666666.59342.peg.3912	None	None	fig|6666666.59345.peg.1290	None	fig|6666666.59347.peg.2311	fig|6666666.59346.peg.4360	fig|6666666.59348.peg.5471	fig|6666666.59349.peg.3221	None	fig|6666666.59351.peg.3757	None	None	None

481	Histone deacetylase AcuC AphA family protein	fig|6666666.59329.peg.6533	fig|6666666.59330.peg.1202	None	fig|6666666.59332.peg.3136	None	fig|6666666.59335.peg.1442	fig|6666666.59336.peg.3175	fig|6666666.59333.peg.2468	fig|6666666.59338.peg.4938	fig|6666666.59339.peg.187	None	fig|6666666.59337.peg.3140	None	None	None	fig|6666666.59345.peg.1152	fig|6666666.59341.peg.1119	fig|6666666.59347.peg.2581	fig|6666666.59346.peg.94	fig|6666666.59348.peg.1319	fig|6666666.59349.peg.1909	fig|6666666.59350.peg.854	fig|6666666.59351.peg.470	None	None	fig|6666666.59354.peg.1722

4224	Predicted Fe-S protein	fig|6666666.59329.peg.1438	fig|6666666.59330.peg.3567	None	fig|6666666.59332.peg.1479	None	fig|6666666.59335.peg.5265	fig|6666666.59336.peg.2380	None	fig|6666666.59338.peg.508	fig|6666666.59339.peg.554	None	fig|6666666.59337.peg.3655	None	fig|6666666.71181.peg.2940	fig|6666666.59344.peg.3505	fig|6666666.59345.peg.1235	fig|6666666.59341.peg.5124	None	None	fig|6666666.59348.peg.362	fig|6666666.59349.peg.3768	None	fig|6666666.59351.peg.4287	fig|6666666.59353.peg.1379	fig|6666666.59355.peg.2494	fig|6666666.59354.peg.777

8317	Protein export cytoplasm protein SecA ATPase RNA helicase TC 3.A.5.1.1	fig|6666666.59329.peg.1003	fig|6666666.59330.peg.3948	fig|6666666.59331.peg.5644	fig|6666666.59332.peg.4457	fig|6666666.59334.peg.3444	fig|6666666.59335.peg.2517	fig|6666666.59336.peg.4022	fig|6666666.59333.peg.1536	fig|6666666.59338.peg.1473	fig|6666666.59339.peg.3132	None	fig|6666666.59337.peg.4371	fig|6666666.59342.peg.1963	None	None	fig|6666666.59345.peg.1496	fig|6666666.59341.peg.2384	fig|6666666.59347.peg.2626	fig|6666666.59346.peg.1435	fig|6666666.59348.peg.2975	None	fig|6666666.59350.peg.3271	None	None	None	None

5833	Probable hydrogenase cytochrome b-type subunit	fig|6666666.59329.peg.2948	fig|6666666.59330.peg.3264	None	fig|6666666.59332.peg.3443	None	fig|6666666.59335.peg.2813	fig|6666666.59336.peg.2137	None	fig|6666666.59338.peg.676	fig|6666666.59339.peg.1959	fig|6666666.59340.peg.2736	fig|6666666.59337.peg.1237	fig|6666666.59342.peg.5763	None	None	fig|6666666.59345.peg.4422	fig|6666666.59341.peg.2034	fig|6666666.59347.peg.390	fig|6666666.59346.peg.1045	fig|6666666.59348.peg.4075	fig|6666666.59349.peg.3388	None	fig|6666666.59351.peg.3366	None	None	fig|6666666.59354.peg.4509

9913	1,4-alpha-glucan branching enzyme EC 2.4.1.18	None	fig|6666666.59330.peg.870	None	fig|6666666.59332.peg.1922	fig|6666666.59334.peg.750	fig|6666666.59335.peg.5432	fig|6666666.59336.peg.3502	fig|6666666.59333.peg.57	fig|6666666.59338.peg.2328	fig|6666666.59339.peg.2052	None	fig|6666666.59337.peg.3290	fig|6666666.59342.peg.5503	None	None	fig|6666666.59345.peg.553	fig|6666666.59341.peg.1217	fig|6666666.59347.peg.3638	fig|6666666.59346.peg.4234	fig|6666666.59348.peg.2756	fig|6666666.59349.peg.965	None	fig|6666666.59351.peg.1581	None	None	fig|6666666.59354.peg.505

3421	Hydroxylamine reductase EC 1.7.-.-	fig|6666666.59329.peg.3302	None	fig|6666666.59331.peg.3212	fig|6666666.59332.peg.4528	fig|6666666.59334.peg.2027	fig|6666666.59335.peg.1829	None	fig|6666666.59333.peg.4582	fig|6666666.59338.peg.367	fig|6666666.59339.peg.1992	fig|6666666.59340.peg.2921	fig|6666666.59337.peg.3677	fig|6666666.59342.peg.3988	fig|6666666.71181.peg.4137	fig|6666666.59344.peg.391	fig|6666666.59345.peg.1252	fig|6666666.59341.peg.2942	fig|6666666.59347.peg.1540	None	None	None	None	None	fig|6666666.59353.peg.1611	fig|6666666.59355.peg.1110	None

11405	FIG00920344 hypothetical protein	fig|6666666.59329.peg.660	fig|6666666.59330.peg.1893	fig|6666666.59331.peg.419	fig|6666666.59332.peg.436	fig|6666666.59334.peg.2612	fig|6666666.59335.peg.3145	fig|6666666.59336.peg.784	fig|6666666.59333.peg.3764	fig|6666666.59338.peg.244	fig|6666666.59339.peg.5452	fig|6666666.59340.peg.710	fig|6666666.59337.peg.241	fig|6666666.59342.peg.1834	None	None	fig|6666666.59345.peg.5259	fig|6666666.59341.peg.3814	fig|6666666.59347.peg.13	fig|6666666.59346.peg.5111	fig|6666666.59348.peg.2955	None	None	None	None	None	None

10113	Cobalt-zinc-cadmium resistance protein	fig|6666666.59329.peg.2879	fig|6666666.59330.peg.918	fig|6666666.59331.peg.5849	fig|6666666.59332.peg.4308	fig|6666666.59334.peg.3309	fig|6666666.59335.peg.1454	fig|6666666.59336.peg.5149	fig|6666666.59333.peg.5007	fig|6666666.59338.peg.2906	fig|6666666.59339.peg.4444	fig|6666666.59340.peg.3754	fig|6666666.59337.peg.3944	None	fig|6666666.71181.peg.5901	None	fig|6666666.59345.peg.431	fig|6666666.59341.peg.5507	fig|6666666.59347.peg.1471	fig|6666666.59346.peg.3387	fig|6666666.59348.peg.4736	None	None	None	None	None	None

7131	Type IV pilus Tfp assembly protein PilF	fig|6666666.59329.peg.1934	None	fig|6666666.59331.peg.1762	fig|6666666.59332.peg.4425	None	fig|6666666.59335.peg.2010	fig|6666666.59336.peg.4332	fig|6666666.59333.peg.2386	fig|6666666.59338.peg.2712	fig|6666666.59339.peg.3948	None	fig|6666666.59337.peg.2657	fig|6666666.59342.peg.4333	fig|6666666.71181.peg.1768	fig|6666666.59344.peg.1642	fig|6666666.59345.peg.699	fig|6666666.59341.peg.724	fig|6666666.59347.peg.3444	None	fig|6666666.59348.peg.1722	None	None	None	fig|6666666.59353.peg.2707	fig|6666666.59355.peg.1773	None

1658	Type IV fimbrial biogenesis protein PilV	fig|6666666.59329.peg.1670	fig|6666666.59330.peg.4791	fig|6666666.59331.peg.1066	fig|6666666.59332.peg.4680	None	fig|6666666.59335.peg.1693	fig|6666666.59336.peg.6387	None	fig|6666666.59338.peg.5320	fig|6666666.59339.peg.7261	fig|6666666.59340.peg.295	fig|6666666.59337.peg.4981	fig|6666666.59342.peg.2188	None	None	fig|6666666.59345.peg.3565	fig|6666666.59341.peg.4066	fig|6666666.59347.peg.4337	fig|6666666.59346.peg.3607	None	fig|6666666.59349.peg.575	None	fig|6666666.59351.peg.508	None	None	fig|6666666.59354.peg.1573

467	FIG01199633 hypothetical protein	fig|6666666.59329.peg.3543	fig|6666666.59330.peg.2299	fig|6666666.59331.peg.6337	None	fig|6666666.59334.peg.1404	fig|6666666.59335.peg.6177	None	fig|6666666.59333.peg.3310	fig|6666666.59338.peg.4095	fig|6666666.59339.peg.3746	None	fig|6666666.59337.peg.3586	fig|6666666.59342.peg.3411	None	None	fig|6666666.59345.peg.868	fig|6666666.59341.peg.5466	fig|6666666.59347.peg.2239	fig|6666666.59346.peg.1684	fig|6666666.59348.peg.5454	fig|6666666.59349.peg.1775	None	fig|6666666.59351.peg.2301	None	None	fig|6666666.59354.peg.5632

6958	DNA ligase ATP EC 6.5.1.1	fig|6666666.59329.peg.2337	fig|6666666.59330.peg.4966	fig|6666666.59331.peg.3375	fig|6666666.59332.peg.4859	None	fig|6666666.59335.peg.3871	fig|6666666.59336.peg.4423	fig|6666666.59333.peg.4610	None	fig|6666666.59339.peg.1177	None	fig|6666666.59337.peg.4449	fig|6666666.59342.peg.5429	None	None	fig|6666666.59345.peg.3148	fig|6666666.59341.peg.5916	fig|6666666.59347.peg.1632	fig|6666666.59346.peg.1754	fig|6666666.59348.peg.2623	fig|6666666.59349.peg.3943	None	fig|6666666.59351.peg.418	None	None	fig|6666666.59354.peg.4552

10138	FIG01200281 hypothetical protein	fig|6666666.59329.peg.2921	fig|6666666.59330.peg.3729	fig|6666666.59331.peg.715	fig|6666666.59332.peg.5270	None	fig|6666666.59335.peg.1682	fig|6666666.59336.peg.4587	fig|6666666.59333.peg.4659	fig|6666666.59338.peg.5204	fig|6666666.59339.peg.3672	None	fig|6666666.59337.peg.4134	None	None	None	fig|6666666.59345.peg.4604	fig|6666666.59341.peg.374	fig|6666666.59347.peg.1963	fig|6666666.59346.peg.2508	fig|6666666.59348.peg.892	fig|6666666.59349.peg.1945	None	fig|6666666.59351.peg.2352	None	None	fig|6666666.59354.peg.1727

8957	Histidine permease YuiF	fig|6666666.59329.peg.792	fig|6666666.59330.peg.3383	None	None	fig|6666666.59334.peg.1046	fig|6666666.59335.peg.1201	fig|6666666.59336.peg.3557	fig|6666666.59333.peg.3174	fig|6666666.59338.peg.1828	fig|6666666.59339.peg.46	None	fig|6666666.59337.peg.4418	fig|6666666.59342.peg.1047	None	None	fig|6666666.59345.peg.1132	fig|6666666.59341.peg.2711	fig|6666666.59347.peg.5382	fig|6666666.59346.peg.4473	fig|6666666.59348.peg.81	fig|6666666.59349.peg.1592	None	fig|6666666.59351.peg.1014	None	None	fig|6666666.59354.peg.3516

5422	FIG106692 Outer membrane lipoprotein	fig|6666666.59329.peg.1308	fig|6666666.59330.peg.5078	None	fig|6666666.59332.peg.2650	fig|6666666.59334.peg.877	fig|6666666.59335.peg.5947	fig|6666666.59336.peg.3909	fig|6666666.59333.peg.4584	None	fig|6666666.59339.peg.290	None	fig|6666666.59337.peg.3367	fig|6666666.59342.peg.119	fig|6666666.71181.peg.2932	fig|6666666.59344.peg.5014	fig|6666666.59345.peg.1550	fig|6666666.59341.peg.669	None	fig|6666666.59346.peg.2527	fig|6666666.59348.peg.1670	None	None	None	fig|6666666.59353.peg.1387	fig|6666666.59355.peg.1376	None

9411	Gluconate permease	fig|6666666.59329.peg.5840	fig|6666666.59330.peg.4025	None	None	None	None	fig|6666666.59336.peg.771	fig|6666666.59333.peg.690	fig|6666666.59338.peg.2995	None	None	fig|6666666.59337.peg.2938	fig|6666666.59342.peg.4957	fig|6666666.71181.peg.3493	fig|6666666.59344.peg.2699	fig|6666666.59345.peg.2072	fig|6666666.59341.peg.5195	fig|6666666.59347.peg.3160	None	fig|6666666.59348.peg.2115	fig|6666666.59349.peg.1107	None	fig|6666666.59351.peg.2370	fig|6666666.59353.peg.694	fig|6666666.59355.peg.180	fig|6666666.59354.peg.4500

11378	Cobalt zinc cadmium efflux RND transporter, membrane fusion protein, CzcB family	fig|6666666.59329.peg.5235	fig|6666666.59330.peg.3969	fig|6666666.59331.peg.3677	fig|6666666.59332.peg.5523	None	fig|6666666.59335.peg.1406	fig|6666666.59336.peg.3442	fig|6666666.59333.peg.3850	fig|6666666.59338.peg.4549	fig|6666666.59339.peg.6668	None	fig|6666666.59337.peg.5732	fig|6666666.59342.peg.3810	None	None	fig|6666666.59345.peg.4002	fig|6666666.59341.peg.1492	fig|6666666.59347.peg.3764	fig|6666666.59346.peg.4076	None	fig|6666666.59349.peg.2570	None	fig|6666666.59351.peg.2530	None	None	fig|6666666.59354.peg.4044

6365	Formate--tetrahydrofolate ligase EC 6.3.4.3	fig|6666666.59329.peg.3640	fig|6666666.59330.peg.3423	fig|6666666.59331.peg.3575	fig|6666666.59332.peg.2415	fig|6666666.59334.peg.2628	fig|6666666.59335.peg.3675	fig|6666666.59336.peg.5805	fig|6666666.59333.peg.5343	fig|6666666.59338.peg.6086	fig|6666666.59339.peg.2659	None	fig|6666666.59337.peg.2499	fig|6666666.59342.peg.239	None	None	fig|6666666.59345.peg.145	fig|6666666.59341.peg.6038	fig|6666666.59347.peg.815	fig|6666666.59346.peg.5122	fig|6666666.59348.peg.2731	None	fig|6666666.59350.peg.2407	None	None	None	None

9291	Putative methyltransferase associated with DUF414	None	fig|6666666.59330.peg.392	fig|6666666.59331.peg.5675	fig|6666666.59332.peg.22	None	fig|6666666.59335.peg.54	fig|6666666.59336.peg.1452	fig|6666666.59333.peg.840	fig|6666666.59338.peg.6157	fig|6666666.59339.peg.7594	fig|6666666.59340.peg.1144	fig|6666666.59337.peg.975	fig|6666666.59342.peg.2843	None	None	fig|6666666.59345.peg.2700	fig|6666666.59341.peg.4286	fig|6666666.59347.peg.5044	fig|6666666.59346.peg.1077	fig|6666666.59348.peg.101	fig|6666666.59349.peg.4814	None	fig|6666666.59351.peg.102	None	None	None

5496	Mobile element protein	None	None	None	fig|6666666.59332.peg.6164	fig|6666666.59334.peg.3772	fig|6666666.59335.peg.6233	None	fig|6666666.59333.peg.5568	fig|6666666.59338.peg.1491	None	fig|6666666.59340.peg.3014	None	fig|6666666.59342.peg.6136	fig|6666666.71181.peg.4476	fig|6666666.59344.peg.351	fig|6666666.59345.peg.3600	None	fig|6666666.59347.peg.5435	fig|6666666.59346.peg.5313	fig|6666666.59348.peg.183	fig|6666666.59349.peg.4337	fig|6666666.59350.peg.6245	None	fig|6666666.59353.peg.536	fig|6666666.59355.peg.21	fig|6666666.59354.peg.5250

3230	FIG01200212 hypothetical protein	fig|6666666.59329.peg.2517	fig|6666666.59330.peg.2098	fig|6666666.59331.peg.4035	fig|6666666.59332.peg.2671	fig|6666666.59334.peg.4406	fig|6666666.59335.peg.6119	fig|6666666.59336.peg.1994	fig|6666666.59333.peg.3959	fig|6666666.59338.peg.3075	fig|6666666.59339.peg.2857	fig|6666666.59340.peg.1756	fig|6666666.59337.peg.3533	fig|6666666.59342.peg.964	None	None	fig|6666666.59345.peg.487	fig|6666666.59341.peg.4952	fig|6666666.59347.peg.664	fig|6666666.59346.peg.4259	fig|6666666.59348.peg.2524	None	None	None	None	None	None

11119	Adenosylcobinamide-phosphate guanylyltransferase EC 2.7.7.62	fig|6666666.59329.peg.3345	fig|6666666.59330.peg.2661	fig|6666666.59331.peg.1673	fig|6666666.59332.peg.6074	None	fig|6666666.59335.peg.2168	fig|6666666.59336.peg.5744	fig|6666666.59333.peg.1784	fig|6666666.59338.peg.3801	fig|6666666.59339.peg.3907	None	fig|6666666.59337.peg.1982	fig|6666666.59342.peg.2060	None	None	fig|6666666.59345.peg.4674	fig|6666666.59341.peg.547	fig|6666666.59347.peg.3353	fig|6666666.59346.peg.2035	fig|6666666.59348.peg.3562	None	fig|6666666.59350.peg.2437	fig|6666666.59351.peg.3916	None	None	None

4832	Integral membrane protein TerC	None	fig|6666666.59330.peg.4776	None	fig|6666666.59332.peg.5307	None	fig|6666666.59335.peg.2358	fig|6666666.59336.peg.6582	fig|6666666.59333.peg.3778	fig|6666666.59338.peg.2875	fig|6666666.59339.peg.2885	None	fig|6666666.59337.peg.4958	fig|6666666.59342.peg.548	fig|6666666.71181.peg.4840	fig|6666666.59344.peg.5653	fig|6666666.59345.peg.121	fig|6666666.59341.peg.1758	fig|6666666.59347.peg.755	fig|6666666.59346.peg.2896	fig|6666666.59348.peg.2913	None	None	None	fig|6666666.59353.peg.2394	fig|6666666.59355.peg.5595	None

8141	hypothetical protein	None	None	None	fig|6666666.59332.peg.5761	None	fig|6666666.59335.peg.2148	fig|6666666.59336.peg.4144	fig|6666666.59333.peg.4470	fig|6666666.59338.peg.4816	fig|6666666.59339.peg.6765	None	fig|6666666.59337.peg.2307	fig|6666666.59342.peg.3913	fig|6666666.71181.peg.1548	fig|6666666.59344.peg.2323	fig|6666666.59345.peg.1291	fig|6666666.59341.peg.328	fig|6666666.59347.peg.1377	fig|6666666.59346.peg.4875	fig|6666666.59348.peg.2307	None	fig|6666666.59350.peg.2123	None	fig|6666666.59353.peg.2886	fig|6666666.59355.peg.2853	None

6999	Polyhydroxyalkanoic acid synthase	None	fig|6666666.59330.peg.2099	fig|6666666.59331.peg.4202	fig|6666666.59332.peg.2670	None	fig|6666666.59335.peg.1375	fig|6666666.59336.peg.1995	fig|6666666.59333.peg.3958	fig|6666666.59338.peg.1237	fig|6666666.59339.peg.2769	None	fig|6666666.59337.peg.3531	fig|6666666.59342.peg.4009	None	None	fig|6666666.59345.peg.488	fig|6666666.59341.peg.2028	fig|6666666.59347.peg.1602	fig|6666666.59346.peg.4260	fig|6666666.59348.peg.3270	fig|6666666.59349.peg.4438	None	fig|6666666.59351.peg.3588	None	None	fig|6666666.59354.peg.1120

6697	adenylate kinase	fig|6666666.59329.peg.2693	fig|6666666.59330.peg.5084	fig|6666666.59331.peg.6364	fig|6666666.59332.peg.3523	None	fig|6666666.59335.peg.3253	fig|6666666.59336.peg.3585	fig|6666666.59333.peg.3040	None	fig|6666666.59339.peg.1440	None	fig|6666666.59337.peg.1156	fig|6666666.59342.peg.6071	fig|6666666.71181.peg.2111	fig|6666666.59344.peg.5310	fig|6666666.59345.peg.3464	None	None	fig|6666666.59346.peg.1514	fig|6666666.59348.peg.3729	None	fig|6666666.59350.peg.6290	None	fig|6666666.59353.peg.700	fig|6666666.59355.peg.186	None

3801	50S ribosomal subunit maturation GTPase RbgA B. subtilis YlqF	fig|6666666.59329.peg.3731	fig|6666666.59330.peg.5107	None	fig|6666666.59332.peg.3816	fig|6666666.59334.peg.1819	fig|6666666.59335.peg.1882	fig|6666666.59336.peg.4999	fig|6666666.59333.peg.5229	None	fig|6666666.59339.peg.4730	None	fig|6666666.59337.peg.5198	fig|6666666.59342.peg.1823	None	None	fig|6666666.59345.peg.3355	fig|6666666.59341.peg.2477	fig|6666666.59347.peg.896	fig|6666666.59346.peg.4732	fig|6666666.59348.peg.3583	fig|6666666.59349.peg.1722	None	fig|6666666.59351.peg.2372	None	None	fig|6666666.59354.peg.2649

10726	ABC-type tungstate transport system, periplasmic binding protein	fig|6666666.59329.peg.2469	fig|6666666.59330.peg.3811	None	fig|6666666.59332.peg.5968	fig|6666666.59334.peg.2695	fig|6666666.59335.peg.5040	fig|6666666.59336.peg.5623	fig|6666666.59333.peg.3237	fig|6666666.59338.peg.5609	fig|6666666.59339.peg.1216	fig|6666666.59340.peg.2646	fig|6666666.59337.peg.2719	fig|6666666.59342.peg.1126	None	None	fig|6666666.59345.peg.3198	fig|6666666.59341.peg.4006	fig|6666666.59347.peg.1702	fig|6666666.59346.peg.4518	fig|6666666.59348.peg.3731	None	fig|6666666.59350.peg.5769	None	None	None	None

4509	4Fe-4S ferredoxin, iron-sulfur binding	fig|6666666.59329.peg.5261	fig|6666666.59330.peg.5105	fig|6666666.59331.peg.4401	fig|6666666.59332.peg.1246	fig|6666666.59334.peg.1900	fig|6666666.59335.peg.6021	fig|6666666.59336.peg.2224	None	fig|6666666.59338.peg.437	fig|6666666.59339.peg.1948	fig|6666666.59340.peg.1108	fig|6666666.59337.peg.4211	fig|6666666.59342.peg.5954	None	None	fig|6666666.59345.peg.1618	fig|6666666.59341.peg.1304	fig|6666666.59347.peg.1563	fig|6666666.59346.peg.2619	fig|6666666.59348.peg.3449	None	fig|6666666.59350.peg.291	None	None	None	None

66	Ferritin-like protein 2	None	fig|6666666.59330.peg.236	None	fig|6666666.59332.peg.1721	fig|6666666.59334.peg.1194	None	fig|6666666.59336.peg.5075	fig|6666666.59333.peg.674	fig|6666666.59338.peg.77	fig|6666666.59339.peg.7552	None	fig|6666666.59337.peg.952	fig|6666666.59342.peg.5889	fig|6666666.71181.peg.859	fig|6666666.59344.peg.15	fig|6666666.59345.peg.2723	fig|6666666.59341.peg.4152	fig|6666666.59347.peg.4573	fig|6666666.59346.peg.3446	fig|6666666.59348.peg.2085	None	None	None	fig|6666666.59353.peg.355	fig|6666666.59355.peg.1393	None

8260	Glucosamine-link cellobiase EC 3.2.1.21	fig|6666666.59329.peg.855	fig|6666666.59330.peg.3991	fig|6666666.59331.peg.3973	fig|6666666.59332.peg.237	fig|6666666.59334.peg.1249	fig|6666666.59335.peg.1537	fig|6666666.59336.peg.2390	fig|6666666.59333.peg.3002	fig|6666666.59338.peg.3551	fig|6666666.59339.peg.4812	fig|6666666.59340.peg.1610	fig|6666666.59337.peg.650	fig|6666666.59342.peg.4423	None	None	fig|6666666.59345.peg.2386	fig|6666666.59341.peg.1810	fig|6666666.59347.peg.3148	fig|6666666.59346.peg.764	fig|6666666.59348.peg.655	None	None	None	None	None	None

751	FIG01199952 hypothetical protein	fig|6666666.59329.peg.2336	fig|6666666.59330.peg.4967	fig|6666666.59331.peg.6370	fig|6666666.59332.peg.3139	fig|6666666.59334.peg.4149	fig|6666666.59335.peg.3870	fig|6666666.59336.peg.4424	fig|6666666.59333.peg.5084	None	fig|6666666.59339.peg.1178	None	fig|6666666.59337.peg.4450	fig|6666666.59342.peg.5428	None	None	fig|6666666.59345.peg.3149	None	fig|6666666.59347.peg.1633	fig|6666666.59346.peg.91	fig|6666666.59348.peg.2692	fig|6666666.59349.peg.4341	None	fig|6666666.59351.peg.907	None	None	fig|6666666.59354.peg.5809

2950	Probable thiol oxidoreductase with 2 cytochrome c heme-binding sites	fig|6666666.59329.peg.5292	fig|6666666.59330.peg.2306	fig|6666666.59331.peg.2724	fig|6666666.59332.peg.812	None	fig|6666666.59335.peg.4604	fig|6666666.59336.peg.4388	fig|6666666.59333.peg.3694	fig|6666666.59338.peg.4442	fig|6666666.59339.peg.3735	fig|6666666.59340.peg.2959	fig|6666666.59337.peg.1769	fig|6666666.59342.peg.2332	None	None	fig|6666666.59345.peg.875	fig|6666666.59341.peg.4887	fig|6666666.59347.peg.2245	fig|6666666.59346.peg.4446	fig|6666666.59348.peg.3163	None	fig|6666666.59350.peg.1272	None	None	None	None

5732	FIG01200397 hypothetical protein	fig|6666666.59329.peg.5565	fig|6666666.59330.peg.1939	fig|6666666.59331.peg.5878	fig|6666666.59332.peg.4304	fig|6666666.59334.peg.5392	fig|6666666.59335.peg.612	fig|6666666.59336.peg.4952	fig|6666666.59333.peg.3150	fig|6666666.59338.peg.1409	fig|6666666.59339.peg.467	fig|6666666.59340.peg.1705	fig|6666666.59337.peg.4603	fig|6666666.59342.peg.2455	None	None	fig|6666666.59345.peg.1326	fig|6666666.59341.peg.477	fig|6666666.59347.peg.4120	fig|6666666.59346.peg.1567	fig|6666666.59348.peg.1072	None	None	None	None	None	None

2361	4-hydroxyproline epimerase EC 5.1.1.8	fig|6666666.59329.peg.3524	None	None	None	None	fig|6666666.59335.peg.5353	fig|6666666.59336.peg.5246	fig|6666666.59333.peg.3522	fig|6666666.59338.peg.5501	fig|6666666.59339.peg.8775	None	None	fig|6666666.59342.peg.2430	fig|6666666.71181.peg.341	fig|6666666.59344.peg.1287	fig|6666666.59345.peg.5596	None	fig|6666666.59347.peg.137	None	fig|6666666.59348.peg.4940	fig|6666666.59349.peg.3141	fig|6666666.59350.peg.1713	fig|6666666.59351.peg.680	fig|6666666.59353.peg.4203	fig|6666666.59355.peg.5569	fig|6666666.59354.peg.969

5112	FIG00920510 hypothetical protein	fig|6666666.59329.peg.4450	None	fig|6666666.59331.peg.676	fig|6666666.59332.peg.968	None	fig|6666666.59335.peg.5784	fig|6666666.59336.peg.2605	fig|6666666.59333.peg.1668	fig|6666666.59338.peg.2659	fig|6666666.59339.peg.5648	None	fig|6666666.59337.peg.4356	None	fig|6666666.71181.peg.740	fig|6666666.59344.peg.718	fig|6666666.59345.peg.1906	fig|6666666.59341.peg.1949	fig|6666666.59347.peg.1351	fig|6666666.59346.peg.2582	fig|6666666.59348.peg.2137	None	None	None	fig|6666666.59353.peg.961	fig|6666666.59355.peg.2415	None

10484	FIG01199974 hypothetical protein	fig|6666666.59329.peg.5965	fig|6666666.59330.peg.795	fig|6666666.59331.peg.2718	fig|6666666.59332.peg.4916	fig|6666666.59334.peg.4192	fig|6666666.59335.peg.555	fig|6666666.59336.peg.5475	fig|6666666.59333.peg.1530	fig|6666666.59338.peg.1744	fig|6666666.59339.peg.2952	None	fig|6666666.59337.peg.3822	fig|6666666.59342.peg.953	None	None	fig|6666666.59345.peg.66	fig|6666666.59341.peg.2226	fig|6666666.59347.peg.925	fig|6666666.59346.peg.5281	fig|6666666.59348.peg.2435	None	fig|6666666.59350.peg.7208	None	None	None	None

672	conserved hypothetical protein [Pyrococcus horikoshii]; C2102 Predicted ATPases of PP-loop superfamily; IPR002761 Domain of unknown function DUF71	fig|6666666.59329.peg.5080	fig|6666666.59330.peg.4570	fig|6666666.59331.peg.4248	fig|6666666.59332.peg.575	fig|6666666.59334.peg.5011	fig|6666666.59335.peg.5812	fig|6666666.59336.peg.3236	fig|6666666.59333.peg.1464	fig|6666666.59338.peg.3308	fig|6666666.59339.peg.8599	fig|6666666.59340.peg.2673	fig|6666666.59337.peg.1668	fig|6666666.59342.peg.5309	None	None	fig|6666666.59345.peg.5637	fig|6666666.59341.peg.3568	fig|6666666.59347.peg.4657	fig|6666666.59346.peg.1656	fig|6666666.59348.peg.3643	None	None	None	None	None	None

2497	FIG028593 membrane protein	fig|6666666.59329.peg.3500	fig|6666666.59330.peg.4645	fig|6666666.59331.peg.1675	None	None	fig|6666666.59335.peg.5408	None	fig|6666666.59333.peg.5551	fig|6666666.59338.peg.6097	fig|6666666.59339.peg.2838	None	fig|6666666.59337.peg.5669	fig|6666666.59342.peg.1672	fig|6666666.71181.peg.760	fig|6666666.59344.peg.4882	fig|6666666.59345.peg.105	fig|6666666.59341.peg.3253	fig|6666666.59347.peg.836	fig|6666666.59346.peg.3466	fig|6666666.59348.peg.4793	None	None	None	fig|6666666.59353.peg.941	fig|6666666.59355.peg.2437	None

7419	Sucrose-6-phosphate hydrolase EC 3.2.1.26	fig|6666666.59329.peg.5125	None	fig|6666666.59331.peg.1498	fig|6666666.59332.peg.4947	fig|6666666.59334.peg.3334	fig|6666666.59335.peg.3740	fig|6666666.59336.peg.1744	fig|6666666.59333.peg.1030	fig|6666666.59338.peg.2417	None	fig|6666666.59340.peg.1349	None	fig|6666666.59342.peg.3342	fig|6666666.71181.peg.3880	fig|6666666.59344.peg.883	fig|6666666.59345.peg.1725	fig|6666666.59341.peg.392	fig|6666666.59347.peg.3645	None	fig|6666666.59348.peg.4659	None	None	None	fig|6666666.59353.peg.1240	fig|6666666.59355.peg.3346	None

2123	Metal-dependent hydrolase	fig|6666666.59329.peg.5952	fig|6666666.59330.peg.770	fig|6666666.59331.peg.4214	fig|6666666.59332.peg.6203	fig|6666666.59334.peg.5514	fig|6666666.59335.peg.5232	fig|6666666.59336.peg.2636	fig|6666666.59333.peg.2776	None	fig|6666666.59339.peg.2789	None	fig|6666666.59337.peg.5361	None	None	None	fig|6666666.59345.peg.47	fig|6666666.59341.peg.4777	fig|6666666.59347.peg.911	fig|6666666.59346.peg.1207	fig|6666666.59348.peg.1865	fig|6666666.59349.peg.3249	None	fig|6666666.59351.peg.3281	None	None	fig|6666666.59354.peg.6476

6853	BatD	fig|6666666.59329.peg.5585	fig|6666666.59330.peg.3801	None	fig|6666666.59332.peg.5677	None	fig|6666666.59335.peg.4338	fig|6666666.59336.peg.529	None	fig|6666666.59338.peg.4000	fig|6666666.59339.peg.4362	fig|6666666.59340.peg.1568	fig|6666666.59337.peg.3496	fig|6666666.59342.peg.5689	None	None	fig|6666666.59345.peg.2761	fig|6666666.59341.peg.5817	fig|6666666.59347.peg.1489	fig|6666666.59346.peg.4940	fig|6666666.59348.peg.2276	fig|6666666.59349.peg.4452	None	fig|6666666.59351.peg.696	None	None	fig|6666666.59354.peg.4909

9099	FIG01200055 hypothetical protein	fig|6666666.59329.peg.3165	fig|6666666.59330.peg.4880	fig|6666666.59331.peg.1870	fig|6666666.59332.peg.5088	fig|6666666.59334.peg.1500	fig|6666666.59335.peg.4636	fig|6666666.59336.peg.2503	fig|6666666.59333.peg.2282	fig|6666666.59338.peg.1468	fig|6666666.59339.peg.417	fig|6666666.59340.peg.2822	fig|6666666.59337.peg.4367	None	None	fig|6666666.59344.peg.1493	fig|6666666.59345.peg.1494	fig|6666666.59341.peg.3871	fig|6666666.59347.peg.4218	fig|6666666.59346.peg.1431	fig|6666666.59348.peg.1933	None	None	None	None	None	None

5325	Fatty acid cis trans isomerase	fig|6666666.59329.peg.2011	fig|6666666.59330.peg.3183	None	None	fig|6666666.59334.peg.3452	fig|6666666.59335.peg.3673	fig|6666666.59336.peg.822	fig|6666666.59333.peg.3857	fig|6666666.59338.peg.2558	fig|6666666.59339.peg.2661	None	fig|6666666.59337.peg.5749	fig|6666666.59342.peg.2235	None	None	fig|6666666.59345.peg.3301	fig|6666666.59341.peg.5825	fig|6666666.59347.peg.971	fig|6666666.59346.peg.3651	fig|6666666.59348.peg.3497	fig|6666666.59349.peg.2283	None	fig|6666666.59351.peg.4164	None	None	fig|6666666.59354.peg.4738

3766	FIG01206288 hypothetical protein	None	None	fig|6666666.59331.peg.1817	fig|6666666.59332.peg.3379	fig|6666666.59334.peg.3901	fig|6666666.59335.peg.4891	fig|6666666.59336.peg.2946	fig|6666666.59333.peg.4764	fig|6666666.59338.peg.4302	fig|6666666.59339.peg.1635	None	None	fig|6666666.59342.peg.6457	fig|6666666.71181.peg.3874	fig|6666666.59344.peg.872	fig|6666666.59345.peg.4039	fig|6666666.59341.peg.5213	fig|6666666.59347.peg.3700	fig|6666666.59346.peg.3944	fig|6666666.59348.peg.4527	None	None	None	fig|6666666.59353.peg.3402	fig|6666666.59355.peg.3339	None

7892	Hypothetical Zinc-finger containing protein	None	fig|6666666.59330.peg.1931	fig|6666666.59331.peg.749	fig|6666666.59332.peg.5495	None	None	fig|6666666.59336.peg.5837	fig|6666666.59333.peg.2046	fig|6666666.59338.peg.3567	None	fig|6666666.59340.peg.2939	fig|6666666.59337.peg.4597	fig|6666666.59342.peg.4252	fig|6666666.71181.peg.2113	fig|6666666.59344.peg.5313	fig|6666666.59345.peg.1332	fig|6666666.59341.peg.5912	fig|6666666.59347.peg.2391	fig|6666666.59346.peg.1574	None	None	fig|6666666.59350.peg.282	None	fig|6666666.59353.peg.702	fig|6666666.59355.peg.188	None

1740	6-phosphogluconolactonase EC 3.1.1.31 , eukaryotic type	fig|6666666.59329.peg.3058	fig|6666666.59330.peg.3316	fig|6666666.59331.peg.2190	fig|6666666.59332.peg.1387	fig|6666666.59334.peg.3015	fig|6666666.59335.peg.3885	fig|6666666.59336.peg.6549	fig|6666666.59333.peg.1647	None	fig|6666666.59339.peg.2435	None	fig|6666666.59337.peg.3102	fig|6666666.59342.peg.2268	None	None	fig|6666666.59345.peg.4146	None	None	fig|6666666.59346.peg.3575	fig|6666666.59348.peg.3035	fig|6666666.59349.peg.662	fig|6666666.59350.peg.141	fig|6666666.59351.peg.4930	None	None	fig|6666666.59354.peg.4960

10313	Flagellar protein FlgT	fig|6666666.59329.peg.567	fig|6666666.59330.peg.3093	fig|6666666.59331.peg.5956	fig|6666666.59332.peg.2631	None	fig|6666666.59335.peg.2072	fig|6666666.59336.peg.3752	fig|6666666.59333.peg.205	fig|6666666.59338.peg.2319	fig|6666666.59339.peg.5302	None	fig|6666666.59337.peg.3338	None	None	None	fig|6666666.59345.peg.5441	fig|6666666.59341.peg.5370	fig|6666666.59347.peg.2723	fig|6666666.59346.peg.4728	fig|6666666.59348.peg.319	fig|6666666.59349.peg.713	None	fig|6666666.59351.peg.2568	None	None	fig|6666666.59354.peg.4322

7462	FIG01199646 hypothetical protein	fig|6666666.59329.peg.365	fig|6666666.59330.peg.986	fig|6666666.59331.peg.2344	fig|6666666.59332.peg.260	fig|6666666.59334.peg.2189	fig|6666666.59335.peg.17	fig|6666666.59336.peg.185	fig|6666666.59333.peg.1574	fig|6666666.59338.peg.2872	fig|6666666.59339.peg.7541	fig|6666666.59340.peg.2894	fig|6666666.59337.peg.2545	fig|6666666.59342.peg.6109	None	None	fig|6666666.59345.peg.5537	fig|6666666.59341.peg.4838	fig|6666666.59347.peg.5249	fig|6666666.59346.peg.925	fig|6666666.59348.peg.936	None	None	None	None	None	None

8481	2,3,4,5-tetrahydropyridine-2,6-dicarboxylate N-succinyltransferase EC 2.3.1.117	fig|6666666.59329.peg.2670	fig|6666666.59330.peg.2125	fig|6666666.59331.peg.2973	fig|6666666.59332.peg.3747	fig|6666666.59334.peg.3164	fig|6666666.59335.peg.3696	fig|6666666.59336.peg.105	fig|6666666.59333.peg.4531	fig|6666666.59338.peg.2349	fig|6666666.59339.peg.6882	fig|6666666.59340.peg.1384	fig|6666666.59337.peg.5132	fig|6666666.59342.peg.6291	None	None	fig|6666666.59345.peg.2270	fig|6666666.59341.peg.516	fig|6666666.59347.peg.2828	fig|6666666.59346.peg.1096	fig|6666666.59348.peg.1678	None	None	None	None	None	None

10693	FIG01200538 hypothetical protein	fig|6666666.59329.peg.1958	fig|6666666.59330.peg.739	None	fig|6666666.59332.peg.3050	fig|6666666.59334.peg.2487	fig|6666666.59335.peg.4656	fig|6666666.59336.peg.4631	fig|6666666.59333.peg.2250	fig|6666666.59338.peg.2495	fig|6666666.59339.peg.8390	fig|6666666.59340.peg.2063	fig|6666666.59337.peg.3468	fig|6666666.59342.peg.5583	None	None	fig|6666666.59345.peg.1783	fig|6666666.59341.peg.2255	fig|6666666.59347.peg.435	fig|6666666.59346.peg.4680	fig|6666666.59348.peg.1218	None	None	None	None	None	None

8295	FIG01201104 hypothetical protein	fig|6666666.59329.peg.4267	fig|6666666.59330.peg.3632	None	fig|6666666.59332.peg.2793	fig|6666666.59334.peg.381	fig|6666666.59335.peg.1189	fig|6666666.59336.peg.1606	fig|6666666.59333.peg.3281	fig|6666666.59338.peg.793	fig|6666666.59339.peg.26	None	fig|6666666.59337.peg.4491	None	None	None	fig|6666666.59345.peg.2163	fig|6666666.59341.peg.1668	fig|6666666.59347.peg.2857	fig|6666666.59346.peg.2497	None	fig|6666666.59349.peg.649	None	fig|6666666.59351.peg.4919	None	None	fig|6666666.59354.peg.2914

4729	Permease of the major facilitator superfamily	fig|6666666.59329.peg.4444	fig|6666666.59330.peg.849	fig|6666666.59331.peg.207	fig|6666666.59332.peg.3339	fig|6666666.59334.peg.542	fig|6666666.59335.peg.5147	fig|6666666.59336.peg.1339	fig|6666666.59333.peg.76	fig|6666666.59338.peg.2760	fig|6666666.59339.peg.4329	fig|6666666.59340.peg.3220	fig|6666666.59337.peg.3267	fig|6666666.59342.peg.4432	None	None	fig|6666666.59345.peg.531	fig|6666666.59341.peg.5044	fig|6666666.59347.peg.421	fig|6666666.59346.peg.5067	None	None	None	None	None	None	None

4621	FIG01200152 hypothetical protein	fig|6666666.59329.peg.1797	fig|6666666.59330.peg.1560	fig|6666666.59331.peg.5374	fig|6666666.59332.peg.2705	fig|6666666.59334.peg.3681	fig|6666666.59335.peg.6247	fig|6666666.59336.peg.3163	fig|6666666.59333.peg.3184	fig|6666666.59338.peg.2860	fig|6666666.59339.peg.8561	fig|6666666.59340.peg.84	fig|6666666.59337.peg.1644	None	None	None	fig|6666666.59345.peg.5476	fig|6666666.59341.peg.5361	fig|6666666.59347.peg.5060	fig|6666666.59346.peg.3706	fig|6666666.59348.peg.578	None	None	None	None	None	None

10438	FIG01201055 hypothetical protein	fig|6666666.59329.peg.2507	fig|6666666.59330.peg.3923	fig|6666666.59331.peg.5322	fig|6666666.59332.peg.2360	fig|6666666.59334.peg.802	fig|6666666.59335.peg.4192	fig|6666666.59336.peg.4351	fig|6666666.59333.peg.2620	fig|6666666.59338.peg.2109	fig|6666666.59339.peg.3188	fig|6666666.59340.peg.2874	fig|6666666.59337.peg.2678	None	None	None	fig|6666666.59345.peg.1054	fig|6666666.59341.peg.818	fig|6666666.59347.peg.3786	fig|6666666.59346.peg.117	fig|6666666.59348.peg.2018	None	None	None	None	None	None

6955	FIG00919661 hypothetical protein	fig|6666666.59329.peg.3621	fig|6666666.59330.peg.3394	fig|6666666.59331.peg.2870	fig|6666666.59332.peg.4395	fig|6666666.59334.peg.2986	fig|6666666.59335.peg.4532	fig|6666666.59336.peg.5551	fig|6666666.59333.peg.1909	fig|6666666.59338.peg.5932	fig|6666666.59339.peg.3544	None	fig|6666666.59337.peg.3515	fig|6666666.59342.peg.6275	None	None	fig|6666666.59345.peg.933	fig|6666666.59341.peg.3617	fig|6666666.59347.peg.2323	fig|6666666.59346.peg.3900	fig|6666666.59348.peg.3615	None	None	None	None	None	None

5670	FIG01201336 hypothetical protein	fig|6666666.59329.peg.5516	fig|6666666.59330.peg.1949	fig|6666666.59331.peg.4110	fig|6666666.59332.peg.4444	fig|6666666.59334.peg.1303	fig|6666666.59335.peg.1798	fig|6666666.59336.peg.2288	fig|6666666.59333.peg.5273	fig|6666666.59338.peg.3535	fig|6666666.59339.peg.3616	None	fig|6666666.59337.peg.2629	fig|6666666.59342.peg.849	None	None	fig|6666666.59345.peg.713	fig|6666666.59341.peg.3519	fig|6666666.59347.peg.1690	fig|6666666.59346.peg.1807	fig|6666666.59348.peg.1455	None	None	None	None	None	None

9565	Transcriptional regulator	fig|6666666.59329.peg.2224	fig|6666666.59330.peg.3015	fig|6666666.59331.peg.4286	fig|6666666.59332.peg.5828	None	fig|6666666.59335.peg.3134	fig|6666666.59336.peg.5992	None	fig|6666666.59338.peg.234	fig|6666666.59339.peg.5436	fig|6666666.59340.peg.1665	None	None	fig|6666666.71181.peg.1449	fig|6666666.59344.peg.1533	fig|6666666.59345.peg.5273	fig|6666666.59341.peg.3819	fig|6666666.59347.peg.6	fig|6666666.59346.peg.364	None	None	None	None	fig|6666666.59353.peg.4365	fig|6666666.59355.peg.4478	None

3276	ABC transporter substrate-binding protein	fig|6666666.59329.peg.6260	fig|6666666.59330.peg.4359	fig|6666666.59331.peg.2497	fig|6666666.59332.peg.1745	fig|6666666.59334.peg.1654	fig|6666666.59335.peg.4112	fig|6666666.59336.peg.2810	None	fig|6666666.59338.peg.6019	fig|6666666.59339.peg.3125	fig|6666666.59340.peg.1847	fig|6666666.59337.peg.4933	fig|6666666.59342.peg.806	None	None	fig|6666666.59345.peg.1097	fig|6666666.59341.peg.3141	fig|6666666.59347.peg.1038	fig|6666666.59346.peg.550	fig|6666666.59348.peg.4875	None	None	None	None	None	None

5803	DedA protein	fig|6666666.59329.peg.3109	fig|6666666.59330.peg.698	fig|6666666.59331.peg.85	None	None	None	fig|6666666.59336.peg.1544	fig|6666666.59333.peg.2005	fig|6666666.59338.peg.3186	None	fig|6666666.59340.peg.2207	fig|6666666.59337.peg.779	fig|6666666.59342.peg.2882	fig|6666666.71181.peg.4395	fig|6666666.59344.peg.5261	fig|6666666.59345.peg.4790	fig|6666666.59341.peg.4340	fig|6666666.59347.peg.2733	None	fig|6666666.59348.peg.958	None	None	None	fig|6666666.59353.peg.4709	fig|6666666.59355.peg.4781	None

2703	hypothetical protein	None	fig|6666666.59330.peg.2093	fig|6666666.59331.peg.2222	fig|6666666.59332.peg.1240	fig|6666666.59334.peg.2257	fig|6666666.59335.peg.5255	fig|6666666.59336.peg.2897	fig|6666666.59333.peg.1895	fig|6666666.59338.peg.4494	fig|6666666.59339.peg.2419	None	fig|6666666.59337.peg.3542	None	None	None	fig|6666666.59345.peg.477	fig|6666666.59341.peg.4686	fig|6666666.59347.peg.776	fig|6666666.59346.peg.2624	None	fig|6666666.59349.peg.4085	None	fig|6666666.59351.peg.2762	None	None	fig|6666666.59354.peg.466

9107	Pressure-regulated ORF-like protein	fig|6666666.59329.peg.1870	fig|6666666.59330.peg.1074	fig|6666666.59331.peg.4552	fig|6666666.59332.peg.1761	fig|6666666.59334.peg.2824	fig|6666666.59335.peg.1703	fig|6666666.59336.peg.6617	fig|6666666.59333.peg.2831	fig|6666666.59338.peg.723	fig|6666666.59339.peg.7249	fig|6666666.59340.peg.3574	fig|6666666.59337.peg.5652	None	None	None	fig|6666666.59345.peg.3556	fig|6666666.59341.peg.1476	fig|6666666.59347.peg.4349	fig|6666666.59346.peg.2248	fig|6666666.59348.peg.2780	None	None	None	None	None	None

7775	hypothetical protein	fig|6666666.59329.peg.3902	fig|6666666.59330.peg.4338	fig|6666666.59331.peg.4237	fig|6666666.59332.peg.6012	None	fig|6666666.59335.peg.662	fig|6666666.59336.peg.642	fig|6666666.59333.peg.2661	fig|6666666.59338.peg.4428	fig|6666666.59339.peg.4215	None	fig|6666666.59337.peg.5251	None	None	None	fig|6666666.59345.peg.2055	fig|6666666.59341.peg.397	fig|6666666.59347.peg.1348	fig|6666666.59346.peg.3630	fig|6666666.59348.peg.749	fig|6666666.59349.peg.2351	None	fig|6666666.59351.peg.1199	None	None	None

5015	Beta-galactosidase beta-glucuronidase	fig|6666666.59329.peg.3264	fig|6666666.59330.peg.2453	fig|6666666.59331.peg.5978	fig|6666666.59332.peg.3194	fig|6666666.59334.peg.752	fig|6666666.59335.peg.224	fig|6666666.59336.peg.1785	fig|6666666.59333.peg.2815	fig|6666666.59338.peg.129	fig|6666666.59339.peg.8178	None	fig|6666666.59337.peg.4000	fig|6666666.59342.peg.6285	None	None	fig|6666666.59345.peg.4840	fig|6666666.59341.peg.2702	fig|6666666.59347.peg.3836	fig|6666666.59346.peg.1677	fig|6666666.59348.peg.554	None	None	None	None	None	None

4859	Beta-lactamase class C and other penicillin binding proteins	None	None	fig|6666666.59331.peg.785	fig|6666666.59332.peg.4945	fig|6666666.59334.peg.2810	None	fig|6666666.59336.peg.1147	None	fig|6666666.59338.peg.4425	None	None	None	None	fig|6666666.71181.peg.2705	fig|6666666.59344.peg.3840	fig|6666666.59345.peg.1435	fig|6666666.59341.peg.3166	fig|6666666.59347.peg.4697	None	fig|6666666.59348.peg.2903	fig|6666666.59349.peg.129	fig|6666666.59350.peg.3202	fig|6666666.59351.peg.3415	fig|6666666.59353.peg.3341	fig|6666666.59355.peg.3887	fig|6666666.59354.peg.4968

3069	hypothetical protein	fig|6666666.59329.peg.4863	fig|6666666.59330.peg.4353	fig|6666666.59331.peg.3997	fig|6666666.59332.peg.721	fig|6666666.59334.peg.3349	fig|6666666.59335.peg.5932	fig|6666666.59336.peg.6257	None	fig|6666666.59338.peg.3016	fig|6666666.59339.peg.3619	fig|6666666.59340.peg.3775	fig|6666666.59337.peg.1455	fig|6666666.59342.peg.5348	None	None	fig|6666666.59345.peg.2905	fig|6666666.59341.peg.2547	fig|6666666.59347.peg.4061	fig|6666666.59346.peg.4426	fig|6666666.59348.peg.3463	None	None	None	None	None	None

3068	Putative cytoplasmic protein	None	None	fig|6666666.59331.peg.4575	fig|6666666.59332.peg.1599	fig|6666666.59334.peg.2963	fig|6666666.59335.peg.4892	fig|6666666.59336.peg.3282	None	fig|6666666.59338.peg.2444	fig|6666666.59339.peg.1637	fig|6666666.59340.peg.1941	None	None	fig|6666666.71181.peg.4187	fig|6666666.59344.peg.2790	fig|6666666.59345.peg.1983	None	fig|6666666.59347.peg.1425	fig|6666666.59346.peg.2762	fig|6666666.59348.peg.3388	None	fig|6666666.59350.peg.2368	None	fig|6666666.59353.peg.1655	fig|6666666.59355.peg.1066	None

7752	3-deoxy-D-manno-octulosonate 8-phosphate phosphatase EC 3.1.3.45	None	None	None	fig|6666666.59332.peg.5623	fig|6666666.59334.peg.97	fig|6666666.59335.peg.3048	None	None	fig|6666666.59338.peg.110	fig|6666666.59339.peg.8198	fig|6666666.59340.peg.1366	None	None	fig|6666666.71181.peg.1020	fig|6666666.59344.peg.1800	None	fig|6666666.59341.peg.1246	fig|6666666.59347.peg.3249	fig|6666666.59346.peg.636	fig|6666666.59348.peg.2006	fig|6666666.59349.peg.24	None	fig|6666666.59351.peg.3444	fig|6666666.59353.peg.2338	fig|6666666.59355.peg.1612	fig|6666666.59354.peg.5890

5038	hypothetical protein	fig|6666666.59329.peg.752	fig|6666666.59330.peg.4016	None	fig|6666666.59332.peg.959	fig|6666666.59334.peg.4587	fig|6666666.59335.peg.5022	fig|6666666.59336.peg.6214	fig|6666666.59333.peg.3835	fig|6666666.59338.peg.700	fig|6666666.59339.peg.1772	None	fig|6666666.59337.peg.2929	fig|6666666.59342.peg.6476	None	None	fig|6666666.59345.peg.2080	fig|6666666.59341.peg.3728	fig|6666666.59347.peg.3141	fig|6666666.59346.peg.4193	fig|6666666.59348.peg.2357	None	None	fig|6666666.59351.peg.3287	None	None	None

4438	Hydroxyethylthiazole kinase EC 2.7.1.50	fig|6666666.59329.peg.3207	fig|6666666.59330.peg.4729	fig|6666666.59331.peg.4528	fig|6666666.59332.peg.2528	fig|6666666.59334.peg.910	None	fig|6666666.59336.peg.3925	fig|6666666.59333.peg.3400	fig|6666666.59338.peg.3367	None	None	fig|6666666.59337.peg.1222	None	fig|6666666.71181.peg.4217	fig|6666666.59344.peg.6065	fig|6666666.59345.peg.4406	fig|6666666.59341.peg.321	fig|6666666.59347.peg.3679	fig|6666666.59346.peg.4175	None	None	None	None	fig|6666666.59353.peg.2742	fig|6666666.59355.peg.1740	None

314	Hypothetical protein C3496	fig|6666666.59329.peg.2103	fig|6666666.59330.peg.2876	fig|6666666.59331.peg.6135	None	fig|6666666.59334.peg.2690	fig|6666666.59335.peg.606	fig|6666666.59336.peg.4712	None	fig|6666666.59338.peg.1416	None	None	fig|6666666.59337.peg.4608	None	None	None	fig|6666666.59345.peg.1321	fig|6666666.59341.peg.483	fig|6666666.59347.peg.4126	None	fig|6666666.59348.peg.2671	fig|6666666.59349.peg.938	fig|6666666.59350.peg.4079	fig|6666666.59351.peg.1096	None	fig|6666666.59355.peg.5873	fig|6666666.59354.peg.1699

441	Bacterioferritin-associated ferredoxin	fig|6666666.59329.peg.4602	fig|6666666.59330.peg.1668	fig|6666666.59331.peg.5762	fig|6666666.59332.peg.3791	None	fig|6666666.59335.peg.3495	fig|6666666.59336.peg.3888	None	fig|6666666.59338.peg.1397	None	fig|6666666.59340.peg.2009	fig|6666666.59337.peg.1671	fig|6666666.59342.peg.5305	None	None	fig|6666666.59345.peg.5409	fig|6666666.59341.peg.2490	None	fig|6666666.59346.peg.5024	fig|6666666.59348.peg.5546	fig|6666666.59349.peg.4532	None	fig|6666666.59351.peg.3807	None	None	fig|6666666.59354.peg.6278

4061	Electron transfer flavoprotein, alpha subunit	fig|6666666.59329.peg.6459	fig|6666666.59330.peg.4451	None	None	None	fig|6666666.59335.peg.4302	fig|6666666.59336.peg.2883	fig|6666666.59333.peg.3306	fig|6666666.59338.peg.3108	fig|6666666.59339.peg.4646	None	fig|6666666.59337.peg.4801	fig|6666666.59342.peg.3419	None	None	fig|6666666.59345.peg.321	fig|6666666.59341.peg.4905	fig|6666666.59347.peg.1153	None	fig|6666666.59348.peg.3324	fig|6666666.59349.peg.4284	None	fig|6666666.59351.peg.1074	None	fig|6666666.59355.peg.5428	fig|6666666.59354.peg.2725

833	putative esterase	fig|6666666.59329.peg.4882	None	fig|6666666.59331.peg.3266	fig|6666666.59332.peg.2262	fig|6666666.59334.peg.1115	None	None	fig|6666666.59333.peg.5452	fig|6666666.59338.peg.5828	None	None	None	None	fig|6666666.71181.peg.2673	fig|6666666.59344.peg.4299	fig|6666666.59345.peg.1764	None	fig|6666666.59347.peg.5364	fig|6666666.59346.peg.347	fig|6666666.59348.peg.891	fig|6666666.59349.peg.4169	None	fig|6666666.59351.peg.2953	fig|6666666.59353.peg.1732	fig|6666666.59355.peg.988	fig|6666666.59354.peg.4748

4470	Transcriptional regulator LuxT	fig|6666666.59329.peg.3718	fig|6666666.59330.peg.2719	fig|6666666.59331.peg.3510	fig|6666666.59332.peg.999	fig|6666666.59334.peg.2124	fig|6666666.59335.peg.5242	fig|6666666.59336.peg.4894	fig|6666666.59333.peg.4874	fig|6666666.59338.peg.5478	fig|6666666.59339.peg.2491	None	fig|6666666.59337.peg.2450	fig|6666666.59342.peg.3869	None	None	fig|6666666.59345.peg.601	fig|6666666.59341.peg.5512	fig|6666666.59347.peg.639	fig|6666666.59346.peg.3026	fig|6666666.59348.peg.376	None	None	None	None	None	None

6312	Guanine deaminase EC 3.5.4.3	None	fig|6666666.59330.peg.1915	fig|6666666.59331.peg.2804	fig|6666666.59332.peg.1623	None	fig|6666666.59335.peg.1856	fig|6666666.59336.peg.6403	None	None	fig|6666666.59339.peg.8258	None	fig|6666666.59337.peg.4851	None	fig|6666666.71181.peg.3603	fig|6666666.59344.peg.5864	fig|6666666.59345.peg.1356	fig|6666666.59341.peg.4782	fig|6666666.59347.peg.3322	fig|6666666.59346.peg.2100	fig|6666666.59348.peg.405	None	fig|6666666.59350.peg.5353	None	fig|6666666.59353.peg.4150	fig|6666666.59355.peg.4265	None

1642	2-methylcitrate dehydratase FeS dependent EC 4.2.1.79	fig|6666666.59329.peg.1833	None	fig|6666666.59331.peg.5999	fig|6666666.59332.peg.6200	None	fig|6666666.59335.peg.5770	fig|6666666.59336.peg.6191	None	fig|6666666.59338.peg.5493	fig|6666666.59339.peg.4027	None	fig|6666666.59337.peg.5506	fig|6666666.59342.peg.2943	fig|6666666.71181.peg.4192	None	None	fig|6666666.59341.peg.5234	None	fig|6666666.59346.peg.4481	fig|6666666.59348.peg.4217	fig|6666666.59349.peg.1164	fig|6666666.59350.peg.5388	None	None	fig|6666666.59355.peg.6105	fig|6666666.59354.peg.5981

5438	Glyoxalase family protein	fig|6666666.59329.peg.3614	fig|6666666.59330.peg.4026	fig|6666666.59331.peg.5312	fig|6666666.59332.peg.1112	fig|6666666.59334.peg.1608	fig|6666666.59335.peg.1421	fig|6666666.59336.peg.720	fig|6666666.59333.peg.3830	None	fig|6666666.59339.peg.226	None	fig|6666666.59337.peg.2939	fig|6666666.59342.peg.4118	None	None	fig|6666666.59345.peg.3907	None	None	fig|6666666.59346.peg.1853	fig|6666666.59348.peg.1807	None	fig|6666666.59350.peg.6749	fig|6666666.59351.peg.2876	None	None	fig|6666666.59354.peg.542

6550	3-oxoacyl-[acyl-carrier protein] reductase EC 1.1.1.100	fig|6666666.59329.peg.3768	fig|6666666.59330.peg.2610	None	None	None	fig|6666666.59335.peg.4296	fig|6666666.59336.peg.4076	fig|6666666.59333.peg.4045	fig|6666666.59338.peg.4877	fig|6666666.59339.peg.4638	None	fig|6666666.59337.peg.1471	fig|6666666.59342.peg.4308	None	None	fig|6666666.59345.peg.373	fig|6666666.59341.peg.1708	fig|6666666.59347.peg.1157	None	fig|6666666.59348.peg.4581	fig|6666666.59349.peg.2149	fig|6666666.59350.peg.3887	fig|6666666.59351.peg.3707	None	None	fig|6666666.59354.peg.1874

2190	FIG01199666 hypothetical protein	fig|6666666.59329.peg.3461	fig|6666666.59330.peg.1998	fig|6666666.59331.peg.5447	None	fig|6666666.59334.peg.2988	fig|6666666.59335.peg.543	fig|6666666.59336.peg.5547	fig|6666666.59333.peg.1518	fig|6666666.59338.peg.2942	fig|6666666.59339.peg.8421	fig|6666666.59340.peg.2929	fig|6666666.59337.peg.3812	fig|6666666.59342.peg.5224	None	None	fig|6666666.59345.peg.55	fig|6666666.59341.peg.361	fig|6666666.59347.peg.916	fig|6666666.59346.peg.1219	fig|6666666.59348.peg.4724	None	None	None	None	None	None

4390	Stress responsive alpha-beta barrel domain protein Dabb	fig|6666666.59329.peg.3043	fig|6666666.59330.peg.4293	fig|6666666.59331.peg.3778	fig|6666666.59332.peg.339	None	fig|6666666.59335.peg.659	fig|6666666.59336.peg.4228	None	fig|6666666.59338.peg.1036	fig|6666666.59339.peg.4218	fig|6666666.59340.peg.3585	fig|6666666.59337.peg.4085	fig|6666666.59342.peg.5117	None	None	fig|6666666.59345.peg.1681	fig|6666666.59341.peg.3604	fig|6666666.59347.peg.1824	None	fig|6666666.59348.peg.3175	None	fig|6666666.59350.peg.6570	None	None	fig|6666666.59355.peg.5967	None

3192	Phosphorelay protein LuxU	fig|6666666.59329.peg.4648	fig|6666666.59330.peg.1707	fig|6666666.59331.peg.2084	fig|6666666.59332.peg.1518	fig|6666666.59334.peg.294	fig|6666666.59335.peg.5491	fig|6666666.59336.peg.1322	fig|6666666.59333.peg.1334	fig|6666666.59338.peg.1627	fig|6666666.59339.peg.137	None	fig|6666666.59337.peg.4021	fig|6666666.59342.peg.867	None	None	fig|6666666.59345.peg.1116	fig|6666666.59341.peg.2170	fig|6666666.59347.peg.5161	fig|6666666.59346.peg.530	fig|6666666.59348.peg.714	None	None	None	None	None	None

5486	Homeodomain-like	fig|6666666.59329.peg.5523	fig|6666666.59330.peg.321	None	fig|6666666.59332.peg.3431	None	fig|6666666.59335.peg.868	fig|6666666.59336.peg.5408	fig|6666666.59333.peg.1438	fig|6666666.59338.peg.2850	fig|6666666.59339.peg.3250	fig|6666666.59340.peg.3730	fig|6666666.59337.peg.1049	fig|6666666.59342.peg.5415	None	None	fig|6666666.59345.peg.4233	fig|6666666.59341.peg.2672	fig|6666666.59347.peg.2638	fig|6666666.59346.peg.2074	fig|6666666.59348.peg.910	None	fig|6666666.59350.peg.3532	None	None	None	None

8116	FIG01199731 hypothetical protein	fig|6666666.59329.peg.6408	fig|6666666.59330.peg.2707	None	None	None	fig|6666666.59335.peg.4520	fig|6666666.59336.peg.4240	fig|6666666.59333.peg.5206	fig|6666666.59338.peg.1760	fig|6666666.59339.peg.2475	fig|6666666.59340.peg.3141	fig|6666666.59337.peg.2436	None	fig|6666666.71181.peg.1181	fig|6666666.59344.peg.2310	fig|6666666.59345.peg.613	fig|6666666.59341.peg.2369	fig|6666666.59347.peg.2259	None	None	None	fig|6666666.59350.peg.4612	None	fig|6666666.59353.peg.3404	fig|6666666.59355.peg.3502	None

416	Transcriptional regulator	fig|6666666.59329.peg.2865	None	fig|6666666.59331.peg.2151	None	None	fig|6666666.59335.peg.326	None	None	None	fig|6666666.59339.peg.8636	fig|6666666.59340.peg.1735	fig|6666666.59337.peg.5274	None	fig|6666666.71181.peg.4581	fig|6666666.59344.peg.5500	None	fig|6666666.59341.peg.1859	fig|6666666.59347.peg.4887	None	fig|6666666.59348.peg.761	fig|6666666.59349.peg.1208	fig|6666666.59350.peg.8408	fig|6666666.59351.peg.4904	fig|6666666.59353.peg.4475	fig|6666666.59355.peg.4895	fig|6666666.59354.peg.641

533	Putative analog of CcoH, C3198	fig|6666666.59329.peg.1341	fig|6666666.59330.peg.3192	None	fig|6666666.59332.peg.6156	fig|6666666.59334.peg.1462	fig|6666666.59335.peg.3536	fig|6666666.59336.peg.5991	fig|6666666.59333.peg.4307	fig|6666666.59338.peg.4742	fig|6666666.59339.peg.1323	None	fig|6666666.59337.peg.3724	fig|6666666.59342.peg.592	None	None	fig|6666666.59345.peg.3232	None	fig|6666666.59347.peg.1741	fig|6666666.59346.peg.1531	None	fig|6666666.59349.peg.140	None	fig|6666666.59351.peg.831	None	None	fig|6666666.59354.peg.200

8456	FIG01200665 hypothetical protein	fig|6666666.59329.peg.5932	fig|6666666.59330.peg.1980	fig|6666666.59331.peg.4822	None	fig|6666666.59334.peg.1633	fig|6666666.59335.peg.3405	fig|6666666.59336.peg.900	fig|6666666.59333.peg.955	None	fig|6666666.59339.peg.4323	None	fig|6666666.59337.peg.2605	fig|6666666.59342.peg.6124	fig|6666666.71181.peg.75	fig|6666666.59344.peg.3191	fig|6666666.59345.peg.752	fig|6666666.59341.peg.5440	None	None	fig|6666666.59348.peg.694	None	None	None	fig|6666666.59353.peg.1940	fig|6666666.59355.peg.1467	None

7048	hypothetical protein	fig|6666666.59329.peg.926	None	fig|6666666.59331.peg.1489	fig|6666666.59332.peg.2812	fig|6666666.59334.peg.5463	fig|6666666.59335.peg.671	fig|6666666.59336.peg.389	None	fig|6666666.59338.peg.5126	fig|6666666.59339.peg.1689	None	fig|6666666.59337.peg.3615	fig|6666666.59342.peg.4610	fig|6666666.71181.peg.2137	fig|6666666.59344.peg.3548	fig|6666666.59345.peg.1820	fig|6666666.59341.peg.5093	None	None	fig|6666666.59348.peg.2701	None	None	None	fig|6666666.59353.peg.2824	fig|6666666.59355.peg.834	None

8609	FIG01199649 hypothetical protein	fig|6666666.59329.peg.4300	fig|6666666.59330.peg.2525	fig|6666666.59331.peg.978	fig|6666666.59332.peg.4024	fig|6666666.59334.peg.4831	None	fig|6666666.59336.peg.3299	fig|6666666.59333.peg.2439	fig|6666666.59338.peg.4896	fig|6666666.59339.peg.4368	fig|6666666.59340.peg.2246	fig|6666666.59337.peg.2558	fig|6666666.59342.peg.4800	None	None	fig|6666666.59345.peg.3316	fig|6666666.59341.peg.1519	fig|6666666.59347.peg.1486	fig|6666666.59346.peg.4326	fig|6666666.59348.peg.5173	None	None	None	None	None	None

6989	Thymidylate kinase	None	fig|6666666.59330.peg.4455	fig|6666666.59331.peg.4806	fig|6666666.59332.peg.1875	fig|6666666.59334.peg.159	fig|6666666.59335.peg.335	fig|6666666.59336.peg.613	fig|6666666.59333.peg.875	fig|6666666.59338.peg.322	fig|6666666.59339.peg.8327	fig|6666666.59340.peg.427	fig|6666666.59337.peg.1724	fig|6666666.59342.peg.2486	None	None	fig|6666666.59345.peg.5360	fig|6666666.59341.peg.1432	fig|6666666.59347.peg.4911	fig|6666666.59346.peg.3208	fig|6666666.59348.peg.4245	None	None	None	None	None	None

5706	FIG01200136 hypothetical protein	fig|6666666.59329.peg.1356	fig|6666666.59330.peg.2997	fig|6666666.59331.peg.2299	fig|6666666.59332.peg.3230	fig|6666666.59334.peg.224	None	fig|6666666.59336.peg.331	fig|6666666.59333.peg.861	fig|6666666.59338.peg.1151	fig|6666666.59339.peg.7208	fig|6666666.59340.peg.2462	fig|6666666.59337.peg.3933	fig|6666666.59342.peg.2213	None	None	fig|6666666.59345.peg.4334	fig|6666666.59341.peg.5925	fig|6666666.59347.peg.2943	fig|6666666.59346.peg.2006	fig|6666666.59348.peg.1061	None	None	None	None	None	None

7823	DNA-binding response regulator, AraC family	fig|6666666.59329.peg.147	fig|6666666.59330.peg.2781	fig|6666666.59331.peg.4341	fig|6666666.59332.peg.665	None	fig|6666666.59335.peg.3599	fig|6666666.59336.peg.1731	fig|6666666.59333.peg.912	fig|6666666.59338.peg.1960	fig|6666666.59339.peg.2984	None	fig|6666666.59337.peg.4119	None	None	None	fig|6666666.59345.peg.4314	fig|6666666.59341.peg.1570	fig|6666666.59347.peg.4394	None	fig|6666666.59348.peg.1907	fig|6666666.59349.peg.4529	None	fig|6666666.59351.peg.4806	None	None	fig|6666666.59354.peg.3696

9365	Uncharacterized protein with LysM domain, C1652	fig|6666666.59329.peg.67	fig|6666666.59330.peg.647	fig|6666666.59331.peg.5420	fig|6666666.59332.peg.230	None	None	fig|6666666.59336.peg.1990	fig|6666666.59333.peg.1371	fig|6666666.59338.peg.2932	None	fig|6666666.59340.peg.2278	fig|6666666.59337.peg.3903	None	None	None	fig|6666666.59345.peg.4925	fig|6666666.59341.peg.3882	fig|6666666.59347.peg.4746	fig|6666666.59346.peg.1825	fig|6666666.59348.peg.5039	fig|6666666.59349.peg.2819	None	fig|6666666.59351.peg.5058	None	None	fig|6666666.59354.peg.1428

6532	FIG01201258 hypothetical protein	fig|6666666.59329.peg.3709	fig|6666666.59330.peg.3863	None	fig|6666666.59332.peg.2934	fig|6666666.59334.peg.5111	fig|6666666.59335.peg.2925	fig|6666666.59336.peg.3333	None	fig|6666666.59338.peg.3621	fig|6666666.59339.peg.1592	None	fig|6666666.59337.peg.1930	None	fig|6666666.71181.peg.1008	fig|6666666.59344.peg.1785	fig|6666666.59345.peg.4022	fig|6666666.59341.peg.1643	fig|6666666.59347.peg.4453	fig|6666666.59346.peg.5305	None	None	None	None	fig|6666666.59353.peg.2325	fig|6666666.59355.peg.1599	None

4165	Predicted transporter component	fig|6666666.59329.peg.5951	fig|6666666.59330.peg.769	fig|6666666.59331.peg.3415	fig|6666666.59332.peg.345	fig|6666666.59334.peg.5513	fig|6666666.59335.peg.5233	fig|6666666.59336.peg.2635	fig|6666666.59333.peg.2775	None	fig|6666666.59339.peg.2788	fig|6666666.59340.peg.3487	fig|6666666.59337.peg.5360	fig|6666666.59342.peg.1408	None	None	fig|6666666.59345.peg.46	None	fig|6666666.59347.peg.910	fig|6666666.59346.peg.402	fig|6666666.59348.peg.4218	None	fig|6666666.59350.peg.1476	None	None	None	None

7021	FIG01200315 hypothetical protein	fig|6666666.59329.peg.331	fig|6666666.59330.peg.2838	fig|6666666.59331.peg.3771	fig|6666666.59332.peg.6002	None	fig|6666666.59335.peg.3983	fig|6666666.59336.peg.1361	fig|6666666.59333.peg.5321	None	fig|6666666.59339.peg.8908	None	fig|6666666.59337.peg.2035	fig|6666666.59342.peg.3103	None	None	fig|6666666.59345.peg.2439	None	None	fig|6666666.59346.peg.563	fig|6666666.59348.peg.3600	fig|6666666.59349.peg.590	fig|6666666.59350.peg.443	fig|6666666.59351.peg.526	None	None	fig|6666666.59354.peg.4884

2615	Iron-sulfur cluster assembly protein SufD	fig|6666666.59329.peg.3532	None	fig|6666666.59331.peg.6331	fig|6666666.59332.peg.4505	fig|6666666.59334.peg.1174	fig|6666666.59335.peg.4548	fig|6666666.59336.peg.202	None	fig|6666666.59338.peg.3859	fig|6666666.59339.peg.2177	None	None	fig|6666666.59342.peg.1846	fig|6666666.71181.peg.2559	fig|6666666.59344.peg.745	fig|6666666.59345.peg.3616	None	fig|6666666.59347.peg.2746	None	None	fig|6666666.59349.peg.4902	fig|6666666.59350.peg.7503	None	fig|6666666.59353.peg.1357	fig|6666666.59355.peg.2515	None

2613	FIG01202709 hypothetical protein	fig|6666666.59329.peg.5698	fig|6666666.59330.peg.2857	None	fig|6666666.59332.peg.1921	fig|6666666.59334.peg.3291	fig|6666666.59335.peg.5431	fig|6666666.59336.peg.6068	fig|6666666.59333.peg.4427	fig|6666666.59338.peg.2330	fig|6666666.59339.peg.2051	fig|6666666.59340.peg.1405	fig|6666666.59337.peg.1957	fig|6666666.59342.peg.2220	None	None	fig|6666666.59345.peg.4061	fig|6666666.59341.peg.1216	fig|6666666.59347.peg.3639	fig|6666666.59346.peg.4232	fig|6666666.59348.peg.2755	None	None	None	None	None	None

5794	ABC transporter, permease protein YnjC	fig|6666666.59329.peg.5460	fig|6666666.59330.peg.3573	fig|6666666.59331.peg.2159	fig|6666666.59332.peg.2235	fig|6666666.59334.peg.3578	fig|6666666.59335.peg.5268	fig|6666666.59336.peg.4392	fig|6666666.59333.peg.1206	fig|6666666.59338.peg.1986	fig|6666666.59339.peg.550	None	fig|6666666.59337.peg.3668	fig|6666666.59342.peg.4645	None	None	fig|6666666.59345.peg.1246	fig|6666666.59341.peg.1092	fig|6666666.59347.peg.3455	fig|6666666.59346.peg.3493	fig|6666666.59348.peg.1148	None	None	None	None	None	None

6667	Formate dehydrogenase subunit or accessory protein	fig|6666666.59329.peg.4873	fig|6666666.59330.peg.2591	fig|6666666.59331.peg.1228	fig|6666666.59332.peg.5370	fig|6666666.59334.peg.5219	fig|6666666.59335.peg.2192	fig|6666666.59336.peg.3296	fig|6666666.59333.peg.4541	fig|6666666.59338.peg.1310	fig|6666666.59339.peg.1284	None	fig|6666666.59337.peg.3700	fig|6666666.59342.peg.211	None	None	fig|6666666.59345.peg.3211	fig|6666666.59341.peg.2897	fig|6666666.59347.peg.1719	fig|6666666.59346.peg.3284	fig|6666666.59348.peg.5315	None	None	None	None	None	None

882	FIG01202974 hypothetical protein	fig|6666666.59329.peg.3428	fig|6666666.59330.peg.34	None	None	fig|6666666.59334.peg.5225	fig|6666666.59335.peg.243	fig|6666666.59336.peg.338	fig|6666666.59333.peg.248	fig|6666666.59338.peg.86	fig|6666666.59339.peg.8154	fig|6666666.59340.peg.922	fig|6666666.59337.peg.819	fig|6666666.59342.peg.4280	None	None	fig|6666666.59345.peg.4872	fig|6666666.59341.peg.3523	fig|6666666.59347.peg.4587	None	None	fig|6666666.59349.peg.4755	None	fig|6666666.59351.peg.712	None	None	fig|6666666.59354.peg.4759

6864	Inorganic pyrophosphatase EC 3.6.1.1	fig|6666666.59329.peg.1779	fig|6666666.59330.peg.840	None	None	fig|6666666.59334.peg.2598	fig|6666666.59335.peg.177	fig|6666666.59336.peg.921	fig|6666666.59333.peg.573	fig|6666666.59338.peg.305	fig|6666666.59339.peg.7727	None	fig|6666666.59337.peg.333	fig|6666666.59342.peg.5992	fig|6666666.71181.peg.2805	fig|6666666.59344.peg.2130	fig|6666666.59345.peg.2483	fig|6666666.59341.peg.5006	fig|6666666.59347.peg.4871	None	None	None	None	None	fig|6666666.59353.peg.3163	fig|6666666.59355.peg.434	None

9580	Predicted ATPase	fig|6666666.59329.peg.357	fig|6666666.59330.peg.405	None	fig|6666666.59332.peg.2114	None	fig|6666666.59335.peg.2260	fig|6666666.59336.peg.2708	fig|6666666.59333.peg.3738	fig|6666666.59338.peg.5946	fig|6666666.59339.peg.831	fig|6666666.59340.peg.606	fig|6666666.59337.peg.990	fig|6666666.59342.peg.6227	None	None	fig|6666666.59345.peg.2689	fig|6666666.59341.peg.5315	fig|6666666.59347.peg.4454	fig|6666666.59346.peg.1088	fig|6666666.59348.peg.676	None	fig|6666666.59350.peg.59	None	None	None	None

1268	FIG00919732 hypothetical protein	fig|6666666.59329.peg.1792	fig|6666666.59330.peg.1557	fig|6666666.59331.peg.5287	fig|6666666.59332.peg.2708	fig|6666666.59334.peg.3682	fig|6666666.59335.peg.6063	fig|6666666.59336.peg.638	fig|6666666.59333.peg.3186	fig|6666666.59338.peg.2862	fig|6666666.59339.peg.8558	None	fig|6666666.59337.peg.1640	fig|6666666.59342.peg.4796	None	None	fig|6666666.59345.peg.5479	fig|6666666.59341.peg.5359	fig|6666666.59347.peg.5062	fig|6666666.59346.peg.3704	fig|6666666.59348.peg.2611	None	None	None	None	None	None

8515	FIG00919628 hypothetical protein	fig|6666666.59329.peg.1669	fig|6666666.59330.peg.4790	fig|6666666.59331.peg.3293	fig|6666666.59332.peg.393	None	fig|6666666.59335.peg.1692	fig|6666666.59336.peg.6386	fig|6666666.59333.peg.4995	fig|6666666.59338.peg.5319	fig|6666666.59339.peg.7264	fig|6666666.59340.peg.296	fig|6666666.59337.peg.4979	fig|6666666.59342.peg.2189	None	None	fig|6666666.59345.peg.3566	fig|6666666.59341.peg.4067	fig|6666666.59347.peg.4336	fig|6666666.59346.peg.3606	fig|6666666.59348.peg.5383	None	None	None	None	None	None

9228	Phenylalanine-4-hydroxylase EC 1.14.16.1	fig|6666666.59329.peg.6253	fig|6666666.59330.peg.912	fig|6666666.59331.peg.1074	fig|6666666.59332.peg.4268	None	fig|6666666.59335.peg.995	None	fig|6666666.59333.peg.4639	fig|6666666.59338.peg.5301	fig|6666666.59339.peg.4452	None	fig|6666666.59337.peg.5588	fig|6666666.59342.peg.6218	None	None	fig|6666666.59345.peg.426	fig|6666666.59341.peg.3310	fig|6666666.59347.peg.520	fig|6666666.59346.peg.142	None	fig|6666666.59349.peg.2081	None	fig|6666666.59351.peg.2594	None	None	fig|6666666.59354.peg.3554

9221	FIG01201621 hypothetical protein	fig|6666666.59329.peg.5496	fig|6666666.59330.peg.1476	fig|6666666.59331.peg.5531	fig|6666666.59332.peg.6187	None	fig|6666666.59335.peg.5808	fig|6666666.59336.peg.3193	fig|6666666.59333.peg.1220	fig|6666666.59338.peg.3364	fig|6666666.59339.peg.2101	fig|6666666.59340.peg.733	fig|6666666.59337.peg.200	fig|6666666.59342.peg.5998	None	None	fig|6666666.59345.peg.4529	fig|6666666.59341.peg.317	fig|6666666.59347.peg.3681	fig|6666666.59346.peg.5141	fig|6666666.59348.peg.5184	None	None	None	None	None	None

5763	FIG01201095 hypothetical protein	fig|6666666.59329.peg.1639	fig|6666666.59330.peg.3010	fig|6666666.59331.peg.3387	fig|6666666.59332.peg.584	None	fig|6666666.59335.peg.3528	fig|6666666.59336.peg.5735	fig|6666666.59333.peg.4625	fig|6666666.59338.peg.2127	fig|6666666.59339.peg.4057	None	fig|6666666.59337.peg.222	fig|6666666.59342.peg.5098	None	None	fig|6666666.59345.peg.5277	None	fig|6666666.59347.peg.2275	fig|6666666.59346.peg.158	fig|6666666.59348.peg.2462	fig|6666666.59349.peg.687	None	None	None	None	fig|6666666.59354.peg.5324

6592	Flagellar biosynthesis protein FlgN	fig|6666666.59329.peg.571	fig|6666666.59330.peg.2695	None	fig|6666666.59332.peg.1801	None	fig|6666666.59335.peg.2075	fig|6666666.59336.peg.1969	fig|6666666.59333.peg.202	fig|6666666.59338.peg.625	fig|6666666.59339.peg.5306	None	fig|6666666.59337.peg.3341	None	None	None	fig|6666666.59345.peg.5444	fig|6666666.59341.peg.4563	fig|6666666.59347.peg.2719	fig|6666666.59346.peg.1757	fig|6666666.59348.peg.4696	fig|6666666.59349.peg.716	None	fig|6666666.59351.peg.2564	None	None	fig|6666666.59354.peg.1518

6034	ABC-type multidrug transport system, ATPase and permease component	fig|6666666.59329.peg.1020	fig|6666666.59330.peg.1072	fig|6666666.59331.peg.2971	fig|6666666.59332.peg.3750	fig|6666666.59334.peg.2788	fig|6666666.59335.peg.2674	fig|6666666.59336.peg.2722	fig|6666666.59333.peg.2826	fig|6666666.59338.peg.4137	fig|6666666.59339.peg.2676	None	fig|6666666.59337.peg.5649	fig|6666666.59342.peg.448	None	None	fig|6666666.59345.peg.3552	fig|6666666.59341.peg.1195	fig|6666666.59347.peg.2757	fig|6666666.59346.peg.3763	fig|6666666.59348.peg.3512	None	None	None	None	None	None

2908	Choloylglycine hydrolase EC 3.5.1.24	fig|6666666.59329.peg.6233	None	None	fig|6666666.59332.peg.3218	fig|6666666.59334.peg.1726	None	fig|6666666.59336.peg.5450	None	fig|6666666.59338.peg.5013	None	fig|6666666.59340.peg.3143	fig|6666666.59337.peg.4698	fig|6666666.59342.peg.4817	fig|6666666.71181.peg.5739	None	fig|6666666.59345.peg.3101	fig|6666666.59341.peg.1888	fig|6666666.59347.peg.999	None	fig|6666666.59348.peg.1296	fig|6666666.59349.peg.4028	None	fig|6666666.59351.peg.2777	None	fig|6666666.59355.peg.5185	fig|6666666.59354.peg.1016

11085	Histone acetyltransferase HPA2 and related acetyltransferases	fig|6666666.59329.peg.2988	fig|6666666.59330.peg.2070	fig|6666666.59331.peg.149	fig|6666666.59332.peg.5432	None	fig|6666666.59335.peg.4029	fig|6666666.59336.peg.1909	fig|6666666.59333.peg.5259	fig|6666666.59338.peg.5862	fig|6666666.59339.peg.3822	None	fig|6666666.59337.peg.3559	fig|6666666.59342.peg.740	None	None	fig|6666666.59345.peg.835	fig|6666666.59341.peg.1284	fig|6666666.59347.peg.1670	fig|6666666.59346.peg.444	fig|6666666.59348.peg.4598	None	None	fig|6666666.59351.peg.5107	None	None	None

4652	FIG01199685 hypothetical protein	fig|6666666.59329.peg.1382	fig|6666666.59330.peg.2519	fig|6666666.59331.peg.2739	fig|6666666.59332.peg.1087	fig|6666666.59334.peg.2853	fig|6666666.59335.peg.1001	fig|6666666.59336.peg.6079	fig|6666666.59333.peg.2505	fig|6666666.59338.peg.5736	fig|6666666.59339.peg.7475	fig|6666666.59340.peg.1169	fig|6666666.59337.peg.140	fig|6666666.59342.peg.2142	None	None	fig|6666666.59345.peg.3702	fig|6666666.59341.peg.98	fig|6666666.59347.peg.4039	fig|6666666.59346.peg.245	None	None	None	None	None	None	None

4658	General secretion pathway protein K	None	fig|6666666.59330.peg.424	None	fig|6666666.59332.peg.2996	fig|6666666.59334.peg.121	fig|6666666.59335.peg.711	fig|6666666.59336.peg.3115	fig|6666666.59333.peg.512	fig|6666666.59338.peg.1432	fig|6666666.59339.peg.6208	fig|6666666.59340.peg.722	None	None	None	None	fig|6666666.59345.peg.2670	fig|6666666.59341.peg.4544	fig|6666666.59347.peg.4470	fig|6666666.59346.peg.2227	fig|6666666.59348.peg.1390	fig|6666666.59349.peg.2168	None	fig|6666666.59351.peg.4340	None	None	fig|6666666.59354.peg.704

7264	FIG139976 hypothetical protein	fig|6666666.59329.peg.3558	fig|6666666.59330.peg.2479	fig|6666666.59331.peg.6308	fig|6666666.59332.peg.4611	fig|6666666.59334.peg.4840	fig|6666666.59335.peg.947	fig|6666666.59336.peg.2789	fig|6666666.59333.peg.1053	fig|6666666.59338.peg.4020	fig|6666666.59339.peg.331	None	fig|6666666.59337.peg.4541	fig|6666666.59342.peg.1784	None	None	fig|6666666.59345.peg.1524	fig|6666666.59341.peg.2246	fig|6666666.59347.peg.4189	fig|6666666.59346.peg.4180	fig|6666666.59348.peg.1591	None	None	None	None	None	None

8839	Hypotehtical protein in Cytochrome oxidase biogenesis cluster	None	fig|6666666.59330.peg.3216	fig|6666666.59331.peg.610	fig|6666666.59332.peg.3955	fig|6666666.59334.peg.1000	fig|6666666.59335.peg.2865	None	None	fig|6666666.59338.peg.5935	fig|6666666.59339.peg.6632	None	fig|6666666.59337.peg.5080	fig|6666666.59342.peg.5322	None	None	fig|6666666.59345.peg.5619	fig|6666666.59341.peg.5974	fig|6666666.59347.peg.1547	fig|6666666.59346.peg.4310	fig|6666666.59348.peg.1805	fig|6666666.59349.peg.3504	None	fig|6666666.59351.peg.4384	None	None	fig|6666666.59354.peg.2071

3524	FIG01200297 hypothetical protein	fig|6666666.59329.peg.2845	fig|6666666.59330.peg.4804	fig|6666666.59331.peg.3626	fig|6666666.59332.peg.5589	None	fig|6666666.59335.peg.4990	fig|6666666.59336.peg.5627	fig|6666666.59333.peg.3322	fig|6666666.59338.peg.2335	fig|6666666.59339.peg.4694	None	fig|6666666.59337.peg.1189	fig|6666666.59342.peg.4922	None	None	fig|6666666.59345.peg.8	fig|6666666.59341.peg.3024	fig|6666666.59347.peg.855	fig|6666666.59346.peg.4991	fig|6666666.59348.peg.5544	None	fig|6666666.59350.peg.2298	None	None	None	None

1362	FIG001587 exported protein	fig|6666666.59329.peg.6310	fig|6666666.59330.peg.2301	None	fig|6666666.59332.peg.2682	None	fig|6666666.59335.peg.6024	fig|6666666.59336.peg.5128	fig|6666666.59333.peg.3313	None	fig|6666666.59339.peg.3744	None	fig|6666666.59337.peg.1774	fig|6666666.59342.peg.3409	fig|6666666.71181.peg.4360	fig|6666666.59344.peg.2776	fig|6666666.59345.peg.870	fig|6666666.59341.peg.5464	None	fig|6666666.59346.peg.1682	None	None	fig|6666666.59350.peg.579	None	fig|6666666.59353.peg.1667	fig|6666666.59355.peg.1054	None

7205	Carboxynorspermidine dehydrogenase	fig|6666666.59329.peg.966	fig|6666666.59330.peg.2946	None	fig|6666666.59332.peg.2223	fig|6666666.59334.peg.4485	fig|6666666.59335.peg.1328	fig|6666666.59336.peg.1779	fig|6666666.59333.peg.5195	fig|6666666.59338.peg.3040	fig|6666666.59339.peg.932	None	fig|6666666.59337.peg.1745	fig|6666666.59342.peg.2552	None	None	fig|6666666.59345.peg.900	fig|6666666.59341.peg.386	fig|6666666.59347.peg.2286	fig|6666666.59346.peg.1357	fig|6666666.59348.peg.4165	None	None	None	None	fig|6666666.59355.peg.5186	None

6428	hypothetical protein	fig|6666666.59329.peg.2949	fig|6666666.59330.peg.3265	fig|6666666.59331.peg.6230	fig|6666666.59332.peg.3442	None	fig|6666666.59335.peg.2814	fig|6666666.59336.peg.2136	None	fig|6666666.59338.peg.677	fig|6666666.59339.peg.1957	None	fig|6666666.59337.peg.1236	None	None	None	fig|6666666.59345.peg.4421	fig|6666666.59341.peg.2035	fig|6666666.59347.peg.391	fig|6666666.59346.peg.2194	None	fig|6666666.59349.peg.4412	fig|6666666.59350.peg.6633	fig|6666666.59351.peg.5314	None	None	fig|6666666.59354.peg.2029

10687	Anaerobic C4-dicarboxylate transporter DcuC	fig|6666666.59329.peg.1387	fig|6666666.59330.peg.3297	None	None	fig|6666666.59334.peg.1181	fig|6666666.59335.peg.2345	fig|6666666.59336.peg.5338	fig|6666666.59333.peg.3435	None	fig|6666666.59339.peg.2868	None	fig|6666666.59337.peg.2111	None	fig|6666666.71181.peg.210	fig|6666666.59344.peg.260	fig|6666666.59345.peg.3348	fig|6666666.59341.peg.2268	fig|6666666.59347.peg.1504	fig|6666666.59346.peg.510	fig|6666666.59348.peg.3530	None	None	None	fig|6666666.59353.peg.611	fig|6666666.59355.peg.93	None

9958	FIG01200262 hypothetical protein	fig|6666666.59329.peg.143	fig|6666666.59330.peg.828	None	fig|6666666.59332.peg.5687	None	fig|6666666.59335.peg.235	fig|6666666.59336.peg.4639	fig|6666666.59333.peg.554	fig|6666666.59338.peg.1821	fig|6666666.59339.peg.8165	fig|6666666.59340.peg.3019	fig|6666666.59337.peg.1372	fig|6666666.59342.peg.3941	None	None	fig|6666666.59345.peg.2470	fig|6666666.59341.peg.1990	fig|6666666.59347.peg.3099	fig|6666666.59346.peg.3985	fig|6666666.59348.peg.564	None	None	fig|6666666.59351.peg.1836	None	None	None

403	FIG00920311 hypothetical protein	fig|6666666.59329.peg.1781	fig|6666666.59330.peg.842	None	fig|6666666.59332.peg.5110	fig|6666666.59334.peg.60	fig|6666666.59335.peg.176	fig|6666666.59336.peg.924	fig|6666666.59333.peg.575	None	fig|6666666.59339.peg.7726	None	fig|6666666.59337.peg.336	fig|6666666.59342.peg.4404	fig|6666666.71181.peg.2806	fig|6666666.59344.peg.2131	fig|6666666.59345.peg.2486	None	None	fig|6666666.59346.peg.1979	fig|6666666.59348.peg.2841	None	None	None	fig|6666666.59353.peg.3164	fig|6666666.59355.peg.433	None

10325	FIG01207143 hypothetical protein	fig|6666666.59329.peg.6366	fig|6666666.59330.peg.2040	None	fig|6666666.59332.peg.4366	fig|6666666.59334.peg.2664	fig|6666666.59335.peg.5466	fig|6666666.59336.peg.4487	fig|6666666.59333.peg.2612	None	fig|6666666.59339.peg.959	None	fig|6666666.59337.peg.4310	None	fig|6666666.71181.peg.1570	fig|6666666.59344.peg.2016	fig|6666666.59345.peg.3902	fig|6666666.59341.peg.4312	fig|6666666.59347.peg.5298	fig|6666666.59346.peg.1519	None	None	None	None	fig|6666666.59353.peg.2909	fig|6666666.59355.peg.2830	None

7979	FIG01199673 hypothetical protein	None	None	fig|6666666.59331.peg.5463	fig|6666666.59332.peg.4849	None	fig|6666666.59335.peg.4288	None	None	fig|6666666.59338.peg.2657	fig|6666666.59339.peg.447	fig|6666666.59340.peg.3722	None	None	fig|6666666.71181.peg.1260	fig|6666666.59344.peg.143	None	fig|6666666.59341.peg.4926	fig|6666666.59347.peg.1349	fig|6666666.59346.peg.2203	fig|6666666.59348.peg.3379	fig|6666666.59349.peg.2885	None	fig|6666666.59351.peg.869	fig|6666666.59353.peg.457	fig|6666666.59355.peg.2184	fig|6666666.59354.peg.3064

9725	Rho-specific inhibitor of transcription termination YaeO	fig|6666666.59329.peg.5711	fig|6666666.59330.peg.1628	fig|6666666.59331.peg.6013	fig|6666666.59332.peg.5137	None	fig|6666666.59335.peg.661	fig|6666666.59336.peg.743	fig|6666666.59333.peg.5516	None	fig|6666666.59339.peg.4216	None	fig|6666666.59337.peg.1273	None	None	None	fig|6666666.59345.peg.4459	fig|6666666.59341.peg.2916	fig|6666666.59347.peg.1707	fig|6666666.59346.peg.3170	fig|6666666.59348.peg.1729	fig|6666666.59349.peg.5048	None	fig|6666666.59351.peg.967	None	None	fig|6666666.59354.peg.5158

488	Pterin-4-alpha-carbinolamine dehydratase EC 4.2.1.96	fig|6666666.59329.peg.6254	fig|6666666.59330.peg.910	fig|6666666.59331.peg.1075	fig|6666666.59332.peg.4269	None	fig|6666666.59335.peg.994	fig|6666666.59336.peg.4668	None	fig|6666666.59338.peg.5303	fig|6666666.59339.peg.4453	None	fig|6666666.59337.peg.5587	None	None	None	fig|6666666.59345.peg.425	fig|6666666.59341.peg.3311	fig|6666666.59347.peg.521	fig|6666666.59346.peg.143	None	fig|6666666.59349.peg.2080	fig|6666666.59350.peg.6094	fig|6666666.59351.peg.2593	None	None	fig|6666666.59354.peg.3555

6461	Integral membrane protein	fig|6666666.59329.peg.6363	fig|6666666.59330.peg.1917	fig|6666666.59331.peg.757	fig|6666666.59332.peg.1412	fig|6666666.59334.peg.3596	fig|6666666.59335.peg.2970	fig|6666666.59336.peg.4160	fig|6666666.59333.peg.3685	fig|6666666.59338.peg.947	fig|6666666.59339.peg.4568	fig|6666666.59340.peg.2505	fig|6666666.59337.peg.4854	fig|6666666.59342.peg.4362	None	None	fig|6666666.59345.peg.201	fig|6666666.59341.peg.4725	fig|6666666.59347.peg.623	fig|6666666.59346.peg.1502	None	None	None	None	None	None	None

3929	Nicotinate-nucleotide--dimethylbenzimidazole phosphoribosyltransferase EC 2.4.2.21	fig|6666666.59329.peg.5305	fig|6666666.59330.peg.2664	fig|6666666.59331.peg.1671	fig|6666666.59332.peg.3375	None	fig|6666666.59335.peg.2166	fig|6666666.59336.peg.6346	fig|6666666.59333.peg.1786	fig|6666666.59338.peg.3174	fig|6666666.59339.peg.3904	None	fig|6666666.59337.peg.1978	None	None	None	fig|6666666.59345.peg.4672	fig|6666666.59341.peg.549	fig|6666666.59347.peg.3355	fig|6666666.59346.peg.2033	fig|6666666.59348.peg.4809	None	fig|6666666.59350.peg.295	fig|6666666.59351.peg.3919	None	None	None

6090	Zn-dependent hydrolase EC 3.-.-.-	fig|6666666.59329.peg.1210	fig|6666666.59330.peg.1658	fig|6666666.59331.peg.2911	None	None	fig|6666666.59335.peg.5047	fig|6666666.59336.peg.2695	fig|6666666.59333.peg.5302	None	fig|6666666.59339.peg.970	None	fig|6666666.59337.peg.1516	fig|6666666.59342.peg.1107	None	None	fig|6666666.59345.peg.3031	fig|6666666.59341.peg.736	fig|6666666.59347.peg.3432	fig|6666666.59346.peg.2777	fig|6666666.59348.peg.453	fig|6666666.59349.peg.4266	None	fig|6666666.59351.peg.5279	None	None	fig|6666666.59354.peg.1602

3586	Transcriptional regulator, MerR family	fig|6666666.59329.peg.2929	None	fig|6666666.59331.peg.2067	fig|6666666.59332.peg.5579	fig|6666666.59334.peg.2084	fig|6666666.59335.peg.4430	fig|6666666.59336.peg.2463	None	fig|6666666.59338.peg.5601	fig|6666666.59339.peg.2200	fig|6666666.59340.peg.3099	fig|6666666.59337.peg.5768	fig|6666666.59342.peg.4514	None	None	fig|6666666.59345.peg.3992	fig|6666666.59341.peg.3589	fig|6666666.59347.peg.1436	fig|6666666.59346.peg.4015	fig|6666666.59348.peg.2206	None	None	fig|6666666.59351.peg.319	None	None	None

4220	L-lactate permease	fig|6666666.59329.peg.4455	None	None	fig|6666666.59332.peg.1782	None	fig|6666666.59335.peg.5282	fig|6666666.59336.peg.4718	fig|6666666.59333.peg.3627	fig|6666666.59338.peg.3214	fig|6666666.59339.peg.2613	None	None	fig|6666666.59342.peg.3190	fig|6666666.71181.peg.124	fig|6666666.59344.peg.3643	fig|6666666.59345.peg.2817	fig|6666666.59341.peg.3374	fig|6666666.59347.peg.632	fig|6666666.59346.peg.3623	None	None	None	fig|6666666.59351.peg.5072	fig|6666666.59353.peg.3865	fig|6666666.59355.peg.1520	None

5214	FIG01199621 hypothetical protein	None	None	fig|6666666.59331.peg.1608	None	None	fig|6666666.59335.peg.4666	None	fig|6666666.59333.peg.4665	fig|6666666.59338.peg.3181	fig|6666666.59339.peg.1615	None	None	None	fig|6666666.71181.peg.2026	fig|6666666.59344.peg.2709	None	fig|6666666.59341.peg.5149	fig|6666666.59347.peg.4686	fig|6666666.59346.peg.1126	fig|6666666.59348.peg.4100	fig|6666666.59349.peg.2056	fig|6666666.59350.peg.1224	fig|6666666.59351.peg.4291	fig|6666666.59353.peg.3023	fig|6666666.59355.peg.2930	fig|6666666.59354.peg.733

5024	Putative outer membrane lipoprotein YmcA	fig|6666666.59329.peg.829	fig|6666666.59330.peg.1353	fig|6666666.59331.peg.3176	fig|6666666.59332.peg.2134	fig|6666666.59334.peg.3593	None	fig|6666666.59336.peg.79	fig|6666666.59333.peg.475	fig|6666666.59338.peg.1228	fig|6666666.59339.peg.6402	fig|6666666.59340.peg.246	fig|6666666.59337.peg.442	fig|6666666.59342.peg.1935	None	None	fig|6666666.59345.peg.2591	None	fig|6666666.59347.peg.5170	fig|6666666.59346.peg.4837	fig|6666666.59348.peg.1535	None	fig|6666666.59350.peg.820	None	None	None	None

1537	Mannitol-1-phosphate 5-dehydrogenase EC 1.1.1.17	fig|6666666.59329.peg.6093	None	None	fig|6666666.59332.peg.2454	fig|6666666.59334.peg.920	fig|6666666.59335.peg.1286	fig|6666666.59336.peg.2728	fig|6666666.59333.peg.4979	fig|6666666.59338.peg.149	None	fig|6666666.59340.peg.1280	None	fig|6666666.59342.peg.2783	fig|6666666.71181.peg.663	fig|6666666.59344.peg.4126	fig|6666666.59345.peg.3866	fig|6666666.59341.peg.2827	fig|6666666.59347.peg.234	fig|6666666.59346.peg.4437	None	None	None	None	fig|6666666.59353.peg.4106	fig|6666666.59355.peg.2334	None

3464	FIG01199551 hypothetical protein	fig|6666666.59329.peg.3623	fig|6666666.59330.peg.3393	fig|6666666.59331.peg.2869	fig|6666666.59332.peg.4394	fig|6666666.59334.peg.2987	fig|6666666.59335.peg.4531	fig|6666666.59336.peg.5550	fig|6666666.59333.peg.1910	fig|6666666.59338.peg.5931	fig|6666666.59339.peg.3545	None	fig|6666666.59337.peg.3514	fig|6666666.59342.peg.6274	None	None	fig|6666666.59345.peg.932	fig|6666666.59341.peg.3618	fig|6666666.59347.peg.2322	fig|6666666.59346.peg.3899	fig|6666666.59348.peg.3617	None	None	None	None	None	None

946	FIG028593 membrane protein	fig|6666666.59329.peg.1526	fig|6666666.59330.peg.4484	fig|6666666.59331.peg.825	fig|6666666.59332.peg.476	fig|6666666.59334.peg.5437	fig|6666666.59335.peg.4117	fig|6666666.59336.peg.621	fig|6666666.59333.peg.797	fig|6666666.59338.peg.4911	fig|6666666.59339.peg.8538	fig|6666666.59340.peg.3157	fig|6666666.59337.peg.5861	None	None	None	fig|6666666.59345.peg.5493	fig|6666666.59341.peg.3299	fig|6666666.59347.peg.678	fig|6666666.59346.peg.610	fig|6666666.59348.peg.4893	None	None	None	None	None	None

328	Fucose 4-O-acetylase	fig|6666666.59329.peg.2362	fig|6666666.59330.peg.1493	None	fig|6666666.59332.peg.2059	fig|6666666.59334.peg.1301	fig|6666666.59335.peg.746	fig|6666666.59336.peg.1168	fig|6666666.59333.peg.1734	fig|6666666.59338.peg.2517	fig|6666666.59339.peg.5606	fig|6666666.59340.peg.3744	fig|6666666.59337.peg.184	fig|6666666.59342.peg.4447	None	None	fig|6666666.59345.peg.4513	fig|6666666.59341.peg.3329	fig|6666666.59347.peg.3693	fig|6666666.59346.peg.4237	fig|6666666.59348.peg.4016	None	None	None	None	None	None

6178	FIG01200359 hypothetical protein	fig|6666666.59329.peg.46	fig|6666666.59330.peg.627	fig|6666666.59331.peg.3341	fig|6666666.59332.peg.213	None	fig|6666666.59335.peg.2447	fig|6666666.59336.peg.2734	fig|6666666.59333.peg.8	fig|6666666.59338.peg.571	fig|6666666.59339.peg.8071	fig|6666666.59340.peg.529	fig|6666666.59337.peg.3886	fig|6666666.59342.peg.4810	None	None	fig|6666666.59345.peg.4942	fig|6666666.59341.peg.1938	fig|6666666.59347.peg.4762	fig|6666666.59346.peg.4408	fig|6666666.59348.peg.65	None	None	None	None	None	None

4885	Believed to be involved in assembly of Fe-S clusters	None	fig|6666666.59330.peg.1420	None	fig|6666666.59332.peg.1335	None	fig|6666666.59335.peg.2374	fig|6666666.59336.peg.674	fig|6666666.59333.peg.931	fig|6666666.59338.peg.5772	None	fig|6666666.59340.peg.3044	fig|6666666.59337.peg.745	fig|6666666.59342.peg.3303	fig|6666666.71181.peg.1763	fig|6666666.59344.peg.1635	fig|6666666.59345.peg.3646	fig|6666666.59341.peg.6094	fig|6666666.59347.peg.4289	fig|6666666.59346.peg.5152	None	None	None	None	fig|6666666.59353.peg.2701	fig|6666666.59355.peg.1778	None

2474	FIG01200711 hypothetical protein	fig|6666666.59329.peg.654	fig|6666666.59330.peg.1888	None	fig|6666666.59332.peg.2834	fig|6666666.59334.peg.2614	fig|6666666.59335.peg.3139	fig|6666666.59336.peg.779	fig|6666666.59333.peg.4838	fig|6666666.59338.peg.243	fig|6666666.59339.peg.5443	fig|6666666.59340.peg.711	fig|6666666.59337.peg.236	fig|6666666.59342.peg.3612	None	None	fig|6666666.59345.peg.5264	fig|6666666.59341.peg.3815	fig|6666666.59347.peg.12	fig|6666666.59346.peg.4489	fig|6666666.59348.peg.2046	None	None	None	None	None	None

5525	Gamma-D-Glutamyl-meso-Diaminopimelate Amidase	fig|6666666.59329.peg.1709	fig|6666666.59330.peg.1908	fig|6666666.59331.peg.5825	fig|6666666.59332.peg.6229	fig|6666666.59334.peg.4441	None	fig|6666666.59336.peg.1319	fig|6666666.59333.peg.3503	fig|6666666.59338.peg.3596	None	None	fig|6666666.59337.peg.255	None	fig|6666666.71181.peg.1380	fig|6666666.59344.peg.543	fig|6666666.59345.peg.5245	fig|6666666.59341.peg.5553	fig|6666666.59347.peg.2532	fig|6666666.59346.peg.3363	None	None	None	None	fig|6666666.59353.peg.1848	fig|6666666.59355.peg.1165	None

10099	Transcription regulator [contains diacylglycerol kinase catalytic domain]	fig|6666666.59329.peg.5045	None	fig|6666666.59331.peg.57	None	fig|6666666.59334.peg.5299	None	fig|6666666.59336.peg.1525	fig|6666666.59333.peg.4716	fig|6666666.59338.peg.3539	None	None	fig|6666666.59337.peg.2097	None	fig|6666666.71181.peg.4284	fig|6666666.59344.peg.5112	fig|6666666.59345.peg.3334	fig|6666666.59341.peg.4851	fig|6666666.59347.peg.1804	None	None	fig|6666666.59349.peg.1309	None	fig|6666666.59351.peg.4530	fig|6666666.59353.peg.4773	fig|6666666.59355.peg.1722	fig|6666666.59354.peg.2023

3824	FIG01200137 hypothetical protein	fig|6666666.59329.peg.116	None	fig|6666666.59331.peg.5320	fig|6666666.59332.peg.3052	fig|6666666.59334.peg.4721	fig|6666666.59335.peg.810	fig|6666666.59336.peg.5039	fig|6666666.59333.peg.3409	fig|6666666.59338.peg.5641	fig|6666666.59339.peg.5001	fig|6666666.59340.peg.715	fig|6666666.59337.peg.98	fig|6666666.59342.peg.1358	None	None	fig|6666666.59345.peg.3745	fig|6666666.59341.peg.1146	fig|6666666.59347.peg.3065	fig|6666666.59346.peg.4040	fig|6666666.59348.peg.4177	None	None	None	None	None	None

2436	FIG01199752 hypothetical protein	fig|6666666.59329.peg.4739	fig|6666666.59330.peg.4546	fig|6666666.59331.peg.5060	fig|6666666.59332.peg.483	None	fig|6666666.59335.peg.1423	fig|6666666.59336.peg.6348	fig|6666666.59333.peg.2462	fig|6666666.59338.peg.3262	fig|6666666.59339.peg.222	fig|6666666.59340.peg.1078	fig|6666666.59337.peg.3146	fig|6666666.59342.peg.936	None	None	fig|6666666.59345.peg.1146	fig|6666666.59341.peg.1126	fig|6666666.59347.peg.2573	fig|6666666.59346.peg.100	fig|6666666.59348.peg.4290	None	None	None	None	None	None

1492	Na + H + antiporter subunit D	None	None	fig|6666666.59331.peg.5010	None	None	fig|6666666.59335.peg.3879	fig|6666666.59336.peg.5543	fig|6666666.59333.peg.2967	fig|6666666.59338.peg.3061	None	None	None	fig|6666666.59342.peg.4549	fig|6666666.71181.peg.532	fig|6666666.59344.peg.1940	fig|6666666.59345.peg.248	fig|6666666.59341.peg.2679	fig|6666666.59347.peg.2136	None	None	fig|6666666.59349.peg.3067	fig|6666666.59350.peg.2611	fig|6666666.59351.peg.3217	fig|6666666.59353.peg.1162	fig|6666666.59355.peg.314	fig|6666666.59354.peg.2465

9642	Small-conductance mechanosensitive channel	fig|6666666.59329.peg.5827	fig|6666666.59330.peg.3958	fig|6666666.59331.peg.5180	fig|6666666.59332.peg.5797	fig|6666666.59334.peg.1323	fig|6666666.59335.peg.5135	fig|6666666.59336.peg.4206	fig|6666666.59333.peg.2121	fig|6666666.59338.peg.5883	fig|6666666.59339.peg.4170	None	fig|6666666.59337.peg.1920	fig|6666666.59342.peg.4625	None	None	fig|6666666.59345.peg.2822	fig|6666666.59341.peg.764	fig|6666666.59347.peg.838	fig|6666666.59346.peg.1054	fig|6666666.59348.peg.3272	None	None	None	None	None	None

1002	FIG01202410 hypothetical protein	fig|6666666.59329.peg.2375	fig|6666666.59330.peg.3544	None	fig|6666666.59332.peg.2592	None	fig|6666666.59335.peg.2615	fig|6666666.59336.peg.1066	fig|6666666.59333.peg.3594	fig|6666666.59338.peg.2894	fig|6666666.59339.peg.2235	None	fig|6666666.59337.peg.4033	None	fig|6666666.71181.peg.5618	None	fig|6666666.59345.peg.1786	None	fig|6666666.59347.peg.5315	None	fig|6666666.59348.peg.1510	fig|6666666.59349.peg.854	fig|6666666.59350.peg.8376	fig|6666666.59351.peg.1826	None	None	fig|6666666.59354.peg.6379

8408	Phenylalanyl-tRNA synthetase beta subunit	fig|6666666.59329.peg.1527	None	None	fig|6666666.59332.peg.1251	None	fig|6666666.59335.peg.1471	fig|6666666.59336.peg.4419	None	fig|6666666.59338.peg.4148	fig|6666666.59339.peg.4424	fig|6666666.59340.peg.3437	fig|6666666.59337.peg.2581	fig|6666666.59342.peg.5136	None	None	fig|6666666.59345.peg.685	fig|6666666.59341.peg.1622	fig|6666666.59347.peg.405	fig|6666666.59346.peg.2611	None	fig|6666666.59349.peg.1957	fig|6666666.59350.peg.3193	fig|6666666.59351.peg.3273	None	None	fig|6666666.59354.peg.3938

5909	FIG01199691 hypothetical protein	fig|6666666.59329.peg.218	fig|6666666.59330.peg.1746	fig|6666666.59331.peg.4637	fig|6666666.59332.peg.1357	fig|6666666.59334.peg.2087	fig|6666666.59335.peg.1026	fig|6666666.59336.peg.3151	fig|6666666.59333.peg.4647	fig|6666666.59338.peg.194	fig|6666666.59339.peg.7435	fig|6666666.59340.peg.1232	fig|6666666.59337.peg.115	None	None	None	fig|6666666.59345.peg.3726	fig|6666666.59341.peg.3957	fig|6666666.59347.peg.3038	fig|6666666.59346.peg.3105	fig|6666666.59348.peg.1893	None	None	None	None	None	None

9604	Outer membrane protein	fig|6666666.59329.peg.5797	fig|6666666.59330.peg.3309	fig|6666666.59331.peg.5411	fig|6666666.59332.peg.4008	fig|6666666.59334.peg.2399	fig|6666666.59335.peg.2022	fig|6666666.59336.peg.6528	fig|6666666.59333.peg.3338	fig|6666666.59338.peg.3768	fig|6666666.59339.peg.3963	None	fig|6666666.59337.peg.3097	fig|6666666.59342.peg.4709	None	None	fig|6666666.59345.peg.4154	fig|6666666.59341.peg.2021	fig|6666666.59347.peg.1608	fig|6666666.59346.peg.4737	fig|6666666.59348.peg.2886	None	None	None	None	None	None

10036	FIG01200885 hypothetical protein	fig|6666666.59329.peg.3513	fig|6666666.59330.peg.790	fig|6666666.59331.peg.2641	fig|6666666.59332.peg.2431	fig|6666666.59334.peg.4190	fig|6666666.59335.peg.550	fig|6666666.59336.peg.4479	fig|6666666.59333.peg.1525	fig|6666666.59338.peg.5964	fig|6666666.59339.peg.2957	None	fig|6666666.59337.peg.3818	fig|6666666.59342.peg.946	None	None	fig|6666666.59345.peg.61	fig|6666666.59341.peg.2231	fig|6666666.59347.peg.921	fig|6666666.59346.peg.1225	fig|6666666.59348.peg.4169	None	None	None	None	None	None

1493	FIG01201208 hypothetical protein	fig|6666666.59329.peg.4215	fig|6666666.59330.peg.3643	fig|6666666.59331.peg.4759	fig|6666666.59332.peg.3079	None	fig|6666666.59335.peg.353	fig|6666666.59336.peg.2166	fig|6666666.59333.peg.5426	fig|6666666.59338.peg.780	fig|6666666.59339.peg.7143	fig|6666666.59340.peg.642	fig|6666666.59337.peg.5608	fig|6666666.59342.peg.3259	None	None	fig|6666666.59345.peg.2152	fig|6666666.59341.peg.1678	fig|6666666.59347.peg.2847	fig|6666666.59346.peg.3547	fig|6666666.59348.peg.3293	None	None	None	None	None	None

2629	Electron transfer flavoprotein, beta subunit	None	fig|6666666.59330.peg.4449	None	None	None	fig|6666666.59335.peg.4301	fig|6666666.59336.peg.2882	fig|6666666.59333.peg.3813	fig|6666666.59338.peg.3111	fig|6666666.59339.peg.4645	None	fig|6666666.59337.peg.4799	fig|6666666.59342.peg.3418	None	None	fig|6666666.59345.peg.322	fig|6666666.59341.peg.1711	fig|6666666.59347.peg.1154	None	fig|6666666.59348.peg.3517	fig|6666666.59349.peg.4285	fig|6666666.59350.peg.3051	fig|6666666.59351.peg.1075	None	fig|6666666.59355.peg.5427	fig|6666666.59354.peg.2724

76	FIG01200883 hypothetical protein	fig|6666666.59329.peg.2636	fig|6666666.59330.peg.1332	None	fig|6666666.59332.peg.2505	None	fig|6666666.59335.peg.929	fig|6666666.59336.peg.3900	fig|6666666.59333.peg.2546	fig|6666666.59338.peg.3807	fig|6666666.59339.peg.303	None	fig|6666666.59337.peg.5390	fig|6666666.59342.peg.132	None	None	fig|6666666.59345.peg.1542	fig|6666666.59341.peg.1011	fig|6666666.59347.peg.2382	fig|6666666.59346.peg.2028	fig|6666666.59348.peg.4509	fig|6666666.59349.peg.1696	None	fig|6666666.59351.peg.5219	None	None	None

3725	Pyrrolidone-carboxylate peptidase EC 3.4.19.3	fig|6666666.59329.peg.2705	fig|6666666.59330.peg.5114	None	None	None	None	fig|6666666.59336.peg.5939	fig|6666666.59333.peg.5159	fig|6666666.59338.peg.3359	None	None	fig|6666666.59337.peg.5348	fig|6666666.59342.peg.3414	fig|6666666.71181.peg.3229	fig|6666666.59344.peg.3000	fig|6666666.59345.peg.416	fig|6666666.59341.peg.5096	fig|6666666.59347.peg.367	None	None	fig|6666666.59349.peg.2233	None	fig|6666666.59351.peg.884	fig|6666666.59353.peg.2081	fig|6666666.59355.peg.3620	fig|6666666.59354.peg.4578

4113	FIG01199550 hypothetical protein	fig|6666666.59329.peg.3542	fig|6666666.59330.peg.3349	fig|6666666.59331.peg.3312	fig|6666666.59332.peg.5725	fig|6666666.59334.peg.5175	fig|6666666.59335.peg.6176	fig|6666666.59336.peg.4653	fig|6666666.59333.peg.3309	fig|6666666.59338.peg.3464	fig|6666666.59339.peg.3748	None	fig|6666666.59337.peg.3585	fig|6666666.59342.peg.3412	None	None	fig|6666666.59345.peg.867	fig|6666666.59341.peg.5468	fig|6666666.59347.peg.2238	fig|6666666.59346.peg.1685	fig|6666666.59348.peg.5121	None	None	None	None	None	None

9260	NADH ubiquinone oxidoreductase subunit 2	fig|6666666.59329.peg.6527	fig|6666666.59330.peg.3296	fig|6666666.59331.peg.1813	fig|6666666.59332.peg.4801	fig|6666666.59334.peg.4861	fig|6666666.59335.peg.4231	fig|6666666.59336.peg.3677	fig|6666666.59333.peg.3436	fig|6666666.59338.peg.2563	fig|6666666.59339.peg.6509	None	fig|6666666.59337.peg.2108	fig|6666666.59342.peg.3054	None	None	fig|6666666.59345.peg.3345	fig|6666666.59341.peg.3352	fig|6666666.59347.peg.969	fig|6666666.59346.peg.2939	fig|6666666.59348.peg.525	None	None	None	None	None	None

4326	FIG01199991 hypothetical protein	fig|6666666.59329.peg.6165	fig|6666666.59330.peg.1749	fig|6666666.59331.peg.4636	fig|6666666.59332.peg.1356	fig|6666666.59334.peg.4544	fig|6666666.59335.peg.1024	fig|6666666.59336.peg.2369	fig|6666666.59333.peg.4650	fig|6666666.59338.peg.192	fig|6666666.59339.peg.7440	None	fig|6666666.59337.peg.118	fig|6666666.59342.peg.3795	None	None	fig|6666666.59345.peg.3724	fig|6666666.59341.peg.3955	fig|6666666.59347.peg.3036	fig|6666666.59346.peg.3095	fig|6666666.59348.peg.1896	None	None	None	None	None	None

10849	FIG01199672 hypothetical protein	None	fig|6666666.59330.peg.2981	None	fig|6666666.59332.peg.4673	fig|6666666.59334.peg.4562	fig|6666666.59335.peg.1556	fig|6666666.59336.peg.3487	fig|6666666.59333.peg.4511	fig|6666666.59338.peg.4978	fig|6666666.59339.peg.3857	None	fig|6666666.59337.peg.3319	fig|6666666.59342.peg.1074	fig|6666666.71181.peg.733	fig|6666666.59344.peg.708	fig|6666666.59345.peg.4620	fig|6666666.59341.peg.1398	fig|6666666.59347.peg.1951	None	None	None	None	None	fig|6666666.59353.peg.968	fig|6666666.59355.peg.2408	None

8562	FIG01200525 hypothetical protein	fig|6666666.59329.peg.4458	fig|6666666.59330.peg.3959	fig|6666666.59331.peg.613	fig|6666666.59332.peg.5934	fig|6666666.59334.peg.4911	fig|6666666.59335.peg.3514	fig|6666666.59336.peg.4203	fig|6666666.59333.peg.3625	fig|6666666.59338.peg.6074	fig|6666666.59339.peg.6504	None	fig|6666666.59337.peg.1921	fig|6666666.59342.peg.4627	None	None	fig|6666666.59345.peg.2821	fig|6666666.59341.peg.2153	fig|6666666.59347.peg.619	fig|6666666.59346.peg.1049	fig|6666666.59348.peg.4775	None	None	None	None	None	None

5153	FIG01199890 hypothetical protein	fig|6666666.59329.peg.5767	fig|6666666.59330.peg.4278	fig|6666666.59331.peg.2681	fig|6666666.59332.peg.4323	fig|6666666.59334.peg.4781	fig|6666666.59335.peg.1477	fig|6666666.59336.peg.4155	fig|6666666.59333.peg.3683	fig|6666666.59338.peg.1803	fig|6666666.59339.peg.4419	None	fig|6666666.59337.peg.3433	fig|6666666.59342.peg.6295	None	None	fig|6666666.59345.peg.196	fig|6666666.59341.peg.2965	fig|6666666.59347.peg.790	fig|6666666.59346.peg.1925	fig|6666666.59348.peg.4426	None	None	None	None	None	None

277	Nicotinate-nucleotide adenylyltransferase EC 2.7.7.18	fig|6666666.59329.peg.2817	fig|6666666.59330.peg.2712	None	fig|6666666.59332.peg.990	fig|6666666.59334.peg.2856	fig|6666666.59335.peg.1626	fig|6666666.59336.peg.5657	fig|6666666.59333.peg.3908	fig|6666666.59338.peg.5471	fig|6666666.59339.peg.6543	None	fig|6666666.59337.peg.2441	fig|6666666.59342.peg.2954	None	None	fig|6666666.59345.peg.608	fig|6666666.59341.peg.5418	fig|6666666.59347.peg.545	fig|6666666.59346.peg.3019	fig|6666666.59348.peg.1501	None	None	None	None	None	None

6815	EBNA-1 nuclear protein	fig|6666666.59329.peg.5939	fig|6666666.59330.peg.4100	None	fig|6666666.59332.peg.2700	fig|6666666.59334.peg.4778	fig|6666666.59335.peg.5471	fig|6666666.59336.peg.5645	fig|6666666.59333.peg.824	fig|6666666.59338.peg.3709	fig|6666666.59339.peg.4130	fig|6666666.59340.peg.3026	fig|6666666.59337.peg.2862	None	None	None	fig|6666666.59345.peg.1729	fig|6666666.59341.peg.2426	fig|6666666.59347.peg.2469	fig|6666666.59346.peg.4780	None	None	fig|6666666.59350.peg.2944	None	None	None	None

1548	Inosose dehydratase EC 4.2.1.44	fig|6666666.59329.peg.1872	None	None	None	fig|6666666.59334.peg.2711	fig|6666666.59335.peg.3390	fig|6666666.59336.peg.2059	None	None	fig|6666666.59339.peg.695	None	None	fig|6666666.59342.peg.972	fig|6666666.71181.peg.1796	fig|6666666.59344.peg.4860	fig|6666666.59345.peg.1939	fig|6666666.59341.peg.2531	None	None	None	fig|6666666.59349.peg.620	fig|6666666.59350.peg.2183	fig|6666666.59351.peg.978	fig|6666666.59353.peg.2161	fig|6666666.59355.peg.3255	fig|6666666.59354.peg.5020

2181	FIG01199578 hypothetical protein	fig|6666666.59329.peg.4345	fig|6666666.59330.peg.1025	fig|6666666.59331.peg.3403	fig|6666666.59332.peg.4229	fig|6666666.59334.peg.3707	fig|6666666.59335.peg.3364	fig|6666666.59336.peg.5980	fig|6666666.59333.peg.4844	fig|6666666.59338.peg.4553	fig|6666666.59339.peg.1550	None	fig|6666666.59337.peg.3167	fig|6666666.59342.peg.1627	None	None	fig|6666666.59345.peg.3270	fig|6666666.59341.peg.4166	fig|6666666.59347.peg.1785	fig|6666666.59346.peg.3919	None	None	None	None	None	None	None

2184	Polysaccharide biosynthesis chain length regulator SypO	fig|6666666.59329.peg.6449	None	fig|6666666.59331.peg.4963	fig|6666666.59332.peg.2397	fig|6666666.59334.peg.5403	fig|6666666.59335.peg.2717	None	fig|6666666.59333.peg.3786	fig|6666666.59338.peg.1844	fig|6666666.59339.peg.1153	fig|6666666.59340.peg.2676	fig|6666666.59337.peg.4431	fig|6666666.59342.peg.3115	None	None	fig|6666666.59345.peg.3132	fig|6666666.59341.peg.5583	fig|6666666.59347.peg.2339	fig|6666666.59346.peg.5325	fig|6666666.59348.peg.2642	None	None	None	None	None	None

6430	ABC transporter, periplasmic substrate-binding protein YnjB	None	fig|6666666.59330.peg.3571	fig|6666666.59331.peg.5115	fig|6666666.59332.peg.2236	fig|6666666.59334.peg.3485	fig|6666666.59335.peg.5779	fig|6666666.59336.peg.4395	fig|6666666.59333.peg.1205	fig|6666666.59338.peg.2283	fig|6666666.59339.peg.5568	None	fig|6666666.59337.peg.3667	fig|6666666.59342.peg.4755	None	None	fig|6666666.59345.peg.1245	fig|6666666.59341.peg.5636	fig|6666666.59347.peg.3454	fig|6666666.59346.peg.3494	None	None	None	None	None	fig|6666666.59355.peg.5364	None

7148	Small-conductance mechanosensitive channel	fig|6666666.59329.peg.6445	fig|6666666.59330.peg.3900	None	None	fig|6666666.59334.peg.1712	None	fig|6666666.59336.peg.5405	None	None	None	None	fig|6666666.59337.peg.4702	fig|6666666.59342.peg.4790	fig|6666666.71181.peg.2740	fig|6666666.59344.peg.1114	fig|6666666.59345.peg.3104	fig|6666666.59341.peg.5133	fig|6666666.59347.peg.2064	None	None	fig|6666666.59349.peg.2203	None	fig|6666666.59351.peg.4190	fig|6666666.59353.peg.792	fig|6666666.59355.peg.913	fig|6666666.59354.peg.3019

3006	Outer membrane protein OmpK	fig|6666666.59329.peg.4847	fig|6666666.59330.peg.2797	fig|6666666.59331.peg.3693	fig|6666666.59332.peg.863	None	fig|6666666.59335.peg.109	fig|6666666.59336.peg.3462	fig|6666666.59333.peg.1927	None	fig|6666666.59339.peg.5033	None	fig|6666666.59337.peg.2369	fig|6666666.59342.peg.6439	fig|6666666.71181.peg.1212	fig|6666666.59344.peg.201	fig|6666666.59345.peg.2249	None	None	fig|6666666.59346.peg.2711	None	None	None	None	fig|6666666.59353.peg.507	fig|6666666.59355.peg.2233	None

3001	FIG01199686 hypothetical protein	fig|6666666.59329.peg.2757	fig|6666666.59330.peg.1297	None	fig|6666666.59332.peg.1118	fig|6666666.59334.peg.1143	fig|6666666.59335.peg.2096	fig|6666666.59336.peg.4509	fig|6666666.59333.peg.1505	fig|6666666.59338.peg.1289	fig|6666666.59339.peg.4900	fig|6666666.59340.peg.283	fig|6666666.59337.peg.72	None	None	None	fig|6666666.59345.peg.3771	fig|6666666.59341.peg.72	fig|6666666.59347.peg.4019	fig|6666666.59346.peg.1859	fig|6666666.59348.peg.2157	None	None	None	None	None	None

1588	Nitrate reductase cytochrome c550-type subunit	fig|6666666.59329.peg.2515	None	fig|6666666.59331.peg.4037	fig|6666666.59332.peg.2673	fig|6666666.59334.peg.4404	None	fig|6666666.59336.peg.1992	fig|6666666.59333.peg.3961	fig|6666666.59338.peg.3077	fig|6666666.59339.peg.2859	fig|6666666.59340.peg.1754	fig|6666666.59337.peg.3535	fig|6666666.59342.peg.962	None	None	fig|6666666.59345.peg.485	fig|6666666.59341.peg.4950	fig|6666666.59347.peg.666	fig|6666666.59346.peg.4257	fig|6666666.59348.peg.2526	None	None	None	None	None	None

6419	Autoinducer 2-binding periplasmic protein LuxP precursor	fig|6666666.59329.peg.4254	fig|6666666.59330.peg.2116	fig|6666666.59331.peg.1701	fig|6666666.59332.peg.5021	fig|6666666.59334.peg.4628	fig|6666666.59335.peg.5033	fig|6666666.59336.peg.2210	fig|6666666.59333.peg.1461	fig|6666666.59338.peg.3934	fig|6666666.59339.peg.5413	None	fig|6666666.59337.peg.4589	fig|6666666.59342.peg.2322	None	None	fig|6666666.59345.peg.505	fig|6666666.59341.peg.5599	fig|6666666.59347.peg.884	fig|6666666.59346.peg.743	None	None	None	None	None	None	None

5222	FIG01199776 hypothetical protein	fig|6666666.59329.peg.5750	fig|6666666.59330.peg.3348	None	fig|6666666.59332.peg.6228	None	fig|6666666.59335.peg.5263	fig|6666666.59336.peg.4858	fig|6666666.59333.peg.1915	fig|6666666.59338.peg.3267	fig|6666666.59339.peg.556	fig|6666666.59340.peg.2467	fig|6666666.59337.peg.3510	fig|6666666.59342.peg.4060	None	None	fig|6666666.59345.peg.928	fig|6666666.59341.peg.3421	fig|6666666.59347.peg.3564	fig|6666666.59346.peg.4965	fig|6666666.59348.peg.367	None	None	None	None	None	None

6479	FIG023406 hypothetical protein	None	fig|6666666.59330.peg.3231	fig|6666666.59331.peg.3335	fig|6666666.59332.peg.4301	fig|6666666.59334.peg.3991	fig|6666666.59335.peg.6173	fig|6666666.59336.peg.1572	fig|6666666.59333.peg.4723	fig|6666666.59338.peg.2339	fig|6666666.59339.peg.4141	None	None	fig|6666666.59342.peg.3384	None	None	fig|6666666.59345.peg.5605	None	fig|6666666.59347.peg.859	fig|6666666.59346.peg.5335	fig|6666666.59348.peg.1327	fig|6666666.59349.peg.2035	None	fig|6666666.59351.peg.2426	None	None	None

6717	FIG01200523 hypothetical protein	fig|6666666.59329.peg.5903	fig|6666666.59330.peg.1535	fig|6666666.59331.peg.1805	fig|6666666.59332.peg.4287	fig|6666666.59334.peg.1415	fig|6666666.59335.peg.582	fig|6666666.59336.peg.6634	fig|6666666.59333.peg.2918	fig|6666666.59338.peg.3279	fig|6666666.59339.peg.510	None	fig|6666666.59337.peg.2319	fig|6666666.59342.peg.4992	None	None	fig|6666666.59345.peg.1275	fig|6666666.59341.peg.5348	fig|6666666.59347.peg.2300	fig|6666666.59346.peg.4749	None	None	None	None	None	None	None

2570	ABC transporter ATP-binding protein	fig|6666666.59329.peg.1398	fig|6666666.59330.peg.1473	fig|6666666.59331.peg.2889	fig|6666666.59332.peg.891	None	None	fig|6666666.59336.peg.297	fig|6666666.59333.peg.1217	fig|6666666.59338.peg.2669	None	None	fig|6666666.59337.peg.203	fig|6666666.59342.peg.5866	None	None	fig|6666666.59345.peg.4570	fig|6666666.59341.peg.5181	fig|6666666.59347.peg.1125	fig|6666666.59346.peg.3849	fig|6666666.59348.peg.1470	fig|6666666.59349.peg.155	None	None	None	None	fig|6666666.59354.peg.176

5032	FIG01200960 hypothetical protein	fig|6666666.59329.peg.1904	fig|6666666.59330.peg.3328	None	fig|6666666.59332.peg.4527	None	fig|6666666.59335.peg.3397	fig|6666666.59336.peg.3955	fig|6666666.59333.peg.92	fig|6666666.59338.peg.4316	fig|6666666.59339.peg.4311	fig|6666666.59340.peg.3409	fig|6666666.59337.peg.4578	fig|6666666.59342.peg.1646	None	None	fig|6666666.59345.peg.515	fig|6666666.59341.peg.1899	fig|6666666.59347.peg.1522	fig|6666666.59346.peg.4754	fig|6666666.59348.peg.4556	None	None	None	None	None	None

317	Ferrous iron transport protein C	fig|6666666.59329.peg.6425	fig|6666666.59330.peg.4509	fig|6666666.59331.peg.1504	fig|6666666.59332.peg.3927	fig|6666666.59334.peg.5195	fig|6666666.59335.peg.4148	fig|6666666.59336.peg.1736	fig|6666666.59333.peg.917	fig|6666666.59338.peg.2374	fig|6666666.59339.peg.2992	None	fig|6666666.59337.peg.4123	None	None	None	fig|6666666.59345.peg.4311	fig|6666666.59341.peg.5056	fig|6666666.59347.peg.4400	fig|6666666.59346.peg.4283	fig|6666666.59348.peg.2686	None	None	None	None	None	None

592	Type II IV secretion system ATPase TadZ CpaE, associated with Flp pilus assembly	fig|6666666.59329.peg.6257	fig|6666666.59330.peg.664	fig|6666666.59331.peg.2294	fig|6666666.59332.peg.686	fig|6666666.59334.peg.2684	fig|6666666.59335.peg.3503	fig|6666666.59336.peg.702	None	None	fig|6666666.59339.peg.6819	None	fig|6666666.59337.peg.1334	fig|6666666.59342.peg.357	None	None	fig|6666666.59345.peg.2313	fig|6666666.59341.peg.4292	fig|6666666.59347.peg.1929	fig|6666666.59346.peg.4811	fig|6666666.59348.peg.4477	None	fig|6666666.59350.peg.7351	None	None	None	None

6108	hypothetical protein	fig|6666666.59329.peg.1731	None	fig|6666666.59331.peg.165	fig|6666666.59332.peg.4767	fig|6666666.59334.peg.3763	fig|6666666.59335.peg.5388	None	None	None	fig|6666666.59339.peg.8465	None	None	fig|6666666.59342.peg.4989	fig|6666666.71181.peg.1145	fig|6666666.59344.peg.2268	fig|6666666.59345.peg.3186	fig|6666666.59341.peg.934	None	fig|6666666.59346.peg.719	fig|6666666.59348.peg.1255	None	None	fig|6666666.59351.peg.968	fig|6666666.59353.peg.3435	fig|6666666.59355.peg.3536	None

10936	Phosphotransferase system IIA component	fig|6666666.59329.peg.5701	fig|6666666.59330.peg.4641	fig|6666666.59331.peg.2130	fig|6666666.59332.peg.5778	fig|6666666.59334.peg.4763	fig|6666666.59335.peg.2276	fig|6666666.59336.peg.5066	fig|6666666.59333.peg.4506	fig|6666666.59338.peg.5524	None	None	fig|6666666.59337.peg.5663	fig|6666666.59342.peg.1681	None	None	fig|6666666.59345.peg.109	fig|6666666.59341.peg.3248	fig|6666666.59347.peg.832	fig|6666666.59346.peg.2908	fig|6666666.59348.peg.5381	None	None	None	None	None	None

2464	hypothetical protein	fig|6666666.59329.peg.6071	fig|6666666.59330.peg.2670	fig|6666666.59331.peg.1869	fig|6666666.59332.peg.5089	fig|6666666.59334.peg.1681	fig|6666666.59335.peg.4011	fig|6666666.59336.peg.1692	None	None	fig|6666666.59339.peg.1776	None	fig|6666666.59337.peg.4532	fig|6666666.59342.peg.396	None	None	fig|6666666.59345.peg.4668	fig|6666666.59341.peg.2863	None	fig|6666666.59346.peg.1430	fig|6666666.59348.peg.2704	fig|6666666.59349.peg.3250	None	fig|6666666.59351.peg.3351	None	None	None

9456	Acyl-CoA synthetase	fig|6666666.59329.peg.5946	fig|6666666.59330.peg.2555	fig|6666666.59331.peg.627	fig|6666666.59332.peg.2508	None	fig|6666666.59335.peg.5881	fig|6666666.59336.peg.2145	fig|6666666.59333.peg.1697	fig|6666666.59338.peg.2144	fig|6666666.59339.peg.4503	None	fig|6666666.59337.peg.2257	fig|6666666.59342.peg.3896	None	None	fig|6666666.59345.peg.2948	fig|6666666.59341.peg.1167	fig|6666666.59347.peg.4503	fig|6666666.59346.peg.5218	fig|6666666.59348.peg.4963	None	None	None	None	None	None

10959	GlcNAc 2 ABC transporter, ATP-binding component 2	fig|6666666.59329.peg.657	fig|6666666.59330.peg.3993	fig|6666666.59331.peg.421	fig|6666666.59332.peg.236	fig|6666666.59334.peg.1251	fig|6666666.59335.peg.5281	fig|6666666.59336.peg.782	fig|6666666.59333.peg.3766	fig|6666666.59338.peg.3552	fig|6666666.59339.peg.5446	None	fig|6666666.59337.peg.652	None	None	None	fig|6666666.59345.peg.5262	fig|6666666.59341.peg.1811	fig|6666666.59347.peg.3147	fig|6666666.59346.peg.766	fig|6666666.59348.peg.4464	None	None	None	None	None	None

4392	FIG01201756 hypothetical protein	fig|6666666.59329.peg.4148	fig|6666666.59330.peg.4202	None	fig|6666666.59332.peg.5527	None	fig|6666666.59335.peg.6200	fig|6666666.59336.peg.6287	fig|6666666.59333.peg.5450	fig|6666666.59338.peg.2275	None	None	None	fig|6666666.59342.peg.1030	None	None	fig|6666666.59345.peg.4819	fig|6666666.59341.peg.5742	fig|6666666.59347.peg.879	fig|6666666.59346.peg.5198	fig|6666666.59348.peg.826	fig|6666666.59349.peg.836	None	fig|6666666.59351.peg.3380	None	None	fig|6666666.59354.peg.4308

9638	FIG01200641 hypothetical protein	fig|6666666.59329.peg.2552	fig|6666666.59330.peg.2885	fig|6666666.59331.peg.4622	fig|6666666.59332.peg.2643	None	fig|6666666.59335.peg.2057	fig|6666666.59336.peg.848	fig|6666666.59333.peg.3358	None	fig|6666666.59339.peg.7495	None	fig|6666666.59337.peg.148	fig|6666666.59342.peg.533	None	None	fig|6666666.59345.peg.3693	None	None	fig|6666666.59346.peg.255	fig|6666666.59348.peg.395	fig|6666666.59349.peg.4834	None	fig|6666666.59351.peg.1507	None	None	fig|6666666.59354.peg.3501

8943	FIG01200287 hypothetical protein	fig|6666666.59329.peg.2445	fig|6666666.59330.peg.1247	None	None	None	fig|6666666.59335.peg.5121	fig|6666666.59336.peg.2753	fig|6666666.59333.peg.1884	fig|6666666.59338.peg.4771	fig|6666666.59339.peg.3458	None	fig|6666666.59337.peg.2190	None	None	None	fig|6666666.59345.peg.1597	fig|6666666.59341.peg.255	fig|6666666.59347.peg.4097	fig|6666666.59346.peg.131	fig|6666666.59348.peg.3628	fig|6666666.59349.peg.1797	None	fig|6666666.59351.peg.992	None	None	fig|6666666.59354.peg.1801

379	FIG01201392 hypothetical protein	fig|6666666.59329.peg.5909	None	fig|6666666.59331.peg.4055	fig|6666666.59332.peg.166	None	fig|6666666.59335.peg.418	fig|6666666.59336.peg.2170	fig|6666666.59333.peg.1386	fig|6666666.59338.peg.2357	fig|6666666.59339.peg.6364	fig|6666666.59340.peg.2572	fig|6666666.59337.peg.409	fig|6666666.59342.peg.5398	None	None	fig|6666666.59345.peg.2554	fig|6666666.59341.peg.193	fig|6666666.59347.peg.4532	fig|6666666.59346.peg.798	fig|6666666.59348.peg.2311	None	None	None	None	None	None

10972	membrane protein	fig|6666666.59329.peg.2119	fig|6666666.59330.peg.2450	fig|6666666.59331.peg.3531	fig|6666666.59332.peg.3196	fig|6666666.59334.peg.4127	fig|6666666.59335.peg.225	fig|6666666.59336.peg.1782	fig|6666666.59333.peg.2817	None	fig|6666666.59339.peg.8177	fig|6666666.59340.peg.1770	fig|6666666.59337.peg.4793	None	None	None	fig|6666666.59345.peg.4838	fig|6666666.59341.peg.1841	fig|6666666.59347.peg.2753	fig|6666666.59346.peg.1675	fig|6666666.59348.peg.555	None	None	None	None	None	None

4822	ABC-type Fe3+-hydroxamate transport system, periplasmic component	fig|6666666.59329.peg.1854	fig|6666666.59330.peg.4454	None	fig|6666666.59332.peg.1876	fig|6666666.59334.peg.160	fig|6666666.59335.peg.334	fig|6666666.59336.peg.612	fig|6666666.59333.peg.877	None	fig|6666666.59339.peg.8326	None	fig|6666666.59337.peg.1723	fig|6666666.59342.peg.2485	None	None	fig|6666666.59345.peg.5361	None	None	fig|6666666.59346.peg.3207	fig|6666666.59348.peg.4244	fig|6666666.59349.peg.99	None	fig|6666666.59351.peg.156	None	None	fig|6666666.59354.peg.420

7986	Transmembrane regulatory protein ToxS	fig|6666666.59329.peg.4592	fig|6666666.59330.peg.2200	None	fig|6666666.59332.peg.1700	fig|6666666.59334.peg.5429	fig|6666666.59335.peg.2422	fig|6666666.59336.peg.2552	fig|6666666.59333.peg.2062	fig|6666666.59338.peg.5513	fig|6666666.59339.peg.5262	None	fig|6666666.59337.peg.3917	fig|6666666.59342.peg.4084	None	None	fig|6666666.59345.peg.4352	fig|6666666.59341.peg.508	fig|6666666.59347.peg.2835	fig|6666666.59346.peg.3675	fig|6666666.59348.peg.1279	None	None	None	None	None	None

6359	Butyrate-acetoacetate CoA-transferase subunit A EC 2.8.3.9	fig|6666666.59329.peg.4091	None	None	None	None	fig|6666666.59335.peg.3382	fig|6666666.59336.peg.2062	None	None	fig|6666666.59339.peg.686	fig|6666666.59340.peg.1925	None	fig|6666666.59342.peg.974	fig|6666666.71181.peg.4665	fig|6666666.59344.peg.3817	fig|6666666.59345.peg.1941	fig|6666666.59341.peg.5443	None	None	None	fig|6666666.59349.peg.2163	fig|6666666.59350.peg.3321	fig|6666666.59351.peg.3693	fig|6666666.59353.peg.2476	fig|6666666.59355.peg.5094	fig|6666666.59354.peg.6304

11109	Transcriptional regulator, LysR family	None	fig|6666666.59330.peg.2213	None	None	fig|6666666.59334.peg.2506	fig|6666666.59335.peg.6102	fig|6666666.59336.peg.483	fig|6666666.59333.peg.2853	fig|6666666.59338.peg.4726	fig|6666666.59339.peg.4179	None	fig|6666666.59337.peg.1890	None	fig|6666666.71181.peg.4046	fig|6666666.59344.peg.2941	fig|6666666.59345.peg.2853	fig|6666666.59341.peg.2476	fig|6666666.59347.peg.900	fig|6666666.59346.peg.3649	None	None	None	None	fig|6666666.59353.peg.3937	fig|6666666.59355.peg.3164	None

8457	Inner membrane protein YccF	None	fig|6666666.59330.peg.887	fig|6666666.59331.peg.5973	fig|6666666.59332.peg.2946	None	fig|6666666.59335.peg.4570	fig|6666666.59336.peg.1867	fig|6666666.59333.peg.3290	None	fig|6666666.59339.peg.2016	None	fig|6666666.59337.peg.5416	fig|6666666.59342.peg.1699	fig|6666666.71181.peg.269	fig|6666666.59344.peg.1196	fig|6666666.59345.peg.567	None	None	fig|6666666.59346.peg.3396	fig|6666666.59348.peg.466	None	None	None	fig|6666666.59353.peg.1699	fig|6666666.59355.peg.1021	None

2211	Chromosome segregation ATPase	fig|6666666.59329.peg.3976	fig|6666666.59330.peg.4705	fig|6666666.59331.peg.6322	None	fig|6666666.59334.peg.5117	fig|6666666.59335.peg.5205	fig|6666666.59336.peg.5958	fig|6666666.59333.peg.4431	fig|6666666.59338.peg.3777	fig|6666666.59339.peg.812	None	fig|6666666.59337.peg.1851	fig|6666666.59342.peg.1167	None	None	fig|6666666.59345.peg.4203	fig|6666666.59341.peg.5914	fig|6666666.59347.peg.1631	fig|6666666.59346.peg.3478	fig|6666666.59348.peg.3783	None	None	None	None	None	None

1016	FIG01201081 hypothetical protein	fig|6666666.59329.peg.2893	fig|6666666.59330.peg.875	fig|6666666.59331.peg.201	None	fig|6666666.59334.peg.3843	None	fig|6666666.59336.peg.2075	fig|6666666.59333.peg.54	fig|6666666.59338.peg.3128	fig|6666666.59339.peg.5294	fig|6666666.59340.peg.3800	fig|6666666.59337.peg.3294	fig|6666666.59342.peg.2990	None	None	fig|6666666.59345.peg.558	fig|6666666.59341.peg.6022	fig|6666666.59347.peg.1662	fig|6666666.59346.peg.4122	None	None	fig|6666666.59350.peg.1193	None	None	None	None

4354	Cellulose synthase catalytic subunit [UDP-forming] EC 2.4.1.12	None	fig|6666666.59330.peg.2187	fig|6666666.59331.peg.3798	fig|6666666.59332.peg.4336	None	None	fig|6666666.59336.peg.3302	None	fig|6666666.59338.peg.3320	None	None	fig|6666666.59337.peg.4920	fig|6666666.59342.peg.88	fig|6666666.71181.peg.4269	fig|6666666.59344.peg.2440	fig|6666666.59345.peg.4374	fig|6666666.59341.peg.5779	fig|6666666.59347.peg.3284	fig|6666666.59346.peg.4571	None	None	fig|6666666.59350.peg.5311	None	fig|6666666.59353.peg.3666	fig|6666666.59355.peg.4037	None

3245	Ribosome modulation factor	fig|6666666.59329.peg.2244	fig|6666666.59330.peg.3168	fig|6666666.59331.peg.2638	fig|6666666.59332.peg.5385	fig|6666666.59334.peg.1330	fig|6666666.59335.peg.1222	fig|6666666.59336.peg.6630	fig|6666666.59333.peg.2795	fig|6666666.59338.peg.6071	fig|6666666.59339.peg.1511	None	fig|6666666.59337.peg.4558	fig|6666666.59342.peg.2346	None	None	fig|6666666.59345.peg.3250	fig|6666666.59341.peg.913	fig|6666666.59347.peg.1763	fig|6666666.59346.peg.2057	None	None	None	None	None	None	None

7561	Glutaredoxin	fig|6666666.59329.peg.6471	fig|6666666.59330.peg.4649	fig|6666666.59331.peg.1260	fig|6666666.59332.peg.817	fig|6666666.59334.peg.460	fig|6666666.59335.peg.2006	None	fig|6666666.59333.peg.5384	fig|6666666.59338.peg.2464	fig|6666666.59339.peg.3942	None	fig|6666666.59337.peg.2000	fig|6666666.59342.peg.4647	None	None	fig|6666666.59345.peg.4690	fig|6666666.59341.peg.2733	fig|6666666.59347.peg.3341	fig|6666666.59346.peg.5074	fig|6666666.59348.peg.1728	None	None	None	None	None	None

4167	Type IIA topoisomerase, B subunit	fig|6666666.59329.peg.1739	fig|6666666.59330.peg.4923	None	fig|6666666.59332.peg.4875	None	fig|6666666.59335.peg.3864	fig|6666666.59336.peg.5535	fig|6666666.59333.peg.3697	fig|6666666.59338.peg.1027	fig|6666666.59339.peg.1183	None	fig|6666666.59337.peg.2700	fig|6666666.59342.peg.2243	None	None	fig|6666666.59345.peg.3177	fig|6666666.59341.peg.3614	fig|6666666.59347.peg.1812	None	None	fig|6666666.59349.peg.3802	None	fig|6666666.59351.peg.3877	None	None	fig|6666666.59354.peg.3999

2098	hypothetical protein	fig|6666666.59329.peg.6191	fig|6666666.59330.peg.757	fig|6666666.59331.peg.3757	fig|6666666.59332.peg.2761	fig|6666666.59334.peg.3253	None	fig|6666666.59336.peg.4733	None	fig|6666666.59338.peg.2615	fig|6666666.59339.peg.1010	None	fig|6666666.59337.peg.1195	fig|6666666.59342.peg.1434	None	None	fig|6666666.59345.peg.12	fig|6666666.59341.peg.1122	fig|6666666.59347.peg.2579	None	fig|6666666.59348.peg.3903	fig|6666666.59349.peg.2655	None	None	None	None	fig|6666666.59354.peg.4358

7022	dCMP deaminase EC 3.5.4.12	fig|6666666.59329.peg.1093	fig|6666666.59330.peg.4588	fig|6666666.59331.peg.5898	fig|6666666.59332.peg.3353	fig|6666666.59334.peg.2936	fig|6666666.59335.peg.3404	fig|6666666.59336.peg.2900	fig|6666666.59333.peg.1898	fig|6666666.59338.peg.5328	fig|6666666.59339.peg.4322	None	fig|6666666.59337.peg.3544	fig|6666666.59342.peg.655	None	None	fig|6666666.59345.peg.475	None	fig|6666666.59347.peg.2355	fig|6666666.59346.peg.2608	fig|6666666.59348.peg.2539	None	None	None	None	None	None

1205	Probable proline and glycine rich transmembrane protein gene in bax	fig|6666666.59329.peg.4264	fig|6666666.59330.peg.3201	None	fig|6666666.59332.peg.5499	fig|6666666.59334.peg.2621	fig|6666666.59335.peg.2214	fig|6666666.59336.peg.3528	fig|6666666.59333.peg.4849	fig|6666666.59338.peg.4109	fig|6666666.59339.peg.1310	None	fig|6666666.59337.peg.3713	fig|6666666.59342.peg.230	None	None	fig|6666666.59345.peg.3222	fig|6666666.59341.peg.5625	fig|6666666.59347.peg.1732	fig|6666666.59346.peg.1521	fig|6666666.59348.peg.2933	None	None	None	None	None	None

7007	Type IV pilus biogenesis protein PilP	fig|6666666.59329.peg.311	fig|6666666.59330.peg.2633	fig|6666666.59331.peg.892	fig|6666666.59332.peg.1028	None	fig|6666666.59335.peg.2487	fig|6666666.59336.peg.4161	None	fig|6666666.59338.peg.959	fig|6666666.59339.peg.8721	None	fig|6666666.59337.peg.1699	None	None	None	None	fig|6666666.59341.peg.965	fig|6666666.59347.peg.4941	fig|6666666.59346.peg.4	fig|6666666.59348.peg.1914	fig|6666666.59349.peg.525	None	fig|6666666.59351.peg.3848	None	None	fig|6666666.59354.peg.2403

3118	hypothetical protein	None	None	fig|6666666.59331.peg.5670	fig|6666666.59332.peg.5343	fig|6666666.59334.peg.4522	fig|6666666.59335.peg.5749	fig|6666666.59336.peg.6592	fig|6666666.59333.peg.2369	fig|6666666.59338.peg.5606	fig|6666666.59339.peg.2127	fig|6666666.59340.peg.3802	fig|6666666.59337.peg.3415	fig|6666666.59342.peg.4068	None	None	fig|6666666.59345.peg.1978	fig|6666666.59341.peg.3389	fig|6666666.59347.peg.3472	fig|6666666.59346.peg.1018	None	None	fig|6666666.59350.peg.1897	None	None	None	None

5743	FIG00921398 hypothetical protein	fig|6666666.59329.peg.5387	fig|6666666.59330.peg.3861	None	fig|6666666.59332.peg.1658	fig|6666666.59334.peg.4228	fig|6666666.59335.peg.1346	fig|6666666.59336.peg.1478	fig|6666666.59333.peg.1188	fig|6666666.59338.peg.5462	fig|6666666.59339.peg.908	None	fig|6666666.59337.peg.4268	fig|6666666.59342.peg.6031	None	None	fig|6666666.59345.peg.1218	fig|6666666.59341.peg.873	fig|6666666.59347.peg.1994	fig|6666666.59346.peg.228	fig|6666666.59348.peg.2317	None	None	None	None	None	None

1918	FIG01201938 hypothetical protein	fig|6666666.59329.peg.4491	None	None	fig|6666666.59332.peg.5618	fig|6666666.59334.peg.3733	None	fig|6666666.59336.peg.2546	fig|6666666.59333.peg.4375	fig|6666666.59338.peg.4676	None	fig|6666666.59340.peg.1883	fig|6666666.59337.peg.1901	fig|6666666.59342.peg.4107	None	None	fig|6666666.59345.peg.2831	None	fig|6666666.59347.peg.3843	fig|6666666.59346.peg.1920	fig|6666666.59348.peg.5148	fig|6666666.59349.peg.4650	None	fig|6666666.59351.peg.1514	None	None	fig|6666666.59354.peg.3506

7506	FIG002781 Alpha-L-glutamate ligase family protein	fig|6666666.59329.peg.3559	fig|6666666.59330.peg.2478	fig|6666666.59331.peg.1021	fig|6666666.59332.peg.4610	fig|6666666.59334.peg.4841	fig|6666666.59335.peg.946	fig|6666666.59336.peg.2788	fig|6666666.59333.peg.1054	None	fig|6666666.59339.peg.330	fig|6666666.59340.peg.1860	fig|6666666.59337.peg.4540	None	None	None	fig|6666666.59345.peg.1525	fig|6666666.59341.peg.2245	fig|6666666.59347.peg.4188	fig|6666666.59346.peg.4181	fig|6666666.59348.peg.1594	None	None	None	None	None	None

7611	hypothetical protein	fig|6666666.59329.peg.1560	fig|6666666.59330.peg.1381	fig|6666666.59331.peg.4881	fig|6666666.59332.peg.4770	fig|6666666.59334.peg.3188	fig|6666666.59335.peg.5735	fig|6666666.59336.peg.415	fig|6666666.59333.peg.743	fig|6666666.59338.peg.2268	fig|6666666.59339.peg.4596	None	fig|6666666.59337.peg.2883	None	None	None	fig|6666666.59345.peg.1022	fig|6666666.59341.peg.1156	fig|6666666.59347.peg.2500	fig|6666666.59346.peg.5214	fig|6666666.59348.peg.4384	None	None	None	None	None	None

761	FIG01199914 hypothetical protein	fig|6666666.59329.peg.788	fig|6666666.59330.peg.3380	fig|6666666.59331.peg.3723	fig|6666666.59332.peg.2568	fig|6666666.59334.peg.3503	None	None	fig|6666666.59333.peg.4811	fig|6666666.59338.peg.1211	fig|6666666.59339.peg.50	fig|6666666.59340.peg.309	fig|6666666.59337.peg.4415	fig|6666666.59342.peg.1052	None	None	fig|6666666.59345.peg.1129	fig|6666666.59341.peg.2833	fig|6666666.59347.peg.285	fig|6666666.59346.peg.5254	fig|6666666.59348.peg.84	None	None	None	None	None	None

5183	putative membrane protein	fig|6666666.59329.peg.3005	None	fig|6666666.59331.peg.3294	fig|6666666.59332.peg.3396	fig|6666666.59334.peg.2586	fig|6666666.59335.peg.5586	fig|6666666.59336.peg.3199	None	fig|6666666.59338.peg.1600	None	None	None	fig|6666666.59342.peg.3236	fig|6666666.71181.peg.1753	fig|6666666.59344.peg.1622	fig|6666666.59345.peg.1430	fig|6666666.59341.peg.2880	fig|6666666.59347.peg.4423	fig|6666666.59346.peg.4186	None	None	None	None	fig|6666666.59353.peg.2690	fig|6666666.59355.peg.1789	None

3739	Putative protein-S-isoprenylcysteine methyltransferase	fig|6666666.59329.peg.4801	fig|6666666.59330.peg.496	fig|6666666.59331.peg.4986	fig|6666666.59332.peg.4485	None	fig|6666666.59335.peg.1561	fig|6666666.59336.peg.5017	fig|6666666.59333.peg.4353	fig|6666666.59338.peg.3618	fig|6666666.59339.peg.3865	None	fig|6666666.59337.peg.3316	None	None	None	fig|6666666.59345.peg.4623	fig|6666666.59341.peg.1392	fig|6666666.59347.peg.1948	fig|6666666.59346.peg.4466	fig|6666666.59348.peg.3582	None	None	fig|6666666.59351.peg.1711	None	None	None

5166	Putative phosphatidylglycerophosphate synthase	fig|6666666.59329.peg.5685	fig|6666666.59330.peg.3576	fig|6666666.59331.peg.2162	fig|6666666.59332.peg.2232	fig|6666666.59334.peg.3580	fig|6666666.59335.peg.5088	fig|6666666.59336.peg.6331	fig|6666666.59333.peg.1208	None	fig|6666666.59339.peg.546	None	fig|6666666.59337.peg.3671	fig|6666666.59342.peg.4643	None	None	fig|6666666.59345.peg.1248	fig|6666666.59341.peg.1088	fig|6666666.59347.peg.3457	None	fig|6666666.59348.peg.1146	None	fig|6666666.59350.peg.1528	None	None	None	None

3093	Butyrate-acetoacetate CoA-transferase subunit B EC 2.8.3.9	None	None	None	None	None	fig|6666666.59335.peg.3381	fig|6666666.59336.peg.2063	None	None	fig|6666666.59339.peg.685	fig|6666666.59340.peg.924	None	fig|6666666.59342.peg.975	fig|6666666.71181.peg.4592	fig|6666666.59344.peg.3816	fig|6666666.59345.peg.1942	fig|6666666.59341.peg.5442	None	None	fig|6666666.59348.peg.4381	fig|6666666.59349.peg.2164	fig|6666666.59350.peg.6543	fig|6666666.59351.peg.3692	fig|6666666.59353.peg.2477	fig|6666666.59355.peg.570	fig|6666666.59354.peg.6305

9202	Uncharacterized protein DUF547	fig|6666666.59329.peg.123	fig|6666666.59330.peg.813	fig|6666666.59331.peg.2385	fig|6666666.59332.peg.4896	fig|6666666.59334.peg.341	fig|6666666.59335.peg.5237	fig|6666666.59336.peg.3669	fig|6666666.59333.peg.126	fig|6666666.59338.peg.54	fig|6666666.59339.peg.7319	fig|6666666.59340.peg.3098	fig|6666666.59337.peg.1389	None	None	None	fig|6666666.59345.peg.3891	fig|6666666.59341.peg.4412	fig|6666666.59347.peg.3083	fig|6666666.59346.peg.818	None	None	None	None	None	None	None

7407	Putative arylsulfatase regulatory protein	fig|6666666.59329.peg.4139	None	fig|6666666.59331.peg.330	fig|6666666.59332.peg.3527	fig|6666666.59334.peg.5412	None	fig|6666666.59336.peg.5882	fig|6666666.59333.peg.3468	fig|6666666.59338.peg.5633	fig|6666666.59339.peg.4077	fig|6666666.59340.peg.2876	fig|6666666.59337.peg.2118	fig|6666666.59342.peg.2851	None	None	fig|6666666.59345.peg.3313	fig|6666666.59341.peg.1530	fig|6666666.59347.peg.2217	fig|6666666.59346.peg.4395	None	None	None	fig|6666666.59351.peg.5385	None	None	None

4714	FIG01200185 hypothetical protein	fig|6666666.59329.peg.2623	fig|6666666.59330.peg.87	fig|6666666.59331.peg.3700	fig|6666666.59332.peg.3770	fig|6666666.59334.peg.1151	fig|6666666.59335.peg.3635	fig|6666666.59336.peg.4265	None	fig|6666666.59338.peg.608	fig|6666666.59339.peg.5009	fig|6666666.59340.peg.1460	fig|6666666.59337.peg.1300	fig|6666666.59342.peg.4689	None	None	fig|6666666.59345.peg.2338	fig|6666666.59341.peg.4999	fig|6666666.59347.peg.2974	None	fig|6666666.59348.peg.2222	None	None	None	None	None	None

6823	F WD40 repeat	fig|6666666.59329.peg.4720	fig|6666666.59330.peg.1011	None	fig|6666666.59332.peg.873	fig|6666666.59334.peg.932	fig|6666666.59335.peg.5673	fig|6666666.59336.peg.6497	fig|6666666.59333.peg.2667	fig|6666666.59338.peg.4704	fig|6666666.59339.peg.1535	None	fig|6666666.59337.peg.3153	fig|6666666.59342.peg.786	None	None	fig|6666666.59345.peg.3260	fig|6666666.59341.peg.900	fig|6666666.59347.peg.1773	fig|6666666.59346.peg.3398	fig|6666666.59348.peg.1998	None	None	None	None	None	None

8175	Type IV fimbrial biogenesis protein PilW	fig|6666666.59329.peg.1666	fig|6666666.59330.peg.4789	fig|6666666.59331.peg.1068	fig|6666666.59332.peg.392	None	fig|6666666.59335.peg.1691	fig|6666666.59336.peg.3612	fig|6666666.59333.peg.3464	None	fig|6666666.59339.peg.7265	fig|6666666.59340.peg.1147	fig|6666666.59337.peg.4978	fig|6666666.59342.peg.2190	None	None	fig|6666666.59345.peg.3567	fig|6666666.59341.peg.4068	fig|6666666.59347.peg.4335	fig|6666666.59346.peg.4644	fig|6666666.59348.peg.1566	None	None	None	None	None	None

9266	Predicted metal-dependent hydrolase	fig|6666666.59329.peg.300	fig|6666666.59330.peg.1432	fig|6666666.59331.peg.3768	fig|6666666.59332.peg.4350	None	fig|6666666.59335.peg.5524	fig|6666666.59336.peg.1630	fig|6666666.59333.peg.2149	fig|6666666.59338.peg.3377	fig|6666666.59339.peg.6977	fig|6666666.59340.peg.39	fig|6666666.59337.peg.757	None	None	None	fig|6666666.59345.peg.3635	fig|6666666.59341.peg.3031	fig|6666666.59347.peg.4301	fig|6666666.59346.peg.1313	fig|6666666.59348.peg.5212	None	None	None	None	None	None

9265	putative glycoporin	None	None	fig|6666666.59331.peg.1496	fig|6666666.59332.peg.4948	fig|6666666.59334.peg.2600	fig|6666666.59335.peg.4000	fig|6666666.59336.peg.2116	None	fig|6666666.59338.peg.555	None	fig|6666666.59340.peg.826	fig|6666666.59337.peg.2296	fig|6666666.59342.peg.5686	fig|6666666.71181.peg.5045	fig|6666666.59344.peg.6220	fig|6666666.59345.peg.1724	fig|6666666.59341.peg.1213	fig|6666666.59347.peg.3643	None	None	None	None	None	fig|6666666.59353.peg.5087	fig|6666666.59355.peg.5168	None

1208	Azurin	fig|6666666.59329.peg.5086	fig|6666666.59330.peg.2547	None	fig|6666666.59332.peg.5391	fig|6666666.59334.peg.3254	fig|6666666.59335.peg.4297	fig|6666666.59336.peg.6400	fig|6666666.59333.peg.1562	fig|6666666.59338.peg.1787	fig|6666666.59339.peg.4639	None	fig|6666666.59337.peg.3690	fig|6666666.59342.peg.3285	None	None	fig|6666666.59345.peg.668	None	fig|6666666.59347.peg.1903	fig|6666666.59346.peg.1698	None	fig|6666666.59349.peg.2031	None	None	None	None	fig|6666666.59354.peg.2054

9746	FIG01202244 hypothetical protein	None	fig|6666666.59330.peg.2724	fig|6666666.59331.peg.2047	fig|6666666.59332.peg.1529	fig|6666666.59334.peg.1304	fig|6666666.59335.peg.4568	fig|6666666.59336.peg.4813	None	fig|6666666.59338.peg.6010	fig|6666666.59339.peg.2014	fig|6666666.59340.peg.1825	fig|6666666.59337.peg.4055	fig|6666666.59342.peg.4236	None	None	fig|6666666.59345.peg.3404	fig|6666666.59341.peg.5723	fig|6666666.59347.peg.213	fig|6666666.59346.peg.3660	fig|6666666.59348.peg.469	None	None	None	None	None	None

7676	Flagellar biosynthesis protein FliL	fig|6666666.59329.peg.5863	fig|6666666.59330.peg.2909	None	None	None	fig|6666666.59335.peg.915	fig|6666666.59336.peg.986	fig|6666666.59333.peg.4086	fig|6666666.59338.peg.2386	fig|6666666.59339.peg.89	None	fig|6666666.59337.peg.3212	fig|6666666.59342.peg.3229	fig|6666666.71181.peg.4937	fig|6666666.59344.peg.3055	fig|6666666.59345.peg.2878	fig|6666666.59341.peg.122	fig|6666666.59347.peg.4611	None	None	None	None	None	fig|6666666.59353.peg.3509	fig|6666666.59355.peg.3667	None

3523	Tryptophanyl-tRNA synthetase EC 6.1.1.2	fig|6666666.59329.peg.5397	fig|6666666.59330.peg.4742	fig|6666666.59331.peg.4613	fig|6666666.59332.peg.1442	None	fig|6666666.59335.peg.2980	fig|6666666.59336.peg.5720	fig|6666666.59333.peg.2406	None	None	None	fig|6666666.59337.peg.2834	fig|6666666.59342.peg.6399	fig|6666666.71181.peg.1132	fig|6666666.59344.peg.2253	fig|6666666.59345.peg.1368	None	None	fig|6666666.59346.peg.3567	fig|6666666.59348.peg.4058	None	None	None	fig|6666666.59353.peg.4970	fig|6666666.59355.peg.5042	None

2698	FIG01200921 hypothetical protein	fig|6666666.59329.peg.3067	fig|6666666.59330.peg.1215	None	fig|6666666.59332.peg.5577	None	fig|6666666.59335.peg.2946	None	fig|6666666.59333.peg.1085	fig|6666666.59338.peg.4734	fig|6666666.59339.peg.273	None	fig|6666666.59337.peg.3128	fig|6666666.59342.peg.1235	None	None	fig|6666666.59345.peg.1162	fig|6666666.59341.peg.1	fig|6666666.59347.peg.4074	fig|6666666.59346.peg.80	None	fig|6666666.59349.peg.2742	None	fig|6666666.59351.peg.763	None	None	fig|6666666.59354.peg.1294

9182	Periplasmic nitrate reductase component NapE	fig|6666666.59329.peg.4425	fig|6666666.59330.peg.1524	fig|6666666.59331.peg.5372	None	None	fig|6666666.59335.peg.5113	fig|6666666.59336.peg.2590	fig|6666666.59333.peg.3615	fig|6666666.59338.peg.5871	fig|6666666.59339.peg.360	fig|6666666.59340.peg.2546	fig|6666666.59337.peg.2313	fig|6666666.59342.peg.2411	None	None	fig|6666666.59345.peg.1286	fig|6666666.59341.peg.5396	fig|6666666.59347.peg.2307	fig|6666666.59346.peg.4559	fig|6666666.59348.peg.4615	None	None	None	None	None	None

5818	probable lipoprotein protein YPO1422	fig|6666666.59329.peg.1563	fig|6666666.59330.peg.1380	None	fig|6666666.59332.peg.5908	fig|6666666.59334.peg.2821	None	fig|6666666.59336.peg.1226	fig|6666666.59333.peg.745	None	fig|6666666.59339.peg.683	None	fig|6666666.59337.peg.2884	fig|6666666.59342.peg.5558	fig|6666666.71181.peg.258	fig|6666666.59344.peg.1182	fig|6666666.59345.peg.1015	None	None	None	None	fig|6666666.59349.peg.4420	None	None	fig|6666666.59353.peg.1687	fig|6666666.59355.peg.1033	fig|6666666.59354.peg.2302

4617	FIG01200393 hypothetical protein	fig|6666666.59329.peg.4759	fig|6666666.59330.peg.2980	None	fig|6666666.59332.peg.4674	None	fig|6666666.59335.peg.1557	fig|6666666.59336.peg.3486	fig|6666666.59333.peg.4512	fig|6666666.59338.peg.4944	fig|6666666.59339.peg.3858	None	fig|6666666.59337.peg.3318	fig|6666666.59342.peg.1075	None	None	fig|6666666.59345.peg.4621	fig|6666666.59341.peg.1397	fig|6666666.59347.peg.1950	None	None	fig|6666666.59349.peg.2669	None	fig|6666666.59351.peg.5164	None	None	fig|6666666.59354.peg.4644

9700	FIG00921706 hypothetical protein	fig|6666666.59329.peg.1214	fig|6666666.59330.peg.488	None	fig|6666666.59332.peg.5549	fig|6666666.59334.peg.318	fig|6666666.59335.peg.1705	fig|6666666.59336.peg.2413	fig|6666666.59333.peg.2261	None	fig|6666666.59339.peg.7247	fig|6666666.59340.peg.3501	fig|6666666.59337.peg.1561	fig|6666666.59342.peg.2122	None	None	fig|6666666.59345.peg.2220	fig|6666666.59341.peg.2304	fig|6666666.59347.peg.2788	fig|6666666.59346.peg.3535	fig|6666666.59348.peg.2782	None	None	None	None	None	None

6747	FIG01199964 hypothetical protein	None	fig|6666666.59330.peg.2852	fig|6666666.59331.peg.3635	fig|6666666.59332.peg.5472	fig|6666666.59334.peg.3286	fig|6666666.59335.peg.1632	fig|6666666.59336.peg.5799	fig|6666666.59333.peg.3674	fig|6666666.59338.peg.3472	fig|6666666.59339.peg.6535	fig|6666666.59340.peg.3422	fig|6666666.59337.peg.1962	fig|6666666.59342.peg.1259	None	None	fig|6666666.59345.peg.4076	fig|6666666.59341.peg.1219	fig|6666666.59347.peg.3635	fig|6666666.59346.peg.661	None	None	None	None	None	None	None

2131	FIG01200535 hypothetical protein	fig|6666666.59329.peg.1632	fig|6666666.59330.peg.436	None	fig|6666666.59332.peg.2495	fig|6666666.59334.peg.195	fig|6666666.59335.peg.704	fig|6666666.59336.peg.4514	fig|6666666.59333.peg.2903	fig|6666666.59338.peg.4274	fig|6666666.59339.peg.6223	fig|6666666.59340.peg.2340	fig|6666666.59337.peg.5109	None	None	None	fig|6666666.59345.peg.2659	fig|6666666.59341.peg.1420	fig|6666666.59347.peg.4481	fig|6666666.59346.peg.703	fig|6666666.59348.peg.4887	None	None	None	None	None	None

10351	hypothetical cytosolic protein	None	fig|6666666.59330.peg.3141	fig|6666666.59331.peg.2359	fig|6666666.59332.peg.3216	fig|6666666.59334.peg.4327	fig|6666666.59335.peg.3776	fig|6666666.59336.peg.6372	None	fig|6666666.59338.peg.3664	fig|6666666.59339.peg.4557	fig|6666666.59340.peg.806	fig|6666666.59337.peg.5743	fig|6666666.59342.peg.3813	None	None	fig|6666666.59345.peg.418	fig|6666666.59341.peg.5542	fig|6666666.59347.peg.531	fig|6666666.59346.peg.4043	fig|6666666.59348.peg.5239	None	None	None	None	None	None

3949	FIG01200248 hypothetical protein	fig|6666666.59329.peg.4133	fig|6666666.59330.peg.3533	fig|6666666.59331.peg.2225	fig|6666666.59332.peg.2349	fig|6666666.59334.peg.5191	fig|6666666.59335.peg.621	None	fig|6666666.59333.peg.3075	fig|6666666.59338.peg.1016	fig|6666666.59339.peg.4277	fig|6666666.59340.peg.690	fig|6666666.59337.peg.1436	fig|6666666.59342.peg.1686	None	None	fig|6666666.59345.peg.401	fig|6666666.59341.peg.2996	fig|6666666.59347.peg.1218	fig|6666666.59346.peg.3414	None	None	None	None	None	None	None

4200	FIG00919535 hypothetical protein	fig|6666666.59329.peg.1189	fig|6666666.59330.peg.1993	fig|6666666.59331.peg.4798	fig|6666666.59332.peg.802	fig|6666666.59334.peg.4041	fig|6666666.59335.peg.5928	fig|6666666.59336.peg.5708	fig|6666666.59333.peg.1622	fig|6666666.59338.peg.3920	fig|6666666.59339.peg.3532	None	fig|6666666.59337.peg.4649	fig|6666666.59342.peg.204	None	None	fig|6666666.59345.peg.4586	fig|6666666.59341.peg.364	fig|6666666.59347.peg.1971	fig|6666666.59346.peg.5226	None	None	None	None	None	None	None

10322	Response regulator VieB	fig|6666666.59329.peg.5520	fig|6666666.59330.peg.4141	fig|6666666.59331.peg.2017	fig|6666666.59332.peg.3348	None	fig|6666666.59335.peg.6068	fig|6666666.59336.peg.5298	None	fig|6666666.59338.peg.5114	fig|6666666.59339.peg.2648	None	fig|6666666.59337.peg.4167	fig|6666666.59342.peg.3122	None	None	fig|6666666.59345.peg.3435	None	fig|6666666.59347.peg.1227	fig|6666666.59346.peg.4566	None	fig|6666666.59349.peg.2500	None	fig|6666666.59351.peg.4628	None	None	fig|6666666.59354.peg.5628

6114	Uncharacterized protein conserved in bacteria	fig|6666666.59329.peg.4566	fig|6666666.59330.peg.933	None	None	None	fig|6666666.59335.peg.4227	fig|6666666.59336.peg.4179	fig|6666666.59333.peg.4453	None	fig|6666666.59339.peg.6513	None	fig|6666666.59337.peg.3964	fig|6666666.59342.peg.1860	fig|6666666.71181.peg.3435	fig|6666666.59344.peg.4918	fig|6666666.59345.peg.461	None	None	None	fig|6666666.59348.peg.522	fig|6666666.59349.peg.1840	None	fig|6666666.59351.peg.4589	fig|6666666.59353.peg.4615	fig|6666666.59355.peg.4716	None

8894	Tryptophanase EC 4.1.99.1	fig|6666666.59329.peg.895	fig|6666666.59330.peg.2396	None	fig|6666666.59332.peg.4003	fig|6666666.59334.peg.4906	fig|6666666.59335.peg.2917	fig|6666666.59336.peg.6317	fig|6666666.59333.peg.4024	fig|6666666.59338.peg.5563	fig|6666666.59339.peg.5757	None	fig|6666666.59337.peg.1496	fig|6666666.59342.peg.4239	None	None	fig|6666666.59345.peg.3006	fig|6666666.59341.peg.3969	fig|6666666.59347.peg.1941	fig|6666666.59346.peg.4631	fig|6666666.59348.peg.1116	None	None	None	None	None	None

11409	Predicted acetyltransferase	fig|6666666.59329.peg.2573	fig|6666666.59330.peg.723	None	None	fig|6666666.59334.peg.3597	fig|6666666.59335.peg.4732	fig|6666666.59336.peg.3042	fig|6666666.59333.peg.4035	fig|6666666.59338.peg.4574	fig|6666666.59339.peg.1943	fig|6666666.59340.peg.1014	fig|6666666.59337.peg.3774	fig|6666666.59342.peg.2035	None	None	fig|6666666.59345.peg.2806	fig|6666666.59341.peg.5375	fig|6666666.59347.peg.207	fig|6666666.59346.peg.3224	fig|6666666.59348.peg.2567	None	None	None	None	None	None

7133	Hemolysin	fig|6666666.59329.peg.182	None	None	fig|6666666.59332.peg.1748	None	fig|6666666.59335.peg.1068	fig|6666666.59336.peg.3077	None	fig|6666666.59338.peg.5661	fig|6666666.59339.peg.4515	None	fig|6666666.59337.peg.5384	None	fig|6666666.71181.peg.2211	fig|6666666.59344.peg.3575	fig|6666666.59345.peg.2934	fig|6666666.59341.peg.3971	fig|6666666.59347.peg.1943	fig|6666666.59346.peg.2817	fig|6666666.59348.peg.2395	None	None	None	fig|6666666.59353.peg.844	fig|6666666.59355.peg.860	None

10204	MSHA biogenesis protein MshN	fig|6666666.59329.peg.605	fig|6666666.59330.peg.12	fig|6666666.59331.peg.3274	fig|6666666.59332.peg.3981	fig|6666666.59334.peg.2435	fig|6666666.59335.peg.205	fig|6666666.59336.peg.1179	fig|6666666.59333.peg.4444	fig|6666666.59338.peg.4322	fig|6666666.59339.peg.7767	None	fig|6666666.59337.peg.2104	None	None	None	fig|6666666.59345.peg.4897	fig|6666666.59341.peg.5385	fig|6666666.59347.peg.750	fig|6666666.59346.peg.4658	fig|6666666.59348.peg.1249	None	None	None	None	None	None

7094	Alkaline phosphatase EC 3.1.3.1	fig|6666666.59329.peg.6259	fig|6666666.59330.peg.909	fig|6666666.59331.peg.5108	fig|6666666.59332.peg.3512	None	fig|6666666.59335.peg.6235	fig|6666666.59336.peg.4667	fig|6666666.59333.peg.5559	fig|6666666.59338.peg.670	fig|6666666.59339.peg.2781	None	fig|6666666.59337.peg.5586	None	None	None	fig|6666666.59345.peg.424	fig|6666666.59341.peg.4182	fig|6666666.59347.peg.384	fig|6666666.59346.peg.488	fig|6666666.59348.peg.3959	None	None	fig|6666666.59351.peg.407	None	None	None

7098	Chromosome segregation ATPase	None	fig|6666666.59330.peg.2531	None	fig|6666666.59332.peg.2516	fig|6666666.59334.peg.4725	fig|6666666.59335.peg.5360	fig|6666666.59336.peg.5002	fig|6666666.59333.peg.4728	fig|6666666.59338.peg.5323	fig|6666666.59339.peg.4375	fig|6666666.59340.peg.2720	fig|6666666.59337.peg.2566	fig|6666666.59342.peg.3728	None	None	fig|6666666.59345.peg.5645	fig|6666666.59341.peg.1982	fig|6666666.59347.peg.1482	fig|6666666.59346.peg.2275	fig|6666666.59348.peg.3386	None	None	None	None	None	None

6483	FIG01199901 hypothetical protein	fig|6666666.59329.peg.3050	fig|6666666.59330.peg.2984	fig|6666666.59331.peg.4090	fig|6666666.59332.peg.4264	None	fig|6666666.59335.peg.1553	fig|6666666.59336.peg.6514	fig|6666666.59333.peg.3669	fig|6666666.59338.peg.4980	fig|6666666.59339.peg.3853	None	fig|6666666.59337.peg.3322	fig|6666666.59342.peg.1070	None	None	fig|6666666.59345.peg.4617	fig|6666666.59341.peg.1400	fig|6666666.59347.peg.1953	fig|6666666.59346.peg.4894	fig|6666666.59348.peg.3189	None	None	None	None	None	None

7870	Conserved protein containing a Zn-ribbon motif	fig|6666666.59329.peg.3503	fig|6666666.59330.peg.4643	None	None	None	fig|6666666.59335.peg.2280	fig|6666666.59336.peg.6602	fig|6666666.59333.peg.5549	fig|6666666.59338.peg.6099	fig|6666666.59339.peg.2915	None	fig|6666666.59337.peg.5667	fig|6666666.59342.peg.1676	None	None	fig|6666666.59345.peg.107	fig|6666666.59341.peg.3251	fig|6666666.59347.peg.834	fig|6666666.59346.peg.3468	fig|6666666.59348.peg.4369	fig|6666666.59349.peg.3680	None	None	None	None	fig|6666666.59354.peg.1906

10133	FIG01200459 hypothetical protein	fig|6666666.59329.peg.1215	fig|6666666.59330.peg.489	None	fig|6666666.59332.peg.5548	fig|6666666.59334.peg.319	fig|6666666.59335.peg.1704	fig|6666666.59336.peg.2414	None	fig|6666666.59338.peg.277	fig|6666666.59339.peg.7248	fig|6666666.59340.peg.3500	fig|6666666.59337.peg.1560	fig|6666666.59342.peg.2123	None	None	fig|6666666.59345.peg.2221	fig|6666666.59341.peg.2303	fig|6666666.59347.peg.2789	fig|6666666.59346.peg.3536	fig|6666666.59348.peg.2781	None	None	None	None	None	None

9310	FIG01200132 hypothetical protein	fig|6666666.59329.peg.4077	fig|6666666.59330.peg.2140	None	fig|6666666.59332.peg.2477	None	fig|6666666.59335.peg.5136	fig|6666666.59336.peg.1161	fig|6666666.59333.peg.1739	fig|6666666.59338.peg.3570	fig|6666666.59339.peg.251	fig|6666666.59340.peg.779	fig|6666666.59337.peg.180	fig|6666666.59342.peg.3129	None	None	fig|6666666.59345.peg.4509	fig|6666666.59341.peg.530	fig|6666666.59347.peg.2819	fig|6666666.59346.peg.2963	fig|6666666.59348.peg.3273	None	None	None	None	None	None

2260	Cobalamin synthase	fig|6666666.59329.peg.5309	fig|6666666.59330.peg.2662	fig|6666666.59331.peg.6372	None	None	fig|6666666.59335.peg.2167	fig|6666666.59336.peg.6343	fig|6666666.59333.peg.1785	None	fig|6666666.59339.peg.3906	None	fig|6666666.59337.peg.1981	fig|6666666.59342.peg.2059	None	None	fig|6666666.59345.peg.4673	fig|6666666.59341.peg.548	fig|6666666.59347.peg.3354	fig|6666666.59346.peg.2034	fig|6666666.59348.peg.4807	None	fig|6666666.59350.peg.294	fig|6666666.59351.peg.3917	None	None	None

2455	Transcriptional regulator, PadR family	fig|6666666.59329.peg.2453	fig|6666666.59330.peg.1677	None	fig|6666666.59332.peg.1854	fig|6666666.59334.peg.2177	fig|6666666.59335.peg.449	fig|6666666.59336.peg.1594	fig|6666666.59333.peg.3009	fig|6666666.59338.peg.1056	fig|6666666.59339.peg.1145	None	fig|6666666.59337.peg.1679	fig|6666666.59342.peg.4415	None	None	fig|6666666.59345.peg.5401	fig|6666666.59341.peg.947	fig|6666666.59347.peg.4925	fig|6666666.59346.peg.1001	fig|6666666.59348.peg.140	None	None	None	None	None	None

656	Delta 1-pyrroline-5-carboxylate dehydrogenase domain protein	fig|6666666.59329.peg.227	fig|6666666.59330.peg.2291	None	fig|6666666.59332.peg.4004	fig|6666666.59334.peg.403	fig|6666666.59335.peg.3572	fig|6666666.59336.peg.3984	fig|6666666.59333.peg.3587	fig|6666666.59338.peg.5300	None	None	fig|6666666.59337.peg.277	fig|6666666.59342.peg.4360	None	None	fig|6666666.59345.peg.5225	fig|6666666.59341.peg.4449	fig|6666666.59347.peg.4694	fig|6666666.59346.peg.3970	fig|6666666.59348.peg.4756	None	None	fig|6666666.59351.peg.5419	None	None	None

6363	NADPH dependent preQ0 reductase EC 1.7.1.13	fig|6666666.59329.peg.4304	fig|6666666.59330.peg.2346	None	fig|6666666.59332.peg.2590	fig|6666666.59334.peg.2830	fig|6666666.59335.peg.4012	fig|6666666.59336.peg.2802	None	None	fig|6666666.59339.peg.1774	None	fig|6666666.59337.peg.3612	fig|6666666.59342.peg.3718	None	None	fig|6666666.59345.peg.1847	None	fig|6666666.59347.peg.775	fig|6666666.59346.peg.210	fig|6666666.59348.peg.1900	fig|6666666.59349.peg.1589	None	fig|6666666.59351.peg.1007	None	None	fig|6666666.59354.peg.2193

11354	FIG01199602 hypothetical protein	fig|6666666.59329.peg.1748	fig|6666666.59330.peg.3513	None	fig|6666666.59332.peg.42	fig|6666666.59334.peg.5136	fig|6666666.59335.peg.73	fig|6666666.59336.peg.1492	fig|6666666.59333.peg.1172	fig|6666666.59338.peg.3363	fig|6666666.59339.peg.7627	fig|6666666.59340.peg.732	fig|6666666.59337.peg.2785	None	None	None	fig|6666666.59345.peg.2749	fig|6666666.59341.peg.4301	fig|6666666.59347.peg.3682	fig|6666666.59346.peg.2399	fig|6666666.59348.peg.1683	None	None	None	None	None	None

11176	FIG01199933 hypothetical protein	fig|6666666.59329.peg.2846	fig|6666666.59330.peg.4803	None	None	fig|6666666.59334.peg.4980	None	fig|6666666.59336.peg.5625	fig|6666666.59333.peg.3321	fig|6666666.59338.peg.2336	fig|6666666.59339.peg.1009	None	fig|6666666.59337.peg.1190	None	None	None	fig|6666666.59345.peg.10	fig|6666666.59341.peg.3023	fig|6666666.59347.peg.856	fig|6666666.59346.peg.5059	None	fig|6666666.59349.peg.4158	fig|6666666.59350.peg.1364	None	None	fig|6666666.59355.peg.5319	fig|6666666.59354.peg.4064

5921	FIG01199712 hypothetical protein	fig|6666666.59329.peg.100	fig|6666666.59330.peg.2177	None	fig|6666666.59332.peg.176	fig|6666666.59334.peg.653	fig|6666666.59335.peg.2794	fig|6666666.59336.peg.4277	None	fig|6666666.59338.peg.2433	fig|6666666.59339.peg.4921	fig|6666666.59340.peg.337	fig|6666666.59337.peg.85	fig|6666666.59342.peg.3768	None	None	fig|6666666.59345.peg.3757	fig|6666666.59341.peg.82	fig|6666666.59347.peg.4026	fig|6666666.59346.peg.1364	fig|6666666.59348.peg.417	None	None	None	None	None	None

9137	Chromate transport protein ChrA	fig|6666666.59329.peg.3136	fig|6666666.59330.peg.2805	fig|6666666.59331.peg.1246	None	fig|6666666.59334.peg.1325	fig|6666666.59335.peg.3509	fig|6666666.59336.peg.1096	fig|6666666.59333.peg.1132	fig|6666666.59338.peg.3785	fig|6666666.59339.peg.6825	None	fig|6666666.59337.peg.1341	fig|6666666.59342.peg.6081	None	None	fig|6666666.59345.peg.2304	None	fig|6666666.59347.peg.2956	fig|6666666.59346.peg.4743	None	fig|6666666.59349.peg.2202	None	fig|6666666.59351.peg.4617	None	None	None

1822	Predicted transcriptional regulator	fig|6666666.59329.peg.6188	fig|6666666.59330.peg.859	fig|6666666.59331.peg.3522	fig|6666666.59332.peg.5727	None	fig|6666666.59335.peg.4214	fig|6666666.59336.peg.3854	fig|6666666.59333.peg.526	fig|6666666.59338.peg.3311	fig|6666666.59339.peg.8767	None	fig|6666666.59337.peg.5722	fig|6666666.59342.peg.2426	None	None	fig|6666666.59345.peg.4643	fig|6666666.59341.peg.1925	fig|6666666.59347.peg.4722	None	fig|6666666.59348.peg.5446	None	None	fig|6666666.59351.peg.1135	None	None	None

5580	Ubiquitin-protein ligase	fig|6666666.59329.peg.4746	fig|6666666.59330.peg.4911	fig|6666666.59331.peg.4268	None	fig|6666666.59334.peg.3228	fig|6666666.59335.peg.1325	fig|6666666.59336.peg.5558	fig|6666666.59333.peg.3806	fig|6666666.59338.peg.6164	fig|6666666.59339.peg.935	None	fig|6666666.59337.peg.1749	fig|6666666.59342.peg.3713	None	None	fig|6666666.59345.peg.896	fig|6666666.59341.peg.3028	fig|6666666.59347.peg.851	fig|6666666.59346.peg.1352	fig|6666666.59348.peg.3317	None	None	None	None	None	None

7377	ABC-type tungstate transport system, permease protein	fig|6666666.59329.peg.2467	fig|6666666.59330.peg.3813	fig|6666666.59331.peg.5672	None	fig|6666666.59334.peg.4181	fig|6666666.59335.peg.5039	fig|6666666.59336.peg.4578	fig|6666666.59333.peg.3240	None	fig|6666666.59339.peg.1214	None	fig|6666666.59337.peg.2717	fig|6666666.59342.peg.1127	None	None	fig|6666666.59345.peg.3196	fig|6666666.59341.peg.4007	fig|6666666.59347.peg.1701	fig|6666666.59346.peg.4519	fig|6666666.59348.peg.3730	None	fig|6666666.59350.peg.404	None	None	None	None

5344	FIG01199606 hypothetical protein	fig|6666666.59329.peg.3199	fig|6666666.59330.peg.4782	None	fig|6666666.59332.peg.3518	fig|6666666.59334.peg.1272	fig|6666666.59335.peg.2347	fig|6666666.59336.peg.5108	fig|6666666.59333.peg.3744	fig|6666666.59338.peg.4407	fig|6666666.59339.peg.2872	None	fig|6666666.59337.peg.2512	fig|6666666.59342.peg.892	None	None	fig|6666666.59345.peg.132	fig|6666666.59341.peg.3015	fig|6666666.59347.peg.864	fig|6666666.59346.peg.2907	fig|6666666.59348.peg.4203	None	None	None	None	None	None

6380	Iron-regulated protein A precursor	fig|6666666.59329.peg.5294	fig|6666666.59330.peg.2304	fig|6666666.59331.peg.3395	fig|6666666.59332.peg.2676	None	fig|6666666.59335.peg.4607	fig|6666666.59336.peg.4389	fig|6666666.59333.peg.3695	fig|6666666.59338.peg.4443	fig|6666666.59339.peg.3737	None	fig|6666666.59337.peg.1771	fig|6666666.59342.peg.2333	None	None	fig|6666666.59345.peg.874	fig|6666666.59341.peg.4886	fig|6666666.59347.peg.2244	fig|6666666.59346.peg.4447	None	None	fig|6666666.59350.peg.1274	None	None	None	None

2161	FIG01199972 hypothetical protein	fig|6666666.59329.peg.4693	None	fig|6666666.59331.peg.3017	fig|6666666.59332.peg.5732	fig|6666666.59334.peg.3363	fig|6666666.59335.peg.2429	fig|6666666.59336.peg.1507	fig|6666666.59333.peg.2055	fig|6666666.59338.peg.3598	fig|6666666.59339.peg.5252	None	fig|6666666.59337.peg.5070	fig|6666666.59342.peg.1957	None	None	fig|6666666.59345.peg.4360	fig|6666666.59341.peg.1625	fig|6666666.59347.peg.4438	fig|6666666.59346.peg.3681	fig|6666666.59348.peg.1269	None	None	None	None	None	None

73	FIG01199541 hypothetical protein	fig|6666666.59329.peg.6154	fig|6666666.59330.peg.3781	fig|6666666.59331.peg.1029	fig|6666666.59332.peg.5480	fig|6666666.59334.peg.4886	None	fig|6666666.59336.peg.4409	fig|6666666.59333.peg.4231	fig|6666666.59338.peg.3080	None	fig|6666666.59340.peg.1934	fig|6666666.59337.peg.2218	fig|6666666.59342.peg.5610	None	None	fig|6666666.59345.peg.3000	fig|6666666.59341.peg.2661	fig|6666666.59347.peg.210	fig|6666666.59346.peg.896	fig|6666666.59348.peg.3686	None	None	None	None	None	None

4308	FIG00920623 hypothetical protein	fig|6666666.59329.peg.6116	fig|6666666.59330.peg.1166	fig|6666666.59331.peg.4155	fig|6666666.59332.peg.295	None	fig|6666666.59335.peg.1996	fig|6666666.59336.peg.55	fig|6666666.59333.peg.653	None	fig|6666666.59339.peg.6292	fig|6666666.59340.peg.667	fig|6666666.59337.peg.470	fig|6666666.59342.peg.5522	None	None	fig|6666666.59345.peg.2618	fig|6666666.59341.peg.5104	fig|6666666.59347.peg.4513	fig|6666666.59346.peg.1712	fig|6666666.59348.peg.3018	None	None	None	None	None	None

2369	Phospholipid-binding protein	fig|6666666.59329.peg.1253	fig|6666666.59330.peg.3866	fig|6666666.59331.peg.5497	fig|6666666.59332.peg.522	None	fig|6666666.59335.peg.5334	fig|6666666.59336.peg.3330	None	None	fig|6666666.59339.peg.183	None	fig|6666666.59337.peg.1933	None	fig|6666666.71181.peg.2517	fig|6666666.59344.peg.4081	fig|6666666.59345.peg.4025	fig|6666666.59341.peg.2969	None	fig|6666666.59346.peg.186	None	None	None	fig|6666666.59351.peg.4687	fig|6666666.59353.peg.2127	fig|6666666.59355.peg.2693	None

1717	no significant database hits	fig|6666666.59329.peg.2787	None	fig|6666666.59331.peg.714	fig|6666666.59332.peg.5268	fig|6666666.59334.peg.2960	None	fig|6666666.59336.peg.1750	None	None	None	fig|6666666.59340.peg.1814	fig|6666666.59337.peg.1840	fig|6666666.59342.peg.5838	fig|6666666.71181.peg.3816	fig|6666666.59344.peg.5081	fig|6666666.59345.peg.4213	fig|6666666.59341.peg.2961	fig|6666666.59347.peg.1389	fig|6666666.59346.peg.2509	None	None	None	None	fig|6666666.59353.peg.2064	fig|6666666.59355.peg.2764	None

1780	FIG01200289 hypothetical protein	fig|6666666.59329.peg.102	fig|6666666.59330.peg.2179	None	None	None	fig|6666666.59335.peg.4672	fig|6666666.59336.peg.4060	fig|6666666.59333.peg.2394	fig|6666666.59338.peg.2430	fig|6666666.59339.peg.7409	None	fig|6666666.59337.peg.87	fig|6666666.59342.peg.3770	None	None	fig|6666666.59345.peg.3755	fig|6666666.59341.peg.84	fig|6666666.59347.peg.4028	None	fig|6666666.59348.peg.211	fig|6666666.59349.peg.4576	None	fig|6666666.59351.peg.1332	None	None	fig|6666666.59354.peg.3264

6871	FIG01202541 hypothetical protein	fig|6666666.59329.peg.5720	fig|6666666.59330.peg.2726	fig|6666666.59331.peg.5539	fig|6666666.59332.peg.971	fig|6666666.59334.peg.4134	fig|6666666.59335.peg.748	fig|6666666.59336.peg.5600	fig|6666666.59333.peg.4546	fig|6666666.59338.peg.4483	None	fig|6666666.59340.peg.3148	fig|6666666.59337.peg.5899	None	None	None	fig|6666666.59345.peg.3407	fig|6666666.59341.peg.5267	fig|6666666.59347.peg.162	fig|6666666.59346.peg.2579	fig|6666666.59348.peg.4017	None	None	None	None	None	None

2642	hypothetical protein	fig|6666666.59329.peg.1747	fig|6666666.59330.peg.755	fig|6666666.59331.peg.300	fig|6666666.59332.peg.43	None	fig|6666666.59335.peg.1089	fig|6666666.59336.peg.538	fig|6666666.59333.peg.2083	fig|6666666.59338.peg.334	fig|6666666.59339.peg.1869	None	fig|6666666.59337.peg.2786	None	None	None	fig|6666666.59345.peg.2771	None	fig|6666666.59347.peg.4692	fig|6666666.59346.peg.2396	None	fig|6666666.59349.peg.2563	None	fig|6666666.59351.peg.4434	None	None	fig|6666666.59354.peg.5622

5309	FIG01199563 hypothetical protein	fig|6666666.59329.peg.5154	fig|6666666.59330.peg.2587	fig|6666666.59331.peg.2357	None	fig|6666666.59334.peg.4602	fig|6666666.59335.peg.5746	fig|6666666.59336.peg.4844	fig|6666666.59333.peg.4682	fig|6666666.59338.peg.5158	None	None	fig|6666666.59337.peg.2731	fig|6666666.59342.peg.2887	None	None	fig|6666666.59345.peg.3207	fig|6666666.59341.peg.2902	fig|6666666.59347.peg.1715	fig|6666666.59346.peg.3280	fig|6666666.59348.peg.3913	None	fig|6666666.59350.peg.395	None	None	None	None

5178	FIG00920016 hypothetical protein	fig|6666666.59329.peg.5854	fig|6666666.59330.peg.4341	fig|6666666.59331.peg.3831	fig|6666666.59332.peg.5984	None	fig|6666666.59335.peg.569	fig|6666666.59336.peg.2246	fig|6666666.59333.peg.4299	fig|6666666.59338.peg.5425	fig|6666666.59339.peg.2932	None	fig|6666666.59337.peg.3026	fig|6666666.59342.peg.2286	None	None	fig|6666666.59345.peg.81	fig|6666666.59341.peg.5837	fig|6666666.59347.peg.962	fig|6666666.59346.peg.1890	fig|6666666.59348.peg.5282	None	None	None	None	None	None

6586	FIG01199561 hypothetical protein	fig|6666666.59329.peg.5494	fig|6666666.59330.peg.1478	None	None	fig|6666666.59334.peg.2093	fig|6666666.59335.peg.738	fig|6666666.59336.peg.3770	fig|6666666.59333.peg.1222	None	fig|6666666.59339.peg.5616	None	fig|6666666.59337.peg.198	fig|6666666.59342.peg.4002	None	None	fig|6666666.59345.peg.4527	fig|6666666.59341.peg.726	fig|6666666.59347.peg.3442	None	fig|6666666.59348.peg.788	fig|6666666.59349.peg.977	None	fig|6666666.59351.peg.1569	None	None	fig|6666666.59354.peg.2707

8323	Arginine deiminase EC 3.5.3.6	fig|6666666.59329.peg.1442	fig|6666666.59330.peg.65	None	fig|6666666.59332.peg.1287	fig|6666666.59334.peg.4696	fig|6666666.59335.peg.222	fig|6666666.59336.peg.1788	fig|6666666.59333.peg.2812	fig|6666666.59338.peg.127	fig|6666666.59339.peg.6484	None	fig|6666666.59337.peg.852	fig|6666666.59342.peg.428	None	None	fig|6666666.59345.peg.4842	fig|6666666.59341.peg.4997	fig|6666666.59347.peg.2611	fig|6666666.59346.peg.620	fig|6666666.59348.peg.4206	None	None	None	None	None	None

8322	FIG01199582 hypothetical protein	None	fig|6666666.59330.peg.1005	fig|6666666.59331.peg.1509	fig|6666666.59332.peg.3582	fig|6666666.59334.peg.420	fig|6666666.59335.peg.4613	fig|6666666.59336.peg.1642	fig|6666666.59333.peg.3251	fig|6666666.59338.peg.1200	fig|6666666.59339.peg.7683	None	fig|6666666.59337.peg.2525	fig|6666666.59342.peg.5389	None	None	fig|6666666.59345.peg.5517	fig|6666666.59341.peg.4203	fig|6666666.59347.peg.5232	fig|6666666.59346.peg.4510	fig|6666666.59348.peg.3059	None	None	None	None	None	None

10055	Thiol-disulfide isomerase	fig|6666666.59329.peg.1568	None	fig|6666666.59331.peg.6270	fig|6666666.59332.peg.6092	fig|6666666.59334.peg.5556	fig|6666666.59335.peg.3454	fig|6666666.59336.peg.4186	fig|6666666.59333.peg.5173	fig|6666666.59338.peg.5341	fig|6666666.59339.peg.2799	None	fig|6666666.59337.peg.3552	fig|6666666.59342.peg.4792	None	None	fig|6666666.59345.peg.467	fig|6666666.59341.peg.4457	fig|6666666.59347.peg.813	fig|6666666.59346.peg.3458	fig|6666666.59348.peg.823	None	None	None	None	None	None

9097	FIG01199772 hypothetical protein	fig|6666666.59329.peg.5708	fig|6666666.59330.peg.2126	fig|6666666.59331.peg.3070	fig|6666666.59332.peg.3744	fig|6666666.59334.peg.5115	fig|6666666.59335.peg.989	fig|6666666.59336.peg.106	None	fig|6666666.59338.peg.3726	fig|6666666.59339.peg.4458	fig|6666666.59340.peg.3038	fig|6666666.59337.peg.5129	None	None	None	fig|6666666.59345.peg.2269	fig|6666666.59341.peg.1921	fig|6666666.59347.peg.655	fig|6666666.59346.peg.1098	fig|6666666.59348.peg.3675	None	None	None	None	None	None

5094	FIG01200211 hypothetical protein	fig|6666666.59329.peg.6498	fig|6666666.59330.peg.2597	fig|6666666.59331.peg.5429	None	fig|6666666.59334.peg.3093	fig|6666666.59335.peg.2200	fig|6666666.59336.peg.3291	fig|6666666.59333.peg.3222	fig|6666666.59338.peg.1304	fig|6666666.59339.peg.1295	None	fig|6666666.59337.peg.3705	fig|6666666.59342.peg.218	None	None	fig|6666666.59345.peg.3215	fig|6666666.59341.peg.2892	fig|6666666.59347.peg.1723	fig|6666666.59346.peg.3583	fig|6666666.59348.peg.3797	None	None	None	None	None	None

4499	FIG01260270 hypothetical protein	None	fig|6666666.59330.peg.232	fig|6666666.59331.peg.5859	fig|6666666.59332.peg.1718	None	fig|6666666.59335.peg.21	fig|6666666.59336.peg.6105	fig|6666666.59333.peg.678	fig|6666666.59338.peg.74	fig|6666666.59339.peg.7548	fig|6666666.59340.peg.163	fig|6666666.59337.peg.949	fig|6666666.59342.peg.5139	None	None	fig|6666666.59345.peg.2726	fig|6666666.59341.peg.4149	fig|6666666.59347.peg.4570	fig|6666666.59346.peg.3442	fig|6666666.59348.peg.2082	None	None	None	None	None	None

4700	Arsenical resistance operon repressor	fig|6666666.59329.peg.1258	fig|6666666.59330.peg.3871	None	fig|6666666.59332.peg.2815	fig|6666666.59334.peg.2446	fig|6666666.59335.peg.1464	fig|6666666.59336.peg.5533	fig|6666666.59333.peg.5094	fig|6666666.59338.peg.3693	fig|6666666.59339.peg.4431	fig|6666666.59340.peg.1710	fig|6666666.59337.peg.1937	fig|6666666.59342.peg.5259	None	None	fig|6666666.59345.peg.4030	fig|6666666.59341.peg.649	fig|6666666.59347.peg.3941	fig|6666666.59346.peg.2752	None	None	None	None	None	None	None

8774	AraC-type DNA-binding domain-containing protein	fig|6666666.59329.peg.3968	fig|6666666.59330.peg.2714	fig|6666666.59331.peg.5994	fig|6666666.59332.peg.992	fig|6666666.59334.peg.2858	fig|6666666.59335.peg.4526	None	fig|6666666.59333.peg.5171	fig|6666666.59338.peg.5473	fig|6666666.59339.peg.2481	None	fig|6666666.59337.peg.2443	fig|6666666.59342.peg.2957	None	None	fig|6666666.59345.peg.606	fig|6666666.59341.peg.223	fig|6666666.59347.peg.547	fig|6666666.59346.peg.3021	fig|6666666.59348.peg.4375	None	None	None	None	None	None

1749	Putative sugar phosphotransferase component II B	fig|6666666.59329.peg.3781	None	fig|6666666.59331.peg.949	fig|6666666.59332.peg.1725	fig|6666666.59334.peg.2707	None	None	fig|6666666.59333.peg.2785	fig|6666666.59338.peg.3430	fig|6666666.59339.peg.2336	None	None	None	fig|6666666.71181.peg.558	fig|6666666.59344.peg.1907	fig|6666666.59345.peg.2963	fig|6666666.59341.peg.2870	fig|6666666.59347.peg.495	fig|6666666.59346.peg.3450	fig|6666666.59348.peg.2983	None	None	None	fig|6666666.59353.peg.1190	fig|6666666.59355.peg.287	None

6796	Pyridoxamine 5'-phosphate oxidase-related, FMN-binding	None	fig|6666666.59330.peg.3321	fig|6666666.59331.peg.1310	fig|6666666.59332.peg.4566	None	fig|6666666.59335.peg.4981	fig|6666666.59336.peg.5581	fig|6666666.59333.peg.84	fig|6666666.59338.peg.2519	fig|6666666.59339.peg.4341	fig|6666666.59340.peg.2365	fig|6666666.59337.peg.3258	fig|6666666.59342.peg.2222	None	None	fig|6666666.59345.peg.525	fig|6666666.59341.peg.3327	fig|6666666.59347.peg.3691	fig|6666666.59346.peg.4059	None	None	None	None	None	None	None

2142	Predicted manganese transporter, 11 TMS	None	None	fig|6666666.59331.peg.4306	fig|6666666.59332.peg.3685	None	fig|6666666.59335.peg.584	None	fig|6666666.59333.peg.2916	fig|6666666.59338.peg.6182	fig|6666666.59339.peg.508	fig|6666666.59340.peg.1238	fig|6666666.59337.peg.2317	fig|6666666.59342.peg.5201	None	None	None	fig|6666666.59341.peg.5978	fig|6666666.59347.peg.2302	fig|6666666.59346.peg.4714	None	fig|6666666.59349.peg.4108	None	fig|6666666.59351.peg.1090	None	None	fig|6666666.59354.peg.1949

3435	Isopentenyl-diphosphate delta-isomerase EC 5.3.3.2	fig|6666666.59329.peg.3495	fig|6666666.59330.peg.3930	fig|6666666.59331.peg.1923	None	fig|6666666.59334.peg.4742	fig|6666666.59335.peg.6108	fig|6666666.59336.peg.4322	fig|6666666.59333.peg.2334	fig|6666666.59338.peg.885	fig|6666666.59339.peg.2841	None	fig|6666666.59337.peg.5807	None	None	None	fig|6666666.59345.peg.102	fig|6666666.59341.peg.5325	fig|6666666.59347.peg.991	fig|6666666.59346.peg.3463	fig|6666666.59348.peg.4197	None	None	None	None	None	None

8015	Methylcrotonyl-CoA carboxylase carboxyl transferase subunit EC 6.4.1.4	fig|6666666.59329.peg.1766	fig|6666666.59330.peg.4350	None	None	None	fig|6666666.59335.peg.5609	fig|6666666.59336.peg.3643	fig|6666666.59333.peg.5087	fig|6666666.59338.peg.5090	fig|6666666.59339.peg.4617	None	fig|6666666.59337.peg.1458	None	None	None	fig|6666666.59345.peg.384	fig|6666666.59341.peg.1695	fig|6666666.59347.peg.1168	None	None	fig|6666666.59349.peg.2159	fig|6666666.59350.peg.2560	fig|6666666.59351.peg.3698	None	None	fig|6666666.59354.peg.2507

4723	FIG01200178 hypothetical protein	fig|6666666.59329.peg.2251	fig|6666666.59330.peg.3172	fig|6666666.59331.peg.353	fig|6666666.59332.peg.4688	fig|6666666.59334.peg.4691	fig|6666666.59335.peg.1218	None	fig|6666666.59333.peg.2791	None	fig|6666666.59339.peg.1502	None	fig|6666666.59337.peg.4563	fig|6666666.59342.peg.2339	None	None	fig|6666666.59345.peg.3245	fig|6666666.59341.peg.917	fig|6666666.59347.peg.1759	fig|6666666.59346.peg.5100	fig|6666666.59348.peg.4460	None	None	None	None	None	None

3742	FIG01200260 hypothetical protein	fig|6666666.59329.peg.6268	fig|6666666.59330.peg.402	None	fig|6666666.59332.peg.32	None	None	fig|6666666.59336.peg.6357	fig|6666666.59333.peg.851	fig|6666666.59338.peg.1313	None	fig|6666666.59340.peg.2198	fig|6666666.59337.peg.986	None	None	None	fig|6666666.59345.peg.2692	None	fig|6666666.59347.peg.5037	fig|6666666.59346.peg.1084	fig|6666666.59348.peg.671	fig|6666666.59349.peg.3592	None	fig|6666666.59351.peg.4838	None	None	fig|6666666.59354.peg.6487

253	Alkylated DNA repair protein AlkB	fig|6666666.59329.peg.6584	fig|6666666.59330.peg.3782	None	None	fig|6666666.59334.peg.544	None	fig|6666666.59336.peg.4410	fig|6666666.59333.peg.4230	fig|6666666.59338.peg.2697	fig|6666666.59339.peg.4181	None	fig|6666666.59337.peg.2219	fig|6666666.59342.peg.1483	None	None	fig|6666666.59345.peg.2999	fig|6666666.59341.peg.3092	fig|6666666.59347.peg.3583	None	None	fig|6666666.59349.peg.326	None	fig|6666666.59351.peg.3934	None	None	fig|6666666.59354.peg.2167

10180	FIG01202625 hypothetical protein	fig|6666666.59329.peg.1780	fig|6666666.59330.peg.841	fig|6666666.59331.peg.6273	fig|6666666.59332.peg.5109	fig|6666666.59334.peg.3759	None	fig|6666666.59336.peg.922	fig|6666666.59333.peg.574	fig|6666666.59338.peg.304	None	fig|6666666.59340.peg.870	fig|6666666.59337.peg.334	fig|6666666.59342.peg.5993	None	None	fig|6666666.59345.peg.2484	fig|6666666.59341.peg.5007	fig|6666666.59347.peg.4869	fig|6666666.59346.peg.1980	None	None	None	None	None	None	None

794	FIG01200147 hypothetical protein	None	fig|6666666.59330.peg.2653	None	fig|6666666.59332.peg.823	fig|6666666.59334.peg.2170	fig|6666666.59335.peg.2178	fig|6666666.59336.peg.3972	fig|6666666.59333.peg.1775	None	fig|6666666.59339.peg.3918	fig|6666666.59340.peg.2507	fig|6666666.59337.peg.1989	fig|6666666.59342.peg.3509	None	None	fig|6666666.59345.peg.4681	fig|6666666.59341.peg.2720	fig|6666666.59347.peg.3347	fig|6666666.59346.peg.2044	fig|6666666.59348.peg.1911	None	None	None	None	None	None

10356	Inosine-uridine preferring nucleoside hydrolase EC 3.2.2.1	fig|6666666.59329.peg.872	fig|6666666.59330.peg.1220	None	None	None	None	fig|6666666.59336.peg.1814	None	None	None	fig|6666666.59340.peg.349	fig|6666666.59337.peg.1182	fig|6666666.59342.peg.5035	fig|6666666.71181.peg.3217	fig|6666666.59344.peg.1756	fig|6666666.59345.peg.1166	None	None	None	fig|6666666.59348.peg.4343	fig|6666666.59349.peg.4603	fig|6666666.59350.peg.3382	None	fig|6666666.59353.peg.2029	fig|6666666.59355.peg.4436	fig|6666666.59354.peg.5156

7968	Glyoxalase family protein	fig|6666666.59329.peg.4881	None	fig|6666666.59331.peg.6233	fig|6666666.59332.peg.2861	fig|6666666.59334.peg.2721	None	fig|6666666.59336.peg.2928	fig|6666666.59333.peg.2101	fig|6666666.59338.peg.5279	None	None	None	None	None	None	fig|6666666.59345.peg.1763	fig|6666666.59341.peg.5524	fig|6666666.59347.peg.1634	fig|6666666.59346.peg.3656	None	fig|6666666.59349.peg.3999	None	fig|6666666.59351.peg.1259	None	fig|6666666.59355.peg.5888	fig|6666666.59354.peg.554

9172	O-methyltransferase-related protein	fig|6666666.59329.peg.780	fig|6666666.59330.peg.1577	fig|6666666.59331.peg.1721	fig|6666666.59332.peg.565	None	fig|6666666.59335.peg.4042	fig|6666666.59336.peg.1365	fig|6666666.59333.peg.1960	fig|6666666.59338.peg.1971	fig|6666666.59339.peg.8586	None	fig|6666666.59337.peg.1660	None	None	None	fig|6666666.59345.peg.5628	fig|6666666.59341.peg.4971	fig|6666666.59347.peg.4647	fig|6666666.59346.peg.1648	fig|6666666.59348.peg.1070	None	None	None	None	None	None

632	Lactoylglutathione lyase and related lyases	None	fig|6666666.59330.peg.3980	fig|6666666.59331.peg.4503	fig|6666666.59332.peg.2194	None	None	None	fig|6666666.59333.peg.5544	fig|6666666.59338.peg.556	None	None	fig|6666666.59337.peg.2168	fig|6666666.59342.peg.6198	fig|6666666.71181.peg.1373	fig|6666666.59344.peg.4563	fig|6666666.59345.peg.3988	fig|6666666.59341.peg.2040	None	fig|6666666.59346.peg.5133	None	None	fig|6666666.59350.peg.5270	None	fig|6666666.59353.peg.3347	fig|6666666.59355.peg.1159	None

1307	FIG00922492 hypothetical protein	fig|6666666.59329.peg.1403	fig|6666666.59330.peg.4472	fig|6666666.59331.peg.5274	None	fig|6666666.59334.peg.3084	None	fig|6666666.59336.peg.2159	fig|6666666.59333.peg.3554	fig|6666666.59338.peg.3390	fig|6666666.59339.peg.7135	None	fig|6666666.59337.peg.5641	fig|6666666.59342.peg.2563	None	None	fig|6666666.59345.peg.2145	fig|6666666.59341.peg.1686	fig|6666666.59347.peg.2841	fig|6666666.59346.peg.3540	fig|6666666.59348.peg.2631	None	None	None	None	None	None

7753	SH3 domain protein	fig|6666666.59329.peg.2397	fig|6666666.59330.peg.2109	fig|6666666.59331.peg.5824	fig|6666666.59332.peg.6094	None	fig|6666666.59335.peg.985	fig|6666666.59336.peg.2222	fig|6666666.59333.peg.1447	fig|6666666.59338.peg.5236	fig|6666666.59339.peg.4464	None	fig|6666666.59337.peg.5491	fig|6666666.59342.peg.1459	None	None	fig|6666666.59345.peg.497	fig|6666666.59341.peg.1916	fig|6666666.59347.peg.658	None	fig|6666666.59348.peg.3673	None	None	None	None	None	None

2574	Urea ABC transporter, permease protein UrtB	None	None	fig|6666666.59331.peg.5785	fig|6666666.59332.peg.3456	None	fig|6666666.59335.peg.3944	None	fig|6666666.59333.peg.4248	fig|6666666.59338.peg.1461	fig|6666666.59339.peg.1357	None	None	None	fig|6666666.71181.peg.3321	fig|6666666.59344.peg.5596	None	fig|6666666.59341.peg.239	fig|6666666.59347.peg.560	fig|6666666.59346.peg.1307	fig|6666666.59348.peg.551	None	fig|6666666.59350.peg.1467	None	fig|6666666.59353.peg.3711	fig|6666666.59355.peg.5288	None

5593	hypothetical protein	fig|6666666.59329.peg.6051	fig|6666666.59330.peg.2947	fig|6666666.59331.peg.1291	fig|6666666.59332.peg.6136	None	None	fig|6666666.59336.peg.6607	None	fig|6666666.59338.peg.5780	None	None	fig|6666666.59337.peg.3458	fig|6666666.59342.peg.6229	None	None	fig|6666666.59345.peg.182	fig|6666666.59341.peg.5258	fig|6666666.59347.peg.1409	fig|6666666.59346.peg.4029	None	fig|6666666.59349.peg.1834	None	fig|6666666.59351.peg.5267	None	None	fig|6666666.59354.peg.594

4844	Inner membrane protein ygjV	fig|6666666.59329.peg.2745	fig|6666666.59330.peg.4288	fig|6666666.59331.peg.4484	None	fig|6666666.59334.peg.2224	None	fig|6666666.59336.peg.4889	None	fig|6666666.59338.peg.4602	None	fig|6666666.59340.peg.2371	fig|6666666.59337.peg.4032	None	fig|6666666.71181.peg.4389	fig|6666666.59344.peg.5655	fig|6666666.59345.peg.2113	fig|6666666.59341.peg.1263	fig|6666666.59347.peg.183	None	None	None	None	None	fig|6666666.59353.peg.2392	fig|6666666.59355.peg.1665	None

1099	Chitooligosaccharide deacetylase EC 3.5.1.- ; putative uricase	None	None	fig|6666666.59331.peg.6172	fig|6666666.59332.peg.2252	fig|6666666.59334.peg.2226	fig|6666666.59335.peg.1140	None	None	fig|6666666.59338.peg.5028	fig|6666666.59339.peg.1646	None	None	None	fig|6666666.71181.peg.883	fig|6666666.59344.peg.4759	None	fig|6666666.59341.peg.4531	fig|6666666.59347.peg.3330	fig|6666666.59346.peg.4745	fig|6666666.59348.peg.3210	None	fig|6666666.59350.peg.3208	None	fig|6666666.59353.peg.4550	fig|6666666.59355.peg.4647	None

1320	ABC-type multidrug transport system, permease component	None	None	None	None	None	fig|6666666.59335.peg.2614	fig|6666666.59336.peg.5308	fig|6666666.59333.peg.4418	fig|6666666.59338.peg.5338	fig|6666666.59339.peg.2236	fig|6666666.59340.peg.1763	fig|6666666.59337.peg.5313	fig|6666666.59342.peg.5943	fig|6666666.71181.peg.4641	fig|6666666.59344.peg.5936	fig|6666666.59345.peg.4008	fig|6666666.59341.peg.340	fig|6666666.59347.peg.3658	None	None	None	None	None	fig|6666666.59353.peg.5027	fig|6666666.59355.peg.5113	None

8136	Oligoendopeptidase F	fig|6666666.59329.peg.3347	fig|6666666.59330.peg.2659	None	fig|6666666.59332.peg.830	None	fig|6666666.59335.peg.2171	fig|6666666.59336.peg.5747	fig|6666666.59333.peg.1782	fig|6666666.59338.peg.3798	fig|6666666.59339.peg.3911	None	fig|6666666.59337.peg.1984	fig|6666666.59342.peg.2062	None	None	fig|6666666.59345.peg.4676	fig|6666666.59341.peg.544	fig|6666666.59347.peg.3351	fig|6666666.59346.peg.2039	None	None	fig|6666666.59350.peg.284	None	None	None	None

11224	Succinyl-CoA synthetase, alpha subunit	fig|6666666.59329.peg.5756	fig|6666666.59330.peg.2728	fig|6666666.59331.peg.5256	fig|6666666.59332.peg.4665	None	None	fig|6666666.59336.peg.5602	fig|6666666.59333.peg.4547	fig|6666666.59338.peg.3930	None	None	fig|6666666.59337.peg.4391	fig|6666666.59342.peg.3557	None	None	fig|6666666.59345.peg.3409	None	None	fig|6666666.59346.peg.525	None	fig|6666666.59349.peg.646	fig|6666666.59350.peg.2987	fig|6666666.59351.peg.1156	None	None	fig|6666666.59354.peg.4938

3600	FIG01199775 hypothetical protein	None	fig|6666666.59330.peg.1944	fig|6666666.59331.peg.1177	None	fig|6666666.59334.peg.4376	fig|6666666.59335.peg.1802	fig|6666666.59336.peg.3786	fig|6666666.59333.peg.2522	fig|6666666.59338.peg.3191	fig|6666666.59339.peg.3608	None	fig|6666666.59337.peg.5453	fig|6666666.59342.peg.4196	None	None	fig|6666666.59345.peg.719	fig|6666666.59341.peg.3827	fig|6666666.59347.peg.1686	fig|6666666.59346.peg.3138	fig|6666666.59348.peg.5450	None	None	None	None	None	None

11117	hypothetical protein	fig|6666666.59329.peg.2697	fig|6666666.59330.peg.3148	fig|6666666.59331.peg.114	fig|6666666.59332.peg.4775	None	fig|6666666.59335.peg.2255	fig|6666666.59336.peg.3582	None	fig|6666666.59338.peg.3454	fig|6666666.59339.peg.839	None	fig|6666666.59337.peg.1159	fig|6666666.59342.peg.5845	None	None	fig|6666666.59345.peg.4530	fig|6666666.59341.peg.4124	fig|6666666.59347.peg.478	fig|6666666.59346.peg.4678	fig|6666666.59348.peg.4892	None	None	None	None	None	None

9114	FIG01201025 hypothetical protein	None	fig|6666666.59330.peg.1080	None	fig|6666666.59332.peg.5101	fig|6666666.59334.peg.5382	fig|6666666.59335.peg.1696	fig|6666666.59336.peg.6548	fig|6666666.59333.peg.3839	fig|6666666.59338.peg.717	fig|6666666.59339.peg.7258	fig|6666666.59340.peg.1377	fig|6666666.59337.peg.5439	fig|6666666.59342.peg.3514	None	None	fig|6666666.59345.peg.3560	fig|6666666.59341.peg.1482	fig|6666666.59347.peg.4344	None	fig|6666666.59348.peg.1476	None	None	None	None	None	None

3628	Outer membrane lipoprotein-sorting protein	fig|6666666.59329.peg.5426	fig|6666666.59330.peg.529	fig|6666666.59331.peg.3157	fig|6666666.59332.peg.1461	None	fig|6666666.59335.peg.3610	fig|6666666.59336.peg.1707	fig|6666666.59333.peg.530	None	fig|6666666.59339.peg.591	None	fig|6666666.59337.peg.3960	fig|6666666.59342.peg.1864	None	None	fig|6666666.59345.peg.457	fig|6666666.59341.peg.5051	fig|6666666.59347.peg.2074	fig|6666666.59346.peg.3126	fig|6666666.59348.peg.4419	None	None	None	None	None	None

9457	NAD FAD -utilizing dehydrogenase, sll0175 homolog	fig|6666666.59329.peg.2291	fig|6666666.59330.peg.4988	None	fig|6666666.59332.peg.1244	fig|6666666.59334.peg.1899	fig|6666666.59335.peg.6020	fig|6666666.59336.peg.2225	None	fig|6666666.59338.peg.435	fig|6666666.59339.peg.1949	fig|6666666.59340.peg.1415	fig|6666666.59337.peg.4212	fig|6666666.59342.peg.3886	None	None	fig|6666666.59345.peg.1617	fig|6666666.59341.peg.1306	fig|6666666.59347.peg.1562	fig|6666666.59346.peg.2620	None	None	None	None	None	None	None

8583	Membrane protein SypL involved in exopolysaccharide production	fig|6666666.59329.peg.2532	None	fig|6666666.59331.peg.1300	fig|6666666.59332.peg.3460	None	fig|6666666.59335.peg.2722	fig|6666666.59336.peg.3871	fig|6666666.59333.peg.4127	fig|6666666.59338.peg.1848	fig|6666666.59339.peg.1158	fig|6666666.59340.peg.2042	fig|6666666.59337.peg.4433	fig|6666666.59342.peg.5711	None	None	fig|6666666.59345.peg.3135	fig|6666666.59341.peg.2640	fig|6666666.59347.peg.2336	fig|6666666.59346.peg.5235	None	None	None	None	None	None	None

6120	General secretion pathway protein B	fig|6666666.59329.peg.379	fig|6666666.59330.peg.940	None	fig|6666666.59332.peg.5032	fig|6666666.59334.peg.533	fig|6666666.59335.peg.1508	fig|6666666.59336.peg.3310	fig|6666666.59333.peg.186	fig|6666666.59338.peg.803	fig|6666666.59339.peg.7395	None	fig|6666666.59337.peg.4	None	None	None	fig|6666666.59345.peg.3836	fig|6666666.59341.peg.4836	fig|6666666.59347.peg.3128	fig|6666666.59346.peg.3116	fig|6666666.59348.peg.1826	None	None	None	None	None	None

7084	Acyl carrier protein ACP1	fig|6666666.59329.peg.3339	None	None	fig|6666666.59332.peg.5793	None	None	fig|6666666.59336.peg.1054	fig|6666666.59333.peg.1094	fig|6666666.59338.peg.4670	None	None	None	None	fig|6666666.71181.peg.3938	fig|6666666.59344.peg.5380	None	fig|6666666.59341.peg.2755	fig|6666666.59347.peg.2096	fig|6666666.59346.peg.4721	fig|6666666.59348.peg.780	fig|6666666.59349.peg.2867	None	None	fig|6666666.59353.peg.4795	fig|6666666.59355.peg.4849	fig|6666666.59354.peg.6257

4027	Probable transmembrane protein	fig|6666666.59329.peg.3928	fig|6666666.59330.peg.768	fig|6666666.59331.peg.3414	fig|6666666.59332.peg.343	None	fig|6666666.59335.peg.5234	fig|6666666.59336.peg.2633	fig|6666666.59333.peg.2774	fig|6666666.59338.peg.6167	fig|6666666.59339.peg.2787	None	fig|6666666.59337.peg.5359	fig|6666666.59342.peg.1409	None	None	fig|6666666.59345.peg.45	None	fig|6666666.59347.peg.909	fig|6666666.59346.peg.401	None	None	fig|6666666.59350.peg.1477	None	None	None	None

5485	TraF-related protein	fig|6666666.59329.peg.4859	fig|6666666.59330.peg.802	fig|6666666.59331.peg.565	fig|6666666.59332.peg.4467	None	fig|6666666.59335.peg.5032	None	fig|6666666.59333.peg.3896	fig|6666666.59338.peg.4950	fig|6666666.59339.peg.2939	fig|6666666.59340.peg.825	fig|6666666.59337.peg.3829	None	None	None	fig|6666666.59345.peg.76	fig|6666666.59341.peg.5356	fig|6666666.59347.peg.932	fig|6666666.59346.peg.1894	fig|6666666.59348.peg.5215	None	None	None	None	None	None

1192	Prolidase EC 3.4.13.9	fig|6666666.59329.peg.5502	None	fig|6666666.59331.peg.4555	fig|6666666.59332.peg.1620	fig|6666666.59334.peg.4703	None	fig|6666666.59336.peg.3228	None	fig|6666666.59338.peg.618	None	None	fig|6666666.59337.peg.4048	fig|6666666.59342.peg.5580	None	None	fig|6666666.59345.peg.39	fig|6666666.59341.peg.2831	fig|6666666.59347.peg.4665	fig|6666666.59346.peg.4218	None	fig|6666666.59349.peg.4143	None	fig|6666666.59351.peg.728	None	None	fig|6666666.59354.peg.2574

1340	Antibiotic biosynthesis monooxygenase	None	None	fig|6666666.59331.peg.3388	None	fig|6666666.59334.peg.4887	None	None	None	fig|6666666.59338.peg.6036	fig|6666666.59339.peg.6086	None	None	None	fig|6666666.71181.peg.5338	fig|6666666.59344.peg.3745	None	fig|6666666.59341.peg.5256	fig|6666666.59347.peg.1830	fig|6666666.59346.peg.212	None	fig|6666666.59349.peg.3135	fig|6666666.59350.peg.6848	fig|6666666.59351.peg.4585	fig|6666666.59353.peg.657	fig|6666666.59355.peg.140	fig|6666666.59354.peg.1317

2271	Alpha-galactosidase EC 3.2.1.22	fig|6666666.59329.peg.3870	None	fig|6666666.59331.peg.2004	fig|6666666.59332.peg.713	fig|6666666.59334.peg.5347	None	fig|6666666.59336.peg.1469	None	fig|6666666.59338.peg.743	fig|6666666.59339.peg.5906	None	fig|6666666.59337.peg.2896	fig|6666666.59342.peg.2525	None	None	fig|6666666.59345.peg.847	fig|6666666.59341.peg.3868	fig|6666666.59347.peg.3779	fig|6666666.59346.peg.4763	fig|6666666.59348.peg.4422	None	fig|6666666.59350.peg.276	None	None	None	None

7326	FIG01199869 hypothetical protein	fig|6666666.59329.peg.1179	fig|6666666.59330.peg.4271	None	fig|6666666.59332.peg.4784	None	fig|6666666.59335.peg.1671	fig|6666666.59336.peg.1465	fig|6666666.59333.peg.4409	fig|6666666.59338.peg.515	fig|6666666.59339.peg.3690	None	fig|6666666.59337.peg.3570	fig|6666666.59342.peg.1180	None	None	fig|6666666.59345.peg.845	fig|6666666.59341.peg.1776	fig|6666666.59347.peg.2166	fig|6666666.59346.peg.3248	fig|6666666.59348.peg.5043	None	None	None	None	None	None

7061	FIG01199945 hypothetical protein	fig|6666666.59329.peg.770	fig|6666666.59330.peg.2003	None	fig|6666666.59332.peg.792	fig|6666666.59334.peg.3561	fig|6666666.59335.peg.2548	fig|6666666.59336.peg.1222	fig|6666666.59333.peg.1614	None	fig|6666666.59339.peg.3519	None	fig|6666666.59337.peg.4640	fig|6666666.59342.peg.194	None	None	fig|6666666.59345.peg.4578	fig|6666666.59341.peg.356	fig|6666666.59347.peg.1978	fig|6666666.59346.peg.4117	fig|6666666.59348.peg.3885	None	None	None	None	None	None

5511	FIG01200492 hypothetical protein	fig|6666666.59329.peg.3975	fig|6666666.59330.peg.2718	None	fig|6666666.59332.peg.997	None	fig|6666666.59335.peg.4529	fig|6666666.59336.peg.4895	fig|6666666.59333.peg.4873	fig|6666666.59338.peg.5477	fig|6666666.59339.peg.2489	None	fig|6666666.59337.peg.2449	fig|6666666.59342.peg.3870	None	None	fig|6666666.59345.peg.602	fig|6666666.59341.peg.5511	fig|6666666.59347.peg.638	fig|6666666.59346.peg.3025	fig|6666666.59348.peg.375	None	None	None	None	None	None

9024	Xanthine dehydrogenase, iron-sulfur cluster and FAD-binding subunit A 1.17.1.4	None	None	fig|6666666.59331.peg.1321	fig|6666666.59332.peg.4039	None	fig|6666666.59335.peg.1156	None	None	fig|6666666.59338.peg.5492	fig|6666666.59339.peg.1673	fig|6666666.59340.peg.3649	None	None	fig|6666666.71181.peg.4887	fig|6666666.59344.peg.6149	None	fig|6666666.59341.peg.3079	fig|6666666.59347.peg.3319	fig|6666666.59346.peg.4881	fig|6666666.59348.peg.496	None	fig|6666666.59350.peg.4222	None	fig|6666666.59353.peg.4151	fig|6666666.59355.peg.4264	None

3794	Nitrate ABC transporter, nitrate-binding protein	fig|6666666.59329.peg.3989	None	None	None	None	fig|6666666.59335.peg.2890	fig|6666666.59336.peg.5789	fig|6666666.59333.peg.724	fig|6666666.59338.peg.872	fig|6666666.59339.peg.4680	None	fig|6666666.59337.peg.5459	None	fig|6666666.71181.peg.5176	fig|6666666.59344.peg.6035	fig|6666666.59345.peg.285	None	fig|6666666.59347.peg.5146	None	fig|6666666.59348.peg.489	None	fig|6666666.59350.peg.5253	None	fig|6666666.59353.peg.4654	fig|6666666.59355.peg.3870	None

3792	RRNA methylase, putative	fig|6666666.59329.peg.1394	fig|6666666.59330.peg.1466	fig|6666666.59331.peg.5643	fig|6666666.59332.peg.4989	fig|6666666.59334.peg.3521	None	fig|6666666.59336.peg.301	None	fig|6666666.59338.peg.4353	None	fig|6666666.59340.peg.445	fig|6666666.59337.peg.210	None	None	None	None	fig|6666666.59341.peg.2971	fig|6666666.59347.peg.1411	fig|6666666.59346.peg.1610	None	fig|6666666.59349.peg.4558	None	fig|6666666.59351.peg.1539	None	None	fig|6666666.59354.peg.5794

1950	probable peptidase VC1983	fig|6666666.59329.peg.3615	fig|6666666.59330.peg.4232	fig|6666666.59331.peg.2908	fig|6666666.59332.peg.1868	fig|6666666.59334.peg.4114	fig|6666666.59335.peg.1831	fig|6666666.59336.peg.1774	fig|6666666.59333.peg.2939	None	None	None	fig|6666666.59337.peg.1251	None	fig|6666666.71181.peg.2121	fig|6666666.59344.peg.5323	fig|6666666.59345.peg.4436	None	None	fig|6666666.59346.peg.682	None	None	None	None	fig|6666666.59353.peg.711	fig|6666666.59355.peg.198	None

8668	FIG01200682 hypothetical protein	None	fig|6666666.59330.peg.936	fig|6666666.59331.peg.1317	fig|6666666.59332.peg.2305	fig|6666666.59334.peg.5001	fig|6666666.59335.peg.4224	fig|6666666.59336.peg.4182	fig|6666666.59333.peg.4457	fig|6666666.59338.peg.3820	fig|6666666.59339.peg.6516	None	None	fig|6666666.59342.peg.1857	None	None	fig|6666666.59345.peg.464	fig|6666666.59341.peg.3439	fig|6666666.59347.peg.1344	fig|6666666.59346.peg.1940	fig|6666666.59348.peg.519	None	None	None	None	None	None

4085	FIG01200078 hypothetical protein	fig|6666666.59329.peg.735	fig|6666666.59330.peg.578	None	fig|6666666.59332.peg.3503	None	fig|6666666.59335.peg.4756	fig|6666666.59336.peg.168	fig|6666666.59333.peg.390	fig|6666666.59338.peg.5800	fig|6666666.59339.peg.8293	fig|6666666.59340.peg.585	fig|6666666.59337.peg.365	None	None	None	fig|6666666.59345.peg.2512	fig|6666666.59341.peg.778	fig|6666666.59347.peg.4847	fig|6666666.59346.peg.873	fig|6666666.59348.peg.430	None	None	None	None	None	None

4088	NifU-related domain containing protein	fig|6666666.59329.peg.5709	fig|6666666.59330.peg.2127	fig|6666666.59331.peg.3071	fig|6666666.59332.peg.5133	fig|6666666.59334.peg.3949	fig|6666666.59335.peg.991	fig|6666666.59336.peg.108	None	fig|6666666.59338.peg.3728	fig|6666666.59339.peg.4456	None	fig|6666666.59337.peg.2345	fig|6666666.59342.peg.2448	None	None	fig|6666666.59345.peg.2268	fig|6666666.59341.peg.1922	fig|6666666.59347.peg.654	fig|6666666.59346.peg.1099	None	None	None	None	None	None	None

8082	FIG01201193 hypothetical protein	fig|6666666.59329.peg.3452	fig|6666666.59330.peg.4117	fig|6666666.59331.peg.3905	fig|6666666.59332.peg.6168	None	fig|6666666.59335.peg.3293	fig|6666666.59336.peg.3031	fig|6666666.59333.peg.2537	fig|6666666.59338.peg.5170	fig|6666666.59339.peg.3058	fig|6666666.59340.peg.194	fig|6666666.59337.peg.3734	None	None	None	fig|6666666.59345.peg.1197	fig|6666666.59341.peg.2455	fig|6666666.59347.peg.2449	None	fig|6666666.59348.peg.456	None	None	None	None	None	None

2943	Mll3428 protein	fig|6666666.59329.peg.6367	fig|6666666.59330.peg.2042	fig|6666666.59331.peg.2046	fig|6666666.59332.peg.1528	None	fig|6666666.59335.peg.2259	fig|6666666.59336.peg.4489	None	fig|6666666.59338.peg.5104	fig|6666666.59339.peg.833	None	fig|6666666.59337.peg.4311	None	None	None	fig|6666666.59345.peg.3903	fig|6666666.59341.peg.4897	fig|6666666.59347.peg.1149	fig|6666666.59346.peg.3659	fig|6666666.59348.peg.3172	None	fig|6666666.59350.peg.4465	None	None	None	None

5795	FIG01199534 hypothetical protein	fig|6666666.59329.peg.3256	None	None	fig|6666666.59332.peg.4470	None	fig|6666666.59335.peg.1121	fig|6666666.59336.peg.122	fig|6666666.59333.peg.4491	fig|6666666.59338.peg.5820	fig|6666666.59339.peg.6955	fig|6666666.59340.peg.3063	fig|6666666.59337.peg.2357	fig|6666666.59342.peg.3940	None	None	fig|6666666.59345.peg.2259	fig|6666666.59341.peg.527	fig|6666666.59347.peg.2821	fig|6666666.59346.peg.1842	fig|6666666.59348.peg.302	None	None	None	None	None	None

6664	Predicted membrane-associated metal-dependent hydrolase	None	fig|6666666.59330.peg.877	fig|6666666.59331.peg.900	None	fig|6666666.59334.peg.3761	fig|6666666.59335.peg.2067	fig|6666666.59336.peg.2072	fig|6666666.59333.peg.3295	None	fig|6666666.59339.peg.5295	None	fig|6666666.59337.peg.5409	None	fig|6666666.71181.peg.2539	fig|6666666.59344.peg.3508	fig|6666666.59345.peg.560	None	None	fig|6666666.59346.peg.3059	fig|6666666.59348.peg.4253	None	None	None	fig|6666666.59353.peg.1376	fig|6666666.59355.peg.2497	None

8255	Isochorismatase EC 3.3.2.1	fig|6666666.59329.peg.4173	fig|6666666.59330.peg.893	fig|6666666.59331.peg.4232	fig|6666666.59332.peg.6103	None	fig|6666666.59335.peg.767	fig|6666666.59336.peg.1856	None	fig|6666666.59338.peg.4327	fig|6666666.59339.peg.5583	None	fig|6666666.59337.peg.2477	None	None	None	fig|6666666.59345.peg.576	fig|6666666.59341.peg.2819	fig|6666666.59347.peg.1837	fig|6666666.59346.peg.196	fig|6666666.59348.peg.4648	None	None	fig|6666666.59351.peg.4415	None	None	None

7422	DJ-1 YajL PfpI superfamily, includes chaperone protein YajL former ThiJ , parkinsonism-associated protein DJ-1, peptidases PfpI, Hsp31	None	fig|6666666.59330.peg.2799	None	None	fig|6666666.59334.peg.4949	fig|6666666.59335.peg.107	None	fig|6666666.59333.peg.1925	fig|6666666.59338.peg.3152	fig|6666666.59339.peg.5031	None	fig|6666666.59337.peg.2367	None	fig|6666666.71181.peg.2036	fig|6666666.59344.peg.1086	fig|6666666.59345.peg.2251	fig|6666666.59341.peg.535	fig|6666666.59347.peg.2814	None	fig|6666666.59348.peg.4847	None	None	None	fig|6666666.59353.peg.768	fig|6666666.59355.peg.936	None

2139	FIG01200071 hypothetical protein	None	fig|6666666.59330.peg.1265	None	fig|6666666.59332.peg.2101	None	fig|6666666.59335.peg.2818	fig|6666666.59336.peg.465	fig|6666666.59333.peg.4894	fig|6666666.59338.peg.2139	fig|6666666.59339.peg.3493	fig|6666666.59340.peg.3706	fig|6666666.59337.peg.2209	fig|6666666.59342.peg.1090	None	None	fig|6666666.59345.peg.1580	fig|6666666.59341.peg.3535	fig|6666666.59347.peg.4174	fig|6666666.59346.peg.3240	fig|6666666.59348.peg.2045	None	None	None	None	None	None

6596	hypothetical protein	fig|6666666.59329.peg.5683	fig|6666666.59330.peg.1916	fig|6666666.59331.peg.2715	fig|6666666.59332.peg.6181	None	fig|6666666.59335.peg.4874	fig|6666666.59336.peg.6266	fig|6666666.59333.peg.2403	fig|6666666.59338.peg.4921	fig|6666666.59339.peg.4604	None	fig|6666666.59337.peg.4852	fig|6666666.59342.peg.5985	None	None	fig|6666666.59345.peg.1355	None	fig|6666666.59347.peg.3761	fig|6666666.59346.peg.2322	fig|6666666.59348.peg.3511	None	None	None	None	None	None

3096	FIG01199969 hypothetical protein	fig|6666666.59329.peg.5848	fig|6666666.59330.peg.354	fig|6666666.59331.peg.1203	fig|6666666.59332.peg.1317	None	fig|6666666.59335.peg.3570	fig|6666666.59336.peg.3271	None	fig|6666666.59338.peg.2602	fig|6666666.59339.peg.8360	None	fig|6666666.59337.peg.286	fig|6666666.59342.peg.4125	None	None	fig|6666666.59345.peg.2129	fig|6666666.59341.peg.6028	fig|6666666.59347.peg.4733	fig|6666666.59346.peg.3069	fig|6666666.59348.peg.191	None	None	None	None	None	None

4101	SM-20-related protein	None	fig|6666666.59330.peg.1979	fig|6666666.59331.peg.396	fig|6666666.59332.peg.4114	None	None	None	fig|6666666.59333.peg.957	fig|6666666.59338.peg.4853	None	None	fig|6666666.59337.peg.2606	fig|6666666.59342.peg.2930	None	None	fig|6666666.59345.peg.751	fig|6666666.59341.peg.4181	fig|6666666.59347.peg.3650	fig|6666666.59346.peg.5104	fig|6666666.59348.peg.4995	fig|6666666.59349.peg.2962	None	fig|6666666.59351.peg.1629	None	None	fig|6666666.59354.peg.3095

8577	Nitrate ABC transporter, ATP-binding protein	fig|6666666.59329.peg.3987	None	None	None	None	fig|6666666.59335.peg.2894	None	fig|6666666.59333.peg.726	fig|6666666.59338.peg.874	fig|6666666.59339.peg.4685	None	fig|6666666.59337.peg.5462	fig|6666666.59342.peg.3403	fig|6666666.71181.peg.5178	fig|6666666.59344.peg.6037	fig|6666666.59345.peg.283	None	fig|6666666.59347.peg.5144	None	fig|6666666.59348.peg.484	None	fig|6666666.59350.peg.4766	None	fig|6666666.59353.peg.4652	fig|6666666.59355.peg.3872	None

7400	ABC-type transport system, ATPase component	fig|6666666.59329.peg.4835	fig|6666666.59330.peg.3252	None	fig|6666666.59332.peg.1165	None	fig|6666666.59335.peg.4201	None	fig|6666666.59333.peg.1824	fig|6666666.59338.peg.3983	fig|6666666.59339.peg.725	None	fig|6666666.59337.peg.2162	None	None	None	fig|6666666.59345.peg.3978	fig|6666666.59341.peg.1225	fig|6666666.59347.peg.3631	fig|6666666.59346.peg.1163	fig|6666666.59348.peg.3553	fig|6666666.59349.peg.4478	None	None	None	None	fig|6666666.59354.peg.319

267	FIG01200768 hypothetical protein	fig|6666666.59329.peg.3967	fig|6666666.59330.peg.2713	None	fig|6666666.59332.peg.991	fig|6666666.59334.peg.2857	fig|6666666.59335.peg.1627	None	fig|6666666.59333.peg.3910	fig|6666666.59338.peg.5472	fig|6666666.59339.peg.6542	None	fig|6666666.59337.peg.2442	fig|6666666.59342.peg.2956	None	None	fig|6666666.59345.peg.607	fig|6666666.59341.peg.5419	fig|6666666.59347.peg.546	fig|6666666.59346.peg.3020	fig|6666666.59348.peg.1503	None	None	None	None	None	None

2922	Uncharacterized lipoprotein YmcC precursor	fig|6666666.59329.peg.826	fig|6666666.59330.peg.1351	fig|6666666.59331.peg.3174	fig|6666666.59332.peg.3270	fig|6666666.59334.peg.3587	None	fig|6666666.59336.peg.81	fig|6666666.59333.peg.477	fig|6666666.59338.peg.1224	fig|6666666.59339.peg.6407	None	fig|6666666.59337.peg.440	fig|6666666.59342.peg.1938	None	None	fig|6666666.59345.peg.2589	None	fig|6666666.59347.peg.5172	fig|6666666.59346.peg.4835	fig|6666666.59348.peg.1537	None	None	None	None	None	None

1164	Mobile element protein	None	fig|6666666.59330.peg.4765	fig|6666666.59331.peg.1058	fig|6666666.59332.peg.4552	None	None	None	None	fig|6666666.59338.peg.6181	None	fig|6666666.59340.peg.2789	None	fig|6666666.59342.peg.5930	fig|6666666.71181.peg.2359	fig|6666666.59344.peg.3365	None	fig|6666666.59341.peg.4769	None	fig|6666666.59346.peg.5303	None	fig|6666666.59349.peg.270	fig|6666666.59350.peg.7309	None	fig|6666666.59353.peg.3741	fig|6666666.59355.peg.3758	fig|6666666.59354.peg.848

4730	Putative ATP-dependent Lon protease	fig|6666666.59329.peg.1635	fig|6666666.59330.peg.433	None	fig|6666666.59332.peg.2466	None	fig|6666666.59335.peg.701	fig|6666666.59336.peg.4210	fig|6666666.59333.peg.4870	fig|6666666.59338.peg.5911	fig|6666666.59339.peg.6230	fig|6666666.59340.peg.2073	fig|6666666.59337.peg.4262	None	None	None	fig|6666666.59345.peg.2661	fig|6666666.59341.peg.1417	fig|6666666.59347.peg.4479	fig|6666666.59346.peg.2237	fig|6666666.59348.peg.1409	None	None	None	None	None	None

6800	MSHA biogenesis protein MshJ	fig|6666666.59329.peg.5895	fig|6666666.59330.peg.8	fig|6666666.59331.peg.5766	fig|6666666.59332.peg.504	fig|6666666.59334.peg.733	fig|6666666.59335.peg.201	fig|6666666.59336.peg.1173	fig|6666666.59333.peg.579	None	fig|6666666.59339.peg.7761	None	fig|6666666.59337.peg.794	fig|6666666.59342.peg.5471	None	None	fig|6666666.59345.peg.4902	fig|6666666.59341.peg.3148	None	fig|6666666.59346.peg.1947	fig|6666666.59348.peg.5412	None	None	None	None	None	None

5994	Transcription elongation factor	fig|6666666.59329.peg.1219	fig|6666666.59330.peg.4056	None	fig|6666666.59332.peg.1666	None	fig|6666666.59335.peg.132	fig|6666666.59336.peg.2776	fig|6666666.59333.peg.1810	fig|6666666.59338.peg.273	fig|6666666.59339.peg.5062	None	fig|6666666.59337.peg.1554	None	None	None	fig|6666666.59345.peg.2226	fig|6666666.59341.peg.2300	fig|6666666.59347.peg.2792	None	None	fig|6666666.59349.peg.133	None	fig|6666666.59351.peg.842	None	None	fig|6666666.59354.peg.2594

6765	Pectin degradation protein KdgF	None	None	fig|6666666.59331.peg.5332	None	fig|6666666.59334.peg.3114	None	fig|6666666.59336.peg.2194	None	fig|6666666.59338.peg.4601	None	fig|6666666.59340.peg.2522	None	fig|6666666.59342.peg.1487	fig|6666666.71181.peg.564	fig|6666666.59344.peg.1899	fig|6666666.59345.peg.1350	fig|6666666.59341.peg.4549	fig|6666666.59347.peg.321	fig|6666666.59346.peg.2856	None	None	fig|6666666.59350.peg.5710	None	fig|6666666.59353.peg.1195	fig|6666666.59355.peg.281	None

6760	Type III effector HopPmaJ	fig|6666666.59329.peg.6524	fig|6666666.59330.peg.907	fig|6666666.59331.peg.1752	fig|6666666.59332.peg.5746	None	fig|6666666.59335.peg.5199	fig|6666666.59336.peg.4665	fig|6666666.59333.peg.4679	fig|6666666.59338.peg.6130	None	None	fig|6666666.59337.peg.5584	fig|6666666.59342.peg.3819	None	None	fig|6666666.59345.peg.423	fig|6666666.59341.peg.5577	fig|6666666.59347.peg.2113	fig|6666666.59346.peg.4101	fig|6666666.59348.peg.3491	None	None	None	None	None	None

3203	FIG01201134 hypothetical protein	fig|6666666.59329.peg.55	fig|6666666.59330.peg.634	None	fig|6666666.59332.peg.218	None	fig|6666666.59335.peg.2441	fig|6666666.59336.peg.6424	fig|6666666.59333.peg.2	fig|6666666.59338.peg.577	fig|6666666.59339.peg.8082	None	fig|6666666.59337.peg.3893	fig|6666666.59342.peg.4032	None	None	fig|6666666.59345.peg.4935	fig|6666666.59341.peg.1945	fig|6666666.59347.peg.4754	fig|6666666.59346.peg.1814	fig|6666666.59348.peg.57	None	None	None	None	None	None

3202	FIG01199765 hypothetical protein	fig|6666666.59329.peg.4027	None	fig|6666666.59331.peg.2898	fig|6666666.59332.peg.1538	fig|6666666.59334.peg.4356	None	fig|6666666.59336.peg.6427	fig|6666666.59333.peg.4516	None	None	fig|6666666.59340.peg.1001	fig|6666666.59337.peg.1211	fig|6666666.59342.peg.5232	None	None	fig|6666666.59345.peg.4397	fig|6666666.59341.peg.3180	None	None	fig|6666666.59348.peg.3311	fig|6666666.59349.peg.1822	None	fig|6666666.59351.peg.5256	None	None	fig|6666666.59354.peg.3974

5819	YjbG polysaccharide synthesis-related protein	fig|6666666.59329.peg.827	fig|6666666.59330.peg.1352	fig|6666666.59331.peg.3175	fig|6666666.59332.peg.2133	fig|6666666.59334.peg.3588	None	fig|6666666.59336.peg.80	fig|6666666.59333.peg.476	fig|6666666.59338.peg.1225	fig|6666666.59339.peg.6406	None	fig|6666666.59337.peg.441	fig|6666666.59342.peg.1937	None	None	fig|6666666.59345.peg.2590	None	fig|6666666.59347.peg.5171	fig|6666666.59346.peg.4836	fig|6666666.59348.peg.1536	None	None	None	None	None	None

6249	4-hydroxy-2-oxovalerate aldolase	None	None	None	fig|6666666.59332.peg.5364	fig|6666666.59334.peg.4660	fig|6666666.59335.peg.4688	fig|6666666.59336.peg.508	fig|6666666.59333.peg.2181	None	fig|6666666.59339.peg.774	None	None	fig|6666666.59342.peg.2512	fig|6666666.71181.peg.1945	fig|6666666.59344.peg.1417	fig|6666666.59345.peg.769	fig|6666666.59341.peg.5496	fig|6666666.59347.peg.1325	None	None	None	fig|6666666.59350.peg.7342	None	fig|6666666.59353.peg.173	fig|6666666.59355.peg.785	None

8871	FIG01200547 hypothetical protein	fig|6666666.59329.peg.6052	fig|6666666.59330.peg.2949	fig|6666666.59331.peg.3366	None	fig|6666666.59334.peg.5465	None	fig|6666666.59336.peg.6608	fig|6666666.59333.peg.5270	fig|6666666.59338.peg.4370	fig|6666666.59339.peg.2081	None	fig|6666666.59337.peg.3456	fig|6666666.59342.peg.3878	None	None	fig|6666666.59345.peg.183	fig|6666666.59341.peg.2959	fig|6666666.59347.peg.1390	fig|6666666.59346.peg.4975	fig|6666666.59348.peg.4849	None	None	None	None	None	None

10201	D-mannonate oxidoreductase EC 1.1.1.57	fig|6666666.59329.peg.5759	None	None	fig|6666666.59332.peg.5582	fig|6666666.59334.peg.3206	None	fig|6666666.59336.peg.3453	None	None	None	fig|6666666.59340.peg.618	fig|6666666.59337.peg.306	fig|6666666.59342.peg.2049	fig|6666666.71181.peg.5053	fig|6666666.59344.peg.5106	fig|6666666.59345.peg.2109	fig|6666666.59341.peg.4270	fig|6666666.59347.peg.3831	None	None	None	fig|6666666.59350.peg.5018	None	fig|6666666.59353.peg.1099	fig|6666666.59355.peg.1726	None

9716	FIG01203920 hypothetical protein	fig|6666666.59329.peg.5020	fig|6666666.59330.peg.694	fig|6666666.59331.peg.2468	fig|6666666.59332.peg.4887	None	fig|6666666.59335.peg.2473	fig|6666666.59336.peg.2988	None	fig|6666666.59338.peg.2405	fig|6666666.59339.peg.3583	None	fig|6666666.59337.peg.4157	fig|6666666.59342.peg.3424	None	None	fig|6666666.59345.peg.4775	fig|6666666.59341.peg.3773	fig|6666666.59347.peg.2209	fig|6666666.59346.peg.4406	fig|6666666.59348.peg.2853	None	None	None	None	None	None

205	FIG01199939 hypothetical protein	fig|6666666.59329.peg.4188	fig|6666666.59330.peg.2978	fig|6666666.59331.peg.2703	fig|6666666.59332.peg.6141	None	fig|6666666.59335.peg.5528	fig|6666666.59336.peg.813	fig|6666666.59333.peg.2198	fig|6666666.59338.peg.4211	fig|6666666.59339.peg.2828	None	fig|6666666.59337.peg.5155	fig|6666666.59342.peg.665	None	None	fig|6666666.59345.peg.3294	fig|6666666.59341.peg.5622	fig|6666666.59347.peg.893	fig|6666666.59346.peg.3167	None	None	None	None	None	None	None

9727	FIG01199987 hypothetical protein	None	fig|6666666.59330.peg.2083	None	fig|6666666.59332.peg.5813	fig|6666666.59334.peg.2325	fig|6666666.59335.peg.5971	fig|6666666.59336.peg.4804	fig|6666666.59333.peg.3218	fig|6666666.59338.peg.4260	fig|6666666.59339.peg.3717	None	fig|6666666.59337.peg.4681	fig|6666666.59342.peg.3826	None	None	fig|6666666.59345.peg.823	fig|6666666.59341.peg.5714	fig|6666666.59347.peg.2143	fig|6666666.59346.peg.3847	fig|6666666.59348.peg.4804	None	None	None	None	None	None

3589	FIG01201249 hypothetical protein	fig|6666666.59329.peg.3200	fig|6666666.59330.peg.4783	None	fig|6666666.59332.peg.3517	None	fig|6666666.59335.peg.2348	fig|6666666.59336.peg.5109	fig|6666666.59333.peg.3745	fig|6666666.59338.peg.4409	fig|6666666.59339.peg.2873	None	fig|6666666.59337.peg.2513	fig|6666666.59342.peg.893	None	None	fig|6666666.59345.peg.131	fig|6666666.59341.peg.4253	fig|6666666.59347.peg.866	fig|6666666.59346.peg.2906	fig|6666666.59348.peg.4202	None	None	None	None	None	None

4426	lipoprotein Blc	None	fig|6666666.59330.peg.3150	None	fig|6666666.59332.peg.3011	fig|6666666.59334.peg.58	fig|6666666.59335.peg.4021	fig|6666666.59336.peg.3580	fig|6666666.59333.peg.4161	fig|6666666.59338.peg.4507	None	None	fig|6666666.59337.peg.1161	None	None	None	fig|6666666.59345.peg.3469	fig|6666666.59341.peg.737	fig|6666666.59347.peg.3431	fig|6666666.59346.peg.1030	None	fig|6666666.59349.peg.3103	None	fig|6666666.59351.peg.3258	None	None	fig|6666666.59354.peg.5777

1699	PTS system, cellobiose-specific IIB component EC 2.7.1.69	fig|6666666.59329.peg.6326	fig|6666666.59330.peg.2438	None	None	fig|6666666.59334.peg.554	None	fig|6666666.59336.peg.496	fig|6666666.59333.peg.3566	None	None	fig|6666666.59340.peg.2417	fig|6666666.59337.peg.4181	fig|6666666.59342.peg.2111	fig|6666666.71181.peg.2161	fig|6666666.59344.peg.3233	fig|6666666.59345.peg.4827	fig|6666666.59341.peg.1597	fig|6666666.59347.peg.1450	None	None	None	None	None	fig|6666666.59353.peg.2849	fig|6666666.59355.peg.3952	None

4597	N-carbamoyl-L-amino acid hydrolase EC 3.5.1.87	fig|6666666.59329.peg.4164	fig|6666666.59330.peg.4810	fig|6666666.59331.peg.3319	None	fig|6666666.59334.peg.3777	None	None	None	None	None	None	fig|6666666.59337.peg.2827	fig|6666666.59342.peg.1243	fig|6666666.71181.peg.4618	fig|6666666.59344.peg.4783	fig|6666666.59345.peg.1400	fig|6666666.59341.peg.4697	None	None	None	fig|6666666.59349.peg.1661	fig|6666666.59350.peg.2050	None	fig|6666666.59353.peg.4529	fig|6666666.59355.peg.4667	fig|6666666.59354.peg.2062

10011	FIG01199722 hypothetical protein	fig|6666666.59329.peg.5155	fig|6666666.59330.peg.2588	fig|6666666.59331.peg.2358	None	fig|6666666.59334.peg.4603	fig|6666666.59335.peg.5745	fig|6666666.59336.peg.4845	fig|6666666.59333.peg.4681	None	fig|6666666.59339.peg.1278	None	fig|6666666.59337.peg.2732	fig|6666666.59342.peg.5654	None	None	fig|6666666.59345.peg.3208	fig|6666666.59341.peg.2901	fig|6666666.59347.peg.1716	fig|6666666.59346.peg.3281	fig|6666666.59348.peg.3914	None	None	None	None	None	None

1311	FIG01201362 hypothetical protein	fig|6666666.59329.peg.3581	fig|6666666.59330.peg.3419	None	fig|6666666.59332.peg.4695	fig|6666666.59334.peg.3214	fig|6666666.59335.peg.4516	fig|6666666.59336.peg.4616	fig|6666666.59333.peg.5436	fig|6666666.59338.peg.2044	fig|6666666.59339.peg.8455	None	fig|6666666.59337.peg.2502	fig|6666666.59342.peg.235	None	None	fig|6666666.59345.peg.142	fig|6666666.59341.peg.5297	fig|6666666.59347.peg.820	fig|6666666.59346.peg.4548	None	None	None	None	None	None	None

1319	PTS system, cellobiose-specific IIA component EC 2.7.1.69	fig|6666666.59329.peg.3178	fig|6666666.59330.peg.2436	None	None	fig|6666666.59334.peg.1496	None	fig|6666666.59336.peg.499	fig|6666666.59333.peg.3568	fig|6666666.59338.peg.898	None	None	fig|6666666.59337.peg.4179	fig|6666666.59342.peg.2113	fig|6666666.71181.peg.3643	fig|6666666.59344.peg.2364	fig|6666666.59345.peg.4825	fig|6666666.59341.peg.4347	fig|6666666.59347.peg.1452	None	None	None	None	None	fig|6666666.59353.peg.2846	fig|6666666.59355.peg.1296	None

1245	Histone acetyltransferase HPA2 and related acetyltransferases	None	fig|6666666.59330.peg.4395	fig|6666666.59331.peg.466	None	None	None	None	None	fig|6666666.59338.peg.4420	None	fig|6666666.59340.peg.2476	fig|6666666.59337.peg.1828	fig|6666666.59342.peg.987	fig|6666666.71181.peg.1457	fig|6666666.59344.peg.1161	fig|6666666.59345.peg.4228	fig|6666666.59341.peg.5698	fig|6666666.59347.peg.1858	None	fig|6666666.59348.peg.4976	None	None	fig|6666666.59351.peg.4555	fig|6666666.59353.peg.4373	fig|6666666.59355.peg.4469	None

9831	FIG01200951 hypothetical protein	fig|6666666.59329.peg.1863	fig|6666666.59330.peg.2646	fig|6666666.59331.peg.5759	fig|6666666.59332.peg.4341	fig|6666666.59334.peg.3008	None	fig|6666666.59336.peg.603	None	fig|6666666.59338.peg.3668	fig|6666666.59339.peg.7703	fig|6666666.59340.peg.1730	fig|6666666.59337.peg.1712	None	None	None	fig|6666666.59345.peg.5369	fig|6666666.59341.peg.1881	fig|6666666.59347.peg.529	fig|6666666.59346.peg.2392	fig|6666666.59348.peg.3104	None	None	None	None	None	None

1330	FIG01199829 hypothetical protein	fig|6666666.59329.peg.2752	fig|6666666.59330.peg.4064	None	fig|6666666.59332.peg.4913	fig|6666666.59334.peg.3121	fig|6666666.59335.peg.1303	fig|6666666.59336.peg.5596	fig|6666666.59333.peg.2342	fig|6666666.59338.peg.1575	fig|6666666.59339.peg.3436	None	fig|6666666.59337.peg.2173	fig|6666666.59342.peg.486	None	None	fig|6666666.59345.peg.1610	fig|6666666.59341.peg.275	fig|6666666.59347.peg.4064	fig|6666666.59346.peg.2183	None	None	None	None	None	None	None

8347	FIG01199871 hypothetical protein	fig|6666666.59329.peg.2491	fig|6666666.59330.peg.1602	fig|6666666.59331.peg.2399	fig|6666666.59332.peg.5322	None	fig|6666666.59335.peg.5156	fig|6666666.59336.peg.2571	fig|6666666.59333.peg.3758	fig|6666666.59338.peg.3838	fig|6666666.59339.peg.2380	None	fig|6666666.59337.peg.4895	None	None	None	fig|6666666.59345.peg.4492	fig|6666666.59341.peg.3938	fig|6666666.59347.peg.4166	fig|6666666.59346.peg.3372	fig|6666666.59348.peg.4040	None	None	None	None	None	None

3635	putative methyltransferase	fig|6666666.59329.peg.6070	fig|6666666.59330.peg.3911	fig|6666666.59331.peg.5170	None	None	None	fig|6666666.59336.peg.5381	None	fig|6666666.59338.peg.704	None	None	fig|6666666.59337.peg.4710	fig|6666666.59342.peg.4276	fig|6666666.71181.peg.5133	fig|6666666.59344.peg.4706	fig|6666666.59345.peg.3125	fig|6666666.59341.peg.4404	None	None	None	fig|6666666.59349.peg.4512	fig|6666666.59350.peg.1369	fig|6666666.59351.peg.1752	None	None	fig|6666666.59354.peg.5740

9667	Ribose ABC transport system, periplasmic ribose-binding protein RbsB TC 3.A.1.2.1	None	fig|6666666.59330.peg.711	fig|6666666.59331.peg.3106	None	None	fig|6666666.59335.peg.1206	None	None	fig|6666666.59338.peg.5597	fig|6666666.59339.peg.4376	None	fig|6666666.59337.peg.4620	None	fig|6666666.71181.peg.5225	fig|6666666.59344.peg.4966	None	None	fig|6666666.59347.peg.3620	None	None	fig|6666666.59349.peg.3617	fig|6666666.59350.peg.435	fig|6666666.59351.peg.685	fig|6666666.59353.peg.4636	fig|6666666.59355.peg.4622	fig|6666666.59354.peg.6339

4876	NADH dehydrogenase subunit II-related protein	None	fig|6666666.59330.peg.1091	fig|6666666.59331.peg.6248	fig|6666666.59332.peg.73	None	fig|6666666.59335.peg.3920	fig|6666666.59336.peg.5630	fig|6666666.59333.peg.3965	fig|6666666.59338.peg.5332	fig|6666666.59339.peg.8052	fig|6666666.59340.peg.976	fig|6666666.59337.peg.864	None	None	None	fig|6666666.59345.peg.4989	fig|6666666.59341.peg.5110	fig|6666666.59347.peg.4807	fig|6666666.59346.peg.4289	fig|6666666.59348.peg.1211	None	None	None	None	None	None

8105	FIG01203125 hypothetical protein	fig|6666666.59329.peg.1239	fig|6666666.59330.peg.3218	fig|6666666.59331.peg.608	None	fig|6666666.59334.peg.997	fig|6666666.59335.peg.2867	fig|6666666.59336.peg.3601	fig|6666666.59333.peg.5069	fig|6666666.59338.peg.3610	None	fig|6666666.59340.peg.2272	fig|6666666.59337.peg.5082	fig|6666666.59342.peg.6202	None	None	fig|6666666.59345.peg.5617	fig|6666666.59341.peg.3236	fig|6666666.59347.peg.1549	fig|6666666.59346.peg.4308	None	None	None	None	None	None	None

4016	FIG01202125 hypothetical protein	fig|6666666.59329.peg.2661	fig|6666666.59330.peg.318	fig|6666666.59331.peg.2251	fig|6666666.59332.peg.3427	None	fig|6666666.59335.peg.865	fig|6666666.59336.peg.4656	fig|6666666.59333.peg.1434	fig|6666666.59338.peg.2846	fig|6666666.59339.peg.3243	fig|6666666.59340.peg.544	fig|6666666.59337.peg.1046	None	None	None	fig|6666666.59345.peg.4236	None	fig|6666666.59347.peg.2641	fig|6666666.59346.peg.2071	fig|6666666.59348.peg.907	None	None	None	None	None	None

8951	Na + H + antiporter subunit E	None	None	fig|6666666.59331.peg.5008	None	fig|6666666.59334.peg.5147	fig|6666666.59335.peg.4440	fig|6666666.59336.peg.5544	fig|6666666.59333.peg.4706	fig|6666666.59338.peg.4613	None	fig|6666666.59340.peg.3193	None	fig|6666666.59342.peg.4551	None	None	fig|6666666.59345.peg.247	fig|6666666.59341.peg.2680	fig|6666666.59347.peg.2133	None	None	fig|6666666.59349.peg.3895	fig|6666666.59350.peg.85	fig|6666666.59351.peg.1379	None	None	fig|6666666.59354.peg.2460

2818	FIG028274 hypothetical protein	fig|6666666.59329.peg.4654	fig|6666666.59330.peg.3636	fig|6666666.59331.peg.4739	fig|6666666.59332.peg.4170	None	fig|6666666.59335.peg.1193	fig|6666666.59336.peg.1612	fig|6666666.59333.peg.4792	fig|6666666.59338.peg.788	fig|6666666.59339.peg.34	None	fig|6666666.59337.peg.4495	fig|6666666.59342.peg.2940	None	None	fig|6666666.59345.peg.2158	None	fig|6666666.59347.peg.2853	fig|6666666.59346.peg.2502	fig|6666666.59348.peg.3892	None	None	None	None	None	None

4245	Putative transcriptional regulator	fig|6666666.59329.peg.507	fig|6666666.59330.peg.217	fig|6666666.59331.peg.175	fig|6666666.59332.peg.6207	fig|6666666.59334.peg.4250	fig|6666666.59335.peg.4391	None	fig|6666666.59333.peg.3165	None	fig|6666666.59339.peg.6718	None	fig|6666666.59337.peg.1070	fig|6666666.59342.peg.8	None	None	fig|6666666.59345.peg.1648	fig|6666666.59341.peg.1487	None	None	fig|6666666.59348.peg.625	fig|6666666.59349.peg.3048	None	None	None	None	fig|6666666.59354.peg.1773

4243	Uncharacterized protein ImpI VasC	fig|6666666.59329.peg.5196	None	fig|6666666.59331.peg.4530	fig|6666666.59332.peg.3510	None	fig|6666666.59335.peg.4110	fig|6666666.59336.peg.6379	None	None	fig|6666666.59339.peg.3985	None	fig|6666666.59337.peg.4729	fig|6666666.59342.peg.4391	None	fig|6666666.59344.peg.2978	fig|6666666.59345.peg.4116	fig|6666666.59341.peg.4559	fig|6666666.59347.peg.1022	None	fig|6666666.59348.peg.5372	fig|6666666.59349.peg.1640	None	None	None	fig|6666666.59355.peg.3601	None

9613	FIG01200033 hypothetical protein	fig|6666666.59329.peg.5964	fig|6666666.59330.peg.794	None	fig|6666666.59332.peg.6069	None	fig|6666666.59335.peg.554	fig|6666666.59336.peg.5474	fig|6666666.59333.peg.1529	None	fig|6666666.59339.peg.2953	None	fig|6666666.59337.peg.3821	fig|6666666.59342.peg.952	None	None	fig|6666666.59345.peg.65	None	None	fig|6666666.59346.peg.5280	fig|6666666.59348.peg.2433	fig|6666666.59349.peg.1908	None	fig|6666666.59351.peg.469	None	None	fig|6666666.59354.peg.5734

7072	Chromosome segregation ATPase	fig|6666666.59329.peg.3083	fig|6666666.59330.peg.4007	fig|6666666.59331.peg.1754	fig|6666666.59332.peg.941	None	fig|6666666.59335.peg.2034	fig|6666666.59336.peg.3817	fig|6666666.59333.peg.1349	None	fig|6666666.59339.peg.3153	None	fig|6666666.59337.peg.2745	fig|6666666.59342.peg.4699	None	None	fig|6666666.59345.peg.4303	fig|6666666.59341.peg.4250	fig|6666666.59347.peg.4408	fig|6666666.59346.peg.1481	fig|6666666.59348.peg.5231	None	None	None	None	None	None

1508	Membrane-associated phospholipid phosphatase	fig|6666666.59329.peg.6230	fig|6666666.59330.peg.4309	fig|6666666.59331.peg.3884	fig|6666666.59332.peg.4218	None	fig|6666666.59335.peg.5825	fig|6666666.59336.peg.3993	fig|6666666.59333.peg.4959	None	fig|6666666.59339.peg.6075	None	fig|6666666.59337.peg.5811	fig|6666666.59342.peg.5758	None	None	fig|6666666.59345.peg.4091	fig|6666666.59341.peg.4588	fig|6666666.59347.peg.1626	fig|6666666.59346.peg.2576	fig|6666666.59348.peg.2831	None	None	None	None	None	None

3827	3-oxoacyl-[ACP] synthase EC 2.3.1.41 FabV like	fig|6666666.59329.peg.3247	None	None	fig|6666666.59332.peg.3885	None	fig|6666666.59335.peg.5108	fig|6666666.59336.peg.1005	fig|6666666.59333.peg.1104	None	None	None	None	None	fig|6666666.71181.peg.3928	fig|6666666.59344.peg.5393	None	fig|6666666.59341.peg.4968	None	fig|6666666.59346.peg.2113	None	fig|6666666.59349.peg.311	fig|6666666.59350.peg.5352	fig|6666666.59351.peg.431	fig|6666666.59353.peg.4785	fig|6666666.59355.peg.4839	fig|6666666.59354.peg.1136

5133	Uronate isomerase EC 5.3.1.12	fig|6666666.59329.peg.2748	None	None	None	fig|6666666.59334.peg.940	None	fig|6666666.59336.peg.5896	None	fig|6666666.59338.peg.1690	None	fig|6666666.59340.peg.1827	fig|6666666.59337.peg.304	fig|6666666.59342.peg.2046	fig|6666666.71181.peg.4391	fig|6666666.59344.peg.5657	fig|6666666.59345.peg.2110	fig|6666666.59341.peg.4268	fig|6666666.59347.peg.174	None	None	None	fig|6666666.59350.peg.4355	None	fig|6666666.59353.peg.2390	fig|6666666.59355.peg.1663	None

7998	FIG01200181 hypothetical protein	fig|6666666.59329.peg.3372	fig|6666666.59330.peg.3704	None	fig|6666666.59332.peg.4809	None	fig|6666666.59335.peg.5832	fig|6666666.59336.peg.6059	fig|6666666.59333.peg.2692	fig|6666666.59338.peg.2497	fig|6666666.59339.peg.2852	None	fig|6666666.59337.peg.3041	fig|6666666.59342.peg.3694	None	None	fig|6666666.59345.peg.95	fig|6666666.59341.peg.1064	fig|6666666.59347.peg.984	fig|6666666.59346.peg.3636	fig|6666666.59348.peg.3030	None	None	None	None	None	None

8287	FIG01199553 hypothetical protein	fig|6666666.59329.peg.104	fig|6666666.59330.peg.2181	None	fig|6666666.59332.peg.172	None	fig|6666666.59335.peg.4670	fig|6666666.59336.peg.4058	fig|6666666.59333.peg.2392	fig|6666666.59338.peg.2428	fig|6666666.59339.peg.7411	None	fig|6666666.59337.peg.89	fig|6666666.59342.peg.3772	None	None	fig|6666666.59345.peg.3753	fig|6666666.59341.peg.86	fig|6666666.59347.peg.4030	fig|6666666.59346.peg.1369	fig|6666666.59348.peg.213	None	None	None	None	None	None

4170	Leucine-rich repeat protein	fig|6666666.59329.peg.3971	fig|6666666.59330.peg.2716	fig|6666666.59331.peg.4435	fig|6666666.59332.peg.994	None	fig|6666666.59335.peg.4528	fig|6666666.59336.peg.4898	fig|6666666.59333.peg.5168	None	fig|6666666.59339.peg.2487	fig|6666666.59340.peg.930	fig|6666666.59337.peg.2445	None	None	None	fig|6666666.59345.peg.604	fig|6666666.59341.peg.3379	fig|6666666.59347.peg.636	fig|6666666.59346.peg.3023	fig|6666666.59348.peg.3692	None	None	None	None	None	None

7395	C1242 Predicted Fe-S oxidoreductase	fig|6666666.59329.peg.1055	fig|6666666.59330.peg.2174	None	fig|6666666.59332.peg.182	fig|6666666.59334.peg.650	fig|6666666.59335.peg.2791	fig|6666666.59336.peg.2666	fig|6666666.59333.peg.2398	fig|6666666.59338.peg.2435	fig|6666666.59339.peg.4913	None	fig|6666666.59337.peg.81	None	None	None	fig|6666666.59345.peg.3761	fig|6666666.59341.peg.79	fig|6666666.59347.peg.4024	fig|6666666.59346.peg.1361	fig|6666666.59348.peg.422	None	None	None	None	None	None

61	FIG01202077 hypothetical protein	fig|6666666.59329.peg.28	fig|6666666.59330.peg.148	None	fig|6666666.59332.peg.3628	fig|6666666.59334.peg.466	fig|6666666.59335.peg.1948	fig|6666666.59336.peg.266	fig|6666666.59333.peg.2299	fig|6666666.59338.peg.1167	fig|6666666.59339.peg.8828	None	fig|6666666.59337.peg.5195	None	None	None	fig|6666666.59345.peg.5100	fig|6666666.59341.peg.187	fig|6666666.59347.peg.4538	fig|6666666.59346.peg.365	fig|6666666.59348.peg.1969	None	None	None	None	None	None

6521	Endonuclease I	fig|6666666.59329.peg.2234	fig|6666666.59330.peg.4325	fig|6666666.59331.peg.224	fig|6666666.59332.peg.3020	None	fig|6666666.59335.peg.5160	fig|6666666.59336.peg.2402	fig|6666666.59333.peg.1073	None	fig|6666666.59339.peg.2373	None	fig|6666666.59337.peg.3804	None	None	None	fig|6666666.59345.peg.1953	None	None	fig|6666666.59346.peg.3895	fig|6666666.59348.peg.2952	fig|6666666.59349.peg.1174	None	fig|6666666.59351.peg.51	None	None	fig|6666666.59354.peg.4223

6062	hypothetical protein	fig|6666666.59329.peg.1880	None	fig|6666666.59331.peg.2883	fig|6666666.59332.peg.3849	fig|6666666.59334.peg.3072	fig|6666666.59335.peg.3198	fig|6666666.59336.peg.2046	None	fig|6666666.59338.peg.5140	fig|6666666.59339.peg.7560	None	None	fig|6666666.59342.peg.3854	None	None	fig|6666666.59345.peg.1667	fig|6666666.59341.peg.2921	fig|6666666.59347.peg.1706	fig|6666666.59346.peg.880	fig|6666666.59348.peg.1877	None	fig|6666666.59350.peg.8067	None	None	None	None

7375	FIG01201537 hypothetical protein	fig|6666666.59329.peg.3866	fig|6666666.59330.peg.3516	fig|6666666.59331.peg.5461	None	fig|6666666.59334.peg.3292	None	fig|6666666.59336.peg.5348	fig|6666666.59333.peg.1001	fig|6666666.59338.peg.4169	fig|6666666.59339.peg.4308	None	fig|6666666.59337.peg.2431	fig|6666666.59342.peg.5576	None	None	fig|6666666.59345.peg.619	fig|6666666.59341.peg.5038	fig|6666666.59347.peg.1470	fig|6666666.59346.peg.2282	fig|6666666.59348.peg.2348	None	None	None	None	None	None

11145	FIG01199759 hypothetical protein	None	fig|6666666.59330.peg.4284	fig|6666666.59331.peg.702	fig|6666666.59332.peg.4243	None	None	fig|6666666.59336.peg.6471	None	fig|6666666.59338.peg.1189	fig|6666666.59339.peg.5882	fig|6666666.59340.peg.2456	fig|6666666.59337.peg.4029	fig|6666666.59342.peg.4914	None	None	fig|6666666.59345.peg.202	None	fig|6666666.59347.peg.2122	fig|6666666.59346.peg.382	None	fig|6666666.59349.peg.3740	None	fig|6666666.59351.peg.2810	None	None	fig|6666666.59354.peg.3050

2597	FIG00920305 hypothetical protein	fig|6666666.59329.peg.2243	fig|6666666.59330.peg.3167	fig|6666666.59331.peg.2637	fig|6666666.59332.peg.5384	fig|6666666.59334.peg.1331	fig|6666666.59335.peg.1223	None	fig|6666666.59333.peg.2796	fig|6666666.59338.peg.6174	fig|6666666.59339.peg.1514	None	fig|6666666.59337.peg.4557	None	None	None	fig|6666666.59345.peg.3251	fig|6666666.59341.peg.912	fig|6666666.59347.peg.1764	fig|6666666.59346.peg.2056	fig|6666666.59348.peg.2355	None	None	None	None	None	None

6043	MSHA biogenesis protein MshK	fig|6666666.59329.peg.5896	fig|6666666.59330.peg.9	fig|6666666.59331.peg.5767	fig|6666666.59332.peg.505	fig|6666666.59334.peg.734	fig|6666666.59335.peg.202	fig|6666666.59336.peg.1174	fig|6666666.59333.peg.578	None	fig|6666666.59339.peg.7762	None	fig|6666666.59337.peg.795	fig|6666666.59342.peg.5472	None	None	fig|6666666.59345.peg.4901	fig|6666666.59341.peg.3149	None	fig|6666666.59346.peg.1948	fig|6666666.59348.peg.1245	None	None	None	None	None	None

2108	Predicted ATPase related to phosphate starvation-inducible protein PhoH	None	fig|6666666.59330.peg.32	None	fig|6666666.59332.peg.4522	fig|6666666.59334.peg.2676	fig|6666666.59335.peg.241	None	fig|6666666.59333.peg.241	fig|6666666.59338.peg.4960	fig|6666666.59339.peg.8158	None	fig|6666666.59337.peg.817	None	None	None	fig|6666666.59345.peg.4875	fig|6666666.59341.peg.894	fig|6666666.59347.peg.3108	fig|6666666.59346.peg.2138	fig|6666666.59348.peg.1702	fig|6666666.59349.peg.2597	None	fig|6666666.59351.peg.2536	None	None	None

894	Alkaline phosphatase EC 3.1.3.1	fig|6666666.59329.peg.4056	fig|6666666.59330.peg.1226	None	None	None	fig|6666666.59335.peg.2748	fig|6666666.59336.peg.3013	None	None	fig|6666666.59339.peg.259	None	fig|6666666.59337.peg.3755	fig|6666666.59342.peg.1900	fig|6666666.71181.peg.158	fig|6666666.59344.peg.3742	fig|6666666.59345.peg.1172	None	None	None	None	fig|6666666.59349.peg.2099	None	fig|6666666.59351.peg.1638	fig|6666666.59353.peg.660	fig|6666666.59355.peg.143	fig|6666666.59354.peg.882

6872	FIG01200823 hypothetical protein	fig|6666666.59329.peg.4690	None	fig|6666666.59331.peg.3019	fig|6666666.59332.peg.5730	fig|6666666.59334.peg.3361	fig|6666666.59335.peg.2426	fig|6666666.59336.peg.2557	fig|6666666.59333.peg.2058	fig|6666666.59338.peg.3600	fig|6666666.59339.peg.5256	None	fig|6666666.59337.peg.5072	None	None	None	fig|6666666.59345.peg.4357	fig|6666666.59341.peg.1628	fig|6666666.59347.peg.4440	fig|6666666.59346.peg.3678	fig|6666666.59348.peg.1274	None	None	None	None	None	None

1526	Nikel transport family protein NikM	fig|6666666.59329.peg.1656	fig|6666666.59330.peg.1644	fig|6666666.59331.peg.5044	fig|6666666.59332.peg.2293	fig|6666666.59334.peg.4865	None	fig|6666666.59336.peg.1395	fig|6666666.59333.peg.1263	fig|6666666.59338.peg.3793	None	None	fig|6666666.59337.peg.1529	None	None	None	fig|6666666.59345.peg.3044	None	fig|6666666.59347.peg.1009	fig|6666666.59346.peg.2459	fig|6666666.59348.peg.5318	fig|6666666.59349.peg.4477	None	None	None	None	fig|6666666.59354.peg.317

6611	oxidoreductase, short-chain dehydrogenase reductase family	fig|6666666.59329.peg.4120	fig|6666666.59330.peg.3388	None	fig|6666666.59332.peg.5681	fig|6666666.59334.peg.1431	fig|6666666.59335.peg.3122	fig|6666666.59336.peg.6165	fig|6666666.59333.peg.3170	fig|6666666.59338.peg.4238	fig|6666666.59339.peg.173	fig|6666666.59340.peg.1037	fig|6666666.59337.peg.4424	None	None	None	fig|6666666.59345.peg.1137	fig|6666666.59341.peg.2716	fig|6666666.59347.peg.2566	fig|6666666.59346.peg.4377	None	None	None	None	None	None	None

4642	FIG01200830 hypothetical protein	fig|6666666.59329.peg.6283	fig|6666666.59330.peg.1973	None	fig|6666666.59332.peg.4118	None	fig|6666666.59335.peg.4739	fig|6666666.59336.peg.4681	fig|6666666.59333.peg.4080	fig|6666666.59338.peg.3991	fig|6666666.59339.peg.3763	None	fig|6666666.59337.peg.2614	fig|6666666.59342.peg.1779	None	None	fig|6666666.59345.peg.742	fig|6666666.59341.peg.5441	fig|6666666.59347.peg.3316	fig|6666666.59346.peg.4338	fig|6666666.59348.peg.3159	None	None	None	None	None	None

1743	Zn-dependent protease	fig|6666666.59329.peg.6129	fig|6666666.59330.peg.4638	fig|6666666.59331.peg.2127	fig|6666666.59332.peg.5150	None	fig|6666666.59335.peg.2272	fig|6666666.59336.peg.4881	fig|6666666.59333.peg.4509	None	fig|6666666.59339.peg.2907	None	fig|6666666.59337.peg.4967	fig|6666666.59342.peg.5963	None	None	fig|6666666.59345.peg.112	None	None	fig|6666666.59346.peg.2911	None	fig|6666666.59349.peg.4061	None	fig|6666666.59351.peg.3402	None	None	fig|6666666.59354.peg.2127

8487	Periplasmic protein torT precursor	fig|6666666.59329.peg.2005	fig|6666666.59330.peg.3179	fig|6666666.59331.peg.5548	fig|6666666.59332.peg.2859	None	fig|6666666.59335.peg.5650	fig|6666666.59336.peg.818	fig|6666666.59333.peg.4930	None	fig|6666666.59339.peg.8448	None	fig|6666666.59337.peg.5470	fig|6666666.59342.peg.658	None	None	fig|6666666.59345.peg.3298	fig|6666666.59341.peg.773	fig|6666666.59347.peg.975	fig|6666666.59346.peg.3654	fig|6666666.59348.peg.3942	None	None	None	None	None	None

5989	Urease alpha subunit EC 3.5.1.5	fig|6666666.59329.peg.2207	None	fig|6666666.59331.peg.1779	fig|6666666.59332.peg.5419	None	fig|6666666.59335.peg.4462	None	None	fig|6666666.59338.peg.4657	fig|6666666.59339.peg.5359	fig|6666666.59340.peg.3587	None	None	None	None	fig|6666666.59345.peg.4558	fig|6666666.59341.peg.6050	fig|6666666.59347.peg.567	fig|6666666.59346.peg.3222	fig|6666666.59348.peg.543	None	fig|6666666.59350.peg.5027	None	None	fig|6666666.59355.peg.5494	None

8529	FIG01200987 hypothetical protein	fig|6666666.59329.peg.1437	fig|6666666.59330.peg.3566	None	fig|6666666.59332.peg.1481	None	fig|6666666.59335.peg.5264	fig|6666666.59336.peg.2379	fig|6666666.59333.peg.1202	fig|6666666.59338.peg.4995	fig|6666666.59339.peg.555	None	fig|6666666.59337.peg.3654	None	None	None	fig|6666666.59345.peg.1234	fig|6666666.59341.peg.5777	fig|6666666.59347.peg.1938	fig|6666666.59346.peg.3497	fig|6666666.59348.peg.364	None	None	None	None	None	None

5862	FIG01203004 hypothetical protein	fig|6666666.59329.peg.611	fig|6666666.59330.peg.1780	fig|6666666.59331.peg.5961	None	None	None	fig|6666666.59336.peg.652	fig|6666666.59333.peg.1130	fig|6666666.59338.peg.3334	None	None	fig|6666666.59337.peg.2262	fig|6666666.59342.peg.4535	None	None	fig|6666666.59345.peg.2048	fig|6666666.59341.peg.936	fig|6666666.59347.peg.798	fig|6666666.59346.peg.5336	fig|6666666.59348.peg.755	fig|6666666.59349.peg.4395	None	None	None	None	None

6900	Flp pilus assembly surface protein TadF, ATP GTP-binding motif	fig|6666666.59329.peg.5593	fig|6666666.59330.peg.671	None	fig|6666666.59332.peg.695	fig|6666666.59334.peg.1508	fig|6666666.59335.peg.1606	fig|6666666.59336.peg.1099	None	fig|6666666.59338.peg.3696	fig|6666666.59339.peg.1892	None	fig|6666666.59337.peg.5175	fig|6666666.59342.peg.692	None	None	fig|6666666.59345.peg.2307	fig|6666666.59341.peg.4596	fig|6666666.59347.peg.1906	None	fig|6666666.59348.peg.1140	None	None	None	None	None	None

814	FIG01200346 hypothetical protein	fig|6666666.59329.peg.2462	None	None	None	fig|6666666.59334.peg.5176	fig|6666666.59335.peg.5615	None	fig|6666666.59333.peg.3246	fig|6666666.59338.peg.3883	fig|6666666.59339.peg.1204	fig|6666666.59340.peg.2629	fig|6666666.59337.peg.2713	fig|6666666.59342.peg.3665	None	None	fig|6666666.59345.peg.3191	fig|6666666.59341.peg.4011	fig|6666666.59347.peg.1697	fig|6666666.59346.peg.3685	fig|6666666.59348.peg.2825	None	None	None	None	None	None

8738	hypothetical protein	fig|6666666.59329.peg.5977	fig|6666666.59330.peg.1144	fig|6666666.59331.peg.1599	fig|6666666.59332.peg.1869	fig|6666666.59334.peg.1568	None	fig|6666666.59336.peg.2484	None	fig|6666666.59338.peg.2876	None	None	fig|6666666.59337.peg.3644	fig|6666666.59342.peg.6381	None	None	fig|6666666.59345.peg.5203	fig|6666666.59341.peg.1757	fig|6666666.59347.peg.754	fig|6666666.59346.peg.683	fig|6666666.59348.peg.4238	None	None	None	None	None	None

10794	Alpha-galactosidase	fig|6666666.59329.peg.5484	fig|6666666.59330.peg.1529	None	None	None	fig|6666666.59335.peg.6252	fig|6666666.59336.peg.3598	fig|6666666.59333.peg.2914	fig|6666666.59338.peg.6183	fig|6666666.59339.peg.379	fig|6666666.59340.peg.1235	fig|6666666.59337.peg.2316	fig|6666666.59342.peg.6491	None	None	fig|6666666.59345.peg.1282	fig|6666666.59341.peg.5979	fig|6666666.59347.peg.2303	None	fig|6666666.59348.peg.5111	None	None	None	None	None	None

11243	Catalase EC 1.11.1.6	fig|6666666.59329.peg.2870	fig|6666666.59330.peg.3609	None	None	None	None	fig|6666666.59336.peg.1155	fig|6666666.59333.peg.3719	None	None	None	fig|6666666.59337.peg.2995	fig|6666666.59342.peg.1341	fig|6666666.71181.peg.513	fig|6666666.59344.peg.1733	fig|6666666.59345.peg.642	fig|6666666.59341.peg.1787	None	None	None	fig|6666666.59349.peg.3155	None	None	fig|6666666.59353.peg.1140	fig|6666666.59355.peg.336	fig|6666666.59354.peg.4297

5807	2-hydroxychromene-2-carboxylate isomerase DsbA-like thioredoxin domain	fig|6666666.59329.peg.2928	None	fig|6666666.59331.peg.653	fig|6666666.59332.peg.1600	fig|6666666.59334.peg.5092	fig|6666666.59335.peg.4521	fig|6666666.59336.peg.2462	None	fig|6666666.59338.peg.5220	fig|6666666.59339.peg.2476	None	None	None	None	None	fig|6666666.59345.peg.3991	fig|6666666.59341.peg.3898	fig|6666666.59347.peg.4720	fig|6666666.59346.peg.2761	fig|6666666.59348.peg.3143	None	fig|6666666.59350.peg.538	None	None	None	None

9450	Hypothetical protein VC0266 sugar utilization related?	fig|6666666.59329.peg.675	fig|6666666.59330.peg.3770	None	None	fig|6666666.59334.peg.5280	fig|6666666.59335.peg.453	fig|6666666.59336.peg.3841	None	None	fig|6666666.59339.peg.6423	None	fig|6666666.59337.peg.4510	None	fig|6666666.71181.peg.6379	None	fig|6666666.59345.peg.5689	None	None	None	fig|6666666.59348.peg.3196	fig|6666666.59349.peg.3093	fig|6666666.59350.peg.2229	fig|6666666.59351.peg.2959	None	None	fig|6666666.59354.peg.3462

6415	Protocatechuate 3,4-dioxygenase beta chain EC 1.13.11.3	fig|6666666.59329.peg.737	fig|6666666.59330.peg.3061	None	None	None	None	fig|6666666.59336.peg.3218	fig|6666666.59333.peg.1420	fig|6666666.59338.peg.6118	None	None	None	None	fig|6666666.71181.peg.3381	fig|6666666.59344.peg.5764	fig|6666666.59345.peg.343	fig|6666666.59341.peg.1457	fig|6666666.59347.peg.1247	None	None	None	fig|6666666.59350.peg.1838	fig|6666666.59351.peg.4595	fig|6666666.59353.peg.5032	fig|6666666.59355.peg.3213	None

8077	Transcriptional regulator, DeoR family	None	fig|6666666.59330.peg.2363	None	None	None	fig|6666666.59335.peg.966	None	fig|6666666.59333.peg.3082	None	fig|6666666.59339.peg.5863	None	fig|6666666.59337.peg.2233	None	fig|6666666.71181.peg.5965	fig|6666666.59344.peg.3832	None	None	None	None	fig|6666666.59348.peg.4087	fig|6666666.59349.peg.1845	fig|6666666.59350.peg.1454	fig|6666666.59351.peg.4302	fig|6666666.59353.peg.3967	fig|6666666.59355.peg.3883	fig|6666666.59354.peg.4437

10108	membrane protein	fig|6666666.59329.peg.3311	fig|6666666.59330.peg.3690	None	fig|6666666.59332.peg.3232	None	fig|6666666.59335.peg.1352	fig|6666666.59336.peg.1483	fig|6666666.59333.peg.1195	fig|6666666.59338.peg.4571	fig|6666666.59339.peg.900	None	fig|6666666.59337.peg.4264	None	None	None	fig|6666666.59345.peg.1224	fig|6666666.59341.peg.867	fig|6666666.59347.peg.1998	fig|6666666.59346.peg.224	fig|6666666.59348.peg.4531	None	None	None	None	None	None

3457	Lysophospholipase EC 3.1.1.5	None	None	fig|6666666.59331.peg.2077	fig|6666666.59332.peg.4358	fig|6666666.59334.peg.576	fig|6666666.59335.peg.3395	None	None	fig|6666666.59338.peg.5663	fig|6666666.59339.peg.4310	None	None	None	None	None	fig|6666666.59345.peg.978	fig|6666666.59341.peg.5933	fig|6666666.59347.peg.1942	fig|6666666.59346.peg.4755	fig|6666666.59348.peg.4557	fig|6666666.59349.peg.4262	None	fig|6666666.59351.peg.5285	None	None	fig|6666666.59354.peg.1608

971	FIG01199665 hypothetical protein	fig|6666666.59329.peg.3910	fig|6666666.59330.peg.717	None	fig|6666666.59332.peg.3330	fig|6666666.59334.peg.3532	fig|6666666.59335.peg.5476	fig|6666666.59336.peg.4359	fig|6666666.59333.peg.4168	fig|6666666.59338.peg.3617	fig|6666666.59339.peg.2619	None	fig|6666666.59337.peg.3779	None	None	None	fig|6666666.59345.peg.2812	fig|6666666.59341.peg.1391	fig|6666666.59347.peg.1947	fig|6666666.59346.peg.4319	None	None	None	None	None	None	None

311	Ribosome recycling factor	fig|6666666.59329.peg.1879	fig|6666666.59330.peg.3206	None	fig|6666666.59332.peg.512	None	fig|6666666.59335.peg.5193	fig|6666666.59336.peg.2045	fig|6666666.59333.peg.1306	None	fig|6666666.59339.peg.817	None	fig|6666666.59337.peg.5146	fig|6666666.59342.peg.4130	None	None	fig|6666666.59345.peg.1669	fig|6666666.59341.peg.730	fig|6666666.59347.peg.3438	fig|6666666.59346.peg.177	fig|6666666.59348.peg.569	None	None	None	None	None	None

11207	Lysine decarboxylase, inducible EC 4.1.1.18	fig|6666666.59329.peg.1950	fig|6666666.59330.peg.4031	None	None	None	None	fig|6666666.59336.peg.3908	fig|6666666.59333.peg.4116	None	None	None	fig|6666666.59337.peg.4497	fig|6666666.59342.peg.2917	fig|6666666.71181.peg.17	fig|6666666.59344.peg.4358	fig|6666666.59345.peg.5674	fig|6666666.59341.peg.5568	None	None	None	fig|6666666.59349.peg.4638	None	None	fig|6666666.59353.peg.2002	fig|6666666.59355.peg.4107	fig|6666666.59354.peg.813

8694	GlcG protein	None	None	fig|6666666.59331.peg.6297	fig|6666666.59332.peg.5900	None	fig|6666666.59335.peg.2781	None	None	fig|6666666.59338.peg.2468	fig|6666666.59339.peg.5390	None	None	None	fig|6666666.71181.peg.1787	fig|6666666.59344.peg.5157	None	fig|6666666.59341.peg.2736	fig|6666666.59347.peg.3338	fig|6666666.59346.peg.3235	fig|6666666.59348.peg.1610	None	fig|6666666.59350.peg.2689	None	fig|6666666.59353.peg.2727	fig|6666666.59355.peg.1754	None

3045	hypothetical protein	fig|6666666.59329.peg.3923	None	fig|6666666.59331.peg.5893	fig|6666666.59332.peg.2967	fig|6666666.59334.peg.1850	fig|6666666.59335.peg.6153	fig|6666666.59336.peg.2628	None	None	fig|6666666.59339.peg.6136	None	fig|6666666.59337.peg.5354	fig|6666666.59342.peg.1415	None	None	fig|6666666.59345.peg.257	fig|6666666.59341.peg.4758	fig|6666666.59347.peg.4667	fig|6666666.59346.peg.4216	fig|6666666.59348.peg.236	None	None	None	None	None	None

7736	Hydroxymethylglutaryl-CoA reductase EC 1.1.1.34	fig|6666666.59329.peg.2041	fig|6666666.59330.peg.763	None	fig|6666666.59332.peg.5606	fig|6666666.59334.peg.1224	fig|6666666.59335.peg.5526	None	fig|6666666.59333.peg.5058	fig|6666666.59338.peg.4209	fig|6666666.59339.peg.2826	None	fig|6666666.59337.peg.1205	fig|6666666.59342.peg.3297	None	None	fig|6666666.59345.peg.24	fig|6666666.59341.peg.5623	fig|6666666.59347.peg.891	fig|6666666.59346.peg.3165	None	None	None	None	None	None	None

590	Nitrogen regulatory protein P-II	fig|6666666.59329.peg.4692	None	fig|6666666.59331.peg.3061	fig|6666666.59332.peg.5731	fig|6666666.59334.peg.3362	None	fig|6666666.59336.peg.2559	fig|6666666.59333.peg.2057	fig|6666666.59338.peg.3599	fig|6666666.59339.peg.5254	None	fig|6666666.59337.peg.5071	None	None	None	fig|6666666.59345.peg.4358	fig|6666666.59341.peg.1627	fig|6666666.59347.peg.4439	fig|6666666.59346.peg.3679	fig|6666666.59348.peg.1272	None	None	None	None	None	None

4865	FIG01200460 hypothetical protein	fig|6666666.59329.peg.4441	None	None	fig|6666666.59332.peg.2340	None	None	fig|6666666.59336.peg.3695	fig|6666666.59333.peg.217	fig|6666666.59338.peg.3516	fig|6666666.59339.peg.1759	fig|6666666.59340.peg.3335	fig|6666666.59337.peg.3324	fig|6666666.59342.peg.1157	None	None	fig|6666666.59345.peg.5431	fig|6666666.59341.peg.4017	fig|6666666.59347.peg.3660	fig|6666666.59346.peg.4670	fig|6666666.59348.peg.3707	None	None	None	None	None	None

453	Periplasmic nitrate reductase component NapD	fig|6666666.59329.peg.2512	None	fig|6666666.59331.peg.5451	fig|6666666.59332.peg.4192	fig|6666666.59334.peg.4425	fig|6666666.59335.peg.5591	fig|6666666.59336.peg.5879	fig|6666666.59333.peg.1889	None	fig|6666666.59339.peg.2862	None	fig|6666666.59337.peg.3537	fig|6666666.59342.peg.959	None	None	fig|6666666.59345.peg.483	fig|6666666.59341.peg.5205	fig|6666666.59347.peg.668	fig|6666666.59346.peg.503	None	None	None	None	None	None	None

10661	Urea ABC transporter, permease protein UrtC	None	None	fig|6666666.59331.peg.1642	fig|6666666.59332.peg.3455	None	None	None	fig|6666666.59333.peg.5579	fig|6666666.59338.peg.1463	fig|6666666.59339.peg.5369	None	None	None	fig|6666666.71181.peg.4837	fig|6666666.59344.peg.4865	None	fig|6666666.59341.peg.241	fig|6666666.59347.peg.561	fig|6666666.59346.peg.1306	fig|6666666.59348.peg.549	None	fig|6666666.59350.peg.1845	None	fig|6666666.59353.peg.3778	fig|6666666.59355.peg.4956	None

7322	Nicotinamidase EC 3.5.1.19	fig|6666666.59329.peg.4007	fig|6666666.59330.peg.4811	None	None	None	None	fig|6666666.59336.peg.3512	fig|6666666.59333.peg.2102	None	None	None	fig|6666666.59337.peg.2838	fig|6666666.59342.peg.4655	fig|6666666.71181.peg.2973	fig|6666666.59344.peg.2896	fig|6666666.59345.peg.1759	fig|6666666.59341.peg.1467	None	None	fig|6666666.59348.peg.1417	None	fig|6666666.59350.peg.4212	None	fig|6666666.59353.peg.1431	fig|6666666.59355.peg.1980	None

6680	Predicted redox protein	fig|6666666.59329.peg.5996	fig|6666666.59330.peg.693	None	fig|6666666.59332.peg.511	None	fig|6666666.59335.peg.5499	fig|6666666.59336.peg.2986	fig|6666666.59333.peg.2736	None	fig|6666666.59339.peg.2333	None	fig|6666666.59337.peg.4158	fig|6666666.59342.peg.3421	None	None	fig|6666666.59345.peg.4773	fig|6666666.59341.peg.924	None	fig|6666666.59346.peg.176	fig|6666666.59348.peg.2597	None	None	fig|6666666.59351.peg.1288	None	None	None

2427	FIG01201138 hypothetical protein	fig|6666666.59329.peg.2702	fig|6666666.59330.peg.2935	None	fig|6666666.59332.peg.3762	None	fig|6666666.59335.peg.3845	fig|6666666.59336.peg.873	fig|6666666.59333.peg.4977	fig|6666666.59338.peg.564	fig|6666666.59339.peg.569	None	fig|6666666.59337.peg.1735	fig|6666666.59342.peg.4869	None	None	fig|6666666.59345.peg.907	fig|6666666.59341.peg.5352	fig|6666666.59347.peg.2296	fig|6666666.59346.peg.1613	None	None	None	None	None	None	None

3249	Serine threonine protein phosphatase	fig|6666666.59329.peg.1786	fig|6666666.59330.peg.4167	None	None	fig|6666666.59334.peg.1837	None	None	fig|6666666.59333.peg.3053	fig|6666666.59338.peg.5915	None	fig|6666666.59340.peg.1971	fig|6666666.59337.peg.4780	fig|6666666.59342.peg.1251	fig|6666666.71181.peg.2268	fig|6666666.59344.peg.5870	fig|6666666.59345.peg.3444	None	fig|6666666.59347.peg.2421	None	None	None	None	None	fig|6666666.59353.peg.4904	fig|6666666.59355.peg.5323	None

7564	4-carboxymuconolactone decarboxylase EC 4.1.1.44	fig|6666666.59329.peg.538	None	fig|6666666.59331.peg.153	fig|6666666.59332.peg.5325	None	None	fig|6666666.59336.peg.6124	fig|6666666.59333.peg.1816	None	None	None	None	None	fig|6666666.71181.peg.2142	fig|6666666.59344.peg.3212	None	None	fig|6666666.59347.peg.1844	None	fig|6666666.59348.peg.3726	fig|6666666.59349.peg.2144	fig|6666666.59350.peg.1841	None	fig|6666666.59353.peg.2829	fig|6666666.59355.peg.3932	fig|6666666.59354.peg.1880

7366	Outer membrane protein protective antigen OMA87	fig|6666666.59329.peg.2486	None	fig|6666666.59331.peg.5299	fig|6666666.59332.peg.3306	fig|6666666.59334.peg.4794	None	fig|6666666.59336.peg.2576	None	fig|6666666.59338.peg.5232	None	fig|6666666.59340.peg.3805	fig|6666666.59337.peg.4362	fig|6666666.59342.peg.2014	None	None	fig|6666666.59345.peg.1900	fig|6666666.59341.peg.2676	fig|6666666.59347.peg.1053	fig|6666666.59346.peg.2193	fig|6666666.59348.peg.2425	None	None	None	None	None	None

6395	hypothetical protein	None	fig|6666666.59330.peg.2364	None	None	None	fig|6666666.59335.peg.964	fig|6666666.59336.peg.752	fig|6666666.59333.peg.3083	fig|6666666.59338.peg.5452	fig|6666666.59339.peg.4485	None	fig|6666666.59337.peg.2234	None	None	None	fig|6666666.59345.peg.4450	None	fig|6666666.59347.peg.304	None	fig|6666666.59348.peg.4085	fig|6666666.59349.peg.1846	fig|6666666.59350.peg.1453	fig|6666666.59351.peg.4301	None	None	fig|6666666.59354.peg.4436

4967	Alpha-mannosidase EC 3.2.1.24	fig|6666666.59329.peg.3629	None	fig|6666666.59331.peg.2771	None	fig|6666666.59334.peg.4154	fig|6666666.59335.peg.5349	fig|6666666.59336.peg.2349	None	fig|6666666.59338.peg.3047	fig|6666666.59339.peg.1792	None	fig|6666666.59337.peg.5662	fig|6666666.59342.peg.4226	None	None	fig|6666666.59345.peg.4041	fig|6666666.59341.peg.2045	fig|6666666.59347.peg.397	fig|6666666.59346.peg.521	fig|6666666.59348.peg.621	None	None	None	None	None	None

7503	FIG001196 putative membrane protein	fig|6666666.59329.peg.4114	None	None	None	None	fig|6666666.59335.peg.5305	fig|6666666.59336.peg.6145	None	fig|6666666.59338.peg.2915	fig|6666666.59339.peg.1013	None	fig|6666666.59337.peg.3088	fig|6666666.59342.peg.3241	fig|6666666.71181.peg.974	fig|6666666.59344.peg.5335	fig|6666666.59345.peg.4166	None	fig|6666666.59347.peg.69	None	None	None	fig|6666666.59350.peg.3371	None	fig|6666666.59353.peg.2288	fig|6666666.59355.peg.4816	None

10856	FIG01201067 hypothetical protein	fig|6666666.59329.peg.1061	fig|6666666.59330.peg.184	fig|6666666.59331.peg.863	fig|6666666.59332.peg.146	None	fig|6666666.59335.peg.2608	fig|6666666.59336.peg.1297	fig|6666666.59333.peg.2208	None	fig|6666666.59339.peg.2247	None	fig|6666666.59337.peg.4298	None	None	None	fig|6666666.59345.peg.5136	None	None	fig|6666666.59346.peg.776	None	fig|6666666.59349.peg.4258	None	fig|6666666.59351.peg.5289	None	None	fig|6666666.59354.peg.1613

9647	Iron-sulfur cluster-binding protein	fig|6666666.59329.peg.4869	fig|6666666.59330.peg.2589	fig|6666666.59331.peg.4311	fig|6666666.59332.peg.5368	None	fig|6666666.59335.peg.2190	fig|6666666.59336.peg.4846	None	fig|6666666.59338.peg.6060	fig|6666666.59339.peg.1281	None	fig|6666666.59337.peg.3697	None	None	None	fig|6666666.59345.peg.3209	fig|6666666.59341.peg.2899	fig|6666666.59347.peg.1717	fig|6666666.59346.peg.3282	None	None	fig|6666666.59350.peg.396	None	None	None	None

3568	FIG01200906 hypothetical protein	fig|6666666.59329.peg.4622	fig|6666666.59330.peg.2310	None	fig|6666666.59332.peg.3175	None	fig|6666666.59335.peg.4601	None	fig|6666666.59333.peg.3691	fig|6666666.59338.peg.3754	fig|6666666.59339.peg.3731	None	fig|6666666.59337.peg.1766	fig|6666666.59342.peg.2827	None	None	fig|6666666.59345.peg.878	fig|6666666.59341.peg.5270	fig|6666666.59347.peg.2248	fig|6666666.59346.peg.4443	fig|6666666.59348.peg.4586	None	None	None	None	None	None

10640	Superfamily II DNA and RNA helicase	fig|6666666.59329.peg.3049	fig|6666666.59330.peg.2986	None	fig|6666666.59332.peg.4265	fig|6666666.59334.peg.5494	fig|6666666.59335.peg.6032	fig|6666666.59336.peg.2901	fig|6666666.59333.peg.3668	None	fig|6666666.59339.peg.965	None	fig|6666666.59337.peg.4146	fig|6666666.59342.peg.1067	None	None	fig|6666666.59345.peg.4614	fig|6666666.59341.peg.1401	None	fig|6666666.59346.peg.4893	fig|6666666.59348.peg.3081	None	None	None	None	None	None

6827	putative integral membrane protein	fig|6666666.59329.peg.1876	None	fig|6666666.59331.peg.1964	None	fig|6666666.59334.peg.4619	fig|6666666.59335.peg.3385	fig|6666666.59336.peg.2058	None	fig|6666666.59338.peg.4399	fig|6666666.59339.peg.688	None	fig|6666666.59337.peg.4654	fig|6666666.59342.peg.971	None	None	fig|6666666.59345.peg.1937	fig|6666666.59341.peg.6083	fig|6666666.59347.peg.4239	None	None	None	fig|6666666.59350.peg.2853	None	None	None	fig|6666666.59354.peg.3434

1165	Urea ABC transporter, urea binding protein	None	None	None	fig|6666666.59332.peg.3457	None	fig|6666666.59335.peg.4389	None	None	fig|6666666.59338.peg.1457	fig|6666666.59339.peg.4126	fig|6666666.59340.peg.1515	None	None	fig|6666666.71181.peg.1119	fig|6666666.59344.peg.5594	None	fig|6666666.59341.peg.237	fig|6666666.59347.peg.559	fig|6666666.59346.peg.1309	fig|6666666.59348.peg.1870	None	fig|6666666.59350.peg.4831	None	fig|6666666.59353.peg.4883	fig|6666666.59355.peg.4954	None

1369	Flp pilus assembly protein, pilin Flp	fig|6666666.59329.peg.5141	fig|6666666.59330.peg.659	fig|6666666.59331.peg.2136	fig|6666666.59332.peg.682	fig|6666666.59334.peg.3482	fig|6666666.59335.peg.1588	fig|6666666.59336.peg.698	None	fig|6666666.59338.peg.5546	None	None	fig|6666666.59337.peg.5483	fig|6666666.59342.peg.361	None	None	fig|6666666.59345.peg.2317	fig|6666666.59341.peg.3846	fig|6666666.59347.peg.1916	None	fig|6666666.59348.peg.5287	None	None	None	None	None	None

4656	FIGfam138462 Acyl-CoA synthetase, AMP- fatty acid ligase	fig|6666666.59329.peg.3342	None	fig|6666666.59331.peg.3730	fig|6666666.59332.peg.3639	None	None	fig|6666666.59336.peg.1051	fig|6666666.59333.peg.1097	None	None	None	None	None	fig|6666666.71181.peg.3935	fig|6666666.59344.peg.5383	None	fig|6666666.59341.peg.2759	None	fig|6666666.59346.peg.2124	None	fig|6666666.59349.peg.2866	None	fig|6666666.59351.peg.2869	fig|6666666.59353.peg.4792	fig|6666666.59355.peg.4846	fig|6666666.59354.peg.3642

105	Electron transfer flavoprotein-ubiquinone oxidoreductase EC 1.5.5.1	fig|6666666.59329.peg.2138	fig|6666666.59330.peg.4448	None	None	None	fig|6666666.59335.peg.4298	None	fig|6666666.59333.peg.4285	fig|6666666.59338.peg.3113	None	None	fig|6666666.59337.peg.4797	None	None	None	fig|6666666.59345.peg.323	fig|6666666.59341.peg.1709	fig|6666666.59347.peg.1155	None	fig|6666666.59348.peg.3519	fig|6666666.59349.peg.4287	fig|6666666.59350.peg.3700	fig|6666666.59351.peg.1076	None	None	fig|6666666.59354.peg.2723

7267	FIG01201795 hypothetical protein	fig|6666666.59329.peg.2480	fig|6666666.59330.peg.2027	fig|6666666.59331.peg.1340	fig|6666666.59332.peg.411	fig|6666666.59334.peg.1734	fig|6666666.59335.peg.2416	fig|6666666.59336.peg.6289	fig|6666666.59333.peg.4192	None	fig|6666666.59339.peg.5272	None	None	fig|6666666.59342.peg.6383	None	None	fig|6666666.59345.peg.3505	fig|6666666.59341.peg.5906	None	fig|6666666.59346.peg.4060	fig|6666666.59348.peg.3708	None	None	None	None	None	None

10245	Hcp	fig|6666666.59329.peg.2982	fig|6666666.59330.peg.4385	None	None	None	fig|6666666.59335.peg.3426	fig|6666666.59336.peg.5498	fig|6666666.59333.peg.3794	None	fig|6666666.59339.peg.2702	None	fig|6666666.59337.peg.4819	fig|6666666.59342.peg.1624	fig|6666666.71181.peg.285	fig|6666666.59344.peg.1214	fig|6666666.59345.peg.3366	None	None	None	fig|6666666.59348.peg.4176	None	None	None	fig|6666666.59353.peg.1715	fig|6666666.59355.peg.1005	None

5376	haloacid dehalogenase-like hydrolase	fig|6666666.59329.peg.2188	fig|6666666.59330.peg.3240	fig|6666666.59331.peg.4762	fig|6666666.59332.peg.3416	None	fig|6666666.59335.peg.5270	fig|6666666.59336.peg.1664	None	fig|6666666.59338.peg.3762	fig|6666666.59339.peg.5724	None	fig|6666666.59337.peg.2151	None	None	None	fig|6666666.59345.peg.3964	fig|6666666.59341.peg.5446	fig|6666666.59347.peg.3529	fig|6666666.59346.peg.1422	fig|6666666.59348.peg.2076	None	None	None	None	None	None

3033	Chromosome segregation ATPase	fig|6666666.59329.peg.6302	fig|6666666.59330.peg.4761	None	None	None	fig|6666666.59335.peg.1937	fig|6666666.59336.peg.2351	fig|6666666.59333.peg.2405	fig|6666666.59338.peg.5619	fig|6666666.59339.peg.8411	fig|6666666.59340.peg.2449	fig|6666666.59337.peg.5862	fig|6666666.59342.peg.6289	None	None	fig|6666666.59345.peg.1363	fig|6666666.59341.peg.4681	fig|6666666.59347.peg.83	None	fig|6666666.59348.peg.4113	None	None	None	None	None	None

2501	Agmatine deiminase EC 3.5.3.12	None	None	None	fig|6666666.59332.peg.5380	fig|6666666.59334.peg.2604	None	fig|6666666.59336.peg.3087	fig|6666666.59333.peg.5461	fig|6666666.59338.peg.3663	None	fig|6666666.59340.peg.2088	None	fig|6666666.59342.peg.5909	None	None	None	fig|6666666.59341.peg.3990	fig|6666666.59347.peg.379	fig|6666666.59346.peg.1116	None	fig|6666666.59349.peg.1798	fig|6666666.59350.peg.4958	fig|6666666.59351.peg.991	None	None	fig|6666666.59354.peg.1804

5047	FIG01199915 hypothetical protein	None	fig|6666666.59330.peg.3372	None	fig|6666666.59332.peg.5868	None	fig|6666666.59335.peg.5222	fig|6666666.59336.peg.6281	fig|6666666.59333.peg.538	fig|6666666.59338.peg.3301	fig|6666666.59339.peg.3884	None	fig|6666666.59337.peg.4525	fig|6666666.59342.peg.387	None	None	fig|6666666.59345.peg.4661	fig|6666666.59341.peg.447	fig|6666666.59347.peg.1875	fig|6666666.59346.peg.2926	fig|6666666.59348.peg.3545	None	None	None	None	None	None

1520	FIG01200128 hypothetical protein	fig|6666666.59329.peg.1692	fig|6666666.59330.peg.3463	None	fig|6666666.59332.peg.855	fig|6666666.59334.peg.3513	fig|6666666.59335.peg.3068	fig|6666666.59336.peg.3746	fig|6666666.59333.peg.5285	fig|6666666.59338.peg.5007	fig|6666666.59339.peg.5750	None	fig|6666666.59337.peg.5424	None	None	None	fig|6666666.59345.peg.1920	fig|6666666.59341.peg.805	fig|6666666.59347.peg.3793	fig|6666666.59346.peg.1328	None	None	None	None	None	None	None

1122	FIG01201645 hypothetical protein	fig|6666666.59329.peg.2779	None	None	fig|6666666.59332.peg.3013	None	fig|6666666.59335.peg.3127	fig|6666666.59336.peg.1107	fig|6666666.59333.peg.4654	fig|6666666.59338.peg.4774	fig|6666666.59339.peg.5420	fig|6666666.59340.peg.3110	None	fig|6666666.59342.peg.2160	None	None	fig|6666666.59345.peg.1891	None	fig|6666666.59347.peg.1432	fig|6666666.59346.peg.1031	fig|6666666.59348.peg.1812	None	fig|6666666.59350.peg.4790	None	None	None	None

2718	FIG01199598 hypothetical protein	fig|6666666.59329.peg.5514	fig|6666666.59330.peg.1947	None	fig|6666666.59332.peg.4446	None	fig|6666666.59335.peg.1799	None	fig|6666666.59333.peg.5180	fig|6666666.59338.peg.6114	fig|6666666.59339.peg.3615	None	fig|6666666.59337.peg.5456	fig|6666666.59342.peg.844	None	None	fig|6666666.59345.peg.715	fig|6666666.59341.peg.3520	fig|6666666.59347.peg.1689	fig|6666666.59346.peg.2422	fig|6666666.59348.peg.1453	None	None	None	None	None	None

5663	Ribonuclease Z EC 3.1.26.11	fig|6666666.59329.peg.4726	None	fig|6666666.59331.peg.205	None	None	None	fig|6666666.59336.peg.4072	None	None	fig|6666666.59339.peg.2022	fig|6666666.59340.peg.3240	fig|6666666.59337.peg.4400	fig|6666666.59342.peg.2490	None	None	fig|6666666.59345.peg.3421	fig|6666666.59341.peg.2314	fig|6666666.59347.peg.4717	fig|6666666.59346.peg.4118	None	fig|6666666.59349.peg.975	None	fig|6666666.59351.peg.1570	None	None	fig|6666666.59354.peg.5915

5661	hypothetical protein	fig|6666666.59329.peg.1655	fig|6666666.59330.peg.1645	fig|6666666.59331.peg.2201	fig|6666666.59332.peg.2294	fig|6666666.59334.peg.656	None	fig|6666666.59336.peg.1394	fig|6666666.59333.peg.1264	fig|6666666.59338.peg.3794	None	fig|6666666.59340.peg.3698	fig|6666666.59337.peg.1528	None	None	None	fig|6666666.59345.peg.3043	None	fig|6666666.59347.peg.1118	fig|6666666.59346.peg.3788	fig|6666666.59348.peg.5319	None	None	None	None	None	None

2778	Pirin	None	None	fig|6666666.59331.peg.3814	None	None	fig|6666666.59335.peg.3194	fig|6666666.59336.peg.3774	fig|6666666.59333.peg.1225	fig|6666666.59338.peg.3416	fig|6666666.59339.peg.5926	None	None	fig|6666666.59342.peg.4219	fig|6666666.71181.peg.1104	fig|6666666.59344.peg.1848	fig|6666666.59345.peg.4524	None	None	None	fig|6666666.59348.peg.4853	None	None	fig|6666666.59351.peg.2885	fig|6666666.59353.peg.2378	fig|6666666.59355.peg.1650	None

4890	Ccm2-related protein	fig|6666666.59329.peg.2132	fig|6666666.59330.peg.1794	None	None	fig|6666666.59334.peg.3451	None	fig|6666666.59336.peg.1034	fig|6666666.59333.peg.3507	None	fig|6666666.59339.peg.127	fig|6666666.59340.peg.1501	fig|6666666.59337.peg.2297	None	None	None	fig|6666666.59345.peg.1997	fig|6666666.59341.peg.344	fig|6666666.59347.peg.3653	None	None	fig|6666666.59349.peg.4013	None	fig|6666666.59351.peg.1425	None	None	fig|6666666.59354.peg.5553

840	MSHA biogenesis protein MshQ	fig|6666666.59329.peg.4971	fig|6666666.59330.peg.4516	fig|6666666.59331.peg.1328	fig|6666666.59332.peg.3988	fig|6666666.59334.peg.2106	None	fig|6666666.59336.peg.3616	fig|6666666.59333.peg.4747	None	fig|6666666.59339.peg.7787	None	fig|6666666.59337.peg.5507	fig|6666666.59342.peg.4878	None	None	fig|6666666.59345.peg.411	fig|6666666.59341.peg.881	None	fig|6666666.59346.peg.2125	fig|6666666.59348.peg.2477	None	None	None	None	None	None

9939	FIG01202270 hypothetical protein	fig|6666666.59329.peg.2231	None	fig|6666666.59331.peg.1631	fig|6666666.59332.peg.5063	None	fig|6666666.59335.peg.649	fig|6666666.59336.peg.2141	fig|6666666.59333.peg.4617	fig|6666666.59338.peg.2285	fig|6666666.59339.peg.4233	None	fig|6666666.59337.peg.1240	fig|6666666.59342.peg.4899	None	None	fig|6666666.59345.peg.4426	None	fig|6666666.59347.peg.3452	fig|6666666.59346.peg.3857	fig|6666666.59348.peg.3689	None	None	None	None	None	None

3675	FIG01200473 hypothetical protein	fig|6666666.59329.peg.180	fig|6666666.59330.peg.2568	fig|6666666.59331.peg.5314	fig|6666666.59332.peg.1746	None	fig|6666666.59335.peg.1070	fig|6666666.59336.peg.3080	fig|6666666.59333.peg.5064	None	fig|6666666.59339.peg.4513	None	fig|6666666.59337.peg.5386	None	None	None	fig|6666666.59345.peg.2936	fig|6666666.59341.peg.768	fig|6666666.59347.peg.843	fig|6666666.59346.peg.2819	fig|6666666.59348.peg.2394	None	None	None	None	None	None

7176	MSHA biogenesis protein MshI	fig|6666666.59329.peg.3745	fig|6666666.59330.peg.7	None	fig|6666666.59332.peg.503	fig|6666666.59334.peg.732	fig|6666666.59335.peg.200	fig|6666666.59336.peg.5041	fig|6666666.59333.peg.580	None	fig|6666666.59339.peg.7759	None	fig|6666666.59337.peg.793	fig|6666666.59342.peg.5470	None	None	fig|6666666.59345.peg.4903	fig|6666666.59341.peg.3146	None	fig|6666666.59346.peg.1946	fig|6666666.59348.peg.5000	None	None	None	None	None	None

8624	FIG01200120 hypothetical protein	fig|6666666.59329.peg.3844	fig|6666666.59330.peg.3973	fig|6666666.59331.peg.694	fig|6666666.59332.peg.5566	None	fig|6666666.59335.peg.1401	None	fig|6666666.59333.peg.3937	fig|6666666.59338.peg.2792	fig|6666666.59339.peg.6679	None	fig|6666666.59337.peg.5434	None	None	None	fig|6666666.59345.peg.4000	fig|6666666.59341.peg.1488	fig|6666666.59347.peg.3766	fig|6666666.59346.peg.4692	fig|6666666.59348.peg.770	None	None	None	None	None	None

7919	D-Lactate dehydrogenase EC 1.1.2.5	None	fig|6666666.59330.peg.3160	None	fig|6666666.59332.peg.1150	None	fig|6666666.59335.peg.5217	fig|6666666.59336.peg.5439	fig|6666666.59333.peg.4517	None	fig|6666666.59339.peg.643	None	fig|6666666.59337.peg.1174	fig|6666666.59342.peg.3837	fig|6666666.71181.peg.1167	fig|6666666.59344.peg.2294	fig|6666666.59345.peg.3481	None	None	fig|6666666.59346.peg.4293	None	None	None	None	fig|6666666.59353.peg.3416	fig|6666666.59355.peg.3514	None

1313	Flp pilus assembly protein RcpC CpaB	fig|6666666.59329.peg.5142	fig|6666666.59330.peg.660	fig|6666666.59331.peg.2137	fig|6666666.59332.peg.684	None	fig|6666666.59335.peg.1589	fig|6666666.59336.peg.699	None	fig|6666666.59338.peg.4650	None	fig|6666666.59340.peg.2935	fig|6666666.59337.peg.5185	fig|6666666.59342.peg.680	None	None	fig|6666666.59345.peg.2316	fig|6666666.59341.peg.4289	fig|6666666.59347.peg.1932	None	fig|6666666.59348.peg.5285	None	None	None	None	None	None

2560	FIG01200286 hypothetical protein	fig|6666666.59329.peg.1132	fig|6666666.59330.peg.2814	fig|6666666.59331.peg.6268	fig|6666666.59332.peg.2260	None	fig|6666666.59335.peg.3689	fig|6666666.59336.peg.3244	fig|6666666.59333.peg.4499	None	fig|6666666.59339.peg.6869	None	fig|6666666.59337.peg.1359	fig|6666666.59342.peg.4090	None	None	fig|6666666.59345.peg.2284	None	None	fig|6666666.59346.peg.2244	fig|6666666.59348.peg.2556	None	fig|6666666.59350.peg.1669	None	None	None	None

9540	FIG01201453 hypothetical protein	None	fig|6666666.59330.peg.1954	None	fig|6666666.59332.peg.1455	fig|6666666.59334.peg.1297	fig|6666666.59335.peg.5377	fig|6666666.59336.peg.5328	fig|6666666.59333.peg.3635	fig|6666666.59338.peg.3894	fig|6666666.59339.peg.3628	None	fig|6666666.59337.peg.2654	fig|6666666.59342.peg.2404	None	None	fig|6666666.59345.peg.703	fig|6666666.59341.peg.3517	fig|6666666.59347.peg.1692	fig|6666666.59346.peg.4856	None	None	None	None	None	None	None

7702	FIG019175 putative membrane protein	fig|6666666.59329.peg.1659	fig|6666666.59330.peg.1642	None	fig|6666666.59332.peg.1041	fig|6666666.59334.peg.659	None	fig|6666666.59336.peg.943	fig|6666666.59333.peg.1261	fig|6666666.59338.peg.3791	None	fig|6666666.59340.peg.2951	fig|6666666.59337.peg.1531	None	None	None	fig|6666666.59345.peg.3046	None	fig|6666666.59347.peg.1007	fig|6666666.59346.peg.2462	None	fig|6666666.59349.peg.4476	None	None	None	None	fig|6666666.59354.peg.315

11235	FIG01286925 hypothetical protein	fig|6666666.59329.peg.3379	None	fig|6666666.59331.peg.4517	fig|6666666.59332.peg.2970	None	fig|6666666.59335.peg.4188	fig|6666666.59336.peg.6144	None	fig|6666666.59338.peg.1734	fig|6666666.59339.peg.3997	None	fig|6666666.59337.peg.5220	fig|6666666.59342.peg.2710	None	None	fig|6666666.59345.peg.4110	fig|6666666.59341.peg.5949	fig|6666666.59347.peg.1028	None	None	fig|6666666.59349.peg.1635	None	None	None	None	fig|6666666.59354.peg.406

6131	C0398 uncharacterized membrane protein	fig|6666666.59329.peg.2635	fig|6666666.59330.peg.1333	None	fig|6666666.59332.peg.2507	None	fig|6666666.59335.peg.930	fig|6666666.59336.peg.3901	fig|6666666.59333.peg.2545	None	fig|6666666.59339.peg.304	None	fig|6666666.59337.peg.5391	fig|6666666.59342.peg.133	None	None	fig|6666666.59345.peg.1541	None	fig|6666666.59347.peg.2381	fig|6666666.59346.peg.2027	fig|6666666.59348.peg.4508	None	fig|6666666.59350.peg.7326	None	None	None	None

6488	Glycosyltransferase SypN	None	None	fig|6666666.59331.peg.1297	fig|6666666.59332.peg.2396	fig|6666666.59334.peg.5402	fig|6666666.59335.peg.2719	fig|6666666.59336.peg.6107	fig|6666666.59333.peg.3787	fig|6666666.59338.peg.1846	fig|6666666.59339.peg.1154	None	fig|6666666.59337.peg.4432	None	None	None	fig|6666666.59345.peg.3134	fig|6666666.59341.peg.5584	fig|6666666.59347.peg.2338	fig|6666666.59346.peg.5249	fig|6666666.59348.peg.2641	None	None	None	None	None	None

2476	MSHA pilin protein MshD	fig|6666666.59329.peg.598	fig|6666666.59330.peg.20	None	fig|6666666.59332.peg.2047	fig|6666666.59334.peg.4158	fig|6666666.59335.peg.212	fig|6666666.59336.peg.1188	fig|6666666.59333.peg.4387	None	fig|6666666.59339.peg.7778	None	fig|6666666.59337.peg.805	fig|6666666.59342.peg.6140	None	None	fig|6666666.59345.peg.4890	fig|6666666.59341.peg.3665	None	fig|6666666.59346.peg.1960	fig|6666666.59348.peg.2247	None	None	None	None	None	None

4033	putative lipoprotein	fig|6666666.59329.peg.3047	fig|6666666.59330.peg.4624	None	None	None	None	fig|6666666.59336.peg.6347	fig|6666666.59333.peg.1313	fig|6666666.59338.peg.375	None	fig|6666666.59340.peg.2452	fig|6666666.59337.peg.4082	None	None	None	fig|6666666.59345.peg.1678	fig|6666666.59341.peg.2953	fig|6666666.59347.peg.1533	None	None	fig|6666666.59349.peg.2309	fig|6666666.59350.peg.3506	fig|6666666.59351.peg.4015	None	None	fig|6666666.59354.peg.6263

5442	hypothetical protein	None	fig|6666666.59330.peg.2917	fig|6666666.59331.peg.553	fig|6666666.59332.peg.5196	None	fig|6666666.59335.peg.1159	fig|6666666.59336.peg.979	fig|6666666.59333.peg.3027	fig|6666666.59338.peg.2130	None	fig|6666666.59340.peg.3397	fig|6666666.59337.peg.3203	fig|6666666.59342.peg.5630	None	None	fig|6666666.59345.peg.2885	fig|6666666.59341.peg.1465	fig|6666666.59347.peg.2271	fig|6666666.59346.peg.194	None	None	None	None	None	None	None

7682	Lipase-related protein	fig|6666666.59329.peg.6518	fig|6666666.59330.peg.4883	None	fig|6666666.59332.peg.1153	fig|6666666.59334.peg.4983	fig|6666666.59335.peg.2346	fig|6666666.59336.peg.4055	fig|6666666.59333.peg.4787	fig|6666666.59338.peg.2344	fig|6666666.59339.peg.2870	None	fig|6666666.59337.peg.1179	None	None	None	fig|6666666.59345.peg.5706	fig|6666666.59341.peg.3016	fig|6666666.59347.peg.863	fig|6666666.59346.peg.4295	None	None	None	None	None	None	None

1944	Uncharacterized protein ImpA	fig|6666666.59329.peg.6482	fig|6666666.59330.peg.2163	None	None	None	fig|6666666.59335.peg.2685	fig|6666666.59336.peg.3823	fig|6666666.59333.peg.5557	None	fig|6666666.59339.peg.2716	None	fig|6666666.59337.peg.5013	fig|6666666.59342.peg.3107	fig|6666666.71181.peg.294	fig|6666666.59344.peg.1225	fig|6666666.59345.peg.3373	None	None	None	fig|6666666.59348.peg.3042	None	None	None	fig|6666666.59353.peg.1724	fig|6666666.59355.peg.996	None

2850	FIG01203280 hypothetical protein	fig|6666666.59329.peg.5681	fig|6666666.59330.peg.2629	fig|6666666.59331.peg.1249	fig|6666666.59332.peg.5543	None	fig|6666666.59335.peg.4898	fig|6666666.59336.peg.6069	fig|6666666.59333.peg.1408	None	fig|6666666.59339.peg.795	None	fig|6666666.59337.peg.1485	fig|6666666.59342.peg.5154	None	None	fig|6666666.59345.peg.359	fig|6666666.59341.peg.4363	None	fig|6666666.59346.peg.3829	None	None	fig|6666666.59350.peg.389	None	None	None	None

7553	hypothetical protein	fig|6666666.59329.peg.4821	fig|6666666.59330.peg.4663	None	fig|6666666.59332.peg.2788	None	None	fig|6666666.59336.peg.5333	fig|6666666.59333.peg.3940	fig|6666666.59338.peg.1447	None	fig|6666666.59340.peg.2594	fig|6666666.59337.peg.2166	fig|6666666.59342.peg.5492	None	None	fig|6666666.59345.peg.3985	fig|6666666.59341.peg.5504	fig|6666666.59347.peg.3605	fig|6666666.59346.peg.4388	fig|6666666.59348.peg.3638	None	None	None	None	None	None

7557	Putative membrane-bound ClpP-class protease associated with aq 911	fig|6666666.59329.peg.4597	fig|6666666.59330.peg.690	fig|6666666.59331.peg.6053	fig|6666666.59332.peg.5156	fig|6666666.59334.peg.3222	fig|6666666.59335.peg.5884	fig|6666666.59336.peg.4005	fig|6666666.59333.peg.2741	None	fig|6666666.59339.peg.3375	None	fig|6666666.59337.peg.4169	fig|6666666.59342.peg.6039	None	None	fig|6666666.59345.peg.4762	None	None	fig|6666666.59346.peg.3769	fig|6666666.59348.peg.2741	None	None	None	None	None	None

5926	Uncharacterized protein ImpA	fig|6666666.59329.peg.1784	fig|6666666.59330.peg.3110	None	fig|6666666.59332.peg.1094	None	fig|6666666.59335.peg.3350	fig|6666666.59336.peg.5393	fig|6666666.59333.peg.5467	fig|6666666.59338.peg.3588	None	None	fig|6666666.59337.peg.5228	fig|6666666.59342.peg.2907	None	None	fig|6666666.59345.peg.4103	fig|6666666.59341.peg.5193	fig|6666666.59347.peg.2420	None	None	fig|6666666.59349.peg.1630	None	None	None	None	fig|6666666.59354.peg.398

7789	Cellobiose phosphotransferase system YdjC-like protein	fig|6666666.59329.peg.3181	fig|6666666.59330.peg.2434	None	None	None	None	fig|6666666.59336.peg.501	fig|6666666.59333.peg.3571	fig|6666666.59338.peg.901	None	None	fig|6666666.59337.peg.4177	None	fig|6666666.71181.peg.3475	fig|6666666.59344.peg.2174	fig|6666666.59345.peg.4823	fig|6666666.59341.peg.3888	fig|6666666.59347.peg.3707	None	fig|6666666.59348.peg.3192	None	None	None	fig|6666666.59353.peg.226	fig|6666666.59355.peg.2997	None

8612	FIG01199885 hypothetical protein	None	fig|6666666.59330.peg.1089	fig|6666666.59331.peg.1736	fig|6666666.59332.peg.71	None	fig|6666666.59335.peg.4373	fig|6666666.59336.peg.5632	fig|6666666.59333.peg.3967	fig|6666666.59338.peg.5334	fig|6666666.59339.peg.8054	None	fig|6666666.59337.peg.862	None	None	None	fig|6666666.59345.peg.4991	fig|6666666.59341.peg.5758	fig|6666666.59347.peg.4809	fig|6666666.59346.peg.4291	fig|6666666.59348.peg.1213	None	None	None	None	None	None

11332	Conserved hypothetical protein	fig|6666666.59329.peg.1846	fig|6666666.59330.peg.2740	None	fig|6666666.59332.peg.1489	fig|6666666.59334.peg.4655	fig|6666666.59335.peg.1935	None	fig|6666666.59333.peg.3116	fig|6666666.59338.peg.4773	None	None	fig|6666666.59337.peg.4407	fig|6666666.59342.peg.1215	None	None	fig|6666666.59345.peg.3429	fig|6666666.59341.peg.4231	fig|6666666.59347.peg.1433	fig|6666666.59346.peg.3503	fig|6666666.59348.peg.2909	None	None	None	None	None	None

3315	Chemotaxis protein CheV EC 2.7.3.-	None	fig|6666666.59330.peg.4170	fig|6666666.59331.peg.2473	fig|6666666.59332.peg.3362	None	fig|6666666.59335.peg.1065	fig|6666666.59336.peg.5759	None	fig|6666666.59338.peg.376	fig|6666666.59339.peg.4520	None	fig|6666666.59337.peg.2464	fig|6666666.59342.peg.3210	None	None	fig|6666666.59345.peg.589	fig|6666666.59341.peg.2954	fig|6666666.59347.peg.1532	fig|6666666.59346.peg.3910	fig|6666666.59348.peg.917	None	None	None	None	None	None

1029	Metallo-beta-lactamase family protein, RNA-specific	fig|6666666.59329.peg.868	fig|6666666.59330.peg.1335	fig|6666666.59331.peg.2828	fig|6666666.59332.peg.4059	None	None	fig|6666666.59336.peg.1535	fig|6666666.59333.peg.4770	fig|6666666.59338.peg.336	None	fig|6666666.59340.peg.18	fig|6666666.59337.peg.426	fig|6666666.59342.peg.59	None	None	fig|6666666.59345.peg.2564	fig|6666666.59341.peg.2746	fig|6666666.59347.peg.5004	fig|6666666.59346.peg.709	None	None	None	None	None	None	None

5138	Enoyl-[acyl-carrier-protein] reductase [FMN] EC 1.3.1.9	None	None	None	None	None	fig|6666666.59335.peg.5729	None	fig|6666666.59333.peg.2097	fig|6666666.59338.peg.3312	fig|6666666.59339.peg.2370	None	None	None	fig|6666666.71181.peg.1715	fig|6666666.59344.peg.5234	None	fig|6666666.59341.peg.4898	fig|6666666.59347.peg.132	None	None	fig|6666666.59349.peg.4569	fig|6666666.59350.peg.5807	fig|6666666.59351.peg.4591	fig|6666666.59353.peg.3700	fig|6666666.59355.peg.3022	fig|6666666.59354.peg.247

9232	N-acetylmannosaminyltransferase EC 2.4.1.187	None	None	None	None	None	fig|6666666.59335.peg.1614	fig|6666666.59336.peg.1537	None	None	fig|6666666.59339.peg.6560	None	fig|6666666.59337.peg.418	fig|6666666.59342.peg.56	fig|6666666.71181.peg.2442	fig|6666666.59344.peg.2475	None	None	None	fig|6666666.59346.peg.3052	None	fig|6666666.59349.peg.786	fig|6666666.59350.peg.1343	fig|6666666.59351.peg.2646	fig|6666666.59353.peg.3641	fig|6666666.59355.peg.4011	fig|6666666.59354.peg.3013

6679	Zn-ribbon-containing, possibly RNA-binding protein and truncated derivatives	None	fig|6666666.59330.peg.1284	None	None	fig|6666666.59334.peg.3095	None	fig|6666666.59336.peg.958	fig|6666666.59333.peg.4203	None	None	None	fig|6666666.59337.peg.56	None	fig|6666666.71181.peg.1973	fig|6666666.59344.peg.1472	fig|6666666.59345.peg.3786	None	None	None	fig|6666666.59348.peg.255	fig|6666666.59349.peg.2992	None	fig|6666666.59351.peg.217	fig|6666666.59353.peg.145	fig|6666666.59355.peg.755	fig|6666666.59354.peg.4407

2367	FIG01201044 hypothetical protein	fig|6666666.59329.peg.3973	fig|6666666.59330.peg.2717	None	fig|6666666.59332.peg.996	None	fig|6666666.59335.peg.5799	fig|6666666.59336.peg.4896	fig|6666666.59333.peg.4872	None	fig|6666666.59339.peg.422	None	fig|6666666.59337.peg.2448	fig|6666666.59342.peg.3871	None	None	fig|6666666.59345.peg.603	fig|6666666.59341.peg.5510	fig|6666666.59347.peg.637	fig|6666666.59346.peg.3024	fig|6666666.59348.peg.2660	None	None	None	None	None	None

5771	FIG01203160 hypothetical protein	fig|6666666.59329.peg.181	None	fig|6666666.59331.peg.4557	fig|6666666.59332.peg.1747	None	fig|6666666.59335.peg.1069	fig|6666666.59336.peg.3078	fig|6666666.59333.peg.5063	None	fig|6666666.59339.peg.4514	None	fig|6666666.59337.peg.5385	None	fig|6666666.71181.peg.2421	fig|6666666.59344.peg.809	fig|6666666.59345.peg.2935	None	None	fig|6666666.59346.peg.2818	None	None	None	None	fig|6666666.59353.peg.1300	fig|6666666.59355.peg.3281	None

3709	N-acetylglucosamine-regulated outer membrane porin	fig|6666666.59329.peg.6022	fig|6666666.59330.peg.3036	None	fig|6666666.59332.peg.631	None	None	fig|6666666.59336.peg.4672	fig|6666666.59333.peg.4112	None	None	None	fig|6666666.59337.peg.4910	fig|6666666.59342.peg.103	fig|6666666.71181.peg.3804	fig|6666666.59344.peg.5095	fig|6666666.59345.peg.4386	None	fig|6666666.59347.peg.2895	fig|6666666.59346.peg.2375	None	None	None	None	fig|6666666.59353.peg.2051	fig|6666666.59355.peg.2775	None

2125	Aromatic ring-cleaving dioxygenase	fig|6666666.59329.peg.4828	fig|6666666.59330.peg.3844	fig|6666666.59331.peg.1018	fig|6666666.59332.peg.4798	None	fig|6666666.59335.peg.4735	fig|6666666.59336.peg.6516	fig|6666666.59333.peg.3299	None	fig|6666666.59339.peg.3758	None	fig|6666666.59337.peg.4805	fig|6666666.59342.peg.1605	None	None	fig|6666666.59345.peg.316	fig|6666666.59341.peg.2814	None	fig|6666666.59346.peg.2945	fig|6666666.59348.peg.4997	None	None	None	None	None	None

7627	FIG00919622 hypothetical protein	fig|6666666.59329.peg.1350	None	fig|6666666.59331.peg.66	fig|6666666.59332.peg.5671	fig|6666666.59334.peg.3717	fig|6666666.59335.peg.2735	None	fig|6666666.59333.peg.855	fig|6666666.59338.peg.3196	fig|6666666.59339.peg.7199	None	fig|6666666.59337.peg.4102	None	None	None	fig|6666666.59345.peg.4328	fig|6666666.59341.peg.2525	fig|6666666.59347.peg.4379	fig|6666666.59346.peg.2013	fig|6666666.59348.peg.1054	None	None	None	None	None	None

9874	FIG01204839 hypothetical protein	fig|6666666.59329.peg.6415	fig|6666666.59330.peg.4195	None	fig|6666666.59332.peg.2540	None	fig|6666666.59335.peg.638	fig|6666666.59336.peg.4963	fig|6666666.59333.peg.4906	None	fig|6666666.59339.peg.4245	None	fig|6666666.59337.peg.4629	fig|6666666.59342.peg.5594	None	None	fig|6666666.59345.peg.4810	fig|6666666.59341.peg.2088	fig|6666666.59347.peg.575	fig|6666666.59346.peg.484	fig|6666666.59348.peg.1528	None	None	None	None	None	None

2329	Flp pilus assembly membrane protein TadE	fig|6666666.59329.peg.5592	fig|6666666.59330.peg.670	fig|6666666.59331.peg.1975	fig|6666666.59332.peg.694	fig|6666666.59334.peg.3871	fig|6666666.59335.peg.1605	fig|6666666.59336.peg.3213	None	fig|6666666.59338.peg.1793	None	None	fig|6666666.59337.peg.5176	fig|6666666.59342.peg.351	None	None	fig|6666666.59345.peg.2308	fig|6666666.59341.peg.4595	fig|6666666.59347.peg.1924	None	fig|6666666.59348.peg.1139	None	None	None	None	None	None

8773	Phospholipase A1 precursor EC 3.1.1.32, EC 3.1.1.4 ; Outer membrane phospholipase A	None	fig|6666666.59330.peg.1368	None	None	None	None	fig|6666666.59336.peg.2907	fig|6666666.59333.peg.3682	None	None	None	fig|6666666.59337.peg.2910	fig|6666666.59342.peg.4180	fig|6666666.71181.peg.3800	fig|6666666.59344.peg.2499	fig|6666666.59345.peg.4610	fig|6666666.59341.peg.823	None	None	None	fig|6666666.59349.peg.1116	None	fig|6666666.59351.peg.69	fig|6666666.59353.peg.4648	fig|6666666.59355.peg.4765	fig|6666666.59354.peg.310

9255	LfgN	fig|6666666.59329.peg.185	fig|6666666.59330.peg.4951	None	None	None	fig|6666666.59335.peg.892	fig|6666666.59336.peg.6110	fig|6666666.59333.peg.2884	fig|6666666.59338.peg.90	fig|6666666.59339.peg.64	fig|6666666.59340.peg.415	fig|6666666.59337.peg.5381	fig|6666666.59342.peg.3077	None	None	fig|6666666.59345.peg.2931	fig|6666666.59341.peg.3526	fig|6666666.59347.peg.4590	None	None	None	None	None	None	None	None

96	Glycosyltransferase EC 2.4.1.- SypJ	fig|6666666.59329.peg.2535	None	fig|6666666.59331.peg.384	fig|6666666.59332.peg.3464	None	fig|6666666.59335.peg.2725	fig|6666666.59336.peg.3873	fig|6666666.59333.peg.4129	None	fig|6666666.59339.peg.1161	None	fig|6666666.59337.peg.4437	fig|6666666.59342.peg.1293	None	None	fig|6666666.59345.peg.3137	fig|6666666.59341.peg.2642	None	fig|6666666.59346.peg.1740	fig|6666666.59348.peg.4549	None	None	None	None	None	None

3749	FIG01199782 hypothetical protein	fig|6666666.59329.peg.1957	fig|6666666.59330.peg.738	fig|6666666.59331.peg.5602	fig|6666666.59332.peg.3049	None	fig|6666666.59335.peg.4658	fig|6666666.59336.peg.4630	fig|6666666.59333.peg.2249	None	fig|6666666.59339.peg.8389	None	fig|6666666.59337.peg.3759	fig|6666666.59342.peg.5562	None	None	fig|6666666.59345.peg.2785	None	None	fig|6666666.59346.peg.4681	fig|6666666.59348.peg.1219	None	None	None	None	None	None

10513	Iron-regulated protein A precursor	None	fig|6666666.59330.peg.2307	None	fig|6666666.59332.peg.3173	None	fig|6666666.59335.peg.4603	fig|6666666.59336.peg.4387	fig|6666666.59333.peg.3693	None	fig|6666666.59339.peg.3734	None	fig|6666666.59337.peg.1768	fig|6666666.59342.peg.2825	None	None	fig|6666666.59345.peg.876	None	fig|6666666.59347.peg.2246	fig|6666666.59346.peg.4445	fig|6666666.59348.peg.3164	None	fig|6666666.59350.peg.1271	None	None	None	None

10252	NrfD protein	fig|6666666.59329.peg.4231	None	None	fig|6666666.59332.peg.5837	None	fig|6666666.59335.peg.364	fig|6666666.59336.peg.4926	None	fig|6666666.59338.peg.5383	fig|6666666.59339.peg.7127	None	fig|6666666.59337.peg.1762	fig|6666666.59342.peg.2872	None	None	fig|6666666.59345.peg.882	fig|6666666.59341.peg.5274	fig|6666666.59347.peg.1862	fig|6666666.59346.peg.5277	fig|6666666.59348.peg.1554	None	None	None	None	None	None

2728	Hypothetical protein in aerobactin uptake cluster	fig|6666666.59329.peg.2849	None	fig|6666666.59331.peg.2627	fig|6666666.59332.peg.2313	None	fig|6666666.59335.peg.6030	fig|6666666.59336.peg.1902	fig|6666666.59333.peg.3319	fig|6666666.59338.peg.3524	fig|6666666.59339.peg.1801	None	None	None	None	None	fig|6666666.59345.peg.4066	fig|6666666.59341.peg.3485	fig|6666666.59347.peg.3057	fig|6666666.59346.peg.1933	fig|6666666.59348.peg.1349	None	None	None	None	None	None

5264	Exonuclease SbcC	fig|6666666.59329.peg.6442	None	fig|6666666.59331.peg.3109	fig|6666666.59332.peg.6171	fig|6666666.59334.peg.4416	fig|6666666.59335.peg.4497	fig|6666666.59336.peg.5887	fig|6666666.59333.peg.4163	None	fig|6666666.59339.peg.4710	None	None	fig|6666666.59342.peg.4588	None	None	None	fig|6666666.59341.peg.3318	None	fig|6666666.59346.peg.4614	fig|6666666.59348.peg.1926	None	None	None	None	None	fig|6666666.59354.peg.2691

6439	ABC transporter in pyoverdin gene cluster, periplasmic component	fig|6666666.59329.peg.1648	fig|6666666.59330.peg.1650	fig|6666666.59331.peg.3756	fig|6666666.59332.peg.2760	fig|6666666.59334.peg.3248	None	fig|6666666.59336.peg.1387	fig|6666666.59333.peg.1270	fig|6666666.59338.peg.5701	None	None	fig|6666666.59337.peg.1523	None	None	None	fig|6666666.59345.peg.3038	None	fig|6666666.59347.peg.1123	fig|6666666.59346.peg.3783	fig|6666666.59348.peg.3495	None	None	None	None	None	None

1133	Flagellar rod protein flaI	fig|6666666.59329.peg.2523	fig|6666666.59330.peg.4631	None	fig|6666666.59332.peg.2461	None	fig|6666666.59335.peg.381	fig|6666666.59336.peg.1440	None	fig|6666666.59338.peg.4285	fig|6666666.59339.peg.7110	None	fig|6666666.59337.peg.2949	None	None	None	fig|6666666.59345.peg.5353	fig|6666666.59341.peg.5156	fig|6666666.59347.peg.2697	fig|6666666.59346.peg.4632	fig|6666666.59348.peg.2818	None	None	None	None	None	None

7969	ATPase of the AAA+ class	fig|6666666.59329.peg.1429	fig|6666666.59330.peg.2371	fig|6666666.59331.peg.2780	fig|6666666.59332.peg.329	None	fig|6666666.59335.peg.6128	fig|6666666.59336.peg.1738	fig|6666666.59333.peg.2783	None	fig|6666666.59339.peg.7035	None	fig|6666666.59337.peg.2245	None	None	None	fig|6666666.59345.peg.2965	fig|6666666.59341.peg.3215	None	fig|6666666.59346.peg.397	fig|6666666.59348.peg.4677	None	None	None	None	None	None

7930	hypothetical protein	None	fig|6666666.59330.peg.3142	fig|6666666.59331.peg.647	fig|6666666.59332.peg.1593	fig|6666666.59334.peg.4433	fig|6666666.59335.peg.4836	None	None	fig|6666666.59338.peg.505	fig|6666666.59339.peg.3791	fig|6666666.59340.peg.535	fig|6666666.59337.peg.5744	None	fig|6666666.71181.peg.5395	None	None	fig|6666666.59341.peg.3224	fig|6666666.59347.peg.2172	fig|6666666.59346.peg.4299	None	None	None	None	None	None	None

9451	FIG01201888 hypothetical protein	fig|6666666.59329.peg.1684	fig|6666666.59330.peg.4601	None	None	None	fig|6666666.59335.peg.1939	fig|6666666.59336.peg.221	fig|6666666.59333.peg.2757	None	fig|6666666.59339.peg.8415	None	fig|6666666.59337.peg.5032	fig|6666666.59342.peg.853	None	None	fig|6666666.59345.peg.3575	None	None	None	fig|6666666.59348.peg.697	fig|6666666.59349.peg.1175	None	fig|6666666.59351.peg.48	None	None	fig|6666666.59354.peg.4222

6084	lipoprotein, putative	fig|6666666.59329.peg.5500	fig|6666666.59330.peg.764	fig|6666666.59331.peg.2543	fig|6666666.59332.peg.1618	fig|6666666.59334.peg.4702	None	fig|6666666.59336.peg.2626	None	fig|6666666.59338.peg.611	None	None	fig|6666666.59337.peg.5019	fig|6666666.59342.peg.1417	None	None	fig|6666666.59345.peg.40	fig|6666666.59341.peg.1292	fig|6666666.59347.peg.1674	fig|6666666.59346.peg.4217	None	None	None	None	None	None	None

5011	FIG01201545 hypothetical protein	fig|6666666.59329.peg.5808	fig|6666666.59330.peg.3400	None	None	None	fig|6666666.59335.peg.5539	fig|6666666.59336.peg.2971	fig|6666666.59333.peg.2023	fig|6666666.59338.peg.4819	fig|6666666.59339.peg.5682	fig|6666666.59340.peg.590	fig|6666666.59337.peg.3521	fig|6666666.59342.peg.3755	None	None	fig|6666666.59345.peg.940	None	fig|6666666.59347.peg.1376	None	fig|6666666.59348.peg.4303	None	None	None	None	None	None

4788	Serine threonine protein kinase	fig|6666666.59329.peg.636	fig|6666666.59330.peg.206	fig|6666666.59331.peg.1122	fig|6666666.59332.peg.51	None	None	fig|6666666.59336.peg.4908	fig|6666666.59333.peg.1180	None	None	None	fig|6666666.59337.peg.2792	None	fig|6666666.71181.peg.2275	None	fig|6666666.59345.peg.2742	None	fig|6666666.59347.peg.5209	fig|6666666.59346.peg.2814	None	None	None	None	fig|6666666.59353.peg.4896	fig|6666666.59355.peg.5010	None

9536	FIG01200881 hypothetical protein	fig|6666666.59329.peg.879	fig|6666666.59330.peg.1223	None	fig|6666666.59332.peg.1344	None	fig|6666666.59335.peg.2956	fig|6666666.59336.peg.1257	fig|6666666.59333.peg.1092	fig|6666666.59338.peg.2305	fig|6666666.59339.peg.260	None	fig|6666666.59337.peg.3758	None	None	None	fig|6666666.59345.peg.1171	None	fig|6666666.59347.peg.2438	fig|6666666.59346.peg.71	fig|6666666.59348.peg.1975	None	None	None	None	None	None

5412	FIG01200730 hypothetical protein	fig|6666666.59329.peg.3506	fig|6666666.59330.peg.1942	None	None	fig|6666666.59334.peg.4375	fig|6666666.59335.peg.1803	None	fig|6666666.59333.peg.2524	None	fig|6666666.59339.peg.3607	None	fig|6666666.59337.peg.5450	fig|6666666.59342.peg.3512	None	None	fig|6666666.59345.peg.722	fig|6666666.59341.peg.3829	fig|6666666.59347.peg.1684	fig|6666666.59346.peg.3135	fig|6666666.59348.peg.981	None	None	None	None	None	None

9671	hypothetical protein	fig|6666666.59329.peg.4913	fig|6666666.59330.peg.866	fig|6666666.59331.peg.3662	fig|6666666.59332.peg.3561	None	fig|6666666.59335.peg.1473	None	fig|6666666.59333.peg.61	fig|6666666.59338.peg.2338	None	None	fig|6666666.59337.peg.3286	fig|6666666.59342.peg.3549	None	None	fig|6666666.59345.peg.549	fig|6666666.59341.peg.3022	fig|6666666.59347.peg.858	fig|6666666.59346.peg.1872	None	None	None	None	None	None	None

10106	Putative phospholipase C	fig|6666666.59329.peg.4042	fig|6666666.59330.peg.4762	None	None	None	fig|6666666.59335.peg.1936	fig|6666666.59336.peg.2353	fig|6666666.59333.peg.2404	fig|6666666.59338.peg.5621	fig|6666666.59339.peg.8410	None	fig|6666666.59337.peg.5250	None	None	None	fig|6666666.59345.peg.1361	fig|6666666.59341.peg.4680	fig|6666666.59347.peg.85	None	fig|6666666.59348.peg.4111	None	None	fig|6666666.59351.peg.3308	None	None	None

8903	FIG01202679 hypothetical protein	fig|6666666.59329.peg.2586	fig|6666666.59330.peg.2868	fig|6666666.59331.peg.4287	None	fig|6666666.59334.peg.4501	fig|6666666.59335.peg.598	fig|6666666.59336.peg.3105	fig|6666666.59333.peg.5370	None	fig|6666666.59339.peg.485	None	fig|6666666.59337.peg.5577	fig|6666666.59342.peg.538	None	None	fig|6666666.59345.peg.1313	None	None	fig|6666666.59346.peg.1561	fig|6666666.59348.peg.5497	None	None	None	None	None	None

9803	Putative oxidoreductase	fig|6666666.59329.peg.3134	None	fig|6666666.59331.peg.3971	fig|6666666.59332.peg.1256	None	fig|6666666.59335.peg.3277	fig|6666666.59336.peg.1089	fig|6666666.59333.peg.1135	None	fig|6666666.59339.peg.6848	None	fig|6666666.59337.peg.1344	fig|6666666.59342.peg.3165	None	None	fig|6666666.59345.peg.2300	fig|6666666.59341.peg.3325	None	fig|6666666.59346.peg.3259	fig|6666666.59348.peg.3754	None	None	None	None	None	None

3976	FIG01201054 hypothetical protein	None	fig|6666666.59330.peg.2630	fig|6666666.59331.peg.1250	fig|6666666.59332.peg.5545	None	fig|6666666.59335.peg.4899	fig|6666666.59336.peg.4755	fig|6666666.59333.peg.1409	None	fig|6666666.59339.peg.796	None	fig|6666666.59337.peg.1486	fig|6666666.59342.peg.5153	None	None	fig|6666666.59345.peg.358	fig|6666666.59341.peg.4362	None	fig|6666666.59346.peg.3830	fig|6666666.59348.peg.4062	None	None	None	None	None	None

9453	Muramoyltetrapeptide carboxypeptidase EC 3.4.17.13	fig|6666666.59329.peg.1149	fig|6666666.59330.peg.926	fig|6666666.59331.peg.5882	fig|6666666.59332.peg.5621	None	fig|6666666.59335.peg.4321	None	None	fig|6666666.59338.peg.1693	None	None	fig|6666666.59337.peg.3952	None	None	None	fig|6666666.59345.peg.438	None	fig|6666666.59347.peg.172	fig|6666666.59346.peg.3727	None	fig|6666666.59349.peg.2917	None	fig|6666666.59351.peg.4292	None	None	fig|6666666.59354.peg.5177

10119	FIG01199656 hypothetical protein	None	fig|6666666.59330.peg.801	None	fig|6666666.59332.peg.4016	fig|6666666.59334.peg.1007	fig|6666666.59335.peg.562	None	fig|6666666.59333.peg.3893	fig|6666666.59338.peg.4951	fig|6666666.59339.peg.2941	None	fig|6666666.59337.peg.3827	None	None	None	fig|6666666.59345.peg.75	fig|6666666.59341.peg.5357	fig|6666666.59347.peg.931	fig|6666666.59346.peg.1895	fig|6666666.59348.peg.3669	None	None	None	None	None	None

6121	conserved hypothetical membrane protein	fig|6666666.59329.peg.6235	fig|6666666.59330.peg.2859	fig|6666666.59331.peg.4800	fig|6666666.59332.peg.1919	fig|6666666.59334.peg.3343	None	fig|6666666.59336.peg.5009	fig|6666666.59333.peg.4425	None	None	None	fig|6666666.59337.peg.1955	None	None	None	fig|6666666.59345.peg.4059	fig|6666666.59341.peg.1285	fig|6666666.59347.peg.1671	fig|6666666.59346.peg.4230	fig|6666666.59348.peg.2752	None	None	None	None	None	None

4632	Integral membrane protein	None	None	fig|6666666.59331.peg.2424	fig|6666666.59332.peg.1408	None	None	None	None	fig|6666666.59338.peg.4415	None	None	None	fig|6666666.59342.peg.4382	fig|6666666.71181.peg.1300	fig|6666666.59344.peg.3796	None	fig|6666666.59341.peg.5545	fig|6666666.59347.peg.3324	fig|6666666.59346.peg.4599	None	fig|6666666.59349.peg.983	None	None	fig|6666666.59353.peg.3945	fig|6666666.59355.peg.4217	fig|6666666.59354.peg.2718

7089	FIG143263 Glycosyl transferase Lysophospholipid acyltransferase	fig|6666666.59329.peg.2410	None	None	fig|6666666.59332.peg.3635	fig|6666666.59334.peg.3359	None	fig|6666666.59336.peg.1049	fig|6666666.59333.peg.1098	None	None	None	fig|6666666.59337.peg.2264	None	fig|6666666.71181.peg.3933	fig|6666666.59344.peg.5385	None	fig|6666666.59341.peg.5329	None	fig|6666666.59346.peg.2122	fig|6666666.59348.peg.2536	None	None	None	fig|6666666.59353.peg.4790	fig|6666666.59355.peg.4844	None

5482	3',5'-cyclic-nucleotide phosphodiesterase EC 3.1.4.17	fig|6666666.59329.peg.1467	fig|6666666.59330.peg.3368	fig|6666666.59331.peg.6241	fig|6666666.59332.peg.5289	None	fig|6666666.59335.peg.3189	fig|6666666.59336.peg.4873	None	None	fig|6666666.59339.peg.607	None	fig|6666666.59337.peg.4522	None	None	None	fig|6666666.59345.peg.4654	None	None	fig|6666666.59346.peg.4028	fig|6666666.59348.peg.2999	None	fig|6666666.59350.peg.4911	None	None	None	fig|6666666.59354.peg.663

5055	FIG01203067 hypothetical protein	None	None	fig|6666666.59331.peg.5669	fig|6666666.59332.peg.4669	fig|6666666.59334.peg.2780	fig|6666666.59335.peg.4693	fig|6666666.59336.peg.6320	fig|6666666.59333.peg.2368	fig|6666666.59338.peg.4596	fig|6666666.59339.peg.765	fig|6666666.59340.peg.2979	None	fig|6666666.59342.peg.4069	None	None	fig|6666666.59345.peg.774	None	fig|6666666.59347.peg.3402	None	None	None	fig|6666666.59350.peg.7163	None	None	None	None

10977	Iron-sulfur cluster assembly protein SufB	None	None	fig|6666666.59331.peg.4910	None	None	fig|6666666.59335.peg.4383	fig|6666666.59336.peg.199	None	fig|6666666.59338.peg.3860	fig|6666666.59339.peg.2176	None	None	fig|6666666.59342.peg.1844	fig|6666666.71181.peg.2561	fig|6666666.59344.peg.748	fig|6666666.59345.peg.3618	None	fig|6666666.59347.peg.2747	None	None	None	fig|6666666.59350.peg.5432	None	fig|6666666.59353.peg.1355	fig|6666666.59355.peg.2517	None

1972	Uncharacterized iron-regulated protein	fig|6666666.59329.peg.877	fig|6666666.59330.peg.1222	None	fig|6666666.59332.peg.1345	fig|6666666.59334.peg.2898	fig|6666666.59335.peg.2954	fig|6666666.59336.peg.1809	fig|6666666.59333.peg.1091	fig|6666666.59338.peg.1952	fig|6666666.59339.peg.262	None	fig|6666666.59337.peg.3120	None	None	None	fig|6666666.59345.peg.1170	None	fig|6666666.59347.peg.2437	fig|6666666.59346.peg.72	None	None	None	None	None	None	None

2844	hypothetical protein	None	fig|6666666.59330.peg.3906	fig|6666666.59331.peg.6290	None	None	None	fig|6666666.59336.peg.4590	None	fig|6666666.59338.peg.4009	fig|6666666.59339.peg.6080	None	fig|6666666.59337.peg.5557	fig|6666666.59342.peg.3983	None	None	fig|6666666.59345.peg.783	fig|6666666.59341.peg.2919	fig|6666666.59347.peg.730	fig|6666666.59346.peg.4854	None	fig|6666666.59349.peg.1546	None	None	None	None	fig|6666666.59354.peg.3691

4357	Beta-1,4-galactosyltransferase	fig|6666666.59329.peg.833	None	fig|6666666.59331.peg.1927	None	None	None	fig|6666666.59336.peg.67	fig|6666666.59333.peg.471	None	fig|6666666.59339.peg.6303	None	fig|6666666.59337.peg.447	fig|6666666.59342.peg.4337	None	None	fig|6666666.59345.peg.2597	fig|6666666.59341.peg.3699	None	None	None	fig|6666666.59349.peg.450	fig|6666666.59350.peg.477	fig|6666666.59351.peg.2255	None	None	fig|6666666.59354.peg.4634

5934	FIG01200786 hypothetical protein	fig|6666666.59329.peg.1498	fig|6666666.59330.peg.4503	fig|6666666.59331.peg.3881	fig|6666666.59332.peg.405	None	fig|6666666.59335.peg.4421	fig|6666666.59336.peg.758	None	fig|6666666.59338.peg.4918	fig|6666666.59339.peg.5172	None	fig|6666666.59337.peg.1263	None	None	None	fig|6666666.59345.peg.4446	fig|6666666.59341.peg.2851	fig|6666666.59347.peg.2919	fig|6666666.59346.peg.4067	None	None	None	None	None	None	None

607	Exonuclease SbcC	None	fig|6666666.59330.peg.3986	None	None	None	fig|6666666.59335.peg.4496	None	fig|6666666.59333.peg.4164	None	fig|6666666.59339.peg.4709	None	fig|6666666.59337.peg.1152	None	fig|6666666.71181.peg.2749	fig|6666666.59344.peg.1126	fig|6666666.59345.peg.3461	fig|6666666.59341.peg.3319	fig|6666666.59347.peg.765	None	fig|6666666.59348.peg.1924	None	None	None	fig|6666666.59353.peg.802	fig|6666666.59355.peg.904	None

608	FIG01199664 hypothetical protein	fig|6666666.59329.peg.5093	fig|6666666.59330.peg.2324	None	None	None	fig|6666666.59335.peg.5897	fig|6666666.59336.peg.1259	fig|6666666.59333.peg.4051	fig|6666666.59338.peg.4385	None	fig|6666666.59340.peg.2896	fig|6666666.59337.peg.3588	None	None	None	fig|6666666.59345.peg.1881	fig|6666666.59341.peg.3475	fig|6666666.59347.peg.355	None	None	fig|6666666.59349.peg.1549	None	None	None	None	fig|6666666.59354.peg.3688

3115	FIG01200537 hypothetical protein	None	fig|6666666.59330.peg.897	fig|6666666.59331.peg.234	fig|6666666.59332.peg.323	None	fig|6666666.59335.peg.3516	fig|6666666.59336.peg.6373	fig|6666666.59333.peg.4676	fig|6666666.59338.peg.993	fig|6666666.59339.peg.6502	None	fig|6666666.59337.peg.5351	fig|6666666.59342.peg.3815	None	None	fig|6666666.59345.peg.419	None	fig|6666666.59347.peg.1279	None	fig|6666666.59348.peg.5240	None	None	None	None	None	None

7501	Na + H + antiporter subunit C	None	None	fig|6666666.59331.peg.200	None	fig|6666666.59334.peg.5573	fig|6666666.59335.peg.3880	None	fig|6666666.59333.peg.4703	fig|6666666.59338.peg.3059	None	None	None	fig|6666666.59342.peg.6010	None	None	fig|6666666.59345.peg.249	fig|6666666.59341.peg.2678	fig|6666666.59347.peg.2135	None	None	fig|6666666.59349.peg.3897	fig|6666666.59350.peg.87	fig|6666666.59351.peg.1381	None	None	fig|6666666.59354.peg.2463

2585	FIG01200755 hypothetical protein	fig|6666666.59329.peg.658	fig|6666666.59330.peg.1892	fig|6666666.59331.peg.420	fig|6666666.59332.peg.434	fig|6666666.59334.peg.2613	fig|6666666.59335.peg.3142	fig|6666666.59336.peg.783	fig|6666666.59333.peg.3765	None	fig|6666666.59339.peg.5449	None	fig|6666666.59337.peg.240	fig|6666666.59342.peg.1836	None	None	fig|6666666.59345.peg.5261	None	None	None	fig|6666666.59348.peg.3902	None	None	None	None	None	None

10263	Ser-tRNA Ala deacylase; Gly-tRNA Ala deacylase	None	None	None	None	fig|6666666.59334.peg.4311	fig|6666666.59335.peg.4322	None	fig|6666666.59333.peg.3144	None	fig|6666666.59339.peg.1727	None	None	fig|6666666.59342.peg.5111	fig|6666666.71181.peg.1738	fig|6666666.59344.peg.6126	fig|6666666.59345.peg.3106	fig|6666666.59341.peg.5430	None	None	fig|6666666.59348.peg.3700	None	fig|6666666.59350.peg.5137	None	fig|6666666.59353.peg.3675	fig|6666666.59355.peg.5457	None

3548	similar to DBJ BAB55450.1 AB051073 percent identity 45 in 140 aa	None	None	fig|6666666.59331.peg.940	fig|6666666.59332.peg.2816	None	None	None	fig|6666666.59333.peg.3026	None	fig|6666666.59339.peg.5846	None	None	None	fig|6666666.71181.peg.4758	fig|6666666.59344.peg.4482	None	None	fig|6666666.59347.peg.729	fig|6666666.59346.peg.2753	None	fig|6666666.59349.peg.639	None	fig|6666666.59351.peg.1045	fig|6666666.59353.peg.4192	fig|6666666.59355.peg.1681	fig|6666666.59354.peg.2420

2964	Transcriptional regulator, AsnC family	fig|6666666.59329.peg.482	fig|6666666.59330.peg.1618	None	fig|6666666.59332.peg.844	fig|6666666.59334.peg.2572	fig|6666666.59335.peg.3301	fig|6666666.59336.peg.4798	fig|6666666.59333.peg.3190	None	fig|6666666.59339.peg.2406	None	fig|6666666.59337.peg.1287	None	None	None	fig|6666666.59345.peg.4474	None	None	fig|6666666.59346.peg.4275	fig|6666666.59348.peg.4135	None	fig|6666666.59350.peg.5929	None	None	None	None

6597	Protein TadG, associated with Flp pilus assembly	fig|6666666.59329.peg.6204	fig|6666666.59330.peg.672	fig|6666666.59331.peg.4040	fig|6666666.59332.peg.696	fig|6666666.59334.peg.3874	None	fig|6666666.59336.peg.1098	None	fig|6666666.59338.peg.1791	None	None	fig|6666666.59337.peg.5174	fig|6666666.59342.peg.349	None	None	fig|6666666.59345.peg.2306	fig|6666666.59341.peg.5206	fig|6666666.59347.peg.1905	None	fig|6666666.59348.peg.4325	None	None	None	None	None	None

2652	Protein of avirulence locus ImpE	fig|6666666.59329.peg.2960	fig|6666666.59330.peg.3114	None	None	None	None	fig|6666666.59336.peg.4624	fig|6666666.59333.peg.1938	fig|6666666.59338.peg.5505	None	None	fig|6666666.59337.peg.5336	fig|6666666.59342.peg.3253	fig|6666666.71181.peg.2270	fig|6666666.59344.peg.5872	fig|6666666.59345.peg.3449	None	fig|6666666.59347.peg.2416	None	None	None	None	None	fig|6666666.59353.peg.4902	fig|6666666.59355.peg.5016	None

4482	Integrase	fig|6666666.59329.peg.5856	fig|6666666.59330.peg.2426	fig|6666666.59331.peg.214	fig|6666666.59332.peg.3867	None	fig|6666666.59335.peg.4638	None	fig|6666666.59333.peg.2280	None	None	None	fig|6666666.59337.peg.5128	fig|6666666.59342.peg.283	fig|6666666.71181.peg.4830	None	None	fig|6666666.59341.peg.759	fig|6666666.59347.peg.2544	None	None	None	fig|6666666.59350.peg.5426	None	None	fig|6666666.59355.peg.5807	None

8574	FIG01201475 hypothetical protein	None	fig|6666666.59330.peg.691	None	None	None	fig|6666666.59335.peg.4717	fig|6666666.59336.peg.5704	fig|6666666.59333.peg.2740	fig|6666666.59338.peg.2277	fig|6666666.59339.peg.4383	None	fig|6666666.59337.peg.4168	fig|6666666.59342.peg.3124	None	None	fig|6666666.59345.peg.4765	fig|6666666.59341.peg.5743	fig|6666666.59347.peg.878	fig|6666666.59346.peg.3602	fig|6666666.59348.peg.3385	None	None	None	None	None	None

260	Type I restriction-modification system, DNA-methyltransferase subunit M EC 2.1.1.72	fig|6666666.59329.peg.2260	fig|6666666.59330.peg.2503	fig|6666666.59331.peg.961	None	fig|6666666.59334.peg.3974	None	None	None	fig|6666666.59338.peg.1351	fig|6666666.59339.peg.7299	None	fig|6666666.59337.peg.2761	fig|6666666.59342.peg.14	None	None	fig|6666666.59345.peg.5155	None	None	None	None	fig|6666666.59349.peg.2044	fig|6666666.59350.peg.2999	fig|6666666.59351.peg.2445	None	None	fig|6666666.59354.peg.5573

6018	Mannose-1-phosphate guanylyltransferase GDP EC 2.7.7.22	None	None	None	None	None	fig|6666666.59335.peg.415	None	None	fig|6666666.59338.peg.326	fig|6666666.59339.peg.6348	fig|6666666.59340.peg.3180	None	None	fig|6666666.71181.peg.234	fig|6666666.59344.peg.289	None	fig|6666666.59341.peg.2745	fig|6666666.59347.peg.4992	None	fig|6666666.59348.peg.15	None	fig|6666666.59350.peg.3939	fig|6666666.59351.peg.4044	fig|6666666.59353.peg.587	fig|6666666.59355.peg.70	None

2925	Small-conductance mechanosensitive channel	fig|6666666.59329.peg.3591	None	None	fig|6666666.59332.peg.5520	None	fig|6666666.59335.peg.1385	fig|6666666.59336.peg.899	fig|6666666.59333.peg.2486	None	fig|6666666.59339.peg.6748	None	fig|6666666.59337.peg.263	fig|6666666.59342.peg.4172	None	None	fig|6666666.59345.peg.5240	fig|6666666.59341.peg.5796	None	None	None	fig|6666666.59349.peg.2416	None	fig|6666666.59351.peg.394	None	None	fig|6666666.59354.peg.6353

5855	FIG01200001 hypothetical protein	fig|6666666.59329.peg.2223	fig|6666666.59330.peg.3014	fig|6666666.59331.peg.1051	fig|6666666.59332.peg.587	None	fig|6666666.59335.peg.3524	fig|6666666.59336.peg.5993	fig|6666666.59333.peg.5017	None	None	None	fig|6666666.59337.peg.226	None	None	None	fig|6666666.59345.peg.5274	None	None	fig|6666666.59346.peg.152	fig|6666666.59348.peg.5185	fig|6666666.59349.peg.684	None	None	None	None	fig|6666666.59354.peg.5321

8020	Inner membrane protein CreD	fig|6666666.59329.peg.917	None	None	None	None	fig|6666666.59335.peg.4714	fig|6666666.59336.peg.403	fig|6666666.59333.peg.5573	None	fig|6666666.59339.peg.4390	None	fig|6666666.59337.peg.5214	fig|6666666.59342.peg.5464	fig|6666666.71181.peg.3734	fig|6666666.59344.peg.3679	fig|6666666.59345.peg.1833	None	None	None	None	None	None	fig|6666666.59351.peg.2620	fig|6666666.59353.peg.3836	fig|6666666.59355.peg.1551	None

3757	Inosose isomerase EC 5.3.99.-	fig|6666666.59329.peg.3997	None	fig|6666666.59331.peg.267	None	None	fig|6666666.59335.peg.4879	fig|6666666.59336.peg.3421	None	None	fig|6666666.59339.peg.707	fig|6666666.59340.peg.1612	None	fig|6666666.59342.peg.756	fig|6666666.71181.peg.1089	fig|6666666.59344.peg.3591	fig|6666666.59345.peg.1927	fig|6666666.59341.peg.2540	None	None	None	None	None	None	fig|6666666.59353.peg.2151	fig|6666666.59355.peg.1846	None

4812	Putative PRS2 protein	None	fig|6666666.59330.peg.3905	None	None	None	fig|6666666.59335.peg.6100	None	None	fig|6666666.59338.peg.6049	fig|6666666.59339.peg.4185	fig|6666666.59340.peg.3429	fig|6666666.59337.peg.5556	None	None	None	fig|6666666.59345.peg.3111	fig|6666666.59341.peg.314	fig|6666666.59347.peg.3686	None	fig|6666666.59348.peg.2445	fig|6666666.59349.peg.4793	None	fig|6666666.59351.peg.5188	None	None	fig|6666666.59354.peg.1436

7202	FIG01199791 hypothetical protein	fig|6666666.59329.peg.5249	fig|6666666.59330.peg.3796	fig|6666666.59331.peg.1381	fig|6666666.59332.peg.6014	None	None	fig|6666666.59336.peg.5660	fig|6666666.59333.peg.705	fig|6666666.59338.peg.1375	None	None	fig|6666666.59337.peg.3870	fig|6666666.59342.peg.3034	None	None	fig|6666666.59345.peg.298	fig|6666666.59341.peg.1381	fig|6666666.59347.peg.22	fig|6666666.59346.peg.2088	None	None	None	None	None	None	None

1776	Uncharacterized low-complexity protein	fig|6666666.59329.peg.3149	fig|6666666.59330.peg.161	None	fig|6666666.59332.peg.2923	fig|6666666.59334.peg.2350	None	fig|6666666.59336.peg.252	fig|6666666.59333.peg.427	fig|6666666.59338.peg.3735	None	None	fig|6666666.59337.peg.4331	None	None	None	fig|6666666.59345.peg.5113	fig|6666666.59341.peg.176	fig|6666666.59347.peg.4551	fig|6666666.59346.peg.983	fig|6666666.59348.peg.365	None	None	None	None	None	None

9287	FIG01199787 hypothetical protein	fig|6666666.59329.peg.6273	fig|6666666.59330.peg.4704	None	None	None	fig|6666666.59335.peg.5204	fig|6666666.59336.peg.5289	fig|6666666.59333.peg.4432	fig|6666666.59338.peg.3778	None	fig|6666666.59340.peg.3164	fig|6666666.59337.peg.1850	fig|6666666.59342.peg.1166	None	None	fig|6666666.59345.peg.4204	fig|6666666.59341.peg.4591	None	fig|6666666.59346.peg.3477	fig|6666666.59348.peg.3782	None	None	None	None	None	None

5041	Cytochrome c552 precursor EC 1.7.2.2	fig|6666666.59329.peg.5032	None	None	fig|6666666.59332.peg.4715	None	fig|6666666.59335.peg.360	fig|6666666.59336.peg.6404	fig|6666666.59333.peg.4357	fig|6666666.59338.peg.2594	fig|6666666.59339.peg.7132	None	fig|6666666.59337.peg.1759	None	None	None	fig|6666666.59345.peg.885	fig|6666666.59341.peg.5145	fig|6666666.59347.peg.1865	fig|6666666.59346.peg.1341	fig|6666666.59348.peg.1551	None	None	None	None	None	None

8696	FIG01202308 hypothetical protein	fig|6666666.59329.peg.3977	fig|6666666.59330.peg.4706	fig|6666666.59331.peg.4446	fig|6666666.59332.peg.2808	None	None	fig|6666666.59336.peg.5959	fig|6666666.59333.peg.4430	fig|6666666.59338.peg.842	None	None	fig|6666666.59337.peg.1852	fig|6666666.59342.peg.1168	None	None	fig|6666666.59345.peg.4202	None	fig|6666666.59347.peg.460	fig|6666666.59346.peg.415	fig|6666666.59348.peg.4300	None	None	None	None	None	None

10190	putative SpoOM-related protein	fig|6666666.59329.peg.5159	fig|6666666.59330.peg.513	fig|6666666.59331.peg.106	fig|6666666.59332.peg.3281	None	fig|6666666.59335.peg.5226	fig|6666666.59336.peg.2090	fig|6666666.59333.peg.3581	None	fig|6666666.59339.peg.3880	None	fig|6666666.59337.peg.3301	None	None	None	fig|6666666.59345.peg.4632	None	None	None	None	fig|6666666.59349.peg.3317	None	fig|6666666.59351.peg.628	None	None	fig|6666666.59354.peg.3986

10443	Putative sulfite oxidase subunit YedY	None	fig|6666666.59330.peg.3138	None	None	None	fig|6666666.59335.peg.5306	fig|6666666.59336.peg.6147	None	fig|6666666.59338.peg.2914	fig|6666666.59339.peg.1011	None	fig|6666666.59337.peg.5742	None	fig|6666666.71181.peg.975	fig|6666666.59344.peg.5333	fig|6666666.59345.peg.4167	None	fig|6666666.59347.peg.70	None	None	None	fig|6666666.59350.peg.3370	None	fig|6666666.59353.peg.2289	fig|6666666.59355.peg.4814	None

5259	Ribose 5-phosphate isomerase B EC 5.3.1.6	None	None	None	None	fig|6666666.59334.peg.3471	None	fig|6666666.59336.peg.1748	None	fig|6666666.59338.peg.2021	None	None	None	fig|6666666.59342.peg.2761	fig|6666666.71181.peg.3963	fig|6666666.59344.peg.6097	fig|6666666.59345.peg.2971	fig|6666666.59341.peg.639	fig|6666666.59347.peg.1305	None	fig|6666666.59348.peg.1879	None	fig|6666666.59350.peg.7998	None	fig|6666666.59353.peg.2960	fig|6666666.59355.peg.2781	None

929	Transcriptional regulator, MerR family, associated with photolyase	fig|6666666.59329.peg.1963	fig|6666666.59330.peg.742	None	None	None	fig|6666666.59335.peg.1928	fig|6666666.59336.peg.2761	fig|6666666.59333.peg.2426	fig|6666666.59338.peg.1482	fig|6666666.59339.peg.8398	None	fig|6666666.59337.peg.3471	fig|6666666.59342.peg.5884	None	None	fig|6666666.59345.peg.2781	fig|6666666.59341.peg.2259	fig|6666666.59347.peg.438	None	fig|6666666.59348.peg.2762	None	None	None	None	None	None

7159	Na + H + antiporter subunit G	None	None	None	None	fig|6666666.59334.peg.5148	fig|6666666.59335.peg.4442	fig|6666666.59336.peg.2696	fig|6666666.59333.peg.4708	fig|6666666.59338.peg.4611	None	fig|6666666.59340.peg.1952	None	None	None	None	fig|6666666.59345.peg.245	fig|6666666.59341.peg.2681	fig|6666666.59347.peg.2131	None	None	fig|6666666.59349.peg.3893	fig|6666666.59350.peg.83	fig|6666666.59351.peg.1377	None	None	fig|6666666.59354.peg.2458

1459	LafD	fig|6666666.59329.peg.4705	fig|6666666.59330.peg.2907	None	None	None	fig|6666666.59335.peg.917	fig|6666666.59336.peg.989	fig|6666666.59333.peg.4084	fig|6666666.59338.peg.2384	fig|6666666.59339.peg.92	fig|6666666.59340.peg.3156	fig|6666666.59337.peg.3214	fig|6666666.59342.peg.5251	None	None	fig|6666666.59345.peg.2876	fig|6666666.59341.peg.124	fig|6666666.59347.peg.4613	None	None	None	None	None	None	None	None

7172	GGDEF family protein	fig|6666666.59329.peg.3088	fig|6666666.59330.peg.737	fig|6666666.59331.peg.2340	fig|6666666.59332.peg.3048	None	fig|6666666.59335.peg.4659	fig|6666666.59336.peg.4628	fig|6666666.59333.peg.2248	None	fig|6666666.59339.peg.8388	None	fig|6666666.59337.peg.3760	fig|6666666.59342.peg.1879	None	None	fig|6666666.59345.peg.2787	None	None	fig|6666666.59346.peg.4683	fig|6666666.59348.peg.1220	None	None	None	None	None	None

10905	MSHA biogenesis protein MshO	fig|6666666.59329.peg.597	fig|6666666.59330.peg.21	None	fig|6666666.59332.peg.2048	fig|6666666.59334.peg.2109	fig|6666666.59335.peg.213	fig|6666666.59336.peg.1189	fig|6666666.59333.peg.4388	None	fig|6666666.59339.peg.7780	None	fig|6666666.59337.peg.806	None	None	None	fig|6666666.59345.peg.4889	fig|6666666.59341.peg.3666	None	fig|6666666.59346.peg.1961	fig|6666666.59348.peg.2246	None	None	None	None	None	None

4533	LysR-family transcriptional regulator clustered with PA0057	None	None	fig|6666666.59331.peg.5843	fig|6666666.59332.peg.1838	None	fig|6666666.59335.peg.4877	None	fig|6666666.59333.peg.3756	None	fig|6666666.59339.peg.1254	None	None	fig|6666666.59342.peg.5245	fig|6666666.71181.peg.95	fig|6666666.59344.peg.3167	None	fig|6666666.59341.peg.1966	None	fig|6666666.59346.peg.500	fig|6666666.59348.peg.4722	None	None	None	fig|6666666.59353.peg.1918	fig|6666666.59355.peg.1489	None

10622	FIG01201575 hypothetical protein	fig|6666666.59329.peg.1654	fig|6666666.59330.peg.1646	fig|6666666.59331.peg.2202	fig|6666666.59332.peg.1045	fig|6666666.59334.peg.3982	None	fig|6666666.59336.peg.1393	fig|6666666.59333.peg.1265	None	None	fig|6666666.59340.peg.3699	fig|6666666.59337.peg.1527	None	None	None	fig|6666666.59345.peg.3042	None	fig|6666666.59347.peg.1119	fig|6666666.59346.peg.3787	fig|6666666.59348.peg.5320	None	None	None	None	None	None

3050	Putative membrane protein precursor	fig|6666666.59329.peg.1660	fig|6666666.59330.peg.1641	None	None	None	fig|6666666.59335.peg.3964	fig|6666666.59336.peg.942	None	None	fig|6666666.59339.peg.3137	None	fig|6666666.59337.peg.1532	None	fig|6666666.71181.peg.2507	fig|6666666.59344.peg.4067	fig|6666666.59345.peg.3047	None	fig|6666666.59347.peg.3751	None	None	None	fig|6666666.59350.peg.7670	None	fig|6666666.59353.peg.2116	fig|6666666.59355.peg.2705	None

8	FIG01200249 hypothetical protein	None	fig|6666666.59330.peg.2961	None	None	None	fig|6666666.59335.peg.3995	None	fig|6666666.59333.peg.3326	None	fig|6666666.59339.peg.867	None	fig|6666666.59337.peg.3442	fig|6666666.59342.peg.5416	fig|6666666.71181.peg.2519	fig|6666666.59344.peg.4084	fig|6666666.59345.peg.193	None	None	None	fig|6666666.59348.peg.2183	fig|6666666.59349.peg.4802	None	None	fig|6666666.59353.peg.2129	fig|6666666.59355.peg.2691	None

10010	Potassium channel protein	fig|6666666.59329.peg.5870	fig|6666666.59330.peg.2342	fig|6666666.59331.peg.523	None	fig|6666666.59334.peg.4145	None	fig|6666666.59336.peg.2443	fig|6666666.59333.peg.3387	fig|6666666.59338.peg.5747	None	None	fig|6666666.59337.peg.5027	fig|6666666.59342.peg.1990	None	None	fig|6666666.59345.peg.32	fig|6666666.59341.peg.3025	fig|6666666.59347.peg.2119	fig|6666666.59346.peg.3762	None	None	None	None	None	None	None

3948	FIG01200233 hypothetical protein	fig|6666666.59329.peg.3891	fig|6666666.59330.peg.477	None	None	fig|6666666.59334.peg.1031	fig|6666666.59335.peg.3904	fig|6666666.59336.peg.6504	fig|6666666.59333.peg.2233	None	fig|6666666.59339.peg.7180	None	fig|6666666.59337.peg.1573	None	None	None	fig|6666666.59345.peg.2209	None	None	fig|6666666.59346.peg.5001	None	fig|6666666.59349.peg.1350	None	fig|6666666.59351.peg.5485	None	None	fig|6666666.59354.peg.2627

4206	XdhC protein assists in molybdopterin insertion into xanthine dehydrogenase	None	None	None	fig|6666666.59332.peg.1624	None	fig|6666666.59335.peg.1154	None	None	fig|6666666.59338.peg.4309	fig|6666666.59339.peg.1671	None	None	None	fig|6666666.71181.peg.1856	fig|6666666.59344.peg.4329	None	fig|6666666.59341.peg.4780	fig|6666666.59347.peg.3321	fig|6666666.59346.peg.3505	fig|6666666.59348.peg.3585	None	fig|6666666.59350.peg.7875	None	fig|6666666.59353.peg.4153	fig|6666666.59355.peg.3103	None

3420	Urate oxidase EC 1.7.3.3	None	None	fig|6666666.59331.peg.6171	fig|6666666.59332.peg.2251	None	fig|6666666.59335.peg.1141	None	None	fig|6666666.59338.peg.5026	fig|6666666.59339.peg.1648	None	None	None	fig|6666666.71181.peg.901	fig|6666666.59344.peg.5189	None	fig|6666666.59341.peg.4530	fig|6666666.59347.peg.3329	fig|6666666.59346.peg.4746	fig|6666666.59348.peg.3209	None	None	None	fig|6666666.59353.peg.918	fig|6666666.59355.peg.789	None

585	FIG00919855 hypothetical protein	fig|6666666.59329.peg.998	fig|6666666.59330.peg.3952	None	fig|6666666.59332.peg.3906	None	fig|6666666.59335.peg.3967	fig|6666666.59336.peg.5173	fig|6666666.59333.peg.1541	fig|6666666.59338.peg.3865	fig|6666666.59339.peg.157	None	fig|6666666.59337.peg.3846	None	None	None	fig|6666666.59345.peg.1082	fig|6666666.59341.peg.2388	fig|6666666.59347.peg.2622	fig|6666666.59346.peg.501	None	None	None	None	None	None	None

1358	FIG01200784 hypothetical protein	fig|6666666.59329.peg.6064	fig|6666666.59330.peg.2119	None	fig|6666666.59332.peg.4382	None	None	None	fig|6666666.59333.peg.99	fig|6666666.59338.peg.2273	fig|6666666.59339.peg.2812	None	fig|6666666.59337.peg.4585	fig|6666666.59342.peg.2317	None	None	fig|6666666.59345.peg.508	fig|6666666.59341.peg.5602	fig|6666666.59347.peg.881	fig|6666666.59346.peg.3307	fig|6666666.59348.peg.828	None	None	None	None	None	None

8544	unknown	fig|6666666.59329.peg.5169	fig|6666666.59330.peg.2913	None	None	None	fig|6666666.59335.peg.911	fig|6666666.59336.peg.982	fig|6666666.59333.peg.3030	fig|6666666.59338.peg.1250	fig|6666666.59339.peg.84	fig|6666666.59340.peg.2285	fig|6666666.59337.peg.3208	fig|6666666.59342.peg.3233	None	None	fig|6666666.59345.peg.2882	fig|6666666.59341.peg.116	fig|6666666.59347.peg.4606	None	None	None	None	None	None	None	None

9418	5-deoxy-glucuronate isomerase EC 5.3.1.-	fig|6666666.59329.peg.4001	None	None	None	None	fig|6666666.59335.peg.5935	fig|6666666.59336.peg.3418	None	None	fig|6666666.59339.peg.702	fig|6666666.59340.peg.1611	None	fig|6666666.59342.peg.753	fig|6666666.71181.peg.1094	fig|6666666.59344.peg.3596	fig|6666666.59345.peg.1930	fig|6666666.59341.peg.2541	None	None	None	None	fig|6666666.59350.peg.2184	None	fig|6666666.59353.peg.2155	fig|6666666.59355.peg.1850	None

9615	FIG01201386 hypothetical protein	fig|6666666.59329.peg.4145	fig|6666666.59330.peg.1176	fig|6666666.59331.peg.3996	fig|6666666.59332.peg.722	None	None	fig|6666666.59336.peg.3347	fig|6666666.59333.peg.667	fig|6666666.59338.peg.1184	None	None	fig|6666666.59337.peg.485	None	None	None	fig|6666666.59345.peg.2635	fig|6666666.59341.peg.5034	fig|6666666.59347.peg.2126	fig|6666666.59346.peg.1388	fig|6666666.59348.peg.126	None	None	None	None	None	None

8440	Catalyzes the cleavage of p-aminobenzoyl-glutamate to p-aminobenzoate and glutamate, subunit A	None	None	None	None	None	fig|6666666.59335.peg.1897	None	None	fig|6666666.59338.peg.5766	fig|6666666.59339.peg.4748	None	None	fig|6666666.59342.peg.2171	fig|6666666.71181.peg.1408	fig|6666666.59344.peg.505	None	fig|6666666.59341.peg.2118	fig|6666666.59347.peg.3548	None	None	fig|6666666.59349.peg.1672	fig|6666666.59350.peg.1784	fig|6666666.59351.peg.2662	fig|6666666.59353.peg.3903	fig|6666666.59355.peg.3744	None

7393	Protein phosphatase ImpM	fig|6666666.59329.peg.1787	fig|6666666.59330.peg.4166	None	None	None	None	fig|6666666.59336.peg.5511	fig|6666666.59333.peg.3052	fig|6666666.59338.peg.5972	None	None	fig|6666666.59337.peg.4779	fig|6666666.59342.peg.1253	fig|6666666.71181.peg.2266	fig|6666666.59344.peg.2989	fig|6666666.59345.peg.3443	None	fig|6666666.59347.peg.2422	None	None	None	None	None	fig|6666666.59353.peg.3562	fig|6666666.59355.peg.3611	None

9136	Arginine ABC transporter, ATP-binding protein ArtP	fig|6666666.59329.peg.741	fig|6666666.59330.peg.3066	None	fig|6666666.59332.peg.4532	None	None	None	fig|6666666.59333.peg.1415	None	None	None	fig|6666666.59337.peg.5373	fig|6666666.59342.peg.1738	fig|6666666.71181.peg.3909	fig|6666666.59344.peg.373	fig|6666666.59345.peg.347	fig|6666666.59341.peg.2077	None	fig|6666666.59346.peg.3179	None	None	None	None	fig|6666666.59353.peg.519	fig|6666666.59355.peg.4	None

1003	FIG01200898 hypothetical protein	fig|6666666.59329.peg.6034	None	fig|6666666.59331.peg.3964	fig|6666666.59332.peg.2763	fig|6666666.59334.peg.2455	fig|6666666.59335.peg.4992	fig|6666666.59336.peg.4731	fig|6666666.59333.peg.3316	fig|6666666.59338.peg.2337	fig|6666666.59339.peg.4696	None	None	fig|6666666.59342.peg.1436	None	None	fig|6666666.59345.peg.14	None	fig|6666666.59347.peg.857	fig|6666666.59346.peg.3782	None	None	None	None	None	None	None

4345	FIG019993 putative exported protein	fig|6666666.59329.peg.1658	fig|6666666.59330.peg.1643	fig|6666666.59331.peg.5043	fig|6666666.59332.peg.1042	fig|6666666.59334.peg.4866	None	fig|6666666.59336.peg.944	fig|6666666.59333.peg.1262	fig|6666666.59338.peg.3792	None	fig|6666666.59340.peg.2953	fig|6666666.59337.peg.1530	None	None	None	fig|6666666.59345.peg.3045	None	fig|6666666.59347.peg.1116	fig|6666666.59346.peg.2460	None	None	None	None	None	None	None

9275	Potassium uptake protein, integral membrane component, KtrB	fig|6666666.59329.peg.5058	fig|6666666.59330.peg.281	fig|6666666.59331.peg.1409	fig|6666666.59332.peg.4182	None	None	fig|6666666.59336.peg.5988	fig|6666666.59333.peg.2913	fig|6666666.59338.peg.3135	None	fig|6666666.59340.peg.2930	fig|6666666.59337.peg.1011	None	None	None	fig|6666666.59345.peg.4269	fig|6666666.59341.peg.2159	fig|6666666.59347.peg.840	fig|6666666.59346.peg.1057	None	None	None	None	None	None	None

7371	FIG01200257 hypothetical protein	None	fig|6666666.59330.peg.4777	None	fig|6666666.59332.peg.5308	fig|6666666.59334.peg.1359	fig|6666666.59335.peg.2359	None	fig|6666666.59333.peg.3779	None	fig|6666666.59339.peg.2886	None	fig|6666666.59337.peg.4959	fig|6666666.59342.peg.546	None	None	fig|6666666.59345.peg.120	fig|6666666.59341.peg.1759	fig|6666666.59347.peg.756	fig|6666666.59346.peg.2922	fig|6666666.59348.peg.2914	None	None	None	None	None	None

6383	FIG01200141 hypothetical protein	fig|6666666.59329.peg.468	fig|6666666.59330.peg.1828	None	None	fig|6666666.59334.peg.5211	fig|6666666.59335.peg.879	fig|6666666.59336.peg.1948	fig|6666666.59333.peg.2351	fig|6666666.59338.peg.2056	None	None	fig|6666666.59337.peg.1064	fig|6666666.59342.peg.4449	None	None	fig|6666666.59345.peg.1653	None	fig|6666666.59347.peg.2551	fig|6666666.59346.peg.2087	fig|6666666.59348.peg.1048	None	None	None	None	None	None

617	Probable aspartoacylase EC 3.5.1.15	fig|6666666.59329.peg.4305	fig|6666666.59330.peg.2347	None	fig|6666666.59332.peg.4748	None	fig|6666666.59335.peg.5329	fig|6666666.59336.peg.2803	fig|6666666.59333.peg.3498	fig|6666666.59338.peg.1942	fig|6666666.59339.peg.6780	None	fig|6666666.59337.peg.3613	fig|6666666.59342.peg.4608	None	None	fig|6666666.59345.peg.1846	fig|6666666.59341.peg.2882	None	fig|6666666.59346.peg.2147	None	None	None	None	None	None	None

3334	hypothetical protein	fig|6666666.59329.peg.2323	fig|6666666.59330.peg.4950	None	None	None	fig|6666666.59335.peg.893	fig|6666666.59336.peg.6111	fig|6666666.59333.peg.2885	fig|6666666.59338.peg.91	fig|6666666.59339.peg.65	fig|6666666.59340.peg.414	fig|6666666.59337.peg.5380	fig|6666666.59342.peg.3076	None	None	fig|6666666.59345.peg.2930	fig|6666666.59341.peg.3527	fig|6666666.59347.peg.4591	None	None	None	None	None	None	None	None

5698	transcriptional regulator, LuxR family	fig|6666666.59329.peg.3046	None	None	None	None	None	None	fig|6666666.59333.peg.1314	fig|6666666.59338.peg.4282	None	None	fig|6666666.59337.peg.4083	fig|6666666.59342.peg.5600	fig|6666666.71181.peg.4258	fig|6666666.59344.peg.5702	fig|6666666.59345.peg.1679	fig|6666666.59341.peg.5612	fig|6666666.59347.peg.5126	None	None	None	None	fig|6666666.59351.peg.4559	fig|6666666.59353.peg.2505	fig|6666666.59355.peg.596	None

5961	FIG01202576 hypothetical protein	fig|6666666.59329.peg.1198	fig|6666666.59330.peg.2410	None	fig|6666666.59332.peg.6267	None	fig|6666666.59335.peg.5203	None	fig|6666666.59333.peg.2026	None	fig|6666666.59339.peg.810	None	fig|6666666.59337.peg.1510	None	fig|6666666.71181.peg.1874	fig|6666666.59344.peg.4351	fig|6666666.59345.peg.3022	None	None	fig|6666666.59346.peg.1918	None	None	None	None	fig|6666666.59353.peg.3240	fig|6666666.59355.peg.3123	None

9852	Protein ydhR precursor	fig|6666666.59329.peg.2778	None	None	fig|6666666.59332.peg.3014	None	fig|6666666.59335.peg.3126	fig|6666666.59336.peg.1108	fig|6666666.59333.peg.4653	fig|6666666.59338.peg.4775	None	fig|6666666.59340.peg.3111	None	None	None	None	fig|6666666.59345.peg.1892	fig|6666666.59341.peg.4322	fig|6666666.59347.peg.1431	fig|6666666.59346.peg.1032	fig|6666666.59348.peg.1811	None	fig|6666666.59350.peg.4789	None	None	None	None

712	Anaerobic nitric oxide reductase flavorubredoxin	fig|6666666.59329.peg.584	None	fig|6666666.59331.peg.1799	fig|6666666.59332.peg.3070	fig|6666666.59334.peg.4359	None	fig|6666666.59336.peg.5760	fig|6666666.59333.peg.5044	fig|6666666.59338.peg.5844	None	fig|6666666.59340.peg.1125	None	fig|6666666.59342.peg.1810	None	None	fig|6666666.59345.peg.659	fig|6666666.59341.peg.5628	fig|6666666.59347.peg.450	None	fig|6666666.59348.peg.3555	None	None	None	None	None	None

9683	FIG01201672 hypothetical protein	fig|6666666.59329.peg.1640	fig|6666666.59330.peg.3009	None	fig|6666666.59332.peg.582	None	None	fig|6666666.59336.peg.314	fig|6666666.59333.peg.4624	fig|6666666.59338.peg.2126	fig|6666666.59339.peg.4059	None	fig|6666666.59337.peg.221	fig|6666666.59342.peg.5099	None	None	fig|6666666.59345.peg.5278	None	fig|6666666.59347.peg.2276	fig|6666666.59346.peg.159	None	fig|6666666.59349.peg.1041	None	None	None	None	None

8802	FIG01200772 hypothetical protein	None	fig|6666666.59330.peg.3671	None	None	fig|6666666.59334.peg.2959	fig|6666666.59335.peg.2557	fig|6666666.59336.peg.5774	fig|6666666.59333.peg.337	None	fig|6666666.59339.peg.6903	fig|6666666.59340.peg.252	fig|6666666.59337.peg.698	fig|6666666.59342.peg.4190	None	None	fig|6666666.59345.peg.3689	fig|6666666.59341.peg.6004	fig|6666666.59347.peg.4254	None	fig|6666666.59348.peg.4237	None	None	None	None	None	None

11155	Xanthine dehydrogenase, molybdenum binding subunit EC 1.17.1.4	None	None	None	fig|6666666.59332.peg.1625	None	fig|6666666.59335.peg.1155	None	None	fig|6666666.59338.peg.4308	fig|6666666.59339.peg.1672	None	None	None	fig|6666666.71181.peg.1854	fig|6666666.59344.peg.5867	None	fig|6666666.59341.peg.4779	fig|6666666.59347.peg.3320	fig|6666666.59346.peg.3504	fig|6666666.59348.peg.498	None	fig|6666666.59350.peg.4219	None	fig|6666666.59353.peg.3219	fig|6666666.59355.peg.4263	None

7123	Metallo-beta-lactamase superfamily protein PA0057	fig|6666666.59329.peg.2120	None	fig|6666666.59331.peg.4709	fig|6666666.59332.peg.1839	None	fig|6666666.59335.peg.4820	fig|6666666.59336.peg.718	fig|6666666.59333.peg.3755	None	fig|6666666.59339.peg.1253	None	None	fig|6666666.59342.peg.2383	fig|6666666.71181.peg.96	fig|6666666.59344.peg.3166	None	None	None	None	fig|6666666.59348.peg.4645	None	None	None	fig|6666666.59353.peg.1917	fig|6666666.59355.peg.5947	None

6002	Benzoate transport protein	None	None	fig|6666666.59331.peg.2727	fig|6666666.59332.peg.5447	fig|6666666.59334.peg.2501	None	None	None	fig|6666666.59338.peg.1682	fig|6666666.59339.peg.1390	fig|6666666.59340.peg.354	None	None	fig|6666666.71181.peg.2914	fig|6666666.59344.peg.3473	None	fig|6666666.59341.peg.3982	fig|6666666.59347.peg.4701	None	None	None	fig|6666666.59350.peg.55	None	fig|6666666.59353.peg.1406	fig|6666666.59355.peg.2468	None

9253	FIG01202703 hypothetical protein	None	fig|6666666.59330.peg.3175	fig|6666666.59331.peg.356	fig|6666666.59332.peg.4687	None	fig|6666666.59335.peg.1217	fig|6666666.59336.peg.5640	None	None	fig|6666666.59339.peg.1499	None	fig|6666666.59337.peg.4565	None	None	None	fig|6666666.59345.peg.3243	None	None	fig|6666666.59346.peg.5102	None	fig|6666666.59349.peg.3806	None	fig|6666666.59351.peg.3872	None	None	fig|6666666.59354.peg.5689

1769	hypothetical protein	None	None	None	fig|6666666.59332.peg.5454	None	None	None	None	None	fig|6666666.59339.peg.6000	None	None	fig|6666666.59342.peg.3688	fig|6666666.71181.peg.3309	fig|6666666.59344.peg.4851	None	fig|6666666.59341.peg.933	None	fig|6666666.59346.peg.956	None	fig|6666666.59349.peg.880	None	fig|6666666.59351.peg.5087	fig|6666666.59353.peg.3476	fig|6666666.59355.peg.3246	fig|6666666.59354.peg.3878

5056	FIG027190 Putative transmembrane protein	fig|6666666.59329.peg.2406	None	fig|6666666.59331.peg.2353	fig|6666666.59332.peg.4951	None	None	fig|6666666.59336.peg.1045	fig|6666666.59333.peg.1101	None	None	None	None	None	fig|6666666.71181.peg.3931	fig|6666666.59344.peg.5388	None	fig|6666666.59341.peg.5738	None	fig|6666666.59346.peg.2117	fig|6666666.59348.peg.1821	None	None	None	fig|6666666.59353.peg.4788	fig|6666666.59355.peg.4842	None

9173	protein of unknown function DUF358	fig|6666666.59329.peg.4028	fig|6666666.59330.peg.4446	fig|6666666.59331.peg.2896	fig|6666666.59332.peg.1537	None	None	fig|6666666.59336.peg.5210	fig|6666666.59333.peg.4515	None	None	None	fig|6666666.59337.peg.1212	fig|6666666.59342.peg.5231	None	None	fig|6666666.59345.peg.4398	fig|6666666.59341.peg.2683	None	fig|6666666.59346.peg.3670	fig|6666666.59348.peg.5455	None	None	None	None	None	None

3000	hypothetical protein	fig|6666666.59329.peg.5849	fig|6666666.59330.peg.2069	fig|6666666.59331.peg.168	fig|6666666.59332.peg.5697	fig|6666666.59334.peg.3710	None	fig|6666666.59336.peg.1910	None	None	None	None	fig|6666666.59337.peg.3560	fig|6666666.59342.peg.741	None	None	fig|6666666.59345.peg.836	fig|6666666.59341.peg.4759	fig|6666666.59347.peg.1681	fig|6666666.59346.peg.2584	None	None	None	None	None	None	None

3641	FIG01199928 hypothetical protein	fig|6666666.59329.peg.4540	fig|6666666.59330.peg.4745	None	fig|6666666.59332.peg.1249	None	fig|6666666.59335.peg.3887	None	fig|6666666.59333.peg.1653	None	fig|6666666.59339.peg.2432	None	fig|6666666.59337.peg.3108	fig|6666666.59342.peg.4852	None	None	fig|6666666.59345.peg.4140	fig|6666666.59341.peg.1760	None	fig|6666666.59346.peg.2614	fig|6666666.59348.peg.3033	None	None	None	None	None	None

2554	FIG01200499 hypothetical protein	fig|6666666.59329.peg.5209	fig|6666666.59330.peg.1227	None	None	None	fig|6666666.59335.peg.2749	fig|6666666.59336.peg.1254	None	None	fig|6666666.59339.peg.258	None	fig|6666666.59337.peg.3753	fig|6666666.59342.peg.5666	None	None	fig|6666666.59345.peg.1173	None	None	None	None	fig|6666666.59349.peg.298	None	fig|6666666.59351.peg.308	None	fig|6666666.59355.peg.5241	fig|6666666.59354.peg.2477

4416	Long-chain fatty acid transport protein	fig|6666666.59329.peg.4819	fig|6666666.59330.peg.4665	fig|6666666.59331.peg.1828	fig|6666666.59332.peg.3535	fig|6666666.59334.peg.4211	None	None	fig|6666666.59333.peg.3944	None	fig|6666666.59339.peg.3402	None	fig|6666666.59337.peg.2164	fig|6666666.59342.peg.6041	None	None	fig|6666666.59345.peg.3982	fig|6666666.59341.peg.856	None	fig|6666666.59346.peg.4371	None	None	None	None	None	None	None

4436	FIG01202107 hypothetical protein	fig|6666666.59329.peg.2950	fig|6666666.59330.peg.3267	fig|6666666.59331.peg.3786	fig|6666666.59332.peg.5487	None	fig|6666666.59335.peg.2815	fig|6666666.59336.peg.2135	None	None	fig|6666666.59339.peg.3609	None	fig|6666666.59337.peg.1235	fig|6666666.59342.peg.5596	None	None	fig|6666666.59345.peg.4420	fig|6666666.59341.peg.2036	None	fig|6666666.59346.peg.3142	None	None	None	None	None	None	None

1668	FIG01201543 hypothetical protein	fig|6666666.59329.peg.4111	fig|6666666.59330.peg.1516	fig|6666666.59331.peg.1165	fig|6666666.59332.peg.4704	None	fig|6666666.59335.peg.5997	fig|6666666.59336.peg.2601	fig|6666666.59333.peg.4966	None	fig|6666666.59339.peg.353	None	fig|6666666.59337.peg.2303	None	None	None	fig|6666666.59345.peg.1294	None	None	fig|6666666.59346.peg.4358	fig|6666666.59348.peg.2304	None	None	None	None	None	None

2826	Mesenchymal stem cell protein DSCD75	None	None	None	None	None	None	None	None	fig|6666666.59338.peg.3212	None	fig|6666666.59340.peg.82	None	None	fig|6666666.71181.peg.3363	fig|6666666.59344.peg.5448	None	fig|6666666.59341.peg.802	fig|6666666.59347.peg.3737	None	None	fig|6666666.59349.peg.1414	fig|6666666.59350.peg.624	fig|6666666.59351.peg.1404	fig|6666666.59353.peg.4460	fig|6666666.59355.peg.4567	fig|6666666.59354.peg.2311

3195	hypothetical protein	fig|6666666.59329.peg.1425	None	fig|6666666.59331.peg.4741	fig|6666666.59332.peg.1907	fig|6666666.59334.peg.1003	fig|6666666.59335.peg.3473	fig|6666666.59336.peg.2838	None	fig|6666666.59338.peg.656	fig|6666666.59339.peg.2115	None	None	None	None	None	fig|6666666.59345.peg.2974	fig|6666666.59341.peg.932	fig|6666666.59347.peg.2519	None	fig|6666666.59348.peg.3075	None	None	None	None	None	None

11412	NTP pyrophosphohydrolase including oxidative damage repair enzymes	fig|6666666.59329.peg.5073	fig|6666666.59330.peg.2731	fig|6666666.59331.peg.2824	fig|6666666.59332.peg.886	None	None	fig|6666666.59336.peg.4735	fig|6666666.59333.peg.4550	None	None	None	fig|6666666.59337.peg.4394	fig|6666666.59342.peg.3519	None	None	fig|6666666.59345.peg.3412	None	None	fig|6666666.59346.peg.3853	None	fig|6666666.59349.peg.4510	None	None	None	None	fig|6666666.59354.peg.6402

2460	hypothetical protein	None	None	fig|6666666.59331.peg.2807	fig|6666666.59332.peg.6011	fig|6666666.59334.peg.834	None	fig|6666666.59336.peg.2705	None	fig|6666666.59338.peg.4589	None	fig|6666666.59340.peg.3658	fig|6666666.59337.peg.4088	fig|6666666.59342.peg.4759	None	None	fig|6666666.59345.peg.1688	fig|6666666.59341.peg.3644	fig|6666666.59347.peg.3687	fig|6666666.59346.peg.3329	None	None	None	None	None	None	None

5432	FIG01199584 hypothetical protein	None	fig|6666666.59330.peg.3187	fig|6666666.59331.peg.1447	fig|6666666.59332.peg.5112	None	fig|6666666.59335.peg.3543	None	None	fig|6666666.59338.peg.5194	fig|6666666.59339.peg.1329	None	fig|6666666.59337.peg.4569	fig|6666666.59342.peg.586	None	None	None	fig|6666666.59341.peg.5952	fig|6666666.59347.peg.1746	fig|6666666.59346.peg.1537	fig|6666666.59348.peg.2000	None	None	None	None	None	None

5885	HPT domain containing protein	fig|6666666.59329.peg.1852	fig|6666666.59330.peg.4142	fig|6666666.59331.peg.1650	None	fig|6666666.59334.peg.1066	None	fig|6666666.59336.peg.5243	None	fig|6666666.59338.peg.4943	None	None	fig|6666666.59337.peg.4769	fig|6666666.59342.peg.1223	None	None	fig|6666666.59345.peg.3434	fig|6666666.59341.peg.2098	fig|6666666.59347.peg.2154	fig|6666666.59346.peg.2849	None	None	None	None	None	None	None

6499	FIG01201525 hypothetical protein	fig|6666666.59329.peg.3516	fig|6666666.59330.peg.4308	None	fig|6666666.59332.peg.2434	fig|6666666.59334.peg.4191	None	fig|6666666.59336.peg.5698	fig|6666666.59333.peg.2556	fig|6666666.59338.peg.5474	None	None	None	fig|6666666.59342.peg.950	None	None	fig|6666666.59345.peg.63	fig|6666666.59341.peg.2229	fig|6666666.59347.peg.923	fig|6666666.59346.peg.1227	None	None	None	None	None	None	None

10143	FIG01202023 hypothetical protein	fig|6666666.59329.peg.2010	fig|6666666.59330.peg.3182	None	None	fig|6666666.59334.peg.5197	fig|6666666.59335.peg.3674	fig|6666666.59336.peg.821	fig|6666666.59333.peg.3858	None	fig|6666666.59339.peg.2660	None	fig|6666666.59337.peg.5748	fig|6666666.59342.peg.2237	None	None	fig|6666666.59345.peg.3300	fig|6666666.59341.peg.5826	fig|6666666.59347.peg.972	None	None	None	None	None	None	None	None

2862	Cytochrome c-type protein NrfB precursor	fig|6666666.59329.peg.5031	None	None	None	None	fig|6666666.59335.peg.362	fig|6666666.59336.peg.4924	fig|6666666.59333.peg.4359	fig|6666666.59338.peg.2597	fig|6666666.59339.peg.7129	None	fig|6666666.59337.peg.1760	fig|6666666.59342.peg.2874	None	None	fig|6666666.59345.peg.884	fig|6666666.59341.peg.5277	fig|6666666.59347.peg.1864	None	fig|6666666.59348.peg.1552	None	None	None	None	None	None

370	hypothetical protein	fig|6666666.59329.peg.4171	fig|6666666.59330.peg.891	fig|6666666.59331.peg.2895	None	None	fig|6666666.59335.peg.769	fig|6666666.59336.peg.1859	fig|6666666.59333.peg.1820	fig|6666666.59338.peg.3571	fig|6666666.59339.peg.5581	None	fig|6666666.59337.peg.2479	None	None	None	fig|6666666.59345.peg.574	None	fig|6666666.59347.peg.598	None	fig|6666666.59348.peg.2652	None	None	None	None	None	None

8458	Virulence factor VirK	None	fig|6666666.59330.peg.2554	None	None	None	fig|6666666.59335.peg.4241	fig|6666666.59336.peg.2146	fig|6666666.59333.peg.2872	None	fig|6666666.59339.peg.1030	None	fig|6666666.59337.peg.2256	fig|6666666.59342.peg.5432	fig|6666666.71181.peg.4133	None	fig|6666666.59345.peg.2949	None	None	None	fig|6666666.59348.peg.2714	None	None	None	fig|6666666.59353.peg.1615	fig|6666666.59355.peg.1106	None

1480	Ascorbate-specific PTS system, EIIA component EC 2.7.1.-	fig|6666666.59329.peg.2156	None	fig|6666666.59331.peg.1413	fig|6666666.59332.peg.2908	None	None	fig|6666666.59336.peg.3185	None	None	None	None	None	fig|6666666.59342.peg.1767	fig|6666666.71181.peg.2692	fig|6666666.59344.peg.3856	fig|6666666.59345.peg.4535	fig|6666666.59341.peg.4119	None	fig|6666666.59346.peg.2344	None	None	None	None	fig|6666666.59353.peg.3986	fig|6666666.59355.peg.3902	None

1485	Tricarboxylate transport protein TctB	None	fig|6666666.59330.peg.4108	fig|6666666.59331.peg.1170	None	fig|6666666.59334.peg.2668	fig|6666666.59335.peg.2024	None	fig|6666666.59333.peg.3044	None	None	None	fig|6666666.59337.peg.3116	fig|6666666.59342.peg.2645	None	None	fig|6666666.59345.peg.4127	fig|6666666.59341.peg.4872	fig|6666666.59347.peg.1613	None	fig|6666666.59348.peg.3362	None	fig|6666666.59350.peg.2217	None	None	None	None

8479	probable binding protein component of ABC transporter	fig|6666666.59329.peg.6053	None	None	None	None	fig|6666666.59335.peg.5356	fig|6666666.59336.peg.5064	fig|6666666.59333.peg.462	fig|6666666.59338.peg.4637	fig|6666666.59339.peg.8779	None	None	fig|6666666.59342.peg.3599	None	None	fig|6666666.59345.peg.5598	fig|6666666.59341.peg.2400	fig|6666666.59347.peg.139	None	None	None	fig|6666666.59350.peg.185	None	None	fig|6666666.59355.peg.5258	None

11321	Secreted and surface protein containing fasciclin-like repeats	fig|6666666.59329.peg.4682	fig|6666666.59330.peg.2237	None	None	fig|6666666.59334.peg.1703	None	fig|6666666.59336.peg.4147	fig|6666666.59333.peg.4048	fig|6666666.59338.peg.4027	None	None	fig|6666666.59337.peg.5046	None	fig|6666666.71181.peg.4651	None	fig|6666666.59345.peg.1883	fig|6666666.59341.peg.1830	fig|6666666.59347.peg.3594	None	None	None	fig|6666666.59350.peg.2202	None	None	None	None

4146	Rhs-family protein	fig|6666666.59329.peg.3535	fig|6666666.59330.peg.3982	None	None	None	fig|6666666.59335.peg.3787	fig|6666666.59336.peg.5666	fig|6666666.59333.peg.3224	None	None	None	fig|6666666.59337.peg.2815	fig|6666666.59342.peg.4994	fig|6666666.71181.peg.5143	fig|6666666.59344.peg.676	fig|6666666.59345.peg.3457	None	None	None	None	None	None	None	fig|6666666.59353.peg.4500	fig|6666666.59355.peg.4609	None

10730	Twin-arginine translocation pathway signal	fig|6666666.59329.peg.5509	None	None	fig|6666666.59332.peg.1096	None	fig|6666666.59335.peg.3348	fig|6666666.59336.peg.6533	None	fig|6666666.59338.peg.3592	fig|6666666.59339.peg.4008	None	None	fig|6666666.59342.peg.4809	None	None	fig|6666666.59345.peg.4102	fig|6666666.59341.peg.5194	fig|6666666.59347.peg.1036	None	None	fig|6666666.59349.peg.1647	None	None	None	None	fig|6666666.59354.peg.752

7344	FIG01201749 hypothetical protein	fig|6666666.59329.peg.4187	fig|6666666.59330.peg.2977	fig|6666666.59331.peg.2705	fig|6666666.59332.peg.5608	None	fig|6666666.59335.peg.5527	fig|6666666.59336.peg.812	fig|6666666.59333.peg.2199	None	fig|6666666.59339.peg.2827	None	fig|6666666.59337.peg.5156	fig|6666666.59342.peg.666	None	None	fig|6666666.59345.peg.3293	None	None	fig|6666666.59346.peg.3166	None	None	None	None	None	None	None

10022	Nitroreductase family protein	fig|6666666.59329.peg.5431	fig|6666666.59330.peg.156	None	None	None	fig|6666666.59335.peg.438	fig|6666666.59336.peg.258	None	None	fig|6666666.59339.peg.6394	None	fig|6666666.59337.peg.5187	fig|6666666.59342.peg.2307	None	None	fig|6666666.59345.peg.5108	None	None	None	None	fig|6666666.59349.peg.3966	fig|6666666.59350.peg.3540	fig|6666666.59351.peg.4201	None	None	fig|6666666.59354.peg.3369

4315	membrane protein	fig|6666666.59329.peg.3648	fig|6666666.59330.peg.1263	None	None	None	fig|6666666.59335.peg.2821	fig|6666666.59336.peg.467	fig|6666666.59333.peg.1843	None	None	None	fig|6666666.59337.peg.2207	fig|6666666.59342.peg.1092	None	None	fig|6666666.59345.peg.1582	None	None	None	fig|6666666.59348.peg.2041	fig|6666666.59349.peg.2948	None	fig|6666666.59351.peg.2825	None	None	fig|6666666.59354.peg.5604

5267	2'-5' RNA ligase	None	fig|6666666.59330.peg.3750	None	None	fig|6666666.59334.peg.1207	None	fig|6666666.59336.peg.1753	None	None	None	None	fig|6666666.59337.peg.1836	None	fig|6666666.71181.peg.2836	fig|6666666.59344.peg.4444	fig|6666666.59345.peg.4217	None	None	None	None	fig|6666666.59349.peg.1459	None	fig|6666666.59351.peg.2475	fig|6666666.59353.peg.3187	fig|6666666.59355.peg.3069	fig|6666666.59354.peg.4730

662	FIG01202705 hypothetical protein	fig|6666666.59329.peg.1044	fig|6666666.59330.peg.2795	None	fig|6666666.59332.peg.121	None	None	fig|6666666.59336.peg.3466	fig|6666666.59333.peg.1929	None	None	fig|6666666.59340.peg.842	fig|6666666.59337.peg.2371	fig|6666666.59342.peg.3540	None	None	fig|6666666.59345.peg.2247	fig|6666666.59341.peg.2069	fig|6666666.59347.peg.2357	fig|6666666.59346.peg.1152	None	None	None	None	None	None	None

8760	Citrate 6-N-acetyl-6-N-hydroxy-L-lysine ligase, alpha subunit EC 6.3.2.27 , aerobactin biosynthesis protein IucA Siderophore synthetase superfamily, group C Siderophore synthetase component, ligase	fig|6666666.59329.peg.4123	None	fig|6666666.59331.peg.779	None	None	fig|6666666.59335.peg.2647	fig|6666666.59336.peg.6140	fig|6666666.59333.peg.1127	None	fig|6666666.59339.peg.1815	fig|6666666.59340.peg.676	None	None	fig|6666666.71181.peg.886	fig|6666666.59344.peg.4765	None	None	None	fig|6666666.59346.peg.5013	None	None	None	None	fig|6666666.59353.peg.4547	fig|6666666.59355.peg.4650	None

9045	Urease accessory protein UreG	fig|6666666.59329.peg.2204	None	fig|6666666.59331.peg.1777	fig|6666666.59332.peg.3042	None	fig|6666666.59335.peg.4467	None	None	fig|6666666.59338.peg.3562	fig|6666666.59339.peg.5352	None	None	None	None	None	fig|6666666.59345.peg.4541	fig|6666666.59341.peg.4509	fig|6666666.59347.peg.572	fig|6666666.59346.peg.3218	fig|6666666.59348.peg.1495	None	fig|6666666.59350.peg.4192	None	None	None	None

1075	Putative metal chaperone, involved in Zn homeostasis, GTPase of C0523 family	None	None	None	fig|6666666.59332.peg.2591	None	None	fig|6666666.59336.peg.5161	fig|6666666.59333.peg.964	fig|6666666.59338.peg.565	None	None	None	fig|6666666.59342.peg.6095	fig|6666666.71181.peg.1391	fig|6666666.59344.peg.529	fig|6666666.59345.peg.220	None	None	None	fig|6666666.59348.peg.1901	None	fig|6666666.59350.peg.6980	None	fig|6666666.59353.peg.1837	fig|6666666.59355.peg.1177	None

9468	Serine threonine protein kinase	fig|6666666.59329.peg.3874	fig|6666666.59330.peg.5022	fig|6666666.59331.peg.115	fig|6666666.59332.peg.4776	None	fig|6666666.59335.peg.3129	fig|6666666.59336.peg.1928	None	None	fig|6666666.59339.peg.5422	None	fig|6666666.59337.peg.2408	fig|6666666.59342.peg.5526	None	None	fig|6666666.59345.peg.953	None	None	fig|6666666.59346.peg.4677	fig|6666666.59348.peg.4264	None	None	None	None	None	None

6599	Outer membrane protein OmpT	None	fig|6666666.59330.peg.1312	None	fig|6666666.59332.peg.4313	fig|6666666.59334.peg.1452	fig|6666666.59335.peg.6101	fig|6666666.59336.peg.855	fig|6666666.59333.peg.1016	fig|6666666.59338.peg.769	fig|6666666.59339.peg.376	None	None	None	None	None	fig|6666666.59345.peg.1560	fig|6666666.59341.peg.4584	fig|6666666.59347.peg.2361	fig|6666666.59346.peg.678	None	None	None	None	None	None	None

9848	Glutaredoxin 2	fig|6666666.59329.peg.3805	None	fig|6666666.59331.peg.230	fig|6666666.59332.peg.2786	None	None	None	fig|6666666.59333.peg.1745	None	None	None	None	None	fig|6666666.71181.peg.1899	fig|6666666.59344.peg.2544	fig|6666666.59345.peg.3897	fig|6666666.59341.peg.622	fig|6666666.59347.peg.3408	fig|6666666.59346.peg.388	None	None	None	None	fig|6666666.59353.peg.1512	fig|6666666.59355.peg.2062	None

3153	hypothetical protein	None	None	fig|6666666.59331.peg.1589	None	None	fig|6666666.59335.peg.6033	None	None	fig|6666666.59338.peg.5417	fig|6666666.59339.peg.991	fig|6666666.59340.peg.549	fig|6666666.59337.peg.166	None	None	None	None	None	fig|6666666.59347.peg.192	fig|6666666.59346.peg.2977	fig|6666666.59348.peg.5356	fig|6666666.59349.peg.3113	None	fig|6666666.59351.peg.4236	None	None	fig|6666666.59354.peg.1640

126	ADP-ribosylglycohydrolase YegU EC 3.2.-.-	None	None	fig|6666666.59331.peg.445	fig|6666666.59332.peg.4381	None	None	fig|6666666.59336.peg.5225	None	None	fig|6666666.59339.peg.4575	None	None	None	fig|6666666.71181.peg.6195	fig|6666666.59344.peg.149	None	fig|6666666.59341.peg.4727	None	fig|6666666.59346.peg.1268	fig|6666666.59348.peg.866	None	None	fig|6666666.59351.peg.737	fig|6666666.59353.peg.462	fig|6666666.59355.peg.2189	None

3178	hypothetical protein	None	fig|6666666.59330.peg.4662	None	None	None	fig|6666666.59335.peg.1905	fig|6666666.59336.peg.3323	fig|6666666.59333.peg.4025	fig|6666666.59338.peg.410	fig|6666666.59339.peg.3069	None	fig|6666666.59337.peg.917	fig|6666666.59342.peg.2087	None	None	fig|6666666.59345.peg.3584	fig|6666666.59341.peg.5045	fig|6666666.59347.peg.423	None	fig|6666666.59348.peg.4553	None	None	None	None	None	None

8008	PAS factor	fig|6666666.59329.peg.3954	fig|6666666.59330.peg.4854	fig|6666666.59331.peg.2787	fig|6666666.59332.peg.6242	None	None	fig|6666666.59336.peg.3906	fig|6666666.59333.peg.1815	fig|6666666.59338.peg.4876	None	None	fig|6666666.59337.peg.2584	None	None	None	fig|6666666.59345.peg.782	fig|6666666.59341.peg.935	fig|6666666.59347.peg.1156	fig|6666666.59346.peg.4591	None	None	None	None	None	None	None

7670	Mu-like prophage protein gp16	fig|6666666.59329.peg.391	fig|6666666.59330.peg.3344	None	fig|6666666.59332.peg.6108	None	fig|6666666.59335.peg.3837	fig|6666666.59336.peg.4863	fig|6666666.59333.peg.1920	None	fig|6666666.59339.peg.561	None	fig|6666666.59337.peg.3506	fig|6666666.59342.peg.1724	None	None	fig|6666666.59345.peg.924	None	None	fig|6666666.59346.peg.4961	fig|6666666.59348.peg.371	None	None	None	None	None	None

8499	Glutaredoxin-like protein NrdH, required for reduction of Ribonucleotide reductase class Ib	None	None	None	fig|6666666.59332.peg.2071	fig|6666666.59334.peg.2022	fig|6666666.59335.peg.4381	None	None	fig|6666666.59338.peg.3862	fig|6666666.59339.peg.2174	None	None	None	fig|6666666.71181.peg.2619	fig|6666666.59344.peg.3885	None	None	fig|6666666.59347.peg.2749	None	fig|6666666.59348.peg.3683	None	fig|6666666.59350.peg.5089	None	fig|6666666.59353.peg.4692	fig|6666666.59355.peg.4074	None

6789	dTDP-4-dehydrorhamnose 3,5-epimerase EC 5.1.3.13	None	None	None	None	fig|6666666.59334.peg.3136	fig|6666666.59335.peg.504	None	None	fig|6666666.59338.peg.328	fig|6666666.59339.peg.6323	None	None	None	fig|6666666.71181.peg.221	fig|6666666.59344.peg.271	None	None	fig|6666666.59347.peg.5009	None	fig|6666666.59348.peg.882	None	fig|6666666.59350.peg.6787	fig|6666666.59351.peg.240	fig|6666666.59353.peg.601	fig|6666666.59355.peg.83	None

1140	FIG01200213 hypothetical protein	fig|6666666.59329.peg.2886	fig|6666666.59330.peg.2901	None	None	None	fig|6666666.59335.peg.922	fig|6666666.59336.peg.4344	fig|6666666.59333.peg.2862	None	fig|6666666.59339.peg.100	fig|6666666.59340.peg.1102	fig|6666666.59337.peg.3219	fig|6666666.59342.peg.3199	None	None	fig|6666666.59345.peg.2871	fig|6666666.59341.peg.130	fig|6666666.59347.peg.4617	None	None	None	None	None	None	None	None

385	FIG01200623 hypothetical protein	fig|6666666.59329.peg.5297	fig|6666666.59330.peg.3673	None	None	None	None	fig|6666666.59336.peg.5776	fig|6666666.59333.peg.339	fig|6666666.59338.peg.1865	None	fig|6666666.59340.peg.251	fig|6666666.59337.peg.700	fig|6666666.59342.peg.4188	None	None	fig|6666666.59345.peg.3687	fig|6666666.59341.peg.6003	fig|6666666.59347.peg.4255	fig|6666666.59346.peg.2549	None	None	None	None	None	None	None

7208	Malate dehydrogenase EC 1.1.1.37	None	None	fig|6666666.59331.peg.739	None	None	None	None	None	fig|6666666.59338.peg.5118	None	fig|6666666.59340.peg.1099	None	None	fig|6666666.71181.peg.1261	fig|6666666.59344.peg.1276	None	fig|6666666.59341.peg.3210	fig|6666666.59347.peg.3825	fig|6666666.59346.peg.4539	None	None	fig|6666666.59350.peg.391	fig|6666666.59351.peg.2895	fig|6666666.59353.peg.456	fig|6666666.59355.peg.4172	None

800	Potassium uptake protein, integral membrane component, KtrA	None	fig|6666666.59330.peg.280	fig|6666666.59331.peg.1406	fig|6666666.59332.peg.4180	fig|6666666.59334.peg.2348	None	None	fig|6666666.59333.peg.2912	fig|6666666.59338.peg.3137	None	fig|6666666.59340.peg.2839	fig|6666666.59337.peg.1010	None	None	None	fig|6666666.59345.peg.4270	fig|6666666.59341.peg.2158	fig|6666666.59347.peg.839	fig|6666666.59346.peg.1055	None	None	None	None	None	None	None

1127	FIG01200680 hypothetical protein	fig|6666666.59329.peg.2392	fig|6666666.59330.peg.2104	None	None	fig|6666666.59334.peg.1101	None	fig|6666666.59336.peg.2000	fig|6666666.59333.peg.1441	fig|6666666.59338.peg.878	None	None	fig|6666666.59337.peg.3525	fig|6666666.59342.peg.2583	None	None	fig|6666666.59345.peg.493	fig|6666666.59341.peg.3402	fig|6666666.59347.peg.5141	None	fig|6666666.59348.peg.604	None	None	None	None	None	None

2712	hypothetical protein	fig|6666666.59329.peg.3356	fig|6666666.59330.peg.2362	None	None	None	fig|6666666.59335.peg.1213	fig|6666666.59336.peg.1126	fig|6666666.59333.peg.3081	fig|6666666.59338.peg.4917	fig|6666666.59339.peg.1496	None	fig|6666666.59337.peg.2232	None	None	None	fig|6666666.59345.peg.2984	fig|6666666.59341.peg.5527	fig|6666666.59347.peg.2041	None	fig|6666666.59348.peg.1126	None	None	None	None	None	None

10704	FIG01203174 hypothetical protein	fig|6666666.59329.peg.3715	None	fig|6666666.59331.peg.4049	fig|6666666.59332.peg.5839	None	fig|6666666.59335.peg.5462	fig|6666666.59336.peg.3429	fig|6666666.59333.peg.952	None	fig|6666666.59339.peg.955	None	fig|6666666.59337.peg.2600	fig|6666666.59342.peg.4495	None	None	fig|6666666.59345.peg.755	None	None	fig|6666666.59346.peg.3159	fig|6666666.59348.peg.4548	None	None	None	None	None	None

8725	MotA TolQ ExbB proton channel family protein	fig|6666666.59329.peg.6216	None	fig|6666666.59331.peg.1002	fig|6666666.59332.peg.1147	None	fig|6666666.59335.peg.4861	None	None	None	fig|6666666.59339.peg.6263	None	None	fig|6666666.59342.peg.3433	None	fig|6666666.59344.peg.5271	None	None	None	fig|6666666.59346.peg.4622	fig|6666666.59348.peg.120	None	None	fig|6666666.59351.peg.3339	fig|6666666.59353.peg.4702	fig|6666666.59355.peg.4788	None

3903	FIG01200563 hypothetical protein	fig|6666666.59329.peg.1546	fig|6666666.59330.peg.2208	fig|6666666.59331.peg.6250	None	fig|6666666.59334.peg.3393	fig|6666666.59335.peg.3234	fig|6666666.59336.peg.2536	fig|6666666.59333.peg.2859	None	fig|6666666.59339.peg.2452	fig|6666666.59340.peg.308	fig|6666666.59337.peg.1896	None	None	None	fig|6666666.59345.peg.2839	None	None	fig|6666666.59346.peg.3642	None	None	None	None	None	None	None

926	DUF819 domain-containing protein	None	fig|6666666.59330.peg.2955	None	fig|6666666.59332.peg.454	None	None	fig|6666666.59336.peg.4562	None	fig|6666666.59338.peg.3992	None	None	fig|6666666.59337.peg.3450	None	None	None	fig|6666666.59345.peg.187	fig|6666666.59341.peg.5166	fig|6666666.59347.peg.3315	fig|6666666.59346.peg.2483	None	fig|6666666.59349.peg.2620	None	fig|6666666.59351.peg.4657	None	None	fig|6666666.59354.peg.2791

7978	Acyl carrier protein ACP2	fig|6666666.59329.peg.3340	None	fig|6666666.59331.peg.3733	fig|6666666.59332.peg.5792	None	None	fig|6666666.59336.peg.1053	fig|6666666.59333.peg.1095	None	None	None	None	None	fig|6666666.71181.peg.3937	fig|6666666.59344.peg.5381	None	fig|6666666.59341.peg.2756	None	fig|6666666.59346.peg.4720	fig|6666666.59348.peg.779	None	None	None	fig|6666666.59353.peg.4794	fig|6666666.59355.peg.4848	None

9938	Methyl-accepting chemotaxis protein	fig|6666666.59329.peg.4737	fig|6666666.59330.peg.4545	None	None	None	None	fig|6666666.59336.peg.6350	fig|6666666.59333.peg.2461	fig|6666666.59338.peg.3263	None	fig|6666666.59340.peg.1079	fig|6666666.59337.peg.3147	fig|6666666.59342.peg.935	None	None	fig|6666666.59345.peg.1145	fig|6666666.59341.peg.1127	fig|6666666.59347.peg.2572	None	fig|6666666.59348.peg.4291	None	None	None	None	None	None

3587	FIG01202704 hypothetical protein	fig|6666666.59329.peg.6153	fig|6666666.59330.peg.3780	fig|6666666.59331.peg.1357	None	None	fig|6666666.59335.peg.4707	fig|6666666.59336.peg.4408	None	fig|6666666.59338.peg.1362	fig|6666666.59339.peg.4395	None	fig|6666666.59337.peg.2217	fig|6666666.59342.peg.5608	None	None	fig|6666666.59345.peg.3001	fig|6666666.59341.peg.1744	fig|6666666.59347.peg.5425	None	None	None	None	None	None	None	None

8931	MbtH-like protein	None	None	None	fig|6666666.59332.peg.1203	None	fig|6666666.59335.peg.3162	None	fig|6666666.59333.peg.5453	None	fig|6666666.59339.peg.1049	None	None	None	fig|6666666.71181.peg.767	fig|6666666.59344.peg.4895	None	None	None	None	fig|6666666.59348.peg.4762	fig|6666666.59349.peg.909	None	fig|6666666.59351.peg.11	fig|6666666.59353.peg.1733	fig|6666666.59355.peg.2446	fig|6666666.59354.peg.3470

11251	Flagellar protein FliJ	fig|6666666.59329.peg.1714	fig|6666666.59330.peg.2215	None	None	None	fig|6666666.59335.peg.2392	fig|6666666.59336.peg.479	fig|6666666.59333.peg.2850	fig|6666666.59338.peg.2983	fig|6666666.59339.peg.122	None	fig|6666666.59337.peg.1887	fig|6666666.59342.peg.5912	None	None	fig|6666666.59345.peg.2856	fig|6666666.59341.peg.149	fig|6666666.59347.peg.4632	None	None	None	None	None	None	None	None

10013	putative orphan protein; putative membrane protein	None	fig|6666666.59330.peg.3293	fig|6666666.59331.peg.1235	fig|6666666.59332.peg.4386	fig|6666666.59334.peg.4407	fig|6666666.59335.peg.5202	None	None	None	fig|6666666.59339.peg.809	None	None	None	None	None	None	fig|6666666.59341.peg.2628	None	fig|6666666.59346.peg.3304	fig|6666666.59348.peg.3780	fig|6666666.59349.peg.2967	None	fig|6666666.59351.peg.4123	None	None	fig|6666666.59354.peg.3103

1410	FIG01199530 hypothetical protein	fig|6666666.59329.peg.5679	fig|6666666.59330.peg.1500	fig|6666666.59331.peg.502	fig|6666666.59332.peg.4652	None	fig|6666666.59335.peg.5139	fig|6666666.59336.peg.1159	fig|6666666.59333.peg.1741	None	fig|6666666.59339.peg.4177	None	fig|6666666.59337.peg.178	None	None	None	fig|6666666.59345.peg.4507	None	None	fig|6666666.59346.peg.2967	fig|6666666.59348.peg.4917	None	None	None	None	None	None

10379	2-deoxy-D-gluconate 3-dehydrogenase EC 1.1.1.125	fig|6666666.59329.peg.5099	None	None	None	fig|6666666.59334.peg.945	None	None	None	fig|6666666.59338.peg.4603	None	fig|6666666.59340.peg.2596	None	fig|6666666.59342.peg.3683	fig|6666666.71181.peg.4331	fig|6666666.59344.peg.830	None	fig|6666666.59341.peg.1264	fig|6666666.59347.peg.182	None	None	None	fig|6666666.59350.peg.5707	None	fig|6666666.59353.peg.1281	fig|6666666.59355.peg.3301	None

9144	MSHA biogenesis protein MshF	fig|6666666.59329.peg.602	None	None	fig|6666666.59332.peg.2043	fig|6666666.59334.peg.741	fig|6666666.59335.peg.208	fig|6666666.59336.peg.1183	fig|6666666.59333.peg.5459	None	fig|6666666.59339.peg.7773	None	fig|6666666.59337.peg.801	None	None	None	fig|6666666.59345.peg.4894	fig|6666666.59341.peg.3661	None	fig|6666666.59346.peg.1954	fig|6666666.59348.peg.3910	None	None	None	None	None	None

8121	Cdc6-related protein	fig|6666666.59329.peg.1143	None	fig|6666666.59331.peg.1931	fig|6666666.59332.peg.3624	fig|6666666.59334.peg.2459	None	fig|6666666.59336.peg.6390	None	fig|6666666.59338.peg.5239	None	None	fig|6666666.59337.peg.2723	fig|6666666.59342.peg.4640	None	None	fig|6666666.59345.peg.3202	fig|6666666.59341.peg.4027	fig|6666666.59347.peg.1709	fig|6666666.59346.peg.4708	None	None	None	None	None	None	None

39	Ferric iron ABC transporter, iron-binding protein	None	None	None	fig|6666666.59332.peg.6210	None	None	None	fig|6666666.59333.peg.4667	fig|6666666.59338.peg.5944	None	fig|6666666.59340.peg.2747	None	None	fig|6666666.71181.peg.2027	fig|6666666.59344.peg.2708	None	fig|6666666.59341.peg.5150	fig|6666666.59347.peg.4685	fig|6666666.59346.peg.1125	fig|6666666.59348.peg.4099	None	None	None	fig|6666666.59353.peg.3024	fig|6666666.59355.peg.2931	None

11403	FIG01204260 hypothetical protein	fig|6666666.59329.peg.2643	None	None	None	fig|6666666.59334.peg.3856	fig|6666666.59335.peg.5149	fig|6666666.59336.peg.1343	fig|6666666.59333.peg.78	fig|6666666.59338.peg.808	fig|6666666.59339.peg.4333	fig|6666666.59340.peg.221	fig|6666666.59337.peg.3265	fig|6666666.59342.peg.1285	None	None	fig|6666666.59345.peg.529	fig|6666666.59341.peg.171	None	None	None	None	None	None	None	None	None

6324	FIG035331 hypothetical protein	fig|6666666.59329.peg.3248	None	fig|6666666.59331.peg.2606	fig|6666666.59332.peg.3886	None	None	fig|6666666.59336.peg.5615	fig|6666666.59333.peg.1103	None	None	None	None	None	fig|6666666.71181.peg.3929	fig|6666666.59344.peg.5391	None	fig|6666666.59341.peg.4969	None	fig|6666666.59346.peg.2114	fig|6666666.59348.peg.1818	None	None	None	fig|6666666.59353.peg.4786	fig|6666666.59355.peg.4840	None

6323	5-Hydroxyisourate Hydrolase HIUase EC 3.5.2.17	None	None	fig|6666666.59331.peg.6155	fig|6666666.59332.peg.2254	None	fig|6666666.59335.peg.1139	None	None	None	fig|6666666.59339.peg.1645	None	None	None	fig|6666666.71181.peg.902	None	None	fig|6666666.59341.peg.4532	fig|6666666.59347.peg.3331	fig|6666666.59346.peg.4744	fig|6666666.59348.peg.3212	None	fig|6666666.59350.peg.3209	None	fig|6666666.59353.peg.917	fig|6666666.59355.peg.5381	None

4264	FIG01201083 hypothetical protein	fig|6666666.59329.peg.3316	fig|6666666.59330.peg.1483	None	fig|6666666.59332.peg.962	None	fig|6666666.59335.peg.4253	fig|6666666.59336.peg.3777	fig|6666666.59333.peg.1228	None	fig|6666666.59339.peg.5637	None	fig|6666666.59337.peg.193	fig|6666666.59342.peg.4216	None	None	fig|6666666.59345.peg.4522	None	None	fig|6666666.59346.peg.2590	fig|6666666.59348.peg.1851	None	None	None	None	None	None

9168	putative exported protein	fig|6666666.59329.peg.3399	fig|6666666.59330.peg.3666	None	None	None	fig|6666666.59335.peg.3563	fig|6666666.59336.peg.210	fig|6666666.59333.peg.2003	fig|6666666.59338.peg.6077	fig|6666666.59339.peg.8372	None	fig|6666666.59337.peg.777	None	None	None	fig|6666666.59345.peg.3611	fig|6666666.59341.peg.2112	fig|6666666.59347.peg.402	None	None	None	fig|6666666.59350.peg.3860	None	None	None	None

1594	Uncharacterized protein conserved in bacteria	None	None	None	fig|6666666.59332.peg.2116	fig|6666666.59334.peg.3678	None	None	None	fig|6666666.59338.peg.4035	None	fig|6666666.59340.peg.2856	None	None	fig|6666666.71181.peg.1123	fig|6666666.59344.peg.2242	None	fig|6666666.59341.peg.4618	fig|6666666.59347.peg.2176	fig|6666666.59346.peg.3937	fig|6666666.59348.peg.3450	None	None	None	fig|6666666.59353.peg.4960	fig|6666666.59355.peg.5051	None

3408	Fe2+-dicitrate sensor, membrane component	fig|6666666.59329.peg.1701	None	None	fig|6666666.59332.peg.3147	None	fig|6666666.59335.peg.4063	fig|6666666.59336.peg.1085	None	None	fig|6666666.59339.peg.6254	None	None	fig|6666666.59342.peg.747	fig|6666666.71181.peg.3595	fig|6666666.59344.peg.5554	None	None	None	fig|6666666.59346.peg.4786	None	None	None	fig|6666666.59351.peg.3337	fig|6666666.59353.peg.4141	fig|6666666.59355.peg.4274	None

3181	hypothetical protein	fig|6666666.59329.peg.1203	None	None	None	None	fig|6666666.59335.peg.3274	fig|6666666.59336.peg.2690	fig|6666666.59333.peg.4100	fig|6666666.59338.peg.1555	fig|6666666.59339.peg.6844	fig|6666666.59340.peg.2667	None	None	None	None	fig|6666666.59345.peg.3026	fig|6666666.59341.peg.2076	fig|6666666.59347.peg.584	None	fig|6666666.59348.peg.4034	None	None	fig|6666666.59351.peg.1929	None	None	None

5797	TPR repeat containing protein	fig|6666666.59329.peg.4046	fig|6666666.59330.peg.2377	None	fig|6666666.59332.peg.4986	None	None	fig|6666666.59336.peg.2121	fig|6666666.59333.peg.830	None	None	None	fig|6666666.59337.peg.2866	fig|6666666.59342.peg.5364	None	None	fig|6666666.59345.peg.1719	None	None	fig|6666666.59346.peg.4862	None	fig|6666666.59349.peg.4120	None	fig|6666666.59351.peg.1187	None	None	fig|6666666.59354.peg.1642

3216	Fucose permease	fig|6666666.59329.peg.5373	None	None	None	None	None	fig|6666666.59336.peg.5830	None	None	None	None	None	fig|6666666.59342.peg.2462	fig|6666666.71181.peg.4971	fig|6666666.59344.peg.1397	None	fig|6666666.59341.peg.5773	None	fig|6666666.59346.peg.2355	None	fig|6666666.59349.peg.2051	None	fig|6666666.59351.peg.3786	fig|6666666.59353.peg.548	fig|6666666.59355.peg.33	fig|6666666.59354.peg.4352

9419	Conserved domain protein	fig|6666666.59329.peg.1262	None	None	fig|6666666.59332.peg.1293	fig|6666666.59334.peg.3642	None	fig|6666666.59336.peg.5530	None	fig|6666666.59338.peg.5803	None	None	fig|6666666.59337.peg.1940	fig|6666666.59342.peg.3041	None	None	fig|6666666.59345.peg.4034	fig|6666666.59341.peg.3899	None	fig|6666666.59346.peg.2289	None	None	fig|6666666.59350.peg.3410	fig|6666666.59351.peg.3241	None	None	None

7993	Nicotinate phosphoribosyltransferase EC 2.4.2.11	fig|6666666.59329.peg.5535	fig|6666666.59330.peg.4813	None	None	None	None	fig|6666666.59336.peg.3516	fig|6666666.59333.peg.2105	None	None	None	fig|6666666.59337.peg.2841	None	fig|6666666.71181.peg.4355	fig|6666666.59344.peg.2771	fig|6666666.59345.peg.1756	fig|6666666.59341.peg.1466	None	None	None	None	fig|6666666.59350.peg.4596	None	fig|6666666.59353.peg.1672	fig|6666666.59355.peg.1049	None

9991	Urease accessory protein UreE	fig|6666666.59329.peg.2206	None	fig|6666666.59331.peg.3330	fig|6666666.59332.peg.5420	None	fig|6666666.59335.peg.4464	None	None	fig|6666666.59338.peg.3565	fig|6666666.59339.peg.5356	None	None	None	None	None	fig|6666666.59345.peg.4555	fig|6666666.59341.peg.6049	fig|6666666.59347.peg.568	fig|6666666.59346.peg.3221	fig|6666666.59348.peg.539	None	fig|6666666.59350.peg.4190	None	None	None	None

5470	L-xylulose 5-phosphate 3-epimerase EC 5.1.3.-	fig|6666666.59329.peg.2160	None	fig|6666666.59331.peg.669	fig|6666666.59332.peg.2912	fig|6666666.59334.peg.1227	None	fig|6666666.59336.peg.3190	None	None	None	None	None	None	fig|6666666.71181.peg.3565	fig|6666666.59344.peg.3962	fig|6666666.59345.peg.4531	fig|6666666.59341.peg.5771	None	fig|6666666.59346.peg.2358	None	None	None	None	fig|6666666.59353.peg.4387	fig|6666666.59355.peg.3067	None

8993	GlpM protein	fig|6666666.59329.peg.1310	fig|6666666.59330.peg.5077	None	None	None	None	fig|6666666.59336.peg.3520	None	None	None	None	fig|6666666.59337.peg.2843	fig|6666666.59342.peg.3489	fig|6666666.71181.peg.351	fig|6666666.59344.peg.2353	fig|6666666.59345.peg.1753	fig|6666666.59341.peg.5286	fig|6666666.59347.peg.1711	None	None	None	None	None	fig|6666666.59353.peg.245	fig|6666666.59355.peg.1285	None

8283	Transcriptional regulator, TetR family	fig|6666666.59329.peg.3773	fig|6666666.59330.peg.524	fig|6666666.59331.peg.4405	fig|6666666.59332.peg.1458	None	fig|6666666.59335.peg.3614	None	fig|6666666.59333.peg.527	None	fig|6666666.59339.peg.586	None	fig|6666666.59337.peg.5723	fig|6666666.59342.peg.2197	None	None	fig|6666666.59345.peg.4644	None	None	fig|6666666.59346.peg.3130	fig|6666666.59348.peg.4366	None	None	None	None	None	None

7398	Hypothetical protein, specific for	fig|6666666.59329.peg.566	fig|6666666.59330.peg.3094	fig|6666666.59331.peg.3943	fig|6666666.59332.peg.2630	None	fig|6666666.59335.peg.5213	fig|6666666.59336.peg.3751	fig|6666666.59333.peg.206	None	fig|6666666.59339.peg.5293	None	fig|6666666.59337.peg.3335	fig|6666666.59342.peg.1507	None	None	fig|6666666.59345.peg.5440	None	None	fig|6666666.59346.peg.4729	None	None	None	None	None	None	None

8744	FIG01203465 hypothetical protein	fig|6666666.59329.peg.5770	None	fig|6666666.59331.peg.4861	fig|6666666.59332.peg.915	fig|6666666.59334.peg.1360	None	fig|6666666.59336.peg.4159	fig|6666666.59333.peg.3686	fig|6666666.59338.peg.5723	None	fig|6666666.59340.peg.3112	fig|6666666.59337.peg.3430	None	None	None	fig|6666666.59345.peg.200	None	fig|6666666.59347.peg.786	fig|6666666.59346.peg.1542	None	None	None	None	None	None	None

3108	FIG01200821 hypothetical protein	fig|6666666.59329.peg.4918	fig|6666666.59330.peg.1148	fig|6666666.59331.peg.2561	fig|6666666.59332.peg.5986	None	fig|6666666.59335.peg.5655	fig|6666666.59336.peg.2488	fig|6666666.59333.peg.5293	None	fig|6666666.59339.peg.2512	None	fig|6666666.59337.peg.3648	None	None	None	fig|6666666.59345.peg.5199	None	None	fig|6666666.59346.peg.2307	None	None	None	None	None	None	fig|6666666.59354.peg.1504

5734	BatD	fig|6666666.59329.peg.5999	None	fig|6666666.59331.peg.4765	fig|6666666.59332.peg.3799	fig|6666666.59334.peg.4748	None	fig|6666666.59336.peg.6576	fig|6666666.59333.peg.2444	None	None	None	fig|6666666.59337.peg.2080	fig|6666666.59342.peg.4605	None	None	fig|6666666.59345.peg.3321	fig|6666666.59341.peg.1522	fig|6666666.59347.peg.707	fig|6666666.59346.peg.738	None	None	None	None	None	None	None

5730	Urease beta subunit EC 3.5.1.5	fig|6666666.59329.peg.2208	None	fig|6666666.59331.peg.1780	fig|6666666.59332.peg.5719	None	fig|6666666.59335.peg.4461	None	None	fig|6666666.59338.peg.4656	fig|6666666.59339.peg.5361	None	None	None	None	None	fig|6666666.59345.peg.4559	fig|6666666.59341.peg.247	fig|6666666.59347.peg.566	fig|6666666.59346.peg.1300	fig|6666666.59348.peg.544	None	fig|6666666.59350.peg.5028	None	None	None	None

4957	Macrophage infectivity potentiator-related protein	None	None	fig|6666666.59331.peg.3455	fig|6666666.59332.peg.2713	None	None	None	fig|6666666.59333.peg.2779	None	fig|6666666.59339.peg.5493	fig|6666666.59340.peg.1741	None	fig|6666666.59342.peg.1854	None	None	fig|6666666.59345.peg.254	None	None	fig|6666666.59346.peg.4687	None	fig|6666666.59349.peg.1656	fig|6666666.59350.peg.7068	fig|6666666.59351.peg.3242	None	None	fig|6666666.59354.peg.2057

5130	Evolved beta-D-galactosidase, beta subunit	fig|6666666.59329.peg.3132	None	None	fig|6666666.59332.peg.1253	None	fig|6666666.59335.peg.4076	fig|6666666.59336.peg.1088	fig|6666666.59333.peg.1136	None	fig|6666666.59339.peg.6849	None	fig|6666666.59337.peg.1345	fig|6666666.59342.peg.3164	None	None	fig|6666666.59345.peg.2299	fig|6666666.59341.peg.3324	None	fig|6666666.59346.peg.3261	fig|6666666.59348.peg.5051	None	None	None	None	None	None

1271	Probable type IV pilus assembly FimV-related transmembrane protein	fig|6666666.59329.peg.5172	fig|6666666.59330.peg.1598	None	fig|6666666.59332.peg.2755	None	fig|6666666.59335.peg.5878	fig|6666666.59336.peg.6640	fig|6666666.59333.peg.4800	None	fig|6666666.59339.peg.2966	None	fig|6666666.59337.peg.5671	fig|6666666.59342.peg.3883	None	None	fig|6666666.59345.peg.1206	None	None	fig|6666666.59346.peg.50	None	None	None	fig|6666666.59351.peg.3569	None	None	None

3331	hypothetical protein	fig|6666666.59329.peg.5979	fig|6666666.59330.peg.4235	fig|6666666.59331.peg.2400	fig|6666666.59332.peg.5321	None	fig|6666666.59335.peg.5762	fig|6666666.59336.peg.1769	fig|6666666.59333.peg.5127	None	fig|6666666.59339.peg.1837	None	fig|6666666.59337.peg.1254	None	None	None	fig|6666666.59345.peg.4440	None	fig|6666666.59347.peg.1656	fig|6666666.59346.peg.3371	None	None	None	None	None	None	None

2593	Predicted DNA-binding protein	fig|6666666.59329.peg.2900	fig|6666666.59330.peg.4547	fig|6666666.59331.peg.5511	fig|6666666.59332.peg.1242	None	fig|6666666.59335.peg.5333	None	fig|6666666.59333.peg.3202	None	fig|6666666.59339.peg.418	None	fig|6666666.59337.peg.4053	None	None	None	fig|6666666.59345.peg.280	fig|6666666.59341.peg.803	None	fig|6666666.59346.peg.2622	fig|6666666.59348.peg.2658	None	None	None	None	None	None

10866	Urease gamma subunit EC 3.5.1.5	fig|6666666.59329.peg.2209	None	fig|6666666.59331.peg.1781	fig|6666666.59332.peg.5718	None	fig|6666666.59335.peg.4460	None	None	fig|6666666.59338.peg.4655	fig|6666666.59339.peg.5362	None	None	None	None	None	fig|6666666.59345.peg.4560	fig|6666666.59341.peg.245	fig|6666666.59347.peg.565	fig|6666666.59346.peg.1301	fig|6666666.59348.peg.545	None	fig|6666666.59350.peg.5029	None	None	None	None

2104	hypothetical protein	None	None	fig|6666666.59331.peg.1362	fig|6666666.59332.peg.4996	fig|6666666.59334.peg.5045	None	None	None	fig|6666666.59338.peg.2112	None	fig|6666666.59340.peg.469	None	None	None	None	None	fig|6666666.59341.peg.821	fig|6666666.59347.peg.787	fig|6666666.59346.peg.4902	None	fig|6666666.59349.peg.2339	fig|6666666.59350.peg.4061	fig|6666666.59351.peg.1218	None	None	fig|6666666.59354.peg.2105

10489	Ribosomal large subunit pseudouridine synthase A EC 4.2.1.70	fig|6666666.59329.peg.5604	fig|6666666.59330.peg.4964	fig|6666666.59331.peg.4004	fig|6666666.59332.peg.3730	None	fig|6666666.59335.peg.5157	None	fig|6666666.59333.peg.5545	fig|6666666.59338.peg.714	fig|6666666.59339.peg.2377	None	fig|6666666.59337.peg.4766	None	None	None	fig|6666666.59345.peg.4495	None	fig|6666666.59347.peg.4252	fig|6666666.59346.peg.4129	None	None	None	None	None	None	None

3558	FIG01201079 hypothetical protein	None	fig|6666666.59330.peg.4268	fig|6666666.59331.peg.1423	fig|6666666.59332.peg.3388	None	fig|6666666.59335.peg.1675	fig|6666666.59336.peg.6541	fig|6666666.59333.peg.4412	None	fig|6666666.59339.peg.3685	None	fig|6666666.59337.peg.3574	fig|6666666.59342.peg.3196	None	None	fig|6666666.59345.peg.853	None	None	fig|6666666.59346.peg.4765	fig|6666666.59348.peg.2868	None	None	None	None	None	None

2341	FIG01057823 hypothetical protein	fig|6666666.59329.peg.2690	fig|6666666.59330.peg.4134	None	None	None	fig|6666666.59335.peg.910	fig|6666666.59336.peg.3472	fig|6666666.59333.peg.5042	fig|6666666.59338.peg.1249	fig|6666666.59339.peg.83	fig|6666666.59340.peg.2284	fig|6666666.59337.peg.3192	None	None	None	fig|6666666.59345.peg.2916	fig|6666666.59341.peg.115	fig|6666666.59347.peg.4604	None	None	None	None	None	None	None	None

1888	Small-conductance mechanosensitive channel	fig|6666666.59329.peg.1420	fig|6666666.59330.peg.2540	fig|6666666.59331.peg.4732	None	None	fig|6666666.59335.peg.1470	fig|6666666.59336.peg.3627	fig|6666666.59333.peg.604	fig|6666666.59338.peg.5990	None	None	fig|6666666.59337.peg.3687	None	None	None	fig|6666666.59345.peg.680	fig|6666666.59341.peg.1762	fig|6666666.59347.peg.759	fig|6666666.59346.peg.2610	None	None	None	None	None	None	None

4912	MSHA pilin protein MshC	None	fig|6666666.59330.peg.19	fig|6666666.59331.peg.6105	None	None	fig|6666666.59335.peg.211	fig|6666666.59336.peg.1187	fig|6666666.59333.peg.4386	None	fig|6666666.59339.peg.7776	None	fig|6666666.59337.peg.804	fig|6666666.59342.peg.6141	None	None	fig|6666666.59345.peg.4891	fig|6666666.59341.peg.3664	None	fig|6666666.59346.peg.1958	fig|6666666.59348.peg.2248	None	None	None	None	None	None

11092	FIG01203048 hypothetical protein	fig|6666666.59329.peg.1907	fig|6666666.59330.peg.3325	fig|6666666.59331.peg.2402	fig|6666666.59332.peg.3129	None	fig|6666666.59335.peg.1540	fig|6666666.59336.peg.4739	None	None	fig|6666666.59339.peg.3833	None	fig|6666666.59337.peg.4573	fig|6666666.59342.peg.1642	None	None	fig|6666666.59345.peg.518	fig|6666666.59341.peg.2017	None	fig|6666666.59346.peg.3369	None	None	None	None	None	None	None

11098	FIG01201466 hypothetical protein	fig|6666666.59329.peg.1473	fig|6666666.59330.peg.530	None	fig|6666666.59332.peg.5771	None	fig|6666666.59335.peg.3609	fig|6666666.59336.peg.1709	fig|6666666.59333.peg.531	None	fig|6666666.59339.peg.592	None	fig|6666666.59337.peg.3981	fig|6666666.59342.peg.3280	None	None	fig|6666666.59345.peg.4647	None	None	fig|6666666.59346.peg.3125	fig|6666666.59348.peg.4282	None	None	None	None	None	None

7296	hypothetical protein	None	None	fig|6666666.59331.peg.1512	fig|6666666.59332.peg.3573	fig|6666666.59334.peg.2006	None	fig|6666666.59336.peg.2406	fig|6666666.59333.peg.5038	fig|6666666.59338.peg.1579	None	fig|6666666.59340.peg.2177	None	fig|6666666.59342.peg.2787	None	None	fig|6666666.59345.peg.3980	fig|6666666.59341.peg.4234	fig|6666666.59347.peg.417	fig|6666666.59346.peg.425	None	None	None	None	None	None	None

5848	lipoprotein, putative	None	None	None	fig|6666666.59332.peg.5935	None	None	None	None	fig|6666666.59338.peg.991	None	None	fig|6666666.59337.peg.5353	fig|6666666.59342.peg.3817	fig|6666666.71181.peg.1448	fig|6666666.59344.peg.1534	fig|6666666.59345.peg.421	None	fig|6666666.59347.peg.1281	fig|6666666.59346.peg.1050	None	None	None	None	fig|6666666.59353.peg.4364	fig|6666666.59355.peg.4479	None

7212	FIG01199876 hypothetical protein	fig|6666666.59329.peg.2396	fig|6666666.59330.peg.2108	None	None	None	fig|6666666.59335.peg.983	None	fig|6666666.59333.peg.1446	fig|6666666.59338.peg.5238	fig|6666666.59339.peg.4465	None	fig|6666666.59337.peg.5492	fig|6666666.59342.peg.1458	None	None	fig|6666666.59345.peg.496	fig|6666666.59341.peg.1914	fig|6666666.59347.peg.660	None	None	None	None	None	None	None	None

7235	FIG01201498 hypothetical protein	fig|6666666.59329.peg.744	fig|6666666.59330.peg.3076	fig|6666666.59331.peg.972	fig|6666666.59332.peg.5083	None	None	fig|6666666.59336.peg.2620	fig|6666666.59333.peg.1413	fig|6666666.59338.peg.3954	None	None	fig|6666666.59337.peg.1493	None	None	None	fig|6666666.59345.peg.348	None	fig|6666666.59347.peg.1385	fig|6666666.59346.peg.2210	None	None	None	None	None	None	None

10698	Glyoxalase family protein	None	None	fig|6666666.59331.peg.3654	None	fig|6666666.59334.peg.5575	fig|6666666.59335.peg.1386	None	None	None	fig|6666666.59339.peg.6747	None	None	None	None	None	None	fig|6666666.59341.peg.3513	None	fig|6666666.59346.peg.4820	fig|6666666.59348.peg.859	fig|6666666.59349.peg.2279	fig|6666666.59350.peg.3719	fig|6666666.59351.peg.731	None	None	fig|6666666.59354.peg.4105

8734	Type I restriction-modification system, specificity subunit S EC 3.1.21.3	fig|6666666.59329.peg.2259	fig|6666666.59330.peg.1815	fig|6666666.59331.peg.962	None	fig|6666666.59334.peg.3973	None	None	None	fig|6666666.59338.peg.1042	None	None	fig|6666666.59337.peg.932	fig|6666666.59342.peg.3866	None	None	fig|6666666.59345.peg.5154	None	None	None	fig|6666666.59348.peg.29	fig|6666666.59349.peg.2045	None	None	None	None	fig|6666666.59354.peg.5574

4190	HupE-UreJ family metal transporter	None	None	None	fig|6666666.59332.peg.3039	fig|6666666.59334.peg.2134	fig|6666666.59335.peg.4465	None	None	fig|6666666.59338.peg.3564	fig|6666666.59339.peg.5355	fig|6666666.59340.peg.1157	None	None	None	None	None	fig|6666666.59341.peg.4507	fig|6666666.59347.peg.569	fig|6666666.59346.peg.3220	fig|6666666.59348.peg.1493	None	fig|6666666.59350.peg.5276	None	None	None	None

5675	hypothetical protein	fig|6666666.59329.peg.6214	fig|6666666.59330.peg.4833	fig|6666666.59331.peg.5635	None	None	None	fig|6666666.59336.peg.6316	fig|6666666.59333.peg.2613	fig|6666666.59338.peg.2902	None	None	fig|6666666.59337.peg.5844	fig|6666666.59342.peg.4545	None	None	fig|6666666.59345.peg.793	None	None	fig|6666666.59346.peg.2873	fig|6666666.59348.peg.4538	None	None	None	None	None	None

7494	Putative dihydroxyacetone kinase EC 2.7.1.29 , dihydroxyacetone binding subunit	None	None	None	None	fig|6666666.59334.peg.1587	None	None	fig|6666666.59333.peg.107	fig|6666666.59338.peg.2022	None	None	None	fig|6666666.59342.peg.2362	fig|6666666.71181.peg.3028	fig|6666666.59344.peg.6103	None	fig|6666666.59341.peg.1453	fig|6666666.59347.peg.4233	None	fig|6666666.59348.peg.2454	None	None	None	fig|6666666.59353.peg.2955	fig|6666666.59355.peg.2785	None

4720	Glycosidase	fig|6666666.59329.peg.5539	fig|6666666.59330.peg.4330	fig|6666666.59331.peg.4872	fig|6666666.59332.peg.4987	None	None	fig|6666666.59336.peg.2397	fig|6666666.59333.peg.1069	None	None	fig|6666666.59340.peg.2388	fig|6666666.59337.peg.3426	fig|6666666.59342.peg.1710	None	None	fig|6666666.59345.peg.1956	None	None	fig|6666666.59346.peg.1786	None	None	None	None	None	None	None

10348	ABC transporter permease protein	None	fig|6666666.59330.peg.3943	None	None	fig|6666666.59334.peg.1656	fig|6666666.59335.peg.5831	fig|6666666.59336.peg.2808	fig|6666666.59333.peg.3608	fig|6666666.59338.peg.5877	fig|6666666.59339.peg.3126	None	None	fig|6666666.59342.peg.809	None	None	fig|6666666.59345.peg.1095	None	fig|6666666.59347.peg.2630	None	fig|6666666.59348.peg.4601	None	None	None	None	None	None

443	Na + H + antiporter subunit F	None	None	None	None	None	fig|6666666.59335.peg.4441	None	fig|6666666.59333.peg.4707	fig|6666666.59338.peg.4612	None	fig|6666666.59340.peg.3192	None	fig|6666666.59342.peg.4552	None	None	fig|6666666.59345.peg.246	None	fig|6666666.59347.peg.2132	None	None	fig|6666666.59349.peg.3894	fig|6666666.59350.peg.84	fig|6666666.59351.peg.1378	None	None	fig|6666666.59354.peg.2459

8356	Transcriptional regulator, LysR family	fig|6666666.59329.peg.3172	None	None	fig|6666666.59332.peg.4166	fig|6666666.59334.peg.4199	None	fig|6666666.59336.peg.3278	fig|6666666.59333.peg.1041	fig|6666666.59338.peg.1899	None	None	fig|6666666.59337.peg.3407	None	None	None	fig|6666666.59345.peg.1986	fig|6666666.59341.peg.4830	fig|6666666.59347.peg.1147	fig|6666666.59346.peg.4331	None	None	None	None	None	None	None

951	FIG01200798 hypothetical protein	None	None	None	fig|6666666.59332.peg.4586	fig|6666666.59334.peg.4807	fig|6666666.59335.peg.3228	fig|6666666.59336.peg.512	fig|6666666.59333.peg.2177	fig|6666666.59338.peg.5835	fig|6666666.59339.peg.779	None	None	fig|6666666.59342.peg.1535	None	None	fig|6666666.59345.peg.766	None	fig|6666666.59347.peg.3421	None	None	None	fig|6666666.59350.peg.1891	None	None	None	None

1405	Retron-type RNA-directed DNA polymerase EC 2.7.7.49	None	fig|6666666.59330.peg.2065	None	fig|6666666.59332.peg.5018	None	None	None	None	fig|6666666.59338.peg.2322	None	None	None	fig|6666666.59342.peg.3028	fig|6666666.71181.peg.713	fig|6666666.59344.peg.688	None	None	fig|6666666.59347.peg.2011	fig|6666666.59346.peg.830	None	fig|6666666.59349.peg.4880	None	None	fig|6666666.59353.peg.986	fig|6666666.59355.peg.2389	None

10939	Gamma-glutamyl-GABA hydrolase EC 3.5.1.94	None	None	None	fig|6666666.59332.peg.2917	None	None	None	fig|6666666.59333.peg.4573	fig|6666666.59338.peg.3923	None	None	None	None	fig|6666666.71181.peg.1218	fig|6666666.59344.peg.193	None	fig|6666666.59341.peg.5650	fig|6666666.59347.peg.374	fig|6666666.59346.peg.1109	None	None	fig|6666666.59350.peg.4858	None	fig|6666666.59353.peg.501	fig|6666666.59355.peg.2225	None

4473	putative flagellar motor switch protein	fig|6666666.59329.peg.3933	fig|6666666.59330.peg.2226	None	None	None	fig|6666666.59335.peg.2403	fig|6666666.59336.peg.470	fig|6666666.59333.peg.2871	fig|6666666.59338.peg.1770	fig|6666666.59339.peg.111	None	None	fig|6666666.59342.peg.3736	None	None	fig|6666666.59345.peg.2864	fig|6666666.59341.peg.138	fig|6666666.59347.peg.4624	None	None	None	None	None	None	None	None

11076	DNA RNA endonuclease G	fig|6666666.59329.peg.451	None	fig|6666666.59331.peg.3307	None	None	fig|6666666.59335.peg.5506	fig|6666666.59336.peg.1877	None	None	fig|6666666.59339.peg.7160	None	None	fig|6666666.59342.peg.4861	fig|6666666.71181.peg.315	fig|6666666.59344.peg.1252	None	None	None	fig|6666666.59346.peg.1733	None	None	None	None	fig|6666666.59353.peg.4232	fig|6666666.59355.peg.4194	None

4636	hypothetical protein	None	None	fig|6666666.59331.peg.4590	fig|6666666.59332.peg.3480	fig|6666666.59334.peg.5256	fig|6666666.59335.peg.5250	None	None	fig|6666666.59338.peg.4023	fig|6666666.59339.peg.3697	fig|6666666.59340.peg.919	None	None	None	None	None	fig|6666666.59341.peg.1826	fig|6666666.59347.peg.3591	fig|6666666.59346.peg.2083	fig|6666666.59348.peg.2954	None	None	None	None	None	None

2078	FIG01199976 hypothetical protein	fig|6666666.59329.peg.4710	None	fig|6666666.59331.peg.955	None	None	fig|6666666.59335.peg.5675	fig|6666666.59336.peg.3837	None	fig|6666666.59338.peg.1773	fig|6666666.59339.peg.1533	None	None	fig|6666666.59342.peg.3553	None	None	None	fig|6666666.59341.peg.903	None	None	None	fig|6666666.59349.peg.3351	None	fig|6666666.59351.peg.3154	None	None	fig|6666666.59354.peg.5359

9801	Ribonucleotide reduction protein NrdI	None	None	None	fig|6666666.59332.peg.2070	None	fig|6666666.59335.peg.4380	None	None	fig|6666666.59338.peg.3842	fig|6666666.59339.peg.2173	None	None	None	fig|6666666.71181.peg.2620	fig|6666666.59344.peg.3886	None	None	fig|6666666.59347.peg.2750	None	fig|6666666.59348.peg.3684	None	fig|6666666.59350.peg.5088	None	fig|6666666.59353.peg.4691	fig|6666666.59355.peg.4073	None

9324	hypothetical protein	fig|6666666.59329.peg.5017	fig|6666666.59330.peg.696	fig|6666666.59331.peg.5181	fig|6666666.59332.peg.5798	None	None	fig|6666666.59336.peg.2990	None	fig|6666666.59338.peg.883	None	None	fig|6666666.59337.peg.4155	None	None	None	fig|6666666.59345.peg.4777	fig|6666666.59341.peg.763	fig|6666666.59347.peg.993	fig|6666666.59346.peg.1053	None	None	None	None	None	None	None

8850	Alkyl sulfatase EC 3.1.6.-	None	None	fig|6666666.59331.peg.3107	fig|6666666.59332.peg.1581	fig|6666666.59334.peg.3266	None	None	None	fig|6666666.59338.peg.293	None	fig|6666666.59340.peg.1204	None	None	fig|6666666.71181.peg.3257	fig|6666666.59344.peg.4011	None	fig|6666666.59341.peg.4128	None	fig|6666666.59346.peg.4379	None	None	None	None	fig|6666666.59353.peg.4039	fig|6666666.59355.peg.3996	None

5279	YafQ toxin protein	None	None	None	fig|6666666.59332.peg.612	fig|6666666.59334.peg.5371	None	fig|6666666.59336.peg.6489	fig|6666666.59333.peg.5447	fig|6666666.59338.peg.1886	None	None	fig|6666666.59337.peg.5741	None	fig|6666666.71181.peg.3515	fig|6666666.59344.peg.4625	None	None	fig|6666666.59347.peg.532	None	None	None	None	None	fig|6666666.59353.peg.2413	fig|6666666.59355.peg.504	None

1977	Type I restriction-modification system, restriction subunit R EC 3.1.21.3	fig|6666666.59329.peg.954	fig|6666666.59330.peg.2499	None	None	None	None	None	None	fig|6666666.59338.peg.1355	fig|6666666.59339.peg.7292	None	fig|6666666.59337.peg.933	None	None	None	fig|6666666.59345.peg.5153	None	None	None	fig|6666666.59348.peg.30	fig|6666666.59349.peg.2048	fig|6666666.59350.peg.2994	fig|6666666.59351.peg.2449	None	None	fig|6666666.59354.peg.6061

5478	Transglycosylase associated protein	None	fig|6666666.59330.peg.1235	None	None	fig|6666666.59334.peg.4687	None	fig|6666666.59336.peg.1246	fig|6666666.59333.peg.5292	None	None	None	fig|6666666.59337.peg.3747	None	fig|6666666.71181.peg.4059	fig|6666666.59344.peg.3769	fig|6666666.59345.peg.1181	None	fig|6666666.59347.peg.1828	None	None	None	None	None	fig|6666666.59353.peg.4024	fig|6666666.59355.peg.3982	None

9894	Putative isomerase	fig|6666666.59329.peg.1492	fig|6666666.59330.peg.1633	fig|6666666.59331.peg.5025	fig|6666666.59332.peg.1258	None	fig|6666666.59335.peg.3273	fig|6666666.59336.peg.1091	None	None	fig|6666666.59339.peg.6843	None	fig|6666666.59337.peg.1268	fig|6666666.59342.peg.5056	None	None	fig|6666666.59345.peg.2302	fig|6666666.59341.peg.938	None	None	None	None	None	None	None	None	None

2219	FIG01204124 hypothetical protein	fig|6666666.59329.peg.5538	fig|6666666.59330.peg.5076	None	None	None	None	fig|6666666.59336.peg.3519	fig|6666666.59333.peg.2106	fig|6666666.59338.peg.5161	None	None	fig|6666666.59337.peg.2842	fig|6666666.59342.peg.3490	None	None	fig|6666666.59345.peg.1754	fig|6666666.59341.peg.5285	fig|6666666.59347.peg.1712	None	fig|6666666.59348.peg.2390	None	None	None	None	None	None

10839	Arginase agmatinase formimionoglutamate hydrolase, arginase family	fig|6666666.59329.peg.1892	fig|6666666.59330.peg.4974	None	None	None	None	fig|6666666.59336.peg.3530	fig|6666666.59333.peg.4340	fig|6666666.59338.peg.5613	None	None	fig|6666666.59337.peg.5508	fig|6666666.59342.peg.2370	None	None	fig|6666666.59345.peg.410	fig|6666666.59341.peg.2073	fig|6666666.59347.peg.587	None	fig|6666666.59348.peg.3937	None	None	None	None	None	None

5357	Transmembrane component MtsC of energizing module of methionine-regulated ECF transporter	fig|6666666.59329.peg.6422	fig|6666666.59330.peg.729	fig|6666666.59331.peg.3285	fig|6666666.59332.peg.3719	None	fig|6666666.59335.peg.2519	fig|6666666.59336.peg.2851	fig|6666666.59333.peg.2240	None	fig|6666666.59339.peg.1928	None	fig|6666666.59337.peg.3767	None	None	None	fig|6666666.59345.peg.2794	None	None	fig|6666666.59346.peg.1335	None	None	None	None	None	None	None

1995	FIG01202211 hypothetical protein	fig|6666666.59329.peg.1121	None	fig|6666666.59331.peg.1566	fig|6666666.59332.peg.764	None	None	fig|6666666.59336.peg.1647	None	fig|6666666.59338.peg.4205	None	fig|6666666.59340.peg.1543	fig|6666666.59337.peg.717	fig|6666666.59342.peg.6204	None	None	fig|6666666.59345.peg.3672	fig|6666666.59341.peg.4940	fig|6666666.59347.peg.2112	None	None	None	None	None	None	None	None

2392	transcriptional regulator, LysR family	None	None	fig|6666666.59331.peg.2585	fig|6666666.59332.peg.5800	fig|6666666.59334.peg.5289	None	None	None	fig|6666666.59338.peg.2174	None	None	None	None	fig|6666666.71181.peg.4260	fig|6666666.59344.peg.316	None	None	None	fig|6666666.59346.peg.1463	None	None	fig|6666666.59350.peg.4263	fig|6666666.59351.peg.5319	fig|6666666.59353.peg.2501	fig|6666666.59355.peg.594	None

8106	Queuosine biosynthesis QueD, PTPS-I	None	fig|6666666.59330.peg.2067	None	None	None	None	fig|6666666.59336.peg.1912	fig|6666666.59333.peg.5260	None	None	None	fig|6666666.59337.peg.3562	None	fig|6666666.71181.peg.3401	fig|6666666.59344.peg.2215	fig|6666666.59345.peg.838	fig|6666666.59341.peg.2093	None	None	None	None	fig|6666666.59350.peg.1070	None	fig|6666666.59353.peg.194	fig|6666666.59355.peg.2963	None

1352	FIG01205819 hypothetical protein	fig|6666666.59329.peg.4038	None	None	None	fig|6666666.59334.peg.2935	None	fig|6666666.59336.peg.1113	fig|6666666.59333.peg.2266	fig|6666666.59338.peg.1923	None	fig|6666666.59340.peg.3377	fig|6666666.59337.peg.4363	fig|6666666.59342.peg.726	None	None	fig|6666666.59345.peg.1898	fig|6666666.59341.peg.4581	fig|6666666.59347.peg.4215	None	None	None	None	None	None	None	None

5910	Chitinase EC 3.2.1.14	fig|6666666.59329.peg.5487	fig|6666666.59330.peg.3702	None	None	None	fig|6666666.59335.peg.5327	fig|6666666.59336.peg.4582	None	None	fig|6666666.59339.peg.6782	fig|6666666.59340.peg.2623	fig|6666666.59337.peg.3691	fig|6666666.59342.peg.3283	None	None	fig|6666666.59345.peg.667	None	fig|6666666.59347.peg.3759	None	fig|6666666.59348.peg.4799	None	None	None	None	None	None

10870	Gfa-like protein	fig|6666666.59329.peg.4939	None	None	None	None	fig|6666666.59335.peg.1215	fig|6666666.59336.peg.5378	fig|6666666.59333.peg.5468	None	fig|6666666.59339.peg.1498	None	fig|6666666.59337.peg.3118	fig|6666666.59342.peg.2647	None	None	fig|6666666.59345.peg.4125	None	fig|6666666.59347.peg.5436	None	fig|6666666.59348.peg.4470	None	fig|6666666.59350.peg.2083	None	None	None	None

5688	Opine oxidase subunit A	None	None	fig|6666666.59331.peg.2690	None	None	fig|6666666.59335.peg.5426	None	None	None	fig|6666666.59339.peg.2044	None	None	None	fig|6666666.71181.peg.344	fig|6666666.59344.peg.2344	None	None	None	None	None	fig|6666666.59349.peg.3939	fig|6666666.59350.peg.4480	fig|6666666.59351.peg.3237	fig|6666666.59353.peg.238	fig|6666666.59355.peg.1278	fig|6666666.59354.peg.1635

6603	Phage Integrase	fig|6666666.59329.peg.520	fig|6666666.59330.peg.219	fig|6666666.59331.peg.173	fig|6666666.59332.peg.58	fig|6666666.59334.peg.4270	fig|6666666.59335.peg.97	None	None	fig|6666666.59338.peg.860	None	fig|6666666.59340.peg.278	fig|6666666.59337.peg.939	None	None	None	None	None	None	None	fig|6666666.59348.peg.34	None	None	fig|6666666.59351.peg.2226	None	None	None

10606	hypothetical protein	None	None	None	fig|6666666.59332.peg.6048	fig|6666666.59334.peg.4449	None	None	fig|6666666.59333.peg.5265	fig|6666666.59338.peg.3106	None	None	None	None	None	None	None	fig|6666666.59341.peg.4903	fig|6666666.59347.peg.1151	fig|6666666.59346.peg.1516	None	fig|6666666.59349.peg.4139	fig|6666666.59350.peg.6069	fig|6666666.59351.peg.4694	None	None	fig|6666666.59354.peg.6007

7636	FIG00809136 hypothetical protein	None	None	None	fig|6666666.59332.peg.5941	None	None	fig|6666666.59336.peg.6389	None	None	None	None	None	fig|6666666.59342.peg.3691	None	fig|6666666.59344.peg.5069	fig|6666666.59345.peg.97	fig|6666666.59341.peg.5614	fig|6666666.59347.peg.986	fig|6666666.59346.peg.3638	None	None	fig|6666666.59350.peg.6722	None	fig|6666666.59353.peg.4048	None	fig|6666666.59354.peg.4247

1539	FIG01200188 hypothetical protein	fig|6666666.59329.peg.6299	fig|6666666.59330.peg.4884	None	None	None	None	fig|6666666.59336.peg.6391	fig|6666666.59333.peg.4786	fig|6666666.59338.peg.4242	None	None	fig|6666666.59337.peg.1178	None	None	None	fig|6666666.59345.peg.5707	None	fig|6666666.59347.peg.1228	None	None	fig|6666666.59349.peg.2940	None	fig|6666666.59351.peg.3969	None	None	fig|6666666.59354.peg.5967

4108	Uncharacterized protein conserved in bacteria	None	None	None	None	fig|6666666.59334.peg.5624	None	None	fig|6666666.59333.peg.5509	None	fig|6666666.59339.peg.4657	None	None	fig|6666666.59342.peg.6070	fig|6666666.71181.peg.5217	fig|6666666.59344.peg.2757	None	None	None	None	None	fig|6666666.59349.peg.1847	None	fig|6666666.59351.peg.4072	fig|6666666.59353.peg.2983	fig|6666666.59355.peg.2889	fig|6666666.59354.peg.1372

5314	FIG01203188 hypothetical protein	fig|6666666.59329.peg.1505	fig|6666666.59330.peg.2361	fig|6666666.59331.peg.6014	fig|6666666.59332.peg.5136	None	None	fig|6666666.59336.peg.1127	None	fig|6666666.59338.peg.2895	None	None	fig|6666666.59337.peg.2231	None	None	None	fig|6666666.59345.peg.2985	fig|6666666.59341.peg.5478	fig|6666666.59347.peg.796	fig|6666666.59346.peg.3171	None	None	None	None	None	None	None

3375	Carbonic anhydrase EC 4.2.1.1	fig|6666666.59329.peg.3822	fig|6666666.59330.peg.3957	None	None	None	fig|6666666.59335.peg.1480	fig|6666666.59336.peg.4207	fig|6666666.59333.peg.2122	None	fig|6666666.59339.peg.4417	None	fig|6666666.59337.peg.1919	None	None	None	fig|6666666.59345.peg.4539	fig|6666666.59341.peg.3464	fig|6666666.59347.peg.361	None	fig|6666666.59348.peg.4500	None	None	None	None	None	None

7248	FIG01201060 hypothetical protein	fig|6666666.59329.peg.3195	None	None	fig|6666666.59332.peg.1058	None	fig|6666666.59335.peg.4395	fig|6666666.59336.peg.6171	fig|6666666.59333.peg.5033	None	fig|6666666.59339.peg.8485	None	fig|6666666.59337.peg.2507	fig|6666666.59342.peg.887	None	None	fig|6666666.59345.peg.137	None	None	fig|6666666.59346.peg.4009	fig|6666666.59348.peg.5074	None	None	None	None	None	None

8899	Substrate-specific component MtsA of methionine-regulated ECF transporter	None	fig|6666666.59330.peg.733	None	fig|6666666.59332.peg.3721	None	fig|6666666.59335.peg.5805	fig|6666666.59336.peg.2849	fig|6666666.59333.peg.2244	None	fig|6666666.59339.peg.1932	None	fig|6666666.59337.peg.3765	fig|6666666.59342.peg.1885	None	None	fig|6666666.59345.peg.2792	None	None	fig|6666666.59346.peg.1337	fig|6666666.59348.peg.3905	None	None	None	None	None	None

3176	FIG017861 hypothetical protein	fig|6666666.59329.peg.3341	None	fig|6666666.59331.peg.3731	fig|6666666.59332.peg.5791	None	None	fig|6666666.59336.peg.1052	fig|6666666.59333.peg.1096	None	None	None	None	None	fig|6666666.71181.peg.3936	fig|6666666.59344.peg.5382	None	fig|6666666.59341.peg.2757	None	fig|6666666.59346.peg.4719	None	None	None	None	fig|6666666.59353.peg.4793	fig|6666666.59355.peg.4847	None

248	FIG01199754 hypothetical protein	fig|6666666.59329.peg.4103	fig|6666666.59330.peg.3837	None	None	fig|6666666.59334.peg.2601	fig|6666666.59335.peg.1411	None	fig|6666666.59333.peg.4970	fig|6666666.59338.peg.4593	fig|6666666.59339.peg.6661	None	fig|6666666.59337.peg.2019	None	None	None	fig|6666666.59345.peg.3085	fig|6666666.59341.peg.4586	fig|6666666.59347.peg.1624	None	None	None	None	None	None	None	None

7441	FIG01205227 hypothetical protein	fig|6666666.59329.peg.1562	None	fig|6666666.59331.peg.1822	fig|6666666.59332.peg.2198	None	None	fig|6666666.59336.peg.413	fig|6666666.59333.peg.744	None	None	None	None	fig|6666666.59342.peg.5905	fig|6666666.71181.peg.5529	None	fig|6666666.59345.peg.1020	fig|6666666.59341.peg.5202	fig|6666666.59347.peg.1680	fig|6666666.59346.peg.4205	None	None	None	None	None	None	None

10509	Inner membrane protein YjgN	None	fig|6666666.59330.peg.1201	fig|6666666.59331.peg.3161	fig|6666666.59332.peg.3198	None	fig|6666666.59335.peg.247	fig|6666666.59336.peg.6422	None	None	fig|6666666.59339.peg.8148	None	fig|6666666.59337.peg.3141	fig|6666666.59342.peg.3145	None	None	fig|6666666.59345.peg.1151	None	None	fig|6666666.59346.peg.1673	None	None	None	fig|6666666.59351.peg.1453	None	None	None

4286	Manganese transport protein MntH	fig|6666666.59329.peg.177	None	None	None	None	None	fig|6666666.59336.peg.3085	None	None	None	None	fig|6666666.59337.peg.5293	fig|6666666.59342.peg.3016	fig|6666666.71181.peg.1633	fig|6666666.59344.peg.5028	fig|6666666.59345.peg.2940	fig|6666666.59341.peg.930	None	None	None	None	None	fig|6666666.59351.peg.1763	fig|6666666.59353.peg.326	fig|6666666.59355.peg.2848	None

6198	FIG01199882 hypothetical protein	None	fig|6666666.59330.peg.2851	None	fig|6666666.59332.peg.5471	None	None	fig|6666666.59336.peg.5798	fig|6666666.59333.peg.3673	fig|6666666.59338.peg.2080	None	None	fig|6666666.59337.peg.1963	fig|6666666.59342.peg.1260	None	None	fig|6666666.59345.peg.4077	fig|6666666.59341.peg.2217	fig|6666666.59347.peg.1499	fig|6666666.59346.peg.662	None	None	None	None	None	None	None

9186	FIG01199992 hypothetical protein	fig|6666666.59329.peg.4844	fig|6666666.59330.peg.446	None	fig|6666666.59332.peg.427	None	fig|6666666.59335.peg.5865	fig|6666666.59336.peg.3380	fig|6666666.59333.peg.1157	None	None	None	fig|6666666.59337.peg.1604	fig|6666666.59342.peg.2984	None	None	fig|6666666.59345.peg.2179	None	None	fig|6666666.59346.peg.3700	fig|6666666.59348.peg.639	None	None	None	None	None	None

808	FIG138576 3-oxoacyl-[ACP] synthase EC 2.3.1.41	fig|6666666.59329.peg.3241	None	fig|6666666.59331.peg.3657	None	None	None	fig|6666666.59336.peg.1009	fig|6666666.59333.peg.1107	None	None	None	None	None	fig|6666666.71181.peg.3925	fig|6666666.59344.peg.5396	None	fig|6666666.59341.peg.4965	None	fig|6666666.59346.peg.2110	fig|6666666.59348.peg.2996	None	None	None	fig|6666666.59353.peg.4782	fig|6666666.59355.peg.4835	None

10413	FIG01202129 hypothetical protein	fig|6666666.59329.peg.6123	fig|6666666.59330.peg.1145	None	None	None	fig|6666666.59335.peg.5341	fig|6666666.59336.peg.2485	fig|6666666.59333.peg.5415	fig|6666666.59338.peg.1805	fig|6666666.59339.peg.6614	None	fig|6666666.59337.peg.3645	None	None	None	fig|6666666.59345.peg.5202	fig|6666666.59341.peg.5399	fig|6666666.59347.peg.793	None	None	None	None	None	None	None	None

6761	Permease of the major facilitator superfamily	None	None	fig|6666666.59331.peg.3908	fig|6666666.59332.peg.2711	fig|6666666.59334.peg.3686	None	None	None	fig|6666666.59338.peg.3694	None	fig|6666666.59340.peg.1711	fig|6666666.59337.peg.1636	None	None	None	fig|6666666.59345.peg.5483	fig|6666666.59341.peg.648	fig|6666666.59347.peg.3940	None	fig|6666666.59348.peg.2614	None	None	None	None	None	fig|6666666.59354.peg.3153

10199	hypothetical protein	None	None	None	None	None	fig|6666666.59335.peg.1469	None	None	None	fig|6666666.59339.peg.4428	None	None	None	fig|6666666.71181.peg.2623	fig|6666666.59344.peg.3889	None	None	None	None	fig|6666666.59348.peg.2722	fig|6666666.59349.peg.2470	fig|6666666.59350.peg.3586	fig|6666666.59351.peg.1630	fig|6666666.59353.peg.4688	fig|6666666.59355.peg.4070	fig|6666666.59354.peg.985

1128	FIG01199970 hypothetical protein	fig|6666666.59329.peg.1760	None	None	None	fig|6666666.59334.peg.1099	fig|6666666.59335.peg.2616	fig|6666666.59336.peg.3573	None	fig|6666666.59338.peg.2091	fig|6666666.59339.peg.2232	None	fig|6666666.59337.peg.5316	None	None	None	fig|6666666.59345.peg.4006	fig|6666666.59341.peg.5407	fig|6666666.59347.peg.825	None	fig|6666666.59348.peg.1511	None	None	None	None	None	None

4615	hypothetical protein	None	None	fig|6666666.59331.peg.713	fig|6666666.59332.peg.5267	fig|6666666.59334.peg.447	None	fig|6666666.59336.peg.1751	None	fig|6666666.59338.peg.3951	None	fig|6666666.59340.peg.1813	None	fig|6666666.59342.peg.5837	None	None	fig|6666666.59345.peg.4215	fig|6666666.59341.peg.2963	fig|6666666.59347.peg.1388	fig|6666666.59346.peg.2510	None	None	None	None	None	None	None

1190	FIG01200235 hypothetical protein	fig|6666666.59329.peg.4338	fig|6666666.59330.peg.2111	fig|6666666.59331.peg.3313	None	None	fig|6666666.59335.peg.1422	fig|6666666.59336.peg.2220	fig|6666666.59333.peg.1449	None	fig|6666666.59339.peg.223	None	fig|6666666.59337.peg.5489	fig|6666666.59342.peg.1462	None	None	fig|6666666.59345.peg.499	None	None	fig|6666666.59346.peg.897	None	None	None	None	None	None	None

7920	FIG01199830 hypothetical protein	fig|6666666.59329.peg.60	fig|6666666.59330.peg.638	fig|6666666.59331.peg.5139	fig|6666666.59332.peg.222	None	fig|6666666.59335.peg.2438	fig|6666666.59336.peg.3765	fig|6666666.59333.peg.1381	None	fig|6666666.59339.peg.8087	None	fig|6666666.59337.peg.3897	None	None	None	fig|6666666.59345.peg.4932	None	None	fig|6666666.59346.peg.1818	None	None	None	None	None	None	None

201	probable deca-heme c-type cytochrome	fig|6666666.59329.peg.6163	None	fig|6666666.59331.peg.2503	fig|6666666.59332.peg.3658	fig|6666666.59334.peg.2310	None	fig|6666666.59336.peg.5162	None	fig|6666666.59338.peg.4320	None	None	fig|6666666.59337.peg.2072	fig|6666666.59342.peg.4175	None	None	fig|6666666.59345.peg.3310	None	fig|6666666.59347.peg.700	fig|6666666.59346.peg.729	None	None	None	None	None	None	None

7154	FIG01205142 hypothetical protein	fig|6666666.59329.peg.4617	fig|6666666.59330.peg.1964	fig|6666666.59331.peg.649	fig|6666666.59332.peg.1595	None	fig|6666666.59335.peg.2458	fig|6666666.59336.peg.2674	fig|6666666.59333.peg.5354	None	fig|6666666.59339.peg.3555	None	None	fig|6666666.59342.peg.2799	None	None	fig|6666666.59345.peg.729	None	None	fig|6666666.59346.peg.4297	None	None	None	None	None	None	None

1837	Possible glyoxylase family protein Lactoylglutathione lyase EC 4.4.1.5	fig|6666666.59329.peg.5362	fig|6666666.59330.peg.3551	fig|6666666.59331.peg.2304	fig|6666666.59332.peg.2715	None	fig|6666666.59335.peg.2601	fig|6666666.59336.peg.2077	None	None	fig|6666666.59339.peg.2254	None	fig|6666666.59337.peg.2835	None	None	None	fig|6666666.59345.peg.1771	None	None	fig|6666666.59346.peg.2696	fig|6666666.59348.peg.4890	None	None	None	None	None	None

5839	hypothetical protein	None	None	fig|6666666.59331.peg.3466	None	None	fig|6666666.59335.peg.3878	fig|6666666.59336.peg.5587	fig|6666666.59333.peg.2969	fig|6666666.59338.peg.5422	fig|6666666.59339.peg.1079	fig|6666666.59340.peg.3287	None	fig|6666666.59342.peg.1852	None	None	fig|6666666.59345.peg.251	None	fig|6666666.59347.peg.1466	fig|6666666.59346.peg.3941	None	None	None	None	None	None	None

7741	hypothetical protein	fig|6666666.59329.peg.3354	None	None	None	None	None	fig|6666666.59336.peg.4519	None	fig|6666666.59338.peg.1680	None	None	None	fig|6666666.59342.peg.4766	fig|6666666.71181.peg.3437	fig|6666666.59344.peg.4920	fig|6666666.59345.peg.2982	fig|6666666.59341.peg.2845	fig|6666666.59347.peg.4702	None	None	None	None	None	fig|6666666.59353.peg.4613	fig|6666666.59355.peg.4718	None

8747	N-acetylneuraminate synthase EC 2.5.1.56	None	fig|6666666.59330.peg.1365	None	fig|6666666.59332.peg.3645	None	None	fig|6666666.59336.peg.93	None	fig|6666666.59338.peg.159	None	fig|6666666.59340.peg.810	None	fig|6666666.59342.peg.46	None	None	None	None	None	fig|6666666.59346.peg.4222	fig|6666666.59348.peg.4	fig|6666666.59349.peg.4848	None	fig|6666666.59351.peg.2030	None	None	fig|6666666.59354.peg.858

25	hypothetical protein	None	fig|6666666.59330.peg.1857	fig|6666666.59331.peg.148	fig|6666666.59332.peg.4939	None	fig|6666666.59335.peg.5087	fig|6666666.59336.peg.6330	None	fig|6666666.59338.peg.6052	fig|6666666.59339.peg.545	None	fig|6666666.59337.peg.3675	None	None	None	fig|6666666.59345.peg.1249	None	fig|6666666.59347.peg.5297	fig|6666666.59346.peg.446	None	None	None	None	None	None	None

183	FIG01201388 hypothetical protein	fig|6666666.59329.peg.288	fig|6666666.59330.peg.599	None	fig|6666666.59332.peg.1198	None	fig|6666666.59335.peg.168	fig|6666666.59336.peg.2827	fig|6666666.59333.peg.3129	None	fig|6666666.59339.peg.7715	None	fig|6666666.59337.peg.344	fig|6666666.59342.peg.3956	None	None	fig|6666666.59345.peg.2492	None	None	fig|6666666.59346.peg.1971	None	None	None	None	None	None	None

187	Mll0646 protein	fig|6666666.59329.peg.928	None	fig|6666666.59331.peg.1491	fig|6666666.59332.peg.2810	fig|6666666.59334.peg.5462	fig|6666666.59335.peg.1166	fig|6666666.59336.peg.388	None	fig|6666666.59338.peg.2192	fig|6666666.59339.peg.1688	fig|6666666.59340.peg.3373	None	fig|6666666.59342.peg.6167	None	None	fig|6666666.59345.peg.1819	None	None	None	None	None	None	None	None	None	None

2025	Urease accessory protein UreF	fig|6666666.59329.peg.2205	None	None	fig|6666666.59332.peg.3041	None	fig|6666666.59335.peg.4466	None	None	fig|6666666.59338.peg.3563	fig|6666666.59339.peg.5353	None	None	None	None	None	fig|6666666.59345.peg.4554	fig|6666666.59341.peg.4508	fig|6666666.59347.peg.570	fig|6666666.59346.peg.3219	fig|6666666.59348.peg.1494	None	fig|6666666.59350.peg.4191	None	None	None	None

1418	PhnB protein; putative DNA binding 3-demethylubiquinone-9 3-methyltransferase domain protein	None	None	fig|6666666.59331.peg.1839	fig|6666666.59332.peg.2870	fig|6666666.59334.peg.4662	fig|6666666.59335.peg.3980	None	None	None	fig|6666666.59339.peg.8905	None	None	None	fig|6666666.71181.peg.4062	fig|6666666.59344.peg.3765	None	None	None	fig|6666666.59346.peg.1658	fig|6666666.59348.peg.2806	None	None	None	fig|6666666.59353.peg.3920	fig|6666666.59355.peg.4242	None

6116	hypothetical protein	fig|6666666.59329.peg.5275	fig|6666666.59330.peg.4956	None	fig|6666666.59332.peg.1154	None	fig|6666666.59335.peg.4448	fig|6666666.59336.peg.4050	None	None	fig|6666666.59339.peg.2072	None	None	fig|6666666.59342.peg.3357	None	None	fig|6666666.59345.peg.5703	None	None	None	None	fig|6666666.59349.peg.3953	None	fig|6666666.59351.peg.5109	None	None	fig|6666666.59354.peg.2747

9541	Translation initiation inhibitor	fig|6666666.59329.peg.4068	fig|6666666.59330.peg.520	None	None	None	fig|6666666.59335.peg.5727	fig|6666666.59336.peg.5086	None	None	None	None	fig|6666666.59337.peg.5819	fig|6666666.59342.peg.2193	fig|6666666.71181.peg.2917	fig|6666666.59344.peg.3479	fig|6666666.59345.peg.4640	None	None	None	None	None	None	None	fig|6666666.59353.peg.1402	fig|6666666.59355.peg.2471	None

1654	Hydrogen cyanide synthase HcnA Opine oxidase subunit C	None	None	fig|6666666.59331.peg.2691	None	None	fig|6666666.59335.peg.3733	None	None	None	fig|6666666.59339.peg.2042	None	None	None	fig|6666666.71181.peg.343	fig|6666666.59344.peg.2343	None	None	None	None	None	fig|6666666.59349.peg.3938	fig|6666666.59350.peg.4478	fig|6666666.59351.peg.3238	fig|6666666.59353.peg.4200	fig|6666666.59355.peg.4164	fig|6666666.59354.peg.1632

9472	Integrase-like protein	None	fig|6666666.59330.peg.3879	fig|6666666.59331.peg.3830	fig|6666666.59332.peg.5238	fig|6666666.59334.peg.2269	fig|6666666.59335.peg.570	None	None	fig|6666666.59338.peg.3817	fig|6666666.59339.peg.6160	None	None	None	None	None	None	fig|6666666.59341.peg.5838	fig|6666666.59347.peg.963	fig|6666666.59346.peg.4423	fig|6666666.59348.peg.3863	None	None	None	None	None	None

2904	C1830 DhnA-type fructose-1,6-bisphosphate aldolase and related enzymes	None	None	None	None	None	None	None	None	fig|6666666.59338.peg.6041	None	None	None	None	fig|6666666.71181.peg.839	fig|6666666.59344.peg.38	None	fig|6666666.59341.peg.2974	fig|6666666.59347.peg.2054	None	fig|6666666.59348.peg.5430	fig|6666666.59349.peg.542	fig|6666666.59350.peg.6536	None	fig|6666666.59353.peg.376	fig|6666666.59355.peg.1413	fig|6666666.59354.peg.2992

406	FIG01201143 hypothetical protein	fig|6666666.59329.peg.2642	fig|6666666.59330.peg.846	fig|6666666.59331.peg.2767	fig|6666666.59332.peg.4603	None	None	fig|6666666.59336.peg.1344	fig|6666666.59333.peg.79	None	None	None	fig|6666666.59337.peg.3264	fig|6666666.59342.peg.1284	None	None	fig|6666666.59345.peg.528	fig|6666666.59341.peg.3386	None	fig|6666666.59346.peg.419	None	None	None	None	None	None	None

2452	Mannonate dehydratase EC 4.2.1.8	fig|6666666.59329.peg.2385	None	None	fig|6666666.59332.peg.2157	None	None	fig|6666666.59336.peg.5921	None	None	None	None	fig|6666666.59337.peg.314	None	fig|6666666.71181.peg.3172	fig|6666666.59344.peg.1679	fig|6666666.59345.peg.2104	fig|6666666.59341.peg.5724	fig|6666666.59347.peg.3827	None	None	None	None	None	fig|6666666.59353.peg.1098	fig|6666666.59355.peg.380	None

3822	T4-like phage baseplate hub + tail lysozyme	None	None	None	None	None	fig|6666666.59335.peg.296	None	None	None	fig|6666666.59339.peg.2547	None	fig|6666666.59337.peg.5404	None	fig|6666666.71181.peg.3952	fig|6666666.59344.peg.5438	None	None	None	None	fig|6666666.59348.peg.2942	fig|6666666.59349.peg.4786	None	fig|6666666.59351.peg.1503	fig|6666666.59353.peg.4809	fig|6666666.59355.peg.4863	fig|6666666.59354.peg.3347

4839	MSHA biogenesis protein MshP	fig|6666666.59329.peg.596	fig|6666666.59330.peg.22	None	fig|6666666.59332.peg.2049	None	fig|6666666.59335.peg.214	fig|6666666.59336.peg.1190	fig|6666666.59333.peg.4389	None	fig|6666666.59339.peg.7782	None	fig|6666666.59337.peg.807	None	None	None	fig|6666666.59345.peg.4888	fig|6666666.59341.peg.3667	None	fig|6666666.59346.peg.1962	None	None	None	None	None	None	None

10211	Flagellar hook-associated protein FliD	fig|6666666.59329.peg.2521	fig|6666666.59330.peg.4630	None	fig|6666666.59332.peg.2462	None	None	fig|6666666.59336.peg.1439	fig|6666666.59333.peg.5167	None	fig|6666666.59339.peg.7112	None	fig|6666666.59337.peg.2948	None	None	None	fig|6666666.59345.peg.5354	fig|6666666.59341.peg.5155	fig|6666666.59347.peg.2698	fig|6666666.59346.peg.4633	None	None	None	None	None	None	None

9990	hypothetical protein	None	None	fig|6666666.59331.peg.5577	fig|6666666.59332.peg.5747	None	None	fig|6666666.59336.peg.4664	fig|6666666.59333.peg.4678	fig|6666666.59338.peg.1882	None	None	fig|6666666.59337.peg.5583	None	None	None	fig|6666666.59345.peg.422	fig|6666666.59341.peg.1878	fig|6666666.59347.peg.539	fig|6666666.59346.peg.4102	fig|6666666.59348.peg.3947	None	None	None	None	None	None

2335	4-carboxymuconolactone decarboxylase domain alkylhydroperoxidase AhpD family core domain protein	None	None	None	None	fig|6666666.59334.peg.2946	fig|6666666.59335.peg.2466	None	None	None	fig|6666666.59339.peg.3572	None	None	None	fig|6666666.71181.peg.2618	fig|6666666.59344.peg.3883	None	None	None	None	None	fig|6666666.59349.peg.2139	fig|6666666.59350.peg.1332	fig|6666666.59351.peg.1738	fig|6666666.59353.peg.4693	fig|6666666.59355.peg.4075	fig|6666666.59354.peg.4195

7830	Uncharacterized protein ImpI VasC	fig|6666666.59329.peg.5976	fig|6666666.59330.peg.4138	None	None	None	None	None	None	fig|6666666.59338.peg.4066	None	None	fig|6666666.59337.peg.4773	fig|6666666.59342.peg.2557	fig|6666666.71181.peg.2267	fig|6666666.59344.peg.5869	fig|6666666.59345.peg.3438	None	fig|6666666.59347.peg.2427	None	None	None	None	None	fig|6666666.59353.peg.4905	fig|6666666.59355.peg.5322	None

522	Phosphoenolpyruvate-dihydroxyacetone phosphotransferase EC 2.7.1.121 , ADP-binding subunit DhaL	None	None	None	None	fig|6666666.59334.peg.1588	None	None	fig|6666666.59333.peg.106	fig|6666666.59338.peg.2023	None	None	None	fig|6666666.59342.peg.2363	fig|6666666.71181.peg.3029	fig|6666666.59344.peg.4447	None	fig|6666666.59341.peg.4805	fig|6666666.59347.peg.4234	None	fig|6666666.59348.peg.2453	None	None	None	fig|6666666.59353.peg.4167	fig|6666666.59355.peg.1710	None

5928	FIG01199745 hypothetical protein	fig|6666666.59329.peg.5160	None	fig|6666666.59331.peg.107	fig|6666666.59332.peg.1865	None	fig|6666666.59335.peg.5224	fig|6666666.59336.peg.2089	fig|6666666.59333.peg.3582	None	fig|6666666.59339.peg.3881	None	fig|6666666.59337.peg.3300	None	None	None	fig|6666666.59345.peg.4633	fig|6666666.59341.peg.812	None	fig|6666666.59346.peg.4573	None	None	None	None	None	None	None

4506	Uncharacterized protein conserved in bacteria	fig|6666666.59329.peg.1079	fig|6666666.59330.peg.2760	None	None	None	None	fig|6666666.59336.peg.1845	fig|6666666.59333.peg.3235	None	None	None	fig|6666666.59337.peg.323	None	fig|6666666.71181.peg.669	fig|6666666.59344.peg.4118	fig|6666666.59345.peg.2093	None	None	None	fig|6666666.59348.peg.2776	None	None	None	fig|6666666.59353.peg.4099	fig|6666666.59355.peg.2340	None

10876	Putative lipoprotein	None	None	fig|6666666.59331.peg.4027	fig|6666666.59332.peg.5031	fig|6666666.59334.peg.1267	None	None	None	fig|6666666.59338.peg.2524	None	None	None	None	fig|6666666.71181.peg.1897	fig|6666666.59344.peg.2547	None	fig|6666666.59341.peg.388	fig|6666666.59347.peg.2283	fig|6666666.59346.peg.5062	None	None	None	None	fig|6666666.59353.peg.1514	fig|6666666.59355.peg.2064	None

11337	FIG01200332 hypothetical protein	fig|6666666.59329.peg.6130	fig|6666666.59330.peg.883	None	None	fig|6666666.59334.peg.4296	fig|6666666.59335.peg.1846	fig|6666666.59336.peg.2067	None	fig|6666666.59338.peg.853	fig|6666666.59339.peg.2009	None	fig|6666666.59337.peg.5412	fig|6666666.59342.peg.1696	None	None	fig|6666666.59345.peg.565	None	fig|6666666.59347.peg.453	None	None	None	None	None	None	None	None

7576	putative ABC transporter	fig|6666666.59329.peg.1395	fig|6666666.59330.peg.1471	fig|6666666.59331.peg.4409	fig|6666666.59332.peg.890	None	None	fig|6666666.59336.peg.300	fig|6666666.59333.peg.1216	None	None	None	fig|6666666.59337.peg.205	None	None	None	fig|6666666.59345.peg.4571	fig|6666666.59341.peg.5178	None	fig|6666666.59346.peg.3850	fig|6666666.59348.peg.1468	None	None	None	None	None	None

4569	3R -hydroxymyristoyl-[ACP] dehydratase EC 4.2.1.-	fig|6666666.59329.peg.3343	None	fig|6666666.59331.peg.3015	fig|6666666.59332.peg.3638	None	None	fig|6666666.59336.peg.1050	None	None	None	None	None	None	fig|6666666.71181.peg.3934	fig|6666666.59344.peg.5384	None	fig|6666666.59341.peg.5331	None	fig|6666666.59346.peg.2123	fig|6666666.59348.peg.2537	None	None	None	fig|6666666.59353.peg.4791	fig|6666666.59355.peg.4845	None

864	DNA-directed RNA polymerase specialized sigma subunit	fig|6666666.59329.peg.639	fig|6666666.59330.peg.202	fig|6666666.59331.peg.1124	fig|6666666.59332.peg.47	None	None	fig|6666666.59336.peg.1485	fig|6666666.59333.peg.1178	fig|6666666.59338.peg.3486	None	None	fig|6666666.59337.peg.2789	None	None	None	fig|6666666.59345.peg.2744	None	fig|6666666.59347.peg.5211	fig|6666666.59346.peg.2394	None	None	None	None	None	None	None

4119	conserved hypothetical membrane protein, paralogue of Y20848	None	None	fig|6666666.59331.peg.2145	fig|6666666.59332.peg.2247	None	fig|6666666.59335.peg.1144	None	None	fig|6666666.59338.peg.5384	fig|6666666.59339.peg.1654	None	None	None	None	None	None	fig|6666666.59341.peg.4527	fig|6666666.59347.peg.3326	fig|6666666.59346.peg.5341	fig|6666666.59348.peg.2418	None	fig|6666666.59350.peg.6892	None	None	fig|6666666.59355.peg.5204	None

11157	FIG01200334 hypothetical protein	fig|6666666.59329.peg.1120	None	fig|6666666.59331.peg.1186	fig|6666666.59332.peg.763	fig|6666666.59334.peg.4477	None	fig|6666666.59336.peg.1648	None	None	None	None	fig|6666666.59337.peg.716	None	None	None	fig|6666666.59345.peg.3673	fig|6666666.59341.peg.3364	fig|6666666.59347.peg.4268	fig|6666666.59346.peg.4428	fig|6666666.59348.peg.4574	None	None	None	None	None	None

6007	Putative cytoplasmic protein	fig|6666666.59329.peg.582	None	fig|6666666.59331.peg.5768	fig|6666666.59332.peg.4513	fig|6666666.59334.peg.1549	None	fig|6666666.59336.peg.4565	fig|6666666.59333.peg.3717	fig|6666666.59338.peg.5845	None	None	None	fig|6666666.59342.peg.2626	None	None	fig|6666666.59345.peg.657	fig|6666666.59341.peg.3922	fig|6666666.59347.peg.449	None	None	None	None	None	None	None	None

5151	DNA-damage-inducible protein J	None	None	None	fig|6666666.59332.peg.611	fig|6666666.59334.peg.5370	None	fig|6666666.59336.peg.6488	fig|6666666.59333.peg.5446	fig|6666666.59338.peg.1885	None	None	fig|6666666.59337.peg.5740	None	fig|6666666.71181.peg.3516	fig|6666666.59344.peg.4624	None	None	fig|6666666.59347.peg.533	None	None	None	None	None	fig|6666666.59353.peg.2412	fig|6666666.59355.peg.503	None

274	Transcriptional regulator, AsnC family	fig|6666666.59329.peg.459	None	fig|6666666.59331.peg.3431	fig|6666666.59332.peg.5786	None	fig|6666666.59335.peg.5699	fig|6666666.59336.peg.2218	None	None	fig|6666666.59339.peg.4760	None	None	None	None	None	None	None	None	fig|6666666.59346.peg.5188	fig|6666666.59348.peg.4069	None	fig|6666666.59350.peg.1787	None	None	None	fig|6666666.59354.peg.5508

97	FIG01201945 hypothetical protein	fig|6666666.59329.peg.4055	fig|6666666.59330.peg.4954	None	None	None	None	fig|6666666.59336.peg.3014	fig|6666666.59333.peg.701	fig|6666666.59338.peg.4025	None	None	fig|6666666.59337.peg.3866	fig|6666666.59342.peg.1898	None	None	fig|6666666.59345.peg.303	fig|6666666.59341.peg.1829	fig|6666666.59347.peg.3593	None	None	None	None	None	None	None	None

7661	FIG01200679 hypothetical protein	fig|6666666.59329.peg.6337	fig|6666666.59330.peg.1971	None	None	None	fig|6666666.59335.peg.647	fig|6666666.59336.peg.3577	fig|6666666.59333.peg.5110	None	fig|6666666.59339.peg.4236	None	fig|6666666.59337.peg.2616	fig|6666666.59342.peg.3276	None	None	fig|6666666.59345.peg.736	None	None	None	fig|6666666.59348.peg.3691	None	None	None	None	None	None

9502	FIG01206467 hypothetical protein	fig|6666666.59329.peg.2872	fig|6666666.59330.peg.3611	fig|6666666.59331.peg.4171	None	None	None	fig|6666666.59336.peg.1157	None	None	None	None	fig|6666666.59337.peg.2993	fig|6666666.59342.peg.1343	None	None	fig|6666666.59345.peg.644	None	fig|6666666.59347.peg.2527	fig|6666666.59346.peg.4128	None	None	None	fig|6666666.59351.peg.4527	None	None	None

1151	Membrane protein with DUF350 domain	fig|6666666.59329.peg.1627	fig|6666666.59330.peg.3429	None	None	None	None	fig|6666666.59336.peg.5026	fig|6666666.59333.peg.1997	None	None	None	fig|6666666.59337.peg.4374	None	fig|6666666.71181.peg.2825	fig|6666666.59344.peg.2155	fig|6666666.59345.peg.1499	None	None	None	None	None	None	None	fig|6666666.59353.peg.3183	fig|6666666.59355.peg.413	None

4296	Suppression of copper sensitivity putative copper binding protein ScsA	fig|6666666.59329.peg.3395	fig|6666666.59330.peg.1373	None	None	None	fig|6666666.59335.peg.2288	fig|6666666.59336.peg.2604	fig|6666666.59333.peg.1667	None	fig|6666666.59339.peg.4147	None	fig|6666666.59337.peg.2904	None	None	None	fig|6666666.59345.peg.993	fig|6666666.59341.peg.5551	None	None	fig|6666666.59348.peg.2227	None	None	None	None	None	None

6189	Hypothetical with regulatory P domain of a subtilisin-like proprotein convertase	fig|6666666.59329.peg.5184	fig|6666666.59330.peg.754	fig|6666666.59331.peg.911	fig|6666666.59332.peg.2960	None	None	fig|6666666.59336.peg.539	fig|6666666.59333.peg.2082	None	None	None	fig|6666666.59337.peg.3484	fig|6666666.59342.peg.4353	None	None	fig|6666666.59345.peg.2772	None	None	fig|6666666.59346.peg.3438	None	None	None	None	None	None	None

10537	putative transposase	None	fig|6666666.59330.peg.1824	None	None	fig|6666666.59334.peg.4973	None	None	None	None	None	None	fig|6666666.59337.peg.1067	fig|6666666.59342.peg.404	fig|6666666.71181.peg.3705	None	fig|6666666.59345.peg.1650	fig|6666666.59341.peg.425	None	None	None	fig|6666666.59349.peg.1002	None	fig|6666666.59351.peg.2459	None	None	fig|6666666.59354.peg.584

9921	FIG01201687 hypothetical protein	fig|6666666.59329.peg.172	fig|6666666.59330.peg.2563	None	None	None	None	fig|6666666.59336.peg.4364	fig|6666666.59333.peg.5532	fig|6666666.59338.peg.1842	None	None	fig|6666666.59337.peg.5290	fig|6666666.59342.peg.6326	None	None	fig|6666666.59345.peg.2943	fig|6666666.59341.peg.4988	fig|6666666.59347.peg.5310	None	None	None	None	None	None	None	None

9819	Uncharacterized protein ImpA	fig|6666666.59329.peg.5409	fig|6666666.59330.peg.2151	None	None	None	fig|6666666.59335.peg.2696	None	fig|6666666.59333.peg.3920	None	fig|6666666.59339.peg.2732	fig|6666666.59340.peg.3324	fig|6666666.59337.peg.4076	fig|6666666.59342.peg.5193	None	None	fig|6666666.59345.peg.3381	None	None	None	fig|6666666.59348.peg.2013	None	None	None	None	None	None

5031	FIG022199 FAD-binding protein	fig|6666666.59329.peg.4803	None	fig|6666666.59331.peg.3033	fig|6666666.59332.peg.2629	None	None	fig|6666666.59336.peg.1059	fig|6666666.59333.peg.2908	None	None	None	None	None	None	None	None	None	None	fig|6666666.59346.peg.4730	fig|6666666.59348.peg.786	fig|6666666.59349.peg.4500	None	fig|6666666.59351.peg.3611	None	None	fig|6666666.59354.peg.3713

6296	hypothetical protein	None	None	None	fig|6666666.59332.peg.972	None	fig|6666666.59335.peg.4314	None	fig|6666666.59333.peg.2377	fig|6666666.59338.peg.2905	fig|6666666.59339.peg.1736	None	None	None	None	None	None	fig|6666666.59341.peg.3807	fig|6666666.59347.peg.3601	fig|6666666.59346.peg.2577	fig|6666666.59348.peg.1627	None	None	fig|6666666.59351.peg.3636	None	None	None

878	hypothetical protein	fig|6666666.59329.peg.3111	fig|6666666.59330.peg.700	fig|6666666.59331.peg.3035	fig|6666666.59332.peg.5398	fig|6666666.59334.peg.762	None	fig|6666666.59336.peg.1547	fig|6666666.59333.peg.4324	None	None	None	None	None	None	None	fig|6666666.59345.peg.4792	fig|6666666.59341.peg.4346	None	fig|6666666.59346.peg.165	None	None	None	None	None	None	None

3044	Aminotransferase ScrA	fig|6666666.59329.peg.4329	None	None	None	fig|6666666.59334.peg.2202	None	fig|6666666.59336.peg.2428	fig|6666666.59333.peg.4097	None	None	None	fig|6666666.59337.peg.1905	None	None	None	fig|6666666.59345.peg.2828	None	None	None	None	fig|6666666.59349.peg.4265	fig|6666666.59350.peg.4467	fig|6666666.59351.peg.5280	None	None	fig|6666666.59354.peg.1604

1092	Serine phosphatase RsbU, regulator of sigma subunit	fig|6666666.59329.peg.2999	fig|6666666.59330.peg.4551	None	fig|6666666.59332.peg.4624	None	None	fig|6666666.59336.peg.3651	fig|6666666.59333.peg.3208	fig|6666666.59338.peg.1520	None	None	None	fig|6666666.59342.peg.5093	None	None	None	fig|6666666.59341.peg.4115	fig|6666666.59347.peg.3286	fig|6666666.59346.peg.5125	None	None	None	None	None	None	None

3958	FIG01207175 hypothetical protein	fig|6666666.59329.peg.3722	None	None	fig|6666666.59332.peg.6233	fig|6666666.59334.peg.2395	None	fig|6666666.59336.peg.1866	None	fig|6666666.59338.peg.2451	None	None	fig|6666666.59337.peg.5417	fig|6666666.59342.peg.1700	None	None	fig|6666666.59345.peg.568	None	fig|6666666.59347.peg.1356	fig|6666666.59346.peg.3228	None	None	None	None	None	None	None

1541	transposase, putative	fig|6666666.59329.peg.2967	fig|6666666.59330.peg.221	fig|6666666.59331.peg.171	fig|6666666.59332.peg.61	fig|6666666.59334.peg.4052	fig|6666666.59335.peg.99	None	None	fig|6666666.59338.peg.857	None	None	fig|6666666.59337.peg.941	None	None	None	None	None	None	None	fig|6666666.59348.peg.37	None	None	fig|6666666.59351.peg.2223	None	None	None

8533	FIG01199674 hypothetical protein	fig|6666666.59329.peg.5062	fig|6666666.59330.peg.1686	None	fig|6666666.59332.peg.3470	None	fig|6666666.59335.peg.4749	fig|6666666.59336.peg.6486	fig|6666666.59333.peg.1850	None	None	None	fig|6666666.59337.peg.1689	fig|6666666.59342.peg.4981	None	None	fig|6666666.59345.peg.5392	None	None	None	fig|6666666.59348.peg.2449	None	None	None	None	None	None

2018	Electron transport complex protein RnfC	None	None	None	fig|6666666.59332.peg.4738	None	fig|6666666.59335.peg.888	fig|6666666.59336.peg.1331	fig|6666666.59333.peg.3624	None	fig|6666666.59339.peg.59	None	None	None	None	fig|6666666.59344.peg.3876	fig|6666666.59345.peg.1123	None	None	fig|6666666.59346.peg.5092	fig|6666666.59348.peg.92	None	None	fig|6666666.59351.peg.5077	None	None	None

10598	hypothetical protein	None	None	None	None	fig|6666666.59334.peg.1625	None	fig|6666666.59336.peg.4154	None	None	None	None	fig|6666666.59337.peg.3439	fig|6666666.59342.peg.5419	None	None	fig|6666666.59345.peg.812	fig|6666666.59341.peg.5431	None	None	None	fig|6666666.59349.peg.2455	fig|6666666.59350.peg.4680	fig|6666666.59351.peg.4648	None	None	fig|6666666.59354.peg.394

10251	Glutathione S-transferase domain protein	None	None	fig|6666666.59331.peg.3856	None	fig|6666666.59334.peg.3598	None	fig|6666666.59336.peg.4158	None	fig|6666666.59338.peg.1188	None	fig|6666666.59340.peg.2454	fig|6666666.59337.peg.3431	None	None	None	fig|6666666.59345.peg.199	None	fig|6666666.59347.peg.2123	fig|6666666.59346.peg.3174	None	None	fig|6666666.59350.peg.4799	None	None	None	None

587	Proteinase inhibitor I11, ecotin precursor	fig|6666666.59329.peg.1497	fig|6666666.59330.peg.4504	None	None	None	None	fig|6666666.59336.peg.757	None	None	None	None	fig|6666666.59337.peg.1264	fig|6666666.59342.peg.4184	fig|6666666.71181.peg.3860	fig|6666666.59344.peg.345	fig|6666666.59345.peg.4447	None	None	None	None	None	None	None	fig|6666666.59353.peg.543	fig|6666666.59355.peg.28	None

7868	putative nonspecific acid phosphatase precursor	fig|6666666.59329.peg.4292	None	fig|6666666.59331.peg.5408	fig|6666666.59332.peg.2983	fig|6666666.59334.peg.5414	None	fig|6666666.59336.peg.1528	fig|6666666.59333.peg.3466	None	None	None	fig|6666666.59337.peg.2101	fig|6666666.59342.peg.3407	None	None	fig|6666666.59345.peg.3337	fig|6666666.59341.peg.2684	None	None	None	None	None	None	None	None	None

5535	Ribosyl nicotinamide transporter, PnuC-like	None	None	None	None	None	None	fig|6666666.59336.peg.4855	fig|6666666.59333.peg.1244	None	None	None	fig|6666666.59337.peg.4626	None	fig|6666666.71181.peg.2496	None	fig|6666666.59345.peg.4807	None	None	None	None	fig|6666666.59349.peg.2424	None	fig|6666666.59351.peg.2701	fig|6666666.59353.peg.2105	fig|6666666.59355.peg.2716	fig|6666666.59354.peg.5871

5534	hypothetical protein	None	None	None	None	None	fig|6666666.59335.peg.5024	None	None	None	fig|6666666.59339.peg.1769	None	None	None	fig|6666666.71181.peg.6343	fig|6666666.59344.peg.2291	None	None	None	None	fig|6666666.59348.peg.870	fig|6666666.59349.peg.1896	None	fig|6666666.59351.peg.819	fig|6666666.59353.peg.3418	fig|6666666.59355.peg.3517	fig|6666666.59354.peg.3970

4821	Membrane protein, putative	fig|6666666.59329.peg.5302	None	fig|6666666.59331.peg.526	fig|6666666.59332.peg.3832	None	None	fig|6666666.59336.peg.4959	None	fig|6666666.59338.peg.2508	None	None	fig|6666666.59337.peg.1210	fig|6666666.59342.peg.1995	None	None	fig|6666666.59345.peg.4687	None	fig|6666666.59347.peg.1677	fig|6666666.59346.peg.3759	None	None	None	None	None	None	None

8854	FIG01202926 hypothetical protein	fig|6666666.59329.peg.2964	fig|6666666.59330.peg.220	fig|6666666.59331.peg.172	fig|6666666.59332.peg.59	fig|6666666.59334.peg.4274	fig|6666666.59335.peg.98	None	None	fig|6666666.59338.peg.859	None	fig|6666666.59340.peg.280	fig|6666666.59337.peg.940	None	None	None	None	None	None	None	fig|6666666.59348.peg.35	None	None	None	None	None	None

6962	Putative protease	fig|6666666.59329.peg.5299	None	None	None	None	None	fig|6666666.59336.peg.4272	None	None	fig|6666666.59339.peg.6908	None	None	fig|6666666.59342.peg.4186	None	fig|6666666.59344.peg.5113	None	fig|6666666.59341.peg.6001	None	fig|6666666.59346.peg.2547	None	fig|6666666.59349.peg.3076	None	None	None	fig|6666666.59355.peg.1721	fig|6666666.59354.peg.77

1387	Trp operon leader peptide	fig|6666666.59329.peg.403	fig|6666666.59330.peg.4898	fig|6666666.59331.peg.2408	fig|6666666.59332.peg.5106	None	None	fig|6666666.59336.peg.881	None	None	None	None	fig|6666666.59337.peg.1728	fig|6666666.59342.peg.765	None	None	fig|6666666.59345.peg.914	None	None	fig|6666666.59346.peg.1606	fig|6666666.59348.peg.4340	None	None	None	None	None	None

9345	Oligopeptide transport ATP-binding protein OppF TC 3.A.1.5.1	None	None	fig|6666666.59331.peg.3906	None	fig|6666666.59334.peg.4883	fig|6666666.59335.peg.6167	fig|6666666.59336.peg.4416	fig|6666666.59333.peg.4599	None	None	None	None	fig|6666666.59342.peg.3475	None	None	fig|6666666.59345.peg.1420	fig|6666666.59341.peg.576	fig|6666666.59347.peg.2496	fig|6666666.59346.peg.2214	None	None	None	None	None	None	None

5515	carboxyl-terminal protease	fig|6666666.59329.peg.4657	fig|6666666.59330.peg.1956	None	None	None	None	fig|6666666.59336.peg.5325	fig|6666666.59333.peg.3529	None	None	None	fig|6666666.59337.peg.2656	fig|6666666.59342.peg.4856	None	None	fig|6666666.59345.peg.701	None	None	None	None	fig|6666666.59349.peg.1179	None	fig|6666666.59351.peg.44	None	None	fig|6666666.59354.peg.3445

3296	hypothetical protein	fig|6666666.59329.peg.1555	None	fig|6666666.59331.peg.2448	fig|6666666.59332.peg.4833	fig|6666666.59334.peg.3313	None	fig|6666666.59336.peg.419	None	fig|6666666.59338.peg.4749	None	None	None	fig|6666666.59342.peg.5951	None	None	fig|6666666.59345.peg.1026	None	fig|6666666.59347.peg.1110	None	fig|6666666.59348.peg.2734	None	None	None	None	None	None

10833	Predicted zinc-binding protein	fig|6666666.59329.peg.287	fig|6666666.59330.peg.597	None	None	fig|6666666.59334.peg.353	None	fig|6666666.59336.peg.2826	fig|6666666.59333.peg.3128	fig|6666666.59338.peg.350	None	None	fig|6666666.59337.peg.345	None	None	None	None	None	fig|6666666.59347.peg.4863	None	fig|6666666.59348.peg.3609	None	fig|6666666.59350.peg.1853	None	None	None	None

10123	Transcriptional regulator, ArsR family	fig|6666666.59329.peg.3142	fig|6666666.59330.peg.3874	None	fig|6666666.59332.peg.2411	None	fig|6666666.59335.peg.280	fig|6666666.59336.peg.4442	fig|6666666.59333.peg.4766	None	None	None	fig|6666666.59337.peg.1948	fig|6666666.59342.peg.6480	None	None	fig|6666666.59345.peg.4053	None	None	fig|6666666.59346.peg.3487	None	None	None	None	None	None	None

9013	L-ornithine 5-monooxygenase EC 1.13.12.- , PvdA of pyoverdin biosynthesis	None	fig|6666666.59330.peg.4575	None	None	None	fig|6666666.59335.peg.4415	None	None	None	fig|6666666.59339.peg.1075	None	fig|6666666.59337.peg.3970	None	fig|6666666.71181.peg.885	fig|6666666.59344.peg.640	None	fig|6666666.59341.peg.5100	None	None	None	None	None	fig|6666666.59351.peg.31	fig|6666666.59353.peg.647	fig|6666666.59355.peg.129	None

6884	PhnH protein	None	None	None	None	None	None	None	None	fig|6666666.59338.peg.833	fig|6666666.59339.peg.7014	fig|6666666.59340.peg.2313	None	None	fig|6666666.71181.peg.3521	fig|6666666.59344.peg.4619	None	fig|6666666.59341.peg.4385	fig|6666666.59347.peg.107	None	None	None	fig|6666666.59350.peg.6628	None	fig|6666666.59353.peg.2407	fig|6666666.59355.peg.498	None

11045	Chromosome segregation ATPase	fig|6666666.59329.peg.637	fig|6666666.59330.peg.204	fig|6666666.59331.peg.1123	fig|6666666.59332.peg.49	None	None	fig|6666666.59336.peg.1484	fig|6666666.59333.peg.1179	None	None	None	fig|6666666.59337.peg.2791	None	None	None	fig|6666666.59345.peg.2743	None	fig|6666666.59347.peg.5210	fig|6666666.59346.peg.2815	None	None	None	None	None	None	None

2372	Hypothetical Nudix-like regulator	fig|6666666.59329.peg.4008	fig|6666666.59330.peg.4812	None	None	None	None	fig|6666666.59336.peg.3513	fig|6666666.59333.peg.2103	None	None	None	fig|6666666.59337.peg.2839	fig|6666666.59342.peg.4654	None	None	fig|6666666.59345.peg.1758	None	None	None	fig|6666666.59348.peg.2059	fig|6666666.59349.peg.1652	None	None	None	None	fig|6666666.59354.peg.759

2114	FIG01199963 hypothetical protein	fig|6666666.59329.peg.1155	fig|6666666.59330.peg.921	fig|6666666.59331.peg.549	fig|6666666.59332.peg.2954	None	None	fig|6666666.59336.peg.4772	fig|6666666.59333.peg.2170	None	None	None	fig|6666666.59337.peg.3947	fig|6666666.59342.peg.3262	None	None	fig|6666666.59345.peg.434	None	None	fig|6666666.59346.peg.3392	None	None	None	None	None	None	None

9829	Predicted esterase of the alpha-beta hydrolase superfamily	None	None	None	fig|6666666.59332.peg.3021	None	None	fig|6666666.59336.peg.6434	None	fig|6666666.59338.peg.6080	None	fig|6666666.59340.peg.3362	fig|6666666.59337.peg.2139	fig|6666666.59342.peg.5063	None	None	None	fig|6666666.59341.peg.42	None	fig|6666666.59346.peg.120	fig|6666666.59348.peg.2022	None	None	None	None	None	fig|6666666.59354.peg.4431

5583	Magnesium and cobalt transport protein CorA	None	None	fig|6666666.59331.peg.2042	None	None	None	None	fig|6666666.59333.peg.3445	None	None	None	None	None	fig|6666666.71181.peg.1374	fig|6666666.59344.peg.548	None	None	None	fig|6666666.59346.peg.2837	None	fig|6666666.59349.peg.2421	None	fig|6666666.59351.peg.2698	fig|6666666.59353.peg.1853	fig|6666666.59355.peg.1160	fig|6666666.59354.peg.5050

6595	N-acetylneuraminic acid outer membrane channel protein NanC	None	None	fig|6666666.59331.peg.4259	None	fig|6666666.59334.peg.4245	None	fig|6666666.59336.peg.381	None	fig|6666666.59338.peg.3906	None	fig|6666666.59340.peg.915	None	fig|6666666.59342.peg.2066	None	None	fig|6666666.59345.peg.1381	fig|6666666.59341.peg.2338	fig|6666666.59347.peg.1077	fig|6666666.59346.peg.5114	None	None	None	None	None	None	None

1388	Copper-sensing two-component system response regulator CpxR	fig|6666666.59329.peg.5550	fig|6666666.59330.peg.5130	fig|6666666.59331.peg.6046	fig|6666666.59332.peg.4546	None	fig|6666666.59335.peg.2671	fig|6666666.59336.peg.6027	None	None	fig|6666666.59339.peg.2672	None	None	fig|6666666.59342.peg.673	None	None	fig|6666666.59345.peg.3305	None	None	None	fig|6666666.59348.peg.1434	None	None	None	None	None	None

8004	FIG021862 membrane protein, exporter	fig|6666666.59329.peg.2404	None	fig|6666666.59331.peg.2607	fig|6666666.59332.peg.3887	fig|6666666.59334.peg.4553	None	fig|6666666.59336.peg.5614	None	fig|6666666.59338.peg.764	None	fig|6666666.59340.peg.3718	None	None	None	None	None	fig|6666666.59341.peg.4970	None	fig|6666666.59346.peg.2115	fig|6666666.59348.peg.1819	None	None	None	None	None	None

101	Predicted permease	fig|6666666.59329.peg.4686	fig|6666666.59330.peg.2199	None	None	None	fig|6666666.59335.peg.2423	fig|6666666.59336.peg.2553	fig|6666666.59333.peg.2061	None	fig|6666666.59339.peg.5261	None	fig|6666666.59337.peg.3916	fig|6666666.59342.peg.4085	None	None	fig|6666666.59345.peg.4353	None	None	None	fig|6666666.59348.peg.1277	None	None	None	None	None	None

10247	Hypothetical protein in cluster with HutR, VCA0067 homolog	fig|6666666.59329.peg.4994	None	fig|6666666.59331.peg.1431	fig|6666666.59332.peg.2220	None	None	fig|6666666.59336.peg.537	fig|6666666.59333.peg.2085	None	None	None	fig|6666666.59337.peg.3486	None	None	None	fig|6666666.59345.peg.2770	None	None	fig|6666666.59346.peg.4485	None	fig|6666666.59349.peg.1778	None	fig|6666666.59351.peg.2305	None	None	None

246	FIG01200645 hypothetical protein	fig|6666666.59329.peg.4805	None	None	fig|6666666.59332.peg.2627	None	None	None	fig|6666666.59333.peg.2906	None	None	None	None	None	fig|6666666.71181.peg.3941	fig|6666666.59344.peg.5378	None	fig|6666666.59341.peg.2752	None	fig|6666666.59346.peg.4724	fig|6666666.59348.peg.783	None	None	None	fig|6666666.59353.peg.4798	fig|6666666.59355.peg.4852	None

5876	FIG01201986 hypothetical protein	fig|6666666.59329.peg.5212	fig|6666666.59330.peg.4567	None	None	None	fig|6666666.59335.peg.2678	fig|6666666.59336.peg.6154	fig|6666666.59333.peg.3647	None	fig|6666666.59339.peg.2687	fig|6666666.59340.peg.1538	fig|6666666.59337.peg.4811	fig|6666666.59342.peg.5841	None	None	fig|6666666.59345.peg.3359	None	None	None	None	None	None	None	None	None	None

9188	Endoglucanase precursor EC 3.2.1.4	None	fig|6666666.59330.peg.2190	None	None	None	None	fig|6666666.59336.peg.1515	None	None	None	fig|6666666.59340.peg.120	fig|6666666.59337.peg.5060	fig|6666666.59342.peg.4683	fig|6666666.71181.peg.4267	fig|6666666.59344.peg.2443	fig|6666666.59345.peg.4370	None	None	None	None	None	None	None	fig|6666666.59353.peg.3664	fig|6666666.59355.peg.4035	None

6229	Urease accessory protein UreD	fig|6666666.59329.peg.5868	None	None	None	None	fig|6666666.59335.peg.5941	None	None	fig|6666666.59338.peg.1467	fig|6666666.59339.peg.5364	None	None	None	None	None	fig|6666666.59345.peg.4561	fig|6666666.59341.peg.244	fig|6666666.59347.peg.564	fig|6666666.59346.peg.1303	fig|6666666.59348.peg.546	None	fig|6666666.59350.peg.3561	None	None	None	None

9705	Metal-dependent hydrolases of the beta-lactamase superfamily I; PhnP protein	None	None	None	None	None	None	None	None	fig|6666666.59338.peg.2999	fig|6666666.59339.peg.7027	fig|6666666.59340.peg.398	None	None	fig|6666666.71181.peg.3529	fig|6666666.59344.peg.2068	None	fig|6666666.59341.peg.4331	fig|6666666.59347.peg.100	None	None	None	fig|6666666.59350.peg.2221	None	fig|6666666.59353.peg.3107	fig|6666666.59355.peg.5390	None

5669	HipB protein	fig|6666666.59329.peg.404	None	None	fig|6666666.59332.peg.4359	fig|6666666.59334.peg.5469	None	None	None	fig|6666666.59338.peg.1072	None	None	None	None	fig|6666666.71181.peg.2985	fig|6666666.59344.peg.3984	fig|6666666.59345.peg.3592	None	fig|6666666.59347.peg.4320	None	None	None	None	None	fig|6666666.59353.peg.2449	fig|6666666.59355.peg.542	None

19	hypothetical protein	None	None	fig|6666666.59331.peg.3566	fig|6666666.59332.peg.3653	None	None	None	None	fig|6666666.59338.peg.5361	None	None	None	None	fig|6666666.71181.peg.1411	fig|6666666.59344.peg.1582	None	fig|6666666.59341.peg.1288	fig|6666666.59347.peg.1673	fig|6666666.59346.peg.2219	None	None	None	None	fig|6666666.59353.peg.2666	fig|6666666.59355.peg.1816	None

4401	hypothetical protein	fig|6666666.59329.peg.3801	None	fig|6666666.59331.peg.3084	fig|6666666.59332.peg.4364	fig|6666666.59334.peg.3832	None	fig|6666666.59336.peg.4036	None	fig|6666666.59338.peg.1684	None	fig|6666666.59340.peg.356	None	fig|6666666.59342.peg.2994	None	None	fig|6666666.59345.peg.3972	None	fig|6666666.59347.peg.4699	None	None	None	None	None	None	None	None

4798	2-pyrone-4,6-dicarboxylic acid hydrolase	None	None	fig|6666666.59331.peg.345	fig|6666666.59332.peg.518	fig|6666666.59334.peg.2085	None	None	fig|6666666.59333.peg.982	None	None	None	None	None	fig|6666666.71181.peg.3867	fig|6666666.59344.peg.864	None	None	None	fig|6666666.59346.peg.182	None	None	fig|6666666.59350.peg.3478	None	fig|6666666.59353.peg.1254	fig|6666666.59355.peg.3331	None

1069	N- 3-hydroxybutanoyl -L- homoserine lactone synthase LuxM	fig|6666666.59329.peg.394	fig|6666666.59330.peg.3341	None	None	None	fig|6666666.59335.peg.3840	fig|6666666.59336.peg.2661	fig|6666666.59333.peg.1771	None	fig|6666666.59339.peg.1976	None	fig|6666666.59337.peg.3504	fig|6666666.59342.peg.1720	None	None	fig|6666666.59345.peg.922	None	fig|6666666.59347.peg.1420	None	None	None	None	None	None	None	None

9918	hypothetical protein	fig|6666666.59329.peg.1553	None	fig|6666666.59331.peg.2450	fig|6666666.59332.peg.4831	fig|6666666.59334.peg.3315	None	fig|6666666.59336.peg.421	None	fig|6666666.59338.peg.5948	None	None	None	fig|6666666.59342.peg.5952	None	None	fig|6666666.59345.peg.1028	None	fig|6666666.59347.peg.1108	None	fig|6666666.59348.peg.2737	None	None	None	None	None	None

968	Ribokinase EC 2.7.1.15	None	None	fig|6666666.59331.peg.444	None	None	None	fig|6666666.59336.peg.5226	None	None	fig|6666666.59339.peg.4576	None	None	None	fig|6666666.71181.peg.1254	fig|6666666.59344.peg.151	None	fig|6666666.59341.peg.4728	None	fig|6666666.59346.peg.1267	fig|6666666.59348.peg.865	None	None	None	fig|6666666.59353.peg.463	fig|6666666.59355.peg.2190	None

7112	hypothetical protein	None	None	None	fig|6666666.59332.peg.3128	None	fig|6666666.59335.peg.4025	None	None	fig|6666666.59338.peg.2897	fig|6666666.59339.peg.3818	fig|6666666.59340.peg.3396	None	None	None	None	None	fig|6666666.59341.peg.2906	None	None	fig|6666666.59348.peg.1960	fig|6666666.59349.peg.4481	None	fig|6666666.59351.peg.2892	None	None	fig|6666666.59354.peg.321

4892	PhnI protein	None	None	None	None	None	None	None	None	fig|6666666.59338.peg.834	fig|6666666.59339.peg.7015	fig|6666666.59340.peg.2312	None	None	fig|6666666.71181.peg.3522	fig|6666666.59344.peg.4618	None	fig|6666666.59341.peg.4384	fig|6666666.59347.peg.106	None	None	None	fig|6666666.59350.peg.6629	None	fig|6666666.59353.peg.2406	fig|6666666.59355.peg.497	None

3427	hypothetical protein	fig|6666666.59329.peg.474	fig|6666666.59330.peg.2742	None	None	None	None	fig|6666666.59336.peg.6078	fig|6666666.59333.peg.3138	fig|6666666.59338.peg.4024	None	None	fig|6666666.59337.peg.5533	fig|6666666.59342.peg.702	None	None	fig|6666666.59345.peg.4482	fig|6666666.59341.peg.1827	fig|6666666.59347.peg.3592	None	None	None	None	None	None	None	None

5691	L-asparaginase EC 3.5.1.1	fig|6666666.59329.peg.3238	None	None	None	fig|6666666.59334.peg.1892	None	fig|6666666.59336.peg.3287	fig|6666666.59333.peg.1310	None	None	None	fig|6666666.59337.peg.4078	None	fig|6666666.71181.peg.3163	fig|6666666.59344.peg.2908	fig|6666666.59345.peg.1675	None	None	None	None	None	None	None	fig|6666666.59353.peg.1643	fig|6666666.59355.peg.3191	None

6482	FIG01200392 hypothetical protein	fig|6666666.59329.peg.6082	None	None	None	None	None	fig|6666666.59336.peg.6553	fig|6666666.59333.peg.5409	None	None	None	fig|6666666.59337.peg.4962	fig|6666666.59342.peg.4373	fig|6666666.71181.peg.76	fig|6666666.59344.peg.3190	fig|6666666.59345.peg.116	None	None	None	None	None	None	None	fig|6666666.59353.peg.1939	fig|6666666.59355.peg.1468	None

5310	FIG01201735 hypothetical protein	fig|6666666.59329.peg.4036	fig|6666666.59330.peg.2250	fig|6666666.59331.peg.4443	fig|6666666.59332.peg.5309	None	None	fig|6666666.59336.peg.1111	fig|6666666.59333.peg.1674	None	None	None	fig|6666666.59337.peg.5057	fig|6666666.59342.peg.6131	None	None	fig|6666666.59345.peg.1896	None	None	fig|6666666.59346.peg.5046	None	None	None	None	None	None	None

3236	N6-hydroxylysine O-acetyltransferase EC 2.3.1.102 , aerobactin biosynthesis protein IucB Siderophore synthetase small component, acetyltransferase	None	None	None	None	None	fig|6666666.59335.peg.4414	fig|6666666.59336.peg.3861	None	None	fig|6666666.59339.peg.1819	None	None	None	fig|6666666.71181.peg.887	fig|6666666.59344.peg.4764	None	fig|6666666.59341.peg.6031	None	None	fig|6666666.59348.peg.4162	None	None	fig|6666666.59351.peg.4073	fig|6666666.59353.peg.4546	fig|6666666.59355.peg.4651	None

2268	Uncharacterized conserved protein	None	fig|6666666.59330.peg.4171	fig|6666666.59331.peg.2472	fig|6666666.59332.peg.3361	None	fig|6666666.59335.peg.3622	None	None	None	fig|6666666.59339.peg.4299	None	fig|6666666.59337.peg.2462	fig|6666666.59342.peg.3208	None	None	fig|6666666.59345.peg.591	None	None	fig|6666666.59346.peg.3909	None	None	fig|6666666.59350.peg.3052	None	None	None	None

10217	Putative lipase	None	None	fig|6666666.59331.peg.1796	None	None	fig|6666666.59335.peg.6197	fig|6666666.59336.peg.6047	None	None	fig|6666666.59339.peg.1840	None	None	None	fig|6666666.71181.peg.4428	fig|6666666.59344.peg.2611	None	None	None	fig|6666666.59346.peg.429	None	None	fig|6666666.59350.peg.1222	None	fig|6666666.59353.peg.1567	fig|6666666.59355.peg.2115	None

10216	Xylose isomerase EC 5.3.1.5	None	None	None	fig|6666666.59332.peg.4480	fig|6666666.59334.peg.3804	None	None	None	fig|6666666.59338.peg.5491	None	fig|6666666.59340.peg.1854	None	None	fig|6666666.71181.peg.4714	fig|6666666.59344.peg.5980	None	None	fig|6666666.59347.peg.3374	None	None	None	fig|6666666.59350.peg.3647	None	fig|6666666.59353.peg.3582	fig|6666666.59355.peg.4374	None

4998	hypothetical protein	fig|6666666.59329.peg.4965	fig|6666666.59330.peg.5128	fig|6666666.59331.peg.4550	fig|6666666.59332.peg.606	None	fig|6666666.59335.peg.3779	fig|6666666.59336.peg.2981	None	fig|6666666.59338.peg.1259	None	None	fig|6666666.59337.peg.4881	fig|6666666.59342.peg.1972	None	None	fig|6666666.59345.peg.177	None	None	None	None	None	None	None	None	None	None

7035	PUTATIVE PERIPLASMIC PROTEIN	fig|6666666.59329.peg.2215	fig|6666666.59330.peg.3135	None	None	None	None	fig|6666666.59336.peg.3950	fig|6666666.59333.peg.5395	None	fig|6666666.59339.peg.6089	None	fig|6666666.59337.peg.1420	fig|6666666.59342.peg.5811	None	None	fig|6666666.59345.peg.3861	None	None	None	None	fig|6666666.59349.peg.4157	None	None	None	None	fig|6666666.59354.peg.4065

9134	FIG002337 predicted inner membrane protein	None	fig|6666666.59330.peg.3042	None	None	None	None	fig|6666666.59336.peg.4725	None	None	None	fig|6666666.59340.peg.2375	fig|6666666.59337.peg.4915	fig|6666666.59342.peg.97	fig|6666666.71181.peg.5194	fig|6666666.59344.peg.5275	fig|6666666.59345.peg.4380	None	None	None	None	None	None	None	fig|6666666.59353.peg.3624	fig|6666666.59355.peg.4809	None

3577	hypothetical protein	None	None	None	fig|6666666.59332.peg.1375	fig|6666666.59334.peg.628	fig|6666666.59335.peg.5269	fig|6666666.59336.peg.4334	None	fig|6666666.59338.peg.4764	None	None	None	fig|6666666.59342.peg.989	None	None	fig|6666666.59345.peg.4230	fig|6666666.59341.peg.3228	fig|6666666.59347.peg.71	None	None	None	fig|6666666.59350.peg.5308	None	None	None	None

500	FIG00641944 hypothetical protein	None	fig|6666666.59330.peg.4022	None	None	fig|6666666.59334.peg.2890	None	None	fig|6666666.59333.peg.3729	None	None	None	fig|6666666.59337.peg.2933	fig|6666666.59342.peg.2716	fig|6666666.71181.peg.2102	fig|6666666.59344.peg.5296	fig|6666666.59345.peg.2076	None	None	None	None	None	None	None	fig|6666666.59353.peg.691	fig|6666666.59355.peg.177	None

755	Response regulator	fig|6666666.59329.peg.271	fig|6666666.59330.peg.1819	fig|6666666.59331.peg.176	None	fig|6666666.59334.peg.5017	fig|6666666.59335.peg.2221	None	None	None	None	None	fig|6666666.59337.peg.5660	fig|6666666.59342.peg.9	None	None	fig|6666666.59345.peg.1647	fig|6666666.59341.peg.5690	None	None	fig|6666666.59348.peg.624	None	None	None	None	None	None

1022	Transcriptional regulator	fig|6666666.59329.peg.4562	fig|6666666.59330.peg.2414	fig|6666666.59331.peg.427	fig|6666666.59332.peg.3877	None	fig|6666666.59335.peg.4161	None	fig|6666666.59333.peg.2582	None	fig|6666666.59339.peg.7046	None	None	fig|6666666.59342.peg.2091	None	None	fig|6666666.59345.peg.3583	None	None	fig|6666666.59346.peg.701	None	None	None	None	None	None	None

5731	3-oxoacyl-[acyl-carrier protein] reductase EC 1.1.1.100 , paralog	None	None	fig|6666666.59331.peg.1093	fig|6666666.59332.peg.340	None	fig|6666666.59335.peg.6047	fig|6666666.59336.peg.4485	None	None	fig|6666666.59339.peg.1262	None	None	fig|6666666.59342.peg.3895	None	None	fig|6666666.59345.peg.3900	fig|6666666.59341.peg.623	None	fig|6666666.59346.peg.173	fig|6666666.59348.peg.3382	None	None	None	None	None	None

6657	F TPR repeat protein	fig|6666666.59329.peg.6085	fig|6666666.59330.peg.4287	fig|6666666.59331.peg.2021	fig|6666666.59332.peg.4662	None	None	None	None	None	fig|6666666.59339.peg.3778	None	fig|6666666.59337.peg.4031	None	fig|6666666.71181.peg.4366	None	None	fig|6666666.59341.peg.937	None	None	None	None	None	None	fig|6666666.59353.peg.4913	fig|6666666.59355.peg.4983	None

10274	Similarity with glutathionylspermidine synthase EC 6.3.1.8 , group 1	fig|6666666.59329.peg.1629	fig|6666666.59330.peg.3427	None	None	None	None	fig|6666666.59336.peg.5024	fig|6666666.59333.peg.2287	None	None	None	fig|6666666.59337.peg.4372	None	fig|6666666.71181.peg.3025	fig|6666666.59344.peg.4451	fig|6666666.59345.peg.1497	None	None	None	None	None	None	None	fig|6666666.59353.peg.4171	fig|6666666.59355.peg.1706	None

6875	FIG01201380 hypothetical protein	fig|6666666.59329.peg.1062	fig|6666666.59330.peg.183	None	None	fig|6666666.59334.peg.37	fig|6666666.59335.peg.456	fig|6666666.59336.peg.1298	fig|6666666.59333.peg.2209	None	fig|6666666.59339.peg.6461	None	fig|6666666.59337.peg.4299	None	None	None	fig|6666666.59345.peg.5135	None	None	None	fig|6666666.59348.peg.3667	None	None	None	None	None	None

7109	TonB-dependent heme and hemoglobin receptor HutA ; TonB-dependent hemin , ferrichrome receptor	fig|6666666.59329.peg.2542	None	None	fig|6666666.59332.peg.5914	None	fig|6666666.59335.peg.2281	fig|6666666.59336.peg.6003	fig|6666666.59333.peg.2700	fig|6666666.59338.peg.3209	None	None	None	fig|6666666.59342.peg.3619	None	None	None	None	None	fig|6666666.59346.peg.5108	fig|6666666.59348.peg.4368	None	None	None	None	None	fig|6666666.59354.peg.1181

6025	Response regulator NasT	None	None	None	None	None	fig|6666666.59335.peg.2889	fig|6666666.59336.peg.3023	fig|6666666.59333.peg.720	fig|6666666.59338.peg.871	fig|6666666.59339.peg.4678	None	fig|6666666.59337.peg.5457	fig|6666666.59342.peg.4150	None	None	fig|6666666.59345.peg.286	None	fig|6666666.59347.peg.5147	None	fig|6666666.59348.peg.1983	None	None	None	None	None	None

4321	Exodeoxyribonuclease V beta chain EC 3.1.11.5	None	None	None	fig|6666666.59332.peg.5345	None	fig|6666666.59335.peg.1123	None	fig|6666666.59333.peg.4488	None	fig|6666666.59339.peg.6952	fig|6666666.59340.peg.994	None	None	None	fig|6666666.59344.peg.5799	None	fig|6666666.59341.peg.525	None	None	None	None	None	None	fig|6666666.59353.peg.1955	fig|6666666.59355.peg.1452	fig|6666666.59354.peg.3080

6619	FIG01200445 hypothetical protein	fig|6666666.59329.peg.2252	fig|6666666.59330.peg.3173	fig|6666666.59331.peg.3751	None	None	None	fig|6666666.59336.peg.5639	fig|6666666.59333.peg.2790	None	None	None	fig|6666666.59337.peg.4564	fig|6666666.59342.peg.2338	None	None	fig|6666666.59345.peg.3244	None	None	fig|6666666.59346.peg.5101	fig|6666666.59348.peg.4459	None	None	None	None	None	None

3501	putative membrane protein	None	None	None	fig|6666666.59332.peg.324	None	fig|6666666.59335.peg.2607	None	fig|6666666.59333.peg.3681	None	None	None	fig|6666666.59337.peg.4887	None	fig|6666666.71181.peg.2718	None	None	None	None	None	fig|6666666.59348.peg.5334	fig|6666666.59349.peg.2129	None	None	fig|6666666.59353.peg.3960	fig|6666666.59355.peg.3875	fig|6666666.59354.peg.1970

7624	Putative exported protein	None	None	fig|6666666.59331.peg.54	None	None	None	None	fig|6666666.59333.peg.5374	None	None	None	None	None	fig|6666666.71181.peg.464	fig|6666666.59344.peg.1382	None	None	None	None	None	fig|6666666.59349.peg.1323	fig|6666666.59350.peg.7789	fig|6666666.59351.peg.4544	fig|6666666.59353.peg.32	fig|6666666.59355.peg.639	fig|6666666.59354.peg.153

7620	Oligopeptide transport system permease protein OppB TC 3.A.1.5.1	fig|6666666.59329.peg.3777	None	fig|6666666.59331.peg.974	fig|6666666.59332.peg.5873	None	fig|6666666.59335.peg.4918	fig|6666666.59336.peg.3937	fig|6666666.59333.peg.3724	fig|6666666.59338.peg.2269	None	None	None	None	None	None	fig|6666666.59345.peg.1418	None	fig|6666666.59347.peg.2498	fig|6666666.59346.peg.2211	None	None	None	None	None	None	None

3160	hypothetical protein	None	None	None	None	fig|6666666.59334.peg.5199	fig|6666666.59335.peg.3888	fig|6666666.59336.peg.3884	None	None	fig|6666666.59339.peg.2431	None	None	fig|6666666.59342.peg.4364	None	None	fig|6666666.59345.peg.227	fig|6666666.59341.peg.3102	fig|6666666.59347.peg.1659	None	fig|6666666.59348.peg.5534	None	fig|6666666.59350.peg.969	None	None	None	None

10556	Fosfomycin resistance protein FosA	None	fig|6666666.59330.peg.5142	None	None	None	fig|6666666.59335.peg.2637	fig|6666666.59336.peg.1755	None	None	fig|6666666.59339.peg.1835	None	fig|6666666.59337.peg.1835	fig|6666666.59342.peg.6460	None	None	fig|6666666.59345.peg.4218	None	None	None	None	fig|6666666.59349.peg.4283	None	fig|6666666.59351.peg.4554	None	None	fig|6666666.59354.peg.2726

8482	FIG01200099 hypothetical protein	fig|6666666.59329.peg.519	fig|6666666.59330.peg.218	fig|6666666.59331.peg.174	fig|6666666.59332.peg.57	fig|6666666.59334.peg.3386	fig|6666666.59335.peg.96	None	None	fig|6666666.59338.peg.861	None	fig|6666666.59340.peg.2658	fig|6666666.59337.peg.937	None	None	None	None	None	None	None	fig|6666666.59348.peg.33	None	None	None	None	None	None

2687	NADP-specific glutamate dehydrogenase EC 1.4.1.4	None	None	None	None	None	None	None	fig|6666666.59333.peg.2734	None	None	None	None	None	fig|6666666.71181.peg.1064	fig|6666666.59344.peg.4967	None	None	None	None	None	fig|6666666.59349.peg.643	fig|6666666.59350.peg.4167	fig|6666666.59351.peg.2738	fig|6666666.59353.peg.4511	fig|6666666.59355.peg.4621	fig|6666666.59354.peg.3194

6810	Thioesterase	None	fig|6666666.59330.peg.4576	None	None	None	fig|6666666.59335.peg.5443	None	None	None	None	None	fig|6666666.59337.peg.3969	None	None	fig|6666666.59344.peg.647	None	None	None	None	fig|6666666.59348.peg.5208	fig|6666666.59349.peg.4659	None	fig|6666666.59351.peg.2861	None	fig|6666666.59355.peg.2353	fig|6666666.59354.peg.6464

5057	hypothetical protein	fig|6666666.59329.peg.3765	None	None	None	fig|6666666.59334.peg.4559	fig|6666666.59335.peg.5553	fig|6666666.59336.peg.4974	None	None	fig|6666666.59339.peg.2369	None	None	None	None	None	None	fig|6666666.59341.peg.4315	None	None	None	fig|6666666.59349.peg.4022	None	fig|6666666.59351.peg.1743	None	None	fig|6666666.59354.peg.1010

9006	putative exported protein	fig|6666666.59329.peg.3887	fig|6666666.59330.peg.4468	None	None	None	fig|6666666.59335.peg.6158	fig|6666666.59336.peg.5257	fig|6666666.59333.peg.4671	None	fig|6666666.59339.peg.8807	None	fig|6666666.59337.peg.5252	None	None	None	fig|6666666.59345.peg.2060	None	None	None	fig|6666666.59348.peg.5214	None	None	None	None	None	None

155	FIG01200531 hypothetical protein	None	fig|6666666.59330.peg.4098	None	None	None	fig|6666666.59335.peg.4386	fig|6666666.59336.peg.5577	fig|6666666.59333.peg.821	None	fig|6666666.59339.peg.4121	None	fig|6666666.59337.peg.2859	fig|6666666.59342.peg.2548	None	None	fig|6666666.59345.peg.1732	None	None	None	fig|6666666.59348.peg.4921	None	None	None	None	None	None

6183	hypothetical protein	None	None	None	None	fig|6666666.59334.peg.5320	None	None	fig|6666666.59333.peg.2264	fig|6666666.59338.peg.5012	None	None	None	fig|6666666.59342.peg.728	fig|6666666.71181.peg.2936	fig|6666666.59344.peg.3498	None	None	fig|6666666.59347.peg.1667	None	None	None	None	None	fig|6666666.59353.peg.1383	fig|6666666.59355.peg.2489	None

10374	hypothetical protein	fig|6666666.59329.peg.5271	None	None	fig|6666666.59332.peg.1456	None	fig|6666666.59335.peg.1388	None	None	fig|6666666.59338.peg.892	fig|6666666.59339.peg.6745	fig|6666666.59340.peg.999	None	None	None	None	None	fig|6666666.59341.peg.2408	fig|6666666.59347.peg.5362	fig|6666666.59346.peg.4857	None	None	None	None	None	None	None

6231	FIG01201234 hypothetical protein	fig|6666666.59329.peg.1424	None	fig|6666666.59331.peg.2962	fig|6666666.59332.peg.2036	fig|6666666.59334.peg.5498	None	fig|6666666.59336.peg.2839	None	fig|6666666.59338.peg.2757	None	None	None	None	None	None	fig|6666666.59345.peg.2975	None	fig|6666666.59347.peg.3409	fig|6666666.59346.peg.1167	None	None	None	None	None	None	None

10697	Uncharacterized protein similar to VCA0109	None	fig|6666666.59330.peg.2158	None	None	None	fig|6666666.59335.peg.2689	fig|6666666.59336.peg.3829	fig|6666666.59333.peg.3396	None	fig|6666666.59339.peg.2721	None	fig|6666666.59337.peg.5017	fig|6666666.59342.peg.3110	None	None	fig|6666666.59345.peg.3376	None	None	None	fig|6666666.59348.peg.4492	None	None	None	None	None	None

9519	FIG01058644 hypothetical protein	None	fig|6666666.59330.peg.4756	fig|6666666.59331.peg.150	fig|6666666.59332.peg.5431	fig|6666666.59334.peg.4371	fig|6666666.59335.peg.4938	None	None	None	fig|6666666.59339.peg.630	None	None	None	None	None	fig|6666666.59345.peg.803	None	None	fig|6666666.59346.peg.443	None	None	fig|6666666.59350.peg.6849	None	None	None	None

5808	Anaerobic dimethyl sulfoxide reductase chain C EC 1.8.99.-	None	None	None	None	fig|6666666.59334.peg.3556	None	fig|6666666.59336.peg.2283	None	fig|6666666.59338.peg.5025	None	None	fig|6666666.59337.peg.2646	fig|6666666.59342.peg.3374	None	None	fig|6666666.59345.peg.710	fig|6666666.59341.peg.406	fig|6666666.59347.peg.2254	None	None	None	fig|6666666.59350.peg.1333	None	None	None	None

10885	D-alanine-D-alanine ligase	fig|6666666.59329.peg.5279	None	fig|6666666.59331.peg.5590	fig|6666666.59332.peg.3090	None	fig|6666666.59335.peg.3201	None	None	None	fig|6666666.59339.peg.1752	None	None	None	None	None	None	None	None	fig|6666666.59346.peg.883	fig|6666666.59348.peg.1617	fig|6666666.59349.peg.2326	None	None	None	None	fig|6666666.59354.peg.4227

210	Salicylate hydroxylase EC 1.14.13.1	None	None	None	None	None	None	None	None	fig|6666666.59338.peg.2650	fig|6666666.59339.peg.3610	None	None	None	fig|6666666.71181.peg.4890	fig|6666666.59344.peg.5955	None	None	fig|6666666.59347.peg.2085	None	None	None	fig|6666666.59350.peg.634	fig|6666666.59351.peg.3556	fig|6666666.59353.peg.4159	fig|6666666.59355.peg.4907	None

5821	FIG01200456 hypothetical protein	fig|6666666.59329.peg.2482	fig|6666666.59330.peg.2026	fig|6666666.59331.peg.1339	fig|6666666.59332.peg.409	fig|6666666.59334.peg.1735	None	fig|6666666.59336.peg.6288	fig|6666666.59333.peg.4191	None	None	None	None	None	None	None	fig|6666666.59345.peg.3506	None	None	fig|6666666.59346.peg.4061	None	None	None	None	None	None	None

9818	Dihydroneopterin triphosphate epimerase	None	None	None	fig|6666666.59332.peg.952	None	fig|6666666.59335.peg.3119	None	None	fig|6666666.59338.peg.4965	None	None	None	None	fig|6666666.71181.peg.566	fig|6666666.59344.peg.1897	None	None	None	None	None	fig|6666666.59349.peg.4416	None	fig|6666666.59351.peg.4732	fig|6666666.59353.peg.1197	fig|6666666.59355.peg.279	None

8330	hypothetical protein	None	None	fig|6666666.59331.peg.1486	None	fig|6666666.59334.peg.4914	None	fig|6666666.59336.peg.2296	None	fig|6666666.59338.peg.5353	None	fig|6666666.59340.peg.1427	None	fig|6666666.59342.peg.2015	None	None	None	fig|6666666.59341.peg.1842	fig|6666666.59347.peg.2199	fig|6666666.59346.peg.4956	None	None	None	None	None	None	None

4048	Methylthioribose-1-phosphate isomerase EC 5.3.1.23	None	None	None	None	None	fig|6666666.59335.peg.5370	None	None	None	None	None	None	None	fig|6666666.71181.peg.909	fig|6666666.59344.peg.5182	None	None	None	None	None	fig|6666666.59349.peg.3407	fig|6666666.59350.peg.808	fig|6666666.59351.peg.5006	fig|6666666.59353.peg.909	fig|6666666.59355.peg.798	fig|6666666.59354.peg.4831

3597	FIG00921036 hypothetical protein	None	fig|6666666.59330.peg.5008	fig|6666666.59331.peg.2862	fig|6666666.59332.peg.5113	None	None	fig|6666666.59336.peg.5844	fig|6666666.59333.peg.4819	None	None	None	fig|6666666.59337.peg.4568	fig|6666666.59342.peg.4884	None	None	fig|6666666.59345.peg.3239	None	None	fig|6666666.59346.peg.1538	None	None	None	None	None	None	None

4437	FIG076926 outer membrane protein	fig|6666666.59329.peg.4258	None	fig|6666666.59331.peg.1004	fig|6666666.59332.peg.1145	None	fig|6666666.59335.peg.4056	fig|6666666.59336.peg.1076	None	None	fig|6666666.59339.peg.6262	None	None	fig|6666666.59342.peg.3432	None	None	None	None	None	fig|6666666.59346.peg.4624	fig|6666666.59348.peg.119	None	None	None	None	None	None

10474	FIG01199591 hypothetical protein	fig|6666666.59329.peg.3546	fig|6666666.59330.peg.4387	None	None	None	fig|6666666.59335.peg.3429	fig|6666666.59336.peg.5385	fig|6666666.59333.peg.3792	None	fig|6666666.59339.peg.2707	None	fig|6666666.59337.peg.5008	fig|6666666.59342.peg.3927	None	None	fig|6666666.59345.peg.3368	None	None	None	None	None	None	None	None	None	None

2290	Pseudaminic acid cytidylyltransferase EC 2.7.7.43	None	fig|6666666.59330.peg.4936	None	None	None	fig|6666666.59335.peg.4204	None	None	fig|6666666.59338.peg.1298	fig|6666666.59339.peg.6333	None	None	fig|6666666.59342.peg.1522	None	None	None	None	None	None	fig|6666666.59348.peg.3	fig|6666666.59349.peg.762	None	fig|6666666.59351.peg.2032	None	None	fig|6666666.59354.peg.877

3040	Signal transduction histidine kinase SypF	None	None	fig|6666666.59331.peg.389	fig|6666666.59332.peg.2823	fig|6666666.59334.peg.5538	None	fig|6666666.59336.peg.4457	fig|6666666.59333.peg.4988	None	None	None	fig|6666666.59337.peg.4443	fig|6666666.59342.peg.1299	None	None	fig|6666666.59345.peg.3143	None	None	fig|6666666.59346.peg.1744	None	None	None	None	None	None	None

1097	hypothetical protein	fig|6666666.59329.peg.6301	None	fig|6666666.59331.peg.1620	fig|6666666.59332.peg.5298	None	fig|6666666.59335.peg.2137	fig|6666666.59336.peg.2350	None	None	fig|6666666.59339.peg.5688	None	None	fig|6666666.59342.peg.6290	None	None	fig|6666666.59345.peg.1364	None	None	fig|6666666.59346.peg.4049	None	None	None	None	None	None	None

6452	2-haloalkanoic acid dehalogenase EC 3.8.1.2	fig|6666666.59329.peg.2525	fig|6666666.59330.peg.3506	fig|6666666.59331.peg.6019	None	None	fig|6666666.59335.peg.967	fig|6666666.59336.peg.1204	fig|6666666.59333.peg.1866	None	None	None	fig|6666666.59337.peg.2779	None	None	None	fig|6666666.59345.peg.2754	None	None	None	None	None	None	fig|6666666.59351.peg.142	None	None	None

7732	FIG01199528 hypothetical protein	fig|6666666.59329.peg.492	fig|6666666.59330.peg.376	fig|6666666.59331.peg.5302	None	None	None	fig|6666666.59336.peg.5363	fig|6666666.59333.peg.2745	None	None	None	fig|6666666.59337.peg.557	fig|6666666.59342.peg.4752	None	None	fig|6666666.59345.peg.5047	None	None	fig|6666666.59346.peg.325	None	None	None	None	None	None	None

10107	Phosphogluconate dehydratase EC 4.2.1.12	None	None	fig|6666666.59331.peg.1910	None	None	None	fig|6666666.59336.peg.6013	fig|6666666.59333.peg.696	fig|6666666.59338.peg.4803	None	fig|6666666.59340.peg.3299	None	None	fig|6666666.71181.peg.5606	fig|6666666.59344.peg.2696	None	None	None	None	fig|6666666.59348.peg.74	None	None	None	None	None	fig|6666666.59354.peg.294

6102	Pathogenesis-related protein	None	None	None	None	None	None	fig|6666666.59336.peg.1501	None	fig|6666666.59338.peg.1705	fig|6666666.59339.peg.1480	None	fig|6666666.59337.peg.3093	None	fig|6666666.71181.peg.3709	None	None	fig|6666666.59341.peg.431	fig|6666666.59347.peg.2548	None	None	None	fig|6666666.59350.peg.4321	fig|6666666.59351.peg.5468	None	None	None

10930	L-Proline Glycine betaine transporter ProP	None	fig|6666666.59330.peg.3256	None	None	None	fig|6666666.59335.peg.78	None	None	None	fig|6666666.59339.peg.7635	None	fig|6666666.59337.peg.1246	None	fig|6666666.71181.peg.1407	fig|6666666.59344.peg.1331	None	None	None	None	fig|6666666.59348.peg.5127	None	None	None	fig|6666666.59353.peg.4763	fig|6666666.59355.peg.1196	None

2011	hypothetical protein	fig|6666666.59329.peg.1305	None	fig|6666666.59331.peg.311	fig|6666666.59332.peg.4242	None	fig|6666666.59335.peg.1212	None	None	None	fig|6666666.59339.peg.1491	None	None	None	None	None	fig|6666666.59345.peg.1750	fig|6666666.59341.peg.633	None	fig|6666666.59346.peg.4990	fig|6666666.59348.peg.1628	None	None	None	None	None	None

5431	Phosphonates transport ATP-binding protein PhnK	None	None	None	None	None	None	None	None	fig|6666666.59338.peg.836	fig|6666666.59339.peg.7021	None	None	None	fig|6666666.71181.peg.3524	fig|6666666.59344.peg.4615	None	fig|6666666.59341.peg.4382	fig|6666666.59347.peg.104	None	None	None	fig|6666666.59350.peg.2341	None	fig|6666666.59353.peg.2404	fig|6666666.59355.peg.495	None

10667	SECRETION ACTIVATOR PROTEIN	fig|6666666.59329.peg.5602	None	None	None	None	None	None	None	None	fig|6666666.59339.peg.1080	None	None	None	fig|6666666.71181.peg.2865	fig|6666666.59344.peg.4398	None	None	None	None	None	fig|6666666.59349.peg.1258	fig|6666666.59350.peg.1733	None	fig|6666666.59353.peg.4310	fig|6666666.59355.peg.4392	fig|6666666.59354.peg.5286

4634	FIG01205119 hypothetical protein	fig|6666666.59329.peg.3276	fig|6666666.59330.peg.4297	None	None	None	fig|6666666.59335.peg.2514	fig|6666666.59336.peg.2521	None	None	fig|6666666.59339.peg.405	None	fig|6666666.59337.peg.4099	fig|6666666.59342.peg.6318	None	None	fig|6666666.59345.peg.1697	fig|6666666.59341.peg.1795	None	None	None	None	None	None	None	None	None

4254	FIG01200723 hypothetical protein	fig|6666666.59329.peg.5728	fig|6666666.59330.peg.1103	None	None	None	fig|6666666.59335.peg.777	None	None	fig|6666666.59338.peg.1269	fig|6666666.59339.peg.8035	None	fig|6666666.59337.peg.876	None	None	None	None	None	fig|6666666.59347.peg.4797	fig|6666666.59346.peg.1194	fig|6666666.59348.peg.314	None	None	None	None	None	None

377	FIG01203597 hypothetical protein	fig|6666666.59329.peg.3668	fig|6666666.59330.peg.272	None	fig|6666666.59332.peg.4765	None	None	fig|6666666.59336.peg.5487	None	fig|6666666.59338.peg.977	None	None	fig|6666666.59337.peg.1000	fig|6666666.59342.peg.4536	None	None	fig|6666666.59345.peg.4282	None	fig|6666666.59347.peg.3933	None	None	None	None	None	None	None	None

7983	FIG01200582 hypothetical protein	None	fig|6666666.59330.peg.3475	None	None	None	None	fig|6666666.59336.peg.2176	fig|6666666.59333.peg.1393	None	None	None	fig|6666666.59337.peg.417	fig|6666666.59342.peg.252	None	None	fig|6666666.59345.peg.2561	None	None	None	None	fig|6666666.59349.peg.293	None	fig|6666666.59351.peg.263	None	None	fig|6666666.59354.peg.1018

8153	Electron transfer flavoprotein-ubiquinone oxidoreductase EC 1.5.5.1	fig|6666666.59329.peg.2139	None	None	None	None	fig|6666666.59335.peg.4299	fig|6666666.59336.peg.2881	None	None	fig|6666666.59339.peg.4644	None	fig|6666666.59337.peg.4798	None	None	None	None	fig|6666666.59341.peg.1710	None	None	fig|6666666.59348.peg.3518	fig|6666666.59349.peg.4286	fig|6666666.59350.peg.6746	None	None	None	None

9988	Cs C2378	fig|6666666.59329.peg.5375	None	None	fig|6666666.59332.peg.1101	fig|6666666.59334.peg.372	None	None	None	fig|6666666.59338.peg.480	None	None	fig|6666666.59337.peg.4366	fig|6666666.59342.peg.1471	None	None	None	None	fig|6666666.59347.peg.1195	None	fig|6666666.59348.peg.2088	None	fig|6666666.59350.peg.1287	None	None	None	None

1382	putative two-component system sensor histidine kinase, putative heat shock protein	fig|6666666.59329.peg.265	None	None	None	None	None	fig|6666666.59336.peg.2360	None	None	fig|6666666.59339.peg.6270	None	None	None	fig|6666666.71181.peg.2990	fig|6666666.59344.peg.3989	fig|6666666.59345.peg.2123	None	None	None	None	None	None	fig|6666666.59351.peg.4505	fig|6666666.59353.peg.2454	fig|6666666.59355.peg.547	None

4514	FIG01199723 hypothetical protein	fig|6666666.59329.peg.2098	fig|6666666.59330.peg.256	fig|6666666.59331.peg.2435	None	None	None	fig|6666666.59336.peg.897	fig|6666666.59333.peg.5360	None	None	None	fig|6666666.59337.peg.965	fig|6666666.59342.peg.5186	None	None	fig|6666666.59345.peg.2709	None	fig|6666666.59347.peg.4640	None	None	None	None	None	None	None	None

5283	FIG01201311 hypothetical protein	fig|6666666.59329.peg.4221	fig|6666666.59330.peg.5019	None	None	None	fig|6666666.59335.peg.1713	None	None	fig|6666666.59338.peg.4031	fig|6666666.59339.peg.7234	None	fig|6666666.59337.peg.5603	None	None	None	None	fig|6666666.59341.peg.3053	fig|6666666.59347.peg.5270	None	fig|6666666.59348.peg.5017	None	None	None	None	None	None

9020	FIG01202596 hypothetical protein	fig|6666666.59329.peg.4931	None	fig|6666666.59331.peg.266	None	None	fig|6666666.59335.peg.5002	fig|6666666.59336.peg.723	None	None	fig|6666666.59339.peg.4069	None	fig|6666666.59337.peg.4319	None	None	None	fig|6666666.59345.peg.3910	fig|6666666.59341.peg.3976	None	fig|6666666.59346.peg.404	None	None	None	None	None	None	None

7826	C4-type zinc finger protein, DksA TraR family	None	None	None	None	None	None	fig|6666666.59336.peg.5158	fig|6666666.59333.peg.965	fig|6666666.59338.peg.566	None	None	None	fig|6666666.59342.peg.5936	fig|6666666.71181.peg.864	fig|6666666.59344.peg.9	fig|6666666.59345.peg.219	None	None	None	None	None	None	None	fig|6666666.59353.peg.350	fig|6666666.59355.peg.1388	None

1224	membrane protein	fig|6666666.59329.peg.3953	fig|6666666.59330.peg.5151	None	None	None	fig|6666666.59335.peg.4014	fig|6666666.59336.peg.516	fig|6666666.59333.peg.2767	None	fig|6666666.59339.peg.3804	None	fig|6666666.59337.peg.2589	fig|6666666.59342.peg.1537	None	None	fig|6666666.59345.peg.764	None	None	None	None	None	None	None	None	None	None

2232	putative transposition protein	None	fig|6666666.59330.peg.1154	None	None	fig|6666666.59334.peg.4249	fig|6666666.59335.peg.1267	None	None	None	None	None	fig|6666666.59337.peg.1069	fig|6666666.59342.peg.402	fig|6666666.71181.peg.3706	None	fig|6666666.59345.peg.1649	fig|6666666.59341.peg.424	None	None	None	None	None	fig|6666666.59351.peg.2458	None	None	None

6642	Leucine-, isoleucine-, valine-, threonine-, and alanine-binding protein	None	None	None	None	None	None	None	None	None	fig|6666666.59339.peg.1355	None	None	None	fig|6666666.71181.peg.4616	fig|6666666.59344.peg.4869	None	None	None	None	None	fig|6666666.59349.peg.2268	fig|6666666.59350.peg.5164	fig|6666666.59351.peg.4831	fig|6666666.59353.peg.3776	fig|6666666.59355.peg.3263	fig|6666666.59354.peg.1849

2170	putative plasmid-related protein	None	None	fig|6666666.59331.peg.4173	None	None	None	fig|6666666.59336.peg.977	None	fig|6666666.59338.peg.544	fig|6666666.59339.peg.1629	fig|6666666.59340.peg.313	None	fig|6666666.59342.peg.2981	None	None	fig|6666666.59345.peg.2887	fig|6666666.59341.peg.2038	None	None	fig|6666666.59348.peg.1482	None	None	None	None	None	None

10373	Ca2+ H+ antiporter	None	None	fig|6666666.59331.peg.86	None	fig|6666666.59334.peg.757	None	None	None	fig|6666666.59338.peg.5368	None	fig|6666666.59340.peg.1408	None	None	fig|6666666.71181.peg.3757	fig|6666666.59344.peg.5581	None	fig|6666666.59341.peg.4341	None	None	None	None	None	None	fig|6666666.59353.peg.4667	fig|6666666.59355.peg.3856	None

4558	Uncharacterized protein ImpF	fig|6666666.59329.peg.2959	fig|6666666.59330.peg.3115	None	None	None	None	fig|6666666.59336.peg.4625	fig|6666666.59333.peg.1939	fig|6666666.59338.peg.5506	None	None	fig|6666666.59337.peg.5335	fig|6666666.59342.peg.3254	None	None	fig|6666666.59345.peg.3450	None	fig|6666666.59347.peg.2415	None	None	None	None	None	None	None	None

748	FIG01201101 hypothetical protein	fig|6666666.59329.peg.3634	fig|6666666.59330.peg.2336	fig|6666666.59331.peg.517	fig|6666666.59332.peg.2246	None	None	fig|6666666.59336.peg.2449	fig|6666666.59333.peg.3390	None	None	None	fig|6666666.59337.peg.3600	None	None	None	fig|6666666.59345.peg.1860	None	None	fig|6666666.59346.peg.3510	None	None	None	None	None	None	None

6665	hypothetical protein	fig|6666666.59329.peg.1924	None	None	None	fig|6666666.59334.peg.4233	None	fig|6666666.59336.peg.1761	None	None	fig|6666666.59339.peg.4140	None	fig|6666666.59337.peg.1832	fig|6666666.59342.peg.981	None	None	fig|6666666.59345.peg.4224	fig|6666666.59341.peg.4131	None	None	fig|6666666.59348.peg.1328	None	None	None	None	None	None

2117	FIG01203526 hypothetical protein	fig|6666666.59329.peg.3112	fig|6666666.59330.peg.702	None	None	fig|6666666.59334.peg.2392	None	fig|6666666.59336.peg.1549	fig|6666666.59333.peg.4325	None	None	None	fig|6666666.59337.peg.4149	fig|6666666.59342.peg.2879	None	None	fig|6666666.59345.peg.4793	None	fig|6666666.59347.peg.2484	None	None	None	None	None	None	None	None

11312	hypothetical protein	fig|6666666.59329.peg.2345	None	None	fig|6666666.59332.peg.3612	None	None	fig|6666666.59336.peg.3227	None	fig|6666666.59338.peg.3353	None	None	fig|6666666.59337.peg.5256	None	None	None	fig|6666666.59345.peg.4747	fig|6666666.59341.peg.463	fig|6666666.59347.peg.1890	fig|6666666.59346.peg.3764	None	None	None	None	None	None	None

8766	FIG01200999 hypothetical protein	None	fig|6666666.59330.peg.4997	None	None	None	fig|6666666.59335.peg.6208	fig|6666666.59336.peg.1883	fig|6666666.59333.peg.456	None	fig|6666666.59339.peg.632	None	fig|6666666.59337.peg.2009	fig|6666666.59342.peg.639	None	None	fig|6666666.59345.peg.3066	None	None	None	fig|6666666.59348.peg.4279	None	None	None	None	None	None

1917	Cytochrome c-type heme lyase subunit nrfG, nitrite reductase complex assembly	None	None	fig|6666666.59331.peg.3192	fig|6666666.59332.peg.4712	None	fig|6666666.59335.peg.359	fig|6666666.59336.peg.6406	None	None	None	None	fig|6666666.59337.peg.1757	None	None	None	fig|6666666.59345.peg.887	fig|6666666.59341.peg.440	fig|6666666.59347.peg.1870	None	fig|6666666.59348.peg.2630	None	None	None	None	None	None

3549	Biotin carboxylase of acetyl-CoA carboxylase EC 6.3.4.14	fig|6666666.59329.peg.3751	None	fig|6666666.59331.peg.1710	None	None	None	fig|6666666.59336.peg.2273	None	None	fig|6666666.59339.peg.6441	None	fig|6666666.59337.peg.4289	None	None	None	fig|6666666.59345.peg.5145	None	fig|6666666.59347.peg.4495	None	None	None	None	fig|6666666.59351.peg.3696	None	fig|6666666.59355.peg.4950	None

2786	L-fuconolactone hydrolase	None	None	None	None	fig|6666666.59334.peg.3739	None	None	None	None	None	None	None	None	fig|6666666.71181.peg.4974	fig|6666666.59344.peg.1401	None	None	None	None	None	fig|6666666.59349.peg.1780	fig|6666666.59350.peg.6956	fig|6666666.59351.peg.2307	fig|6666666.59353.peg.19	fig|6666666.59355.peg.625	fig|6666666.59354.peg.5388

6622	Permease of the major facilitator superfamily	None	None	fig|6666666.59331.peg.4353	fig|6666666.59332.peg.4272	None	fig|6666666.59335.peg.3135	None	None	fig|6666666.59338.peg.235	fig|6666666.59339.peg.5437	fig|6666666.59340.peg.1664	None	None	None	None	None	None	fig|6666666.59347.peg.7	fig|6666666.59346.peg.146	fig|6666666.59348.peg.2050	None	None	None	None	None	None

10979	Acetate permease ActP cation acetate symporter	None	None	None	None	fig|6666666.59334.peg.34	None	fig|6666666.59336.peg.1302	fig|6666666.59333.peg.2212	None	None	fig|6666666.59340.peg.452	fig|6666666.59337.peg.4301	fig|6666666.59342.peg.2744	None	fig|6666666.59344.peg.5781	None	None	None	fig|6666666.59346.peg.778	None	None	None	None	None	None	fig|6666666.59354.peg.3592

8812	Flagellin protein FlaE	fig|6666666.59329.peg.903	fig|6666666.59330.peg.3028	fig|6666666.59331.peg.4837	fig|6666666.59332.peg.6024	None	None	fig|6666666.59336.peg.2335	fig|6666666.59333.peg.4290	None	None	None	fig|6666666.59337.peg.5712	None	None	None	fig|6666666.59345.peg.4396	None	None	fig|6666666.59346.peg.4795	None	None	None	None	None	None	None

3171	Phenazine biosynthesis protein PhzF	None	None	None	fig|6666666.59332.peg.1609	None	None	None	None	None	fig|6666666.59339.peg.6690	None	None	None	fig|6666666.71181.peg.2284	fig|6666666.59344.peg.4689	None	None	None	fig|6666666.59346.peg.2835	None	None	fig|6666666.59350.peg.3326	fig|6666666.59351.peg.4974	fig|6666666.59353.peg.4497	fig|6666666.59355.peg.4605	None

3772	LSU ribosomal protein L4p L1e	None	None	None	fig|6666666.59332.peg.3795	fig|6666666.59334.peg.1481	None	fig|6666666.59336.peg.141	fig|6666666.59333.peg.2689	fig|6666666.59338.peg.1393	fig|6666666.59339.peg.8264	None	fig|6666666.59337.peg.392	None	None	None	None	fig|6666666.59341.peg.2494	None	None	None	None	None	None	None	None	fig|6666666.59354.peg.1751

7880	PhnG protein	None	None	None	None	None	None	None	None	fig|6666666.59338.peg.831	fig|6666666.59339.peg.7013	None	None	None	fig|6666666.71181.peg.3520	fig|6666666.59344.peg.4620	None	fig|6666666.59341.peg.4386	fig|6666666.59347.peg.108	None	None	None	fig|6666666.59350.peg.2337	None	fig|6666666.59353.peg.2408	fig|6666666.59355.peg.499	None

6802	hypothetical protein	fig|6666666.59329.peg.5270	None	fig|6666666.59331.peg.6377	fig|6666666.59332.peg.1457	None	fig|6666666.59335.peg.1389	None	None	fig|6666666.59338.peg.891	fig|6666666.59339.peg.6744	None	None	None	None	None	None	fig|6666666.59341.peg.2409	fig|6666666.59347.peg.5363	fig|6666666.59346.peg.4858	None	None	None	None	None	None	None

5990	FIG01204954 hypothetical protein	fig|6666666.59329.peg.1482	None	None	None	None	None	fig|6666666.59336.peg.4753	None	fig|6666666.59338.peg.4795	None	None	fig|6666666.59337.peg.4064	fig|6666666.59342.peg.148	None	None	fig|6666666.59345.peg.3391	None	fig|6666666.59347.peg.898	None	None	fig|6666666.59349.peg.3652	None	fig|6666666.59351.peg.1919	None	None	None

244	RNA signal recognition particle 4.5S RNA	None	None	None	None	fig|6666666.59334.peg.4088	None	None	fig|6666666.59333.peg.3148	fig|6666666.59338.peg.2278	None	None	None	None	fig|6666666.71181.peg.3392	fig|6666666.59344.peg.4826	None	fig|6666666.59341.peg.2911	fig|6666666.59347.peg.877	None	None	None	None	None	fig|6666666.59353.peg.3498	fig|6666666.59355.peg.3225	None

7491	Sodium alanine symporter	None	fig|6666666.59330.peg.1730	None	None	fig|6666666.59334.peg.4723	fig|6666666.59335.peg.3632	fig|6666666.59336.peg.5037	None	None	None	fig|6666666.59340.peg.114	None	fig|6666666.59342.peg.1164	None	fig|6666666.59344.peg.1334	fig|6666666.59345.peg.3743	None	None	None	fig|6666666.59348.peg.4909	None	None	None	None	None	None

3697	Unknown	fig|6666666.59329.peg.1131	fig|6666666.59330.peg.2813	None	None	fig|6666666.59334.peg.686	None	fig|6666666.59336.peg.3245	None	fig|6666666.59338.peg.318	None	fig|6666666.59340.peg.1732	fig|6666666.59337.peg.1358	fig|6666666.59342.peg.4092	None	None	None	None	fig|6666666.59347.peg.2726	None	None	None	None	None	None	None	None

2696	Citrate lyase beta chain EC 4.1.3.6	fig|6666666.59329.peg.1725	None	None	None	fig|6666666.59334.peg.1766	None	fig|6666666.59336.peg.2509	None	None	None	None	None	fig|6666666.59342.peg.2233	fig|6666666.71181.peg.1726	fig|6666666.59344.peg.6208	None	None	None	None	None	None	fig|6666666.59350.peg.2856	None	fig|6666666.59353.peg.3688	fig|6666666.59355.peg.3011	None

7207	hypothetical protein	None	None	fig|6666666.59331.peg.1814	fig|6666666.59332.peg.4800	None	None	None	None	fig|6666666.59338.peg.1982	None	None	None	None	fig|6666666.71181.peg.1152	fig|6666666.59344.peg.2274	None	None	fig|6666666.59347.peg.3459	fig|6666666.59346.peg.2940	None	None	None	None	fig|6666666.59353.peg.3429	fig|6666666.59355.peg.3530	None

6766	corresponds to STY3950 from Accession AL513382 Salmonella typhi CT18	fig|6666666.59329.peg.5610	None	None	None	fig|6666666.59334.peg.584	None	fig|6666666.59336.peg.2611	None	fig|6666666.59338.peg.2581	None	None	fig|6666666.59337.peg.4361	fig|6666666.59342.peg.4433	None	None	fig|6666666.59345.peg.1901	fig|6666666.59341.peg.2430	fig|6666666.59347.peg.2465	None	None	None	None	None	None	None	None

142	FIG01199650 hypothetical protein	fig|6666666.59329.peg.1817	fig|6666666.59330.peg.1132	None	fig|6666666.59332.peg.3285	None	None	None	None	None	None	None	fig|6666666.59337.peg.3625	fig|6666666.59342.peg.1655	None	None	fig|6666666.59345.peg.5215	fig|6666666.59341.peg.1543	fig|6666666.59347.peg.691	None	fig|6666666.59348.peg.2464	None	None	None	None	None	None

10526	Propionate catabolism operon transcriptional regulator of GntR family [predicted]	None	None	fig|6666666.59331.peg.4746	fig|6666666.59332.peg.3975	fig|6666666.59334.peg.3890	None	fig|6666666.59336.peg.3701	fig|6666666.59333.peg.3526	None	fig|6666666.59339.peg.8770	fig|6666666.59340.peg.852	fig|6666666.59337.peg.5310	None	None	None	None	None	None	None	None	None	None	fig|6666666.59351.peg.1226	None	None	None

8870	Chaperone protein HtpG	None	fig|6666666.59330.peg.2202	None	None	None	fig|6666666.59335.peg.2420	fig|6666666.59336.peg.2549	None	None	fig|6666666.59339.peg.5265	fig|6666666.59340.peg.672	None	fig|6666666.59342.peg.1795	None	fig|6666666.59344.peg.5517	fig|6666666.59345.peg.4350	None	None	None	fig|6666666.59348.peg.2491	None	None	None	None	None	None

10205	3-oxoacyl-[ACP] synthase	None	None	fig|6666666.59331.peg.3681	None	None	None	fig|6666666.59336.peg.1056	fig|6666666.59333.peg.2905	None	None	None	None	None	fig|6666666.71181.peg.3940	None	None	fig|6666666.59341.peg.2753	None	fig|6666666.59346.peg.4723	fig|6666666.59348.peg.782	None	None	None	fig|6666666.59353.peg.4797	fig|6666666.59355.peg.4851	None

4182	Beta-lactamase EC 3.5.2.6	fig|6666666.59329.peg.2932	fig|6666666.59330.peg.3977	None	None	None	fig|6666666.59335.peg.875	fig|6666666.59336.peg.2469	fig|6666666.59333.peg.3938	None	fig|6666666.59339.peg.3263	None	fig|6666666.59337.peg.5770	None	None	None	fig|6666666.59345.peg.3996	fig|6666666.59341.peg.4578	None	None	None	None	None	None	None	None	None

836	FIG01200865 hypothetical protein	fig|6666666.59329.peg.3269	None	fig|6666666.59331.peg.1173	fig|6666666.59332.peg.1381	None	fig|6666666.59335.peg.5258	None	fig|6666666.59333.peg.1652	None	fig|6666666.59339.peg.2424	None	fig|6666666.59337.peg.3104	None	None	None	None	None	None	fig|6666666.59346.peg.3010	fig|6666666.59348.peg.4657	None	None	None	None	None	None

8886	Phage replication protein	None	None	fig|6666666.59331.peg.4767	None	None	fig|6666666.59335.peg.314	None	None	None	None	fig|6666666.59340.peg.319	None	None	fig|6666666.71181.peg.4828	None	None	fig|6666666.59341.peg.1864	fig|6666666.59347.peg.4891	None	fig|6666666.59348.peg.239	fig|6666666.59349.peg.74	None	None	None	None	fig|6666666.59354.peg.3288

5390	Sensor histidine kinase	None	fig|6666666.59330.peg.3424	fig|6666666.59331.peg.2501	fig|6666666.59332.peg.4700	None	None	fig|6666666.59336.peg.2507	fig|6666666.59333.peg.2284	fig|6666666.59338.peg.1472	None	None	fig|6666666.59337.peg.4370	fig|6666666.59342.peg.5425	None	None	None	None	None	fig|6666666.59346.peg.1434	None	None	None	None	None	None	None

2898	Two-component response regulator CreC	None	None	None	None	None	fig|6666666.59335.peg.4715	fig|6666666.59336.peg.404	fig|6666666.59333.peg.3374	None	None	None	fig|6666666.59337.peg.5215	None	fig|6666666.71181.peg.3733	None	fig|6666666.59345.peg.1834	None	None	None	None	None	None	fig|6666666.59351.peg.2619	fig|6666666.59353.peg.3835	fig|6666666.59355.peg.1552	None

1894	FIG01206951 hypothetical protein	None	None	fig|6666666.59331.peg.1823	fig|6666666.59332.peg.2199	fig|6666666.59334.peg.4305	None	fig|6666666.59336.peg.411	None	None	None	None	None	fig|6666666.59342.peg.5587	None	None	fig|6666666.59345.peg.1018	fig|6666666.59341.peg.5991	fig|6666666.59347.peg.2215	fig|6666666.59346.peg.4206	None	None	None	None	None	None	None

7749	Flp pilus assembly protein RcpC CpaB	fig|6666666.59329.peg.4557	None	fig|6666666.59331.peg.240	None	fig|6666666.59334.peg.3488	fig|6666666.59335.peg.1093	None	None	None	fig|6666666.59339.peg.6815	None	fig|6666666.59337.peg.1327	None	None	None	None	None	None	fig|6666666.59346.peg.4788	fig|6666666.59348.peg.989	None	fig|6666666.59350.peg.5692	None	None	None	None

11448	Acetyltransferase EC 2.3.1.-	fig|6666666.59329.peg.6272	None	fig|6666666.59331.peg.2710	None	None	None	fig|6666666.59336.peg.4927	None	None	fig|6666666.59339.peg.3831	None	fig|6666666.59337.peg.3229	None	None	None	fig|6666666.59345.peg.1815	fig|6666666.59341.peg.923	None	fig|6666666.59346.peg.5308	fig|6666666.59348.peg.3795	None	None	None	None	None	None

908	hypothetical protein	fig|6666666.59329.peg.1554	None	fig|6666666.59331.peg.2449	fig|6666666.59332.peg.4832	fig|6666666.59334.peg.3314	None	fig|6666666.59336.peg.420	None	fig|6666666.59338.peg.4750	None	None	None	None	None	None	fig|6666666.59345.peg.1027	None	fig|6666666.59347.peg.1109	None	fig|6666666.59348.peg.2735	None	None	None	None	None	None

4229	hypothetical protein	fig|6666666.59329.peg.175	None	fig|6666666.59331.peg.481	fig|6666666.59332.peg.6152	None	None	fig|6666666.59336.peg.996	fig|6666666.59333.peg.2882	None	None	None	fig|6666666.59337.peg.5292	fig|6666666.59342.peg.6429	None	None	fig|6666666.59345.peg.1446	None	None	fig|6666666.59346.peg.204	None	None	None	None	None	None	None

6705	Methyl-accepting chemotaxis protein	fig|6666666.59329.peg.4084	None	None	None	None	None	None	None	None	fig|6666666.59339.peg.1461	fig|6666666.59340.peg.594	None	None	None	None	None	fig|6666666.59341.peg.5953	fig|6666666.59347.peg.4902	fig|6666666.59346.peg.4133	fig|6666666.59348.peg.2339	fig|6666666.59349.peg.84	None	None	None	None	fig|6666666.59354.peg.440

4054	Cyclic di-GMP binding protein precursor	None	fig|6666666.59330.peg.2189	None	None	None	None	fig|6666666.59336.peg.6542	None	None	None	fig|6666666.59340.peg.118	None	fig|6666666.59342.peg.86	fig|6666666.71181.peg.4268	fig|6666666.59344.peg.2441	fig|6666666.59345.peg.4371	None	None	None	None	None	None	None	fig|6666666.59353.peg.3665	fig|6666666.59355.peg.4036	None

1536	FIG01205684 hypothetical protein	None	None	None	None	None	None	fig|6666666.59336.peg.1029	None	None	None	None	fig|6666666.59337.peg.2283	fig|6666666.59342.peg.826	fig|6666666.71181.peg.751	fig|6666666.59344.peg.4797	fig|6666666.59345.peg.2015	fig|6666666.59341.peg.215	None	None	None	None	None	None	fig|6666666.59353.peg.4977	fig|6666666.59355.peg.5059	None

7115	Mannose-6-phosphate isomerase EC 5.3.1.8	fig|6666666.59329.peg.6456	None	None	None	fig|6666666.59334.peg.924	None	fig|6666666.59336.peg.2084	fig|6666666.59333.peg.5056	None	fig|6666666.59339.peg.419	fig|6666666.59340.peg.1490	None	fig|6666666.59342.peg.1989	None	None	None	fig|6666666.59341.peg.3995	None	None	fig|6666666.59348.peg.1170	None	None	None	None	None	None

10320	hypothetical protein	fig|6666666.59329.peg.3004	None	None	fig|6666666.59332.peg.5879	None	None	fig|6666666.59336.peg.3200	fig|6666666.59333.peg.2605	fig|6666666.59338.peg.1872	None	None	None	fig|6666666.59342.peg.3235	None	None	fig|6666666.59345.peg.1431	None	fig|6666666.59347.peg.147	None	fig|6666666.59348.peg.4092	None	None	None	None	None	None

4895	pentapeptide repeat family protein	fig|6666666.59329.peg.3548	fig|6666666.59330.peg.4390	None	None	None	fig|6666666.59335.peg.3432	fig|6666666.59336.peg.4884	fig|6666666.59333.peg.4569	None	fig|6666666.59339.peg.2711	None	fig|6666666.59337.peg.5010	fig|6666666.59342.peg.2840	None	None	fig|6666666.59345.peg.3370	None	None	None	None	None	None	None	None	None	None

4852	Prolidase EC 3.4.13.9	None	None	fig|6666666.59331.peg.4869	fig|6666666.59332.peg.1621	None	None	fig|6666666.59336.peg.2622	None	fig|6666666.59338.peg.620	None	None	None	fig|6666666.59342.peg.1420	None	None	None	None	None	fig|6666666.59346.peg.4845	None	fig|6666666.59349.peg.4349	None	fig|6666666.59351.peg.915	None	None	fig|6666666.59354.peg.6403

11187	FIG01289452 hypothetical protein	None	None	fig|6666666.59331.peg.6256	None	fig|6666666.59334.peg.4428	None	None	None	fig|6666666.59338.peg.1996	None	fig|6666666.59340.peg.1993	None	None	fig|6666666.71181.peg.2061	fig|6666666.59344.peg.1056	None	None	None	None	None	None	None	fig|6666666.59351.peg.4061	fig|6666666.59353.peg.743	fig|6666666.59355.peg.962	None

11188	FIG01199688 hypothetical protein	fig|6666666.59329.peg.5267	fig|6666666.59330.peg.2150	None	None	None	fig|6666666.59335.peg.2697	fig|6666666.59336.peg.3637	fig|6666666.59333.peg.3921	None	fig|6666666.59339.peg.2733	None	fig|6666666.59337.peg.4075	fig|6666666.59342.peg.6214	None	None	fig|6666666.59345.peg.3382	None	None	None	None	None	None	None	None	None	None

8127	hypothetical protein	None	None	fig|6666666.59331.peg.5840	fig|6666666.59332.peg.19	None	fig|6666666.59335.peg.5027	None	None	fig|6666666.59338.peg.135	fig|6666666.59339.peg.1763	None	None	None	None	None	None	fig|6666666.59341.peg.3578	fig|6666666.59347.peg.221	fig|6666666.59346.peg.1074	fig|6666666.59348.peg.2204	None	None	None	None	None	None

7703	POSSIBLE METHYLTRANSFERASE METHYLASE EC 2.1.1.-	fig|6666666.59329.peg.3003	None	None	fig|6666666.59332.peg.5880	fig|6666666.59334.peg.1544	None	fig|6666666.59336.peg.3202	fig|6666666.59333.peg.2606	fig|6666666.59338.peg.1871	None	None	None	fig|6666666.59342.peg.3234	None	None	fig|6666666.59345.peg.1433	None	fig|6666666.59347.peg.146	None	None	None	None	None	None	None	None

4074	Membrane protein, putative	fig|6666666.59329.peg.2048	None	fig|6666666.59331.peg.525	None	None	None	fig|6666666.59336.peg.4956	None	None	None	None	fig|6666666.59337.peg.1996	fig|6666666.59342.peg.5586	None	None	fig|6666666.59345.peg.30	fig|6666666.59341.peg.5199	fig|6666666.59347.peg.1678	fig|6666666.59346.peg.3760	None	None	None	None	None	None	None

3961	hypothetical protein	fig|6666666.59329.peg.4905	None	None	fig|6666666.59332.peg.5069	None	fig|6666666.59335.peg.2380	fig|6666666.59336.peg.687	None	None	fig|6666666.59339.peg.6806	None	None	fig|6666666.59342.peg.4612	None	None	None	fig|6666666.59341.peg.3906	None	fig|6666666.59346.peg.4926	fig|6666666.59348.peg.3881	None	None	None	None	None	None

3630	type 1 fimbriae anchoring protein FimD	None	None	None	None	None	None	fig|6666666.59336.peg.1028	None	None	None	None	fig|6666666.59337.peg.2282	fig|6666666.59342.peg.825	fig|6666666.71181.peg.4378	fig|6666666.59344.peg.4799	fig|6666666.59345.peg.2848	fig|6666666.59341.peg.1738	None	None	None	None	None	None	fig|6666666.59353.peg.4332	fig|6666666.59355.peg.1505	None

6307	NADP-dependent malic enzyme EC 1.1.1.40	fig|6666666.59329.peg.4605	None	None	None	None	None	None	None	None	fig|6666666.59339.peg.6378	None	None	None	None	None	fig|6666666.59345.peg.5406	fig|6666666.59341.peg.5323	None	None	fig|6666666.59348.peg.4559	fig|6666666.59349.peg.516	fig|6666666.59350.peg.6065	fig|6666666.59351.peg.3839	None	None	fig|6666666.59354.peg.928

9315	L-threonine 3-dehydrogenase EC 1.1.1.103	None	fig|6666666.59330.peg.3482	fig|6666666.59331.peg.1398	None	fig|6666666.59334.peg.2555	None	fig|6666666.59336.peg.4218	fig|6666666.59333.peg.2131	fig|6666666.59338.peg.6064	fig|6666666.59339.peg.4406	None	None	fig|6666666.59342.peg.6000	None	None	None	None	None	None	fig|6666666.59348.peg.4778	None	None	None	None	None	None

5522	FIG01201985 hypothetical protein	fig|6666666.59329.peg.187	fig|6666666.59330.peg.1871	None	None	fig|6666666.59334.peg.4656	fig|6666666.59335.peg.1082	fig|6666666.59336.peg.2873	None	None	fig|6666666.59339.peg.1858	None	fig|6666666.59337.peg.2963	fig|6666666.59342.peg.4349	None	None	fig|6666666.59345.peg.5340	None	None	None	None	None	None	None	None	None	None

9659	FIG01201555 hypothetical protein	None	None	None	fig|6666666.59332.peg.5547	fig|6666666.59334.peg.1727	fig|6666666.59335.peg.1763	None	None	fig|6666666.59338.peg.2	fig|6666666.59339.peg.5195	fig|6666666.59340.peg.2381	None	None	None	None	None	fig|6666666.59341.peg.5011	fig|6666666.59347.peg.2912	None	fig|6666666.59348.peg.4157	None	None	None	None	None	None

8380	GTPases - Sulfate adenylate transferase subunit 1	None	fig|6666666.59330.peg.2916	None	None	None	None	fig|6666666.59336.peg.980	fig|6666666.59333.peg.3028	None	None	None	fig|6666666.59337.peg.3204	fig|6666666.59342.peg.5629	None	None	fig|6666666.59345.peg.2884	None	None	None	None	fig|6666666.59349.peg.4483	None	fig|6666666.59351.peg.4588	None	None	fig|6666666.59354.peg.323

2879	FIG01199641 hypothetical protein	fig|6666666.59329.peg.641	fig|6666666.59330.peg.201	None	fig|6666666.59332.peg.46	None	None	fig|6666666.59336.peg.1487	fig|6666666.59333.peg.1176	None	None	None	fig|6666666.59337.peg.2788	fig|6666666.59342.peg.4780	None	None	fig|6666666.59345.peg.2746	None	None	fig|6666666.59346.peg.2395	None	None	None	None	None	None	None

3356	FIG01205237 hypothetical protein	None	fig|6666666.59330.peg.3214	None	None	None	None	None	None	fig|6666666.59338.peg.5796	fig|6666666.59339.peg.8123	None	fig|6666666.59337.peg.5653	fig|6666666.59342.peg.1771	None	None	None	None	fig|6666666.59347.peg.3728	None	None	fig|6666666.59349.peg.357	None	fig|6666666.59351.peg.5118	None	None	fig|6666666.59354.peg.382

5441	FIG01204047 hypothetical protein	None	fig|6666666.59330.peg.3873	None	None	None	None	fig|6666666.59336.peg.4600	None	fig|6666666.59338.peg.4685	None	None	fig|6666666.59337.peg.1947	fig|6666666.59342.peg.3666	None	None	fig|6666666.59345.peg.4052	fig|6666666.59341.peg.231	fig|6666666.59347.peg.552	fig|6666666.59346.peg.5017	None	None	None	None	None	None	None

5001	FIG00877092 hypothetical protein	None	None	fig|6666666.59331.peg.3167	None	None	None	None	fig|6666666.59333.peg.1949	fig|6666666.59338.peg.3590	None	None	fig|6666666.59337.peg.5229	fig|6666666.59342.peg.2331	None	None	None	None	fig|6666666.59347.peg.1035	None	fig|6666666.59348.peg.3120	fig|6666666.59349.peg.1649	None	None	None	None	fig|6666666.59354.peg.755

2200	FIG01199615 hypothetical protein	fig|6666666.59329.peg.4339	fig|6666666.59330.peg.2110	None	None	None	fig|6666666.59335.peg.986	fig|6666666.59336.peg.2221	fig|6666666.59333.peg.1448	None	None	None	fig|6666666.59337.peg.5490	fig|6666666.59342.peg.1461	None	None	fig|6666666.59345.peg.498	None	None	fig|6666666.59346.peg.5286	None	None	None	None	None	None	None

5298	ankyrin repeat protein, putative	None	None	fig|6666666.59331.peg.4551	fig|6666666.59332.peg.607	None	fig|6666666.59335.peg.3780	fig|6666666.59336.peg.2980	None	fig|6666666.59338.peg.1261	None	fig|6666666.59340.peg.475	None	fig|6666666.59342.peg.4016	None	None	fig|6666666.59345.peg.178	None	fig|6666666.59347.peg.5413	None	None	None	None	None	None	None	None

11036	FIG01202195 hypothetical protein	fig|6666666.59329.peg.5774	fig|6666666.59330.peg.2094	fig|6666666.59331.peg.2223	None	None	None	fig|6666666.59336.peg.2896	fig|6666666.59333.peg.1894	None	None	None	fig|6666666.59337.peg.3541	fig|6666666.59342.peg.954	None	None	fig|6666666.59345.peg.478	None	None	fig|6666666.59346.peg.689	None	None	None	None	None	None	None

2856	Malonyl CoA-acyl carrier protein transacylase EC 2.3.1.39	fig|6666666.59329.peg.4855	fig|6666666.59330.peg.3622	None	None	fig|6666666.59334.peg.3239	None	fig|6666666.59336.peg.3343	fig|6666666.59333.peg.1590	None	fig|6666666.59339.peg.3176	fig|6666666.59340.peg.408	fig|6666666.59337.peg.2689	fig|6666666.59342.peg.1384	None	None	None	None	None	None	None	None	None	None	None	None	None

529	Transcriptional regulator, MarR family	fig|6666666.59329.peg.4161	fig|6666666.59330.peg.4808	None	None	None	None	fig|6666666.59336.peg.2960	fig|6666666.59333.peg.2410	fig|6666666.59338.peg.2986	None	None	fig|6666666.59337.peg.2825	None	None	None	fig|6666666.59345.peg.1402	None	fig|6666666.59347.peg.302	None	fig|6666666.59348.peg.2654	None	None	None	None	None	None

7552	putative phage integrase	None	None	None	None	None	None	None	None	fig|6666666.59338.peg.732	None	None	None	fig|6666666.59342.peg.5003	fig|6666666.71181.peg.688	fig|6666666.59344.peg.659	fig|6666666.59345.peg.3852	None	fig|6666666.59347.peg.2542	None	fig|6666666.59348.peg.3760	None	None	None	fig|6666666.59353.peg.1009	fig|6666666.59355.peg.2363	None

9018	Phosphoenolpyruvate-dihydroxyacetone phosphotransferase operon regulatory protein DhaR	None	None	None	None	None	None	None	fig|6666666.59333.peg.104	fig|6666666.59338.peg.3829	None	None	None	fig|6666666.59342.peg.2674	fig|6666666.71181.peg.3031	fig|6666666.59344.peg.4445	None	fig|6666666.59341.peg.3411	fig|6666666.59347.peg.4230	None	None	None	None	None	fig|6666666.59353.peg.4165	fig|6666666.59355.peg.1712	None

8617	FMN reductase, NADPH-dependent	None	None	None	None	None	fig|6666666.59335.peg.2244	None	None	None	fig|6666666.59339.peg.856	None	None	None	fig|6666666.71181.peg.4810	None	None	None	None	None	fig|6666666.59348.peg.2168	fig|6666666.59349.peg.4772	None	fig|6666666.59351.peg.4145	fig|6666666.59353.peg.2862	fig|6666666.59355.peg.5021	fig|6666666.59354.peg.5591

2660	Alginate lyase precursor EC 4.2.2.3	None	None	fig|6666666.59331.peg.477	None	fig|6666666.59334.peg.3371	None	fig|6666666.59336.peg.2198	None	fig|6666666.59338.peg.4279	None	fig|6666666.59340.peg.1639	None	fig|6666666.59342.peg.4361	None	None	fig|6666666.59345.peg.1353	None	fig|6666666.59347.peg.299	fig|6666666.59346.peg.5151	None	None	None	None	None	None	None

3480	putative lipoprotein	fig|6666666.59329.peg.1423	None	None	None	None	None	fig|6666666.59336.peg.2840	None	None	None	None	fig|6666666.59337.peg.2241	fig|6666666.59342.peg.6299	fig|6666666.71181.peg.851	fig|6666666.59344.peg.26	fig|6666666.59345.peg.2976	None	None	None	None	None	None	None	fig|6666666.59353.peg.364	fig|6666666.59355.peg.1401	None

9030	Alginate lyase precursor EC 4.2.2.3	None	None	fig|6666666.59331.peg.1540	None	fig|6666666.59334.peg.1624	None	fig|6666666.59336.peg.434	None	fig|6666666.59338.peg.1958	None	fig|6666666.59340.peg.3150	None	fig|6666666.59342.peg.5877	None	None	fig|6666666.59345.peg.1372	None	fig|6666666.59347.peg.2430	fig|6666666.59346.peg.2143	None	None	None	None	None	None	None

732	Mobile element protein	fig|6666666.59329.peg.6193	fig|6666666.59330.peg.5117	fig|6666666.59331.peg.567	None	None	None	fig|6666666.59336.peg.4496	None	None	None	None	fig|6666666.59337.peg.4566	None	None	None	fig|6666666.59345.peg.3241	None	fig|6666666.59347.peg.1756	fig|6666666.59346.peg.1892	None	None	fig|6666666.59350.peg.4323	None	None	None	None

8090	Exodeoxyribonuclease V gamma chain EC 3.1.11.5	None	None	None	None	fig|6666666.59334.peg.3054	None	fig|6666666.59336.peg.115	None	None	fig|6666666.59339.peg.6944	fig|6666666.59340.peg.1661	None	None	None	None	None	fig|6666666.59341.peg.522	None	fig|6666666.59346.peg.1837	fig|6666666.59348.peg.297	fig|6666666.59349.peg.2650	None	None	None	None	fig|6666666.59354.peg.5841

4953	Transcriptional regulator, LysR family	fig|6666666.59329.peg.3041	None	fig|6666666.59331.peg.2177	fig|6666666.59332.peg.3821	None	None	fig|6666666.59336.peg.5068	None	None	None	None	fig|6666666.59337.peg.4091	fig|6666666.59342.peg.4676	None	None	fig|6666666.59345.peg.1691	fig|6666666.59341.peg.2480	None	fig|6666666.59346.peg.4448	None	None	None	None	None	None	None

9838	Membrane-associated phospholipid phosphatase	fig|6666666.59329.peg.5757	fig|6666666.59330.peg.2727	None	None	None	None	fig|6666666.59336.peg.5601	None	fig|6666666.59338.peg.3280	None	None	fig|6666666.59337.peg.4388	fig|6666666.59342.peg.3558	None	None	fig|6666666.59345.peg.3408	fig|6666666.59341.peg.5349	fig|6666666.59347.peg.2299	None	None	None	None	None	None	None	None

3845	FIG01200167 hypothetical protein	None	None	fig|6666666.59331.peg.4817	fig|6666666.59332.peg.4422	fig|6666666.59334.peg.1100	fig|6666666.59335.peg.2014	None	None	None	fig|6666666.59339.peg.3952	None	None	None	None	None	None	fig|6666666.59341.peg.729	fig|6666666.59347.peg.3439	fig|6666666.59346.peg.1449	fig|6666666.59348.peg.2879	None	None	None	None	None	None

6672	Superoxide dismutase [Cu-Zn] precursor EC 1.15.1.1	fig|6666666.59329.peg.4006	None	None	None	None	None	fig|6666666.59336.peg.3511	fig|6666666.59333.peg.4096	None	None	None	None	fig|6666666.59342.peg.3632	fig|6666666.71181.peg.2937	fig|6666666.59344.peg.3500	fig|6666666.59345.peg.1760	None	None	None	None	None	None	None	fig|6666666.59353.peg.1382	fig|6666666.59355.peg.2490	None

4939	HDED PROTEIN	fig|6666666.59329.peg.4732	fig|6666666.59330.peg.3901	None	None	None	None	None	None	None	None	None	None	fig|6666666.59342.peg.5110	fig|6666666.71181.peg.139	fig|6666666.59344.peg.3663	fig|6666666.59345.peg.3105	fig|6666666.59341.peg.4338	None	None	None	None	None	None	fig|6666666.59353.peg.3851	fig|6666666.59355.peg.1535	None

2109	FIG01200274 hypothetical protein	fig|6666666.59329.peg.4909	fig|6666666.59330.peg.869	None	None	None	fig|6666666.59335.peg.2461	fig|6666666.59336.peg.3501	fig|6666666.59333.peg.58	None	fig|6666666.59339.peg.3563	None	fig|6666666.59337.peg.3289	None	None	None	fig|6666666.59345.peg.552	fig|6666666.59341.peg.668	None	None	None	None	None	None	None	None	None

6028	membrane protein, putative	fig|6666666.59329.peg.5139	fig|6666666.59330.peg.3397	None	None	None	fig|6666666.59335.peg.5540	fig|6666666.59336.peg.2974	fig|6666666.59333.peg.4972	None	fig|6666666.59339.peg.5680	fig|6666666.59340.peg.1652	fig|6666666.59337.peg.3519	fig|6666666.59342.peg.3821	None	None	None	None	None	None	None	None	None	None	None	None	None

3144	Carboxylesterase	None	None	None	None	None	None	None	None	None	fig|6666666.59339.peg.6060	None	None	None	fig|6666666.71181.peg.4372	fig|6666666.59344.peg.5674	None	None	None	None	None	fig|6666666.59349.peg.1561	fig|6666666.59350.peg.8331	fig|6666666.59351.peg.4754	fig|6666666.59353.peg.4907	fig|6666666.59355.peg.4977	fig|6666666.59354.peg.1458

5778	Flp pilus assembly protein TadD, contains TPR repeat	fig|6666666.59329.peg.5878	None	fig|6666666.59331.peg.1247	None	fig|6666666.59334.peg.512	fig|6666666.59335.peg.3508	None	None	None	fig|6666666.59339.peg.6824	None	fig|6666666.59337.peg.1338	None	None	None	None	None	None	fig|6666666.59346.peg.4742	fig|6666666.59348.peg.2706	None	None	fig|6666666.59351.peg.4083	None	None	None

133	Transcriptional repressor of the lac operon	None	None	None	None	None	None	fig|6666666.59336.peg.1467	None	None	fig|6666666.59339.peg.5904	None	None	fig|6666666.59342.peg.2527	fig|6666666.71181.peg.1719	fig|6666666.59344.peg.5239	fig|6666666.59345.peg.846	None	None	fig|6666666.59346.peg.2640	None	None	None	None	fig|6666666.59353.peg.3695	fig|6666666.59355.peg.3017	None

8804	methyl-accepting chemotaxis protein	fig|6666666.59329.peg.757	fig|6666666.59330.peg.2768	None	None	None	None	fig|6666666.59336.peg.4547	None	None	None	None	fig|6666666.59337.peg.2928	None	None	None	fig|6666666.59345.peg.2085	fig|6666666.59341.peg.5029	fig|6666666.59347.peg.2562	None	None	fig|6666666.59349.peg.1534	None	fig|6666666.59351.peg.1722	None	None	None

1503	poly beta-D-mannuronate lyase EC 4.2.2.3	None	None	None	None	fig|6666666.59334.peg.5362	None	fig|6666666.59336.peg.2196	None	fig|6666666.59338.peg.3908	None	fig|6666666.59340.peg.1077	None	fig|6666666.59342.peg.1488	None	None	fig|6666666.59345.peg.1351	fig|6666666.59341.peg.3152	fig|6666666.59347.peg.298	fig|6666666.59346.peg.2857	None	None	None	None	None	None	None

1297	FIG01200268 hypothetical protein	fig|6666666.59329.peg.3547	fig|6666666.59330.peg.4389	None	None	None	fig|6666666.59335.peg.3430	fig|6666666.59336.peg.5386	fig|6666666.59333.peg.3790	None	fig|6666666.59339.peg.2708	None	fig|6666666.59337.peg.5009	fig|6666666.59342.peg.3928	None	None	fig|6666666.59345.peg.3369	None	None	None	None	None	None	None	None	None	None

991	FIG01200778 hypothetical protein	None	None	None	None	None	None	fig|6666666.59336.peg.2440	fig|6666666.59333.peg.5565	fig|6666666.59338.peg.4907	None	fig|6666666.59340.peg.1775	fig|6666666.59337.peg.3607	fig|6666666.59342.peg.2754	None	None	fig|6666666.59345.peg.1851	fig|6666666.59341.peg.561	fig|6666666.59347.peg.1854	None	None	None	None	None	None	None	None

11090	Cellulose synthase, putative	None	fig|6666666.59330.peg.2186	None	None	None	None	fig|6666666.59336.peg.3304	None	None	None	None	fig|6666666.59337.peg.4919	fig|6666666.59342.peg.89	fig|6666666.71181.peg.4270	fig|6666666.59344.peg.2438	fig|6666666.59345.peg.4375	None	None	None	None	None	None	None	fig|6666666.59353.peg.3667	fig|6666666.59355.peg.4038	None

10655	Acetyltransferase SypM	None	None	fig|6666666.59331.peg.1299	None	fig|6666666.59334.peg.2797	fig|6666666.59335.peg.2721	None	None	fig|6666666.59338.peg.1847	fig|6666666.59339.peg.1157	None	None	None	None	None	None	fig|6666666.59341.peg.2639	fig|6666666.59347.peg.2337	fig|6666666.59346.peg.5233	fig|6666666.59348.peg.2639	None	None	None	None	None	None

7479	Protein of unknown function UPF0060	None	None	None	None	fig|6666666.59334.peg.643	None	None	fig|6666666.59333.peg.3451	None	fig|6666666.59339.peg.5994	None	None	None	fig|6666666.71181.peg.3590	fig|6666666.59344.peg.5561	None	fig|6666666.59341.peg.1469	fig|6666666.59347.peg.1657	None	None	None	None	None	fig|6666666.59353.peg.4136	fig|6666666.59355.peg.5782	None

10966	Osmotically inducible protein OsmY	None	None	fig|6666666.59331.peg.53	None	None	None	None	fig|6666666.59333.peg.5373	None	None	None	None	None	fig|6666666.71181.peg.465	fig|6666666.59344.peg.1383	None	None	None	None	None	fig|6666666.59349.peg.1320	None	fig|6666666.59351.peg.4539	fig|6666666.59353.peg.31	fig|6666666.59355.peg.638	fig|6666666.59354.peg.149

8557	Phosphonates transport ATP-binding protein PhnL	None	None	None	None	None	None	None	None	fig|6666666.59338.peg.837	fig|6666666.59339.peg.7022	fig|6666666.59340.peg.3663	None	None	fig|6666666.71181.peg.3525	fig|6666666.59344.peg.4614	None	None	fig|6666666.59347.peg.103	None	None	None	fig|6666666.59350.peg.2342	None	fig|6666666.59353.peg.2403	fig|6666666.59355.peg.494	None

99	FIG01202392 hypothetical protein	fig|6666666.59329.peg.4876	fig|6666666.59330.peg.2736	None	None	None	None	fig|6666666.59336.peg.6455	fig|6666666.59333.peg.3113	fig|6666666.59338.peg.3541	None	None	fig|6666666.59337.peg.4404	None	None	None	fig|6666666.59345.peg.3426	None	fig|6666666.59347.peg.1806	None	None	None	None	None	None	None	None

391	Mobile element protein	fig|6666666.59329.peg.5935	fig|6666666.59330.peg.4800	None	fig|6666666.59332.peg.4360	None	None	None	None	fig|6666666.59338.peg.3876	None	None	fig|6666666.59337.peg.5904	None	None	None	None	None	fig|6666666.59347.peg.2027	fig|6666666.59346.peg.2829	None	None	fig|6666666.59350.peg.4731	None	None	None	None

4620	Sugar efflux transporter B	None	None	None	None	None	fig|6666666.59335.peg.3775	None	None	None	fig|6666666.59339.peg.4285	None	None	None	fig|6666666.71181.peg.3177	fig|6666666.59344.peg.1673	None	None	None	None	fig|6666666.59348.peg.4983	None	fig|6666666.59350.peg.1514	None	fig|6666666.59353.peg.1093	fig|6666666.59355.peg.386	None

172	Nicotinate-nucleotide adenylyltransferase EC 2.7.7.18	None	None	None	None	None	None	None	None	None	None	None	None	None	fig|6666666.71181.peg.4764	fig|6666666.59344.peg.4494	None	None	None	None	None	fig|6666666.59349.peg.2761	fig|6666666.59350.peg.3976	fig|6666666.59351.peg.3626	fig|6666666.59353.peg.2023	fig|6666666.59355.peg.4430	fig|6666666.59354.peg.2323

8714	FIG048548 ATP synthase protein I2	fig|6666666.59329.peg.6000	None	None	None	fig|6666666.59334.peg.2490	None	fig|6666666.59336.peg.5854	None	None	fig|6666666.59339.peg.2285	None	fig|6666666.59337.peg.2081	fig|6666666.59342.peg.4606	None	None	fig|6666666.59345.peg.3322	fig|6666666.59341.peg.3685	None	None	None	None	None	None	None	None	None

3684	hypothetical protein	None	None	None	None	None	None	None	None	None	fig|6666666.59339.peg.6409	None	None	None	fig|6666666.71181.peg.2017	fig|6666666.59344.peg.2722	None	None	None	None	None	fig|6666666.59349.peg.1759	None	fig|6666666.59351.peg.2288	fig|6666666.59353.peg.3013	fig|6666666.59355.peg.2921	fig|6666666.59354.peg.489

10182	Transcriptional regulator, Cro CI family	None	None	None	fig|6666666.59332.peg.4840	None	None	None	None	None	None	None	None	None	fig|6666666.71181.peg.848	fig|6666666.59344.peg.29	None	None	None	fig|6666666.59346.peg.4190	None	None	fig|6666666.59350.peg.4562	fig|6666666.59351.peg.2616	fig|6666666.59353.peg.367	fig|6666666.59355.peg.1404	None

10188	3-oxoadipate CoA-transferase subunit B EC 2.8.3.6	fig|6666666.59329.peg.1956	None	None	None	None	None	None	None	None	None	fig|6666666.59340.peg.925	None	None	fig|6666666.71181.peg.4664	fig|6666666.59344.peg.3815	None	None	None	None	None	None	fig|6666666.59350.peg.5758	None	fig|6666666.59353.peg.4994	fig|6666666.59355.peg.5095	fig|6666666.59354.peg.6306

1157	Chloramphenicol-sensitive protein rarD	None	None	None	None	None	None	None	None	fig|6666666.59338.peg.4386	None	None	None	None	fig|6666666.71181.peg.1735	fig|6666666.59344.peg.6138	None	fig|6666666.59341.peg.3473	fig|6666666.59347.peg.354	fig|6666666.59346.peg.4346	None	None	None	None	fig|6666666.59353.peg.3678	fig|6666666.59355.peg.4941	None

7142	hypothetical protein	None	None	fig|6666666.59331.peg.6210	fig|6666666.59332.peg.4237	fig|6666666.59334.peg.2513	fig|6666666.59335.peg.5000	None	None	None	fig|6666666.59339.peg.4066	None	None	None	None	None	None	fig|6666666.59341.peg.4313	None	fig|6666666.59346.peg.1454	fig|6666666.59348.peg.4045	None	None	None	None	None	None

3471	Na + Citrate OH - antiporter	fig|6666666.59329.peg.6018	None	None	None	fig|6666666.59334.peg.3105	None	fig|6666666.59336.peg.2512	None	None	None	None	None	fig|6666666.59342.peg.2229	fig|6666666.71181.peg.3825	fig|6666666.59344.peg.858	None	None	None	None	None	None	None	None	fig|6666666.59353.peg.1258	fig|6666666.59355.peg.4063	None

10109	hypothetical protein	fig|6666666.59329.peg.3602	None	None	None	fig|6666666.59334.peg.5277	fig|6666666.59335.peg.3297	fig|6666666.59336.peg.5012	None	None	fig|6666666.59339.peg.2402	None	fig|6666666.59337.peg.1415	fig|6666666.59342.peg.3848	None	None	fig|6666666.59345.peg.3872	None	None	None	None	None	None	None	None	None	None

7907	Putative phospholipase A accessory protein	fig|6666666.59329.peg.4005	None	None	None	None	None	fig|6666666.59336.peg.3510	None	None	None	None	None	fig|6666666.59342.peg.3631	fig|6666666.71181.peg.1434	fig|6666666.59344.peg.1552	fig|6666666.59345.peg.1761	None	None	None	None	None	None	None	fig|6666666.59353.peg.2640	fig|6666666.59355.peg.1841	None

6274	FIG01200762 hypothetical protein	fig|6666666.59329.peg.4543	None	fig|6666666.59331.peg.236	None	fig|6666666.59334.peg.1695	fig|6666666.59335.peg.3496	None	None	None	fig|6666666.59339.peg.6812	None	fig|6666666.59337.peg.1323	None	None	None	None	None	None	fig|6666666.59346.peg.4912	fig|6666666.59348.peg.3986	None	None	None	None	None	None

1683	FIG01200382 hypothetical protein	None	fig|6666666.59330.peg.129	None	fig|6666666.59332.peg.1008	None	None	fig|6666666.59336.peg.243	fig|6666666.59333.peg.4693	None	None	None	None	None	None	None	fig|6666666.59345.peg.2376	fig|6666666.59341.peg.5389	fig|6666666.59347.peg.3979	None	fig|6666666.59348.peg.4935	None	None	None	None	None	None

1688	Polysaccharide deacetylase	None	None	None	None	None	None	None	None	fig|6666666.59338.peg.1292	None	None	None	None	fig|6666666.71181.peg.6118	fig|6666666.59344.peg.4430	None	None	None	None	None	fig|6666666.59349.peg.2199	None	fig|6666666.59351.peg.4371	fig|6666666.59353.peg.3199	fig|6666666.59355.peg.3080	fig|6666666.59354.peg.6466

7297	DNA-binding protein	None	None	None	None	None	None	None	None	None	None	None	None	None	fig|6666666.71181.peg.933	fig|6666666.59344.peg.3536	None	None	None	None	None	fig|6666666.59349.peg.332	fig|6666666.59350.peg.5161	fig|6666666.59351.peg.3939	fig|6666666.59353.peg.881	fig|6666666.59355.peg.823	fig|6666666.59354.peg.4179

6455	Maleate cis-trans isomerase EC 5.2.1.1	None	None	None	None	None	fig|6666666.59335.peg.1896	None	None	None	fig|6666666.59339.peg.4745	None	None	None	fig|6666666.71181.peg.4858	fig|6666666.59344.peg.6160	None	fig|6666666.59341.peg.5692	None	None	None	None	fig|6666666.59350.peg.3374	None	fig|6666666.59353.peg.4157	fig|6666666.59355.peg.4258	None

6454	Quorum-sensing transcriptional activator YspR	None	None	None	None	None	fig|6666666.59335.peg.3239	None	None	None	None	None	None	None	fig|6666666.71181.peg.6049	fig|6666666.59344.peg.2079	None	fig|6666666.59341.peg.2266	None	None	None	None	fig|6666666.59350.peg.2547	fig|6666666.59351.peg.5397	fig|6666666.59353.peg.4115	fig|6666666.59355.peg.2324	None

6457	hypothetical protein	fig|6666666.59329.peg.3350	None	fig|6666666.59331.peg.3500	None	None	None	fig|6666666.59336.peg.4522	None	fig|6666666.59338.peg.4030	None	None	fig|6666666.59337.peg.2237	None	None	None	fig|6666666.59345.peg.2979	fig|6666666.59341.peg.2172	fig|6666666.59347.peg.5269	None	None	None	None	None	None	None	None

3602	4-amino-4-deoxy-L-arabinose transferase	fig|6666666.59329.peg.6172	fig|6666666.59330.peg.4908	None	None	fig|6666666.59334.peg.1607	None	None	None	None	None	None	fig|6666666.59337.peg.4035	fig|6666666.59342.peg.3858	None	None	fig|6666666.59345.peg.212	None	fig|6666666.59347.peg.1529	None	fig|6666666.59348.peg.2181	None	None	None	None	None	None

10105	FIG01203892 hypothetical protein	fig|6666666.59329.peg.3042	None	None	None	None	fig|6666666.59335.peg.1773	fig|6666666.59336.peg.5069	fig|6666666.59333.peg.5342	None	fig|6666666.59339.peg.5788	None	fig|6666666.59337.peg.4090	fig|6666666.59342.peg.4677	None	None	fig|6666666.59345.peg.1690	None	None	None	None	None	None	None	None	None	None

2821	putative inner membrane or exported protein	fig|6666666.59329.peg.5130	None	None	None	None	fig|6666666.59335.peg.1885	fig|6666666.59336.peg.4249	fig|6666666.59333.peg.1128	None	fig|6666666.59339.peg.4735	None	None	fig|6666666.59342.peg.5920	None	None	fig|6666666.59345.peg.2034	None	fig|6666666.59347.peg.3630	None	None	None	None	None	None	None	None

10937	Alcohol dehydrogenase, class IV	fig|6666666.59329.peg.1494	fig|6666666.59330.peg.4506	None	None	None	None	fig|6666666.59336.peg.749	None	None	None	None	fig|6666666.59337.peg.1266	None	None	None	fig|6666666.59345.peg.4453	None	None	None	None	fig|6666666.59349.peg.3126	None	fig|6666666.59351.peg.4572	None	None	fig|6666666.59354.peg.5918

9115	Alternative oxidase 2, mitochondrial precursor EC 1.-.-.-	None	fig|6666666.59330.peg.1734	None	None	fig|6666666.59334.peg.3131	None	fig|6666666.59336.peg.2438	None	fig|6666666.59338.peg.3228	None	None	fig|6666666.59337.peg.102	None	None	None	fig|6666666.59345.peg.3740	fig|6666666.59341.peg.1282	fig|6666666.59347.peg.3418	None	None	None	None	None	None	None	None

2014	Glyoxalase family protein	None	None	None	None	None	fig|6666666.59335.peg.4313	None	None	None	fig|6666666.59339.peg.1737	None	None	None	fig|6666666.71181.peg.3991	fig|6666666.59344.peg.5636	None	None	None	None	fig|6666666.59348.peg.2203	None	fig|6666666.59350.peg.3080	None	fig|6666666.59353.peg.2970	fig|6666666.59355.peg.2875	None

9444	Endoribonuclease L-PSP	fig|6666666.59329.peg.3878	None	None	None	None	fig|6666666.59335.peg.759	fig|6666666.59336.peg.3089	None	None	fig|6666666.59339.peg.5595	None	None	fig|6666666.59342.peg.6040	None	None	fig|6666666.59345.peg.4544	fig|6666666.59341.peg.5220	None	None	fig|6666666.59348.peg.1858	None	None	None	None	None	None

8118	4-deoxy-L-threo-5-hexosulose-uronate ketol-isomerase EC 5.3.1.17	fig|6666666.59329.peg.5100	None	None	None	None	None	None	None	None	None	None	None	None	fig|6666666.71181.peg.4330	fig|6666666.59344.peg.829	None	fig|6666666.59341.peg.2194	fig|6666666.59347.peg.3814	None	None	None	fig|6666666.59350.peg.5708	None	fig|6666666.59353.peg.1282	fig|6666666.59355.peg.3300	None

7712	Uncharacterized GST-like protein yibF	None	None	None	None	None	None	None	None	None	None	None	None	None	fig|6666666.71181.peg.677	fig|6666666.59344.peg.4108	None	None	None	None	None	fig|6666666.59349.peg.3157	fig|6666666.59350.peg.4550	fig|6666666.59351.peg.3082	fig|6666666.59353.peg.4088	fig|6666666.59355.peg.2351	fig|6666666.59354.peg.2835

9300	hypothetical protein	None	None	fig|6666666.59331.peg.673	None	None	None	fig|6666666.59336.peg.4171	None	fig|6666666.59338.peg.3028	fig|6666666.59339.peg.740	None	None	None	None	None	fig|6666666.59345.peg.2796	fig|6666666.59341.peg.3202	fig|6666666.59347.peg.3824	fig|6666666.59346.peg.2363	None	None	None	None	None	None	None

8965	YheO-like PAS domain	fig|6666666.59329.peg.2597	fig|6666666.59330.peg.4126	None	None	fig|6666666.59334.peg.2769	None	fig|6666666.59336.peg.1241	None	None	None	None	fig|6666666.59337.peg.3744	fig|6666666.59342.peg.3904	None	None	fig|6666666.59345.peg.1185	None	None	None	fig|6666666.59348.peg.3103	None	None	None	None	None	None

5880	hypothetical protein	fig|6666666.59329.peg.4598	None	None	None	None	None	None	fig|6666666.59333.peg.3870	None	None	None	fig|6666666.59337.peg.1012	None	None	None	fig|6666666.59345.peg.4268	None	None	None	fig|6666666.59348.peg.4003	fig|6666666.59349.peg.1987	None	fig|6666666.59351.peg.1756	None	None	fig|6666666.59354.peg.2774

11075	FIG01205343 hypothetical protein	None	fig|6666666.59330.peg.663	None	None	None	None	fig|6666666.59336.peg.701	None	fig|6666666.59338.peg.5795	None	None	fig|6666666.59337.peg.5183	fig|6666666.59342.peg.358	None	None	fig|6666666.59345.peg.2314	fig|6666666.59341.peg.4291	fig|6666666.59347.peg.1930	None	None	None	None	None	None	None	None

3199	FIG01201582 hypothetical protein	fig|6666666.59329.peg.2707	fig|6666666.59330.peg.5115	None	None	None	None	fig|6666666.59336.peg.5938	fig|6666666.59333.peg.5161	None	None	None	fig|6666666.59337.peg.5346	fig|6666666.59342.peg.3415	None	None	fig|6666666.59345.peg.415	fig|6666666.59341.peg.5477	None	None	None	None	None	None	None	None	None

1626	FIG01201118 hypothetical protein	fig|6666666.59329.peg.4559	None	fig|6666666.59331.peg.242	None	fig|6666666.59334.peg.3491	fig|6666666.59335.peg.3502	None	None	None	fig|6666666.59339.peg.6817	None	fig|6666666.59337.peg.1329	None	None	None	None	None	None	fig|6666666.59346.peg.4790	fig|6666666.59348.peg.991	None	None	None	None	None	None

1625	putative exported protein	fig|6666666.59329.peg.3271	fig|6666666.59330.peg.4744	None	None	fig|6666666.59334.peg.1435	None	fig|6666666.59336.peg.4229	None	None	None	None	fig|6666666.59337.peg.4086	fig|6666666.59342.peg.4849	None	None	fig|6666666.59345.peg.1685	None	None	fig|6666666.59346.peg.5229	None	None	None	None	None	None	None

2050	FIG01201199 hypothetical protein	fig|6666666.59329.peg.1628	None	None	None	None	None	fig|6666666.59336.peg.5025	None	None	None	None	fig|6666666.59337.peg.4373	None	fig|6666666.71181.peg.3026	fig|6666666.59344.peg.4450	fig|6666666.59345.peg.1498	None	None	None	None	None	None	None	fig|6666666.59353.peg.4170	fig|6666666.59355.peg.1707	None

2423	FIG01201640 hypothetical protein	fig|6666666.59329.peg.2216	fig|6666666.59330.peg.1797	None	None	None	None	fig|6666666.59336.peg.1037	fig|6666666.59333.peg.2379	None	None	None	fig|6666666.59337.peg.3396	None	None	None	fig|6666666.59345.peg.1995	None	None	None	None	fig|6666666.59349.peg.4827	None	None	None	None	fig|6666666.59354.peg.1125

2426	ADP-ribosylglycohydrolase family protein	fig|6666666.59329.peg.1165	fig|6666666.59330.peg.3694	None	None	None	None	fig|6666666.59336.peg.4816	None	fig|6666666.59338.peg.4962	None	None	fig|6666666.59337.peg.2986	fig|6666666.59342.peg.6053	None	None	fig|6666666.59345.peg.652	None	fig|6666666.59347.peg.767	None	None	None	None	None	None	None	None

6354	Phosphoserine phosphatase EC 3.1.3.3	fig|6666666.59329.peg.484	fig|6666666.59330.peg.1620	None	None	None	None	fig|6666666.59336.peg.6302	fig|6666666.59333.peg.3995	fig|6666666.59338.peg.1834	None	None	fig|6666666.59337.peg.1285	None	None	None	fig|6666666.59345.peg.4472	None	fig|6666666.59347.peg.5301	None	None	None	None	None	None	None	None

1974	Fic family protein	None	None	fig|6666666.59331.peg.5262	fig|6666666.59332.peg.2962	None	None	None	None	None	None	None	None	None	fig|6666666.71181.peg.4380	fig|6666666.59344.peg.4803	None	None	None	None	None	None	fig|6666666.59350.peg.3268	fig|6666666.59351.peg.2448	fig|6666666.59353.peg.4109	fig|6666666.59355.peg.2330	None

8459	Excinuclease ABC, C subunit-like	fig|6666666.59329.peg.5907	None	None	fig|6666666.59332.peg.383	None	None	None	fig|6666666.59333.peg.1387	fig|6666666.59338.peg.262	None	fig|6666666.59340.peg.25	None	fig|6666666.59342.peg.5397	None	None	None	fig|6666666.59341.peg.5728	fig|6666666.59347.peg.5202	None	None	None	None	None	None	None	None

11008	Cytochrome c553	None	None	None	None	None	None	fig|6666666.59336.peg.3795	fig|6666666.59333.peg.5336	fig|6666666.59338.peg.5509	None	None	None	fig|6666666.59342.peg.500	None	None	fig|6666666.59345.peg.3474	fig|6666666.59341.peg.2822	fig|6666666.59347.peg.1237	None	fig|6666666.59348.peg.920	None	None	None	None	None	None

1431	FIG01199934 hypothetical protein	fig|6666666.59329.peg.455	fig|6666666.59330.peg.327	None	None	fig|6666666.59334.peg.5400	None	fig|6666666.59336.peg.6500	fig|6666666.59333.peg.4942	None	None	None	fig|6666666.59337.peg.1057	fig|6666666.59342.peg.5804	None	None	fig|6666666.59345.peg.1662	None	None	None	None	None	None	None	None	None	None

9364	FIG01201414 hypothetical protein	None	None	None	fig|6666666.59332.peg.5720	fig|6666666.59334.peg.3674	fig|6666666.59335.peg.2360	None	None	None	fig|6666666.59339.peg.2887	None	None	None	None	None	fig|6666666.59345.peg.119	None	None	fig|6666666.59346.peg.2921	fig|6666666.59348.peg.2915	None	fig|6666666.59350.peg.2489	None	None	None	None

2234	FIG01202607 hypothetical protein	fig|6666666.59329.peg.3895	fig|6666666.59330.peg.2919	None	None	None	fig|6666666.59335.peg.4015	fig|6666666.59336.peg.910	None	None	fig|6666666.59339.peg.3807	None	fig|6666666.59337.peg.3201	fig|6666666.59342.peg.5060	None	None	fig|6666666.59345.peg.2900	None	None	None	None	None	None	None	None	None	None

7792	probable exported protein YPO3473	None	None	fig|6666666.59331.peg.4115	None	None	None	fig|6666666.59336.peg.382	None	None	None	fig|6666666.59340.peg.801	None	fig|6666666.59342.peg.2067	None	None	fig|6666666.59345.peg.1382	None	None	fig|6666666.59346.peg.4417	None	fig|6666666.59349.peg.2059	None	None	None	None	fig|6666666.59354.peg.738

1033	FIG00514064 hypothetical protein	None	None	fig|6666666.59331.peg.3604	fig|6666666.59332.peg.5342	None	fig|6666666.59335.peg.3465	None	fig|6666666.59333.peg.2172	None	fig|6666666.59339.peg.2128	None	fig|6666666.59337.peg.3416	None	None	None	fig|6666666.59345.peg.1977	None	None	None	fig|6666666.59348.peg.3134	None	None	None	None	None	None

8081	FIG01206490 hypothetical protein	fig|6666666.59329.peg.4658	fig|6666666.59330.peg.1955	None	None	None	None	fig|6666666.59336.peg.5327	fig|6666666.59333.peg.3531	None	None	None	fig|6666666.59337.peg.2655	fig|6666666.59342.peg.4857	None	None	fig|6666666.59345.peg.702	None	None	None	None	None	None	fig|6666666.59351.peg.43	None	None	None

6881	Ureidoglycolate hydrolase EC 3.5.3.19	None	None	None	fig|6666666.59332.peg.2248	None	fig|6666666.59335.peg.1143	None	None	fig|6666666.59338.peg.5385	fig|6666666.59339.peg.1650	None	None	None	None	None	None	fig|6666666.59341.peg.4528	fig|6666666.59347.peg.3327	None	fig|6666666.59348.peg.2417	None	fig|6666666.59350.peg.3207	None	None	None	None

9809	hypothetical protein	fig|6666666.59329.peg.2137	fig|6666666.59330.peg.3310	fig|6666666.59331.peg.1361	fig|6666666.59332.peg.4997	None	None	None	None	None	None	None	fig|6666666.59337.peg.3098	fig|6666666.59342.peg.5122	None	None	fig|6666666.59345.peg.328	None	None	fig|6666666.59346.peg.4903	None	None	None	None	None	None	None

7581	Glucose-methanol-choline GMC oxidoreductase NAD binding site	None	None	None	None	None	None	None	None	None	None	None	None	None	fig|6666666.71181.peg.3876	fig|6666666.59344.peg.875	None	None	None	None	None	fig|6666666.59349.peg.2111	fig|6666666.59350.peg.520	fig|6666666.59351.peg.981	fig|6666666.59353.peg.3854	fig|6666666.59355.peg.3341	fig|6666666.59354.peg.3959

2944	hypothetical protein	None	None	fig|6666666.59331.peg.1458	None	None	fig|6666666.59335.peg.2188	fig|6666666.59336.peg.4593	None	None	fig|6666666.59339.peg.3932	fig|6666666.59340.peg.3814	None	fig|6666666.59342.peg.3586	None	None	None	fig|6666666.59341.peg.5309	None	fig|6666666.59346.peg.342	None	None	None	None	None	None	None

4316	Phosphodiesterase alkaline phosphatase D	None	fig|6666666.59330.peg.5062	None	None	None	None	fig|6666666.59336.peg.2903	fig|6666666.59333.peg.3665	None	None	None	fig|6666666.59337.peg.4144	fig|6666666.59342.peg.4178	None	None	fig|6666666.59345.peg.4612	None	None	None	None	fig|6666666.59349.peg.3613	None	fig|6666666.59351.peg.2992	None	None	None

3114	N-acetylmannosamine-6-phosphate 2-epimerase EC 5.1.3.9	None	None	None	None	None	None	None	None	None	fig|6666666.59339.peg.737	None	None	fig|6666666.59342.peg.4376	fig|6666666.71181.peg.3917	fig|6666666.59344.peg.385	None	fig|6666666.59341.peg.1617	None	None	fig|6666666.59348.peg.5297	None	None	None	fig|6666666.59353.peg.1605	fig|6666666.59355.peg.1116	None

1870	FIG01205881 hypothetical protein	None	fig|6666666.59330.peg.519	fig|6666666.59331.peg.2993	fig|6666666.59332.peg.1397	None	None	fig|6666666.59336.peg.5085	None	None	fig|6666666.59339.peg.6027	None	fig|6666666.59337.peg.5820	fig|6666666.59342.peg.3573	None	None	fig|6666666.59345.peg.4639	None	None	None	None	None	None	None	None	None	None

4947	Phosphohydrolase MutT nudix family protein	None	fig|6666666.59330.peg.3077	None	None	None	None	None	fig|6666666.59333.peg.3140	None	None	None	fig|6666666.59337.peg.1489	None	fig|6666666.71181.peg.4295	fig|6666666.59344.peg.6253	None	None	None	None	None	None	fig|6666666.59350.peg.2321	None	fig|6666666.59353.peg.4004	fig|6666666.59355.peg.3963	None

2116	hypothetical protein	fig|6666666.59329.peg.3705	None	fig|6666666.59331.peg.5041	fig|6666666.59332.peg.3335	fig|6666666.59334.peg.4977	fig|6666666.59335.peg.6015	fig|6666666.59336.peg.5053	None	None	None	None	None	fig|6666666.59342.peg.4665	None	None	None	None	None	fig|6666666.59346.peg.3902	None	None	None	None	None	None	None

9238	hypothetical protein	None	None	fig|6666666.59331.peg.1567	fig|6666666.59332.peg.3307	fig|6666666.59334.peg.2743	None	None	None	fig|6666666.59338.peg.2912	None	None	fig|6666666.59337.peg.2288	fig|6666666.59342.peg.5170	None	None	None	None	fig|6666666.59347.peg.689	fig|6666666.59346.peg.1334	None	None	None	None	None	None	None

8783	DNA sulfur modification protein DndB	None	None	None	fig|6666666.59332.peg.5314	fig|6666666.59334.peg.4513	None	fig|6666666.59336.peg.3326	None	None	fig|6666666.59339.peg.8948	None	None	None	fig|6666666.71181.peg.694	fig|6666666.59344.peg.665	None	None	None	None	None	None	None	None	fig|6666666.59353.peg.1003	fig|6666666.59355.peg.2369	None

5687	Metal-dependent hydrolase involved in phosphonate metabolism	None	None	None	None	None	None	None	None	fig|6666666.59338.peg.838	fig|6666666.59339.peg.7024	None	None	None	fig|6666666.71181.peg.3526	fig|6666666.59344.peg.4613	None	None	fig|6666666.59347.peg.102	None	None	None	fig|6666666.59350.peg.2345	None	fig|6666666.59353.peg.2402	fig|6666666.59355.peg.493	None

5978	Transcriptional regulator, HxlR family	None	None	None	None	None	None	fig|6666666.59336.peg.6376	None	None	fig|6666666.59339.peg.2082	None	None	None	fig|6666666.71181.peg.4214	fig|6666666.59344.peg.1185	fig|6666666.59345.peg.781	None	None	None	None	fig|6666666.59349.peg.4958	None	None	fig|6666666.59353.peg.2739	fig|6666666.59355.peg.1743	None

10005	FIG01200677 hypothetical protein	None	fig|6666666.59330.peg.3926	None	None	None	fig|6666666.59335.peg.4195	fig|6666666.59336.peg.4355	fig|6666666.59333.peg.2625	None	None	None	fig|6666666.59337.peg.2674	fig|6666666.59342.peg.5486	None	None	fig|6666666.59345.peg.1050	None	None	None	fig|6666666.59348.peg.2020	None	None	None	None	None	None

648	hypothetical protein	fig|6666666.59329.peg.3721	None	fig|6666666.59331.peg.4136	None	None	None	fig|6666666.59336.peg.1865	None	fig|6666666.59338.peg.2450	None	fig|6666666.59340.peg.2475	fig|6666666.59337.peg.5418	None	None	None	fig|6666666.59345.peg.569	None	None	fig|6666666.59346.peg.3229	None	None	None	None	None	None	None

5774	Oxalate formate antiporter	None	None	fig|6666666.59331.peg.443	None	None	None	fig|6666666.59336.peg.3593	fig|6666666.59333.peg.3437	None	fig|6666666.59339.peg.4577	None	None	fig|6666666.59342.peg.3053	None	fig|6666666.59344.peg.2054	None	None	None	fig|6666666.59346.peg.1265	fig|6666666.59348.peg.864	None	None	None	None	None	None

10280	FIG01202831 hypothetical protein	None	fig|6666666.59330.peg.4096	None	None	None	None	fig|6666666.59336.peg.6324	fig|6666666.59333.peg.816	None	None	None	fig|6666666.59337.peg.2856	fig|6666666.59342.peg.4949	None	None	fig|6666666.59345.peg.1740	fig|6666666.59341.peg.2812	None	None	fig|6666666.59348.peg.1806	None	None	None	None	None	None

7406	PhnJ protein	None	None	None	None	None	None	None	None	fig|6666666.59338.peg.835	None	None	None	None	fig|6666666.71181.peg.3523	fig|6666666.59344.peg.4616	None	fig|6666666.59341.peg.4383	fig|6666666.59347.peg.105	None	None	None	fig|6666666.59350.peg.2340	None	fig|6666666.59353.peg.2405	fig|6666666.59355.peg.496	None

3718	Hydrolases of the alpha beta superfamily	None	None	fig|6666666.59331.peg.1989	fig|6666666.59332.peg.331	None	fig|6666666.59335.peg.668	None	None	None	None	None	None	fig|6666666.59342.peg.5124	None	None	fig|6666666.59345.peg.326	None	None	None	None	fig|6666666.59349.peg.2360	fig|6666666.59350.peg.2840	None	None	None	fig|6666666.59354.peg.4665

3394	Permeases of the drug metabolite transporter DMT superfamily	None	None	fig|6666666.59331.peg.495	None	fig|6666666.59334.peg.3575	None	None	None	fig|6666666.59338.peg.1018	None	None	None	None	None	None	None	None	None	fig|6666666.59346.peg.3471	None	fig|6666666.59349.peg.1545	fig|6666666.59350.peg.2142	fig|6666666.59351.peg.4741	None	None	fig|6666666.59354.peg.3538

1209	tRNA Cytosine32 -2-thiocytidine synthetase	None	None	None	fig|6666666.59332.peg.5668	fig|6666666.59334.peg.2624	None	fig|6666666.59336.peg.3524	fig|6666666.59333.peg.4757	None	None	None	None	fig|6666666.59342.peg.599	None	fig|6666666.59344.peg.550	None	None	None	None	None	None	None	None	fig|6666666.59353.peg.1855	fig|6666666.59355.peg.1158	None

7889	2,3-bisphosphoglycerate-independent phosphoglycerate mutase EC 5.4.2.1	None	None	fig|6666666.59331.peg.2867	None	None	None	fig|6666666.59336.peg.1821	None	fig|6666666.59338.peg.1165	fig|6666666.59339.peg.8825	fig|6666666.59340.peg.937	None	None	None	fig|6666666.59344.peg.968	None	None	None	None	fig|6666666.59348.peg.1967	None	None	None	fig|6666666.59353.peg.2570	None	None

10383	Succinylarginine dihydrolase EC 3.5.3.23	None	None	None	None	fig|6666666.59334.peg.3197	None	None	None	None	None	None	None	None	fig|6666666.71181.peg.4526	fig|6666666.59344.peg.2605	None	None	None	None	None	fig|6666666.59349.peg.3830	None	fig|6666666.59351.peg.1136	fig|6666666.59353.peg.1562	fig|6666666.59355.peg.2111	fig|6666666.59354.peg.4207

6203	Alpha-ketoglutarate-dependent taurine dioxygenase EC 1.14.11.17	None	fig|6666666.59330.peg.4408	None	None	None	fig|6666666.59335.peg.1687	None	None	None	fig|6666666.59339.peg.3665	None	fig|6666666.59337.peg.2421	None	fig|6666666.71181.peg.4301	fig|6666666.59344.peg.3119	None	None	None	None	None	None	None	None	fig|6666666.59353.peg.2262	fig|6666666.59355.peg.1955	None

9243	Sodium-dependent transporter	None	None	None	None	fig|6666666.59334.peg.3301	None	None	None	None	fig|6666666.59339.peg.6054	None	None	None	fig|6666666.71181.peg.2021	None	None	None	None	None	None	fig|6666666.59349.peg.2920	None	fig|6666666.59351.peg.4581	fig|6666666.59353.peg.3018	fig|6666666.59355.peg.2925	fig|6666666.59354.peg.5179

3690	2-keto-3-deoxy-D-arabino-heptulosonate-7-phosphate synthase II EC 2.5.1.54	None	None	None	None	None	None	None	None	None	None	None	None	None	fig|6666666.71181.peg.2910	fig|6666666.59344.peg.3467	None	None	None	None	None	fig|6666666.59349.peg.4154	fig|6666666.59350.peg.596	fig|6666666.59351.peg.1705	fig|6666666.59353.peg.1409	fig|6666666.59355.peg.2465	fig|6666666.59354.peg.3316

5872	MoaE protein	None	None	None	None	None	None	None	None	None	None	None	fig|6666666.59337.peg.453	None	fig|6666666.71181.peg.4251	fig|6666666.59344.peg.5695	fig|6666666.59345.peg.2602	None	None	fig|6666666.59346.peg.3336	None	None	None	fig|6666666.59351.peg.2860	fig|6666666.59353.peg.2512	fig|6666666.59355.peg.603	None

3756	putative ORF1 [Plasmid pTOM9]	None	None	fig|6666666.59331.peg.4585	None	fig|6666666.59334.peg.3677	fig|6666666.59335.peg.2988	fig|6666666.59336.peg.4994	None	None	fig|6666666.59339.peg.4588	None	None	fig|6666666.59342.peg.5435	None	None	None	None	None	None	fig|6666666.59348.peg.4210	None	fig|6666666.59350.peg.4801	None	None	None	None

165	putative sugar transport system permease	fig|6666666.59329.peg.1441	fig|6666666.59330.peg.3570	fig|6666666.59331.peg.2020	None	None	None	fig|6666666.59336.peg.4574	fig|6666666.59333.peg.3868	None	None	None	fig|6666666.59337.peg.1013	fig|6666666.59342.peg.4736	None	None	fig|6666666.59345.peg.4267	None	None	None	None	None	None	None	None	None	None

9769	FIG01203265 hypothetical protein	fig|6666666.59329.peg.5039	fig|6666666.59330.peg.3745	None	None	fig|6666666.59334.peg.4022	None	fig|6666666.59336.peg.6543	fig|6666666.59333.peg.4434	None	None	None	fig|6666666.59337.peg.1843	fig|6666666.59342.peg.4971	None	None	fig|6666666.59345.peg.4210	None	None	None	None	None	None	None	None	None	None

1771	poly beta-D-mannuronate lyase EC 4.2.2.3	None	None	None	None	None	None	fig|6666666.59336.peg.2197	None	fig|6666666.59338.peg.3989	None	fig|6666666.59340.peg.1075	None	fig|6666666.59342.peg.1489	None	None	fig|6666666.59345.peg.1352	fig|6666666.59341.peg.3153	fig|6666666.59347.peg.1075	fig|6666666.59346.peg.2858	None	None	None	None	None	None	None

4771	Tryptophan synthase beta chain like EC 4.2.1.20	None	None	None	None	None	None	None	None	fig|6666666.59338.peg.3756	None	None	None	None	fig|6666666.71181.peg.2427	fig|6666666.59344.peg.2492	None	fig|6666666.59341.peg.5269	fig|6666666.59347.peg.2249	None	None	None	None	fig|6666666.59351.peg.1533	fig|6666666.59353.peg.3626	fig|6666666.59355.peg.4998	None

4406	FIG01200163 hypothetical protein	fig|6666666.59329.peg.3551	fig|6666666.59330.peg.4391	None	None	None	None	fig|6666666.59336.peg.4885	fig|6666666.59333.peg.4570	None	fig|6666666.59339.peg.2712	None	fig|6666666.59337.peg.5011	fig|6666666.59342.peg.2839	None	None	fig|6666666.59345.peg.3371	None	None	None	None	None	None	None	None	None	None

783	ATP-dependent protease La EC 3.4.21.53 Type II	None	None	None	fig|6666666.59332.peg.3329	fig|6666666.59334.peg.3458	fig|6666666.59335.peg.5477	None	fig|6666666.59333.peg.4167	None	fig|6666666.59339.peg.2620	fig|6666666.59340.peg.918	None	None	None	None	None	None	None	None	None	fig|6666666.59349.peg.4127	None	fig|6666666.59351.peg.3631	None	None	None

7487	Carbamate kinase EC 2.7.2.2	None	None	None	None	fig|6666666.59334.peg.5306	fig|6666666.59335.peg.467	fig|6666666.59336.peg.4887	None	fig|6666666.59338.peg.2819	fig|6666666.59339.peg.6481	None	None	fig|6666666.59342.peg.3608	None	None	None	fig|6666666.59341.peg.4434	fig|6666666.59347.peg.2612	None	None	None	None	None	None	None	None

1104	O-antigen acetylase	None	None	None	None	None	None	None	fig|6666666.59333.peg.3115	None	None	None	fig|6666666.59337.peg.4406	None	fig|6666666.71181.peg.3187	fig|6666666.59344.peg.1658	fig|6666666.59345.peg.3428	None	fig|6666666.59347.peg.5366	None	None	None	None	None	fig|6666666.59353.peg.1081	fig|6666666.59355.peg.398	None

8601	FIG01201424 hypothetical protein	fig|6666666.59329.peg.3633	fig|6666666.59330.peg.2338	None	None	fig|6666666.59334.peg.1617	None	fig|6666666.59336.peg.2447	None	fig|6666666.59338.peg.434	None	fig|6666666.59340.peg.1417	fig|6666666.59337.peg.3602	None	None	None	fig|6666666.59345.peg.1858	None	None	None	None	None	None	None	None	None	None

849	Phosphoenolpyruvate-dihydroxyacetone phosphotransferase EC 2.7.1.121 , subunit DhaM; DHA-specific IIA component DHA-specific phosphocarrier protein HPr DHA-specific EI component	None	None	None	None	None	None	None	fig|6666666.59333.peg.105	fig|6666666.59338.peg.1738	None	None	None	None	fig|6666666.71181.peg.3030	fig|6666666.59344.peg.4446	None	fig|6666666.59341.peg.1455	fig|6666666.59347.peg.4235	None	None	None	None	None	fig|6666666.59353.peg.4166	fig|6666666.59355.peg.1711	None

1470	Mobile element protein	None	None	None	None	None	None	None	fig|6666666.59333.peg.5566	None	None	None	None	None	fig|6666666.71181.peg.4475	fig|6666666.59344.peg.350	None	None	None	None	None	fig|6666666.59349.peg.4987	fig|6666666.59350.peg.8371	None	fig|6666666.59353.peg.537	fig|6666666.59355.peg.22	fig|6666666.59354.peg.5251

5589	FIG01199847 hypothetical protein	None	None	fig|6666666.59331.peg.1997	fig|6666666.59332.peg.5303	None	fig|6666666.59335.peg.2801	None	None	fig|6666666.59338.peg.3647	fig|6666666.59339.peg.1972	None	None	None	None	None	None	None	fig|6666666.59347.peg.294	fig|6666666.59346.peg.3422	fig|6666666.59348.peg.1581	None	None	None	None	None	None

2056	FIG01202483 hypothetical protein	fig|6666666.59329.peg.918	None	None	None	None	fig|6666666.59335.peg.4711	fig|6666666.59336.peg.401	fig|6666666.59333.peg.3019	None	fig|6666666.59339.peg.4392	None	fig|6666666.59337.peg.5213	fig|6666666.59342.peg.5466	None	None	fig|6666666.59345.peg.1832	None	None	None	None	None	None	None	None	None	None

869	FIG01201732 hypothetical protein	fig|6666666.59329.peg.4045	None	fig|6666666.59331.peg.2041	fig|6666666.59332.peg.6232	None	None	fig|6666666.59336.peg.2120	fig|6666666.59333.peg.829	None	None	None	None	fig|6666666.59342.peg.5363	None	None	fig|6666666.59345.peg.1720	None	None	fig|6666666.59346.peg.2838	None	None	None	None	None	None	None

6449	hypothetical protein	None	fig|6666666.59330.peg.2804	None	None	fig|6666666.59334.peg.1849	fig|6666666.59335.peg.4232	fig|6666666.59336.peg.726	None	None	fig|6666666.59339.peg.1021	None	None	fig|6666666.59342.peg.4940	None	None	None	None	None	fig|6666666.59346.peg.5038	fig|6666666.59348.peg.3165	None	None	None	None	None	None

965	GCN5-related N-acetyltransferase	None	None	None	None	None	None	None	None	fig|6666666.59338.peg.5740	None	None	None	None	fig|6666666.71181.peg.2338	None	None	fig|6666666.59341.peg.234	fig|6666666.59347.peg.554	None	fig|6666666.59348.peg.5134	fig|6666666.59349.peg.3531	None	fig|6666666.59351.peg.1057	None	None	fig|6666666.59354.peg.4214

5563	2,3,4,5-tetrahydropyridine-2,6-dicarboxylate N-succinyltransferase EC 2.3.1.117	None	None	None	None	None	None	None	None	None	None	None	None	None	fig|6666666.71181.peg.38	fig|6666666.59344.peg.2648	None	None	None	None	None	fig|6666666.59349.peg.1395	fig|6666666.59350.peg.212	fig|6666666.59351.peg.74	fig|6666666.59353.peg.1980	fig|6666666.59355.peg.4087	fig|6666666.59354.peg.306

9101	FIG01200247 hypothetical protein	fig|6666666.59329.peg.3110	fig|6666666.59330.peg.699	None	None	fig|6666666.59334.peg.756	None	fig|6666666.59336.peg.1546	fig|6666666.59333.peg.4322	None	None	None	fig|6666666.59337.peg.4150	fig|6666666.59342.peg.2880	None	None	fig|6666666.59345.peg.4791	None	None	None	None	None	None	None	None	None	None

11407	FIG01199998 hypothetical protein	None	None	fig|6666666.59331.peg.3816	None	fig|6666666.59334.peg.1962	None	None	fig|6666666.59333.peg.4068	fig|6666666.59338.peg.5412	None	fig|6666666.59340.peg.2957	None	fig|6666666.59342.peg.3083	None	None	fig|6666666.59345.peg.1389	None	fig|6666666.59347.peg.2478	None	None	None	None	None	None	None	None

8768	tRNA pseudouridine synthase B EC 4.2.1.70	None	None	None	None	fig|6666666.59334.peg.775	fig|6666666.59335.peg.2097	fig|6666666.59336.peg.6413	fig|6666666.59333.peg.2322	None	fig|6666666.59339.peg.4899	fig|6666666.59340.peg.955	None	None	None	None	None	None	None	None	None	fig|6666666.59349.peg.1437	None	None	None	None	fig|6666666.59354.peg.5106

6325	3-keto-L-gulonate 6-phosphate decarboxylase	fig|6666666.59329.peg.2159	None	fig|6666666.59331.peg.671	fig|6666666.59332.peg.2911	fig|6666666.59334.peg.1228	None	fig|6666666.59336.peg.3189	None	None	None	None	None	None	None	None	fig|6666666.59345.peg.4532	fig|6666666.59341.peg.4122	None	fig|6666666.59346.peg.2360	None	None	None	None	None	None	None

6320	hypothetical protein	None	fig|6666666.59330.peg.3401	None	None	None	fig|6666666.59335.peg.973	fig|6666666.59336.peg.2975	None	None	fig|6666666.59339.peg.4476	None	None	None	None	None	fig|6666666.59345.peg.937	fig|6666666.59341.peg.225	None	None	None	fig|6666666.59349.peg.2528	None	None	None	None	fig|6666666.59354.peg.5127

9664	Oxygen-independent coproporphyrinogen III oxidase, Fe-S oxidoreductase	fig|6666666.59329.peg.2616	None	None	fig|6666666.59332.peg.3062	fig|6666666.59334.peg.5033	None	fig|6666666.59336.peg.735	None	None	None	None	None	None	None	None	fig|6666666.59345.peg.3917	fig|6666666.59341.peg.3230	fig|6666666.59347.peg.808	fig|6666666.59346.peg.4840	None	None	None	None	None	None	None

10066	Cell division protein MraZ	None	None	None	None	None	None	None	None	None	None	None	None	None	fig|6666666.71181.peg.370	fig|6666666.59344.peg.1491	None	None	None	None	None	fig|6666666.59349.peg.2975	fig|6666666.59350.peg.3520	fig|6666666.59351.peg.199	fig|6666666.59353.peg.128	fig|6666666.59355.peg.739	fig|6666666.59354.peg.3670

3185	FIG107774 hypothetical protein	fig|6666666.59329.peg.4257	None	fig|6666666.59331.peg.1006	None	None	fig|6666666.59335.peg.4058	fig|6666666.59336.peg.1078	None	None	fig|6666666.59339.peg.6261	None	None	fig|6666666.59342.peg.743	None	None	None	None	None	fig|6666666.59346.peg.4625	fig|6666666.59348.peg.118	None	None	None	None	None	None

10428	ATPase involved in DNA repair	fig|6666666.59329.peg.2990	fig|6666666.59330.peg.2352	None	None	fig|6666666.59334.peg.3702	None	fig|6666666.59336.peg.406	fig|6666666.59333.peg.3376	None	None	None	fig|6666666.59337.peg.3622	None	None	None	fig|6666666.59345.peg.1836	fig|6666666.59341.peg.1840	None	None	None	None	None	None	None	None	None

2810	AmpG permease	None	None	fig|6666666.59331.peg.6159	fig|6666666.59332.peg.3946	None	fig|6666666.59335.peg.684	None	None	None	None	None	None	None	fig|6666666.71181.peg.4911	fig|6666666.59344.peg.6084	None	None	None	None	fig|6666666.59348.peg.5295	None	None	None	fig|6666666.59353.peg.4347	fig|6666666.59355.peg.5201	None

10166	Glucokinase EC 2.7.1.2	None	None	None	None	None	None	None	None	None	None	None	None	None	fig|6666666.71181.peg.1545	fig|6666666.59344.peg.2051	None	None	None	None	None	fig|6666666.59349.peg.4199	fig|6666666.59350.peg.3128	fig|6666666.59351.peg.896	fig|6666666.59353.peg.2882	fig|6666666.59355.peg.2856	fig|6666666.59354.peg.2537

11376	FIG01207182 hypothetical protein	fig|6666666.59329.peg.2129	fig|6666666.59330.peg.1790	None	None	fig|6666666.59334.peg.1319	None	fig|6666666.59336.peg.3148	None	None	None	None	fig|6666666.59337.peg.2277	None	None	None	fig|6666666.59345.peg.2030	fig|6666666.59341.peg.2368	fig|6666666.59347.peg.3616	None	None	None	None	None	None	None	None

5448	Pyruvate 2-oxoglutarate dehydrogenase complex, dihydrolipoamide dehydrogenase component	fig|6666666.59329.peg.897	fig|6666666.59330.peg.3987	None	None	None	None	fig|6666666.59336.peg.5166	fig|6666666.59333.peg.4162	None	fig|6666666.59339.peg.2418	None	fig|6666666.59337.peg.1637	fig|6666666.59342.peg.4589	None	None	fig|6666666.59345.peg.5482	None	None	None	None	None	None	None	None	None	None

8639	Glutamate racemase EC 5.1.1.3	fig|6666666.59329.peg.2695	None	None	None	fig|6666666.59334.peg.3520	None	fig|6666666.59336.peg.3583	None	None	None	fig|6666666.59340.peg.1679	fig|6666666.59337.peg.1158	None	None	None	fig|6666666.59345.peg.3466	fig|6666666.59341.peg.4265	None	None	None	None	fig|6666666.59350.peg.3980	None	None	None	None

1422	hypothetical protein	fig|6666666.59329.peg.1558	None	None	fig|6666666.59332.peg.4768	fig|6666666.59334.peg.3311	None	fig|6666666.59336.peg.417	None	fig|6666666.59338.peg.4747	None	None	None	None	None	None	fig|6666666.59345.peg.1024	None	fig|6666666.59347.peg.1112	None	fig|6666666.59348.peg.5495	None	None	None	None	None	None

2439	Catabolite control protein A	None	None	None	None	fig|6666666.59334.peg.1497	None	None	None	fig|6666666.59338.peg.1613	None	None	None	None	fig|6666666.71181.peg.2157	fig|6666666.59344.peg.3229	None	fig|6666666.59341.peg.5649	fig|6666666.59347.peg.1453	None	None	None	None	None	fig|6666666.59353.peg.2845	fig|6666666.59355.peg.3948	None

1001	TsgA protein homolog	None	None	None	None	None	None	None	None	None	None	None	fig|6666666.59337.peg.2007	fig|6666666.59342.peg.641	fig|6666666.71181.peg.1820	fig|6666666.59344.peg.3078	fig|6666666.59345.peg.3064	None	None	None	None	None	None	fig|6666666.59351.peg.4411	fig|6666666.59353.peg.2230	fig|6666666.59355.peg.1924	None

8014	Spermidine synthase EC 2.5.1.16	None	None	None	None	None	None	None	None	None	None	None	None	None	fig|6666666.71181.peg.2854	fig|6666666.59344.peg.4423	None	None	None	None	None	fig|6666666.59349.peg.2207	fig|6666666.59350.peg.1111	fig|6666666.59351.peg.4185	fig|6666666.59353.peg.3206	fig|6666666.59355.peg.3088	fig|6666666.59354.peg.3028

2665	Alginate lyase precursor EC 4.2.2.3	None	None	fig|6666666.59331.peg.5818	None	fig|6666666.59334.peg.5015	None	fig|6666666.59336.peg.2955	None	fig|6666666.59338.peg.4576	None	fig|6666666.59340.peg.3225	None	None	None	None	fig|6666666.59345.peg.1387	fig|6666666.59341.peg.2340	fig|6666666.59347.peg.1079	None	None	None	None	None	None	None	None

3272	Alcohol dehydrogenase EC 1.1.1.1 ; Acetaldehyde dehydrogenase EC 1.2.1.10	None	fig|6666666.59330.peg.3389	None	None	None	None	fig|6666666.59336.peg.6164	fig|6666666.59333.peg.3169	None	fig|6666666.59339.peg.237	fig|6666666.59340.peg.1036	None	fig|6666666.59342.peg.2377	None	None	None	fig|6666666.59341.peg.2717	None	None	fig|6666666.59348.peg.2720	None	None	None	None	None	None

5489	hypothetical protein	fig|6666666.59329.peg.5415	None	fig|6666666.59331.peg.4652	None	fig|6666666.59334.peg.1786	None	None	None	fig|6666666.59338.peg.5784	None	fig|6666666.59340.peg.3336	None	None	None	None	None	fig|6666666.59341.peg.4498	fig|6666666.59347.peg.1849	None	fig|6666666.59348.peg.2303	None	None	None	None	None	None

69	Chaperone protein DnaK	fig|6666666.59329.peg.1661	None	None	None	None	fig|6666666.59335.peg.5648	None	fig|6666666.59333.peg.3459	None	fig|6666666.59339.peg.7269	fig|6666666.59340.peg.2026	None	None	None	fig|6666666.59344.peg.1326	None	fig|6666666.59341.peg.4072	None	fig|6666666.59346.peg.4648	None	None	None	None	None	None	None

503	Rare lipoprotein A precursor	None	None	fig|6666666.59331.peg.3475	fig|6666666.59332.peg.5280	fig|6666666.59334.peg.2926	None	None	None	fig|6666666.59338.peg.1545	None	fig|6666666.59340.peg.2468	None	None	None	None	None	fig|6666666.59341.peg.2686	fig|6666666.59347.peg.4684	fig|6666666.59346.peg.1025	None	None	None	None	None	None	None

5900	Phosphate acetyltransferase EC 2.3.1.8	fig|6666666.59329.peg.842	None	None	None	None	None	fig|6666666.59336.peg.2938	None	fig|6666666.59338.peg.6211	fig|6666666.59339.peg.3419	None	None	None	None	None	None	None	None	None	fig|6666666.59348.peg.4713	None	fig|6666666.59350.peg.1493	fig|6666666.59351.peg.2820	None	None	fig|6666666.59354.peg.4254

5453	3-hydroxydecanoyl-[ACP] dehydratase EC 4.2.1.60	None	None	None	None	None	None	None	fig|6666666.59333.peg.1105	None	None	None	None	None	fig|6666666.71181.peg.3927	fig|6666666.59344.peg.5394	None	fig|6666666.59341.peg.4967	None	fig|6666666.59346.peg.2112	fig|6666666.59348.peg.2994	None	None	None	fig|6666666.59353.peg.4784	fig|6666666.59355.peg.4837	None

5737	Transcriptional regulator, LysR family	None	None	fig|6666666.59331.peg.1815	fig|6666666.59332.peg.4211	None	None	fig|6666666.59336.peg.2948	fig|6666666.59333.peg.4366	None	None	None	None	None	None	None	fig|6666666.59345.peg.4037	None	fig|6666666.59347.peg.3698	fig|6666666.59346.peg.3942	fig|6666666.59348.peg.616	None	None	None	None	None	None

8093	tRNA-guanine transglycosylase EC 2.4.2.29	None	fig|6666666.59330.peg.1404	fig|6666666.59331.peg.2296	None	None	None	None	None	None	fig|6666666.59339.peg.6894	fig|6666666.59340.peg.554	None	fig|6666666.59342.peg.1146	None	None	None	None	None	None	fig|6666666.59348.peg.1562	fig|6666666.59349.peg.1924	None	None	None	None	fig|6666666.59354.peg.1289

2386	Rhodanese domain protein	None	None	None	None	None	None	fig|6666666.59336.peg.1831	None	fig|6666666.59338.peg.5424	fig|6666666.59339.peg.618	None	None	None	None	None	fig|6666666.59345.peg.2102	None	fig|6666666.59347.peg.241	None	None	fig|6666666.59349.peg.3970	fig|6666666.59350.peg.1265	fig|6666666.59351.peg.4195	None	None	None

6898	CcdB	fig|6666666.59329.peg.2814	fig|6666666.59330.peg.5006	None	None	None	None	None	fig|6666666.59333.peg.5596	None	None	None	None	None	fig|6666666.71181.peg.4212	fig|6666666.59344.peg.6058	None	None	None	None	None	None	fig|6666666.59350.peg.2838	None	fig|6666666.59353.peg.2737	fig|6666666.59355.peg.1745	None

8426	Cellulose synthase operon protein C	None	fig|6666666.59330.peg.2191	None	None	None	None	fig|6666666.59336.peg.1514	None	None	None	None	fig|6666666.59337.peg.5062	None	fig|6666666.71181.peg.4266	fig|6666666.59344.peg.2445	fig|6666666.59345.peg.4367	None	None	None	None	None	None	None	fig|6666666.59353.peg.3662	fig|6666666.59355.peg.4034	None

5468	Msl2237 protein	fig|6666666.59329.peg.3924	fig|6666666.59330.peg.765	None	None	None	None	fig|6666666.59336.peg.2629	fig|6666666.59333.peg.3029	None	None	None	fig|6666666.59337.peg.5355	fig|6666666.59342.peg.1413	None	None	fig|6666666.59345.peg.42	None	None	None	None	fig|6666666.59349.peg.2545	None	None	None	None	None

10276	Capsular polysaccharide synthesis enzyme CpsB	fig|6666666.59329.peg.549	fig|6666666.59330.peg.3965	None	None	None	fig|6666666.59335.peg.2300	None	fig|6666666.59333.peg.994	None	fig|6666666.59339.peg.4162	None	fig|6666666.59337.peg.3012	None	None	None	fig|6666666.59345.peg.626	fig|6666666.59341.peg.740	None	None	None	None	None	None	None	None	None

79	Chaperone protein DnaK	fig|6666666.59329.peg.4675	fig|6666666.59330.peg.1940	None	fig|6666666.59332.peg.5992	None	None	fig|6666666.59336.peg.5415	fig|6666666.59333.peg.2525	None	None	None	None	fig|6666666.59342.peg.3511	None	None	fig|6666666.59345.peg.723	None	None	fig|6666666.59346.peg.3132	None	None	None	None	None	None	None

10483	Adenylate cyclase EC 4.6.1.1	fig|6666666.59329.peg.6492	None	fig|6666666.59331.peg.4506	None	None	None	fig|6666666.59336.peg.4591	fig|6666666.59333.peg.5203	None	fig|6666666.59339.peg.6029	None	fig|6666666.59337.peg.5558	fig|6666666.59342.peg.3588	None	None	fig|6666666.59345.peg.3113	None	None	None	None	None	None	None	None	None	None

1521	Ribosomal-protein-S18p-alanine acetyltransferase EC 2.3.1.-	fig|6666666.59329.peg.3610	None	fig|6666666.59331.peg.5200	fig|6666666.59332.peg.3126	None	None	fig|6666666.59336.peg.5419	None	fig|6666666.59338.peg.5432	None	None	fig|6666666.59337.peg.5878	None	None	None	fig|6666666.59345.peg.979	None	fig|6666666.59347.peg.1176	None	None	None	None	None	None	None	None

9217	Type-1 fimbrial protein, A chain precursor	None	None	None	None	None	None	fig|6666666.59336.peg.3144	None	None	None	None	fig|6666666.59337.peg.2281	fig|6666666.59342.peg.823	fig|6666666.71181.peg.1076	fig|6666666.59344.peg.6000	fig|6666666.59345.peg.2018	None	None	None	None	None	None	None	fig|6666666.59353.peg.4974	fig|6666666.59355.peg.5056	None

4914	Alkanal monooxygenase alpha chain EC 1.14.14.3	None	fig|6666666.59330.peg.1135	None	None	None	None	None	fig|6666666.59333.peg.4493	None	None	None	fig|6666666.59337.peg.3632	None	fig|6666666.71181.peg.3577	fig|6666666.59344.peg.3258	None	None	None	None	None	None	fig|6666666.59350.peg.5395	None	fig|6666666.59353.peg.4398	fig|6666666.59355.peg.3055	None

7258	Aerolysin 4 precursor	None	fig|6666666.59330.peg.3743	None	None	None	fig|6666666.59335.peg.31	fig|6666666.59336.peg.5171	None	None	fig|6666666.59339.peg.7557	None	fig|6666666.59337.peg.1846	fig|6666666.59342.peg.5412	None	None	fig|6666666.59345.peg.4208	None	None	None	fig|6666666.59348.peg.4630	None	None	None	None	None	None

1397	FIG01202522 hypothetical protein	None	None	None	None	None	None	None	fig|6666666.59333.peg.1494	None	None	None	fig|6666666.59337.peg.65	fig|6666666.59342.peg.5537	fig|6666666.71181.peg.3086	fig|6666666.59344.peg.4223	fig|6666666.59345.peg.3778	None	None	None	None	None	None	None	fig|6666666.59353.peg.4263	fig|6666666.59355.peg.4333	None

8567	Mobile element protein	None	fig|6666666.59330.peg.5172	None	fig|6666666.59332.peg.5433	None	fig|6666666.59335.peg.6232	None	None	None	None	None	None	None	fig|6666666.71181.peg.5851	None	None	None	None	None	None	fig|6666666.59349.peg.4975	fig|6666666.59350.peg.6119	None	None	fig|6666666.59355.peg.5869	fig|6666666.59354.peg.1934

6831	hypothetical protein	None	fig|6666666.59330.peg.3211	None	None	None	None	None	None	fig|6666666.59338.peg.4666	fig|6666666.59339.peg.8130	None	fig|6666666.59337.peg.5150	None	None	None	None	None	fig|6666666.59347.peg.3730	None	None	fig|6666666.59349.peg.354	None	fig|6666666.59351.peg.5120	None	None	fig|6666666.59354.peg.386

11175	[Citrate [pro-3S]-lyase] ligase EC 6.2.1.22	fig|6666666.59329.peg.1727	None	None	None	fig|6666666.59334.peg.3620	None	fig|6666666.59336.peg.2511	None	None	None	None	None	fig|6666666.59342.peg.2230	fig|6666666.71181.peg.1724	fig|6666666.59344.peg.6206	None	None	None	None	None	None	None	None	fig|6666666.59353.peg.3690	fig|6666666.59355.peg.3013	None

10881	CDP-diacylglycerol--serine O-phosphatidyltransferase EC 2.7.8.8	fig|6666666.59329.peg.2425	fig|6666666.59330.peg.4560	None	None	fig|6666666.59334.peg.2117	None	None	None	None	None	fig|6666666.59340.peg.888	None	fig|6666666.59342.peg.1703	None	fig|6666666.59344.peg.3923	None	None	None	None	fig|6666666.59348.peg.24	None	None	None	None	None	None

9756	FIG01203844 hypothetical protein	fig|6666666.59329.peg.26	fig|6666666.59330.peg.150	None	fig|6666666.59332.peg.2737	None	None	fig|6666666.59336.peg.264	fig|6666666.59333.peg.2301	None	None	None	fig|6666666.59337.peg.5193	None	None	None	fig|6666666.59345.peg.5102	None	None	None	None	None	None	None	None	None	None

1767	CRISPR-associated protein NE0113	None	None	fig|6666666.59331.peg.11	None	fig|6666666.59334.peg.1086	None	None	None	fig|6666666.59338.peg.183	None	fig|6666666.59340.peg.3159	None	fig|6666666.59342.peg.2522	None	None	fig|6666666.59345.peg.3176	None	fig|6666666.59347.peg.2006	None	None	None	None	None	None	None	None

11111	hypothetical protein	None	None	None	fig|6666666.59332.peg.2736	None	fig|6666666.59335.peg.2254	None	None	fig|6666666.59338.peg.3561	fig|6666666.59339.peg.840	None	None	None	None	None	None	fig|6666666.59341.peg.4510	fig|6666666.59347.peg.573	fig|6666666.59346.peg.4555	None	None	None	None	None	None	None

9522	2-oxo-hepta-3-ene-1,7-dioic acid hydratase EC 4.2.-.-	None	None	None	None	None	None	None	None	None	None	None	None	None	fig|6666666.71181.peg.4555	fig|6666666.59344.peg.1418	None	fig|6666666.59341.peg.5497	fig|6666666.59347.peg.1326	None	None	None	fig|6666666.59350.peg.7341	None	fig|6666666.59353.peg.8	fig|6666666.59355.peg.614	None

9966	hypothetical protein	fig|6666666.59329.peg.3438	fig|6666666.59330.peg.2748	None	None	None	None	fig|6666666.59336.peg.3267	fig|6666666.59333.peg.3229	None	None	None	fig|6666666.59337.peg.290	fig|6666666.59342.peg.4129	None	None	fig|6666666.59345.peg.2125	None	None	None	None	None	None	None	None	None	None

3538	hypothetical protein	fig|6666666.59329.peg.4865	None	fig|6666666.59331.peg.2819	fig|6666666.59332.peg.3579	None	None	fig|6666666.59336.peg.6307	None	None	None	None	fig|6666666.59337.peg.1453	None	None	None	fig|6666666.59345.peg.163	None	None	fig|6666666.59346.peg.1283	None	None	None	None	None	None	None

10763	FIG073159 hypothetical protein	None	None	None	None	None	None	fig|6666666.59336.peg.6126	None	None	None	None	None	fig|6666666.59342.peg.4383	fig|6666666.71181.peg.1299	fig|6666666.59344.peg.2954	None	None	fig|6666666.59347.peg.1842	None	None	None	None	None	fig|6666666.59353.peg.3299	fig|6666666.59355.peg.3153	None

10768	hypothetical protein	None	None	fig|6666666.59331.peg.2916	fig|6666666.59332.peg.5247	None	None	None	None	fig|6666666.59338.peg.1583	None	None	None	fig|6666666.59342.peg.5840	None	None	None	fig|6666666.59341.peg.1290	fig|6666666.59347.peg.716	fig|6666666.59346.peg.2637	None	None	None	None	None	None	None

10432	FIG01202089 hypothetical protein	fig|6666666.59329.peg.2240	None	None	fig|6666666.59332.peg.4397	fig|6666666.59334.peg.1920	None	fig|6666666.59336.peg.5448	None	None	fig|6666666.59339.peg.3543	None	None	None	None	fig|6666666.59344.peg.5245	None	None	None	None	fig|6666666.59348.peg.1309	None	None	None	None	None	None

5868	Transcriptional regulator, LysR family	None	None	None	fig|6666666.59332.peg.4490	None	fig|6666666.59335.peg.3873	fig|6666666.59336.peg.2048	None	fig|6666666.59338.peg.945	None	None	None	None	None	None	fig|6666666.59345.peg.1665	None	fig|6666666.59347.peg.625	None	fig|6666666.59348.peg.3384	None	None	None	None	None	None

6237	hypothetical protein	None	fig|6666666.59330.peg.2957	None	None	None	None	None	None	fig|6666666.59338.peg.2725	fig|6666666.59339.peg.6077	None	fig|6666666.59337.peg.3448	None	None	None	fig|6666666.59345.peg.189	None	fig|6666666.59347.peg.1252	None	None	None	None	fig|6666666.59351.peg.4702	None	None	None

9776	Transcriptional regulator	None	fig|6666666.59330.peg.4839	fig|6666666.59331.peg.4261	fig|6666666.59332.peg.1233	None	None	fig|6666666.59336.peg.1935	fig|6666666.59333.peg.2010	None	None	None	fig|6666666.59337.peg.2401	None	None	None	fig|6666666.59345.peg.961	None	None	None	None	None	None	None	None	None	None

4192	FIG003269 Prophage tail protein	None	None	None	None	None	fig|6666666.59335.peg.304	None	None	None	fig|6666666.59339.peg.2538	None	None	None	fig|6666666.71181.peg.2891	fig|6666666.59344.peg.4374	None	None	None	None	fig|6666666.59348.peg.5312	None	None	None	fig|6666666.59353.peg.4287	fig|6666666.59355.peg.4416	None

239	Putative membrane protein	None	None	None	None	None	None	None	None	None	None	None	None	None	fig|6666666.71181.peg.1170	fig|6666666.59344.peg.2298	None	None	None	None	None	fig|6666666.59349.peg.2079	None	fig|6666666.59351.peg.3576	fig|6666666.59353.peg.3413	fig|6666666.59355.peg.3511	fig|6666666.59354.peg.3556

5076	Mlr1851 protein	fig|6666666.59329.peg.3852	None	fig|6666666.59331.peg.1243	fig|6666666.59332.peg.1377	None	fig|6666666.59335.peg.3264	None	None	None	fig|6666666.59339.peg.6829	None	fig|6666666.59337.peg.1340	None	None	None	None	None	None	fig|6666666.59346.peg.3079	None	None	None	None	None	None	None

930	FIG01200052 hypothetical protein	fig|6666666.59329.peg.2363	fig|6666666.59330.peg.1491	None	None	None	None	fig|6666666.59336.peg.1169	fig|6666666.59333.peg.1733	None	None	None	fig|6666666.59337.peg.185	fig|6666666.59342.peg.4445	None	None	fig|6666666.59345.peg.4515	None	None	None	None	None	None	None	None	None	None

9388	PTS system, mannose-specific IIA component EC 2.7.1.69 PTS system, mannose-specific IIB component EC 2.7.1.69	fig|6666666.59329.peg.618	None	None	None	None	None	None	None	fig|6666666.59338.peg.4056	None	None	None	None	fig|6666666.71181.peg.4237	fig|6666666.59344.peg.2568	None	fig|6666666.59341.peg.3085	None	None	None	None	None	None	fig|6666666.59353.peg.1531	fig|6666666.59355.peg.2080	None

2888	FIG01207299 hypothetical protein	None	fig|6666666.59330.peg.2985	None	None	None	fig|6666666.59335.peg.3250	fig|6666666.59336.peg.6515	None	None	fig|6666666.59339.peg.1437	None	fig|6666666.59337.peg.4147	fig|6666666.59342.peg.1069	None	None	fig|6666666.59345.peg.4616	None	None	None	None	None	None	None	None	None	None

3287	Dihydroneopterin triphosphate pyrophosphohydrolase type 2 nudB	None	None	None	None	None	None	None	None	None	None	None	None	None	fig|6666666.71181.peg.1919	fig|6666666.59344.peg.2519	None	None	None	None	None	fig|6666666.59349.peg.1984	None	fig|6666666.59351.peg.1753	fig|6666666.59353.peg.1490	fig|6666666.59355.peg.2040	fig|6666666.59354.peg.2777

2550	Protein kinase-like protein	None	None	None	fig|6666666.59332.peg.4372	fig|6666666.59334.peg.2966	fig|6666666.59335.peg.980	None	fig|6666666.59333.peg.819	None	fig|6666666.59339.peg.4469	None	None	None	None	None	fig|6666666.59345.peg.1737	None	None	None	fig|6666666.59348.peg.3173	None	None	None	None	None	None

913	Regulator of nucleoside diphosphate kinase	fig|6666666.59329.peg.3877	None	None	None	None	None	None	fig|6666666.59333.peg.3889	None	None	None	None	None	fig|6666666.71181.peg.999	fig|6666666.59344.peg.1775	fig|6666666.59345.peg.4543	None	None	None	None	None	None	None	fig|6666666.59353.peg.2316	fig|6666666.59355.peg.1590	None

10645	Glutamate receptor 1 precursor	fig|6666666.59329.peg.1307	fig|6666666.59330.peg.5079	None	None	None	None	fig|6666666.59336.peg.3910	None	None	None	None	fig|6666666.59337.peg.2845	fig|6666666.59342.peg.3487	None	None	fig|6666666.59345.peg.1751	None	None	None	None	None	fig|6666666.59350.peg.1601	None	None	None	None

11440	ABC transporter ATP-binding protein	fig|6666666.59329.peg.1396	fig|6666666.59330.peg.1472	fig|6666666.59331.peg.4410	None	None	None	fig|6666666.59336.peg.298	None	None	None	None	fig|6666666.59337.peg.204	fig|6666666.59342.peg.5865	None	None	None	fig|6666666.59341.peg.5179	None	None	None	None	None	None	None	None	None

634	Alpha-amylase EC 3.2.1.1	None	None	fig|6666666.59331.peg.6116	None	fig|6666666.59334.peg.2578	fig|6666666.59335.peg.4914	None	None	None	fig|6666666.59339.peg.4071	None	None	None	None	None	None	fig|6666666.59341.peg.5549	None	None	fig|6666666.59348.peg.4870	None	None	None	None	None	fig|6666666.59354.peg.2547

4724	Lipoprotein	fig|6666666.59329.peg.1299	fig|6666666.59330.peg.4846	None	None	None	None	fig|6666666.59336.peg.3363	fig|6666666.59333.peg.812	None	None	None	fig|6666666.59337.peg.2850	fig|6666666.59342.peg.3187	None	None	fig|6666666.59345.peg.1744	None	None	None	None	None	None	None	None	None	None

4727	D-amino-acid oxidase EC 1.4.3.3	None	None	fig|6666666.59331.peg.2692	None	None	None	None	None	None	fig|6666666.59339.peg.2046	None	None	None	fig|6666666.71181.peg.342	fig|6666666.59344.peg.2342	None	None	None	None	None	None	fig|6666666.59350.peg.6146	None	fig|6666666.59353.peg.4201	fig|6666666.59355.peg.4165	None

11261	FIG01199570 hypothetical protein	None	None	None	fig|6666666.59332.peg.2363	None	None	fig|6666666.59336.peg.2639	None	None	None	None	fig|6666666.59337.peg.3237	fig|6666666.59342.peg.3094	None	None	fig|6666666.59345.peg.1804	fig|6666666.59341.peg.4920	None	fig|6666666.59346.peg.2793	None	None	None	None	None	None	None

8333	UDP-N-acetylglucosamine 1-carboxyvinyltransferase EC 2.5.1.7	None	fig|6666666.59330.peg.57	None	None	None	None	fig|6666666.59336.peg.360	None	None	None	fig|6666666.59340.peg.838	fig|6666666.59337.peg.844	fig|6666666.59342.peg.438	None	None	None	fig|6666666.59341.peg.2692	None	fig|6666666.59346.peg.627	None	None	None	None	None	None	None

4043	Putative membrane protein	None	None	None	None	None	fig|6666666.59335.peg.4884	None	None	None	fig|6666666.59339.peg.714	None	None	None	fig|6666666.71181.peg.500	fig|6666666.59344.peg.1716	None	None	None	None	None	None	fig|6666666.59350.peg.6356	None	fig|6666666.59353.peg.1128	fig|6666666.59355.peg.348	None

3664	FIG01200910 hypothetical protein	fig|6666666.59329.peg.4927	None	None	None	None	fig|6666666.59335.peg.5285	fig|6666666.59336.peg.3852	None	None	fig|6666666.59339.peg.2609	None	fig|6666666.59337.peg.3276	fig|6666666.59342.peg.5511	None	None	fig|6666666.59345.peg.540	None	None	None	None	None	None	None	None	None	None

5035	FIG01064529 hypothetical protein	None	fig|6666666.59330.peg.2703	fig|6666666.59331.peg.2905	None	None	None	None	None	None	None	None	None	None	None	None	None	None	fig|6666666.59347.peg.2067	fig|6666666.59346.peg.2406	None	fig|6666666.59349.peg.2694	None	fig|6666666.59351.peg.5170	None	None	fig|6666666.59354.peg.4456

10477	hypothetical protein	None	None	None	fig|6666666.59332.peg.5248	fig|6666666.59334.peg.2656	None	None	None	fig|6666666.59338.peg.1584	None	None	None	fig|6666666.59342.peg.5839	None	None	None	fig|6666666.59341.peg.1291	fig|6666666.59347.peg.717	fig|6666666.59346.peg.2636	None	None	None	None	None	None	None

6298	FIG01201229 hypothetical protein	None	None	fig|6666666.59331.peg.1017	None	None	fig|6666666.59335.peg.2806	None	None	fig|6666666.59338.peg.1217	fig|6666666.59339.peg.1966	fig|6666666.59340.peg.1310	None	None	None	None	None	None	None	fig|6666666.59346.peg.2946	fig|6666666.59348.peg.4358	None	None	None	None	None	None

3431	2,3-diketo-5-methylthiopentyl-1-phosphate enolase-phosphatase EC 3.1.3.77	None	None	None	None	None	None	None	None	None	None	None	None	None	fig|6666666.71181.peg.907	fig|6666666.59344.peg.5184	None	None	None	None	None	fig|6666666.59349.peg.2218	None	fig|6666666.59351.peg.5008	fig|6666666.59353.peg.911	fig|6666666.59355.peg.796	fig|6666666.59354.peg.3602

2743	PTS system, mannose-specific IID component EC 2.7.1.69	fig|6666666.59329.peg.620	None	None	None	None	None	None	None	fig|6666666.59338.peg.4058	None	None	None	None	fig|6666666.71181.peg.4239	fig|6666666.59344.peg.2571	None	fig|6666666.59341.peg.3082	None	None	None	None	None	None	fig|6666666.59353.peg.1533	fig|6666666.59355.peg.2082	None

6331	TesB-like acyl-CoA thioesterase 1	None	None	None	None	None	None	None	None	fig|6666666.59338.peg.296	None	fig|6666666.59340.peg.1395	None	None	None	None	None	fig|6666666.59341.peg.1376	fig|6666666.59347.peg.31	None	None	fig|6666666.59349.peg.4424	None	fig|6666666.59351.peg.3740	None	None	fig|6666666.59354.peg.2307

752	Nitroreductase family protein	None	None	fig|6666666.59331.peg.6226	None	fig|6666666.59334.peg.2397	fig|6666666.59335.peg.437	fig|6666666.59336.peg.257	None	None	fig|6666666.59339.peg.6393	None	None	None	None	None	None	None	None	None	None	None	fig|6666666.59350.peg.3539	None	None	None	fig|6666666.59354.peg.3368

2804	Periplasmic protein YqjC	None	fig|6666666.59330.peg.3304	None	None	None	None	None	fig|6666666.59333.peg.2901	None	None	None	None	None	fig|6666666.71181.peg.4397	fig|6666666.59344.peg.1856	None	fig|6666666.59341.peg.629	None	None	None	None	None	None	fig|6666666.59353.peg.2385	fig|6666666.59355.peg.1657	None

5433	Capsular polysaccharide synthesis enzyme CpsE	fig|6666666.59329.peg.545	fig|6666666.59330.peg.3962	None	None	None	None	fig|6666666.59336.peg.5032	fig|6666666.59333.peg.990	None	None	None	fig|6666666.59337.peg.3008	fig|6666666.59342.peg.4581	None	None	fig|6666666.59345.peg.629	None	None	None	None	None	None	None	None	None	None

5435	FIG01204004 hypothetical protein	fig|6666666.59329.peg.3275	fig|6666666.59330.peg.4296	None	None	None	None	fig|6666666.59336.peg.2520	None	None	fig|6666666.59339.peg.406	None	None	fig|6666666.59342.peg.6319	None	None	fig|6666666.59345.peg.1696	fig|6666666.59341.peg.1794	None	None	None	None	None	None	None	None	None

5129	P pilus assembly protein, porin PapC	fig|6666666.59329.peg.1572	None	None	None	fig|6666666.59334.peg.1263	None	fig|6666666.59336.peg.689	None	fig|6666666.59338.peg.4331	None	None	fig|6666666.59337.peg.1316	None	None	None	fig|6666666.59345.peg.2324	None	None	fig|6666666.59346.peg.1791	None	None	None	None	None	None	None

9792	Chaperone protein HscA	None	None	None	fig|6666666.59332.peg.1333	fig|6666666.59334.peg.3069	None	None	fig|6666666.59333.peg.929	None	None	fig|6666666.59340.peg.3042	None	None	None	None	None	None	None	None	None	None	None	None	fig|6666666.59353.peg.2699	fig|6666666.59355.peg.1780	fig|6666666.59354.peg.5181

3220	Transcriptional regulator, LysR family	None	None	None	None	None	None	fig|6666666.59336.peg.5119	None	None	None	None	None	fig|6666666.59342.peg.4380	fig|6666666.71181.peg.1298	fig|6666666.59344.peg.2955	None	None	fig|6666666.59347.peg.1839	None	None	None	None	None	fig|6666666.59353.peg.3300	fig|6666666.59355.peg.3152	None

6493	O-antigen export system permease protein RfbD	None	None	None	None	None	fig|6666666.59335.peg.506	None	None	None	None	None	None	None	fig|6666666.71181.peg.236	fig|6666666.59344.peg.291	None	fig|6666666.59341.peg.2742	None	None	None	None	fig|6666666.59350.peg.7682	None	fig|6666666.59353.peg.585	fig|6666666.59355.peg.68	None

5487	hypothetical protein	None	None	None	None	fig|6666666.59334.peg.1454	None	None	None	fig|6666666.59338.peg.3500	None	None	None	None	fig|6666666.71181.peg.100	fig|6666666.59344.peg.3160	None	None	fig|6666666.59347.peg.3812	None	None	None	None	None	fig|6666666.59353.peg.1912	fig|6666666.59355.peg.1496	None

9639	HTH-type transcriptional regulator PtxR	None	None	None	None	None	fig|6666666.59335.peg.5294	None	None	None	fig|6666666.59339.peg.8501	None	None	None	fig|6666666.71181.peg.1469	fig|6666666.59344.peg.5737	fig|6666666.59345.peg.4180	None	None	None	None	None	None	None	fig|6666666.59353.peg.4385	fig|6666666.59355.peg.4456	None

3348	FIG01201458 hypothetical protein	fig|6666666.59329.peg.3560	fig|6666666.59330.peg.2476	None	None	None	None	fig|6666666.59336.peg.2787	fig|6666666.59333.peg.1055	None	None	None	fig|6666666.59337.peg.4539	fig|6666666.59342.peg.2859	None	None	fig|6666666.59345.peg.1526	None	None	None	None	None	None	None	None	None	None

2051	Glucose ABC transporter, ATP-binding subunit EC 3.6.3.-	None	None	None	fig|6666666.59332.peg.5743	None	None	None	None	fig|6666666.59338.peg.4290	None	fig|6666666.59340.peg.3569	None	None	fig|6666666.71181.peg.6345	None	None	fig|6666666.59341.peg.5282	None	None	fig|6666666.59348.peg.3397	None	fig|6666666.59350.peg.6304	None	None	None	None

6683	ABC transporter ATP-binding protein	None	None	None	fig|6666666.59332.peg.619	None	None	None	None	fig|6666666.59338.peg.669	None	None	None	None	fig|6666666.71181.peg.99	fig|6666666.59344.peg.3162	None	None	fig|6666666.59347.peg.2511	None	None	None	None	None	fig|6666666.59353.peg.1913	fig|6666666.59355.peg.1495	None

6357	Bll2871 protein	fig|6666666.59329.peg.6090	fig|6666666.59330.peg.4738	fig|6666666.59331.peg.4492	None	None	None	None	None	None	None	None	fig|6666666.59337.peg.2829	fig|6666666.59342.peg.1240	None	None	fig|6666666.59345.peg.1397	fig|6666666.59341.peg.4695	None	None	None	None	None	None	None	None	None

531	5-methylthioribose kinase EC 2.7.1.100	None	None	None	None	None	fig|6666666.59335.peg.5369	None	None	None	None	None	None	None	fig|6666666.71181.peg.910	fig|6666666.59344.peg.5180	None	None	None	None	None	fig|6666666.59349.peg.3408	None	fig|6666666.59351.peg.5005	fig|6666666.59353.peg.907	fig|6666666.59355.peg.799	None

6163	Deoxycytidine triphosphate deaminase EC 3.5.4.13	None	None	None	None	None	None	None	None	None	None	None	None	None	fig|6666666.71181.peg.208	fig|6666666.59344.peg.256	None	None	None	None	None	fig|6666666.59349.peg.3743	None	fig|6666666.59351.peg.2808	fig|6666666.59353.peg.613	fig|6666666.59355.peg.95	fig|6666666.59354.peg.3054

2213	Possible monoamine oxidase	None	fig|6666666.59330.peg.1467	None	None	fig|6666666.59334.peg.2644	fig|6666666.59335.peg.624	None	None	fig|6666666.59338.peg.2756	None	None	fig|6666666.59337.peg.208	None	None	None	None	None	fig|6666666.59347.peg.3410	None	None	None	fig|6666666.59350.peg.1885	None	None	None	None

9025	AttF component of AttEFGH ABC transport system AttG component of AttEFGH ABC transport system	fig|6666666.59329.peg.607	fig|6666666.59330.peg.4333	fig|6666666.59331.peg.5166	None	fig|6666666.59334.peg.5329	None	fig|6666666.59336.peg.647	fig|6666666.59333.peg.2655	None	None	None	None	None	None	None	None	None	None	None	fig|6666666.59348.peg.751	None	None	None	None	None	None

9471	Phenylalanyl-tRNA synthetase alpha chain EC 6.1.1.20	fig|6666666.59329.peg.1471	None	None	None	None	None	None	fig|6666666.59333.peg.532	None	fig|6666666.59339.peg.594	None	None	None	None	None	fig|6666666.59345.peg.4650	fig|6666666.59341.peg.451	fig|6666666.59347.peg.1879	None	fig|6666666.59348.peg.3242	None	None	None	None	None	None

7041	Apo-citrate lyase phosphoribosyl-dephospho-CoA transferase EC 2.7.7.61	fig|6666666.59329.peg.1723	None	None	None	fig|6666666.59334.peg.1770	None	None	None	None	None	None	None	fig|6666666.59342.peg.3710	fig|6666666.71181.peg.1728	fig|6666666.59344.peg.6290	None	None	None	None	None	None	None	None	fig|6666666.59353.peg.3685	fig|6666666.59355.peg.3009	None

7049	Hypothetical flavoprotein YqcA clustered with tRNA pseudouridine synthase C	None	None	None	None	None	None	None	None	None	None	None	None	None	fig|6666666.71181.peg.40	fig|6666666.59344.peg.2646	None	None	None	None	None	fig|6666666.59349.peg.1354	None	fig|6666666.59351.peg.5489	fig|6666666.59353.peg.1978	fig|6666666.59355.peg.4085	fig|6666666.59354.peg.2623

11004	FIG01201474 hypothetical protein	fig|6666666.59329.peg.5779	None	None	None	None	None	None	None	None	None	fig|6666666.59340.peg.2493	None	None	None	None	None	fig|6666666.59341.peg.1868	fig|6666666.59347.peg.4895	fig|6666666.59346.peg.4983	None	fig|6666666.59349.peg.78	None	None	None	None	fig|6666666.59354.peg.3284

432	ABC transporter substrate-binding protein	None	None	fig|6666666.59331.peg.1706	None	None	None	None	fig|6666666.59333.peg.3611	None	fig|6666666.59339.peg.3124	fig|6666666.59340.peg.1846	fig|6666666.59337.peg.4932	fig|6666666.59342.peg.805	None	None	None	None	None	fig|6666666.59346.peg.549	None	None	None	None	None	None	None

1484	FIG01206948 hypothetical protein	None	fig|6666666.59330.peg.3902	None	None	None	fig|6666666.59335.peg.992	None	None	None	fig|6666666.59339.peg.4455	None	fig|6666666.59337.peg.5552	fig|6666666.59342.peg.5113	None	None	fig|6666666.59345.peg.3107	None	None	None	None	None	None	fig|6666666.59351.peg.2795	None	None	None

516	hypothetical protein	None	None	None	None	None	fig|6666666.59335.peg.3832	None	None	None	fig|6666666.59339.peg.1788	None	None	None	fig|6666666.71181.peg.5601	None	None	None	fig|6666666.59347.peg.876	None	fig|6666666.59348.peg.5059	None	fig|6666666.59350.peg.4786	fig|6666666.59351.peg.1435	None	None	None

6539	Xaa-Pro aminopeptidase EC 3.4.11.9	fig|6666666.59329.peg.5608	None	None	None	None	fig|6666666.59335.peg.6049	fig|6666666.59336.peg.180	None	None	fig|6666666.59339.peg.7535	None	None	fig|6666666.59342.peg.2676	None	None	None	None	None	None	fig|6666666.59348.peg.874	None	fig|6666666.59350.peg.1054	None	None	None	None

11300	Inner membrane protein YqiK	None	fig|6666666.59330.peg.3212	None	None	None	None	None	None	None	fig|6666666.59339.peg.8129	None	fig|6666666.59337.peg.5151	fig|6666666.59342.peg.1769	None	None	None	None	fig|6666666.59347.peg.3729	None	None	fig|6666666.59349.peg.355	None	fig|6666666.59351.peg.5119	None	None	None

1031	hypothetical protein	None	None	None	None	None	None	fig|6666666.59336.peg.5840	fig|6666666.59333.peg.2043	None	fig|6666666.59339.peg.5717	None	None	None	fig|6666666.71181.peg.1332	fig|6666666.59344.peg.2918	None	None	None	None	None	None	None	None	fig|6666666.59353.peg.3268	fig|6666666.59355.peg.3184	None

5722	54K polar flagellar sheath protein A	fig|6666666.59329.peg.3578	fig|6666666.59330.peg.1298	None	fig|6666666.59332.peg.6000	None	None	None	fig|6666666.59333.peg.2673	None	None	None	fig|6666666.59337.peg.2212	fig|6666666.59342.peg.5762	None	None	None	None	None	fig|6666666.59346.peg.3834	None	None	None	None	None	None	None

1857	FIG01201992 hypothetical protein	fig|6666666.59329.peg.2307	fig|6666666.59330.peg.92	None	None	None	None	fig|6666666.59336.peg.3566	fig|6666666.59333.peg.2314	None	None	None	fig|6666666.59337.peg.1295	fig|6666666.59342.peg.4543	None	None	fig|6666666.59345.peg.2343	None	None	None	None	None	None	None	None	None	None

1421	Transcriptional repressor of aerobactin receptor iutR	None	None	fig|6666666.59331.peg.875	fig|6666666.59332.peg.1161	fig|6666666.59334.peg.3519	None	None	None	fig|6666666.59338.peg.1020	None	None	None	None	None	None	None	None	fig|6666666.59347.peg.255	fig|6666666.59346.peg.1159	None	None	fig|6666666.59350.peg.1006	None	None	None	None

1420	Deacetylase DA1	None	fig|6666666.59330.peg.2439	None	None	None	None	fig|6666666.59336.peg.495	fig|6666666.59333.peg.2982	fig|6666666.59338.peg.895	None	None	fig|6666666.59337.peg.4182	None	None	None	fig|6666666.59345.peg.4828	None	fig|6666666.59347.peg.5360	None	None	None	None	None	None	None	None

10772	Phage integrase	None	None	None	None	fig|6666666.59334.peg.470	None	fig|6666666.59336.peg.3495	None	None	None	fig|6666666.59340.peg.3593	fig|6666666.59337.peg.5267	fig|6666666.59342.peg.287	None	None	None	fig|6666666.59341.peg.756	None	None	None	None	None	fig|6666666.59351.peg.4496	None	None	None

3857	Ribulose-5-phosphate 4-epimerase and related epimerases and aldolases	None	None	None	None	None	None	None	None	None	None	None	None	None	fig|6666666.71181.peg.820	fig|6666666.59344.peg.57	None	None	None	None	None	None	fig|6666666.59350.peg.3749	fig|6666666.59351.peg.3109	fig|6666666.59353.peg.394	fig|6666666.59355.peg.2117	fig|6666666.59354.peg.5692

1702	FIG01200675 hypothetical protein	fig|6666666.59329.peg.6371	None	None	None	None	None	None	None	fig|6666666.59338.peg.2511	None	None	fig|6666666.59337.peg.3609	fig|6666666.59342.peg.5509	None	None	fig|6666666.59345.peg.1849	fig|6666666.59341.peg.557	fig|6666666.59347.peg.2115	None	None	None	None	None	None	None	None

1878	FIG01199761 hypothetical protein	None	fig|6666666.59330.peg.157	None	fig|6666666.59332.peg.744	None	None	fig|6666666.59336.peg.256	fig|6666666.59333.peg.431	None	None	None	fig|6666666.59337.peg.4537	None	None	None	fig|6666666.59345.peg.5109	None	None	fig|6666666.59346.peg.974	None	None	None	None	None	None	None

9889	FIG00560454 hypothetical protein	None	None	None	fig|6666666.59332.peg.3125	fig|6666666.59334.peg.3789	None	fig|6666666.59336.peg.3885	None	fig|6666666.59338.peg.4607	None	None	None	fig|6666666.59342.peg.4366	None	None	None	fig|6666666.59341.peg.2925	fig|6666666.59347.peg.711	None	None	None	None	None	None	None	None

1911	Methyl-accepting chemotaxis protein	fig|6666666.59329.peg.995	fig|6666666.59330.peg.2384	None	None	None	None	fig|6666666.59336.peg.798	fig|6666666.59333.peg.2629	None	None	None	fig|6666666.59337.peg.2875	fig|6666666.59342.peg.4007	None	None	fig|6666666.59345.peg.1711	None	None	None	None	None	None	None	None	None	None

5685	Maltose phosphorylase EC 2.4.1.8 Trehalose phosphorylase EC 2.4.1.64	None	None	fig|6666666.59331.peg.4358	None	fig|6666666.59334.peg.4697	fig|6666666.59335.peg.3736	None	None	None	None	fig|6666666.59340.peg.272	None	fig|6666666.59342.peg.608	None	None	fig|6666666.59345.peg.2004	fig|6666666.59341.peg.3835	None	None	None	None	None	None	None	None	None

5681	DNA helicase IV	fig|6666666.59329.peg.5924	None	fig|6666666.59331.peg.4179	fig|6666666.59332.peg.2864	None	None	fig|6666666.59336.peg.815	None	None	fig|6666666.59339.peg.2832	None	None	fig|6666666.59342.peg.662	None	None	None	None	None	None	fig|6666666.59348.peg.4487	None	None	None	None	None	None

2639	FIG01204755 hypothetical protein	None	None	fig|6666666.59331.peg.1829	fig|6666666.59332.peg.5726	fig|6666666.59334.peg.2105	None	None	fig|6666666.59333.peg.3942	None	None	None	None	None	None	None	fig|6666666.59345.peg.3983	fig|6666666.59341.peg.1845	None	fig|6666666.59346.peg.4372	None	None	None	None	None	None	None

10262	hypothetical protein	None	None	None	None	None	None	None	None	None	None	None	None	None	fig|6666666.71181.peg.3311	fig|6666666.59344.peg.4854	None	None	None	None	None	fig|6666666.59349.peg.153	None	fig|6666666.59351.peg.3675	fig|6666666.59353.peg.3474	fig|6666666.59355.peg.3248	fig|6666666.59354.peg.178

4332	Bacteriophage tail sheath protein	None	None	None	None	None	fig|6666666.59335.peg.286	None	None	None	fig|6666666.59339.peg.2566	None	None	None	fig|6666666.71181.peg.2884	fig|6666666.59344.peg.4381	None	None	None	None	fig|6666666.59348.peg.3701	None	None	None	fig|6666666.59353.peg.4293	fig|6666666.59355.peg.4410	None

5760	FIG01199977 hypothetical protein	fig|6666666.59329.peg.3620	fig|6666666.59330.peg.4230	None	None	None	None	fig|6666666.59336.peg.4313	fig|6666666.59333.peg.2936	None	None	None	fig|6666666.59337.peg.1249	None	None	None	fig|6666666.59345.peg.4431	None	None	None	fig|6666666.59348.peg.3500	None	None	None	None	None	None

1040	FIG00922618 hypothetical protein	None	None	None	None	fig|6666666.59334.peg.5445	None	fig|6666666.59336.peg.3833	fig|6666666.59333.peg.2709	None	None	fig|6666666.59340.peg.816	fig|6666666.59337.peg.2024	fig|6666666.59342.peg.6052	None	None	fig|6666666.59345.peg.2452	None	None	None	None	None	None	None	None	None	None

5168	Glutathione synthetase EC 6.3.2.3	None	None	fig|6666666.59331.peg.2494	fig|6666666.59332.peg.5590	None	None	fig|6666666.59336.peg.2050	None	fig|6666666.59338.peg.4763	None	None	None	None	None	None	fig|6666666.59345.peg.3894	None	fig|6666666.59347.peg.72	fig|6666666.59346.peg.1876	None	None	None	None	None	None	None

2782	Mannose-6-phosphate isomerase	fig|6666666.59329.peg.5816	None	None	None	None	None	None	None	fig|6666666.59338.peg.2899	None	None	None	None	fig|6666666.71181.peg.5393	None	None	None	None	None	None	fig|6666666.59349.peg.2536	fig|6666666.59350.peg.6728	fig|6666666.59351.peg.5228	None	None	fig|6666666.59354.peg.4583

9696	FIG01200221 hypothetical protein	fig|6666666.59329.peg.1735	fig|6666666.59330.peg.3825	fig|6666666.59331.peg.762	None	None	None	fig|6666666.59336.peg.5373	fig|6666666.59333.peg.3701	None	None	None	fig|6666666.59337.peg.2703	None	None	None	fig|6666666.59345.peg.3181	None	None	None	None	None	None	None	None	None	None

6030	Citrate lyase gamma chain, acyl carrier protein EC 4.1.3.6	fig|6666666.59329.peg.1726	None	None	None	fig|6666666.59334.peg.1765	None	fig|6666666.59336.peg.2510	None	None	None	None	None	fig|6666666.59342.peg.2232	fig|6666666.71181.peg.1725	None	None	None	None	None	None	None	None	None	fig|6666666.59353.peg.3689	fig|6666666.59355.peg.3012	None

4480	Transcriptional regulator	None	None	None	None	None	None	None	None	None	None	None	None	None	fig|6666666.71181.peg.2123	fig|6666666.59344.peg.5325	None	None	None	None	None	fig|6666666.59349.peg.857	None	fig|6666666.59351.peg.4100	fig|6666666.59353.peg.713	fig|6666666.59355.peg.200	fig|6666666.59354.peg.5129

705	2-keto-3-deoxy-D-arabino-heptulosonate-7-phosphate synthase I alpha EC 2.5.1.54	fig|6666666.59329.peg.5985	None	fig|6666666.59331.peg.5182	fig|6666666.59332.peg.5008	fig|6666666.59334.peg.3585	None	None	fig|6666666.59333.peg.4142	None	None	fig|6666666.59340.peg.2690	None	fig|6666666.59342.peg.2591	None	None	None	None	None	None	None	None	None	None	None	None	None

3710	hypothetical protein	fig|6666666.59329.peg.47	None	None	None	None	None	None	None	fig|6666666.59338.peg.569	fig|6666666.59339.peg.3290	fig|6666666.59340.peg.531	None	None	None	None	None	fig|6666666.59341.peg.1937	fig|6666666.59347.peg.4763	fig|6666666.59346.peg.1810	None	None	None	None	None	None	None

8810	5-methyltetrahydropteroyltriglutamate--homocysteine methyltransferase EC 2.1.1.14	None	None	None	fig|6666666.59332.peg.5712	fig|6666666.59334.peg.1990	None	fig|6666666.59336.peg.5950	fig|6666666.59333.peg.1912	None	None	None	fig|6666666.59337.peg.3512	None	None	None	fig|6666666.59345.peg.930	None	fig|6666666.59347.peg.3566	None	None	None	None	None	None	None	None

9087	Mobile element protein	fig|6666666.59329.peg.412	None	fig|6666666.59331.peg.3673	fig|6666666.59332.peg.6106	fig|6666666.59334.peg.4486	fig|6666666.59335.peg.5400	None	None	fig|6666666.59338.peg.4310	None	None	None	None	None	None	None	None	None	None	None	None	None	fig|6666666.59351.peg.3021	None	None	None

5082	ABC transporter, periplasmic spermidine putrescine-binding protein PotD TC 3.A.1.11.1	None	fig|6666666.59330.peg.2601	fig|6666666.59331.peg.936	fig|6666666.59332.peg.1323	None	fig|6666666.59335.peg.2206	None	None	None	None	None	None	None	None	None	None	None	None	fig|6666666.59346.peg.5301	fig|6666666.59348.peg.3373	None	fig|6666666.59350.peg.6432	None	None	None	None

1168	Putative sensor-like histidine kinase YfhK	None	None	None	None	None	None	None	None	None	None	None	None	None	fig|6666666.71181.peg.4874	fig|6666666.59344.peg.1594	None	None	None	None	None	fig|6666666.59349.peg.891	None	fig|6666666.59351.peg.4518	fig|6666666.59353.peg.2671	fig|6666666.59355.peg.1810	fig|6666666.59354.peg.2994

7886	FIG01201686 hypothetical protein	fig|6666666.59329.peg.1422	fig|6666666.59330.peg.4981	None	None	None	None	fig|6666666.59336.peg.2842	fig|6666666.59333.peg.2383	None	None	None	fig|6666666.59337.peg.2240	fig|6666666.59342.peg.988	None	None	fig|6666666.59345.peg.4229	None	None	None	None	None	None	None	None	None	None

8763	Capsular polysaccharide synthesis enzyme CpsH	fig|6666666.59329.peg.540	fig|6666666.59330.peg.3600	None	None	None	None	fig|6666666.59336.peg.1143	fig|6666666.59333.peg.986	None	None	None	fig|6666666.59337.peg.3005	fig|6666666.59342.peg.4313	None	None	fig|6666666.59345.peg.632	None	None	None	None	None	None	None	None	None	None

5996	Putative arylsulfatase regulatory protein	None	None	fig|6666666.59331.peg.885	None	fig|6666666.59334.peg.5376	None	None	None	fig|6666666.59338.peg.5631	None	fig|6666666.59340.peg.3237	None	fig|6666666.59342.peg.3405	None	None	None	fig|6666666.59341.peg.5886	None	fig|6666666.59346.peg.5209	None	None	None	None	None	None	None

6780	DedA family inner membrane protein YdjX	fig|6666666.59329.peg.5090	None	None	None	None	None	fig|6666666.59336.peg.4396	None	None	None	None	fig|6666666.59337.peg.3666	fig|6666666.59342.peg.4754	fig|6666666.71181.peg.3704	None	fig|6666666.59345.peg.1244	None	None	None	None	None	None	None	None	fig|6666666.59355.peg.3713	None

10953	PTS system, mannose-specific IIC component EC 2.7.1.69	fig|6666666.59329.peg.619	None	None	None	None	None	None	None	fig|6666666.59338.peg.4057	None	None	None	None	fig|6666666.71181.peg.4238	fig|6666666.59344.peg.2569	None	fig|6666666.59341.peg.3084	None	None	None	None	None	None	fig|6666666.59353.peg.1532	fig|6666666.59355.peg.2081	None

7193	Cation transport ATPase	None	None	fig|6666666.59331.peg.6186	fig|6666666.59332.peg.6080	None	fig|6666666.59335.peg.4625	None	None	fig|6666666.59338.peg.3769	None	None	None	None	None	None	None	fig|6666666.59341.peg.2020	fig|6666666.59347.peg.1609	fig|6666666.59346.peg.3579	None	None	None	None	None	None	None

7196	FIG01201774 hypothetical protein	fig|6666666.59329.peg.1732	fig|6666666.59330.peg.3821	None	None	None	None	fig|6666666.59336.peg.6482	fig|6666666.59333.peg.3704	None	None	None	fig|6666666.59337.peg.2707	fig|6666666.59342.peg.4988	None	None	fig|6666666.59345.peg.3185	None	None	None	None	None	None	None	None	None	None

7442	FIG01201814 hypothetical protein	fig|6666666.59329.peg.4620	fig|6666666.59330.peg.1960	None	None	None	None	fig|6666666.59336.peg.2670	fig|6666666.59333.peg.2530	None	None	None	fig|6666666.59337.peg.2621	fig|6666666.59342.peg.4214	None	None	fig|6666666.59345.peg.726	None	None	None	None	None	None	None	None	None	None

4814	Transport ATP-binding protein CydC	None	fig|6666666.59330.peg.3723	None	None	fig|6666666.59334.peg.5113	None	fig|6666666.59336.peg.4040	None	None	fig|6666666.59339.peg.3655	None	fig|6666666.59337.peg.4126	fig|6666666.59342.peg.1555	None	None	None	None	None	fig|6666666.59346.peg.2518	None	None	None	None	None	None	None

6226	Putative uncharacterized protein ydbL, may be related to amine metabolism	None	None	None	None	None	None	None	None	None	None	None	None	None	fig|6666666.71181.peg.1363	fig|6666666.59344.peg.564	None	None	None	None	None	fig|6666666.59349.peg.2794	None	fig|6666666.59351.peg.5197	fig|6666666.59353.peg.1868	fig|6666666.59355.peg.1145	fig|6666666.59354.peg.5500

8706	hypothetical protein	fig|6666666.59329.peg.3766	None	None	fig|6666666.59332.peg.6054	fig|6666666.59334.peg.796	None	fig|6666666.59336.peg.4973	None	fig|6666666.59338.peg.2327	None	None	None	None	None	None	fig|6666666.59345.peg.985	fig|6666666.59341.peg.4936	None	None	None	None	None	None	None	None	None

6425	Oxidoreductase, short chain dehydrogenase reductase family	None	None	None	None	None	None	fig|6666666.59336.peg.4131	None	None	None	None	fig|6666666.59337.peg.2576	fig|6666666.59342.peg.2847	None	fig|6666666.59344.peg.6242	fig|6666666.59345.peg.690	None	None	None	None	None	None	None	fig|6666666.59353.peg.4343	fig|6666666.59355.peg.4526	None

10778	hypothetical protein	None	None	None	None	fig|6666666.59334.peg.4903	fig|6666666.59335.peg.4770	None	None	fig|6666666.59338.peg.2402	fig|6666666.59339.peg.938	None	None	None	None	None	None	fig|6666666.59341.peg.3772	fig|6666666.59347.peg.2210	None	fig|6666666.59348.peg.4104	None	None	None	None	None	None

8040	Mobile element protein	fig|6666666.59329.peg.5742	fig|6666666.59330.peg.5139	fig|6666666.59331.peg.5031	None	fig|6666666.59334.peg.5359	None	None	None	fig|6666666.59338.peg.5872	None	None	fig|6666666.59337.peg.2098	None	None	None	None	None	None	None	None	None	None	fig|6666666.59351.peg.4154	None	None	None

8042	ATPase involved in DNA repair	None	None	fig|6666666.59331.peg.1980	fig|6666666.59332.peg.3189	None	None	None	None	fig|6666666.59338.peg.1131	None	None	None	None	None	None	None	fig|6666666.59341.peg.6055	fig|6666666.59347.peg.2310	fig|6666666.59346.peg.4556	None	None	None	fig|6666666.59351.peg.4847	None	None	None

9701	FIG001957 putative hydrolase	None	None	None	None	None	None	None	None	None	None	None	None	None	fig|6666666.71181.peg.1841	fig|6666666.59344.peg.5475	None	None	None	None	None	fig|6666666.59349.peg.2286	None	fig|6666666.59351.peg.4167	fig|6666666.59353.peg.2208	fig|6666666.59355.peg.1900	fig|6666666.59354.peg.4741

829	FIG00951794 hypothetical protein	None	None	None	None	None	None	None	None	None	None	None	None	None	fig|6666666.71181.peg.3694	fig|6666666.59344.peg.3290	None	None	None	None	None	fig|6666666.59349.peg.1920	None	fig|6666666.59351.peg.483	fig|6666666.59353.peg.2789	fig|6666666.59355.peg.3703	fig|6666666.59354.peg.3298

3019	Fimbrial subunit	None	None	None	None	None	None	None	None	None	None	None	None	fig|6666666.59342.peg.5274	fig|6666666.71181.peg.111	fig|6666666.59344.peg.3145	fig|6666666.59345.peg.2851	fig|6666666.59341.peg.217	None	None	None	None	None	None	fig|6666666.59353.peg.1902	fig|6666666.59355.peg.1507	None

4186	Asp-tRNAAsn Glu-tRNAGln amidotransferase A subunit and related amidases	None	None	None	None	fig|6666666.59334.peg.2378	None	None	None	None	None	None	None	None	fig|6666666.71181.peg.3724	fig|6666666.59344.peg.3693	None	None	None	None	None	None	fig|6666666.59350.peg.449	fig|6666666.59351.peg.190	fig|6666666.59353.peg.4539	fig|6666666.59355.peg.1562	None

15	hypothetical protein	fig|6666666.59329.peg.4518	None	None	None	None	None	None	None	None	None	None	None	None	fig|6666666.71181.peg.2861	fig|6666666.59344.peg.4402	None	None	None	None	None	fig|6666666.59349.peg.1232	None	None	fig|6666666.59353.peg.4314	fig|6666666.59355.peg.4388	fig|6666666.59354.peg.5885

18	Polymyxin resistance protein ArnA DH, UDP-glucuronic acid decarboxylase EC 4.1.1.- Polymyxin resistance protein ArnA FT, UDP-4-amino-4-deoxy-L-arabinose formylase EC 2.1.2.-	None	None	None	None	None	None	None	None	fig|6666666.59338.peg.3627	None	None	None	None	fig|6666666.71181.peg.2405	fig|6666666.59344.peg.791	None	fig|6666666.59341.peg.2061	fig|6666666.59347.peg.2349	None	None	None	None	None	fig|6666666.59353.peg.1316	fig|6666666.59355.peg.2555	None

9959	Arginine pathway regulatory protein ArgR, repressor of arg regulon	None	None	None	None	fig|6666666.59334.peg.5398	fig|6666666.59335.peg.462	None	None	fig|6666666.59338.peg.2822	fig|6666666.59339.peg.6471	None	None	None	None	None	None	fig|6666666.59341.peg.4993	fig|6666666.59347.peg.2614	None	fig|6666666.59348.peg.1226	None	None	None	None	None	None

4795	FIG00959802 hypothetical protein	None	None	fig|6666666.59331.peg.350	fig|6666666.59332.peg.524	None	None	None	None	None	None	None	None	None	fig|6666666.71181.peg.3870	fig|6666666.59344.peg.867	None	None	None	fig|6666666.59346.peg.188	None	None	None	None	fig|6666666.59353.peg.1251	fig|6666666.59355.peg.3334	None

6265	Curli production assembly transport component CsgE	None	None	None	None	None	None	None	None	fig|6666666.59338.peg.3447	None	fig|6666666.59340.peg.3089	None	None	None	None	None	fig|6666666.59341.peg.1973	None	None	fig|6666666.59348.peg.4745	fig|6666666.59349.peg.3058	None	fig|6666666.59351.peg.3058	None	None	fig|6666666.59354.peg.3700

4902	FIG01199982 hypothetical protein	fig|6666666.59329.peg.5691	fig|6666666.59330.peg.1521	None	None	None	None	fig|6666666.59336.peg.2593	fig|6666666.59333.peg.4474	None	None	None	fig|6666666.59337.peg.2310	fig|6666666.59342.peg.3910	None	None	fig|6666666.59345.peg.1288	None	None	None	None	None	None	None	None	None	None

9935	hypothetical protein	None	None	fig|6666666.59331.peg.1914	fig|6666666.59332.peg.1561	fig|6666666.59334.peg.2388	None	None	None	fig|6666666.59338.peg.2713	None	fig|6666666.59340.peg.1489	None	fig|6666666.59342.peg.4386	None	None	fig|6666666.59345.peg.4158	None	None	None	None	None	None	None	None	None	None

2548	DNA replication terminus site-binding protein	None	None	None	None	None	None	None	None	None	None	None	None	None	fig|6666666.71181.peg.2713	fig|6666666.59344.peg.3830	None	None	None	None	None	fig|6666666.59349.peg.4155	None	fig|6666666.59351.peg.1706	fig|6666666.59353.peg.3965	fig|6666666.59355.peg.3881	fig|6666666.59354.peg.6182

3583	SAM-dependent methyltransferase	fig|6666666.59329.peg.4804	None	fig|6666666.59331.peg.3032	fig|6666666.59332.peg.2628	None	None	fig|6666666.59336.peg.1058	fig|6666666.59333.peg.2907	None	None	None	None	None	None	None	None	fig|6666666.59341.peg.2751	None	None	fig|6666666.59348.peg.785	None	None	None	None	None	None

9559	DNA polymerase III delta subunit EC 2.7.7.7	fig|6666666.59329.peg.2164	None	fig|6666666.59331.peg.5578	fig|6666666.59332.peg.5142	None	None	None	fig|6666666.59333.peg.2137	None	None	None	None	fig|6666666.59342.peg.906	None	fig|6666666.59344.peg.4495	None	None	None	fig|6666666.59346.peg.3301	None	None	None	None	None	None	None

1692	hypothetical protein	None	None	None	None	None	None	None	None	None	None	None	None	None	fig|6666666.71181.peg.2325	fig|6666666.59344.peg.4541	None	None	None	None	None	fig|6666666.59349.peg.1662	None	fig|6666666.59351.peg.2402	fig|6666666.59353.peg.3329	fig|6666666.59355.peg.3465	fig|6666666.59354.peg.2064

1066	FIG027785 Fimbriae usher protein StfC	None	None	None	None	None	None	None	None	None	None	None	None	fig|6666666.59342.peg.824	fig|6666666.71181.peg.752	fig|6666666.59344.peg.733	fig|6666666.59345.peg.2849	fig|6666666.59341.peg.1739	None	None	None	None	None	None	fig|6666666.59353.peg.949	fig|6666666.59355.peg.2429	None

3947	Lipopolysaccharide biosynthesis protein WzxC	None	None	None	None	None	None	None	fig|6666666.59333.peg.5380	None	None	None	None	None	fig|6666666.71181.peg.226	fig|6666666.59344.peg.276	None	None	fig|6666666.59347.peg.5010	None	None	None	fig|6666666.59350.peg.843	None	fig|6666666.59353.peg.596	fig|6666666.59355.peg.78	None

4446	Tagatose-6-phosphate kinase AgaZ EC 2.7.1.144	fig|6666666.59329.peg.615	None	None	None	None	None	None	None	None	None	None	None	None	fig|6666666.71181.peg.2665	fig|6666666.59344.peg.4309	None	fig|6666666.59341.peg.6010	fig|6666666.59347.peg.4709	None	None	None	None	None	fig|6666666.59353.peg.3880	fig|6666666.59355.peg.3814	None

5195	Accessory colonization factor AcfD precursor	fig|6666666.59329.peg.4349	None	fig|6666666.59331.peg.2334	None	None	None	fig|6666666.59336.peg.5768	None	None	None	None	fig|6666666.59337.peg.4713	fig|6666666.59342.peg.4279	None	None	fig|6666666.59345.peg.3127	None	None	fig|6666666.59346.peg.2943	None	None	None	None	None	None	None

305	Type III secretion chaperone protein for YopD SycD	fig|6666666.59329.peg.533	fig|6666666.59330.peg.3281	None	None	None	fig|6666666.59335.peg.6080	None	None	None	fig|6666666.59339.peg.1370	None	fig|6666666.59337.peg.2809	fig|6666666.59342.peg.4695	None	None	fig|6666666.59345.peg.1453	None	None	None	None	None	None	None	None	None	None

10583	hypothetical protein	None	fig|6666666.59330.peg.5108	None	None	None	None	None	None	fig|6666666.59338.peg.4104	fig|6666666.59339.peg.8120	None	fig|6666666.59337.peg.4305	fig|6666666.59342.peg.3533	None	None	None	None	fig|6666666.59347.peg.3726	None	None	None	fig|6666666.59350.peg.3956	None	None	None	None

1080	3-methyl-2-oxobutanoate hydroxymethyltransferase EC 2.1.2.11	None	None	None	None	None	fig|6666666.59335.peg.2328	fig|6666666.59336.peg.2744	None	None	fig|6666666.59339.peg.4766	None	fig|6666666.59337.peg.621	fig|6666666.59342.peg.1574	None	fig|6666666.59344.peg.4435	None	None	None	None	fig|6666666.59348.peg.1696	None	None	None	None	None	None

4468	Chromosomal replication initiator protein DnaA	None	None	None	None	None	None	None	None	None	None	None	None	None	fig|6666666.71181.peg.3268	fig|6666666.59344.peg.4024	None	None	None	None	None	fig|6666666.59349.peg.3586	None	fig|6666666.59351.peg.4430	fig|6666666.59353.peg.4027	fig|6666666.59355.peg.3985	fig|6666666.59354.peg.6358

2837	FIG01200226 hypothetical protein	None	None	None	None	None	fig|6666666.59335.peg.1679	None	None	fig|6666666.59338.peg.3879	fig|6666666.59339.peg.3676	fig|6666666.59340.peg.2755	None	None	None	None	None	fig|6666666.59341.peg.1407	fig|6666666.59347.peg.1960	None	fig|6666666.59348.peg.4392	None	None	None	None	None	None

4262	Permeases of the major facilitator superfamily	None	None	None	None	None	None	None	None	None	None	None	None	fig|6666666.59342.peg.5681	fig|6666666.71181.peg.2296	fig|6666666.59344.peg.4700	fig|6666666.59345.peg.4182	None	None	None	None	None	fig|6666666.59350.peg.3126	None	fig|6666666.59353.peg.4485	fig|6666666.59355.peg.4592	None

2793	hypothetical protein	None	fig|6666666.59330.peg.3251	fig|6666666.59331.peg.4242	fig|6666666.59332.peg.53	fig|6666666.59334.peg.5475	fig|6666666.59335.peg.4875	None	None	None	None	None	fig|6666666.59337.peg.2160	None	None	None	None	None	None	fig|6666666.59346.peg.2812	None	None	None	None	None	None	None

8363	Methyl-accepting chemotaxis protein I serine chemoreceptor protein	fig|6666666.59329.peg.4041	None	fig|6666666.59331.peg.1392	fig|6666666.59332.peg.5449	None	fig|6666666.59335.peg.2465	None	None	None	fig|6666666.59339.peg.3567	None	None	None	None	None	None	None	None	None	fig|6666666.59348.peg.3639	None	None	fig|6666666.59351.peg.2234	None	None	None

1442	Uncharacterized protein ybfE	None	None	None	None	None	None	None	None	None	None	None	None	None	fig|6666666.71181.peg.3811	fig|6666666.59344.peg.5087	None	None	None	None	None	fig|6666666.59349.peg.1815	None	fig|6666666.59351.peg.1114	fig|6666666.59353.peg.2058	fig|6666666.59355.peg.2769	fig|6666666.59354.peg.4091

10632	FIG01200413 hypothetical protein	None	fig|6666666.59330.peg.3850	None	fig|6666666.59332.peg.2497	None	None	fig|6666666.59336.peg.5145	None	None	None	None	fig|6666666.59337.peg.4809	fig|6666666.59342.peg.4630	None	None	fig|6666666.59345.peg.311	None	None	fig|6666666.59346.peg.704	None	None	None	None	None	None	None

9617	SAM-dependent methyltransferases	None	None	None	None	None	None	None	None	None	None	None	None	None	fig|6666666.71181.peg.3999	fig|6666666.59344.peg.5628	None	None	None	None	None	fig|6666666.59349.peg.3106	None	fig|6666666.59351.peg.2983	fig|6666666.59353.peg.2978	fig|6666666.59355.peg.2883	fig|6666666.59354.peg.2692

11080	Chitin catabolic cascade sensor histidine kinase ChiS	None	fig|6666666.59330.peg.134	fig|6666666.59331.peg.4454	fig|6666666.59332.peg.4882	None	None	None	None	None	None	fig|6666666.59340.peg.2173	fig|6666666.59337.peg.658	None	None	None	None	None	None	fig|6666666.59346.peg.274	fig|6666666.59348.peg.1284	None	None	None	None	None	None

1375	probable integral membrane protein NMA1777	None	None	fig|6666666.59331.peg.1996	fig|6666666.59332.peg.5304	None	fig|6666666.59335.peg.2800	None	None	None	fig|6666666.59339.peg.1977	None	None	None	None	None	None	None	None	fig|6666666.59346.peg.3424	fig|6666666.59348.peg.1583	None	None	fig|6666666.59351.peg.1132	None	None	None

1374	Histidyl-tRNA synthetase EC 6.1.1.21	None	fig|6666666.59330.peg.1427	None	fig|6666666.59332.peg.5276	None	None	fig|6666666.59336.peg.684	None	fig|6666666.59338.peg.2291	None	None	None	fig|6666666.59342.peg.1063	None	None	None	None	None	fig|6666666.59346.peg.1317	None	None	None	None	None	fig|6666666.59355.peg.5532	None

7854	Anaerobic glycerol-3-phosphate dehydrogenase subunit B EC 1.1.5.3	None	None	None	None	None	fig|6666666.59335.peg.3449	None	None	fig|6666666.59338.peg.5435	None	None	None	None	fig|6666666.71181.peg.4494	fig|6666666.59344.peg.5723	None	None	fig|6666666.59347.peg.2081	None	None	None	None	None	fig|6666666.59353.peg.4945	fig|6666666.59355.peg.3348	None

10408	Glucose-6-phosphate 1-dehydrogenase EC 1.1.1.49	fig|6666666.59329.peg.1810	None	None	fig|6666666.59332.peg.1390	fig|6666666.59334.peg.3017	None	None	None	None	fig|6666666.59339.peg.2438	None	None	fig|6666666.59342.peg.2266	None	None	None	fig|6666666.59341.peg.3392	None	None	None	None	fig|6666666.59350.peg.5944	None	None	None	None

1611	Putative periplasmic protein YibQ, distant homology with nucleoside diphosphatase and polysaccharide deacetylase	None	None	None	None	None	None	None	None	None	None	None	None	None	fig|6666666.71181.peg.3674	fig|6666666.59344.peg.965	None	None	None	None	None	fig|6666666.59349.peg.2483	None	fig|6666666.59351.peg.369	fig|6666666.59353.peg.2572	fig|6666666.59355.peg.2629	fig|6666666.59354.peg.57

5440	4-hydroxyphenylacetate 3-monooxygenase, reductase component EC 1.6.8.-	None	None	None	None	None	None	None	None	None	None	None	None	None	fig|6666666.71181.peg.4560	fig|6666666.59344.peg.1408	None	fig|6666666.59341.peg.3443	fig|6666666.59347.peg.1331	None	None	None	fig|6666666.59350.peg.2043	None	fig|6666666.59353.peg.13	fig|6666666.59355.peg.619	None

4522	Putative hydroxymethyltransferase	None	fig|6666666.59330.peg.1783	None	None	None	fig|6666666.59335.peg.3656	fig|6666666.59336.peg.661	None	None	fig|6666666.59339.peg.3322	None	fig|6666666.59337.peg.2268	fig|6666666.59342.peg.3291	None	None	fig|6666666.59345.peg.2037	None	None	None	None	None	None	None	None	None	None

8143	FIG01200041 hypothetical protein	fig|6666666.59329.peg.1180	fig|6666666.59330.peg.4272	None	None	None	None	fig|6666666.59336.peg.1464	fig|6666666.59333.peg.4408	None	None	None	fig|6666666.59337.peg.3569	fig|6666666.59342.peg.1179	None	None	fig|6666666.59345.peg.844	None	None	None	None	None	None	None	None	None	None

8147	Curli production assembly transport component CsgF	None	None	None	None	None	None	None	None	fig|6666666.59338.peg.5372	None	None	None	None	None	None	None	fig|6666666.59341.peg.1974	fig|6666666.59347.peg.1141	None	fig|6666666.59348.peg.4746	fig|6666666.59349.peg.3059	None	fig|6666666.59351.peg.3059	None	None	fig|6666666.59354.peg.3699

9299	FIG01200462 hypothetical protein	fig|6666666.59329.peg.4219	fig|6666666.59330.peg.3109	fig|6666666.59331.peg.1858	fig|6666666.59332.peg.2337	None	None	None	fig|6666666.59333.peg.222	None	None	None	fig|6666666.59337.peg.5630	None	None	None	None	None	None	fig|6666666.59346.peg.3348	None	None	None	None	None	None	None

2990	Adenylosuccinate synthetase EC 6.3.4.4	fig|6666666.59329.peg.3855	fig|6666666.59330.peg.3203	None	None	None	None	None	fig|6666666.59333.peg.1308	None	None	None	fig|6666666.59337.peg.5143	fig|6666666.59342.peg.3751	None	None	fig|6666666.59345.peg.1672	fig|6666666.59341.peg.2750	None	None	None	None	None	None	None	None	None

2992	Protocatechuate 4,5-dioxygenase beta chain EC 1.13.11.8	None	None	fig|6666666.59331.peg.347	fig|6666666.59332.peg.520	None	None	None	None	None	None	None	None	None	fig|6666666.71181.peg.3866	fig|6666666.59344.peg.862	None	None	None	fig|6666666.59346.peg.185	None	None	None	None	fig|6666666.59353.peg.1255	fig|6666666.59355.peg.3329	None

420	GTP cyclohydrolase I EC 3.5.4.16 type 2	None	None	None	None	None	None	None	None	None	None	None	None	None	fig|6666666.71181.peg.1393	fig|6666666.59344.peg.527	None	None	None	None	None	fig|6666666.59349.peg.3649	fig|6666666.59350.peg.1770	fig|6666666.59351.peg.290	fig|6666666.59353.peg.1835	fig|6666666.59355.peg.1179	None

2434	Mobile element protein	None	fig|6666666.59330.peg.5132	None	None	None	fig|6666666.59335.peg.5558	None	None	None	None	None	fig|6666666.59337.peg.2924	None	fig|6666666.71181.peg.5380	None	fig|6666666.59345.peg.5161	None	fig|6666666.59347.peg.1213	None	None	None	fig|6666666.59350.peg.4634	None	None	None	None

1942	Metal-dependent phosphohydrolase, HD subdomain	None	fig|6666666.59330.peg.4552	None	None	fig|6666666.59334.peg.3342	None	fig|6666666.59336.peg.6444	None	fig|6666666.59338.peg.5725	None	None	None	fig|6666666.59342.peg.1013	None	None	fig|6666666.59345.peg.266	None	fig|6666666.59347.peg.788	None	None	None	None	None	None	None	None

1235	Alpha-1,2-mannosidase	fig|6666666.59329.peg.5133	fig|6666666.59330.peg.3545	None	None	None	None	fig|6666666.59336.peg.4310	None	None	None	None	fig|6666666.59337.peg.3251	fig|6666666.59342.peg.2053	None	None	fig|6666666.59345.peg.1776	None	None	None	None	None	None	fig|6666666.59351.peg.4719	None	None	None

7319	Type III secretion inner membrane protein YscD,homologous to flagellar export components	fig|6666666.59329.peg.2727	fig|6666666.59330.peg.3754	None	None	None	fig|6666666.59335.peg.5182	None	fig|6666666.59333.peg.2991	None	None	None	fig|6666666.59337.peg.4463	fig|6666666.59342.peg.625	None	None	fig|6666666.59345.peg.1486	None	None	None	None	None	None	None	None	None	None

7034	hypothetical protein	None	None	None	None	fig|6666666.59334.peg.4564	None	fig|6666666.59336.peg.657	None	fig|6666666.59338.peg.3418	None	fig|6666666.59340.peg.473	None	fig|6666666.59342.peg.5239	None	None	fig|6666666.59345.peg.2041	None	fig|6666666.59347.peg.3717	None	None	None	None	None	None	None	None

9588	1,2-dihydroxy-3-keto-5-methylthiopentene dioxygenase EC 1.13.11.54	None	None	None	None	None	None	None	None	None	None	None	None	None	fig|6666666.71181.peg.908	fig|6666666.59344.peg.5183	None	None	None	None	None	fig|6666666.59349.peg.2219	None	fig|6666666.59351.peg.5007	fig|6666666.59353.peg.910	fig|6666666.59355.peg.797	fig|6666666.59354.peg.3601

9377	hypothetical protein	None	fig|6666666.59330.peg.4815	None	fig|6666666.59332.peg.1123	None	None	fig|6666666.59336.peg.5895	None	None	None	None	None	fig|6666666.59342.peg.5189	None	None	fig|6666666.59345.peg.4783	None	None	None	fig|6666666.59348.peg.5338	None	None	fig|6666666.59351.peg.3323	None	None	None

63	2-dehydro-3-deoxygalactonokinase EC 2.7.1.58	None	None	None	None	fig|6666666.59334.peg.3894	None	None	None	None	fig|6666666.59339.peg.2330	None	None	None	fig|6666666.71181.peg.631	fig|6666666.59344.peg.4184	None	None	None	None	None	None	fig|6666666.59350.peg.273	None	fig|6666666.59353.peg.3048	fig|6666666.59355.peg.2298	None

1216	FIG01202033 hypothetical protein	None	fig|6666666.59330.peg.1364	fig|6666666.59331.peg.4149	fig|6666666.59332.peg.1734	None	None	fig|6666666.59336.peg.72	None	fig|6666666.59338.peg.1293	None	None	None	fig|6666666.59342.peg.3742	None	None	None	None	None	fig|6666666.59346.peg.5219	None	None	None	None	None	None	None

8261	Lipoate-protein ligase A	fig|6666666.59329.peg.4932	fig|6666666.59330.peg.2049	None	None	None	None	fig|6666666.59336.peg.722	fig|6666666.59333.peg.1751	None	None	None	fig|6666666.59337.peg.4318	None	None	None	fig|6666666.59345.peg.3909	None	None	fig|6666666.59346.peg.957	None	None	None	None	None	None	None

10782	Dipeptide-binding ABC transporter, periplasmic substrate-binding component TC 3.A.1.5.2	None	None	None	None	fig|6666666.59334.peg.4330	None	None	None	None	None	None	None	None	fig|6666666.71181.peg.3751	fig|6666666.59344.peg.4530	None	fig|6666666.59341.peg.2982	None	None	None	None	fig|6666666.59350.peg.5735	None	fig|6666666.59353.peg.4656	fig|6666666.59355.peg.3474	None

2958	FIG01201879 hypothetical protein	fig|6666666.59329.peg.2897	fig|6666666.59330.peg.4550	None	None	None	None	fig|6666666.59336.peg.3654	fig|6666666.59333.peg.3206	None	None	None	fig|6666666.59337.peg.4050	fig|6666666.59342.peg.4728	None	None	fig|6666666.59345.peg.277	None	None	None	None	None	None	None	None	None	None

3103	NADPH-dependent 7-cyano-7-deazaguanine reductase EC 1.7.1.13	fig|6666666.59329.peg.2943	None	None	None	None	None	fig|6666666.59336.peg.3261	None	None	fig|6666666.59339.peg.7148	None	fig|6666666.59337.peg.1115	None	None	None	fig|6666666.59345.peg.3518	None	None	None	None	None	None	None	None	fig|6666666.59355.peg.3795	fig|6666666.59354.peg.2635

5739	Succinylglutamate desuccinylase EC 3.5.1.96	None	None	None	fig|6666666.59332.peg.828	None	None	None	fig|6666666.59333.peg.1780	fig|6666666.59338.peg.5581	fig|6666666.59339.peg.3913	None	None	fig|6666666.59342.peg.2064	None	None	None	None	None	None	None	None	None	fig|6666666.59351.peg.1107	None	None	fig|6666666.59354.peg.4948

5469	Transcriptional regulator, ArsR family	None	None	None	None	None	None	None	None	None	None	None	None	None	fig|6666666.71181.peg.160	fig|6666666.59344.peg.3744	None	fig|6666666.59341.peg.5255	None	None	None	None	fig|6666666.59350.peg.309	fig|6666666.59351.peg.4587	fig|6666666.59353.peg.658	fig|6666666.59355.peg.141	None

3848	Phosphate ABC transporter, periplasmic phosphate-binding protein PstS TC 3.A.1.7.1	None	None	fig|6666666.59331.peg.4315	fig|6666666.59332.peg.1268	fig|6666666.59334.peg.1110	None	None	None	None	fig|6666666.59339.peg.6916	None	fig|6666666.59337.peg.707	None	None	None	None	None	None	fig|6666666.59346.peg.2540	fig|6666666.59348.peg.3207	None	None	None	None	None	None

2625	Flavodoxin reductases ferredoxin-NADPH reductases family 1	None	None	None	fig|6666666.59332.peg.598	fig|6666666.59334.peg.2983	None	fig|6666666.59336.peg.4842	None	fig|6666666.59338.peg.3717	None	None	None	fig|6666666.59342.peg.1530	None	None	fig|6666666.59345.peg.1438	None	fig|6666666.59347.peg.1104	None	None	None	None	None	None	None	None

1047	Quinolinate synthetase EC 2.5.1.72	fig|6666666.59329.peg.1625	None	fig|6666666.59331.peg.5120	fig|6666666.59332.peg.4696	fig|6666666.59334.peg.2374	None	None	None	fig|6666666.59338.peg.1479	None	None	None	None	None	fig|6666666.59344.peg.4054	None	None	None	None	None	fig|6666666.59349.peg.2656	None	None	None	None	None

2750	hypothetical protein	None	None	fig|6666666.59331.peg.1488	None	fig|6666666.59334.peg.1823	None	fig|6666666.59336.peg.2294	None	fig|6666666.59338.peg.5326	None	None	None	fig|6666666.59342.peg.4539	None	None	fig|6666666.59345.peg.1410	None	fig|6666666.59347.peg.2201	None	None	None	None	None	None	None	None

10861	lambda repressor-like DNA-binding protein	None	fig|6666666.59330.peg.4302	None	fig|6666666.59332.peg.464	None	None	fig|6666666.59336.peg.3879	None	None	None	None	fig|6666666.59337.peg.4003	fig|6666666.59342.peg.5764	None	None	fig|6666666.59345.peg.1701	None	None	fig|6666666.59346.peg.2475	None	None	None	None	None	None	None

10867	FIG01204022 hypothetical protein	fig|6666666.59329.peg.4875	fig|6666666.59330.peg.2737	None	None	None	None	fig|6666666.59336.peg.6454	None	fig|6666666.59338.peg.3540	None	None	fig|6666666.59337.peg.4405	None	None	None	fig|6666666.59345.peg.3427	None	fig|6666666.59347.peg.1805	None	None	None	None	None	None	None	None

3726	Hydantoin racemase EC 5.1.99.-	None	None	None	None	fig|6666666.59334.peg.2227	None	None	None	None	fig|6666666.59339.peg.6146	None	None	None	fig|6666666.71181.peg.882	fig|6666666.59344.peg.4758	None	None	None	None	None	None	fig|6666666.59350.peg.5349	None	fig|6666666.59353.peg.4551	fig|6666666.59355.peg.4646	None

7609	Putative transport protein	None	None	None	None	None	None	None	None	None	None	None	None	None	fig|6666666.71181.peg.2039	fig|6666666.59344.peg.1083	None	None	None	None	None	fig|6666666.59349.peg.4449	None	fig|6666666.59351.peg.3413	fig|6666666.59353.peg.765	fig|6666666.59355.peg.939	fig|6666666.59354.peg.6196

9179	Outer membrane lipoprotein carrier protein LolA	None	fig|6666666.59330.peg.1927	None	None	None	fig|6666666.59335.peg.3959	fig|6666666.59336.peg.2307	None	fig|6666666.59338.peg.2186	None	None	fig|6666666.59337.peg.4864	None	None	None	fig|6666666.59345.peg.1338	None	fig|6666666.59347.peg.2395	None	None	None	None	None	None	None	None

9859	ParD protein antitoxin to ParE	None	fig|6666666.59330.peg.4837	None	None	None	None	None	None	None	None	None	fig|6666666.59337.peg.5130	None	fig|6666666.71181.peg.4832	fig|6666666.59344.peg.5603	None	None	None	None	None	None	fig|6666666.59350.peg.3570	None	fig|6666666.59353.peg.4890	fig|6666666.59355.peg.4961	None

9178	Selenide,water dikinase EC 2.7.9.3	None	None	None	None	None	None	None	None	None	None	None	fig|6666666.59337.peg.5127	None	fig|6666666.71181.peg.4905	fig|6666666.59344.peg.2900	None	fig|6666666.59341.peg.3074	None	None	None	None	fig|6666666.59350.peg.80	None	fig|6666666.59353.peg.1427	fig|6666666.59355.peg.1976	None

3343	FIG01061772 hypothetical protein	None	None	fig|6666666.59331.peg.4061	fig|6666666.59332.peg.6105	None	None	None	None	fig|6666666.59338.peg.3318	None	None	None	None	None	None	None	fig|6666666.59341.peg.3112	fig|6666666.59347.peg.3282	fig|6666666.59346.peg.4569	None	None	fig|6666666.59350.peg.1199	None	None	None	None

10048	3-hydroxyisobutyrate dehydrogenase and related beta-hydroxyacid dehydrogenases	None	None	fig|6666666.59331.peg.276	fig|6666666.59332.peg.528	None	None	None	None	None	None	None	None	None	fig|6666666.71181.peg.3873	fig|6666666.59344.peg.871	None	None	None	fig|6666666.59346.peg.2999	None	None	None	None	fig|6666666.59353.peg.1248	fig|6666666.59355.peg.3338	None

9077	Allantoicase EC 3.5.3.4	None	None	None	fig|6666666.59332.peg.2249	None	fig|6666666.59335.peg.1142	None	None	None	fig|6666666.59339.peg.1649	None	None	None	None	None	None	fig|6666666.59341.peg.4529	fig|6666666.59347.peg.3328	fig|6666666.59346.peg.4747	fig|6666666.59348.peg.2416	None	None	None	None	None	None

3551	FIG01202573 hypothetical protein	None	None	fig|6666666.59331.peg.4826	fig|6666666.59332.peg.912	None	None	None	None	fig|6666666.59338.peg.3867	None	fig|6666666.59340.peg.663	None	None	None	None	None	fig|6666666.59341.peg.2835	fig|6666666.59347.peg.326	fig|6666666.59346.peg.5202	None	None	None	None	None	None	None

2910	FIG01204495 hypothetical protein	None	fig|6666666.59330.peg.4404	None	None	fig|6666666.59334.peg.4976	None	None	fig|6666666.59333.peg.2022	None	fig|6666666.59339.peg.437	None	fig|6666666.59337.peg.2414	None	None	None	fig|6666666.59345.peg.946	None	None	None	fig|6666666.59348.peg.3733	None	None	None	None	None	None

2913	Uncharacterized protein ynbE; probable lipoprotein STY1424	None	None	None	None	None	None	None	None	None	None	None	None	None	fig|6666666.71181.peg.1364	fig|6666666.59344.peg.563	None	None	None	None	None	fig|6666666.59349.peg.2795	None	fig|6666666.59351.peg.5196	fig|6666666.59353.peg.1867	fig|6666666.59355.peg.1146	fig|6666666.59354.peg.5501

6613	Phosphoribosylformimino-5-aminoimidazole carboxamide ribotide isomerase EC 5.3.1.16	None	None	fig|6666666.59331.peg.4534	None	fig|6666666.59334.peg.3965	None	fig|6666666.59336.peg.4088	None	None	fig|6666666.59339.peg.500	None	None	None	None	fig|6666666.59344.peg.306	None	None	None	None	None	None	None	None	fig|6666666.59353.peg.574	fig|6666666.59355.peg.58	None

10845	DNA primase EC 2.7.7.-	None	None	None	None	fig|6666666.59334.peg.2468	None	None	None	fig|6666666.59338.peg.696	None	fig|6666666.59340.peg.1275	None	fig|6666666.59342.peg.1636	None	None	None	fig|6666666.59341.peg.3723	None	None	fig|6666666.59348.peg.2744	None	None	None	None	None	fig|6666666.59354.peg.13

1886	hypothetical protein	None	None	None	None	None	fig|6666666.59335.peg.4446	fig|6666666.59336.peg.4051	None	None	fig|6666666.59339.peg.2074	None	None	fig|6666666.59342.peg.3358	None	None	fig|6666666.59345.peg.5704	None	None	None	None	None	None	fig|6666666.59351.peg.5108	None	None	fig|6666666.59354.peg.6116

4915	Sodium glutamate symporter	fig|6666666.59329.peg.4486	fig|6666666.59330.peg.1966	None	None	fig|6666666.59334.peg.4268	None	fig|6666666.59336.peg.2678	fig|6666666.59333.peg.4337	None	None	None	None	fig|6666666.59342.peg.2801	None	None	fig|6666666.59345.peg.732	None	None	None	None	None	None	None	None	None	None

5173	S-adenosylmethionine decarboxylase proenzyme EC 4.1.1.50 , prokaryotic class 1A	None	None	None	None	None	None	None	None	None	None	None	None	None	fig|6666666.71181.peg.2855	fig|6666666.59344.peg.4422	None	None	None	None	None	fig|6666666.59349.peg.3800	None	fig|6666666.59351.peg.3879	fig|6666666.59353.peg.3207	fig|6666666.59355.peg.3089	fig|6666666.59354.peg.3997

7257	FIG01204073 hypothetical protein	fig|6666666.59329.peg.3807	fig|6666666.59330.peg.3136	None	None	None	fig|6666666.59335.peg.4445	fig|6666666.59336.peg.3951	None	None	fig|6666666.59339.peg.2075	None	fig|6666666.59337.peg.1419	None	None	None	fig|6666666.59345.peg.3862	None	None	None	None	None	None	None	None	None	None

7250	1-hydroxy-2-methyl-2- E -butenyl 4-diphosphate synthase EC 1.17.7.1	None	None	fig|6666666.59331.peg.1633	None	fig|6666666.59334.peg.1253	fig|6666666.59335.peg.1111	None	None	fig|6666666.59338.peg.2295	fig|6666666.59339.peg.6969	None	None	None	None	None	None	None	fig|6666666.59347.peg.4295	None	None	None	None	None	None	None	fig|6666666.59354.peg.84

7621	Aspartate--ammonia ligase EC 6.3.1.1	None	None	None	None	None	None	None	None	None	None	None	None	None	fig|6666666.71181.peg.586	fig|6666666.59344.peg.4258	None	None	None	None	None	fig|6666666.59349.peg.4706	None	fig|6666666.59351.peg.313	fig|6666666.59353.peg.3094	fig|6666666.59355.peg.2254	fig|6666666.59354.peg.4397

9193	FIG01202450 hypothetical protein	fig|6666666.59329.peg.1144	None	None	None	fig|6666666.59334.peg.1065	None	fig|6666666.59336.peg.4018	fig|6666666.59333.peg.2162	None	None	None	None	fig|6666666.59342.peg.834	None	None	fig|6666666.59345.peg.442	None	fig|6666666.59347.peg.2151	None	None	None	None	None	None	None	None

9877	LSU ribosomal protein L13p L13Ae	None	None	None	None	None	fig|6666666.59335.peg.3436	fig|6666666.59336.peg.1587	None	fig|6666666.59338.peg.4447	None	fig|6666666.59340.peg.1136	None	None	None	None	None	None	None	None	fig|6666666.59348.peg.1450	None	fig|6666666.59350.peg.5608	None	None	None	fig|6666666.59354.peg.356

6001	Cytochrome d ubiquinol oxidase subunit I EC 1.10.3.-	fig|6666666.59329.peg.2856	None	fig|6666666.59331.peg.4302	fig|6666666.59332.peg.1963	None	fig|6666666.59335.peg.2500	None	None	fig|6666666.59338.peg.6147	fig|6666666.59339.peg.386	None	None	fig|6666666.59342.peg.4140	None	None	None	None	None	None	None	None	None	None	None	None	None

9092	FIG01201854 hypothetical protein	fig|6666666.59329.peg.1184	fig|6666666.59330.peg.4275	None	None	None	None	fig|6666666.59336.peg.1917	fig|6666666.59333.peg.940	None	None	None	fig|6666666.59337.peg.3566	fig|6666666.59342.peg.1175	None	None	fig|6666666.59345.peg.841	None	None	None	None	None	None	None	None	None	None

8560	FIG015373 Membrane protein	None	None	None	None	None	None	None	None	None	None	None	None	None	fig|6666666.71181.peg.3227	fig|6666666.59344.peg.4605	None	None	None	None	None	fig|6666666.59349.peg.2235	None	fig|6666666.59351.peg.886	fig|6666666.59353.peg.2079	fig|6666666.59355.peg.2746	fig|6666666.59354.peg.6095

3760	FIG01200986 hypothetical protein	fig|6666666.59329.peg.2214	fig|6666666.59330.peg.3134	None	None	None	None	fig|6666666.59336.peg.3949	fig|6666666.59333.peg.5396	None	None	None	fig|6666666.59337.peg.1421	None	None	None	fig|6666666.59345.peg.3860	fig|6666666.59341.peg.2889	None	None	None	None	None	None	None	None	None

6836	Cell division protein FtsK	None	None	None	fig|6666666.59332.peg.3208	None	None	fig|6666666.59336.peg.2305	fig|6666666.59333.peg.4332	None	fig|6666666.59339.peg.3426	None	None	None	None	fig|6666666.59344.peg.2823	None	None	None	None	None	None	None	None	None	fig|6666666.59355.peg.1091	fig|6666666.59354.peg.5451

10558	ATP-dependent helicase HrpA	None	None	None	None	None	None	None	None	fig|6666666.59338.peg.3536	fig|6666666.59339.peg.3621	None	None	fig|6666666.59342.peg.2410	None	fig|6666666.59344.peg.566	None	None	None	None	None	None	None	None	fig|6666666.59353.peg.1870	fig|6666666.59355.peg.1143	fig|6666666.59354.peg.6038

1748	hypothetical protein	fig|6666666.59329.peg.4309	fig|6666666.59330.peg.2654	None	fig|6666666.59332.peg.3848	None	None	None	None	None	None	None	fig|6666666.59337.peg.1988	None	fig|6666666.71181.peg.5962	None	fig|6666666.59345.peg.807	None	None	fig|6666666.59346.peg.1512	None	None	None	None	None	None	None

8483	putative Glutathione-regulated potassium-efflux system protein KefB	fig|6666666.59329.peg.1948	None	fig|6666666.59331.peg.5619	None	None	None	None	None	fig|6666666.59338.peg.5708	None	fig|6666666.59340.peg.2918	None	fig|6666666.59342.peg.1034	None	None	None	fig|6666666.59341.peg.285	None	None	fig|6666666.59348.peg.3021	None	None	None	None	None	None

7464	hypothetical protein	None	None	None	fig|6666666.59332.peg.4104	None	None	fig|6666666.59336.peg.6328	None	None	None	None	fig|6666666.59337.peg.2764	fig|6666666.59342.peg.5202	None	None	fig|6666666.59345.peg.790	None	None	fig|6666666.59346.peg.5223	None	None	None	fig|6666666.59351.peg.1324	None	None	None

5980	Nucleoside-diphosphate-sugar epimerases	None	None	None	None	None	None	None	None	None	None	None	None	None	fig|6666666.71181.peg.4582	fig|6666666.59344.peg.5498	None	None	None	None	None	None	None	fig|6666666.59351.peg.2687	fig|6666666.59353.peg.4826	fig|6666666.59355.peg.4894	fig|6666666.59354.peg.3777

6798	hypothetical protein	None	None	None	fig|6666666.59332.peg.629	None	None	fig|6666666.59336.peg.4729	None	None	None	None	None	fig|6666666.59342.peg.101	None	None	fig|6666666.59345.peg.4384	None	fig|6666666.59347.peg.2892	fig|6666666.59346.peg.2373	None	None	None	None	None	None	None

3516	LpsA protein	fig|6666666.59329.peg.554	fig|6666666.59330.peg.1163	None	None	None	None	fig|6666666.59336.peg.57	fig|6666666.59333.peg.649	None	None	None	fig|6666666.59337.peg.467	None	None	None	fig|6666666.59345.peg.2615	None	None	None	None	None	None	None	None	None	None

3515	RNA polymerase sigma factor RpoD	fig|6666666.59329.peg.679	None	fig|6666666.59331.peg.795	fig|6666666.59332.peg.3923	None	fig|6666666.59335.peg.1641	None	None	None	None	fig|6666666.59340.peg.347	None	None	None	None	None	None	None	fig|6666666.59346.peg.2563	None	None	None	None	None	None	None

2036	Nonspecific acid phosphatase precursor EC 3.1.3.2	None	None	None	None	None	None	fig|6666666.59336.peg.5604	None	None	None	None	None	None	fig|6666666.71181.peg.629	fig|6666666.59344.peg.4182	fig|6666666.59345.peg.208	None	None	None	None	None	None	None	fig|6666666.59353.peg.3050	fig|6666666.59355.peg.2296	None

10397	Sodium-dependent phosphate transporter	None	None	None	None	None	None	None	None	None	None	None	None	None	fig|6666666.71181.peg.2022	fig|6666666.59344.peg.2714	None	None	None	None	None	None	fig|6666666.59350.peg.2014	fig|6666666.59351.peg.4987	fig|6666666.59353.peg.3019	fig|6666666.59355.peg.2926	None

6774	FIG01199617 hypothetical protein	fig|6666666.59329.peg.2698	fig|6666666.59330.peg.3149	None	None	None	None	fig|6666666.59336.peg.3581	None	None	None	None	fig|6666666.59337.peg.1160	fig|6666666.59342.peg.5847	None	None	fig|6666666.59345.peg.3468	None	None	None	None	None	None	None	None	None	None

6772	Oligosaccharide translocase SypK	None	None	None	fig|6666666.59332.peg.3463	None	None	None	None	fig|6666666.59338.peg.2157	None	fig|6666666.59340.peg.2291	fig|6666666.59337.peg.4436	None	None	None	None	fig|6666666.59341.peg.506	None	fig|6666666.59346.peg.1739	None	None	None	None	None	None	None

8520	Uncharacterized paraquat-inducible protein B	None	None	None	None	None	fig|6666666.59335.peg.4280	None	None	fig|6666666.59338.peg.890	fig|6666666.59339.peg.2847	fig|6666666.59340.peg.3311	None	None	None	None	None	None	None	None	fig|6666666.59348.peg.3222	None	None	None	None	None	fig|6666666.59354.peg.2169

10623	Glutamate synthase [NADPH] small chain EC 1.4.1.13	None	None	None	fig|6666666.59332.peg.5204	None	None	None	fig|6666666.59333.peg.1512	fig|6666666.59338.peg.3180	None	None	None	None	None	None	None	fig|6666666.59341.peg.4349	None	fig|6666666.59346.peg.4185	None	None	fig|6666666.59350.peg.4864	None	None	None	None

10168	Malate synthase G EC 2.3.3.9	None	None	None	None	fig|6666666.59334.peg.4594	None	None	None	fig|6666666.59338.peg.3020	None	fig|6666666.59340.peg.3315	None	None	None	None	None	None	None	fig|6666666.59346.peg.3598	None	None	fig|6666666.59350.peg.2690	None	None	None	fig|6666666.59354.peg.216

5867	FIG01202733 hypothetical protein	fig|6666666.59329.peg.1504	fig|6666666.59330.peg.5175	None	None	None	None	fig|6666666.59336.peg.1128	None	fig|6666666.59338.peg.1802	None	None	None	None	None	None	fig|6666666.59345.peg.2986	None	None	None	fig|6666666.59348.peg.3323	None	None	None	None	None	None

6234	putative periplasmic protein	fig|6666666.59329.peg.3955	fig|6666666.59330.peg.4914	None	None	None	None	None	None	None	None	None	None	None	fig|6666666.71181.peg.4819	fig|6666666.59344.peg.2980	None	None	None	None	None	None	None	None	fig|6666666.59353.peg.3571	fig|6666666.59355.peg.3603	None

7932	FIG01200292 hypothetical protein	fig|6666666.59329.peg.5354	None	None	None	None	None	fig|6666666.59336.peg.1453	fig|6666666.59333.peg.839	None	None	None	fig|6666666.59337.peg.974	fig|6666666.59342.peg.2844	None	None	fig|6666666.59345.peg.2701	None	None	None	None	None	None	None	None	None	None

4577	Kef-type K+ transport system, predicted NAD-binding component	None	None	None	None	None	fig|6666666.59335.peg.1973	None	None	None	fig|6666666.59339.peg.6999	None	None	None	None	None	None	None	None	None	fig|6666666.59348.peg.5404	fig|6666666.59349.peg.2132	None	fig|6666666.59351.peg.2722	None	None	fig|6666666.59354.peg.1973

11132	L-2,4-diaminobutyric acid acetyltransferase EC 2.3.1.-	fig|6666666.59329.peg.2936	None	None	None	None	None	fig|6666666.59336.peg.3711	fig|6666666.59333.peg.4578	None	None	None	fig|6666666.59337.peg.1865	fig|6666666.59342.peg.418	None	None	fig|6666666.59345.peg.4190	None	None	None	None	None	None	None	None	None	None

9394	FIG00653109 hypothetical protein	None	None	fig|6666666.59331.peg.2458	fig|6666666.59332.peg.1398	None	None	None	None	fig|6666666.59338.peg.5676	None	None	None	None	None	None	None	fig|6666666.59341.peg.579	fig|6666666.59347.peg.2488	fig|6666666.59346.peg.4594	None	None	None	None	None	None	None

6433	FIG01199900 hypothetical protein	None	None	fig|6666666.59331.peg.3230	fig|6666666.59332.peg.3091	None	fig|6666666.59335.peg.3200	None	None	None	fig|6666666.59339.peg.1753	None	None	None	None	None	None	None	None	fig|6666666.59346.peg.882	fig|6666666.59348.peg.1618	None	None	None	None	None	None

9946	GTP-binding protein Era	None	None	fig|6666666.59331.peg.4031	None	None	None	fig|6666666.59336.peg.2097	None	None	None	None	None	None	None	None	None	fig|6666666.59341.peg.1332	None	None	None	fig|6666666.59349.peg.4320	fig|6666666.59350.peg.6233	fig|6666666.59351.peg.603	None	None	None

2145	Putative inner membrane protein	None	None	None	None	fig|6666666.59334.peg.3953	None	None	None	None	None	None	None	None	fig|6666666.71181.peg.3557	fig|6666666.59344.peg.5504	None	fig|6666666.59341.peg.1996	None	None	None	None	None	None	fig|6666666.59353.peg.4823	fig|6666666.59355.peg.4897	None

8051	hypothetical protein	fig|6666666.59329.peg.445	fig|6666666.59330.peg.1048	None	None	None	None	fig|6666666.59336.peg.1871	None	None	None	None	fig|6666666.59337.peg.1126	fig|6666666.59342.peg.3658	None	None	fig|6666666.59345.peg.3529	None	None	None	None	None	None	None	None	None	None

2887	Aminoglycoside phosphotransferase	None	None	None	None	None	None	None	None	None	None	None	None	None	fig|6666666.71181.peg.2751	None	None	None	None	None	fig|6666666.59348.peg.4005	fig|6666666.59349.peg.1443	None	None	fig|6666666.59353.peg.804	fig|6666666.59355.peg.902	fig|6666666.59354.peg.5767

11246	2-methylcitrate dehydratase FeS dependent EC 4.2.1.79	fig|6666666.59329.peg.1836	None	None	fig|6666666.59332.peg.3970	None	None	None	None	None	fig|6666666.59339.peg.4024	None	None	None	fig|6666666.71181.peg.4951	fig|6666666.59344.peg.482	None	None	None	None	None	None	None	None	None	None	fig|6666666.59354.peg.5983

5800	hypothetical protein	None	fig|6666666.59330.peg.2963	None	None	None	fig|6666666.59335.peg.3994	None	None	None	fig|6666666.59339.peg.866	None	fig|6666666.59337.peg.3440	fig|6666666.59342.peg.5418	None	None	fig|6666666.59345.peg.194	None	None	None	None	None	None	None	None	None	None

7965	FIG01205558 hypothetical protein	None	None	fig|6666666.59331.peg.1115	fig|6666666.59332.peg.5988	fig|6666666.59334.peg.4414	fig|6666666.59335.peg.3317	None	None	None	fig|6666666.59339.peg.881	None	None	None	None	None	None	None	None	fig|6666666.59346.peg.511	None	None	None	None	None	None	None

9177	FIG00919685 hypothetical protein	None	None	None	fig|6666666.59332.peg.973	None	fig|6666666.59335.peg.6164	fig|6666666.59336.peg.4567	None	None	fig|6666666.59339.peg.819	None	None	fig|6666666.59342.peg.2629	None	None	fig|6666666.59345.peg.656	None	None	None	None	None	None	None	None	None	None

3479	DNA or RNA helicase of superfamily II	None	fig|6666666.59330.peg.3692	fig|6666666.59331.peg.5873	fig|6666666.59332.peg.1652	fig|6666666.59334.peg.5373	None	None	None	None	fig|6666666.59339.peg.903	None	None	None	None	None	None	None	None	None	None	None	None	None	None	None	fig|6666666.59354.peg.166

9106	Predicted gluconate TRAP family transporter, DctP subunit	None	None	None	fig|6666666.59332.peg.5781	None	fig|6666666.59335.peg.6206	None	None	None	fig|6666666.59339.peg.4186	None	None	None	None	None	None	None	fig|6666666.59347.peg.4424	fig|6666666.59346.peg.5006	fig|6666666.59348.peg.2446	None	None	None	None	None	None

7776	FIG01199536 hypothetical protein	None	None	fig|6666666.59331.peg.5959	fig|6666666.59332.peg.6263	None	fig|6666666.59335.peg.581	None	None	None	fig|6666666.59339.peg.512	None	None	None	None	None	None	None	None	fig|6666666.59346.peg.4750	None	None	fig|6666666.59350.peg.1236	None	None	None	None

3918	FIG01199600 hypothetical protein	fig|6666666.59329.peg.1953	fig|6666666.59330.peg.4848	None	None	None	None	fig|6666666.59336.peg.5517	fig|6666666.59333.peg.5243	None	None	None	fig|6666666.59337.peg.3797	None	None	None	fig|6666666.59345.peg.1946	None	None	None	None	None	None	None	None	None	None

6734	L-ectoine synthase EC 4.2.1.-	fig|6666666.59329.peg.2938	None	None	None	None	None	fig|6666666.59336.peg.3713	fig|6666666.59333.peg.4580	None	None	None	fig|6666666.59337.peg.1867	fig|6666666.59342.peg.414	None	None	fig|6666666.59345.peg.4188	None	None	None	None	None	None	None	None	None	None

5019	Outer membrane receptor protein	fig|6666666.59329.peg.5781	None	None	None	None	None	None	None	None	None	fig|6666666.59340.peg.2335	None	None	None	None	None	fig|6666666.59341.peg.1870	fig|6666666.59347.peg.4897	fig|6666666.59346.peg.4985	fig|6666666.59348.peg.2343	None	None	None	None	None	None

10828	hypothetical protein	fig|6666666.59329.peg.892	fig|6666666.59330.peg.2394	None	None	None	None	fig|6666666.59336.peg.5105	None	None	None	None	fig|6666666.59337.peg.1494	fig|6666666.59342.peg.3953	None	None	fig|6666666.59345.peg.3004	None	None	None	None	None	None	None	None	None	None

9732	Copper resistance protein C precursor	None	None	None	None	None	None	None	None	None	None	None	None	None	fig|6666666.71181.peg.858	fig|6666666.59344.peg.17	None	None	None	None	None	None	fig|6666666.59350.peg.1689	fig|6666666.59351.peg.2526	fig|6666666.59353.peg.356	fig|6666666.59355.peg.1394	None

858	hypothetical protein	fig|6666666.59329.peg.3613	fig|6666666.59330.peg.3539	fig|6666666.59331.peg.136	None	None	None	fig|6666666.59336.peg.1070	None	None	None	None	None	fig|6666666.59342.peg.4777	None	None	None	None	None	fig|6666666.59346.peg.2870	None	None	None	None	None	None	None

6712	Protein chain release factor A	None	None	None	fig|6666666.59332.peg.1485	None	fig|6666666.59335.peg.6110	None	None	None	fig|6666666.59339.peg.3769	None	None	None	None	None	None	None	fig|6666666.59347.peg.2130	fig|6666666.59346.peg.3500	fig|6666666.59348.peg.4354	None	None	None	None	None	None

5036	Endolysin EC 3.2.1.-	None	None	None	None	None	fig|6666666.59335.peg.5059	None	None	None	None	None	None	None	fig|6666666.71181.peg.3974	fig|6666666.59344.peg.1970	None	None	None	fig|6666666.59346.peg.4135	None	None	None	None	fig|6666666.59353.peg.2947	fig|6666666.59355.peg.2792	None

5037	FIG01202968 hypothetical protein	fig|6666666.59329.peg.4966	fig|6666666.59330.peg.3662	None	None	None	None	fig|6666666.59336.peg.4530	None	None	None	None	fig|6666666.59337.peg.772	fig|6666666.59342.peg.5114	None	None	fig|6666666.59345.peg.3621	None	None	None	None	None	None	None	None	None	None

8928	Putative amino acid ABC transporter, permease protein	None	None	None	None	fig|6666666.59334.peg.2383	None	None	None	None	None	None	None	None	fig|6666666.71181.peg.897	fig|6666666.59344.peg.4779	None	fig|6666666.59341.peg.5830	None	None	None	None	None	None	fig|6666666.59353.peg.4533	fig|6666666.59355.peg.4663	None

1460	Na+ H+ antiporter NhaD type	fig|6666666.59329.peg.2366	None	fig|6666666.59331.peg.4467	None	None	None	None	None	None	fig|6666666.59339.peg.5645	None	None	fig|6666666.59342.peg.5830	None	None	None	None	None	None	fig|6666666.59348.peg.4716	None	None	None	None	None	fig|6666666.59354.peg.1375

11209	Dihydroxy-acid dehydratase EC 4.2.1.9	None	None	fig|6666666.59331.peg.1207	None	None	None	None	None	fig|6666666.59338.peg.2477	fig|6666666.59339.peg.8062	None	None	None	None	None	None	fig|6666666.59341.peg.2121	None	None	fig|6666666.59348.peg.72	None	None	fig|6666666.59351.peg.82	None	None	None

10120	FIG01055796 hypothetical protein	None	None	None	None	None	None	None	None	None	None	None	None	None	fig|6666666.71181.peg.1594	fig|6666666.59344.peg.2404	None	None	None	None	None	None	fig|6666666.59350.peg.5509	fig|6666666.59351.peg.2957	fig|6666666.59353.peg.287	fig|6666666.59355.peg.1327	None

7928	Coenzyme PQQ synthesis protein E	None	None	None	None	None	None	None	None	None	None	None	None	None	fig|6666666.71181.peg.1283	None	None	None	None	None	None	fig|6666666.59349.peg.59	None	fig|6666666.59351.peg.3017	fig|6666666.59353.peg.3316	fig|6666666.59355.peg.3136	fig|6666666.59354.peg.4474

7705	putative exported protein	None	None	None	None	fig|6666666.59334.peg.835	None	fig|6666666.59336.peg.754	None	fig|6666666.59338.peg.1608	None	None	None	None	None	None	fig|6666666.59345.peg.4449	fig|6666666.59341.peg.4335	fig|6666666.59347.peg.55	None	None	None	None	None	None	None	None

873	FIG01202941 hypothetical protein	fig|6666666.59329.peg.919	None	None	None	None	None	fig|6666666.59336.peg.400	fig|6666666.59333.peg.3020	None	None	None	fig|6666666.59337.peg.5212	fig|6666666.59342.peg.5467	None	None	fig|6666666.59345.peg.1831	None	None	None	None	None	None	None	None	None	None

4841	3-phytase precursor EC 3.1.3.8	None	None	None	None	None	None	None	None	None	None	None	None	None	fig|6666666.71181.peg.2198	fig|6666666.59344.peg.1166	None	None	None	None	None	None	fig|6666666.59350.peg.7570	fig|6666666.59351.peg.1830	fig|6666666.59353.peg.831	fig|6666666.59355.peg.873	None

3047	Dihydropteroate synthase EC 2.5.1.15	None	None	None	None	None	fig|6666666.59335.peg.2848	None	None	None	fig|6666666.59339.peg.4861	fig|6666666.59340.peg.1220	None	None	None	None	None	None	None	None	fig|6666666.59348.peg.1542	None	fig|6666666.59350.peg.2892	None	None	None	fig|6666666.59354.peg.1195

953	Unknown, probably involved in type III secretion	fig|6666666.59329.peg.6208	fig|6666666.59330.peg.3761	None	None	None	None	None	fig|6666666.59333.peg.1660	None	None	None	fig|6666666.59337.peg.4456	fig|6666666.59342.peg.4357	None	None	fig|6666666.59345.peg.1492	None	None	None	None	None	None	None	None	None	None

6334	FIG01201844 hypothetical protein	None	None	fig|6666666.59331.peg.3970	fig|6666666.59332.peg.1257	None	None	fig|6666666.59336.peg.1090	None	None	None	None	None	fig|6666666.59342.peg.3167	None	None	fig|6666666.59345.peg.2301	None	None	fig|6666666.59346.peg.3258	None	None	None	None	None	None	None

6338	hypothetical protein	None	None	None	fig|6666666.59332.peg.5525	fig|6666666.59334.peg.2829	None	None	None	fig|6666666.59338.peg.4028	None	None	None	None	None	None	None	fig|6666666.59341.peg.1831	fig|6666666.59347.peg.3595	fig|6666666.59346.peg.4125	None	None	None	None	None	None	None

6339	DNA-binding response regulator, AraC family	fig|6666666.59329.peg.146	None	fig|6666666.59331.peg.5224	None	None	fig|6666666.59335.peg.3600	None	None	fig|6666666.59338.peg.1959	fig|6666666.59339.peg.2986	None	None	None	None	None	None	None	None	fig|6666666.59346.peg.4280	None	None	None	None	None	None	None

9675	Glutamate decarboxylase EC 4.1.1.15	None	fig|6666666.59330.peg.1233	None	None	None	None	fig|6666666.59336.peg.1250	None	None	None	None	fig|6666666.59337.peg.3749	None	None	None	fig|6666666.59345.peg.1178	fig|6666666.59341.peg.816	fig|6666666.59347.peg.306	None	None	None	None	None	None	None	None

1663	hypothetical protein	None	fig|6666666.59330.peg.1203	None	None	None	None	fig|6666666.59336.peg.3176	fig|6666666.59333.peg.2469	None	None	None	fig|6666666.59337.peg.3139	fig|6666666.59342.peg.6423	None	None	fig|6666666.59345.peg.1153	None	None	None	None	None	None	None	None	None	None

5576	Polymyxin resistance protein PmrM	None	None	None	None	None	None	None	None	None	None	None	None	None	fig|6666666.71181.peg.2401	fig|6666666.59344.peg.786	None	fig|6666666.59341.peg.2053	fig|6666666.59347.peg.2345	None	None	None	None	None	fig|6666666.59353.peg.1321	fig|6666666.59355.peg.2550	None

3263	FIG01200472 hypothetical protein	None	None	None	fig|6666666.59332.peg.630	fig|6666666.59334.peg.4367	None	None	None	None	None	None	None	fig|6666666.59342.peg.102	None	None	fig|6666666.59345.peg.4385	None	fig|6666666.59347.peg.2894	fig|6666666.59346.peg.2374	None	None	None	None	None	None	None

5570	Hydroxymethylglutaryl-CoA reductase EC 1.1.1.34	fig|6666666.59329.peg.2040	None	None	None	None	None	fig|6666666.59336.peg.5912	fig|6666666.59333.peg.5059	None	fig|6666666.59339.peg.2825	None	fig|6666666.59337.peg.1204	fig|6666666.59342.peg.3296	None	None	None	None	None	None	None	None	None	None	None	None	None

5799	ATP synthase A chain EC 3.6.3.14	None	None	None	None	fig|6666666.59334.peg.2492	None	fig|6666666.59336.peg.5852	None	None	fig|6666666.59339.peg.2287	None	fig|6666666.59337.peg.2083	None	None	None	None	fig|6666666.59341.peg.3687	fig|6666666.59347.peg.2033	None	None	None	None	None	None	None	None

11113	Flagellar biosynthesis protein FlhB	None	None	None	None	None	fig|6666666.59335.peg.405	None	None	None	fig|6666666.59339.peg.1337	fig|6666666.59340.peg.1937	fig|6666666.59337.peg.5231	fig|6666666.59342.peg.2076	None	None	None	None	None	None	None	None	fig|6666666.59350.peg.5838	None	None	None	None

9442	Endo-beta-1,3-1,4 glucanase Licheninase EC 3.2.1.73	None	None	fig|6666666.59331.peg.1647	fig|6666666.59332.peg.5940	None	None	None	None	fig|6666666.59338.peg.2306	None	None	None	None	None	None	None	fig|6666666.59341.peg.2161	fig|6666666.59347.peg.5151	None	None	None	fig|6666666.59350.peg.2587	None	None	None	None

8370	hypothetical protein	fig|6666666.59329.peg.6467	fig|6666666.59330.peg.4807	None	None	None	None	None	None	None	None	None	fig|6666666.59337.peg.1180	fig|6666666.59342.peg.4271	None	None	fig|6666666.59345.peg.1	None	None	None	fig|6666666.59348.peg.2105	None	None	None	None	None	None

1026	FIG01201377 hypothetical protein	None	None	None	None	fig|6666666.59334.peg.4532	None	None	fig|6666666.59333.peg.4006	fig|6666666.59338.peg.2816	None	None	None	fig|6666666.59342.peg.2010	None	None	None	fig|6666666.59341.peg.809	fig|6666666.59347.peg.1650	None	None	None	None	None	None	None	None

2801	S-adenosylmethionine synthetase EC 2.5.1.6	None	None	None	fig|6666666.59332.peg.1932	None	fig|6666666.59335.peg.3100	None	None	None	fig|6666666.59339.peg.7843	fig|6666666.59340.peg.3052	None	None	None	None	None	None	None	None	None	None	None	fig|6666666.59351.peg.3638	None	None	fig|6666666.59354.peg.1957

2012	FIG01206455 hypothetical protein	fig|6666666.59329.peg.650	fig|6666666.59330.peg.1402	None	None	None	None	fig|6666666.59336.peg.5757	None	None	None	None	fig|6666666.59337.peg.726	fig|6666666.59342.peg.5287	None	None	fig|6666666.59345.peg.3663	None	None	None	None	None	None	None	None	None	None

11290	Malonyl CoA-acyl carrier protein transacylase EC 2.3.1.39	None	None	None	None	None	fig|6666666.59335.peg.687	None	None	None	fig|6666666.59339.peg.4091	None	None	None	None	None	None	None	None	None	None	fig|6666666.59349.peg.907	fig|6666666.59350.peg.5459	fig|6666666.59351.peg.2684	None	None	fig|6666666.59354.peg.5137

4804	FIG01201965 hypothetical protein	None	fig|6666666.59330.peg.3909	None	None	fig|6666666.59334.peg.5549	fig|6666666.59335.peg.3946	fig|6666666.59336.peg.5330	None	fig|6666666.59338.peg.862	None	None	None	None	None	None	None	None	None	fig|6666666.59346.peg.438	None	None	None	None	None	None	None

9795	FIG00613574 hypothetical protein	None	None	None	None	None	None	None	None	None	None	None	None	None	fig|6666666.71181.peg.3162	fig|6666666.59344.peg.2805	None	None	None	None	None	fig|6666666.59349.peg.2574	None	fig|6666666.59351.peg.5526	fig|6666666.59353.peg.1642	fig|6666666.59355.peg.1079	None

9794	Internalin-like protein LPXTG motif Lmo0331 homolog	None	fig|6666666.59330.peg.3058	None	None	None	None	fig|6666666.59336.peg.3223	None	None	None	None	fig|6666666.59337.peg.5367	None	None	None	fig|6666666.59345.peg.332	None	None	None	None	fig|6666666.59349.peg.3255	None	None	None	None	fig|6666666.59354.peg.3118

8998	transcriptional regulator, LysR family	None	None	fig|6666666.59331.peg.1868	None	None	None	None	None	None	None	None	None	None	fig|6666666.71181.peg.741	fig|6666666.59344.peg.719	None	None	None	fig|6666666.59346.peg.1429	None	None	None	None	fig|6666666.59353.peg.960	fig|6666666.59355.peg.2416	None

4025	FIG01202029 hypothetical protein	None	fig|6666666.59330.peg.2245	None	None	fig|6666666.59334.peg.4027	None	fig|6666666.59336.peg.1100	fig|6666666.59333.peg.4484	None	None	None	fig|6666666.59337.peg.5052	fig|6666666.59342.peg.1657	None	None	None	None	None	None	None	None	None	None	None	None	None

11385	hypothetical protein	None	None	None	None	None	None	None	None	None	fig|6666666.59339.peg.4097	None	None	None	fig|6666666.71181.peg.1887	fig|6666666.59344.peg.4363	None	None	None	None	None	fig|6666666.59349.peg.310	None	fig|6666666.59351.peg.2682	None	None	fig|6666666.59354.peg.1135

1198	Alpha-glucosidase EC 3.2.1.20	None	None	None	None	None	None	None	None	None	None	None	None	None	fig|6666666.71181.peg.1781	fig|6666666.59344.peg.5149	None	fig|6666666.59341.peg.3414	fig|6666666.59347.peg.4228	None	None	None	None	None	fig|6666666.59353.peg.2720	fig|6666666.59355.peg.1760	None

1195	para-nitrobenzyl esterase intracellular esterase B	None	None	None	None	fig|6666666.59334.peg.3687	None	None	fig|6666666.59333.peg.2267	fig|6666666.59338.peg.4769	None	None	fig|6666666.59337.peg.2668	fig|6666666.59342.peg.725	None	None	None	None	fig|6666666.59347.peg.1654	None	None	None	None	None	None	None	None

6575	Structural protein P5	fig|6666666.59329.peg.4088	None	None	None	None	fig|6666666.59335.peg.3080	None	None	None	None	fig|6666666.59340.peg.597	None	None	None	None	None	fig|6666666.59341.peg.988	fig|6666666.59347.peg.956	None	None	fig|6666666.59349.peg.86	None	None	None	None	None

3837	hypothetical protein	None	None	fig|6666666.59331.peg.5688	fig|6666666.59332.peg.2522	None	None	None	None	fig|6666666.59338.peg.5141	None	None	None	None	None	None	None	fig|6666666.59341.peg.4025	fig|6666666.59347.peg.1705	fig|6666666.59346.peg.4072	None	None	None	None	None	None	None

8852	DNA recombination protein RmuC	None	None	fig|6666666.59331.peg.6075	None	None	None	None	fig|6666666.59333.peg.2515	None	fig|6666666.59339.peg.7565	None	None	None	None	fig|6666666.59344.peg.3420	None	None	None	None	None	None	None	None	None	fig|6666666.59355.peg.3391	fig|6666666.59354.peg.226

6968	putative membrane protein	fig|6666666.59329.peg.3612	None	None	None	None	None	fig|6666666.59336.peg.3587	fig|6666666.59333.peg.3831	None	None	None	None	fig|6666666.59342.peg.6375	None	None	fig|6666666.59345.peg.2069	None	None	None	None	None	fig|6666666.59350.peg.8347	None	None	None	None

11360	Leucine-responsive regulatory protein, regulator for leucine or lrp regulon and high-affinity branched-chain amino acid transport system	None	None	None	None	None	fig|6666666.59335.peg.4723	None	None	None	fig|6666666.59339.peg.2161	None	None	None	fig|6666666.71181.peg.4105	fig|6666666.59344.peg.5352	None	None	None	None	None	None	None	None	fig|6666666.59353.peg.4743	fig|6666666.59355.peg.4701	None

1973	hypothetical protein	None	None	None	None	None	None	fig|6666666.59336.peg.994	None	None	None	None	None	None	None	None	fig|6666666.59345.peg.1447	fig|6666666.59341.peg.2917	None	None	None	fig|6666666.59349.peg.2904	None	fig|6666666.59351.peg.736	None	None	fig|6666666.59354.peg.3073

9631	Chromosome segregation ATPase	fig|6666666.59329.peg.638	fig|6666666.59330.peg.203	fig|6666666.59331.peg.6051	fig|6666666.59332.peg.48	None	None	None	None	fig|6666666.59338.peg.3485	None	None	fig|6666666.59337.peg.2790	None	None	None	None	None	None	None	None	None	None	None	None	None	None

538	Alkaline phosphatase EC 3.1.3.1 ; Type I phosphodiesterase nucleotide pyrophosphatase precursor	None	None	fig|6666666.59331.peg.1704	fig|6666666.59332.peg.5022	None	None	None	None	None	None	None	fig|6666666.59337.peg.5042	None	None	None	None	fig|6666666.59341.peg.1527	fig|6666666.59347.peg.747	fig|6666666.59346.peg.745	None	None	None	None	None	None	None

2216	Transcription-repair coupling factor	fig|6666666.59329.peg.4433	fig|6666666.59330.peg.1245	None	fig|6666666.59332.peg.2406	None	None	None	fig|6666666.59333.peg.1882	None	None	None	fig|6666666.59337.peg.2188	None	None	None	fig|6666666.59345.peg.1599	None	None	None	None	None	None	None	None	None	None

9425	Microcystin dependent protein	None	None	fig|6666666.59331.peg.2631	fig|6666666.59332.peg.1894	None	None	None	None	None	None	None	None	None	None	None	None	None	None	None	None	fig|6666666.59349.peg.3620	fig|6666666.59350.peg.7254	fig|6666666.59351.peg.5447	None	None	fig|6666666.59354.peg.4491

7615	FIG01060252 hypothetical protein	None	None	None	fig|6666666.59332.peg.4337	None	None	None	None	fig|6666666.59338.peg.3319	None	None	None	None	None	None	None	fig|6666666.59341.peg.6005	fig|6666666.59347.peg.3283	fig|6666666.59346.peg.4570	None	None	fig|6666666.59350.peg.1209	None	None	None	None

7047	ATP-dependent Clp protease ATP-binding subunit ClpA	fig|6666666.59329.peg.2571	None	None	None	None	fig|6666666.59335.peg.5998	None	None	fig|6666666.59338.peg.5086	fig|6666666.59339.peg.352	None	None	fig|6666666.59342.peg.115	None	None	None	None	None	None	fig|6666666.59348.peg.1388	None	None	None	None	None	None

2982	FIG00731820 hypothetical protein	fig|6666666.59329.peg.2381	None	None	None	None	fig|6666666.59335.peg.3386	None	None	None	fig|6666666.59339.peg.689	None	None	fig|6666666.59342.peg.6342	None	None	fig|6666666.59345.peg.1788	None	None	None	fig|6666666.59348.peg.2147	None	None	None	None	None	None

2987	Putative cytoplasmic protein	None	None	None	fig|6666666.59332.peg.773	None	None	None	None	None	None	None	None	None	fig|6666666.71181.peg.1011	fig|6666666.59344.peg.1789	None	None	None	None	None	None	None	None	fig|6666666.59353.peg.2329	fig|6666666.59355.peg.1602	fig|6666666.59354.peg.579

1010	FIG01204570 hypothetical protein	fig|6666666.59329.peg.1697	fig|6666666.59330.peg.806	None	None	None	None	fig|6666666.59336.peg.2913	fig|6666666.59333.peg.120	None	None	None	fig|6666666.59337.peg.1397	None	None	None	fig|6666666.59345.peg.3886	None	None	None	None	None	None	None	None	None	None

9497	Transcriptional regulator, LysR family	None	None	None	None	None	None	None	None	fig|6666666.59338.peg.1979	None	None	None	None	fig|6666666.71181.peg.1403	fig|6666666.59344.peg.511	None	None	fig|6666666.59347.peg.3461	None	None	None	None	None	fig|6666666.59353.peg.1824	fig|6666666.59355.peg.1192	None

10837	putative short-chain dehydrogenase	None	fig|6666666.59330.peg.2440	None	None	None	fig|6666666.59335.peg.5759	fig|6666666.59336.peg.493	fig|6666666.59333.peg.2983	None	None	None	fig|6666666.59337.peg.4783	None	None	None	fig|6666666.59345.peg.4829	None	None	None	None	None	None	None	None	None	None

3793	VapC toxin protein	None	None	None	None	None	None	None	None	None	fig|6666666.59339.peg.6051	None	None	None	fig|6666666.71181.peg.2240	fig|6666666.59344.peg.3023	None	None	None	None	fig|6666666.59348.peg.5359	None	None	None	fig|6666666.59353.peg.3537	fig|6666666.59355.peg.3638	None

1954	hypothetical protein	fig|6666666.59329.peg.1559	None	None	fig|6666666.59332.peg.4769	None	None	fig|6666666.59336.peg.416	None	fig|6666666.59338.peg.4746	None	None	None	None	None	None	fig|6666666.59345.peg.1023	None	fig|6666666.59347.peg.1113	None	None	None	None	None	None	None	None

1489	Protein-export membrane protein SecD TC 3.A.5.1.1	fig|6666666.59329.peg.2395	None	None	None	fig|6666666.59334.peg.761	None	None	None	fig|6666666.59338.peg.5283	None	fig|6666666.59340.peg.3620	None	fig|6666666.59342.peg.1457	None	None	None	fig|6666666.59341.peg.1912	None	None	None	None	None	None	None	None	None

2060	Chitobiose phosphorylase EC 2.4.1.-	None	fig|6666666.59330.peg.1774	None	None	fig|6666666.59334.peg.3728	None	None	None	fig|6666666.59338.peg.5122	fig|6666666.59339.peg.4805	fig|6666666.59340.peg.2960	fig|6666666.59337.peg.645	None	None	None	None	None	None	None	None	None	None	None	None	None	None

512	Isochorismatase EC 3.3.2.1	None	fig|6666666.59330.peg.4822	None	None	None	None	None	None	None	None	None	None	None	fig|6666666.71181.peg.1134	fig|6666666.59344.peg.2256	fig|6666666.59345.peg.804	None	None	None	None	None	None	None	fig|6666666.59353.peg.3446	fig|6666666.59355.peg.3547	None

1228	FIG01201903 hypothetical protein	fig|6666666.59329.peg.5490	fig|6666666.59330.peg.3852	None	None	None	None	fig|6666666.59336.peg.5142	fig|6666666.59333.peg.4595	None	None	None	fig|6666666.59337.peg.3861	None	None	None	fig|6666666.59345.peg.309	None	None	None	None	None	None	None	None	None	None

6536	RstA phage-related replication protein	fig|6666666.59329.peg.3597	fig|6666666.59330.peg.4714	fig|6666666.59331.peg.3791	None	None	None	None	None	fig|6666666.59338.peg.6107	None	None	None	fig|6666666.59342.peg.2621	None	None	None	fig|6666666.59341.peg.5484	None	None	None	None	None	None	None	None	None

6533	AmpE protein	None	None	None	None	None	None	None	None	None	None	None	None	None	fig|6666666.71181.peg.1960	fig|6666666.59344.peg.1453	None	None	None	None	None	None	None	fig|6666666.59351.peg.281	fig|6666666.59353.peg.159	fig|6666666.59355.peg.770	fig|6666666.59354.peg.6334

6531	hypothetical protein	None	None	fig|6666666.59331.peg.1492	fig|6666666.59332.peg.2809	None	None	None	None	fig|6666666.59338.peg.2369	None	fig|6666666.59340.peg.906	None	None	None	None	None	fig|6666666.59341.peg.4244	fig|6666666.59347.peg.4404	None	None	None	None	None	None	None	None

8796	FIG01200372 hypothetical protein	None	None	fig|6666666.59331.peg.680	fig|6666666.59332.peg.965	None	fig|6666666.59335.peg.5786	None	None	None	fig|6666666.59339.peg.5644	None	None	None	None	None	None	None	None	fig|6666666.59346.peg.2587	fig|6666666.59348.peg.4717	None	None	None	None	None	None

3877	FIG01200909 hypothetical protein	fig|6666666.59329.peg.4912	fig|6666666.59330.peg.867	None	None	None	None	None	fig|6666666.59333.peg.60	None	None	None	fig|6666666.59337.peg.3287	fig|6666666.59342.peg.3550	None	None	fig|6666666.59345.peg.550	None	None	None	None	None	None	None	None	None	None

5939	Phosphoethanolamine transferase specific for the outer Kdo residue of lipopolysaccharide	None	None	None	None	None	None	None	None	None	None	None	None	None	fig|6666666.71181.peg.365	fig|6666666.59344.peg.2369	None	fig|6666666.59341.peg.4954	fig|6666666.59347.peg.5196	None	None	None	None	None	fig|6666666.59353.peg.3613	fig|6666666.59355.peg.1299	None

3498	Chromosome segregation ATPase	None	None	None	None	None	fig|6666666.59335.peg.74	None	None	None	fig|6666666.59339.peg.7629	None	None	None	None	None	None	None	None	None	fig|6666666.59348.peg.629	fig|6666666.59349.peg.3569	None	fig|6666666.59351.peg.2421	None	None	fig|6666666.59354.peg.4615

7360	hypothetical protein	None	None	fig|6666666.59331.peg.2403	fig|6666666.59332.peg.2081	None	None	None	None	None	None	None	None	None	None	None	None	None	None	fig|6666666.59346.peg.3368	None	fig|6666666.59349.peg.3293	None	fig|6666666.59351.peg.4228	None	None	fig|6666666.59354.peg.3244

5725	Transcriptional regulator, HxlR family	None	None	None	fig|6666666.59332.peg.6104	None	fig|6666666.59335.peg.3130	None	None	None	fig|6666666.59339.peg.5425	None	None	None	None	None	None	None	None	fig|6666666.59346.peg.496	fig|6666666.59348.peg.4263	None	None	fig|6666666.59351.peg.4727	None	None	None

6649	bifunctional outer membrane translocase extracellular lipase, PlpD	None	fig|6666666.59330.peg.1763	None	None	None	None	None	fig|6666666.59333.peg.320	fig|6666666.59338.peg.1975	fig|6666666.59339.peg.4783	None	fig|6666666.59337.peg.631	None	None	None	None	None	None	None	fig|6666666.59348.peg.2606	None	None	None	None	None	None

10812	FIG01202349 hypothetical protein	None	None	None	None	fig|6666666.59334.peg.3799	None	fig|6666666.59336.peg.3830	fig|6666666.59333.peg.2707	None	None	None	fig|6666666.59337.peg.2021	fig|6666666.59342.peg.4714	None	None	fig|6666666.59345.peg.2455	None	None	None	None	None	None	None	None	None	None

1856	FIG01200721 hypothetical protein	fig|6666666.59329.peg.6207	fig|6666666.59330.peg.3762	None	None	None	None	None	fig|6666666.59333.peg.1659	None	None	None	fig|6666666.59337.peg.4455	fig|6666666.59342.peg.4354	None	None	fig|6666666.59345.peg.1493	None	None	None	None	None	None	None	None	None	None

5694	FUPA27 P-type ATPase	None	None	fig|6666666.59331.peg.2452	None	fig|6666666.59334.peg.989	None	None	None	None	fig|6666666.59339.peg.5278	None	None	None	fig|6666666.71181.peg.6244	None	None	fig|6666666.59341.peg.5610	None	None	fig|6666666.59348.peg.5083	None	None	None	None	None	None

7945	hypothetical protein	fig|6666666.59329.peg.2299	None	None	fig|6666666.59332.peg.3874	None	fig|6666666.59335.peg.4158	None	fig|6666666.59333.peg.2574	None	None	None	None	fig|6666666.59342.peg.2004	None	None	fig|6666666.59345.peg.5151	None	None	None	None	None	None	None	None	None	None

8278	Prevent host death protein, Phd antitoxin	fig|6666666.59329.peg.2826	fig|6666666.59330.peg.4823	None	None	None	None	None	fig|6666666.59333.peg.5576	None	None	None	fig|6666666.59337.peg.5706	None	None	None	None	None	fig|6666666.59347.peg.5322	fig|6666666.59346.peg.4140	None	None	None	None	None	None	None

8101	FIG01202273 hypothetical protein	None	fig|6666666.59330.peg.3276	fig|6666666.59331.peg.2707	None	None	None	None	None	None	None	None	fig|6666666.59337.peg.2812	fig|6666666.59342.peg.5048	None	None	fig|6666666.59345.peg.1450	None	None	fig|6666666.59346.peg.3184	None	None	None	None	None	None	None

10773	hypothetical protein	None	fig|6666666.59330.peg.4426	None	fig|6666666.59332.peg.2594	None	fig|6666666.59335.peg.5530	None	fig|6666666.59333.peg.3139	None	fig|6666666.59339.peg.3825	None	fig|6666666.59337.peg.5034	None	None	None	None	None	None	None	None	None	None	None	None	None	None

8417	SSU ribosomal protein S2p SAe	None	None	None	None	None	None	None	None	None	fig|6666666.59339.peg.5375	None	None	None	fig|6666666.71181.peg.1010	fig|6666666.59344.peg.5848	None	None	None	None	fig|6666666.59348.peg.325	None	None	None	fig|6666666.59353.peg.2473	fig|6666666.59355.peg.1601	None

6952	CRISPR-associated protein, Csy3 family	None	fig|6666666.59330.peg.3732	None	None	fig|6666666.59334.peg.707	None	None	None	None	None	None	fig|6666666.59337.peg.4207	fig|6666666.59342.peg.24	None	None	fig|6666666.59345.peg.1637	None	None	None	None	fig|6666666.59349.peg.4905	None	None	None	None	None

4144	Serine--pyruvate aminotransferase EC 2.6.1.51 L-alanine glyoxylate aminotransferase EC 2.6.1.44	fig|6666666.59329.peg.6047	None	None	None	None	None	None	None	None	None	None	None	None	None	fig|6666666.59344.peg.4782	None	None	fig|6666666.59347.peg.3265	fig|6666666.59346.peg.1593	None	None	None	None	fig|6666666.59353.peg.4530	fig|6666666.59355.peg.4666	None

10025	Chaperone protein YscY Yop proteins translocation protein Y	fig|6666666.59329.peg.527	fig|6666666.59330.peg.3287	None	None	None	None	None	fig|6666666.59333.peg.3361	None	None	None	fig|6666666.59337.peg.5244	fig|6666666.59342.peg.1913	None	None	fig|6666666.59345.peg.1458	None	None	None	None	None	None	None	None	None	None

7349	Phosphonoacetaldehyde hydrolase EC 3.11.1.1	None	None	None	None	None	None	None	fig|6666666.59333.peg.2788	None	None	None	fig|6666666.59337.peg.5036	fig|6666666.59342.peg.6281	None	fig|6666666.59344.peg.835	None	None	None	None	None	None	None	None	fig|6666666.59353.peg.1278	fig|6666666.59355.peg.3305	None

11047	Uncharacterized paraquat-inducible protein B	fig|6666666.59329.peg.6507	None	None	fig|6666666.59332.peg.5636	None	None	None	fig|6666666.59333.peg.2337	None	fig|6666666.59339.peg.2845	None	None	fig|6666666.59342.peg.719	None	None	None	None	None	None	None	None	None	None	None	None	fig|6666666.59354.peg.2171

8645	FIG01201930 hypothetical protein	fig|6666666.59329.peg.5491	fig|6666666.59330.peg.3851	None	None	None	None	fig|6666666.59336.peg.5143	fig|6666666.59333.peg.4592	None	None	None	fig|6666666.59337.peg.4810	None	None	None	fig|6666666.59345.peg.310	None	None	None	None	None	None	None	None	None	None

10873	hypothetical protein	fig|6666666.59329.peg.4861	None	fig|6666666.59331.peg.5241	fig|6666666.59332.peg.2129	None	fig|6666666.59335.peg.1316	None	None	None	fig|6666666.59339.peg.3451	None	None	None	None	None	None	None	None	fig|6666666.59346.peg.1882	None	None	None	None	None	None	None

4940	Flp pilus assembly protein TadD, contains TPR repeat	fig|6666666.59329.peg.3654	fig|6666666.59330.peg.668	None	None	None	fig|6666666.59335.peg.1603	None	None	fig|6666666.59338.peg.1795	None	None	fig|6666666.59337.peg.5178	None	None	None	None	fig|6666666.59341.peg.4593	None	None	None	None	None	None	None	None	None

5108	ATP-binding protein PhnN; Guanylate kinase EC 2.7.4.8	None	None	None	None	None	None	None	None	None	fig|6666666.59339.peg.7025	None	None	None	fig|6666666.71181.peg.3527	None	None	fig|6666666.59341.peg.4330	fig|6666666.59347.peg.101	None	None	None	fig|6666666.59350.peg.4813	None	None	fig|6666666.59355.peg.492	None

10491	Polymyxin resistance protein PmrL, sucrose-6 phosphate hydrolase	None	None	None	None	None	None	None	None	None	None	None	None	None	fig|6666666.71181.peg.2402	fig|6666666.59344.peg.787	None	fig|6666666.59341.peg.2055	fig|6666666.59347.peg.2346	None	None	None	None	None	fig|6666666.59353.peg.1320	fig|6666666.59355.peg.2551	None

669	DNA transposition protein	None	None	None	None	None	None	None	None	None	None	None	None	None	fig|6666666.71181.peg.2859	fig|6666666.59344.peg.4404	None	None	None	None	None	fig|6666666.59349.peg.1220	None	None	fig|6666666.59353.peg.4316	fig|6666666.59355.peg.4386	fig|6666666.59354.peg.5804

668	Multi antimicrobial extrusion protein Na + drug antiporter , MATE family of MDR efflux pumps	fig|6666666.59329.peg.3905	None	None	None	fig|6666666.59334.peg.2591	None	fig|6666666.59336.peg.644	fig|6666666.59333.peg.2658	None	None	fig|6666666.59340.peg.200	None	fig|6666666.59342.peg.4029	None	None	None	None	None	None	None	None	None	None	None	None	None

664	hypothetical protein	fig|6666666.59329.peg.1561	None	None	None	None	None	fig|6666666.59336.peg.414	None	fig|6666666.59338.peg.138	None	None	None	None	None	None	fig|6666666.59345.peg.1021	fig|6666666.59341.peg.3583	fig|6666666.59347.peg.224	None	None	None	None	None	None	None	None

4129	DNA-directed RNA polymerase beta' subunit EC 2.7.7.6	fig|6666666.59329.peg.628	None	None	None	None	None	fig|6666666.59336.peg.1685	None	None	fig|6666666.59339.peg.8836	fig|6666666.59340.peg.731	None	None	None	None	None	fig|6666666.59341.peg.3795	None	None	fig|6666666.59348.peg.1009	None	None	None	None	None	None

2583	FIG01201447 hypothetical protein	fig|6666666.59329.peg.3901	fig|6666666.59330.peg.1486	None	None	None	None	fig|6666666.59336.peg.5137	fig|6666666.59333.peg.1729	None	None	None	fig|6666666.59337.peg.189	None	None	None	fig|6666666.59345.peg.4519	None	None	None	None	None	None	None	None	None	None

9047	Na + H + antiporter subunit D	None	None	fig|6666666.59331.peg.5009	None	fig|6666666.59334.peg.5146	None	None	None	fig|6666666.59338.peg.4614	None	fig|6666666.59340.peg.3194	None	fig|6666666.59342.peg.4550	None	None	None	None	None	None	None	None	fig|6666666.59350.peg.7269	None	None	None	None

3540	Transcription termination protein NusA	fig|6666666.59329.peg.3443	None	fig|6666666.59331.peg.2124	None	fig|6666666.59334.peg.781	fig|6666666.59335.peg.2102	None	None	None	fig|6666666.59339.peg.4893	None	None	None	None	None	None	fig|6666666.59341.peg.2439	None	None	None	None	None	None	None	None	None

3542	TRAP transporter solute receptor, unknown substrate 8	None	None	None	fig|6666666.59332.peg.3412	fig|6666666.59334.peg.3665	fig|6666666.59335.peg.5273	None	None	None	fig|6666666.59339.peg.5728	None	None	None	None	None	None	None	fig|6666666.59347.peg.61	fig|6666666.59346.peg.1426	None	None	None	None	None	None	None

764	Putative membrane protein	fig|6666666.59329.peg.275	None	None	fig|6666666.59332.peg.5396	None	None	None	None	fig|6666666.59338.peg.1423	None	fig|6666666.59340.peg.1184	fig|6666666.59337.peg.3393	fig|6666666.59342.peg.347	None	None	None	None	None	None	None	None	None	None	None	None	None

4335	hypothetical protein	None	None	None	None	None	fig|6666666.59335.peg.6074	None	None	None	None	fig|6666666.59340.peg.314	None	None	None	None	None	None	fig|6666666.59347.peg.4888	None	fig|6666666.59348.peg.760	fig|6666666.59349.peg.72	None	None	None	None	fig|6666666.59354.peg.3291

3137	FIG01199872 hypothetical protein	None	None	None	None	None	None	fig|6666666.59336.peg.5222	None	None	None	None	fig|6666666.59337.peg.2766	fig|6666666.59342.peg.4724	None	None	fig|6666666.59345.peg.4287	None	None	fig|6666666.59346.peg.707	fig|6666666.59348.peg.3233	None	None	None	None	None	None

3130	Holliday junction DNA helicase RuvB	None	None	fig|6666666.59331.peg.5225	fig|6666666.59332.peg.1962	None	None	None	fig|6666666.59333.peg.1980	None	None	None	None	fig|6666666.59342.peg.4141	None	fig|6666666.59344.peg.2828	None	None	None	None	None	None	None	None	None	None	fig|6666666.59354.peg.1593

11263	hypothetical protein	fig|6666666.59329.peg.4089	None	None	None	None	fig|6666666.59335.peg.272	None	None	None	fig|6666666.59339.peg.1468	fig|6666666.59340.peg.598	None	None	None	None	None	fig|6666666.59341.peg.989	fig|6666666.59347.peg.957	None	None	None	None	None	None	None	None

5366	hypothetical protein	None	None	None	None	None	fig|6666666.59335.peg.5058	None	None	None	fig|6666666.59339.peg.1482	None	None	None	None	None	None	None	None	fig|6666666.59346.peg.4136	fig|6666666.59348.peg.1738	fig|6666666.59349.peg.1248	None	None	None	None	fig|6666666.59354.peg.2735

2358	FIG01201294 hypothetical protein	fig|6666666.59329.peg.1383	fig|6666666.59330.peg.2520	None	None	None	None	fig|6666666.59336.peg.6080	None	None	None	None	fig|6666666.59337.peg.141	fig|6666666.59342.peg.2143	None	None	fig|6666666.59345.peg.3701	None	None	None	None	None	None	None	None	None	None

2783	Phosphopentomutase EC 5.4.2.7	None	None	None	None	fig|6666666.59334.peg.493	fig|6666666.59335.peg.3642	None	None	None	fig|6666666.59339.peg.5020	None	None	fig|6666666.59342.peg.1788	None	fig|6666666.59344.peg.1372	None	None	None	None	None	None	fig|6666666.59350.peg.5245	None	None	None	None

9698	hypothetical protein	fig|6666666.59329.peg.6446	fig|6666666.59330.peg.3899	None	None	None	None	fig|6666666.59336.peg.5404	None	None	None	None	fig|6666666.59337.peg.4701	fig|6666666.59342.peg.4791	None	None	fig|6666666.59345.peg.3103	None	None	None	None	None	None	None	None	None	None

6598	Phage integrase	fig|6666666.59329.peg.5998	None	None	fig|6666666.59332.peg.6110	None	None	None	None	fig|6666666.59338.peg.520	None	fig|6666666.59340.peg.1414	fig|6666666.59337.peg.3391	fig|6666666.59342.peg.348	None	None	None	None	None	None	None	None	None	None	None	None	None

1530	Ferrochelatase, protoheme ferro-lyase EC 4.99.1.1	None	None	None	None	None	None	None	None	None	fig|6666666.59339.peg.5271	fig|6666666.59340.peg.2605	None	None	None	None	None	fig|6666666.59341.peg.513	None	fig|6666666.59346.peg.5166	fig|6666666.59348.peg.3706	None	None	None	None	None	fig|6666666.59354.peg.5005

3099	Exopolyphosphatase-related protein	None	None	fig|6666666.59331.peg.3485	fig|6666666.59332.peg.1749	None	fig|6666666.59335.peg.1067	None	None	None	fig|6666666.59339.peg.4516	None	None	None	None	None	None	None	None	fig|6666666.59346.peg.2816	fig|6666666.59348.peg.914	None	None	None	None	None	None

1991	RelE StbE replicon stabilization toxin	fig|6666666.59329.peg.5111	fig|6666666.59330.peg.4828	fig|6666666.59331.peg.3994	None	None	None	None	None	fig|6666666.59338.peg.5970	None	None	None	None	None	None	fig|6666666.59345.peg.789	None	None	fig|6666666.59346.peg.2876	None	None	None	None	None	None	None

3157	CTP molybdopterin cytidylyltransferase	None	None	fig|6666666.59331.peg.5709	None	None	None	None	None	None	None	None	None	None	fig|6666666.71181.peg.1855	fig|6666666.59344.peg.4327	None	None	None	None	None	None	fig|6666666.59350.peg.5455	None	fig|6666666.59353.peg.3220	fig|6666666.59355.peg.3102	None

10649	Methionine ABC transporter substrate-binding protein	None	None	None	None	fig|6666666.59334.peg.3419	None	None	None	None	fig|6666666.59339.peg.5166	None	None	None	fig|6666666.71181.peg.1174	fig|6666666.59344.peg.2303	None	None	None	None	None	None	None	None	fig|6666666.59353.peg.3409	fig|6666666.59355.peg.3507	None

6624	FIG00919535 hypothetical protein	fig|6666666.59329.peg.1188	fig|6666666.59330.peg.1994	None	None	None	None	fig|6666666.59336.peg.6267	None	None	fig|6666666.59339.peg.3531	None	fig|6666666.59337.peg.4648	None	None	None	None	None	None	fig|6666666.59346.peg.5334	None	None	None	None	None	None	None

10740	hypothetical protein	None	None	fig|6666666.59331.peg.5144	fig|6666666.59332.peg.5570	None	None	None	None	None	None	None	None	fig|6666666.59342.peg.1093	None	None	fig|6666666.59345.peg.67	None	fig|6666666.59347.peg.1835	fig|6666666.59346.peg.3613	None	None	None	None	None	None	None

4710	hypothetical protein	fig|6666666.59329.peg.2813	fig|6666666.59330.peg.2044	None	None	fig|6666666.59334.peg.631	None	None	fig|6666666.59333.peg.5303	None	None	None	None	None	None	None	fig|6666666.59345.peg.3905	None	None	None	fig|6666666.59348.peg.1424	None	None	None	None	None	None

7241	Type III secretion protein YscE	fig|6666666.59329.peg.2728	fig|6666666.59330.peg.3752	None	None	None	None	None	fig|6666666.59333.peg.2992	None	None	None	fig|6666666.59337.peg.4464	fig|6666666.59342.peg.626	None	None	fig|6666666.59345.peg.1485	None	None	None	None	None	None	None	None	None	None

10406	Cs C3339	fig|6666666.59329.peg.4453	fig|6666666.59330.peg.2253	None	None	None	None	fig|6666666.59336.peg.2609	fig|6666666.59333.peg.1671	None	None	None	fig|6666666.59337.peg.4359	None	None	None	fig|6666666.59345.peg.1903	None	None	None	None	None	None	None	None	None	None

262	Bacterial extracellular solute-binding protein, family 3	None	None	None	None	None	fig|6666666.59335.peg.2249	None	None	None	fig|6666666.59339.peg.850	None	None	None	None	None	None	None	None	None	fig|6666666.59348.peg.4944	fig|6666666.59349.peg.2201	None	fig|6666666.59351.peg.4191	None	None	fig|6666666.59354.peg.5835

6016	OmpA-like transmembrane domain protein	fig|6666666.59329.peg.5990	fig|6666666.59330.peg.2553	None	None	None	None	fig|6666666.59336.peg.2147	fig|6666666.59333.peg.2476	None	None	None	fig|6666666.59337.peg.2255	None	None	None	fig|6666666.59345.peg.2950	None	None	None	None	None	None	None	None	None	None

5084	Ubiquinol--cytochrome c reductase, cytochrome B subunit EC 1.10.2.2	None	None	None	None	fig|6666666.59334.peg.3191	None	fig|6666666.59336.peg.1590	None	None	fig|6666666.59339.peg.4942	None	None	fig|6666666.59342.peg.4826	None	None	None	None	None	None	fig|6666666.59348.peg.1447	None	None	None	None	None	fig|6666666.59354.peg.359

2673	putative esterase	None	None	None	None	None	None	None	None	None	fig|6666666.59339.peg.1255	None	None	None	fig|6666666.71181.peg.4698	fig|6666666.59344.peg.4556	None	None	None	None	None	None	fig|6666666.59350.peg.3101	None	fig|6666666.59353.peg.2764	fig|6666666.59355.peg.3452	None

3170	FIG01055585 hypothetical protein	None	None	None	None	None	None	None	None	None	None	None	None	None	fig|6666666.71181.peg.1279	fig|6666666.59344.peg.119	None	fig|6666666.59341.peg.4087	fig|6666666.59347.peg.1588	None	None	None	None	None	fig|6666666.59353.peg.439	fig|6666666.59355.peg.2166	None

1169	PTS system, glucitol sorbitol-specific IIC component EC 2.7.1.69	None	None	None	None	None	None	None	None	fig|6666666.59338.peg.6027	None	None	None	None	fig|6666666.71181.peg.4226	fig|6666666.59344.peg.6067	None	fig|6666666.59341.peg.5555	None	None	None	None	None	None	fig|6666666.59353.peg.2751	fig|6666666.59355.peg.1731	None

1381	D-alanyl-D-alanine dipeptidase EC 3.4.13.- ; Putative metaloprotease associated with hemin utilization	None	None	None	None	fig|6666666.59334.peg.2141	None	None	None	None	None	None	None	None	fig|6666666.71181.peg.2336	fig|6666666.59344.peg.4529	None	None	fig|6666666.59347.peg.735	None	None	None	None	None	fig|6666666.59353.peg.3319	fig|6666666.59355.peg.3475	None

8002	FIG01200071 hypothetical protein	None	None	None	None	fig|6666666.59334.peg.3304	None	fig|6666666.59336.peg.464	None	fig|6666666.59338.peg.2140	fig|6666666.59339.peg.3495	None	None	fig|6666666.59342.peg.1089	None	None	None	None	None	fig|6666666.59346.peg.3239	None	None	None	None	None	None	None

10892	Indole-3-glycerol phosphate synthase EC 4.1.1.48 Phosphoribosylanthranilate isomerase EC 5.3.1.24	None	None	None	fig|6666666.59332.peg.6276	None	fig|6666666.59335.peg.5381	None	None	None	fig|6666666.59339.peg.3634	None	None	None	None	fig|6666666.59344.peg.457	None	None	None	None	fig|6666666.59348.peg.1831	None	None	None	None	None	fig|6666666.59354.peg.5222

2317	FIG01201547 hypothetical protein	None	None	fig|6666666.59331.peg.5695	fig|6666666.59332.peg.5336	None	None	None	None	None	None	None	None	None	None	None	None	None	None	fig|6666666.59346.peg.1275	None	fig|6666666.59349.peg.410	None	fig|6666666.59351.peg.4795	None	None	fig|6666666.59354.peg.1248

108	DNA gyrase inhibitory protein	None	None	None	None	None	None	None	fig|6666666.59333.peg.4669	None	fig|6666666.59339.peg.1679	None	None	None	fig|6666666.71181.peg.2285	fig|6666666.59344.peg.4690	None	None	None	None	None	None	None	None	fig|6666666.59353.peg.4496	fig|6666666.59355.peg.4604	None

109	Leucine-rich repeat protein	fig|6666666.59329.peg.3972	None	None	fig|6666666.59332.peg.995	None	None	fig|6666666.59336.peg.4897	None	None	fig|6666666.59339.peg.2488	None	fig|6666666.59337.peg.2446	None	None	None	None	fig|6666666.59341.peg.5509	None	None	None	None	None	None	None	None	None

684	hypothetical protein	fig|6666666.59329.peg.3725	None	None	fig|6666666.59332.peg.456	None	None	fig|6666666.59336.peg.5603	None	fig|6666666.59338.peg.4389	None	None	None	fig|6666666.59342.peg.6302	None	None	fig|6666666.59345.peg.205	None	None	None	None	None	None	None	None	None	None

5992	P pilus assembly protein, chaperone PapD	fig|6666666.59329.peg.1573	None	None	None	fig|6666666.59334.peg.1264	None	fig|6666666.59336.peg.691	None	None	None	None	fig|6666666.59337.peg.1317	None	None	None	fig|6666666.59345.peg.2323	None	None	fig|6666666.59346.peg.1790	None	None	None	None	None	None	None

1363	Primosomal replication protein N	None	None	None	None	fig|6666666.59334.peg.170	None	None	None	None	None	None	None	fig|6666666.59342.peg.1892	None	None	None	None	None	None	fig|6666666.59348.peg.4967	fig|6666666.59349.peg.3229	None	fig|6666666.59351.peg.5423	None	None	fig|6666666.59354.peg.2013

8022	Agglutination protein	fig|6666666.59329.peg.3030	None	None	None	None	fig|6666666.59335.peg.1334	fig|6666666.59336.peg.5648	fig|6666666.59333.peg.3565	None	fig|6666666.59339.peg.926	None	None	None	None	None	fig|6666666.59345.peg.3286	None	None	None	None	None	None	None	None	None	None

8021	Formate efflux transporter TC 2.A.44 family	None	None	fig|6666666.59331.peg.1981	None	fig|6666666.59334.peg.3582	None	fig|6666666.59336.peg.2595	fig|6666666.59333.peg.4472	None	fig|6666666.59339.peg.2879	None	fig|6666666.59337.peg.2517	None	None	None	None	None	None	None	None	None	None	None	None	None	None

4282	Mobile element protein	None	fig|6666666.59330.peg.4690	None	fig|6666666.59332.peg.4676	None	None	None	None	None	None	None	None	fig|6666666.59342.peg.901	None	None	None	None	None	fig|6666666.59346.peg.5042	None	None	fig|6666666.59350.peg.8345	fig|6666666.59351.peg.1159	None	None	None

3753	Type III secretion cytoplasmic protein YscI	fig|6666666.59329.peg.2732	fig|6666666.59330.peg.4698	None	None	None	None	None	fig|6666666.59333.peg.2997	None	None	None	fig|6666666.59337.peg.4468	fig|6666666.59342.peg.631	None	None	fig|6666666.59345.peg.1481	None	None	None	None	None	None	None	None	None	None

6194	FIG01199609 hypothetical protein	fig|6666666.59329.peg.2186	fig|6666666.59330.peg.3238	None	None	None	None	fig|6666666.59336.peg.1667	None	None	None	None	fig|6666666.59337.peg.2149	fig|6666666.59342.peg.4668	None	None	fig|6666666.59345.peg.3961	None	None	None	None	None	None	None	None	None	None

6192	Histone deacetylase AcuC AphA family protein	None	None	None	None	fig|6666666.59334.peg.5491	None	None	fig|6666666.59333.peg.2467	None	None	None	None	fig|6666666.59342.peg.3143	None	None	None	fig|6666666.59341.peg.1120	None	None	fig|6666666.59348.peg.1318	None	None	None	None	None	fig|6666666.59354.peg.5735

6199	Citrate lyase alpha chain EC 4.1.3.6	fig|6666666.59329.peg.1724	None	None	None	fig|6666666.59334.peg.1767	None	None	None	None	None	None	None	None	fig|6666666.71181.peg.1727	fig|6666666.59344.peg.6209	None	None	None	None	None	None	None	None	fig|6666666.59353.peg.3687	fig|6666666.59355.peg.3010	None

10367	L-proline glycine betaine ABC transport system permease protein ProV TC 3.A.1.12.1	None	None	None	None	None	None	None	None	None	None	None	None	None	fig|6666666.71181.peg.237	fig|6666666.59344.peg.292	None	fig|6666666.59341.peg.2741	None	None	None	None	fig|6666666.59350.peg.5072	None	fig|6666666.59353.peg.584	fig|6666666.59355.peg.67	None

804	Putative oxidoreductase linked to yggC	fig|6666666.59329.peg.5467	None	None	None	None	None	fig|6666666.59336.peg.4604	None	None	None	None	fig|6666666.59337.peg.1943	fig|6666666.59342.peg.4950	None	None	fig|6666666.59345.peg.4047	fig|6666666.59341.peg.4101	None	None	None	None	None	None	None	None	None

9976	FIG01200565 hypothetical protein	fig|6666666.59329.peg.6556	fig|6666666.59330.peg.1205	None	None	None	None	fig|6666666.59336.peg.3178	fig|6666666.59333.peg.2472	None	None	None	fig|6666666.59337.peg.3137	None	None	None	fig|6666666.59345.peg.1155	None	None	None	None	None	None	None	None	None	None

9512	tRNA Ile -lysidine synthetase	None	None	fig|6666666.59331.peg.4956	None	None	None	None	fig|6666666.59333.peg.1151	fig|6666666.59338.peg.2439	None	None	None	None	None	fig|6666666.59344.peg.2674	None	None	None	None	None	None	None	None	fig|6666666.59353.peg.2004	fig|6666666.59355.peg.4109	None

1255	Co-activator of prophage gene expression IbrB	None	None	None	None	None	fig|6666666.59335.peg.4234	fig|6666666.59336.peg.5594	None	None	fig|6666666.59339.peg.1023	None	None	None	None	None	fig|6666666.59345.peg.263	None	None	fig|6666666.59346.peg.845	fig|6666666.59348.peg.4763	None	None	None	None	None	None

1126	FIG01200941 hypothetical protein	fig|6666666.59329.peg.18	fig|6666666.59330.peg.2721	None	None	None	fig|6666666.59335.peg.475	fig|6666666.59336.peg.274	fig|6666666.59333.peg.2978	None	None	None	fig|6666666.59337.peg.1796	None	None	None	None	None	None	None	None	None	None	None	None	None	None

8047	Dentin sialophosphoprotein precursor	None	None	None	None	None	None	fig|6666666.59336.peg.3834	fig|6666666.59333.peg.2710	None	None	fig|6666666.59340.peg.817	fig|6666666.59337.peg.2025	fig|6666666.59342.peg.6051	None	None	fig|6666666.59345.peg.2451	None	None	None	None	None	None	None	None	None	None

10444	hypothetical protein	fig|6666666.59329.peg.3006	None	None	None	fig|6666666.59334.peg.2585	fig|6666666.59335.peg.5587	fig|6666666.59336.peg.3198	None	None	fig|6666666.59339.peg.4212	None	None	None	None	None	None	None	None	None	fig|6666666.59348.peg.4236	None	None	None	None	None	None

4779	hypothetical protein	None	fig|6666666.59330.peg.2962	None	None	None	None	None	None	fig|6666666.59338.peg.504	fig|6666666.59339.peg.865	None	fig|6666666.59337.peg.3441	fig|6666666.59342.peg.5417	None	None	None	None	fig|6666666.59347.peg.2173	None	None	None	None	None	None	None	None

10155	ABC-type amino acid transport, signal transduction systems, periplasmic component domain	fig|6666666.59329.peg.4936	None	None	None	fig|6666666.59334.peg.1040	None	None	None	None	fig|6666666.59339.peg.3971	None	fig|6666666.59337.peg.4719	fig|6666666.59342.peg.1615	None	None	None	None	None	fig|6666666.59346.peg.3645	None	None	None	None	None	None	None

143	PTS system, glucitol sorbitol-specific IIA component EC 2.7.1.69	None	None	None	None	None	None	None	None	fig|6666666.59338.peg.3937	None	None	None	None	fig|6666666.71181.peg.4224	fig|6666666.59344.peg.6070	None	fig|6666666.59341.peg.5557	None	None	None	None	None	None	fig|6666666.59353.peg.2749	fig|6666666.59355.peg.1733	None

8877	hypothetical protein; Some similarities with bacteriophage protein	None	None	None	None	None	fig|6666666.59335.peg.5718	None	None	None	fig|6666666.59339.peg.2144	None	None	None	fig|6666666.71181.peg.2679	fig|6666666.59344.peg.4291	None	None	None	None	None	None	None	None	fig|6666666.59353.peg.3894	fig|6666666.59355.peg.3798	None

821	Putative transport protein	fig|6666666.59329.peg.2986	None	None	None	None	fig|6666666.59335.peg.3709	None	fig|6666666.59333.peg.3382	None	None	None	fig|6666666.59337.peg.3618	fig|6666666.59342.peg.4389	None	None	None	None	None	fig|6666666.59346.peg.1554	None	None	None	None	None	None	None

4180	Large repetitive protein	fig|6666666.59329.peg.6401	fig|6666666.59330.peg.4991	None	None	None	None	None	fig|6666666.59333.peg.3649	None	fig|6666666.59339.peg.8932	None	fig|6666666.59337.peg.5898	None	None	None	fig|6666666.59345.peg.3358	None	None	None	None	None	None	None	None	None	None

14	Phosphate starvation-inducible ATPase PhoH with RNA binding motif	None	fig|6666666.59330.peg.4034	None	None	None	None	None	None	None	None	fig|6666666.59340.peg.860	fig|6666666.59337.peg.1073	fig|6666666.59342.peg.1134	None	None	None	fig|6666666.59341.peg.1639	None	None	None	None	fig|6666666.59350.peg.569	None	None	None	None

3900	hypothetical protein	None	None	fig|6666666.59331.peg.4473	fig|6666666.59332.peg.2781	None	None	None	None	fig|6666666.59338.peg.5958	None	None	None	None	None	None	None	fig|6666666.59341.peg.1289	fig|6666666.59347.peg.714	fig|6666666.59346.peg.5339	None	None	None	None	None	None	None

4403	Type III secretion spans bacterial envelope protein YscO	fig|6666666.59329.peg.1316	fig|6666666.59330.peg.4257	None	None	None	None	None	fig|6666666.59333.peg.3368	None	None	None	fig|6666666.59337.peg.5237	fig|6666666.59342.peg.3605	None	None	fig|6666666.59345.peg.1464	None	None	None	None	None	None	None	None	None	None

1373	Nucleoside triphosphate pyrophosphohydrolase MazG EC 3.6.1.8	None	None	None	None	None	None	None	fig|6666666.59333.peg.4311	None	fig|6666666.59339.peg.7386	fig|6666666.59340.peg.1068	None	None	None	None	None	None	None	fig|6666666.59346.peg.3153	fig|6666666.59348.peg.3648	None	fig|6666666.59350.peg.4273	None	None	None	None

8546	Uncharacterized protein ImpI VasC	None	fig|6666666.59330.peg.2149	None	None	None	None	fig|6666666.59336.peg.3638	fig|6666666.59333.peg.3922	None	fig|6666666.59339.peg.2735	None	fig|6666666.59337.peg.4074	None	None	None	fig|6666666.59345.peg.3383	None	None	None	None	None	None	None	None	None	None

2897	FIG01199741 hypothetical protein	fig|6666666.59329.peg.5917	None	None	None	fig|6666666.59334.peg.1102	None	fig|6666666.59336.peg.5673	None	None	None	None	None	None	None	None	fig|6666666.59345.peg.2631	fig|6666666.59341.peg.399	None	None	fig|6666666.59348.peg.127	None	None	None	None	None	None

1890	bacteriocin resistance protein, putative	None	None	None	None	fig|6666666.59334.peg.5444	None	fig|6666666.59336.peg.3832	fig|6666666.59333.peg.2708	None	None	fig|6666666.59340.peg.815	fig|6666666.59337.peg.2023	None	None	None	fig|6666666.59345.peg.2453	None	None	None	None	None	None	None	None	None	None

4792	Conserved domain protein	fig|6666666.59329.peg.4311	None	None	None	None	fig|6666666.59335.peg.4559	None	None	None	fig|6666666.59339.peg.2056	None	None	None	None	None	None	None	None	fig|6666666.59346.peg.826	None	fig|6666666.59349.peg.3252	None	None	None	None	fig|6666666.59354.peg.3114

6260	hypothetical protein	fig|6666666.59329.peg.1557	None	None	None	fig|6666666.59334.peg.3312	None	fig|6666666.59336.peg.418	None	None	None	None	None	None	None	None	fig|6666666.59345.peg.1025	None	fig|6666666.59347.peg.1111	None	fig|6666666.59348.peg.2732	None	None	None	None	None	None

5233	Type III secretion cytoplasmic protein YscL	fig|6666666.59329.peg.6032	fig|6666666.59330.peg.4701	None	None	None	None	None	fig|6666666.59333.peg.3000	None	None	None	fig|6666666.59337.peg.4471	fig|6666666.59342.peg.635	None	None	fig|6666666.59345.peg.1478	None	None	None	None	None	None	None	None	None	None

1737	Anaerobic nitric oxide reductase transcription regulator NorR	fig|6666666.59329.peg.587	None	None	fig|6666666.59332.peg.5244	None	None	None	None	fig|6666666.59338.peg.5788	fig|6666666.59339.peg.2000	None	None	None	None	None	None	None	None	None	None	fig|6666666.59349.peg.2969	None	None	None	None	fig|6666666.59354.peg.1448

1596	TraF-related protein	None	None	fig|6666666.59331.peg.564	None	None	fig|6666666.59335.peg.563	None	fig|6666666.59333.peg.3895	fig|6666666.59338.peg.2332	fig|6666666.59339.peg.2940	None	fig|6666666.59337.peg.3828	None	None	None	None	None	None	None	None	None	None	None	None	None	None

5644	Excinuclease ABC subunit B	fig|6666666.59329.peg.2669	None	None	None	None	None	fig|6666666.59336.peg.1324	None	None	None	fig|6666666.59340.peg.2299	fig|6666666.59337.peg.4019	None	None	None	None	None	None	None	None	fig|6666666.59349.peg.1862	None	None	None	None	fig|6666666.59354.peg.6299

4826	hypothetical protein	None	None	None	None	None	fig|6666666.59335.peg.4401	None	None	None	fig|6666666.59339.peg.8477	None	None	None	None	None	None	None	None	None	fig|6666666.59348.peg.2544	fig|6666666.59349.peg.885	None	fig|6666666.59351.peg.5094	None	None	fig|6666666.59354.peg.3872

6469	hypothetical protein	fig|6666666.59329.peg.4082	None	None	None	None	fig|6666666.59335.peg.6226	None	None	None	fig|6666666.59339.peg.1458	fig|6666666.59340.peg.2338	None	None	None	None	None	fig|6666666.59341.peg.1873	fig|6666666.59347.peg.951	None	None	None	None	None	None	None	None

6724	FIG01199588 hypothetical protein	fig|6666666.59329.peg.2808	fig|6666666.59330.peg.4213	None	None	None	None	fig|6666666.59336.peg.5559	fig|6666666.59333.peg.1727	None	None	None	None	fig|6666666.59342.peg.3838	None	None	fig|6666666.59345.peg.5084	None	None	None	None	None	None	None	None	None	None

6729	FIG01201465 hypothetical protein	fig|6666666.59329.peg.4689	fig|6666666.59330.peg.2196	None	None	None	None	fig|6666666.59336.peg.2556	None	None	None	None	fig|6666666.59337.peg.5073	fig|6666666.59342.peg.4088	None	None	fig|6666666.59345.peg.4356	None	None	None	None	None	None	None	None	None	None

9937	FIG01200673 hypothetical protein	fig|6666666.59329.peg.6314	None	None	None	fig|6666666.59334.peg.1212	None	fig|6666666.59336.peg.4714	fig|6666666.59333.peg.1793	None	None	None	None	fig|6666666.59342.peg.4782	None	None	fig|6666666.59345.peg.444	None	None	None	None	None	None	None	None	None	None

907	FIG01200937 hypothetical protein	None	None	fig|6666666.59331.peg.948	fig|6666666.59332.peg.1724	None	None	None	None	fig|6666666.59338.peg.3429	None	None	None	None	None	None	None	fig|6666666.59341.peg.2871	fig|6666666.59347.peg.496	fig|6666666.59346.peg.3449	None	None	None	None	None	None	None

23	PTS system, glucitol sorbitol-specific IIB component and second of two IIC components EC 2.7.1.69	None	None	None	None	None	None	None	None	fig|6666666.59338.peg.5720	None	None	None	None	fig|6666666.71181.peg.4225	fig|6666666.59344.peg.6068	None	fig|6666666.59341.peg.5556	None	None	None	None	None	None	fig|6666666.59353.peg.2750	fig|6666666.59355.peg.1732	None

7759	hypothetical protein	None	None	None	None	None	fig|6666666.59335.peg.2705	None	fig|6666666.59333.peg.1807	None	fig|6666666.59339.peg.3090	None	None	None	None	None	None	None	None	None	None	fig|6666666.59349.peg.1346	None	fig|6666666.59351.peg.4230	None	None	fig|6666666.59354.peg.2631

7918	Type III secretion cytoplasmic protein YscF	fig|6666666.59329.peg.2729	fig|6666666.59330.peg.3751	None	None	None	None	None	fig|6666666.59333.peg.2993	None	None	None	fig|6666666.59337.peg.4465	fig|6666666.59342.peg.628	None	None	fig|6666666.59345.peg.1484	None	None	None	None	None	None	None	None	None	None

6287	hypothetical protein	None	None	fig|6666666.59331.peg.3866	fig|6666666.59332.peg.5911	None	None	None	None	fig|6666666.59338.peg.1516	None	None	None	None	None	None	None	fig|6666666.59341.peg.4110	fig|6666666.59347.peg.3290	fig|6666666.59346.peg.1902	None	None	None	None	None	None	None

3441	FIG01205019 hypothetical protein	fig|6666666.59329.peg.3671	fig|6666666.59330.peg.4253	None	None	None	None	fig|6666666.59336.peg.5219	None	None	None	None	fig|6666666.59337.peg.2769	fig|6666666.59342.peg.5315	None	None	fig|6666666.59345.peg.4284	None	None	None	None	None	None	None	None	None	None

5211	Chromate transport protein ChrA	None	None	None	fig|6666666.59332.peg.698	None	None	None	None	None	None	fig|6666666.59340.peg.2616	None	None	None	None	None	fig|6666666.59341.peg.3902	fig|6666666.59347.peg.2957	None	fig|6666666.59348.peg.2705	None	None	None	None	None	fig|6666666.59354.peg.3018

9919	FIG01201599 hypothetical protein	None	None	fig|6666666.59331.peg.3229	fig|6666666.59332.peg.3092	None	fig|6666666.59335.peg.3199	None	None	None	fig|6666666.59339.peg.1754	None	None	None	None	None	None	None	None	fig|6666666.59346.peg.881	fig|6666666.59348.peg.1620	None	None	None	None	None	None

7534	FIG01201743 hypothetical protein	fig|6666666.59329.peg.1444	fig|6666666.59330.peg.63	None	None	None	None	fig|6666666.59336.peg.1791	None	None	None	None	fig|6666666.59337.peg.850	fig|6666666.59342.peg.430	None	None	fig|6666666.59345.peg.4844	None	None	None	None	None	None	None	None	None	None

11420	Curli production assembly transport component CsgG	None	None	None	None	None	None	None	None	fig|6666666.59338.peg.5335	None	None	None	None	None	None	None	fig|6666666.59341.peg.1975	fig|6666666.59347.peg.1479	None	None	fig|6666666.59349.peg.3060	None	fig|6666666.59351.peg.3060	None	None	fig|6666666.59354.peg.4271

4448	Aspartokinase EC 2.7.2.4	fig|6666666.59329.peg.2939	None	None	None	None	None	fig|6666666.59336.peg.3714	fig|6666666.59333.peg.4581	None	None	None	fig|6666666.59337.peg.1868	fig|6666666.59342.peg.413	None	None	fig|6666666.59345.peg.4187	None	None	None	None	None	None	None	None	None	None

8919	L-serine dehydratase EC 4.3.1.17	None	fig|6666666.59330.peg.2074	None	fig|6666666.59332.peg.5504	None	None	None	fig|6666666.59333.peg.4479	None	None	None	None	fig|6666666.59342.peg.5208	None	None	None	None	None	None	fig|6666666.59348.peg.1444	None	fig|6666666.59350.peg.8053	None	None	None	None

640	FIG01201304 hypothetical protein	fig|6666666.59329.peg.3141	fig|6666666.59330.peg.3875	None	None	None	None	fig|6666666.59336.peg.4441	fig|6666666.59333.peg.4767	None	None	None	fig|6666666.59337.peg.1949	None	None	None	fig|6666666.59345.peg.4054	None	None	None	None	None	None	None	None	None	None

1673	FIG01204242 hypothetical protein	None	None	None	fig|6666666.59332.peg.5972	fig|6666666.59334.peg.2082	None	fig|6666666.59336.peg.4302	None	None	None	None	None	fig|6666666.59342.peg.704	None	None	fig|6666666.59345.peg.4483	None	None	None	None	None	None	None	None	fig|6666666.59355.peg.5819	None

1419	hypothetical protein	None	fig|6666666.59330.peg.1849	None	None	None	None	None	None	None	fig|6666666.59339.peg.5880	fig|6666666.59340.peg.931	fig|6666666.59337.peg.2338	None	None	None	None	fig|6666666.59341.peg.3378	fig|6666666.59347.peg.635	None	None	None	None	None	None	None	None

1412	Predicted nucleoside ABC transporter, permease 2 component	None	None	None	None	None	None	None	None	fig|6666666.59338.peg.601	None	fig|6666666.59340.peg.3506	None	None	fig|6666666.71181.peg.6274	None	None	fig|6666666.59341.peg.4763	fig|6666666.59347.peg.2978	None	None	None	fig|6666666.59350.peg.6122	None	None	None	None

4893	putative type III secretion protein SycN	fig|6666666.59329.peg.525	fig|6666666.59330.peg.3289	None	None	None	None	None	fig|6666666.59333.peg.3363	None	None	None	fig|6666666.59337.peg.5242	fig|6666666.59342.peg.1911	None	None	fig|6666666.59345.peg.1460	None	None	None	None	None	None	None	None	None	None

10922	Permease of the major facilitator superfamily	fig|6666666.59329.peg.4511	None	None	fig|6666666.59332.peg.3820	None	None	fig|6666666.59336.peg.3414	fig|6666666.59333.peg.2008	None	None	None	fig|6666666.59337.peg.2398	None	None	None	fig|6666666.59345.peg.963	None	None	None	None	None	None	None	None	None	None

9142	Glycerol-3-phosphate acyltransferase EC 2.3.1.15	None	None	fig|6666666.59331.peg.6085	None	fig|6666666.59334.peg.5040	fig|6666666.59335.peg.4262	None	None	None	fig|6666666.59339.peg.8577	None	None	None	None	None	None	None	None	None	fig|6666666.59348.peg.2966	fig|6666666.59349.peg.1090	None	None	None	None	None

9146	ABC-type amino acid transport, signal transduction systems, periplasmic component domain	None	None	None	None	None	fig|6666666.59335.peg.648	None	None	None	fig|6666666.59339.peg.989	None	None	None	None	None	None	None	None	None	fig|6666666.59348.peg.3690	fig|6666666.59349.peg.4020	None	fig|6666666.59351.peg.1126	None	None	fig|6666666.59354.peg.447

9145	FIG01200622 hypothetical protein	None	fig|6666666.59330.peg.1825	None	None	None	None	None	None	None	None	None	fig|6666666.59337.peg.1066	fig|6666666.59342.peg.2	None	None	fig|6666666.59345.peg.1651	None	None	None	None	fig|6666666.59349.peg.1003	None	None	None	None	fig|6666666.59354.peg.585

4589	FIG01203472 hypothetical protein	None	fig|6666666.59330.peg.3735	None	None	None	fig|6666666.59335.peg.3423	None	fig|6666666.59333.peg.4465	None	None	None	fig|6666666.59337.peg.4813	fig|6666666.59342.peg.1840	None	None	fig|6666666.59345.peg.3363	None	None	None	None	None	None	None	None	None	None

4856	ABC transporter, periplasmic substrate-binding protein YnjB	fig|6666666.59329.peg.5462	None	None	None	fig|6666666.59334.peg.3484	None	fig|6666666.59336.peg.4394	None	None	None	None	None	fig|6666666.59342.peg.4757	None	None	None	fig|6666666.59341.peg.1093	None	None	fig|6666666.59348.peg.5537	None	None	None	None	None	None

1338	Type III secretion outermembrane negative regulator of secretion TyeA	fig|6666666.59329.peg.524	fig|6666666.59330.peg.3290	None	None	None	None	None	fig|6666666.59333.peg.3364	None	None	None	fig|6666666.59337.peg.5241	fig|6666666.59342.peg.1910	None	None	fig|6666666.59345.peg.1461	None	None	None	None	None	None	None	None	None	None

31	hypothetical protein	None	None	None	None	None	fig|6666666.59335.peg.4845	None	fig|6666666.59333.peg.3450	None	fig|6666666.59339.peg.3802	None	None	None	None	None	None	fig|6666666.59341.peg.4317	None	None	fig|6666666.59348.peg.1487	None	fig|6666666.59350.peg.7878	None	None	None	None

11402	MmgE PrpD family protein	None	None	None	None	None	None	None	fig|6666666.59333.peg.978	None	None	None	None	None	fig|6666666.71181.peg.2316	fig|6666666.59344.peg.4551	None	None	None	None	None	None	fig|6666666.59350.peg.7412	None	fig|6666666.59353.peg.3338	fig|6666666.59355.peg.5338	None

8857	hypothetical protein	None	None	None	None	None	fig|6666666.59335.peg.3115	None	None	fig|6666666.59338.peg.2513	fig|6666666.59339.peg.169	None	None	None	None	None	None	fig|6666666.59341.peg.3332	fig|6666666.59347.peg.3697	None	fig|6666666.59348.peg.1459	None	None	None	None	None	None

4469	Two-component sensor protein RcsD EC 2.7.3.-	None	None	None	None	None	None	None	None	None	None	None	None	None	fig|6666666.71181.peg.501	fig|6666666.59344.peg.1717	None	None	None	None	None	fig|6666666.59349.peg.3184	None	fig|6666666.59351.peg.3125	fig|6666666.59353.peg.1129	fig|6666666.59355.peg.347	None

949	FIG01204809 hypothetical protein	fig|6666666.59329.peg.3848	fig|6666666.59330.peg.748	None	None	None	None	fig|6666666.59336.peg.4779	None	None	None	None	fig|6666666.59337.peg.3478	fig|6666666.59342.peg.4078	None	None	fig|6666666.59345.peg.2776	None	None	None	None	None	None	None	None	None	None

944	Hypothetical protein colocalized with Enterobactin receptor VctA	fig|6666666.59329.peg.6572	None	None	None	None	None	fig|6666666.59336.peg.6286	fig|6666666.59333.peg.1229	None	None	None	None	fig|6666666.59342.peg.1029	None	None	fig|6666666.59345.peg.4818	None	None	None	None	None	fig|6666666.59350.peg.6698	None	None	None	None

7134	Cytochrome c-type biogenesis protein DsbD, protein-disulfide reductase EC 1.8.1.8	None	None	None	None	fig|6666666.59334.peg.31	None	None	None	None	None	None	None	fig|6666666.59342.peg.2739	None	None	fig|6666666.59345.peg.5130	None	None	fig|6666666.59346.peg.782	fig|6666666.59348.peg.5062	None	None	None	None	None	fig|6666666.59354.peg.3262

1739	no significant homology	fig|6666666.59329.peg.1536	fig|6666666.59330.peg.334	None	None	None	None	None	fig|6666666.59333.peg.757	None	None	None	fig|6666666.59337.peg.514	fig|6666666.59342.peg.5658	None	None	fig|6666666.59345.peg.5003	None	None	None	None	None	None	None	None	None	None

2836	Type III secretion cytoplasmic plug protein LcrG	fig|6666666.59329.peg.531	fig|6666666.59330.peg.3284	None	None	None	None	None	fig|6666666.59333.peg.4288	None	None	None	fig|6666666.59337.peg.2807	fig|6666666.59342.peg.1916	None	None	fig|6666666.59345.peg.1455	None	None	None	None	None	None	None	None	None	None

7688	FIG01206982 hypothetical protein	None	None	fig|6666666.59331.peg.184	fig|6666666.59332.peg.3373	fig|6666666.59334.peg.4308	None	None	None	None	None	None	None	None	None	None	None	fig|6666666.59341.peg.4465	fig|6666666.59347.peg.2220	fig|6666666.59346.peg.4164	None	None	None	None	None	None	None

5891	hypothetical protein	fig|6666666.59329.peg.6224	None	None	None	None	fig|6666666.59335.peg.6224	None	None	None	None	fig|6666666.59340.peg.2492	None	None	None	None	None	None	fig|6666666.59347.peg.944	fig|6666666.59346.peg.4982	fig|6666666.59348.peg.3341	None	None	None	None	None	None

5892	FIG00924762 hypothetical protein	None	None	None	None	None	None	None	None	None	None	None	None	None	fig|6666666.71181.peg.191	fig|6666666.59344.peg.232	None	None	None	None	None	None	None	fig|6666666.59351.peg.564	fig|6666666.59353.peg.631	fig|6666666.59355.peg.114	fig|6666666.59354.peg.4083

4664	Methyl-accepting chemotaxis protein	None	None	fig|6666666.59331.peg.1402	None	None	None	None	None	None	None	None	None	None	None	None	None	None	fig|6666666.59347.peg.744	fig|6666666.59346.peg.2482	None	fig|6666666.59349.peg.2998	None	fig|6666666.59351.peg.3179	None	None	fig|6666666.59354.peg.38

321	tRNA nucleotidyltransferase EC 2.7.7.21 EC 2.7.7.25	None	None	None	None	fig|6666666.59334.peg.531	fig|6666666.59335.peg.1505	None	fig|6666666.59333.peg.184	None	None	None	None	None	None	fig|6666666.59344.peg.4467	None	None	None	None	None	None	None	None	fig|6666666.59353.peg.4182	fig|6666666.59355.peg.1695	None

10943	FIG01205080 hypothetical protein	fig|6666666.59329.peg.3896	fig|6666666.59330.peg.2921	None	None	None	None	fig|6666666.59336.peg.909	None	None	None	None	fig|6666666.59337.peg.3200	fig|6666666.59342.peg.5234	None	None	fig|6666666.59345.peg.2901	None	None	None	None	None	None	None	None	None	None

9780	Type III secretion inner membrane protein YscQ,homologous to flagellar export components	fig|6666666.59329.peg.1318	fig|6666666.59330.peg.4260	None	None	None	None	None	fig|6666666.59333.peg.3370	None	None	None	fig|6666666.59337.peg.5235	fig|6666666.59342.peg.2072	None	None	fig|6666666.59345.peg.1467	None	None	None	None	None	None	None	None	None	None

8365	Adenosylhomocysteinase EC 3.3.1.1	None	None	fig|6666666.59331.peg.225	None	None	None	None	None	None	None	None	None	None	fig|6666666.71181.peg.1188	fig|6666666.59344.peg.2317	None	None	None	fig|6666666.59346.peg.3894	None	None	None	None	fig|6666666.59353.peg.3397	fig|6666666.59355.peg.3495	None

3189	hypothetical protein	None	fig|6666666.59330.peg.3742	None	None	None	fig|6666666.59335.peg.32	fig|6666666.59336.peg.6205	None	None	fig|6666666.59339.peg.7558	None	None	fig|6666666.59342.peg.1161	None	None	fig|6666666.59345.peg.4207	None	None	None	None	None	None	None	None	None	None

1443	Response regulator	None	None	None	None	None	fig|6666666.59335.peg.75	None	None	None	fig|6666666.59339.peg.7630	None	None	None	None	None	None	None	None	None	fig|6666666.59348.peg.628	fig|6666666.59349.peg.3568	None	fig|6666666.59351.peg.2419	None	None	fig|6666666.59354.peg.4614

2475	ParE toxin protein	None	fig|6666666.59330.peg.4836	None	None	None	fig|6666666.59335.peg.1902	None	None	None	None	None	None	None	fig|6666666.71181.peg.4833	fig|6666666.59344.peg.5602	None	None	None	None	None	None	None	None	fig|6666666.59353.peg.4889	fig|6666666.59355.peg.4960	None

6304	FIG01200390 hypothetical protein	fig|6666666.59329.peg.2725	None	None	None	None	fig|6666666.59335.peg.5849	None	fig|6666666.59333.peg.2578	None	fig|6666666.59339.peg.7306	None	fig|6666666.59337.peg.4194	fig|6666666.59342.peg.2089	None	None	None	None	None	None	None	None	None	None	None	None	None

2819	FIG01200822 hypothetical protein	fig|6666666.59329.peg.1793	fig|6666666.59330.peg.1558	None	None	None	None	fig|6666666.59336.peg.639	None	None	None	None	fig|6666666.59337.peg.1641	fig|6666666.59342.peg.4795	None	None	fig|6666666.59345.peg.5478	None	None	None	None	None	None	None	None	None	None

4247	Mobile element protein	None	None	None	None	None	None	None	None	None	fig|6666666.59339.peg.6002	None	fig|6666666.59337.peg.2020	None	None	None	None	fig|6666666.59341.peg.4479	None	fig|6666666.59346.peg.215	None	fig|6666666.59349.peg.3578	None	None	None	None	fig|6666666.59354.peg.5858

44	FIG01202617 hypothetical protein	fig|6666666.59329.peg.3603	None	None	None	None	None	fig|6666666.59336.peg.5013	None	None	fig|6666666.59339.peg.7870	None	None	fig|6666666.59342.peg.3852	None	None	fig|6666666.59345.peg.3871	fig|6666666.59341.peg.1500	None	None	None	None	None	None	None	None	None

349	Phage-related capsid packaging protein	None	None	None	None	None	fig|6666666.59335.peg.294	None	None	None	fig|6666666.59339.peg.2553	None	None	None	fig|6666666.71181.peg.4474	fig|6666666.59344.peg.349	None	None	None	None	None	None	None	None	fig|6666666.59353.peg.538	fig|6666666.59355.peg.23	None

10963	Serine threonine protein kinase EC 2.7.11.1	None	fig|6666666.59330.peg.4139	None	None	None	None	None	fig|6666666.59333.peg.3212	fig|6666666.59338.peg.3796	None	None	fig|6666666.59337.peg.4771	None	None	None	fig|6666666.59345.peg.3437	None	fig|6666666.59347.peg.2428	None	None	None	None	None	None	None	None

8209	Mobile element protein	None	fig|6666666.59330.peg.5162	fig|6666666.59331.peg.286	None	None	None	None	None	None	fig|6666666.59339.peg.6003	None	None	None	fig|6666666.71181.peg.5816	None	None	None	None	fig|6666666.59346.peg.4943	fig|6666666.59348.peg.4539	None	None	None	None	None	None

2047	FIG01200930 hypothetical protein	fig|6666666.59329.peg.2187	fig|6666666.59330.peg.3239	None	None	None	None	fig|6666666.59336.peg.1666	None	None	None	None	fig|6666666.59337.peg.2150	fig|6666666.59342.peg.4669	None	None	fig|6666666.59345.peg.3962	None	None	None	None	None	None	None	None	None	None

11081	FIG01202850 hypothetical protein	fig|6666666.59329.peg.1733	fig|6666666.59330.peg.3822	None	None	None	None	None	fig|6666666.59333.peg.3703	None	None	None	fig|6666666.59337.peg.2706	fig|6666666.59342.peg.4986	None	None	fig|6666666.59345.peg.3184	None	None	None	None	None	None	None	None	None	None

8234	FIG01203201 hypothetical protein	fig|6666666.59329.peg.6190	fig|6666666.59330.peg.860	None	None	None	None	fig|6666666.59336.peg.3855	None	None	None	None	fig|6666666.59337.peg.3279	fig|6666666.59342.peg.3978	None	None	fig|6666666.59345.peg.543	None	None	None	None	None	None	None	None	None	None

1365	hypothetical protein	None	fig|6666666.59330.peg.3143	fig|6666666.59331.peg.2112	fig|6666666.59332.peg.5593	fig|6666666.59334.peg.3643	None	None	None	None	None	None	None	fig|6666666.59342.peg.4296	None	None	None	None	None	fig|6666666.59346.peg.5191	None	None	None	None	None	None	None

5445	FIG01200843 hypothetical protein	fig|6666666.59329.peg.3280	fig|6666666.59330.peg.4299	None	None	None	None	None	fig|6666666.59333.peg.5341	None	None	None	fig|6666666.59337.peg.4006	fig|6666666.59342.peg.4300	None	None	fig|6666666.59345.peg.1699	None	None	None	None	None	None	None	None	None	None

541	FIG01200306 hypothetical protein	fig|6666666.59329.peg.4058	fig|6666666.59330.peg.3793	None	None	None	None	None	fig|6666666.59333.peg.702	None	None	None	fig|6666666.59337.peg.3868	fig|6666666.59342.peg.1902	None	None	fig|6666666.59345.peg.300	None	None	None	None	None	None	None	None	None	None

3217	putative amino group acetyl transferase	fig|6666666.59329.peg.1433	fig|6666666.59330.peg.3682	None	None	None	None	fig|6666666.59336.peg.2376	None	None	None	None	fig|6666666.59337.peg.3652	fig|6666666.59342.peg.1207	None	None	fig|6666666.59345.peg.1231	None	None	None	None	None	None	None	None	None	None

5299	Glucitol operon activator protein	None	None	None	None	None	None	None	None	fig|6666666.59338.peg.3939	None	None	None	None	fig|6666666.71181.peg.4222	fig|6666666.59344.peg.6072	None	fig|6666666.59341.peg.5866	None	None	None	None	None	None	fig|6666666.59353.peg.2747	fig|6666666.59355.peg.1735	None

8146	Oligopeptide ABC transporter, periplasmic oligopeptide-binding protein OppA TC 3.A.1.5.1	fig|6666666.59329.peg.3982	None	None	None	fig|6666666.59334.peg.3857	None	None	None	None	fig|6666666.59339.peg.5720	None	None	None	None	fig|6666666.59344.peg.1058	None	None	None	None	None	None	None	None	fig|6666666.59353.peg.744	fig|6666666.59355.peg.960	None

10219	hypothetical protein	None	None	fig|6666666.59331.peg.856	fig|6666666.59332.peg.704	None	None	None	None	fig|6666666.59338.peg.2673	None	fig|6666666.59340.peg.706	None	None	None	None	None	None	fig|6666666.59347.peg.1128	None	fig|6666666.59348.peg.347	None	None	None	None	None	None

5516	Ethanolamine ammonia-lyase heavy chain EC 4.3.1.7	None	None	None	None	None	None	None	fig|6666666.59333.peg.5405	fig|6666666.59338.peg.5045	None	None	None	None	fig|6666666.71181.peg.1569	fig|6666666.59344.peg.2017	None	None	None	None	None	None	None	None	fig|6666666.59353.peg.2908	fig|6666666.59355.peg.2831	None

2330	Capsular polysaccharide synthesis enzyme CpsG, Lipid A core - O-antigen ligase	fig|6666666.59329.peg.542	fig|6666666.59330.peg.3599	None	None	None	None	fig|6666666.59336.peg.6635	fig|6666666.59333.peg.988	None	None	None	fig|6666666.59337.peg.3006	None	None	None	fig|6666666.59345.peg.631	None	None	None	None	None	None	None	None	None	None

9352	Phenylacetate-coenzyme A ligase EC 6.2.1.30 PaaF	None	None	None	None	None	None	None	fig|6666666.59333.peg.4120	None	None	None	None	None	fig|6666666.71181.peg.4703	fig|6666666.59344.peg.3323	None	None	None	None	None	None	fig|6666666.59350.peg.3525	None	fig|6666666.59353.peg.2758	fig|6666666.59355.peg.3674	None

521	Uncharacterized protein C3236	None	fig|6666666.59330.peg.3026	fig|6666666.59331.peg.5026	fig|6666666.59332.peg.2835	None	None	fig|6666666.59336.peg.778	None	None	None	None	fig|6666666.59337.peg.235	None	None	None	None	None	None	fig|6666666.59346.peg.4490	None	None	None	None	None	None	None

1231	hypothetical protein	None	None	fig|6666666.59331.peg.2708	None	None	fig|6666666.59335.peg.5529	None	None	fig|6666666.59338.peg.5497	fig|6666666.59339.peg.3824	None	None	None	None	None	None	None	fig|6666666.59347.peg.1382	fig|6666666.59346.peg.5309	None	None	None	None	None	None	None

1232	YheO-like PAS domain	None	None	None	fig|6666666.59332.peg.4628	None	None	fig|6666666.59336.peg.3863	None	None	None	None	fig|6666666.59337.peg.2423	fig|6666666.59342.peg.3678	None	None	fig|6666666.59345.peg.944	fig|6666666.59341.peg.6051	None	None	None	None	None	None	None	None	None

7396	hypothetical protein	None	None	None	None	fig|6666666.59334.peg.3798	None	fig|6666666.59336.peg.3831	None	None	None	fig|6666666.59340.peg.814	fig|6666666.59337.peg.2022	fig|6666666.59342.peg.4713	None	None	fig|6666666.59345.peg.2454	None	None	None	None	None	None	None	None	None	None

7318	FIG00432558 hypothetical protein	fig|6666666.59329.peg.526	fig|6666666.59330.peg.3288	None	None	None	None	None	fig|6666666.59333.peg.3362	None	None	None	fig|6666666.59337.peg.5243	fig|6666666.59342.peg.1912	None	None	fig|6666666.59345.peg.1459	None	None	None	None	None	None	None	None	None	None

7311	Muramoyltetrapeptide carboxypeptidase EC 3.4.17.13	fig|6666666.59329.peg.1150	fig|6666666.59330.peg.925	None	None	None	fig|6666666.59335.peg.4320	None	None	None	fig|6666666.59339.peg.1730	None	fig|6666666.59337.peg.3951	fig|6666666.59342.peg.5851	None	None	None	None	None	None	None	None	None	None	None	None	None

8619	tRNA 5-methylaminomethyl-2-thiouridylate -methyltransferase EC 2.1.1.61	fig|6666666.59329.peg.5937	None	None	None	fig|6666666.59334.peg.4034	fig|6666666.59335.peg.3934	None	None	fig|6666666.59338.peg.1851	fig|6666666.59339.peg.7057	None	None	None	None	None	None	None	None	None	None	None	fig|6666666.59350.peg.1325	None	None	None	None

4974	hypothetical protein	None	None	fig|6666666.59331.peg.1666	fig|6666666.59332.peg.2441	None	fig|6666666.59335.peg.2881	None	None	None	fig|6666666.59339.peg.4665	None	None	None	None	None	None	None	None	fig|6666666.59346.peg.2097	fig|6666666.59348.peg.3418	None	None	None	None	None	None

3270	hypothetical protein	fig|6666666.59329.peg.6003	None	None	None	None	None	fig|6666666.59336.peg.6335	fig|6666666.59333.peg.3121	None	None	None	None	fig|6666666.59342.peg.5861	None	None	fig|6666666.59345.peg.191	fig|6666666.59341.peg.3218	None	None	None	None	None	None	None	None	None

65	FIG01205019 hypothetical protein	None	fig|6666666.59330.peg.4252	None	None	None	None	fig|6666666.59336.peg.5220	fig|6666666.59333.peg.2909	None	None	None	fig|6666666.59337.peg.2768	fig|6666666.59342.peg.5314	None	None	fig|6666666.59345.peg.4285	None	None	None	None	None	None	None	None	None	None

633	FIG01202440 hypothetical protein	None	fig|6666666.59330.peg.3215	None	None	None	None	None	None	fig|6666666.59338.peg.4103	fig|6666666.59339.peg.8122	None	fig|6666666.59337.peg.4304	fig|6666666.59342.peg.3534	None	None	None	None	fig|6666666.59347.peg.3727	None	None	None	None	None	None	None	None

6522	Chitinase EC 3.2.1.14	None	None	None	None	None	fig|6666666.59335.peg.1481	None	None	fig|6666666.59338.peg.6172	fig|6666666.59339.peg.2086	None	None	fig|6666666.59342.peg.2255	None	None	fig|6666666.59345.peg.4744	None	fig|6666666.59347.peg.1097	None	None	None	None	None	None	None	None

7011	Protease precursor	None	None	None	None	None	fig|6666666.59335.peg.1380	None	None	None	fig|6666666.59339.peg.2776	None	None	None	None	None	None	None	None	None	fig|6666666.59348.peg.2320	fig|6666666.59349.peg.440	None	fig|6666666.59351.peg.1608	None	None	fig|6666666.59354.peg.5598

5454	hypothetical protein	None	None	None	None	fig|6666666.59334.peg.2004	None	None	fig|6666666.59333.peg.3346	fig|6666666.59338.peg.4229	None	None	None	fig|6666666.59342.peg.2011	None	None	None	fig|6666666.59341.peg.3509	fig|6666666.59347.peg.1647	None	None	None	None	None	None	None	None

6381	hypothetical protein	fig|6666666.59329.peg.3897	fig|6666666.59330.peg.2922	None	None	None	None	fig|6666666.59336.peg.908	None	None	None	None	fig|6666666.59337.peg.3199	fig|6666666.59342.peg.5236	None	None	fig|6666666.59345.peg.2902	None	None	None	None	None	None	None	None	None	None

11149	possible efflux permease	None	None	fig|6666666.59331.peg.5227	None	fig|6666666.59334.peg.3567	None	None	None	fig|6666666.59338.peg.2459	fig|6666666.59339.peg.3924	fig|6666666.59340.peg.2215	None	None	None	None	None	None	None	fig|6666666.59346.peg.5174	None	None	None	None	None	None	None

1844	Periplasmic chorismate mutase I precursor EC 5.4.99.5	None	None	None	None	None	fig|6666666.59335.peg.2264	None	None	None	fig|6666666.59339.peg.2893	None	None	None	fig|6666666.71181.peg.1209	fig|6666666.59344.peg.204	None	None	None	None	None	None	None	None	fig|6666666.59353.peg.510	fig|6666666.59355.peg.2236	None

5135	T1SS secreted agglutinin RTX	None	fig|6666666.59330.peg.1041	None	None	None	fig|6666666.59335.peg.4034	None	fig|6666666.59333.peg.4170	None	fig|6666666.59339.peg.1571	None	None	fig|6666666.59342.peg.1560	None	None	None	None	None	None	fig|6666666.59348.peg.3560	None	None	None	None	None	None

5234	FIG005274 hypothetical protein	None	fig|6666666.59330.peg.2183	None	None	None	None	fig|6666666.59336.peg.3306	None	None	None	None	fig|6666666.59337.peg.4917	fig|6666666.59342.peg.92	fig|6666666.71181.peg.4272	None	fig|6666666.59345.peg.4378	None	None	None	None	None	None	None	None	None	None

8420	hypothetical protein	None	None	None	None	fig|6666666.59334.peg.1067	None	fig|6666666.59336.peg.2265	None	fig|6666666.59338.peg.4122	None	None	None	None	None	None	fig|6666666.59345.peg.451	fig|6666666.59341.peg.2100	fig|6666666.59347.peg.2152	None	None	None	None	None	None	None	None

9123	hypothetical protein	None	None	None	fig|6666666.59332.peg.4377	None	fig|6666666.59335.peg.4646	None	None	None	fig|6666666.59339.peg.664	None	None	None	None	None	None	None	None	None	fig|6666666.59348.peg.3254	None	fig|6666666.59350.peg.7201	fig|6666666.59351.peg.3188	None	None	None

9125	Death on curing protein, Doc toxin	fig|6666666.59329.peg.2825	fig|6666666.59330.peg.4824	None	None	None	None	None	fig|6666666.59333.peg.5577	None	None	None	fig|6666666.59337.peg.5707	None	None	None	None	None	fig|6666666.59347.peg.5321	fig|6666666.59346.peg.4141	None	None	None	None	None	None	None

2596	hypothetical protein	fig|6666666.59329.peg.5768	fig|6666666.59330.peg.4280	None	None	None	None	fig|6666666.59336.peg.4156	None	None	None	None	fig|6666666.59337.peg.3432	fig|6666666.59342.peg.6297	None	None	fig|6666666.59345.peg.198	None	None	None	None	None	None	None	None	None	None

7357	NAD NADP transhydrogenase beta subunit	None	fig|6666666.59330.peg.3122	None	None	None	None	fig|6666666.59336.peg.5668	fig|6666666.59333.peg.1950	None	None	None	fig|6666666.59337.peg.1148	fig|6666666.59342.peg.4996	None	None	fig|6666666.59345.peg.3456	None	None	None	None	None	None	None	None	None	None

11050	FIG01202548 hypothetical protein	fig|6666666.59329.peg.6196	fig|6666666.59330.peg.4192	None	None	None	None	fig|6666666.59336.peg.4854	fig|6666666.59333.peg.1245	None	None	None	fig|6666666.59337.peg.4625	None	None	None	fig|6666666.59345.peg.4806	None	None	None	None	None	None	None	None	None	None

11058	Hydroxymethylpyrimidine ABC transporter, transmembrane component	None	None	None	None	None	None	None	None	fig|6666666.59338.peg.468	fig|6666666.59339.peg.5550	None	None	None	fig|6666666.71181.peg.818	fig|6666666.59344.peg.6272	None	None	None	fig|6666666.59346.peg.4172	None	None	None	None	None	fig|6666666.59355.peg.3918	None

8653	hypothetical protein	fig|6666666.59329.peg.274	None	None	fig|6666666.59332.peg.5395	None	None	None	None	fig|6666666.59338.peg.1422	None	fig|6666666.59340.peg.1294	fig|6666666.59337.peg.3394	fig|6666666.59342.peg.346	None	None	None	None	None	None	None	None	None	None	None	None	None

5751	FIG01200849 hypothetical protein	fig|6666666.59329.peg.3139	fig|6666666.59330.peg.2862	None	None	None	None	fig|6666666.59336.peg.4439	None	None	None	None	fig|6666666.59337.peg.1951	fig|6666666.59342.peg.5083	None	None	fig|6666666.59345.peg.4056	None	None	None	None	None	None	None	None	None	None

3721	Methyl-accepting chemotaxis protein I serine chemoreceptor protein	None	None	None	None	None	fig|6666666.59335.peg.2819	None	None	fig|6666666.59338.peg.2137	fig|6666666.59339.peg.3490	None	None	None	None	None	None	fig|6666666.59341.peg.3534	fig|6666666.59347.peg.4173	None	fig|6666666.59348.peg.2044	None	None	None	None	None	None

891	Glycosyltransferase EC 2.4.1.-	None	None	None	fig|6666666.59332.peg.1899	fig|6666666.59334.peg.1338	None	fig|6666666.59336.peg.1803	None	None	None	None	None	None	None	None	None	None	None	None	None	fig|6666666.59349.peg.288	None	fig|6666666.59351.peg.257	None	None	fig|6666666.59354.peg.1028

892	L,D-transpeptidase YcbB	fig|6666666.59329.peg.6309	None	None	fig|6666666.59332.peg.2681	None	None	None	None	None	fig|6666666.59339.peg.3743	fig|6666666.59340.peg.3786	None	None	None	None	None	fig|6666666.59341.peg.5463	None	None	None	None	fig|6666666.59350.peg.6837	None	None	None	None

1789	2-oxoglutarate dehydrogenase complex, dehydrogenase component	fig|6666666.59329.peg.1543	fig|6666666.59330.peg.2211	None	None	None	None	fig|6666666.59336.peg.2533	fig|6666666.59333.peg.2855	None	fig|6666666.59339.peg.2442	None	None	None	None	None	fig|6666666.59345.peg.2842	None	None	None	None	None	None	None	None	None	None

6877	FIG001614 Membrane protein	None	None	None	None	None	None	None	None	None	None	None	None	None	fig|6666666.71181.peg.3228	fig|6666666.59344.peg.4604	None	None	None	None	None	fig|6666666.59349.peg.2234	None	fig|6666666.59351.peg.885	fig|6666666.59353.peg.2080	fig|6666666.59355.peg.2745	None

9857	MSHA pilin protein MshA	fig|6666666.59329.peg.599	fig|6666666.59330.peg.18	None	None	None	None	fig|6666666.59336.peg.1186	fig|6666666.59333.peg.5456	None	None	None	fig|6666666.59337.peg.803	None	None	None	fig|6666666.59345.peg.4892	None	None	None	None	None	None	None	None	None	None

4118	Transport ATP-binding protein CydC	None	fig|6666666.59330.peg.3722	None	None	fig|6666666.59334.peg.5112	fig|6666666.59335.peg.5991	None	None	fig|6666666.59338.peg.4688	fig|6666666.59339.peg.3654	None	None	fig|6666666.59342.peg.1556	None	None	None	None	None	None	None	None	None	None	None	None	None

2640	Prolipoprotein	None	None	None	None	None	fig|6666666.59335.peg.5903	fig|6666666.59336.peg.6438	None	fig|6666666.59338.peg.4922	fig|6666666.59339.peg.4408	None	None	fig|6666666.59342.peg.2256	None	None	fig|6666666.59345.peg.4743	None	None	None	None	None	None	None	None	None	None

9075	Methylcrotonyl-CoA carboxylase carboxyl transferase subunit EC 6.4.1.4	None	fig|6666666.59330.peg.4349	None	None	None	fig|6666666.59335.peg.4970	None	fig|6666666.59333.peg.3861	fig|6666666.59338.peg.5123	None	None	fig|6666666.59337.peg.1459	None	None	None	None	None	None	None	fig|6666666.59348.peg.2811	None	None	None	None	None	None

3557	Zinc ABC transporter, inner membrane permease protein ZnuB	fig|6666666.59329.peg.2631	fig|6666666.59330.peg.2058	None	None	None	None	fig|6666666.59336.peg.5436	None	None	None	None	fig|6666666.59337.peg.5594	fig|6666666.59342.peg.1756	None	None	fig|6666666.59345.peg.3923	None	None	None	None	None	None	None	None	None	None

5301	Copper resistance protein D	None	None	None	None	None	None	None	None	None	None	None	None	None	fig|6666666.71181.peg.857	fig|6666666.59344.peg.19	None	None	None	None	None	None	fig|6666666.59350.peg.1688	fig|6666666.59351.peg.2524	fig|6666666.59353.peg.357	fig|6666666.59355.peg.1395	None

5303	Type III secretion cytoplasmic LcrG inhibitor LcrV,secretion and targeting control protein, V antigen	fig|6666666.59329.peg.532	fig|6666666.59330.peg.3283	None	None	None	None	None	fig|6666666.59333.peg.4289	None	None	None	fig|6666666.59337.peg.2808	fig|6666666.59342.peg.1917	None	None	fig|6666666.59345.peg.1454	None	None	None	None	None	None	None	None	None	None

2345	Chemotaxis protein CheD	None	None	None	None	None	fig|6666666.59335.peg.2586	None	None	None	fig|6666666.59339.peg.5478	None	None	None	None	None	None	None	None	None	None	fig|6666666.59349.peg.3492	fig|6666666.59350.peg.3427	fig|6666666.59351.peg.4374	None	None	fig|6666666.59354.peg.5070

6850	FIG01201430 hypothetical protein	fig|6666666.59329.peg.6150	None	None	None	None	None	fig|6666666.59336.peg.5309	fig|6666666.59333.peg.4419	None	None	None	fig|6666666.59337.peg.5314	fig|6666666.59342.peg.5942	None	None	fig|6666666.59345.peg.4007	None	None	None	None	None	None	None	None	None	None

3087	FIG01201008 hypothetical protein	fig|6666666.59329.peg.3616	fig|6666666.59330.peg.4231	None	None	None	None	fig|6666666.59336.peg.4318	fig|6666666.59333.peg.2937	None	None	None	fig|6666666.59337.peg.1250	None	None	None	fig|6666666.59345.peg.4435	None	None	None	None	None	None	None	None	None	None

3086	bacteriophage f237 ORF9	fig|6666666.59329.peg.5940	fig|6666666.59330.peg.5071	fig|6666666.59331.peg.357	None	None	None	None	None	None	fig|6666666.59339.peg.6006	None	fig|6666666.59337.peg.919	fig|6666666.59342.peg.4554	None	None	None	None	None	None	None	None	None	None	None	None	None

9279	Asparagine synthetase [glutamine-hydrolyzing] EC 6.3.5.4	fig|6666666.59329.peg.1947	fig|6666666.59330.peg.3006	None	fig|6666666.59332.peg.5757	None	fig|6666666.59335.peg.4588	None	None	None	None	None	fig|6666666.59337.peg.3925	None	None	None	None	None	None	fig|6666666.59346.peg.1997	None	None	None	None	None	None	None

4492	hypothetical protein	fig|6666666.59329.peg.3066	None	fig|6666666.59331.peg.1947	None	None	None	fig|6666666.59336.peg.1819	None	fig|6666666.59338.peg.4735	None	None	None	None	None	None	None	None	None	fig|6666666.59346.peg.79	fig|6666666.59348.peg.2573	None	None	None	None	None	None

5327	hypothetical protein	None	None	fig|6666666.59331.peg.6304	fig|6666666.59332.peg.4328	None	None	None	None	fig|6666666.59338.peg.2807	None	fig|6666666.59340.peg.2096	None	None	None	None	None	fig|6666666.59341.peg.2190	fig|6666666.59347.peg.2762	None	None	None	None	None	None	None	None

5323	hypothetical protein	fig|6666666.59329.peg.3713	fig|6666666.59330.peg.1983	None	None	None	None	fig|6666666.59336.peg.3427	None	None	fig|6666666.59339.peg.952	None	None	fig|6666666.59342.peg.2933	None	None	None	None	None	None	fig|6666666.59348.peg.4546	None	None	None	None	None	None

11099	hypothetical protein	fig|6666666.59329.peg.1528	None	None	None	None	None	fig|6666666.59336.peg.2323	fig|6666666.59333.peg.599	None	None	None	fig|6666666.59337.peg.2577	fig|6666666.59342.peg.2848	None	None	fig|6666666.59345.peg.688	None	None	None	None	None	None	None	None	None	None

736	Mobile element protein	None	None	None	None	None	None	fig|6666666.59336.peg.5779	fig|6666666.59333.peg.5554	fig|6666666.59338.peg.3372	None	None	None	None	None	None	None	None	None	None	fig|6666666.59348.peg.5191	None	None	None	fig|6666666.59353.peg.5110	fig|6666666.59355.peg.5742	None

10654	Putative lipoprotein	None	None	None	None	None	fig|6666666.59335.peg.3312	None	None	None	fig|6666666.59339.peg.893	None	None	None	fig|6666666.71181.peg.1173	fig|6666666.59344.peg.2301	None	None	None	None	None	None	None	None	fig|6666666.59353.peg.3410	fig|6666666.59355.peg.3508	None

4643	FIG01203131 hypothetical protein	fig|6666666.59329.peg.1928	fig|6666666.59330.peg.4396	None	None	None	None	fig|6666666.59336.peg.5877	None	None	None	None	fig|6666666.59337.peg.1829	fig|6666666.59342.peg.986	None	None	fig|6666666.59345.peg.4227	None	None	None	None	None	None	None	None	None	None

7898	FIG01201263 hypothetical protein	fig|6666666.59329.peg.5847	fig|6666666.59330.peg.2295	None	None	None	None	fig|6666666.59336.peg.3275	None	None	None	None	fig|6666666.59337.peg.283	fig|6666666.59342.peg.5533	None	None	fig|6666666.59345.peg.2132	None	None	None	None	None	None	None	None	None	None

7276	FIG01202540 hypothetical protein	fig|6666666.59329.peg.6270	None	None	fig|6666666.59332.peg.4127	None	None	fig|6666666.59336.peg.6493	fig|6666666.59333.peg.3452	None	None	None	None	None	None	None	None	fig|6666666.59341.peg.2905	None	fig|6666666.59346.peg.441	None	None	None	None	None	None	None

10555	hypothetical protein	None	None	fig|6666666.59331.peg.3481	fig|6666666.59332.peg.549	fig|6666666.59334.peg.3158	None	None	None	fig|6666666.59338.peg.983	None	fig|6666666.59340.peg.526	None	None	None	None	None	None	None	fig|6666666.59346.peg.802	None	None	None	None	None	None	None

8770	Ethidium bromide-methyl viologen resistance protein EmrE	fig|6666666.59329.peg.485	fig|6666666.59330.peg.1621	None	None	None	None	fig|6666666.59336.peg.6301	None	None	None	None	fig|6666666.59337.peg.1284	fig|6666666.59342.peg.4635	None	None	fig|6666666.59345.peg.4471	None	None	None	None	None	None	None	None	None	None

1742	Cold-active serine alkaline protease	None	fig|6666666.59330.peg.3147	None	None	None	fig|6666666.59335.peg.5231	None	None	None	None	None	fig|6666666.59337.peg.1874	None	None	None	None	None	None	None	None	fig|6666666.59349.peg.1740	None	fig|6666666.59351.peg.2393	None	None	fig|6666666.59354.peg.1052

6797	Succinyl-CoA synthetase, alpha subunit-related enzymes	None	None	None	None	None	None	None	None	None	None	None	None	None	fig|6666666.71181.peg.273	fig|6666666.59344.peg.1200	None	None	None	None	None	None	fig|6666666.59350.peg.2740	None	fig|6666666.59353.peg.1703	fig|6666666.59355.peg.1017	None

6795	Membrane fusion protein of RND family multidrug efflux pump	None	None	fig|6666666.59331.peg.4592	None	fig|6666666.59334.peg.348	None	None	None	None	fig|6666666.59339.peg.2206	fig|6666666.59340.peg.2035	None	None	None	None	None	fig|6666666.59341.peg.1131	None	None	None	None	None	None	None	None	None

6793	Protein involved in biosynthesis of mitomycin antibiotics polyketide fumonisin	None	None	None	None	None	None	None	None	None	None	None	None	None	fig|6666666.71181.peg.4743	fig|6666666.59344.peg.5895	None	None	None	None	None	None	fig|6666666.59350.peg.1694	None	fig|6666666.59353.peg.5051	fig|6666666.59355.peg.5079	None

11366	FIG01203773 hypothetical protein	None	None	fig|6666666.59331.peg.5865	None	fig|6666666.59334.peg.3265	None	None	None	fig|6666666.59338.peg.3513	None	None	None	None	None	None	None	fig|6666666.59341.peg.5880	fig|6666666.59347.peg.1513	None	None	None	None	None	None	None	None

10580	DNA polymerase-like protein PA0670	None	None	fig|6666666.59331.peg.4536	None	fig|6666666.59334.peg.359	None	None	None	fig|6666666.59338.peg.2679	None	None	None	None	None	None	None	fig|6666666.59341.peg.39	None	None	fig|6666666.59348.peg.4123	None	None	None	None	None	None

875	putative PnuC protein	fig|6666666.59329.peg.6086	fig|6666666.59330.peg.2967	fig|6666666.59331.peg.4808	None	None	None	None	None	None	None	None	fig|6666666.59337.peg.3437	None	None	None	fig|6666666.59345.peg.794	None	None	None	None	None	None	None	None	None	None

622	hypothetical protein	fig|6666666.59329.peg.6429	fig|6666666.59330.peg.5134	None	None	None	None	None	None	None	None	None	None	fig|6666666.59342.peg.3577	None	None	fig|6666666.59345.peg.802	None	None	fig|6666666.59346.peg.4349	None	None	None	None	None	None	None

8013	Phage tail sheath monomer	None	None	None	None	None	None	None	None	None	fig|6666666.59339.peg.1115	None	None	None	None	None	None	None	None	None	None	fig|6666666.59349.peg.1993	fig|6666666.59350.peg.5393	fig|6666666.59351.peg.1470	None	None	fig|6666666.59354.peg.2846

4594	hypothetical protein	None	None	None	None	None	None	None	None	fig|6666666.59338.peg.4216	None	None	None	fig|6666666.59342.peg.1901	None	None	fig|6666666.59345.peg.301	fig|6666666.59341.peg.4937	fig|6666666.59347.peg.1583	None	None	None	None	None	None	None	None

10887	FIG01203110 hypothetical protein	None	None	fig|6666666.59331.peg.289	None	None	None	None	None	fig|6666666.59338.peg.385	None	None	fig|6666666.59337.peg.4759	None	None	None	None	None	fig|6666666.59347.peg.1366	fig|6666666.59346.peg.3875	None	None	None	None	None	None	None

3741	GTPase EC 3.6.1.-	fig|6666666.59329.peg.6211	None	None	None	fig|6666666.59334.peg.1687	None	None	None	None	None	None	None	fig|6666666.59342.peg.2630	None	None	fig|6666666.59345.peg.655	fig|6666666.59341.peg.6021	None	None	None	None	None	None	None	None	None

10461	Taurine transport ATP-binding protein TauB	None	None	None	None	None	None	None	None	None	None	None	None	None	fig|6666666.71181.peg.4299	fig|6666666.59344.peg.3116	None	None	None	None	None	None	fig|6666666.59350.peg.4838	None	fig|6666666.59353.peg.2259	fig|6666666.59355.peg.1952	None

7213	hypothetical protein	None	None	None	None	fig|6666666.59334.peg.2640	None	None	None	fig|6666666.59338.peg.1700	None	fig|6666666.59340.peg.2916	None	None	None	None	None	fig|6666666.59341.peg.5491	fig|6666666.59347.peg.2183	None	None	None	None	None	None	None	None

6816	putative flagellar motor switch protein	fig|6666666.59329.peg.3932	None	None	None	None	None	fig|6666666.59336.peg.469	None	None	None	fig|6666666.59340.peg.1579	fig|6666666.59337.peg.5790	fig|6666666.59342.peg.4513	None	None	None	None	None	None	None	None	None	None	None	None	None

10576	L-arabinose isomerase EC 5.3.1.4	None	None	None	fig|6666666.59332.peg.1997	None	None	None	None	None	None	None	None	None	fig|6666666.71181.peg.4978	fig|6666666.59344.peg.3870	None	None	None	None	None	None	None	None	fig|6666666.59353.peg.3997	fig|6666666.59355.peg.3914	None

8848	membrane protein	None	fig|6666666.59330.peg.1904	None	None	None	None	fig|6666666.59336.peg.3942	fig|6666666.59333.peg.4404	None	None	None	fig|6666666.59337.peg.250	None	None	None	fig|6666666.59345.peg.5250	None	None	None	None	None	None	None	None	None	None

8843	Periplasmic protein p19 involved in high-affinity Fe2+ transport	fig|6666666.59329.peg.2658	None	None	None	None	None	fig|6666666.59336.peg.912	None	None	None	None	None	fig|6666666.59342.peg.4529	None	None	fig|6666666.59345.peg.2898	fig|6666666.59341.peg.1658	None	None	None	None	None	None	None	None	None

8846	General secretion pathway protein K	fig|6666666.59329.peg.1229	None	fig|6666666.59331.peg.4992	None	None	None	None	None	None	fig|6666666.59339.peg.6209	None	fig|6666666.59337.peg.4252	fig|6666666.59342.peg.5968	None	None	None	None	None	None	None	None	None	None	None	None	None

691	Putative oxidoreductase	fig|6666666.59329.peg.3243	None	None	None	None	None	None	None	None	None	None	None	None	None	fig|6666666.59344.peg.3700	None	None	None	None	None	None	fig|6666666.59350.peg.3915	None	fig|6666666.59353.peg.3818	None	fig|6666666.59354.peg.1629

3534	FIG01200959 hypothetical protein	fig|6666666.59329.peg.595	fig|6666666.59330.peg.2051	None	None	None	None	None	fig|6666666.59333.peg.1755	None	None	None	fig|6666666.59337.peg.4321	None	None	None	fig|6666666.59345.peg.3913	None	None	None	None	None	None	None	None	None	None

8526	Invasin	None	None	None	None	None	None	None	None	None	None	None	fig|6666666.59337.peg.2672	None	fig|6666666.71181.peg.3063	fig|6666666.59344.peg.6111	None	None	None	None	None	None	None	None	fig|6666666.59353.peg.1598	fig|6666666.59355.peg.4287	None

8537	FIG01199865 hypothetical protein	None	None	fig|6666666.59331.peg.5751	fig|6666666.59332.peg.3089	None	fig|6666666.59335.peg.3202	None	None	None	None	None	None	None	None	None	None	None	None	fig|6666666.59346.peg.884	fig|6666666.59348.peg.1616	None	None	None	None	None	None

4603	5-oxo-L-prolinase, putative	None	None	None	None	None	fig|6666666.59335.peg.6218	None	None	fig|6666666.59338.peg.1989	fig|6666666.59339.peg.8554	None	None	None	None	None	None	fig|6666666.59341.peg.3293	fig|6666666.59347.peg.5064	None	None	None	None	None	None	None	None

6181	Adenylosuccinate synthetase EC 6.3.4.4	None	fig|6666666.59330.peg.3204	None	None	None	None	fig|6666666.59336.peg.2042	None	None	fig|6666666.59339.peg.8254	None	None	fig|6666666.59342.peg.3750	None	None	fig|6666666.59345.peg.1671	None	None	None	None	None	None	None	None	None	None

7237	hypothetical protein	None	None	fig|6666666.59331.peg.870	None	None	None	None	None	None	None	None	None	None	fig|6666666.71181.peg.5029	None	None	fig|6666666.59341.peg.2934	fig|6666666.59347.peg.1214	None	fig|6666666.59348.peg.3654	None	None	None	None	None	None

10561	Ethanolamine ammonia-lyase light chain EC 4.3.1.7	None	None	None	None	None	None	None	fig|6666666.59333.peg.2094	None	None	None	None	None	fig|6666666.71181.peg.1568	fig|6666666.59344.peg.2019	None	None	None	None	None	None	None	None	fig|6666666.59353.peg.2907	fig|6666666.59355.peg.2832	None

8737	Aerobic cobaltochelatase CobT subunit EC 6.6.1.2	None	None	None	fig|6666666.59332.peg.3966	None	fig|6666666.59335.peg.3664	None	None	None	fig|6666666.59339.peg.3298	None	None	None	None	None	None	fig|6666666.59341.peg.3211	None	None	None	None	fig|6666666.59350.peg.1058	None	None	None	None

5678	FIG01206232 hypothetical protein	fig|6666666.59329.peg.2185	fig|6666666.59330.peg.3237	None	None	None	None	fig|6666666.59336.peg.1668	None	None	None	None	fig|6666666.59337.peg.2148	None	None	None	fig|6666666.59345.peg.3960	None	None	None	None	None	None	None	None	None	None

6434	hypothetical protein	fig|6666666.59329.peg.6250	None	None	None	None	None	None	fig|6666666.59333.peg.2045	None	None	None	fig|6666666.59337.peg.4596	fig|6666666.59342.peg.4251	None	None	fig|6666666.59345.peg.1333	None	None	None	None	None	None	None	None	None	None

6431	Voltage-gated sodium channel subunit	None	None	None	None	None	None	None	None	fig|6666666.59338.peg.3642	None	None	None	None	None	None	None	None	None	None	None	fig|6666666.59349.peg.4652	fig|6666666.59350.peg.248	fig|6666666.59351.peg.1535	None	None	fig|6666666.59354.peg.2431

8199	hypothetical protein	None	None	None	None	None	fig|6666666.59335.peg.2990	fig|6666666.59336.peg.2016	None	None	fig|6666666.59339.peg.4592	None	None	fig|6666666.59342.peg.6026	None	None	None	None	None	fig|6666666.59346.peg.2836	None	None	None	None	None	None	None

233	Beta-glucanase precursor EC 3.2.1.73	None	None	fig|6666666.59331.peg.692	None	None	None	None	None	fig|6666666.59338.peg.390	None	None	None	None	None	None	None	None	None	fig|6666666.59346.peg.4361	None	fig|6666666.59349.peg.2040	None	fig|6666666.59351.peg.3788	None	None	None

2534	putative membrane protein	fig|6666666.59329.peg.4464	None	fig|6666666.59331.peg.1787	None	None	None	fig|6666666.59336.peg.3508	None	None	fig|6666666.59339.peg.2399	None	None	fig|6666666.59342.peg.5619	None	None	None	None	None	None	None	None	None	None	None	None	None

5072	putative integral membrane protein	None	fig|6666666.59330.peg.4133	None	None	None	None	fig|6666666.59336.peg.3473	fig|6666666.59333.peg.5043	None	None	None	fig|6666666.59337.peg.3193	None	None	None	fig|6666666.59345.peg.2915	None	None	None	None	None	None	None	None	None	None

9561	FIG01055678 hypothetical protein	None	None	None	None	None	None	None	None	None	None	None	None	None	fig|6666666.71181.peg.4339	fig|6666666.59344.peg.5926	None	None	None	None	None	None	fig|6666666.59350.peg.982	None	fig|6666666.59353.peg.5005	fig|6666666.59355.peg.5103	None

5389	5-nucleotidase SurE EC 3.1.3.5 Exopolyphosphatase EC 3.6.1.11	None	None	None	None	None	None	fig|6666666.59336.peg.4463	None	fig|6666666.59338.peg.219	None	None	None	fig|6666666.59342.peg.1271	None	None	None	None	None	None	None	None	None	fig|6666666.59351.peg.5474	None	None	fig|6666666.59354.peg.3145

7144	Alkylated DNA repair protein AlkB	None	None	None	None	None	None	None	None	None	None	None	None	None	fig|6666666.71181.peg.1736	fig|6666666.59344.peg.6130	None	None	None	None	None	None	fig|6666666.59350.peg.5221	None	fig|6666666.59353.peg.3677	fig|6666666.59355.peg.4940	None

4973	Spermidine synthase-like protein	None	None	None	None	None	None	None	None	None	None	None	None	None	fig|6666666.71181.peg.3121	None	None	None	None	None	None	fig|6666666.59349.peg.1114	None	fig|6666666.59351.peg.67	fig|6666666.59353.peg.3351	fig|6666666.59355.peg.3444	None

7492	Response regulator CheB receptor modification enzyme, protein-glutamate methylesterase	None	None	None	None	fig|6666666.59334.peg.5486	None	None	None	None	None	None	None	None	None	None	None	None	None	None	None	fig|6666666.59349.peg.1314	fig|6666666.59350.peg.3477	fig|6666666.59351.peg.4535	None	None	fig|6666666.59354.peg.141

1573	FIG023677 hypothetical protein	None	None	fig|6666666.59331.peg.2278	None	None	None	None	None	fig|6666666.59338.peg.5211	None	None	None	None	None	None	None	None	None	None	fig|6666666.59348.peg.47	fig|6666666.59349.peg.4594	None	None	None	None	fig|6666666.59354.peg.4167

1571	Lysine decarboxylase family	None	None	None	None	None	None	None	None	None	None	None	None	None	fig|6666666.71181.peg.4900	fig|6666666.59344.peg.2906	None	None	None	None	None	None	fig|6666666.59350.peg.4344	None	fig|6666666.59353.peg.1421	fig|6666666.59355.peg.1970	None

10352	Uncharacterized fimbrial chaperone YehC precursor	None	None	None	None	None	None	None	None	None	None	None	None	None	fig|6666666.71181.peg.310	fig|6666666.59344.peg.1244	fig|6666666.59345.peg.2850	None	None	None	None	None	None	None	fig|6666666.59353.peg.4238	fig|6666666.59355.peg.4199	None

6252	Guanine-hypoxanthine permease	None	None	None	None	None	None	None	None	None	None	None	None	None	fig|6666666.71181.peg.3989	fig|6666666.59344.peg.5638	None	fig|6666666.59341.peg.4353	None	None	None	None	None	None	fig|6666666.59353.peg.2968	fig|6666666.59355.peg.2873	None

8540	Tricorn protease N-terminal domain-containing protein	None	fig|6666666.59330.peg.5069	None	None	None	fig|6666666.59335.peg.2680	None	fig|6666666.59333.peg.3645	None	None	None	fig|6666666.59337.peg.4812	None	None	None	fig|6666666.59345.peg.3361	None	None	None	None	None	None	None	None	None	None

8543	C0840 Methyl-accepting chemotaxis protein	None	None	None	None	None	None	None	None	None	fig|6666666.59339.peg.1118	None	None	None	fig|6666666.71181.peg.2888	fig|6666666.59344.peg.4376	None	None	None	None	None	None	None	None	fig|6666666.59353.peg.4289	fig|6666666.59355.peg.4414	None

11288	ClpB protein	fig|6666666.59329.peg.5913	None	None	None	None	None	None	None	fig|6666666.59338.peg.1729	None	None	fig|6666666.59337.peg.4818	fig|6666666.59342.peg.2713	None	None	None	None	None	None	fig|6666666.59348.peg.3064	None	None	None	None	None	None

1720	Secreted trypsin-like serine protease	fig|6666666.59329.peg.649	None	None	None	None	None	None	fig|6666666.59333.peg.4137	None	None	None	fig|6666666.59337.peg.728	fig|6666666.59342.peg.5746	None	None	fig|6666666.59345.peg.3661	None	None	None	None	None	None	None	None	None	None

5481	Adenylylsulfate kinase EC 2.7.1.25	None	None	fig|6666666.59331.peg.4380	None	None	fig|6666666.59335.peg.4595	None	None	None	fig|6666666.59339.peg.8312	None	None	None	None	fig|6666666.59344.peg.2207	None	None	None	fig|6666666.59346.peg.1964	None	None	None	None	None	None	None

5658	putative cytochrome P450 hydroxylase	None	None	None	None	None	None	None	None	None	None	None	None	None	fig|6666666.71181.peg.1653	fig|6666666.59344.peg.643	None	None	None	None	None	None	fig|6666666.59350.peg.1515	None	fig|6666666.59353.peg.1022	fig|6666666.59355.peg.3596	None

3919	helix-turn-helix domain protein	fig|6666666.59329.peg.1196	None	None	None	None	None	fig|6666666.59336.peg.6128	None	None	None	None	fig|6666666.59337.peg.5564	fig|6666666.59342.peg.3428	None	None	fig|6666666.59345.peg.4591	None	None	None	None	None	None	None	None	None	None

217	FIG01205628 hypothetical protein	fig|6666666.59329.peg.3847	fig|6666666.59330.peg.749	None	None	None	None	fig|6666666.59336.peg.4778	None	None	None	None	fig|6666666.59337.peg.3479	fig|6666666.59342.peg.4079	None	None	None	None	None	None	None	None	None	None	None	None	None

3649	Uncharacterized protein conserved in bacteria	None	None	None	None	None	fig|6666666.59335.peg.3949	None	None	None	None	None	None	None	fig|6666666.71181.peg.1307	fig|6666666.59344.peg.2945	None	None	None	None	None	None	None	None	fig|6666666.59353.peg.3293	fig|6666666.59355.peg.3160	None

3644	Metal dependent hydrolase	None	fig|6666666.59330.peg.4204	None	None	None	None	fig|6666666.59336.peg.4971	None	None	None	None	fig|6666666.59337.peg.5571	fig|6666666.59342.peg.3171	None	None	fig|6666666.59345.peg.983	None	None	None	None	None	None	None	None	None	None

914	BatD	fig|6666666.59329.peg.5588	fig|6666666.59330.peg.3803	None	None	None	None	None	fig|6666666.59333.peg.2092	None	None	None	None	fig|6666666.59342.peg.6113	None	None	None	None	None	None	fig|6666666.59348.peg.2275	None	None	None	None	None	None

7166	Chromosome plasmid partitioning protein ParA	None	None	None	None	None	None	None	None	fig|6666666.59338.peg.5650	fig|6666666.59339.peg.6127	None	None	None	None	None	None	None	None	None	fig|6666666.59348.peg.3515	None	fig|6666666.59350.peg.7433	None	None	None	fig|6666666.59354.peg.4865

1215	Protein containing plastocyanin azurin family domain	None	None	None	None	None	fig|6666666.59335.peg.3715	None	None	None	fig|6666666.59339.peg.3644	None	None	None	None	None	None	None	None	None	None	fig|6666666.59349.peg.3629	None	fig|6666666.59351.peg.3865	None	None	fig|6666666.59354.peg.635

8268	Altronate hydrolase EC 4.2.1.7	None	None	None	None	None	None	None	None	None	None	None	None	None	fig|6666666.71181.peg.4390	fig|6666666.59344.peg.5656	None	None	fig|6666666.59347.peg.59	None	None	None	None	None	fig|6666666.59353.peg.2391	fig|6666666.59355.peg.1664	None

4721	Outer membrane protein C precursor	None	None	None	None	None	fig|6666666.59335.peg.2842	None	fig|6666666.59333.peg.1695	None	fig|6666666.59339.peg.4853	None	None	fig|6666666.59342.peg.5591	None	fig|6666666.59344.peg.2603	None	None	None	None	None	None	None	None	None	None	None

4782	Branched-chain acyl-CoA dehydrogenase EC 1.3.99.12	None	fig|6666666.59330.peg.5101	None	None	None	None	None	fig|6666666.59333.peg.4040	fig|6666666.59338.peg.5538	fig|6666666.59339.peg.4627	None	fig|6666666.59337.peg.1466	None	None	None	None	None	None	None	None	None	None	None	None	None	None

11260	FIG01201207 hypothetical protein	None	fig|6666666.59330.peg.4083	None	None	None	None	fig|6666666.59336.peg.6211	fig|6666666.59333.peg.1024	None	None	None	fig|6666666.59337.peg.2134	None	None	None	fig|6666666.59345.peg.3949	None	None	None	None	None	None	None	None	None	None

10450	N-3-oxooctanoyl-L-homoserine lactone synthase N-3-oxohexanoyl-L-homoserine lactone synthase	None	None	None	None	None	None	None	None	None	None	None	None	None	fig|6666666.71181.peg.3537	fig|6666666.59344.peg.2078	None	None	None	None	None	None	None	fig|6666666.59351.peg.186	fig|6666666.59353.peg.3115	fig|6666666.59355.peg.482	None

191	Flagellar hook-length control protein FliK	fig|6666666.59329.peg.188	fig|6666666.59330.peg.1873	None	None	None	None	None	fig|6666666.59333.peg.4561	None	None	None	fig|6666666.59337.peg.2964	None	None	None	fig|6666666.59345.peg.5338	None	None	None	None	None	None	None	None	None	None

10100	Alpha-mannosidase EC 3.2.1.24	None	None	None	fig|6666666.59332.peg.4753	fig|6666666.59334.peg.4153	None	fig|6666666.59336.peg.2943	None	None	None	None	None	fig|6666666.59342.peg.769	None	None	None	fig|6666666.59341.peg.2044	None	None	None	None	None	None	None	None	None

2761	TrkA, Potassium channel-family protein	fig|6666666.59329.peg.763	None	None	fig|6666666.59332.peg.4557	fig|6666666.59334.peg.2873	None	None	None	None	fig|6666666.59339.peg.3509	None	None	None	None	None	None	None	None	fig|6666666.59346.peg.5237	None	None	None	None	None	None	None

7903	DinB family protein	None	None	None	None	None	None	None	None	fig|6666666.59338.peg.1833	None	None	None	None	None	None	None	None	None	None	None	fig|6666666.59349.peg.3747	fig|6666666.59350.peg.5816	fig|6666666.59351.peg.3561	None	None	fig|6666666.59354.peg.5430

7904	putative lipoprotein	None	None	fig|6666666.59331.peg.2098	None	None	None	None	None	None	None	None	None	None	None	None	None	None	None	fig|6666666.59346.peg.1452	None	fig|6666666.59349.peg.1795	None	fig|6666666.59351.peg.995	None	None	fig|6666666.59354.peg.1798

6271	hypothetical protein	fig|6666666.59329.peg.4525	None	None	None	None	None	None	None	None	None	None	None	None	fig|6666666.71181.peg.2862	fig|6666666.59344.peg.4401	None	None	None	None	None	None	None	None	fig|6666666.59353.peg.4313	fig|6666666.59355.peg.4389	None

6272	Beta-glucosidase EC 3.2.1.21	None	None	fig|6666666.59331.peg.138	None	None	None	None	None	fig|6666666.59338.peg.3385	None	None	fig|6666666.59337.peg.4763	None	None	None	None	None	fig|6666666.59347.peg.1373	fig|6666666.59346.peg.2867	None	None	None	None	None	None	None

6478	Homoserine O-acetyltransferase EC 2.3.1.31	None	None	None	None	None	fig|6666666.59335.peg.2270	None	None	None	fig|6666666.59339.peg.2905	None	None	None	fig|6666666.71181.peg.5159	None	None	None	None	None	None	fig|6666666.59349.peg.1108	None	None	None	None	fig|6666666.59354.peg.4499

6471	O-succinylbenzoate synthase EC 4.2.1.113	None	None	None	None	None	None	fig|6666666.59336.peg.2472	None	None	fig|6666666.59339.peg.3234	None	fig|6666666.59337.peg.1041	fig|6666666.59342.peg.3964	None	None	fig|6666666.59345.peg.4241	None	None	None	None	None	None	None	None	None	None

7756	2-dehydro-3-deoxygluconate kinase EC 2.7.1.45	fig|6666666.59329.peg.5098	None	fig|6666666.59331.peg.127	None	None	None	fig|6666666.59336.peg.5897	None	None	None	None	None	None	None	None	None	None	None	fig|6666666.59346.peg.2855	fig|6666666.59348.peg.684	None	None	None	None	None	None

3934	FIG01204434 hypothetical protein	None	None	None	None	None	None	fig|6666666.59336.peg.1237	None	None	None	None	fig|6666666.59337.peg.2893	fig|6666666.59342.peg.3314	None	None	fig|6666666.59345.peg.1000	None	None	fig|6666666.59346.peg.4760	None	None	None	None	None	None	None

3930	Lipopolysaccharide biosynthesis glycosyltransferase	None	None	None	fig|6666666.59332.peg.288	None	fig|6666666.59335.peg.2002	None	None	None	fig|6666666.59339.peg.6300	fig|6666666.59340.peg.2657	None	fig|6666666.59342.peg.5928	None	None	None	None	None	None	None	None	None	None	None	None	None

4044	YpfJ protein, zinc metalloprotease superfamily	None	None	None	None	None	None	None	None	None	None	None	fig|6666666.59337.peg.5148	None	fig|6666666.71181.peg.3249	fig|6666666.59344.peg.4002	None	None	None	None	None	None	None	None	fig|6666666.59353.peg.4047	fig|6666666.59355.peg.4004	None

3666	Sensory box GGDEF family protein	None	fig|6666666.59330.peg.1539	fig|6666666.59331.peg.3362	None	None	None	None	None	None	fig|6666666.59339.peg.8535	None	None	None	None	None	None	None	None	fig|6666666.59346.peg.605	fig|6666666.59348.peg.2097	None	None	None	None	None	None

2578	Phage Mu-like virion morphogenesis protein	None	None	None	None	None	None	None	None	None	None	None	None	None	fig|6666666.71181.peg.2876	fig|6666666.59344.peg.4389	None	None	None	None	None	fig|6666666.59349.peg.1234	None	None	fig|6666666.59353.peg.4301	fig|6666666.59355.peg.4402	None

10476	Secreted alkaline metalloproteinase EC 3.4.24.- , PrtA B C G homolog	None	None	None	None	None	None	None	None	None	None	None	None	None	fig|6666666.71181.peg.4747	fig|6666666.59344.peg.5898	None	None	None	None	None	None	fig|6666666.59350.peg.6414	None	fig|6666666.59353.peg.5054	fig|6666666.59355.peg.5082	None

8924	Nitrilotriacetate monooxygenase component B EC 1.14.13.-	None	None	None	None	None	None	None	None	None	None	None	None	None	fig|6666666.71181.peg.3576	fig|6666666.59344.peg.3950	None	None	None	None	None	None	fig|6666666.59350.peg.877	None	fig|6666666.59353.peg.4397	fig|6666666.59355.peg.3056	None

1685	Exoribonuclease II EC 3.1.13.1	None	None	None	None	None	fig|6666666.59335.peg.623	fig|6666666.59336.peg.6532	fig|6666666.59333.peg.3071	None	fig|6666666.59339.peg.4273	None	None	None	None	None	None	None	None	None	fig|6666666.59348.peg.3046	None	None	None	None	None	None

4213	D-Galactonate repressor DgoR	None	None	None	None	None	None	None	None	None	None	None	None	None	fig|6666666.71181.peg.630	fig|6666666.59344.peg.4183	None	None	None	None	None	None	fig|6666666.59350.peg.2103	None	fig|6666666.59353.peg.3049	fig|6666666.59355.peg.2297	None

442	MSHA pilin protein MshA	None	None	None	fig|6666666.59332.peg.5450	None	None	fig|6666666.59336.peg.4814	fig|6666666.59333.peg.3656	None	None	None	fig|6666666.59337.peg.4056	None	None	None	fig|6666666.59345.peg.3403	None	None	None	None	None	None	None	None	None	None

7292	FIG00638990 hypothetical protein	fig|6666666.59329.peg.6038	None	None	None	None	None	None	None	None	None	None	None	None	fig|6666666.71181.peg.2871	fig|6666666.59344.peg.4393	None	None	None	None	None	None	None	None	fig|6666666.59353.peg.4305	fig|6666666.59355.peg.4397	None

10340	Putative ABC transporter periplasmic binding protein	None	None	None	None	None	None	None	fig|6666666.59333.peg.973	None	None	None	None	None	fig|6666666.71181.peg.3585	fig|6666666.59344.peg.5566	None	None	None	None	None	None	None	None	fig|6666666.59353.peg.4131	fig|6666666.59355.peg.4284	None

4842	Type III secretion low calcium response protein LcrR	None	fig|6666666.59330.peg.3285	None	None	None	None	None	fig|6666666.59333.peg.4287	None	None	None	fig|6666666.59337.peg.2806	fig|6666666.59342.peg.1915	None	None	fig|6666666.59345.peg.1456	None	None	None	None	None	None	None	None	None	None

5204	Site-specific recombinase, phage integrase family	None	None	None	None	None	fig|6666666.59335.peg.329	None	None	None	None	fig|6666666.59340.peg.421	None	None	None	None	None	None	fig|6666666.59347.peg.4908	None	fig|6666666.59348.peg.1733	fig|6666666.59349.peg.91	None	None	None	None	None

3048	Branched-chain amino acid aminotransferase EC 2.6.1.42	None	None	None	None	fig|6666666.59334.peg.3	None	None	None	None	fig|6666666.59339.peg.8064	None	None	None	None	None	None	None	None	None	None	None	fig|6666666.59350.peg.3732	fig|6666666.59351.peg.2213	None	fig|6666666.59355.peg.5717	None

1096	Alpha-acetolactate decarboxylase EC 4.1.1.5	None	None	None	None	None	None	None	fig|6666666.59333.peg.4126	None	None	None	None	None	fig|6666666.71181.peg.1564	fig|6666666.59344.peg.2023	None	None	None	None	None	None	None	None	fig|6666666.59353.peg.2903	fig|6666666.59355.peg.2836	None

1328	Predicted signal transduction protein	fig|6666666.59329.peg.3463	None	None	None	None	None	fig|6666666.59336.peg.6091	None	None	fig|6666666.59339.peg.3525	None	None	None	None	None	None	None	None	None	None	None	None	fig|6666666.59351.peg.997	None	None	fig|6666666.59354.peg.1166

7734	Maltose maltodextrin ABC transporter, permease protein MalF	None	None	None	None	None	None	None	fig|6666666.59333.peg.998	fig|6666666.59338.peg.4516	None	None	fig|6666666.59337.peg.2428	fig|6666666.59342.peg.1364	None	None	None	fig|6666666.59341.peg.1968	None	None	None	None	None	None	None	None	None

8353	Prepilin-type N-terminal cleavage methylation domain-containing protein	fig|6666666.59329.peg.2946	None	None	None	None	None	fig|6666666.59336.peg.2139	fig|6666666.59333.peg.4618	None	None	None	fig|6666666.59337.peg.1239	None	None	None	fig|6666666.59345.peg.4424	None	None	None	None	None	None	None	None	None	None

1546	Fibronectin type III domain protein	None	fig|6666666.59330.peg.4992	None	fig|6666666.59332.peg.5071	None	fig|6666666.59335.peg.6117	None	None	None	fig|6666666.59339.peg.5737	None	None	None	None	None	None	None	None	None	None	None	None	None	None	None	fig|6666666.59354.peg.6439

4065	hypothetical protein	fig|6666666.59329.peg.2004	None	None	fig|6666666.59332.peg.5551	None	None	None	None	None	None	None	fig|6666666.59337.peg.5688	fig|6666666.59342.peg.6121	None	None	None	fig|6666666.59341.peg.3876	None	None	None	None	None	None	None	None	None

717	Threonine dehydratase, catabolic EC 4.3.1.19	None	None	None	None	None	None	None	None	None	None	None	None	None	fig|6666666.71181.peg.1277	fig|6666666.59344.peg.121	None	None	None	None	None	None	fig|6666666.59350.peg.6068	None	fig|6666666.59353.peg.441	fig|6666666.59355.peg.2168	None

8904	hypothetical protein	None	None	None	None	None	None	None	None	None	fig|6666666.59339.peg.716	None	None	fig|6666666.59342.peg.3006	None	None	fig|6666666.59345.peg.225	None	None	None	None	fig|6666666.59349.peg.2138	None	None	None	None	fig|6666666.59354.peg.1979

1669	Signal peptidase I EC 3.4.21.89	None	None	None	None	None	None	None	fig|6666666.59333.peg.2807	None	fig|6666666.59339.peg.7366	fig|6666666.59340.peg.849	None	None	None	None	None	None	None	None	None	None	None	None	fig|6666666.59353.peg.2651	fig|6666666.59355.peg.1831	None

5417	CRISPR-associated protein	None	fig|6666666.59330.peg.3733	None	None	fig|6666666.59334.peg.706	None	None	None	None	None	None	fig|6666666.59337.peg.4208	fig|6666666.59342.peg.25	None	None	fig|6666666.59345.peg.1636	None	None	None	None	None	None	None	None	None	None

4212	Putative ABC transporter membrane protein	None	None	None	None	None	None	None	fig|6666666.59333.peg.972	None	None	None	None	None	fig|6666666.71181.peg.3586	fig|6666666.59344.peg.5565	None	None	None	None	None	None	None	None	fig|6666666.59353.peg.4132	fig|6666666.59355.peg.4283	None

9530	FIG01199698 hypothetical protein	None	None	fig|6666666.59331.peg.6017	fig|6666666.59332.peg.3633	None	fig|6666666.59335.peg.4244	None	None	None	fig|6666666.59339.peg.1709	None	None	None	None	None	None	None	None	fig|6666666.59346.peg.2971	None	None	None	None	None	None	None

2019	L-sorbosone dehydrogenase	None	None	None	None	None	None	None	None	None	None	None	None	None	fig|6666666.71181.peg.1310	fig|6666666.59344.peg.2942	None	None	None	None	None	fig|6666666.59349.peg.822	None	None	fig|6666666.59353.peg.3289	fig|6666666.59355.peg.3163	None

7710	putative orphan protein	None	None	None	None	None	None	None	fig|6666666.59333.peg.2984	None	None	None	None	None	None	None	None	None	None	None	None	fig|6666666.59349.peg.2293	fig|6666666.59350.peg.1668	fig|6666666.59351.peg.3270	None	None	fig|6666666.59354.peg.5046

7719	Probable type IV pilus assembly FimV-related transmembrane protein	fig|6666666.59329.peg.4655	fig|6666666.59330.peg.1597	None	None	fig|6666666.59334.peg.1829	None	None	None	fig|6666666.59338.peg.1857	None	None	None	None	None	None	None	None	None	None	None	None	None	None	None	None	fig|6666666.59354.peg.2622

9435	GGDEF family protein	None	None	fig|6666666.59331.peg.5371	fig|6666666.59332.peg.2698	None	None	None	None	None	None	None	None	None	None	None	None	None	None	None	None	fig|6666666.59349.peg.485	None	fig|6666666.59351.peg.1656	None	None	fig|6666666.59354.peg.5341

4008	Polyhydroxyalkanoic acid synthase	None	None	fig|6666666.59331.peg.3254	fig|6666666.59332.peg.4660	None	None	None	None	fig|6666666.59338.peg.5438	None	None	None	None	None	None	None	None	None	fig|6666666.59346.peg.1798	None	None	fig|6666666.59350.peg.6499	None	None	None	None

3979	Hydrolase, isochorismatase family	None	None	None	None	None	None	None	None	None	fig|6666666.59339.peg.1231	None	None	None	fig|6666666.71181.peg.2508	fig|6666666.59344.peg.4069	None	None	None	None	None	None	None	None	fig|6666666.59353.peg.2117	fig|6666666.59355.peg.2703	None

3622	Cs C3558	None	None	None	None	None	None	None	None	None	None	None	None	None	fig|6666666.71181.peg.495	fig|6666666.59344.peg.1710	None	None	None	None	None	None	fig|6666666.59350.peg.5192	None	fig|6666666.59353.peg.1123	fig|6666666.59355.peg.353	None

4475	protein of unknown function DUF885	None	None	None	None	None	fig|6666666.59335.peg.6089	None	None	None	fig|6666666.59339.peg.7073	None	None	None	None	None	None	None	None	None	None	fig|6666666.59349.peg.3409	None	fig|6666666.59351.peg.5004	None	None	fig|6666666.59354.peg.2219

4476	Lipopolysaccharide biosynthesis protein RffC	None	None	None	None	None	fig|6666666.59335.peg.491	None	None	None	None	None	None	None	fig|6666666.71181.peg.2447	fig|6666666.59344.peg.2468	None	None	None	None	None	None	None	None	fig|6666666.59353.peg.3646	fig|6666666.59355.peg.4016	None

2468	Type III secretion spans bacterial envelope protein YscG	fig|6666666.59329.peg.2730	None	None	None	None	None	None	fig|6666666.59333.peg.2994	None	None	None	fig|6666666.59337.peg.4466	fig|6666666.59342.peg.629	None	None	fig|6666666.59345.peg.1483	None	None	None	None	None	None	None	None	None	None

6319	Acetyl-coenzyme A carboxyl transferase beta chain EC 6.4.1.2	fig|6666666.59329.peg.3494	None	None	None	None	fig|6666666.59335.peg.3997	None	fig|6666666.59333.peg.5495	None	None	None	None	None	None	None	None	None	None	fig|6666666.59346.peg.53	None	None	fig|6666666.59350.peg.8200	None	None	None	None

2808	Bsu YqfO NIF3 CutA domain	None	None	None	fig|6666666.59332.peg.6049	fig|6666666.59334.peg.1684	fig|6666666.59335.peg.4026	None	None	None	fig|6666666.59339.peg.3819	None	None	None	None	None	None	None	None	fig|6666666.59346.peg.1515	None	None	None	None	None	None	None

2803	Methyl parathion hydrolase	None	None	None	None	None	None	None	None	None	None	None	None	None	fig|6666666.71181.peg.2628	fig|6666666.59344.peg.3897	None	None	None	None	None	None	fig|6666666.59350.peg.5801	None	fig|6666666.59353.peg.4681	fig|6666666.59355.peg.4065	None

5559	hypothetical protein	None	None	None	None	fig|6666666.59334.peg.5016	None	None	None	fig|6666666.59338.peg.4723	None	fig|6666666.59340.peg.3534	None	None	None	None	None	fig|6666666.59341.peg.4821	fig|6666666.59347.peg.592	None	None	None	None	None	None	None	None

8589	ClpB protein	fig|6666666.59329.peg.6496	None	None	None	None	None	None	fig|6666666.59333.peg.1944	None	None	None	fig|6666666.59337.peg.5881	fig|6666666.59342.peg.6208	None	fig|6666666.59344.peg.5425	None	None	None	None	None	None	None	None	None	None	None

5889	PilL protein	None	None	None	None	None	None	None	None	None	None	fig|6666666.59340.peg.565	None	None	fig|6666666.71181.peg.722	fig|6666666.59344.peg.697	None	None	None	None	None	None	None	None	fig|6666666.59353.peg.977	fig|6666666.59355.peg.2399	None

6552	ABC-type tungstate transport system, permease protein	None	fig|6666666.59330.peg.3812	None	None	None	None	fig|6666666.59336.peg.4580	fig|6666666.59333.peg.3239	None	None	None	fig|6666666.59337.peg.2718	None	None	None	fig|6666666.59345.peg.3197	None	None	None	None	None	None	None	None	None	None

352	putative membrane protein	None	None	None	None	None	None	None	None	fig|6666666.59338.peg.1894	None	None	None	None	None	None	None	None	fig|6666666.59347.peg.2489	None	None	fig|6666666.59349.peg.3213	None	fig|6666666.59351.peg.3750	None	None	fig|6666666.59354.peg.2454

7508	hypothetical protein	None	None	None	None	None	None	None	None	fig|6666666.59338.peg.417	None	fig|6666666.59340.peg.3399	None	None	None	None	None	fig|6666666.59341.peg.3165	fig|6666666.59347.peg.415	None	fig|6666666.59348.peg.4619	None	None	None	None	None	None

10110	Isopentenyl-diphosphate delta-isomerase, FMN-dependent EC 5.3.3.2	None	fig|6666666.59330.peg.2925	None	None	None	None	fig|6666666.59336.peg.4142	fig|6666666.59333.peg.4905	None	None	None	fig|6666666.59337.peg.3196	None	None	None	fig|6666666.59345.peg.2912	None	None	None	None	None	None	None	None	None	None

10111	Orotate phosphoribosyltransferase EC 2.4.2.10	None	None	None	None	None	None	None	None	fig|6666666.59338.peg.4347	None	fig|6666666.59340.peg.28	None	None	None	None	None	fig|6666666.59341.peg.6062	None	None	None	None	None	fig|6666666.59351.peg.333	None	None	fig|6666666.59354.peg.3332

9799	putative reductase	fig|6666666.59329.peg.5089	None	None	None	None	None	fig|6666666.59336.peg.4398	None	None	None	None	fig|6666666.59337.peg.3665	fig|6666666.59342.peg.3992	None	None	fig|6666666.59345.peg.1243	None	None	None	None	None	None	None	None	None	None

8394	hypothetical protein	None	None	None	None	None	fig|6666666.59335.peg.2520	None	None	fig|6666666.59338.peg.4674	fig|6666666.59339.peg.1927	None	None	None	None	None	None	None	fig|6666666.59347.peg.1577	fig|6666666.59346.peg.359	None	None	None	None	None	None	None

4391	Acyl-CoA thioesterase II EC 3.1.2.-	fig|6666666.59329.peg.464	None	None	fig|6666666.59332.peg.3482	None	None	fig|6666666.59336.peg.1951	None	None	None	fig|6666666.59340.peg.1675	None	None	None	None	None	None	None	None	None	None	None	None	None	fig|6666666.59355.peg.5367	None

478	Transcriptional regulator, ArsR family	None	None	None	None	None	None	None	None	None	None	None	None	None	fig|6666666.71181.peg.4619	fig|6666666.59344.peg.4872	None	None	None	None	None	None	fig|6666666.59350.peg.2538	None	fig|6666666.59353.peg.3456	fig|6666666.59355.peg.3266	None

11389	Putative ACR protein	None	None	None	None	None	None	None	None	None	None	None	None	None	fig|6666666.71181.peg.3505	fig|6666666.59344.peg.5527	None	fig|6666666.59341.peg.2967	None	None	None	None	None	None	fig|6666666.59353.peg.4083	fig|6666666.59355.peg.4159	None

298	hypothetical protein	None	None	None	None	None	None	None	None	None	None	None	None	None	fig|6666666.71181.peg.3245	fig|6666666.59344.peg.4579	None	fig|6666666.59341.peg.4445	None	None	None	None	None	None	fig|6666666.59353.peg.2095	fig|6666666.59355.peg.2727	None

2444	ABC-type antimicrobial peptide transport system, permease component	fig|6666666.59329.peg.2076	None	None	None	None	fig|6666666.59335.peg.4790	fig|6666666.59336.peg.1411	None	None	fig|6666666.59339.peg.3365	None	None	None	None	None	None	fig|6666666.59341.peg.4574	None	None	None	None	None	None	None	None	None

10140	Gamma-glutamyl phosphate reductase EC 1.2.1.41	fig|6666666.59329.peg.5831	None	None	None	fig|6666666.59334.peg.1667	None	None	None	None	None	None	None	fig|6666666.59342.peg.5144	None	fig|6666666.59344.peg.3534	None	None	None	None	None	None	fig|6666666.59350.peg.4617	None	None	None	None

4263	Transporter	fig|6666666.59329.peg.5737	None	None	None	None	None	None	fig|6666666.59333.peg.1707	None	None	fig|6666666.59340.peg.2489	None	None	None	None	None	None	None	None	None	fig|6666666.59349.peg.2319	None	None	None	None	fig|6666666.59354.peg.286

6578	Uncharacterized ABC transporter, permease component YrbE	None	None	None	None	fig|6666666.59334.peg.90	None	None	None	None	None	None	None	None	None	None	None	None	fig|6666666.59347.peg.3244	None	fig|6666666.59348.peg.3525	None	fig|6666666.59350.peg.7410	None	None	None	fig|6666666.59354.peg.344

6145	hypothetical protein	fig|6666666.59329.peg.1076	None	None	None	None	None	None	None	fig|6666666.59338.peg.4504	None	None	fig|6666666.59337.peg.320	fig|6666666.59342.peg.3891	None	None	None	None	fig|6666666.59347.peg.3428	None	None	None	None	None	None	None	None

6500	RNA polymerase sigma-54 factor RpoN	None	None	None	fig|6666666.59332.peg.2740	None	None	None	None	None	fig|6666666.59339.peg.8203	None	None	None	None	fig|6666666.59344.peg.1796	None	None	None	fig|6666666.59346.peg.640	None	None	None	None	None	fig|6666666.59355.peg.1608	None

2052	FIG01199729 hypothetical protein	fig|6666666.59329.peg.1329	None	None	None	None	None	None	fig|6666666.59333.peg.5420	None	None	None	fig|6666666.59337.peg.5730	fig|6666666.59342.peg.3484	None	None	fig|6666666.59345.peg.1473	None	None	None	None	None	None	None	None	None	None

4824	Rhamnogalacturonides degradation protein RhiN	None	None	None	None	None	None	None	None	None	None	None	None	None	fig|6666666.71181.peg.1519	fig|6666666.59344.peg.4991	None	None	None	None	None	None	fig|6666666.59350.peg.4517	None	fig|6666666.59353.peg.4876	fig|6666666.59355.peg.222	None

4532	Hypothetical lysR-family transcriptional regulator YdhB	None	None	None	None	None	None	fig|6666666.59336.peg.43	fig|6666666.59333.peg.15	None	fig|6666666.59339.peg.7996	None	None	None	None	None	fig|6666666.59345.peg.4952	fig|6666666.59341.peg.2125	None	None	None	None	None	None	None	None	None

7982	Probable lipoprotein	None	fig|6666666.59330.peg.3068	None	None	None	None	None	None	None	None	None	None	fig|6666666.59342.peg.1740	None	None	None	None	None	None	None	fig|6666666.59349.peg.2276	None	fig|6666666.59351.peg.4157	None	None	fig|6666666.59354.peg.4108

9408	Phytanoyl-CoA dioxygenase	None	None	None	None	None	None	None	None	fig|6666666.59338.peg.2786	None	None	None	None	None	None	None	fig|6666666.59341.peg.2629	fig|6666666.59347.peg.1598	None	None	fig|6666666.59349.peg.3272	None	None	None	None	fig|6666666.59354.peg.3787

7325	Glycerol dehydrogenase EC 1.1.1.6	None	None	None	None	None	None	None	fig|6666666.59333.peg.108	fig|6666666.59338.peg.3831	None	None	None	fig|6666666.59342.peg.2361	None	None	None	fig|6666666.59341.peg.1450	fig|6666666.59347.peg.4231	None	None	None	None	None	None	None	None

7062	probable MFS transporter	None	None	None	None	None	None	None	None	None	None	None	None	None	fig|6666666.71181.peg.1237	fig|6666666.59344.peg.176	None	None	None	None	None	None	fig|6666666.59350.peg.3847	None	fig|6666666.59353.peg.485	fig|6666666.59355.peg.2208	None

7060	Phage FluMu protein gp47	None	None	None	None	None	fig|6666666.59335.peg.301	None	None	None	fig|6666666.59339.peg.2542	None	None	None	fig|6666666.71181.peg.2894	None	None	None	None	None	None	None	None	None	fig|6666666.59353.peg.4284	fig|6666666.59355.peg.4419	None

11020	DNA topoisomerase I EC 5.99.1.2	None	None	None	None	None	None	None	fig|6666666.59333.peg.2551	fig|6666666.59338.peg.5486	None	None	None	None	None	None	None	fig|6666666.59341.peg.1003	None	fig|6666666.59346.peg.2530	fig|6666666.59348.peg.3006	None	None	None	None	None	None

1384	Lactose permease	None	None	None	None	fig|6666666.59334.peg.3988	None	None	None	None	None	None	None	None	fig|6666666.71181.peg.3879	fig|6666666.59344.peg.3303	None	None	None	None	None	None	None	None	fig|6666666.59353.peg.1241	fig|6666666.59355.peg.3691	None

419	Putative lipoprotein	fig|6666666.59329.peg.6158	None	None	None	None	None	fig|6666666.59336.peg.3020	fig|6666666.59333.peg.697	None	None	None	fig|6666666.59337.peg.3863	None	None	None	fig|6666666.59345.peg.306	None	None	None	None	None	None	None	None	None	None

6682	hypothetical protein	fig|6666666.59329.peg.1194	None	None	None	None	None	fig|6666666.59336.peg.4378	None	None	None	None	fig|6666666.59337.peg.5839	fig|6666666.59342.peg.6013	None	None	fig|6666666.59345.peg.4590	None	None	None	None	None	None	None	None	None	None

5464	1-deoxy-D-xylulose 5-phosphate reductoisomerase EC 1.1.1.267	fig|6666666.59329.peg.1576	None	None	None	fig|6666666.59334.peg.5276	None	None	None	None	None	None	None	None	None	None	None	None	None	None	None	fig|6666666.59349.peg.1370	fig|6666666.59350.peg.1880	None	None	None	fig|6666666.59354.peg.1337

2420	Mu-like prophage protein GP36	fig|6666666.59329.peg.4609	None	None	None	None	None	None	None	None	None	None	None	None	fig|6666666.71181.peg.2880	fig|6666666.59344.peg.4385	None	None	None	None	None	None	None	None	fig|6666666.59353.peg.4297	fig|6666666.59355.peg.4406	None

1971	Putative transport protein	None	None	fig|6666666.59331.peg.63	None	None	None	None	None	None	None	None	None	None	None	None	None	None	None	None	None	fig|6666666.59349.peg.2690	fig|6666666.59350.peg.1697	fig|6666666.59351.peg.773	None	None	fig|6666666.59354.peg.2588

11108	FIG01200650 hypothetical protein	fig|6666666.59329.peg.486	None	None	None	None	None	fig|6666666.59336.peg.2201	None	None	None	None	fig|6666666.59337.peg.1282	fig|6666666.59342.peg.4633	None	None	fig|6666666.59345.peg.4469	None	None	None	None	None	None	None	None	None	None

1902	FIG01202214 hypothetical protein	fig|6666666.59329.peg.1843	None	None	None	None	None	fig|6666666.59336.peg.3702	None	None	None	None	fig|6666666.59337.peg.5311	fig|6666666.59342.peg.4631	None	None	fig|6666666.59345.peg.4100	None	None	None	None	None	None	None	None	None	None

6168	Exodeoxyribonuclease encoded by cryptic prophage CP-933P	None	None	None	None	None	fig|6666666.59335.peg.323	None	None	None	None	None	None	None	fig|6666666.71181.peg.2955	fig|6666666.59344.peg.2876	None	None	None	None	None	None	None	None	fig|6666666.59353.peg.1449	fig|6666666.59355.peg.1998	None

10995	Sorbitol dehydrogenase EC 1.1.1.14	None	None	None	None	None	None	None	None	None	None	None	None	None	fig|6666666.71181.peg.3964	fig|6666666.59344.peg.6098	None	fig|6666666.59341.peg.5372	None	None	None	None	None	None	fig|6666666.59353.peg.2959	fig|6666666.59355.peg.2782	None

4513	FIG01199700 hypothetical protein	None	fig|6666666.59330.peg.3824	None	None	None	None	fig|6666666.59336.peg.5372	None	None	None	None	fig|6666666.59337.peg.2704	fig|6666666.59342.peg.4984	None	None	fig|6666666.59345.peg.3182	None	None	None	None	None	None	None	None	None	None

4517	FIG00642443 hypothetical protein	None	None	None	None	None	None	None	None	None	None	None	fig|6666666.59337.peg.5647	None	fig|6666666.71181.peg.2162	fig|6666666.59344.peg.3234	None	None	None	None	None	None	None	None	fig|6666666.59353.peg.2850	fig|6666666.59355.peg.3953	None

10946	hypothetical protein	None	None	fig|6666666.59331.peg.555	fig|6666666.59332.peg.6053	None	None	None	fig|6666666.59333.peg.2272	None	None	None	None	None	None	None	None	None	None	fig|6666666.59346.peg.192	fig|6666666.59348.peg.1000	None	None	None	None	None	None

9021	hypothetical protein	fig|6666666.59329.peg.2042	None	None	None	None	None	fig|6666666.59336.peg.6555	None	None	None	None	fig|6666666.59337.peg.1206	fig|6666666.59342.peg.2000	None	None	fig|6666666.59345.peg.25	None	None	None	None	None	None	None	None	None	None

7308	Hemolysins and related proteins containing CBS domains	None	fig|6666666.59330.peg.2840	None	fig|6666666.59332.peg.6004	fig|6666666.59334.peg.259	None	None	None	None	None	None	fig|6666666.59337.peg.2037	None	None	None	fig|6666666.59345.peg.2437	None	None	None	None	None	None	None	None	None	None

10060	Peptide deformylase EC 3.5.1.88	None	None	None	None	None	fig|6666666.59335.peg.2257	None	None	None	None	None	None	fig|6666666.59342.peg.5774	None	None	None	None	None	None	fig|6666666.59348.peg.1001	None	fig|6666666.59350.peg.1376	fig|6666666.59351.peg.2947	None	None	None

9491	TPR domain protein in aerotolerance operon	fig|6666666.59329.peg.5584	None	fig|6666666.59331.peg.5148	fig|6666666.59332.peg.6262	None	None	None	fig|6666666.59333.peg.5429	None	None	None	None	None	None	None	None	None	None	fig|6666666.59346.peg.4941	None	None	None	None	None	None	None

7693	Ferric aerobactin ABC transporter, periplasmic substrate binding protein	None	None	None	None	None	fig|6666666.59335.peg.2651	fig|6666666.59336.peg.4745	fig|6666666.59333.peg.3514	None	fig|6666666.59339.peg.1809	None	None	None	None	None	None	None	None	None	fig|6666666.59348.peg.1345	None	None	None	None	None	None

4358	DNA sulfur modification protein DndD	None	None	None	None	None	None	None	None	None	None	None	None	None	fig|6666666.71181.peg.692	fig|6666666.59344.peg.663	None	None	fig|6666666.59347.peg.4244	None	None	None	None	None	fig|6666666.59353.peg.1005	fig|6666666.59355.peg.2367	None

11001	Cro-like protein	None	None	None	None	None	None	None	None	None	None	fig|6666666.59340.peg.3442	None	None	fig|6666666.71181.peg.91	fig|6666666.59344.peg.3171	None	None	None	None	None	None	None	None	fig|6666666.59353.peg.1922	fig|6666666.59355.peg.1485	None

5708	FIG01202810 hypothetical protein	None	fig|6666666.59330.peg.4189	None	None	None	None	fig|6666666.59336.peg.2860	None	None	None	None	fig|6666666.59337.peg.4480	fig|6666666.59342.peg.5730	None	None	fig|6666666.59345.peg.2172	None	None	None	None	None	None	None	None	None	None

1437	Iron-regulated outer membrane protein	fig|6666666.59329.peg.753	None	fig|6666666.59331.peg.3680	fig|6666666.59332.peg.2580	None	None	None	None	None	None	None	None	None	None	None	fig|6666666.59345.peg.2081	None	None	fig|6666666.59346.peg.3197	None	None	None	None	None	None	None

9366	FIG01200794 hypothetical protein	fig|6666666.59329.peg.5323	None	None	None	None	None	None	None	None	None	None	fig|6666666.59337.peg.3956	None	None	None	fig|6666666.59345.peg.453	fig|6666666.59341.peg.5720	fig|6666666.59347.peg.2070	None	None	None	None	None	None	None	None

8985	FIG01203446 hypothetical protein	fig|6666666.59329.peg.3590	None	None	None	None	None	fig|6666666.59336.peg.1313	None	None	None	None	fig|6666666.59337.peg.264	fig|6666666.59342.peg.4170	None	None	fig|6666666.59345.peg.5239	None	None	None	None	None	None	None	None	None	None

8989	Family of unknown function DUF450 family	None	None	None	None	None	None	None	fig|6666666.59333.peg.3378	None	None	None	None	None	fig|6666666.71181.peg.2206	fig|6666666.59344.peg.3582	None	None	None	None	None	None	None	None	fig|6666666.59353.peg.839	fig|6666666.59355.peg.865	None

4985	Cellulose synthase catalytic subunit [UDP-forming] EC 2.4.1.12	None	None	None	None	None	None	fig|6666666.59336.peg.6527	None	None	None	fig|6666666.59340.peg.116	fig|6666666.59337.peg.4921	fig|6666666.59342.peg.87	None	None	fig|6666666.59345.peg.4373	None	None	None	None	None	None	None	None	None	None

4986	Putative protease ydgD EC 3.4.21.-	None	None	None	None	None	None	None	None	None	None	None	None	None	fig|6666666.71181.peg.1360	fig|6666666.59344.peg.570	None	None	None	None	None	None	fig|6666666.59350.peg.2419	None	fig|6666666.59353.peg.1873	fig|6666666.59355.peg.1140	None

1990	Putative cytoplasmic protein	None	None	None	None	None	None	None	None	fig|6666666.59338.peg.5587	None	fig|6666666.59340.peg.757	None	None	None	None	None	fig|6666666.59341.peg.3466	fig|6666666.59347.peg.359	None	None	None	fig|6666666.59350.peg.271	None	None	None	None

514	Unsaturated glucuronyl hydrolase EC 3.2.1.-	fig|6666666.59329.peg.3653	None	None	None	None	None	None	None	fig|6666666.59338.peg.4972	None	fig|6666666.59340.peg.651	None	None	None	None	None	fig|6666666.59341.peg.1269	fig|6666666.59347.peg.166	None	None	None	None	None	None	None	None

7568	hypothetical protein	fig|6666666.59329.peg.2218	None	None	None	None	None	fig|6666666.59336.peg.1039	None	None	None	None	fig|6666666.59337.peg.3399	fig|6666666.59342.peg.5408	None	None	fig|6666666.59345.peg.1993	None	None	None	None	None	None	None	None	None	None

10122	Undecaprenyl-diphosphatase EC 3.6.1.27	None	None	fig|6666666.59331.peg.2591	None	fig|6666666.59334.peg.528	None	None	fig|6666666.59333.peg.182	None	fig|6666666.59339.peg.7915	None	None	None	None	None	None	None	None	None	None	None	None	None	None	None	fig|6666666.59354.peg.6421

5935	LemA protein	fig|6666666.59329.peg.1386	None	None	None	None	None	None	None	fig|6666666.59338.peg.1699	None	None	fig|6666666.59337.peg.2110	None	None	None	fig|6666666.59345.peg.3347	None	fig|6666666.59347.peg.2184	None	None	None	None	None	None	None	None

3492	extensin-like protein	None	None	None	None	None	None	None	None	None	None	None	None	None	fig|6666666.71181.peg.347	fig|6666666.59344.peg.2349	None	None	None	None	None	None	fig|6666666.59350.peg.1821	None	fig|6666666.59353.peg.241	fig|6666666.59355.peg.1281	None

3491	Shikimate kinase III EC 2.7.1.71	None	None	None	None	None	None	None	None	None	None	None	None	None	fig|6666666.71181.peg.2190	fig|6666666.59344.peg.1155	None	None	None	None	None	None	fig|6666666.59350.peg.4725	None	fig|6666666.59353.peg.824	fig|6666666.59355.peg.882	None

8423	NADH-ubiquinone oxidoreductase chain B EC 1.6.5.3	None	None	None	None	None	None	None	None	None	None	None	None	None	fig|6666666.71181.peg.543	fig|6666666.59344.peg.1925	None	None	None	None	None	None	fig|6666666.59350.peg.5713	None	fig|6666666.59353.peg.1172	fig|6666666.59355.peg.303	None

7367	Lysyl-tRNA synthetase class II EC 6.1.1.6	None	None	None	fig|6666666.59332.peg.1362	None	fig|6666666.59335.peg.2115	None	None	None	None	None	fig|6666666.59337.peg.112	fig|6666666.59342.peg.2749	None	None	fig|6666666.59345.peg.3730	None	None	None	None	None	None	None	None	None	None

7028	FIG01201162 hypothetical protein	fig|6666666.59329.peg.5926	fig|6666666.59330.peg.3603	None	None	None	None	None	None	None	None	None	fig|6666666.59337.peg.3001	None	None	None	fig|6666666.59345.peg.635	None	None	None	fig|6666666.59348.peg.1801	None	None	None	None	None	None

9008	putative hemagglutinin hemolysin-related protein	fig|6666666.59329.peg.1414	None	None	None	None	None	fig|6666666.59336.peg.1334	fig|6666666.59333.peg.601	None	None	None	fig|6666666.59337.peg.3684	fig|6666666.59342.peg.4263	None	None	None	None	None	None	None	None	None	None	None	None	None

9832	FIG01056338 hypothetical protein	None	None	None	None	None	None	None	None	None	None	None	None	None	fig|6666666.71181.peg.4055	fig|6666666.59344.peg.3773	None	None	None	None	None	fig|6666666.59349.peg.4789	None	None	fig|6666666.59353.peg.3927	fig|6666666.59355.peg.4234	None

4375	FIG01062720 hypothetical protein	None	None	None	fig|6666666.59332.peg.2779	None	None	None	None	fig|6666666.59338.peg.4985	None	None	None	None	None	None	None	fig|6666666.59341.peg.3677	fig|6666666.59347.peg.3311	fig|6666666.59346.peg.1911	None	None	None	None	None	None	None

11065	cholesterol oxidase	None	None	None	None	None	None	fig|6666666.59336.peg.4264	None	None	None	None	None	fig|6666666.59342.peg.1319	None	None	fig|6666666.59345.peg.4703	None	None	None	None	fig|6666666.59349.peg.4858	None	None	None	None	fig|6666666.59354.peg.5478

11325	Nickel transport ATP-binding protein NikD TC 3.A.1.5.3	None	None	None	fig|6666666.59332.peg.2897	fig|6666666.59334.peg.1539	None	fig|6666666.59336.peg.5948	None	None	None	None	None	None	None	None	fig|6666666.59345.peg.1424	None	fig|6666666.59347.peg.3303	None	None	None	None	None	None	None	None

6641	probable membrane protein STY1534	None	None	None	None	None	None	None	None	None	None	None	None	None	fig|6666666.71181.peg.4708	fig|6666666.59344.peg.5789	None	None	None	None	None	None	fig|6666666.59350.peg.1761	None	fig|6666666.59353.peg.4951	fig|6666666.59355.peg.5034	None

10818	ABC-type amino acid transport signal transduction system	None	None	None	None	None	fig|6666666.59335.peg.1211	None	None	None	fig|6666666.59339.peg.4207	None	None	None	None	None	None	None	None	None	None	fig|6666666.59349.peg.2959	None	fig|6666666.59351.peg.3802	None	None	fig|6666666.59354.peg.3047

5693	Regulator of L-galactonate catabolism YjjM	None	None	None	None	None	None	None	None	None	None	None	None	None	fig|6666666.71181.peg.1129	fig|6666666.59344.peg.2251	None	None	fig|6666666.59347.peg.64	None	None	None	None	None	fig|6666666.59353.peg.4967	fig|6666666.59355.peg.5045	None

5121	Carbamate kinase EC 2.7.2.2	None	None	None	None	None	None	None	None	fig|6666666.59338.peg.2820	fig|6666666.59339.peg.6480	fig|6666666.59340.peg.3413	None	None	None	None	None	fig|6666666.59341.peg.4435	None	None	fig|6666666.59348.peg.2513	None	None	None	None	None	None

6880	predicted 4-deoxy-L-threo-5-hexosulose-uronate ketol-isomerase EC 5.3.1.17	None	None	None	None	fig|6666666.59334.peg.942	None	None	None	fig|6666666.59338.peg.5594	None	None	None	fig|6666666.59342.peg.3685	None	None	None	fig|6666666.59341.peg.1262	fig|6666666.59347.peg.185	None	None	None	None	None	None	None	None

8419	hypothetical protein	fig|6666666.59329.peg.2350	None	None	None	None	None	None	None	None	None	None	None	None	fig|6666666.71181.peg.1295	fig|6666666.59344.peg.2959	None	None	None	None	None	None	None	None	fig|6666666.59353.peg.3303	fig|6666666.59355.peg.3149	None

8265	FIG00953662 hypothetical protein	None	None	None	None	None	None	None	None	None	None	None	None	None	fig|6666666.71181.peg.3574	fig|6666666.59344.peg.3952	None	None	None	None	None	None	fig|6666666.59350.peg.879	None	fig|6666666.59353.peg.4395	fig|6666666.59355.peg.3058	None

5913	FIG01204347 hypothetical protein	fig|6666666.59329.peg.3888	fig|6666666.59330.peg.1785	None	fig|6666666.59332.peg.932	None	None	None	None	None	None	None	fig|6666666.59337.peg.2271	None	None	None	fig|6666666.59345.peg.2033	None	None	None	None	None	None	None	None	None	None

10512	Acetyltransferase EC 2.3.1.-	None	None	None	None	None	None	None	None	fig|6666666.59338.peg.1548	None	None	None	None	fig|6666666.71181.peg.2676	fig|6666666.59344.peg.4295	None	None	None	None	None	None	None	None	fig|6666666.59353.peg.3891	fig|6666666.59355.peg.3801	None

9771	Isoquinoline 1-oxidoreductase beta subunit EC 1.3.99.16	None	None	fig|6666666.59331.peg.4661	None	None	None	None	None	None	None	None	None	None	fig|6666666.71181.peg.4080	None	None	None	None	None	None	None	fig|6666666.59350.peg.891	None	fig|6666666.59353.peg.4840	fig|6666666.59355.peg.4910	None

6078	Putative transmembrane efflux protein	fig|6666666.59329.peg.5498	None	None	None	None	None	None	None	None	None	None	None	None	fig|6666666.71181.peg.2124	fig|6666666.59344.peg.5327	None	None	None	None	None	None	None	None	fig|6666666.59353.peg.715	fig|6666666.59355.peg.202	None

2612	NADH-ubiquinone oxidoreductase chain E EC 1.6.5.3	None	None	None	None	None	None	None	None	None	None	None	None	None	fig|6666666.71181.peg.541	fig|6666666.59344.peg.1927	None	None	None	None	None	None	fig|6666666.59350.peg.3985	None	fig|6666666.59353.peg.1170	fig|6666666.59355.peg.306	None

7348	Cytochrome c heme lyase subunit CcmH	None	None	fig|6666666.59331.peg.5875	None	None	None	None	fig|6666666.59333.peg.2601	fig|6666666.59338.peg.2231	None	fig|6666666.59340.peg.1116	None	None	None	None	None	None	fig|6666666.59347.peg.5098	None	None	None	None	None	None	None	None

7006	FIG01202521 hypothetical protein	fig|6666666.59329.peg.3240	None	None	None	fig|6666666.59334.peg.561	None	None	None	fig|6666666.59338.peg.177	None	None	None	fig|6666666.59342.peg.2280	None	None	fig|6666666.59345.peg.3165	None	None	None	None	None	None	None	None	None	None

747	Glucans biosynthesis glucosyltransferase H EC 2.4.1.-	None	None	None	None	None	None	None	None	None	None	None	None	None	fig|6666666.71181.peg.1587	fig|6666666.59344.peg.2395	None	None	None	None	None	None	fig|6666666.59350.peg.92	None	fig|6666666.59353.peg.279	fig|6666666.59355.peg.1318	None

9387	2- 5''-triphosphoribosyl -3'-dephosphocoenzyme-A synthase EC 2.7.8.25	None	None	None	None	fig|6666666.59334.peg.1771	None	fig|6666666.59336.peg.6458	None	None	None	None	None	fig|6666666.59342.peg.3711	fig|6666666.71181.peg.1729	None	None	None	None	None	None	None	None	None	fig|6666666.59353.peg.3684	None	None

3110	probable exported protein STY4558	None	None	None	None	None	None	None	None	None	None	fig|6666666.59340.peg.561	None	None	fig|6666666.71181.peg.720	fig|6666666.59344.peg.695	None	None	None	None	None	None	None	None	fig|6666666.59353.peg.979	fig|6666666.59355.peg.2397	None

8640	NADH-ubiquinone oxidoreductase chain I EC 1.6.5.3	None	None	None	None	None	None	None	None	None	None	None	None	None	fig|6666666.71181.peg.537	fig|6666666.59344.peg.1935	None	None	None	None	None	None	fig|6666666.59350.peg.2615	None	fig|6666666.59353.peg.1165	fig|6666666.59355.peg.310	None

5747	Vanillate O-demethylase oxygenase subunit EC 1.14.13.82	None	None	None	None	None	None	None	None	None	None	None	None	None	fig|6666666.71181.peg.4387	fig|6666666.59344.peg.4813	None	None	None	None	None	None	fig|6666666.59350.peg.1998	None	fig|6666666.59353.peg.4341	fig|6666666.59355.peg.4528	None

6666	ABC-type amino acid transport signal transduction system	None	None	None	fig|6666666.59332.peg.2837	None	None	None	fig|6666666.59333.peg.4738	None	None	None	fig|6666666.59337.peg.233	None	None	None	None	None	None	fig|6666666.59346.peg.4493	None	None	None	None	None	None	fig|6666666.59354.peg.5834

10875	Flagellar basal-body rod modification protein FlgD	None	None	None	fig|6666666.59332.peg.1793	None	None	None	None	None	fig|6666666.59339.peg.5313	None	None	None	None	None	None	fig|6666666.59341.peg.3254	None	None	None	fig|6666666.59349.peg.723	None	None	None	None	fig|6666666.59354.peg.1526

1876	Transport ATP-binding protein CydD	fig|6666666.59329.peg.6210	None	None	None	None	None	None	None	None	fig|6666666.59339.peg.3659	None	fig|6666666.59337.peg.4129	fig|6666666.59342.peg.1553	None	None	fig|6666666.59345.peg.4600	None	None	None	None	None	None	None	None	None	None

4943	N-formylglutamate deformylase EC 3.5.1.68	None	None	None	None	None	None	None	None	None	None	None	None	None	fig|6666666.71181.peg.1207	fig|6666666.59344.peg.208	None	None	None	None	None	None	fig|6666666.59350.peg.4117	None	fig|6666666.59353.peg.512	fig|6666666.59355.peg.2238	None

5109	hypothetical protein	None	None	fig|6666666.59331.peg.55	None	None	None	None	fig|6666666.59333.peg.5375	None	None	None	None	None	None	None	None	None	None	None	None	fig|6666666.59349.peg.1324	None	fig|6666666.59351.peg.4545	None	None	fig|6666666.59354.peg.154

5102	probable dioxygenase	None	None	None	None	None	None	None	None	None	None	None	None	None	fig|6666666.71181.peg.3696	fig|6666666.59344.peg.3288	None	None	None	None	None	None	fig|6666666.59350.peg.3069	None	fig|6666666.59353.peg.2791	fig|6666666.59355.peg.3705	None

3282	hypothetical protein	None	None	fig|6666666.59331.peg.3540	fig|6666666.59332.peg.1402	None	None	None	None	fig|6666666.59338.peg.865	None	None	None	None	None	None	fig|6666666.59345.peg.4163	None	fig|6666666.59347.peg.2539	None	None	None	None	None	None	None	None

6861	Cell division protein FtsJ Ribosomal RNA large subunit methyltransferase E EC 2.1.1.-	None	None	fig|6666666.59331.peg.1132	None	None	None	fig|6666666.59336.peg.227	None	fig|6666666.59338.peg.5976	fig|6666666.59339.peg.4857	fig|6666666.59340.peg.2859	None	None	None	None	None	None	None	None	None	None	None	None	None	None	None

8787	hypothetical protein	fig|6666666.59329.peg.6039	None	None	None	None	None	None	None	None	None	None	None	None	fig|6666666.71181.peg.2870	fig|6666666.59344.peg.4394	None	None	None	None	None	None	None	None	fig|6666666.59353.peg.4306	fig|6666666.59355.peg.4396	None

8435	Regulatory protein, LysR LysR, substrate-binding	None	None	fig|6666666.59331.peg.4574	fig|6666666.59332.peg.1597	None	fig|6666666.59335.peg.2455	fig|6666666.59336.peg.2676	None	None	None	None	None	None	None	None	None	None	None	fig|6666666.59346.peg.2763	None	None	None	None	None	None	None

8514	SSU ribosomal protein S7p S5e	None	None	None	None	None	fig|6666666.59335.peg.5582	None	None	None	None	None	None	None	fig|6666666.71181.peg.6423	fig|6666666.59344.peg.3132	None	None	None	None	None	None	None	None	fig|6666666.59353.peg.2274	fig|6666666.59355.peg.1967	None

8512	Permease of the drug metabolite transporter DMT superfamily	fig|6666666.59329.peg.5988	None	None	None	None	fig|6666666.59335.peg.3911	fig|6666666.59336.peg.2148	fig|6666666.59333.peg.2477	None	None	fig|6666666.59340.peg.2259	None	None	None	None	None	None	None	None	None	None	None	None	None	None	None

5682	UspA	None	None	None	None	None	None	None	None	None	None	None	None	None	fig|6666666.71181.peg.1203	fig|6666666.59344.peg.2334	None	None	None	None	None	None	fig|6666666.59350.peg.963	None	fig|6666666.59353.peg.3382	fig|6666666.59355.peg.3481	None

6055	hypothetical protein	fig|6666666.59329.peg.3778	None	None	None	None	None	fig|6666666.59336.peg.3938	None	None	None	None	fig|6666666.59337.peg.2820	fig|6666666.59342.peg.2026	None	None	fig|6666666.59345.peg.1417	None	None	None	None	None	None	None	None	None	None

10007	Predicted ATPase with chaperone activity, associated with Flp pilus assembly	None	None	fig|6666666.59331.peg.238	None	None	fig|6666666.59335.peg.3498	None	None	None	fig|6666666.59339.peg.6814	None	fig|6666666.59337.peg.1325	None	None	None	None	None	None	None	fig|6666666.59348.peg.988	None	None	None	None	None	None

2630	Putative DNA-binding protein	None	None	None	None	None	None	None	None	None	None	None	fig|6666666.59337.peg.5804	None	fig|6666666.71181.peg.2236	fig|6666666.59344.peg.3027	None	None	None	None	None	None	None	None	fig|6666666.59353.peg.3533	fig|6666666.59355.peg.3642	None

2965	hypothetical protein	None	None	None	None	fig|6666666.59334.peg.2831	None	fig|6666666.59336.peg.2293	None	None	None	None	None	fig|6666666.59342.peg.4540	None	None	fig|6666666.59345.peg.1409	None	fig|6666666.59347.peg.2205	None	None	None	None	None	None	None	None

2968	Aspartokinase EC 2.7.2.4	None	None	fig|6666666.59331.peg.3068	fig|6666666.59332.peg.2532	None	None	None	None	None	fig|6666666.59339.peg.7742	None	None	None	None	fig|6666666.59344.peg.2716	None	None	None	None	None	None	None	None	None	None	fig|6666666.59354.peg.1298

1079	Pesticidial crystal protein cry6Aa	None	None	fig|6666666.59331.peg.808	fig|6666666.59332.peg.2442	None	fig|6666666.59335.peg.2883	None	None	None	fig|6666666.59339.peg.4669	None	None	None	None	None	None	None	None	fig|6666666.59346.peg.2095	None	None	None	None	None	None	None

5373	L-rhamnose-proton symporter	None	None	None	None	fig|6666666.59334.peg.3742	None	None	None	None	None	None	None	None	fig|6666666.71181.peg.3111	fig|6666666.59344.peg.5983	None	None	None	None	None	None	None	None	fig|6666666.59353.peg.3361	fig|6666666.59355.peg.3433	None

4336	Putative predicted metal-dependent hydrolase	None	fig|6666666.59330.peg.2498	fig|6666666.59331.peg.302	None	None	None	None	None	None	None	None	fig|6666666.59337.peg.935	None	None	None	None	None	None	None	None	fig|6666666.59349.peg.2049	None	None	None	None	fig|6666666.59354.peg.5722

5768	Mu-like prophage FluMu protein gp29	None	None	None	None	None	None	None	None	None	None	None	None	None	fig|6666666.71181.peg.2875	fig|6666666.59344.peg.4390	None	None	None	None	None	fig|6666666.59349.peg.1231	None	None	fig|6666666.59353.peg.4302	fig|6666666.59355.peg.4401	None

5762	FIG01199724 hypothetical protein	None	None	fig|6666666.59331.peg.5446	fig|6666666.59332.peg.797	None	None	None	None	None	fig|6666666.59339.peg.3527	None	None	None	None	None	None	fig|6666666.59341.peg.4428	None	fig|6666666.59346.peg.4112	None	None	None	None	None	None	None

8190	Large repetitive protein	None	fig|6666666.59330.peg.4013	None	None	None	fig|6666666.59335.peg.6115	None	fig|6666666.59333.peg.4065	None	fig|6666666.59339.peg.8417	None	fig|6666666.59337.peg.5202	None	None	None	None	None	None	None	None	None	None	None	None	None	None

6604	Orotidine 5'-phosphate decarboxylase EC 4.1.1.23	None	None	fig|6666666.59331.peg.6021	None	None	None	fig|6666666.59336.peg.425	None	fig|6666666.59338.peg.6054	None	None	None	fig|6666666.59342.peg.1802	None	None	None	None	None	None	None	None	None	None	None	None	fig|6666666.59354.peg.5717

10857	Na + -translocating NADH-quinone reductase subunit C EC 1.6.5.-	None	None	None	None	None	None	None	None	None	None	None	None	fig|6666666.59342.peg.2979	None	fig|6666666.59344.peg.5168	fig|6666666.59345.peg.2888	None	None	None	None	None	None	None	fig|6666666.59353.peg.898	None	fig|6666666.59354.peg.4981

10851	FIG024746 hypothetical protein	fig|6666666.59329.peg.2199	None	None	None	None	None	fig|6666666.59336.peg.4074	None	None	None	None	None	fig|6666666.59342.peg.2492	None	None	fig|6666666.59345.peg.1809	fig|6666666.59341.peg.2312	None	None	None	None	None	None	None	None	None

2974	RNA methyltransferase, TrmH family	fig|6666666.59329.peg.5259	None	fig|6666666.59331.peg.4403	None	None	None	None	None	None	None	fig|6666666.59340.peg.1105	None	None	None	None	None	None	None	None	fig|6666666.59348.peg.4194	None	None	None	None	None	fig|6666666.59354.peg.70

2359	putative membrane protein	None	None	fig|6666666.59331.peg.3950	fig|6666666.59332.peg.2849	None	None	None	None	None	fig|6666666.59339.peg.2773	None	None	None	None	None	None	None	None	fig|6666666.59346.peg.5331	fig|6666666.59348.peg.3479	None	None	None	None	None	None

2355	Probable signal peptide protein	None	None	None	None	None	None	None	None	None	None	None	None	None	fig|6666666.71181.peg.2964	fig|6666666.59344.peg.2885	None	None	None	None	None	None	fig|6666666.59350.peg.550	None	fig|6666666.59353.peg.1440	fig|6666666.59355.peg.1989	None

3735	Short chain dehydrogenase	None	None	None	fig|6666666.59332.peg.678	None	None	fig|6666666.59336.peg.6623	fig|6666666.59333.peg.4496	fig|6666666.59338.peg.5403	None	None	None	None	None	None	None	None	None	None	None	None	None	None	None	None	fig|6666666.59354.peg.2102

4929	hypothetical protein	fig|6666666.59329.peg.2096	fig|6666666.59330.peg.258	None	None	None	None	fig|6666666.59336.peg.1460	None	None	None	None	fig|6666666.59337.peg.967	None	None	None	fig|6666666.59345.peg.2707	None	None	None	None	None	None	None	None	None	None

2787	FIG01201965 hypothetical protein	fig|6666666.59329.peg.4348	None	None	None	None	None	None	None	None	None	None	fig|6666666.59337.peg.4708	fig|6666666.59342.peg.4242	None	None	fig|6666666.59345.peg.3123	None	fig|6666666.59347.peg.303	None	None	None	None	None	None	None	None

10602	FIG01200664 hypothetical protein	None	fig|6666666.59330.peg.2182	None	None	None	None	fig|6666666.59336.peg.3307	None	None	None	None	fig|6666666.59337.peg.4916	fig|6666666.59342.peg.93	None	None	fig|6666666.59345.peg.4379	None	None	None	None	None	None	None	None	None	None

10604	Hydrolase HAD superfamily	None	None	None	None	None	None	None	None	None	None	None	None	None	fig|6666666.71181.peg.4718	fig|6666666.59344.peg.6114	None	None	None	None	None	None	None	fig|6666666.59351.peg.2944	fig|6666666.59353.peg.3578	fig|6666666.59355.peg.4378	None

1267	Inner membrane protein YqiK	None	None	None	None	None	None	None	None	fig|6666666.59338.peg.5050	fig|6666666.59339.peg.8128	None	None	fig|6666666.59342.peg.1770	None	None	None	None	None	None	None	fig|6666666.59349.peg.356	None	None	None	None	fig|6666666.59354.peg.383

1794	hypothetical protein	fig|6666666.59329.peg.2812	None	None	None	None	fig|6666666.59335.peg.4022	None	None	fig|6666666.59338.peg.1837	fig|6666666.59339.peg.3813	None	None	None	None	None	None	None	fig|6666666.59347.peg.5305	None	None	None	None	None	None	None	None

3896	Phage terminase, small subunit	fig|6666666.59329.peg.6037	None	None	None	None	None	None	None	None	None	None	None	None	fig|6666666.71181.peg.2873	fig|6666666.59344.peg.4392	None	None	None	None	None	None	None	None	fig|6666666.59353.peg.4304	fig|6666666.59355.peg.4399	None

5959	GlcNAc 2 ABC transporter, ATP-binding component 1	None	None	None	None	None	None	None	None	fig|6666666.59338.peg.381	None	None	fig|6666666.59337.peg.4756	None	None	None	None	fig|6666666.59341.peg.3195	fig|6666666.59347.peg.3854	fig|6666666.59346.peg.3871	None	None	None	None	None	None	None

2656	Putative metal chaperone, involved in Zn homeostasis, GTPase of C0523 family	None	None	fig|6666666.59331.peg.5520	None	None	None	fig|6666666.59336.peg.5159	fig|6666666.59333.peg.5162	None	fig|6666666.59339.peg.4729	None	None	fig|6666666.59342.peg.6163	None	None	None	None	None	None	None	None	None	None	None	None	None

9061	DNA alkylation repair enzyme	None	None	None	None	fig|6666666.59334.peg.5552	None	None	None	fig|6666666.59338.peg.2208	None	fig|6666666.59340.peg.2696	None	None	None	None	None	None	fig|6666666.59347.peg.510	None	None	None	fig|6666666.59350.peg.1917	None	None	None	None

9062	bacteriophage f237 ORF10	None	None	fig|6666666.59331.peg.6292	None	None	fig|6666666.59335.peg.1767	None	None	fig|6666666.59338.peg.4522	fig|6666666.59339.peg.1442	None	None	None	None	None	None	None	fig|6666666.59347.peg.935	None	None	None	None	None	None	None	None

2901	Mn-dependent transcriptional regulator MntR	None	None	None	None	None	None	None	None	None	None	None	None	None	fig|6666666.71181.peg.1632	fig|6666666.59344.peg.5029	None	None	None	None	None	None	fig|6666666.59350.peg.6641	None	fig|6666666.59353.peg.325	fig|6666666.59355.peg.1364	None

4719	sodium-solute symporter, putative	None	None	fig|6666666.59331.peg.4262	None	fig|6666666.59334.peg.5516	None	None	None	None	None	fig|6666666.59340.peg.1826	None	fig|6666666.59342.peg.2418	None	None	None	None	None	fig|6666666.59346.peg.3766	None	None	None	None	None	None	None

4671	Putative sulfite oxidase subunit YedY	fig|6666666.59329.peg.4113	None	None	None	None	None	fig|6666666.59336.peg.6146	None	None	fig|6666666.59339.peg.1012	None	None	None	None	None	None	fig|6666666.59341.peg.6131	None	None	None	None	fig|6666666.59350.peg.6208	None	None	None	None

5149	Transcriptional regulator, AraC family	None	None	None	fig|6666666.59332.peg.4280	None	fig|6666666.59335.peg.1820	None	None	None	fig|6666666.59339.peg.1984	None	None	None	None	None	None	None	None	None	fig|6666666.59348.peg.5149	None	None	fig|6666666.59351.peg.1129	None	None	None

121	3-hydroxyisobutyrate dehydrogenase EC 1.1.1.31	None	None	None	None	None	fig|6666666.59335.peg.4295	None	None	None	fig|6666666.59339.peg.4636	None	None	fig|6666666.59342.peg.4306	None	None	None	None	None	None	fig|6666666.59348.peg.4579	None	fig|6666666.59350.peg.2370	None	None	None	None

7957	FIG01203515 hypothetical protein	fig|6666666.59329.peg.1328	None	None	None	None	None	None	fig|6666666.59333.peg.5419	None	None	None	fig|6666666.59337.peg.5731	fig|6666666.59342.peg.2078	None	None	fig|6666666.59345.peg.1472	None	None	None	None	None	None	None	None	None	None

10546	Arabinose efflux permease	None	None	None	None	fig|6666666.59334.peg.4757	None	fig|6666666.59336.peg.3135	None	None	None	None	None	fig|6666666.59342.peg.6356	None	None	fig|6666666.59345.peg.387	fig|6666666.59341.peg.5543	None	None	None	None	None	None	None	None	None

8819	contains zinc finger, C3HC4 type RING finger , signature, PROSITE PS00518; go function protein binding [goid 0005515]; go function zinc ion binding [goid 0008270] zinc finger C3HC4-type RING finger family protein	None	None	None	None	None	None	None	None	None	None	None	fig|6666666.59337.peg.5476	fig|6666666.59342.peg.3572	None	None	fig|6666666.59345.peg.1360	None	None	None	None	fig|6666666.59349.peg.3814	None	None	None	None	fig|6666666.59354.peg.339

8210	FIG01023251 hypothetical protein	None	None	fig|6666666.59331.peg.5636	None	None	None	None	None	fig|6666666.59338.peg.165	None	None	None	None	fig|6666666.71181.peg.6150	None	fig|6666666.59345.peg.5193	None	None	None	None	None	fig|6666666.59350.peg.4326	None	None	None	None

8215	Alkanesulfonates ABC transporter ATP-binding protein Sulfonate ABC transporter, ATP-binding subunit SsuB	None	None	None	None	None	None	None	None	None	None	None	None	None	fig|6666666.71181.peg.819	fig|6666666.59344.peg.58	None	None	None	None	None	None	fig|6666666.59350.peg.4114	None	fig|6666666.59353.peg.395	fig|6666666.59355.peg.2118	None

268	Uncharacterized iron-regulated protein	fig|6666666.59329.peg.878	None	None	None	fig|6666666.59334.peg.2897	fig|6666666.59335.peg.2955	None	None	None	fig|6666666.59339.peg.261	None	None	None	None	None	None	None	None	None	fig|6666666.59348.peg.1976	None	None	None	None	None	None

989	FIG01202431 hypothetical protein	None	None	None	None	None	None	fig|6666666.59336.peg.5315	None	None	None	None	fig|6666666.59337.peg.2588	fig|6666666.59342.peg.5289	None	None	fig|6666666.59345.peg.777	None	None	None	None	None	None	fig|6666666.59351.peg.947	None	None	None

982	Histidine permease YuiF	None	None	None	fig|6666666.59332.peg.3347	fig|6666666.59334.peg.1428	fig|6666666.59335.peg.1200	None	None	None	None	fig|6666666.59340.peg.1685	None	None	None	None	None	None	None	None	None	None	None	fig|6666666.59351.peg.1012	None	None	None

2034	Urea ABC transporter, permease protein UrtB	None	None	fig|6666666.59331.peg.1641	None	None	None	None	None	None	fig|6666666.59339.peg.5370	None	None	None	None	None	None	fig|6666666.59341.peg.240	None	None	fig|6666666.59348.peg.550	None	fig|6666666.59350.peg.6567	None	None	None	None

474	Adenylate cyclase EC 4.6.1.1	None	None	None	None	None	None	None	None	fig|6666666.59338.peg.1452	None	fig|6666666.59340.peg.1553	None	None	None	None	None	fig|6666666.59341.peg.4501	fig|6666666.59347.peg.3607	None	None	None	fig|6666666.59350.peg.3504	None	None	None	None

10001	probable DNA alkylation repair enzyme	None	None	None	None	None	None	fig|6666666.59336.peg.5382	None	fig|6666666.59338.peg.5378	None	None	fig|6666666.59337.peg.4712	fig|6666666.59342.peg.4277	None	None	fig|6666666.59345.peg.3126	None	None	None	None	None	None	None	None	None	None

1289	Inosine-uridine preferring nucleoside hydrolase EC 3.2.2.1	fig|6666666.59329.peg.873	None	None	None	None	None	None	None	None	None	None	None	fig|6666666.59342.peg.5036	None	fig|6666666.59344.peg.1757	None	None	None	None	None	None	fig|6666666.59350.peg.4277	None	fig|6666666.59353.peg.2302	None	None

689	hypothetical protein	None	None	None	None	None	None	None	None	None	None	None	None	None	None	None	None	None	fig|6666666.59347.peg.1578	None	None	fig|6666666.59349.peg.3990	fig|6666666.59350.peg.5380	fig|6666666.59351.peg.4515	None	None	fig|6666666.59354.peg.3791

688	FIG01056215 hypothetical protein	None	None	None	None	None	None	None	None	None	None	None	None	None	fig|6666666.71181.peg.3084	fig|6666666.59344.peg.4221	None	None	None	None	None	None	fig|6666666.59350.peg.7251	None	fig|6666666.59353.peg.4261	fig|6666666.59355.peg.4335	None

2150	Galactosamine-6-phosphate isomerase EC 5.3.1.-	fig|6666666.59329.peg.616	None	None	None	None	None	None	None	None	None	None	None	None	fig|6666666.71181.peg.2664	fig|6666666.59344.peg.4310	None	None	None	None	None	None	None	None	fig|6666666.59353.peg.3879	fig|6666666.59355.peg.3816	None

10422	Branched-chain amino acid transport ATP-binding protein LivF TC 3.A.1.4.1	None	None	None	None	None	None	None	None	None	None	None	None	None	fig|6666666.71181.peg.4420	fig|6666666.59344.peg.3436	None	None	None	None	None	None	fig|6666666.59350.peg.4135	None	fig|6666666.59353.peg.3780	fig|6666666.59355.peg.3375	None

387	positive regulator of the middle operon; also called E17	fig|6666666.59329.peg.5603	None	None	None	None	None	None	None	None	None	None	None	None	fig|6666666.71181.peg.2864	fig|6666666.59344.peg.4399	None	None	None	None	None	None	None	None	fig|6666666.59353.peg.4311	fig|6666666.59355.peg.4391	None

2191	putative membrane protein	fig|6666666.59329.peg.4827	fig|6666666.59330.peg.3845	None	None	None	None	None	None	None	None	None	fig|6666666.59337.peg.4806	fig|6666666.59342.peg.1604	None	None	fig|6666666.59345.peg.315	None	None	None	None	None	None	None	None	None	None

10425	Beta-fimbriae chaperone protein	None	None	None	None	None	None	None	fig|6666666.59333.peg.2127	None	None	None	None	None	fig|6666666.71181.peg.83	fig|6666666.59344.peg.3182	None	None	None	None	None	None	None	None	fig|6666666.59353.peg.1932	fig|6666666.59355.peg.1475	None

166	Na + H + antiporter subunit A; Na + H + antiporter subunit B	None	None	None	None	None	None	None	fig|6666666.59333.peg.4701	fig|6666666.59338.peg.3060	None	None	None	fig|6666666.59342.peg.6011	None	None	None	None	None	None	None	None	None	fig|6666666.59351.peg.3141	None	None	fig|6666666.59354.peg.2464

6190	lipoprotein, putative	None	fig|6666666.59330.peg.3071	None	None	None	None	None	None	None	None	None	None	fig|6666666.59342.peg.1743	None	None	None	None	None	None	None	fig|6666666.59349.peg.2278	None	fig|6666666.59351.peg.4159	None	None	fig|6666666.59354.peg.4106

9185	putative membrane protein	fig|6666666.59329.peg.5318	None	None	None	fig|6666666.59334.peg.558	None	None	None	fig|6666666.59338.peg.179	None	None	None	fig|6666666.59342.peg.2278	None	None	fig|6666666.59345.peg.3163	None	None	None	None	None	None	None	None	None	None

805	Selenophosphate-dependent tRNA 2-selenouridine synthase	None	None	None	None	None	None	None	None	None	None	None	None	None	fig|6666666.71181.peg.4904	fig|6666666.59344.peg.2901	None	None	None	None	None	None	fig|6666666.59350.peg.79	None	fig|6666666.59353.peg.1426	fig|6666666.59355.peg.1975	None

10242	Oxidoreductase flavoprotein	None	None	None	None	None	None	None	None	None	None	None	None	None	fig|6666666.71181.peg.3773	fig|6666666.59344.peg.2230	None	None	None	None	None	None	fig|6666666.59350.peg.945	None	fig|6666666.59353.peg.183	fig|6666666.59355.peg.2951	None

9636	Potassium efflux system KefA protein Small-conductance mechanosensitive channel	None	None	fig|6666666.59331.peg.58	None	None	None	None	None	None	None	None	None	None	None	None	None	None	None	fig|6666666.59346.peg.4643	None	fig|6666666.59349.peg.1321	None	fig|6666666.59351.peg.4541	None	None	fig|6666666.59354.peg.150

6768	Peptide transport system permease protein SapC	None	None	None	None	fig|6666666.59334.peg.4073	None	None	None	None	None	None	None	None	None	None	None	None	None	None	None	fig|6666666.59349.peg.673	fig|6666666.59350.peg.5500	fig|6666666.59351.peg.2785	None	None	fig|6666666.59354.peg.1094

2509	Type III secretion host injection and negative regulator protein YopD	fig|6666666.59329.peg.535	fig|6666666.59330.peg.3278	None	None	None	None	None	fig|6666666.59333.peg.3035	None	None	None	fig|6666666.59337.peg.2811	None	None	None	fig|6666666.59345.peg.1451	None	None	None	None	None	None	None	None	None	None

9513	FIG01205087 hypothetical protein	None	None	None	fig|6666666.59332.peg.2017	None	None	fig|6666666.59336.peg.4173	None	None	None	None	fig|6666666.59337.peg.4692	fig|6666666.59342.peg.2151	None	None	fig|6666666.59345.peg.4694	None	None	None	None	None	None	None	None	None	None

1256	Tetrathionate reductase subunit C	None	None	None	None	fig|6666666.59334.peg.788	None	None	fig|6666666.59333.peg.3876	None	None	None	None	fig|6666666.59342.peg.4205	None	None	fig|6666666.59345.peg.1006	fig|6666666.59341.peg.2626	None	None	None	None	None	None	None	None	None

1250	Transcriptional regulator	None	None	None	None	None	None	None	None	None	None	None	None	None	fig|6666666.71181.peg.3070	fig|6666666.59344.peg.4204	None	None	None	None	None	None	fig|6666666.59350.peg.994	None	fig|6666666.59353.peg.4247	fig|6666666.59355.peg.4348	None

5813	Benzoate 1,2-dioxygenase alpha subunit EC 1.14.12.10	None	None	None	None	None	None	None	None	None	None	None	None	None	fig|6666666.71181.peg.4658	fig|6666666.59344.peg.3806	None	None	None	None	None	None	None	fig|6666666.59351.peg.1949	fig|6666666.59353.peg.3953	fig|6666666.59355.peg.4209	None

5815	Methyl-accepting chemotaxis protein	None	None	None	None	None	None	fig|6666666.59336.peg.4017	None	fig|6666666.59338.peg.2448	fig|6666666.59339.peg.2677	None	None	fig|6666666.59342.peg.835	None	None	None	None	None	None	fig|6666666.59348.peg.3513	None	None	None	None	None	None

146	hypothetical protein	None	None	None	None	None	None	fig|6666666.59336.peg.5590	fig|6666666.59333.peg.1979	None	None	None	fig|6666666.59337.peg.4835	fig|6666666.59342.peg.5479	None	None	fig|6666666.59345.peg.5701	None	None	None	None	None	None	None	None	None	None

9161	D-ribulokinase EC 2.7.1.47	None	None	None	fig|6666666.59332.peg.1995	None	None	None	None	None	None	None	None	None	fig|6666666.71181.peg.1059	fig|6666666.59344.peg.3868	None	None	None	None	None	None	None	None	fig|6666666.59353.peg.4516	fig|6666666.59355.peg.4626	None

6248	Probable lipoprotein	None	fig|6666666.59330.peg.3070	None	None	None	None	None	None	None	None	None	None	fig|6666666.59342.peg.1742	None	None	None	None	None	None	None	fig|6666666.59349.peg.2277	None	fig|6666666.59351.peg.4158	None	None	fig|6666666.59354.peg.4107

10524	Periplasmic beta-glucosidase EC 3.2.1.21	None	None	fig|6666666.59331.peg.3878	None	None	None	None	None	None	None	fig|6666666.59340.peg.3340	None	fig|6666666.59342.peg.509	None	fig|6666666.59344.peg.2283	None	None	None	fig|6666666.59346.peg.3795	None	None	None	None	None	None	None

8875	FIG01201389 hypothetical protein	fig|6666666.59329.peg.4594	None	fig|6666666.59331.peg.1819	None	None	None	fig|6666666.59336.peg.4003	None	None	None	None	fig|6666666.59337.peg.4172	None	None	None	fig|6666666.59345.peg.4760	None	None	None	None	None	None	None	None	None	None

5255	D-Tyr-tRNAtyr deacylase	fig|6666666.59329.peg.461	None	None	None	None	None	fig|6666666.59336.peg.1953	fig|6666666.59333.peg.4447	None	None	None	None	fig|6666666.59342.peg.3464	None	None	fig|6666666.59345.peg.1657	None	None	None	None	None	None	None	None	None	None

5942	Metallo-beta-lactamase family protein	fig|6666666.59329.peg.3804	None	fig|6666666.59331.peg.231	None	None	None	None	None	None	None	None	None	None	None	None	None	None	None	fig|6666666.59346.peg.389	None	None	fig|6666666.59350.peg.1144	fig|6666666.59351.peg.2434	None	None	None

8728	Transcriptional regulator LuxT	fig|6666666.59329.peg.5414	fig|6666666.59330.peg.3830	None	None	None	None	None	None	None	None	None	fig|6666666.59337.peg.2012	fig|6666666.59342.peg.5336	None	None	fig|6666666.59345.peg.3079	None	None	None	None	None	None	None	None	None	None

9574	FIG01200683 hypothetical protein	fig|6666666.59329.peg.5953	fig|6666666.59330.peg.771	None	None	None	None	None	None	None	None	None	fig|6666666.59337.peg.5362	fig|6666666.59342.peg.2296	None	None	None	None	None	None	fig|6666666.59348.peg.1866	None	None	None	None	None	None

787	Cobalamin biosynthesis protein BluB 5,6-dimethylbenzimidazole synthase, flavin destructase family	None	None	fig|6666666.59331.peg.6224	None	fig|6666666.59334.peg.2396	fig|6666666.59335.peg.6144	None	None	None	fig|6666666.59339.peg.826	None	None	None	None	None	None	None	None	None	None	None	fig|6666666.59350.peg.2767	None	None	None	None

1109	hypothetical protein	None	None	fig|6666666.59331.peg.3982	fig|6666666.59332.peg.2772	None	None	None	None	None	None	None	None	None	None	None	None	fig|6666666.59341.peg.3682	fig|6666666.59347.peg.3307	fig|6666666.59346.peg.1906	None	None	None	None	None	None	None

4796	Pyruvate decarboxylase EC 4.1.1.1 ; Alpha-keto-acid decarboxylase EC 4.1.1.-	None	None	None	None	None	None	None	None	None	None	None	None	None	fig|6666666.71181.peg.1550	fig|6666666.59344.peg.2043	None	None	None	None	None	None	None	fig|6666666.59351.peg.457	fig|6666666.59353.peg.2888	fig|6666666.59355.peg.2851	None

5831	FIG00922652 hypothetical protein	None	None	None	None	None	fig|6666666.59335.peg.3270	fig|6666666.59336.peg.3064	None	None	fig|6666666.59339.peg.6840	None	None	None	None	None	fig|6666666.59345.peg.2138	fig|6666666.59341.peg.1788	None	None	None	None	None	None	None	None	None

6268	Putative hydroxymethyltransferase	None	None	None	None	None	fig|6666666.59335.peg.3653	fig|6666666.59336.peg.660	None	None	None	None	fig|6666666.59337.peg.2267	fig|6666666.59342.peg.3290	None	None	fig|6666666.59345.peg.2039	None	None	None	None	None	None	None	None	None	None

10078	Alpha-1,2-mannosidase	fig|6666666.59329.peg.2374	None	None	None	None	None	fig|6666666.59336.peg.1060	None	None	None	None	fig|6666666.59337.peg.3249	fig|6666666.59342.peg.3498	None	None	fig|6666666.59345.peg.1779	None	None	None	None	None	None	None	None	None	None

3070	Cation transport protein chaC	None	None	None	None	None	None	None	None	None	None	None	None	None	fig|6666666.71181.peg.3753	fig|6666666.59344.peg.5577	None	None	None	None	None	None	fig|6666666.59350.peg.5361	None	fig|6666666.59353.peg.4664	fig|6666666.59355.peg.3859	None

5642	Tricarboxylate transport protein TctB	None	None	None	None	None	None	None	None	None	None	None	None	None	fig|6666666.71181.peg.1489	fig|6666666.59344.peg.1869	None	None	None	None	None	None	fig|6666666.59350.peg.6742	None	fig|6666666.59353.peg.1221	fig|6666666.59355.peg.255	None

483	Probable secreted protein	None	None	None	None	None	fig|6666666.59335.peg.5984	None	None	None	fig|6666666.59339.peg.2152	None	None	None	None	None	None	None	None	None	None	fig|6666666.59349.peg.2320	None	fig|6666666.59351.peg.2931	None	None	fig|6666666.59354.peg.287

7743	FIG01200568 hypothetical protein	fig|6666666.59329.peg.1263	None	None	None	None	None	fig|6666666.59336.peg.2952	None	None	None	None	fig|6666666.59337.peg.1941	fig|6666666.59342.peg.3042	None	None	fig|6666666.59345.peg.4035	None	None	None	None	None	None	None	None	None	None

6726	Long-chain-fatty-acid--CoA ligase EC 6.2.1.3	None	None	None	None	None	None	None	None	None	fig|6666666.59339.peg.1061	None	None	None	None	None	None	None	None	None	fig|6666666.59348.peg.2961	fig|6666666.59349.peg.895	fig|6666666.59350.peg.7974	None	None	None	fig|6666666.59354.peg.6523

6094	hypothetical protein	None	None	fig|6666666.59331.peg.5665	fig|6666666.59332.peg.5119	None	None	fig|6666666.59336.peg.3690	None	None	None	None	None	fig|6666666.59342.peg.698	None	None	fig|6666666.59345.peg.4480	None	None	None	None	None	None	None	None	None	None

2546	Tryptophan 2,3-dioxygenase EC 1.13.11.11	None	None	None	None	None	None	None	None	None	None	None	None	None	fig|6666666.71181.peg.2286	fig|6666666.59344.peg.4691	None	None	None	None	None	None	fig|6666666.59350.peg.2681	None	fig|6666666.59353.peg.4495	fig|6666666.59355.peg.4603	None

11443	Phenylacetic acid degradation protein PaaD, thioesterase	None	None	None	None	None	None	None	None	None	None	None	None	None	fig|6666666.71181.peg.4678	fig|6666666.59344.peg.3328	None	None	None	None	None	None	fig|6666666.59350.peg.3523	None	fig|6666666.59353.peg.3906	fig|6666666.59355.peg.3671	None

4422	Type III secretion outermembrane contact sensing protein YopN,Yop4b,LcrE	fig|6666666.59329.peg.523	fig|6666666.59330.peg.3291	None	None	None	None	None	fig|6666666.59333.peg.3365	None	None	None	fig|6666666.59337.peg.5240	None	None	None	fig|6666666.59345.peg.1462	None	None	None	None	None	None	None	None	None	None

8934	Phosphonate ABC transporter permease protein phnE1 TC 3.A.1.9.1	None	None	None	fig|6666666.59332.peg.2541	None	None	None	None	None	fig|6666666.59339.peg.7010	fig|6666666.59340.peg.2751	None	None	None	None	None	fig|6666666.59341.peg.4662	None	None	None	None	fig|6666666.59350.peg.4230	None	None	None	None

7824	membrane protein	None	None	None	fig|6666666.59332.peg.3197	None	None	None	fig|6666666.59333.peg.2818	fig|6666666.59338.peg.3846	fig|6666666.59339.peg.8176	None	None	None	None	None	None	None	None	fig|6666666.59346.peg.1674	None	None	None	None	None	None	None

1473	FIG01205627 hypothetical protein	None	None	None	None	None	fig|6666666.59335.peg.1584	fig|6666666.59336.peg.3145	None	None	None	None	fig|6666666.59337.peg.2280	fig|6666666.59342.peg.821	None	None	fig|6666666.59345.peg.2019	None	None	None	None	None	None	None	None	None	None

1474	Ferrichrome transport ATP-binding protein FhuC TC 3.A.1.14.3	None	None	None	fig|6666666.59332.peg.3728	None	None	None	None	None	None	None	None	None	fig|6666666.71181.peg.3193	fig|6666666.59344.peg.1649	None	None	None	None	None	None	None	None	fig|6666666.59353.peg.1074	fig|6666666.59355.peg.404	None

4223	GTP-binding protein Obg	None	None	None	None	None	fig|6666666.59335.peg.4489	None	fig|6666666.59333.peg.550	None	fig|6666666.59339.peg.7881	None	None	None	None	fig|6666666.59344.peg.2115	None	None	None	None	fig|6666666.59348.peg.168	None	None	None	None	None	None

4923	FIG01201235 hypothetical protein	None	None	None	fig|6666666.59332.peg.6062	None	None	None	None	fig|6666666.59338.peg.4495	None	None	None	None	None	None	None	fig|6666666.59341.peg.4687	fig|6666666.59347.peg.777	fig|6666666.59346.peg.2310	None	None	None	None	None	None	None

2283	L,D-transpeptidase YbiS	None	None	None	None	None	None	None	None	None	None	None	None	None	fig|6666666.71181.peg.4448	fig|6666666.59344.peg.5908	None	None	None	None	None	None	fig|6666666.59350.peg.5300	None	fig|6666666.59353.peg.2487	fig|6666666.59355.peg.582	None

8316	L-proline glycine betaine binding ABC transporter protein ProX TC 3.A.1.12.1 Osmotic adaptation	fig|6666666.59329.peg.745	None	None	None	fig|6666666.59334.peg.1720	None	None	None	None	None	None	None	fig|6666666.59342.peg.2836	None	None	fig|6666666.59345.peg.354	fig|6666666.59341.peg.1785	None	None	None	None	None	None	None	None	None

10900	Putative sugar ABC transport system, permease protein YjfF	None	None	None	fig|6666666.59332.peg.2875	None	None	None	None	None	None	None	None	None	fig|6666666.71181.peg.4864	fig|6666666.59344.peg.4238	None	None	None	None	None	None	None	None	fig|6666666.59353.peg.4275	fig|6666666.59355.peg.4321	None

6286	Hemolysin activation secretion protein	None	None	None	None	None	fig|6666666.59335.peg.4856	None	None	None	None	None	None	None	fig|6666666.71181.peg.1034	fig|6666666.59344.peg.1816	None	None	None	None	None	None	None	None	fig|6666666.59353.peg.2352	fig|6666666.59355.peg.1625	None

868	Phosphoribosylformylglycinamidine synthase, synthetase subunit EC 6.3.5.3 Phosphoribosylformylglycinamidine synthase, glutamine amidotransferase subunit EC 6.3.5.3	None	fig|6666666.59330.peg.1078	None	None	None	fig|6666666.59335.peg.1700	None	fig|6666666.59333.peg.3842	None	fig|6666666.59339.peg.7253	None	fig|6666666.59337.peg.5441	None	None	None	None	None	None	None	None	None	None	None	None	None	None

861	Zona occludens toxin	fig|6666666.59329.peg.4890	fig|6666666.59330.peg.4978	fig|6666666.59331.peg.360	None	None	None	None	None	fig|6666666.59338.peg.1619	None	None	None	fig|6666666.59342.peg.3439	None	None	None	None	None	None	None	None	None	None	None	None	None

9835	Oligopeptide ABC transporter, periplasmic oligopeptide-binding protein OppA TC 3.A.1.5.1	None	None	None	None	None	fig|6666666.59335.peg.5275	None	None	fig|6666666.59338.peg.1102	fig|6666666.59339.peg.4820	None	None	None	None	None	None	fig|6666666.59341.peg.3191	None	fig|6666666.59346.peg.5344	None	None	None	None	None	None	None

10156	FIG01200528 hypothetical protein	fig|6666666.59329.peg.2227	fig|6666666.59330.peg.3018	None	None	None	None	fig|6666666.59336.peg.6198	None	None	None	None	None	None	None	None	fig|6666666.59345.peg.5271	fig|6666666.59341.peg.5664	None	None	None	None	None	None	None	None	None

3942	Transcriptional factor MdcH	fig|6666666.59329.peg.4561	None	fig|6666666.59331.peg.1221	None	fig|6666666.59334.peg.3551	None	None	None	None	None	None	None	fig|6666666.59342.peg.2001	fig|6666666.71181.peg.3716	None	None	None	None	None	None	None	None	None	None	None	None

10014	FIG01202545 hypothetical protein	None	None	None	None	None	None	None	fig|6666666.59333.peg.3453	None	None	None	None	None	None	None	None	None	None	None	fig|6666666.59348.peg.1421	fig|6666666.59349.peg.2564	None	fig|6666666.59351.peg.3596	None	None	fig|6666666.59354.peg.1881

3614	Rod shape-determining protein MreB	fig|6666666.59329.peg.705	None	None	None	fig|6666666.59334.peg.1314	None	None	None	fig|6666666.59338.peg.3605	None	None	None	None	None	None	None	None	None	None	None	None	fig|6666666.59350.peg.3074	None	None	None	fig|6666666.59354.peg.3165

2567	NADH-ubiquinone oxidoreductase chain C EC 1.6.5.3 NADH-ubiquinone oxidoreductase chain D EC 1.6.5.3	None	None	None	None	None	None	None	None	None	None	None	None	None	fig|6666666.71181.peg.542	fig|6666666.59344.peg.1926	None	None	None	None	None	None	fig|6666666.59350.peg.2568	None	fig|6666666.59353.peg.1171	fig|6666666.59355.peg.304	None

11427	hypothetical protein	fig|6666666.59329.peg.2346	fig|6666666.59330.peg.675	None	None	None	None	fig|6666666.59336.peg.3226	None	None	None	None	fig|6666666.59337.peg.5257	None	None	None	fig|6666666.59345.peg.4748	None	None	None	None	None	None	None	None	None	None

1535	Chromate transport protein ChrA	None	fig|6666666.59330.peg.2806	None	None	None	None	fig|6666666.59336.peg.1095	None	None	fig|6666666.59339.peg.6826	None	fig|6666666.59337.peg.1342	fig|6666666.59342.peg.6080	None	None	None	None	None	None	None	None	None	None	None	None	None

1247	GTPase	fig|6666666.59329.peg.1493	fig|6666666.59330.peg.4507	None	None	None	None	fig|6666666.59336.peg.748	None	None	None	None	fig|6666666.59337.peg.1267	None	None	None	fig|6666666.59345.peg.4454	None	None	None	None	None	None	None	None	None	None

10460	Pyruvate dehydrogenase E1 component EC 1.2.4.1	fig|6666666.59329.peg.2589	None	None	None	None	None	None	None	fig|6666666.59338.peg.919	fig|6666666.59339.peg.7966	None	None	None	None	None	None	None	None	None	None	fig|6666666.59349.peg.3763	None	None	None	None	fig|6666666.59354.peg.771

5198	FIG01199556 hypothetical protein	fig|6666666.59329.peg.4843	None	None	None	None	None	fig|6666666.59336.peg.3378	None	None	None	None	None	None	None	fig|6666666.59344.peg.5402	fig|6666666.59345.peg.2180	None	None	None	fig|6666666.59348.peg.638	None	None	None	None	None	None

1963	protein of unknown function DUF497	None	None	None	None	None	None	None	None	None	None	None	None	None	fig|6666666.71181.peg.3720	fig|6666666.59344.peg.3698	None	None	None	None	None	None	fig|6666666.59350.peg.3965	None	fig|6666666.59353.peg.3820	fig|6666666.59355.peg.1566	None

1411	hypothetical protein	fig|6666666.59329.peg.2001	None	fig|6666666.59331.peg.134	None	None	None	None	None	None	fig|6666666.59339.peg.6022	None	None	fig|6666666.59342.peg.4943	None	None	None	None	None	fig|6666666.59346.peg.2872	None	None	None	None	None	None	None

7415	putative DNA helicase	None	None	None	fig|6666666.59332.peg.1693	None	None	None	None	None	None	None	None	None	fig|6666666.71181.peg.1668	fig|6666666.59344.peg.626	None	None	None	None	None	None	None	None	fig|6666666.59353.peg.1037	fig|6666666.59355.peg.3581	None

7288	Branched-chain amino acid ABC transporter, amino acid-binding protein TC 3.A.1.4.1	None	None	None	None	None	fig|6666666.59335.peg.2186	None	None	None	fig|6666666.59339.peg.3930	None	None	None	None	None	None	None	None	None	None	fig|6666666.59349.peg.2972	None	fig|6666666.59351.peg.4129	None	None	fig|6666666.59354.peg.1444

4586	FIG01202917 hypothetical protein	fig|6666666.59329.peg.5180	fig|6666666.59330.peg.4510	None	None	None	None	None	fig|6666666.59333.peg.918	None	None	None	fig|6666666.59337.peg.2733	None	None	None	None	fig|6666666.59341.peg.5057	None	None	None	None	None	None	None	None	None

10588	Maebl	fig|6666666.59329.peg.4695	fig|6666666.59330.peg.4488	fig|6666666.59331.peg.1220	None	None	fig|6666666.59335.peg.5070	None	None	None	None	None	None	None	None	None	None	None	fig|6666666.59347.peg.2546	None	None	None	None	None	None	None	None

8623	hypothetical protein	fig|6666666.59329.peg.4975	None	None	None	None	None	fig|6666666.59336.peg.1512	None	None	None	None	fig|6666666.59337.peg.5065	fig|6666666.59342.peg.1952	None	None	fig|6666666.59345.peg.4365	None	None	None	None	None	None	None	None	None	None

10729	putative site specific recombinase	None	None	None	fig|6666666.59332.peg.1303	fig|6666666.59334.peg.981	None	None	None	None	None	None	fig|6666666.59337.peg.4667	None	fig|6666666.71181.peg.4072	None	None	None	None	None	None	fig|6666666.59349.peg.4915	None	None	None	None	None

4076	FIG01200621 hypothetical protein	fig|6666666.59329.peg.6535	None	None	None	None	fig|6666666.59335.peg.4534	fig|6666666.59336.peg.5552	fig|6666666.59333.peg.1908	None	None	None	None	None	None	None	None	None	None	None	fig|6666666.59348.peg.3613	None	None	None	None	None	None

3965	putative membrane protein	None	None	fig|6666666.59331.peg.3432	None	None	fig|6666666.59335.peg.5700	None	None	None	fig|6666666.59339.peg.4758	None	None	None	None	None	None	None	None	fig|6666666.59346.peg.5187	fig|6666666.59348.peg.4068	None	None	None	None	None	None

7510	UDP-glucose 4-epimerase EC 5.1.3.2	None	None	None	None	None	fig|6666666.59335.peg.3520	None	None	None	None	None	None	None	fig|6666666.71181.peg.2474	fig|6666666.59344.peg.5404	None	None	None	None	None	None	None	None	fig|6666666.59353.peg.3605	fig|6666666.59355.peg.4352	None

6328	Phage integrase	fig|6666666.59329.peg.3596	fig|6666666.59330.peg.4712	fig|6666666.59331.peg.1719	None	None	None	None	None	fig|6666666.59338.peg.5004	None	None	None	fig|6666666.59342.peg.2618	None	None	None	None	None	None	None	None	None	None	None	None	None

586	FIG01201196 hypothetical protein	fig|6666666.59329.peg.1570	None	None	None	fig|6666666.59334.peg.3497	None	fig|6666666.59336.peg.688	None	None	None	None	fig|6666666.59337.peg.1314	None	None	None	fig|6666666.59345.peg.2325	None	None	None	None	None	None	None	None	None	None

584	Beta-glucosidase EC 3.2.1.21	None	None	None	None	None	None	None	None	None	None	None	None	None	fig|6666666.71181.peg.2154	fig|6666666.59344.peg.3226	None	fig|6666666.59341.peg.4187	None	None	None	None	None	None	fig|6666666.59353.peg.2842	fig|6666666.59355.peg.3945	None

2831	Protocatechuate 4,5-dioxygenase alpha chain EC 1.13.11.8	None	None	fig|6666666.59331.peg.346	fig|6666666.59332.peg.519	None	None	None	None	None	None	None	None	None	None	fig|6666666.59344.peg.863	None	None	None	fig|6666666.59346.peg.183	None	None	None	None	None	fig|6666666.59355.peg.3330	None

5232	Sensor protein torS EC 2.7.3.-	fig|6666666.59329.peg.2009	None	None	fig|6666666.59332.peg.4979	None	None	None	fig|6666666.59333.peg.3859	None	None	None	None	fig|6666666.59342.peg.2238	None	None	None	None	None	fig|6666666.59346.peg.3652	None	None	None	None	None	None	None

8598	Putative inner membrane protein Fragment	None	None	None	None	None	None	None	None	None	None	None	None	None	fig|6666666.71181.peg.3119	fig|6666666.59344.peg.5963	None	None	None	None	None	fig|6666666.59349.peg.157	None	None	fig|6666666.59353.peg.3353	fig|6666666.59355.peg.3442	None

5893	putative phosphoribosyl-AMP cyclohydrolase	None	None	None	None	fig|6666666.59334.peg.1175	None	None	None	fig|6666666.59338.peg.5246	None	fig|6666666.59340.peg.3660	None	None	None	None	None	None	fig|6666666.59347.peg.2197	None	None	None	fig|6666666.59350.peg.7471	None	None	None	None

5895	Carbamoyl-phosphate synthase large chain EC 6.3.5.5	None	None	None	None	None	None	fig|6666666.59336.peg.2529	None	fig|6666666.59338.peg.1279	fig|6666666.59339.peg.4991	fig|6666666.59340.peg.1643	None	None	None	None	None	None	None	fig|6666666.59346.peg.1849	None	None	None	None	None	None	None

5896	Putative inner membrane protein	None	None	None	fig|6666666.59332.peg.841	None	None	None	None	None	None	None	None	None	fig|6666666.71181.peg.3914	fig|6666666.59344.peg.380	None	None	None	None	None	None	None	None	fig|6666666.59353.peg.1602	fig|6666666.59355.peg.1119	None

3403	Di- tripeptide transporter	None	None	None	None	fig|6666666.59334.peg.2653	None	None	None	None	None	None	None	None	fig|6666666.71181.peg.871	fig|6666666.59344.peg.2	None	None	None	None	None	None	None	None	fig|6666666.59353.peg.343	fig|6666666.59355.peg.1381	None

3407	Beta-ketoadipate enol-lactone hydrolase EC 3.1.1.24	None	None	None	None	None	None	fig|6666666.59336.peg.513	None	fig|6666666.59338.peg.5430	fig|6666666.59339.peg.780	None	None	None	None	None	None	None	fig|6666666.59347.peg.3420	None	None	None	fig|6666666.59350.peg.1889	None	None	None	None

7537	FIG01203854 hypothetical protein	None	fig|6666666.59330.peg.3661	None	None	None	None	fig|6666666.59336.peg.4531	None	None	None	None	fig|6666666.59337.peg.771	fig|6666666.59342.peg.5115	None	None	fig|6666666.59345.peg.3622	None	None	None	None	None	None	None	None	None	None

4872	hypothetical protein	None	None	fig|6666666.59331.peg.3261	fig|6666666.59332.peg.2920	None	None	None	None	fig|6666666.59338.peg.1212	None	None	None	None	None	None	None	None	fig|6666666.59347.peg.286	None	fig|6666666.59348.peg.3483	None	None	None	None	None	None

2799	Para-aminobenzoate synthase, amidotransferase component EC 2.6.1.85 # PabAb	None	fig|6666666.59330.peg.1445	None	None	None	None	None	fig|6666666.59333.peg.306	fig|6666666.59338.peg.3805	None	fig|6666666.59340.peg.2412	fig|6666666.59337.peg.1798	None	None	None	None	None	None	None	None	None	None	None	None	None	None

5696	Deoxyuridine 5'-triphosphate nucleotidohydrolase EC 3.6.1.23	None	None	None	None	None	None	None	None	None	None	None	None	None	fig|6666666.71181.peg.2615	fig|6666666.59344.peg.937	None	None	None	None	None	None	fig|6666666.59350.peg.7555	None	fig|6666666.59353.peg.2596	fig|6666666.59355.peg.2606	None

6486	Multiple sugar ABC transporter, ATP-binding protein	None	None	None	None	None	None	None	None	None	None	None	None	None	fig|6666666.71181.peg.3597	fig|6666666.59344.peg.5552	None	None	fig|6666666.59347.peg.3502	None	None	None	None	None	fig|6666666.59353.peg.4143	fig|6666666.59355.peg.4272	None

8368	Periplasmic beta-glucosidase EC 3.2.1.21	None	None	None	fig|6666666.59332.peg.5408	None	None	None	None	None	None	None	fig|6666666.59337.peg.1170	fig|6666666.59342.peg.506	None	None	None	None	None	fig|6666666.59346.peg.3791	None	None	None	None	None	None	fig|6666666.59354.peg.725

7875	putative DNA-binding protein	None	None	None	None	None	None	None	None	None	None	None	None	None	fig|6666666.71181.peg.5500	fig|6666666.59344.peg.5893	None	None	None	None	None	None	fig|6666666.59350.peg.1695	None	fig|6666666.59353.peg.5103	fig|6666666.59355.peg.5077	None

7872	Ribonuclease E EC 3.1.26.12	None	None	None	fig|6666666.59332.peg.3586	None	None	fig|6666666.59336.peg.5346	None	None	None	fig|6666666.59340.peg.3169	None	fig|6666666.59342.peg.2350	None	None	None	None	None	fig|6666666.59346.peg.4937	None	None	None	None	None	None	None

8954	Phosphoribosylformylglycinamidine synthase, synthetase subunit EC 6.3.5.3 Phosphoribosylformylglycinamidine synthase, glutamine amidotransferase subunit EC 6.3.5.3	None	None	fig|6666666.59331.peg.5570	fig|6666666.59332.peg.5735	None	None	None	None	fig|6666666.59338.peg.720	None	None	None	fig|6666666.59342.peg.6068	None	None	None	None	None	fig|6666666.59346.peg.4694	None	None	None	None	None	None	None

8955	LysR family transcriptional regulator YnfL	None	None	None	None	None	None	None	fig|6666666.59333.peg.685	None	None	None	None	None	fig|6666666.71181.peg.2715	fig|6666666.59344.peg.3828	None	None	None	None	None	None	None	None	fig|6666666.59353.peg.3963	fig|6666666.59355.peg.3878	None

8952	hypothetical protein	fig|6666666.59329.peg.3175	None	None	None	None	None	fig|6666666.59336.peg.3281	None	fig|6666666.59338.peg.3210	None	None	None	None	None	None	fig|6666666.59345.peg.1984	None	fig|6666666.59347.peg.3739	None	None	None	None	None	None	None	None

2909	Permease of the drug metabolite transporter DMT superfamily	None	fig|6666666.59330.peg.4356	None	None	None	fig|6666666.59335.peg.5049	None	fig|6666666.59333.peg.4797	None	fig|6666666.59339.peg.969	None	None	None	None	None	None	None	None	None	fig|6666666.59348.peg.3077	None	None	None	None	None	None

4249	Co-activator of prophage gene expression IbrA	None	None	None	None	fig|6666666.59334.peg.1851	fig|6666666.59335.peg.4233	None	None	None	fig|6666666.59339.peg.1022	None	None	fig|6666666.59342.peg.71	None	None	None	None	None	fig|6666666.59346.peg.846	None	None	None	None	None	None	None

48	FIG01055362 hypothetical protein	fig|6666666.59329.peg.3534	None	None	None	None	None	None	None	None	None	None	None	None	fig|6666666.71181.peg.2563	fig|6666666.59344.peg.750	None	None	None	None	None	None	None	None	fig|6666666.59353.peg.1353	fig|6666666.59355.peg.2519	None

42	Metallo-beta-lactamase precursor	None	None	None	None	None	fig|6666666.59335.peg.2971	None	fig|6666666.59333.peg.5012	None	fig|6666666.59339.peg.1386	None	None	None	None	None	None	None	None	None	fig|6666666.59348.peg.323	None	fig|6666666.59350.peg.1256	None	None	None	None

6540	Aldose 1-epimerase EC 5.1.3.3	None	None	None	None	None	None	None	None	None	fig|6666666.59339.peg.6861	fig|6666666.59340.peg.2575	None	None	fig|6666666.71181.peg.5441	fig|6666666.59344.peg.4063	None	None	None	None	None	None	fig|6666666.59350.peg.6676	None	None	None	None

8302	Large-conductance mechanosensitive channel	None	None	None	None	None	None	None	None	None	None	None	None	None	fig|6666666.71181.peg.3434	fig|6666666.59344.peg.4917	None	None	None	None	None	None	fig|6666666.59350.peg.965	None	fig|6666666.59353.peg.4616	fig|6666666.59355.peg.4715	None

2262	FIG01203735 hypothetical protein	None	fig|6666666.59330.peg.1807	None	None	fig|6666666.59334.peg.709	None	None	None	None	None	None	fig|6666666.59337.peg.4206	fig|6666666.59342.peg.21	None	None	fig|6666666.59345.peg.1639	None	None	None	None	None	None	None	None	None	None

2041	RNA polymerase associated protein RapA EC 3.6.1.-	None	None	None	None	None	None	fig|6666666.59336.peg.4436	fig|6666666.59333.peg.242	None	fig|6666666.59339.peg.8157	fig|6666666.59340.peg.53	None	None	None	None	fig|6666666.59345.peg.4874	None	None	None	None	None	None	None	None	None	None

4818	hypothetical protein	None	None	fig|6666666.59331.peg.4602	fig|6666666.59332.peg.2009	None	None	None	fig|6666666.59333.peg.5324	None	None	None	fig|6666666.59337.peg.1018	None	None	None	None	None	fig|6666666.59347.peg.2663	None	None	None	None	None	None	None	None

11072	FIG01201513 hypothetical protein	fig|6666666.59329.peg.2677	None	None	None	None	None	fig|6666666.59336.peg.2649	None	None	None	None	fig|6666666.59337.peg.3800	fig|6666666.59342.peg.3473	None	None	fig|6666666.59345.peg.1949	None	None	None	None	None	None	None	None	None	None

10161	FIG00921245 hypothetical protein	None	None	fig|6666666.59331.peg.5876	fig|6666666.59332.peg.2778	None	None	None	None	None	None	None	None	None	None	None	None	fig|6666666.59341.peg.3679	fig|6666666.59347.peg.3310	fig|6666666.59346.peg.1910	None	None	None	None	None	None	None

10092	NADH-ubiquinone oxidoreductase chain F EC 1.6.5.3	None	None	None	None	None	None	None	None	None	None	None	None	None	fig|6666666.71181.peg.540	fig|6666666.59344.peg.1929	None	None	None	None	None	None	fig|6666666.59350.peg.2389	None	fig|6666666.59353.peg.1169	fig|6666666.59355.peg.307	None

10096	putative protein PaaI, possibly involved in aromatic compounds catabolism	None	None	None	None	None	None	None	None	None	None	None	None	None	fig|6666666.71181.peg.3801	fig|6666666.59344.peg.2498	None	None	None	None	None	None	fig|6666666.59350.peg.70	None	fig|6666666.59353.peg.4649	fig|6666666.59355.peg.4766	None

10098	hypothetical protein	None	None	None	fig|6666666.59332.peg.3894	None	fig|6666666.59335.peg.667	fig|6666666.59336.peg.4254	None	None	None	None	None	None	None	None	fig|6666666.59345.peg.3097	None	None	None	None	None	fig|6666666.59350.peg.8235	None	None	None	None

6970	7,8-dihydro-8-oxoguanine-triphosphatase, putative EC 3.6.1.-	None	None	None	None	None	None	None	None	None	None	None	None	None	fig|6666666.71181.peg.4734	fig|6666666.59344.peg.6261	None	None	None	None	None	None	fig|6666666.59350.peg.3115	None	fig|6666666.59353.peg.5061	fig|6666666.59355.peg.4669	None

6971	interphotoreceptor retinoid-binding protein	None	None	None	None	None	fig|6666666.59335.peg.2969	None	None	None	fig|6666666.59339.peg.1389	None	None	None	None	None	None	None	None	None	fig|6666666.59348.peg.3089	fig|6666666.59349.peg.3922	None	None	None	None	fig|6666666.59354.peg.2602

8382	Cell division protein FtsH EC 3.4.24.-	None	None	fig|6666666.59331.peg.5919	None	None	None	None	None	None	fig|6666666.59339.peg.4860	fig|6666666.59340.peg.1219	None	fig|6666666.59342.peg.2482	None	fig|6666666.59344.peg.2105	None	None	None	None	None	None	None	None	None	None	None

4032	P-hydroxybenzoate hydroxylase EC 1.14.13.2	None	None	None	None	None	None	None	None	None	None	None	None	None	fig|6666666.71181.peg.3385	fig|6666666.59344.peg.4815	None	None	None	None	None	None	fig|6666666.59350.peg.494	None	fig|6666666.59353.peg.3505	fig|6666666.59355.peg.3217	None

9331	Translation elongation factor Tu	None	fig|6666666.59330.peg.1667	None	None	None	None	None	None	None	None	None	None	None	None	fig|6666666.59344.peg.5469	None	None	None	None	None	None	None	fig|6666666.59351.peg.4152	fig|6666666.59353.peg.4445	fig|6666666.59355.peg.4582	None

9294	Putative nucleoside transporter YegT	None	None	None	fig|6666666.59332.peg.4379	None	None	None	None	None	fig|6666666.59339.peg.4571	None	None	None	fig|6666666.71181.peg.1257	fig|6666666.59344.peg.3104	None	None	None	None	fig|6666666.59348.peg.869	None	None	None	None	None	None

1604	hypothetical protein	None	None	fig|6666666.59331.peg.857	fig|6666666.59332.peg.702	None	None	None	None	fig|6666666.59338.peg.2222	None	fig|6666666.59340.peg.705	None	None	None	None	None	None	fig|6666666.59347.peg.2504	None	None	None	None	None	None	None	None

2870	hypothetical protein	None	fig|6666666.59330.peg.2684	None	None	None	None	fig|6666666.59336.peg.1959	None	None	None	None	fig|6666666.59337.peg.3352	fig|6666666.59342.peg.1494	None	None	fig|6666666.59345.peg.5453	None	None	None	None	None	None	None	None	None	None

3355	Chromate transport protein ChrA	None	None	None	None	fig|6666666.59334.peg.5455	None	None	None	None	None	None	None	None	fig|6666666.71181.peg.4749	fig|6666666.59344.peg.5900	None	None	None	None	None	None	None	None	fig|6666666.59353.peg.5056	fig|6666666.59355.peg.5084	None

5506	hypothetical protein	None	None	fig|6666666.59331.peg.457	fig|6666666.59332.peg.1770	None	None	None	None	None	None	None	None	None	None	None	None	None	None	None	None	fig|6666666.59349.peg.2458	None	fig|6666666.59351.peg.2315	None	None	fig|6666666.59354.peg.998

543	Transposase	fig|6666666.59329.peg.4850	None	None	None	None	None	None	None	None	None	None	None	None	fig|6666666.71181.peg.2858	fig|6666666.59344.peg.4405	None	None	None	None	None	None	None	None	fig|6666666.59353.peg.4317	fig|6666666.59355.peg.4385	None

544	conserved hypothetical protein	fig|6666666.59329.peg.5429	fig|6666666.59330.peg.2144	None	None	None	None	None	None	None	fig|6666666.59339.peg.3273	fig|6666666.59340.peg.682	fig|6666666.59337.peg.4070	None	None	None	None	None	None	None	None	None	None	None	None	None	None

6564	Succinyl-CoA ligase [ADP-forming] alpha chain EC 6.2.1.5	None	fig|6666666.59330.peg.2777	None	None	None	fig|6666666.59335.peg.3906	None	None	None	fig|6666666.59339.peg.7183	None	fig|6666666.59337.peg.4115	fig|6666666.59342.peg.483	None	None	None	None	None	None	None	None	None	None	None	None	None

7680	FIG01200841 hypothetical protein	fig|6666666.59329.peg.2641	None	fig|6666666.59331.peg.2768	fig|6666666.59332.peg.5979	None	None	None	None	None	None	None	fig|6666666.59337.peg.3262	None	None	None	None	None	None	fig|6666666.59346.peg.417	None	None	None	None	None	None	None

3823	FIG01212985 hypothetical protein	None	None	None	None	None	None	None	None	None	None	fig|6666666.59340.peg.3269	None	None	fig|6666666.71181.peg.706	fig|6666666.59344.peg.683	None	None	None	None	None	None	None	None	fig|6666666.59353.peg.991	fig|6666666.59355.peg.2382	None

10981	Type III secretion cytoplasmic protein YscK	None	fig|6666666.59330.peg.4700	None	None	None	None	None	fig|6666666.59333.peg.2999	None	None	None	fig|6666666.59337.peg.4470	fig|6666666.59342.peg.634	None	None	fig|6666666.59345.peg.1479	None	None	None	None	None	None	None	None	None	None

10982	Serine threonine protein phosphatase EC 3.1.3.16	None	None	None	None	None	None	None	None	None	None	fig|6666666.59340.peg.3426	None	None	fig|6666666.71181.peg.4595	fig|6666666.59344.peg.5846	None	None	None	None	None	None	None	None	fig|6666666.59353.peg.2474	fig|6666666.59355.peg.567	None

2203	FIG01205245 hypothetical protein	fig|6666666.59329.peg.5319	None	None	None	fig|6666666.59334.peg.560	None	None	None	fig|6666666.59338.peg.176	None	None	None	fig|6666666.59342.peg.2279	None	None	fig|6666666.59345.peg.3164	None	None	None	None	None	None	None	None	None	None

7997	Putative sugar ABC transporter precursor	None	None	None	None	None	fig|6666666.59335.peg.5367	None	None	None	None	None	None	None	fig|6666666.71181.peg.203	fig|6666666.59344.peg.247	None	None	None	None	None	None	None	None	fig|6666666.59353.peg.619	fig|6666666.59355.peg.102	None

8144	FIG01200690 hypothetical protein	fig|6666666.59329.peg.1552	None	None	None	None	None	fig|6666666.59336.peg.2543	fig|6666666.59333.peg.4377	None	None	None	fig|6666666.59337.peg.1900	None	None	None	fig|6666666.59345.peg.2834	None	None	None	None	None	None	None	None	None	None

9555	FIG01201424 hypothetical protein	None	fig|6666666.59330.peg.2337	None	None	None	None	fig|6666666.59336.peg.2448	None	None	None	fig|6666666.59340.peg.1419	fig|6666666.59337.peg.3601	None	None	None	fig|6666666.59345.peg.1859	None	None	None	None	None	None	None	None	None	None

9026	Type III secretion chaperone protein for YopN SycN,YscB	fig|6666666.59329.peg.6450	fig|6666666.59330.peg.3757	None	None	None	None	None	None	None	None	None	fig|6666666.59337.peg.4460	fig|6666666.59342.peg.622	None	None	fig|6666666.59345.peg.1488	None	None	None	None	None	None	None	None	None	None

57	Alpha-2-macroglobulin	None	None	None	None	None	None	None	None	None	None	None	None	None	fig|6666666.71181.peg.43	None	None	None	fig|6666666.59347.peg.394	None	None	None	fig|6666666.59350.peg.616	fig|6666666.59351.peg.4045	fig|6666666.59353.peg.1974	None	None

9999	Glutamate transport ATP-binding protein	None	None	None	None	fig|6666666.59334.peg.2382	None	None	None	None	None	None	None	None	fig|6666666.71181.peg.898	fig|6666666.59344.peg.4780	None	None	None	None	None	None	None	None	fig|6666666.59353.peg.4532	fig|6666666.59355.peg.4664	None

854	hypothetical protein	None	None	fig|6666666.59331.peg.1648	fig|6666666.59332.peg.5939	None	None	None	None	None	None	None	None	None	None	None	None	fig|6666666.59341.peg.3115	fig|6666666.59347.peg.3279	fig|6666666.59346.peg.2850	None	None	None	None	None	None	None

856	Putative phage-encoded peptidoglycan binding protein	None	None	None	None	None	fig|6666666.59335.peg.2118	fig|6666666.59336.peg.5839	fig|6666666.59333.peg.2044	None	fig|6666666.59339.peg.5716	None	None	fig|6666666.59342.peg.4250	None	None	None	None	None	None	None	None	None	None	None	None	None

11356	hypothetical protein	None	None	None	None	None	fig|6666666.59335.peg.3415	None	None	fig|6666666.59338.peg.4683	fig|6666666.59339.peg.8798	None	None	None	None	None	None	fig|6666666.59341.peg.228	fig|6666666.59347.peg.550	None	None	None	None	None	None	None	None

8258	Adenylate cyclase EC 4.6.1.1 Guanylate cyclase EC 4.6.1.2	None	None	fig|6666666.59331.peg.1438	fig|6666666.59332.peg.3219	None	None	None	None	None	None	None	None	None	None	None	None	None	None	None	None	fig|6666666.59349.peg.2579	None	fig|6666666.59351.peg.5350	None	None	fig|6666666.59354.peg.5651

2332	Antirestriction protein	None	None	None	None	None	None	None	None	None	None	None	None	None	fig|6666666.71181.peg.681	fig|6666666.59344.peg.651	None	None	None	None	None	None	fig|6666666.59350.peg.2865	None	fig|6666666.59353.peg.1015	fig|6666666.59355.peg.2357	None

2432	FIG01200903 hypothetical protein	fig|6666666.59329.peg.2131	None	None	None	None	None	fig|6666666.59336.peg.1033	None	None	None	None	fig|6666666.59337.peg.2286	fig|6666666.59342.peg.5338	None	None	fig|6666666.59345.peg.2012	None	None	None	None	None	None	None	None	None	None

7838	FIG01204095 hypothetical protein	fig|6666666.59329.peg.4928	None	None	None	None	None	fig|6666666.59336.peg.3851	None	None	None	None	fig|6666666.59337.peg.3275	fig|6666666.59342.peg.6369	None	None	fig|6666666.59345.peg.539	None	None	None	None	None	None	None	None	None	None

10174	hypothetical protein	None	None	fig|6666666.59331.peg.56	None	None	None	None	fig|6666666.59333.peg.5376	None	None	None	None	None	fig|6666666.71181.peg.5377	None	None	None	None	None	None	fig|6666666.59349.peg.4366	None	None	None	None	fig|6666666.59354.peg.3435

1238	FIG01056067 hypothetical protein	None	None	None	None	None	None	None	None	None	None	None	fig|6666666.59337.peg.298	None	fig|6666666.71181.peg.1244	fig|6666666.59344.peg.164	None	None	None	None	None	None	None	None	fig|6666666.59353.peg.475	fig|6666666.59355.peg.2200	None

6509	Transcriptional regulators, LysR family	fig|6666666.59329.peg.3272	None	None	None	None	None	fig|6666666.59336.peg.5807	None	None	None	None	fig|6666666.59337.peg.5432	fig|6666666.59342.peg.4851	None	None	fig|6666666.59345.peg.3998	None	None	None	None	None	None	None	None	None	None

8462	hypothetical protein	None	None	None	None	None	None	None	None	None	None	None	None	None	fig|6666666.71181.peg.3304	fig|6666666.59344.peg.4846	None	None	None	None	None	None	None	fig|6666666.59351.peg.2660	fig|6666666.59353.peg.3482	fig|6666666.59355.peg.3241	None

8466	probable membrane protein YPO2863	None	fig|6666666.59330.peg.3489	None	None	None	None	fig|6666666.59336.peg.2530	None	fig|6666666.59338.peg.1755	None	None	None	fig|6666666.59342.peg.5745	None	None	None	None	fig|6666666.59347.peg.123	None	None	None	None	None	None	None	None

2223	Ribosomal protein S3	None	None	None	None	None	None	None	None	None	None	None	None	None	fig|6666666.71181.peg.2608	fig|6666666.59344.peg.928	None	None	None	None	None	None	None	fig|6666666.59351.peg.971	fig|6666666.59353.peg.2603	fig|6666666.59355.peg.2599	None

7784	Glucans biosynthesis protein G precursor	None	None	None	None	None	None	None	None	None	None	None	None	None	fig|6666666.71181.peg.1586	fig|6666666.59344.peg.2393	None	None	None	None	None	None	fig|6666666.59350.peg.93	None	fig|6666666.59353.peg.278	fig|6666666.59355.peg.1317	None

10826	hypothetical protein	None	None	None	None	None	None	fig|6666666.59336.peg.2051	None	None	None	None	fig|6666666.59337.peg.4306	fig|6666666.59342.peg.6432	None	None	fig|6666666.59345.peg.3895	fig|6666666.59341.peg.3603	None	None	None	None	None	None	None	None	None

10824	Phenylacetate-CoA oxygenase, PaaJ subunit	None	None	None	None	None	None	None	None	None	None	None	None	None	fig|6666666.71181.peg.4674	fig|6666666.59344.peg.6175	None	None	None	None	None	None	fig|6666666.59350.peg.2291	None	fig|6666666.59353.peg.3912	fig|6666666.59355.peg.4250	None

7818	hypothetical protein	None	None	None	None	None	None	None	None	None	None	None	None	None	fig|6666666.71181.peg.4627	fig|6666666.59344.peg.5892	None	None	None	None	None	fig|6666666.59349.peg.4904	None	None	fig|6666666.59353.peg.4983	fig|6666666.59355.peg.5066	None

2666	Alkanesulfonates-binding protein	None	None	None	None	None	None	None	None	None	None	None	None	None	fig|6666666.71181.peg.815	fig|6666666.59344.peg.62	None	None	None	None	None	None	None	fig|6666666.59351.peg.1461	fig|6666666.59353.peg.1678	fig|6666666.59355.peg.1042	None

9375	NADH-ubiquinone oxidoreductase chain K EC 1.6.5.3	None	None	None	None	None	None	None	None	None	None	None	None	None	fig|6666666.71181.peg.535	fig|6666666.59344.peg.1937	None	None	None	None	None	None	fig|6666666.59350.peg.2612	None	fig|6666666.59353.peg.1163	fig|6666666.59355.peg.312	None

9376	hypothetical protein	None	None	fig|6666666.59331.peg.10	None	fig|6666666.59334.peg.1092	None	None	None	None	None	None	None	fig|6666666.59342.peg.2276	None	None	fig|6666666.59345.peg.3156	None	fig|6666666.59347.peg.2025	None	None	None	None	None	None	None	None

1921	Ribosomal protein L11 methyltransferase EC 2.1.1.-	None	None	fig|6666666.59331.peg.3198	None	None	None	fig|6666666.59336.peg.2270	None	fig|6666666.59338.peg.3733	None	fig|6666666.59340.peg.818	None	None	None	None	None	None	fig|6666666.59347.peg.4492	None	None	None	None	None	None	None	None

8403	FIG01199888 hypothetical protein	fig|6666666.59329.peg.1080	fig|6666666.59330.peg.2761	None	None	None	None	fig|6666666.59336.peg.1846	None	None	None	None	fig|6666666.59337.peg.324	None	None	None	fig|6666666.59345.peg.2092	None	None	None	None	None	None	None	None	None	None

8334	FIG01200789 hypothetical protein	None	None	None	None	None	None	fig|6666666.59336.peg.6325	fig|6666666.59333.peg.817	None	None	None	fig|6666666.59337.peg.2857	fig|6666666.59342.peg.2542	None	None	fig|6666666.59345.peg.1739	None	None	None	None	None	None	None	None	None	None

5904	Predicted exporter of the RND superfamily	None	None	None	fig|6666666.59332.peg.2174	None	fig|6666666.59335.peg.3612	fig|6666666.59336.peg.4824	None	None	fig|6666666.59339.peg.588	None	None	None	None	None	None	None	None	None	fig|6666666.59348.peg.5167	None	None	None	None	None	None

9606	YbbM seven transmembrane helix protein	None	None	None	None	fig|6666666.59334.peg.1317	None	fig|6666666.59336.peg.2592	None	fig|6666666.59338.peg.4545	None	None	None	fig|6666666.59342.peg.3909	None	None	None	fig|6666666.59341.peg.5394	None	None	None	None	None	None	None	None	None

2608	Dipeptide-binding ABC transporter, periplasmic substrate-binding component TC 3.A.1.5.2 ; Putative hemin-binding lipoprotein	None	None	None	None	None	None	None	None	None	None	None	None	None	fig|6666666.71181.peg.3293	fig|6666666.59344.peg.4650	None	None	None	None	None	None	fig|6666666.59350.peg.805	None	fig|6666666.59353.peg.2433	fig|6666666.59355.peg.526	None

10784	putative acetyltransferase	fig|6666666.59329.peg.3952	None	None	None	None	None	fig|6666666.59336.peg.517	None	None	None	None	fig|6666666.59337.peg.2590	fig|6666666.59342.peg.1538	None	None	fig|6666666.59345.peg.763	None	None	None	None	None	None	None	None	None	None

7010	Cell invasion protein SipD Salmonella invasion protein D	fig|6666666.59329.peg.534	fig|6666666.59330.peg.3280	None	None	None	None	None	None	None	None	None	fig|6666666.59337.peg.2810	fig|6666666.59342.peg.4694	None	None	fig|6666666.59345.peg.1452	None	None	None	None	None	None	None	None	None	None

3780	Flagellar hook-associated protein FlgL	None	None	None	None	None	fig|6666666.59335.peg.5017	fig|6666666.59336.peg.2924	None	None	fig|6666666.59339.peg.5328	fig|6666666.59340.peg.3431	None	None	None	None	None	fig|6666666.59341.peg.3266	None	None	None	None	None	None	None	None	None

4361	3,4-dihydroxyphenylacetate 2,3-dioxygenase EC 1.13.11.15	None	None	None	None	None	None	None	None	None	None	None	None	None	fig|6666666.71181.peg.4553	fig|6666666.59344.peg.1420	None	None	None	None	None	None	fig|6666666.59350.peg.3930	None	fig|6666666.59353.peg.6	fig|6666666.59355.peg.612	None

4360	ParB-like nuclease	fig|6666666.59329.peg.2562	fig|6666666.59330.peg.2504	None	None	None	None	None	None	fig|6666666.59338.peg.1707	None	None	fig|6666666.59337.peg.610	None	None	None	None	fig|6666666.59341.peg.4309	None	None	None	None	None	None	None	None	None

3104	putative lipoprotein	None	None	None	None	None	None	None	None	None	None	fig|6666666.59340.peg.3119	None	None	fig|6666666.71181.peg.704	fig|6666666.59344.peg.681	None	None	None	None	None	None	None	None	fig|6666666.59353.peg.993	fig|6666666.59355.peg.2380	None

8671	CBS-domain containing protein	None	None	None	None	fig|6666666.59334.peg.225	None	None	None	None	fig|6666666.59339.peg.6426	fig|6666666.59340.peg.2341	None	fig|6666666.59342.peg.6146	None	None	None	fig|6666666.59341.peg.1421	None	None	None	None	None	None	None	None	None

11314	Tricarboxylate transport membrane protein TctA	None	None	None	None	fig|6666666.59334.peg.4013	None	None	fig|6666666.59333.peg.3043	None	fig|6666666.59339.peg.3966	None	None	None	None	None	None	None	None	None	fig|6666666.59348.peg.3361	None	fig|6666666.59350.peg.6743	None	None	None	None

8099	2-dehydropantoate 2-reductase EC 1.1.1.169	None	None	fig|6666666.59331.peg.5070	None	fig|6666666.59334.peg.4948	None	None	None	None	None	fig|6666666.59340.peg.2967	None	None	None	None	None	None	None	None	fig|6666666.59348.peg.4846	fig|6666666.59349.peg.1765	None	None	None	None	None

9159	Peptidase, T4 family	None	None	None	None	None	None	None	None	None	None	None	None	None	fig|6666666.71181.peg.1102	fig|6666666.59344.peg.3607	None	None	None	None	None	None	fig|6666666.59350.peg.631	None	fig|6666666.59353.peg.2162	fig|6666666.59355.peg.1857	None

2380	membrane protein, putative	None	None	None	None	None	None	None	None	None	None	None	None	None	fig|6666666.71181.peg.4087	fig|6666666.59344.peg.5951	None	None	None	None	None	None	fig|6666666.59350.peg.7982	None	fig|6666666.59353.peg.4833	fig|6666666.59355.peg.4903	None

9288	Phosphogluconate repressor HexR, RpiR family	None	None	fig|6666666.59331.peg.2517	None	None	fig|6666666.59335.peg.2546	None	None	None	fig|6666666.59339.peg.3516	None	fig|6666666.59337.peg.4638	None	None	None	None	fig|6666666.59341.peg.1901	None	None	None	None	None	None	None	None	None

2169	NADH-ubiquinone oxidoreductase chain J EC 1.6.5.3	None	None	None	None	None	None	None	None	None	None	None	None	None	fig|6666666.71181.peg.536	fig|6666666.59344.peg.1936	None	None	None	None	None	None	fig|6666666.59350.peg.7345	None	fig|6666666.59353.peg.1164	fig|6666666.59355.peg.311	None

3297	L-proline glycine betaine ABC transport system permease protein ProV TC 3.A.1.12.1	None	None	None	None	None	None	None	fig|6666666.59333.peg.971	None	None	None	None	None	fig|6666666.71181.peg.3583	fig|6666666.59344.peg.5568	None	None	None	None	None	None	None	None	fig|6666666.59353.peg.4129	fig|6666666.59355.peg.4286	None

6892	Aldo keto reductase	None	None	None	None	None	None	None	None	None	fig|6666666.59339.peg.7439	None	None	None	None	None	None	None	None	fig|6666666.59346.peg.2615	fig|6666666.59348.peg.1895	fig|6666666.59349.peg.4162	None	None	None	None	fig|6666666.59354.peg.4059

10308	Prophage baseplate assembly protein V	None	None	None	None	None	fig|6666666.59335.peg.303	None	None	None	fig|6666666.59339.peg.2539	None	None	None	fig|6666666.71181.peg.2892	None	None	None	None	None	fig|6666666.59348.peg.4773	None	None	None	None	fig|6666666.59355.peg.4417	None

3335	FIG01203128 hypothetical protein	fig|6666666.59329.peg.1256	fig|6666666.59330.peg.3868	None	None	None	None	fig|6666666.59336.peg.3328	None	None	None	None	fig|6666666.59337.peg.1935	None	None	None	fig|6666666.59345.peg.4027	None	None	None	None	None	None	None	None	None	None

4135	hypothetical protein	None	None	None	None	None	None	None	None	fig|6666666.59338.peg.2061	fig|6666666.59339.peg.3260	None	None	None	None	None	None	None	fig|6666666.59347.peg.2556	None	None	None	fig|6666666.59350.peg.6142	fig|6666666.59351.peg.1913	None	None	None

4136	FIG01055893 hypothetical protein	None	None	None	None	None	None	None	None	None	None	None	None	None	fig|6666666.71181.peg.1055	fig|6666666.59344.peg.4956	None	None	None	None	None	None	fig|6666666.59350.peg.2908	None	fig|6666666.59353.peg.4521	fig|6666666.59355.peg.4631	None

3843	Isocitrate dehydrogenase phosphatase EC 2.7.11.5 kinase EC 3.1.3.-	None	None	None	None	None	None	None	None	None	None	None	None	None	None	fig|6666666.59344.peg.2693	None	None	None	None	None	fig|6666666.59349.peg.2705	None	fig|6666666.59351.peg.5179	fig|6666666.59353.peg.3033	fig|6666666.59355.peg.2940	None

3846	hypothetical protein	None	fig|6666666.59330.peg.5138	None	None	None	None	fig|6666666.59336.peg.2934	None	None	None	None	None	fig|6666666.59342.peg.5504	None	None	fig|6666666.59345.peg.5587	fig|6666666.59341.peg.262	None	None	None	None	None	None	None	None	None

5969	Solute-binding periplasmic protein of iron siderophore ABC transporter	None	None	None	None	None	None	None	None	None	None	None	None	None	fig|6666666.71181.peg.3396	fig|6666666.59344.peg.4831	None	None	None	None	fig|6666666.59348.peg.5276	None	None	None	fig|6666666.59353.peg.3495	fig|6666666.59355.peg.879	None

2624	FIG01201495 hypothetical protein	None	fig|6666666.59330.peg.2185	None	None	None	None	fig|6666666.59336.peg.3305	None	None	None	None	fig|6666666.59337.peg.4918	fig|6666666.59342.peg.90	None	None	fig|6666666.59345.peg.4376	None	None	None	None	None	None	None	None	None	None

9231	Capsular polysaccharide synthesis enzyme CpsJ, membrane protein, export of O-antigen and teichoic acid	None	fig|6666666.59330.peg.3602	None	None	None	None	fig|6666666.59336.peg.1146	fig|6666666.59333.peg.983	None	None	None	fig|6666666.59337.peg.3002	None	None	None	fig|6666666.59345.peg.634	None	None	None	None	None	None	None	None	None	None

9233	Ribonucleotide reductase of class Ib aerobic , alpha subunit EC 1.17.4.1	None	None	fig|6666666.59331.peg.1233	fig|6666666.59332.peg.2069	fig|6666666.59334.peg.2021	None	None	None	None	None	None	None	None	None	None	None	None	None	None	fig|6666666.59348.peg.3685	None	fig|6666666.59350.peg.5087	None	None	None	None

9059	Adenine deaminase EC 3.5.4.2	None	None	None	None	None	None	None	None	None	None	None	None	None	fig|6666666.71181.peg.2073	fig|6666666.59344.peg.1042	None	None	None	None	None	None	fig|6666666.59350.peg.867	None	fig|6666666.59353.peg.732	fig|6666666.59355.peg.973	None

9055	FIG001592 Phosphocarrier protein kinase phosphorylase, nitrogen regulation associated	None	None	fig|6666666.59331.peg.5483	None	fig|6666666.59334.peg.1751	None	fig|6666666.59336.peg.2366	None	None	None	fig|6666666.59340.peg.187	None	None	None	None	fig|6666666.59345.peg.3721	None	None	None	None	None	None	None	None	None	None

774	VgrG protein	fig|6666666.59329.peg.3038	None	None	None	None	fig|6666666.59335.peg.5069	None	None	None	None	None	fig|6666666.59337.peg.3019	None	None	None	fig|6666666.59345.peg.3587	None	fig|6666666.59347.peg.3632	None	None	None	None	None	None	None	None

778	FIG01201851 hypothetical protein	None	None	None	None	None	None	fig|6666666.59336.peg.5605	fig|6666666.59333.peg.3735	None	None	None	fig|6666666.59337.peg.4034	fig|6666666.59342.peg.5890	None	None	fig|6666666.59345.peg.209	None	None	None	None	None	None	None	None	None	None

77	Type III secretion effector protein YopR, encoded by YscH	fig|6666666.59329.peg.2731	fig|6666666.59330.peg.4697	None	None	None	None	None	None	None	None	None	fig|6666666.59337.peg.4467	fig|6666666.59342.peg.630	None	None	fig|6666666.59345.peg.1482	None	None	None	None	None	None	None	None	None	None

2973	Sigma-54 dependent transcriptional regulator	None	None	None	fig|6666666.59332.peg.1447	None	None	None	None	None	fig|6666666.59339.peg.1218	None	None	fig|6666666.59342.peg.2660	None	fig|6666666.59344.peg.1597	None	None	None	fig|6666666.59346.peg.3571	None	None	None	None	None	None	None

7432	FIG01203861 hypothetical protein	fig|6666666.59329.peg.5934	fig|6666666.59330.peg.4236	None	None	fig|6666666.59334.peg.2728	None	fig|6666666.59336.peg.1766	None	None	None	None	None	None	None	None	fig|6666666.59345.peg.4442	None	None	None	None	None	None	None	None	None	None

2364	Uncharacterized protein conserved in bacteria	None	None	None	None	None	None	None	None	None	None	None	None	None	fig|6666666.71181.peg.2323	fig|6666666.59344.peg.4543	None	None	None	None	None	None	fig|6666666.59350.peg.1053	None	fig|6666666.59353.peg.3331	fig|6666666.59355.peg.3463	None

1860	acetyltransferase, GNAT family	None	fig|6666666.59330.peg.3268	fig|6666666.59331.peg.1145	None	None	None	None	None	fig|6666666.59338.peg.4158	None	None	fig|6666666.59337.peg.1233	None	None	None	None	None	None	fig|6666666.59346.peg.5155	None	None	None	None	None	None	None

3727	FIG01055643 hypothetical protein	None	None	None	None	None	None	None	None	None	None	None	None	None	fig|6666666.71181.peg.4786	fig|6666666.59344.peg.2084	None	None	None	None	None	None	fig|6666666.59350.peg.4362	None	fig|6666666.59353.peg.3120	fig|6666666.59355.peg.477	None

3720	Glycerol-3-phosphate transporter	None	fig|6666666.59330.peg.2824	None	None	None	fig|6666666.59335.peg.1735	fig|6666666.59336.peg.6216	None	None	fig|6666666.59339.peg.7340	None	None	fig|6666666.59342.peg.5567	None	None	None	None	None	None	None	None	None	None	None	None	None

5110	Thiol peroxidase, Bcp-type EC 1.11.1.15	None	fig|6666666.59330.peg.3638	None	None	fig|6666666.59334.peg.373	None	fig|6666666.59336.peg.1614	None	None	None	None	None	fig|6666666.59342.peg.2942	None	None	None	None	None	None	fig|6666666.59348.peg.76	None	None	None	None	None	None

2490	hypothetical protein	None	None	None	None	None	fig|6666666.59335.peg.2610	None	None	None	fig|6666666.59339.peg.2245	None	None	None	None	None	None	fig|6666666.59341.peg.3413	fig|6666666.59347.peg.4229	None	fig|6666666.59348.peg.1509	None	None	None	None	None	None

6023	Lysophospholipase L2 EC 3.1.1.5	None	fig|6666666.59330.peg.1545	None	None	None	None	None	fig|6666666.59333.peg.800	None	fig|6666666.59339.peg.8541	None	fig|6666666.59337.peg.1627	None	None	None	None	None	None	None	fig|6666666.59348.peg.744	None	None	None	None	None	None

10077	hypothetical protein	None	None	fig|6666666.59331.peg.5405	fig|6666666.59332.peg.676	None	fig|6666666.59335.peg.3206	None	None	None	fig|6666666.59339.peg.1746	None	None	None	None	None	None	None	None	None	fig|6666666.59348.peg.617	None	None	None	None	None	None

5302	hypothetical protein	fig|6666666.59329.peg.3092	None	None	None	None	None	fig|6666666.59336.peg.2848	None	None	None	None	fig|6666666.59337.peg.3764	fig|6666666.59342.peg.1883	None	None	fig|6666666.59345.peg.2791	None	None	None	None	None	None	None	None	None	None

5305	Type III secretion negative regulator LscZ	None	fig|6666666.59330.peg.3758	None	None	None	None	None	fig|6666666.59333.peg.5139	None	None	None	fig|6666666.59337.peg.4459	fig|6666666.59342.peg.621	None	None	fig|6666666.59345.peg.1489	None	None	None	None	None	None	None	None	None	None

6610	3-oxoacyl-[acyl-carrier-protein] synthase, KASII EC 2.3.1.41	fig|6666666.59329.peg.5938	None	fig|6666666.59331.peg.1747	None	None	None	None	None	None	fig|6666666.59339.peg.7056	None	None	None	None	fig|6666666.59344.peg.1184	None	None	None	None	None	None	fig|6666666.59350.peg.4749	None	None	None	None

2349	Outer membrane protein OmpT	fig|6666666.59329.peg.4528	None	fig|6666666.59331.peg.2477	None	None	None	None	None	None	fig|6666666.59339.peg.454	None	fig|6666666.59337.peg.3378	fig|6666666.59342.peg.2504	None	None	None	None	None	None	None	None	None	None	None	None	None

1884	Bores hole in peptidoglycan layer allowing type IV secretion complex assembly to occur VirB1	fig|6666666.59329.peg.3830	None	None	None	None	None	fig|6666666.59336.peg.3358	None	None	None	None	fig|6666666.59337.peg.3062	None	None	None	fig|6666666.59345.peg.4739	None	None	None	None	None	fig|6666666.59350.peg.5105	None	None	None	None

2121	L-aspartate-beta-decarboxylase	None	None	None	None	fig|6666666.59334.peg.2875	None	None	fig|6666666.59333.peg.4004	fig|6666666.59338.peg.2503	None	None	None	None	None	None	None	fig|6666666.59341.peg.2635	fig|6666666.59347.peg.1664	None	None	None	None	None	None	None	None

4916	tRNA Uracil54-C5- -methyltransferase EC 2.1.1.35	fig|6666666.59329.peg.2301	None	fig|6666666.59331.peg.4348	None	None	None	None	None	fig|6666666.59338.peg.4189	fig|6666666.59339.peg.8594	fig|6666666.59340.peg.2962	None	None	None	None	None	None	None	None	None	None	None	None	None	None	None

9680	putative lipoprotein	fig|6666666.59329.peg.3474	fig|6666666.59330.peg.1801	None	None	None	None	fig|6666666.59336.peg.549	None	None	None	None	fig|6666666.59337.peg.3420	None	None	None	fig|6666666.59345.peg.1969	None	None	None	None	None	None	None	None	None	None

7253	putative chloramphenicol 3-O phosphotransferase	None	fig|6666666.59330.peg.4916	None	fig|6666666.59332.peg.3566	None	None	None	None	None	None	None	None	None	None	None	None	None	None	None	None	fig|6666666.59349.peg.1448	fig|6666666.59350.peg.2360	None	None	None	fig|6666666.59354.peg.2561

82	PvcB protein, related to amino acid oxidizing enzymes	None	None	None	None	None	None	None	None	None	None	None	None	None	fig|6666666.71181.peg.415	fig|6666666.59344.peg.1309	None	None	None	None	None	None	fig|6666666.59350.peg.3655	None	fig|6666666.59353.peg.83	fig|6666666.59355.peg.691	None

6588	Cyclic di-GMP binding protein precursor	None	None	None	None	None	None	fig|6666666.59336.peg.1517	None	None	None	fig|6666666.59340.peg.119	fig|6666666.59337.peg.5059	fig|6666666.59342.peg.85	None	fig|6666666.59344.peg.2442	None	None	None	None	None	None	None	None	None	None	None

9876	Zinc-regulated TonB-dependent outer membrane receptor	fig|6666666.59329.peg.5166	fig|6666666.59330.peg.2056	None	None	None	None	None	fig|6666666.59333.peg.4176	None	None	None	fig|6666666.59337.peg.4326	None	None	None	fig|6666666.59345.peg.3921	None	None	None	None	None	None	None	None	None	None

3368	FIG01205478 hypothetical protein	fig|6666666.59329.peg.48	fig|6666666.59330.peg.628	None	None	None	None	fig|6666666.59336.peg.2735	None	None	None	None	fig|6666666.59337.peg.3887	None	None	None	fig|6666666.59345.peg.4941	None	None	None	None	None	None	None	None	None	None

659	Retron-type RNA-directed DNA polymerase EC 2.7.7.49	None	None	None	None	fig|6666666.59334.peg.3385	None	None	None	None	fig|6666666.59339.peg.602	None	None	None	None	None	None	None	None	None	None	fig|6666666.59349.peg.3044	None	fig|6666666.59351.peg.386	None	None	fig|6666666.59354.peg.1766

3885	Conserved hypothetical protein perhaps related to histidine degradation	None	None	None	None	None	None	None	None	None	None	None	None	None	fig|6666666.71181.peg.3882	fig|6666666.59344.peg.214	None	None	None	None	None	None	fig|6666666.59350.peg.4119	None	fig|6666666.59353.peg.5108	fig|6666666.59355.peg.2242	None

3881	Promiscuous sugar phosphatase YidA, haloacid dehalogenase-like phosphatase family	None	None	fig|6666666.59331.peg.3155	fig|6666666.59332.peg.5610	None	fig|6666666.59335.peg.5823	None	None	None	fig|6666666.59339.peg.4036	None	None	None	None	fig|6666666.59344.peg.4176	None	None	None	None	None	None	None	None	None	None	None

8324	Sucrose-6-phosphate hydrolase EC 3.2.1.26	fig|6666666.59329.peg.5123	None	fig|6666666.59331.peg.3052	fig|6666666.59332.peg.6218	fig|6666666.59334.peg.2039	None	None	None	None	None	None	None	fig|6666666.59342.peg.5642	None	None	None	None	None	None	None	None	None	None	None	None	None

6008	Sulfite reductase [NADPH] hemoprotein beta-component EC 1.8.1.2	None	None	fig|6666666.59331.peg.5707	None	fig|6666666.59334.peg.155	None	fig|6666666.59336.peg.6212	fig|6666666.59333.peg.5488	None	None	fig|6666666.59340.peg.2944	None	None	None	None	None	None	None	None	None	None	None	None	None	None	None

2937	Aerobic cobaltochelatase CobS subunit EC 6.6.1.2	None	None	None	fig|6666666.59332.peg.6061	None	fig|6666666.59335.peg.3665	None	None	None	fig|6666666.59339.peg.3302	None	None	None	None	None	None	fig|6666666.59341.peg.3592	None	None	None	None	fig|6666666.59350.peg.5805	None	None	None	None

2930	hypothetical protein	fig|6666666.59329.peg.2694	fig|6666666.59330.peg.5083	None	None	None	None	fig|6666666.59336.peg.3584	None	None	None	None	fig|6666666.59337.peg.1157	None	None	None	fig|6666666.59345.peg.3465	None	None	None	None	None	None	None	None	None	None

2154	VapC toxin protein	None	None	None	None	None	None	None	None	None	None	None	None	None	fig|6666666.71181.peg.4783	fig|6666666.59344.peg.5482	None	None	None	None	None	None	fig|6666666.59350.peg.6009	None	fig|6666666.59353.peg.2201	fig|6666666.59355.peg.1894	None

7478	FIG01200195 hypothetical protein	fig|6666666.59329.peg.4731	None	fig|6666666.59331.peg.3227	fig|6666666.59332.peg.3094	None	fig|6666666.59335.peg.3197	None	None	None	fig|6666666.59339.peg.1755	None	None	None	None	None	None	None	None	None	None	None	None	None	None	None	None

7476	Putative ABC transporter, ATP-binding protein	None	None	None	None	None	None	None	None	None	None	None	None	None	fig|6666666.71181.peg.3748	fig|6666666.59344.peg.6031	None	None	None	None	None	None	fig|6666666.59350.peg.4261	None	fig|6666666.59353.peg.4657	fig|6666666.59355.peg.3867	None

3761	hypothetical protein	None	fig|6666666.59330.peg.5120	None	None	None	fig|6666666.59335.peg.6006	None	None	None	fig|6666666.59339.peg.7314	None	None	fig|6666666.59342.peg.864	None	None	fig|6666666.59345.peg.3389	None	None	None	None	None	None	None	None	None	None

1293	Galactose methyl galactoside ABC transport system, D-galactose-binding periplasmic protein MglB TC 3.A.1.2.3	None	None	None	None	None	None	None	None	None	None	None	None	None	fig|6666666.71181.peg.194	fig|6666666.59344.peg.235	None	fig|6666666.59341.peg.303	None	None	None	None	None	None	fig|6666666.59353.peg.628	fig|6666666.59355.peg.111	None

7278	Site-specific recombinase, phage integrase family	None	None	fig|6666666.59331.peg.2200	fig|6666666.59332.peg.1170	None	None	None	fig|6666666.59333.peg.2577	None	None	None	fig|6666666.59337.peg.928	None	None	None	None	None	None	None	None	None	fig|6666666.59350.peg.8240	None	None	None	None

8823	Ribosomal protein S6 glutaminyl transferase	fig|6666666.59329.peg.1207	None	None	None	None	None	None	None	fig|6666666.59338.peg.4810	fig|6666666.59339.peg.5074	None	None	None	None	fig|6666666.59344.peg.357	None	None	None	None	fig|6666666.59348.peg.4028	None	None	None	None	None	None

7642	HIPA PROTEIN	fig|6666666.59329.peg.2254	None	fig|6666666.59331.peg.283	None	None	None	None	None	None	None	None	None	None	None	None	None	None	None	fig|6666666.59346.peg.5195	None	None	fig|6666666.59350.peg.1813	fig|6666666.59351.peg.329	None	None	None

7647	Sodium-dependent transporter	None	None	fig|6666666.59331.peg.5184	None	None	None	None	None	fig|6666666.59338.peg.5348	None	fig|6666666.59340.peg.3499	None	None	None	None	None	None	None	fig|6666666.59346.peg.3287	None	None	None	None	None	None	fig|6666666.59354.peg.1295

3308	Type cbb3 cytochrome oxidase biogenesis protein CcoG, involved in Cu oxidation	None	None	None	None	None	None	None	None	None	fig|6666666.59339.peg.8077	fig|6666666.59340.peg.1393	None	None	None	None	None	None	None	None	fig|6666666.59348.peg.60	None	None	fig|6666666.59351.peg.3119	None	None	fig|6666666.59354.peg.1230

5490	Scaffold protein for [4Fe-4S] cluster assembly ApbC, MRP-like	None	None	None	fig|6666666.59332.peg.2119	fig|6666666.59334.peg.4892	None	None	None	None	None	None	None	None	None	None	None	None	None	None	None	None	fig|6666666.59350.peg.8189	fig|6666666.59351.peg.2807	None	None	fig|6666666.59354.peg.3055

5637	FIG01202050 hypothetical protein	None	fig|6666666.59330.peg.5028	None	None	None	None	None	None	fig|6666666.59338.peg.2898	fig|6666666.59339.peg.5958	None	None	fig|6666666.59342.peg.6148	None	None	None	None	fig|6666666.59347.peg.2547	None	None	None	None	None	None	None	None

5983	Outer membrane lipoprotein YidQ	None	None	None	None	None	None	None	None	None	None	None	None	None	fig|6666666.71181.peg.637	fig|6666666.59344.peg.4194	None	None	None	None	None	None	None	None	fig|6666666.59353.peg.3042	fig|6666666.59355.peg.2306	None

5985	Integral inner membrane protein of type IV secretion complex VirB6	None	None	None	None	None	None	fig|6666666.59336.peg.1723	None	None	None	None	fig|6666666.59337.peg.3068	None	None	None	fig|6666666.59345.peg.4733	None	None	None	None	None	fig|6666666.59350.peg.3713	None	None	None	None

270	Predicted nucleoside ABC transporter, permease 1 component	None	None	None	None	None	None	None	None	fig|6666666.59338.peg.603	None	None	None	None	None	None	None	fig|6666666.59341.peg.4762	fig|6666666.59347.peg.2977	None	None	None	fig|6666666.59350.peg.2280	None	None	None	None

279	cAMP-binding proteins - catabolite gene activator and regulatory subunit of cAMP-dependent protein kinases	None	None	fig|6666666.59331.peg.5335	None	None	None	None	None	None	None	None	None	None	None	None	None	fig|6666666.59341.peg.4180	fig|6666666.59347.peg.1297	fig|6666666.59346.peg.721	None	None	None	None	None	None	None

10708	FIG01201170 hypothetical protein	None	None	None	None	None	None	fig|6666666.59336.peg.6405	fig|6666666.59333.peg.4356	None	None	None	fig|6666666.59337.peg.1758	None	None	None	fig|6666666.59345.peg.886	None	None	None	None	None	None	None	None	None	None

9258	extracellular secretory protein nucE	None	None	None	None	None	None	None	None	None	None	None	None	None	fig|6666666.71181.peg.3951	fig|6666666.59344.peg.5436	None	None	None	None	None	None	None	None	fig|6666666.59353.peg.4808	fig|6666666.59355.peg.4862	None

2682	FIG00784179 hypothetical protein	None	None	None	None	None	None	None	None	fig|6666666.59338.peg.712	None	None	fig|6666666.59337.peg.4454	None	None	None	None	None	None	None	fig|6666666.59348.peg.3786	None	None	fig|6666666.59351.peg.3018	None	None	None

91	PutR, transcriptional activator of PutA and PutP	fig|6666666.59329.peg.1610	None	fig|6666666.59331.peg.5326	None	None	None	None	None	fig|6666666.59338.peg.5682	None	None	None	None	None	None	None	None	None	None	None	None	None	None	None	fig|6666666.59355.peg.5851	None

95	transposase, putative	fig|6666666.59329.peg.2966	None	None	fig|6666666.59332.peg.60	None	None	None	None	fig|6666666.59338.peg.858	None	fig|6666666.59340.peg.281	None	None	None	None	None	None	None	None	None	None	None	None	None	None	None

1173	MrfF	None	None	None	None	None	None	None	None	None	None	None	None	None	fig|6666666.71181.peg.749	fig|6666666.59344.peg.727	None	None	None	None	None	None	None	None	fig|6666666.59353.peg.952	fig|6666666.59355.peg.2425	None

1170	PTS system, mannose-specific IIB component EC 2.7.1.69 PTS system, mannose-specific IIA component EC 2.7.1.69	fig|6666666.59329.peg.621	None	None	None	None	None	None	None	fig|6666666.59338.peg.4055	None	None	None	None	None	None	None	fig|6666666.59341.peg.3086	None	None	None	None	fig|6666666.59350.peg.5375	None	None	None	None

7186	Glutamyl-tRNA synthetase EC 6.1.1.17	None	None	None	None	None	None	fig|6666666.59336.peg.3749	fig|6666666.59333.peg.208	None	None	None	fig|6666666.59337.peg.3333	None	None	None	None	fig|6666666.59341.peg.2517	None	None	None	None	None	None	None	None	None

872	Aspartate dehydrogenase homolog	None	None	None	None	None	None	None	None	None	None	None	None	None	fig|6666666.71181.peg.5038	fig|6666666.59344.peg.6080	None	None	None	None	None	None	None	None	fig|6666666.59353.peg.5075	fig|6666666.59355.peg.5164	None

8019	Beta-glucoside bgl operon antiterminator, BglG family	None	None	None	None	None	None	None	None	None	None	None	None	None	fig|6666666.71181.peg.2176	fig|6666666.59344.peg.1134	None	None	None	None	None	None	None	None	fig|6666666.59353.peg.807	fig|6666666.59355.peg.899	None

871	Molybdenum cofactor biosynthesis protein MoaA	None	None	None	None	None	None	None	None	fig|6666666.59338.peg.2311	None	fig|6666666.59340.peg.3676	None	None	None	fig|6666666.59344.peg.4100	None	None	None	None	None	None	None	None	None	fig|6666666.59355.peg.2678	None

8012	Aminopeptidase Y Arg, Lys, Leu preference EC 3.4.11.15	None	None	None	None	None	fig|6666666.59335.peg.4917	None	None	None	fig|6666666.59339.peg.4074	None	None	None	None	None	None	None	None	None	None	fig|6666666.59349.peg.4109	None	fig|6666666.59351.peg.1088	None	None	None

7456	hypothetical protein	None	None	None	None	None	None	fig|6666666.59336.peg.1217	fig|6666666.59333.peg.1608	None	None	None	None	fig|6666666.59342.peg.188	None	None	fig|6666666.59345.peg.4574	None	None	None	None	None	None	None	None	None	None

625	hypothetical protein	None	None	None	None	None	None	None	None	None	None	None	None	None	fig|6666666.71181.peg.2366	fig|6666666.59344.peg.3359	None	None	None	None	None	None	None	None	fig|6666666.59353.peg.3735	fig|6666666.59355.peg.3752	None

7450	major facilitator superfamily MFS 1	None	None	None	None	None	None	None	None	None	None	None	None	None	fig|6666666.71181.peg.2313	fig|6666666.59344.peg.4555	None	None	None	None	None	None	None	None	fig|6666666.59353.peg.3342	fig|6666666.59355.peg.3453	None

624	Galactoside O-acetyltransferase EC 2.3.1.18	None	None	None	fig|6666666.59332.peg.5556	None	None	fig|6666666.59336.peg.6460	None	None	None	None	None	None	None	None	None	fig|6666666.59341.peg.1647	None	None	fig|6666666.59348.peg.2900	None	None	None	None	None	None

7458	FIG01056244 hypothetical protein	None	None	None	None	None	None	None	None	None	None	None	None	None	fig|6666666.71181.peg.849	fig|6666666.59344.peg.28	None	None	None	None	None	None	None	None	fig|6666666.59353.peg.366	fig|6666666.59355.peg.1403	None

4595	SSU ribosomal protein S19p S15e	None	None	None	None	None	fig|6666666.59335.peg.4347	fig|6666666.59336.peg.145	None	None	None	None	None	None	None	None	fig|6666666.59345.peg.2536	None	None	None	fig|6666666.59348.peg.414	None	None	None	None	None	None

10886	hypothetical protein	None	None	None	None	None	fig|6666666.59335.peg.3258	None	fig|6666666.59333.peg.5147	None	None	None	fig|6666666.59337.peg.3047	None	None	None	None	None	None	None	fig|6666666.59348.peg.241	None	None	None	None	None	None

8291	Short chain dehydrogenase	None	None	None	None	None	None	None	None	None	None	None	None	None	fig|6666666.71181.peg.3794	fig|6666666.59344.peg.2508	None	None	None	None	None	None	None	None	fig|6666666.59353.peg.4641	fig|6666666.59355.peg.4758	None

4599	2-ketogluconate utilization repressor PtxS	None	None	None	None	None	None	None	None	None	None	None	None	None	fig|6666666.71181.peg.648	fig|6666666.59344.peg.4148	None	None	None	None	None	None	None	None	fig|6666666.59353.peg.4123	fig|6666666.59355.peg.2317	None

4734	FIG01055756 hypothetical protein	None	None	None	None	None	None	None	None	None	None	None	None	None	fig|6666666.71181.peg.3588	fig|6666666.59344.peg.5563	None	None	None	None	None	None	None	None	fig|6666666.59353.peg.4134	fig|6666666.59355.peg.4281	None

4843	Predicted amidohydrolase RutB in novel pyrimidine catabolism pathway	None	None	None	None	None	None	None	None	None	None	None	None	None	fig|6666666.71181.peg.326	fig|6666666.59344.peg.1265	None	None	None	None	None	None	None	None	fig|6666666.59353.peg.4220	fig|6666666.59355.peg.4183	None

392	probable exported protein YPO2521	None	None	None	None	None	None	None	None	None	None	None	None	None	fig|6666666.71181.peg.3994	fig|6666666.59344.peg.5633	None	None	None	None	None	None	None	None	fig|6666666.59353.peg.2973	fig|6666666.59355.peg.2878	None

2302	WD-40 repeat protein	None	None	None	None	None	None	None	None	None	None	None	None	None	fig|6666666.71181.peg.2477	fig|6666666.59344.peg.5408	None	None	None	None	None	None	None	None	fig|6666666.59353.peg.3602	fig|6666666.59355.peg.4355	None

2305	Potassium uptake protein, integral membrane component, KtrB	None	None	fig|6666666.59331.peg.1407	fig|6666666.59332.peg.4181	None	None	None	None	fig|6666666.59338.peg.3136	None	None	None	None	None	None	None	None	None	fig|6666666.59346.peg.1056	None	None	None	None	None	None	None

11285	1-pyrroline-4-hydroxy-2-carboxylate deaminase EC 3.5.4.22	None	None	None	None	None	fig|6666666.59335.peg.5358	fig|6666666.59336.peg.5061	None	None	fig|6666666.59339.peg.8782	None	None	None	None	None	None	None	None	None	None	None	None	None	None	None	fig|6666666.59354.peg.965

11284	FIG01200954 hypothetical protein	fig|6666666.59329.peg.3826	None	None	None	None	None	fig|6666666.59336.peg.3356	None	None	None	None	fig|6666666.59337.peg.3060	None	None	None	fig|6666666.59345.peg.4741	None	None	None	None	None	None	None	None	None	None

5849	LysR family transcriptional regulator YafC	None	None	None	None	None	None	None	None	None	None	None	None	None	fig|6666666.71181.peg.3923	fig|6666666.59344.peg.5399	None	None	None	None	None	None	None	None	fig|6666666.59353.peg.4780	fig|6666666.59355.peg.4833	None

3748	Homoserine kinase EC 2.7.1.39	None	None	fig|6666666.59331.peg.4924	None	None	None	None	None	fig|6666666.59338.peg.3317	fig|6666666.59339.peg.7416	None	None	None	None	None	None	None	None	None	None	fig|6666666.59349.peg.3475	None	None	None	None	None

4622	FIG01045568 hypothetical protein	None	None	None	None	None	None	None	None	None	None	None	None	None	fig|6666666.71181.peg.711	fig|6666666.59344.peg.687	None	None	None	None	None	None	None	None	fig|6666666.59353.peg.987	fig|6666666.59355.peg.2387	None

4625	Amino acid permease	None	None	None	None	None	None	None	None	None	None	None	None	None	fig|6666666.71181.peg.4263	fig|6666666.59344.peg.6021	None	None	None	None	None	None	None	None	fig|6666666.59353.peg.2498	fig|6666666.59355.peg.591	None

4627	FMN reductase EC 1.5.1.29	None	None	None	None	None	None	None	None	None	None	None	None	None	fig|6666666.71181.peg.4773	fig|6666666.59344.peg.1172	None	None	None	None	None	None	None	None	fig|6666666.59353.peg.1679	fig|6666666.59355.peg.1041	None

4628	Serine threonine protein kinase	None	None	fig|6666666.59331.peg.4442	fig|6666666.59332.peg.838	None	None	None	None	None	None	None	None	None	None	None	None	fig|6666666.59341.peg.3401	None	fig|6666666.59346.peg.3994	None	None	None	None	None	None	None

176	hypothetical protein	None	None	fig|6666666.59331.peg.1108	fig|6666666.59332.peg.5122	None	None	None	None	None	None	None	fig|6666666.59337.peg.936	None	None	None	None	None	None	fig|6666666.59346.peg.1261	None	None	None	None	None	None	None

10469	hypothetical protein	None	None	None	None	None	None	None	None	None	None	None	None	None	fig|6666666.71181.peg.2959	fig|6666666.59344.peg.2879	None	None	None	None	None	None	None	None	fig|6666666.59353.peg.1445	fig|6666666.59355.peg.1994	None

10390	Spermidine synthase EC 2.5.1.16	None	None	None	None	None	None	None	None	None	None	None	None	None	None	None	None	None	None	None	None	fig|6666666.59349.peg.4135	fig|6666666.59350.peg.418	fig|6666666.59351.peg.119	None	None	fig|6666666.59354.peg.4930

7218	Periplasmic binding protein	None	None	None	None	None	None	None	None	None	None	None	None	None	fig|6666666.71181.peg.1636	fig|6666666.59344.peg.5023	None	None	None	None	None	None	None	None	fig|6666666.59353.peg.329	fig|6666666.59355.peg.1368	None

7216	Putative membrane protein	None	None	None	None	None	None	None	None	None	None	None	None	None	fig|6666666.71181.peg.1502	fig|6666666.59344.peg.5813	None	None	None	None	None	None	None	None	fig|6666666.59353.peg.1234	fig|6666666.59355.peg.241	None

6216	hypothetical protein	None	None	None	None	None	None	None	None	None	None	None	None	None	fig|6666666.71181.peg.3256	fig|6666666.59344.peg.4010	None	None	None	None	None	None	None	None	fig|6666666.59353.peg.4040	fig|6666666.59355.peg.3997	None

6214	FIG01056174 hypothetical protein	None	None	None	None	None	None	None	None	None	None	None	None	None	fig|6666666.71181.peg.1229	fig|6666666.59344.peg.183	None	None	None	None	None	None	None	None	fig|6666666.59353.peg.491	fig|6666666.59355.peg.2215	None

6212	formate dehydrogenase formation protein FdhE	None	None	None	None	None	None	None	None	None	None	None	None	None	fig|6666666.71181.peg.675	fig|6666666.59344.peg.4110	None	None	None	None	None	None	None	None	fig|6666666.59353.peg.4090	fig|6666666.59355.peg.2349	None

6218	FIG01055459 hypothetical protein	None	None	None	None	None	None	None	None	None	None	None	None	None	fig|6666666.71181.peg.1567	fig|6666666.59344.peg.2020	None	None	None	None	None	None	None	None	fig|6666666.59353.peg.2906	fig|6666666.59355.peg.2833	None

6814	Putative uncharacterized protein ydbH	None	None	None	None	None	None	None	None	None	None	None	None	None	fig|6666666.71181.peg.1365	fig|6666666.59344.peg.562	None	None	None	None	None	None	None	None	fig|6666666.59353.peg.1866	fig|6666666.59355.peg.1147	None

6817	putative sugar phosphotransferase component II B	None	None	None	None	None	None	None	None	None	None	None	None	None	fig|6666666.71181.peg.2696	fig|6666666.59344.peg.3852	None	None	None	None	None	None	None	None	fig|6666666.59353.peg.3982	fig|6666666.59355.peg.3898	None

9754	hypothetical protein	None	None	None	None	None	None	None	None	None	None	None	None	None	fig|6666666.71181.peg.1799	fig|6666666.59344.peg.3103	None	None	None	None	None	None	None	None	fig|6666666.59353.peg.2252	fig|6666666.59355.peg.1944	None

9759	hypothetical protein	None	None	None	None	None	None	None	None	None	None	None	None	None	fig|6666666.71181.peg.1605	fig|6666666.59344.peg.2415	None	None	None	None	None	None	None	None	fig|6666666.59353.peg.297	fig|6666666.59355.peg.1337	None

8849	Threonine dehydratase, catabolic EC 4.3.1.19	None	None	None	None	None	None	None	None	None	None	None	None	None	fig|6666666.71181.peg.628	fig|6666666.59344.peg.4180	None	None	None	None	None	None	None	None	fig|6666666.59353.peg.3051	fig|6666666.59355.peg.2295	None

1761	FIG01203009 hypothetical protein	None	None	fig|6666666.59331.peg.1234	fig|6666666.59332.peg.4385	fig|6666666.59334.peg.4157	None	None	None	None	None	None	None	None	None	None	None	None	None	fig|6666666.59346.peg.3305	None	None	None	None	None	None	None

692	Transcriptional regulator, Cro CI family	None	None	None	None	None	None	None	None	None	None	None	None	None	fig|6666666.71181.peg.320	fig|6666666.59344.peg.1257	None	None	None	None	None	None	None	None	fig|6666666.59353.peg.4227	fig|6666666.59355.peg.4189	None

695	Alkaline phosphatase EC 3.1.3.1	fig|6666666.59329.peg.1955	None	None	None	None	None	fig|6666666.59336.peg.2064	None	None	None	None	fig|6666666.59337.peg.3795	None	None	None	fig|6666666.59345.peg.1943	None	None	None	None	None	None	None	None	None	None

8719	hypothetical protein	None	None	None	None	None	None	None	None	None	None	None	None	None	fig|6666666.71181.peg.745	fig|6666666.59344.peg.723	None	None	None	None	None	None	None	None	fig|6666666.59353.peg.956	fig|6666666.59355.peg.2421	None

10043	hypothetical protein	None	None	None	None	None	None	None	None	None	None	None	None	None	fig|6666666.71181.peg.348	fig|6666666.59344.peg.2350	None	None	None	None	None	None	None	None	fig|6666666.59353.peg.242	fig|6666666.59355.peg.1282	None

5619	Transcriptional regulator, LysR family	None	None	None	None	None	None	None	None	None	None	None	None	None	fig|6666666.71181.peg.2315	fig|6666666.59344.peg.4553	None	None	None	None	None	None	None	None	fig|6666666.59353.peg.3340	fig|6666666.59355.peg.3455	None

10696	Coenzyme PQQ synthesis protein B	None	None	None	None	None	None	None	None	None	None	None	None	None	fig|6666666.71181.peg.1286	fig|6666666.59344.peg.2971	None	None	None	None	None	None	None	None	fig|6666666.59353.peg.3313	fig|6666666.59355.peg.3139	None

9525	Rare lipoprotein A precursor	None	None	None	None	None	None	None	None	None	None	None	None	None	fig|6666666.71181.peg.5518	fig|6666666.59344.peg.3334	None	None	None	None	None	None	None	None	fig|6666666.59353.peg.3716	fig|6666666.59355.peg.3731	None

6775	hypothetical protein	None	None	None	None	None	None	fig|6666666.59336.peg.387	None	None	None	None	fig|6666666.59337.peg.3228	fig|6666666.59342.peg.6165	None	None	fig|6666666.59345.peg.1818	None	None	None	None	None	None	None	None	None	None

6777	Major facilitator superfamily	None	None	None	None	None	None	None	None	None	None	None	None	None	fig|6666666.71181.peg.1470	fig|6666666.59344.peg.3878	None	None	None	None	None	None	None	None	fig|6666666.59353.peg.4698	fig|6666666.59355.peg.4080	None

9960	hypothetical protein	None	None	None	None	None	None	None	None	None	None	None	None	None	fig|6666666.71181.peg.2965	fig|6666666.59344.peg.2886	None	None	None	None	None	None	None	None	fig|6666666.59353.peg.1439	fig|6666666.59355.peg.1988	None

4665	Osmotically inducible lipoprotein B precursor	None	None	None	None	None	None	None	None	None	None	None	None	None	fig|6666666.71181.peg.4201	fig|6666666.59344.peg.494	None	None	None	None	None	None	None	None	fig|6666666.59353.peg.1810	fig|6666666.59355.peg.1206	None

9509	FIG01055482 hypothetical protein	None	None	None	None	None	None	None	None	None	None	None	None	None	fig|6666666.71181.peg.1084	fig|6666666.59344.peg.6008	None	None	None	None	None	None	None	None	fig|6666666.59353.peg.4972	fig|6666666.59355.peg.5064	None

9507	ABC-type cobalamin Fe3+-siderophores transport systems, ATPase components	None	None	None	None	None	None	None	None	None	None	None	None	None	fig|6666666.71181.peg.1156	fig|6666666.59344.peg.2278	None	None	None	None	None	None	None	None	fig|6666666.59353.peg.3426	fig|6666666.59355.peg.3526	None

3689	Manganese ABC transporter, ATP-binding protein SitB	None	None	None	None	fig|6666666.59334.peg.1846	None	None	None	None	None	None	None	fig|6666666.59342.peg.4909	None	fig|6666666.59344.peg.821	None	None	None	None	None	None	fig|6666666.59350.peg.6436	None	None	None	None

3688	hypothetical protein	fig|6666666.59329.peg.1939	None	None	fig|6666666.59332.peg.5024	None	None	None	None	None	None	None	fig|6666666.59337.peg.3788	None	fig|6666666.71181.peg.4601	None	None	None	None	None	None	None	None	None	None	None	None

3683	MrfB	None	None	None	None	None	None	None	None	None	None	None	None	None	fig|6666666.71181.peg.753	fig|6666666.59344.peg.734	None	None	None	None	None	None	None	None	fig|6666666.59353.peg.948	fig|6666666.59355.peg.2430	None

3685	hypothetical protein	None	None	None	None	None	None	None	None	None	None	None	None	None	fig|6666666.71181.peg.101	fig|6666666.59344.peg.3159	None	None	None	None	None	None	None	None	fig|6666666.59353.peg.1911	fig|6666666.59355.peg.1497	None

2511	Phosphatidylserine phosphatidylglycerophosphate cardiolipi n synthases and related enzymes	None	None	None	None	None	None	None	None	None	None	None	None	None	fig|6666666.71181.peg.4455	fig|6666666.59344.peg.6024	None	None	None	None	None	None	None	None	fig|6666666.59353.peg.2495	fig|6666666.59355.peg.589	None

3532	HipA protein	None	None	None	None	None	None	None	None	None	None	None	None	None	fig|6666666.71181.peg.2986	fig|6666666.59344.peg.3985	None	None	None	None	None	None	None	None	fig|6666666.59353.peg.2450	fig|6666666.59355.peg.543	None

3535	HigB toxin protein	None	None	None	None	None	None	None	None	None	None	None	None	None	fig|6666666.71181.peg.4845	fig|6666666.59344.peg.5648	None	None	None	None	None	None	None	None	fig|6666666.59353.peg.2399	fig|6666666.59355.peg.1672	None

3536	hypothetical protein	None	None	None	None	None	None	None	None	None	None	None	None	None	fig|6666666.71181.peg.1109	fig|6666666.59344.peg.3616	None	None	None	None	None	None	None	None	fig|6666666.59353.peg.2169	fig|6666666.59355.peg.1864	None

5054	hypothetical protein	None	None	None	None	None	None	None	None	None	None	None	None	None	fig|6666666.71181.peg.923	fig|6666666.59344.peg.5161	None	None	None	None	None	None	None	None	fig|6666666.59353.peg.891	fig|6666666.59355.peg.813	None

10760	Holin	None	None	None	None	None	None	None	None	None	None	None	None	None	fig|6666666.71181.peg.3973	fig|6666666.59344.peg.1969	None	None	None	None	None	None	None	None	fig|6666666.59353.peg.2948	fig|6666666.59355.peg.2791	None

10765	3-demethylubiquinone-9 3-methyltransferase	None	None	None	None	None	None	None	None	None	None	None	None	None	fig|6666666.71181.peg.3391	fig|6666666.59344.peg.4825	None	None	None	None	None	None	None	None	fig|6666666.59353.peg.3499	fig|6666666.59355.peg.3224	None

10439	FIG01205831 hypothetical protein	None	None	fig|6666666.59331.peg.2954	None	None	fig|6666666.59335.peg.1884	None	None	None	None	None	None	None	None	None	None	None	None	fig|6666666.59346.peg.5168	fig|6666666.59348.peg.1491	None	None	None	None	None	None

10185	hypothetical protein	None	None	None	None	None	None	None	None	None	None	None	None	None	fig|6666666.71181.peg.1698	fig|6666666.59344.peg.3929	None	None	None	None	None	None	None	None	fig|6666666.59353.peg.3715	fig|6666666.59355.peg.3038	None

10186	hypothetical protein	None	None	None	None	None	fig|6666666.59335.peg.4725	None	None	None	fig|6666666.59339.peg.2163	None	None	None	None	None	None	None	None	None	fig|6666666.59348.peg.1106	None	None	fig|6666666.59351.peg.3510	None	None	None

8037	Cold shock protein CspI	None	None	None	None	None	None	None	None	None	None	None	None	None	fig|6666666.71181.peg.4188	fig|6666666.59344.peg.2791	None	None	None	None	None	None	None	None	fig|6666666.59353.peg.1654	fig|6666666.59355.peg.1067	None

8031	N-methyl-L-amino-acid oxidase EC 1.5.3.2 ; N-methyl-L-tryptophan oxidase EC 1.5.3.-	None	None	None	None	None	None	None	None	None	None	None	None	None	fig|6666666.71181.peg.1607	fig|6666666.59344.peg.2417	None	None	None	None	None	None	None	None	fig|6666666.59353.peg.299	fig|6666666.59355.peg.1339	None

4295	3-oxoacyl-[acyl-carrier protein] reductase EC 1.1.1.100	None	None	None	None	None	None	None	None	None	None	None	None	None	fig|6666666.71181.peg.2472	fig|6666666.59344.peg.5295	None	None	None	None	None	None	None	None	fig|6666666.59353.peg.3607	fig|6666666.59355.peg.4792	None

4291	hemolysin expression modulating family protein	None	None	None	None	None	None	None	None	None	None	None	None	None	fig|6666666.71181.peg.2255	fig|6666666.59344.peg.3004	None	None	None	None	None	None	None	None	fig|6666666.59353.peg.3552	fig|6666666.59355.peg.3623	None

4292	Methyl-accepting chemotaxis protein	None	None	None	None	None	fig|6666666.59335.peg.2589	None	None	None	fig|6666666.59339.peg.5483	None	None	None	None	None	None	None	None	None	fig|6666666.59348.peg.2970	None	None	fig|6666666.59351.peg.865	None	None	None

4749	VgrG protein	None	None	None	None	None	fig|6666666.59335.peg.93	fig|6666666.59336.peg.1930	None	None	fig|6666666.59339.peg.5955	None	fig|6666666.59337.peg.2405	None	None	None	None	None	None	None	None	None	None	None	None	None	None

4743	hypothetical protein	None	None	None	None	None	None	None	None	None	None	None	None	None	fig|6666666.71181.peg.1491	fig|6666666.59344.peg.1866	None	None	None	None	None	None	None	None	fig|6666666.59353.peg.1223	fig|6666666.59355.peg.252	None

5865	hypothetical protein	None	None	None	None	None	None	None	None	None	None	None	None	None	fig|6666666.71181.peg.4685	fig|6666666.59344.peg.5541	None	None	None	None	None	None	None	None	fig|6666666.59353.peg.4855	fig|6666666.59355.peg.4925	None

5860	hypothetical protein	None	None	None	None	None	None	None	None	None	None	None	None	None	fig|6666666.71181.peg.4688	fig|6666666.59344.peg.5538	None	None	None	None	None	None	None	None	fig|6666666.59353.peg.4852	fig|6666666.59355.peg.4922	None

5863	ATP-dependent Clp protease proteolytic subunit EC 3.4.21.92	None	None	None	None	None	None	None	None	None	None	None	None	None	fig|6666666.71181.peg.828	fig|6666666.59344.peg.49	None	None	None	None	None	None	None	None	fig|6666666.59353.peg.386	fig|6666666.59355.peg.1425	None

6906	FIG01056553 hypothetical protein	None	None	None	None	None	None	None	None	None	None	None	None	None	fig|6666666.71181.peg.2269	fig|6666666.59344.peg.5871	None	None	None	None	None	None	None	None	fig|6666666.59353.peg.4903	fig|6666666.59355.peg.5017	None

6903	Cupin 2 conserved barrel domain protein	None	None	None	None	None	None	None	None	None	None	None	None	None	fig|6666666.71181.peg.2141	fig|6666666.59344.peg.3211	None	None	None	None	None	None	None	None	fig|6666666.59353.peg.2828	fig|6666666.59355.peg.3931	None

154	Lipopolysaccharide core biosynthesis protein RfaZ	None	None	None	None	None	None	None	None	None	None	None	None	None	fig|6666666.71181.peg.3668	fig|6666666.59344.peg.956	None	None	None	None	None	None	None	None	fig|6666666.59353.peg.2579	fig|6666666.59355.peg.2623	None

159	Glutathione synthetase EC 6.3.2.3	None	None	None	fig|6666666.59332.peg.1939	None	None	None	fig|6666666.59333.peg.774	None	None	fig|6666666.59340.peg.206	None	None	None	None	None	fig|6666666.59341.peg.2576	None	None	None	None	None	None	None	None	None

8134	ABC transporter, periplasmic spermidine putrescine-binding protein PotD TC 3.A.1.11.1	None	None	None	None	None	None	None	None	None	None	None	None	None	fig|6666666.71181.peg.4727	fig|6666666.59344.peg.1605	None	None	None	None	None	None	None	None	fig|6666666.59353.peg.2678	fig|6666666.59355.peg.1803	None

8689	Virulence protein msgA	None	None	None	None	None	None	None	None	None	None	None	None	None	fig|6666666.71181.peg.1416	fig|6666666.59344.peg.1573	None	None	None	None	None	None	None	None	fig|6666666.59353.peg.2660	fig|6666666.59355.peg.1822	None

2725	Mu-like prophage FluMu protein gp37	None	None	None	None	None	None	None	None	None	None	None	None	None	fig|6666666.71181.peg.2882	fig|6666666.59344.peg.4383	None	None	None	None	None	None	None	None	fig|6666666.59353.peg.4295	fig|6666666.59355.peg.4408	None

2729	Chromosome partition protein MukB	None	None	fig|6666666.59331.peg.2938	None	fig|6666666.59334.peg.589	None	None	None	None	fig|6666666.59339.peg.322	None	None	None	None	None	None	None	None	None	fig|6666666.59348.peg.4806	None	None	None	None	None	None

7234	Phage major tail tube protein	None	None	None	None	None	None	None	None	None	fig|6666666.59339.peg.1116	None	None	None	None	None	None	None	None	None	None	fig|6666666.59349.peg.1992	None	fig|6666666.59351.peg.1469	None	None	fig|6666666.59354.peg.2847

10562	Putative deaminase YahJ	None	None	None	None	None	None	None	None	None	None	None	None	None	fig|6666666.71181.peg.4717	fig|6666666.59344.peg.6113	None	None	None	None	None	None	None	None	fig|6666666.59353.peg.3579	fig|6666666.59355.peg.4377	None

10560	hypothetical protein	None	None	None	None	None	None	None	None	None	None	None	None	None	fig|6666666.71181.peg.4883	fig|6666666.59344.peg.3939	None	None	None	None	None	None	None	None	fig|6666666.59353.peg.4407	fig|6666666.59355.peg.3046	None

6238	putative translation initiation inhibitor	None	None	None	None	None	None	None	None	None	None	None	None	None	fig|6666666.71181.peg.862	fig|6666666.59344.peg.11	None	None	None	None	None	None	None	None	fig|6666666.59353.peg.352	fig|6666666.59355.peg.1390	None

6230	Phosphatase NagD predicted to act in N-acetylglucosamine utilization subsystem	None	None	None	None	None	None	None	None	None	None	None	None	None	fig|6666666.71181.peg.3202	fig|6666666.59344.peg.4521	None	None	None	None	None	None	None	None	fig|6666666.59353.peg.2044	fig|6666666.59355.peg.4451	None

6233	Ner-like regulatory protein	None	None	None	None	None	None	None	None	None	None	None	None	None	fig|6666666.71181.peg.2797	fig|6666666.59344.peg.2121	None	None	None	None	None	None	None	None	fig|6666666.59353.peg.3154	fig|6666666.59355.peg.442	None

10518	hypothetical protein	None	None	None	None	None	None	None	None	None	None	None	None	None	fig|6666666.71181.peg.4381	fig|6666666.59344.peg.4804	None	None	None	None	None	None	None	None	fig|6666666.59353.peg.4335	fig|6666666.59355.peg.4534	None

8868	hypothetical protein	None	None	None	None	None	None	None	None	None	None	None	None	None	fig|6666666.71181.peg.4548	fig|6666666.59344.peg.2387	None	None	None	None	None	None	None	None	fig|6666666.59353.peg.273	fig|6666666.59355.peg.1312	None

8869	hypothetical protein	None	None	None	None	None	None	None	None	None	None	None	None	None	fig|6666666.71181.peg.1168	fig|6666666.59344.peg.2296	None	None	None	None	None	None	None	None	fig|6666666.59353.peg.3415	fig|6666666.59355.peg.3513	None

817	hypothetical protein	None	None	None	None	None	None	None	None	None	None	None	None	None	fig|6666666.71181.peg.3612	fig|6666666.59344.peg.5058	None	None	None	None	None	None	None	None	fig|6666666.59353.peg.4057	fig|6666666.59355.peg.4133	None

1705	Secreted alkaline metalloproteinase EC 3.4.24.- , PrtA B C G homolog	None	None	None	None	None	None	None	None	None	None	None	None	None	fig|6666666.71181.peg.4501	fig|6666666.59344.peg.5716	None	None	None	None	None	None	None	None	fig|6666666.59353.peg.4938	fig|6666666.59355.peg.3355	None

2096	Molybdenum-binding periplasmic protein	None	None	None	None	None	None	None	None	None	None	None	None	None	fig|6666666.71181.peg.1155	fig|6666666.59344.peg.2277	None	None	None	None	None	None	None	None	fig|6666666.59353.peg.3427	fig|6666666.59355.peg.3527	None

10231	hypothetical protein	None	None	None	None	None	None	None	None	None	None	None	None	None	fig|6666666.71181.peg.2485	fig|6666666.59344.peg.5417	None	None	None	None	None	None	None	None	fig|6666666.59353.peg.3595	fig|6666666.59355.peg.4362	None

11135	hypothetical protein	None	None	None	None	None	None	None	None	None	None	None	None	None	fig|6666666.71181.peg.3740	fig|6666666.59344.peg.3673	None	None	None	None	None	None	None	None	fig|6666666.59353.peg.3842	fig|6666666.59355.peg.1545	None

11131	hypothetical protein	None	None	None	None	None	None	None	None	None	None	None	None	None	fig|6666666.71181.peg.3990	fig|6666666.59344.peg.5637	None	None	None	None	None	None	None	None	fig|6666666.59353.peg.2969	fig|6666666.59355.peg.2874	None

11138	3-oxoacyl-[acyl-carrier-protein] synthase, KASII EC 2.3.1.41	None	None	None	None	None	None	None	None	None	None	None	None	None	fig|6666666.71181.peg.114	fig|6666666.59344.peg.3142	None	None	None	None	None	None	None	None	fig|6666666.59353.peg.1899	fig|6666666.59355.peg.1510	None

3028	Cs C3146	None	None	None	None	None	None	None	None	None	None	None	None	None	None	None	None	None	None	None	None	fig|6666666.59349.peg.4261	fig|6666666.59350.peg.6092	fig|6666666.59351.peg.5286	None	None	fig|6666666.59354.peg.1610

3027	Sulfate and thiosulfate binding protein CysP	None	None	None	None	None	None	None	None	None	None	None	None	None	fig|6666666.71181.peg.4462	fig|6666666.59344.peg.1991	None	None	None	None	None	None	None	None	fig|6666666.59353.peg.2930	fig|6666666.59355.peg.2810	None

3025	Putative lipoprotein	None	None	None	None	None	None	None	None	None	None	None	None	None	fig|6666666.71181.peg.2433	fig|6666666.59344.peg.2486	None	None	None	None	None	None	None	None	fig|6666666.59353.peg.3632	fig|6666666.59355.peg.4992	None

8736	Uncharacterized membrane protein YqjD	None	None	None	None	None	None	None	None	None	None	None	None	None	fig|6666666.71181.peg.4398	fig|6666666.59344.peg.1855	None	None	None	None	None	None	None	None	fig|6666666.59353.peg.2384	fig|6666666.59355.peg.1656	None

8730	hypothetical protein	None	None	None	None	None	None	None	None	None	None	None	None	None	fig|6666666.71181.peg.4644	fig|6666666.59344.peg.5933	None	None	None	None	None	None	None	None	fig|6666666.59353.peg.5024	fig|6666666.59355.peg.5116	None

4197	hypothetical protein	None	None	None	None	None	None	None	None	None	None	None	None	None	fig|6666666.71181.peg.2408	fig|6666666.59344.peg.794	None	None	None	None	None	None	None	None	fig|6666666.59353.peg.1313	fig|6666666.59355.peg.2558	None

4193	N-3-oxooctanoyl-L-homoserine lactone synthase; N-3-oxohexanoyl-L-homoserine lactone synthase	None	None	None	None	None	None	None	None	None	None	None	None	None	fig|6666666.71181.peg.656	fig|6666666.59344.peg.4136	None	None	None	None	None	None	None	None	fig|6666666.59353.peg.4113	fig|6666666.59355.peg.2326	None

5672	C2110, Macro domain, possibly ADP-ribose binding module	None	None	None	None	None	fig|6666666.59335.peg.658	None	None	fig|6666666.59338.peg.643	fig|6666666.59339.peg.4219	None	None	None	None	None	None	None	fig|6666666.59347.peg.37	None	None	None	None	None	None	None	None

5679	Putative exported protein precursor	None	None	None	None	None	None	None	None	None	None	None	None	None	fig|6666666.71181.peg.2412	fig|6666666.59344.peg.798	None	None	None	None	None	None	None	None	fig|6666666.59353.peg.1309	fig|6666666.59355.peg.2562	None

10235	2-Keto-D-gluconate dehydrogenase EC 1.1.99.4 , membrane-bound, gamma subunit	None	None	None	None	None	None	None	None	None	None	None	None	None	fig|6666666.71181.peg.135	fig|6666666.59344.peg.3659	None	None	None	None	None	None	None	None	fig|6666666.59353.peg.3855	fig|6666666.59355.peg.1531	None

6436	Predicted transcriptional regulator for fatty acid degradation FadQ, TetR family	None	None	fig|6666666.59331.peg.1367	fig|6666666.59332.peg.1221	None	None	None	None	None	fig|6666666.59339.peg.439	None	None	None	None	None	None	None	None	fig|6666666.59346.peg.5081	None	None	None	None	None	None	None

6432	putative hemagglutinin hemolysin-related protein	None	None	None	None	None	None	fig|6666666.59336.peg.1333	fig|6666666.59333.peg.600	None	None	None	fig|6666666.59337.peg.3683	None	None	None	fig|6666666.59345.peg.683	None	None	None	None	None	None	None	None	None	None

10309	SgrR, sugar-phosphate stress, transcriptional activator of SgrS small RNA	fig|6666666.59329.peg.3504	None	None	None	None	None	fig|6666666.59336.peg.6601	fig|6666666.59333.peg.5548	None	None	None	fig|6666666.59337.peg.5666	None	None	None	None	None	None	None	None	None	None	None	None	None	None

6758	Segregation and condensation protein B	None	None	None	None	None	None	None	None	None	None	None	None	None	None	None	None	None	None	None	None	fig|6666666.59349.peg.4267	fig|6666666.59350.peg.3958	fig|6666666.59351.peg.795	None	None	fig|6666666.59354.peg.3825

6752	FIG138928 iron-regulated membrane protein	None	None	None	None	fig|6666666.59334.peg.662	None	None	None	None	None	None	None	None	None	None	None	None	None	None	None	fig|6666666.59349.peg.3097	None	fig|6666666.59351.peg.2963	None	None	fig|6666666.59354.peg.3458

9941	Transcriptional regulator of xylitol utilization, LacI family	None	None	None	None	None	None	None	None	None	None	None	None	None	fig|6666666.71181.peg.3567	fig|6666666.59344.peg.3960	None	None	None	None	None	None	None	None	fig|6666666.59353.peg.4389	fig|6666666.59355.peg.3065	None

10692	DNA primase EC 2.7.7.-	None	None	None	None	None	None	None	None	None	None	None	None	None	fig|6666666.71181.peg.2351	fig|6666666.59344.peg.3374	None	None	None	None	None	None	None	None	fig|6666666.59353.peg.3747	fig|6666666.59355.peg.3765	None

236	Heat-inducible transcription repressor HrcA	None	None	None	None	None	None	None	None	None	None	None	None	None	None	None	None	None	None	None	None	fig|6666666.59349.peg.2384	fig|6666666.59350.peg.1498	fig|6666666.59351.peg.645	None	None	fig|6666666.59354.peg.4835

10304	ABC transporter ATP-binding protein uup	None	None	None	fig|6666666.59332.peg.5382	None	fig|6666666.59335.peg.1224	None	None	None	None	None	None	None	None	None	None	None	None	None	None	None	None	fig|6666666.59351.peg.3169	None	None	fig|6666666.59354.peg.720

2535	MSHA biogenesis protein MshQ	None	fig|6666666.59330.peg.23	None	None	None	fig|6666666.59335.peg.215	None	None	None	None	None	fig|6666666.59337.peg.808	None	None	None	fig|6666666.59345.peg.4886	None	None	None	None	None	None	None	None	None	None

5078	ATP synthase delta chain EC 3.6.3.14	None	None	None	None	None	None	None	None	None	None	None	None	None	fig|6666666.71181.peg.2179	fig|6666666.59344.peg.1138	None	None	None	None	None	None	None	None	fig|6666666.59353.peg.811	fig|6666666.59355.peg.895	None

5079	Phenylacetate-coenzyme A ligase EC 6.2.1.30 PaaF	None	None	None	None	None	None	None	None	None	None	None	None	None	None	fig|6666666.59344.peg.3325	None	None	None	None	None	None	fig|6666666.59350.peg.3524	None	fig|6666666.59353.peg.2757	fig|6666666.59355.peg.3673	None

5073	Death on curing protein, Doc toxin	None	None	None	None	None	None	None	None	None	None	None	None	None	fig|6666666.71181.peg.3979	fig|6666666.59344.peg.1975	None	None	None	None	None	None	None	None	fig|6666666.59353.peg.2942	fig|6666666.59355.peg.2797	None

5070	Aldo-keto reductase	None	None	None	None	None	None	None	None	None	None	None	None	None	fig|6666666.71181.peg.1451	fig|6666666.59344.peg.1531	None	None	None	None	None	None	None	None	fig|6666666.59353.peg.4367	fig|6666666.59355.peg.4476	None

938	Agmatinase EC 3.5.3.11	None	None	None	None	None	None	None	None	fig|6666666.59338.peg.1450	None	fig|6666666.59340.peg.43	None	None	None	None	None	fig|6666666.59341.peg.1319	fig|6666666.59347.peg.3162	None	None	None	None	None	None	None	None

932	hypothetical protein	None	None	None	None	None	None	None	None	None	fig|6666666.59339.peg.4736	None	None	fig|6666666.59342.peg.6199	None	None	fig|6666666.59345.peg.3987	None	fig|6666666.59347.peg.5318	None	None	None	None	None	None	None	None

937	Fatty acid cis trans isomerase	None	None	None	None	fig|6666666.59334.peg.3454	None	fig|6666666.59336.peg.6095	None	fig|6666666.59338.peg.2561	None	None	None	None	None	None	None	None	None	None	None	None	None	None	None	None	fig|6666666.59354.peg.4735

934	3-polyprenyl-4-hydroxybenzoate carboxy-lyase EC 4.1.1.-	None	None	None	None	fig|6666666.59334.peg.422	None	None	None	None	fig|6666666.59339.peg.7688	None	None	None	None	None	None	None	None	None	None	None	None	fig|6666666.59351.peg.4763	None	None	fig|6666666.59354.peg.1081

5380	hypothetical protein	None	None	None	None	None	None	None	None	None	None	None	None	None	fig|6666666.71181.peg.2566	fig|6666666.59344.peg.752	None	None	None	None	None	None	None	None	fig|6666666.59353.peg.1350	fig|6666666.59355.peg.2522	None

5383	Flagellar transcriptional activator FlhD	None	None	None	None	None	None	None	None	None	None	None	None	None	fig|6666666.71181.peg.2253	fig|6666666.59344.peg.3006	None	None	None	None	None	None	None	None	fig|6666666.59353.peg.3550	fig|6666666.59355.peg.3625	None

5384	Mobile element protein	None	None	None	None	None	None	None	None	None	None	None	None	None	fig|6666666.71181.peg.737	fig|6666666.59344.peg.715	None	None	None	None	None	None	None	None	fig|6666666.59353.peg.964	fig|6666666.59355.peg.2412	None

5385	FIG01201672 hypothetical protein	None	fig|6666666.59330.peg.3008	None	fig|6666666.59332.peg.581	None	None	None	None	None	None	None	None	fig|6666666.59342.peg.5100	None	None	None	None	None	fig|6666666.59346.peg.160	None	None	None	None	None	None	None

10795	Sialic acid utilization regulator, RpiR family	None	None	None	None	None	None	None	None	None	None	None	None	None	fig|6666666.71181.peg.1268	fig|6666666.59344.peg.132	None	None	None	None	None	None	None	None	fig|6666666.59353.peg.449	fig|6666666.59355.peg.2176	None

792	Ribose xylose arabinose galactoside ABC-type transport systems, ATP-binding protein EC 3.6.3.17	None	None	None	None	None	None	None	None	None	None	None	None	None	fig|6666666.71181.peg.1062	fig|6666666.59344.peg.4965	None	None	None	None	None	None	None	None	fig|6666666.59353.peg.4513	fig|6666666.59355.peg.4623	None

798	hypothetical protein	None	None	None	None	None	None	None	None	None	None	None	None	None	fig|6666666.71181.peg.1331	fig|6666666.59344.peg.2919	None	None	None	None	None	None	None	None	fig|6666666.59353.peg.3269	fig|6666666.59355.peg.3183	None

2144	Putative lipoprotein	fig|6666666.59329.peg.6156	None	None	None	None	None	fig|6666666.59336.peg.3021	None	None	None	None	fig|6666666.59337.peg.3862	None	None	None	fig|6666666.59345.peg.308	None	None	None	None	None	None	None	None	None	None

2415	Molybdopterin biosynthesis Mog protein, molybdochelatase	None	None	None	None	None	None	None	None	None	None	None	None	None	fig|6666666.71181.peg.430	fig|6666666.59344.peg.1332	None	None	None	None	None	None	None	None	fig|6666666.59353.peg.68	fig|6666666.59355.peg.675	None

2141	Phosphate ABC transporter, periplasmic phosphate-binding protein PstS TC 3.A.1.7.1	fig|6666666.59329.peg.4131	None	None	None	None	None	None	None	fig|6666666.59338.peg.6134	None	None	fig|6666666.59337.peg.1434	None	None	None	None	None	None	fig|6666666.59346.peg.3416	None	None	None	None	None	None	None

1132	hypothetical protein	None	None	None	None	None	None	None	None	None	None	None	None	None	fig|6666666.71181.peg.4826	fig|6666666.59344.peg.2987	None	None	None	None	None	None	None	None	fig|6666666.59353.peg.3564	fig|6666666.59355.peg.3609	None

7145	Type IV secretory pathway, VirD2 component	None	fig|6666666.59330.peg.4492	fig|6666666.59331.peg.429	None	None	None	None	None	None	None	None	None	None	None	None	fig|6666666.59345.peg.3590	None	fig|6666666.59347.peg.1190	None	None	None	None	None	None	None	None

2149	transcriptional regulator, LacI family	None	None	None	None	fig|6666666.59334.peg.3468	fig|6666666.59335.peg.3146	None	None	None	None	None	None	None	None	None	fig|6666666.59345.peg.2010	fig|6666666.59341.peg.3225	None	None	None	None	None	None	None	None	None

8057	hypothetical protein	None	None	None	None	None	None	None	None	None	None	None	None	None	fig|6666666.71181.peg.80	fig|6666666.59344.peg.3184	None	None	None	None	None	None	None	None	fig|6666666.59353.peg.1935	fig|6666666.59355.peg.1472	None

8056	Uncharacterized protein ImpA	None	None	fig|6666666.59331.peg.4417	None	None	None	fig|6666666.59336.peg.5366	None	None	fig|6666666.59339.peg.4005	None	fig|6666666.59337.peg.5227	None	None	None	None	None	None	None	None	None	None	None	None	None	None

8055	FIG01199641 hypothetical protein	fig|6666666.59329.peg.640	None	None	None	None	None	fig|6666666.59336.peg.1486	fig|6666666.59333.peg.1177	None	None	None	None	None	None	None	fig|6666666.59345.peg.2745	None	None	None	None	None	None	None	None	None	None

8508	FIG004599 Hypothetical protein	None	None	None	None	None	fig|6666666.59335.peg.5516	None	None	None	None	None	None	None	None	None	None	fig|6666666.59341.peg.2353	None	None	fig|6666666.59348.peg.5211	None	None	None	None	None	fig|6666666.59354.peg.3307

8059	hypothetical protein	None	None	None	None	None	None	None	None	None	None	None	None	None	fig|6666666.71181.peg.3721	fig|6666666.59344.peg.3697	None	None	None	None	None	None	None	None	fig|6666666.59353.peg.3821	fig|6666666.59355.peg.1565	None

8058	hypothetical protein	None	None	None	None	fig|6666666.59334.peg.5242	None	None	None	fig|6666666.59338.peg.132	None	fig|6666666.59340.peg.572	None	None	None	None	None	None	fig|6666666.59347.peg.217	None	None	None	None	None	None	None	None

10457	hypothetical protein	None	None	None	None	None	None	None	None	None	None	None	None	None	fig|6666666.71181.peg.2863	fig|6666666.59344.peg.4400	None	None	None	None	None	None	None	None	fig|6666666.59353.peg.4312	fig|6666666.59355.peg.4390	None

2883	hypothetical protein	None	None	None	None	None	None	None	None	None	None	None	None	None	fig|6666666.71181.peg.3687	fig|6666666.59344.peg.3297	None	None	None	None	None	None	None	None	fig|6666666.59353.peg.2781	fig|6666666.59355.peg.3696	None

2885	hypothetical protein	None	None	None	None	None	None	None	None	None	None	None	None	None	fig|6666666.71181.peg.4691	fig|6666666.59344.peg.5534	None	None	None	None	None	None	None	None	fig|6666666.59353.peg.4849	fig|6666666.59355.peg.4920	None

10793	L-fucose mutarotase, type 2	None	None	None	None	None	None	None	None	None	None	None	None	None	fig|6666666.71181.peg.4972	fig|6666666.59344.peg.1398	None	None	None	None	None	None	None	None	fig|6666666.59353.peg.21	fig|6666666.59355.peg.627	None

2	hypothetical protein	None	None	None	None	None	None	None	None	None	None	None	None	None	fig|6666666.71181.peg.4817	fig|6666666.59344.peg.3255	None	None	None	None	None	None	None	None	fig|6666666.59353.peg.2869	fig|6666666.59355.peg.5028	None

10455	hypothetical protein	None	None	None	None	None	None	None	None	None	None	None	None	None	fig|6666666.71181.peg.4325	fig|6666666.59344.peg.825	None	None	None	None	None	None	None	None	fig|6666666.59353.peg.1286	fig|6666666.59355.peg.3295	None

1922	Biotin carboxylase of acetyl-CoA carboxylase EC 6.3.4.14	None	None	None	None	fig|6666666.59334.peg.48	fig|6666666.59335.peg.2627	None	None	None	None	None	None	None	fig|6666666.71181.peg.6311	None	None	None	None	None	None	None	None	None	None	None	fig|6666666.59354.peg.4664

3273	Multidrug efflux pump component MtrF	None	None	None	None	None	fig|6666666.59335.peg.4080	None	None	None	fig|6666666.59339.peg.5163	None	None	None	None	None	None	None	None	fig|6666666.59346.peg.3528	None	None	None	None	None	None	fig|6666666.59354.peg.2712

1926	twin-arginine translocation pathway signal	None	None	None	None	None	None	None	None	None	None	None	None	None	fig|6666666.71181.peg.2143	fig|6666666.59344.peg.3213	None	None	None	None	None	None	None	None	fig|6666666.59353.peg.2830	fig|6666666.59355.peg.3933	None

11161	ADP-heptose LPS heptosyltransferase	None	None	None	None	None	None	None	None	None	None	None	None	None	fig|6666666.71181.peg.1666	fig|6666666.59344.peg.629	None	None	None	None	None	None	None	None	fig|6666666.59353.peg.1035	fig|6666666.59355.peg.3583	None

4769	unknown	None	None	None	None	None	None	None	None	None	None	None	None	None	fig|6666666.71181.peg.1712	fig|6666666.59344.peg.5230	None	None	None	None	None	None	None	None	fig|6666666.59353.peg.3703	fig|6666666.59355.peg.3026	None

7189	hypothetical protein	None	None	None	None	None	None	None	None	None	None	None	None	None	fig|6666666.71181.peg.193	fig|6666666.59344.peg.234	None	None	None	None	None	None	None	None	fig|6666666.59353.peg.629	fig|6666666.59355.peg.112	None

5802	hypothetical protein	None	None	None	None	None	None	None	None	None	None	None	None	None	fig|6666666.71181.peg.4669	fig|6666666.59344.peg.6179	None	None	None	None	None	None	None	None	fig|6666666.59353.peg.3916	fig|6666666.59355.peg.4246	None

5805	hypothetical protein	None	None	None	None	fig|6666666.59334.peg.1084	None	None	None	fig|6666666.59338.peg.2100	None	None	None	fig|6666666.59342.peg.3366	None	None	fig|6666666.59345.peg.3162	None	None	None	None	None	None	None	None	None	None

5804	Nucleoside-diphosphate-sugar epimerases	None	None	None	None	None	None	None	None	None	None	None	None	None	fig|6666666.71181.peg.4056	fig|6666666.59344.peg.3772	None	None	None	None	None	None	None	None	fig|6666666.59353.peg.3926	fig|6666666.59355.peg.4235	None

1570	ABC transporter, periplasmic binding protein	None	None	None	None	None	None	None	None	None	None	None	None	None	fig|6666666.71181.peg.2318	fig|6666666.59344.peg.4549	None	None	None	None	None	None	None	None	fig|6666666.59353.peg.3335	fig|6666666.59355.peg.3459	None

2708	Ribose xylose arabinose galactoside ABC-type transport systems, permease component 1	None	None	None	None	None	None	None	None	None	None	None	None	None	fig|6666666.71181.peg.1060	fig|6666666.59344.peg.4963	None	None	None	None	None	None	None	None	fig|6666666.59353.peg.4515	fig|6666666.59355.peg.4625	None

4881	TagA-related protein	fig|6666666.59329.peg.3009	None	None	None	None	None	None	None	None	None	None	fig|6666666.59337.peg.2010	fig|6666666.59342.peg.2679	None	None	fig|6666666.59345.peg.3069	None	None	None	None	None	None	None	None	None	None

4882	hypothetical protein	None	fig|6666666.59330.peg.3139	None	None	None	fig|6666666.59335.peg.2463	None	None	None	fig|6666666.59339.peg.3564	None	None	None	None	None	None	fig|6666666.59341.peg.2089	None	None	None	None	None	None	None	None	None

7962	hypothetical protein	None	None	None	None	None	None	None	None	None	None	None	None	None	fig|6666666.71181.peg.1883	fig|6666666.59344.peg.4359	None	None	None	None	None	None	None	None	fig|6666666.59353.peg.3247	fig|6666666.59355.peg.3130	None

6257	FIG01055413 hypothetical protein	None	None	None	None	None	None	None	None	None	None	None	None	None	fig|6666666.71181.peg.4190	fig|6666666.59344.peg.2793	None	None	None	None	None	None	None	None	fig|6666666.59353.peg.1652	fig|6666666.59355.peg.1069	None

10532	hypothetical protein	None	None	None	None	fig|6666666.59334.peg.4971	None	None	None	fig|6666666.59338.peg.4869	None	None	None	None	None	None	None	None	fig|6666666.59347.peg.2216	fig|6666666.59346.peg.1489	None	None	None	None	None	None	None

10535	Multiple antibiotic resistance protein marC	fig|6666666.59329.peg.4839	fig|6666666.59330.peg.2241	None	None	None	None	fig|6666666.59336.peg.4150	fig|6666666.59333.peg.4487	None	None	None	None	None	None	None	None	None	None	None	None	None	None	None	None	None	None

9712	hypothetical protein	None	None	None	None	None	fig|6666666.59335.peg.5642	None	None	None	fig|6666666.59339.peg.8376	None	None	None	None	None	None	None	None	None	None	fig|6666666.59349.peg.1884	None	None	None	None	fig|6666666.59354.peg.1381

8547	DNA-damage-inducible protein I	None	None	None	None	None	None	None	None	None	None	None	None	None	fig|6666666.71181.peg.1610	fig|6666666.59344.peg.2419	None	None	None	None	None	None	None	None	fig|6666666.59353.peg.301	fig|6666666.59355.peg.1341	None

3477	conserved hypothetical protein	None	None	None	None	None	None	None	None	None	None	None	None	None	fig|6666666.71181.peg.3998	fig|6666666.59344.peg.5629	None	None	None	None	None	None	None	None	fig|6666666.59353.peg.2977	fig|6666666.59355.peg.2882	None

8884	hypothetical protein	None	None	None	None	None	None	None	None	None	None	None	None	None	fig|6666666.71181.peg.4820	fig|6666666.59344.peg.2981	None	None	None	None	None	None	None	None	fig|6666666.59353.peg.3570	fig|6666666.59355.peg.3604	None

7457	Guanine deaminase EC 3.5.4.3 ; Hydroxydechloroatrazine ethylaminohydrolase EC 3.5.99.3	None	None	fig|6666666.59331.peg.806	None	None	fig|6666666.59335.peg.4894	None	None	None	None	None	None	None	None	None	None	fig|6666666.59341.peg.3183	None	fig|6666666.59346.peg.2094	None	None	None	None	None	None	None

8687	hypothetical protein	None	None	None	None	None	None	None	None	None	None	None	None	None	fig|6666666.71181.peg.87	fig|6666666.59344.peg.3176	None	None	None	None	None	None	None	None	fig|6666666.59353.peg.1927	fig|6666666.59355.peg.1480	None

9007	FIG01200506 hypothetical protein	fig|6666666.59329.peg.4796	None	None	None	None	fig|6666666.59335.peg.5409	None	None	None	fig|6666666.59339.peg.2213	None	None	None	None	None	None	None	None	None	fig|6666666.59348.peg.2927	None	None	None	None	None	None

5248	DcrB protein precursor	None	None	None	None	None	None	None	None	None	None	None	None	None	fig|6666666.71181.peg.4496	fig|6666666.59344.peg.5721	None	None	None	None	None	None	None	None	fig|6666666.59353.peg.4943	fig|6666666.59355.peg.3350	None

1726	Accessory cholera enterotoxin	fig|6666666.59329.peg.4889	fig|6666666.59330.peg.4977	fig|6666666.59331.peg.1714	None	None	None	None	None	None	None	None	None	fig|6666666.59342.peg.3440	None	None	None	None	None	None	None	None	None	None	None	None	None

5241	Uncharacterized protein YidR	None	None	None	None	None	None	None	None	None	None	None	None	None	fig|6666666.71181.peg.636	fig|6666666.59344.peg.4193	None	None	None	None	None	None	None	None	fig|6666666.59353.peg.3043	fig|6666666.59355.peg.2305	None

9004	Oxidoreductase	None	None	None	None	None	None	None	None	None	None	None	None	None	fig|6666666.71181.peg.4296	fig|6666666.59344.peg.6254	None	None	None	None	None	None	None	None	fig|6666666.59353.peg.4003	fig|6666666.59355.peg.3962	None

5626	FIG01203911 hypothetical protein	None	None	None	None	None	None	fig|6666666.59336.peg.3146	None	None	None	None	fig|6666666.59337.peg.2279	fig|6666666.59342.peg.820	None	None	fig|6666666.59345.peg.2020	None	None	None	None	None	None	None	None	None	None

3003	Hydroxymethylglutaryl-CoA reductase EC 1.1.1.34	None	None	None	None	None	None	None	fig|6666666.59333.peg.5057	fig|6666666.59338.peg.4210	None	None	None	fig|6666666.59342.peg.3298	None	None	None	None	fig|6666666.59347.peg.892	None	None	None	None	None	None	None	None

3009	hypothetical protein	None	None	None	None	None	None	None	None	None	None	None	None	None	fig|6666666.71181.peg.2957	fig|6666666.59344.peg.2877	None	None	None	None	None	None	None	None	fig|6666666.59353.peg.1447	fig|6666666.59355.peg.1996	None

1580	hypothetical protein	None	None	None	None	None	None	None	None	None	None	None	None	None	fig|6666666.71181.peg.2386	fig|6666666.59344.peg.768	None	None	None	None	None	None	None	None	fig|6666666.59353.peg.1336	fig|6666666.59355.peg.2535	None
[truncated: 1,695,741 more chars]
